# IMIREG: a lineage-resolved regulon signature unveiling immune engagement archetypes and predicting immunotherapy response across diverse cancers

**DOI:** 10.1038/s41698-026-01453-7

**Published:** 2026-05-06

**Authors:** Bashir Lawal, Renu Sharma, Akshat Gupta, Rohit Bhargava, Laizhi Zhang, Yue Wang, Huayan Ren, Xiao-Song Wang

**Affiliations:** 1 UPMC Hillman Cancer Center, University of Pittsburgh, Pittsburgh, PA USA; 2 Department of Pathology, University of Pittsburgh, Pittsburgh, PA USA; 3 UPMC Magee-Womens Hospital, Pittsburgh, PA USA; 4 Department of Human Genetics, School of Public Health, University of Pittsburgh, Pittsburgh, PA USA

**Keywords:** Biomarkers, Cancer, Computational biology and bioinformatics, Immunology, Oncology

## Abstract

A critical barrier to maximizing immune checkpoint blockade (ICB) efficacy is the lack of biomarkers that capture the core regulatory dynamics of the tumor microenvironment. We developed IMIREG, a 14-regulon transcriptional signature derived from inferred transcription factor activity that quantifies immune engagement. Validated across 50 immunotherapy cohorts (52 treatment arms) spanning 16 cancer types, IMIREG robustly predicts clinical benefit (mean AUROC = 0.71), consistently outperforming established response and resistance signatures, including the T cell–inflamed, IFN-γ, CD8 T effector, and antigen presentation signatures. Crucially, its predictive utility is specific to the immunotherapy context rather than general prognosis. Single-cell analysis of 21 datasets revealed selective IMIREG enrichment in checkpoint-restrained exhausted/memory/effector T cells and pro-inflammatory M1 macrophages, defining an “immune-engaged but restrained” state primed for therapeutic reactivation. Our lineage-resolved framework classifies IMIREG-high tumors into T cell–, macrophage–, and dual-driven archetypes, revealing that the dual-driven phenotype is markedly diminished in metastatic compared to primary tumors. In triple-negative breast cancer, longitudinal single-cell analysis of neoadjuvant anti-PD-1/radiotherapy biopsies establishes IMIREG as a dynamic pharmacodynamic marker that discriminates spatial response trajectories, and predicts pathological complete response in on-treatment biopsies collected prior to radiation initiation. Together, IMIREG provides a mechanism-based biomarker that captures anti-tumor immune regulatory circuitry, improving ICB patient stratification and illuminating immune dynamics in cancer progression.

## Introduction

The advent of immune checkpoint blockade (ICB) has fundamentally transformed the landscape of cancer therapy, offering durable clinical responses in a growing number of malignancies^1,2^. However, a substantial number of patients do not benefit from these treatments, and primary or acquired resistance remains a central clinical challenge^3,4^. This underscores an urgent need for robust, mechanistically informed biomarkers that can accurately identify responders and elucidate the underlying determinants of therapeutic success or failure^5–7^. Current predictive strategies, often relying on tumor mutational burden (TMB)^8^ or broad immune gene expression signatures^9,10^, frequently lack the resolution to capture the complex, dynamic interactions within the tumor microenvironment (TME) that dictate response to ICB^11,12^. For instance, while high TMB is associated with increased neoantigen load and T cell infiltration^13^, it is not universally predictive across all cancer types or patient cohorts, and many patients with high TMB still do not respond^14^. Similarly, broad immune signatures may indicate inflammation but fail to pinpoint the functional state or precise cellular subsets critical for effective anti-tumor immunity^15^. A deeper understanding of the core transcriptional programs that govern anti-tumor immunity is, therefore, critical for advancing patient stratification and developing more effective therapeutic interventions.

Previous efforts to identify ICB response biomarkers have primarily focused on downstream readouts of immune activation, such as interferon-gamma (IFN-γ) gene expression signatures^9^, T cell inflamed gene expression profiles^11^, and the quantification of tumor-infiltrating lymphocytes (TILs)^16^. While these markers have provided valuable insights, their ability to predict response remains limited due to their descriptive nature, often overlooking the upstream regulatory circuitry that orchestrates effective anti-tumor immune responses. The inherent heterogeneity of the TME, encompassing diverse immune cell subsets and their functional states, further complicates the identification of universally applicable predictive signatures^17^. Thus, there is a pressing need for a biomarker that delves deeper into the fundamental transcriptional control mechanisms driving immune engagement rather than merely reflecting its consequences.

In this study, we introduce IMIREG (immune microenvironment regulon signature), a novel 14-regulon transcriptional signature systematically inferred through the DoRothEA v0.11 resource^18^ and VIPER algorithm^19^, designed to predict clinical benefit from immunotherapy, with primary focus on anti-PD-1/PD-L1 therapies and additional evaluation across other immunotherapy modalities. Distinguishing our work from prior studies, IMIREG provides a unique mechanistic lens, moving beyond descriptive immune metrics to quantify the activity of master transcription factors (TFs) governing the anti-tumor immune response. Through extensive multi-cohort validation across 50 independent ICB clinical trial datasets and pan-cancer analyses, we demonstrate that IMIREG consistently and robustly identifies patients likely to respond to ICB, outperforming existing biomarkers. Mechanistically, we show that IMIREG defines a distinct, physiologically restricted immune-competent program, selectively enriched in functionally engaged yet checkpoint-restrained T cells and M1-polarized macrophages.

Our novel lineage-resolved framework further dissects the cellular drivers of IMIREG activity, revealing diverse immune archetypes within tumors. Integrating spatial transcriptomics to contextualize these findings and translate intricate spatial patterns into actionable biomarkers, this approach provided a novel, lineage-informed interpretation of immune organization, defining functional immune niches relevant to cancer progression and therapy response. Critically, we demonstrate a profound erosion of the most immunologically active phenotype during metastatic progression. Additionally, we establish IMIREG as a dynamic, real-time pharmacodynamic marker in triple-negative breast cancer (TNBC), correlating with early immune engagement and long-term pathological complete response (pCR).

Collectively, our findings position IMIREG as a powerful, mechanistically grounded biomarker that not only enhances patient stratification for ICB but also provides critical insights into the dynamic nature of anti-tumor immunity, paving the way for more precise and effective immunotherapy strategies.

## Results

### Discovery and conceptual framework of IMIREG, a transcriptional regulon signature of ICB sensitivity

**Fig. 1:**
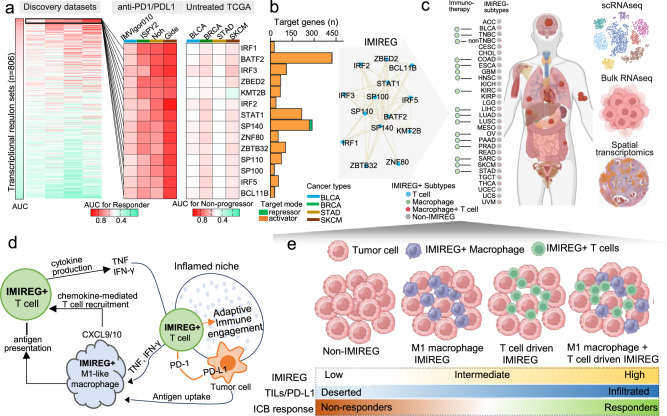
Discovery and conceptual framework of IMIREG, a transcriptional regulon signature of ICB sensitivity. **a** *Regulon screening for ICB-predictive activity*. *Left*: heatmap summarizing AUROC values for 806 inferred transcriptional regulons evaluated across four discovery ICB cohorts (IMvigor210/BLCA, I-SPY2/BRCA, Noh/STAD, and Gide/SKCM). *Middle*: AUROC values for the final 14 selected regulons. Regulons were selected if they consistently predicted clinical response across all discovery datasets with no trend toward resistance in any dataset, achieved an unpaired two-tailed t-test *p* < 0.05 in ≥50% of cohorts, and mean AUROC ≥ 0.69 across the four discovery datasets. *Right*: evaluation of these 14 regulons in matched untreated TCGA tumor types using disease progression endpoints, showing lack of consistent association with non-progression in the absence of ICB. **b** bar plot indicating the number of target genes in the corresponding DoRothEA regulon, partitioned by regulatory mode (activating vs. repressing edges). **c** Schematic overview of IMIREG+ subtype distributions across pan-cancer (T cell-driven, macrophage-driven, dual-driven, or non-IMIREG) and the multi-scale datasets used for downstream analyses (scRNA-seq, bulk RNA-seq, and spatial transcriptomics). **d** *Mechanistic hypothesis*. Conceptual model in which IMIREG+ T cells produce effector cytokines (TNF, IFN-γ) while engaging inhibitory checkpoints. T cell-derived IFN-γ activates IMIREG+ M1 macrophages to upregulate antigen presentation and secrete CXCL9/CXCL10, recruiting additional CXCR3+ T cells. This reciprocal signaling loop establishes a self-reinforcing inflammatory circuit primed for ICB response. **e** Cartoon depiction of four archetypal tumor immune states (non-IMIREG, macrophage-driven, T cell-driven, and dual-driven) aligned to an increasing continuum of IMIREG activity, immune infiltration/PD-L1 features, and probability of ICB response.

To identify transcriptional regulatory programs that predict benefit from ICB, we profiled the inferred activity of 806 transcriptional regulons across four discovery immunotherapy cohorts spanning distinct tumor types: IMvigor210 (BLCA)^20^, I-SPY2 (BRCA)^21^, Noh (STAD)^22^, and Gide (SKCM)^23^. Regulon activities were inferred from bulk tumor transcriptomes using DoRothEA/VIPER, which estimates TF activity from the coordinated expression of signed TF-target relationships (Fig. S1). Within each cohort, we quantified predictive performance for clinical benefit using ROC/AUROC and selected a convergent set of 14 regulons that reproducibly discriminated responders from nonresponders across the discovery compendium (Fig. 1a). To distinguish predictive from purely prognostic signals, we evaluated these same regulons in matched untreated TCGA tumor types using disease progression endpoints and observed no consistent association with non-progression in the absence of ICB (Fig. 1a, *right*), supporting ICB-context specificity rather than baseline prognosis. To examine the regulatory landscape of the 14 TF regulons, we quantified regulon size and target-gene overlap. Regulon sizes varied widely (28–425 target genes; Fig. 1b). SP100 and SP110 showed particularly high target sharing, and SP140 overlapped substantially with ZBED2, ZBTB32, and ZNF80 (Fig. S2), consistent with co-regulation or partial redundancy within IMIREG. In contrast, many TF pairs exhibited low overlap, supporting distinct, nonredundant regulatory contributions across the program.

We next derived a composite score, IMIREG (immune microenvironment regulon signature), by averaging the activity of these 14 regulons. Across discovery cohorts, IMIREG was consistently higher in responders, with AUROC of 0.83 (SKCM), 0.79 (STAD), 0.74 (BRCA) and 0.54 (BLCA) (Fig. S3a). Notably, even in BLCA, where discriminatory accuracy was modest, higher IMIREG was significantly associated with improved overall survival (HR = 0.66, *p* = 0.02), paralleling the survival benefit observed in melanoma (HR = 0.24, *p* = 0.01) (Fig. S3b). While these findings suggest IMIREG’s predictive value on overall survival following ICB therapy, survival data limitations for the STAD and BRCA cohorts constrain the generalizability in these specific groups.

Pan-cancer application of IMIREG across immunotherapy cohorts motivated a compartmental framework in which IMIREG can be T cell-driven, macrophage-driven, or dual-lineage, providing a conceptual basis for subtype resolution across cancers and data modalities (Fig. 1c). In this model, IMIREG-high T cells are hypothesized to couple immune activation with cytokine programs (e.g., TNF/IFN-γ) and checkpoint engagement, whereas IMIREG-high M1-like macrophages secrete CXCL9/CXCL10 and reinforce antigen-presentation programs; together, these programs create an inflamed niche permissive for adaptive immune activity and ICB responsiveness (Fig. 1d). These lineage-resolved IMIREG states therefore map onto tumor ecosystems spanning immune-desert/IMIREG-low tumors, intermediate single-lineage IMIREG states, and highly inflamed dual-lineage IMIREG tumors, with concordant increases in TIL/PD-L1, immune infiltration, and clinical benefit (Fig. 1e).

### IMIREG encodes an immune-competent program with physiological tissue restriction and exclusion from immune-privileged niches

**Fig. 2:**
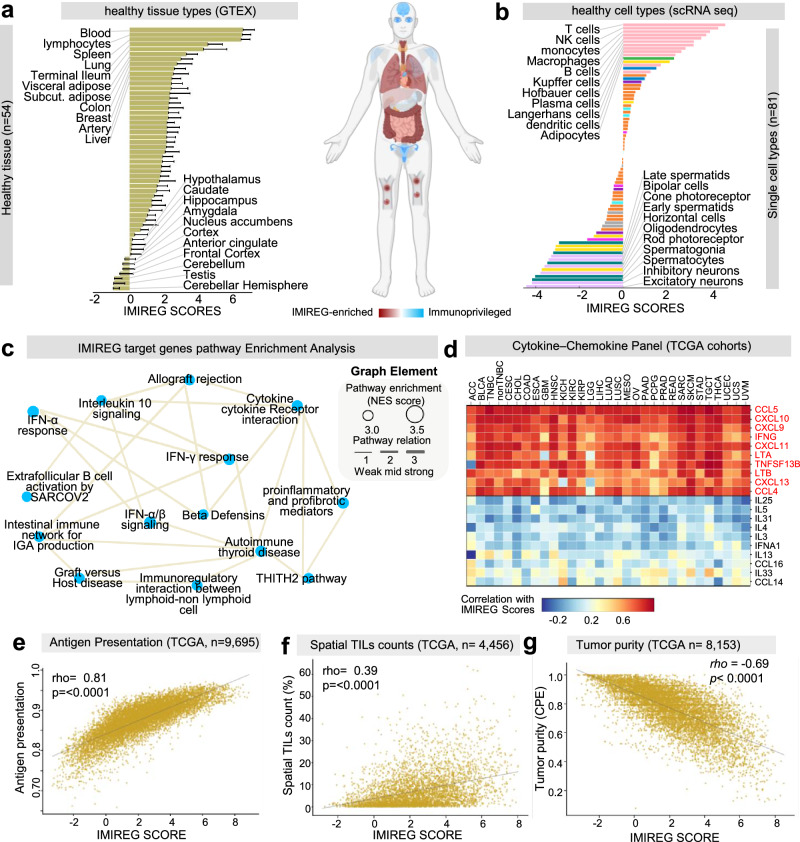
IMIREG defines a physiologically restricted, immune-competent transcriptional program. **a** Ranked bar plot of mean IMIREG scores across healthy GTEx tissues (*n* = 54); error bars indicate standard deviation across samples per tissue. Middle panel: a human body schematic highlights anatomical sites consistent with relative IMIREG enrichment versus immune-privileged compartments (color key). **b** Ranked mean IMIREG scores across healthy scRNA-seq cell types (*n* = 81), with high IMIREG in immune lineages (T cells, macrophages, NK cells, and B cells) and low IMIREG in immune-privileged/non-immune lineages (e.g., neurons, germ cells). **c** Pathway enrichment network for IMIREG-associated/target genes: nodes represent the top significantly enriched pathways after redundancy-aware disambiguation of CSEA results (Bonferroni-adjusted *p* = 0.01); node size is proportional to NES. Edges denote functional similarity between pathways computed from gene-set overlap using the precomputed Ochiai-based redundancy matrix, and edge thickness scales with association strength. **d** Heatmap of correlations between IMIREG scores and a curated cytokine-chemokine gene panel across TCGA cohorts (columns = cancer types; rows = genes). Scatterplots across TCGA tumors with fitted trend lines and Spearman rho and *p*-values: **e** antigen-presentation score, **f** spatial TIL (T-cell) count metric, and **g** tumor purity estimate. The per-tumor TIL and tumor purity metrics were used as provided by their respective TCGA source resources and analyzed as continuous variables; sample sizes differ across (**e**–**g**) because not all samples have available data for each metric. GTEx genotype-tissue expression, TCGA the Cancer Genome Atlas, scRNA-seq single-cell RNA sequencing, TILs tumor-infiltrating lymphocytes, NES normalized enrichment score, CSEA concept set enrichment analysis.

Our initial characterization of the IMIREG program sought to define its physiological relevance by examining its distribution across 54 healthy human tissues (GTEx) and 81 distinct cell types (scRNA-seq atlases). This analysis provided crucial insights into the normal immune contexts where IMIREG is naturally expressed and environments where it is actively excluded. Our findings revealed that IMIREG is most enriched in lymphoid-rich tissues, including blood, lymphocytes, spleen, and lung (Fig. 2a), all of which are typically engaged in immune surveillance and leukocyte trafficking. In stark contrast, brain regions (e.g., hypothalamus, cortex, cerebellum), testis, and retina-associated structures displayed the lowest IMIREG scores, aligning with known immune-privileged compartments.

This physiological pattern was further supported by IMIREG expression across 81 healthy cell types profiled in single-cell RNA-seq reference datasets (Fig. 2b). IMIREG scores were consistently highest in T cells, macrophages, monocytes, NK cells, and B cells, further validating its restriction to immune effector lineages. Notably, IMIREG was absent or negatively enriched in neurons, photoreceptors, germ cells, and other non-immune lineages, reinforcing its role as a lineage-specific immune signature. Together, these findings robustly support the notion that IMIREG represents an immune-competent transcriptional program with physiological restrictions to tissues primarily involved in immunological defense.

To mechanistically annotate the IMIREG regulons, we performed a pathway enrichment analysis of their transcriptional targets using our custom-developed IndepthPathway tool. The resulting network (Fig. 2c) revealed a coordinated enrichment of pathways including interferon-γ and -α responses, cytokine–cytokine receptor interaction, B cell activation and immunoglobulin production, IL-10 signaling, and autoimmune and graft-versus-host pathways (e.g., thyroid autoimmunity, intestinal IgA response). These pathways collectively reflect a transcriptional landscape consistent with active immune engagement rather than quiescent or suppressive immune states. The simultaneous enrichment of both pro-inflammatory (e.g., IFN-gamma) and regulatory signaling (e.g., IL-10) suggests that IMIREG operates within a tightly regulated immune feedback system. Collectively, these data position IMIREG as a physiologically grounded immune transcriptional program, selectively expressed in immune-responsive compartments and centered on IFN-driven immune activation, antigen response, and tissue-specific immune regulation.

### IMIREG is a hallmark of inflamed, immune-recognized tumors primed for checkpoint response

Building on its physiological characterization, we next examined the impact of IMIREG on the tumor microenvironment (TME) by estimating immune cell fractions using the CIBERSORT algorithm. Across most TCGA cancer types, IMIREG scores were most significantly and positively associated with key anti-tumor immune populations, including CD8+ T cells, M1 macrophages, NK cells, and neutrophils (*p* < 0.0001; Fig. S4), indicating its consistent enrichment in immune-inflamed tumors. We further assessed TME features associated with IMIREG expression. IMIREG correlated positively with a wide array of pro-inflammatory cytokines and chemokines (Fig. 2d), antigen presentation machinery (rho = 0.81, *p* < 0.0001; Fig. 2e), and TILs as measured by spatial histopathology (rho = 0.39, *p* < 0.0001; Fig. 2f). Conversely, it was negatively correlated with tumor purity (rho = −0.69, *p* < 0.0001; Fig. 2g), reinforcing its association with immune-rich tumor ecosystems.

To systematically define its immunological context, we profiled IMIREG against key immunoregulatory genes and metrics across TCGA. IMIREG showed robust positive correlations with both stimulatory and inhibitory checkpoint molecules, including PDCD1, CTLA4, TIGIT, LAG3, CD80, and CD86—across most tumor types (Fig. S5a). It was also positively associated with TCR and BCR Shannon diversity and richness, though not evenness, consistent with oligoclonal immune expansion (Fig. S5b). Consistent with its immune-recognition role, IMIREG was tightly linked to APM gene expression, including MHC class I/II molecules and B2M (Fig. S5c). Finally, IMIREG correlated positively with immunophenoscore (IPS) in all checkpoint contexts, with the strongest association under combined CTLA4 + PD1 blockade, and the weakest in checkpoint-negative tumors (Fig. S5d). Collectively, these findings indicate that IMIREG marks an inflamed, antigen-presenting tumor state primed for response to ICB.

### Tumors engage IMIREG through distinct immune compartments across the single-cell landscape

**Fig. 3:**
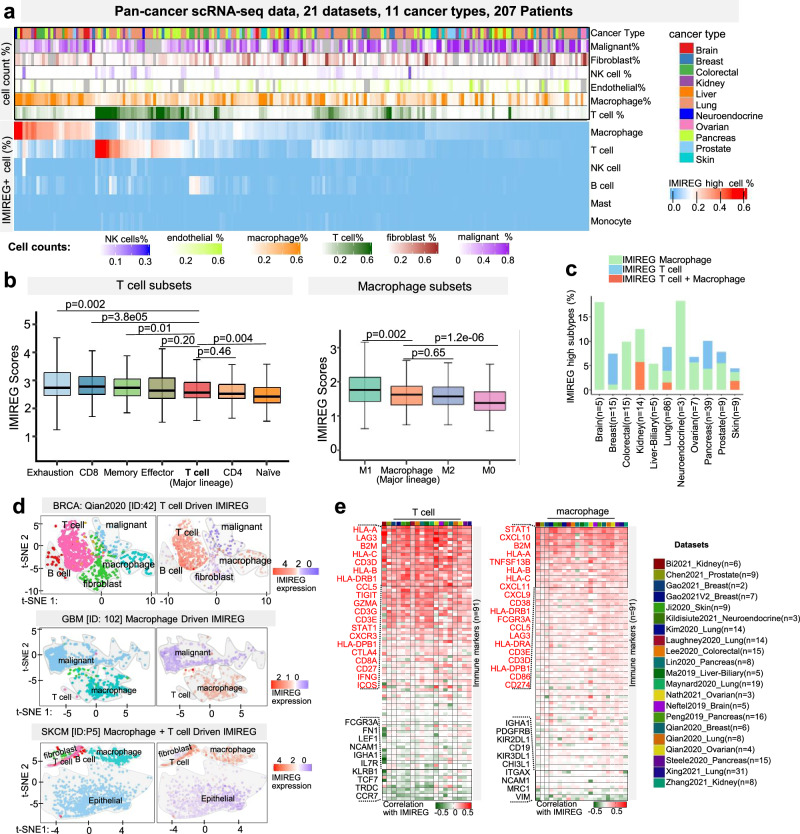
Pan-cancer single-cell landscape of IMIREG-high states. **a** Heatmap of patient-level global IMIREG frequency (GlobalFract) across major lineages, computed as IMIREG-high cells in a lineage/ total profiled cells per patient (all compartments); columns are annotated by cancer type and proportional abundance of major compartments (e.g., malignant, fibroblast/endothelial, macrophage, and T cell). **b** Box-and-whisker plots display the mean IMIREG expression scores for various macrophage and T-cell subsets. The center line of each box denotes the median, while the box bounds represent the interquartile range (IQR). *P*-values are based on two-sided Wilcoxon rank-sum tests, with each subset compared against its respective major lineage baseline (“macrophage” or “T_cell”). **c** Stacked bars show mean IMIREG subtype enrichment per cancer type, defined as the average % of total IMIREG-high cells within each subtype (IMIREG-high macrophages and/or T cell. Patients were first classified into subtypes based on a Z-score threshold (>0.5) of their IMIREG-global high fraction relative to their dataset. For each cancer type, subtype burden was computed by averaging the subtype-specific percent contributions across all patients, with non-enriched patients contributing zero. Bars are stacked to show the relative contribution of macrophage-driven, T-cell-driven, and dual-high IMIREG states. **d** t-SNE embeddings show compartment-specific IMIREG expression in representative tumors for each subtype (T cell-driven breast cancer, macrophage-driven glioblastoma, and dual-driven skin tumors). For each example, the left panel is colored by broad lineage to depict cellular architecture, and the right panel overlays the continuous IMIREG score on the same embedding. Embeddings were generated by PCA (top 20 principal components) followed by t-SNE (perplexity = 20, theta = 0.8, max_iter = 300; set.seed = 42) using the Rtsne package. **e** Heatmaps summarize Spearman correlation coefficients (rho) between cell-level IMIREG score and indicated immune markers, computed separately within T cells (left) and macrophages (right) for each dataset (columns; dataset identities and sample sizes listed at right). Correlations were computed only when ≥20 complete cell-level observations were available for a given marker.

To determine the cellular origin of IMIREG activity, we analyzed a pan-cancer scRNA-seq compendium comprising 21 datasets, spanning 11 cancer types and 207 patient tumors (Figs. 3a and S6a). Cells were assigned to major TME compartments using dataset-provided subtype annotations, and IMIREG activity was quantified using a regulon-scoring pipeline. Across all annotated cell types, IMIREG was almost exclusively enriched in T cells and macrophages, with minimal to no activity in fibroblasts, endothelial cells, malignant cells, or other immune populations (Fig. 3a). This lineage restriction was consistent across datasets and tumor types (Fig. S6a), indicating a conserved confinement of IMIREG to effector immune compartments.

To resolve IMIREG-high immune subpopulations, we performed marker-based and reference-guided subtyping of T cells and macrophages using Seurat module scoring. Within T cells, IMIREG signal was concentrated in functionally engaged states—including CD8, exhausted, memory and effector programs—and was reduced in naïve-like states (Fig. 3b). Within macrophages, IMIREG activity was primarily confined to M1-polarized pro-inflammatory macrophages, with lower levels in M2 and minimal signal in M0 (resting) macrophages, linking IMIREG to immune-stimulatory myeloid programs (Fig. 3b).

We next examined tumor-context dependence by stratifying IMIREG-high tumors according to the dominant immune compartment driving IMIREG burden (Fig. 3c). Macrophage-driven IMIREG was most prominent in relatively stroma-rich settings, including brain (glioblastoma), neuroendocrine, and liver–biliary tumors, and was also evident in kidney, ovarian, pancreatic, and colorectal cancers. In contrast, T cell-driven IMIREG was more frequent in immune-inflamed cancers, such as breast and lung, and was also observed at high frequency in pancreatic and prostate tumors, with a smaller contribution in subsets of ovarian tumors. Several cancer types—including kidney, skin, and subsets of lung-exhibited dual T cell- and macrophage-driven IMIREG patterns (Fig. 3c).

These lineage-specific patterns were visualized by t-SNE in representative tumors (Fig. 3d): IMIREG localized to the lymphoid compartment in a T cell-driven breast tumor, to the myeloid compartment in a macrophage-driven glioblastoma, and to both compartments in a dual-driven skin tumor. Collectively, these data establish IMIREG as a lineage- and state-restricted transcriptional program reproducibly activated in tumor-infiltrating T cells and macrophages, supporting its use for mechanistic subtyping of IMIREG+ tumors and for informing immune-context-specific strategies for immunotherapy prediction and immune-targeted intervention.

### IMIREG represents a signature of immune engagement in the face of inhibitory signals

To uncover the immune states driving IMIREG, we correlated its expression with a curated panel of 91 immune-related marker genes in single-cell RNA-seq datasets (Fig. 3e). In T cells, IMIREG strongly correlated with activation/trafficking (CD3D/E/G, CXCR3), effector/cytotoxic functions (CCL5, GZMA, IFNG, and CD8A), MHC class I presentation (HLA-A/B/C, B2M), and checkpoint/exhaustion markers (LAG3, TIGIT, CTLA4, and ICOS). This profile—characteristic of antigen-experienced, exhausted T cells—was confirmed in TCGA bulk RNA-seq data (Figs. S7 and S5a, b). In macrophages, IMIREG aligned with pro-inflammatory M1 polarization and antigen presentation (CD86, CXCL10, and HLA-DRA), while showing low to negative correlation with M2/tissue-repair (MRC1, CHI3L1) and stromal programs (PDGFRB, VIM) (Fig. 3e). Together, this pattern indicates a TME that is enriched in antigen-experienced, clonally expanded T cells that are functionally engaged yet restrained by inhibitory checkpoints

Expanding to TCGA bulk data, IMIREG positively associated with Th1-type chemokines and cytokines (CXCL9/10/11, CCL4/5, IFNG), supporting an effector-rich, pro-inflammatory TME (Fig. 2d). Conversely, it negatively correlated with Th2 and immunosuppressive cytokines (IL4/5/25/3/31, IFNA1), highlighting an exclusion of tissue-healing programs. Across additional cohorts, IMIREG consistently linked with TILs and inflamed phenotypes (Fig. S8a, b). Notably, IMIREG strongly correlated with CD8 expression in the IMmotion150 renal cancer cohort (rho = 0.64, *p* < 0.0001; Fig. S8c) and with PD-L1 expression across multiple immunotherapy trials (Fig. S8d).

Ultimately, these data position IMIREG as a mechanistically grounded biomarker of immune engagement under inhibitory constraint. Rather than marking immune-deserted or suppressive tumors, IMIREG-high tumors harbor active, IFN-γ-driven T cell and macrophage responses limited by checkpoint-mediated exhaustion. Consequently, IMIREG serves as a distinct mechanistic fingerprint for tumors primed for immune reactivation, offering a powerful tool to identify patients most likely to benefit from checkpoint blockade therapies.

### Lineage-resolved IMIREG subtypes reveal diminished immune engagement in metastatic cancer

**Fig. 4:**
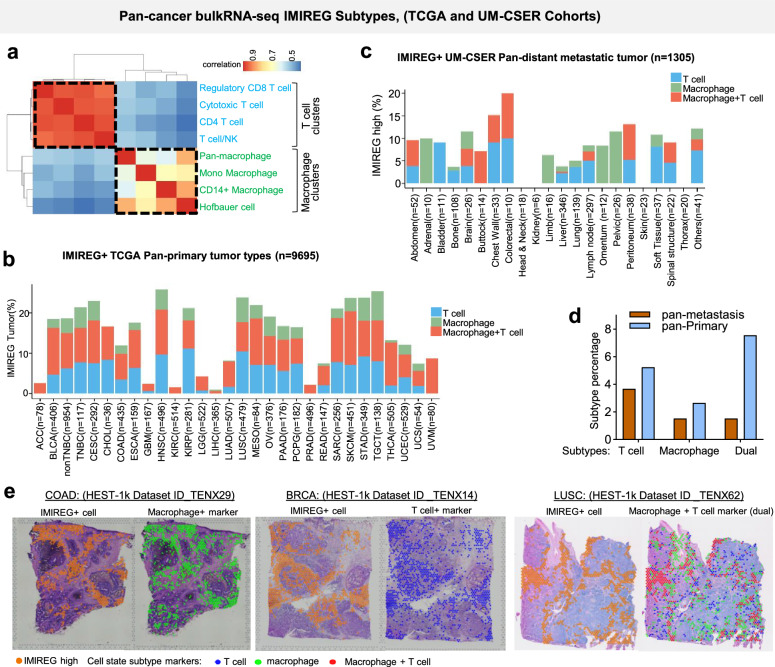
Lineage-resolved IMIREG subtypes reveal immune disengagement in metastasis and define the functional architecture of IMIREG-high tumors. **a** Heatmap showing pan-cancer correlations between bulk IMIREG and the finalized compartment-surrogate marker signatures used for subtyping (T-cell and macrophage panels). Values represent Spearman correlation coefficients, clustered to highlight co-association structure among T-cell-linked and macrophage-linked programs. **b** Prevalence of IMIREG-high subtypes across TCGA solid primary tumors (*n* = 9,695). Tumors were required to meet a global IMIREG-high prerequisite (IMIREG > median + MAD across solid tumors; MAD constant = 1). Among IMIREG-high tumors, subtype assignment was performed within each cancer type using cancer-type-specific z-scoring of the T-cell and macrophage composite driver scores (mean of validated marker signatures; driver-high defined as *z* > 1): IMIREG T cell, IMIREG macrophage, or IMIREG macrophage + T cell (dual). Bars show subtype frequencies by cancer type. **c** Prevalence of IMIREG-high subtypes across a pan-cancer cohort of distant metastatic tumors (UM-CSER; *n* = 1305), using the same global IMIREG-high prerequisite and cancer-type driver-high definitions as in (**b**). Bars show subtype frequencies by metastatic cancer category. **d** Bar plot showing the distribution of IMIREG+ tumor subtypes across pan-TCGA primary tumors and pan-distant metastatic tumors for comparative analysis. **e** Spatial transcriptomic validation of IMIREG localization in HEST-1k dataset: representative COAD (TENX29), LUSC (TENX62), and BRCA sample (TENX14) cases. Spot-level IMIREG scores are overlaid on H&E images, with corresponding overlays of T-cell-marker scores and macrophage-marker scores, illustrating spatial concordance between IMIREG-high regions and lineage-associated immune programs.

To further resolve the mechanistic diversity of IMIREG and pinpoint its cellular drivers, we developed a lineage-resolved classification framework based on immune cell state-specific gene signatures derived from our pan-cancer single-cell RNA-seq (scRNA-seq) dataset (*n* = 21). We correlated IMIREG expression with 1265 curated cell state gene sets, prioritizing T cell- and macrophage-specific signatures surpassing a predefined enrichment threshold (Fig. S9a). To ensure robustness, we refined these signatures by selecting those with strong correlation (Spearman rho > 0.5) to IMIREG in bulk RNA-seq data from the TCGA pan-cancer cohort (Fig. S9b). This dual approach—integrating single-cell specificity with bulk cohort reproducibility—identified lineage-specific immune programs driving IMIREG activation. Hierarchical clustering of these gene sets in TCGA data revealed two distinct modules: macrophage-associated and T cell-associated (Fig. 4a). Using these cell marker signatures, we stratified IMIREG-high tumors into three subtypes based on lineage-specific enrichment: T cell-driven, macrophage-driven, and dual-driven (T cell + macrophage). Interestingly, our subtyping assignment is robust against different strategies (Fig. S10a).

In the TCGA primary tumor cohort, 15.5% of tumors were IMIREG-high, with notable variation across cancer types (Fig. S10b). HNSC (26%), TGCT (25%), LUAD (24%), STAD (24%), SKCM (24%), CESC (23%), LUSC (22%), and TNBC (21%) showed the highest prevalence, reflecting their immune-rich microenvironments. Conversely, LGG (1%), GBM (2%), PRAD (2%), KICH (2%), and ACC (3%), were predominantly IMIREG-low, indicating immune-deserted niches. Among IMIREG-high tumors, while KIRC, LUAD, and STAD are mostly T-cell-driven IMIREG, the dual-driven subtype dominated in immune-infiltrated cancers like SKCM, BLCA, LUSC, and HNSC, suggesting coordinated T cell and macrophage activation (Fig. 4b). Immunologically quiescent tumors (e.g., LGG, GBM, and ACC) showed low subtype prevalence, underscoring their immune disengagement. This inter-patient heterogeneity in subtype distribution, even within histologically similar cancers, highlighted diverse tumor–immune interactions.

To assess whether these subtypes persist in advanced disease, we applied our stratification to metastatic tumors. In the UM-CSER pan-metastatic cohort (Figs. 4c and S10c), The proportion of IMIREG-high tumors dropped to 6.74%, with detectable levels in specific metastatic sites (e.g., colorectal: 20%; chest wall: 15.15%; peritoneum: 13.15%). Most immunologically active sites including head and neck, kidney, skin and thorax lacked IMIREG-high tumors in the metastatic settings (Fig. 4c). Strikingly, the dual-driven subtype, prevalent in primary tumors (7.6%), was nearly absent in metastases (1.5%, Fig. 4d). This reduction suggests an association between metastatic status and diminished coordinated T cell-macrophage programs, potentially driven by immune selection, immunosuppressive metastatic niches, or failure to sustain functional immune populations. Notably, this comparison is cross-sectional between independent primary (TCGA) and metastatic (UM-CSER) cohorts with differing cancer-type compositions; therefore, compositional differences and cohort-specific factors may partly contribute to the observed pattern. Representative histopathological images of TCGA tumors, illustrating the distinct TMEs associated with IMIREG subtypes (inflamed TME in IMIREG-high, deserted TME in IMIREG-low samples), are presented in Fig. S10d.

To further validate and spatially contextualize these IMIREG subtypes, we analyzed spatial transcriptomics data. This analysis revealed localized enrichment of IMIREG scores across tumor sections. Specifically, IMIREG-high (IMIREG+) spots exhibited clear spatial co-localization with high expression of macrophage and T cell marker gene sets (Fig. 4e). These distinct spatial patterns support the presence of macrophage-driven and T cell-driven IMIREG+ niches within the TME. This spatial mapping confirmed that IMIREG expression is not uniformly distributed but rather spatially restricted to immune-enriched areas of the TME, directly consistent with our scRNA-seq-based subtype classifications and validating the spatial localization and subtype heterogeneity of IMIREG+ cells in tumor tissue.

Altogether, these findings support a dynamic, context-dependent model of IMIREG regulation, wherein immune subtypes are sculpted by both tumor-intrinsic programs and the immune landscape. The near-complete loss of dual-driven IMIREG-high tumors in metastatic settings indicates that coordinated T cell-macrophage engagement progressively collapses during cancer dissemination. This lineage-informed framework integrates single-cell, bulk, and spatial transcriptomics to illuminate the mechanistic diversity of IMIREG and its utility as a biomarker for immune state and therapeutic stratification.

### IMIREG maintains high predictive accuracy across diverse immunotherapy trial datasets

**Fig. 5:**
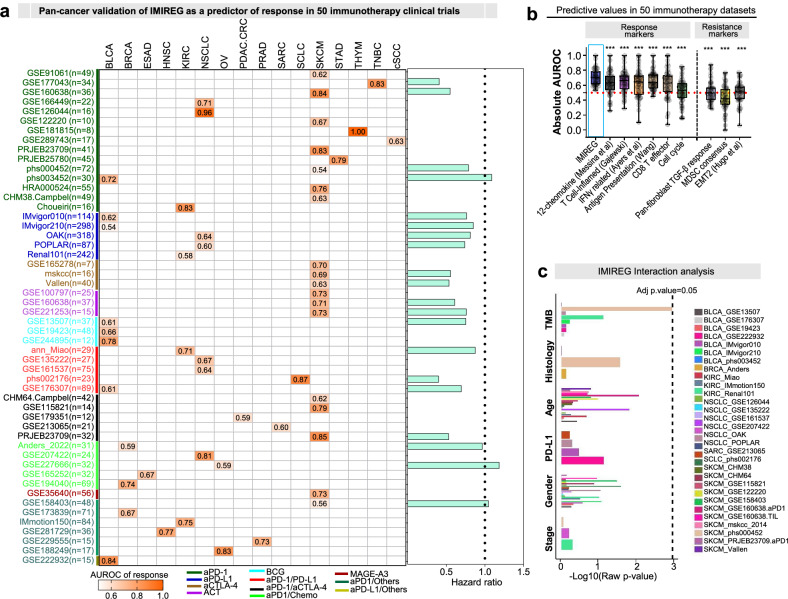
IMIREG maintains high predictive accuracy across diverse immunotherapy trial datasets and cancer types. **a** Left, heatmap summarizing IMIREG predictive accuracy across 50 independent immunotherapy (52 treatment arms) cohorts (4 discovery and 46 validation cohorts) spanning 16 cancer types. Each row represents a cohort (sample size shown as *n*), and the shaded cells report the AUROC for discriminating clinical benefit. Row label colors denote cancer type, and the color scale indicates increasing AUROC (higher = better discrimination). Right, for cohorts with time-to-event endpoints, the adjacent horizontal bars present hazard ratios (HRs) from time-to-event analyses, where HR < 1 denotes improved survival in IMIREG-high tumors. **b** Bar plot of AUROC distribution, highlighting IMIREG performance relative to established immune-response predictors (e.g., T cell-inflamed, CD8 effector, IFNG, antigen presentation) and resistance-linked programs (e.g., EMT, fibroblast/TGF-β). Statistical differences were assessed by paired Wilcoxon tests (paired across cohorts; *p < 0.05, **p < 0.01, ***p < 0.001). The red dashed line denotes AUROC = 0.5 (random prediction). Two cohorts contributed separate treatment arms evaluated independently, yielding 52 entries in (**a**, **b**). **c** Interaction analysis testing the stability of IMIREG’s predictive effect across major clinical covariates (e.g., age, sex, smoking, PD-L1, TMB, stage; cohort-specific availability). Bars summarize the significance of IMIREG×covariate interaction terms across cohorts; the dashed line denotes the multiple-testing-adjusted significance threshold used in the analysis.

To assess the clinical utility of IMIREG, we benchmarked its predictive performance across our compendium of 50 immunotherapy cohorts (52 treatment arms) spanning 16 cancer types. IMIREG achieved high predictive accuracy (mean AUROC = 0.71) consistently across the datasets, with AUROC values exceeding 0.80 in more than 10 datasets (Fig. 5a). In datasets with time-to-event outcomes, IMIREG-high tumors were associated with lower hazard ratios (Fig. 5a, *right panel*), indicating a trend toward improved outcomes in treated patients. Stratification by treatment modality revealed that IMIREG maintained predictive accuracy across diverse immunotherapy classes. Notably, performance was highest in anti-PD-1 cohorts (median AUROC = 0.76), consistent with the signature’s focus on checkpoint-restrained immune populations (Fig. S13a). When compared with multiple published response and resistance signatures, IMIREG consistently outperformed established immune-based predictors, including the T cell-inflamed, CD8 T effector, IFNG, and antigen processing and presentation signatures, and surpassed resistance-associated features, such as EMT, MDSC consensus signature and pan-fibroblast TGF-β response in predictive power (Fig. 5b). Overall, IMIREG maintained robust discriminatory performance across trials (mean AUROC = 0.71; sensitivity = 0.75; and specificity = 0.71; Supplementary Table 1). A random-effects meta-analysis (DerSimonian–Laird on logit-transformed AUROCs) across all 50 cohorts (52 treatment arms) confirmed low-to-moderate heterogeneity (*I*² = 26.9%, *τ*² = 0.06), supporting the stability of IMIREG’s predictive accuracy across independent datasets (Fig. S11). Notably, in treatment-naïve TCGA pan-cancer cohorts, IMIREG did not show consistent associations with overall survival or progression-related outcomes, indicating that its predictive utility is specific to the immunotherapy context rather than reflecting general prognosis (Fig. S12).

### IMIREG provides independent, nonredundant predictive power beyond immune cell enumeration

To determine whether IMIREG adds value beyond T-cell abundance, we performed multivariable logistic regression and found that IMIREG was the strongest predictor of response, significantly outperforming T-cell-based abundance proxies (e.g., CD8 T-effector and T-inflamed signatures) (Fig. S13b). We then assessed incremental predictive value using multivariable models that jointly included IMIREG and T cell infiltration-derived metrics. IMIREG remained a significant predictor and improved model performance relative to T-cell abundance metrics alone (Fig. S13c), supporting that it captures immune regulatory activation beyond simple cell quantification. Finally, interaction analyses across cohorts showed that IMIREG’s predictive performance is stable across key clinical covariates (age, sex, smoking status, PD-L1, TMB, and stage), indicating robustness across clinical contexts and diverse patient demographics (Fig. 5c).

### Temporal dynamics of IMIREG track early immune engagement and distinguish response trajectories in TNBC immunotherapy

**Fig. 6:**
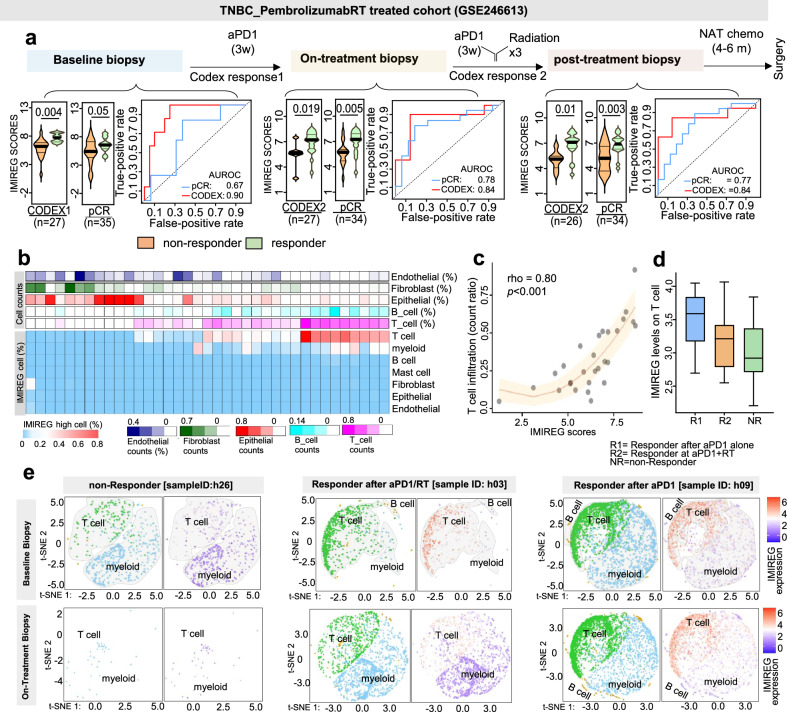
Temporal IMIREG dynamics mark early immune engagement and associate with response patterns in TNBC. **a** Violin plot shows longitudinal IMIREG scores in biopsies collected at baseline, post-cycle 1 pembrolizumab, and post-pembrolizumab + radiotherapy, stratified by CODEX-defined response trajectories and by pCR at surgery; ROC curves summarize discrimination at each timepoint. In the study, CODEX-derived spatial “district” features were used to assign response trajectory groups (R1, R2, and NR) via unsupervised clustering of tumors based on their longitudinal spatial patterns. **b** Heatmaps from single-cell RNA-seq of baseline biopsies indicate that IMIREG-high cells are predominantly T cells, with moderate contributions from myeloid populations. **c** Scatter plot of Spearman correlation between IMIREG scores and T cell infiltration. **d** Boxplot stratification showing T-cell IMIREG levels are highest in tumors assigned to an early responder-like trajectory, intermediate in delayed responder-like trajectory tumors (maximal engagement emerging after addition of RT), and lowest in nonresponder-like tumors; pCR status is shown as the surgical endpoint. **e** Single-cell t-SNE maps of IMIREG activity in paired baseline and on-treatment biopsies (post-cycle 1 pembrolizumab; pre-RT), stratified by the response trajectory labels used in this analysis. Representative patients include a nonresponder-like tumor (h26), an early responder-like trajectory tumor (h09), and a delayed responder-like trajectory tumor (h03). For each patient and timepoint, cells are colored by major immune lineages and separately by IMIREG signal intensity. Sample sizes vary across timepoints and outcome stratifications because CODEX trajectory and pCR represent independent classifications with different numbers of evaluable patients; all available samples per timepoint from GSE246613 were included.

To characterize the clinical dynamics of IMIREG during ICB, we analyzed longitudinal single-cell RNA-seq data from TNBC patients treated on a pembrolizumab-based neoadjuvant regimen with focal radiotherapy delivered during the second cycle (GSE246613), leveraging biopsies collected at baseline, after one pembrolizumab cycle, and after pembrolizumab + radiotherapy (pre-chemotherapy) (Fig. 6a–e).

We evaluated IMIREG against two complementary outcome labels: (i) CODEX (Co-detection by indEXing)-derived response trajectory groups (early responder-like, late responder-like, and nonresponder-like trajectories) defined by spatial immune remodeling patterns, and (ii) pathologic response at curative-intent surgery, where pCR is defined as the absence of invasive disease in the breast and lymph nodes (Fig. 6a). Across all timepoints, IMIREG scores were significantly higher in tumors assigned to responder-like CODEX trajectories versus nonresponder-like trajectories, achieving AUROCs of 0.90 (baseline), 0.84 (post-cycle 1), and 0.84 (post pembrolizumab + radiotherapy) for distinguishing these trajectory-defined response patterns (Fig. 6a). In parallel, IMIREG scores from baseline, post-cycle 1, and post-combination biopsies predicted pCR status at surgery with AUROCs of 0.67, 0.78, and 0.77, respectively (Fig. 6a), supporting IMIREG as an early biomarker associated with downstream pathologic outcome in this regimen. Single-cell analyses of baseline biopsies indicated that IMIREG was enriched predominantly in T cells, with a smaller contribution from myeloid populations (Fig. 6b), and IMIREG strongly correlated with T cell infiltration across samples (rho = 0.80, *p* < 0.001; Fig. 6c). Stratified by response trajectory, T cells from early responder-like tumors (immune engagement evident after pembrolizumab alone) exhibited the highest IMIREG levels, followed by late responder-like tumors (maximal immune engagement emerging after pembrolizumab + radiotherapy), whereas nonresponder-like tumors showed minimal IMIREG expression (Fig. 6d).

To further localize IMIREG and track its dynamics, we visualized single-cell embeddings (t-SNE) from baseline and post-cycle 1 pembrolizumab biopsies (pre-RT), using representative patients and the outcome labels applied here (Fig. 6e). At baseline, the nonresponder (h26; nonresponder-like trajectory and/or non-pCR) showed negligible IMIREG in T cells and myeloid cells, consistent with limited immune engagement. In contrast, responders (h03, h09) exhibited moderate-to-high IMIREG at baseline, strongest in T cells with additional myeloid signal, consistent with a pre-existing immune-active state. After one pembrolizumab cycle, this divergence sharpened: h26 remained IMIREG-low, whereas responders showed marked IMIREG upregulation—predominantly in T cells with robust induction in myeloid cells as well. Notably, the early responder-like case (h09; immune engagement after pembrolizumab alone, prior to RT) displayed broader and higher T cell IMIREG than the delayed responder-like case (h03; maximal engagement emerging later with RT in the treatment course).

Together, these data establish IMIREG as a dynamic, lineage-enriched marker of immune competence in TNBC: enriched in T cells (with inducible myeloid expression), graded by response intensity, and associated with both CODEX-defined response trajectories and downstream pCR at surgery, supporting its use as an early pharmacodynamic biomarker for treatment stratification.

### IMIREG-high tumors are characterized by an immune-enriched non-fibrotic TME in TNBC

**Fig. 7:**
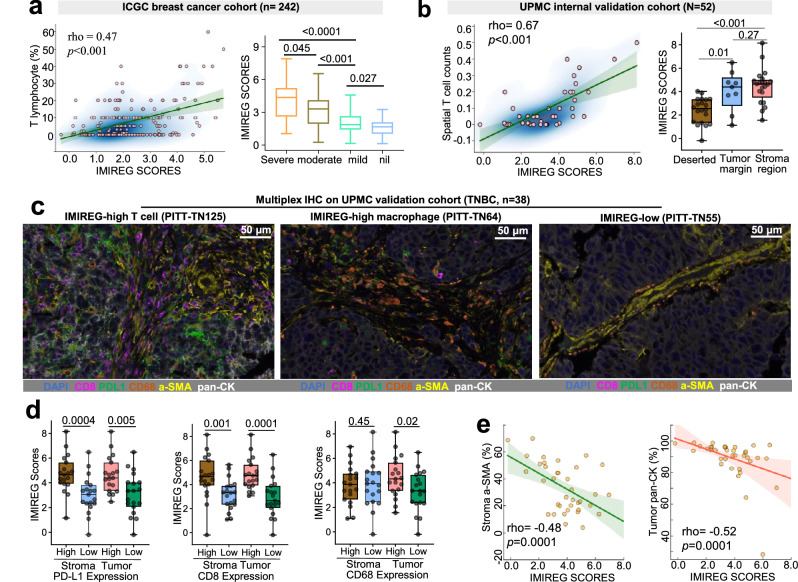
IMIREG-high tumors define an inflamed, non-fibrotic TME in TNBC. **a** Scatter plot shows a positive association between IMIREG scores and pathologist-assessed tumor-infiltrating lymphocyte (TIL) percentages in the ICGC breast cancer cohort. Adjacent bar plots depict IMIREG scores across pathological immune phenotypes in the TME (severe, moderate, mild, and nil). **b** Scatter plots demonstrate a strong positive association between IMIREG scores and spatial T-cell infiltration in the UPMC validation cohort. Box-and-whisker plots further show that IMIREG expression is higher in stromal regions relative to tumor margins and immune-deserted zones (center line = median; box = IQR; whiskers = min–max). **c** Representative multiplex immunohistochemistry (mIHC) indicates that IMIREG-high tumors exhibit T cell- and macrophage-enriched microenvironments with elevated PD-L1 and CD8 expression, whereas IMIREG-low tumors show sparse immune infiltration consistent with an immune-desert phenotype. Scale bars, 50 μm. **d** Box-and-whisker plots show IMIREG expression stratified by multiplex-defined immune-profile status (PD-L1, CD8, and CD68; high vs low). Center line = median; box = IQR; whiskers = min–max; points = individual samples. **e** Scatter plots show that IMIREG scores are inversely associated with stromal fibrosis (α-SMA%) and epithelial burden (pan-CK%), consistent with reduced non-immune content in IMIREG-high tumors. All correlations were computed using Spearman’s rank correlation (two-tailed). Lines indicate linear regression fits with 95% confidence intervals. Different sample sizes across modalities reflect archival tissue availability and assay-specific technical evaluability (RNA-seq *n* = 67; H&E *n* = 52; multiplex IHC *n* = 38).

We next investigated the association between IMIREG expression and the immune landscape in a large cohort of 242 breast cancer patients from the ICGC breast cancer dataset^24^. This analysis demonstrated a strong positive correlation between IMIREG scores and T cell infiltration, as well as inflamed immune phenotypes (Fig. 7a). To further validate these findings, we examined our independent in-house cohort of 67 TNBC patients from UPMC Hillman Cancer Center, utilizing bulk RNA sequencing and histopathological assessment of TILs. Our cohort confirmed the association between IMIREG scores and immune activity. Spatial immune profiling revealed a robust positive correlation between IMIREG scores and spatial T cell density (rho = 0.67), with elevated scores in stroma-rich regions compared to immune-deserted or tumor-margin areas (Fig. 7b). CIBERSORT-based immune TME deconvolution further identified strong positive correlations between IMIREG expression and M1 macrophages, CD8+ T cells, and memory CD4+ T cells (Fig. S14). Multiplex immunohistochemistry validated a T cell- and macrophage-enriched TME in IMIREG-high tumors, characterized by increased PD-L1 and CD8 expression in both stromal and tumor compartments (Fig. 7c, d), whereas IMIREG-low tumors exhibited an immune-deserted TME. Additionally, IMIREG scores showed an inverse association with stromal α-SMA and tumor pan-CK area (Fig. 7e), suggesting reduced stromal fibrosis and epithelial content in immune-inflamed tumors. Collectively, these results establish IMIREG as a spatially and transcriptionally validated predictor of immune activation in TNBC. Overall, these findings comprehensively characterize IMIREG as a robust and mechanistically grounded biomarker of immune engagement and therapeutic responsiveness, providing a foundation for its clinical translation across diverse cancer contexts.

## Discussion

The transformative potential of ICB in cancer therapy is increasingly constrained by the substantial proportion of patients who fail to respond, highlighting an urgent unmet need for robust, mechanistic biomarkers^25,26^. Current clinical predictors, while useful, often fall short in comprehensively capturing the complex interplay within the TME that dictates ICB efficacy^25,27^. Building on our earlier work with intragenic-rearrangement burden and tumor-associated antigen load—metrics that successfully identify responders even in low-TMB, PD-L1-negative tumors^28,29^—we now introduce IMIREG, a novel 14-regulon transcriptional signature derived from a systems-biology approach, which powerfully predicts clinical benefit from ICB across a diverse range of cancer types. Our findings position IMIREG as a dynamic, predictive biomarker that not only outperforms established signatures but also provides unprecedented mechanistic insight into a distinct, functionally engaged yet checkpoint-restrained immune state driving anti-tumor responses and their evolution during cancer progression.

A key strength of IMIREG lies in its mechanistic foundation. By integrating DoRothEA and VIPER algorithms^18,19^, we moved beyond static gene expression levels to infer core transcriptional regulatory programs. This approach, which quantifies TF activity by integrating target gene expression and their inferred regulatory modes, allows us to dissect the upstream regulatory circuitry governing immune responses rather than merely observing their downstream consequences. This approach revealed that IMIREG activity is predominantly enriched in lymphoid-rich tissues and critical immune effector cells, notably T cells and macrophages. Our physiological mapping across healthy tissues and single-cell atlases^30^ rigorously established IMIREG as a lineage-specific signature, almost exclusively confined to T cells and M1-polarized macrophages within the TME. This cellular restriction is pivotal, distinguishing IMIREG from broad inflammatory signatures and highlighting its direct association with anti-tumor immune operatives^31^. Specifically, within T cells, IMIREG is enriched in effector/exhausted CD8+ T cells, suggesting its role in functionally active, antigen-experienced states rather than naïve T cells. In the macrophage compartment, IMIREG expression is largely confined to M1-polarized, pro-inflammatory macrophages, indicating a link with immune-stimulatory programs necessary for effective antigen presentation and cytokine secretion.

Mechanistically, IMIREG-high tumors exhibit a transcriptionally inflamed TME, distinguished by elevated expression of pro-inflammatory cytokines and chemokines (e.g., CXCL9/10, IFN-γ), robust antigen presentation machinery (APM), and high infiltration of TILs. Pathway enrichment of IMIREG regulon target genes revealed coordinated activation of interferon-α and -γ responses, cytokine–cytokine receptor interactions, B cell activation and immunoglobulin production, and IL-10 signaling. Notably, IRF1 and IRF3—two core TFs in IMIREG—have been shown to promote antitumor CD8+ T-cell responses by reinforcing interferon-driven programs that support T-cell priming, trafficking, and effector function^32,33^, whereas IRF5 exerts antitumor activity largely by programming inflammatory (M1-like) macrophage states^34^. Together, these observations support IMIREG as a mechanistic readout of transcriptional programs that govern anti-tumor immunity. These findings reinforce that IMIREG reflects a state in which the immune system is actively recognizing and engaging tumor antigens, mediated by an orchestrated network of pro-inflammatory TFs. Furthermore, the concurrent enrichment of both effector (e.g., IFN-γ) and regulatory (e.g., IL-10) pathways suggests that IMIREG captures a dynamically regulated immune state, balancing immune activation with feedback control to preserve anti-tumor efficacy while limiting immune-mediated toxicity, a concept well-supported by studies on cytokine pleiotropy^35^.

Crucially, IMIREG expression correlates positively with key co-stimulatory and co-inhibitory checkpoint molecules (e.g., PD-1, CTLA-4, LAG-3, TIGIT, CD80, and CD86), suggesting its enrichment in immune compartments that are actively responding to tumor antigens but are simultaneously under significant inhibitory restraint^36^. This “immune-engaged but restrained” phenotype is a hallmark of ICB-sensitive tumors, providing a tangible mechanistic link between IMIREG and favorable therapeutic outcomes. The T cells captured by IMIREG are not merely present but are functionally active, having undergone antigen recognition and clonal expansion, yet they are simultaneously undergoing a process of checkpoint-mediated exhaustion or TME-driven dysfunction. By identifying this precise state, IMIREG not only reflects immunotherapy sensitivity but also serves as a mechanistic fingerprint of tumors primed for immune reactivation, offering a powerful tool for identifying patients most likely to benefit from checkpoint blockade therapies. The strong correlation with Immunophenoscore (IPS), particularly under combined CTLA-4/PD-1 blockade, further reinforces IMIREG’s capacity to identify tumors primed for therapeutic immune reactivation, where overcoming multiple inhibitory pathways is key.

Our lineage-resolved classification framework represents a significant advance in dissecting inter-tumoral immune heterogeneity. By categorizing IMIREG-high tumors into T cell-driven, macrophage-driven, and dual-driven archetypes, we revealed a spectrum of immune engagement. The observation that the dual-driven subtype, indicative of coordinated T cell and macrophage activation, is largely diminished in metastatic settings is particularly striking. This suggests a profound immunological erosion during metastatic progression, likely due to a combination of selective pressures favoring immune-evasive tumor cells, the establishment of immunosuppressive metastatic niches with distinct microenvironmental factors (e.g., in the liver or brain) that lead to immune cell functional impairment or exclusion, and the inability to sustain a robust multi-lineage immune response given the systemic burden of advanced disease or specific metabolic adaptations within metastatic tumor cells. This finding has critical implications for understanding ICB resistance in advanced disease and may guide strategies for restoring effective anti-tumor immunity in metastatic sites, potentially through therapies that specifically target these suppressive mechanisms or reinforce dual-lineage immune engagement.

The robust and consistent predictive accuracy of IMIREG across 50 independent immunotherapy cohorts (52 treatment arms), outperforming established biomarkers, such as IFN-γ gene expression signatures^9^, T cell-inflamed gene expression profiles^11^ and antigen presentation signature, underscores its clinical utility. Its predictive, rather than prognostic, nature ensures specificity for ICB response, avoiding confounding by general disease aggressiveness. This distinction is critical for patient stratification, ensuring that patients receive ICB based on their likelihood of response to the specific therapy, rather than their general disease trajectory. Furthermore, the dynamic tracking of IMIREG in longitudinal TNBC biopsies demonstrates its potential as a real-time pharmacodynamic marker. The increased baseline and on-treatment IMIREG levels in responders, particularly within T cells, highlight its sensitivity to early immune engagement and its capacity to predict both immediate and pCRs. This suggests that IMIREG can serve as a valuable tool for early assessment of treatment efficacy and for guiding adaptive treatment strategies, such as dose adjustments or combination therapy initiation, based on observed immune activation. The strong spatial correlation of IMIREG with T cell infiltration and its inverse association with stromal fibrosis (α-SMA) and epithelial content (pan-CK) in TNBC further validates its connection to an immune-permissive, anti-TME. This mechanistic link indicates that IMIREG-high tumors are characterized by a less dense, less immunosuppressive stroma, allowing for better immune cell infiltration and function^37^. Reduced fibrosis, for example, can facilitate leukocyte trafficking and nutrient exchange, thereby fostering an environment conducive to effective anti-tumor immunity^38^.

In conclusion, IMIREG provides a novel, mechanistically grounded biomarker that extends beyond enumerating immune components to measuring the core regulatory circuitry driving anti-tumor immune responses. This signature significantly enhances patient stratification for ICB, offers a powerful framework for understanding the dynamic, multi-lineage nature of immune engagement and its collapse in advanced cancer, and paves the way for more refined immunotherapy strategies and combination approaches.

Despite the comprehensive nature of our study, certain limitations warrant consideration. While IMIREG demonstrates broad predictive utility, the precise contribution of each of the 14 regulons may vary across different cancer types or treatment regimens, meriting further investigation into context-specific regulatory network dynamics. The observed reduction of the dual-driven IMIREG phenotype in metastasis warrants deeper mechanistic studies to elucidate the specific molecular and cellular mechanisms driving this immune collapse, potentially informing strategies to reverse this state. Future research will focus on prospective clinical validation of IMIREG in larger cohorts, exploring its predictive capacity in combination with other therapeutic modalities, and investigating the therapeutic targeting of specific IMIREG-associated regulons to re-establish robust anti-tumor immunity in refractory or advanced disease settings.

## Methods

### Data sources and preprocessing

We assembled a multi-platform, multi-cohort dataset to evaluate the immunotherapy-sensitivity regulon signature (IMIREG). The overall workflow and data types are summarized in Supplementary Fig. S1. Normal tissue single-cell RNA-seq data spanning 81 cell types across 31 tissues were obtained from the Human Protein Atlas (HPA, RRID:SCR_006710)^30^. Tumor-derived single-cell RNA-seq data (21 datasets across 11 cancer types) were retrieved from the Curated Cancer Cell Atlas (3CA)^39^. The normal tissue bulk transcriptomes for 54 tissue types from the genotype-tissue expression (GTEx) project were obtained via HPA^30^. The International Cancer Genome Consortium (ICGC) breast cancer dataset (*n* = 242) was downloaded from the ICGC data portal^24^. Bulk RNA-seq from 1305 metastatic tumors was retrieved from dbGaP (accession phs000673.v4.p1). H&E whole-slide images and raw spatial transcriptomics data were downloaded from the HEST-1k repository^40^. Immunotherapy clinical trial datasets spanning multiple cancer types were curated from repositories including GEO (RRID:SCR_005012)^41^, EGA (RRID:SCR_004944), and dbGaP (RRID:SCR_002709)^42^. For each immunotherapy cohort, we obtained (i) an expression matrix (RNA-seq or microarray) and (ii) a clinical response label. A complete cohort inventory (accession, platform, cancer type, treatment, sample size, and response definition) is provided in Supplementary Table 1.

#### TCGA dataset usage and scope

Bulk RNA-seq and associated clinical data for The Cancer Genome Atlas (TCGA) pan-cancer cohort were accessed through the UCSC Xena portal^43^. TCGA pan-cancer were used as an untreated reference for prognosis-oriented analyses and for mechanistic correlates, including immune infiltration, antigen presentation, and tumor purity; unless otherwise specified in the text or figure legends, analyses included all available TCGA tumor types. Spatial histopathology estimates of TILs for TCGA were obtained from Saltz et al.^44^. Immunophenoscore (IPS) components, and adaptive immune receptor repertoire metrics (BCR/TCR Shannon, richness, and evenness) were obtained from Bagaev et al.^45^. Consensus purity estimates (CPE) were obtained from Aran et al.^46^.

### Bulk expression standardization, processing, and harmonization

Expression data were retrieved as unlogged TPM for RNA-seq whenever available. When only raw counts (HTSeq/featureCounts) were provided, counts were converted to TPM using GENCODE v38 gene lengths by first computing reads per kilobase (RPK) as counts divided by gene length (kb), and then computing TPM (TPM = RPK/ΣRPK × 10^6^). When a cohort provided log-transformed TPM, values were converted back to unlogged TPM only if the exact transformation was explicitly specified (e.g., log2(TPM + 1)); otherwise, expression values were retained at the provided scale, and all downstream scoring was performed within-cohort. Microarray datasets were retained at their native normalized scale. All expression matrices were mapped to HGNC gene symbols using either the NCBI GRCh38.p13 annotation table (for NCBI-hosted matrices) or GENCODE v38 mappings (for count-derived TPM). When multiple features mapped to the same HGNC symbol, duplicates were collapsed by taking the mean expression across duplicated entries.

### Cohort-level analysis strategy

To minimize batch artifacts introduced by platform- and study-specific effects, all downstream computations used a cohort-wise workflow: regulon scoring, biomarker scoring, and ROC/AUROC analyses were performed independently within each cohort using that cohort’s processed expression matrix and clinical labels. Expression matrices were not merged across cohorts prior to ROC/AUROC. Cross-cohort performance was summarized by comparing AUROC values across cohorts.

### Clinical cohort and tissue procurement

This study was performed in accordance with the Declaration of Helsinki, and the study protocol was approved by the University of Pittsburgh Institutional Review Board (IRB) under Honest Broker approval number HB015. Formalin-fixed paraffin-embedded (FFPE) tissue blocks from 67 female patients diagnosed with TNBC between September 1991 and May 2016 at the UPMC Hillman Cancer Center were procured through the Pitt Biospecimen Core at the University of Pittsburgh. All samples were deidentified and obtained through a certified honest broker, with no direct patient contact or recruitment involved. The requirement for informed consent was waived by the University of Pittsburgh Institutional Review Board under the honest broker protocol, as only pre-existing deidentified archival specimens were analyzed without any patient interaction, and no participant compensation was applicable. Demographic and clinical characteristics are detailed in Supplementary Table 2.

All 67 cases had RNA-seq data available for IMIREG quantification. Due to section quality and tissue availability constraints inherent to archival FFPE material, evaluable slides for spatial histopathology (H&E-based TIL scoring) were available for a subset of tumors (*n* = 52). A further subset had sufficient remaining tissue and passed technical quality control for multiplex immunohistochemistry (opal platform; staining quality and spectral unmixing), yielding *n* = 38 for multiplex IHC analyses. Sex and gender were not incorporated as variables in the study design, given that TNBC occurs in female patients and no male cases were represented in this archival cohort, rendering sex-disaggregated analysis inapplicable.

### Histopathological analysis

Tissue sections were reviewed by a board-certified pathologist (Dr. Rohit Bhargava), who was blinded to transcriptomic data. Immune infiltration was quantified using hematoxylin and eosin (H&E) staining, scoring TILs within tumor, stromal, and combined compartments according to standardized guidelines.

### Multiplex immunohistochemistry (IHC)

Multiplex IHC was conducted using the Opal 6-Plex Detection Kit (Akoya Biosciences; NEL871001KT) at the Translational Pathology Imaging Lab, UPMC. Antibody optimization and panel assembly followed the Opal Assay Development Guide. Automated staining was executed on the Leica Bond RX system. Whole-slide imaging and spectral unmixing were performed with the Akoya PhenoImager HT and InForm® software (v2.8). Primary antibody details are provided in Supplementary Table 3.

### Bulk RNA-seq library preparation and sequencing

RNA was extracted from FFPE samples using TRIzol reagent. Libraries were prepared by poly(A)-selection, followed by cDNA synthesis, end-repair, adapter ligation, and amplification. Library QC included bioanalyzer-based size profiling and qubit quantification. Paired-end 150 bp reads were generated using the Illumina NovaSeq platform by Novogene Co., Ltd.

### Regulon activity inference using DoRothEA

A compendium of 806 human TFs, each linked to a regulon of ≥25 target genes, was accessed via the DoRothEA v0.11 R package (RRID:SCR_027126)^18^, which integrates multiple lines of evidence (including peaks, binding site motifs, and gene expression interactions) to define high-confidence TF-target relationships. Only regulons with ≥25 target genes were retained for analysis. The VIPER algorithm^19^, as implemented in the DoRothEA package, was used to infer TF activity scores based on signed gene-target relationships, accounting for activation versus repression^18^. Positive scores reflect inferred protein activation, and negative values indicate repression. The IMIREG score was computed by averaging, per sample, the VIPER activity scores of the 14 regulons that consistently predicted clinical response across all discovery datasets with no trend toward resistance in any dataset, achieved an unpaired two-tailed t-test *p* < 0.05 in ≥50% of cohorts, and mean AUROC ≥ 0.69 across the four discovery datasets. Target genes for the IMIREG regulons are provided in Supplementary Table 4.

### Immune cell deconvolution and computation of cell type-specific signatures

Transcriptomic datasets were normalized and processed for immune deconvolution using CIBERSORT (v1.04, RRID:SCR_016955)^47^ to estimate immune cell fractions. Immune pathway scores and cell type-specific signature scores were computed using curated gene sets from MSigDB (RRID:SCR_016863)^48^ and PanglaoDB (RRID:SCR_022580)^49^. Detailed information of the cell state signatures is provided in Supplementary Table 5. Analyses were restricted to cell markers with at least 5 gene set members. Gene set enrichment analyses were performed using the concept signature enrichment analysis^50^ algorithm implemented in the IndepthPathway tool^51^, with enrichment significance set at *p* < 0.05. Pathway networks were visualized using the “igraph” R package (v1.2.4.2).

### Single-cell preprocessing and lineage cell-state annotation

All scRNA-seq datasets were processed in R using a standardized workflow. Cells were retained if they expressed >1500 genes and had <25% mitochondrial reads. Count matrices were normalized to counts-per-million (CPM), and CPM expression was used to compute per-cell IMIREG scores. Cell identities were assigned using a three-tier strategy. Broad lineages were taken directly from the original study metadata to preserve published labeling conventions^39^. Within these lineages, functional sub-states were resolved by reference-based module scoring in Seurat (v 5.4.0)^52^, which quantifies relative signature enrichment against background gene sets to mitigate depth and dropout effects. T-cell states were defined using human reference signatures^53,54^, and macrophage polarization states (M1, M2) were defined using validated pan-cancer myeloid atlases^55,56^ (Supplementary Table 6). Following established signature-gating protocols^57,58^, a module-score threshold of 0.2 was applied to enforce dominant program assignment: macrophages below 0.2 threshold for both M1 and M2 were labeled M0 (resting).

#### Pan-cancer scRNA-seq IMIREG landscape, burden, and correlates

Within each dataset, cells were designated IMIREG-high if their IMIREG score exceeded a dataset-specific cutoff defined as median(IMIREG) + 1 × MAD(IMIREG). Dataset-specific thresholds were used to account for technical variation across single-cell platforms and processing pipelines. Samples were retained only if they contained ≥100 total cells and included both macrophage and T-cell compartments. For each sample and cell type, the IMIREG-high ratio (global frequency) was calculated as the number of IMIREG-high cells in that cell type divided by the total number of profiled cells in the sample across all cell types. These GlobalFract values were visualized using ComplexHeatmap (v2.26.0) with annotations indicating major lineage abundances per patient. In parallel, cell-type-specific mean IMIREG activity was computed per patient as the average cell-level IMIREG score within macrophage or T-cell subsets and displayed as stacked ComplexHeatmap panels. To quantify subtype burden, macrophage and T-cell GlobalFract values were jointly Z-score standardized within each dataset, and patients were classified as IMIREG macrophage, IMIREG T cell, or dual IMIREG if the corresponding lineage Z-score exceeded 0.5; mean subtype burden per cancer type was reported as the average percent of total profiled cells contributed by the corresponding lineage(s) across all patients, assigning zero contribution to non-enriched samples. Within each dataset, lineage-restricted (macrophage or T cell) cell-level Spearman correlations between IMIREG and immune-marker features were computed using complete pairs (*n* ≥ 20).

#### Dimensionality reduction and visualization

Dimensionality reduction and visualization were implemented in R (v4.3.2) using tidyverse for data handling, prcomp for PCA, Rtsne (v0.16) for t-SNE, and ggplot2 (v3.4.4) for plotting. To ensure reproducibility of stochastic steps, a fixed seed was used (set.seed(42)). Numeric features were first denoised by PCA (prcomp; rank. = 20), and the top 20 PCs were used as input to t-SNE (Rtsne; dims = 2, perplexity = 20, theta = 0.8, max_iter = 300, pca = FALSE, check_duplicates = FALSE). Plots were generated with ggplot2, using manual palettes for discrete cell types and scale_color_gradient2 for continuous overlays.

#### Compartment-specific IMIREG marker discovery and bulk validation

A total of 1265 cell-type-specific gene sets were compiled from publicly available resources, such as MSigDB^48^ and PanglaoDB^49^. Detailed information of the cell state signatures is provided in Supplementary Table 5. The associations between IMIREG and the cell-type-specific gene sets signatures were quantified using Spearman correlation (rho) within each sample × compartment (T cell or macrophage), restricting to pairwise complete observations (*n*_pairs ≥ 20) and computing correlations via vectorized matrix operations. A two-stage inverse-variance meta-analysis was performed on Fisher z-transformed correlations (*z* = atanh(ρ), *v* = 1/(*n* − 3)): sample-level effects were pooled within each dataset using DerSimonian–Laird random-effects models, and dataset-level random-effects estimates (*z* and SE) were then pooled across datasets to obtain pan-cancer random-effects estimates per compartment and feature. Heterogeneity was summarized using Cochran’s *Q*, *τ*², and *I*², and *p*-values were Benjamini–Hochberg adjusted. Compartment specificity was evaluated by comparing pan-cancer T-cell versus macrophage effects using a Z-test on Fisher-Z differences, and candidates were prioritized for strong target-compartment association with weak off-target association; full results are reported in Supplementary Tables 7 and 8. scRNA-seq-derived candidates were then validated in TCGA bulk RNA-seq by computing pan-cancer Spearman correlations between TCGA IMIREG and each cell-state score using complete pairs with BH correction (Supplementary Table 9), retaining only candidates with pan-cancer rho > 0.5 for downstream bulk subtyping.

### IMIREG subtyping in TCGA bulk RNA-seq

TCGA bulk IMIREG subtyping was restricted to solid tumors, excluding DLBC and THYM to avoid confounding from immune-lineage-dominant malignancies. A global prerequisite for IMIREG-high status was defined as an IMIREG score exceeding the global median plus one median absolute deviation (MAD), calculated across the pan-cancer solid tumor compendium. For each sample, T-cell and macrophage driver composite scores were derived from the mean of the selected TCGA-validated compartment marker signatures. To assess threshold sensitivity, driver-high status was defined using two independent methodologies: a relative within-tumor Z-score threshold (*z* > 1.0) and an absolute within-tumor threshold (score > median + 1.0 × MAD). Samples exceeding the global IMIREG cutoff were classified into immune archetypes—*IMIREG_Tcell*, *IMIREG_macrophage*, or *IMIREG_dual-high*—based on these respective driver-high statuses. Prevalence and subtype distributions were summarized and visualized using stacked barplots in ggplot2.

### Processing and analysis of spatial transcriptomic data

To spatially contextualize IMIREG expression and its associated immune subtypes, we analyzed publicly available spatial transcriptomics datasets from the HEST-1k collection^40^. We selected three representative cases: a colorectal adenocarcinoma (COAD) sample (TENX29), characteristic of Macrophage-driven IMIREG, BRCA sample (TENX14) characteristic T cell-driven IMIREG, and a lung squamous cell carcinoma (LUSC) sample (TENX62), illustrative of macrophage + T cell-driven IMIREG. Raw spatial transcriptomic counts were normalized to CPM. Stringent quality control was applied to exclude low-quality spots based on the following criteria: (i) fewer than 1500 uniquely detected genes, or (ii) greater than 25% mitochondrial RNA fraction. Following spot-level filtration, the dataset was further filtered to reduce gene sparsity, retaining only genes with ≥2 counts in at least two distinct spots. IMIREG scores were calculated from the raw spatial transcriptomics data for each spot within the samples. The spatial distribution of IMIREG-high (IMIREG+) spots was then visually mapped onto the corresponding H&E images.

### Statistical analyses

Statistical tests were performed using R or GraphPad Prism version 10.2.3 (RRID: SCR_002798). Group comparisons (responders vs. nonresponders) used two-tailed unpaired t-tests. Receiver operating characteristic (ROC) curves were utilized to quantify predictive accuracy. Survival analyses used Kaplan–Meier curves and log-rank tests, with patients dichotomized by median + MAD (constant = 1) of the IMIREG scores. Immune correlates and signature associations were tested using Spearman’s correlation. Response classification was standardized across datasets based on available clinical metrics. For datasets with RECIST-based outcomes, patients with complete response (CR) or partial response (PR) were classified as responders, while those with stable disease (SD) or progressive disease (PD) were deemed nonresponders. In datasets with pCR data, patients achieving pCR were considered responders, and those without were classified as nonresponders. For clinical trial cohorts with protocol-specific definitions, the original criteria were preserved to ensure consistency and fidelity to each study design (see Supplementary Table 1).

## Supplementary information

Merged Supplementary Information

## Data Availability

The RNA-seq gene expression data of the UPMC TNBC cohort generated in this study has been deposited in the Gene Expression Omnibus database, under the accession code GSE312235. Further information and requests for resources and reagents should be directed to and will be fulfilled by the Lead Contact, Xiaosong Wang (xiaosongw@pitt.edu).

## Code availability

The key scripts used in this study have been deposited in Github (https://github.com/wangxlab/IMIREG). Source data are provided with this paper.
