# Supplementary material for: IMIREG: a lineage-resolved regulon signature unveiling immune engagement archetypes and predicting immunotherapy response across diverse cancers
Source: NPJ Precis Oncol. 2026 May 6;10:201. doi: 10.1038/s41698-026-01453-7 (PMC13234301; doi:10.1038/s41698-026-01453-7)
Supplement: Supplementary file 1 — Merged Supplementary Information [file 41698_2026_1453_MOESM1_ESM.pdf]

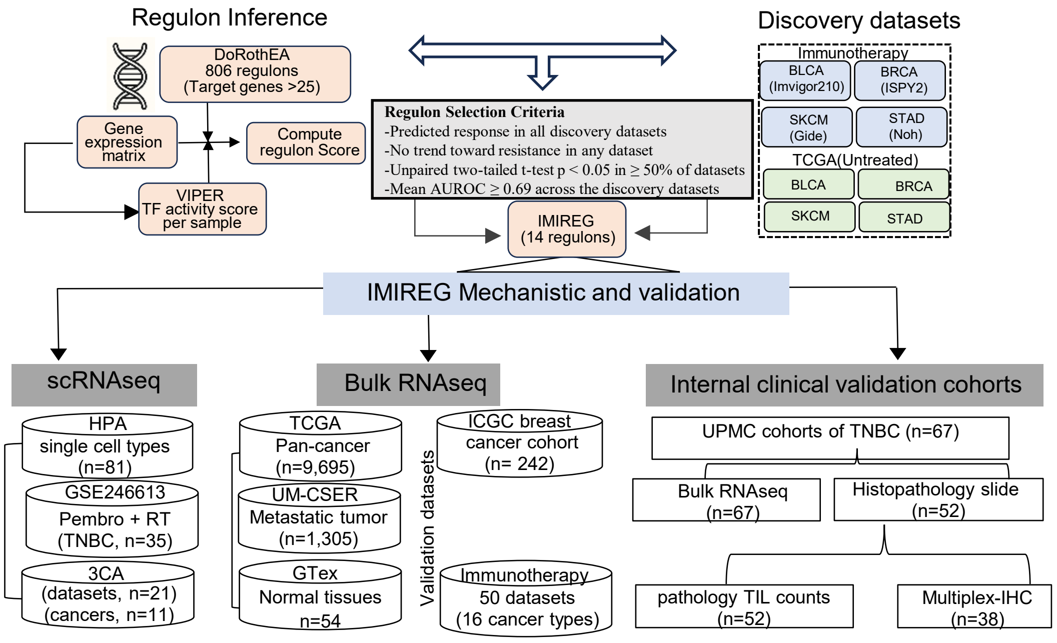

Figure S1: IMIREG discovery, scoring workflow, and multi-cohort mechanistic validation. Gene-expression matrices were converted to transcription-factor (TF) regulon activity profiles by applying DoRothEA (806 regulons; target-gene set size >25) and computing per-sample VIPER TF-activity/regulon scores. Regulon activity was screened across four discovery cohorts—BLCA (IMvigor210), BRCA (I-SPY2), SKCM (Gide), and STAD (Noh)—to identify 14 consistently predictive regulons, which were aggregated to form the IMIREG signature. Regulons were retained if they predicted response (never resistance), had two-tailed unpaired t-test $p < 0.05$ in ≥50% of discovery cohorts, and achieved mean AUROC ≥ 0.69 across four datasets. IMIREG mechanistic characterization and validation were then performed across complementary data types and cohorts: scRNA-seq reference/clinical datasets (HPA single-cell types, n=81; GSE246613 pembrolizumab+RT TNBC, n=35; 3CA datasets, n=21 across 11 cancers), bulk RNA-seq compendia (TCGA pan-cancer, n=9,695; UM-CSER distant metastatic tumors, n=1,305; GTEx normal tissues, n=54; ICGC breast cancer cohort, n=242), and immunotherapy collections (50 datasets spanning 16 cancer types, 52 treatment arms), with additional internal clinical validation cohorts (n=67) from UPMC. Different sample sizes in UPMC cohorts across modalities reflect archival tissue availability and assay-specific technical evaluability (RNA-seq n=67; H&E n=52; multiplex IHC n=38). **Abbreviations:** HPA, Human Protein Atlas; TNBC, triple-negative breast cancer; 3CA, single-cell cancer atlas (compendium); TCGA, The Cancer Genome Atlas; GTEx, Genotype-Tissue Expression Project; ICGC, International Cancer Genome Consortium.

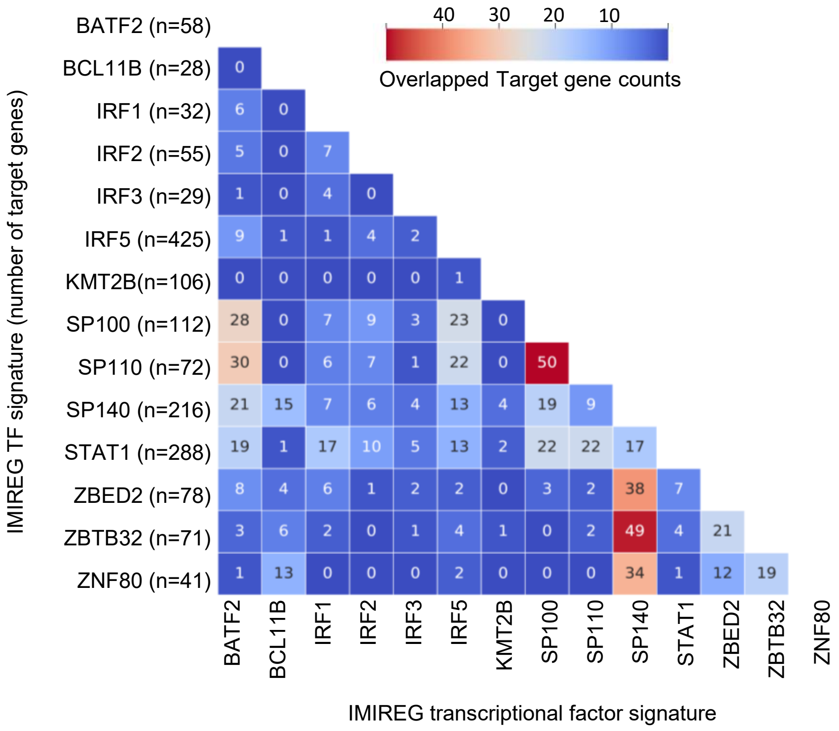

Figure S2: Overlap of IMIREG Transcription Factor Target Gene Signatures. This heatmap depicts pairwise overlap among the target gene sets (regulons) of the 14 transcription factors (TFs) that constitute the IMIREG signature. Each matrix entry reports the number of target genes shared between a given TF pair, with color intensity encoding overlap magnitude (red = greater overlap; blue = lesser overlap). Row and column labels indicate TF identity and regulon size (total number of target genes). Prominent overlap is observed between SP100 and SP110, and among ZBED2, ZBTB32, and ZNF80, each of which shares substantial target gene overlap with SP140—consistent with potential co-regulatory relationships or partial functional redundancy within the IMIREG regulatory program.

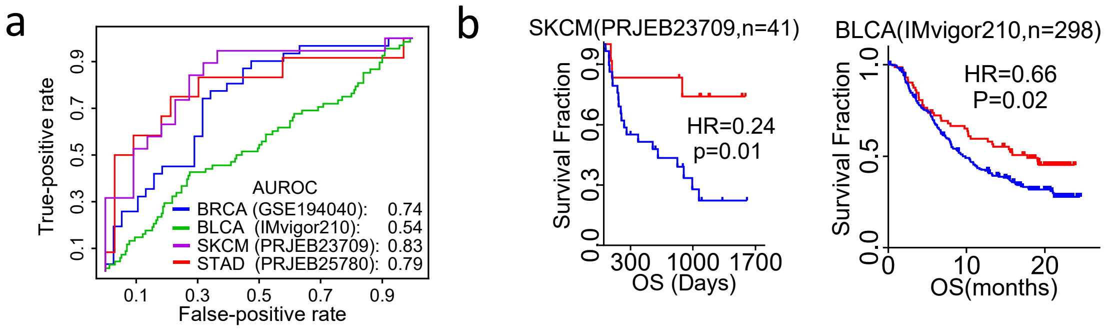

Figure S3: Performance of the IMIREG across discovery immunotherapy cohorts. (**a**) Receiver Operating Characteristic (ROC) curves demonstrating the predictive performance of IMIREG scores for distinguishing immunotherapy responders from non-responders across the discovery datasets. Area under the curve (AUC) values indicate classification accuracy, with 0.5 representing no predictive power and 1.0 indicating perfect discrimination. **(b)** Kaplan–Meier overall survival (OS) analyses comparing IMIREG-high versus IMIREG-low tumors in the Gide (SKCM) and IMvigor210 (BLCA) cohorts. Patients were stratified using a cohort-specific cutoff defined as median + MAD of IMIREG scores (MAD constant = 1). Hazard ratios (HRs) and corresponding p-values from log-rank testing are displayed on each plot. Abbreviations: BLCA, bladder urothelial carcinoma; BRCA, breast invasive carcinoma; SKCM, skin cutaneous melanoma; STAD, stomach adenocarcinoma.

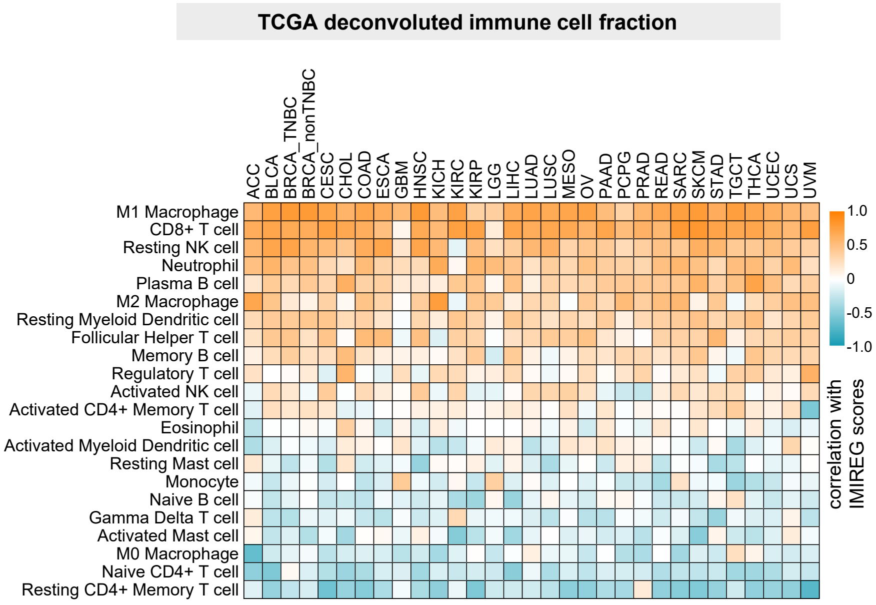

Figure S4. TCGA immune composition correlates of IMIREG. Heatmap showing the association between IMIREG scores and CIBERSORT deconvoluted immune cell fractions across TCGA pan-cancer tumors (DLBC and THYM were excluded to avoid confounding from immune-lineage dominant malignancies). Columns denote TCGA cancer types (standard TCGA abbreviations), and rows denote inferred immune cell populations from bulk RNA-seq deconvolution. Each heatmap cell reports the within–cancer type Spearman correlation coefficient (ρ) between the IMIREG score and the estimated fraction of the corresponding immune cell type across tumors in that cohort. Color indicates the direction and magnitude of correlation

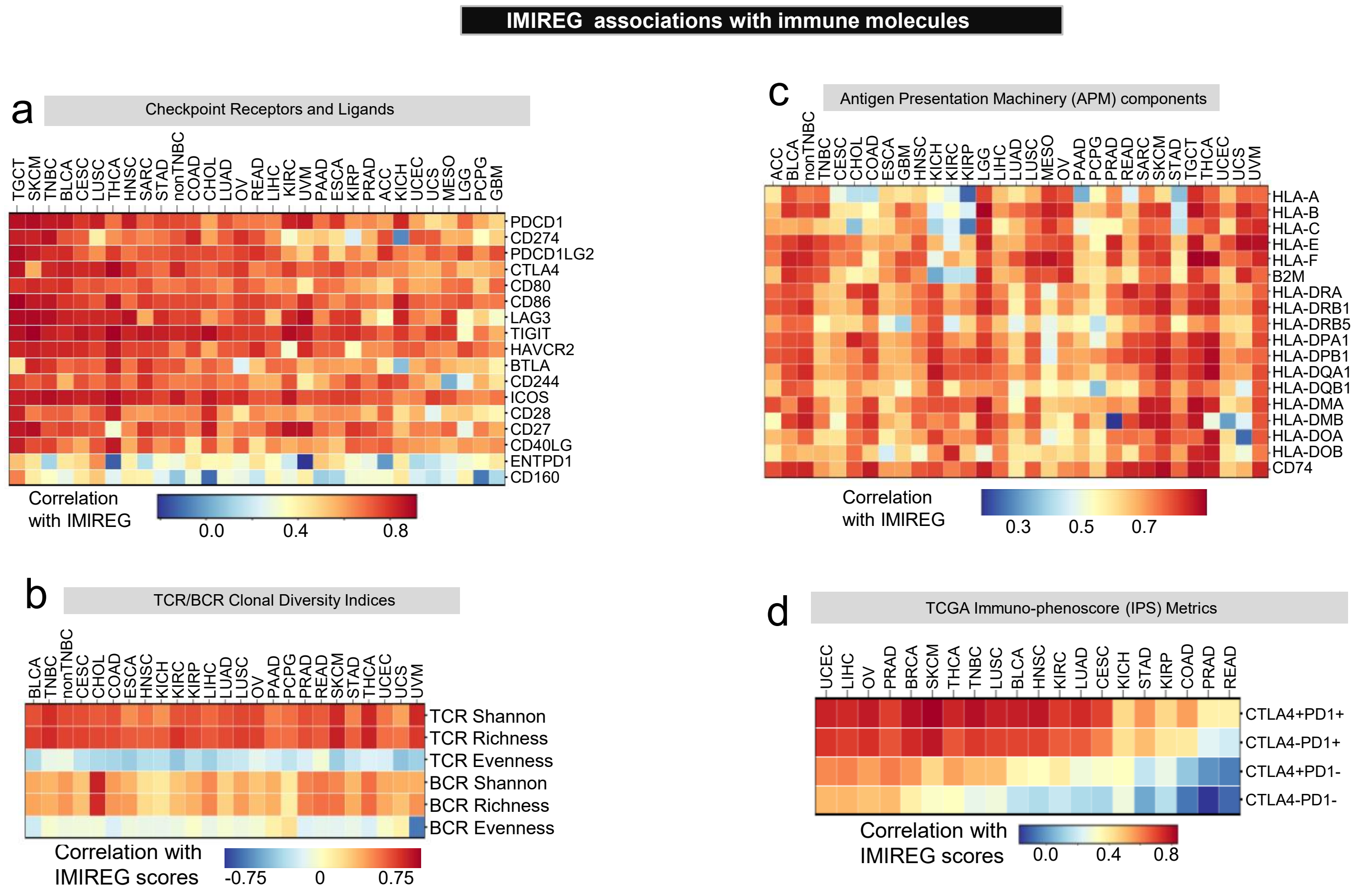

Figure S5: IMIREG associations with immune pathways and immune competence metrics in TCGA: Heatmaps summarize within–cancer-type Spearman correlations between the TCGA bulk IMIREG score and immune features (DLBC and THYM were excluded to avoid confounding from immune-lineage dominant malignancies). Panels: **(a)** checkpoint receptors/ligands, **(b)** TCR/BCR repertoire diversity indices (Shannon, richness, evenness), **(c)** antigen-presentation machinery (MHC class I/II and core APM genes), and **(d)** Immunophenoscore (IPS) components. Columns denote TCGA tumor types; rows list genes/metrics in each category. Each cell shows the Spearman correlation coefficient computed across patients in that tumor type using complete (finite) pairs. Colors indicate direction and magnitude on a diverging scale (red = positive, blue = negative). Heatmaps were rendered in R using ComplexHeatmap without additional row/column scaling (values are the raw Spearman correlation coefficients).

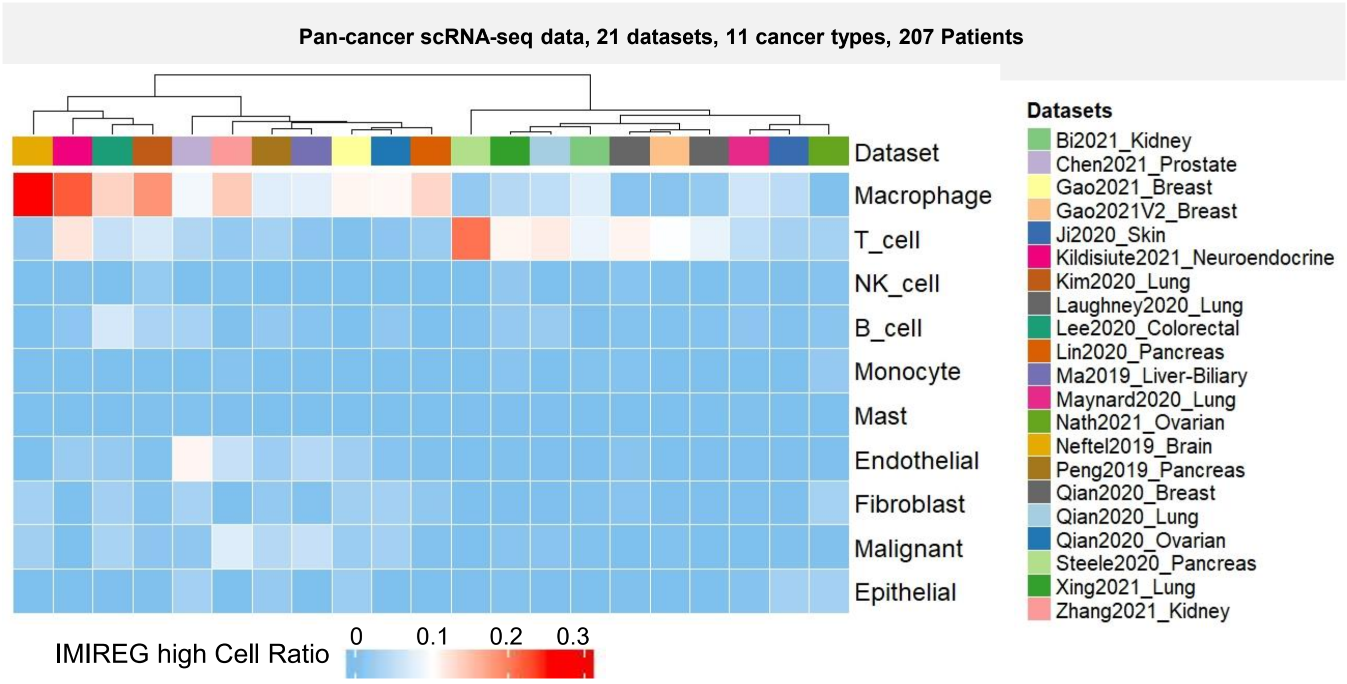

Figure S6: IMIREG-High Cell Distribution across Dataset and T Cell Correlation in Pan-Cancer Single-Cell Data. Heatmap depicting the percentage of IMIREG-high cells across cell types in single-cell RNA-seq data from 21 datasets spanning 11 cancer types (207 tumors). Cell types (x-axis) include T cell, Macrophage, NK cell, Monocyte, Mast cell, B cell, Malignant, Endothelial, Fibroblast, Epithelial. Color gradient represents IMIREG-high cell fractions relative to total cells per type.

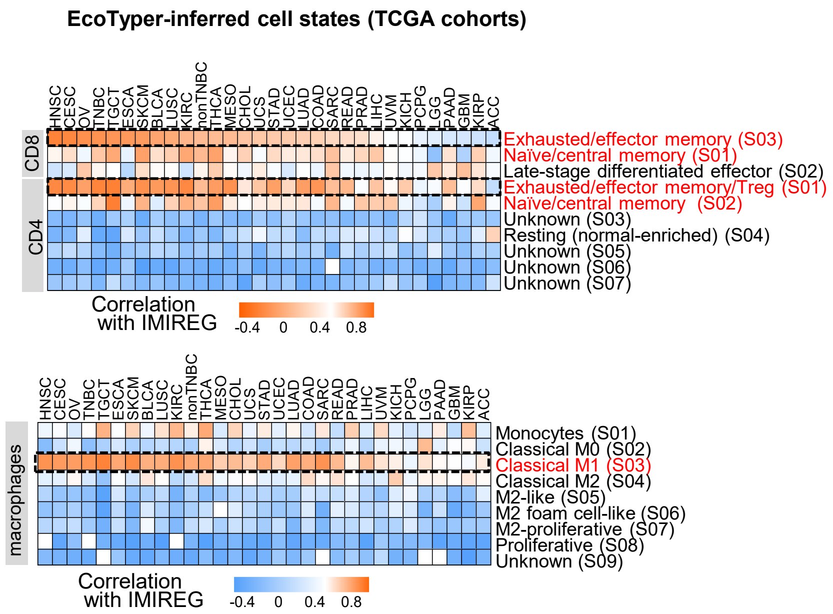

Figure S7. IMIREG Correlates with EcoTyper Immune Cell States Across TCGA Pan-Cancer. Heatmap depicting Spearman correlations between the IMIREG signature and EcoTyper-inferred immune cell states across TCGA pan-cancer tumors profiled by bulk RNA-seq. Rows correspond to immune lineages ($CD4^+$ T cells, $CD8^+$ T cells, and macrophages), and columns correspond to individual cancer types. IMIREG shows positive correlations with exhausted and effector memory–like $CD4^+$/$CD8^+$ T-cell states and M1-like macrophage states, while exhibiting negative correlations with naïve T-cell states and M2-like macrophage states.

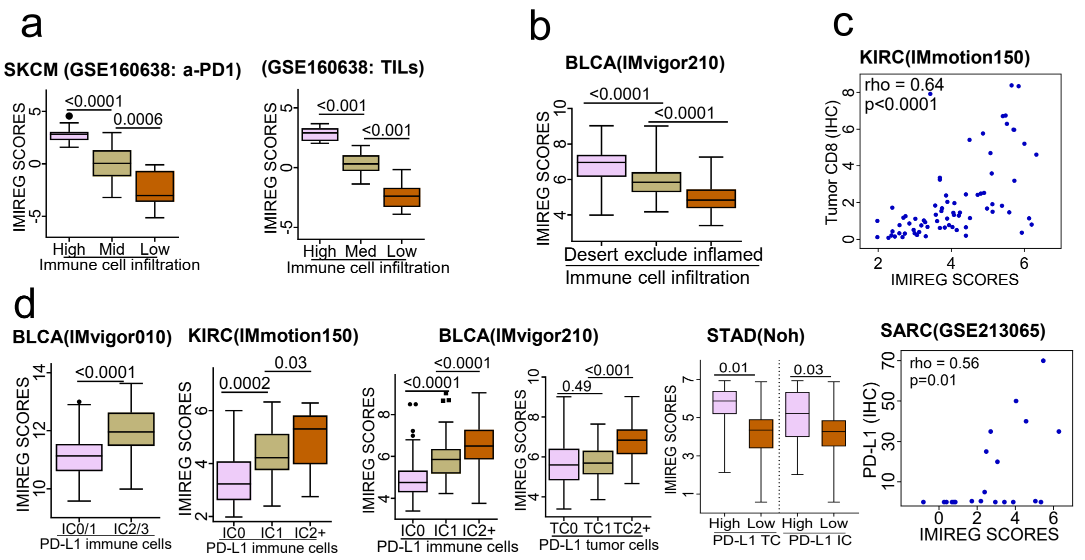

Figure S8. IMIREG score associations with immune landscape features and checkpoint expression across clinical immunotherapy cohorts. (a) Box and whiskers plot of IMIREG scores across immune infiltration strata (High, Med, Low) shown separately for patients treated with anti-PD-1 (a-PD1) and adoptive T-cell therapy (ATC) in GSE160638 (SKCM) dataset. Immune infiltration groups were defined using the cohort ImmuneScore clustering.**(b)** BLCA (IMvigor210): Box and whiskers plot of IMIREG scores across tumor immune phenotypes (immune desert, immune excluded, immune inflamed) as defined in the original study.**(c)** Scatter plot showing a positive correlation (r = 0.64, p < 0.0001) between IMIREG scores and tumor CD8 IHC expression in the KIRC IMmotion150 cohort. **(d)** IMIREG associations with PD-L1 IHC across cohorts. Left: boxplots of IMIREG scores stratified by PD-L1 categories in immune cells (IC) and/or tumor cells (TC) where available (BLCA IMvigor010; KIRC IMmotion150; BLCA IMvigor210; STAD Noh). Right: Spearman correlation between IMIREG and PD-L1 IHC in SARC (GSE213065). Boxplots show median and interquartile range; points represent individual samples. P-values and correlation statistics are displayed in each panel.

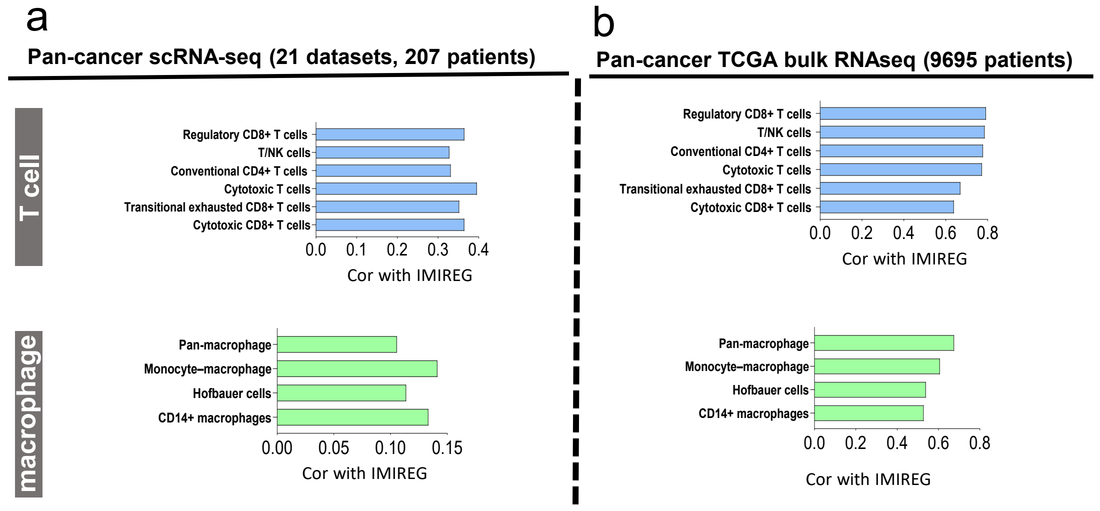

Figure S9. IMIREG correlation screen identifies compartment-specific surrogate markers for IMIREG subtype identification. (A) From a compendium of n = 1,265 cell-state signature scores we computed lineage-restricted IMIREG correlations in single-cell datasets using hierarchical random-effects meta-analysis to obtain ρ_random for T cells and macrophages, then cross-validated candidates by pan-cancer TCGA Spearman correlation with bulk IMIREG. Final compartment-specific candidates were required to be both strongly associated in the target lineage and specific against the off-target lineage, with significant between-lineage difference (P_adj_diff < 0.01) and bulk validation (TCGA ρ > 0.5). T-cell candidates satisfied: ρ_random_T_cell > 0.3, ρ_random_Macrophage < 0.2, and negative Δ_M (ρ_T_cell > ρ_Macrophage); macrophage candidates satisfied: ρ_random_Macrophage > 0.1, ρ_random_T_cell < 0.051, and negative Δ_T (ρ_Macrophage > ρ_T_cell). Only 4 macrophage signatures met these criteria and were retained as surrogates for the IMIREG-macrophage subtype; 6 T-cell signatures met criteria, of which the top 4 were used as surrogates for IMIREG–T-cell subtyping. (B) Pan-cancer TCGA validation of the selected surrogate signatures, showing Spearman correlations between bulk IMIREG scores and the finalized T-cell and macrophage cell-state signatures across TCGA solid tumors; the top-ranked signature(s) for each compartment remained strongly and significantly associated with IMIREG in bulk tissue.

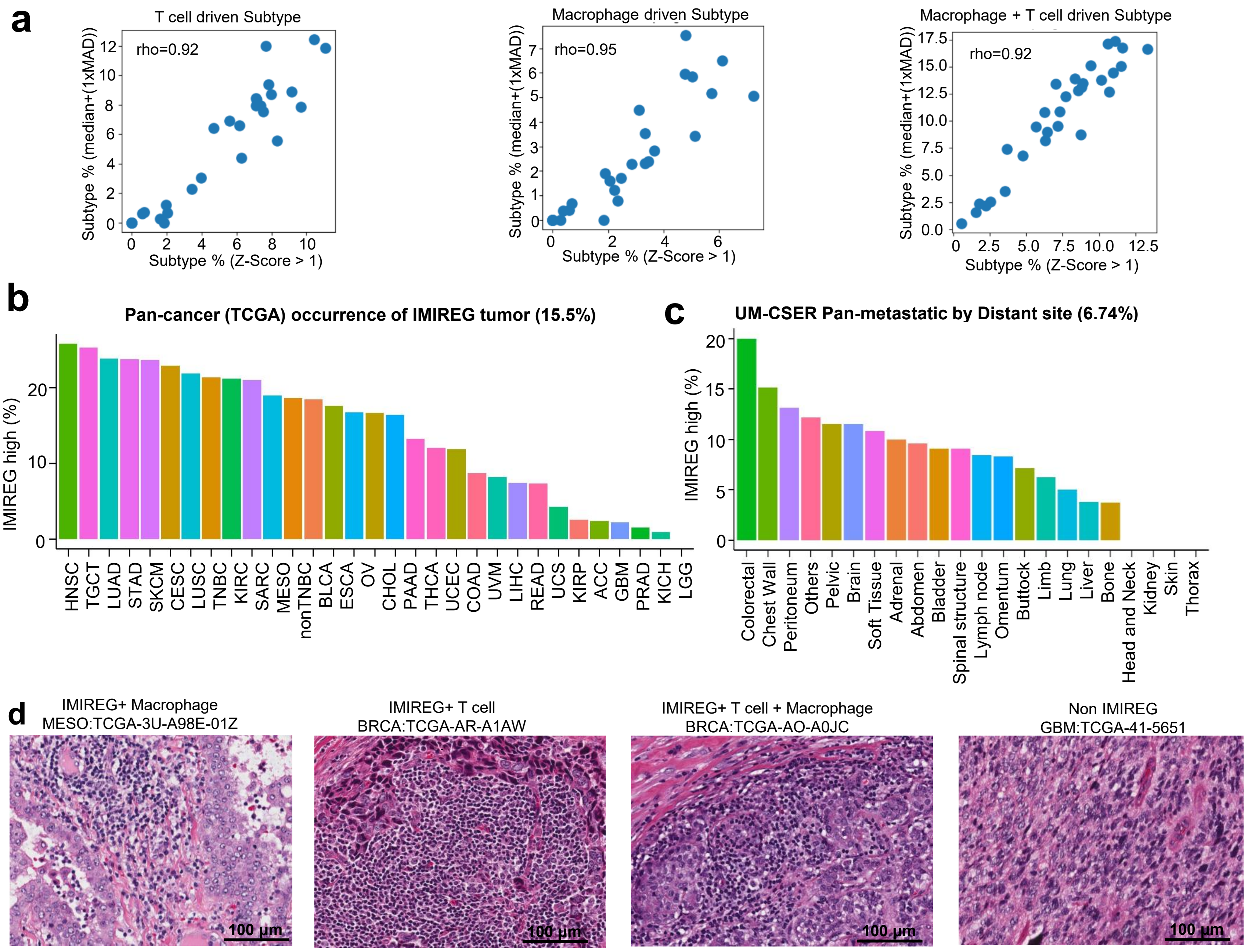

Figure S10: IMIREG-High Tumor Prevalence Across Cohorts: (A) Scatter plots show the relationship between z-score–derived IMIREG subtype frequency and median+MAD–derived IMIREG subtype frequency across TCGA solid tumors. Each point represents a cancer type. Associations were assessed using Spearman's rank correlation. **(B)** Bar plot of IMIREG-high tumor percentages across TCGA cancer types, sorted by prevalence. **(C)** Bar plot of IMIREG-high tumor percentages in UM-CSER pan-metastatic cohorts, sorted by distant metastatic location. **(D)** Representative H&E examples illustrating histologic context for IMIREG subtypes in TCGA: macrophage-driven, T-cell–driven, dual-driven IMIREG-high, and non-IMIREG tumors. Scale bars, 100 µm.

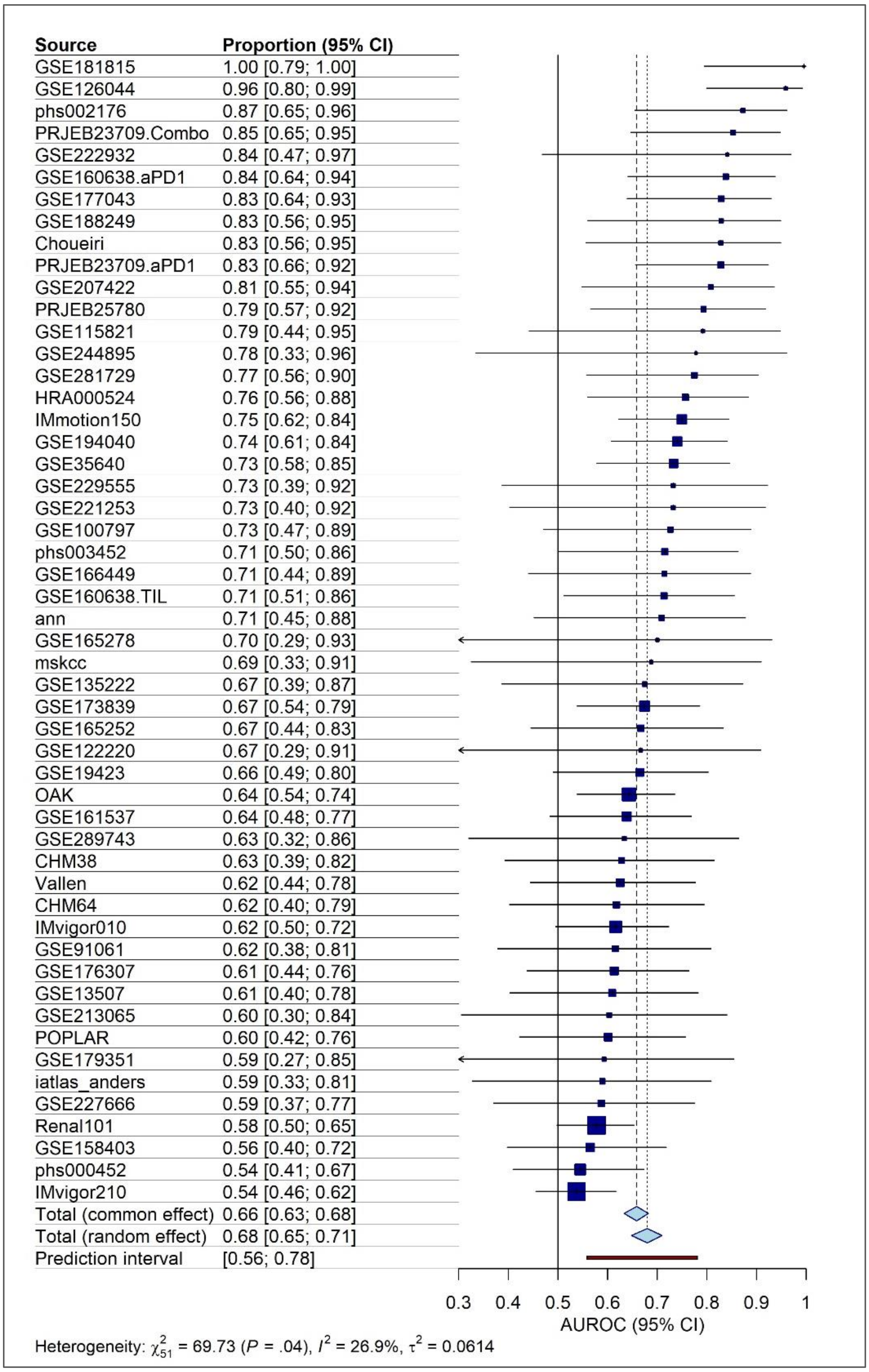

Figure S11: Forest Plot of IMIREG Performance. The forest plot summarizes the predictive performance (AUROC) of IMIREG across 50 independent immunotherapy cohorts. Two cohorts contributed separate treatment arms evaluated independently, yielding 52 entries in the plot. Each study is represented by a square, with the size corresponding to its statistical weight. Horizontal lines indicate the 95% CI. AUROCs were logit-transformed prior to DerSimonian–Laird random-effects pooling; all displayed values and confidence intervals were obtained by inverse-logit back-transformation. The diamond at the base represents the pooled random-effects estimate. Heterogeneity was low-to-moderate (I^2 = 26.9%, τ^2 = 0.06), supporting consistency of IMIREG's predictive accuracy across diverse cancer types and immunotherapy regimens.

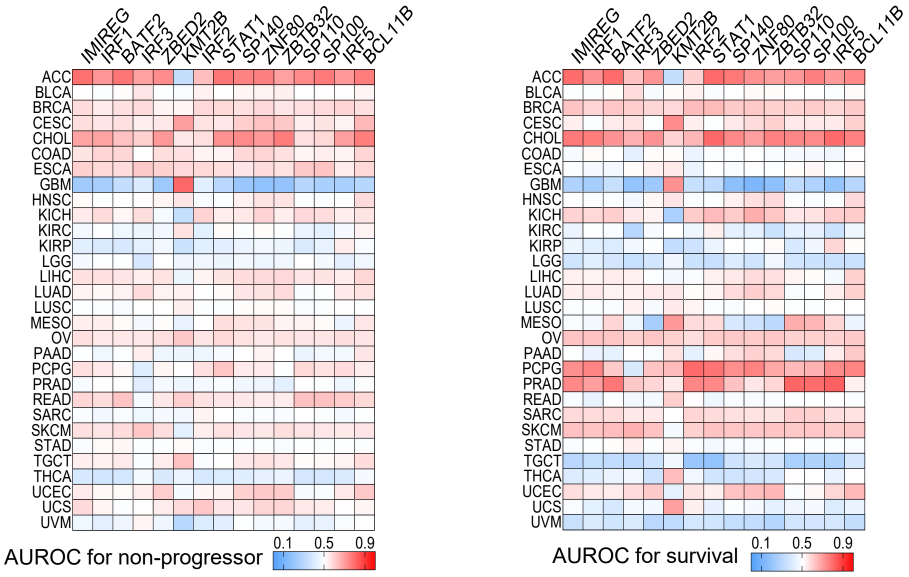

Figure S12: TCGA pan-cancer evaluation of IMIREG for non-progression and survival. Heatmaps summarize AUROC values for IMIREG and its constituent transcription-factor regulons in predicting non-progression and survival status across TCGA cancer types using bulk RNA-seq. Predictive performance is heterogeneous across cancers and does not indicate a consistent pan-cancer prognostic association.

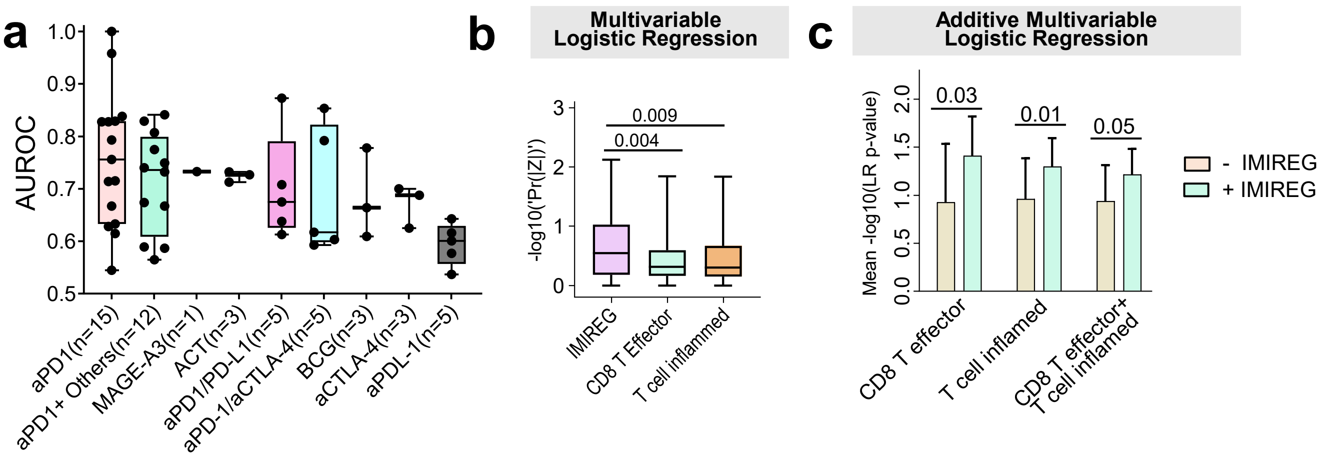

Figure S13: Pan-immunotherapy performance and independent predictive value of IMIREG. (**A**) Box-and-whisker plots illustrating the distribution of Area Under the Receiver Operating Characteristic (AUROC) values for IMIREG across different treatment classes. Each individual data point represents an independent patient cohort. box components: The center line denotes the median AUROC, while the box bounds represent the interquartile range (IQR). Whiskers: Whiskers extend to the minimum and maximum values. (**B**) Multivariable logistic regression comparing IMIREG to T-cell abundance–based predictors (e.g., CD8 effector/T cell–inflamed), highlighting IMIREG's relative contribution. (**C)** Additive multivariable models combining IMIREG with infiltration-derived metrics, showing improved predictive performance over T-cell abundance metrics alone.

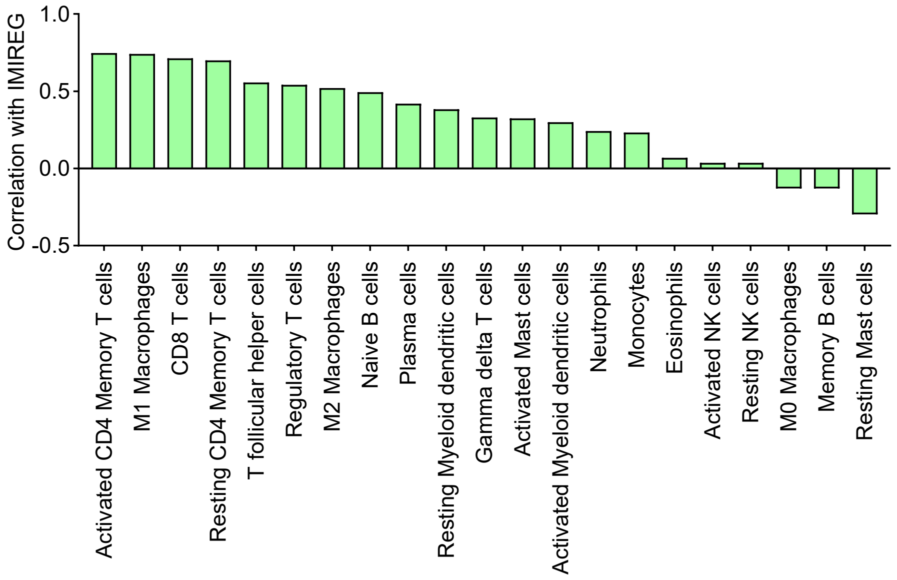

Figure S14. Bar chart illustrating the association between IMIREG scores and immune cell populations inferred via CIBERSORT deconvolution in the UPMC TNBC validation cohort. IMIREG expression showed strong positive correlations with M1 macrophages, CD8+ T cells, and memory CD4+ T cells, suggesting immune-active features in IMIREG-high TNBC tumors.

Supplementary Table Legends

Supplementary Table S1. Overview of publicly available datasets analyzed in this study. Cohort inventory of immunotherapy trial datasets and reference resources used throughout the study. Fields include dataset accession/identifier, cancer type, study/author metadata, treatment context, sample size and responder/non-responder counts, platform, response endpoint definition, and cohort-level predictive-performance summaries. ACT: Adoptive Cell Therapy; BCG: Bacillus Calmette–Guérin; DCR: Disease Control Rate; DP: Disease Progression; DR: Disease Recurrence; ORR: Objective Response Rate; OS: Overall Survival; pCR: Pathological Complete Response; PFS: Progression-Free Survival; PSA50: ≥50% reduction in Prostate-Specific Antigen; RECIST: Response Evaluation Criteria in Solid Tumors; RFS: Relapse-Free Survival

Supplementary Table S2. Clinical and demographic information for the UPMC internal validation cohort. Summary of clinical and demographic characteristics for the UPMC TNBC validation cohort, including cohort size and key clinicopathologic variables .

Supplementary Table S3. Details of antibodies used for multiplex immunohistochemistry (IHC). Reagent list for the multiplex IHC panel used in the UPMC validation experiments, including antibody target, clone, working concentration/dilution, and vendor/supplier information.

Supplementary Table S4. Comprehensive list of target genes regulated by transcription factors in the IMIREG signature. Complete TF–target regulon membership for the 14 IMIREG transcription factors from the DoRothEA resource.

Supplementary Table S5. Cell-state marker gene list. Curated cell-state gene-set compendium used for module scoring/enrichment analyses in single-cell and bulk datasets.

Supplementary Table S6. T cell and macrophage subset markers. Reference marker panels used to annotate immune sub-states in single-cell analyses (T-cell subsets and macrophage polarization states).

Supplementary Table S7. Dataset-level meta-analysis of IMIREG association with cell-state markers. Random- and fixed-effects correlation meta-analysis results computed within each scRNA-seq dataset and immune compartment. Columns include dataset, cancer type category, compartment (T cell vs macrophage), feature (gene-set score), number of samples contributing, pooled correlation estimates, significance, and heterogeneity statistics (e.g., Q and I^2) with BH-adjusted p-values.

Supplementary Table S8. Pan-cancer scRNA-seq meta-analysis of IMIREG association with cell-state markers. Pan-cancer summary of compartment-resolved random-effects correlations between IMIREG and each candidate cell-state score across scRNA-seq datasets. Includes macrophage- and T-cell-specific pooled effects, dataset counts, and statistical comparison of compartment specificity (e.g., Z-test difference and adjusted p-values).

Supplementary Table S9. Spearman correlation between IMIREG expression and cell-state markers in TCGA pan-cancer cohorts. Validation of scRNA-seq–derived candidate markers in TCGA bulk RNA-seq: pan-cancer Spearman correlations between TCGA IMIREG and each cell-state score, including sample size, correlation coefficient, nominal p-value, and BH-adjusted p-value.

Abbreviations: AUROC, area under the receiver operating characteristic curve; BH, Benjamini–Hochberg; ICB, immune checkpoint blockade; IHC, immunohistochemistry; TCGA, The Cancer Genome Atlas.

Table S1: Overview of publicly available datasets analyzed in this study

Dataset	CancerType	Author	PMID	Treatment	TotalSample	Responders	NonResponders		PLATFORM	Endpoint	AUROC	Sensitivity	Specificity
GSE13507	BLCA	Kim	20059769	BCG	37	24	13		Array	Recurrence	0.609	0.75	0.538
GSE176307	BLCA	Rose	34294892	anti-PD1/PD-L1	89	16	73		RNAseq	RECIST	0.613	0.562	0.767
GSE19423	BLCA	Kim	20233890	BCG	48	26	22		Array	DR	0.664	0.577	0.818
GSE222932	BLCA	Jang	36928921	anti-PDL1+gua…	15	4	11		RNA-seq	ORR	0.841	0.75	1
GSE244895	BLCA	Lee	38203315	BCG	12	3	9		RNAseq	DP	0.778	0.667	1
IMvigor010	BLCA	Powles	37500339	anti-PD-L1	114	38	76		RNA-seq	OS	0.616	0.395	0.868
IMvigor210	BLCA	Mariathasan	29443960	anti-PD-L1	298	68	230		RNA-seq	RECIST	0.537	0.368	0.761
phs003452	BLCA	Beckabir	38789460	anti-PD-1	30	10	20		RNA-seq	pCR	0.715	1	0.5
GSE194040	BRCA	Wolf	35623341	anti-PD-1+ Pa…	69	31	38		Array	pCR	0.74	0.774	0.658
GSE173839	BRCA	Pusztai	34143979	anti-PD-1/ola…	71	29	42		Array	pCR	0.674	0.931	0.405
GSE177043	TNBC	Hammerl	34580291	anti-PD-1	34	20	14		RNAseq	OS	0.829	0.85	0.786
iatlas_anders_2022	BRCA	Anders	35121644	aPD1/Chemo	31	7	24		RNAseq	RECIST	0.589	0.571	0.708
GSE289743	cSCC	Baruch	40836096	anti-PD-1	17	12	5		RNAseq	PathologyResp…	0.633	0.5	0.4
GSE165252	ESCA	PERFECT	33504550	anti-PD-L1/Ch…	32	12	20		RNAseq	pCR	0.667	0.583	0.85
GSE281729	HNSC	Mastrolonardo	39585339	aPD1+IDO	36	21	15		RNA-seq	pCR	0.775	0.905	0.667
ann_Miao	KIRC	Miao	29301960	anti-PD-1/PD-…	29	5	24		RNAseq	RECIST	0.708	1	0.458
Choueiri	KIRC	Choueiri	27169994	anti-PD-1	16	8	8		Array	RECIST	0.828	1	0.625
IMmotion150	KIRC	IMmotion150	29867230	anti-PD-1+Bev	84	28	56		RNA-seq	RECIST	0.749	0.786	0.661
Renal101	KIRC	Motzer	32895571	anti-PDL-1	242	160	82		RNA-seq	PFS	0.577	0.519	0.646
GSE126044	NSCLC	Cho	32879421	anti-PD-1	16	4	12		RNAseq	RECIST	0.958	1	0.833
GSE135222	NSCLC	Jung	31537801	anti-PD-1/PD-…	27	6	21		RNAseq	PFS	0.675	0.833	0.667
GSE161537	NSCLC	Foy	36038492	anti-PD-1/PD-…	75	19	56		RNA-seq	RECIST	0.638	0.789	0.536
GSE166449	NSCLC	SELECT	33857424	anti-PD-1	22	7	15		RNA-seq	RECIST	0.714	0.857	0.6
GSE207422	NSCLC	Hu	36869384	anti-PD-1/Che…	24	9	15		RNAseq	pCR	0.807	0.778	0.867
OAK	NSCLC	Patil	35216676	anti-PD-L1	318	48	270		RNAseq	RECIST	0.643	0.562	0.789
POPLAR	NSCLC	Fehrenbacher	26970723	anti-PD-L1	87	13	74		RNA-seq	RECIST	0.601	0.538	0.73
GSE188249	OV	Chen	35671108	anti-PD-1/gua…	17	7	10		Array	RECIST	0.829	1	0.7
GSE227666	OV	Ray-Coquard	39013870	anti-PD-1/Che…	32	13	19		RNA-seq	RECIST	0.587	0.462	0.789
GSE179351	PDAC.CRC	Parikh	35122060	aPD-1/aCTLA-4	12	3	9		RNA-seq	RECIST	0.593	1	0.444
GSE229555	PRAD	COMBAT	38167882	anti-PD-1 +BAT	15	7	8		RNAseq	PSA50	0.732	0.571	1
GSE213065	SARC	Subramanian	38429415	anti-CTLA-4/a…	21	4	17		RNAseq	RECIST	0.603	1	0.353
phs002176	SCLC	Roper	34162872	anti-PD-1/PD-…	23	6	17		RNA-seq	RECIST	0.873	0.833	0.824
CHM38.Campbell	SKCM	Campbell	37037616	anti-PD-1	49	10	39		RNA-seq	RECIST	0.628	0.4	0.949
CHM64.Campbell	SKCM	Campbell	37037616	aPD-1/aCTLA-4	42	14	28		RNA-seq	RECIST	0.617	0.571	0.75
GSE100797	SKCM	Lause	29170503	ACT	25	10	15		RNAseq	RECIST	0.727	0.7	0.867
GSE115821	SKCM	Auslander	30127394	aPD-1/aCTLA-4	14	2	12		RNA-seq		0.792	1	0.667
GSE122220	SKCM	Jaiswal	na	anti-PD-1	10	4	6		Array	RECIST	0.667	0.75	0.667
GSE158403	SKCM	Ribas	33288749	Durv/mek inhib	48	22	26		RNA-seq		0.565	0.5	0.692
GSE160638	SKCM	Markovits	37074069	anti-PD-1	36	22	14		RNAseq	RECIST	0.838	0.773	0.857
GSE160638	SKCM	Markovits	37074069	ACT	37	19	18		RNAseq	RECIST	0.713	0.895	0.611
GSE165278	SKCM	Zappasodi	33588426	anti-CTLA-4	7	2	5		RNA-seq	RECIST	0.7	1	0.6
GSE221253	SKCM	Barras	38306416	ACT	15	7	8		RNAseq	RECIST	0.732	0.857	0.625
GSE35640	SKCM	Ulloa-Montoya	23715562	MAGE-A3	56	22	34		Array	RECIST	0.733	0.818	0.618
GSE91061	SKCM	Riaz	29033130	anti-PD1	49	10	39		RNA-seq	RECIST	0.615	0.4	0.949
HRA000524	SKCM	Cui	33542239	anti-PD-1	55	14	41		RNA-seq	RECIST	0.756	0.786	0.707
mskcc	SKCM	Snyder	25409260	anti-CTLA-4	16	4	12		RNA-seq	RECIST	0.688	0.75	0.75
phs000452	SKCM	Liu	31792460	anti-PD-1	72	32	40		RNA-seq	RECIST	0.545	0.812	0.325
PRJEB23709	SKCM	Gide	30753825	anti-PD-1	41	19	22		RNA-seq	RECIST	0.828	0.947	0.636
PRJEB23709	SKCM	Gide	30753825	anti-CTLA-4/a…	32	26	6		RNA-seq	RECIST	0.853	0.692	1
Van Allen	SKCM	Van Allen	26359337	anti-CTLA-4	40	8	32		RNA-seq	RECIST	0.625	1	0.5
PRJEB25780	STAD	Noh	33547412	anti-PD-1	45	12	33		RNA-seq	RECIST	0.793	0.75	0.788
GSE181815	THYM	He	34622229	anti-PD1	8	4	4		RNA-seq	RECIST	1	1	1

Table S2: Clinical and demographic information for the UPMC internal validation cohorts

Characteristic	Prospective s…
Median age, years (range)	54 (range 29–…
Sex	67 (100%)
Median survival (range)	61.5 (range 5…
Median CS tumor size (range)	22 (2-995)
Race	Black: 9(13.4…
	White: 53(79.…
	Unknown: 5 (7…
Origin	Non-Spanish; …
	Unknown origi…
Post-menopause	Yes: 34 (50.7…
	No: 22 (32.83…
	Unknown :13 (…
Previous abortion:	Yes: 13(19.40…
	No: 41 (61.19…
	Unknown: 13 (…
gravida	Nulligravida:…
	Monogravida: …
	Multi-gravida…
	Unknown:9 (13…

Table S3: Details of antibodies used for multiplex immunohistochemistry (IHC)

Antibody	Clone	Concentration	Supplier
CD8	C8/144B	1:150	Biocare Medical
PD-L1	E1L3N	1:500	Cell Signaling
a-SMA	D4K9N	1:500	Cell Signaling
pan-cytokeratin	AE1/AE3	1:150	Santa Cruz
CD68	D4B9C	1:700	Cell Signaling

tf	confidence	target	mor
IRF1	A	DST	-1
IRF1	A	FOXP3	-1
IRF3	C	PURA	-1
IRF5	E	PDLIM7	-1
SP100	E	ETS1	-1
STAT1	A	AGFG2	-1
STAT1	A	CDC7	-1
STAT1	A	CXCL8	-1
STAT1	A	FCGRT	-1
STAT1	A	FGFR3	-1
STAT1	A	HCFC1	-1
STAT1	A	HSP90AA1	-1
STAT1	A	IFNA1	-1
STAT1	A	IFNB1	-1
STAT1	A	IFNG	-1
STAT1	A	IL6	-1
STAT1	A	MMP13	-1
STAT1	A	MMP9	-1
STAT1	A	NAA35	-1
STAT1	A	NR1H4	-1
STAT1	A	PRKCE	-1
STAT1	A	RPS24	-1
STAT1	A	ZNF367	-1
BATF2	E	APOL1	1
BATF2	E	APOL3	1
BATF2	E	APOL6	1
BATF2	E	CASP1	1
BATF2	E	CCL7	1
BATF2	E	CCL8	1
BATF2	E	CXCL10	1
BATF2	E	CXCL11	1
BATF2	E	CXCL9	1
BATF2	E	CXCR3	1
BATF2	E	DHX58	1
BATF2	E	EPSTI1	1
BATF2	E	ETV7	1
BATF2	E	GBP1	1
BATF2	E	GBP4	1
BATF2	E	GBP5	1
BATF2	E	GZMA	1
BATF2	E	HLA-C	1
BATF2	E	HLA-DMA	1
BATF2	E	HLA-E	1
BATF2	E	HLA-F	1
BATF2	E	IFI30	1
BATF2	E	IFI35	1
BATF2	E	IFI44L	1
BATF2	E	IFIH1	1
BATF2	E	IFIT3	1
BATF2	E	IFITM1	1
BATF2	E	IRF1	1
BATF2	E	IRF9	1
BATF2	E	KLHDC7B	1
BATF2	E	LAG3	1
BATF2	E	LAP3	1
BATF2	E	NKG7	1
BATF2	E	NUB1	1
BATF2	E	OAS1	1
BATF2	E	OAS2	1
BATF2	E	OASL	1
BATF2	E	PARP12	1
BATF2	E	PARP9	1
BATF2	E	PPP1R14D	1
BATF2	E	PSMB9	1
BATF2	E	PSME1	1
BATF2	E	RAB28	1
BATF2	E	RHOF	1
BATF2	E	RTP4	1
BATF2	E	SAMD9L	1
BATF2	E	SLA2	1
BATF2	E	SOCS1	1
BATF2	E	SP140L	1
BATF2	E	STAT1	1
BATF2	E	TAP1	1
BATF2	E	TAP2	1
BATF2	E	TRIM21	1
BATF2	E	UBE2L6	1
BATF2	E	USP18	1
BATF2	E	WARS1	1
BATF2	E	XAF1	1
BATF2	E	ZBP1	1
BCL11B	E	CCND2	1
BCL11B	E	CD2	1
BCL11B	E	CD247	1
BCL11B	E	CD3E	1
BCL11B	E	CD3G	1
BCL11B	E	CD40LG	1
BCL11B	E	CD6	1
BCL11B	E	CD96	1
BCL11B	E	FCRL1	1
BCL11B	E	GRAP2	1
BCL11B	E	IKZF1	1
BCL11B	E	ITK	1
BCL11B	E	ITPRIPL1	1
BCL11B	E	LTB	1
BCL11B	E	NELL2	1
BCL11B	E	NLRC3	1
BCL11B	E	NLRC5	1
BCL11B	E	POU3F1	1
BCL11B	E	PYHIN1	1
BCL11B	E	RUNX3	1
BCL11B	E	SIRPG	1
BCL11B	E	SLAMF1	1
BCL11B	E	TENM1	1

Table S4: Comprehensive list of target genes regulated by transcription factors in the IMIREG signature

tf	confidence	target	mor
BCL11B	E	THEMIS	1
BCL11B	E	TRAT1	1
BCL11B	E	UBASH3A	1
BCL11B	E	ZAP70	1
BCL11B	E	ZNF831	1
IRF1	A	CASP7	1
IRF1	A	CCL5	1
IRF1	A	CD40	1
IRF1	A	CEACAM1	1
IRF1	A	CIITA	1
IRF1	A	CXCL10	1
IRF1	A	CYBB	1
IRF1	A	EIF2AK2	1
IRF1	A	HLA-A	1
IRF1	A	HLA-G	1
IRF1	A	IFIT3	1
IRF1	A	IFNA1	1
IRF1	A	IFNB1	1
IRF1	A	IFNG	1
IRF1	A	IL10	1
IRF1	A	IL12A	1
IRF1	A	IL12B	1
IRF1	A	IL15	1
IRF1	A	IL27	1
IRF1	A	IL4	1
IRF1	A	IL5	1
IRF1	A	IRF2	1
IRF1	A	OGN	1
IRF1	A	OPTN	1
IRF1	A	PSMB9	1
IRF1	A	SOCS1	1
IRF1	A	TAP1	1
IRF1	A	TAP2	1
IRF1	A	TNFSF10	1
IRF1	A	TP53	1
IRF2	C	CIITA	1
IRF2	C	TAP1	1
IRF2	C	CASP7	1
IRF2	C	CASP8	1
IRF2	C	NR3C1	1
IRF2	C	ADAR	1
IRF2	C	AFF1	1
IRF2	C	APOL2	1
IRF2	C	APOL3	1
IRF2	C	BBC3	1
IRF2	C	BTN3A1	1
IRF2	C	BTN3A3	1
IRF2	C	CALCOCO2	1
IRF2	C	CEACAM1	1
IRF2	C	CEP128	1
IRF2	C	DDX3X	1
IRF2	C	FAM111A	1
IRF2	C	FAM13A	1
IRF2	C	FANCA	1
IRF2	C	FLT3LG	1
IRF2	C	FMR1	1
IRF2	C	FOXP1	1
IRF2	C	HOXB3	1
IRF2	C	IFIT3	1
IRF2	C	IKZF4	1
IRF2	C	IL15RA	1
IRF2	C	IL1R1	1
IRF2	C	IL4	1
IRF2	C	ISG15	1
IRF2	C	MAX	1
IRF2	C	MDK	1
IRF2	C	MICB	1
IRF2	C	NFIC	1
IRF2	C	NFIX	1
IRF2	C	NMI	1
IRF2	C	NOD1	1
IRF2	C	PARP14	1
IRF2	C	PER1	1
IRF2	C	PLAAT4	1
IRF2	C	PRKD2	1
IRF2	C	PTMA	1
IRF2	C	SEMA4C	1
IRF2	C	SLC25A22	1
IRF2	C	SOCS1	1
IRF2	C	SP140L	1
IRF2	C	SRBD1	1
IRF2	C	TAL1	1
IRF2	C	TASOR2	1
IRF2	C	TGIF1	1
IRF2	C	WDR74	1
IRF2	C	ZC3HAV1	1
IRF2	C	ZEB2	1
IRF2	C	ZFP36	1
IRF2	C	ZNF566	1
IRF2	C	ZNF93	1
IRF3	C	CXCL10	1
IRF3	C	IFNA1	1
IRF3	C	IFNB1	1
IRF3	C	IRF7	1
IRF3	C	ABCA7	1
IRF3	C	AKAP8L	1
IRF3	C	APOBEC3C	1
IRF3	C	APOBEC3D	1
IRF3	C	APOBEC3F	1
IRF3	C	APOBEC3G	1
IRF3	C	BCAS3	1
IRF3	C	BST2	1
IRF3	C	CCL5	1
IRF3	C	CYTH2	1

Table S4: Comprehensive list of target genes regulated by transcription factors in the IMIREG signature

tf	confidence	target	mor
IRF3	C	DCAF15	1
IRF3	C	FOXK2	1
IRF3	C	GLCCI1	1
IRF3	C	IFNL1	1
IRF3	C	KCNAB2	1
IRF3	C	KMT5C	1
IRF3	C	LMBR1L	1
IRF3	C	PGP	1
IRF3	C	RHPN1	1
IRF3	C	SPSB3	1
IRF3	C	TEPSIN	1
IRF3	C	TMEM160	1
IRF3	C	TRIP10	1
IRF3	C	ZNF574	1
IRF5	E	TNF	1
IRF5	E	IFNA1	1
IRF5	E	ACSS1	1
IRF5	E	ACTR10	1
IRF5	E	ACY3	1
IRF5	E	ADAP2	1
IRF5	E	ADARB2	1
IRF5	E	ADORA1	1
IRF5	E	ADPGK	1
IRF5	E	AHR	1
IRF5	E	AHRR	1
IRF5	E	AKAP11	1
IRF5	E	AKAP8	1
IRF5	E	AKR1B1	1
IRF5	E	ALDH3B1	1
IRF5	E	AMER1	1
IRF5	E	ANKRD34A	1
IRF5	E	ANKRD7	1
IRF5	E	ANO5	1
IRF5	E	APOC4-A…	1
IRF5	E	ARAF	1
IRF5	E	ARHGAP1…	1
IRF5	E	ARHGAP1…	1
IRF5	E	ARHGAP27	1
IRF5	E	ARHGEF1	1
IRF5	E	ATF3	1
IRF5	E	ATP10A	1
IRF5	E	ATP13A1	1
IRF5	E	ATP9B	1
IRF5	E	BCL7B	1
IRF5	E	BFSP1	1
IRF5	E	BICDL2	1
IRF5	E	BPNT1	1
IRF5	E	BRME1	1
IRF5	E	BROX	1
IRF5	E	BST2	1
IRF5	E	BTK	1
IRF5	E	C12orf75	1
IRF5	E	C17orf97	1
IRF5	E	C19orf44	1
IRF5	E	C1QTNF6	1
IRF5	E	C6orf62	1
IRF5	E	C9orf135	1
IRF5	E	CA13	1
IRF5	E	CALR3	1
IRF5	E	CARD16	1
IRF5	E	CARD17	1
IRF5	E	CARD9	1
IRF5	E	CASP1	1
IRF5	E	CC2D1A	1
IRF5	E	CCDC167	1
IRF5	E	CCDC30	1
IRF5	E	CCDC34	1
IRF5	E	CCDC42	1
IRF5	E	CCDC69	1
IRF5	E	CCDC88C	1
IRF5	E	CCR3	1
IRF5	E	CCR6	1
IRF5	E	CD300C	1
IRF5	E	CD5	1
IRF5	E	CD68	1
IRF5	E	CD86	1
IRF5	E	CDC42BPB	1
IRF5	E	CDH11	1
IRF5	E	CEBPA	1
IRF5	E	CEBPG	1
IRF5	E	CEP131	1
IRF5	E	CEP43	1
IRF5	E	CFAP44	1
IRF5	E	CFAP91	1
IRF5	E	CFI	1
IRF5	E	CGGBP1	1
IRF5	E	CHEK2	1
IRF5	E	CHRNA10	1
IRF5	E	CHST12	1
IRF5	E	CIP2A	1
IRF5	E	CKS1B	1
IRF5	E	CLDN1	1
IRF5	E	CLDN9	1
IRF5	E	CNOT9	1
IRF5	E	CNTN1	1
IRF5	E	COL26A1	1
IRF5	E	COLEC11	1
IRF5	E	COX7A1	1
IRF5	E	CPVL	1
IRF5	E	CRX	1
IRF5	E	CSE1L	1
IRF5	E	CSF3R	1
IRF5	E	CTNND2	1
IRF5	E	CTTN	1

Table S4: Comprehensive list of target genes regulated by transcription factors in the IMIREG signature

tf	confidence	target	mor
IRF5	E	CYFIP2	1
IRF5	E	CYP4F2	1
IRF5	E	CYP4F3	1
IRF5	E	CYTH1	1
IRF5	E	DAGLB	1
IRF5	E	DDAH1	1
IRF5	E	DDX42	1
IRF5	E	DDX55	1
IRF5	E	DDX60	1
IRF5	E	DDX60L	1
IRF5	E	DEF8	1
IRF5	E	DHX37	1
IRF5	E	DHX58	1
IRF5	E	DIS3L2	1
IRF5	E	DNAJA1	1
IRF5	E	DPEP2	1
IRF5	E	DRD5	1
IRF5	E	DTX3L	1
IRF5	E	DUT	1
IRF5	E	DUX4	1
IRF5	E	DYNLRB1	1
IRF5	E	ECE1	1
IRF5	E	EHF	1
IRF5	E	EIF2B4	1
IRF5	E	ELAVL2	1
IRF5	E	ENC1	1
IRF5	E	EPHA8	1
IRF5	E	EPSTI1	1
IRF5	E	ERCC5	1
IRF5	E	ETHE1	1
IRF5	E	ETV5	1
IRF5	E	EVI5	1
IRF5	E	EZH1	1
IRF5	E	EZR	1
IRF5	E	FABP7	1
IRF5	E	FAM107B	1
IRF5	E	FAM131A	1
IRF5	E	FAM167A	1
IRF5	E	FAM25A	1
IRF5	E	FAM47E	1
IRF5	E	FANCA	1
IRF5	E	FAR2	1
IRF5	E	FASTKD3	1
IRF5	E	FBLN7	1
IRF5	E	FBRS	1
IRF5	E	FBXO17	1
IRF5	E	FBXO6	1
IRF5	E	FBXW9	1
IRF5	E	FCGR2A	1
IRF5	E	FCRL2	1
IRF5	E	FGD2	1
IRF5	E	FGFR2	1
IRF5	E	FIS1	1
IRF5	E	FKBP11	1
IRF5	E	FMR1	1
IRF5	E	FZD3	1
IRF5	E	GALNT9	1
IRF5	E	GATA6	1
IRF5	E	GBP6	1
IRF5	E	GGT6	1
IRF5	E	GJC1	1
IRF5	E	GLYCTK	1
IRF5	E	GPHN	1
IRF5	E	GPR84	1
IRF5	E	GRXCR1	1
IRF5	E	GTF2I	1
IRF5	E	H2AB2	1
IRF5	E	H2BC5	1
IRF5	E	H4C14	1
IRF5	E	H4C7	1
IRF5	E	HAT1	1
IRF5	E	HECW2	1
IRF5	E	HERPUD1	1
IRF5	E	HEXD	1
IRF5	E	HEYL	1
IRF5	E	HLA-C	1
IRF5	E	HMGN3	1
IRF5	E	HOMEZ	1
IRF5	E	HSH2D	1
IRF5	E	HSP90AA1	1
IRF5	E	HYLS1	1
IRF5	E	ICE2	1
IRF5	E	IFI27	1
IRF5	E	IFI44	1
IRF5	E	IFIT1	1
IRF5	E	IFIT5	1
IRF5	E	IFITM3	1
IRF5	E	IKBKE	1
IRF5	E	IKZF4	1
IRF5	E	IL13RA2	1
IRF5	E	IL4I1	1
IRF5	E	IL6R	1
IRF5	E	ING4	1
IRF5	E	INVS	1
IRF5	E	ISG15	1
IRF5	E	ITGAX	1
IRF5	E	ITSN1	1
IRF5	E	JAG1	1
IRF5	E	KANSL3	1
IRF5	E	KATNAL1	1
IRF5	E	KCNE4	1
IRF5	E	KCNJ6	1
IRF5	E	KCNMB1	1
IRF5	E	KCP	1

Table S4: Comprehensive list of target genes regulated by transcription factors in the IMIREG signature

tf	confidence	target	mor
IRF5	E	KDM5B	1
IRF5	E	KLHL18	1
IRF5	E	KLHL21	1
IRF5	E	KLHL7	1
IRF5	E	KRTCAP2	1
IRF5	E	LARS2	1
IRF5	E	LIPA	1
IRF5	E	LIPG	1
IRF5	E	LLGL1	1
IRF5	E	LNP1	1
IRF5	E	LNPEP	1
IRF5	E	LRP5L	1
IRF5	E	LRRC29	1
IRF5	E	LRRC74A	1
IRF5	E	LSM6	1
IRF5	E	LTA	1
IRF5	E	LYL1	1
IRF5	E	MAGOH	1
IRF5	E	MAJIN	1
IRF5	E	MAP2K2	1
IRF5	E	MAP2K3	1
IRF5	E	MAP3K10	1
IRF5	E	MAP3K13	1
IRF5	E	MAP3K21	1
IRF5	E	MAPK8IP2	1
IRF5	E	MBD1	1
IRF5	E	MBNL1	1
IRF5	E	MBOAT1	1
IRF5	E	MED31	1
IRF5	E	MFSD11	1
IRF5	E	MIA2	1
IRF5	E	MICAL3	1
IRF5	E	MID2	1
IRF5	E	MLEC	1
IRF5	E	MLPH	1
IRF5	E	MORN2	1
IRF5	E	MRPL14	1
IRF5	E	MRPL39	1
IRF5	E	MRPS12	1
IRF5	E	MSX2	1
IRF5	E	MTFR1L	1
IRF5	E	MTHFD1L	1
IRF5	E	MTMR4	1
IRF5	E	MX1	1
IRF5	E	MYO1F	1
IRF5	E	NCF1	1
IRF5	E	NCK2	1
IRF5	E	NCOR1	1
IRF5	E	NDST3	1
IRF5	E	NDUFS7	1
IRF5	E	NECTIN4	1
IRF5	E	NIBAN2	1
IRF5	E	NINJ1	1
IRF5	E	NLRC5	1
IRF5	E	NPAT	1
IRF5	E	NR5A2	1
IRF5	E	NRDC	1
IRF5	E	NSMCE4A	1
IRF5	E	NSUN2	1
IRF5	E	NUP50	1
IRF5	E	NYAP2	1
IRF5	E	NYNRIN	1
IRF5	E	OAS2	1
IRF5	E	OAS3	1
IRF5	E	OAZ1	1
IRF5	E	OGFR	1
IRF5	E	OR2V2	1
IRF5	E	OSMR	1
IRF5	E	OTOGL	1
IRF5	E	PARP15	1
IRF5	E	PARP9	1
IRF5	E	PARVG	1
IRF5	E	PBX1	1
IRF5	E	PCDHGC4	1
IRF5	E	PCNA	1
IRF5	E	PEA15	1
IRF5	E	PFKFB2	1
IRF5	E	PFKL	1
IRF5	E	PHF19	1
IRF5	E	PIGR	1
IRF5	E	PIP4K2C	1
IRF5	E	PKP4	1
IRF5	E	PLAAT2	1
IRF5	E	PLEKHA2	1
IRF5	E	PLPPR3	1
IRF5	E	PLXNA1	1
IRF5	E	PMFBP1	1
IRF5	E	POFUT2	1
IRF5	E	POLR3B	1
IRF5	E	POLR3GL	1
IRF5	E	PPM1M	1
IRF5	E	PPP1R12A	1
IRF5	E	PRKCZ	1
IRF5	E	PRR29	1
IRF5	E	PRSS54	1
IRF5	E	PSRC1	1
IRF5	E	PTPN6	1
IRF5	E	RAB36	1
IRF5	E	RAP1B	1
IRF5	E	RASAL3	1
IRF5	E	RASIP1	1
IRF5	E	RBM24	1
IRF5	E	RBM26	1
IRF5	E	RBM43	1

Table S4: Comprehensive list of target genes regulated by transcription factors in the IMIREG signature

tf	confidence	target	mor
IRF5	E	RBM48	1
IRF5	E	RCCD1	1
IRF5	E	RFX5	1
IRF5	E	RGPD1	1
IRF5	E	RGS19	1
IRF5	E	RHBDF2	1
IRF5	E	RIC8B	1
IRF5	E	RNF121	1
IRF5	E	RNF122	1
IRF5	E	RNF213	1
IRF5	E	RPA2	1
IRF5	E	RPS15A	1
IRF5	E	RTN4	1
IRF5	E	RTP4	1
IRF5	E	RUFY2	1
IRF5	E	S100A2	1
IRF5	E	SAMD15	1
IRF5	E	SAMD9	1
IRF5	E	SARS2	1
IRF5	E	SC5D	1
IRF5	E	SCARB2	1
IRF5	E	SCMH1	1
IRF5	E	SCRN2	1
IRF5	E	SEC24C	1
IRF5	E	SEC61G	1
IRF5	E	SFI1	1
IRF5	E	SFPQ	1
IRF5	E	SFT2D3	1
IRF5	E	SHC1	1
IRF5	E	SIAE	1
IRF5	E	SIRT2	1
IRF5	E	SLC30A10	1
IRF5	E	SLC38A3	1
IRF5	E	SLC44A2	1
IRF5	E	SLC6A5	1
IRF5	E	SLC6A9	1
IRF5	E	SLCO5A1	1
IRF5	E	SMAD3	1
IRF5	E	SMC6	1
IRF5	E	SNIP1	1
IRF5	E	SNX17	1
IRF5	E	SNX8	1
IRF5	E	SORBS3	1
IRF5	E	SOWAHD	1
IRF5	E	SPA17	1
IRF5	E	SPHK1	1
IRF5	E	SPI1	1
IRF5	E	SPIRE1	1
IRF5	E	SPPL2B	1
IRF5	E	SPTLC2	1
IRF5	E	SREBF1	1
IRF5	E	SRP54	1
IRF5	E	SRRM1	1
IRF5	E	STAT2	1
IRF5	E	SWT1	1
IRF5	E	SYK	1
IRF5	E	SYNGR3	1
IRF5	E	SYNJ2	1
IRF5	E	SYNJ2BP	1
IRF5	E	SYNJ2BP…	1
IRF5	E	SYT14	1
IRF5	E	SYT4	1
IRF5	E	TAS2R42	1
IRF5	E	TBR1	1
IRF5	E	TCEA2	1
IRF5	E	TDRD7	1
IRF5	E	TENM2	1
IRF5	E	TEX264	1
IRF5	E	TFAP2A	1
IRF5	E	THAP3	1
IRF5	E	THRAP3	1
IRF5	E	TINAG	1
IRF5	E	TMEM182	1
IRF5	E	TMEM208	1
IRF5	E	TMTC1	1
IRF5	E	TNFAIP2	1
IRF5	E	TNFRSF1…	1
IRF5	E	TNFRSF14	1
IRF5	E	TNFSF11	1
IRF5	E	TNFSF13	1
IRF5	E	TRAF5	1
IRF5	E	TRIM11	1
IRF5	E	TRIM14	1
IRF5	E	TRIM21	1
IRF5	E	TRIM46	1
IRF5	E	TRIO	1
IRF5	E	TRMT1L	1
IRF5	E	TRMU	1
IRF5	E	TRUB2	1
IRF5	E	TSC2	1
IRF5	E	TSPAN18	1
IRF5	E	TSPAN33	1
IRF5	E	TTC30B	1
IRF5	E	TTC7A	1
IRF5	E	TTLL5	1
IRF5	E	TYROBP	1
IRF5	E	UBE2D3	1
IRF5	E	UBL5	1
IRF5	E	UHRF2	1
IRF5	E	USP15	1
IRF5	E	USP45	1
IRF5	E	VCX2	1
IRF5	E	WDR90	1
IRF5	E	WDTC1	1

Table S4: Comprehensive list of target genes regulated by transcription factors in the IMIREG signature

tf	confidence	target	mor
IRF5	E	WSCD1	1
IRF5	E	XAGE3	1
IRF5	E	XAGE5	1
IRF5	E	XIRP2	1
IRF5	E	XPC	1
IRF5	E	XRCC6	1
IRF5	E	XRN1	1
IRF5	E	ZBP1	1
IRF5	E	ZBTB24	1
IRF5	E	ZBTB6	1
IRF5	E	ZC3H4	1
IRF5	E	ZMYND15	1
IRF5	E	ZNF18	1
IRF5	E	ZNF260	1
IRF5	E	ZNF287	1
IRF5	E	ZNF532	1
IRF5	E	ZNF544	1
IRF5	E	ZNF568	1
IRF5	E	ZNF674	1
IRF5	E	ZNFX1	1
IRF5	E	ZNRF2	1
IRF5	E	ZYX	1
KMT2B	D	ABCB9	1
KMT2B	D	AC00648…	1
KMT2B	D	ACOX3	1
KMT2B	D	AP4B1	1
KMT2B	D	ARHGEF7	1
KMT2B	D	ARID1A	1
KMT2B	D	ATM	1
KMT2B	D	ATP5MG	1
KMT2B	D	ATXN2L	1
KMT2B	D	BCLAF1	1
KMT2B	D	BLK	1
KMT2B	D	BRWD1	1
KMT2B	D	BUB1	1
KMT2B	D	C12ORF73	1
KMT2B	D	C1ORF109	1
KMT2B	D	CARF	1
KMT2B	D	CBLL1	1
KMT2B	D	CCAR1	1
KMT2B	D	CCDC12	1
KMT2B	D	CCDC77	1
KMT2B	D	CCNT2	1
KMT2B	D	CD53	1
KMT2B	D	CD81	1
KMT2B	D	CDC45	1
KMT2B	D	CNOT1	1
KMT2B	D	CNR2	1
KMT2B	D	COA1	1
KMT2B	D	COMMD1	1
KMT2B	D	COPB1	1
KMT2B	D	COPS7B	1
KMT2B	D	CTD	1
KMT2B	D	CTDNEP1	1
KMT2B	D	CYB5D2	1
KMT2B	D	DHX9	1
KMT2B	D	DOCK8	1
KMT2B	D	EIPR1	1
KMT2B	D	ELP5	1
KMT2B	D	EP300	1
KMT2B	D	ESF1	1
KMT2B	D	EXOSC3	1
KMT2B	D	GPBP1L1	1
KMT2B	D	INTS4	1
KMT2B	D	IRAG2	1
KMT2B	D	IRF8	1
KMT2B	D	ISG20	1
KMT2B	D	KMT2A	1
KMT2B	D	LAMTOR5	1
KMT2B	D	LINC009…	1
KMT2B	D	LINC013…	1
KMT2B	D	MCM7	1
KMT2B	D	MOB3A	1
KMT2B	D	MRPS27	1
KMT2B	D	MYBL1	1
KMT2B	D	NSUN6	1
KMT2B	D	NUP160	1
KMT2B	D	NUP88	1
KMT2B	D	OGFOD2	1
KMT2B	D	PCBP2	1
KMT2B	D	PCM1	1
KMT2B	D	PIPOX	1
KMT2B	D	PISD	1
KMT2B	D	PNPLA6	1
KMT2B	D	POLE	1
KMT2B	D	PPP1R37	1
KMT2B	D	PPP6R1	1
KMT2B	D	PRPF40A	1
KMT2B	D	PSMA3	1
KMT2B	D	RBM39	1
KMT2B	D	RECQL5	1
KMT2B	D	RHO	1
KMT2B	D	RIC8A	1
KMT2B	D	RNU7-1	1
KMT2B	D	RPL6	1
KMT2B	D	RRM2B	1
KMT2B	D	SF3B1	1
KMT2B	D	SF3B3	1
KMT2B	D	SMARCD2	1
KMT2B	D	SMC1A	1
KMT2B	D	SMC4	1
KMT2B	D	SNORA4.3	1
KMT2B	D	SNX1	1
KMT2B	D	SNX8	1

Table S4: Comprehensive list of target genes regulated by transcription factors in the IMIREG signature

tf	confidence	target	mor
KMT2B	D	SUPT5H	1
KMT2B	D	SYNE2	1
KMT2B	D	SYVN1	1
KMT2B	D	TARDBP	1
KMT2B	D	TATDN3	1
KMT2B	D	TMEM126A	1
KMT2B	D	TMEM69	1
KMT2B	D	TNRC6B	1
KMT2B	D	TPR	1
KMT2B	D	UBC	1
KMT2B	D	USP39	1
KMT2B	D	USP48	1
KMT2B	D	WASHC4	1
KMT2B	D	WRAP53	1
KMT2B	D	XRCC5	1
KMT2B	D	ZC3H12D	1
KMT2B	D	ZC3H7A	1
KMT2B	D	ZCCHC10	1
KMT2B	D	ZNF106	1
KMT2B	D	ZNF263	1
KMT2B	D	ZNF569	1
KMT2B	D	ZNF570	1
KMT2B	D	ZNF668	1
KMT2B	D	ZNF773	1
SP100	E	APOBEC3D	1
SP100	E	APOBEC3G	1
SP100	E	APOL1	1
SP100	E	APOL6	1
SP100	E	B2M	1
SP100	E	BLMH	1
SP100	E	BST2	1
SP100	E	BTN3A1	1
SP100	E	BTN3A2	1
SP100	E	BTN3A3	1
SP100	E	CARD16	1
SP100	E	CASKIN1	1
SP100	E	CASP1	1
SP100	E	CASP4	1
SP100	E	CASP8	1
SP100	E	CAV2	1
SP100	E	CDH24	1
SP100	E	CERS1	1
SP100	E	CIR1	1
SP100	E	CMPK2	1
SP100	E	DAPP1	1
SP100	E	DDX58	1
SP100	E	DDX60	1
SP100	E	DDX60L	1
SP100	E	DTX3L	1
SP100	E	EHD1	1
SP100	E	EPSTI1	1
SP100	E	FAH	1
SP100	E	GAPT	1
SP100	E	GBP1	1
SP100	E	GBP3	1
SP100	E	GIMAP4	1
SP100	E	GRAMD1C	1
SP100	E	HERC5	1
SP100	E	HERC6	1
SP100	E	HLA-B	1
SP100	E	HLA-DMA	1
SP100	E	HLA-E	1
SP100	E	HLA-F	1
SP100	E	HLA-G	1
SP100	E	IDH2	1
SP100	E	IFI16	1
SP100	E	IFI35	1
SP100	E	IFI44	1
SP100	E	IFI44L	1
SP100	E	IFI6	1
SP100	E	IFIH1	1
SP100	E	IFIT1	1
SP100	E	IFIT2	1
SP100	E	IFIT3	1
SP100	E	IFIT5	1
SP100	E	IFITM1	1
SP100	E	IFITM3	1
SP100	E	IL15	1
SP100	E	IPO4	1
SP100	E	IRF2	1
SP100	E	IRF9	1
SP100	E	ISG15	1
SP100	E	LAMP3	1
SP100	E	LCP2	1
SP100	E	LGALS3	1
SP100	E	LGALS3BP	1
SP100	E	LGALS9C	1
SP100	E	MBD2	1
SP100	E	MX1	1
SP100	E	MYOF	1
SP100	E	NCEH1	1
SP100	E	NMI	1
SP100	E	NT5DC2	1
SP100	E	NUB1	1
SP100	E	OAS1	1
SP100	E	OAS2	1
SP100	E	ODF3B	1
SP100	E	OPTN	1
SP100	E	PARP12	1
SP100	E	PARP14	1
SP100	E	PARP9	1
SP100	E	PARS2	1
SP100	E	PHF11	1
SP100	E	PLSCR1	1

Table S4: Comprehensive list of target genes regulated by transcription factors in the IMIREG signature

tf	confidence	target	mor
SP100	E	PPM1M	1
SP100	E	PSMB8	1
SP100	E	PSMB9	1
SP100	E	RBM14	1
SP100	E	RBM43	1
SP100	E	RRAS	1
SP100	E	RSAD2	1
SP100	E	RTP4	1
SP100	E	SAMD9	1
SP100	E	SAMD9L	1
SP100	E	SCAP	1
SP100	E	SERPINB6	1
SP100	E	SLC12A3	1
SP100	E	SP110	1
SP100	E	SP140L	1
SP100	E	STAT1	1
SP100	E	TAP1	1
SP100	E	TDRD7	1
SP100	E	THOP1	1
SP100	E	TICAM2	1
SP100	E	TLR2	1
SP100	E	TRIM21	1
SP100	E	TRIM22	1
SP100	E	TRIM34	1
SP100	E	TRIM38	1
SP100	E	UBA7	1
SP100	E	UBE2L6	1
SP100	E	USP15	1
SP100	E	XAF1	1
SP100	E	ZNF77	1
SP100	E	ZNFX1	1
SP110	E	APOL1	1
SP110	E	APOL2	1
SP110	E	APOL6	1
SP110	E	B2M	1
SP110	E	BATF2	1
SP110	E	CASP1	1
SP110	E	CASP4	1
SP110	E	CMPK2	1
SP110	E	DDX58	1
SP110	E	DDX60	1
SP110	E	DDX60L	1
SP110	E	DHX58	1
SP110	E	DTX3L	1
SP110	E	EIF2AK2	1
SP110	E	EPSTI1	1
SP110	E	HERC6	1
SP110	E	HLA-B	1
SP110	E	HLA-E	1
SP110	E	HSH2D	1
SP110	E	IFI35	1
SP110	E	IFI44	1
SP110	E	IFI44L	1
SP110	E	IFI6	1
SP110	E	IFIH1	1
SP110	E	IFIT1	1
SP110	E	IFIT2	1
SP110	E	IFIT3	1
SP110	E	IFIT5	1
SP110	E	IFITM3	1
SP110	E	IRF7	1
SP110	E	IRF9	1
SP110	E	ISG15	1
SP110	E	MVP	1
SP110	E	MX1	1
SP110	E	MX2	1
SP110	E	NMI	1
SP110	E	NUB1	1
SP110	E	OAS1	1
SP110	E	OAS2	1
SP110	E	OAS3	1
SP110	E	OASL	1
SP110	E	PARP10	1
SP110	E	PARP12	1
SP110	E	PARP14	1
SP110	E	PARP9	1
SP110	E	PLSCR1	1
SP110	E	PSMB9	1
SP110	E	PSME1	1
SP110	E	RSAD2	1
SP110	E	RTP4	1
SP110	E	SAMD9	1
SP110	E	SAMD9L	1
SP110	E	SFXN1	1
SP110	E	SHFL	1
SP110	E	SP100	1
SP110	E	SP140L	1
SP110	E	SQOR	1
SP110	E	STAT1	1
SP110	E	TAP1	1
SP110	E	TAP2	1
SP110	E	TNFSF10	1
SP110	E	TRIM14	1
SP110	E	TRIM21	1
SP110	E	TRIM22	1
SP110	E	TRIM34	1
SP110	E	TRIM38	1
SP110	E	TRIM5	1
SP110	E	UBE2L6	1
SP110	E	USP18	1
SP110	E	XAF1	1
SP110	E	ZBP1	1
SP110	E	ZNFX1	1
SP140	E	ACAP1	1

Table S4: Comprehensive list of target genes regulated by transcription factors in the IMIREG signature

tf	confidence	target	mor
SP140	E	ADGRG5	1
SP140	E	AIM2	1
SP140	E	APOBEC3D	1
SP140	E	APOBEC3G	1
SP140	E	APOL1	1
SP140	E	APOL3	1
SP140	E	ARHGAP30	1
SP140	E	ARHGAP9	1
SP140	E	ARHGDIB	1
SP140	E	ASB2	1
SP140	E	BANK1	1
SP140	E	BATF	1
SP140	E	BIN2	1
SP140	E	BLK	1
SP140	E	BTK	1
SP140	E	BTLA	1
SP140	E	BTN2A2	1
SP140	E	BTN3A1	1
SP140	E	BTN3A3	1
SP140	E	C5orf58	1
SP140	E	CALHM6	1
SP140	E	CARD16	1
SP140	E	CARMIL2	1
SP140	E	CCL5	1
SP140	E	CCR5	1
SP140	E	CD19	1
SP140	E	CD2	1
SP140	E	CD247	1
SP140	E	CD27	1
SP140	E	CD37	1
SP140	E	CD38	1
SP140	E	CD3D	1
SP140	E	CD3E	1
SP140	E	CD3G	1
SP140	E	CD48	1
SP140	E	CD5	1
SP140	E	CD52	1
SP140	E	CD53	1
SP140	E	CD6	1
SP140	E	CD7	1
SP140	E	CD72	1
SP140	E	CD79A	1
SP140	E	CD79B	1
SP140	E	CD80	1
SP140	E	CD86	1
SP140	E	CD96	1
SP140	E	CIITA	1
SP140	E	CLEC12A	1
SP140	E	CLEC4C	1
SP140	E	CORO1A	1
SP140	E	CST7	1
SP140	E	CTLA4	1
SP140	E	CTSS	1
SP140	E	CXCL10	1
SP140	E	CXCL11	1
SP140	E	CXCL13	1
SP140	E	CXCR3	1
SP140	E	CXCR6	1
SP140	E	CYTIP	1
SP140	E	DAPP1	1
SP140	E	DAZL	1
SP140	E	DOK2	1
SP140	E	DPPA4	1
SP140	E	DUSP2	1
SP140	E	EPSTI1	1
SP140	E	ERMN	1
SP140	E	ETV7	1
SP140	E	EVI2B	1
SP140	E	FCRL2	1
SP140	E	FCRL3	1
SP140	E	FCRL5	1
SP140	E	FCRLA	1
SP140	E	FGD2	1
SP140	E	FOXP3	1
SP140	E	FUT7	1
SP140	E	GBP4	1
SP140	E	GBP5	1
SP140	E	GPR171	1
SP140	E	GPR174	1
SP140	E	GPR18	1
SP140	E	GPR55	1
SP140	E	GZMA	1
SP140	E	GZMK	1
SP140	E	HCST	1
SP140	E	HLA-DMA	1
SP140	E	HLA-DOB	1
SP140	E	HLA-DQA1	1
SP140	E	HLA-DRB5	1
SP140	E	HLA-E	1
SP140	E	HLA-F	1
SP140	E	HNMT	1
SP140	E	HSF5	1
SP140	E	HSH2D	1
SP140	E	ICOS	1
SP140	E	IDO1	1
SP140	E	IKZF1	1
SP140	E	IL10RA	1
SP140	E	IL12RB1	1
SP140	E	IL15	1
SP140	E	IL21R	1
SP140	E	IL27	1
SP140	E	IL2RA	1
SP140	E	IL2RB	1
SP140	E	IL2RG	1

Table S4: Comprehensive list of target genes regulated by transcription factors in the IMIREG signature

tf	confidence	target	mor
SP140	E	IL32	1
SP140	E	IL9R	1
SP140	E	IRF1	1
SP140	E	IRF4	1
SP140	E	IRF8	1
SP140	E	ITGAL	1
SP140	E	ITGB7	1
SP140	E	ITK	1
SP140	E	JAK3	1
SP140	E	JAKMIP1	1
SP140	E	KCNJ10	1
SP140	E	KIR3DL2	1
SP140	E	KLHL6	1
SP140	E	KLRC1	1
SP140	E	LAG3	1
SP140	E	LAIR2	1
SP140	E	LAMP3	1
SP140	E	LCK	1
SP140	E	LCP2	1
SP140	E	LILRB1	1
SP140	E	LILRB4	1
SP140	E	LIMD2	1
SP140	E	LPXN	1
SP140	E	LSP1	1
SP140	E	LTA	1
SP140	E	LTB	1
SP140	E	LY75	1
SP140	E	LY9	1
SP140	E	MAP4K1	1
SP140	E	MCOLN2	1
SP140	E	MEI1	1
SP140	E	MYO1G	1
SP140	E	NCF1	1
SP140	E	NCR3	1
SP140	E	NKG7	1
SP140	E	NUGGC	1
SP140	E	P2RY10	1
SP140	E	PARP15	1
SP140	E	PATL2	1
SP140	E	PCED1B	1
SP140	E	PDCD1	1
SP140	E	PLA2G2D	1
SP140	E	POU2AF1	1
SP140	E	PSTPIP1	1
SP140	E	PTPN22	1
SP140	E	PTPN7	1
SP140	E	PTPRC	1
SP140	E	PTPRCAP	1
SP140	E	PVRIG	1
SP140	E	PYHIN1	1
SP140	E	RAB33A	1
SP140	E	RAC2	1
SP140	E	RASAL3	1
SP140	E	RGL4	1
SP140	E	RHOH	1
SP140	E	RTP5	1
SP140	E	RUBCNL	1
SP140	E	RUFY4	1
SP140	E	S1PR4	1
SP140	E	SAMD3	1
SP140	E	SAMD9L	1
SP140	E	SASH3	1
SP140	E	SCIMP	1
SP140	E	SCML4	1
SP140	E	SELL	1
SP140	E	SELPLG	1
SP140	E	SEPTIN1	1
SP140	E	SH2D1A	1
SP140	E	SIGLEC10	1
SP140	E	SIRPG	1
SP140	E	SIT1	1
SP140	E	SLA2	1
SP140	E	SLAMF1	1
SP140	E	SLAMF6	1
SP140	E	SLAMF7	1
SP140	E	SLAMF8	1
SP140	E	SNX20	1
SP140	E	SP110	1
SP140	E	SP140L	1
SP140	E	SPIB	1
SP140	E	SPN	1
SP140	E	STAP1	1
SP140	E	STAT4	1
SP140	E	SUSD3	1
SP140	E	TAGAP	1
SP140	E	TAP1	1
SP140	E	TBC1D10C	1
SP140	E	TBX21	1
SP140	E	TCL1A	1
SP140	E	TESPA1	1
SP140	E	TIFAB	1
SP140	E	TIGIT	1
SP140	E	TIMD4	1
SP140	E	TLR10	1
SP140	E	TLR8	1
SP140	E	TMC8	1
SP140	E	TMEM156	1
SP140	E	TMEM268	1
SP140	E	TNFRSF1…	1
SP140	E	TNFSF13B	1
SP140	E	TRAF3IP3	1
SP140	E	TRAT1	1
SP140	E	TRIM22	1
SP140	E	TTC24	1

Table S4: Comprehensive list of target genes regulated by transcription factors in the IMIREG signature

tf	confidence	target	mor
SP140	E	UBASH3A	1
SP140	E	VAV1	1
SP140	E	WAS	1
SP140	E	ZAP70	1
SP140	E	ZBP1	1
SP140	E	ZBTB32	1
SP140	E	ZC3H12D	1
STAT1	A	A2M	1
STAT1	A	ADAR	1
STAT1	A	AIM2	1
STAT1	A	AOX1	1
STAT1	A	APOE	1
STAT1	A	APOL4	1
STAT1	A	APP	1
STAT1	A	ATG4C	1
STAT1	A	B3GAT3	1
STAT1	A	BAX	1
STAT1	A	BCL2	1
STAT1	A	BCL6	1
STAT1	A	BRD8	1
STAT1	A	BST2	1
STAT1	A	CASP4	1
STAT1	A	CCL2	1
STAT1	A	CCL3	1
STAT1	A	CCND1	1
STAT1	A	CCND2	1
STAT1	A	CCND3	1
STAT1	A	CCR1	1
STAT1	A	CCR5	1
STAT1	A	CD274	1
STAT1	A	CD40	1
STAT1	A	CD86	1
STAT1	A	CDKN1A	1
STAT1	A	CEBPE	1
STAT1	A	CFTR	1
STAT1	A	CIITA	1
STAT1	A	CISH	1
STAT1	A	CLC	1
STAT1	A	CLIC2	1
STAT1	A	COL1A1	1
STAT1	A	COL1A2	1
STAT1	A	CREM	1
STAT1	A	CSF1	1
STAT1	A	CTSB	1
STAT1	A	CTSL	1
STAT1	A	CX3CL1	1
STAT1	A	CXCL10	1
STAT1	A	CXCL9	1
STAT1	A	CYP19A1	1
STAT1	A	DCLRE1C	1
STAT1	A	DDX50	1
STAT1	A	DIMT1	1
STAT1	A	DUOX2	1
STAT1	A	EDEM2	1
STAT1	A	EDN1	1
STAT1	A	EGFR	1
STAT1	A	EGLN3	1
STAT1	A	EIF2AK1	1
STAT1	A	EIF2AK2	1
STAT1	A	EIF2AK3	1
STAT1	A	ELF5	1
STAT1	A	ELP3	1
STAT1	A	ESR1	1
STAT1	A	ESR2	1
STAT1	A	ETV7	1
STAT1	A	FAS	1
STAT1	A	FBXO5	1
STAT1	A	FCGR1A	1
STAT1	A	FCGR3A	1
STAT1	A	FGF2	1
STAT1	A	FOS	1
STAT1	A	FOXP3	1
STAT1	A	GATA3	1
STAT1	A	GBP1	1
STAT1	A	GCK	1
STAT1	A	GLS	1
STAT1	A	GREB1	1
STAT1	A	GSTK1	1
STAT1	A	HDAC7	1
STAT1	A	HLA-DMA	1
STAT1	A	HLA-DMB	1
STAT1	A	HLA-DQA2	1
STAT1	A	HLA-DRB1	1
STAT1	A	HLA-E	1
STAT1	A	HMOX1	1
STAT1	A	HSPB1	1
STAT1	A	ICAM1	1
STAT1	A	IDO1	1
STAT1	A	IFI35	1
STAT1	A	IFI6	1
STAT1	A	IFIT1	1
STAT1	A	IFIT3	1
STAT1	A	IFITM1	1
STAT1	A	IFITM3	1
STAT1	A	IFNLR1	1
STAT1	A	IGF1	1
STAT1	A	IGFBP3	1
STAT1	A	IGFLR1	1
STAT1	A	IL10	1
STAT1	A	IL12A	1
STAT1	A	IL12B	1
STAT1	A	IL12RB1	1
STAT1	A	IL12RB2	1
STAT1	A	IL17D	1

Table S4: Comprehensive list of target genes regulated by transcription factors in the IMIREG signature

tf	confidence	target	mor
STAT1	A	IL18BP	1
STAT1	A	IL1B	1
STAT1	A	IL1R1	1
STAT1	A	IL2RA	1
STAT1	A	IL6ST	1
STAT1	A	INO80	1
STAT1	A	IRF1	1
STAT1	A	IRF2	1
STAT1	A	IRF7	1
STAT1	A	IRF8	1
STAT1	A	IRF9	1
STAT1	A	ISG15	1
STAT1	A	ISG20	1
STAT1	A	JAK2	1
STAT1	A	JUN	1
STAT1	A	JUNB	1
STAT1	A	KIF2A	1
STAT1	A	KRT17	1
STAT1	A	LAP3	1
STAT1	A	LY96	1
STAT1	A	MAN1A1	1
STAT1	A	MAT2A	1
STAT1	A	MCL1	1
STAT1	A	MED16	1
STAT1	A	MET	1
STAT1	A	MTMR9	1
STAT1	A	MUC1	1
STAT1	A	MUC4	1
STAT1	A	MVP	1
STAT1	A	MX1	1
STAT1	A	MYC	1
STAT1	A	MYD88	1
STAT1	A	NMI	1
STAT1	A	NOS2	1
STAT1	A	NOX5	1
STAT1	A	OAS1	1
STAT1	A	OAS2	1
STAT1	A	OAS3	1
STAT1	A	OASL	1
STAT1	A	OPRM1	1
STAT1	A	PARP14	1
STAT1	A	PIM1	1
STAT1	A	PLAU	1
STAT1	A	PLSCR1	1
STAT1	A	PML	1
STAT1	A	PPARA	1
STAT1	A	PPIP5K2	1
STAT1	A	PRF1	1
STAT1	A	PROS1	1
STAT1	A	PSMB9	1
STAT1	A	PTGFR	1
STAT1	A	PTGS2	1
STAT1	A	PTTG1IP	1
STAT1	A	PWP2	1
STAT1	A	RBAK	1
STAT1	A	REV3L	1
STAT1	A	RIPK1	1
STAT1	A	RNF213	1
STAT1	A	S100A10	1
STAT1	A	SBNO2	1
STAT1	A	SCARB1	1
STAT1	A	SEMA4B	1
STAT1	A	SLC2A2	1
STAT1	A	SMARCA4	1
STAT1	A	SNRNP27	1
STAT1	A	SOCS1	1
STAT1	A	SOCS2	1
STAT1	A	SOCS3	1
STAT1	A	SOCS4	1
STAT1	A	SOCS5	1
STAT1	A	SOCS6	1
STAT1	A	SOCS7	1
STAT1	A	STAT2	1
STAT1	A	STAT3	1
STAT1	A	TAP1	1
STAT1	A	TBX21	1
STAT1	A	TFF1	1
STAT1	A	TH	1
STAT1	A	TIMP1	1
STAT1	A	TNFRSF8	1
STAT1	A	TNFSF11	1
STAT1	A	TNFSF13B	1
STAT1	A	TP53	1
STAT1	A	TRIM25	1
STAT1	A	UBR1	1
STAT1	A	UPP1	1
STAT1	A	VEGFA	1
STAT1	A	VEGFC	1
STAT1	A	VEGFD	1
STAT1	A	VIP	1
STAT1	A	VPS33B	1
STAT1	A	WARS1	1
STAT1	A	ZC3HAV1	1
STAT1	A	ZFP1	1
STAT1	A	ZFP30	1
STAT1	A	ZNF107	1
STAT1	A	ZNF112	1
STAT1	A	ZNF114	1
STAT1	A	ZNF12	1
STAT1	A	ZNF124	1
STAT1	A	ZNF140	1
STAT1	A	ZNF155	1
STAT1	A	ZNF157	1
STAT1	A	ZNF169	1

Table S4: Comprehensive list of target genes regulated by transcription factors in the IMIREG signature

tf	confidence	target	mor
STAT1	A	ZNF175	1
STAT1	A	ZNF181	1
STAT1	A	ZNF184	1
STAT1	A	ZNF19	1
STAT1	A	ZNF200	1
STAT1	A	ZNF205	1
STAT1	A	ZNF222	1
STAT1	A	ZNF226	1
STAT1	A	ZNF227	1
STAT1	A	ZNF23	1
STAT1	A	ZNF230	1
STAT1	A	ZNF235	1
STAT1	A	ZNF25	1
STAT1	A	ZNF251	1
STAT1	A	ZNF254	1
STAT1	A	ZNF267	1
STAT1	A	ZNF268	1
STAT1	A	ZNF273	1
STAT1	A	ZNF282	1
STAT1	A	ZNF302	1
STAT1	A	ZNF311	1
STAT1	A	ZNF331	1
STAT1	A	ZNF333	1
STAT1	A	ZNF337	1
STAT1	A	ZNF33B	1
STAT1	A	ZNF343	1
STAT1	A	ZNF354A	1
STAT1	A	ZNF398	1
STAT1	A	ZNF41	1
STAT1	A	ZNF417	1
STAT1	A	ZNF418	1
STAT1	A	ZNF419	1
STAT1	A	ZNF425	1
STAT1	A	ZNF460	1
STAT1	A	ZNF490	1
STAT1	A	ZNF510	1
STAT1	A	ZNF519	1
STAT1	A	ZNF529	1
STAT1	A	ZNF546	1
STAT1	A	ZNF550	1
STAT1	A	ZNF567	1
STAT1	A	ZNF585A	1
STAT1	A	ZNF589	1
STAT1	A	ZNF596	1
STAT1	A	ZNF599	1
STAT1	A	ZNF613	1
STAT1	A	ZNF619	1
STAT1	A	ZNF630	1
STAT1	A	ZNF655	1
STAT1	A	ZNF669	1
STAT1	A	ZNF670	1
STAT1	A	ZNF680	1
STAT1	A	ZNF689	1
STAT1	A	ZNF713	1
STAT1	A	ZNF721	1
STAT1	A	ZNF746	1
STAT1	A	ZNF764	1
STAT1	A	ZNF777	1
STAT1	A	ZNF778	1
STAT1	A	ZNF782	1
STAT1	A	ZNF786	1
STAT1	A	ZNF79	1
STAT1	A	ZNF8	1
STAT1	A	ZNF81	1
STAT1	A	ZNF84	1
STAT1	A	ZNF860	1
STAT1	A	ZNF875	1
STAT1	A	ZNF878	1
STAT1	A	ZNF879	1
ZBED2	E	ACAP1	1
ZBED2	E	ADAMDEC1	1
ZBED2	E	ADGRA3	1
ZBED2	E	AHNAK2	1
ZBED2	E	ANXA2	1
ZBED2	E	ANXA8L1	1
ZBED2	E	ARNTL2	1
ZBED2	E	BCAR3	1
ZBED2	E	BTLA	1
ZBED2	E	C10orf55	1
ZBED2	E	CAV1	1
ZBED2	E	CAV2	1
ZBED2	E	CCL18	1
ZBED2	E	CCL5	1
ZBED2	E	CCR5	1
ZBED2	E	CD2	1
ZBED2	E	CD27	1
ZBED2	E	CD300LF	1
ZBED2	E	CD3D	1
ZBED2	E	CD3E	1
ZBED2	E	CD7	1
ZBED2	E	CD8B	1
ZBED2	E	CD96	1
ZBED2	E	CHST11	1
ZBED2	E	CTLA4	1
ZBED2	E	CXCL10	1
ZBED2	E	CXCL13	1
ZBED2	E	CXCL9	1
ZBED2	E	CXCR3	1
ZBED2	E	EHD1	1
ZBED2	E	EHD2	1
ZBED2	E	FOXP3	1
ZBED2	E	GBP5	1
ZBED2	E	GFI1	1
ZBED2	E	GSDME	1

Table S4: Comprehensive list of target genes regulated by transcription factors in the IMIREG signature

tf	confidence	target	mor
ZBED2	E	HK3	1
ZBED2	E	ICOS	1
ZBED2	E	IDO1	1
ZBED2	E	IFNG	1
ZBED2	E	IL21R	1
ZBED2	E	IL2RB	1
ZBED2	E	IL2RG	1
ZBED2	E	IL4I1	1
ZBED2	E	KCNJ11	1
ZBED2	E	KLHDC7B	1
ZBED2	E	LAG3	1
ZBED2	E	LAMA3	1
ZBED2	E	LAMC2	1
ZBED2	E	LCK	1
ZBED2	E	LTA	1
ZBED2	E	LYPD5	1
ZBED2	E	MYO1G	1
ZBED2	E	NCF2	1
ZBED2	E	NT5E	1
ZBED2	E	PDCD1	1
ZBED2	E	PLA2G2D	1
ZBED2	E	PPP1R14C	1
ZBED2	E	PTPN22	1
ZBED2	E	PTPN7	1
ZBED2	E	PTPRCAP	1
ZBED2	E	RALB	1
ZBED2	E	REP15	1
ZBED2	E	RNF144B	1
ZBED2	E	SERPINB7	1
ZBED2	E	SIRPG	1
ZBED2	E	SIT1	1
ZBED2	E	SLAMF8	1
ZBED2	E	SPN	1
ZBED2	E	TAP1	1
ZBED2	E	TAP2	1
ZBED2	E	TBC1D2	1
ZBED2	E	TIGIT	1
ZBED2	E	TLR8	1
ZBED2	E	TNFRSF1…	1
ZBED2	E	TNFRSF9	1
ZBED2	E	TTC24	1
ZBED2	E	WNT10A	1
ZBED2	E	ZNF620	1
ZBTB32	E	ACAP1	1
ZBTB32	E	ADAM8	1
ZBTB32	E	ARHGAP45	1
ZBTB32	E	ARHGAP9	1
ZBTB32	E	CARMIL2	1
ZBTB32	E	CCL22	1
ZBTB32	E	CCL5	1
ZBTB32	E	CD19	1
ZBTB32	E	CD2	1
ZBTB32	E	CD27	1
ZBTB32	E	CD37	1
ZBTB32	E	CD3D	1
ZBTB32	E	CD3E	1
ZBTB32	E	CD6	1
ZBTB32	E	CD7	1
ZBTB32	E	CD79A	1
ZBTB32	E	CTLA4	1
ZBTB32	E	CXCL13	1
ZBTB32	E	CXCR5	1
ZBTB32	E	DEF6	1
ZBTB32	E	FCER2	1
ZBTB32	E	FCMR	1
ZBTB32	E	FCRL2	1
ZBTB32	E	FCRL3	1
ZBTB32	E	FCRL5	1
ZBTB32	E	FMNL1	1
ZBTB32	E	FOXP3	1
ZBTB32	E	GZMM	1
ZBTB32	E	HEATR9	1
ZBTB32	E	ICOS	1
ZBTB32	E	IL21R	1
ZBTB32	E	IL9R	1
ZBTB32	E	IRF4	1
ZBTB32	E	ISG20	1
ZBTB32	E	JAK3	1
ZBTB32	E	LAG3	1
ZBTB32	E	LAT	1
ZBTB32	E	LAX1	1
ZBTB32	E	LILRB1	1
ZBTB32	E	LTA	1
ZBTB32	E	MAP4K1	1
ZBTB32	E	MMP25	1
ZBTB32	E	MYO1G	1
ZBTB32	E	NIBAN3	1
ZBTB32	E	NUGGC	1
ZBTB32	E	OASL	1
ZBTB32	E	PDCD1	1
ZBTB32	E	PSTPIP1	1
ZBTB32	E	PTPN7	1
ZBTB32	E	PTPRCAP	1
ZBTB32	E	PVRIG	1
ZBTB32	E	RASAL3	1
ZBTB32	E	RELT	1
ZBTB32	E	S1PR4	1
ZBTB32	E	SEPTIN1	1
ZBTB32	E	SIRPG	1
ZBTB32	E	SIT1	1
ZBTB32	E	SLAMF1	1
ZBTB32	E	SLAMF7	1
ZBTB32	E	SOCS6	1
ZBTB32	E	SP140	1

Table S4: Comprehensive list of target genes regulated by transcription factors in the IMIREG signature

tf	confidence	target	mor
ZBTB32	E	SPATC1	1
ZBTB32	E	TBC1D10C	1
ZBTB32	E	TIGIT	1
ZBTB32	E	TNFRSF1…	1
ZBTB32	E	TNFRSF4	1
ZBTB32	E	TRAF3IP3	1
ZBTB32	E	TTC16	1
ZBTB32	E	WAS	1
ZBTB32	E	ZAP70	1
ZBTB32	E	ZBP1	1
ZNF80	E	ACAP1	1
ZNF80	E	ARHGAP15	1
ZNF80	E	ARHGAP9	1
ZNF80	E	CD2	1
ZNF80	E	CD3E	1
ZNF80	E	CD5	1
ZNF80	E	CD6	1
ZNF80	E	CD96	1
ZNF80	E	CTLA4	1
ZNF80	E	CXCL13	1
ZNF80	E	CYTIP	1
ZNF80	E	EOMES	1
ZNF80	E	FCRL3	1
ZNF80	E	GFI1	1
ZNF80	E	GZMK	1
ZNF80	E	ICOS	1
ZNF80	E	IKZF1	1
ZNF80	E	IL2RA	1
ZNF80	E	ITGAL	1
ZNF80	E	ITK	1
ZNF80	E	JAKMIP1	1
ZNF80	E	KLRK1	1
ZNF80	E	MAP4K1	1
ZNF80	E	NLRP1	1
ZNF80	E	PARP15	1
ZNF80	E	PDCD1	1
ZNF80	E	PYHIN1	1
ZNF80	E	RHOH	1
ZNF80	E	SEPTIN1	1
ZNF80	E	SH2D1A	1
ZNF80	E	SIRPG	1
ZNF80	E	SIT1	1
ZNF80	E	SLA2	1
ZNF80	E	SLAMF1	1
ZNF80	E	TBC1D10C	1
ZNF80	E	THEMIS	1
ZNF80	E	TIGIT	1
ZNF80	E	TRAF3IP3	1
ZNF80	E	TRAT1	1
ZNF80	E	ZAP70	1
ZNF80	E	ZNF831	1

CELLMARKERCAN_ACINAR_CELLS		CTRB1	KLK1	RBPJL	PTF1A	CELA3A	PRSS1	SPINK1	ZG16	CEL	CELA2A	CPB1	CELA1	RNASE1	AMY2B	CPA2	CPA1	CELA3B	PNLIP	CTRB2	PLA2G1B	PRSS2	CLPS	REG1A	SYCN
CELLMARKERCAN_ADIPOCYTE_PROGENITOR_CELLS		NA	FGF10	WT1	SCARA5	HOXC8	TCF7L1	HOXC9	EBF2	NRG4															
CELLMARKERCAN_ADRENERGIC_NEURONS		TH	DDC	DBH	PNMT	SLC18A2	NPFF	SLC12A7	SYT1																
CELLMARKERCAN_AIRWAY_EPITHELIAL_CELLS		AQP1	ADH7	CDH1	SEC14L3																				
CELLMARKERCAN_ALPHA_CELLS		GCG	TTR	NA	ARX	RESP18	PYY	PCSK2	SLC38A5	TM4SF4	CRYBA2	NKX6-1	IRX2	ALDH1A1	PEMT	CHGA	SMIM24	F10	SCGN	SLC30A8	SH3GL2	SCGB2A1	FAP	DPP4	GPR119
CELLMARKERCAN_ALVEOLAR_MACROPHAGES		NA	ITGAX	MRC1	MARCO	S100A4	GNGT2																		
CELLMARKERCAN_ASTROCYTES		S100B	SLC1A2	GFAP	BYSL	ALDH1L1	CD40	SLC1A3	FGFR3	GJB6	AQP4	HTRA1	S100A8	CLDN10	ITIH3	NKAIN4	NA	ACSBG1	TRIL	AGT	GPR37L1	SLC4A4	NTSR2	SLC6A11	HSPA2
CELLMARKERCAN_B_CELLS		NA	CD5	MS4A1	CR2	CD22	FCER2	CD40	CD79A	CD79B	CD80	CD86	TNFRSF13C	IGHD	IGHM	HLA-DRA	MME	SPN	CD19	CD24	CD27	CD72	PAX5	LY6D	FCMR
CELLMARKERCAN_B_CELLS_MEMORY		CD38	CD80	CD84	CD86	NT5E	PAX5	PTPRC	SPIB	ADAM28	AIM2	ALOX5	BACH2	BANK1	BLK	CCR6	CD180	CD19	CD1C	CD22	CD27	CD37	CD69	CD72	CD79A
CELLMARKERCAN_B_CELLS_NAIVE		CD19	NA	CD72	ABCB4	ADAM28	BACH2	BANK1	BCL7A	BEND5	BLK	BRAF	CD180	CD1C	CD22	CD37	CD69	CD79A	CD79B	CR2	CXCR5	EAF2	FCER2	FCGR2B	FCRL2
CELLMARKERCAN_BASOPHILS		CD69	IL3RA	CCR3	ENPP3	FCER1A	TLR4	LTC4S	CPA3	ITGAL	ITGAM	ITGAX	IL4R	ITGB7	IL4	MS4A2	MS4A3	HDC	GATA2	CCL3	CD33	CD44	CDH1	HTR1B	IGFBP7
CELLMARKERCAN_BERGMANN_GLIA		GFAP	GDF10	GJB6	GRIA4	GRM3	NA	ITIH3	A2M	PTCH1	CDC42EP4	BTBD17	AQP4	SLC4A4	HOPX	GPR37L1	ACSBG1	ALDH1L1	SLC1A3	SLC1A2	S100B	VIM	FABP7	TTC21B	HEPACAM
CELLMARKERCAN_BETA_CELLS		GCGR	JPH3	CD40	HAMP	EZH1	NTRK1	PDX1	SLC2A2	NKX6-2	FXYD2	NA	INS	RIMS1	MAFA	EFNA5	LMX1A	NKX2-2	NKX6-1	PAX4	IAPP	PCSK2	G6PC2	SLC30A8	PCSK1
CELLMARKERCAN_CAJAL_RETZIUS_CELLS		RELN	NA	EBF3	CALB2	EOMES	CNR1	HAR1A																	
CELLMARKERCAN_CARDIAC_STEM_AND_PRECURSOR_…		NA	NKX2-5	ISL1	MESP1	ADGRL2	TBX3	TBX4	WNT5A	ANKRD1															
CELLMARKERCAN_CARDIOMYOCYTES		TNNC1	ADM	ALCAM	NPPA	FGF2	NPPB	TNNI3	TNNT2	ANKRD1	NPPC	DES	HAND2	HAND1	GATA4	GATA6	MYL2	NA	PNMT	ACTA1	CD36	VLDLR	ALPK3	RBM24	FABP4
CELLMARKERCAN_CHOLANGIOCYTES		SSTR2	SLC4A2	SCTR	KRT7	KCNN2	ITGB4	GGT6	CFTR	CCKBR	AQP4	AQP1	PIGR	GGT1	JAG1	GPBAR1	GGT7	KRT19	ONECUT2	ALPL	HNF1B	ALB	AGR2	TFF3	TFF1
CELLMARKERCAN_CHOLINERGIC_NEURONS		CHAT	SLC18A3	ACHE	SLC5A7	TAC1	BRCA1	ACLY																	
CELLMARKERCAN_CHONDROCYTES		PRG4	TNC	CRTAC1	CREB3L2	RUNX2	ACAN	COL2A1	COMP	MATN3	RUNX3	SOX6	BMP2	NA	FGF18	GLI3	PBX3	PTHLH	SMO	STAT1	MMP13	VEGFA	MATN1	COL9A1	COL10A1
CELLMARKERCAN_CHOROID_PLEXUS_CELLS		TTR	KL	SOSTDC1	CLIC6	SLC4A10	NA	AQP1	SLC26A11	CHMP1A															
CELLMARKERCAN_CILIATED_CELLS		SEC14L3	NA	FOXJ1	TUBB4B																				
CELLMARKERCAN_DELTA_CELLS		PDX1	FFAR4	SST	PCSK9	RBP4	PAX4	RESP18	HHEX	ISL1	SCGN	KCNK16	IAPP	FXYD2	NPTX2	GPC5-AS1	UCP2	LEPR	BAIAP3	MS4A8	CASR	BCHE	GABRB3	UNC5B	EDN3
CELLMARKERCAN_DOPAMINERGIC_NEURONS		TH	SLC6A3	NA	DDC	SLC18A2	SLC18A3	NEUROD6	SMAD3	PITX3															
CELLMARKERCAN_DUCTAL_CELLS		CFTR	HNF1B	KRT20	MUC1	NA	AMBP	HHEX	ANXA4	SPP1	PDX1	SERPINA3	CFB	GDF15	AKR1C3	MMP7	DEFB1	SERPING1	TSPAN8	CLDN10	SLPI	SERPINA5	PIGR	CLDN1	LGALS4
CELLMARKERCAN_EMBRYONIC_STEM_CELLS		NA	FOXD3	POU5F1	FUT4	CD24	CDH1	TEX19	NANOG	SALL4	TDGF1	GJC1	TRIM6	PUM2	GJB4	SOX15	UTF1	GJB1	LMNA	FBXO15	PML	THY1	KIT	EPCAM	CD9
CELLMARKERCAN_ENTERIC_GLIA_CELLS		GFRA3	NA	SOX10	GFAP	S100B	NOS2	FOXD3	ALDH1A3	SLC18A2															
CELLMARKERCAN_ENTERIC_NEURONS		NA	TAC1	GAL	CARTPT	NMU	VIP	CCK																	
CELLMARKERCAN_ENTEROCYTES		GATA4	LYPD8	NLRP6	KRT20	MYO1A	PPARG	SLC15A1	FLOT2	APOBEC1	NA	SLC10A2	SLC40A1	SLC5A1	TTC7A	NPC1L1	FABP1	ANPEP	CA1	CPS1	DPP4	SLC2A5	ALPI	FABP2	LCT
CELLMARKERCAN_ENTEROENDOCRINE_CELLS		NPSR1	FFAR3	SCN3A	NEUROG3	FFAR1	ARX	NKX2-2	SCT	CCK	GFRA3	VWA5B2	NEUROD1	SCGN	PCSK1	ISL1	FABP5	TAC1	RIMBP2	PAX6	GIP	GLP1R	PYY	GLP2R	GAST
CELLMARKERCAN_EPENDYMAL_CELLS		FOXJ1	TRIM71	SIX3	USP18	AQP4	CELSR2	AQP1	GFAP	ENKUR	NA	KRT15	ODF3B	CROCC	HSPA2	CALML4	HDC	PCP4L1	RIIAD1	TMEM212	CCDC153	S100B	AK7	RARRES2	PVALB
CELLMARKERCAN_EPSILON_CELLS		GHRL	ANXA13	PHGR1	BMP4	HMGCS2	TM4SF5	OLFML3	ASGR1	COX6A2	NPY1R	FAXDC2	SLC7A9	C2ORF54	GHRLOS	BHMT	OPRK1	PTGER4	ITGA4	EYA4	XYLB	ELOVL2	AFF3	CORIN	MYO1A
CELLMARKERCAN_FOLLICULAR_CELLS		NA	TG	IYD	PRLR	TPO	DIO1	SLC26A7	SLC16A11	FOXE1															
CELLMARKERCAN_FOVEOLAR_CELLS		NA	MUC5AC	TFF1	CLDN18	KRT20	LGALS4	AKR1B10	IL33																
CELLMARKERCAN_GAMMA_DELTA_T_CELLS		GNLY	PTGDS	GZMB	S100B	FGFBP2	NKG7	PRF1	KLRF1	HOPX	CST7	KLRD1	CTSW	SPON2	IFITM1	GZMA	CD247	CLIC3	CD7	ADGRG1	CCL5	TRDC	STMN1	HMGB2	TYMS
CELLMARKERCAN_GERM_CELLS		NA	POU5F1	TFAP2C	DAZL	DDX4	ZAR1	PRM1	TNP2	ODF2	SPATA18	PGK2	CABS1	H1FNT	GAPDHS	PRM3	TNP1	SMCP	PRM2	FKBP6	TAF7L	SYCP1	SOHLH1	SLC25A31	PHF13
CELLMARKERCAN_GLUTAMINERGIC_NEURONS		SLC17A7	SLC17A6	NA	MEIS2	SLC17A8	SLC1A1	SLC1A2	SLC1A6																
CELLMARKERCAN_GRANULOSA_CELLS		AMH	MCAM	NA	INHA	FSHR	FOXL2	CYP19A1																	
CELLMARKERCAN_HEMATOPOIETIC_STEM_CELLS		THY1	MPL	KIT	PROM1	FLT3	SLAMF1	CXCR4	TEK	ABCB1	ABCG2	BAALC	SPN	CDCP1	MYADM	KITLG	TGFB2	PTPRE	TAF7	GATA2	GPR171	ANGPT1	PROCR	NFE2	NKX2-3
CELLMARKERCAN_IMMATURE_NEURONS		PAX6	PCLO	CUX2	SLC12A5	POMC	STIM2	RAX	CUL5	HIVEP2	FUT9	TBR1	PROX1	NTRK3	RND2	FEZF2	SOX5	ROBO1	EPHA3	YY2	SLC1A1	NOTCH3	NOTCH1	LHX6	NES
CELLMARKERCAN_INTERNEURONS		CALB1	CCK	CHAT	VSX2	EN1	ETV1	EVX1	GAD1	ISL1	LHX1	LHX5	LHX3	LHX6	GRM1	NPY	OPRM1	GRIN2D	NOS1	PVALB	PAX2	SST	TACR1	VIP	CRHR1
CELLMARKERCAN_IONOCYTES		FOXI1	CFTR	ASCL3	NA	FOXN4	TFCP2L1	SLC12A2	CLCNKB	KCNMA1															
CELLMARKERCAN_KERATINOCYTES		CALML5	CD24	FLG	NA	IVL	TGM1	KRT1	KRT5	KRT14	KRT17	LCN2	PLEC	SPRR1A	SPRR1B	GJB3	IRF3	LAMC2	SIRT7	RIPK4	REL	IL13RA1	SGPP1	PKP3	PKP1
CELLMARKERCAN_KUPFFER_CELLS		TNF	LYZ	NA	VSIG4	PROK2	STARD5	MSR1	TLR9	TREM1	TLR4	MMP13	CD14	VDR	CHIT1	OSM	CLEC4F	ADGRE1	SLC40A1	G6PD	TIMD4	DNASE1L3	MARCO	HFE	CD38
CELLMARKERCAN_LEYDIG_CELLS		HSD11B2	NA	ALDH1A1	SLC9A8	PDE8A	PTCH1	HSD3B1	CYP17A1	CYP11A1	IGF1	INSL3	LHCGR	STAR	THBS2	PTGDS	LCN2	HSD17B3	PRLR	IGF2	IGFBP5	CFD	DLK1	IGFBP3	INHBA
CELLMARKERCAN_LOOP_OF_HENLE_CELLS		CLDN16	NA	UMOD	FABP4	AQP1	CLDN10	CLDN19	SLC12A1	DEFB1	CLCNKA	RGS5	COX4I2	CYP4B1	PARVB	SH3RF2	HEY2	NKAIN4	ADAMTS5	CXCL1	GEM	FGA	PLA2G7	THBS1	ENC1
CELLMARKERCAN_LUMINAL_EPITHELIAL_CELLS		FGFR2	FGG	KRT18	ANKRD30A	SLPI	PROM1	KRT19	SYTL2	CD74	AGR2	LTF	SAA2	KRT23	SERPINB4	SERPINB3	WFDC2	LCN2	BTG1	CLDN4	ANXA1	HMGA1	STC2	AREG	TNFSF10
CELLMARKERCAN_LUTEAL_CELLS		AKT1	LHCGR	NA	PRLR																				
CELLMARKERCAN_MEGAKARYOCYTES		ITGA2B	GP1BA	ITGB3	MPL	LAT	PLEK	S100A9	S100A8	PF4	F13A1	CD9	EPOR	CXCR4	GP5	CXCR1	CXCR2	SLAMF1	PDE6B	MATK	SPON2	MKL2	TLR9	KIF5B	GATA1
CELLMARKERCAN_MELANOCYTES		NA	MC1R	PMEL	PTGDS	MLANA	MITF	TYR	DCT	GPNMB	HSPB1	PAX3	TRPM1	CTNS	ENPP1	SOX9	BNC2	MEN1	POMC	TFAP2A	RAB38	TP63	CRABP1	BMI1	PTN
CELLMARKERCAN_MESANGIAL_CELLS		NA	PDGFRB	ACTA2	TNS1	ANGPT2	GATA3	ITGA8	ARID5B	CCR7	FN1	PAWR	PTN	SERPINE2	TBX18	TEK	THY1	MYL9	TPM2	CALD1	HOPX	NR4A2	ACTN1	AKAP12	ROCK1
CELLMARKERCAN_MESOTHELIAL_CELLS		NA	MSLN	LRRN4	FRAS1	RSPO1																			
CELLMARKERCAN_MICROFOLD_CELLS		GP2	SPIB	PGLYRP1	EHF	ANXA5	SOX8	MIER3	NFIC	CXCL16	CTSD	CCL20	CTSH	MARCKSL1	RAC2	TNFAIP2	FABP5	GJB2	MFGE8	CYBA	TMPRSS2	AHR	MTF1	RELB	TULP4
CELLMARKERCAN_MICROGLIA		ITGAM	ITGAX	C5AR1	CX3CR1	P2RY12	P2RX7	CD40	TREM2	TMEM119	GPR34	NA	SALL1	TCIRG1	GPR183	VAV1	SLC2A5	LAG3	ENTPD1	ADRB2	P2RY6	P2RY13	CEBPA	CD180	OLFML3
CELLMARKERCAN_MONOCYTES		RGS1	APOBEC3A	CD7	TET2	CD40	DYSF	CMKLR1	MEFV	HCK	FCGR3B	PADI4	GHSR	ITGAX	SELE	TLR4	AR	CXCR4	CD86	CCR2	TNFRSF14	ADA2	MGMT	CD14	CD33
CELLMARKERCAN_MOTOR_NEURONS		ISL1	MNX1	NA																					
CELLMARKERCAN_MULLER_CELLS		TLR2	NA	SLC38A5	SLC38A3	IL33	RDH10	COL9A1	AQP4	RLBP1	DKK3	VSX2	COL1A1	COL2A1	COL3A1	COL4A2	COL5A1	COL6A1	COL11A1	GLUL	CLU	S100A16	SOX2	APOE	DBI
CELLMARKERCAN_MYELOID_DERIVED_SUPPRESSOR_…		NA	S100A9	S100A4																					
CELLMARKERCAN_MYOBLASTS		MYOG	LIF	NGF	IGF2	ANGPT2	ACHE	ADAM12	NA	DES	CDH15	MYF6	MYF5	MYOD1	PAX3	PAX7	TBX3	TTN	MUSK	FLNC	FGFR4	STK40	CXCL1	TNFRSF12A	CCL2
CELLMARKERCAN_MYOEPITHELIAL_CELLS		NA	KRT14	ACTG2	KRT5	MYLK	MYL9																		
CELLMARKERCAN_MYOFIBROBLASTS		NA	ACTA2	CALD1	MYL9	TAGLN																			
CELLMARKERCAN_NEUROBLASTS		NTNG1	TSHZ1	SP9	SP8	PROK2	ASCL1	DLL3	SALL3	DRAXIN	ERBB4	TRIM32	IGFBPL1	EOMES	PBX1	CUX2	ISL1	PHF1	NEUROG1	NKX6-1	NEUROG2	MAPK7	PROKR2	POU6F2	ITGA6
CELLMARKERCAN_NEUROENDOCRINE_CELLS		NA	SST	NPY	CCK	PRSS12																			
CELLMARKERCAN_NEURONS		ENO2	RBFOX3	CHAT	TH	CALB1	CALB2	ISL1	EPO	CSF3	NEFH	NEFM	DLG4	SLC12A5	GABRA1	AQP1	IFIT3	SLC11A1	TFF3	SLC1A1	CXCL1	CXCR5	AIM2	MATN2	SLC36A4
CELLMARKERCAN_NEUTROPHILS		MME	ITGAM	ITGAX	CD14	FUT4	FCGR3A	PECAM1	CD33	SELL	CEACAM8	C5AR1	CXCR1	CXCR2	JAML	TLR2	MYLK	S100A9	MPO	CD24	CEACAM1	NA	LCN2	DEFA1	DEFA3
CELLMARKERCAN_NK_CELLS		ITGAM	ITGAX	FCGR3A	CD69	KLRD1	IL2RB	KLRB1	CD244	KLRK1	SLAMF7	SIGLEC7	NCR1	SLAMF6	KIT	CD27	KLRC1	KLRF1	GNLY	NKG7	NA	GZMH	FGFBP2	GZMM	CTSW
CELLMARKERCAN_NORADRENERGIC_NEURONS		TH	DDC	DBH	SLC18A2	SLC18A3	SLC6A2	SLC39A11	SLC9B2																
CELLMARKERCAN_NUOCYTES		ICOS	IL1RL1	NA	IL7R	CRLF2	ARG1	GATA3	IL5	IL13															
CELLMARKERCAN_OLIGODENDROCYTES		GALC	MOG	OLIG1	OLIG2	TF	OLIG3	SOX10	GJC2	GAMT	BCAS1	ENPP6	TMEM63A	GPR62	BACE1	CREB5	FGFR2	SLC25A38	HDAC11	TYRO3	S1PR5	ASPA	EPHB1	HEPACAM	SOX8
CELLMARKERCAN_OSTEOBLASTS		SLC6A6	VDR	LRP4	EGFL6	MMP13	GPER1	RUNX2	NF2	LRP5	PHEX	HIF1A	C5AR1	CXCL12	COL1A1	PLCD4	FOXO1	SOCS3	IL33	MAGED1	PARD6G	CD44	NLRP3	ALPL	TNFSF11
CELLMARKERCAN_OSTEOCLAST_PRECURSOR_CELLS		NA	TNFRSF11A	CYSLTR1	TLR4																				
CELLMARKERCAN_OSTEOCLASTS		SOST	ZBTB7A	SEMA4D	KCNMA1	TNFRSF11B	PTPRA	TNFRSF11A	MMP9	ACP5	RAB3B	ITPR3	NRP2	THRA	AGER	RGS18	PTH1R	LTB4R	TRPV5	SNX10	CTSK	OSCAR	SLC2A1	DCSTAMP	EGFL7
CELLMARKERCAN_OSTEOCYTES		SOST	PFN1	EFNB2	PDPN	FGF23																			
CELLMARKERCAN_PANETH_CELLS		DEFA5	MMP7	AGR2	DEFA1	DEFA6	CSF2	LYZ	PLA2G2A	PRSS1	GUCA2B	MUC2	NA	GUCA2A	REG3G	ITLN1	GPX2	FABP4	CFD	ADIPOQ	PNLIPRP2	DMBT1	NOD2	LGR4	RIPK2
CELLMARKERCAN_PARATHYROID_CHIEF_CELLS		KL	CASR	VDR	FGFR1	CYP27B1	LRP2	PTH	RAB3A																
CELLMARKERCAN_PERICYTES		PECAM1	PDGFRB	CSPG4	ANPEP	ACTA2	DES	RGS5	ABCC9	KCNJ8	CD248	DLK1	TEK	NOTCH3	NA	ANGPT1	ZIC1	HIGD1B	MCAM	COLEC11	VTN	STEAP4	ATP13A5	AOC3	ANGPT2
CELLMARKERCAN_PERITUBULAR_MYOID_CELLS		NA	MYH11	TPM1	TPM2	TPM4	MYL9	WNT2B																	
CELLMARKERCAN_PHOTORECEPTOR_CELLS		RCVRN	NA	RHO	CRX																				
CELLMARKERCAN_PLASMA_CELLS		SDC1	IRF4	CD38	PRDM1	CD93	CD27	ENPP1	KLF4	MS4A4A	PIAS1	CYCS	DIABLO	HTRA2	MS4A1	CD9	CD19	CD24	CD37	CD40	CD74	CXCR4	GZMB	TNFRSF17	TNFRSF13B
CELLMARKERCAN_PLASMACYTOID_DENDRITIC_CELLS		CD4	CD8A	NA	CD80	CD83	LILRA4	CD86	IL3RA	CCR7	CLEC4C	CXCR3	LAMP5	GPR183	PTCRA	ERN1	ETV6	MAP3K8	FAM129C	SPIB	GZMB	CXCR4	PACSIN1	TLR7	TLR9
CELLMARKERCAN_PODOCYTES		PODXL	ADAMTS13	LCN2	VEGFA	SLC2A4	NLRP3	SYNPO	NPHS1	NPHS2	LAMA3	FOXC2	WT1	CD80	DES	PTPRO	LMX1B	MYOC	CDH13	SEMA3G	THSD7A	MAFB	NA	CLIC5	RAB3B
CELLMARKERCAN_PROXIMAL_TUBULE_CELLS		AQP1	CLDN2	DPP4	ENPEP	SLC36A2	SLC5A1	SLC6A18	SLC6A19	SLC5A2	LRP2	PDZK1	MIOX	SLC34A1	TTC36	ASS1	PCK1	FUT9	KHK	SLC4A4	GLYAT	FBP1	SLC22A6	SLC22A12	NA
CELLMARKERCAN_PULMONARY_ALVEOLAR_TYPE_I_C…		MMP11	ICAM1	CTGF	SEMA3B	SCNN1B	SEMA3E	SCNN1G	SCNN1A	COL4A4	CYP4B1	RTKN2	CLIC5	AKAP5	GPRC5A	SEC14L3	COL4A3	CLDN18	AGER	HOPX	AQP5	EMP2	VEGFA	KRT7	IGFBP6
CELLMARKERCAN_PULMONARY_ALVEOLAR_TYPE_II_…		IRX1	EGFL6	CEBPA	NKX2-1	MUC1	CLDN18	AGER	LAMP3	SFTA2	SFTPD	SLC34A2	SFTPB	SFTPA1	SFTPC	NA	ADGRF5	ABCD3	SOAT1	ETV5	CD36	LPCAT1	NAPSA	ABCA3	S100G
CELLMARKERCAN_PURKINJE_NEURONS		CALB1	NA	GAD1	PCP4	SLC1A6	GRID2	GAD2																	
CELLMARKERCAN_RETICULOCYTES		RUVBL1	TFRC	UCP3	FOLR1	UBE2O	CD36	ITGA4	SLC11A2																
CELLMARKERCAN_RETINAL_GANGLION_CELLS		NA	POU4F2	ISL1	SLC17A6	RBPMS	POU4F1																		
CELLMARKERCAN_RETINAL_PROGENITOR_CELLS		PAX6	NA	LGR5	RAX	NOTCH1	HES1	MSI1	SOX9																
CELLMARKERCAN_SATELLITE_CELLS		PAX7	NA	PAX3	MYF5	CDH15	CXCR4	ITGA7	MET	CALCR	ASB5	SRXN1	BDKRB1	CDCP1	VCAM1	MYOD1	SOX8	SPRY1	CXCL1	HMOX1	TNFRSF12A	RUNX1	BARX2	TRIM32	VAMP2
CELLMARKERCAN_SCHWANN_CELLS		MPZ	ITGB4	NA	S100B	POU3F1	GFAP	MAG	OLIG1	NDRG1	CTHRC1	46274	AGR2	NGFR	CLDN19	PRX	BCAS1	ASPA	RXRG	SOX10	CMTM5	EGFL8	GFRA3	SEMA3B	MATN2
CELLMARKERCAN_SEROTONERGIC_NEURONS		NA	SLC6A4	FEV	TPH2	DDC	SLC18A2	ESM1	SLC22A3																
CELLMARKERCAN_SERTOLI_CELLS		KITLG	AMH	INHBB	SHBG	GATA1	GATA4	DHH	AR	GDNF	MAFB	GAMT	MEIG1	HES5	NF2	INHA	DES	NA	FASLG	TF	WT1	AARD	CST9	TMEM184A	WNT6
CELLMARKERCAN_SMOOTH_MUSCLE_CELLS		ACTA2	CNN1	HEXIM1	TAGLN	RGS5	WFDC1	NOTCH3	RPRM	DES	FHL2	FBLN5	LOX	NF2	ANGPT1	CRABP1	MYH11	FABP4	ACTC1	ANKRD1	NA	PDE4DIP	LMO7	MYH6	NEXN
CELLMARKERCAN_STROMAL_CELLS		NA	COL15A1	GDF10	COL4A1	BMP4	WNT2	BMP5																	
CELLMARKERCAN_T_CELLS		CD3D	CD3G	CD3E	NA	CD4	LTB	CXCR6	IL7R	LEF1	ITK	TRBC2	CD7	TRAC	CD69	IL32	CXCR4	CD2	CTSW	TXK	TRAF1	BATF3	GZMK	CD247	TRDC
CELLMARKERCAN_T_CYTOTOXIC_CELLS		NA	GZMB	TRAC																					
CELLMARKERCAN_T_FOLLICULAR_HELPER_CELLS		NA	ICOS	CD200	P2RX7																				
CELLMARKERCAN_T_MEMORY_CELLS		NA	CCR7	PTPRC	ENPP1																				
CELLMARKERCAN_TRANSIENT_CELLS		NA	PARM1	SYT7																					
CELLMARKERCAN_TRICHOCYTES		KRT86	DSG2	KRT81	DSG4	KRT37	HOXC13																		
CELLMARKERCAN_TRIGEMINAL_NEURONS		NA	PIEZO2	VAMP1	SCN10A																				
CELLMARKERCAN_TROPHOBLAST_CELLS		NA	KRT7	PERP	PARP1	ERVFRD-1	HLA-G																		
CELLMARKERCAN_TROPHOBLAST_PROGENITOR_CELLS		ISG20	FOXO4	TFAP2C																					
CELLMARKERCAN_TUFT_CELLS		SUCNR1	FABP1	POU2F3	CDHR2	AVIL	ESPN	LRMP	TRPM5	DCLK1	TAS1R3	SOX9	CAMK2B	GNAT3	IL25	PLCB2	GFI1B	ATOH1	ASIC5	KLF3	KLF6	DRD3	GNG13	NREP	RGS2
CELLMARKERCANENDOTHELIALCELLSAORTA		NA	BMX	EHD3																					
CELLMARKERCUR_N_ABNORMAL_MYELOID_CELL		PRTN3	ELANE	AZU1	CST7																				

Table S5: Cell state marker gene list

CELLMARKERCAN_ACINAR_CELLS		CTRB1	KLK1	RBPJL	PTF1A	CELA3A	PRSS1	SPINK1	ZG16	CEL	CELA2A	CPB1	CELA1	RNASE1	AMY2B	CPA2	CPA1	CELA3B	PNLIP	CTRB2	PLA2G1B	PRSS2	CLPS	REG1A	SYCN
CELLMARKERCUR_N_ABNORMAL_PLASMA_CELL		SDC1	CD38	NCAM1																					
CELLMARKERCUR_N_ACTIVATED_DENDRITIC_CELL		CCR7	BIRC3	CCL22	IDO1	LAMP3	OAS3	EBI3	CCL1																
CELLMARKERCUR_N_ACTIVATED_EFFECTOR_CELL		MKI67	IL32	CCL5	TNFRSF4	HLA-DRA																			
CELLMARKERCUR_N_ACTIVATED_HEPATIC_STELLAT…		COL3A1	COL1A2	COL1A1	ACTA2																				
CELLMARKERCUR_N_ACTIVATED_LANGERHANS_CELL		CD83	PIM3	PXDC1	MMP9	STK17B	ARL4C	C15orf48																	
CELLMARKERCUR_N_ACTIVATED_MACROPHAGE		PTPRC	CD68	MRC1																					
CELLMARKERCUR_N_ACTIVATED_MEMORY_T_CELL		CD69	IL2RA	GZMB																					
CELLMARKERCUR_N_ACTIVATED_NATURAL_KILLER_…		LILRB1	NCAM1	KLRC2	B3GAT1	CD2	CD69	NCR1																	
CELLMARKERCUR_N_ACTIVATED_REGULATORY_T_CE…		IL2RA	CCR4	CD4																					
CELLMARKERCUR_N_ADIPOCYTE_PROGENITOR_CELL		ALCAM	THY1	LUM	COL1A2	MGP	CFD	CCN5	APOD																
CELLMARKERCUR_N_ADRENAL_MEDULLA_CELL		CHGA	TH	DBH	PNMT	CHGB																			
CELLMARKERCUR_N_ADULT_SPERMATOGONIA		PLPPR3	DCAF4L1	EGR4	SIX1																				
CELLMARKERCUR_N_ADVENTITIAL_CELL		PECAM1	PTPRC	CD34	NCAM1	NT5E	CD44	CSPG4	THY1																
CELLMARKERCUR_N_AEROCYTE		EDNRB	HPGD	SOSTDC1																					
CELLMARKERCUR_N_AIRWAY_SMOOTH_MUSCLE_CELL		ACTA2	TAGLN	CNN1	LGR6	DES																			
CELLMARKERCUR_N_ALVEOLAR_CELL_TYPE_1		RTKN2	AGER	SFTPC	SLPI	EPCAM	PDPN																		
CELLMARKERCUR_N_ALVEOLAR_CELL_TYPE_2		AGER	SLPI	EPCAM	SFTPC	ABCA3	SFTPB																		
CELLMARKERCUR_N_ALVEOLAR_EPITHELIAL_CELL_…		AGER	PDPN	CAV1																					
CELLMARKERCUR_N_ALVEOLAR_EPITHELIAL_CELL_…		SFTPB	SFTPD	SFTPA1	SFTPC	HHIP																			
CELLMARKERCUR_N_ALVEOLAR_PNEUMOCYTE_TYPE_I		CLIC5	CAV1	CAV2																					
CELLMARKERCUR_N_ALVEOLAR_PNEUMOCYTE_TYPE_…		SFTA3	PGC	SFTPC	WIF1																				
CELLMARKERCUR_N_AMACRINE_CELL		CRABP1	CALB1	CHAT	GAD1	C1QL2	TFAP2B	GAD2																	
CELLMARKERCUR_N_AMNIOTIC_FLUID_STEM_CELL		CD44	NT5E	THY1																					
CELLMARKERCUR_N_ANTERIOR_CELL		PAX6	SIX1	DLX5																					
CELLMARKERCUR_N_ANTERIOR_HINDBRAIN_CELL		GBX2	IRX3	HOXA2	HOXB2																				
CELLMARKERCUR_N_ANTI_TUMOR_IMMUNE_CELL		PRF1	GZMH	GZMB																					
CELLMARKERCUR_N_ANTIGEN_PRESENTATION_CANC…		CAV1	CD74	CLU	SAA3P																				
CELLMARKERCUR_N_ASTROCYTE		GFAP	AQP4	AQP1	CD80	CD86	CD40	C5AR1	NFIA	ALDH1L1	S100B	SOX9	GJA1	SLC1A3	KCNJ10	LGR6	OLIG2	CHI3L1	MLC1	ID3	SLC14A1	MT1F	PRDM16…	DIO2	SLC1A2
CELLMARKERCUR_N_ATRIAL_CELL		NR2F2	KCNJ3	MYL7	MYH6																				
CELLMARKERCUR_N_ATRIOVENTRICULAR_CANAL_CE…		MEIS1	TGFB2	JUN	ISL1																				
CELLMARKERCUR_N_ATYPICAL_MEMORY_B_CELL		MS4A1	CD19	CD27	MME	CR2	FCRL5																		
CELLMARKERCUR_N_B_2_CELL		STK11	CDKN2A	FHIT	MYCL	CD79A	IGLC2																		
CELLMARKERCUR_N_B1_CELL		MS4A1	SPN	CD27	CD5																				
CELLMARKERCUR_N_B10_REGULATORY_B_CELL		CD24	CD19	CD38	CD27	CD5																			
CELLMARKERCUR_N_BASAL_CELL		KRT17	KRT5	KRT14	KRT1	KRT16	KRT6C	CDH3	KRT6B	CD44	KRT72	KRT15	TFAP2C	STAT3	KRT6A	CD38	MYLK	ACTA2	CD24	ESR1	KIT	PGR	CD151	TAFAZZIN	KRT19
CELLMARKERCUR_N_BASAL_EPITHELIAL_CELL		KRT14	ITGB4	ITGA6	SCPEP1	KRT5	KRT17	ITGB1																	
CELLMARKERCUR_N_BASAL_KERATINOCYTE		CXCL14	KRT14	KRT5																					
CELLMARKERCUR_N_BASAL_LIKE_CELL		KRT5	KRT14	S100A2																					
CELLMARKERCUR_N_BASAL_PROGENITOR_CELL		ITGA6	CD44	CD24	WIF1	ACTG2	MYLK																		
CELLMARKERCUR_N_BASOPHIL		FUT4	IL3RA	KIT	ENPP3	SMAD1	CD19	CD22	CD63	CD69	CCR3	PTPRC	MS4A2	CLC	CPA3	GATA2	SLC18…	CCR5	TPSAB1	PTGDR2	CD9	CD33	CD38	CD44	CEBPA
CELLMARKERCUR_N_BILIARY_CELL		CFTR	EPCAM	KRT7	KRT19																				
CELLMARKERCUR_N_BONE_MARROW_STEM_CELL		CD44	THY1	CD34	ALCAM	CD14	ITGB1	PTPRC	NT5E																
CELLMARKERCUR_N_BONE_SPECIFIC_CELL		BGLAP	BMP2	RUNX2	ALPL	SPP1																			
CELLMARKERCUR_N_BRAIN_CELL		FOXG1	ZIC3	NRP1	ZIC2																				
CELLMARKERCUR_N_CANCER_CELL		KRT19	ALDH1A1	CD44	MS4A1	ABCB5	CSPG4	MCAM	NGFR	SALL4	ERBB2	KLK3	FOLH1	CDH1	MUC1	KRT8	KRT18	EPCAM	CD24	PTPRC	IL3RA	CD33	COLEC10	TSHR	CCR7
CELLMARKERCUR_N_CANCER_STEM_LIKE_CELL		FUT4	CDH5	PROM1	CXCR4	CD44	ALDH1A1	ALCAM	ANPEP																
CELLMARKERCUR_N_CAPILLARY_CELL		IGFBP3	ARL15	RGCC	SPARC	SGK1	CA4	PRX	EMCN	FOS															
CELLMARKERCUR_N_CAR_T_CELL		BTK	PLCG2	NFKBIE	BIRC3	SF3B1	XPO1	RIPK1	MGA	CD19															
CELLMARKERCUR_N_CARDIAC_CELL		MYBPC3	TNNT2	NPPA																					
CELLMARKERCUR_N_CARDIOMYOCYTE		VCAM1	SIRPA	ALCAM	TNNI3	MYL7	MYH6	TNNT2	MYL2	MYH7	BMP4	FABP3	GATA4	GATA6	GJA4	GJA5	MEF2C	TBX5	HCN4	LRG1	NKX2-5	KCNJ2	NPPA	GJA1	DCN
CELLMARKERCUR_N_CARDIOSPHERE_DERIVED_CELL		TBX5	CXCL1	CXCL6	IL1B	NKX2-5	GATA4	CXCL8																	
CELLMARKERCUR_N_CARDIOVASCULAR_PROGENITOR…		FLT1	FLT4	NKX2-5	ISL1	PDGFRA	HAND1	ANPEP																	
CELLMARKERCUR_N_CAUDAL_CORTEX_CELL		NEFL	NPY	LPL																					
CELLMARKERCUR_N_CD14_MONOCYTE		S100A8	CD14	LYZ	FCN1																				
CELLMARKERCUR_N_CD16_MONOCYTE		LYZ	FCGR3A	FCN1																					
CELLMARKERCUR_N_CD4_CD28_T_CELL		ITGB2	B3GAT1	IL2RA	TNFRSF8	BCL2	FAS																		
CELLMARKERCUR_N_CD4_PERIPHERALLY_DERIVED_…		KLRB1	CD69	CD38	LAG3																				
CELLMARKERCUR_N_CD4_T_CELL		CD3E	CD4	CD3G	CD3D	CD8A	CD8B	CD40LG	PTPRC																
CELLMARKERCUR_N_CD56BRIGHT_NATURAL_KILLER…		CD82	SELL	CD55	KLRC1	TNFSF10																			
CELLMARKERCUR_N_CD56DIM_NATURAL_KILLER_CE…		CD58	CX3CR1	ATP1B3	BSG	B3GAT1	CXCR1	HLA-E																	
CELLMARKERCUR_N_CD8_T_CELL		CD8B	CD3E	CD8A	CD3D	PDCD1	CD3G	CD40LG	TBCC	ABT1	ARHGAP8	KAT6A	SLC16A7	LEPROTL1	ZNF609	TMC6	SRSF7	HAUS3	PRF1	RBM3	VAMP2	PPP1R2	ZEB1	ZNF22	TMEM259
CELLMARKERCUR_N_CENTRAL_MEMORY_T_CELL		CD8A	CD4	CD27	SELL	CCR7	PASK	IL7R	FAS	GZMA	CD3D	S100A4	ITGAE	CD69	CD28	HNRNPH1	KLF12	RPP38	PHC3	TRAF3I…	REPS1	SLC7A6	N4BP2L…	CDC14A	POLR2J2
CELLMARKERCUR_N_CENTRAL_MEMORY_T_HELPER_1…		CD4	CCR7	TBX21																					
CELLMARKERCUR_N_CENTRAL_NERVOUS_SYSTEM_ST…		CD34	PTPRC	PROM1	CD24																				
CELLMARKERCUR_N_CHANDELIER_CELL		NOG	UNC5B	COL15A1																					
CELLMARKERCUR_N_CHIEF_CELL		PGA3	PGA4	LIPF	PGA5	CBLIF	PGC																		
CELLMARKERCUR_N_CHONDROCYTE		COL2A1	SOX9	ACAN	COMP	MIA																			
CELLMARKERCUR_N_CHROMAFFIN_CELL		NCAM1	TH	CHGB	CHRNB4	NPY																			
CELLMARKERCUR_N_CIRCULATING_ANGIOGENIC_CE…		KDR	PROM1	CD34																					
CELLMARKERCUR_N_CIRCULATING_FETAL_CELL		TFRC	MCAM	CLDN6	KCNQ4	MMP14	F3	CTNNB1	TCF3	CRYL1	JUN	EN1	SELE	SOX13	PF4	HMGA1	CD68	PBLD	CD33	ATF4	PDGFC	SELPLG	CRABP1	TNC	SMAD3
CELLMARKERCUR_N_CIRCULATING_NATURAL_KILLE…		TBX21	FCGR3A	CX3CR1																					
CELLMARKERCUR_N_CIRCULATING_PRECURSOR_CELL		PTPRC	CD34	PROM1																					
CELLMARKERCUR_N_CIRCULATING_PROGENITOR_CE…		CD34	MCAM	PROM1	CD14																				
CELLMARKERCUR_N_CIRCULATING_T_FOLLICULAR_…		CXCR5	CD4	CCR6	CXCR3																				
CELLMARKERCUR_N_CIRCULATING_TUMOR_CELL		CEACAM5	MAGEA3	MCAM																					
CELLMARKERCUR_N_CLASS_SWITCHED_PLASMABLAST		CD27	CD38	CD19																					
CELLMARKERCUR_N_CLASSICAL_B_CELL		CD24	FCER2	CD38																					
CELLMARKERCUR_N_CLASSICAL_CANCER_ASSOCIAT…		MMP11	SFRP2	COL1A1	FAP																				
CELLMARKERCUR_N_CLASSICAL_MONOCYTE		CD14	FCGR3A	CD33	FUT4	IL3RA	CEACAM8	CD9	CD93	ITGB3	C5AR1	FCAR	PTPRC	BASP1	CXCL8	S100A9	IFITM3	S100A8	VNN2	IFIT3	ALOX5AP	S100A12	HLA-DQ…	IFI6	HLA-DRB1
CELLMARKERCUR_N_CLAUDIN_LOW_CELL		CLDN4	CLDN7	ZEB2	ZEB1	VIM	CDH1	CLDN3	TWIST1																
CELLMARKERCUR_N_COLLECTING_DUCTAL_CELL		AQP2	TMEM213	ATP6V1…	ATP6V0D2																				
CELLMARKERCUR_N_COLONCYTE		GUCA2A	CEACAM1	CA1																					
CELLMARKERCUR_N_COLORECTAL_STEM_CELL		ITGB1	ALDH1A1	LGR5	DCLK1	MSI1																			
CELLMARKERCUR_N_COLUMNAR_EPITHELIAL_CELL		KRT7	AGR2	KRT18	GDA																				
CELLMARKERCUR_N_COMMON_LYMPHOID_PROGENITO…		SPINK2	DNTT	CD34	CD38	MME																			
CELLMARKERCUR_N_COMMON_MYELOID_PROGENITOR…		CSF3R	CSF2RB	CD7	CD34	CD38	FLT3	MME	THY1																
CELLMARKERCUR_N_COMPLEMENT_SECRETING_CANC…		CFI	C3	CFD	CFH	CALCA																			
CELLMARKERCUR_N_CONE_PHOTORECEPTOR_CELL		PDE6C	PDE6H	GUCA1C	THRB	GRK7	GNGT2	RXRG	OPN1LW	ARR3															
CELLMARKERCUR_N_CONNECTING_TUBULAR_CELL		AKR1B1	KRT17	MUC6	STXBP6	CALB1																			
CELLMARKERCUR_N_CONVENTIONAL_CD4_T_CELL		CD3E	CD3G	CD3D																					
CELLMARKERCUR_N_CORNEAL_ENDOTHELIAL_CELL		CD200	GPC4	VEGFA	PROM1	FLT1																			
CELLMARKERCUR_N_CORNEAL_EPITHELIAL_CELL		KRT12	KRT14	TGFBI																					
CELLMARKERCUR_N_CORNEOCYTE_LIKE_CELL		IL1RN	LYPD2	FXYD3																					
CELLMARKERCUR_N_CORTICAL_CELL		TBR1	BCL11B	SLC17A7	SLC17A6	LY75																			
CELLMARKERCUR_N_CORTICAL_STROMAL_CELL		SERPIN…	DES	COL3A1	DCN																				
CELLMARKERCUR_N_CORTICOTROPH_TUMOR_CELL		PITX1	CHGB	PCSK1N	EPCAM	SCG2	TBX19	RASD1	POMC																
CELLMARKERCUR_N_CRANIAL_NEURAL_CREST_CELL		POSTN	PLP1	TFAP2A	MPZ	PAX3	SOX10																		
CELLMARKERCUR_N_CRYPT_CELL		TNFRSF…	LGR5	OLFM4																					
CELLMARKERCUR_N_CRYPT_STEM_CELL		FOXQ1	LGR5	ASCL2	PROM1	SOX9																			
CELLMARKERCUR_N_CYCLING_B_CELL		HMGB2	STMN1	MKI67																					
CELLMARKERCUR_N_CYCLING_EPITHELIAL_CELL		TUBA1B	EEF2	MKI67																					
CELLMARKERCUR_N_CYTOTOXIC_CD8_T_CELL		GZMB	CD8A	CCL5	CD2	KNSTRN																			
CELLMARKERCUR_N_CYTOTOXIC_DOUBLE_POSITIVE…		GZMH	KLRG1	GZMB	GNLY	FGFBP2	TNF																		
CELLMARKERCUR_N_CYTOTOXIC_T_CELL		CD8A	GZMA	PRF1	KLRG1	GZMB	CX3CR1	FGFBP2	NKG7	CD3D	CD8B	GNLY	GZMH	IL2RA	CD3E	CD2	VIM	LAPTM5	PTPRC	CD3G	CD28	IDO1	IFNG	CCL4	CCL3

CELLMARKERCAN_ACINAR_CELLS		CTRB1	KLK1	RBPJL	PTF1A	CELA3A	PRSS1	SPINK1	ZG16	CEL	CELA2A	CPB1	CELA1	RNASE1	AMY2B	CPA2	CPA1	CELA3B	PNLIP	CTRB2	PLA2G1B	PRSS2	CLPS	REG1A	SYCN
CELLMARKERCUR_N_CYTOTOXIC_T_CELL_2		GZMH	PRF1	GNLY																					
CELLMARKERCUR_N_DAMAGE_ASSOCIATED_TRANSIE…		CDKN1A	KRT8	CLDN4																					
CELLMARKERCUR_N_DARK_ZONE_CELL		FOXP1	CXCR4	AICDA																					
CELLMARKERCUR_N_DEDIFFERENTIATION_ADIPOCY…		VCAM1	ITGB1	PTPRC	CD34	CD44																			
CELLMARKERCUR_N_DEEP_LAYER_NEURON		CRYM	SOX5	BCL11B	TBR1	FOXP2	FEZF2																		
CELLMARKERCUR_N_DEFINITIVE_ENDODERM_CELL		HDHD5-…	LEFTY1	SOX17	CXCR4	EOMES	GATA6	CER1	MIXL1	FOXA2															
CELLMARKERCUR_N_DELTA_CELL		RGS2	SLC38A1	FFAR4	LEPR	SEC11C	CD9	PPY	SST																
CELLMARKERCUR_N_DEMYELINATING_MICROGLIAL_…		TMEM119	CD68	PLP1																					
CELLMARKERCUR_N_DENDRITIC_CELL_2A		CLEC4A	CLEC10A	FCER1A	AREG	CD1C	LTB	CD3E																	
CELLMARKERCUR_N_DENDRITIC_CELL_LINEAGE		SIGLEC6	IL3RA	AXL	CD33	CD2	SIRPA																		
CELLMARKERCUR_N_DENTAL_PULP_CELL		ALCAM	THY1	NT5E																					
CELLMARKERCUR_N_DERMAL_CELL		DPP4	THY1	PTPRC	CD34	SFRP2	TCF4	WIF1	APCDD1																
CELLMARKERCUR_N_DEUTEROSOMAL_CELL		FOXN4	DEUP1	CDC20B	FOXJ1	PLK4																			
CELLMARKERCUR_N_DIFFUSE_GASTRIC_CANCER_CE…		COL1A1	S100A4	COL1A2																					
CELLMARKERCUR_N_DISTAL_CELL		MND1	HMGB3	TACC3																					
CELLMARKERCUR_N_DISTAL_STEM_CELL		PTGDR	LEFTY1	SMOC2																					
CELLMARKERCUR_N_DISTAL_TUBULAR_CELL		POU3F3	SOX9	CALB1	UMOD	DEFB1	CDH1	SLC8A1	SLC12A3	CKB															
CELLMARKERCUR_N_DIVIDING_CELL		MKI67	CDK1	H2AZ1	TUBB																				
CELLMARKERCUR_N_DIVIDING_PLASMA_B_CELL		MKI67	MZB1	CD38																					
CELLMARKERCUR_N_DIVIDING_T_CELL		HMGB2	CD3D	CORO1A	GAPDH	ACTG1	IL7R	TUBA1B																	
CELLMARKERCUR_N_DOPAMINERGIC_NEURON		FOLR1	KCNJ6	TH	NR4A2	FOXA2	LMX1B																		
CELLMARKERCUR_N_DORSAL_ANTERIOR_HINDBRAIN…		PAX3	MSX1	PAX7																					
CELLMARKERCUR_N_DORSAL_LATERAL_GANGLIONIC…		NRG1	ETV1	PAX6																					
CELLMARKERCUR_N_DORSAL_MEDIAL_GANGLIONIC_…		ZFHX3	NR2F1	ZNF503																					
CELLMARKERCUR_N_DOUBLE_NEGATIVE_B_CELL		CD19	CD27	MS4A1	FOXP4																				
CELLMARKERCUR_N_DOUBLE_NEGATIVE_T_CELL		PTPRC	CD3E	TRAC																					
CELLMARKERCUR_N_DOUBLE_POSITIVE_T_CELL		CD8A	PTPRC	CD4																					
CELLMARKERCUR_N_DOUBLE_POSITIVE_TREG_CELL		ID3	BATF	FOXP3																					
CELLMARKERCUR_N_EARLY_HEMANGIOBLAST		SIX1	FOXC2	ETV2																					
CELLMARKERCUR_N_EARLY_PROXIMAL_TUBULE_CELL		SMIM24	CUBN	GLYAT	APOE																				
CELLMARKERCUR_N_EARLY_RESPONSE_SECRETORY_…		EGR1	NR4A1	FOS	JUN																				
CELLMARKERCUR_N_EFFECTOR_CHONDROCYTE		KIF9	CHRDL2	CYSTM1	TF	TPM4	RARRES2	DSC2																	
CELLMARKERCUR_N_EFFECTOR_MEMORY_T_CELL		CD4	CD27	CD8A	SELL	CCR7	GZMK	KLRB1	IL7R	TNFSF1…	TIMP1	LTB	TNFRSF4	CD52	AQP3	CXCR5	B3GAT1	TCF7	KLRG1	PTGER2	TNFRSF1A	PDCD1	CTLA4	RIPK3	RIPK1
CELLMARKERCUR_N_EFFECTOR_NATURAL_KILLER_C…		CD2	NKG7	CD8B																					
CELLMARKERCUR_N_ELONGATING_CELL		TNP1	PRM2	PRM1	SPATA3	TSSK6																			
CELLMARKERCUR_N_EMBRYONIC_CELL		FOXA2	ISL1	PDX1																					
CELLMARKERCUR_N_EMBRYONIC_STEM_CELL		CDH3	CD9	LIN28A	SOX2	FUT4	LEFTY2	TERT	REXO1	POU5F1	B3GAT1	ATF3	MT1G	H4C3	CDH1	ANXA1	PEG10	KHDC3L	TPM1	FABP5	KRT19	ITGA6	DPPA5	MT1X	GATA6
CELLMARKERCUR_N_ENDOCARDIAL_CELL		NFATC1	GATA5	KLF2	MSX1	CDH5	NOTCH4	TBX3	ENG	TBX20															
CELLMARKERCUR_N_ENDOCRINE_PROGENITOR_CELL		CXCR4	DNER	DDR1																					
CELLMARKERCUR_N_ENDODERM_PROGENITOR_CELL		PECAM1	CD34	PROM1																					
CELLMARKERCUR_N_ENDODERMAL_CELL		PAF1	SOX17	FOXA2																					
CELLMARKERCUR_N_ENDOMETRIAL_STROMAL_CELL		RGS5	PLN	SUSD2																					
CELLMARKERCUR_N_ENDOMETRIAL_STROMAL_STEM_…		PTPRC	ITGB1	CD34	THY1																				
CELLMARKERCUR_N_ENDOTHELIAL_PRECURSOR_CELL		PECAM1	KDR	PROM1	FLT1	TEK																			
CELLMARKERCUR_N_ENSHEATHING_GLIAL_CELL		MPZ	PLP1	S100B	PMP2	ALX3																			
CELLMARKERCUR_N_ENTERIC_NEURON		PENK	ELAVL4	NFASC																					
CELLMARKERCUR_N_EOSINOPHIL		SIGLEC8	CEACAM8	CCR3	FCGR3A	FUT4	CD9	IL5RA	CSF3R	IL4R	IL3RA	IL6R	CSF2RA	CD44	CEACAM5	IL2RA	CD14	CD33	CD69	CD48	CD81	ICAM1	IL13	IL5	IL4
CELLMARKERCUR_N_EPENDYMAL_CELL		FOXJ1	CFAP299	AQP4																					
CELLMARKERCUR_N_EPIBLAST_CELL		SOX2	HESX1	NODAL	POU5F1	PRDM14	KLF17	FGF4																	
CELLMARKERCUR_N_EPICARDIAL_CELL		TCF21	GATA6	GATA4	PDGFRB	TBX5	COL1A1	WT1	TBX18																
CELLMARKERCUR_N_EPICARDIUM_DERIVED_CELL		ACTA2	VIM	DDR2																					
CELLMARKERCUR_N_EPIDERMAL_CELL		ITGA6	WNT6	LEF1	KRT14	KRT10	KRT1																		
CELLMARKERCUR_N_EPIDERMAL_STEM_CELL		CD34	CD200	YAP1	CSPG4	ITGA6	ITGB1	KRT15																	
CELLMARKERCUR_N_EPITHELIAL_CELL		VWF	KDR	EPCAM	KRT8	KRT18	CLDN3	KRT78	SCGB1A1	MUC5B	ETFA	KRT5	CDH3	EGFR	KRT19	ITGA6	PPARG	GATA3	KRT17	CDH12	KRT13	CDH18	SOX2	PAX9	KRT7
CELLMARKERCUR_N_EPITHELIAL_DUCTAL_CELL		KRT19	AQP5	CDH1	SOX9																				
CELLMARKERCUR_N_EPITHELIAL_MESENCHYMAL_CE…		VIM	CDH2	TWIST1	ZEB2	ZEB1	FOXC2	CDH11	CDH1	EPCAM															
CELLMARKERCUR_N_EPITHELIOID_MACROPHAGE		CD163	CD274	CD33																					
CELLMARKERCUR_N_EPSILON_CELL		APOH	ASCL1	SPINK1	FRZB																				
CELLMARKERCUR_N_ERYTHROBLAST		TFRC	AHSP	HBB	HBA2	CA1	ALAS2	HBE1																	
CELLMARKERCUR_N_ERYTHROID_CELL		GYPA	TFRC	HBB	HBA2	HBA1	ALAS2	HBG1	GATA1																
CELLMARKERCUR_N_ERYTHROID_PROGENITOR_CELL		CA1	HBD	KIT	GATA1																				
CELLMARKERCUR_N_ESOPHAGEAL_CELL		KRT4	KRT13	KRT14																					
CELLMARKERCUR_N_ESOPHAGEAL_PROGENITOR_CELL		RPE65	COL17A1	KRT15																					
CELLMARKERCUR_N_EXCITATORY_NEURON		NEUROD2	NRGN	SLC17A7	NEUROD1	PROX1	MEIS2	SEMA5A	PID1	MGAT4C	SULF2	NEUROD6	NRIP3	DPF3	MPPED1	SCGN	RANBP…	KCNQ3	CNKSR2	PHF21A	ELP4	EOMES	BCL11B	STMN2	SATB2
CELLMARKERCUR_N_EXHAUSTED_DOUBLE_POSITIVE…		CXCL13	TNFRSF18	TNFRSF4	CTLA4	DUSP4																			
CELLMARKERCUR_N_EXTRAVILLOUS_TROPHOBLAST		ITGA5	CD9	MMP9	HLA-G	ITGA1	MMP2																		
CELLMARKERCUR_N_F_PERICYTE		ITGA1	NR2F2	CXCL12	STEAP4	COL4A1																			
CELLMARKERCUR_N_FETAL_MYOBLAST		NFIX	PAX3	CD82																					
CELLMARKERCUR_N_FIBROBLAST_LIKE_CELL		COL1A1	COL1A2	POSTN	A2M	PTGDS																			
CELLMARKERCUR_N_FIBROCHONDROCYTE		COL6A1	COL1A1	COL3A1																					
CELLMARKERCUR_N_FIBROCHONDROCYTE_PROGENIT…		COL3A1	COL1A1	MYLK	MCAM																				
CELLMARKERCUR_N_FOAM_CELL		MMP9	ABCG1	ABCA1	OLR1																				
CELLMARKERCUR_N_FOLLICULAR_B_CELL		MS4A1	CD79A	HK2	CD74	PLAC8	BANK1	REL	GPR183	NR4A2	FCMR	SELL	FCER2	HLA-DRB5	CD83	SETMAR	LINC0…	CD69	CR2	CD19	CD22	TCL1A	IL4R	IGHD	YBX3
CELLMARKERCUR_N_FOLLICULAR_CELL		CD19	HLA-DRA	CD74	BANK1	VPREB3	PAX8	NKX2-1																	
CELLMARKERCUR_N_FOLLICULAR_DENDRITIC_CELL		CR2	FCER2	CR1																					
CELLMARKERCUR_N_FOREBRAIN_CELL		EMX1	FOXG1	OTX2	SOX1																				
CELLMARKERCUR_N_GABAERGIC_NEURON		GAD1	SLC32A1	GAD2																					
CELLMARKERCUR_N_GANGLION_CELL		SNCG	NEFM	ALCAM	CNTN2	NRN1	EBF3	EBF1	GAP43	POU4F2															
CELLMARKERCUR_N_GASTRIC_NEUROENDOCRINE_CE…		GAST	CHGA	EPCAM	SST	TFF3																			
CELLMARKERCUR_N_GASTRIC_STEM_CELL		LGR5	TNFRSF19	BHLHA15	RUNX1	SOX2																			
CELLMARKERCUR_N_GERM_CELL		ZAR1	BMP15	GDF9	DDX4	NLRP5	POU5F1	DAZL	DPPA3	PRDM1	TFAP2C	SOX17	SYCP3	ZP4	CTDSPL	SALL4	AFP	FGFR3	UTF1	YBX2	YBX1	TEX101	ACTL7B	TNP1	H1-9P
CELLMARKERCUR_N_GERMINAL_CENTER_B_CELL		MS4A1	NEIL1	H2AZ2	PCLAF	HMGB2	PTTG1	SLAMF7	STMN1	DERL3	CCDC144A	JSRP1	IRAG2	HMGA1	H4C3	RGS13	AICDA	LMO2	GCSAM	SNX29P2	SYNE2	MARCKSL1	DAAM1	UCP2	HMCES
CELLMARKERCUR_N_GERMINAL_CENTER_CELL		BANK1	HLA-DRA	VPREB3	CD19	CD74	MEF2B	BCL6																	
CELLMARKERCUR_N_GERMINAL_CENTRE_B_CELL		CD19	TFRC	MME	MS4A1																				
CELLMARKERCUR_N_GLIAL_CELL		GFAP	S100A1	SLC1A3	ITGAM	MBP	CLDN5	SLC1A2	SOX2	MOG	RLBP1	BMP4	PLVAP	CHI3L1	XCR1	ACTA2	WNT2B	CD68	RSPO3	S100B	MADCAM1	CD36	WNT5B	SOX17	VWA1
CELLMARKERCUR_N_GLIOMA_STEM_CELL		PROM1	CD44	FUT4	EGFR	SOX2																			
CELLMARKERCUR_N_GLOBOSE_BASAL_CELL		NEUROD1	CXCR4	EZH2	ASCL1	SOX2	HES6	NEUROG1																	
CELLMARKERCUR_N_GLOMERULAR_PARIETAL_EPITH…		KRT8	CD24	VCAM1	KRT18																				
CELLMARKERCUR_N_GLUTAMATERGIC_NEURON		TBR1	BDNF	SLC17A6	SLC17A7	BCL11B	SATB2	OTX1	GRIN1	GRIN2B															
CELLMARKERCUR_N_GOBLET_CELL		GABRP	MUC5AC	MUC5B	KRT78	EPCAM	SCGB1A1	TSPAN13	AGR2	TFF3	SPDEF	CLDN10	TSPAN8	MSMB	WFDC2	SCGB3A1	SLPI	BPIFB1	GP2	TFF2	TFF1	MUC2	ZG16	FFAR4	SPINK4
CELLMARKERCUR_N_GONADOTROPE		FSHB	GNRHR	LHB																					
CELLMARKERCUR_N_GONADOTROPH_CELL		NR5A1	GATA2	LHB	ESR1																				
CELLMARKERCUR_N_GRANULOCYTE		CEACAM8	CD14	PTPRC	FCGR3A	ENPP3	FUT4	CD63	CCR3	CD44	CD33	IL3RA	FCGR3B	MPO	AZU1	PRTN3	GATA2	NKX2-1	CLC	S100A12	ITGAM	AMH	GSTA1	SERPINE2	HSD17B1
CELLMARKERCUR_N_GRANULOCYTE_MONOCYTE_PROG…		CD34	CD38	IL3RA	FLT3	MME																			
CELLMARKERCUR_N_GRANULOCYTIC_UNK		MPO	RETN	LYZ																					
CELLMARKERCUR_N_GRANULOSA_CELL		FOXL2	CHST8	MGARP	GLDC	FSHR	GPX3	INHBA	AMH	MCAM															
CELLMARKERCUR_N_HAEMATOPOIETIC_CELL		CD9	PTPRC	CD14																					
CELLMARKERCUR_N_HAIR_FOLLICLE_CELL		KRT15	RUNX1	CD34	SOX9																				
CELLMARKERCUR_N_HEMANGIOBLAST		CD34	EPHB4	CDH1	CDH5	ACE	KDR	PECAM1	PROM1																
CELLMARKERCUR_N_HEMATOPOIETIC_CELL		LRP1	PTPRC	PROM1	CD34	KIT	SPINK2	GATA2	HBE1	HBB	NKG7	FCER1G	CD53	ITGA2B	SPN	UCP2	TYROBP	CD44	ATP5MC3	HCST	SRGN	RUNX1	SPI1	CDH5	GJA4
CELLMARKERCUR_N_HEMATOPOIETIC_PRECURSOR_C…		KIT	PTPRC	CD34	CD14	THY1	IL3RA																		
CELLMARKERCUR_N_HEMATOPOIETIC_PROGENITOR_…		CD34	ITGA2B	GYPA	CD38	AVP	MSRB3	MEIS1	CRHBP	FAM124B	CRYGD	HOXA3	KCNJ13	HOXB6	LAPTM4B	EXD2	ATP8B4	CLEC3B	ELN	HTR1F	ANGPT1	MYCT1	FLT3	HLF	KLF1
CELLMARKERCUR_N_HEPATIC_CELL		HP	APOE	TF	ASS1	APOB	CYP2E1	PCK1	ALB	ASGR1															
CELLMARKERCUR_N_HEPATIC_PROGENITOR_CELL		EPCAM	CD24	SOX9	JAG1	KRT19	TACSTD2																		

Table S5: Cell state marker gene list

CELLMARKERCAN_ACINAR_CELLS		CTRB1	KLK1	RBPJL	PTF1A	CELA3A	PRSS1	SPINK1	ZG16	CEL	CELA2A	CPB1	CELA1	RNASE1	AMY2B	CPA2	CPA1	CELA3B	PNLIP	CTRB2	PLA2G1B	PRSS2	CLPS	REG1A	SYCN
CELLMARKERCUR_N_HEPATIC_STELLATE_CELL		HGF	DCN	FRZB	GEM	PDGFRB	PDGFRA	LHX2	FOXF1	HLX															
CELLMARKERCUR_N_HEPATOBLAST		TFRC	PTPRC	DLK1	DAPK3	GJB1	HNF4A	AFP	FXYD1																
CELLMARKERCUR_N_HEPATOCYTE		APOC3	KRT8	MME	ALB	AFP	MET	ABCC2	TJP1	CYP3A4	ARG1	CPS1	TAT	CEBPA	TBX3	GPC3	APOE	APOA2	ASGR1	TF	KRT18	ICAM1	TTR	SLC17A5	HNF4A
CELLMARKERCUR_N_HISTIOCYTE		CD163	CD68	CD14	CD4																				
CELLMARKERCUR_N_HMSC_TREATED_B_CELL		CD40	CD27	CD5	FCER2	CD24	CD38	SPN	CR2																
CELLMARKERCUR_N_HOFBAUER_CELL		LYVE1	CD68	CD14	CD163	FCGR1A																			
CELLMARKERCUR_N_HOMEOSTATIC_CHONDROCYTE		IER2	IER5	NXF1	EIF4A3	NR4A1	RASD1	HEXIM1	CHMP1B	TSC22D2	CCNL1	DDIT3	ZFP36	KLF2	CDKN2AIP	CKS2	PCF11	NUFIP2	KLF10	SLC40A1	KMT2E	TIPARP	ING1	ARL6IP1	PLK2
CELLMARKERCUR_N_HOMEOSTATIC_MICROGLIAL_CE…		ADGRG1	GPR34	GPR183	CX3CR1	P2RY13	P2RY12																		
CELLMARKERCUR_N_HORIZONTAL_BASAL_CELL		TP63	CXCL14	MEG3	KRT5	SOX2																			
CELLMARKERCUR_N_HORIZONTAL_CELL		ONECUT1	ONECUT2	LHX1																					
CELLMARKERCUR_N_HYPOBLAST_CELL		COL4A1	SOX17	FOXA2	GATA6	GATA4																			
CELLMARKERCUR_N_IDIOPATHIC_PULMONARY_FIBR…		RUNX1	FSTL1	CALD1	FBN1	PPIC	POSTN	SPARC	CTHRC1																
CELLMARKERCUR_N_IGA_MEMORY_B_CELL		LOC102…	MS4A1	CD27																					
CELLMARKERCUR_N_IGG_MEMORY_B_CELL		CD27	MS4A1	CD19																					
CELLMARKERCUR_N_IGM_MEMORY_B_CELL		MS4A1	CD27	CD38	PTPRC																				
CELLMARKERCUR_N_IGM_ONLY_B_CELL		CD19	CD27	MS4A1																					
CELLMARKERCUR_N_IL7R_T_HELPER_CELL		GZMH	GZMA	CD3D	IFNG	GZMK	CD69	CD40LG																	
CELLMARKERCUR_N_IMMATURE_ERYTHROID_CELL		HBM	CA2	CA1	HBD																				
CELLMARKERCUR_N_IMMATURE_MYELOID_CELL		IL3RA	FCGR3A	CEACAM8	CD14	CD33	FUT4																		
CELLMARKERCUR_N_IMMATURE_NEUTROPHIL		FCGR3A	FUT4	CD101	CEACAM8	TOP2A	CST3	CTSS																	
CELLMARKERCUR_N_IMMATURE_TRANSITIONAL_B_C…		CD19	CD38	CD24																					
CELLMARKERCUR_N_IMMUNE_CELL		PTPRC	CD86	ANPEP	CD14	CD209	CD83	PECAM1	FCGR3A	LYZ	CD3D	NKG7	MS4A1	CPA3	TPSAB1	CD19	CEACA…	CD8A	NCAM1	CD68	CD4	ITGAX	TPSB2	FOXP3	FCN1
CELLMARKERCUR_N_INDUCED_PLURIPOTENT_STEM_…		ALPL	KLF5	SMAD2	SMAD3	SOX2	STAT3	TBX2	TBX5	TBX3	ZIC3	UTF1	KLF4	ABCG2	LEFTY1	SUZ12	L1TD1	THAP11	LIN28A	CBX2	ZFP42	DPPA3	LIN28B	TEX19	ACVR1B
CELLMARKERCUR_N_INFILTRATING_MACROPHAGE		PTPRC	CCR2	CD68	CD14																				
CELLMARKERCUR_N_INFLAMMATORY_CANCER_ASSOC…		CFD	CXCL12	IL6	ACTA2																				
CELLMARKERCUR_N_INFLAMMATORY_DENDRITIC_CE…		CD209	CD163	FCGR1A	CD14																				
CELLMARKERCUR_N_INFLAMMATORY_MONOCYTE		FCGR3A	CD14	CCR2																					
CELLMARKERCUR_N_INHIBITORY_NEURON		GAD1	PAX6	CDH12	VIP	CCK	LHX6	CALB2	SST	GAD2	DLX1	NR2F2	LHX5	DLX5	DLX2	PNOC	TNFAI…	CRHBP	CALB1	NDNF	PVALB	NOS1	NPY	RELN	TAC3
CELLMARKERCUR_N_INNATE_LYMPHOID_CELL		PTGDR2	KLRB1	IL7R	CD200R1	GATA3	CXCR4	CXCR3	CXCR5	CCR6	GZMB	CSF2	CXCR6	KLRD1	NCR1	ITGAE	NCAM1	ITGA1	NCR2	CXCL2	ID2	KIT	TNFSF1…	AREG	DDIT4
CELLMARKERCUR_N_INNER_CELL_MASS_CELL		GDF3	CUBN	IGF1																					
CELLMARKERCUR_N_INNER_RADIAL_GLIAL_CELL		TOP2A	NUSAP1	CENPU	PCLAF																				
CELLMARKERCUR_N_INTER_MEDIATE_PROGENITOR_…		PPP1R17	NHLH2	NHLH1	TP53I11																				
CELLMARKERCUR_N_INTERCALATED_CELL		SLC4A1	SLC26A7	ATP6V1…	FOXI1																				
CELLMARKERCUR_N_INTERFERON_RESPONSIVE_SEC…		MX1	IFITM3	IFI6																					
CELLMARKERCUR_N_INTERMEDIATE_MONOCYTE		FCGR3A	CD14	IL3RA	CD33	FUT4	CEACAM8	S100A8																	
CELLMARKERCUR_N_INTERMEDIATE_MONOCYTE_CELL		CD14	APOBEC3A	GBP4	MARCO	MARCKSL1	FCGR3A																		
CELLMARKERCUR_N_INTERSTITIAL_MACROPHAGE		CXCL11	CXCL10	CCL4	ISG20	ISG15																			
CELLMARKERCUR_N_INTERSTITIAL_PROGENITOR_C…		FOXD1	GDNF	TYMS	CENPK	PCLAF	RRM2																		
CELLMARKERCUR_N_INTESTINAL_EPITHELIAL_STE…		PHLDA1	CD44	CD24																					
CELLMARKERCUR_N_INTESTINAL_GASTRIC_CANCER…		REG4	MUC13	CDH17	CEACAM5	KRT8																			
CELLMARKERCUR_N_INTESTINAL_METAPLASIA_CELL		CDX2	CDX1	ENO1	PGC	TFF3																			
CELLMARKERCUR_N_INTRAEPITHELIAL_LYMPHOCYT…		ITGAE	KIR3DL2	KIR3DL1																					
CELLMARKERCUR_N_IONOCYTE		CFTR	ASCL3	FOXI1																					
CELLMARKERCUR_N_IRIS_PUTTATIVE_STEM_CELL		ZIC2	LHX2	RAX																					
CELLMARKERCUR_N_KUPFFER_CELL		CD68	ITGB2	CD14	FCGR2A	CD163	CLEC4F	VSIG4	C1QA																
CELLMARKERCUR_N_LACTOTROPH_CELL		POU1F1	ESR1	PRL	DRD2																				
CELLMARKERCUR_N_LANGERHANS_CELL		CD1A	CD207	TACSTD2	FCER1A	S100B																			
CELLMARKERCUR_N_LEUKOCYTE		PTPRC	FCGR3A	CD38	MS4A1	HAVCR2	IL2RA	PDCD1	CD40LG	ITGAM	ITGAL	LAG3	CD274	CD69	NCAM1	ITGAX	FAS	PRF1	CTLA4	CCR4	CD14	CD19	MKI67	CD4	TBX21
CELLMARKERCUR_N_LIMBAL_EPITHELIAL_STEM_CE…		ALCAM	MCAM	CXCR4	KIT	CDH5																			
CELLMARKERCUR_N_LIMBAL_STEM_CELL		KRT5	KRT19	ABCG2	KRT15	VIM	ITGA9	TP63	KRT14	SOD2															
CELLMARKERCUR_N_LIPOFIBROBLAST		COL1A1	PDGFRA	PLIN2	APOE																				
CELLMARKERCUR_N_LIVER_PROGENITOR_CELL		KRT19	CD24	NOTCH3																					
CELLMARKERCUR_N_LIVER_STEM_CELL		ITGB1	THY1	H19	GSTP1	EGF	HGF	KRT19	NOTCH3																
CELLMARKERCUR_N_LMMLEYDIG_CELL		CFD	DLK1	SFRP1	IGF1	NR2F2	DCN	IGF2																	
CELLMARKERCUR_N_LONOCYTE		CFTR	ASCL3	RARRES2	FOXI1																				
CELLMARKERCUR_N_LOOP_OF_HENLE_CELL		UMOD	CLDN16	SLC12A1																					
CELLMARKERCUR_N_LOOP_PROGENITOR_CELL		TOP2A	HMGB2	UBE2C																					
CELLMARKERCUR_N_LOW_DENSITY_NEUTROPHIL		CD226	ITGB3	CD36	ITGA2B																				
CELLMARKERCUR_N_LUMINAL_PROGENITOR_CELL		ITGA6	CD44	CD24	KRT5	KIT	ALDH1A3	GABRP	TNFRSF1…	EZH2															
CELLMARKERCUR_N_LUNG_CELL		SOX2	SFTPB	SFTPA1	SFTPA2																				
CELLMARKERCUR_N_LYMPH_NODE_CELL		FAS	ENTPD1	RB1	CD38	NT5E	CD27																		
CELLMARKERCUR_N_LYMPHATIC_CELL		LYVE1	PECAM1	CCL21																					
CELLMARKERCUR_N_LYMPHATIC_ENDOTHELIAL_CELL		PDPN	LYVE1	PECAM1	MMRN1	FLT4	PROX1	CCL21	CD34																
CELLMARKERCUR_N_LYMPHOBLASTOID_CELL		CD52	ENTPD1	FCER2	CD70	CD58																			
CELLMARKERCUR_N_LYMPHOCYTE		GATA3	FOXP3	NCAM1	CD8A	CD4	MS4A1	PTPRC	CX3CR1	ITGAE	CD79A	NKG7	CD3D	LTB	MME	TNFRSF8	SET	CD2	CD14	CD19	CD22	CD5	SASH3	LCK	GZMA
CELLMARKERCUR_N_LYMPHOID_CELL		PTPRC	CD247	NCAM1	TIA1	CD19	CD4	CD8A	CXCL9	CXCL10	GMFG	ITGA2B	IL7R	CD7	CD27	CD34	CD38	CD68	CD69	CD81	FLT3	CXCR3	ZAP70	KLRG1	CD160
CELLMARKERCUR_N_LYMPHOID_PROGENITOR_CELL		SELL	CD7	IL7R																					
CELLMARKERCUR_N_LYMPHOID_STRUCTURE_CELL		CD4	CD8A	CR2	MS4A1	FOXP3																			
CELLMARKERCUR_N_M_PERICYTE		MCAM	TAGLN	CRIP1	MYL9	ADIRF																			
CELLMARKERCUR_N_M1_MACROPHAGE		CD68	TREM2	CD40	CD80	CCL5	CCR7	TNF	CD86	TLR2	TLR4	FCGR3A	CXCL10	FCGR1A	FCGR2A	SELP	IL7R	CCL4	TSPO	NOX1	CD163	FPR3	SIGLEC1	CCL2	PTGS2
CELLMARKERCUR_N_M1_MICROGLIAL_CELL		FCGR3A	CSF1R	TYROBP	TREM2	CX3CR1	P2RY13	P2RY12																	
CELLMARKERCUR_N_M2_MACROPHAGE		CD163	FOLR2	MRC1	IL10	MMP14	VTCN1	EGF	TGFB1	MSR1	ARG1	CLEC4A	FCER2	IL1B	TNFSF10	RETNLB	CXCR4	CD209	CD36	CD14	CHI3L1	MS4A4A	ABL2	CCL2	CD68
CELLMARKERCUR_N_MACROGLIAL_CELL		COL4A3	FOS	FTL																					
CELLMARKERCUR_N_MALIGNANT_CELL		FXYD2	CD3D	CD14	KRT19	EPCAM	KRT5	TP63																	
CELLMARKERCUR_N_MALIGNANT_PLASMA_CELL		FRZB	IFI27	IL6R	DKK1	EDNRB																			
CELLMARKERCUR_N_MARGINAL_ASSOCIATED_MACRO…		APOE	TREM2	CX3CR1	LYVE1																				
CELLMARKERCUR_N_MAST_CELL		CPA3	KIT	TPSAB1	CD69	CCL4	ENPP3	CD2	IL2RA	CD33	TPSD1	CMA1	CTSG	VWA5A	MS4A2	HDC	CLU	GATA2	TPSB2	CD9	FCER1A	SLC18A2	HPGDS	FCER1G	CD74
CELLMARKERCUR_N_MATURE_ASTROCYTE		AQP4	GFAP	AGT	SPARCL1	TOP2A	TNC																		
CELLMARKERCUR_N_MATURE_B_CELL		LYN	CD37	CD74	HLA-DRA	CD79A	IGHM	CD19	DHX9	IL2RA															
CELLMARKERCUR_N_MATURE_DENDRITIC_CELL		CD14	CD1C	CD83	NFKB1	LAMP3	CCL21	CCL19	CCR7	CD80															
CELLMARKERCUR_N_MATURE_ENDOTHELIAL_CELL		PECAM1	CD34	FLT1	CDH5																				
CELLMARKERCUR_N_MATURE_LUMINAL_CELL		ANKRD3…	ESR1	AGR2	FOXA1	PGR																			
CELLMARKERCUR_N_MATURE_NEURON		MAP2	EBF2	GNG13	CBX8	RTP1																			
CELLMARKERCUR_N_MATURE_NEURONAL		MAP2	TUBB3	MAPT																					
CELLMARKERCUR_N_MATURE_OLIGODENDROCYTE		MAG	CNP	PLP1	MBP	MOG																			
CELLMARKERCUR_N_MATURE_OSTEOBLAST		SPP1	BGLAP	SPARC	CALR	MMP9	NFATC1																		
CELLMARKERCUR_N_MATURE_PODOCYTE		PTPRO	CLIC5	NPHS2	NPHS1	OLFM3																			
CELLMARKERCUR_N_MEDIAL_AND_DISTAL_SEGMENT…		MITF	CD24	IRX1	TSPAN15																				
CELLMARKERCUR_N_MEDIAL_GANGLIONIC_EMINENC…		LHX8	NR2F1	NKX2-1	LHX6																				
CELLMARKERCUR_N_MEGAKARYOCYTE		GP9	ITGA2B	ITGB3	CD34	PF4	PPBP	SPARC	TUBB1	NRGN	GNG11	CAVIN2	MYL9	CCL5	FLI1	CD79A	PTPRJ	CD48	SELP	GP1BA	CD9	CTSW	PLEK	RASGRP2	RAB27B
CELLMARKERCUR_N_MEGAKARYOCYTE_ERYTHROID_C…		CD2	CD4	CD5	CD7	CD9	CD27	CD80	CD86	CD34	CD38	CD48	CD68	CD69	CD81	FLT3	CCR10	CXCR3	CD200	ZAP70	CD84	CD83	KLRG1	CD160	CD244
CELLMARKERCUR_N_MEGAKARYOCYTE_ERYTHROID_P…		CD34	CD38	IL3RA	CD7	FLT3	MME																		
CELLMARKERCUR_N_MEGAKARYOCYTE_PROGENITOR_…		TFRC	PF4	PLA2G1…	PPBP	PLEK																			
CELLMARKERCUR_N_MELANOMA_CELL		POT1	RAP1A	TPP1																					
CELLMARKERCUR_N_MEMORY_B_CELL		FCRL5	CD86	CD79A	CD27	SPIB	CD19	CR2	MS4A1	NEDD4L	KIF16B	AQP5	CLECL1	TCF7	AIM2	TFEC	KLK1	KLF10	EBI3	CD70	ZBTB32	CXCR3	IGHA1	CD38	IGHG1
CELLMARKERCUR_N_MEMORY_B_CELL_PRECURSOR		CCR6	BANK1	RASGRP2	CELF2	STAT1	IFITM2	IFNGR1	IFITM1																
CELLMARKERCUR_N_MEMORY_CD27_B_CELL		CD19	MME	CD27																					
CELLMARKERCUR_N_MEMORY_CD4_T_CELL		CD28	SELL	CCR7																					
CELLMARKERCUR_N_MEMORY_DOUBLE_POSITIVE_T_…		CD8B	HLA-DQA2	XCL2	CD69	CD8A																			
CELLMARKERCUR_N_MEMORY_REGULATORY_T_CELL		CCR4	CD4	IL2RA	FOXP3																				
CELLMARKERCUR_N_MEMORY_T_CELL		S100A4	CD3D	CCR7	CD44	SELL	CD4	IL32	S100A10	LGALS3	ALOX5AP	MYL12A	S100A6	LGALS1	CD8A	IL7R	EOMES	RUNX2	ABCB1	KLRB1	CD69	ITGAE	CD27	CXCR3	IL2RA
CELLMARKERCUR_N_MERKEL_CELL		NCAM1	ATOH1	ISL1	KRT20	KRT8	KRT18	SOX2																	
CELLMARKERCUR_N_MESENCHYMAL_CELL		MMP1	VIM	CDH2	THY1	NT5E	CSF1	CCL2	CXCL2	HIF1A	CHI3L1	MET	ZEB2	CCN2	RUNX1	LUM	COL1A1	ZEB1	FN1	ETS1	CD44	EGFR	PTK2	TIMP1	TGFB1
CELLMARKERCUR_N_MESENCHYMAL_EPITHELIAL_TR…		VIM	ITK	LHX3	LY6H	EPCAM																			

Table S5: Cell state marker gene list

CELLMARKERCAN_ACINAR_CELLS		CTRB1	KLK1	RBPJL	PTF1A	CELA3A	PRSS1	SPINK1	ZG16	CEL	CELA2A	CPB1	CELA1	RNASE1	AMY2B	CPA2	CPA1	CELA3B	PNLIP	CTRB2	PLA2G1B	PRSS2	CLPS	REG1A	SYCN
CELLMARKERCUR_N_MESENCHYMAL_GLIOBLASTOMA_…		COL1A2	S100A1	COL1A1	TGFB1	THBS1	DAB2	CD44	CHI3L1																
CELLMARKERCUR_N_MESENCHYMAL_STEM_CELL		CD19	CD34	CD44	ALCAM	ANPEP	ERBB2	ITGA5	ITGAV	ITGB1	KIT	MME	NCAM1	NGFR	NT5E	PECAM1	PTPRC	TFRC	THY1	TNFRSF…	VCAM1	CD70	CD14	UCP1	SOX2
CELLMARKERCUR_N_MESENCHYMAL_STROMAL_CELL		NGFR	SUSD2	CD34	CD44	NT5E	THY1	MCAM	ALCAM	CSPG4	CD19	CD14	PECAM1	PTPRC	BSG	CD276	CD59	CD81	SLC3A2	CD47	ITGB1	CD151	ANPEP	ISLR	PDGFRB
CELLMARKERCUR_N_MESENDODERMAL_CELL		MIXL1	SOX17	FOXA2	EOMES																				
CELLMARKERCUR_N_MESODERMAL_EMBRYONIC_STEM…		CD34	CDH2	PECAM1																					
CELLMARKERCUR_N_MESODERMAL_PROGENITOR_CELL		NES	MCAM	CSPG4																					
CELLMARKERCUR_N_METAPLASTIC_STEM_CELL		REG1A	OLFM4	CLDN3																					
CELLMARKERCUR_N_MEYLOID_CELL		LYZ	CD68	MARCO	AIF1																				
CELLMARKERCUR_N_MICROGLIAL_CELL		TMEM119	CX3CR1	P2RY12	AIF1	BTG2	TRA2B	RGS2	JUN	MALAT1	DUSP1	SON	RGS1	ATF3	FUS	MEF2C	MEF2A	FOS	CD68	PTPRC	CD74	ARHGDIB	ITGAM	ABI3	FCER1G
CELLMARKERCUR_N_MICROVILLAR_CELL		CFTR	ASCL3	FOXL1	HEPACAM2																				
CELLMARKERCUR_N_MIDBRAIN_DOPAMINERGIC_NEU…		FOXA2	LMX1A	TH																					
CELLMARKERCUR_N_MONO_MACROPHAGE		CSF1R	FCGR3A	C1QA																					
CELLMARKERCUR_N_MONOCYTE_DERIVED_CELL		CD68	SDS	STARD13	FPR3	PMP22																			
CELLMARKERCUR_N_MONOCYTE_DERIVED_MYELOID_…		CD209	CLEC12A	CLEC4E																					
CELLMARKERCUR_N_MONOCYTE_LINEAGE		NCAM1	CD14	CD19																					
CELLMARKERCUR_N_MONOCYTE_PRECURSOR		MS4A6A	CCL3	CST3	LYZ	S100A9																			
CELLMARKERCUR_N_MONOCYTE_PROGENITOR_CELL		CD33	MPEG1	CD14	IRF8																				
CELLMARKERCUR_N_MUELLER_GLIA_CELL		SLC1A3	DKK3	CRABP1																					
CELLMARKERCUR_N_MULTIPOTENT_MESENCHYMAL_S…		THY1	NT5E	PTPRC	CD34	CD14	CD19	NGFR																	
CELLMARKERCUR_N_MULTIPOTENT_RETINAL_PROGE…		LIN28B	IGFBP5	LIN28A	BMP7	PFN1	TMSB10	GJA1	ATP1A2	JAG1															
CELLMARKERCUR_N_MULTIPOTENT_STEM_CELL		CD34	PTPRC	THY1	CD44	NT5E																			
CELLMARKERCUR_N_MULTIPOTENT_STROMAL_CELL		THY1	ITGAV	NT5E	MCAM																				
CELLMARKERCUR_N_MUSCLE_CELL		ACTA2	MYH11	MCAM	MYL9	ACTC1	TTN	TNNC2																	
CELLMARKERCUR_N_MUSCLE_DERIVED_CELL		CD34	KIT	VCAM1	NCAM1																				
CELLMARKERCUR_N_MUSCLE_SATELLITE_CELL		PAX7	NCAM1	CD82	CDCP1	SOX8																			
CELLMARKERCUR_N_MYELOBLAST		CD33	ANPEP	CSF2RA	CSF3R	FUT4	LRP1																		
CELLMARKERCUR_N_MYELOID_DENDRITIC_CELL		CLEC10A	CD4	CD80	CD86	CD40	CD83	CD209	CD207	CCR7	LY75	NRP1	PDCD1LG2	CD1C	IL3RA	FCER1A	ID2	ZEB2	KLF4	IRF4	ITGAX	CLEC4A	SIRPA	LYZ	BATF3
CELLMARKERCUR_N_MYELOID_DENDRITIC_CELL_1		PTPRC	PLD4	CLEC9A	LAMP3	CD1C																			
CELLMARKERCUR_N_MYELOID_DERIVED_CELL		CD68	CSF3R	LYZ																					
CELLMARKERCUR_N_MYELOID_DERIVED_SUPPRESSO…		CD33	ENTPD1	CD14	CD46	ICAM1	SIGLEC7	INSR	TFRC	ITGA6	NCAM1	CD52	CD53	ITGA1	CEACAM6	ITGA5	CD81	CD24	CEACAM5	SELP	SELL	CD244	CD84	CD69	CD226
CELLMARKERCUR_N_MYELOID_PROGENITOR_CELL		CEBPA	CD34	MPO	KIT																				
CELLMARKERCUR_N_MYO_EPITHELIAL_CELL		KRT5	KRT17	KRT14																					
CELLMARKERCUR_N_MYOBLAST		MYL1	MYOD1	MEF2C	LEMD2	TGFB1	MET	SMTN	TCF12	MYF5	EYA1	MYF6	TEAD4	SIX2	GLI2	PAX7	MEOX1	EYA2	EMD	SIX1	MYOG	NRG1	MAPK12	MEF2A	PAX3
CELLMARKERCUR_N_MYOCYTE		MYO18B	MYOG	MYH3	ACTA1	MYOD1	MYH1	MYH6	MYH7	MYH2															
CELLMARKERCUR_N_MYOFIBROBLAST		ACTA2	CST1	TAGLN	CCN2	MYLK	MYL9	SMN1	COL3A1	VIM	SDC1	COL1A1	SERPINH1	PLN	SERPINE1	THY1	CAV1	DCN	COL14A1	LUM	CD44	PDGFRB	CENPF	WNT10A	IL18
CELLMARKERCUR_N_MYOGENIC_ENDOTHELIAL_CELL		NCAM1	CD34	PTPRC	CDH5																				
CELLMARKERCUR_N_MYOGENIC_PROGENITOR_CELL		MYOD1	PDGFRA	ISL1																					
CELLMARKERCUR_N_MYOID_CELL		DES	PTCH1	MYH11	ACTA2	MYL9	MYOD1	BEX1	MYOG																
CELLMARKERCUR_N_NAIVE_B_CELL		CD79A	CD27	CD38	CD19	CD24	MS4A1	SEMA4A	TCL1B	YBX3	TCL1A	IL21R	IL4R	PCDH9	SATB1	HOXB2	GCNT1	CXCR4	PAX5	BCL7A	PLPP5	IGHD	APLP2	IGHM	FCER2
CELLMARKERCUR_N_NAIVE_CD8_T_CELL		CCR7	TCF7	MAL	TSHZ2	SELL	RCAN3	SOCS3	MYC	LEF1	NOSIP														
CELLMARKERCUR_N_NAIVE_DOUBLE_POSITIVE_T_C…		ZFP36L2	FTH1	GPR183	CCR7																				
CELLMARKERCUR_N_NAIVE_PROGENITOR_CELL		ITGB2	CD44	CD33																					
CELLMARKERCUR_N_NAIVE_T_CELL		CD4	CCR7	SELL	CD8A	CD27	CCR5	IL7R																	
CELLMARKERCUR_N_NASCENT_NEPHRON_CELL		PAX8	SIX1	JAG1	LHX1	EPCAM																			
CELLMARKERCUR_N_NATURAL_KILLER_CD56_BRIGH…		MPPED1	DUSP4	TRAPPC9	MADD	PLA2G6	RRAD	FOXJ1	XCL1																
CELLMARKERCUR_N_NATURAL_KILLER_CD56_DIM_C…		KIR2DS5	S1PR5	IL21R	KIR3DL1	KIR3DL3	GTF3C1	PMEPA1	GZMB	KIR2DL3															
CELLMARKERCUR_N_NEGATIVE_MESENCHYMAL_STEM…		CD34	CD19	PTPRC																					
CELLMARKERCUR_N_NEURAL_CELL		NES	SOX1	PAX6	MAP2																				
CELLMARKERCUR_N_NEURAL_CREST_STEM_CELL		TFAP2A	BGN	SOX10	KANK4	NGFR	B3GAT1																		
CELLMARKERCUR_N_NEURAL_CREST_STEMLIKE_CELL		GFRA2	AQP1	NGFR	GDNF																				
CELLMARKERCUR_N_NEURAL_PRECURSOR_CELL		DCX	NES	PAX6	SOX2																				
CELLMARKERCUR_N_NEURAL_STEM_CELL		PAX6	SOX2	PROM1	ASCL1	BMI1	CD81	NOTCH1	SOX9	CXCR4	CD24	ITGA6	ITGB1	MCAM	ABCG2	SOX1	ADGRG1	DCX	SLITRK6	NGFR	HMGB2	HES5	MAPT	VIM	CD44
CELLMARKERCUR_N_NEUROECTODERM_CELL		PAX6	OTX1	HES5	PAX3																				
CELLMARKERCUR_N_NEURONAL_INTERMEDIATE_PRO…		PPP1R17	EOMES	NEUROG1																					
CELLMARKERCUR_N_NEURONAL_PROGENITOR_CELL		TOP2A	PAX6	GMNN	DLX6-AS1	ASCL1																			
CELLMARKERCUR_N_NEUTROPHIL_PROGENITOR_CELL		CAMP	MPO	AZU1	LTF	LCN2																			
CELLMARKERCUR_N_NON_NEURON		PAX6	S100B	REST	MKI67	SLC1A3	SOX9																		
CELLMARKERCUR_N_NON_PT_TUBULAR_CELL		UMOD	SLC24A1	SLC12A1	SLC4A1	UPK3A	AQP2	KRT17																	
CELLMARKERCUR_N_NON_RECIRCULATING_TISSUE_…		ITGAE	ITGA1	PDCD1																					
CELLMARKERCUR_N_NONMYELINATING_SCHWANN_CE…		SCN7A	NGFR	L1CAM	NCAM1																				
CELLMARKERCUR_N_NOTOCHORD_CELL		KRT8	SALL3	TBXT																					
CELLMARKERCUR_N_NUCLEATED_CELL		HLA-B	HLA-C	HLA-A																					
CELLMARKERCUR_N_NUCLEUS_PULPOSUS_PROGENIT…		PRRX1	PDGFRA	IGF1																					
CELLMARKERCUR_N_OLIGODENDROCYTE		OLIG1	RTN4	OLIG2	PLP1	CNP	MOG	IGKV1D-37	MAG	CSPG4	MBP	TF	TNR	ALCAM	PLLP	OMG	CLDN11	OPALIN	KLK6	MOBP	SOX10	GPR37	CRYAB	EPHX2	PTGDS
CELLMARKERCUR_N_OSTEOBLAST		RUNX2	CD44	DLX5	PTH1R	TBX2	TBX5	TBX3	TNFRSF1…	TCIRG1	WDR5	EBF2	SP7	OSCAR	ALCAM	ITGAV	NFIL3	NT5E	PTPRC	THY1	OCSTAMP	MKI67	ACAN	CDH11	COL1A1
CELLMARKERCUR_N_OUTER_RADIAL_GLIA		FAM107A	PTPRZ1	HOPX	MOXD1																				
CELLMARKERCUR_N_OVALBUMIN_SPECIFIC_REGULA…		CD40LG	IL10	FOXP3	TNFRSF9																				
CELLMARKERCUR_N_OVARIAN_CELL		NECTIN3	EPCAM	ALDH1A1	MMP7	CD44	KLK6	PROM1																	
CELLMARKERCUR_N_OVARIAN_STROMAL_CELL		DLK1	FOXL2	STAR																					
CELLMARKERCUR_N_P53_LIKE_CELL		PCP4	ACTC1	PGM5	DES	ACTG2	MFAP4	MYH11	CNN1	FLNC															
CELLMARKERCUR_N_PAN_B_CELL		CXCR5	CCR6	CD19																					
CELLMARKERCUR_N_PAN_CANCER_ASSOCIATED_FIB…		FN1	TAGLN	ACTA2																					
CELLMARKERCUR_N_PAN_MACROPHAGE		CD68	CD163	CD69																					
CELLMARKERCUR_N_PAN_MONOCYTE		CD68	LYZ	CD14																					
CELLMARKERCUR_N_PAN_NEURONAL_CELL		MAP2	RBFOX3	SYN1	NRCAM																				
CELLMARKERCUR_N_PAN_TROPHECTODERM_CELL		ABCG2	MUC15	GATA3	CYP19A1	GATA2	OVOL1																		
CELLMARKERCUR_N_PANCREATIC_ENDODERM_CELL		F3	FOXA2	PDX1	PAX6	SOX17	NKX6-1																		
CELLMARKERCUR_N_PANCREATIC_POLYPEPTIDE_CE…		PPA1	SPP1	FOLR1																					
CELLMARKERCUR_N_PANCREATIC_PROGENITOR_CELL		SLC2A2	PROM1	NKX6-1	GP2	CD24	F3																		
CELLMARKERCUR_N_PANCREATIC_STEM_CELL		PROM1	CXCR4	LGR5																					
CELLMARKERCUR_N_PANETH_CELL		IL9	DEFA5	PLA2G2A	DEFA6	ITLN2	AMACR																		
CELLMARKERCUR_N_PANNEURON		DCLK2	STMN2	KIF1A	EEF1A2	CALY	NEFL	KCNIP2	SERPINF1																
CELLMARKERCUR_N_PANVASCULAR_CELL		ITM2A	ACKR1	ENG	ESAM	CLEC14A	CLEC1A	PCDH17																	
CELLMARKERCUR_N_PAPILLARY_FIBROBLAST		DPP4	NTN1	PDPN	COL6A1	APCDD1	COL23A1	PTGDS																	
CELLMARKERCUR_N_PARIETAL_CELL		ATP4A	ATP4B	CBLIF																					
CELLMARKERCUR_N_PARVALBUMIN_INTERNEURON		PVALB	SLIT2	ERBB4	CRABP1																				
CELLMARKERCUR_N_PATELET		PF4	TUBB1	PPBP																					
CELLMARKERCUR_N_PATHOGENIC_T_PERIPHERAL_H…		CD4	ICOS	PDCD1																					
CELLMARKERCUR_N_PELVIC_EPITHELIAL_CELL		DUOX2	SLPI	SAA2	S100P	KRT17	MUC4	SAA1	LCN2	S100A9															
CELLMARKERCUR_N_PERICYTE		CD34	MCAM	NGFR	PECAM1	PTPRC	CSPG4	ACTA2	RGS5	NT5E	PPARG	DLK1	DPP4	ICAM2	THY1	CD44	NOTCH3	PDGFRB	AMBP	ANPEP	ABCC9	CD248	FOXC1	FOXF2	KCNJ8
CELLMARKERCUR_N_PERIODONTAL_LIGAMENT_STEM…		CD34	PECAM1	PTPRC																					
CELLMARKERCUR_N_PERIOSTEUM_DERIVED_PROGEN…		ALCAM	CD9	PTPRC	THY1																				
CELLMARKERCUR_N_PERITUBULAR_MYOID_CELL		ACTA2	PTCH1	AEBP1																					
CELLMARKERCUR_N_PERIVASCULAR_LIKE_CELL		PDGFRB	MCAM	ACTA2																					
CELLMARKERCUR_N_PHAGOCYTE		PODXL	PTPRO	NPHS2																					
CELLMARKERCUR_N_PHOTORECEPTOR_CELL		PRRT2	CRX	RPE65	OTX2	RCVRN																			
CELLMARKERCUR_N_PIT_CELL		MUC5AC	TFF1	GKN1	GKN2																				
CELLMARKERCUR_N_PIT_MUCOUS_CELL		TFF1	MUC5AC	GKN1	GKN2																				
CELLMARKERCUR_N_PITUITARY_STEM_CELL		SOX2	LHX3	SOX9																					
CELLMARKERCUR_N_PLACENTAL_TISSUE_CELL		LIMCH1	FLT1	TAP1																					
CELLMARKERCUR_N_PLACODE_CELL		SIX1	PAX3	PAX8	PAX6																				
CELLMARKERCUR_N_PLASMABLAST_B_CELL		CD27	CD38	CD19																					
CELLMARKERCUR_N_PLATELET		PF4	ITGA2B	CD9	CD36	CD47	CD48	CD63	CD69	CD151	CSF3R	CD46	CCL5	CD84	CD226	CD109	BSG	CCR3	CNGB1	FCGR2A	ICAM2	ITGA6	ITGAV	ITGB1	ITGB3
CELLMARKERCUR_N_PLURIPOTENT_STEM_CELL		POU5F1	FUT4	ALPL	SOX2	PAX1	FOXC2	COL2A1	NODAL	TBXT	TBX18	FOXC1	MEOX1	PAX3	CDH2	MEOX2	HES7	UNCX	MIXL1	SOX9	RIPPLY1	TCF15	ANPEP	KLF4	ITGA6

Table S5: Cell state marker gene list

CELLMARKERCAN_ACINAR_CELLS		CTRB1	KLK1	RBPJL	PTF1A	CELA3A	PRSS1	SPINK1	ZG16	CEL	CELA2A	CPB1	CELA1	RNASE1	AMY2B	CPA2	CPA1	CELA3B	PNLIP	CTRB2	PLA2G1B	PRSS2	CLPS	REG1A	SYCN
CELLMARKERCUR_N_PODOCYTE_PRECURSOR		C17orf…	PPIB	OLFM3	IL1R1																				
CELLMARKERCUR_N_PORTAL_FIBROBLAST		MSLN	UPK1B	UPK3B																					
CELLMARKERCUR_N_POSTERIOR_CELL		GBX2	PAX8	SIX1																					
CELLMARKERCUR_N_PRE_B_CELL		CD38	CD34	MME	CD19	TCL1A																			
CELLMARKERCUR_N_PRE_CONVENTIONAL_DENDRITI…		CD33	NRP1	CLEC4C	IL3RA	KIT	CSF2RA																		
CELLMARKERCUR_N_PRE_DENDRITIC_CELL		CD74	HLA-DPA1	FCER1A																					
CELLMARKERCUR_N_PRECURSOR_CELL		ITGA4	TUBA1A	CD34																					
CELLMARKERCUR_N_PREHYPERTROPHIC_CHONDROCY…		TGFBI	TNFAIP6	THBS3	PTGES	PPIC	SRPX2	CRLF1	GOLGA7B	CRTAC1	ASPN	TNC	GALNT16	COL5A1	SOX11	FAP	TPPP3	ADAMTS5	MXRA5	CRIP1	F5	OSTC	AMTN	GALNT15	SEMA3C
CELLMARKERCUR_N_PRESOMITE_CELL		TBX6	MSGN1	MESP1	TBXT																				
CELLMARKERCUR_N_PRETUBULAR_AGGREGATE_CELL		CCND1	MACROH2A1	CALCA	RANBP1	LHX1	WNT4	RAN																	
CELLMARKERCUR_N_PRIMITIVE_ENDODERM_CELL		SOX17	CPN1	APOA1	GATA4	OTX2	CDH2																		
CELLMARKERCUR_N_PRINCIPAL_CELL		FXYD4	SCNN1G	AQP3	AQP2	TMEM213																			
CELLMARKERCUR_N_PRO_B_CELL		RAG1	VPREB1	CD34	CD19	MME																			
CELLMARKERCUR_N_PRO_INFLAMMATORY_MACROPHA…		MARCO	CD14	IL1B																					
CELLMARKERCUR_N_PRO_NEUTROPHIL		CD63	SPN	CEACAM8	CD81	FUT4																			
CELLMARKERCUR_N_PROLIFERATING_CELL		DUT	TYMS	MKI67	TOP2A																				
CELLMARKERCUR_N_PROLIFERATIVE_CHONDROCYTE		NOTCH2	BMP6	NGF	KDM7A-DT	GOT1	EIF4EB…	ERCC1	PUM3	GLIS3	ATP13A3	CDV3	LRRC8C	ASNS	ACTN1	CAVIN1	B3GNT5	NOP16	GPATCH4	C3orf52	SLC7A1	METRNL	RPF2	INHBA-AS1	ST3GAL1
CELLMARKERCUR_N_PROLIFERATIVE_DUCTAL_CELL		PLK1	AURKA	CDK1																					
CELLMARKERCUR_N_PROLIFERATIVE_NEURAL_PROG…		PAX6	GLI3	SOX2	TOP2A																				
CELLMARKERCUR_N_PRONEURAL_GLIOBLASTOMA_CE…		STMN4	ERBB3	P2RX7	SOX10																				
CELLMARKERCUR_N_PROSPERMATOGONIA		PAGE2B	RHOXF1	DDX4	PIWIL4	TEX15	MORC1	MAGEC2	MAGEB2																
CELLMARKERCUR_N_PROSTATE_CELL		AR	NKX3-1	ERG																					
CELLMARKERCUR_N_PROXIMAL_CELL		TK1	CENPM	UBE2T																					
CELLMARKERCUR_N_PROXIMAL_SEGMENT_CELL		C1orf2…	PIFO	CCDC198	CFAP126																				
CELLMARKERCUR_N_PROXIMAL_STEM_CELL		PLA2G2A	CASP1	GRIA4																					
CELLMARKERCUR_N_PROXIMAL_TUBULE_CELL		ACE2	GPX3	ALDOB																					
CELLMARKERCUR_N_PULMONARY_NEUROENDOCRINE_…		INSM1	ASCL1	HOXB5	CALCA	CHGA																			
CELLMARKERCUR_N_PYRAMIDAL_CELL		PCP2	CD3E	EMX1	MAP2	TBR1	FEZF2	DLG4	POU3F1																
CELLMARKERCUR_N_QUIESCENT_T_STEM_CELL		S1PR1	SELL	CD27	IL7R	KLF2	LEF1	TCF7																	
CELLMARKERCUR_N_REGULATORY_B_CELL		CD19	CD27	CD38	CD24																				
CELLMARKERCUR_N_REGULATORY_INNATE_LYMPHOI…		IL7R	IL10	KLRB1																					
CELLMARKERCUR_N_RENAL_TUBULAR_CELL		CCL2	ALCAM	TFPI																					
CELLMARKERCUR_N_RESIDENT_MEMORY_T_CELL		F13A1	MRC1	LYVE1	ITGAE	CD8A																			
CELLMARKERCUR_N_RESPIRATORY_HORIZONTAL_BA…		SOX2	KRT5	TP63																					
CELLMARKERCUR_N_RESPIRATORY_SECRETORY_CELL		TFF3	CYP4B1	MUC5AC																					
CELLMARKERCUR_N_RESPONDER_T_CELL		CD4	IL2RA	CD101	CNGB1																				
CELLMARKERCUR_N_RESPONDING_CONVENTIONAL_T…		IFNG	CSF2	IL22	ZBED2	GZMB																			
CELLMARKERCUR_N_RESTING_MEMORY_B_CELL		CD19	CR2	CD27																					
CELLMARKERCUR_N_RESTING_NAIVE_B_CELL		CD24	CD38	CD19																					
CELLMARKERCUR_N_RESTING_REGULATORY_T_CELL		LEF1	FOXP3	IL2RA																					
CELLMARKERCUR_N_RETICULAR_FIBROBLAST		CNN1	ACTA2	COL11A1	CD36	MGP	MFAP5																		
CELLMARKERCUR_N_RETINA_CELL		SPP1	STAC2	PCDH9																					
CELLMARKERCUR_N_ROD_CELL		CNGA1	PDE6A	RHO	PPEF2	NR2E3	NRL	GNGT1	SAG																
CELLMARKERCUR_N_SATELLITE_CELL		APOC1	APOE	PAX7																					
CELLMARKERCUR_N_SEPTUM_TRANSVERSUM_MESENC…		PDGFRB	HLX	PDGFRA	LHX2	FOXF1																			
CELLMARKERCUR_N_SEROUS_CELL		AMY1A	LPO	STATH																					
CELLMARKERCUR_N_SIDE_POPULATION_CELL		ITGA6	CD24	CD44																					
CELLMARKERCUR_N_SKELETAL_MUSCLE_CELL		MYL1	MYBPH	TNNC2	TNNI1																				
CELLMARKERCUR_N_SKELETAL_MUSCLE_STEM_CELL		NGFR	PAX7	CD34																					
CELLMARKERCUR_N_SMOOTH_MUSCLE_CELL		PECAM1	MCAM	TAGLN	ACTA2	CNN1	MYH11	TPM2	DES	ACTG2	NOTCH3	ACTG1	MYLK	MYL9	RGS5	CALD1	MLNR	SYNPO2	MYOCD	ANGPT2	GATA3	HOPX	MEF2C	PDGFRB	PLN
CELLMARKERCUR_N_SMOOTH_MUSCLE_LIKE_CELL		ACTA2	TPM2	TAGLN																					
CELLMARKERCUR_N_SOMATIC_CELL		SOX9	PECAM1	HSD3B1	ACTA2	NR2F2	VIM																		
CELLMARKERCUR_N_SOMATIC_STEM_CELL		LGR5	PROM1	NCAM1	TACSTD2																				
CELLMARKERCUR_N_SOMATOSTATIN_INTERNEURON		SST	CALB1	NPY	PDE1A	RALYL																			
CELLMARKERCUR_N_SPECIALIST_ANTIGEN_PRESEN…		CD5	CD80	CD86	CD33	CD34	CD38	CD40	CD48	CD68	CD81	CXCR3	CD83	CD209	CD207	TLR7	ENTPD1	CDH1	CCR6	CR2	CSF1R	FCER2	IL3RA	IL7R	ITGAE
CELLMARKERCUR_N_SPERMATOGENIC_CELL		CD9	GFRA1	KIT	LIN28A	ADGRA3	DDX4	EPCAM	UTF1	UCHL1															
CELLMARKERCUR_N_SPERMATOGONIAL_CELL		SYCP3	NR6A1	MORC1	PIWIL1	YBX2	ACRV1	SPACA4	UCHL1	DAZL	SPATA16	FMR1	BRDT	ZBTB43	SYCP1	L1TD1	DMRT1	SYCP2	H1-7	BEND4	ID4	SPACA3	ITGA6	PLPPR3	UTF1
CELLMARKERCUR_N_SPERMATOGONIAL_STEM_CELL		THY1	ADGRA3	ID4	UCHL1	DDX4	FGFR3	UTF1	GFRA1	ITGA6															
CELLMARKERCUR_N_SQUAMOUS_DIFFERENTIATION_…		DSC3	DSG3	DSC1	DSG1	DSC2	S100A7	S100A8																	
CELLMARKERCUR_N_STELLATE_CELL		ADIRF	PDGFRB	RGS5	ACTA2	NOTCH3	COL1A2																		
CELLMARKERCUR_N_STEM_CELL_DERIVED_ALPHA_C…		ISL1	CD36	GCG	IRX2	ARX																			
CELLMARKERCUR_N_STEM_CELL_DERIVED_BETA_CE…		ISL1	PAX4	NKX6-1	PDX1	INS																			
CELLMARKERCUR_N_STEM_CELL_DERIVED_ENDOTHE…		TPH1	FEV	SLC18A1	LMX1A																				
CELLMARKERCUR_N_STEM_LIKE_CD8_T_CELL		CD8A	PDCD1	HNF1A																					
CELLMARKERCUR_N_STROMAL_CELL		NT5E	CD44	THY1	GREM1	ANPEP	MME	ITGB1	PTPRC	ITGA1	ALCAM	CD34	MCAM	NGFR	FAP	ACTA2	TAGLN	CD3D	APOE	CXCL12	JUNB	PECAM1	CD248	HLA-A	HLA-B
CELLMARKERCUR_N_SUBPALLIUM_CELL		ANGPT2	NR2F1	NR2F2	NKX2-1	MEIS2	ETV1	ZFHX3	LHX6																
CELLMARKERCUR_N_SUBPLATE_CELL		CRYM	NR4A2	HS3ST4	WNT7B																				
CELLMARKERCUR_N_SUPPRESSIVE_MONOCYTE		CD33	CD14	IL3RA	CEACAM8	FUT4	FCGR3A																		
CELLMARKERCUR_N_SUPPRESSOR_T_CELL		SELL	CD4	CD8A																					
CELLMARKERCUR_N_SUPRABASAL_CELL		NOTCH3	KRT19	KRT5																					
CELLMARKERCUR_N_SURFACE_ECTODERM_CELL		TFAP2A	HAND1	WNT6																					
CELLMARKERCUR_N_SUSTENTACULAR_CELL		GPX6	ERMN	SOX2	CYP2A13	CYP2J2																			
CELLMARKERCUR_N_SYMPATHOADRENAL_CELL		GATA3	PHOX2A	PHOX2B	CARTPT	ISL1	STMN2	HAND1																	
CELLMARKERCUR_N_SYNCYTIOTROPHOBLAST		SDC1	CD63	CGA	S100P																				
CELLMARKERCUR_N_SYNTHETIC_SMOOTH_MUSCLE_C…		TPM4	MGP	MYH10																					
CELLMARKERCUR_N_T_CELL_LARGE_GRANULAR_LYM…		FOS	FCGR3A	CCL4	IFNG	CCL5																			
CELLMARKERCUR_N_T_CELL_LINEAGE		DPP4	CD2	CD5	CD7	CD4																			
CELLMARKERCUR_N_T_HELPER_CELL		CD4	SELL	CCR6	CCR4	CD52	TRAC	TRBC2	CD3D	CD40LG	CCR7	IL32	CD3E	IL7R	CXCR3	CXCR5	PDCD1	BTLA	B3GAT1	ICOS	CD5	CD7	CD34	CD38	CCR10
CELLMARKERCUR_N_T_MEMORY_STEM_CELL		CD58	CCR7	FAS																					
CELLMARKERCUR_N_T2_MZP_REGULATORY_B_CELL		CR2	PTPRC	FCER2																					
CELLMARKERCUR_N_TENDON_CELL		SCX	COL1A1	TNMD	DCN																				
CELLMARKERCUR_N_TERMINAL_DIFFERENTIATED_B…		CD38	CD27	MME	CD19																				
CELLMARKERCUR_N_THALAMIC_CELL		PAX6	TCF7L2	OTX2	DBX1	GBX2																			
CELLMARKERCUR_N_THECA_INTERNA_CELL		CYP17A1	OGN	RAMP2	FBLN5																				
CELLMARKERCUR_N_THYMIC_EPITHELIAL_CELL		ITGA6	ZBED2	CCN2																					
CELLMARKERCUR_N_TISSUE_LIKE_MEMORY_B_CELL		FCRL4	CR2	CD27																					
CELLMARKERCUR_N_TISSUE_RESIDENT_CELL		CXCR6	CD69	CXCR3	ITGAE																				
CELLMARKERCUR_N_TISSUE_RESIDENT_EFFECTOR_…		CD69	ITGAE	ITGA1																					
CELLMARKERCUR_N_TISSUE_RESIDENT_MACROPHAGE		PTPRC	CD68	CD14																					
CELLMARKERCUR_N_TISSUE_RESIDENT_MEMORY_LI…		HNF1A	ITGAE	CD69																					
CELLMARKERCUR_N_TOLDC_INDUCED_REGULATORY_…		CCR4	PDCD1	ENTPD1	ICOS	CD28	CD38																		
CELLMARKERCUR_N_TONSIL_CELL		CD27	FAS	ENTPD1	CD38	RB1	NT5E																		
CELLMARKERCUR_N_TRABECULAR_MESHWORK_CELL		CHI3L1	MYOC	MGP																					
CELLMARKERCUR_N_TRANSITIONAL_B_CELL		CD19	CD27	CD38	CD24	MS4A1	CD5	MME	PTPRC																
CELLMARKERCUR_N_TRANSITIONAL_DENDRITIC_CE…		CD5	CD33	THBD	CX3CR1																				
CELLMARKERCUR_N_TRUNCATED_RADIAL_GLIAL_CE…		FOXJ1	NR4A1	CRYAB	DDIT3	ENO1	PGK1																		
CELLMARKERCUR_N_TUBULAR_CELL		SPP1	LRP2	SLC22A6	ATP1A1																				
CELLMARKERCUR_N_TUMOR_ASSOCIATED_MICROGLI…		AIF1	CX3CR1	PTPRC																					
CELLMARKERCUR_N_TUMOR_ENDOTHELIAL_CELL		SBSN	ACKR1	POSTN																					
CELLMARKERCUR_N_TUMOR_EPITHELIAL_CELL		MDK	SOX4	EPCAM																					
CELLMARKERCUR_N_TUMOR_INFILTRATING_REGULA…		CD83	CCRL2	TNFRSF9	TIGIT	CCR8	ICOS																		
CELLMARKERCUR_N_TUMOR_INFILTRATING_T_CELL		CD8A	CD4	FOXP3																					
CELLMARKERCUR_N_TUMOR_INITIATING_CELL		CD44	CD24	CD47	PBX3																				
CELLMARKERCUR_N_TUMOR_REGULATORY_T_CELL		CCR8	FOXP3	RTKN2																					

CELLMARKERCAN_ACINAR_CELLS		CTRB1	KLK1	RBPJL	PTF1A	CELA3A	PRSS1	SPINK1	ZG16	CEL	CELA2A	CPB1	CELA1	RNASE1	AMY2B	CPA2	CPA1	CELA3B	PNLIP	CTRB2	PLA2G1B	PRSS2	CLPS	REG1A	SYCN
CELLMARKERCUR_N_UMBRELLA_CELL		KRT20	UPK2	UPK3A	TSPAN1	PLAT	FXYD3																		
CELLMARKERCUR_N_UNRESTRICTED_SOMATIC_STEM…		THY1	ALCAM	PTPRC	PECAM1	CD34	NT5E	CD44																	
CELLMARKERCUR_N_UNSWITCHED_PLASMABLAST		CD38	CD19	CD27																					
CELLMARKERCUR_N_URETERIC_BUD_CELL		KRT18	KRT8	MMP7	RET																				
CELLMARKERCUR_N_UROTHELIAL_CELL		GATA3	RPE65	KRT8	KRT18	EPCAM																			
CELLMARKERCUR_N_UROTHELIUM_CELL		TP63	DHRS2	KRT5	UPK1B	S100P	NECTIN4																		
CELLMARKERCUR_N_VASCULATURE_CELL		TIMP3	PLVAP	SLC14A1	PECAM1																				
CELLMARKERCUR_N_VEIN_CELL		MMRN1	ACKR1	NR2F2																					
CELLMARKERCUR_N_VENOUS_CELL		EPHB4	NR2F2	NRP2	ICAM1	FOS	LRG1	EMCN																	
CELLMARKERCUR_N_VENTRAL_LATERAL_GANGLIONI…		ISL1	EBF1	IGFBP5	ZNF503	TAC1																			
CELLMARKERCUR_N_VENTRAL_MEDIAL_GANGLIONIC…		LHX8	ZIC1	ZIC2	ZIC4																				
CELLMARKERCUR_N_VENTRICULAR_CELL		MYL2	IRX4	HEY2																					
CELLMARKERCUR_N_YOUNG_NEURON		DCX	NCAM1	STMN1	SYP																				
CELLMARKERCUR_N_ZONA_FASCICULATA_CELL		CYP11B1	CYP11A1	STAR	CYP17A1																				
CELLMARKERCUR_N_ZONA_GLOMERULOSA_CELL		CYP11B2	DAB2	AGTR1																					
CELLMARKERCUR_N_ZONA_RETICULARIS_CELL		AKR1B1	STAR	CYP17A1	TSPAN12	SULT2A1																			
CELLMARKERCUR_N_ZYGOTE		H1-8	ACCSL	CLEC10A																					
CELLMARKERCURNACTIVATEDEFFECTORMEMORYCD4T…		PDCD1	CD28	CD4	FAS																				
CELLMARKERCURNACTIVATEDTISSUERESIDENTMEMO…		CD28	FAS	PDCD1	CD4																				
CELLMARKERCURNACTIVATEDTISSUERESIDENTMEMO…		ITGAE	CXCR5	FCGR3A	CD69	CD8A	FAS																		
CELLMARKERCURNALPHACELLACELL		ARX	FAP	FEV	TM4SF4	GCG	PAX6																		
CELLMARKERCURNALVEOLARTYPEIIATIICELL		LAMP3	SFTPC	MUC1	ABCA3	SFTPB	SFTPA1																		
CELLMARKERCURNATYPICALBCELLABC		CD72	LILRB2	FCRL5	ITGAX																				
CELLMARKERCURNAXLCD11CPLASMACYTOIDDENDRIT…		CD5	CD33	CD2																					
CELLMARKERCURNAXLCD123PLASMACYTOIDDENDRIT…		CLEC4C	IL3RA	CD2	CD5																				
CELLMARKERCURNBASALFOREBRAINCHOLINERGICNE…		NGFR	SLC18A3	CHAT	ISL1																				
CELLMARKERCURNBRUSHCELLTUFTCELL		DCLK1	RGS13	ASCL2	POU2F3	IRAG2	TRPM5	AVIL																	
CELLMARKERCURNCANCERASSOCIATEDFIBROBLASTC…		FAP	ACTA2	DCN																					
CELLMARKERCURNCAPSULARFIBROBLASTFB2		SEMA3D	FBN1	PI16																					
CELLMARKERCURNCCR7DENDRITICCELL		CCL22	CD83	CCR7	LAMP3	FSCN1	IDO1	BIRC3																	
CELLMARKERCURNCD106MESENCHYMALSTEMCELL		PLAUR	VCAM1	CD248	CD47																				
CELLMARKERCURNCD14MACROPHAGE		CD209	CD14	FCGR1A																					
CELLMARKERCURNCD16DENDRITICCELL		IL3RA	CD14	CEACAM8	CD33	FUT4	FCGR3A																		
CELLMARKERCURNCD25LAG3TCELL		IL2RA	LAG3	CD4																					
CELLMARKERCURNCD34GRANULOCYTICPROGENITORC…		PRTN3	ELANE	PRSS57																					
CELLMARKERCURNCD34HEMATOPOIETICSTEMCELL		PRDX1	AVP	SPINK2																					
CELLMARKERCURNCD34PREBCELL		IGLL1	STMN1	VPREB1	CD79B																				
CELLMARKERCURNCD34PROGENITORCELL		CD34	HOXA9	PROM1																					
CELLMARKERCURNCD34THYMOCYTE		CD7	IL3RA	CD5	CD34	CD44																			
CELLMARKERCURNCD40LGTHELPERCELL		GZMK	CD40LG	IFNG	GZMA	GZMH	CD69	CD3D																	
CELLMARKERCURNCD4CD25REGULATORYTCELL		CD4	IL2RA	PTPRC																					
CELLMARKERCURNCD4CENTRALMEMORYLIKETTCMLIK…		SELL	CD28	CD3E	CCR7	CD27	CD4	TCF7																	
CELLMARKERCURNCD4CYTOTOXICT1CELL		CCL4	CD27	PRF1	CD28	GZMB	CD4	CCL3	NKG7	CD3E															
CELLMARKERCURNCD4CYTOTOXICT2CELL		CCL5	CD28	NKG7	CD3E	CD4	GZMB	CD27																	
CELLMARKERCURNCD4THELPERCELL		CD4	PTPRC	CD3D	KLRB1	PTGDR2																			
CELLMARKERCURNCHRONICALLYACTIVATEDCD4TCELL		PTPRC	CD4	CD38																					
CELLMARKERCURNCHRONICALLYACTIVATEDCD8TCELL		CD8A	PTPRC	CD38																					
CELLMARKERCURNCLASSSWITCHEDMEMORYBIGGU03B…		CD19	MS4A1	CD27																					
CELLMARKERCURNCLEC10ADENDRITICCELL		LYZ	CLEC10A	CST3	C1QA	CD1C	CD74	C1QB	FCER1A																
CELLMARKERCURNCLEC9ADENDRITICCELL		CLEC9A	LYZ	THBD	CD74	CST3																			
CELLMARKERCURNCONVENTIONALDENDRITICCELL2B…		CD33	CD163	CD36																					
CELLMARKERCURNCONVENTIONALDENDRITICCELL2B…		CD300E	FCER1G	TSPO	CD14	CD163	FCN1	IL1B	F13A1	ZEB2	S100A9	VCAN	ANXA2	LGALS3	LYZ	CCR2									
CELLMARKERCURNCORTICALSOMATOSTATINSSTINTE…		CORT	NPY	SST																					
CELLMARKERCURNCYTOTOXICCD4T2CELL		NKG7	PRF1	GZMB																					
CELLMARKERCURNCYTOTOXICCD4TCELL		B3GAT1	PRF1	GZMB	CD4	GZMA	GNLY	NKG7																	
CELLMARKERCURND1MSNMEDIUMSPINYNEURON		TAC1	ISL1	EBF1																					
CELLMARKERCURND2MSNMEDIUMSPINYNEURON		GRIK3	SIX3	SP9	SIX3-AS1																				
CELLMARKERCURNDISTALTUBULELOOPOFHENLECELL		MAL	HOXD8	TFCP2L1	SLC12A1																				
CELLMARKERCURNEARLYCD34MEGAKARYOCYTEERYTH…		SERPIN…	HSP90AB1	CYTL1	TMEM14C																				
CELLMARKERCURNEARLYTCELLPROGENITORETP		CD7	LYL1	CD34																					
CELLMARKERCURNEFFECTORMEMORYCD4TCELL		CD28	FAS	IL2RA	FOXP3	IL7R	CXCR5	CD4	GPR183	GZMA	S100A4	TSPAN14	SPATA7	SATB1	SKAP2	TEF	TMED10	LMAN2	SBNO2	PRR7-A…	TBC1D5	TNFSF15	PRR7	UQCRQ	PHF5A
CELLMARKERCURNEFFECTORREGULATORYTETREGCELL		CD4	IL2RA	GNLY	PRF1	GZMH	GZMA	CD44	ICOS																
CELLMARKERCURNEFFECTORTEFFCELL		B3GAT1	CD4	IL2RA	GZMK	PRDM1	TBX21	CXCR3	RORC	RUNX3	STAT4	IL23R	CD2	NKG7	CD8B	CCR5	PDCD1	FGFBP2	CX3CR1	FCGR3A	KLRF1	MKI67	PRF1	CD27	GZMA
CELLMARKERCURNEXHAUSTEDCD4TCELL		PDCD1	LAG3	TIGIT	HAVCR2	CD4	CD3D	CD3E	CXCL13	CTLA4															
CELLMARKERCURNEXHAUSTEDCD8TCELL		TIGIT	CTLA4	PDCD1	HAVCR2	LAG3	CD3E	CD8A	CD3D	HLA-DR…	ENTPD1	KRT86	LY6E	IFI6	IFI27	HLA-DQA2	IFI44	OAS1	PRF1	CXCL13	HLA-DRB5	HLA-DRA	ALOX5AP	MX1	GZMB
CELLMARKERCURNEXHAUSTEDCD8TPROGENITORCELL		PDCD1	HNF1A	CXCR5	CD8A																				
CELLMARKERCURNEXHAUSTEDTTEXCELL		TIGIT	CD160	LAG3	PDCD1	CTLA4	CD244	HAVCR2	GZMB	GPR25	INPP5F	B3GAT1	ZEB2	EOMES	TNFRSF1A	PRDM1	TOX2	NFATC3	TNFRSF1B	PTGER4	IRF7	NFATC1	NFATC2	PTGER2	BHLHE40
CELLMARKERCURNEXTRATELENCEPHALICETCELL		POU3F1	ANKRD34B	LRATD2																					
CELLMARKERCURNFINALLYHIGHLYEFFECTORTEMRAM…		GZMH	CST7	NKG7	GZMA	CCL5	CCL4	GNLY																	
CELLMARKERCURNFOLLICULARREGULATORYTTFRCELL		IL1R2	CTLA4	CXCR5																					
CELLMARKERCURNFOXP3NATURALREGULATORYTTREG…		CD6	CD4	IL2RA																					
CELLMARKERCURNGAMMADELTAU03B3DTCELL		NCAM1	TRDV2	TRGV9	TRDC	CD3D	TRGC1	GZMH	SLC4A10	GNLY	NKG7	CCL5	CD5	CD7	CD2	CD8B	CD8A	KLRB1	CD4	CD3E	TRGC2	CXCR6	CCR5	FFAR4	SERTM1
CELLMARKERCURNGERMINALCENTREGCLIKECELL		BHLHE40	BCL6	HMGB2	MKI67																				
CELLMARKERCURNGLOMERULARENDOTHELIALCELLECG		EMCN	PECAM1	PLVAP																					
CELLMARKERCURNGRANULOCYTICMYELOIDDERIVEDS…		FUT4	CD80	CD33																					
CELLMARKERCURNGRBREGULATORYBCELL		CD38	BSG	CD19																					
CELLMARKERCURNGZMKTCELL		IFNG	GZMA	GZMH	GZMK	CD8B	CD3D	LAG3	CD8A	CD69															
CELLMARKERCURNHIGHLYDIFFERENTIATEDEFFECTO…		CD244	KLRG1	B3GAT1																					
CELLMARKERCURNINDUCEDREGULATORYTTREGCELL		CD4	IL2RA	IL7R	FOXP3																				
CELLMARKERCURNINFLAMMATORYCANCERASSOCIATE…		COL1A1	PDGFRA	DPT	CFD																				
CELLMARKERCURNINNATELYMPHOIDCELLILCS		ID2	IL1R1	KIT	ZBTB16																				
CELLMARKERCURNINTRATHYMICFIBROBLASTFB1		GDF10	COLEC11	ALDH1A2																					
CELLMARKERCURNITOCELLHEPATICSTELLATECELL		FAP	GFAP	LRAT	NGFR																				
CELLMARKERCURNLYMPHANGIOLEIOMYOMATOSISCEL…		CTSK	PMEL	ESR1	MYH11	PGR	ACTA2	VEGFD																	
CELLMARKERCURNMARGINALZONEMZLIKEBCELL		CD27	CD19	MS4A1																					
CELLMARKERCURNMEMORYSTEMCELLTSCM		CD28	CXCR3	FAS	ITGAL	IL7R	CD27	IL2RB	CCR7	SELL															
CELLMARKERCURNMICROGLIADERIVEDTUMORASSOCI…		TMIGD3	APOC2	SCIN																					
CELLMARKERCURNMKERYTHCELL		HBZ	HBM	ITGB3	GP6																				
CELLMARKERCURNMYOFIBROBLASTICCANCERASSOCI…		RGS5	TAGLN	TPM1	POSTN	MYL9	TPM2	HOPX	MMP11																
CELLMARKERCURNNAIVEREGULATORYTTREGCELL		CD4	IL2RA	IL7R																					
CELLMARKERCURNNATURALREGULATORYTTREGCELL		CD4	IL2RA	IL7R	IKZF2	CD6																			
CELLMARKERCURNPERIPHERALBLOODMONONUCLEARC…		CD33	CD4	CD19																					
CELLMARKERCURNPITUITARYSTEMCELLPSC		RBPMS	SOX9	LGR4																					
CELLMARKERCURNPLASMACYTOIDDENDRITICCELLPDC		SPIB	IL3RA	LILRA4	TLR7	CLEC4C	CD4	NRP1	IRF7	TLR9	TCF4	CCR7	CD19	CD38	CD27	MS4A1	MZB1	ITM2C	GZMB	SERPIN…	CYTL1	GATA2	IRF8	JCHAIN	MPEG1
CELLMARKERCURNPOLYMORPHONUCLEARMYELOIDDER…		ITGAM	CAMP	S100A9	S100A8	MMP8	OLR1	ARG1	LCN2																
CELLMARKERCURNPOLYMORPHONUCLEARNEUTROPHIL…		ALPL	ADGRG3	PROK2	IL1R2	CSF3R																			
CELLMARKERCURNPOSTERIORPRESOMITICMESODERM…		TBX6	RSPO3	MSGN1																					
CELLMARKERCURNPROLIFERATIVECD4TCELL		TYMS	CD4	CD3E	CD3D	PTPRC																			
CELLMARKERCURNPROLIFERATIVECD8TCELL		CD8A	CD3E	TYMS	CD3D	PTPRC																			
CELLMARKERCURNREDBLOODCELLERYTHROCYTE		HBA1	HBB	HBA2	CD24	GYPA	PTPRC	TFRC	ITGA4	SPI1	ALAS2	AHSP	HBQ1	SLC4A1	RHCE	HBG2	HBG1	HBM	CA1	KIT	CD36	CD34	HBD	ENG	TFR2
CELLMARKERCURNREGULATORYBBREGCELL		CD24	CD19	CD27	TFRC	IL10	IL2RA	CD38	CD5	CD1D															
CELLMARKERCURNREGULATORYCD4TCELL		IL2RA	CD4	TIGIT	CTLA4	FOXP3	CD3E	CD3D	GEM	TNFRSF4															
CELLMARKERCURNREGULATORYCD8TCELL		CD8A	CD3D	FOXP3	CD3E	CD28	IL10																		
CELLMARKERCURNREGULATORYTTREGCELL		FAS	IL2RA	FOXP3	PDCD1	ENTPD1	GZMB	CD4	HAVCR2	CD8A	CTLA4	TNFRSF4	TIGIT	TNFRSF9	TNFRSF18	IL7R	CCR7	CCR4	KLRB1	ICOS	CORO1B	FANK1	ARID5B	RTKN2	GBP2

Table S5: Cell state marker gene list

CELLMARKERCAN_ACINAR_CELLS		CTRB1	KLK1	RBPJL	PTF1A	CELA3A	PRSS1	SPINK1	ZG16	CEL	CELA2A	CPB1	CELA1	RNASE1	AMY2B	CPA2	CPA1	CELA3B	PNLIP	CTRB2	PLA2G1B	PRSS2	CLPS	REG1A	SYCN
CELLMARKERCURNREGULATORYTTREGCELL		CD4	FOXP3	IL2RA	IL2RB	ENTPD1	CD101	TNFRSF18	CNGB1	FCRL3	TIGIT	IL7R	CCR6	CCR4	TRAC	IL32	TRBC2	CD3D	CTLA4	IL10	NT5E	NRP1	IKZF2	LAG3	STAT3
CELLMARKERCURNRENALVESICLECOMMASHAPEDBODY…		KLK6	LAMP5	SFRP2	CCND1	PCP4	PAX8	LHX1	EMID1																
CELLMARKERCURNRESIDENTMEMORYCD8TCELL		GPR15	ITGAE	IL7R	CD69																				
CELLMARKERCURNREVERTANTMEMORYCD8TTEMRACELL		CD8A	CX3CR1	PTPRC																					
CELLMARKERCURNSCARASSOCIATEDMACROPHAGESAM		CD9	TREM2	CAPG	GPNMB																				
CELLMARKERCURNSENESCENTCD8TCELL		B3GAT1	TIGIT	CD27	CD28	KLRG1																			
CELLMARKERCURNSTEMPROGENITORCELL		KRT5	KRT15	TP63																					
CELLMARKERCURNSWITCHEDMEMORYBCELLSWMB		CD19	CD27	CD38	PTPRC																				
CELLMARKERCURNT1TRANSITIONALBCELL		CD19	MME	PTPRC																					
CELLMARKERCURNT2TRANSITIONALBCELL		PTPRC	CD19	MME																					
CELLMARKERCURNTCELLNATURALKILLERCELL		TRAC	KLRB1	CD3E	GZMA	CD2																			
CELLMARKERCURNTERMINALEFFECTORCD8TCELL		NKG7	GZMA	PRF1	GZMB																				
CELLMARKERCURNTERMINALLYDIFFERENTIATEDCD8…		ENTPD1	HAVCR2	TOX																					
CELLMARKERCURNTFOLLICULARHELPERTFHCELL		ICOS	CXCR3	CCR6	CXCR5	CD4	PDCD1	CXCL13	BCL6	MME	TIGIT	IL21	CD200	ICA1	TOX2	CD40LG	GPR183	MALAT1	LTB	TNFRSF4	MAF	CCR5	APCS	MYO7A	SLC7A10
CELLMARKERCURNTHELPER1TH1CELL		CXCR3	CD4	CCR6	CCR1	CCR5	STAT1	STAT4	TNF	DPP4	IFNGR1	IL2	IL12RB1	IL18	KLRD1	TNFSF11	IL18R1	CXCR6	TBX21	HAVCR2	IL27				
CELLMARKERCURNTHELPER1TH1CELL		CXCR3	CD4	CXCR5	CCR5	KLRB1	CCR6	CCR4	IFNG	CCL4	TBX21	TNF	STAT1	STAT4	ZEB2	ATOH7	PTPRC	LRP8	DPP4	SGCB	IGHD	LTA	IL12RB2	CTLA4	BST2
CELLMARKERCURNTHELPER2TH2CELL		CCR4	CD4	CCR6	GATA3	CCR3	CXCR3	TAF1B	IFIT3	IFIT2	STAT6	IL13	STAT5A	HPGDS	PTGDR2	IL1RL1	IL10	PDCD1	IL4	CD200R1	TCN1	PTPRC	AADACL2	KIAA0319	FLG
CELLMARKERCURNTHELPER2TH2CELL		IL4	CCR4	CD4	TNFRSF8	CCR6	CXCR3	CCR8	GATA3	IRF4	STAT6	BATF	CCR3	CCR7	IL2	IL5	IL6	IL9	IL10	IL13	STAT5A	CXCR4	PTGDR2	HAVCR1	ICOS
CELLMARKERCURNTHELPER9TH9CELL		CD4	GATA3	IRF4	STAT6	IL4	IL9	IL10																	
CELLMARKERCURNTHELPERTHCELL		CD4	WAS	RORC	IL17RE	IL6ST	CD3E	IL2RA	KIR2DL4	KIR3DP1	CXCR5	ZFP36L2	CD38	PDCD1	CXCR3	GZMA	CCL5	EGFR	STAT6	TP63	PRDM1	TBX21	BCL6	GATA3	CCR6
CELLMARKERCURNTHYMICEPITHELIALCELLTEC		CXCL12	KRT5	PSMB11	CCL25	KRT8	DLL4	PRSS16	FOXN1																
CELLMARKERCURNTISSUERESIDENTMEMORYCD4TCELL		FAS	IL2RA	CD69	CD4	CXCR6	RUNX3																		
CELLMARKERCURNTISSUERESIDENTMEMORYCD8TCELL		CD8A	CD69	CXCR5																					
CELLMARKERCURNTISSUERESIDENTMEMORYTRMCELL		CD69	ITGAE	CD101	ITGA1	CCR5	PDCD1	CXCR6	FASLG	NUSAP1	DUSP4	CCR8	CD44	AHR	ZNF683	S1PR1	CRTAM	FOLR2	COLEC12	LYVE1	C1QA	F13A1	KLRB1	IL7R	CCL4L2
CELLMARKERCURNTPERIPHERALHELPERTPHCELL		CXCR3	PDCD1	CCR2	CD4																				
CELLMARKERCURNTRANSIENTAMPLIFYINGTACELL		MYC	CCL2	OLFM4																					
CELLMARKERCURNTRANSITIONALEXHAUSTEDCD8TCE…		CSF1	DUSP4	PDCD1	GZMK	HAVCR2																			
CELLMARKERCURNTUMORINFILTRATINGLYMPHOCYTE…		CD8A	CD4	FOXP3																					
CELLMARKERCURNUNSWITCHEDMEMORYBCELLUNSWMB		CD19	CD27	MS4A1																					
CELLMARKERCURNVASARECTAENDOTHELIALCELLECVR		CLDN5	FLT1	PECAM1																					
CELLMARKERCURNVONECONOMONEURONVEN		ITGA4	GABRQ	BMP3	ADRA1A	POU3F1																			
CELLMARKERCURNYOLKSACDERIVEDMYELOIDBIASED…		CD44	PTPRC	CD34	MYB																				
CELLMARKEREXP_ACINAR_CELLS		CTRB1	KLK1	RBPJL	PTF1A	CELA3A	PRSS1	SPINK1	ZG16	CEL	CELA2A	CPB1	CELA1	RNASE1	AMY2B	CPA2	CPA1	CELA3B	PNLIP	CTRB2	PLA2G1B	PRSS2	CLPS	REG1A	SYCN
CELLMARKEREXP_ADRENERGIC_NEURONS		TH	DDC	DBH	PNMT	SLC18A2	NPFF	SLC12A7	SYT1																
CELLMARKEREXP_AIRWAY_EPITHELIAL_CELLS		AQP1	ADH7	CDH1	SEC14L3																				
CELLMARKEREXP_AIRWAY_SMOOTH_MUSCLE_CELLS		NOG	ACTA2	FOXF1	GATA5																				
CELLMARKEREXP_ALPHA_CELLS		GCG	TTR	GLP1R	ARX	POU3F4	NKX2-2	RESP18	PYY	PCSK2	SLC38A5	TM4SF4	CRYBA2	NKX6-1	KCNK16	PCSK1	PRRG2	IRX2	ALDH1A1	PEMT	CHGA	SMIM24	F10	SCGN	SLC30A8
CELLMARKEREXP_ANTERIOR_PITUITARY_GLAND_CE…		AGA	ANGPT1	CHGA	CPEB4	IGSF1	MEST	NKTR	NUCB2	PITX2	POU1F1	PRL	RAB27A	RCN3	SEZ6L2	SIX6	TBRG1	TMEM130	POMC	GMPPA	GNB3	HEPACAM2	ASB4	RAX	NKX2-1
CELLMARKEREXP_ASTROCYTES		S100B	SLC1A2	GFAP	BYSL	ALDH1L1	CD40	SLC1A3	FGFR3	GJB6	AQP4	HTRA1	S100A8	CLDN10	ITIH3	NKAIN4	ACSL6	ACSBG1	TRIL	AGT	GPR37L1	SLC4A4	NTSR2	SLC6A11	HSPA2
CELLMARKEREXP_B_CELLS		CD2	CD5	MS4A1	CR2	CD22	FCER2	CD40	CD69	CD70	CD79A	CD79B	CD80	CD86	TNFRSF9	SDC1	TNFSF4	TNFRSF1…	TNFRSF1…	PDCD1	IGHD	IGHM	RASGRP3	HLA-DRA	LTB
CELLMARKEREXP_B_CELLS_MEMORY		CD38	CD80	CD84	CD86	NT5E	PAX5	PTPRC	SPIB	ADAM28	AIM2	BCL7A	BACH2	BANK1	BLK	CCR6	CD180	CD19	CD1C	CD22	CD27	CD37	CD69	CD72	CD79A
CELLMARKEREXP_B_CELLS_NAIVE		CD19	CD2	CD72	IL2RA	MME	ABCB4	ADAM28	BACH2	BANK1	BCL7A	BEND5	BLK	BRAF	CD180	CD1C	CD22	CD37	CD69	CD79A	CD79B	CR2	CXCR5	EAF2	FCER2
CELLMARKEREXP_BASAL_CELLS		KRT5	KRT17	BCL2	KRT14	TP63	CDH3	KRT6B	METTL5	ANXA11	TMEM14A	BNIP3	TYMP	DPP7	OPTN	PLAU	CAPN1	BACE2	CTNNB1	ILK	PKP3	ITGA3	MT1L	SRSF4	RAB38
CELLMARKEREXP_BASOPHILS		CD69	IL3RA	CCR3	ENPP3	FCER1A	TLR4	LTC4S	CPA3	ITGAL	ITGAM	ITGAX	IL4R	ITGB7	IL4	MS4A2	MS4A3	HDC	GATA2	CCL3	CD33	CD44	CDH1	HTR1B	IGFBP7
CELLMARKEREXP_BERGMANN_GLIA		GFAP	GDF10	GJB6	GRIA4	GRM3	NTSR2	ITIH3	A2M	PTCH1	CDC42EP4	BTBD17	AQP4	SLC4A4	HOPX	GPR37L1	ACSBG1	ALDH1L1	SLC1A3	SLC1A2	S100B	VIM	FABP7	TTC21B	HEPACAM
CELLMARKEREXP_BETA_CELLS		GCGR	JPH3	CD40	HAMP	EZH1	NTRK1	PDX1	SLC2A2	NKX6-2	FXYD2	NPY	INS	RIMS1	MAFA	EFNA5	LMX1A	NKX2-2	NKX6-1	PAX4	IAPP	PCSK2	G6PC2	SLC30A8	PCSK1
CELLMARKEREXP_CAJAL_RETZIUS_CELLS		RELN	DIABLO	ST3GAL5	ISOC1	SMOC2	DYNC1I1	NHLH2	CLSTN2	TMEM163	CACNA2D2	CIB2	NDNF	LINGO1	LNPK	RNF138	TMEM…	ABCC5	CNRIP1	EBF3	FUT9	PABPN1	GNL3L	NUDT2	SLC25A46
CELLMARKEREXP_CHOLANGIOCYTES		SSTR2	SLC4A2	SCTR	KRT7	KCNN2	ITGB4	GGT6	CFTR	CCKBR	AQP4	AQP1	PIGR	GGT1	JAG1	GPBAR1	GGT7	KRT19	ONECUT2	ALPL	HNF1B	ALB	AGR2	TFF3	TFF1
CELLMARKEREXP_CHOLINERGIC_NEURONS		CHAT	SLC18A3	ACHE	SLC5A7	TAC1	BRCA1	ACLY																	
CELLMARKEREXP_CHONDROCYTES		PRG4	TNC	CRTAC1	CREB3L2	RUNX2	ACAN	COL2A1	COMP	MATN3	RUNX3	SOX6	BMP2	CTNNB1	FGF18	GLI3	PBX3	PTHLH	SMO	STAT1	MMP13	VEGFA	MATN1	COL9A1	COL10A1
CELLMARKEREXP_DELTA_CELLS		PDX1	FFAR4	SST	PCSK9	RBP4	PAX4	RESP18	HHEX	ISL1	SCGN	KCNK16	IAPP	FXYD2	NPTX2	GPC5-AS1	UCP2	LEPR	BAIAP3	MS4A8	CASR	BCHE	GABRB3	UNC5B	EDN3
CELLMARKEREXP_DISTAL_TUBULE_CELLS		CLDN8	SLC12A3	UMOD	JAG1	CALB1	PVALB	PGAM2	WNK4	SGMS2	SLC16A7	LHX1	ABCA13	EMX1	TRPM6	CWH43	TRPM7	UROC1	TFRC	TSC22D2	LARP1B	CLCNKB	KLHL3	KLK1	KCNJ1
CELLMARKEREXP_DUCTAL_CELLS		CFTR	HNF1B	KRT20	MUC1	ONECUT1	AMBP	HHEX	ANXA4	SPP1	PDX1	SERPINA3	CFB	GDF15	AKR1C3	MMP7	DEFB1	SERPING1	TSPAN8	CLDN10	SLPI	SERPINA5	PIGR	CLDN1	LGALS4
CELLMARKEREXP_EMBRYONIC_STEM_CELLS		MYC	FOXD3	POU5F1	FUT4	CD24	ITGA4	CDH1	ABCG2	PODXL	ALPP	ZIC1	PRDM5	ANO6	CDK8	SLC46A2	TEX19	ZSCAN10	KCNIP3	PCGF2	NANOG	GAL	PITX2	BCL3	SALL4
CELLMARKEREXP_ENTERIC_NEURONS		HIVEP3	PLEKHA5	IGF2BP3	ARHGAP26	TTBK2	ASTN1	CACNA1E	TUB	FILIP1	GCNT2	VEZT	RET	AHDC1	MAP6	RGMB	FBXO7	PPP2R5B	SNX21	FSTL5	LIX1	RGS3	CYTH3	TSHZ1	STXBP5
CELLMARKEREXP_ENTEROENDOCRINE_CELLS		NPSR1	FFAR3	SCN3A	NEUROG3	FFAR1	ARX	NKX2-2	SCT	CCK	GFRA3	VWA5B2	NEUROD1	SCGN	PCSK1	ISL1	FABP5	TAC1	RIMBP2	PAX6	GIP	GLP1R	PYY	GLP2R	GAST
CELLMARKEREXP_EPENDYMAL_CELLS		FOXJ1	TRIM71	SIX3	USP18	AQP4	CELSR2	AQP1	GFAP	ENKUR	FAM216B	KRT15	CATIP	DNAH12	FAM166B	IQCG	SPEF2	MYO16	ODF3B	PLXNB2	TEKT1	CHEK2	CROCC	NME9	CFAP126
CELLMARKEREXP_EPSILON_CELLS		GHRL	ANXA13	PHGR1	BMP4	HMGCS2	TM4SF5	OLFML3	ASGR1	COX6A2	NPY1R	FAXDC2	SLC7A9	C2ORF54	GHRLOS	BHMT	OPRK1	PTGER4	ITGA4	EYA4	XYLB	ELOVL2	AFF3	CORIN	MYO1A
CELLMARKEREXP_ERYTHROID_LIKE_AND_ERYTHROI…		ALAS2	HEXA	AQP1	CCNA2	PKLR	LOX	ARG1	HBB	HBA1	HBA2	HBG1	HBG2	CEACAM1	CD36	GYPA	THBS1	ITGA4	GYPB	AHSP	HBM	FECH	EPOR	KIT	BPGM
CELLMARKEREXP_GAMMA_DELTA_T_CELLS		GNLY	PTGDS	GZMB	S100B	FGFBP2	NKG7	PRF1	KLRF1	HOPX	CST7	KLRD1	CTSW	SPON2	IFITM1	GZMA	CD247	CLIC3	CD7	ADGRG1	CCL5	TRDC	STMN1	HMGB2	TYMS
CELLMARKEREXP_GOBLET_CELLS		CREB3L1	NLRP6	MUC5AC	MUC2	SLC9A8	OTOGL	ITLN1	AQP3	CDON	MUC4	MUC5B	TMEM207	TFF3	AGR2	CDX2	KRT7	KRT20	GUCA2A	PHGR1	MUC13	FCGBP	CLCA1	ZG16	TPSG1
CELLMARKEREXP_HEMANGIOBLASTS		ETV2	T	TAL1																					
CELLMARKEREXP_HEMATOPOIETIC_STEM_CELLS		THY1	MPL	KIT	PROM1	FLT3	SLAMF1	CXCR4	TEK	ABCB1	ABCG2	BAALC	SPN	CDCP1	MYADM	KITLG	TGFB2	PTPRE	TAF7	GATA2	GPR171	ANGPT1	PROCR	NFE2	NKX2-3
CELLMARKEREXP_HEPATIC_STELLATE_CELLS		ADAMTS…	CYGB	PPARG	PDGFRA	RGS5	PPARA	FOXF1	ALB	NR1H4	CTGF	SEMA7A	AGTR1	PNPLA3	WT1	FGF10	GFAP	NGFR	VEGFA	ACTA2	MYB	DES	FAP	SLC8A1	RELN
CELLMARKEREXP_HIS_BUNDLE_CELLS		GJA5	GJC1	KCNK3																					
CELLMARKEREXP_IMMATURE_NEURONS		PAX6	PCLO	CUX2	SLC12A5	POMC	STIM2	RAX	CUL5	HIVEP2	FUT9	TBR1	PROX1	NTRK3	RND2	FEZF2	SOX5	ROBO1	EPHA7	EPHA3	YY2	SLC1A1	NOTCH3	NOTCH1	LHX6
CELLMARKEREXP_JUXTAGLOMERULAR_CELLS		TRPV4	REN	TH	ACTA2	MYH11	CNN1																		
CELLMARKEREXP_KERATINOCYTES		CALML5	CD24	FLG	KPRP	ICAM1	IVL	TGM1	KRT1	KRT5	KRT14	KRT16	KRT17	LAMA5	LCN2	PLEC	PI3	SPRR1A	SPRR1B	GJB3	IRF3	LAMC2	SIRT7	RIPK4	REL
CELLMARKEREXP_KIDNEY_PROGENITOR_CELLS		OSR1	PAX2	EYA1	WT1	SIX2	SALL1	CITED1																	
CELLMARKEREXP_KUPFFER_CELLS		TNF	LYZ	MPO	VSIG4	PROK2	STARD5	MSR1	TLR9	PPARA	TREM1	SLC11A1	TLR4	MMP13	CD14	VDR	CHIT1	OSM	PPARD	CLEC4F	ADGRE1	IL1B	SLC40A1	G6PD	TIMD4
CELLMARKEREXP_LEYDIG_CELLS		HSD11B2	MAFB	ALDH1A1	SLC9A8	KISS1	PDE8A	LEP	CXCL10	PTCH1	GATA4	ACSBG1	INHA	HSD3B1	DHCR7	CALB2	CYP17…	CYP11A1	IGF1	INSL3	NR5A2	LHCGR	MLANA	RLN1	RLN2
CELLMARKEREXP_LOOP_OF_HENLE_CELLS		CLDN16	CLDN8	UMOD	FABP4	AQP1	CLDN10	CLDN19	SLC12A1	DEFB1	CLCNKA	TRPM7	CDH6	LCN2	DKK1	DACH1	SLC12…	SLC11A2	ARVCF	RGS5	COX4I2	CYP4B1	PARVB	SH3RF2	HEY2
CELLMARKEREXP_LUMINAL_EPITHELIAL_CELLS		FGFR2	FGG	KRT18	ANKRD30A	SLPI	PROM1	KRT19	SYTL2	CD74	AGR2	LTF	SAA2	KRT23	SERPINB4	SERPINB3	WFDC2	LCN2	BTG1	CLDN4	ANXA1	HMGA1	STC2	AREG	TNFSF10
CELLMARKEREXP_MAMMARY_EPITHELIAL_CELLS		CSN2	BTN1A1	BTN2A2	BTN3A1	MMP14	TOX3	CDH1	FFAR1	PYGO2	SERPINB5	RUNX2	CITED1	IRF6	SULT1E1	KRT14	KDM3A	SLC30A2	PADI2	CLDN1	ABCG2	PIP	KRT5	PRLR	CLDN4
CELLMARKEREXP_MEGAKARYOCYTES		ITGA2B	GP1BA	ITGB3	MPL	LAT	PLEK	S100A9	S100A8	PF4	F13A1	CD9	EPOR	CXCR4	GP5	CXCR1	CXCR2	SLAMF1	PDE6B	MATK	SPON2	MKL2	TLR9	KIF5B	GATA1
CELLMARKEREXP_MELANOCYTES		AHR	MC1R	PMEL	PTGDS	SLC45A2	MLANA	MITF	TYR	TYRP1	DCT	FILIP1L	GPNMB	LOXL2	ZC3H7A	SLK	DKK3	AHNAK2	HSPB1	PAX3	TRPM1	CTNS	ENPP1	SOX9	BNC2
CELLMARKEREXP_MESANGIAL_CELLS		PDGFB	PDGFRB	ACTA2	TNS1	SFRP2	ECM1	ANGPT2	FGF1	CTGF	PLAU	GATA3	LEF1	MRC1	PML	OGT	NOCT	NCOA2	PER3	PLA2R1	TRIP4	AMOTL2	LATS2	YAP1	LRP5
CELLMARKEREXP_MESOTHELIAL_CELLS		CALB2	MUC16	KRT5	KRT6A	DES	CDH1	HAS2	KRT7	ME1	ME2	MSLN	THBD	WT1	LRRN4	IL17RE	LOX	KCNAB1	PKHD1L1	SULF1	DUSP5	MIR22HG	COBLL1	ST3GAL1	FBXO33
CELLMARKEREXP_MICROFOLD_CELLS		GP2	SPIB	PGLYRP1	EHF	ANXA5	SOX8	MIER3	NFIC	CXCL16	CTSD	CCL20	CTSH	MARCKSL1	RAC2	TNFAIP2	FABP5	GJB2	MFGE8	CYBA	TMPRSS2	AHR	MTF1	RELB	TULP4
CELLMARKEREXP_MICROGLIA		ITGAM	ITGAX	C5AR1	CX3CR1	P2RY12	P2RX7	CD40	TREM2	TMEM119	GPR34	TFF3	SALL1	CSF3R	TCIRG1	SUSD3	GPR183	VAV1	SLC2A5	IL21R	IL10RA	PTAFR	LAG3	ENTPD1	ADRB2
CELLMARKEREXP_MONOCYTES		RGS1	APOBEC3A	CD7	TET2	CD40	DYSF	CMKLR1	MEFV	HCK	FCGR3B	PADI4	GHSR	ITGAX	SELE	TLR4	AR	CXCR4	CD86	CCR2	TNFRSF14	ADA2	MGMT	CD14	CD33
CELLMARKEREXP_MULLER_CELLS		TLR2	IL6	CNR2	VEGFA	SLC38A5	SLC38A3	IL33	RDH10	SLITRK2	SYNPR	CROT	CRYM	COL9A1	SIX3OS1	DAPL1	AQP4	RLBP1	DKK3	VSX2	COL1A1	COL2A1	COL3A1	COL4A2	COL5A1
CELLMARKEREXP_MYOBLASTS		MYOG	LIF	NGF	IGF2	ANGPT2	ACHE	ADAM12	TPM2	ENO3	DES	CDH15	CAPN1	MYF6	MYF5	MYOD1	PAX3	PAX7	TBX3	TTN	MUSK	FLNC	FGFR4	STK40	CXCL1
CELLMARKEREXP_MYOCYTES		ACTC1	FHL2	ACTA1	TNNI1	TNNI2	TNNT1	TNNT3	TNNC1	TNNC2	ACTN2	ACTN3	ANKRD1	ANKRD2	CAPZA3	CLOCK	DES	DMD	IL4	ITGA7	LAMA2	LAMB1	LDB1	LDB3	MSTN
CELLMARKEREXP_MYOFIBROBLASTS		CDH11	DES	PALLD	ACTA2	GFAP	TNS1	CALD1	MYL9	TAGLN															
CELLMARKEREXP_NEUROBLASTS		NTNG1	TSHZ1	SP9	SP8	PROK2	ASCL1	DLL3	SALL3	DRAXIN	ERBB4	TRIM32	IGFBPL1	EOMES	PBX1	CUX2	ISL1	PHF1	NEUROG1	NKX6-1	NEUROG2	MAPK7	PROKR2	POU6F2	ITGA6
CELLMARKEREXP_NEUTROPHILS		MME	ITGAM	ITGAX	CD14	FUT4	FCGR3A	PECAM1	CD33	SELL	CEACAM8	C5AR1	CXCR1	CXCR2	JAML	TLR2	MYLK	S100A9	MPO	CD24	CEACAM1	FCGR1A	CRP	LCN2	DEFA1
CELLMARKEREXP_NK_CELLS		ITGAM	ITGAX	FCGR3A	CD69	KLRD1	IL2RB	KLRB1	CD244	KLRK1	SLAMF7	SIGLEC7	NCR1	SLAMF6	KIT	CD27	KLRC1	KLRF1	GNLY	NKG7	IL32	GZMH	FGFBP2	GZMM	CTSW
CELLMARKEREXP_NORADRENERGIC_NEURONS		TH	DDC	DBH	SLC18A2	SLC18A3	SLC6A2	SLC39A11	SLC9B2																
CELLMARKEREXP_OLIGODENDROCYTES		GALC	MOG	OLIG1	OLIG2	TF	OLIG3	SOX10	GJC2	GAMT	BCAS1	ENPP6	TMEM63A	GPR62	BACE1	CREB5	FGFR2	SLC25A38	HDAC11	TYRO3	S1PR5	ASPA	EPHB1	HEPACAM	SOX8
CELLMARKEREXP_OSTEOBLASTS		SLC6A6	VDR	LRP4	EGFL6	MMP13	GPER1	RUNX2	NF2	LRP5	PHEX	HIF1A	C5AR1	CXCL12	COL1A1	PLCD4	FOXO1	SOCS3	IL33	MAGED1	PARD6G	CD44	NLRP3	ALPL	TNFSF11
CELLMARKEREXP_OSTEOCLAST_PRECURSOR_CELLS		MITF	NFKB1	NFKB2	TNFRSF1…	TRAF6	TREM2	CYSLTR1	TLR4																
CELLMARKEREXP_OSTEOCLASTS		SOST	ZBTB7A	SEMA4D	KCNMA1	TNFRSF11B	PTPRA	TNFRSF11A	MMP9	ACP5	RAB3B	ITPR3	NRP2	THRA	AGER	RGS18	PTH1R	LTB4R	TRPV5	SNX10	CTSK	OSCAR	SLC2A1	DCSTAMP	EGFL7
CELLMARKEREXP_OSTEOCYTES		SOST	PFN1	EFNB2	PDPN	FGF23																			
CELLMARKEREXP_PANETH_CELLS		DEFA5	MMP7	AGR2	DEFA1	DEFA6	CSF2	LYZ	PLA2G2A	PRSS1	GUCA2B	MUC2	CLCA1	GUCA2A	REG3G	ITLN1	RETNLB	GPX2	LGALS2	SPINK4	FABP4	CFD	ADIPOQ	PNLIPRP2	DMBT1
CELLMARKEREXP_PARATHYROID_CHIEF_CELLS		KL	CASR	VDR	FGFR1	CYP27B1	LRP2	PTH	RAB3A																
CELLMARKEREXP_PERICYTES		PECAM1	PDGFRB	CSPG4	ANPEP	ACTA2	DES	RGS5	ABCC9	KCNJ8	CD248	DLK1	TEK	NOTCH3	GLI1	ICAM1	ADM	ANGPT1	VEGFA	ZIC1	FOXC1	POSTN	COX4I2	HIGD1B	PDZD2
CELLMARKEREXP_PHOTORECEPTOR_CELLS		RCVRN	PDC	NRL	NR2E3	SAG	RHO	ROM1	CRX	PDE6G	TPBG	GABRA1	PCBP3	FRMD3	ADAMTS5	RD3L	GABRG2	CNTN4	RNF152	SYNE1	SEBOX	HSPA12A	CNTNAP2	NLK	NTNG1
CELLMARKEREXP_PLASMA_CELLS		SDC1	IRF4	CD38	PRDM1	CD93	CD27	ENPP1	KLF4	MS4A4A	PIAS1	CYCS	DIABLO	HTRA2	MS4A1	CD9	CD19	CD24	CD37	CD40	CD74	CXCR4	GZMB	TNFRSF17	TNFRSF13B
CELLMARKEREXP_PLASMACYTOID_DENDRITIC_CELLS		CD4	CD8A	CD40	CD80	CD83	LILRA4	CD86	IL3RA	CCR7	CLEC4C	CXCR3	TRADD	ARHGAP9	LAMP5	GPR183	MAP3K2	TNFSF13B	PTCRA	ERN1	ETV6	MAP3K8	FAM129C	SPIB	GZMB
CELLMARKEREXP_PODOCYTES		PODXL	ADAMTS13	LCN2	VEGFA	SLC2A4	NLRP3	SYNPO	NPHS1	NPHS2	LAMA3	FOXC2	WT1	CD80	DES	PTPRO	LMX1B	MYOC	CDH13	SEMA3G	THSD7A	MAFB	ENPEP	CLIC5	RAB3B
CELLMARKEREXP_PRINCIPAL_CELLS		AQP2	SCNN1A	AQP3	KCNJ1	AVPR2	SCNN1G	HSD11B2	FXYD4	APELA	NPNT	SCNN1B	KCNE1	CDH16	ADGRG1	TSPAN8	CD01	TMEM45B	TACSTD2	PDZK1I…	L1CAM	CLDN4	TBCK	TMEM229A	GPRC5B
CELLMARKEREXP_PROXIMAL_TUBULE_CELLS		AQP1	CLDN2	DPP4	ENPEP	SLC36A2	SLC5A1	SLC6A18	SLC6A19	SLC5A2	LRP2	PDZK1	MIOX	SLC34A1	TTC36	ASS1	PCK1	FUT9	KHK	SLC4A4	GLYAT	FBP1	SLC22A6	SLC22A12	SLC47A1
CELLMARKEREXP_PULMONARY_ALVEOLAR_TYPE_I_C…		MMP11	ICAM1	CTGF	SEMA3B	SCNN1B	SEMA3E	SCNN1G	SCNN1A	COL4A4	CYP4B1	RTKN2	CLIC5	AKAP5	GPRC5A	SEC14L3	COL4A3	CLDN18	AGER	HOPX	AQP5	EMP2	VEGFA	KRT7	IGFBP6

Table S5: Cell state marker gene list

CELLMARKERCAN_ACINAR_CELLS		CTRB1	KLK1	RBPJL	PTF1A	CELA3A	PRSS1	SPINK1	ZG16	CEL	CELA2A	CPB1	CELA1	RNASE1	AMY2B	CPA2	CPA1	CELA3B	PNLIP	CTRB2	PLA2G1B	PRSS2	CLPS	REG1A	SYCN
CELLMARKEREXP_PULMONARY_ALVEOLAR_TYPE_II_…		IRX1	EGFL6	CEBPA	NKX2-1	MUC1	CLDN18	AGER	LAMP3	SFTA2	SFTPD	SLC34A2	SFTPB	SFTPA1	SFTPC	SDC1	ADGRF5	ABCD3	SOAT1	ETV5	CD36	LPCAT1	NAPSA	ABCA3	S100G
CELLMARKEREXP_PURKINJE_FIBER_CELLS		GJA5	B3GAT1	GJC1	KCNK3	NEFM																			
CELLMARKEREXP_PURKINJE_NEURONS		CALB1	ABAT	GRIN1	GAD1	HDAC6	PRKG1	ATP2B2	PDE5A	ITPR3	PDE1B	PDE1A	OMP	PDE9A	CBLN1	MRPS35	PVALB	SHANK2	CACNA2D2	NKAIN4	CNTNAP2	TH	CERK	CD3G	KCNA2
CELLMARKEREXP_PYRAMIDAL_CELLS		CD3E	DLG4	CAMK2A	GRM1	GRM5	TBR1	POU3F1	EMX1	FEZF2	NRGN	GRIA3	SPINK8	PCP4	SYNPR	NR4A2	AGER	GRIN2D	YWHAZ	KCNN4	DAB1	CCM2	MAP2	CALB1	ADRB2
CELLMARKEREXP_RETICULOCYTES		RUVBL1	TFRC	UCP3	FOLR1	UBE2O	CD36	ITGA4	SLC11A2																
CELLMARKEREXP_RETINAL_GANGLION_CELLS		PRKCA	NGF	POU4F2	NHLH2	RNU1-1	ATF6B	SNX16	IRX5	YDJC	DPP10	SHD	SMIM18	GALNT14	CHRNB3	THEM4	SCAPER	GALNTL6	CCDC184	NKAIN3	LHFPL5	SRRM3	GALNT9	MTUS2	IGLON5
CELLMARKEREXP_SALIVARY_MUCOUS_CELLS		KRT14	PIP	MUCL1	CNN1	THBS1	MYH11	KRT17																	
CELLMARKEREXP_SATELLITE_CELLS		PAX7	DMD	CDH1	ERBB2	PAX3	MYF5	CDH15	CXCR4	FOXK1	ITGA7	TCF21	ISL1	MESP1	MET	CALCR	ASB5	SRXN1	BDKRB1	CDCP1	VCAM1	MYOD1	SOX8	SPRY1	CXCL1
CELLMARKEREXP_SCHWANN_CELLS		MPZ	ITGB4	LAMC1	S100B	POU3F1	GFAP	MAG	OLIG1	TST	NDRG1	CTHRC1	NF2	46274	AGR2	NGFR	CLDN19	PRX	NTRK1	ART3	BCAS1	NPY	ASPA	LGI4	BAG3
CELLMARKEREXP_SEROTONERGIC_NEURONS		TPH1	SLC6A4	FEV	TPH2	DDC	SLC18A2	ESM1	SLC22A3																
CELLMARKEREXP_SERTOLI_CELLS		KITLG	AMH	INHBB	SHBG	GATA1	GATA4	DHH	AR	GDNF	MAFB	GAMT	MEIG1	HES5	NF2	INHA	DES	FAS	FASLG	TF	WT1	AARD	CST9	TMEM184A	WNT6
CELLMARKEREXP_SMOOTH_MUSCLE_CELLS		ACTA2	CNN1	HEXIM1	TAGLN	RGS5	WFDC1	NOTCH3	RPRM	DES	FHL2	FBLN5	LOX	NF2	ANGPT1	CRABP1	MYH11	FABP4	ACTC1	ANKRD1	OBSCN	PDE4DIP	LMO7	MYH6	NEXN
CELLMARKEREXP_SPERMATOZOA		ABHD2	HVCN1	GPR55	ABCA1	SLC31A1	LDHC	PRKAA2	HSPA2	ODF1															
CELLMARKEREXP_STROMAL_CELLS		MMP1	MMP2	MMP9	TLR3	MME	PECAM1	ITGA4	ICAM3	ITGAV	ICAM1	VCAM1	KIT	PDGFRA	PDGFRB	MADCAM1	B4GAL…	TLR1	TLR2	TLR4	CDH11	CALB2	DES	MMRN2	CD248
CELLMARKEREXP_T_CELLS		CD3D	CD3G	CD3E	HOPX	CCL3	CCL4	GIMAP2	SYT3	NOTCH3	SEMA6D	DKK3	IFIT3	CERK	PMCH	CD4	LTB	CXCR6	IL7R	SATB1	LEF1	ITK	TRBC2	PTPRCAP	GEM
CELLMARKEREXP_T_CELLS_NAIVE		CD3D	CD4	SELL	CCR7																				
CELLMARKEREXP_T_CYTOTOXIC_CELLS		CD2	CD27	CD28	CD5	CD69	CD8A	GZMB	TRAC																
CELLMARKEREXP_T_HELPER_CELLS		IFNG	IL2	TBX21	DPP4	KLRD1	CXCR3	CCR1	CCR5	TNFSF11	HAVCR2	IL18R1	LTBR	TNF	LTA	GATA3	CSF2	IL4	IL5	IL6	IL10	IL13	CCR8	CXCR4	CCR3
CELLMARKEREXP_T_MEMORY_CELLS		SELL	CCR7	CD4	CCR5	CD69	ITGB5	BCL2	GRAP2	TBC1D1…	CYTIP	KPNA4	CD247	EPSTI1	APBB1IP	CD27	RHOH	GIMAP1	ACP5	IKZF1	SP100	DGKA	IL7R	ITGB7	NKG7
CELLMARKEREXP_TRICHOCYTES		KRT86	DSG2	KRT81	DSG4	KRT37	HOXC13																		
CELLMARKEREXP_TRIGEMINAL_NEURONS		CREBRF	PRKX	PRDM12	DHCR24	CALCA	CALCB	VWA5A	PPP1R1C	AVIL	PIRT	TAC1	PRPH	SCN9A	RUFY2	FAM189B	CLGN	SCN7A	NEFH	GCNT2	ZDHHC2	PARM1	AHNAK2	PHF24	KCNAB2
CELLMARKEREXP_TROPHOBLAST_CELLS		CD4	TTPA	TNFRSF9	STAR	HOPX	S100P	ADAM19	CYP24A1	LIF	HAND1	ELF5	SIRT1	PTHLH	ACKR2	IGF2	SIGLE…	CDX2	KRT7	DLX3	GCM1	ASCL2	CGB3	INHBA	MLANA
CELLMARKEREXP_TROPHOBLAST_PROGENITOR_CELLS		ISG20	FOXO4	TFAP2C																					
CELLMARKEREXP_TUFT_CELLS		SUCNR1	FABP1	POU2F3	CDHR2	AVIL	ESPN	LRMP	TRPM5	DCLK1	TAS1R3	SOX9	CAMK2B	GNAT3	IL25	PLCB2	GFI1B	ATOH1	ASIC5	KLF3	KLF6	DRD3	GNG13	NREP	RGS2
CELLMARKEREXP_VASCULAR_SMOOTH_MUSCLE_CELLS		ACTA2	MYH11	PDGFRB	SEMA3D	TBX18	WT1																		
CELLMARKEREXPENDOTHELIALCELLSAORTA		ITGA5	TMTC2	NR1D1	HFE	PKN3	SULT1A1	TMTC1	SHE	ITGBL1	GRASP	ENPP4	ARHGEF15	TMEM184B	FOXC2	DHH	DENND…	KAZALD1	FGD5	FAS	ITGB4	EMILIN1	MICAL2	NKX2-3	STXBP6
CELLMARKEREXPNEURALSTEMPRECURSORCELLS		SOX1	PROM1	IGF1R	SP8	GFAP	RBM3	SOX3	S100B	SLC1A3	NEUROD4	NEUROG1	NEUROG2	NHLH1	PPP1R17	TMTC4	EOMES	PLEKHH2	TLR3	PARP12	P2RX7	INAFM2	ISG15	IGFBP3	IFIT1
CELLMARKERMSIGDB_AIZARANI_LIVER_C12_NK_NK…		HSPA1A	HSPA1B	DNAJB1	HSPA6	HSP90AA1	HSPH1	IL2RB	NKG7	CD7	IFNG	HSPA8	KLRK1	KLRD1	GNLY	DUSP2	CMC1	HSPD1	BAG3	CST7	CACYBP	CCL5	ITGAL	STK17A	DNAJA1
CELLMARKERMSIGDB_AIZARANI_LIVER_C22_RESID…		MZB1	DERL3	SSR4	BLOC1S5…	ITM2C	HERPUD1	FKBP11	SEC11C	XBP1	FOSB	FCRL5	POU2AF1	RAB30	ERLEC1	LY9	CREB3…	MEI1	TENT5C	HSP90B1	CD79A	SLAMF7	H1-10	SDF2L1	PRDX4
CELLMARKERMSIGDB_AIZARANI_LIVER_C28_NK_NK…		SRGN	TNFAIP3	ZFP36L2	CRYBG1	CREM	ZNF331	CXCR4	RGS1	IL7R	XCL1	BTG1	CNOT6L	CD44	SPOCK2	CD69	AREG	EMB	GPR171	SARAF	NR3C1	STK4	NIBAN1	ATP1B3	TSC22D3
CELLMARKERMSIGDB_AIZARANI_LIVER_C29_MVECS…		MGP	EMP1	FAM107A	TM4SF1	VWF	CLEC14A	ADAMTS1	SPARCL1	CAV1	CPE	LMNA	C7	VIM	C11orf96	ITGA5	IGFBP7	CD9	MMRN2	FOXC1	EPAS1	RNASE1	CCN2	CAVIN2	PRSS23
CELLMARKERMSIGDB_AIZARANI_LIVER_C30_HEPAT…		TNFSF14	CRP	CYP4F3	CYP2B6	RN7SL403P	WDR31	CYP3A5	NSUN6	MLXIPL	ACSM2A	ECHDC2	CP	SLC13A5	CYP2A13	MST1	F5	ASGR1	PLIN5	CYP2C19	IL6R	TENT5A	HGD	ZKSCAN1	LINC01554
CELLMARKERMSIGDB_AIZARANI_LIVER_C8_RESIDE…		JCHAIN	MZB1	DERL3	BLOC1S5…	SSR4	ITM2C	XBP1	HERPUD1	FKBP11	FCRL5	SEC11C	POU2AF1	RAB30	HSP90B1	CD79A	CD27	MEI1	PRDX4	SPAG4	CREB3L2	TENT5C	SLAMF7	SEL1L3	FKBP2
CELLMARKERMSIGDB_AIZARANI_LIVER_C9_LSECS_1		ADM	CTSL	AKAP12	OIT3	CLEC4M	FCN3	FLT1	DNASE1L3	STAB1	SOCS3	TIMP1	FCN2	FGFR1	CLEC4G	LYVE1	ADAMT…	STAB2	NID1	C11orf…	CAVIN2	TCF4	CD14	NR2F1	ITPRIP
CELLMARKERMSIGDB_BUSSLINGER_DUODENAL_BCHE…		BEST4	GUCA2B	GUCA2A	CA7	KRT72	CD24	CFTR	SPIB	MT1G	MT2A	CPA2	MT1H	HEPACAM2	CTSE	GSN	KRT20	MT1E	ADGRG4	RBPJ	MT1X	LYZ	MT1F	NAPSB	PROX1
CELLMARKERMSIGDB_BUSSLINGER_DUODENAL_DIFF…		REG1A	H4C3	TUBA1B	DMBT1	CCL25	MT1G	HSPD1	HMGB2	H2AZ1	STMN1	TUBB	PTMA	PPP1R1B	SLC12A2	MKI67	MCM7	MT2A	HMGA1	PPIA	RPL10A	RPS8	RPS2	HSPE1	RPLP0
CELLMARKERMSIGDB_BUSSLINGER_DUODENAL_EC_C…		CHGA	REG4	LCN15	FEV	CHGB	PCSK1N	ADGRG4	SCG2	GC	TIMP1	NEUROD1	KCTD12	PCSK2	TFF3	SCT	PCSK1	DDC	TTR	CRYBA2	RGS2	MAP1B	RAB3C	CDHR3	IGFBP3
CELLMARKERMSIGDB_BUSSLINGER_DUODENAL_IMMU…		CCL5	TMSB4X	GZMA	LCP1	CD2	PTPRC	VIM	ARHGDIB	RAC2	CD7	PTPRCAP	CD8A	TARP	CD3E	LAPTM5	SRGN	CD3D	RGS1	IFITM1	KLRB1	CD52	TNFAIP3	SH3BGRL3	CD53
CELLMARKERMSIGDB_BUSSLINGER_DUODENAL_STEM…		OLFM4	REG3A	SLC12A2	RGMB	CDK6	SOX4	LYZ	REG1A	SMOC2	SNHG5	VDR	CDCA7	CEL	XIST	ITGA6	HSP90…	ELAPOR1	ASCL2	SPINK1	TSPAN8	PKDCC	MARCKS…	SOX9	GPX2
CELLMARKERMSIGDB_BUSSLINGER_DUODENAL_TUFT…		SH2D6	TRPM5	IRAG2	FYB1	HCK	AVIL	PTGS1	IL17RB	B4GALN…	BMX	MATK	ALOX5	HPGDS	PTPN18	AZGP1	PLCG2	MELTF	GNG13	HTR3E	AOC1	GABRP	ATP2A3	ALOX12B	ZFHX3
CELLMARKERMSIGDB_BUSSLINGER_ESOPHAGEAL_EA…		MT2A	STMN1	CDK1	KRT5	H2AZ1	RRM2	HMGB2	TUBA1B	ASPM	PCNA	HMGB1	TOP2A	H4C3	PTMA	CCNB1	PRC1	H2AZ2	RRM1	TUBB	DUT	DEK	MKI67	CBX3	PTTG1
CELLMARKERMSIGDB_BUSSLINGER_ESOPHAGEAL_PR…		KRT14	DST	ASS1	TOP2A	COL17A1	KRT15	SLC7A5	MKI67	GPNMB	CENPF	CAV1	STMN1	H4C3	ASPM	TUBB	TUBA1B	ITGA6	NUSAP1	HMGB2	RRM2	IL1R2	SLC7A1	LAMB3	MT2A
CELLMARKERMSIGDB_BUSSLINGER_GASTRIC_CHIEF…		PGA5	LIPF	CHIA	PGC	ITIH5	UGT2B15	AZGP1	MT1G	GPER1	SOX4	XYLT2	NR2F2	IL33	ITLN1	IRX2	PDIA2	MT1X	MT2A	MT1F	TMED6	FMOD	FNDC3B	CDKN1C	PLIN5
CELLMARKERMSIGDB_BUSSLINGER_GASTRIC_G_CEL…		GAST	TFF3	PCSK1N	PAX6	ERO1B	CHGB	CPE	SCG5	CES1	PCSK2	SHISAL2B	PDK3	PAM	CACNA1A	SCGB2A1	INSM1	ARX	HAP1	MAP1B	MS4A8	CLTRN	DDC	KCTD12	ACVR1C
CELLMARKERMSIGDB_BUSSLINGER_GASTRIC_ISTHM…		MUCL3	S100A14	KRT8	CRIP1	KRT19	TFF1	NMU	NKX6-2	FER1L6	AGR2	SFN	MKI67	S100A6	HSPB1	CENPF	SYTL2	CAPN8	TOP2A	TAGLN2	IL1R2	SDF2L1	PKP3	CCND1	AKR1B10
CELLMARKERMSIGDB_BUSSLINGER_GASTRIC_NECK_…		MUC6	TFF2	GP2	AQP5	FMOD	GOLM1	ETV5	LYZ	FUT9	PPP1R1B	PGC	MMP1	SLC4A4	CLU	A4GNT	COL11…	NR4A1	C6orf58	DYNC2H1	C16orf89	MSMB	PDIA2	KCNN4	SLC12A2
CELLMARKERMSIGDB_BUSSLINGER_GASTRIC_OXYNT…		HDC	CHGA	CGA	MAOB	SLC18A2	PTF1A	SCG2	GPX3	GFRA3	SCG3	RBPJL	PAM	KCTD12	KLK11	UCP2	MS4A8	CHGB	CPE	CDHR3	PAPPA2	PCSK1	ERP27	VGF	PRSS1
CELLMARKERMSIGDB_BUSSLINGER_GASTRIC_PREZY…		MUC6	LIPF	NR2F2	LTF	FAM3D	PDIA2	SOX4	FUT9	PIGR	RGMB	XYLT2	SLC12A2	PIK3C2G	HMGCS2	THSD4	PTPRZ1	ANKRD50	ENAH	FOSB	SPDEF	PALM3	RHOBTB1	NFIB	EGR1
CELLMARKERMSIGDB_BUSSLINGER_GASTRIC_REG3A…		REG3A	HLA-DRA	CD74	HLA-DPA1	HLA-DRB1	HLA-DQ…	LTF	LCN2	UBD	RGS5	HLA-DPB1	HLA-DQB1	BIRC3	PIGR	DUOX2	REG1A	TYROBP	CXCL11	IFI30	HLA-DMA	CXCL9	VNN2	CXCL10	IFITM3
CELLMARKERMSIGDB_CUI_DEVELOPING_HEART_5TH…		MYH7	MYL2	MASP1	VCAN	S100A4	LBH	HAPLN1	TRDN-AS1	LGALS1	HAND1	HIGD1A	IRX3	HNRNPL	LAMA4	CRNDE	SCUBE3	TM4SF1	CXCL12	PDGFD	FABP5	TMSB4X	RAMP2	CDIN1	FRMD3
CELLMARKERMSIGDB_CUI_DEVELOPING_HEART_5TH…		MYH6	PAM	CALB2	NR2F1	VSNL1	PGAM2	COL2A1	GPX3	GJA5	LINC018…	TESC	NR2F1-AS1	RELN	PDLIM3	TECRL	DKK3	SH3KBP1	RABGAP1L	ATP2A2	FOS	PIFO	CLIC5	PDLIM4	ABRA
CELLMARKERMSIGDB_CUI_DEVELOPING_HEART_C1_…		KRT19	KRT8	TNNT1	PGAM4	KRT18	DMKN	MSMO1	ANXA3	SPINT2	SLC2A1	EZR	PARD6B	PRDX2	SQLE	MLLT11	MGST1	CDH3	TPM2	NAXE	ISYNA1	HACD1	HEY1	NPPB	CSRP2
CELLMARKERMSIGDB_CUI_DEVELOPING_HEART_C2_…		MYL7	TNNT2	TNNC1	ACTC1	MYL4	MYL9	TNNI1	CSRP3	PLN	TNNI3	CRYAB	PGAM2	HSPB7	SLC25A4	FABP3	MYOZ2	CKM	SMPX	LDB3	ACTN2	NEBL	FGF12	NKX2-5	DES
CELLMARKERMSIGDB_CUI_DEVELOPING_HEART_COM…		COL3A1	COL1A1	DCN	COL1A2	POSTN	TNNI3	DES	FN1	SPARCL1	MGP	OGN	COL6A3	LUM	PLAC9	CXCL14	LGALS1	DLK1	VIM	HSPB1	FBN1	ELN	COL5A1	MMP2	CRYAB
CELLMARKERMSIGDB_CUI_DEVELOPING_HEART_COR…		A2M	FABP4	CD36	COL15A1	IGFBP7	FABP5	TNNT3	CLDN5	TCF4	TM4SF18	PDGFB	TCIM	CAV1	APLNR	COL4A1	RGCC	DIPK2B	VWF	JAM2	UACA	COL4A2	UTRN	HLA-B	DST
CELLMARKERMSIGDB_CUI_DEVELOPING_HEART_END…		CDH11	NPR3	CTHRC1	COLEC11	MEST	ITGA8	KRT18	ADGRG6	PCDH7	TFPI	MYL7	CGNL1	ENG	POSTN	NPPA	MASP1	OLFML3	OST4	C4orf48	ISYNA1	FXYD6	LRIG3	ITM2C	LEPR
CELLMARKERMSIGDB_CUI_DEVELOPING_HEART_LEF…		TM4SF1	MYH7	LGALS1	ESAM	PLVAP	COL3A1	COLEC11	CCL2	IRX3	SLIT2	ENG	CTHRC1	TRDN-AS1	THY1	HES1	TFPI	HAPLN1	RAMP2	ECSCR	HAND1	CXCL14	CRNDE	PLXND1	TMEM88
CELLMARKERMSIGDB_CUI_DEVELOPING_HEART_RIG…		KLK6	ACTA1	ABRA	PAM	UCP2	NR4A1	CALB2	CAV1	ULK4	HAMP	HCN4	PNP	NMRK2	SLC30A3	CDKN1A	DUSP2	TAF10	CITED4	PEDS1	LRAT	HHIP-AS1	GNAL	MYPN	EFNB1
CELLMARKERMSIGDB_CUI_DEVELOPING_HEART_SMO…		SFRP2	COL3A1	COL1A2	S100A6	C1R	COL1A1	PCOLCE	ITM2A	CPZ	SERPINF1	PI16	C1S	CRABP2	SPARC	FBLN1	OSR1	ELN	PDGFRL	CCDC80	PI15	IGFBP4	COL14A1	ANGPTL1	RCN3
CELLMARKERMSIGDB_DESCARTES_FETAL_ADRENAL_…		ENSG00…	MYCBPAP	LINC00…	SLC26A4	TEX41	LINC02…	GPRC6A	USP51	LINC02…	LY6E-DT	UICLM	IL1A	MTERF2	MAPK10-…	KDM4A-A…	VMO1	BTBD3-A…	NPHP4	RPL38P1	ZC3H12A	LINC024…	LINC03…	LINC02718	CASC8
CELLMARKERMSIGDB_DESCARTES_FETAL_CEREBELL…		GALNT15	NTF3	SLC26A7	CDH7	FGF5	TRHDE	MIR217HG	GRIK2	ST18	GRIN2A	PCSK9	HS3ST3A1	MIR548X…	KCNA1	BMP5	ENSG0…	LINC016…	LINC028…	TRHDE-…	LINC02476	GRIN2C	TLX3	LINC00992	DIO3
CELLMARKERMSIGDB_DESCARTES_FETAL_CEREBELL…		SLC6A5	PAX2	TFAP2B	NXPH2	LRRC38	CPLX4	CACNG3	KIT	SLC35F4	SPOCK1	IGFBP5	CCBE1	VCAN-AS1	KRT222	ROBO3	ENSG0…	SLC30A3	INHBA	LBX1-A…	NEUROG2	FNDC1	PRR35	ENSG0000…	LINC02408
CELLMARKERMSIGDB_DESCARTES_FETAL_CEREBELL…		CLEC7A	OLR1	LINC02…	FCGR1A	P2RY12	FGD2	SPP1	FCGR2A	DOCK8	VSIG4	SAMSN1	TREM2	CX3CR1	C3	ITGAX	CYTH4	TLR7	PTPRC	CD86	CD14	SIGLEC10	FCGR3A	FOLR2	IKZF1
CELLMARKERMSIGDB_DESCARTES_FETAL_CEREBELL…		SKOR2	COL6A6	POSTN	PDE11A	IL1RAPL2	ITPR1	ESRRB	ANO1	COL24A1	NPSR1	CA8	PRMT8	GPC3	DAB1	NDNF	FOXP2	SLC1A6	TAF4B	RORA	CDS1	HS3ST2	CALB1	PTGER3	HSPA12A
CELLMARKERMSIGDB_DESCARTES_FETAL_CEREBELL…		LINC01…	C8orf34	C8orf3…	SLC24A4	GRIN3A	ZFHX4-…	L3MBTL4	GPR149	PEX5L	ZIM2-AS1	SAMD5	DCHS2	PTPRK	KCNQ5	VSTM2A-…	LGI2	SCN7A	OTOF	HTR1E	DMBX1	TRPC7	HNF4G	PCP4L1	ZPLD1
CELLMARKERMSIGDB_DESCARTES_FETAL_CEREBELL…		LMX1A	EOMES	RPH3A	RSPO2	DYNC1I1	MID1	CHST9	CDH9	FBXL21P	SLC27A6	TMEM237	CHRM5	COL13A1	OTX2	TPBG	QRFPR	OTX2-AS1	LINC029…	RPGRIP1	HTR5A	LINC020…	RPS3P6	CNGB1	LINC01885
CELLMARKERMSIGDB_DESCARTES_FETAL_CEREBELL…		FOXC1	ROBO4	BGN	MYCT1	ESAM	GJA4	ECSCR	DIPK2B	SLC38A5	SEMA3G	GGT5	TIE1	TM4SF18	SOX18	SRARP	APLNR	ZNF366	CAVIN1	ITGA1	GIMAP7	VWF	CD34	ADGRF5	HSPA12B
CELLMARKERMSIGDB_DESCARTES_FETAL_CEREBRUM…		RELN	PTCHD4	RARB	SYNPR	RXRG	ARX	LINC01210	TFAP2B	PDYN	TLE1-DT	ISL1	DLX1	LINC013…	TFAP2D	SYTL5	DLX6	PAX2	CCK	KCNA1	AVP	BMP3	DMBX1	HTR4	CRABP1
CELLMARKERMSIGDB_DESCARTES_FETAL_CEREBRUM…		HPSE2	NTS	PTPN3	CRTAC1	PROX1-AS1	RNF220	CPNE4	SYNPO2	SAMD5	SLC17A6…	OPRK1	HACD1	BDNF	NTF3	CHRNA2	HSD11…	FSHR	CRH	GLB1L3	DMKN	SH2D5	ENSG00…	OVCH2	PYDC2-AS1
CELLMARKERMSIGDB_DESCARTES_FETAL_CEREBRUM…		PPBP	PF4	ITGB3	THBS1	LTBP1	MMRN1	PLEK	RAB27B	GPX1	STON2	PRKAR2B	TLN1	RAP1B	IQGAP2	CAVIN2	PDGFC	LINC002…	STOM	CMTM6	CD226	ANO6	ARHGAP6	IRAG1	FXYD5
CELLMARKERMSIGDB_DESCARTES_FETAL_CEREBRUM…		SIGLEC…	TLR7	C1QC	P2RY13	PLD4	FGD2	CSF2RA	FCGR3A	ITGAX	CSF3R	C1QA	TYROBP	HPGDS	CD86	FOLR2	BLNK	CYBB	MS4A7	SAMSN1	IRF8	ITGB2	SMIM35	TFEC	SPI1
CELLMARKERMSIGDB_DESCARTES_FETAL_CEREBRUM…		BCAS1	GPR17	SOX10	PDGFRA	COL20A1	APOD	ENPP6	UGT8	FA2H	ERBB3	AGMO	LINC01170	ENSG000…	CLDN11	PCDH15	PLPP4	PLAAT1	LHFPL3	OLIG2	TNR	CHRM3	ENSG00…	ZNF365	NBEAP2
CELLMARKERMSIGDB_DESCARTES_FETAL_CEREBRUM…		SKOR2	NPSR1	PDE11A	COL6A6	POSTN	TFAP2A	ESRRB	IL1RAPL2	CALB1	CA8	EBF3	NDNF	PTGER3	TAF4B	KCTD8	ZNF38…	ADGRL2	FOXP4	PRMT8	RORA	ITPR1	GPC3	PCP4	HSPA12A
CELLMARKERMSIGDB_DESCARTES_FETAL_CEREBRUM…		FOXQ1	BGN	ADGRF5	C2CD4B	TM4SF18	FOXC1	FOXF2	GGT5	DIPK2B	ECSCR	CD34	TIE1	HSPA12B	ACVRL1	SRARP	STRA6	CD248	SLC38A11	ZNF366	FOXS1	TBX2	PCAT19	ADCY4	CFH
CELLMARKERMSIGDB_DESCARTES_FETAL_EYE_ASTR…		WIF1	KCNMB2	TNC	DCLK1	CST3	CLYBL	LMO3	MIR4300…	ZIC1	RBM20	ADAMTS3	LINC02231	PAX2	PAX5	RIMS4	PAX8	MLC1	GINS3	HMCN2	CDC20B	NFIA-AS2	AQP4	ZIC4	CLEC18B
CELLMARKERMSIGDB_DESCARTES_FETAL_EYE_GANG…		CHRNB3	OBI1-AS1	POU4F1	GPR149	TMEM163	NRN1	INA	SLC17A6	ISLR2	KIF5A	GAP43	SYT5	PRPH	CHRNA6	LINC016…	CLEC2L	EOMES	TFAP2D	P2RX3	SYNGR3	POU4F2	OPN4	CEND1	LINC00348
CELLMARKERMSIGDB_DESCARTES_FETAL_EYE_LENS…		LGSN	ENSG0000…	CRYBA4	CRYBA1	CRYBB1	SLC24A2	CRYAA	CRYGC	LINC03…	LSAMP	RMST	MED12L	BFSP1	CRYBB2	PALM2AK…	CRYGD	CRYGA	CRYBA2	CRYBB3	MIP	BFSP2	CRYGS	VIT	LINC02024
CELLMARKERMSIGDB_DESCARTES_FETAL_EYE_PHOT…		MPP4	EGFLAM	IMPG2	RPGRIP1	GPC5	EYS	TMEM244	MAK	CDHR1	CHRNB4	CERKL	USH2A	IMPG1	PDC	LINC029…	ST3GA…	ATP6V0D2	PRICKLE…	PCAT4	GPC5-IT1	CNGB1	LINC00…	KCNV2	KDM4A-AS1
CELLMARKERMSIGDB_DESCARTES_FETAL_EYE_RETI…		SLC38A…	COL8A1	PLD5	LINC002…	BEST1	ENPP2	ITGAV	CRISPLD1	VAT1L	FYB2	RPE65	BMP6	VEPH1	CCBE1	PRDM16	LINC0…	GEM	ACOT11	FRZB	DCDC2	CNBD1	NPFFR1	MYRF	PTGDS
CELLMARKERMSIGDB_DESCARTES_FETAL_EYE_SMOO…		LINC03…	TYRP1	CABLES1	CPEB2	STK32A	ARHGAP6	MYO5A	TEX41	KIT	ARID5B	PAX3	SLC35D2	CDH19	MPZ	CRYL1	LINC0…	COL24A1	PPP2R5A	RAB38	MLANA	P2RX7	MME	AP1S3	PAMR1
CELLMARKERMSIGDB_DESCARTES_FETAL_EYE_STRO…		POSTN	COL1A2	MFAP5	COL12A1	PI16	COL1A1	COL14A1	COL3A1	PCOLCE	DCN	LUM	COL6A6	COL5A1	OGN	SPARC	COL5A2	FN1	COL6A2	MXRA5	FBN1	SFRP2	LOX	MGP	COL6A1
CELLMARKERMSIGDB_DESCARTES_FETAL_EYE_VASC…		FLT1	ARL15	LINC02…	ARHGAP29	PLVAP	CALCRL	ENG	EPAS1	PRKCH	PLXND1	EGFL7	ELK3	APLNR	ADGRF5	SHANK3	ST8SI…	RAMP2	TM4SF18	PODXL	TIE1	ERG	RGCC	CDH5	UPB1
CELLMARKERMSIGDB_DESCARTES_FETAL_HEART_EL…		SCGB3A2	SFTPB	AGR2	ZMYND10	ELF3	SFTPC	PIFO	IL20RA	TMC5	CP	AGBL2	LINC02455	CFAP57	FOXJ1	CLXN	LINC0…	CFAP276	LINC018…	SCGB1A1	PIH1D2	DNAH9	CRLF1	DNAH3	FAM216B
CELLMARKERMSIGDB_DESCARTES_FETAL_HEART_EN…		APCDD1L	NPPC	SLC6A4	LEPR	HAPLN1	APCDD1	FOXC1	SLCO2A1	CPXM2	TMEM132C	TMEM100	INHBA	CDH11	SMOC1	HS3ST1	HMCN1	CGNL1	QSOX1	ITGA8	SALL1	PWWP3B	SALL3	CA12	MPZL2
CELLMARKERMSIGDB_DESCARTES_FETAL_HEART_LY…		STAB2	CCL21	NTS	PROX1	MPP7	FLT4	TFF3	LINC021…	PARD6G	TBX1	TSPEAR	PROX1-AS1	PTX3	PIK3C2G	SLC38A4	CNTNA…	TNFAIP8…	SPHK1	LINC01…	EFCC1	ANKRD36…	GPR146	CMKLR2	HOXD8
CELLMARKERMSIGDB_DESCARTES_FETAL_HEART_SC…		MPZ	PLP1	CDH19	XKR4	LINC01505	TENM3	OLFML2A	ITIH5	S100B	ZNF536	ERBB3	TSPAN11	GFRA3	ALDH1A1	SOX10	COL2A1	PLEKHA4	FOXD3	LGI4	MEGF10	SHC4	INSC	PRIMA1	COL28A1
CELLMARKERMSIGDB_DESCARTES_FETAL_HEART_SM…		THBS4	PDGFRB	ENPEP	ACTA2	KCNJ8	NOTCH3	KCNAB1	NDUFA4L2	MYH11	TAGLN	ABCC9	PLA2G5	SULT1E1	CYGB	AOC3	SEMA5B	AGT	CARMN	C11orf…	COX4I2	OR51E1	MYO3A	MDFIC2	ARHGEF17
CELLMARKERMSIGDB_DESCARTES_FETAL_HEART_ST…		DPT	MFAP5	LUM	ABCA10	NRK	PDGFRA	ADAMTS19	COL1A1	FBLN1	ISLR	TCF21	GPC3	DCN	SERPINF1	COL1A2	PCOLCE	HTRA3	ABCA9	ADAMTS2	PRELP	PTN	SCARA5	CCN3	TARID
CELLMARKERMSIGDB_DESCARTES_FETAL_HEART_VI…		CNTN5	LINC03000	PRPH	SST	ASTN2	ELAVL4	EML5	CHRNA3	KCTD16	FST	PHOX2B	RAB3C	CHGB	ELAVL2	RGS4	NXPH1	PTPRR	PRUNE2	STAC	SPOCK3	RAB3B	C14orf…	SCG2	GNG4
CELLMARKERMSIGDB_DESCARTES_FETAL_INTESTIN…		CDH19	SOX2-OT	PTPRZ1	NKAIN3	SLC35F1	ERBB4	SNTB1	COL25A1	GRIK3	PLP1	COL20A1	PRIMA1	LGI4	METRN	MPZ	EGFLAM	ENSG000…	SOX10	GINS3	SOX2	TFAP2A	MEGF10	S100B	BCAN
CELLMARKERMSIGDB_DESCARTES_FETAL_INTESTIN…		TFPI	RALGAPA2	CCL21	PROX1	MPP7	PLSCR4	STAB2	MMRN1	FLT4	PARD6G	LINC021…	PLA2G4C	TSPEAR	IL7	CRACR2B	PLIN5	LETR1	TNFAIP8…	MIR200…	CMKLR2	TBX1	IRAG1-…	ELMOD1	TP63
CELLMARKERMSIGDB_DESCARTES_FETAL_INTESTIN…		PTPRD	SEMA3D	MSLN	RBFOX1	ALDH1A2	CNTN6	PTGIS	ITGA3	COL11A1	KCTD8	MYRF	LRP2	CFH	C3	GFPT2	WT1	CAVIN2-…	WNT2B	PTPRQ	HAS1	BNC1	TNNT1	CSDC2	GRIN2A
CELLMARKERMSIGDB_DESCARTES_FETAL_INTESTIN…		ACTG2	GREM2	DES	MYH11	TAGLN	ACTA2	MYLK	MYOCD	CHRM2	MYL9	KCNMA1	FLNA	PDLIM7	CARMN	CNN1	NTM	RGS5	DSTN	ANO1	FRMD6-AS2	LMOD1	KCND3	WIF1	PSD
CELLMARKERMSIGDB_DESCARTES_FETAL_INTESTIN…		CXCL14	PDGFRA	LINC03…	BMP5	FNDC1	FBLN1	PRR16	SHISA3	MUSK	SPON2	GABRG3	DPT	ADAMTS14	LUM	LRP4	TMEM1…	ADRA1A	TCF21	PDGFRB	SYNDIG1	ITGA11	NPY	LINC01082	MSC-AS1
CELLMARKERMSIGDB_DESCARTES_FETAL_INTESTIN…		FLT1	APLNR	MADCAM1	CD34	PLVAP	COL15A1	HSPG2	EMCN	CLEC3B	SOX7	CYYR1	NOTCH4	SLCO2A1	ESAM	BTNL9	CD320	GASK1B	ARHGEF15	PCDH12	PRND	TM4SF18	CLEC14A	MMRN2	MEOX1
CELLMARKERMSIGDB_DESCARTES_FETAL_KIDNEY_M…		ALX1	NKAIN2	TNC	APCDD1	RERG	LAMC3	ATRNL1	TCF21	PALLD	GRID2	CCBE1	TMEFF2	CCN4	LAMA2	AGTR2	KLHL13	ENPP1	LRRIQ1	METTL24	SYNPO2	COL25A1	AGTR1	NKD1	CPA6
CELLMARKERMSIGDB_DESCARTES_FETAL_KIDNEY_U…		PSCA	UPK1A	ELF5	AQP2	EHF	DHRS2	FXYD3	S100P	KRT7	SNX31	SCNN1G	MUC6	ADIRF	ADH1C	HMGCS2	TACST…	PIK3C2G	UPK2	GGT6	SCNN1B	SCIN	ELF3	TMEM45B	KRT19
CELLMARKERMSIGDB_DESCARTES_FETAL_LIVER_ER…		SLC25A…	C17orf99	RHCE	EIF5AP2	SLC5A4-A…	ANK1	KEL	SLC14A1	OSBP2	SPTA1	TMOD1	SEC14L4	TFRC	SLC4A1	SPTB	TRIM10	GYPA	SLC25A37	ADD2	SLC6A9	ABCB10	PIGQ	GYPB	CPOX
CELLMARKERMSIGDB_DESCARTES_FETAL_LIVER_HE…		CALN1	RNF220	NKAIN2	MPO	CHRM3	C1QTNF4	PRTN3	AZU1	KIT	SPINK2	PRSS57	LINC01122	PRDM16	HMGA2-A…	NOG	LINC0…	LINC029…	LINC021…	TRH	ANKRD20A…	TNFRSF1…	MS4A3	TFAP2E-A…	CHRDL1
CELLMARKERMSIGDB_DESCARTES_FETAL_LIVER_HE…		HNF1A-…	SNAP25-A…	SLC22A…	ONECUT1	PIK3C2G	LINC02…	SLCO1B1	SLC17A1	SLCO1B3	CPS1	NPSR1-A…	PAH	HAL	DPYS	ABCC6	PKHD1	LINC025…	FST	SERPIN…	FTCD	GLDC	F2	TM4SF4	AGMO
CELLMARKERMSIGDB_DESCARTES_FETAL_LIVER_ME…		BNC2	LSAMP	RYR2	CTNNA3	PTPRD	IGFBP5	PTPRF	THSD4	PALLD	PDE7B	SEMA3C	KRT18	EZR	ALDH1A2	PAPPA	SLIT3	SULF1	CDON	KCTD8	PLCE1	WNT2B	RHPN2	FLRT3	ABI3BP
CELLMARKERMSIGDB_DESCARTES_FETAL_LUNG_BRO…		PKHD1	ROS1	SFTPC	C10orf90	PIK3C2G	LOC283…	SLC22A3	CLDN18	KCNJ15	CLDN6	AGER	LINC01331	LAMP3	ATP11A	CRLF1	FGFR2	SFTA3	RNF220	CPM	SLC34A2	ETV5	SFTPB	ACOXL	HAS3
CELLMARKERMSIGDB_DESCARTES_FETAL_LUNG_CIL…		CFAP157	CFAP52	SNTN	TTC29	C20orf85	DNAI1	ZBBX	VWA3B	TMEM212	DNAH9	HOATZ	FAM216B	DTHD1	ROPN1L	DNAH12	FAM18…	CAPS	CFAP73	C5orf49	DNAH3	MAP3K19	CCDC113	FABP6	VWA3A
CELLMARKERMSIGDB_DESCARTES_FETAL_LUNG_LYM…		STAB2	RELN	PTX3	CCL21	NTS	PARD6G	FLT4	PRR5L	LINC01…	NRP2	LOX	KLHL4	TSPEAR	PPFIBP1	KCNIP1	CMKLR2	CTHRC1	LINC021…	LETR1	RAB11FIP5	GJC2	GALNT15	PLIN5	GNG12-AS1

Table S5: Cell state marker gene list

CELLMARKERCAN_ACINAR_CELLS		CTRB1	KLK1	RBPJL	PTF1A	CELA3A	PRSS1	SPINK1	ZG16	CEL	CELA2A	CPB1	CELA1	RNASE1	AMY2B	CPA2	CPA1	CELA3B	PNLIP	CTRB2	PLA2G1B	PRSS2	CLPS	REG1A	SYCN
CELLMARKERMSIGDB_DESCARTES_FETAL_LUNG_NEU…		GRP	GHRL	SEC11C	ASCL1	CACNA1A	ACSL1	CHGA	CPE	NOL4	TMEM132D	SPHKAP	DDC	LINC002…	HEPACAM2	NEUROD1	INSM1	RFX6	CALCA	TPH1	KCND3	CA8	HES6	ABCC8	KCNH6
CELLMARKERMSIGDB_DESCARTES_FETAL_LUNG_STR…		TARID	BMP5	PDGFRA	GABRG3	ASPA	LAMA2	ITGA8	HPSE2	OCA2	PRR16	SLIT2	ZNF385D	LUM	TMEM132C	RSPO2	CACNA…	FREM1	ABCA6	FGF10-…	ROR1-AS1	COL13A1	MYOCD	FAP	S1PR3
CELLMARKERMSIGDB_DESCARTES_FETAL_LUNG_VAS…		NOTCH4	EDN1	APLNR	RXFP1	CLEC1A	KIT	ACE	LHX6	IL33	TCIM	ANO2	CD93	SOX7	NEURL1B	PLK2	RGCC	VWF	JAM2	NOSTRIN	PRND	CLEC3B	SLC22A…	NPR3	MMRN2
CELLMARKERMSIGDB_DESCARTES_FETAL_MUSCLE_L…		CCL21	PROX1	GJA1	NRP2	EFNA5	FLT4	KLHL4	STAB2	PPFIBP1	SHC1	TFF3	NRG3	PCLO	NTS	TBX1	PTX3	KLHL3	SNTG2	ARL4A	CMKLR2	SCAMP5	MYZAP	IL7	GPM6A
CELLMARKERMSIGDB_DESCARTES_FETAL_MUSCLE_S…		TACR3	PAX7	FGFR4	CADM2	GRIA4	MEGF10	SPATS2L	CLCN5	NTN4	TSPAN12	C1QTNF3	EDN3	NXPH2	MYF5	PITX3	CALN1	LRRTM3	POU4F1	SOX9	ERFE	NPY	OTOF	RGR	GSG1L
CELLMARKERMSIGDB_DESCARTES_FETAL_MUSCLE_S…		ACTA2	GUCY1A2	PLCL1	BMP5	GPC6	PDGFRB	ABCC9	CACNA1C	KCNAB1	MYH11	SLIT3	EDNRA	RGS5	SEMA5B	SULT1E1	TAGLN	ENPEP	AOC3	CCDC3	RERG	CARMN	ANGPT2	NDUFA4L2	PAWR
CELLMARKERMSIGDB_DESCARTES_FETAL_MUSCLE_V…		FLT1	PRND	PLVAP	PRCP	CD93	ADGRL4	PTPRM	RAPGEF5	SOX7	CYYR1	ANO2	TM4SF1	KITLG	EGLN1	NOTCH4	IL33	EFCAB14	SLC9C1	MMRN2	EYS	BTNL9	CLIC5	LINC01695	NOX4
CELLMARKERMSIGDB_DESCARTES_FETAL_PANCREAS…		GP2	CELP	REG1A	CELA3A	CTRB1	AQP8	PRSS1	CPA2	CEL	CTRC	PNLIPRP1	CTRB2	SYCN	REG3G	CPA1	CLPS	PLA2G1B	RBPJL	MT1G	CELA3B	AKR7A3	LOC100…	MT1H	SERPINI2
CELLMARKERMSIGDB_DESCARTES_FETAL_PANCREAS…		CCL19	CCL21	APOE	CXCL12	C1S	PTGDS	VCAM1	CSF1	FDCSP	PI15	CXCL14	SLC22A3	TNC	PGF	CXCL13	MSC	ADM2	SHISA3	LGI2	TNFSF11	SLC26A7	UBD	ADAMDEC1	FGF11
CELLMARKERMSIGDB_DESCARTES_FETAL_PANCREAS…		CDH19	SOX10	NKAIN3	MPZ	COL20A1	PLP1	PTPRZ1	S100B	OLFML2A	MDGA2	SOX2-OT	GRIK3	ERBB3	GFRA1	COL25A1	NKAIN2	LGI4	PRIMA1	COL28A1	ADAM23	TSPAN11	ARHGEF…	PRNP	SDK2
CELLMARKERMSIGDB_DESCARTES_FETAL_PANCREAS…		RALGAP…	NRG3	TFPI	PGM5	STON2	MMRN1	MPP7	NFATC1	TLL1	NTS	ENSG000…	LINC02147	STAB2	PARD6G	PTGS2	TRMT9B	ENTREP1	CTHRC1	GPM6A	RASSF9	TNFRSF1…	ANO9	ENSG0000…	F8
CELLMARKERMSIGDB_DESCARTES_FETAL_PANCREAS…		NFATC2	GAS1	CCBE1	GFPT2	MSLN	MYRF	WNT2B	CLDN1	KLF5	RARRES1	WT1	ITPKC	LINC026…	COL8A1	GPRC5A	TFPI2	HAS1	ADGRD1	COL11A1	UPK3B	SLAIN1	ALDH1A2	TMEM151A	PLA2G2A
CELLMARKERMSIGDB_DESCARTES_FETAL_PANCREAS…		CBLN1	THBS4	RGS5	MYH11	GJC1	NDUFA4…	CSPG4	ACTA2	AOC3	CCDC3	AVPR1A	SEPTIN4	HIGD1B	CARMN	FAM162B	ACTG2	NPTX1	ENSG000…	BMP5	DCLK3	OR51E1	GPR20	FOXF2	SLC6A1
CELLMARKERMSIGDB_DESCARTES_FETAL_PANCREAS…		PRND	FLT1	CLEC3B	NOSTRIN	ARHGEF15	NOTCH4	TM4SF18	PLVAP	CD34	SOX7	BTNL9	CLEC14A	MMRN2	PCDH12	CD93	JAM2	F2RL3	COL15A1	SEMA6B	CYYR1	CD109	JAG2	HSPG2	ESAM
CELLMARKERMSIGDB_DESCARTES_FETAL_PLACENTA…		PRG2	HLA-G	NOTUM	NOG	TAC3	FSTL3	DIO2	HTRA4	AOC1	FN1	ISM2	HSPG2	QSOX1	LVRN	PTPRF	CERCAM	HTRA1	FSTL1	HEXB	GPR78	ITM2B	TNFSF10	ASCL2	MYCNUT
CELLMARKERMSIGDB_DESCARTES_FETAL_PLACENTA…		LINC01…	AFF2	AGTR1	CCDC102B	EGFEM1P	PLA2G2A	GUCY1A2	PLCB1	LAMC3	ADAMTS18	CACNA1C	TBX5	PAG1	LINC023…	PLA2G5	EGFLAM	LIPG	GJC1	ITGA9	CCDC68	COL5A3	ARHGAP…	CNTN4	SCN7A
CELLMARKERMSIGDB_DESCARTES_FETAL_PLACENTA…		SLC24A2	CNTN5	KCNIP4	ITGBL1	LINC02307	FREM1	SLIT2	PCDH10	RSPO2	SLC16A10	MATN2	CADM3	MSC-AS1	STC1	NFASC	CLMP	LINC016…	CNTN6	POU6F2	LINC02268	NALCN	ELN	TCF21	LINC02006
CELLMARKERMSIGDB_DESCARTES_FETAL_PLACENTA…		TP63	HS6ST2	LRP2	CCDC54-…	SLC13A3	LGR5	MAPK4	TENM3	SMAGP	SLC22A11	NRXN3	SLC13A4	KANK1	TMEM150C	ZNRF3	SLC6A4	GNA12	SLC38A9	ISYNA1	GRAMD2A	IL1RAPL2	ALDH7A1	RASSF6	SLC27A2
CELLMARKERMSIGDB_DESCARTES_FETAL_PLACENTA…		PSG1	CSH2	PSG6	KISS1	INHBA	GH2	PSG9	PSG4	PSG5	ANGPT2	PSG3	TFPI2	HOPX	CGA	PSG2	SDC1	CSH1	HSD3B1	CYP19A1	GDF15	HSD17B1	SEMA3B	RHOBTB3	AFF1
CELLMARKERMSIGDB_DESCARTES_FETAL_SPLEEN_E…		ANK1	TFRC	XACT	SLC25A21	SPTA1	RHAG	FAM178B	GYPA	SLC4A1	KEL	MYB	C17orf99	GYPB	KCNH2	PPM1H	FAM11…	HEMGN	SLC38A5	ABCB10	KLF1	RHCE	CPOX	HMBS	ICAM4
CELLMARKERMSIGDB_DESCARTES_FETAL_SPLEEN_S…		KLHL29	LRRC4C	LINC03…	CACNA1D	LSAMP	NPAS3	CNTNAP2	UNC5C	TMTC1	RBMS3	NR2F2-A…	KCNMB2	MAPK10	ESRRG	GLIS3	LINC0…	CADPS	CARMN	TARID	KLHL13	PKNOX2	RARB	VLDLR-AS1	CIBAR1-DT
CELLMARKERMSIGDB_DESCARTES_FETAL_SPLEEN_V…		SNTG1	MYRIP	MECOM	PTPRB	FGF12	RAPGEF5	MAGI1	SIPA1L1	MAGI1-…	ADAMTSL1	CHRM3	BBOX1-AS1	ERG	HECW2	STAB2	CYYR1	CALCRL	ADGRL4	THSD7A	AFAP1L1	BTNL9	GABBR2	GALNT18	LRIG3
CELLMARKERMSIGDB_DESCARTES_FETAL_STOMACH_…		DNAH12	CDHR3	LRRIQ1	NEK10	DNAH9	CFAP299	CAPS	DNAH3	CFAP54	CFAP157	DNAH11	STK33	DNAH7	DCDC1	TMEM232	VWA3A	CFAP43	DNAAF1	KIAA20…	SPAG17	HYDIN	WDR49	ULK4	ARMC3
CELLMARKERMSIGDB_DESCARTES_FETAL_STOMACH_…		NRXN1	PTPRZ1	ZNF536	SOX2-OT	NKAIN3	SLC35F1	PLP1	NKAIN4	NKAIN2	EDNRB	OLFML2A	TFAP2A	S100B	CDH19	MOXD1	MPZ	CHL1	COL5A3	KIRREL3	ABCA8	ADAM23	TSPAN11	FAM78B	LGI4
CELLMARKERMSIGDB_DESCARTES_FETAL_STOMACH_…		CNTNAP2	KCNIP4	UNC5C	SYT1	CTNNA2	DSCAM	EML5	DPP6	GAP43	CNTN5	ELAVL4	OLFM1	STMN2	SYN2	PTPRR	SPOCK1	DCX	PHOX2B-…	MAP6	FSTL5	CUX2	PLD5	SHROOM1	CHRNA3
CELLMARKERMSIGDB_DESCARTES_FETAL_STOMACH_…		BACH2	SKAP1	LEF1	BCL11B	IL7R	CD247	TCF7	THEMIS	TRBC2	CD52	LTB	PTPRCAP	TMC8	CD3E	CD6	CD69	NIBAN3	ITK	CD3D	SCML4	CD7	CD3G	LCK	SAMD3
CELLMARKERMSIGDB_DESCARTES_FETAL_STOMACH_…		SULF1	PDZRN4	GFPT2	CDON	WT1	WNT2B	C3	RSPO3	CAVIN2…	ALDH1A2	GAS1	TFPI2	COL8A1	PTPRQ	LINC022…	UPK3B	HAS1	BNC1	NPHS1	LINC02643	LRP2	AOX1	LINC02360	FAM110C
CELLMARKERMSIGDB_DESCARTES_FETAL_STOMACH_…		DMBT1	APOA1	MUC13	APOB	ALDOB	MT1G	APOA4	CPS1	SLC15A1	CDHR5	MAMDC4	GDA	MT1H	PHGR1	MTTP	SLC19…	ONECUT2	AMN	MYO7B	SI	MALRD1	SPIB	CDH17	RBP2
CELLMARKERMSIGDB_DESCARTES_FETAL_STOMACH_…		CD74	CST3	HLA-DRA	HLA-DRB1	HLA-DPA1	TNFRSF…	HLA-DPB1	CD83	C1QC	SPI1	CPVL	CSF2RA	JAML	MYO1F	PLAUR	WDFY4	HLA-DRB6	IRF8	CD4	C1QA	HLA-DQA1	NR4A3	CYBB	HLA-DRB5
CELLMARKERMSIGDB_DESCARTES_FETAL_STOMACH_…		GHRL	GAST	CACNA1A	CHGA	SST	SLC38A…	TTR	GCG	TPH1	LINC002…	HS3ST4	MAOB	RFX6	ST18	HDC	KCNH7	LINC009…	GC	GHRLOS	NEUROD1	PPY	PAX6	SEC11C	VWA5B2
CELLMARKERMSIGDB_DESCARTES_FETAL_STOMACH_…		ATP4B	ATP4A	MFSD4A	CBLIF	ESRRG	B4GALN…	GREM2	PRDM16-…	ALB	DUSP4	KCNJ16	KCNE2	TRIM50	SIGLEC11	GPT2	FAM16…	RAB11FI…	CKM	CYB5R1	FGA	CDH10	AOC3	PDHA1	PAQR5
CELLMARKERMSIGDB_DESCARTES_FETAL_STOMACH_…		KRT4	KRT13	KRT5	AQP3	S100A2	SFN	S100A9	KRT15	SERPIN…	DSG3	TACSTD2	KRT6A	NECTIN1	TMEM40	RHOV	SPINK5	SERPINB5	TP63	PKP1	LYPD3	CALML3	LINC01…	ZNF750	CAPN14
CELLMARKERMSIGDB_DESCARTES_FETAL_STOMACH_…		COL3A1	DCN	COL1A1	PDGFRA	FBLN1	MFAP4	MYH11	CXCL14	COL6A3	EMILIN1	CD248	PTCH1	FENDRR	ACTG2	HSPB6	NELL1	ACTA2	SFRP2	NRK	EBF2	PCDH18	FOXF1	C7	LINC03007
CELLMARKERMSIGDB_DESCARTES_FETAL_STOMACH_…		FLT1	APLNR	PLVAP	EPAS1	CD34	KITLG	A2M	MADCAM1	SOX7	EMCN	CYYR1	KANK3	NOTCH4	CLEC3B	GPRC5A	PRND	PCDH12	MEOX1	NKX2-3	TM4SF18	CLEC14A	CCM2L	ARHGEF15	CPNE5
CELLMARKERMSIGDB_DESCARTES_FETAL_THYMUS_T…		THEMIS	CD96	LINC01…	TSHR	LZTFL1	LINC01…	PXYLP1	ARHGAP1…	NEIL3	LIN28B	GVINP1	SKA3	UBASH3A	CDK6-AS1	AOX3P	PLK4	BEST3	PHGDH	HKDC1	KLRC4-KL…	LIG4	LINC00…	FBXO41	UBIAD1
CELLMARKERMSIGDB_DESCARTES_MAIN_FETAL_ACI…		FBXO2	CTRC	CELA2A	AKR7A3	RNF186	CELA3B	CELA3A	DIRAS3	BTG2	POMC	REG3G	REG1A	CPB1	SERPINI2	CCDC110	SPINK1	LMAN2	CLPS	LOC285…	LINC01624	CPA2	CPA1	PRSS1	RARRES2
CELLMARKERMSIGDB_DESCARTES_MAIN_FETAL_ADR…		UTS2	CASP9	LINC01…	DHCR24	PTPRVP	LINC00…	PLB1	HSPD1	HSPE1	MOB4	INHA	LINC01807	SH3BP5-…	SH3BP5	SCAP	KLHDC…	ALAS1	LINC014…	LINC02…	GNRHR	GASK1B-…	MSMO1	HMGCR	JAKMIP2
CELLMARKERMSIGDB_DESCARTES_MAIN_FETAL_AST…		CACHD1	WARS2-IT1	ATP1A2	LGR6	ENSG0000…	ZNF863P	CYP26B1	NCKAP5-…	RFTN2	PAX3	CCDC140	SLC6A11	BOC	DCLK2	SNCAIP	SLC25…	ELOVL2-…	NHSL1-A…	MMD2	ADCYAP1R1	PTN	RPS20P…	RGS20	RNU6-133…
CELLMARKERMSIGDB_DESCARTES_MAIN_FETAL_BIP…		LACTBL1	MIR3671	LINC01…	ACKR1	LHX4	GS1-27…	RPL11P1	LINC012…	NCKAP5	ZNF385B	KRT8P15	ASIC4	NYAP2	AGAP1	KCTD6	FEZF2	CADPS	RPL14P2	PRICKL…	TAFA4	CRYBG3	GABRR3	C3orf70	FSTL5
CELLMARKERMSIGDB_DESCARTES_MAIN_FETAL_BRO…		FAM41C	CHIA	SFTPB	ACOXL	ESYT3	ETV5	LINC02026	BMP3	NDNF	SMARCA5	GUSBP5	ENSG0000…	IRX2	IRX1	NT5ELP	LINC0…	LINC013…	AGER	ROS1	TSPAN13	LINC030…	LINC01…	SFTPC	BAMBI
CELLMARKERMSIGDB_DESCARTES_MAIN_FETAL_CAR…		TNNI3K	MYBPHL	NTMT2	PPP1R12B	MIXL1	RYR2	LINC01954	SLC8A1	VDAC2P5	PRSS42P	FGF12	FGF12-AS3	ENSG000…	LINC025…	SORBS2	MARCH…	EIF3KP1	SLC27A6	MLIP	FILIP1	TRDN-AS1	HRAT17	TRIQK	ENSG0000…
CELLMARKERMSIGDB_DESCARTES_MAIN_FETAL_CIL…		TTLL10	CFAP74	MORN1	TTC34	TPRG1L	TP73	LNCTAM34A	CENPS	CFAP107	FHAD1	CROCC	UBXN10	STPG1	UBXN11	NUDC	CD164…	DCDC2B	TEKT2	ADPRS	OSCP1	DNALI1	MYCBP	ZMYND12	CCDC30
CELLMARKERMSIGDB_DESCARTES_MAIN_FETAL_DUC…		HES4	KLHDC7A	IFFO2	GPR3	CITED4	GSTM3	ATP1A1	ANXA9	ADORA1	LEFTY1	CYS1	LINC01829	ANXA4	GMCL1	SUCLG1	SCTR	CHDH	FGFR3	SLC4A4	ART3	ADAMTS16	ZBED3	ID4	GMNN
CELLMARKERMSIGDB_DESCARTES_MAIN_FETAL_END…		NBL1	ECE1	ZBTB8A	GJA4	LEPR	ITGA10	NPR1	THBS3	HMCN1	FMOD	ELK4	AGPS	CALCRL	INPP1	NAB1	NPPC	ACKR3	FBLN2	CLEC3B	RHOA	HYAL2	HEG1	ACKR4	HS3ST1
CELLMARKERMSIGDB_DESCARTES_MAIN_FETAL_ENS…		OPRD1	CELF3	RGSL1	TLX2	LINC01238	MYH15	IQCJ	JAKMIP1…	PHOX2B…	TNPO1	LIX1	HMGN1P14	NRG2	PCDHA13	DPYSL3	HTR4	NRSN1	LRFN2	SMIM28	VIP	ETV1	TNS3	NAMA	GARNL3
CELLMARKERMSIGDB_DESCARTES_MAIN_FETAL_EPI…		TNFRSF8	PLA2G2A	S100A10	SPRR2B	ITLN1	PRG4	KCNT2	RAB6D	CCNYL1	LINC006…	CCDC80	BCHE	ENSG000…	BST1	EPGN	ANXA3	NPNT	RN7SL68…	GUCA1A	TBX18	RAET1E	RAET1G	SMOC2	INMT
CELLMARKERMSIGDB_DESCARTES_MAIN_FETAL_GAN…		MFSD14A	SUSD4	CPNE9	DDX50P2	LINC02505	POU4F2	LRRTM2	TFAP2D	NEFM	RBPMS	CHRNB3	CHRNA6	LINC016…	TMEM74	TRHR	OPN4	INA	SLC17A6	P2RX3	MMP20	TRHDE	LINC00…	OBI1-AS1	RPL31P54
CELLMARKERMSIGDB_DESCARTES_MAIN_FETAL_GOB…		FAM177B	CAPN9	GKN2	SLC9A4	SLC9A2	CLASP1…	ENSG0000…	A4GNT	ARL14	LRRC31	PRKCI	UGT2B15	CXCL5	CXCL3	BMP2K-DT	ANXA10	FBXW11	FOXQ1	GMDS	MUCL3	SPDEF	PGC	FUT9	MARCKSL1…
CELLMARKERMSIGDB_DESCARTES_MAIN_FETAL_HEM…		SNHG3	TFAP2E-A…	IL12RB2	CD244	CSRP1-AS1	RPS10P7	TARBP1	GCC2-AS1	FASTKD1	CDCA7	ENSG000…	HSPE1-MO…	AOX2P	RNU1-13…	DYTN	EMC3-…	FANCD2OS	CBX3P10	BAP1	TMCC1-DT	LRRFIP1…	RFC4	SEPSECS	RN7SL558P
CELLMARKERMSIGDB_DESCARTES_MAIN_FETAL_HOR…		LINC02…	TPM3	TNR	TNR-IT1	DCTN1-AS1	HS6ST1	ARHGEF4	TANC1	CNTN4	IL12A-AS1	NDST3	SEMA6A-A…	FCHSD1	ARHGAP2…	PLAC8L1	TFAP2B	B3GAT2	RPL17P23	RNU1-3…	EPM2A	SEM1	LINC02…	LINC02155	MIR2052HG
CELLMARKERMSIGDB_DESCARTES_MAIN_FETAL_INH…		AJAP1	DISP3	EPHA8	MYT1L-A…	SLC30A3	GAD1	CHL1	KIT	UNC5C	NEUROG2	LINC021…	HCRTR2	FNDC1-I…	MSRA	RNU6-72…	PRSS51	XKR4	ENSG000…	BHLHE22	SH3GL2	PBX3	ADARB2…	GAD2	SLC6A5
CELLMARKERMSIGDB_DESCARTES_MAIN_FETAL_INH…		LHX8	DRD3	DLX6-A…	GRM5P1	PMCH	SLAIN1	PDYN																	
CELLMARKERMSIGDB_DESCARTES_MAIN_FETAL_INT…		C1QTNF…	AGMAT	GUCA2A	CLCA1	RPL13AP8	LPGAT1	KHK	XDH	RNU6-4…	LBX2	LINC015…	LINC01123	LCT	VIL1	GPR35	APRG1	C3orf85	RBP2	ZBTB38	SI	TMEM207	MELTF	FAM157A	LINC00955
CELLMARKERMSIGDB_DESCARTES_MAIN_FETAL_ISL…		C1orf1…	FAM131C	CAMK2N1	HPCA	RAB3B	ERO1B	EGR4	ACVR1C	GCG	G6PC2	FEV	ENSG0000…	PTPRN	ECEL1	CASR	VWA5B2	SST	NKX6-1	HADH	LINC02106	PCSK1	SCGN	HSPA1A	KCNK16
CELLMARKERMSIGDB_DESCARTES_MAIN_FETAL_LYM…		SELENON	TIE1	GNG12-…	C2CD4D	S100A16	SLC27A3	SHC1	FCGR2C	RNU7-1…	GJC2	RAB11FI…	TFPI	NRP2	LINC020…	LINC006…	EFCC1	PLSCR4	PTX3	WFS1	SMAD1	SMAD1-A…	MAP3K1	LRRC70	LINC02058
CELLMARKERMSIGDB_DESCARTES_MAIN_FETAL_MES…		MEGF6	PODN	ADAMTS…	LAMC2	EGOT	GADL1	PSAPL1	RN7SL10…	LINC02…	GPT2	NSA2P7	MAP3K8	SFTPD	SFTPD-A…	HDAC1P1	PDZRN4	RND1	RPL31P52	FGF9	BDKRB1	FENDRR	ALOX15	GLP2R	TSHZ3-AS1
CELLMARKERMSIGDB_DESCARTES_MAIN_FETAL_MET…		TRABD2B	LINC01781	NPHS2	HSPE1P13	SIX2	LINC01…	LYPD1	HAGLR	HOXD1	C1QTNF7	LINC022…	SLC34A1	HOXA9	HOXA11-…	BMPER	SLC13…	XRCC6P4	EYA1	DMRT3	LINC02893	CEP164P1	COL13A1	FGF8	THEM7P
CELLMARKERMSIGDB_DESCARTES_MAIN_FETAL_MYE…		C1QA	C1QC	C1QB	THEMIS2	C1orf162	CTSS	TNFAIP8L2	S100A12	RGL1	IL10	CR1	NLRP3	CD207	MARCO	STAB1	FRMD4B	CD86	RNPS1P1	MIR394…	CD180	HBEGF	CD14	TIMD4	HK3
CELLMARKERMSIGDB_DESCARTES_MAIN_FETAL_NEU…		TAS1R3	PRORSD1P	BCAN-A…	POTEI	POTEJ	FAR2P3	HES6	GHRLOS	NUDT16…	ACSL1	LINC021…	TENM2-AS1	VWA7	RFX6	RPL7AP37	SLC35…	CNKSR3	LFNG	SLC26A5	MBOAT4	ENTPD8	PKD2L1	CALCA	TPH1
CELLMARKERMSIGDB_DESCARTES_MAIN_FETAL_OLI…		PDE4B	BCAN	BCAN-A…	BRINP3	CAMSAP2	RNF144A	GPR17	NBEAP2	SCN1A	C2orf80	RTP5	CSPG5	PLAAT1	FIP1L1	PDGFRA	LINC0…	UGT8	SCRG1	GLRA3	TRIO	NIM1K	LINC02…	PPP2R2B	COL9A1
CELLMARKERMSIGDB_DESCARTES_MAIN_FETAL_PDE…		GABRD	NEGR1-IT1	CCDC88A	ANKRD20…	CACNB4	PDE11A	API5P2	SH3BP4	FANCD2…	ANKRD28	GALNT15	FILIP1L	TAGLN3	SNORA58	KCNAB1	RNU6-…	SLIT2	SLIT2-I…	MIR218…	PCDH7	GABRA2	TMPRSS…	RASGEF1B	NPM1P41
CELLMARKERMSIGDB_DESCARTES_MAIN_FETAL_PDE…		MIR200B	TNFRSF14…	CLCNKB	MDS2	LIN28A	PPIEL	RLF	ATPAF1	C1orf1…	YIPF1	MYSM1	SRSF11	CNN3-DT	SEC63P1	ST7L	WARS2	VPS45	UBAP2L	INSRR	TADA1	FMO6P	RABGAP…	TNN	CDC73
CELLMARKERMSIGDB_DESCARTES_MAIN_FETAL_RET…		HES5	UQCRHL	EPHB2	MIR3659…	ILDR2	CRB1	OPTC	LHCGR	FBLN7	DAPL1	TNS1-AS1	PRSS56	RARB	ARHGEF2…	ARHGEF26	LINC0…	FAM184B	KLHL8	PKD2	RN7SKP35	GATB	C5orf34	RNF180	LINC01621
CELLMARKERMSIGDB_DESCARTES_MAIN_FETAL_SAT…		CELA2B	NBPF1	PAX7	FNDC5	PTGFRN	LINC02…	OLFML2B	NCOA1	LINC01…	LSP1P4	CHRNA1	MSTN	SPATS2L	RNY4P34	DIRC3-A…	ERFE	DAG1	SLC25A26	GXYLT2	NXPE3	TACR3	C1QTNF3	SKP2	FGFR4
CELLMARKERMSIGDB_DESCARTES_MAIN_FETAL_SCH…		ANGPTL7	EXTL1	BMP8B	LINC004…	FOXD3	MPZ	DUTP6	C1orf198	SCN7A	ADAM23	CHL1-AS2	SEMA3G	GFRA3	COL28A1	GJC3	TMEM1…	PLAT	LINC016…	AQP7P1	LINC01505	OLFML2A	ENTPD2	LGI1	TECTB
CELLMARKERMSIGDB_DESCARTES_MAIN_FETAL_SLC…		CCNL2	MRPL20	PRKCZ-…	RN7SL57…	NPHP4	ZBTB48	TMEM201	ANO7L1	CPLANE2	ENSG000…	AUNIP	ZPLD2P	ZNF683	ZSCAN20	ZMYM1	ZC3H1…	EXO5-DT	KDM4A-A…	TOE1	EFCAB14-…	ACADM	RPL7P9	VAV3-AS1	AKNAD1
CELLMARKERMSIGDB_DESCARTES_MAIN_FETAL_SMO…		PLA2G5	WNT3A	ADCY3	ACTG2	GPAT2	RFX8	COL6A3	MDFIC2	MYLK	THBS4	CARMN	PDGFRB	KCNMB1	SEPTIN7…	EBF2	GPR20	LAMC3	TACR2	ACTA2-…	ACTA2	ANO1	TAGLN	LINC02398	KCNJ8
CELLMARKERMSIGDB_DESCARTES_MAIN_FETAL_STE…		RN7SL6…	NBPF3	ALPL	ECM1	COLEC11	ALLC	EHBP1	BMPR2	RBMS3	RPS12P5	PTH1R	LINC00698	ADAMTS9	RN7SL75…	AGTR1	MASP1	COL25A1	LINC004…	HHIP	RBM46	ADAMTS12	B3GNTL…	CLMAT3	ADRA1B
CELLMARKERMSIGDB_DESCARTES_MAIN_FETAL_SYM…		NT5C1A	C1orf226	ZDBF2	UNC80	SCG2	TMIE	NT5DC2	SIDT1	QDPR	LINC025…	LINC029…	BMPR1B-DT	UNC5C-A…	CARTPT	FKBP5	MRAP2	VWDE	PPP1R17	TMEM130	SLC18A1	GOT1L1	PENK	ARC	ZNF483
CELLMARKERMSIGDB_DESCARTES_MAIN_FETAL_UNI…		LMX1A	LEMD1-DT	UPP2	EOMES	STXBP5L	ZIC4	LINC02032	LINC020…	NAALAD…	NWD2	NDST4	TRPC3	BLTP1	QKILA	GRIA2	FBXL2…	GRM1	VWC2	CACNA2…	CACNA2D1…	HTR5A	EN2	CNPY1	BARHL1
CELLMARKERMSIGDB_DESCARTES_MAIN_FETAL_URE…		MTCO1P…	PLA2G2F	LINC02…	ASTL	MYO3B	NUDT16…	NAALADL2	ENSG000…	TBC1D9	MTND3P19	SHROOM1	MTND4LP30	NIPAL4	CLPSL1	MYO6	C7orf…	KCP	RPL10P19	SNX31	ACER2	PTGR1	GPR15LG	EMX2OS	EMX2
CELLMARKERMSIGDB_DESCARTES_MAIN_FETAL_VAS…		SRARP	FCN3	MTMR9LP	FAM167B	ADGRL4	FCGR2B	SELE	HLX	EGLN1	ENSG000…	SLC39A10	FAM124B	SNRK	SLC9C1	TM4SF18	LXN	LINC020…	LDB2	LINC02…	ESM1	CRHBP	FOXF2	NOTCH4	RAPGEF5
CELLMARKERMSIGDB_DESCARTES_MAIN_FETAL_VIS…		RNU6-1…	RNU6-123…	PATJ-DT	MTX1	RUSC1	CFHR5	SEPTIN14…	HLX-AS1	FKBP1B	CTNNA2-…	LINC019…	SLC5A7	HAGLROS	BZW1-AS1	RNU7-11…	RAB6B	RPL8P3	SLC7A14	OPA1-A…	TMEM271	PDE6B-A…	UCHL1	NAP1L5	NPY2R
CELLMARKERMSIGDB_DURANTE_ADULT_OLFACTORY_…		MS4A1	CD37	LTB	CD79A	CD79B	BANK1	HLA-DQA1	TNFRSF1…	VPREB3	LINC017…	LINC009…	CD52	HLA-DQB1	IGHM	MARCHF1	IRF8	LAPTM5	HLA-DPB1	HLA-DRA	CD83	HLA-DRB1	CD69	CXCR4	EVI2B
CELLMARKERMSIGDB_DURANTE_ADULT_OLFACTORY_…		STATH	LYZ	ZG16B	FDCSP	LTF	TCN1	SLPI	PIP	AZGP1	ODAM	BPIFB1	PIGR	RNASE1	C6orf58	DMBT1	HP	S100A1	CXCL17	PPP1R1B	LRRC26	CLDN10	NDRG2	XBP1	ELAPOR1
CELLMARKERMSIGDB_DURANTE_ADULT_OLFACTORY_…		LTB	IL7R	CD52	TRAC	CXCR4	TRBC2	GPR183	CD3D	IL32	TRBC1	CD69	CD48	PTPRC	CD2	KLRB1	CORO1A	FYB1	CD3E	ACAP1	CD3G	TAGAP	CLEC2D	RPS29	HCST
CELLMARKERMSIGDB_DURANTE_ADULT_OLFACTORY_…		CCL5	CCL4	KLRB1	CXCR4	CCL4L2	TRAC	CD2	CD3D	IL32	CD52	CD69	GZMA	TRBC2	HCST	IL7R	PTPRC	KLRC1	CD8A	CORO1A	CD3G	CD3E	CD7	TRBC1	BTG1
CELLMARKERMSIGDB_DURANTE_ADULT_OLFACTORY_…		LUM	DCN	APOD	FBLN1	IGFBP6	MGP	SFRP1	SFRP2	CPE	NBL1	PCOLCE	PTN	COL1A2	COL3A1	SERPING1	IGFBP7	CRABP2	CSRP2	INMT	LGALS1	RARRES2	C1S	POSTN	SERPINF1
CELLMARKERMSIGDB_DURANTE_ADULT_OLFACTORY_…		NEUROD1	ELAVL4	SOX11	HES6	NHLH1	MEX3A	GNG8	TUBB2B	LHX2	ELAVL3	STMN2	FEZF1	STMN4	UCHL1	RPRM	EMX2	STMN1	MDM1	CALB2	PCBP4	CKB	JPT1	MARCKSL1	PTMA
CELLMARKERMSIGDB_DURANTE_ADULT_OLFACTORY_…		HLA-DRA	HLA-DPB1	HLA-DP…	HLA-DRB1	HLA-DQB1	HLA-DQ…	CD74	RGS1	HLA-DR…	C1QA	TYROBP	CCL3L1	AIF1	CCL3	C1QB	MS4A6A	IL1B	RGS2	C1QC	FCER1A	FCER1G	CD83	HLA-DMA	SGK1
CELLMARKERMSIGDB_DURANTE_ADULT_OLFACTORY_…		TPSB2	TPSAB1	CPA3	MS4A2	CTSG	HPGDS	RHEX	RGS13	IL1RL1	HDC	SLC45A3	FCER1G	SLC18A2	CD69	TYROBP	KIT	LIF	VWA5A	ALOX5AP	GPR65	SAMSN1	FCER1A	RGS1	LTC4S
CELLMARKERMSIGDB_DURANTE_ADULT_OLFACTORY_…		MS4A7	FCER1A	MS4A6A	C1QC	MNDA	C1QA	C1QB	AIF1	LST1	IL1B	FCER1G	TYROBP	PHACTR1	CCL3L1	IFI30	HLA-D…	CD83	HLA-DQA1	FGL2	HLA-DQB1	CCL3	RGS1	GPR183	RGS10
CELLMARKERMSIGDB_DURANTE_ADULT_OLFACTORY_…		GNLY	NKG7	GZMB	PRF1	KLRD1	FGFBP2	CST7	MYOM2	GZMA	CCL4	GZMH	KLRF1	TRDC	CTSW	CCL5	HCST	CD247	CD7	TYROBP	KLRB1	CLIC3	GZMM	FCGR3A	ITGB2
CELLMARKERMSIGDB_DURANTE_ADULT_OLFACTORY_…		HEPACA…	GNGT1	FOXI1	TMEM61	ASCL3	STAP1	CLGN	CLCNKB	ATP6V1…	LINC011…	PCP4	BSND	CFTR	CRTAC1	DMRT2	RGS13	HES6	IGF1	ALKAL1	QPCT	SCNN1B	TUBB2B	EPCAM	SLC14A1
CELLMARKERMSIGDB_DURANTE_ADULT_OLFACTORY_…		ACKR1	SELE	GNG11	SPARCL1	RAMP2	CLDN5	ITM2A	EMCN	VWF	A2M	AQP1	IFI27	CAV1	LIFR	EGFL7	CLEC1…	SPRY1	ECSCR	ADGRL4	SOX17	NPDC1	THBD	TM4SF1	CD34
CELLMARKERMSIGDB_DURANTE_ADULT_OLFACTORY_…		SFN	AQP3	S100A2	KRT19	NTS	ALDH3A1	TACSTD2	PERP	MT1X	CD9	KRT6A	CLDN4	KRT5	SERPINB5	KLF5	HSPB1	KRT8	LYPD3	PKM	SLC25A5	FXYD3	S100A16	KRT18	KRT23
CELLMARKERMSIGDB_DURANTE_ADULT_OLFACTORY_…		MSMB	LYPD2	MUC5AC	TFF3	WFDC2	S100P	CYP2A13	AGR2	SERPIN…	SEC14L3	UGT2A1	PSCA	LCN2	SCGB3A1	CXCL17	PI3	GSTP1	ADH1C	TSPAN8	CXCL1	KRT18	MT1G	VMO1	KRT8
CELLMARKERMSIGDB_DURANTE_ADULT_OLFACTORY_…		PTTG1	MNS1	TPPP3	CFAP276	STMN1	FAM183A	C9orf24	PIERCE1	CKB	TUBB4B	CAPS	NQO1	RSPH1	PRDX1	ALDH3A1	CALM2	DYNLT1	C20orf85	TSPAN1	PIFO	GSTA1	MORN2	PRDX5	CETN2
CELLMARKERMSIGDB_DURANTE_ADULT_OLFACTORY_…		MYH11	TAGLN	MYL9	C11orf96	ACTA2	TPM2	RGS5	RGS16	CALD1	TPM1	MYLK	FILIP1L	NDUFA4L2	DSTN	ADAMTS1	FHL1	THBS1	MFGE8	FRZB	GUCY1A1	CAV1	RERGL	GEM	TIMP3
CELLMARKERMSIGDB_FAN_EMBRYONIC_CTX_ASTROC…		SPARCL1	NCAN	FAM107A	ID2	EEPD1	GRM3	VEPH1	LINC009…	TNC	ADGRV1	SLCO1C1	CDC42EP4	PEA15	DOK5	EZR	SLC1A3	RGS20	LRRC3B	PAX6	LGALS3	HEY1	FABP7	ALDOC	OLFM2
CELLMARKERMSIGDB_FAN_EMBRYONIC_CTX_BIG_GR…		IGFBP7	COL4A1	ITM2A	ITIH5	RGS5	FN1	FOXS1	BGN	PDGFRB	CLDN5	COL4A2	ESAM	SLC6A12	GGT5	EDNRA	PRELP	ECSCR	CA4	NDUFA4…	COL1A2	NOTCH3	DPEP1	CD248	GPER1
CELLMARKERMSIGDB_FAN_EMBRYONIC_CTX_BIG_GR…		RELN	WFIKKN2	IGFBP5	PRPH	PCP4	LINC01…	NDNF	ACHE	TRH	ISOC1	MAB21L1	CBLN1	C7	PLSCR4	C1QTNF3	RSPO3	HHATL	AMIGO2	TAGLN2	PCP4L1	STK32A	CRABP2	OTOS	NEFH
CELLMARKERMSIGDB_FAN_EMBRYONIC_CTX_BIG_GR…		MEIS2	ARPP21	NEUROD6	CAMK2B	SEMA3C	CSRP2	SATB2	DOK5	CCBE1	SEZ6	ENC1	NELL2	NEUROD2	CACNA2D1	MIAT	BHLHE…	POU3F2	ZBTB18	CAMKV	PCLO	SYBU	DOCK9	LINC01102	SLA
CELLMARKERMSIGDB_FAN_EMBRYONIC_CTX_BIG_GR…		VSIG4	P2RY13	P2RY12	CX3CR1	TREM2	CH25H	CSF1R	C3	C1QB	RGS1	TMEM52B	C1QA	SPP1	FOLR2	FPR1	BHLHE…	FCGR1A	LILRB4	TMIGD3	PTAFR	CD14	PDK4	C1QC	GPR34
CELLMARKERMSIGDB_FAN_EMBRYONIC_CTX_BRAIN_…		CLDN5	ITM2A	MFSD2A	APCDD1	SLC38A5	ESAM	RAMP2	CDH5	SLC7A5	ADGRL4	PECAM1	FLT1	IGFBP7	SRARP	GNG11	SLC2A1	IFI27	KDR	CD34	PCAT19	ROBO4	CAV1	PODXL	BSG
CELLMARKERMSIGDB_FAN_EMBRYONIC_CTX_BRAIN_…		RGS5	PDGFRB	CALD1	NOTCH3	ATP1A2	BGN	COL4A1	LAMC3	NDUFA4…	SPARC	COL4A2	PTN	ITIH5	COL1A2	SLC6A1	FN1	CD248	SLC6A12	KCNJ8	CCDC3	TFPI	PRELP	PLXDC1	GGT5
CELLMARKERMSIGDB_FAN_EMBRYONIC_CTX_EX_1_E…		ENC1	PANTR1	LINC01…	MEIS2	SLA	CSRP2	EIF1B																	
CELLMARKERMSIGDB_FAN_EMBRYONIC_CTX_IN_2_I…		RBP1	ACKR3	BRINP2	LHX6	PDZRN4	MAF	SST																	
CELLMARKERMSIGDB_FAN_EMBRYONIC_CTX_IN_3_I…		PDE4DIP	ERBB4	GAD1	MAF	LHX6	BOD1L1																		
CELLMARKERMSIGDB_FAN_EMBRYONIC_CTX_IN_5_I…		DLX5	DLX1	SCGN	CALB2	CNR1	DLX6-A…																		
CELLMARKERMSIGDB_FAN_EMBRYONIC_CTX_IN_6_I…		TMEM16…	DLX1	BTG1	HSPA1A	SCGN	DLX6-A…																		
CELLMARKERMSIGDB_FAN_EMBRYONIC_CTX_MICROG…		CCL3	FOSB	IL1B	CD83	CCL4	IER2	MIR23AHG	IER3	PPP1R1…															
CELLMARKERMSIGDB_FAN_EMBRYONIC_CTX_OLIG		MOBP	ERMN	PLP1	TF	MOG	KLK6	ADAMTS4	MYLK	MBP	CNP	PADI2	MAG	CLDN11	PTGDS	PPP1R14A	ENPP2	SEPTIN4	SLC31A2	CYB5R2	CAPN3	APLP1	TMEM63A	AOPEP	CDK18
CELLMARKERMSIGDB_FAN_EMBRYONIC_CTX_OPC		PDGFRA	EPN2	SCRG1	PLPPR1	APOD	ARL4A	COL9A1	PCDH15	BEX1	MEG3	GRIA2	THY1	PLAT	SERTAD4…	PLLP	BCHE	LHFPL3	LRRC4C	GPR17	LIMA1	COL20A1	CA10	CNTN1	PHLDA1

Table S5: Cell state marker gene list

CELLMARKERCAN_ACINAR_CELLS	CTRB1	KLK1	RBPJL	PTF1A	CELA3A	PRSS1	SPINK1	ZG16	CEL	CELA2A	CPB1	CELA1	RNASE1	AMY2B	CPA2	CPA1	CELA3B	PNLIP	CTRB2	PLA2G1B	PRSS2	CLPS	REG1A	SYCN
CELLMARKERMSIGDB_FAN_OVARY_CL1_GPRC5A_TNF…	TCF21	TNFRSF12A	DCN	GPRC5A	IQCG	ARID5B	SLC3A2	ERRFI1	C1R	CFD	C7	C1S	FGF7	RTN4	LUM	UAP1	GLIS3	DIRAS3	LMNA	KRT19	DNAJA1	MYC	EIF4A3	GLRX
CELLMARKERMSIGDB_FAN_OVARY_CL11_MURAL_GRA…	HSPA6	DSP	LRAT	ACSM3	KRT8	KRT18	PPP1R12B	CITED2	GJA1	GSTA1	AMH	SOWAHC	HIF1A	LCMT1	HSPA1A	HSPA1B	HSPH1	TCAF1	KLF6	P3H2	PTGER2	IGF2BP2	MAGED2	NECTIN2
CELLMARKERMSIGDB_FAN_OVARY_CL13_MONOCYTE_…	HLA-DRA	LYZ	CXCL8	HLA-DPB1	HLA-DPA1	HLA-DR…	C15orf48	CD74	HLA-DQ…	HLA-DQA1	TYROBP	CST3	FCER1G	AIF1	CCR7	HLA-D…	GPR183	LST1	S100A4	HLA-DMA	SOD2	CTSS	EMP3	SRGN
CELLMARKERMSIGDB_FAN_OVARY_CL14_MATURE_SM…	RGS5	ACTA2	TAGLN	ADIRF	MYL9	PLN	NDUFA4L2	RERGL	TIMP3	SOD3	TPM2	CD9	CRIP1	MYH11	GADD45B	TINAG…	MFGE8	PLAC9	LBH	DSTN	FRZB	BCAM	MT1E	MT2A
CELLMARKERMSIGDB_FAN_OVARY_CL15_SMALL_ANT…	KRT18	CKS2	GATM	KRT8	CHORDC1	CITED2	ZFAND2A	LIPH	EGR4	HSPE1	SNAPC1	DNAJB4	UBXN8	GADD45G	ARL5B	GMNN	HEY2	INHA	HSPA6	TNNI3	ATP5IF1	BAG3	NECTIN2	NOP58
CELLMARKERMSIGDB_FAN_OVARY_CL18_B_LYMPHOC…	IGKC	JCHAIN	GPR183	PTGDS	CD74	GZMB	HLA-DRA	HLA-DQB1	ALOX5AP	HLA-DRB1	CD83	CCR7	EZR	CTSC	HLA-DQA1	TXN	CYBA	PLEK	IL3RA	CD79A	LAPTM5	IRF8	LTB	PLD4
CELLMARKERMSIGDB_FAN_OVARY_CL2_PUTATIVE_E…	TCEAL4	IGFBP5	OGN	HSPA1B	JUN	LUM	FOS	DCN	EGR1	LGALS1	RARRES2	CALD1	C1S	TIMP2	COL1A2	APOE	DDIT4	PDK4	MFAP4	SELENOP	JUNB	SERPIN…	SERPING1	COL6A1
CELLMARKERMSIGDB_FAN_OVARY_CL5_HEALTHY_SE…	COL3A1	APOD	MEST	COL1A2	COL1A1	APOE	WFDC1	COLEC11	COL6A1	SPARC	APOC1	CCN1	BGN	ENC1	MATN2	PCOLCE	CYTOR	COL15A1	MIR443…	COL6A3	COL6A2	TCIM	NBEAL1	NCALD
CELLMARKERMSIGDB_FAN_OVARY_CL6_PUTATIVE_E…	COL1A1	COL1A2	JUN	FOS	C7	LUM	CXCL12	SERPINF1	COL3A1	C1S	OGN	DCN	EGR1	ID2	C1R	JUNB	HSPA1B	MMP2	OSR2	ZFP36	NBL1	SPARC	CCN1	CFD
CELLMARKERMSIGDB_FAN_OVARY_CL8_MATURE_CUM…	AMH	TNNI3	INHBB	ATP5IF1	SERPINE2	PPP1R1…	RBP1	HSD17B1	IFI27	PRKAR2B	SPINT2	APOA1	VCAN	HSPG2	LY6E	CD99	IHH	PSMA3	BEX1	GATM	C4orf48	GSTA1	FDXR	LPL
CELLMARKERMSIGDB_FAN_OVARY_CL9_PUTATIVE_A…	TM4SF1	CCN2	ANGPT2	NNMT	TM4SF18	SOD2	CLDN5	HMOX1	TNFSF10	CALCRL	FAM241A	ACKR1	GIMAP7	CD59	ARL4A	EMP1	GNG11	TCF4	ZNF385D	PDLIM1	MYCT1	ETS2	PCAT19	CLEC2B
CELLMARKERMSIGDB_GAO_ESOPHAGUS_25W_C1_CIL…	AARS2	ABCA13	ABCB10	ADAP1	ADGB	ADGRE5	AGBL2	AGBL5	AK8	AK9	AKAP14	ALDH16A1	ALDH1A1	ALDH3B1	ALMS1	ANKMY1	ANKRD18A	ANKRD54	ANKRD66	ANKUB1	AP3M2	APBB2	APCDD1	APH1B
CELLMARKERMSIGDB_GAO_ESOPHAGUS_25W_C2_KRT…	ADAM28	ADM	AGFG2	APOBEC3A	BMAL2	ATP10B	BAHD1	BLNK	CACNB1	CALHM2	YJU2	CDKN2A	CEACAM1	CTSV	CYP2B7P	DUOX2	DUOXA2	DUSP10	DUSP5	EPS8L1	GAN	GBP4	GDPD3	GIT2
CELLMARKERMSIGDB_GAO_ESOPHAGUS_25W_C3_FGF…	ACSF3	ARRDC2	C19orf…	CCDC15	CD52	CD69	CDIP1	CLCA2	CLOCK	CRTC1	CYTIP	DCAKD	DIAPH3	DNAJC4	FABP3	HYCC1	SHLD2	DIPK1B	GAS2L1	GAS7	GPC1	IL10RA	IRGQ	ISYNA1
CELLMARKERMSIGDB_GAO_LARGE_INTESTINE_24W_…	ADAMTS1	ADAMTS2	ADAMTS4	ASPN	AZIN2	BASP1	BOC	C1QTNF3	C1QTNF6	C1R	C1S	C3	C7	CCDC80	CCL2	CERCAM	CLDN11	CLEC11A	COL14A1	COL16A1	COL1A1	COL1A2	COL3A1	COL5A1
CELLMARKERMSIGDB_GAO_LARGE_INTESTINE_24W_…	ANPEP	AOC1	AQP8	C21orf62	CA7	CDHR5	CEACAM7	CLDN23	CNKSR1	CYP2W1	CYP3A5	DGAT1	DPEP1	EFNA2	EPS8L3	GPANK1	GSTA1	GUCA2A	GUCA2B	HTATIP2	IGF2BP2	KCNJ5	LAD1	LPAR5
CELLMARKERMSIGDB_GAO_LARGE_INTESTINE_24W_…	ABCA1	ABCG1	ABI3	ACP5	ACTRT3	ADAP2	ADCY7	ADCY9	ADGRG5	AIF1	AOAH	APOBEC3A	ARHGDIB	ARL4C	ARRB2	ATF5	ATP11A	ATP1B3	ATP6V1…	BCL2A1	BIRC3	BTK	C1QA	C1QB
CELLMARKERMSIGDB_GAO_LARGE_INTESTINE_24W_…	GJC1	MEIS3	PTGES3L	PART1	ALCAM	SGCA	RAB9B																	
CELLMARKERMSIGDB_GAO_LARGE_INTESTINE_24W_…	ACAD10	ACVR1C	ADH1C	ARSL	ATP10B	C16orf…	C6orf136	CDCA7	CFTR	CHMP4C	CHP2	CLDN15	CLDN2	CPA6	DAPK2	DDC	EFNA3	EVPL	F2RL1	FOXD2-AS1	GATA6-A…	GDF15	GJB1	GJB2
CELLMARKERMSIGDB_GAO_LARGE_INTESTINE_24W_…	ANO7	ATP2C2	B3GNT6	BCAS1	CAPN8	CEACAM5	CEACAM6	CHGA	CLDN8	DHRS9	ENTPD8	ERN2	FAM110C	FER1L6	GALNT3	GPA33	HLA-F	IFI27	LGALS9C	MAP7	MLPH	MUC4	MUC5B	OSBPL7
CELLMARKERMSIGDB_GAO_LARGE_INTESTINE_ADUL…	ABAT	ACP3	ADAMTS…	ADCY1	AFF3	AGTR1	AKAP6	ALPK3	AMER3	AMIGO2	ANK2	AOAH	AP3B2	APBB1	APLP1	APOD	ARC	ARHGEF3…	ARNT2	ARVCF	ASPA	ATP6V1…	B3GAT1	BAIAP3
CELLMARKERMSIGDB_GAO_LARGE_INTESTINE_ADUL…	AADACP1	ABCA9	ABCB6	ABCG1	ACSL1	ADAP2	ADGRF2	ADH1A	ADHFE1	AIRE	AKR1D1	ALKBH1	AMER2	ANKDD1B	ANKMY1	ANKS1B	ANTXR1	AOC3	AP3M2	APBB1	APOBEC3A	AQP11	BMAL1	ARRB2
CELLMARKERMSIGDB_GAO_LARGE_INTESTINE_ADUL…	ACBD7	ADAMTS4	ANKRD45	ANTXR1	ARGFX	AZU1	BNC2	C1S	CEACAM…	CLEC14A	DCN	DEFA4	DNAH5	DSC3	FERMT2	FGD5P1	ACTE1P	FYB1	FZD4	GABRB2	GAN	GNRHR2	GRM6	HAND2-AS1
CELLMARKERMSIGDB_GAO_LARGE_INTESTINE_ADUL…	A1BG	ABCB9	ABI3	ACAP1	ADA	ADAM19	ADAM28	ADAM8	ADGRG5	AIM2	ALOX5	ALOX5AP	AMPD1	ANKRD20…	ANKRD28	ANKRD…	ANKRD44	ANXA6	APBB1IP	APOBEC3G	AQP3	ARHGAP…	ARHGAP25	ARHGAP30
CELLMARKERMSIGDB_GAO_LARGE_INTESTINE_ADUL…	A2M	A2M-AS1	A4GALT	ABCA8	ABI3BP	ABLIM3	ACKR3	ACSL4	ACTA2	ADAM19	ADAM28	ADAM33	ADAMDEC1	ADAMTS1	ADAMTS5	ADGRA2	ADGRB3	ADGRF5	ADGRL3	ADH1B	AEBP1	AGT	AICDA	ALDH1A3
CELLMARKERMSIGDB_GAO_SMALL_INTESTINE_24W_…	AADAC	ADH1C	ATP2A1	BOP1	C17orf78	CA3	CAMKV	CASC2	CCL25	CD24	CDK5	CHCHD10	COMMD1	CYP2J2	DMBT1	DNAJC…	DRG2	DUS3L	ENPP4	FCSK	GCNT1	GSTA1	GSTA2	HDDC3
CELLMARKERMSIGDB_GAO_SMALL_INTESTINE_24W_…	ABCC2	ABRACL	ACP5	AFP	AGT	ALDOB	ALPI	ANXA4	AOC1	APOA1	APOA4	APOB	APOC3	AQP1	ASAH1	ATP1B3	C11orf86	C8G	CELP	CFB	CFI	CHST13	CNKSR1	CTSA
CELLMARKERMSIGDB_GAO_SMALL_INTESTINE_24W_…	ABCA4	ACSS2	ATAD5	BCAS1	C8orf44	CCDC160	CENPM	CFHR4	CLCA1	ERN2	ESR1	FAM193B	FAM3B	FCGBP	LINC019…	FUT6	GRPEL2	GSN	GSTCD	HEPACAM2	KLK1	KRT19	GAU1	MAGEF1
CELLMARKERMSIGDB_GAO_SMALL_INTESTINE_24W_…	ABCA7	ADAMTS16	AMER3	BANK1	BCAM	C12orf…	LINC02903	CACNB4	CC2D2A	CCDC146	CHD5	CHM	COL9A3	CSN2	EPHA10	EVC2	EZH1	FADS6	FOXD2	GABRB2	GAD2	GLIS3	GNAO1	H1-10-AS1
CELLMARKERMSIGDB_GAO_SMALL_INTESTINE_24W_…	ACBD4	ACSM1	AKR1D1	AOX1	APCDD1	ARSI	ASB8	BEND4	ARMH4	C1QTNF3	LINC029…	SCP2D1-A…	CHMP1B2P	CPA1	CPZ	DPT	ELANE	FAM162B	FBXL7	GFRA2	HOXC11	LINC02…	ISLR	ITIH4-AS1
CELLMARKERMSIGDB_GAO_STOMACH_24W_C1_PROCR…	ANKRD37	C2CD2	INTS15	CASP3	CDC45	CENPQ	DDX31	DNAL4	DTX3L	FAM229A	GJC2	GPT2	H2AX	KIAA1586	KLHL11	LGSN	LRRC57	MAML2	MTA1	MTOR	NUDT8	PITPNM2	PLD1	PLK4
CELLMARKERMSIGDB_GAO_STOMACH_24W_C2_TFF2P…	SUPT7L	ECE1	PTP4A3	TCIM	PROM1	TBC1D15	UBE2D1																	
CELLMARKERMSIGDB_GAO_STOMACH_24W_C4_PARIE…	CCDC80	ANXA1	CFH	H6PD	FKBP14	KERA	FGD5	HBB																
CELLMARKERMSIGDB_GAO_STOMACH_24W_C5_PUTAT…	ACTC1	ADRA1D	AK4	ALDH1B1	BARX1	BCL11A	BCL2	BMPER	CAPS	CFAP221	CFL2	CNN1	CORO1C	DHX35	DIXDC1	TAFA4	FBXO32	GBP2	HACD1	HSPB8	IL17B	KCNMB1	LAMA5	LDB3
CELLMARKERMSIGDB_GAUTAM_EYE_IRIS_CILIARY_…	ARFIP1	MRPL3	YTHDC1	NOD1	IKBIP	PPME1	USF3	BCL6B	KEAP1	ABHD17A	ATF7	TNFRSF10A	ZMYM6	DHRS4L2	HINFP	PAPOLG	DENND2C	TMOD3	ADARB1	CARS2	TWNK	EIF4EB…	FAM114A2	UIMC1
CELLMARKERMSIGDB_GAUTAM_EYE_IRIS_CILIARY_…	COX7A2L	RERE	KLF10	VTI1B	CSNK1E	JAM3	NLGN1	MAFF	CDK6	S1PR3	SLC36A4	ZDHHC2	MAB21L1	DSC2	SGCB	SLC20…	SETBP1	STX2	RBP1	PLCXD3	MIDEAS	NCEH1	TTC3	COMMD6
CELLMARKERMSIGDB_GAUTAM_EYE_IRIS_CILIARY_…	DLEU1	S1PR4	GRIPAP1	NRROS	NAA50	RPL5	ADGRE5	IRAK2	TLK2	PRXL2C	RACK1	RCOR3	PPTC7	STK10	PCED1B	TRIM4	PKHD1L1	DBF4	HIBCH	RBM38	DMXL1	DOP1B	THUMPD1	CYP20A1
CELLMARKERMSIGDB_GAUTAM_EYE_IRIS_CILIARY_…	SLC39A…	YWHAB	TMEM70	NFIA	NASP	P4HB	SQOR	IFNGR2	CNN3	TSC22D1	GMDS	SERPINF1	RORA	NTRK3	PTP4A2	ABCA9	SSR3	MXRA8	C1RL	CHEK2	LPIN1	TOP1	MYOC	SCARA5
CELLMARKERMSIGDB_GAUTAM_EYE_IRIS_CILIARY_…	TOM1L2	NSD1	SNX4	GPR146	PPP3CC	LIPA	CHRM3	MAP2K7	RANBP17	MOB2	POMT1	NIBAN2	MMP17	G2E3	FPGT	CHORD…	LBR	LIPT1	APOL6	FRY	IZUMO4	HYCC1	STRN3	NRDE2
CELLMARKERMSIGDB_HAY_BONE_MARROW_CD34_POS…	ACY3	NREP	NEGR1	NPTX2	TRH	CRMA	PRSS2	SDK2																
CELLMARKERMSIGDB_HAY_BONE_MARROW_CD34_POS…	GLDC	FABP5	VAT1L	LDHA	CAPSL	BCAT1	KCNK9																	
CELLMARKERMSIGDB_HAY_BONE_MARROW_CD34_POS…	DEPTOR	HACD1	SVOPL	CPB1	FREM1	SPINK4	HYAL3	ABCC4																
CELLMARKERMSIGDB_HAY_BONE_MARROW_CD34_POS…	ITGA2B	ENSG0000…	VWF	LTBP1	SPX	WFDC1	ITPKA	PBX1	PLEK	MMRN1	IGSF10	TPM1	PRTFDC1	GJA4	TGFB1I1	ANXA3	COL2A1	MED12L	GATA2	ST6GAL2	DAD1	NAA38	GUCY1B1	TCEAL9
CELLMARKERMSIGDB_HAY_BONE_MARROW_CD8_T_CE…	CCL5	GZMH	IL32	CD8A	DUSP2	KLRG1	TRGC2	GZMK	LYAR	HLA-B	LINC018…	CD8B	LAG3	PPP2R5C	CD99	RGS1	PTPRC	CXCR4	SH2D1A	JAKMIP1	FYN	PATL2	PTPN7	TIGIT
CELLMARKERMSIGDB_HAY_BONE_MARROW_EARLY_ER…	APOC1	NMU	CNRIP1	GAL	NPM1	CXADR	FAM89A	PNMT	GCSH	C1QBP	NME1	FSCN1	CDH1	PVT1	GRTP1	UNG	MYC	DNPH1	DES	DHRS11	CCDC26	CENPV	RPL37A	SRM
CELLMARKERMSIGDB_HAY_BONE_MARROW_EOSINOPH…	RNASE2	LYZ	LGALS1	RETN	MT-CO2	H1-10	MT-CO3	RPLP1	MT-ND4	MT-ATP6	COX8A	SLC44A1	RNASE2CP	RFLNB	SERPINB…	RAB32	DBI	MT-ND4L	MT-ND1	H2AJ	MT-ND5	MS4A4A	BLOC1S1	NT5DC2
CELLMARKERMSIGDB_HAY_BONE_MARROW_NAIVE_T…	RPS27	RPL30	RPS29	RPS25	RPL21	RPS27A	RPL13	RPL34	RPL31	RPLP2	RPS15A	RPL23A	SARAF	IL7R	RPS14	RPS12	RPL11	RPL13A	CD3E	RPL32	RPL10	LTB	EEF1A1	RPL35A
CELLMARKERMSIGDB_HAY_BONE_MARROW_NEUTROPH…	S100A9	S100A8	S100A12	VCAN	FCN1	S100A6	CSTA	TSPO	CD14	TYROBP	MNDA	S100A4	CTSD	PLBD1	TYMP	FOS	GPX1	ATP5MJ	AP1S2	RBP7	DUSP1	CYBA	QPCT	SRGN
CELLMARKERMSIGDB_HAY_BONE_MARROW_NK_CELLS	GNLY	NKG7	KLRF1	CTSW	PRF1	KLRD1	CD7	CST7	HOPX	GZMA	CD247	SPON2	GZMB	FGFBP2	TRDC	IL2RB	MATK	CLIC3	IFITM2	GZMM	HLA-C	CMC1	KLRB1	S1PR5
CELLMARKERMSIGDB_HAY_BONE_MARROW_PRE_DEND…	CTSV	SAMHD1	AXL	AREG	SPATS2L																			
CELLMARKERMSIGDB_HAY_BONE_MARROW_PRO_B	STMN1	IGLL1	HMGN2	HMGB2	TUBA1B	NUSAP1	PTMA	TUBB	HMGB1	MKI67	FAM111B	AURKB	CD24	TYMS	TOP2A	H2AZ1	SMC4	PDLIM1	H3-3A	MAD2L2	UBB	BIRC5	CARHSP1	H4C3
CELLMARKERMSIGDB_HAY_BONE_MARROW_STROMAL	CXCL12	APOE	CP	LEPR	DCN	PCOLCE	SELENOP	ESM1	VCAM1	RARRES2	GGT5	OLFML3	C1S	PLPP3	LUM	IGF2	C1R	IGFBP5	LHFPL6	TF	NNMT	CHRDL1	BMP5	SERPING1
CELLMARKERMSIGDB_HE_LIM_SUN_FETAL_LUNG_C0…	MYH11	LMOD1	ACTG2	CNN1	ACTC1	TES	CSRP2	LIMS2	SLMAP	CARMN	MYOCD	FHL2	KRT8	IGFBP2	FOXP2	FRMD6…	PDLIM5	PDGFC	TPBG	LINC02381	HMGA2	SYNPO2	MSRB3	SORBS1
CELLMARKERMSIGDB_HE_LIM_SUN_FETAL_LUNG_C0…	SERPIN…	LOX	ENTPD2	RCN3	COL12A1	PDGFRL	ANXA1	CD34	PAMR1	NPDC1	SPON2	IGFBP6	PRELP	CRABP2	TENT5A	PID1	KDELR3	THBS2	C1R	CTSK	ANGPTL1	PDLIM3	C1QTNF3	SH3BP5
CELLMARKERMSIGDB_HE_LIM_SUN_FETAL_LUNG_C0…	HNRNPA…	HMGA1	TYMS	IGF2BP1	LIMD2	NNAT	XIST	LMNB1	HMGA2	HSPH1	MCM7	UQCRHL	CRABP2	SINHCAF	SFRP1	SNCG	SERINC2	H2AC25	MAD2L1	GINS2	KRT8	CKS2	CDCA7	UBE2C
CELLMARKERMSIGDB_HE_LIM_SUN_FETAL_LUNG_C0…	KRT19	KRT8	IGFBP2	KRT18	ALDH1A2	SBSPON	TNNT1	WT1	COL9A3	REC8	SPINT2	UPK3B	EFNA1	WNT2B	LINC023…	MYRF	UCHL1	PODXL	SMTNL2	DMKN	PAK4	NET1	PLP2	RDH10
CELLMARKERMSIGDB_HE_LIM_SUN_FETAL_LUNG_C0…	COL9A2	COL2A1	COL9A3	COL9A1	SOX6	CNMD	WWP2	MATN4	SOX9	FRZB	RARG	COL11A2	S100B	COL11A1	YPEL2	ACAN	MECOM	DOK5	FGFR2	EYA4	THSD4	HAPLN1	SNAI1	CAPN6
CELLMARKERMSIGDB_HE_LIM_SUN_FETAL_LUNG_C0…	CNTNAP2	CFD	PAMR1	ANXA1	CNTN1	ALDH1A1	FMO1	CA3	GGT5															
CELLMARKERMSIGDB_HE_LIM_SUN_FETAL_LUNG_C0…	MSLN	UPK3B	TM4SF1	C3	TFPI2	KRT19	CLIC3	ADIRF	KRT8	SLPI	KLK11	EFEMP1	CFH	C4B	CD9	KRT18	SPOCK2	ALDH1A2	ANXA1	SBSPON	C4A	TMEM17…	ANXA8L1	PTPRF
CELLMARKERMSIGDB_HE_LIM_SUN_FETAL_LUNG_C0…	HMGA2	FBN2	MEIS2	LINC010…	HHIP	HHIP-A…	PITX1	FRZB	FOXP2	MSC	MAPK10	NCAM1	SULF1	IGFBP2	HOXA4	PITX2	AMOTL2	TES	NSG1	FENDRR	PDLIM3	CISD1	TPBG	TWIST2
CELLMARKERMSIGDB_HE_LIM_SUN_FETAL_LUNG_C0…	IGF1	HTRA1	ACTG2	MYH11	MYOCD	HHIP	SEMA3C	ARL4A	ADAMTS…	ARHGAP6	ENC1	PLP1	WFDC1	CEP120	HHIP-AS1	FENDRR	SPEG	KCNMB1	RHCG	SEMA6D	TSPAN2	FHL2	FBXL22	PMEPA1
CELLMARKERMSIGDB_HE_LIM_SUN_FETAL_LUNG_C0…	KRT19	UPK3B	SBSPON	IGFBP2	KRT8	ALDH1A2	MSLN	TNNT1	WNT2B	KRT18	KLK11	FNDC1	SPOCK2	CLIC3	TM4SF1	GAS6	COL18A1	SULF1	SPINT2	CRIP1	EFEMP1	COL9A3	PTGS1	PODXL
CELLMARKERMSIGDB_HE_LIM_SUN_FETAL_LUNG_C0…	PLPPR4	CXCL14	HTRA1	CT45A1	CT45A3	KCNK17	ARHGAP6	ENC1	EYA4	FENDRR	TPST2	STC1	IGSF1	CTXND1	NKD1	SLC4A4	SEMA3C	CT45A2	PTCH1	CT45A10	CT45A8	ETV5	NOTUM	MMGT1
CELLMARKERMSIGDB_HE_LIM_SUN_FETAL_LUNG_C1…	ADIRF	MSLN	ICAM1	UPK3B	ICAM4	SUSD2	FILIP1	CAV2	SLC2A3	ANXA1	CYBRD1	CLIC5	CAVIN1	STOM	COL4A4	NMU	ABCA1	ST6GALN…	ALDH3B1	EHD2	AQP5	PKDCC	MMP28	SFTPD
CELLMARKERMSIGDB_HE_LIM_SUN_FETAL_LUNG_C1…	AGR3	CFH	FMO2	CP	ANXA1	CCNO	STEAP4	SCNN1B	KDR	MET	KLK11	LMO3	IFITM1	KLK10	SPARCL1	ARFGE…	SERPINA1	ERP27	C3	SCGB1A1	CFHR1	RUNX1	ARHGEF38	ST6GALNA…
CELLMARKERMSIGDB_HE_LIM_SUN_FETAL_LUNG_C1…	CYTL1	XIST	CTNND2	ADM	GPC3	ALPL	MOXD1	TMEM47	IGDCC3	PLPP2	TMEM178A	MEG3	MITF	MTTP	CRH	GADD4…	CDH2	HS3ST1	OPRK1	PCDH17	ASCL1	ZMAT4	TBX1	INSIG1
CELLMARKERMSIGDB_HE_LIM_SUN_FETAL_LUNG_C1…	CTNND2	FADS2	MAD2L1	UHRF1	HNRNPA1L3	UBE2T	BIRC5	CENPU	SHISAL…	CDC20	UBE2C	SCD	CENPW	CCNB2	ORC6	VRK1	CENPN	NUSAP1	PGP	CCNA2	TOP2A	CENPF	CLSPN	TMEFF1
CELLMARKERMSIGDB_HE_LIM_SUN_FETAL_LUNG_C1…	CHGA	GHRL	HEPACA…	CFC1B	NEUROD1	SCG3	CFC1	VSTM2L	ACSL1	PROX1	BEX1	ABCC8	SSTR2	APLP1	SEZ6L2	CXXC4	VWA5B2	CHGB	ARFGEF3	CAMK2B	INSM1	NPDC1	KIF1A	MAP1B
CELLMARKERMSIGDB_HE_LIM_SUN_FETAL_LUNG_C1…	FILIP1	ADIRF	ICAM4	MSLN	ST6GALNA…	COL4A3	SUSD3																	
CELLMARKERMSIGDB_HE_LIM_SUN_FETAL_LUNG_C1…	NOTUM	AQP1	CD36	NAPSA	SFTPA1	RASGRF1	TNC	CLDN2	TMEM163	PPP1R1B	CDH16	C10orf90	ADH1C	ASPG	MGLL	LINC0…	LINC013…	ENG	C11orf…	PGC	SHISA2	C16orf…	ACOXL	DLG2
CELLMARKERMSIGDB_HE_LIM_SUN_FETAL_LUNG_C1…	ASCL1	SMOC2	PCP4	CALML3	MYCL	MUC5AC	ABCG1	SULT2B1	SPX	HES6	G0S2	ESPN	SCNN1A	RASD1	FMOD	MAB21…	CDH2	DLL3	NLGN1	CACNA2D1	CPXM2	RPRM	SARDH	CRISPLD2
CELLMARKERMSIGDB_HE_LIM_SUN_FETAL_LUNG_C1…	SLPI	SCGB3A1	CP	MUC16	MEG3	ANXA1	KLK11	KRT7	SPINK5	TIMP1	FAM3D	SCGB1A1	ARFGEF3	EHF	CD82	MUC20	GK	PLPP2	CHST9	S100P	PROM1	SERPIN…	KLK10	RHOV
CELLMARKERMSIGDB_HE_LIM_SUN_FETAL_LUNG_C1…	GRP	ASCL1	HES6	NPDC1	CHGA	SCG3	DPYSL3	DDC	PROX1	DLL3	RPRM	SCG2	GFRA3	MAP1B	MIAT	HEPAC…	KCNH6	RASD1	APLP1	CA8	KLK11	KLK12	MS4A8	SCG5
CELLMARKERMSIGDB_HE_LIM_SUN_FETAL_LUNG_C1…	KRT15	VCAN	TM4SF1	IGFBP4	TPM2	LGALS1	RASSF6	NTRK2	COL4A1	KRT5	MYC	PAX9	DKK3	MEG3	KREMEN2	THY1	HS3ST1	TFAP2A	NBL1	COL14A1	SH3BP5	COL9A1	HLA-B	DST
CELLMARKERMSIGDB_HE_LIM_SUN_FETAL_LUNG_C1…	SLPI	FOXC1	LTF	TM4SF1	FAM3D	KRT7	EHF	SCGB3A1	PROM1	TFAP2A	TIMP1	LHFPL2	CD82	DSC2	ELAPOR1	ANXA1	PLPP2	GRHL1	RUNX3	ALDH1A3	TF	NOXO1	NUPR1	MUC5B
CELLMARKERMSIGDB_HE_LIM_SUN_FETAL_LUNG_C2…	ACP2	PLA2G7	CD82	APOE	GPNMB	BCAP29	LMNA	SLC38A7	VAT1	GPR137B	CD59	IL18	NECTIN2	APOC1	SMPDL3A	PLA2G…	TMEM37	TCEAL3	XAF1	C2	MSR1	SLC7A8	KCNMA1	IFI6
CELLMARKERMSIGDB_HE_LIM_SUN_FETAL_LUNG_C2…	C1orf54	CLEC9A	WDFY4	S100B	RGCC	CLNK	RAB7B	IDO1	DNASE1…	TAP1	CADM1	BATF3	CAMK2D	SLAMF7	GCSAM	VAC14	FLT3	TMEM14A	STK17A	CSRP1	ASB2	HLA-DOB	FKBP1B	ANPEP
CELLMARKERMSIGDB_HE_LIM_SUN_FETAL_LUNG_C2…	CD1C	GPAT3	FCER1A	LGALS2	HLA-DOA	NDRG2	LPXN	ADAM28	VOPP1	PKIB	ARL4C	CD69	S100B	NR4A3	ADAM8	NAPSA	CD1E	MAP4K1	TLR10	IL1R2	P2RY14	PPP1R1…	CYP2S1	IL18BP
CELLMARKERMSIGDB_HE_LIM_SUN_FETAL_LUNG_C2…	S100A8	RNASE2	NRGN	UPK3A	CES1	CD2	MTMR11																	
CELLMARKERMSIGDB_HE_LIM_SUN_FETAL_LUNG_C2…	AZU1	PRTN3	MPO	MS4A3	NUCB2	CLEC11A	RNASE2	RNASE3	CTSG	PRSS57	SERPINB…	NKG7	MLC1	MYB	ELANE	DEFA4	RGL4	TRGC1	DEFB1	SERPINB8	MTARC1	FUT4	C1QTNF4	HERC5
CELLMARKERMSIGDB_HE_LIM_SUN_FETAL_LUNG_C2…	SPINK2	HOPX	PRSS57	SSBP2	PTPRCAP	JUP	CD34	SMIM24	MZB1	NREP	C1QTNF4	IGHM	CD200	ITM2A	CYTL1	KDM5B	CD82	TSC22D1	MSI2	ADGRG1	RBPMS	HEMGN	HOXA9	GNG11
CELLMARKERMSIGDB_HE_LIM_SUN_FETAL_LUNG_C2…	GP9	CAVIN2	GP1BA	CLEC1B	TUBB1	PF4	ITGA2B	PPBP	STOM	RAB27B	LAT	ESAM	GATA1	MYLK	NRGN	PDLIM1	ARHGAP6	ICAM2	CMTM5	THBS1	TMEM40	MPIG6B	TUBA4A	GUCY1B1
CELLMARKERMSIGDB_HE_LIM_SUN_FETAL_LUNG_C2…	S100A12	S100A8	CDA	RBP7	PADI4	SLC11A1	VNN2	S100P	FCGR3B	LILRA5	ORM1	G0S2	MCEMP1	PELATON	LRRK2	BST1	CKAP4	NFE2	CYP1B1	ASGR1	MEGF9	DYSF	QPCT	FOLR3
CELLMARKERMSIGDB_HE_LIM_SUN_FETAL_LUNG_C2…	MPIG6B	RIPOR3	ZNF778	TAL1	H2BC4	H4C8	GRAP2	SOCS2	TSC22D1	HERC1	C2orf88	MEG3	LAT	PIM1	H3C10	CAVIN2	CNST	KEL	DNM3	TUBB1	ANK1	MMD	ARL15	NT5C3A
CELLMARKERMSIGDB_HE_LIM_SUN_FETAL_LUNG_C2…	C12orf…	IL3RA	TNNI2	LTB	BCL11A	CD7	SPIB	APP	TSPAN13	NREP	ITGB7	CYB561A3	BCL7A	RUNX2	SCT	UGCG	DCPS	CXCR3	PPP1R1…	CLEC4C	LILRA4	CBFA2T3	TSTD1	TPM2
CELLMARKERMSIGDB_HE_LIM_SUN_FETAL_LUNG_C2…	AZU1	MPO	PRTN3	RNASE2	NKG7	CLEC11A	MS4A3	RNASE3	NUCB2	MCM7	CTSG	CDT1	CLSPN	CENPM	CITED4	MKI67	UHRF1	S100P	CENPU	RGL4	SERPINB8	NCAPD2	HP	PRSS57
CELLMARKERMSIGDB_HE_LIM_SUN_FETAL_LUNG_C2…	S100A8	VCAN	FCN1	S100A12	RBP7	SLC11A1	CDA	ASGR1	PELATON	PADI4	LRRK2	BST1	NFE2	LILRA5	MCEMP1	MEGF9	HK3	TREM1	S100P	CKAP4	CLEC4E	RNASE2	ASGR2	THBS1
CELLMARKERMSIGDB_HE_LIM_SUN_FETAL_LUNG_C2…	RNASE1	SELENOP	LYVE1	C1QB	LILRB5	NRP1	A2M	AGR2	WWP1	PLTP	CCL2	VSIG4	SLCO2B1	EGFL7	HPGDS	SPP1	CTSL	IGF1	MAN1A1	CD163L1	SIGLEC1	HRH1	CD28	IGFBP4
CELLMARKERMSIGDB_HE_LIM_SUN_FETAL_LUNG_C3…	KITLG	TBX2	S100A4	EDNRB	SLCO2A1	PRX	RGS9	VIPR1	COLEC10	ITGA4	MYLK	APLN	S100A3	ADIRF	LMO7	TNFRS…	DISP1	SPON2	CDH13	HLX	NOS2	SLC12A2	MGLL	ITGA1
CELLMARKERMSIGDB_HE_LIM_SUN_FETAL_LUNG_C3…	KLF1	GYPA	HEMGN	ALAD	MYL4	RHAG	CPOX	GATA1	GYPB	TFRC	UBAC1	TESC	RFESD	ANK1	EPB42	TANGO2	TLCD4	MKI67	NFE2	DHRS13	CENPF	NUSAP1	TFDP1	EPOR
CELLMARKERMSIGDB_HE_LIM_SUN_FETAL_LUNG_C3…	TUBB2B	TPM2	THY1	CD24	CMTM8	HIC1	ANGPTL4	CYP26B1	RAB34	HMGA2	CTSC	NOSTRIN	FBLN1	XIST	MACROH2…	IGF2B…	HAPLN1	CA2	SERINC2	BAALC	SLC16A3	TEAD4	TMEM37	ACOT7
CELLMARKERMSIGDB_HE_LIM_SUN_FETAL_LUNG_C3…	PTPRE	TFF3	DKK3	CCL21	MPP7	SULF2	PROX1	NTS	NUPR1	LGALS3	ARID5B	C4orf48	LTBP4	NFATC1	PLAC9	PGM5	PLPP1	RADIL	SEMA3D	PARD6G	RAB11FI…	KLHL4	FLRT2	HOXD9
CELLMARKERMSIGDB_HE_LIM_SUN_FETAL_LUNG_C3…	CCL21	PTX3	STAB2	PROX1	TFF3	PRSS23	GJA1	RELN	PDPN	MMRN1	NR2F1	LGALS3	IGF1	NUPR1	LAPTM5	TBX1	CTHRC1	LOX	PGM5	UBTD1	FAM174B	MRC1	LYN	CYTL1
CELLMARKERMSIGDB_HE_LIM_SUN_FETAL_LUNG_C3…	KIT	POSTN	SCARB1	ADM	NEURL1B	LRATD1	LEPR	ADGRL2	CLEC1A	JAM3	EGLN1	LONRF1	TMEM37	PLAT	APCDD1	MCAM	NENF	HLX	SLC35F2	GLCE	GPX3	DTL	DACH1	FCN3
CELLMARKERMSIGDB_HE_LIM_SUN_FETAL_LUNG_C3…	COL1A2	SULF1	MGP	FBLN5	LTBP4	GJA5	CFH	FBLN2	COL3A1	SERPINE2	SULT1E1	SYNPO	PTGIS	DKK2	CXCL12	COL5A1	CPE	NR4A1	OMD	IL11RA	PLAC9	HMCN1	RSPO3	ITPR2
CELLMARKERMSIGDB_HE_LIM_SUN_FETAL_LUNG_C3…	SBSPON	NFATC1	TCTN3	RADIL	C4orf48	PLAC9	TFF3	PROX1	PTPRE	PLPP1	DKK3	GDF10	DPP4	PDE1A	COL9A3	NTS	LGALS3	SEMA3D	ARL15	PELI2	IER3	GSTM3	ARID5B	PDE4D
CELLMARKERMSIGDB_HE_LIM_SUN_FETAL_LUNG_C3…	SLCO2A1	MGP	CPE	ACKR1	LTC4S	PLVAP	ENTPD1	MMP28	HDAC9	PTGDS	FBN1	SELP	ACKR3	SRGN	VEGFC	LTBP3	SERPINE2	HMCN1	ADGRG6	NUAK1	DUSP23	LHX6	MALL	TLL1
CELLMARKERMSIGDB_HE_LIM_SUN_FETAL_LUNG_C4…	TNFRSF9	CRTAM	SLAMF7	MAP1LC3A	TNFRSF18	GFOD1	FCRL3	RNF19A	TRAF1	RAMP1	IRF8	TSPAN17	STARD4	APLP2	LIM2	MIR15…	NME1	PRR5L	HYOU1	PIM3	BCL2L1	MRTO4	PHLDA1	FABP5
CELLMARKERMSIGDB_HE_LIM_SUN_FETAL_LUNG_C4…	CCR7	LRRN3	CD40LG	ACTN1	IL6ST	CD5	CD6	MAL	SUSD3	TRAT1	CHI3L2	CD4	SIT1	RCAN3	LIMS2	INPP4B	ARMH1	BACH2	SERINC5	TMEM204	GPR183	FCGRT	SOCS3	FHIT
CELLMARKERMSIGDB_HE_LIM_SUN_FETAL_LUNG_C4…	GZMK	TOX2	CCL3L1	CXCR6	KRT86	PDLIM1																		
CELLMARKERMSIGDB_HE_LIM_SUN_FETAL_LUNG_C4…	CD8B	CCR7	ARMH1	LINC024…	LRRN3	ACTN1	PASK	CHI3L2	SIRPG	SPINT2	NELL2	LIMS2	CD5	CPNE2	LDLRAP1	A1BG	FBLN2	STMN3	PECAM1	CD248	S100B	SIT1	THEMIS	YBX3
CELLMARKERMSIGDB_HE_LIM_SUN_FETAL_LUNG_C4…	TYMS	MKI67	CDK1	TOP2A	ASF1B	CENPM	NUSAP1	BIRC5	UBE2C	KIFC1	TMEM106C	CENPU	CDCA5	TPX2	KIF22	ZWINT	UBE2T	TK1	CKS1B	MCM7	USP1	CENPF	RRM2	RAD51AP1
CELLMARKERMSIGDB_HE_LIM_SUN_FETAL_LUNG_C4…	TOP2A	MKI67	NUSAP1	UBE2C	CENPF	TPX2	TYMS	SMC2	UBE2T	KIFC1	EZH2	BIRC5	CKS1B	H2AC14	CDK1	LMNB1	AURKB	ASPM	ASF1B	CDT1	TACC3	GTSE1	CENPK	CENPM
CELLMARKERMSIGDB_HE_LIM_SUN_FETAL_LUNG_C4…	CCR6	IL4I1	LST1	RORC	JAML	IL23R	KIT	PRNP	CA2	SLC16A3	SLAMF1	SCART1	GPR25	MAEA	ADAM12	AFF3	REEP3	MAGED1	PHACTR2	TNFSF13B	CAPG	TTC39C…	DPP4	CD82

Table S5: Cell state marker gene list

CELLMARKERCAN_ACINAR_CELLS		CTRB1	KLK1	RBPJL	PTF1A	CELA3A	PRSS1	SPINK1	ZG16	CEL	CELA2A	CPB1	CELA1	RNASE1	AMY2B	CPA2	CPA1	CELA3B	PNLIP	CTRB2	PLA2G1B	PRSS2	CLPS	REG1A	SYCN
CELLMARKERMSIGDB_HE_LIM_SUN_FETAL_LUNG_C4…		IL23R	SLC4A10	TRDV2	CXCR6	RORC	PHACTR2	BLK	IGFBP4	CCR6															
CELLMARKERMSIGDB_HE_LIM_SUN_FETAL_LUNG_C4…		CD1E	ARPP21	CD1B	MZB1	RAG1	CCR9	DAPK1	CD4	SSBP2	RAG2	GSE1	ELOVL4	AEBP1	MIR181A…	MTRFR	EDEM1	PITPNM2	CD8B	LDLRAD4	CD1A	CD1C	TSHR	ITPR2	ARHGEF7
CELLMARKERMSIGDB_HE_LIM_SUN_FETAL_LUNG_C4…		CTLA4	FOXP3	SIRPG	CD5	CD4	SIT1	CD28	NCF4	RTKN2	TBC1D4	IL2RA	ICOS	DUSP4	BIRC3	PELI1	SLAMF1	TRAF1	RGPD2	RGPD1	SESN3	PERP	CCR4	PTMS	TNFRSF4
CELLMARKERMSIGDB_HE_LIM_SUN_FETAL_LUNG_C5…		SLC2A3	LINC00926	CD83	SELL	NR4A1	GPR183	TRBC2	SP140	CD1C	DUSP2	NR4A2	BIN1	CXCR5	BIRC3	LY6E	ATP2B1	FCER2	LY9	HLA-DOB	MARCHF1	CD44	AHNAK	ARHGAP24	CCR7
CELLMARKERMSIGDB_HE_LIM_SUN_FETAL_LUNG_C5…		ARHGAP…	RGS10	DNPH1	S100A4	BCL2A1	CD44	VOPP1	TNFRSF1…	RHOF	RHEX	CLECL1P	APOBEC3G	RASA4	S100A10	CCND2	BCL2	SH3BP1	FCRL2	CD82	TRAC	MYO1E	CTSH	PLEK	NFKBIE
CELLMARKERMSIGDB_HE_LIM_SUN_FETAL_LUNG_C5…		ACSM3	AMN	DEPP1	BMP3	CEBPD	LINC00…																		
CELLMARKERMSIGDB_HE_LIM_SUN_FETAL_LUNG_C5…		DNTT	SOCS2	RAG1	LINC010…	EGFL7	LINC00…	GBP4	SMIM3	ERG	TRGV9	ELK3	ADA	ENG	GNG11	FHIT	SLC8A…	H1-0	CD34	SORBS3	SCHIP1	SH2D4B	PECAM1	TNFRSF1A	C1QTNF4
CELLMARKERMSIGDB_HE_LIM_SUN_FETAL_LUNG_C5…		SOCS2	DNTT	UHRF1	TYMS	CDT1	ZWINT	ADA	MKI67	SMC2	AIF1	MCM3	CDCA7	MAD2L1	CENPU	TMEM106C	CBX5	CENPM	DNAJC9	NUSAP1	TOP2A	RRM1	UBE2T	KIFC1	ASF1B
CELLMARKERMSIGDB_HE_LIM_SUN_FETAL_LUNG_C6…		MUC16	PRR15	PLEKHS1	CXCL17	ANKUB1	MB	VCAN	SCPEP1	MUC20	MUC4	TNNT3	SLC23A1	FYB2	PALLD	POU2AF1	PLAAT3	CCN2	EPPIN	UACA	GBP6	COL28A1	MAOB	ITGA2	TXLNB
CELLMARKERMSIGDB_HE_LIM_SUN_FETAL_LUNG_C7…		COL20A1	ALDH1A1	IGFBP7	ANXA1	IFI16	PDLIM1	BGN	ITM2A	ACTC1	TMEM176A	DOCK8	PLAC9	PDGFA	TMEM176B	ITGA1	IL11RA	CD44	KLF4	USP53	S100A4	FST	CYTL1	FBN1	P3H2
CELLMARKERMSIGDB_HE_LIM_SUN_FETAL_LUNG_C7…		RNASE1	SELENOP	NFIX	ADM	SCGB3A2	FGFBP2	RAPSN	RIMS3	METTL7A	BCL11A	EPAS1	DOK4	VIP	DGKB	DDC	SYN2	CCSER1	VWDE	PLCD4	AQP1	VEGFA	ERV3-1	MFAP4	PLAGL2
CELLMARKERMSIGDB_HE_LIM_SUN_FETAL_LUNG_C7…		TMEM17…	TMEM176B	OLFML2A	EGFL8	AHNAK	S100A11	ENTPD2	IFITM2	COL4A1	ALDH1A1	RPS4Y1	LAMB1	SCN7A	FBLN2	TSPAN11	GFRA3	MATN2	RXRG	IGFBP7	FST	LGALS3	HSPG2	EMP3	COL5A3
CELLMARKERMSIGDB_HE_LIM_SUN_FETAL_LUNG_C7…		GATA3	ISL1	TMOD1	PENK	FAIM2	PCP4	PLPP4	ARHGEF28	NSG2	ELAVL2	ACHE	GNAO1	ATP6V1G2	LY6H	SCN3B	SLC8A1	RUNDC3B	SYBU	TMEM130	SULT4A1	SST	SNCG	PRSS3	RGS4
CELLMARKERMSIGDB_HE_LIM_SUN_FETAL_LUNG_C7…		PENK	TMOD1	SLC18A3	PLPPR5	LY6H	CNTN1	SCN3B	SLC8A1	SLC5A7	FAIM2	TTC9B	SYT4	PPP1R1A	CELF4	NSG2	NRCAM	PPP2R2B	SST	ACHE	ISL1	ENTPD3	CDKN2D	RAB6B	ASTN2
CELLMARKERMSIGDB_HE_LIM_SUN_FETAL_LUNG_C7…		TAC3	NRSN1	SLC5A7	TM4SF4	AKAP6	GPR22	NDUFA4L2	MAB21L2	CHODL	PHYHIPL	CXADR	NXPH4	POU2F2	PPP1R1A	ARHGEF28	MYT1	CDK5R2	CNTNAP2	IGLON5	TSHZ3	ST7	OLFM3	CPT1C	IGFBPL1
CELLMARKERMSIGDB_HU_FETAL_RETINA_AMACRINE		TFAP2B	TFAP2A	GAD2	LHX9	MIR181A1…	MEIS2	RUNX1T1	NRXN3	ZNF385D	FBXW7	CELF4	NSG2	MGARP	FAT3	PPFIA2	GRIA4	MYT1L	NRXN1	DLG2	RGS8	RND3	NRP1	CABP1	SRRM4
CELLMARKERMSIGDB_HU_FETAL_RETINA_BIPOLAR		VSX1	GRM6	PRDM8	CADPS	NEUROD4	CA10	VSX2	FSTL5	CHN2	AANAT	PCP2	LRTM1	RIMS2	SLC1A7	RCVRN	PLXDC1	GNB3	TMEM176B	WIF1	SLC17A7	ST18	TMEM215	KCNMA1	NETO1
CELLMARKERMSIGDB_HU_FETAL_RETINA_FIBROBLA…		COL3A1	COL1A1	FOXC1	PCOLCE	PITX2	COL1A2	LGALS1	S100A11	GMDS	BGN	LAMA4	CPED1	PCDH18	EDNRA	SULT1E1	TGFBI	DCN	HBE1	RAMP2	FN1	SCUBE1	FBN2	OLFML3	CXCL12
CELLMARKERMSIGDB_HU_FETAL_RETINA_HORIZONT…		ONECUT2	ONECUT1	TFAP2B	ONECUT3	PROX1	PLXNA2	CACNG3	SULF2	PTF1A	CNTNAP2	TFAP2A	NSG2	STMN2	MIR124-…	RORB	ADD2	NTRK1	MAGI1	MIR124…	STMN4	SYT4	PTPRF	INA	NDRG4
CELLMARKERMSIGDB_HU_FETAL_RETINA_MICROGLIA		AIF1	TYROBP	LAPTM5	CD74	CX3CR1	FCER1G	C1QA	C3	SRGN	RGS1	C1QB	MEF2C	ARHGDIB	ITGB2	RNASET2	HLA-D…	C1QC	ALOX5AP	LST1	CSF2RA	CSF1R	CD53	SLCO2B1	MED12L
CELLMARKERMSIGDB_HU_FETAL_RETINA_MULLER		WIF1	TF	PLP1	ATP1A2	SLC1A3	PRSS35	ANGPTL1	GPM6B	FABP7	LGI4	SPP1	SPON1	SEL1L3	PMEPA1	SOX2	CRYM	DKK3	HES1	MYO10	TTYH1	PTPRZ1	MIR9-1…	LINC00461	CCND1
CELLMARKERMSIGDB_HU_FETAL_RETINA_PHOTOREC…		PDC	AIPL1	RCVRN	NRL	NR2E3	GNB3	ROM1	RP1	NEUROD1	GNAT1	CRX	KCNV2	SLC1A7	GADD45G	CNGB1	TMEM…	MPP4	GNGT2	AMER2	ANKRD33B	SLC38A5	MRLN	RAX2	BTBD8
CELLMARKERMSIGDB_HU_FETAL_RETINA_RGC		PRPH	ELAVL4	SNCG	ISL1	GAP43	NRN1	OLFM1	CNTN2	EBF1	NSG1	SYT4	NHLH2	EBF3	POU4F2	POU6F2	NEFL	NEFM	DCX	RTN1	S100A10	SST	INA	TUBB2A	CHRNB3
CELLMARKERMSIGDB_LAKE_ADULT_KIDNEY_C1_EPI…		SPATA22	PAMR1	CMC2	CSMD2	TAOK1	NHS	NRG3	PTH1R	DLGAP1	TRABD2B	SLC13A3	PBX1	LRMDA	PTPRD	LRP2	MACRO…	GMDS-DT	MAST4	CALD1	MEIS2	FARP1	EPB41L3	CPQ	GPX3
CELLMARKERMSIGDB_LAKE_ADULT_KIDNEY_C10_TH…		SPP1	SERPINA1	SOD2	TCIM	ADAMTS1	G0S2	ITGB6	MT2A	EMP1	LDHA	GADD45B	BTG1	S100A10	GOLM1	S100A6	CIB1	ENO1	IFITM3	CD59	TXNIP	SLC4A7	DUSP23	SLPI	SAT1
CELLMARKERMSIGDB_LAKE_ADULT_KIDNEY_C12_TH…		UMOD	SLC12A1	PP7080	KNG1	MFSD4A	CLCNKA	PLAU	PPP1R1A	HSPA2	THY1	KCNJ1	EGF	CLCNKB	CKB	PRDM16-…	CST3	ATP1B1	RAP1GAP	CYFIP2	MTATP6P1	TMEM72	DEFB1	TSPAN33	COX5B
CELLMARKERMSIGDB_LAKE_ADULT_KIDNEY_C18_CO…		AQP2	AQP3	FXYD4	ST6GAL1	KRT19	HSD11B2	ID1	TMSB4X	PAPPA	WFDC2	TSPAN1	TGFBR1	RASD1	PGGHG	SLC38A1	MMP7	RALBP1	PRDX1	IGFBP3	PDK4	BTG2	MNS1	GSTP1	CTSH
CELLMARKERMSIGDB_LAKE_ADULT_KIDNEY_C19_CO…		CALCA	TMEM213	KIT	ATP6V0D2	FOXI1	STAP1	ERP27	DMRT2	IL18	SLC26A7	LINC011…	ADGRF1	HSP90AA1	ACAT1	MARCHF1	ATP6V…	HSPD1	PTGR1	FTL	LITAF	LGALS3	C12orf…	B2M	CRYAB
CELLMARKERMSIGDB_LAKE_ADULT_KIDNEY_C25_EN…		MMRN1	SEMA3A	TSHZ2	PROX1	LDB2	ST6GAL…	RALGAPA2	TCF4	KALRN	PLCB4	TFPI	FBXL7	ASAP1	EFNA5	STOX2	PDE7B	DOCK5	UTRN	PPP3CA	TPM4	DOCK9	EPB41L…	CPNE8	ARL15
CELLMARKERMSIGDB_LAKE_ADULT_KIDNEY_C28_IN…		C7	MGP	TSHZ2	A2M	LAMA2	TNC	DCN	RBMS3	CALD1	NEGR1	CCN1	THBS1	MEIS1	MEIS2	SLIT3	EBF1	ITGA1	ZEB2	LHFPL6	PALLD	AKAP12	CACNA1C	TIMP3	COL4A1
CELLMARKERMSIGDB_LAKE_ADULT_KIDNEY_C29_UN…		CFH	LINC01435	MGAT4C	ALDH1A2	ZFPM2	KCNT2	RBFOX1	NRG3	ENTREP2	CTTNBP2	FRMD4A	FBXL7	TSHZ2	EDIL3	TENM3	CCN2	SYNE1	PDE1A	PLCB4	SLIT3	RERG	CASC15	ITGB8	ACSM3
CELLMARKERMSIGDB_LAKE_ADULT_KIDNEY_C5_PRO…		GRIN2B	DRD3	PRSS51	RNA5SP4…	RRH	KLHDC8A	ADAMTS8	LINC006…	ANK1	LINC012…	DNAH2	BTNL10P	GACAT3	CSMD2	GNGT2	GABBR2	LINC004…	IL36B	LINGO1	TEX36	CDON	FRMD6-…	TMEM214	SLC26A3
CELLMARKERMSIGDB_MANNO_MIDBRAIN_NEUROTYPE…		ACTB	ACTG1	ATF4	ATP5F1B	ATP5F1E	ATP5PB	ATP5ME	ATP5MG	ATP5PO	BRK1	BZW1	ATP5MJ	FMC1-LU…	CFL1	CHCHD2	COX4I1	COX6B1	COX6C	DDX5	DYNLL1	EEF1A1	EEF1G	EEF2	EIF4A2
CELLMARKERMSIGDB_MANNO_MIDBRAIN_NEUROTYPE…		ACOT7	ACSL4	ACSL5	ACTL6B	ACVR1B	ADARB2	ADCY2	ADCY8	AJAP1	AKAP1	AKAP12	AKR1C1	AKR1C2	AMPH	ANK1	ANK2	ANK3	ANKRD12	ANKRD1…	ANKRD36B	ANKRD55	AP1S2	APLP1	APOL6
CELLMARKERMSIGDB_MANNO_MIDBRAIN_NEUROTYPE…		ABLIM3	ACSL4	ACSL6	ACTL6B	ACVR2A	ADAM2	ADAM22	ADARB2	ADCY1	ADCY2	ADCY8	AJAP1	AKAP9	AKR1C1	AKR1C2	AMER2	AMPH	ANK1	ANK2	ANK3	ANKRD36B	ANKRD44	AP1S2	APBA1
CELLMARKERMSIGDB_MANNO_MIDBRAIN_NEUROTYPE…		AATK	ABCA3	ABCC8	ABLIM3	ACOT7	ACTL6B	ADARB2	AJAP1	AK5	AKR1C1	AKR1C2	ALDH1A1	ALX4	AMER3	AMN1	ANK3	ANKH	ANKRD44	APBA1	APLP1	ARG2	ARHGAP…	ARHGEF3	ASB4
CELLMARKERMSIGDB_MANNO_MIDBRAIN_NEUROTYPE…		A2M	AAMDC	ABCA1	ABCB1	ABCB4	ABCG1	ABCG2	ABHD17A	ABLIM1	ABLIM3	ACE	ACOT9	ACSL5	ACSS1	ACTN1	ACTN4	ACVR1	ACVRL1	ADAM1A	ADAM15	ADAMTS1	ADAMTS…	ADAMTSL4…	ADCY4
CELLMARKERMSIGDB_MANNO_MIDBRAIN_NEUROTYPE…		A2M	ABCG1	ABI3	ACSF2	ACSL5	ACY3	ADAM28	ADAMTSL…	ADAP2	ADAT2	ADCK2	ADCY7	ADORA3	ADPGK	AFF1	AGR2	ALOX5	ALOX5AP	ANKRD44	ANXA11	ANXA4	ANXA5	AP1B1	APBB1IP
CELLMARKERMSIGDB_MANNO_MIDBRAIN_NEUROTYPE…		AATK	ABLIM3	ACSL4	ACSL6	ACVR1B	ADAM23	ADAMTS5	ADCK2	ADCY1	ADCY2	ADORA2A…	GRK3	AHDC1	AJAP1	AK5	AKAP1	AKAP7	AKAP9	AKR1C1	AKR1C2	AMPH	ANK1	ANK2	ANK3
CELLMARKERMSIGDB_MANNO_MIDBRAIN_NEUROTYPE…		ACTL6B	ACVR1B	ACVR2B	AKR1C1	AKR1C2	AMER2	ANK2	ANK3	AP1S2	ARPP21	ASIC4	ASNS	ASXL3	ATCAY	ATL1	ATP11C	ATP1A3	BASP1	BCL11A	BIN1	BRSK2	BZW2	C1orf35	C1QL1
CELLMARKERMSIGDB_MANNO_MIDBRAIN_NEUROTYPE…		AASDHP…	AATK	ABLIM3	ACTL6B	ACVR1B	ACVR2A	ADARB2	ADAT2	ADD2	AFF2	AJAP1	AKAP1	AKR1C1	AMER2	AMER3	AMPH	ANK2	ANK3	ANKIB1	ANKRD13B	ANKRD44	ANKS1B	APBA2	APC
CELLMARKERMSIGDB_MANNO_MIDBRAIN_NEUROTYPE…		AATK	ABCA3	ABCC8	ABLIM1	ACOT7	ACSL4	ACTL6B	ACVR1B	ADAM22	ADCY1	ADCY2	AHNAK2	AKR1C1	AKR1C2	ALCAM	ANK2	ANK3	APC2	APLP1	ARG2	ASNS	ATCAY	ATL1	ATP11C
CELLMARKERMSIGDB_MANNO_MIDBRAIN_NEUROTYPE…		ACSS1	ADAM22	ADAMTS3	ADAMTS6	ADCYAP1R1	AFAP1L2	AFF1	AGA	AIF1L	AK4	AKAP7	ANGPTL2	ANKRD44	ANKS1B	APOD	APOL6	ARC	ARHGAP1…	CHN1	ARHGAP23	ARHGAP42	ARL8A	ASCL1	ASTN1
CELLMARKERMSIGDB_MANNO_MIDBRAIN_NEUROTYPE…		A2M	AAMDC	ABCA1	ABCA9	ABCC9	ABHD17A	ABHD2	ACOT9	ACTA2	ACTN1	ADA	ADAM12	ADAM9	ADAMTS1	ADAMTS18	ADAMT…	ADAP2	ADCY4	ADORA2B	GRK3	AFAP1L2	AGAP2-…	AGRN	AKAP12
CELLMARKERMSIGDB_MANNO_MIDBRAIN_NEUROTYPE…		AAMDC	ACTL6A	AJUBA	ANLN	ANP32E	ANTXR1	NAXE	ARHGAP1…	ARHGEF…	ARL4A	ASB2	ASCL1	ASPM	ATAD2	AURKA	AURKB	BCAT1	BDH2	BIRC5	BOC	BRCA1	BRCA2	BUB1	BUB1B
CELLMARKERMSIGDB_MANNO_MIDBRAIN_NEUROTYPE…		AASS	ACVR2B	ADAMTS6	AJUBA	AK4	SLF1	ANLN	AP5M1	NAXE	ARHGEF26	ARL4A	ARX	ASPM	ATAD2	ATAD5	AURKA	AURKB	B3GNT5	BARD1	BDH2	BIRC5	BMP7	BRCA1	BRCA2
CELLMARKERMSIGDB_MANNO_MIDBRAIN_NEUROTYPE…		ACTL6A	ADAMTS5	ADAMTS6	AJUBA	AK4	ALDH6A1	SLF1	ANLN	ANP32E	ANTXR1	AP5M1	ARHGAP11A	ARHGEF26	ARL4A	ARX	ASPM	ATAD2	PGGHG	AURKA	AURKB	BIRC5	BMP4	BMP7	BRCA1
CELLMARKERMSIGDB_MANNO_MIDBRAIN_NEUROTYPE…		ABCA8	ACTL6A	ACVR1	ADSS2	AGO1	AJUBA	ALCAM	SLF1	ANXA5	APOA1	AQP4	ARAP2	ARHGAP24	ARHGAP5…	ARHGEF26	ARHGE…	ARL4A	ARMC3	ARSJ	ARX	AURKB	BDH2	BIRC5	BMP1
CELLMARKERMSIGDB_MANNO_MIDBRAIN_NEUROTYPE…		AASS	ACSS1	ADAMTS6	AFF1	AHNAK	ANTXR1	APCDD1	AQP4	ARHGAP5	ARHGAP5…	ARHGEF26	ARHGEF26…	ARMC3	ARX	ASIC3	ATP2B2	ATXN1	B3GLCT	B3GNT5	BCAN	BCO2	BLVRB	BMP6	BMP7
CELLMARKERMSIGDB_MANNO_MIDBRAIN_NEUROTYPE…		AASS	ABAT	ABCA5	ABHD2	ACSS1	ACTRT3	ADAM33	ADAMTSL…	ADCYAP…	ADIRF	AFF2	AHNAK	AIF1L	AK4	AKAP6	ALDH6…	ALDH9A1	ALDOC	ANTXR1	ANXA5	ANXA6	APC	AQP4	ARAP2
CELLMARKERMSIGDB_MANNO_MIDBRAIN_NEUROTYPE…		AAMDC	ABAT	ACBD7	ACSL6	ACSS1	ADAMTS6	ADAMTSL4…	ADCYAP1…	AFF2	AHNAK	AIF1L	AK4	AKAP7	ALDH6A1	AQP4	ARAP2	ARHGEF26	ARHGEF4	ARHGEF6	ASCL1	ASIC3	ATP13A4	ATP1A2	ATP1B1
CELLMARKERMSIGDB_MANNO_MIDBRAIN_NEUROTYPE…		ABHD17A	ACBD7	ACSL6	ACSS1	ADAMTS3	ADAMTS6	ADCYAP1R1	AFF1	ALDH9A1	ANXA5	APBA1	AQP4	ARAP2	ARC	ARHGAP42	ARHGE…	ARHGEF6	ATP13A4	ATP1B1	ATP1B2	ATP2B2	ADGRB1	ADGRB3	BCAN
CELLMARKERMSIGDB_MANNO_MIDBRAIN_NEUROTYPE…		ABCA8	ACSS1	ACVR1	ADAM9	ADAMTSL4…	ADCY2	ADCYAP1R1	AFF2	AGA	AGBL2	AGT	AHNAK	ALCAM	ALDH6A1	ALDH9A1	ANKRD9	ANTXR1	ANXA1	ANXA2	ANXA5	ANXA6	APLP2	APPL2	AQP4
CELLMARKERMSIGDB_MANNO_MIDBRAIN_NEUROTYPE…		ACSL6	ACTL6B	ACVR1B	ACVR2A	ADAMTS5	ADARB2	AKAP6	AKR1C1	AKR1C2	ALDH1L2	AMER2	ANK2	ANK3	ANKRD44	AP1S2	APLP1	ARRB1	ASIC4	ASXL3	ATCAY	ATL1	ATP1A3	ATP6V1H	AUTS2
CELLMARKERMSIGDB_MANNO_MIDBRAIN_NEUROTYPE…		AATK	ABHD17A	ABLIM1	ABR	ACOT7	ACSL6	ACTL6B	ADAM22	ADCY1	AFF2	AK5	AKAP7	AKR1C1	AKR1C2	ANK2	ANKRD…	ANO8	AP3B2	APBA1	APLP1	ARRB1	ASB2	ASXL3	ATCAY
CELLMARKERMSIGDB_MENON_FETAL_KIDNEY_0_CAP…		H19	ITGA8	IGF2	XIST	LYPD1	NR2F1	NASP	CCDC88A	NNAT	PTMAP5	PTMA	NCAM1	NCL	NR2F2	LAMA4	NTRK2	HMGB1P6	NAP1L1	MEIS2	PSIP1	PTPN14	CENPF	RBP1	DEK
CELLMARKERMSIGDB_MENON_FETAL_KIDNEY_10_IM…		LST1	HLA-DPA1	SRGN	MNDA	CORO1A	LCP1	HLA-DRB1	HLA-DRA	CYBB	TYROBP	PTPRC	IGSF6	LAPTM5	HLA-DRB6	HLA-DPB1	SAMHD1	FYB1	ARHGDIB	CD74	AIF1	HLA-B	S100A4	ARPC1B	B2M
CELLMARKERMSIGDB_MENON_FETAL_KIDNEY_2_NEP…		LIMCH1	CD24	POU3F3	MECOM	PLEKHA1	UTRN	RPS2	CCDC198	DCDC2	EMX2	HOXB3	HSP90AA1	ERBB4	RPLP0	CCND1	LHX1	HOXB7	MST1L	RPSAP58	RPL7P1	HES1	EPCAM	RPL3	HMGA1
CELLMARKERMSIGDB_MENON_FETAL_KIDNEY_3_STR…		COL1A2	COL3A1	COL1A1	COL21A1	TNC	COL6A3	LGALS1	SULT1E1	PTN	CALD1	TPM2	POSTN	CLDN11	H19	AKAP12	ZEB2	CYP1B1	EMCN	IGF2	COL5A2	FN1	FLRT2	MYH10	TMSB4X
CELLMARKERMSIGDB_MENON_FETAL_KIDNEY_6_COL…		GATA3	ELF5	AGR2	ADH1C	BCAT1	SCIN	STC1	TBX3	ALDH1A1	RDH10	CLU	BTG2	CTSH	CLDN4	AHI1	MYO6	WFDC2	KRT8	KRT19	TMSB4X	GSTM3	EMX2	EPCAM	RALBP1
CELLMARKERMSIGDB_MURARO_PANCREAS_ACINAR_C…		PNLIP	REG1B	PRSS1	ALB	PRSS3P2	CPA2	CTRB2	CEL	PLA2G1B	CELA3A	CPA1	REG1A	PNLIPRP2	CTRB1	CPB1	REG3A	SPINK1	GSTA2	ALDOB	CELA2A	CLPS	CTRC	AMY2A	GP2
CELLMARKERMSIGDB_MURARO_PANCREAS_ALPHA_CE…		PRKAR1A	CD63	LUC7L3	KCNQ1OT1	ACLY	ATP5MG	OAZ1	TUBA1A	TMEM59	RAP1GAP2	MAB21L3	KDELR1	CDC42EP3	SNU13	SERF2	ATRAID	CAPZB	COPZ1	HSD17B…	UQCRQ	SNHG6	PNMA2	ATP5MF	ESYT1
CELLMARKERMSIGDB_MURARO_PANCREAS_BETA_CELL		INS	IAPP	MAFA	NPTX2	DLK1	ADCYAP1	PFKFB2	PDX1	TGFBR3	SYT13	HADH	MEG3	SCD5	CDKN1C	SORL1	ERO1B	ABCC8	SCD	UCHL1	SCGN	G6PC2	SRXN1	VEGFA	RBP4
CELLMARKERMSIGDB_MURARO_PANCREAS_ENDOTHEL…		FLT1	KDR	CD93	ESAM	SOX18	PECAM1	ESM1	PASK	SLCO2A1	PLVAP	ADGRL4	RGCC	PODXL	COL13A1	PLPP3	THBS1	SELE	CALCRL	EXOC3L2	PDGFB	ACVRL1	HTRA1	ANGPT2	SMAD6
CELLMARKERMSIGDB_MURARO_PANCREAS_EPSILON_…		GHRL	ANXA13	PHGR1	ACSL1	FRZB	SPTSSB	ASGR1	HEPACAM2	VTN	SERPINA1	SUCNR1	FGF14	TM4SF5	LINC002…	TMEM45B	SERPI…	EBF1	FAXDC2	AGT	NEDD9	DEFB1	DIRAS3	BMP7	SEMA3E
CELLMARKERMSIGDB_MURARO_PANCREAS_MESENCHY…		COL1A1	COL1A2	COL3A1	COL6A3	FN1	SFRP2	COL5A1	SPARC	SERPIN…	COL15A1	COL5A2	COL6A1	PDGFRB	LUM	COL4A1	COL6A2	BGN	VCAN	CYGB	THBS2	COL5A3	PXDN	COL4A2	IGFBP5
CELLMARKERMSIGDB_RUBENSTEIN_SKELETAL_MUSC…		S100A8	LYZ	AIF1	S100A9	LST1	FCN1	CLEC7A	SERPINA1	IGSF6	CSTA	MS4A6A	IL1B	S100A12	HCK	SPI1	CFP	IFI30	MS4A7	MPEG1	LILRA5	C5AR1	RGS18	FPR1	CSF2RA
CELLMARKERMSIGDB_RUBENSTEIN_SKELETAL_MUSC…		APOC1	APOE	MYF5	TRDN	MYF6	MEST	MT1X	TUBB2B	MEG3	CHRNA1	SPATS2L	FOSB	STAC3	FOS	PAX7	CXCL14	CRYAB	CSRP2	EGR1	IGFBP5	DLK1	RPL7	JUNB	JUN
CELLMARKERMSIGDB_RUBENSTEIN_SKELETAL_MUSC…		RERGL	MYH11	PLN	CASQ2	ACTG2	PTN	PHLDA2	ENTPD3	LBH	RCAN2	TPM2	ACTA2	MTHFD2	CNN1	SORBS2	MYLK	MAP3K7CL	CCDC3	TAGLN	FRZB	SLC38A11	ITGA8	FBXL13	KCNMB1
CELLMARKERMSIGDB_TRAVAGLINI_LUNG_ALVEOLAR…		EMP2	SPOCK2	AGER	COL4A2	SCEL	TIMP3	UPK3B	RTKN2	CAV1	FSTL3	COL12A1	COL8A1	NCKAP5	SPARC	IL32	CLIC5	HBEGF	UNC13D	S100A10	COL4A1	ANKRD29	TUBB6	FADS3	WFS1
CELLMARKERMSIGDB_TRAVAGLINI_LUNG_BASOPHIL…		LMNA	TPSAB1	TPSB2	CPA3	MS4A2	BIRC3	NFKBIA	HECW2-A…	HPGDS	GADD45B	CSF1	RGS13	SELENOK	IL1RL1	RGS2	ANXA1	VWA5A	CPM	HDC	TNFRSF9	GPR65	NR4A1	RHEX	LAPTM4A
CELLMARKERMSIGDB_TRAVAGLINI_LUNG_CAPILLAR…		HPGD	EDNRB	S100A3	IL1RL1	SOSTDC1	S100A4	CYP3A5	CA4	PRX	IL32	HOPX	AQP1	ICAM2	SERPINE1	TBX2	SLC14…	CD74	SPARC	B2M	EMCN	TGFBR3	SLC12A2	DHRS7	S100A6
CELLMARKERMSIGDB_TRAVAGLINI_LUNG_CAPILLAR…		TMSB4X	SLC9A3R2	TMSB10	PCAT19	SPTBN1	PTMA	KTN1	CSDE1	GUK1	UQCR11	ATP5ME	C4orf3	CAVIN1	BRK1	TMA7	ISCU	ELOB	ANAPC16	RAB13	SEC62	ATP5MK	DYNLRB1	UBL5	SNHG6
CELLMARKERMSIGDB_TRAVAGLINI_LUNG_CD4_MEMO…		IL32	CD52	CD3D	INPP4B	TNFRSF25	CD3E	LDHB	LCK	CD28	IL7R	TTC39C	CD2	ITGB1	CD5	RASGRP1	RPL23A	CD6	RPS27	GIMAP7	RPS29	SYNE2	RCAN3	IL10RA	CD40LG
CELLMARKERMSIGDB_TRAVAGLINI_LUNG_EREG_DEN…		RAB31	NAMPT	CLEC5A	CTSB	IER3	EREG	VEGFA	CD93	THBS1	GPX1	PLAUR	FPR3	FCGR2A	CD163	THBD	MXD1	HLA-DQB1	C5AR1	SAT1	MS4A6A	HBEGF	GPR183	ACSL1	CXCL2
CELLMARKERMSIGDB_TRAVAGLINI_LUNG_IGSF21_D…		FOLR2	RNASE1	SLCO2B1	MRC1	C1QC	C1QB	C1QA	IGSF21	F13A1	CCL2	TMEM176B	MSR1	GFRA2	MAMDC2	MS4A4A	PLTP	PDK4	CCL13	SERPIN…	STAB1	VSIG4	GPX3	LGMN	CTSL
CELLMARKERMSIGDB_TRAVAGLINI_LUNG_MESOTHEL…		CALB2	KRT8	CFB	CPA4	HP	KRT19	RPL28	ITLN1	KLK11	MSLN	EMB	UPK3B	TFPI2	CXADR	RPL39	OCIAD2	CLDN1	PDPN	MIF	ASPHD1	C19orf33	CXCL6	RPS19	REC8
CELLMARKERMSIGDB_TRAVAGLINI_LUNG_MYOFIBRO…		ASPN	CLU	TSPAN8	PAMR1	SCARA3	WIF1	TGFBI	TNC	BCHE	FGF18	LUZP2	CD9	CFH	DPT	TYRP1	LINC0…	ROBO2	HS3ST3A1	SFTA1P	COL16A1	NOTUM	RARRES1	THBD	TNFRSF19
CELLMARKERMSIGDB_TRAVAGLINI_LUNG_NATURAL_…		HLA-C	XCL1	KLRC3	KLRC1	XCL2	CTSW	KLRD1	HOPX	ID2	PRF1	ZNF683	IL2RB	KRT86	TNFRSF18	GNLY	KLRC2	B2M	CXCR4	DUSP2	CD69	NKG7	SOCS1	SLA2	SH2D1B
CELLMARKERMSIGDB_TRAVAGLINI_LUNG_NEUTROPH…		FCGR3B	IL1R2	CYP4F3	S100A9	IFITM2	S100A8	S100P	CSF3R	NAMPT	FPR1	MMP25	ADGRG3	G0S2	BASP1	CXCR1	SLC11…	APOBEC3A	MME	SLC25A…	ACSL1	MXD1	FTH1P20	LUCAT1	CXCR2
CELLMARKERMSIGDB_TRAVAGLINI_LUNG_OLR1_CLA…		SOD2	TIMP1	AQP9	MARCKS	ATP13A3	NAMPT	IL1RN	IL1B	HIF1A	ACSL1	IFNGR2	C15orf48	PPIF	G0S2	CTSL	CD44	SLC43A2	CD93	UPP1	GK	BCL2A1	SERPIN…	IER3	SPHK1
CELLMARKERMSIGDB_TRAVAGLINI_LUNG_PLATELET…		GNG11	RGS18	PPBP	NRGN	ACRBP	SPARC	NCOA4	PF4	TUBB1	RUFY1	LIMS1	GRAP2	PGRMC1	MAX	MMD	CAVIN2	CA2	RAP1B	ODC1	CTSA	RGS10	NT5C3A	MPP1	FERMT3
CELLMARKERMSIGDB_TRAVAGLINI_LUNG_PROLIFER…		H2AZ1	HMGB2	TUBA1B	STMN1	PBK	BIRC5	MKI67	TUBB	UBE2C	TOP2A	TK1	PCLAF	ASPM	NUSAP1	AURKB	CDKN3	NUF2	PTTG1	HMMR	ASF1B	GTSE1	CENPF	ZWINT	KIFC1
CELLMARKERMSIGDB_TRAVAGLINI_LUNG_PROLIFER…		TUBA1B	H2AZ1	TUBB	HMGN2	TK1	PCLAF	TYMS	CDK1	UBE2C	STMN1	BIRC5	NUSAP1	MKI67	CENPW	RAD51AP1	MARCO	MAD2L1	CEP55	HMMR	FABP4	CCNB2	ZWINT	TOP2A	GGH
CELLMARKERMSIGDB_TRAVAGLINI_LUNG_PROXIMAL…		PRSS23	KRT15	AQP3	MIR205HG	KRT5	ADH7	LMO4	KRT19	GAPDH	BTF3	GLUL	SERPINB3	CSTA	RACK1	CLCA2	DAPL1	DDIT4	RHOC	EEF2	RPL10A	FAM107A	SNCA	IGFBP3	S100A2
CELLMARKERMSIGDB_TRAVAGLINI_LUNG_SEROUS_C…		LYZ	ZG16B	PRR4	C6orf58	PIP	LTF	TCN1	S100A1	NDRG2	AZGP1	SLC12A2	LPO	PRB3	LRRC26	S100B	DMBT1	SLPI	LINC018…	PRB1	PART1	PRH2	MIA	PRH1	GNAS
CELLMARKERMSIGDB_TRAVAGLINI_LUNG_SIGNALIN…		SLC22A…	PEBP4	SFTPD	CLDN18	PXMP4	PGC	SFTPA2	GSTA4	SFTPA1	LRRK2	SECISBP…	SFTPB	NPC2	SFTA3	MUC1	C16or…	AKAP13	CACNA2D2	NECAB3	PTPN13	CPM	PMM1	RGS16	RNASE1
CELLMARKERMSIGDB_TRAVAGLINI_LUNG_TREM2_DE…		APOE	LIPA	TREM2	GPNMB	CHI3L1	CCL18	CHIT1	LILRB4	ACP5	CTSB	CTSZ	PRDX1	CSTB	APOC1	CAPG	NUPR1	PLD3	CD68	GRN	CTSD	HLA-DMB	LGMN	ANXA2	BRI3
CELLMARKERMSIGDB_TRAVAGLINI_LUNG_VASCULAR…		COX4I2	NOTCH3	TINAGL1	ESAM	MCAM	TAGLN	EGFL6	KRT18	ADGRF5	LRRC32	WFDC1	HEYL	ACTA2	EPAS1	TM4SF1	HES4	PDGFA	ACTG2	MYL9	RGS5	ECRG4	EPS8	EFHD1	CNN1
CELLMARKERMSIGDB_TRAVAGLINI_LUNG_VEIN_CELL		ACKR1	PLA1A	CPE	C7	VCAM1	PLAT	HDAC9	CLU	FTH1	NAMPT	CCL23	SELP	LYVE1	PRSS23	NNMT	SRPX	SOD2	CYP1B1	NCOA7	CXCL2	IL1R1	VWF	FSTL3	SOX7
CELLMARKERMSIGDB_VANGURP_PANCREATIC_ALPHA…		ALDH1A1	B2M	CFC1	CLU	CRYBA2	CYSTM1	DPP4	F10	FAP	FXYD3	FXYD5	GC	GCG	GLS	GPX3	HIGD1A	HLA-A	HLA-E	IRX2	ITGB1	KCTD12	LOXL4	MUC13	NAA20
CELLMARKERMSIGDB_VANGURP_PANCREATIC_DELTA…		ABCC9	ABI3BP	ADGRL2	AKAP12	AMIGO2	ANK1	AQP3	ARFGEF3	BAIAP3	BCHE	BHLHE41	C22orf42	CADM1	CALB1	CASR	CBLN4	CD9	CPB1	CXADR	DHRS2	DIRAS3	DPYSL3	EDN3	EHF
CELLMARKERMSIGDB_VANGURP_PANCREATIC_GAMMA…		ABCB1	ABCC9	AKAP12	AMOTL1	AQP3	ARX	BMERB1	CALB1	CARTPT	CHN2	CHRM3	CPB1	DPYSL3	ETV1	FGFR1	FXYD2	FXYD6-F…	GC	GCNT3	ID1	ID2	ID4	INPP5F	MEIS2
CELLMARKERMSIGDB_ZHENG_CORD_BLOOD_C1_PUTA…		ABCC3	ABCC4	ACTN1	ARHGAP18	ARHGAP6	BANK1	BIN2	C2orf88	C3orf52	CA2	CALD1	CAMK1	CCDC68	CCL5	CCND1	CD36	CD9	CENPA	CLEC1B	CLU	CMAS	CMTM5	COQ2	CTTN
CELLMARKERMSIGDB_ZHENG_CORD_BLOOD_C10_MUL…		ACAP2	ACTG1	ACY3	ADA	AFF1	JAML	ARHGAP25	ARHGEF7	ARL6IP5	ARPC5L	ARRB2	BAALC	BTK	VSIR	RSRP1	C9orf…	CARD19	CARD11	CD47	CD52	CD74	CD99	CLNK	COBLL1
CELLMARKERMSIGDB_ZHENG_CORD_BLOOD_C2_PUTA…		AFF2	ALOX5AP	ANXA1	ARSGL1	ATF4	BACE2	BRIX1	CALR	CANX	CASP3	CCDC88A	CD63	CD82	CHP1	CHPT1	CNRIP1	CSF2RB	CTNNBL1	DNAJC9	EMP3	FAM178B	TENT5A	FBXO33	FBXO7
CELLMARKERMSIGDB_ZHENG_CORD_BLOOD_C4_PUTA…		ABCA1	ARHGAP15	ATP6V0…	BMP2K	CAMLG	CCND2	CD69	CD84	CDK6	CELF2	CKAP2	CPA3	CRHBP	CTNNBL1	CYCS	DDX21	DEPTOR	DGKE	DTD1	EIF2AK1	ELF1	ELMO1	EREG	ERG
CELLMARKERMSIGDB_ZHENG_CORD_BLOOD_C5_SIMI…		RNU2-5…	RNU2-3P	RNVU1-…	RNVU1-15	RNVU1-19	SNORD13	RNU4-2	RNU2-63P	RNU2-5P	RNU4ATAC	LINC009…	RNVU1-6	RNU5A-1	RNU12	RNU5F-1	RNU2-…	RNU5E-1	RNVU1-24	ZFP36	RNU5E-4P	H2BC8	DUSP1	H2BC5	RNU1-34P
CELLMARKERMSIGDB_ZHENG_CORD_BLOOD_C6_HSC_…		ALDH1A1	AREG	ARPC5L	AVP	BEX1	CCNH	CCNY	CD109	CD74	CEBPB	CRHBP	CXCR4	DDIT4	DUSP1	DUSP2	EZR	FAM3C	FOSL2	FOXO1	FTH1	GADD45A	GATA3	GBP4	TAMALIN
CELLMARKERMSIGDB_ZHENG_CORD_BLOOD_C7_PUTA…		LRRC75A	RPL30-AS1	SNHG29	MT-ND6	SFXN1	BEST1	NAP1L1	CSF3R	PABPC1	TSIX	NUCB2	MALAT1	PIM2	PIK3R1	GYPC	MAP3K8	CALR	ZFP36L2	CD99	TPT1	ANKRD28	TPM4	C6orf62	XIST
CELLMARKERMSIGDB_ZHENG_CORD_BLOOD_C8_PUTA…		MAP3K8	FAM30A	CD99	GYPC	C1QTNF4	CD52	SMIM24	TMSB4X	CSF3R	MT-CO3	TPT1	BAALC	SPINK2	MZB1	B2M	RACK1	GAPDH	CD74	TSC22D3	ANGPT1	ZFAS1	ITM2C	HLA-A	TPM4
CELLMARKERMSIGDB_ZHONG_PFC_C1_ASTROCYTE		ANOS1	SELENBP1	PGM2	AGT	GPX3	EFNA1	TMEM126B	LRATD2	ADORA2B	EIF6	SLC25A18	SPTLC1	DDX24	MRPL19	MMAB	TBC1D…	SCRG1	APOE	ETV1	RIT2	MASP1	SPARCL1	VEPH1	CD82
CELLMARKERMSIGDB_ZHONG_PFC_C1_DLX5_POS_IN…		BTG1	DLX5	DLX6-A…	PDZRN3	PRKCA	RDX	SCGN																	
CELLMARKERMSIGDB_ZHONG_PFC_C2_ASCL1_POS_A…		ASCL1	PPP4R3B	NDUFA12	TRIB2	DDIT4	HPF1																		

Table S5: Cell state marker gene list

CELLMARKERCAN_ACINAR_CELLS		CTRB1	KLK1	RBPJL	PTF1A	CELA3A	PRSS1	SPINK1	ZG16	CEL	CELA2A	CPB1	CELA1	RNASE1	AMY2B	CPA2	CPA1	CELA3B	PNLIP	CTRB2	PLA2G1B	PRSS2	CLPS	REG1A	SYCN
CELLMARKERMSIGDB_ZHONG_PFC_C2_UNKNOWN_NPC		AMBN	ARHGAP11A	ARL6IP1	ASPM	AURKA	AURKB	CALB2	CCNA2	CCNB1	CCNB2	CDC20	CDC25B	CDCA2	CDCA3	CDCA8	CDKN3	CENPE	CENPF	CEP70	CIT	CKAP2L	CKS2	CNOT7	DCXR
CELLMARKERMSIGDB_ZHONG_PFC_C3_ASTROCYTE		MIAT	FBN3	ENSG00…	CPNE7	PCDH11X	LINC00…	CAVIN4	SSTR2	GOLGA8B	CHD5	PRKAR2B	KIAA0319	NCOR2	SRRM3	PODXL2	TMEM6…	SYCP2	MEG3	SLC4A7	AP1G2	SH3PXD2A	CHST2	CNTNAP1	ULK2
CELLMARKERMSIGDB_ZHONG_PFC_C3_HOPX_POS_OPC		ETV1	TLK1	HOPX	DLL3	RARRES2	SEZ6	PANTR1	CST3																
CELLMARKERMSIGDB_ZHONG_PFC_C3_MICROGLIA		SDK2	GPC1	MARCKS…	FAM229B	SDK1	EPM2AI…	CELF2-AS2	DBNDD1	FOXP1	TLE5	STK25	C12orf73	RIT2	SULT4A1	WWC1	MDGA1	HMGCR	NRXN1	SAMD4B	JPH3	C1orf216	ZFTA	PIK3R2	RAB3A
CELLMARKERMSIGDB_ZHONG_PFC_C3_UNKNOWN_INP		APOLD1	BIRC5	BUB1	CCDC112	CCNA2	CCNB2	CDCA2	CDCA8	CDK1	CDKN2C	CENPE	CENPF	CKAP2L	CNR1	DLGAP5	ECT2	FAM83D	H2BC9	HJURP	HYLS1	INSM1	KIF23	KPNA2	MXD3
CELLMARKERMSIGDB_ZHONG_PFC_C4_UNKNOWN_INP		ACTR10	ARHGEF26	ARL4D	ASCL1	ATG3	ATP6V0…	BBS10	BRD7	C19orf…	CFAP298	RBIS	CADPS	CIR1	COA1	COMMD9	COX17	DANCR	DDIT4	DENND1B	DLL3	DOCK7	DPF2	DYRK4	EBF2
CELLMARKERMSIGDB_ZHONG_PFC_C6_PLXNA4_POS_…		CCBE1	EIF1B	GNAL	LINC011…	PLXNA4	SCG2	SRM																	
CELLMARKERMSIGDB_ZHONG_PFC_C7_ORG_UNDERGO…		ABHD6	ACYP2	ADGRG6	ADRA2A	AGPAT3	ARPP21	ATAD2B	C8orf34	CACNG8	CEP126	CHL1	CPTP	CRYM	DLG2	DNAL4	DOCK4	DPP6	DYNC1I1	ENC1	EPHA3	EPHB1	ESYT2	FGF9	GAREM1
CELLMARKERMSIGDB_ZHONG_PFC_C8_ORG_PROLIFE…		ANXA2	ARL6IP1	ASPM	AURKA	BIRC5	BUB1	BUB1B	CCDC34	CCNA1	CCNB1	CCNB2	CDC20	CDCA3	CDCA8	CDKN3	CENPA	CENPE	CENPF	CEP70	CKAP2	CKS2	CRNDE	CRYZ	DCXR
CELLMARKERMSIGDB_ZHONG_PFC_C8_UNKNOWN_NEU…		ABLIM1	ACTN2	ADCYAP…	ALCAM	ARHGAP23	AUTS2	B3GAT1	B4GALT2	BHLHE22	CACNA1A	CACNG8	CALN1	CAMK2N2	CAPNS1	CCBE1	CEP126	CHD5	CNTN1	CNTN3	COPRS	CPE	CRYM	CSRP2	DEAF1
CELLMARKERMSIGDB_ZHONG_PFC_C9_ORG_OTHER		ATP1A2	ATP1B2	AXL	BCAN	BMP7	TCIM	CDC42EP1	CDO1	CLU	COL11A1	CRYL1	CST3	CXCR4	DBI	DKK3	EEPD1	ENHO	ETV5	F3	FABP5	FABP7	FAM107A	FOLH1	GATM
CELLMARKERMSIGDB_ZHONG_PFC_HES1_POS_C1_NPC		HES1	HEY1	HMGA2	LIX1	MCM3	ZFP36L1																		
CELLMARKERMSIGDB_ZHONG_PFC_MAJOR_TYPES_AS…		SPARCL1	CST3	AGT	CLU	CXCL14	TNC	ALDOC	ATP1A2	APOE	AQP4	SLCO1C1	MT1E	BTBD17	MLC1	HOPX	CLDN10	S100A16	MFGE8	S1PR1	PON2	ATP1B2	CD9	MT3	S100A13
CELLMARKERMSIGDB_ZHONG_PFC_MAJOR_TYPES_EX…		NEUROD6	SLA	CSRP2	IGFBPL1	NEUROD1	RBFOX1	PPP2R2B	NEUROD2																
CELLMARKERMSIGDB_ZHONG_PFC_MAJOR_TYPES_MI…		CCL3	AIF1	C3	CCL4	SPP1	C1QB	P2RY12	CX3CR1	RGS1	VSIG4	CSF1R	C1QA	LAPTM5	TYROBP	TREM2	FOLR2	FCER1G	FCGR3A	BHLHE41	P2RY13	FCGRT	PLD4	CD53	LTC4S
CELLMARKERMSIGDB_ZHONG_PFC_MAJOR_TYPES_OPC		APOD	OLIG1	PDGFRA	SCRG1	S100B	PMP2	GPR17	SOX10	OLIG2	COL20A1	PLAT	FIBIN	PLLP	ARC	NKX2-2	BCAN	BCHE	COL9A1	ARL4A	OMG	RAMP1	CA10	ANGPTL2	PCDH15

Table S5: Cell state marker gene list

CELLMARKERCAN_ACINAR_CELLS	PNLIPRP1	CTRC	REG3A	SERPINA3	PRSS3	REG1B	CFB	GDF15	MUC1	C15ORF48	DUOXA2	AKR1C3	OLFM4	GSTA1	LGALS2	PDZK1IP1	RARRES2	CXCL17	UBD	GSTA2	ANPEP	LYZ	ANGPTL4	ALDOB	
CELLMARKERCAN_ADIPOCYTE_PROGENITOR_CELLS																									
CELLMARKERCAN_ADRENERGIC_NEURONS																									
CELLMARKERCAN_AIRWAY_EPITHELIAL_CELLS																									
CELLMARKERCAN_ALPHA_CELLS	MAFB	PAX6	NEUROD1	LOXL4	PLCE1	GC	KLHL41	FEV	PTGER3	RFX6	SMARCA1	PGR	LDB2	IRX1	UCP2	FXYD5	RGS4	GLS							
CELLMARKERCAN_ALVEOLAR_MACROPHAGES																									
CELLMARKERCAN_ASTROCYTES	SLC16A2	SYNE1	DIO2	SLC7A10	ENTPD2	HSPB6	PLXNB1	LCAT	PLA2G7	CMTM5	S1PR1	SLC39A12	LUZP2	GSTA4	SLC25A28	SLC25A46	WNT3	FZD2	TEAD1	RLBP1	ALDOC	GJA1	APOE	LCN2	SOX9
CELLMARKERCAN_B_CELLS	BANK1	VPREB3	POU2AF1	CXCR4	IGLC2	IGLC3	VPREB1	LRMP	KLHL6	FAM129C	BST1	BACH2	PXK	CD38	PTPRC	EBF1	BCL11A	CCR7	CD55	CD74	CD52	TLR9	SWAP70	HMGA1	
CELLMARKERCAN_B_CELLS_MEMORY	CD79B	CLCA3P	CR2	CXCR5	DENND5B	FCGR2B	FCRL2	FRK	GNG7	GPR18	GUSBP11	HHEX	HLA-DOB	IFNA10	IGHD	IGHM	IGKC	IGLL3P	IL7	IRF8	LTB	LY86	MBL2	MS4A1	NMBR
CELLMARKERCAN_B_CELLS_NAIVE	FRK	GPR18	GUSBP11	HHEX	HLA-DOB	IGHD	IGHM	IGKC	IGLL3P	IL4R	IRF8	LTB	LY86	MEP1A	MICAL3	MS4A1	NMBR	P2RX5	P2RY14	PNOC	PSG2	PTPRCAP	RALGPS2	RASGRP2	SELL
CELLMARKERCAN_BASOPHILS	DNASE2B	FADS2	GPC4	GRM6	ACSS2	ADORA2B	AQP9	BACE1	BMP4	CABLES1	CCL4	CEBPA	E2F8	FADS1	FAR2	FBXO9	GABBR1	GPR183	HGF	HIST1H1D	HIST1H2AC	IFITM1	INF2	JAZF1	L1CAM
CELLMARKERCAN_BERGMANN_GLIA	ZEB2	UFL1	NRXN3	ATP7A	BICD2	GJA1	METRN	P2RX7	EGFR	GRIA1	P2RY1	GRIN2B	GRIN3A	46269	DAO	ITGB1	KIF1BP								
CELLMARKERCAN_BETA_CELLS	GJD2	SCGN	IGF2	SYT13	FFAR2	NPTX2	PFKFB2	EDARADD	HOPX	SH3GL2	ADCYAP1	SCGB2A1	CASR	MAFB	PAX6	NEUROD1	ISL1	TGFBR3	SMAD9	SIX3	SIX2	BMP5	PIR	STXBP5	DLK1
CELLMARKERCAN_CAJAL_RETZIUS_CELLS																									
CELLMARKERCAN_CARDIAC_STEM_AND_PRECURSOR_…																									
CELLMARKERCAN_CARDIOMYOCYTES	CKMT2	MYH7B	MYOM2	PDLIM5	MYH7	PLN	TCAP	SORBS2	RYR2	ACTC1	MYL3	PDE4D	NEXN	FHL2	MYBPC3	MYH6	TTN	GPIHBP1	AQP1	MFN2	ASB2	ART3	RRAD	JPH2	HSPB7
CELLMARKERCAN_CHOLANGIOCYTES	SOX9	EPCAM	CLDN4	MMP7	TFF2	SCGB3A1	FXYD2	DEFB1	CD24	LCN2	CXCL1	CXCL6	LGALS2	TACSTD2	ELF3	SPP1	MUC5B	LGALS4							
CELLMARKERCAN_CHOLINERGIC_NEURONS																									
CELLMARKERCAN_CHONDROCYTES	IGF2	MIA	SDC3	EMP1	CYTL1	LCN2	LUM	GADD45B	SCRG1	S100A9	CHAD	DLK1	S100A8	CHODL	ATF4	SHOX	MGP	TCF4	VDR	ATRX	NOG	AGER	IL10RA		
CELLMARKERCAN_CHOROID_PLEXUS_CELLS																									
CELLMARKERCAN_CILIATED_CELLS																									
CELLMARKERCAN_DELTA_CELLS	PRG4	GHSR	FRZB	PCSK1	GABRG2	POU3F1	BHLHE41	EHF	LCORL	ETV1															
CELLMARKERCAN_DOPAMINERGIC_NEURONS																									
CELLMARKERCAN_DUCTAL_CELLS	PERP	PDLIM3	WFDC2	SLC3A1	AQP1	ALDH1A3	VTCN1	KRT19	TFF1	TFF2	KRT7	CLDN4	LAMB3	TACSTD2	CCL2	DCDC2	CXCL2	CTSH	S100A10						
CELLMARKERCAN_EMBRYONIC_STEM_CELLS	ITGB1	CD59	PROM1	PECAM1	ITGA6	KITLG	SOX2	KLF4	ZFP42	ZFX	TAF8	HMGA2	NACC1	NR6A1	STAT3	LEF1	L1TD1	KHDC3L	GDF3	DPPA5	DPPA4	DPPA2	DPPA3	SMAD1	SMAD5
CELLMARKERCAN_ENTERIC_GLIA_CELLS																									
CELLMARKERCAN_ENTERIC_NEURONS																									
CELLMARKERCAN_ENTEROCYTES	SI	VIL1	FUOM	FAHD1	OTC	CDH17	KHK	VDR	RNF186	KLF5	CLDN15	ALDOB	PRAP1	CCL25	LGALS2	GFRA2	L1CAM	SLC11A2	SLC26A3	APOA1	APOA4	CBR1	EPHX2	GSTM3	CREB3L3
CELLMARKERCAN_ENTEROENDOCRINE_CELLS	NTS	SST	MLN	GPR119	GCG	CHGB	CPE	GAL	NUCB2	IAPP	GHRL	CHGA	TRPA1	ENPP2	AFP	VEGFA	FFAR4	MC4R	INSL5	SSTR2	ALCAM	TAS1R2	TAS1R3	GNAT3	
CELLMARKERCAN_EPENDYMAL_CELLS	ANGPTL2	STOML3	PLTP	SSPO	EFNB3	MYB	RFX2	ZMYND10	AK8	CFAP44	TM4SF1														
CELLMARKERCAN_EPSILON_CELLS	HRH2	CALCR	SLC6A16	PCSK6	ADAMTS6	COL22A1	FAM124A	COL12A1	CD109	THSD4	ARX	NKX2-2	ACSL1	FRZB	VTN	APOH	SPINK1	VSTM2L	SPTSSB	S100A6	KRT8				
CELLMARKERCAN_FOLLICULAR_CELLS																									
CELLMARKERCAN_FOVEOLAR_CELLS																									
CELLMARKERCAN_GAMMA_DELTA_T_CELLS	MKI67	UBE2C	TUBA1B	ASPM	CENPA	TOP2A	PCNA	AURKB	BIRC5	NUSAP1	TROAP	TUBB	H2AFX	CENPF	CCNB1	H2AFZ	NA								
CELLMARKERCAN_GERM_CELLS	EGR4	SMC1B	NR6A1	RSBN1L	DYNLT1	PARN	MKI67	CHEK1	BRCA1	DMC1	MRPL14	MLH3	SPAG6	ZPBP2	DNAH6	CAMK4	CREM	MYO1D	HOOK1	SPATA12					
CELLMARKERCAN_GLUTAMINERGIC_NEURONS																									
CELLMARKERCAN_GRANULOSA_CELLS																									
CELLMARKERCAN_HEMATOPOIETIC_STEM_CELLS	HHEX	MEIS1	LYL1	ELF1	HLF	RGS18	MYCT1	ADGRG1	DNMT3B	MYCN	ARID3A	ITGA2B	GATA3	ROBO4	PITX2	TXNIP	RUNX1	PTPRS	PTPRC	CD44	GFI1	CD48	HMGB3	TLR2	APC
CELLMARKERCAN_IMMATURE_NEURONS	RBFOX3	CREB1	NEUROD1	ASCL1	NTRK1	SYN1	NF1	BCL2	SLC6A5	FOXD2	ICAM5	SSTR2	NEUROG2												
CELLMARKERCAN_INTERNEURONS	NA	NXPH1	PTPRO	CXCL14	PRKCG	GABRA5	PAX6	PNOC	HTR3A	RELN	CALB2	PDE1A	ADGRL2	KCNIP2	RGS10	NOV	CPNE5	SLC5A7	CRH	GDA	SEMA3E	DLX1	DLX2	DLX5	ARX
CELLMARKERCAN_IONOCYTES																									
CELLMARKERCAN_KERATINOCYTES	TRIM29	DSC3	FST	AQP3	KRT15	S100A14	DMKN	ENTPD2	NGFR	TFAP2A	LGALS7	CCL2	SFN	KRT10	SBSN	KRT84	KRT36								
CELLMARKERCAN_KUPFFER_CELLS	CD163	CCR5	CLEC1B	CLEC4G	GPIHBP1	FOLR2	PLTP	FTL	IRF7	SPIC	CSF1R	C1QC	C1QA	C1QB	CLEC4E										
CELLMARKERCAN_LEYDIG_CELLS	VIT	ALDH1A3	NOS1	EDNRA	SMOC2	ADAM21	ESRRG	GPRC6A	GJA1	GAS6	SULT1E1	PLAUR	FABP9												
CELLMARKERCAN_LOOP_OF_HENLE_CELLS	SPSB1	ARID5A	CD40	SLC14A2	NRGN	RNF182	PCSK9	DRAM1	ARC	NEK6	CDKN1C	CALCRL	SMARCD3	KCNT1	PTGS2	IRX2	PPP1R1B	LIPG	TNNI1	LRRC66	IGF1				
CELLMARKERCAN_LUMINAL_EPITHELIAL_CELLS	SERPINA1	PIP	ATP7B	HIF1A	FGFR4	DDR1	CEBPD	PGR	KRT8	UXT	PTH1R	CD9	AR	AQP3	ATP2C2	WNT5A	SLC12A2	ESR1	AQP5	RUNX1	CDH1	MUC1	ANPEP	KLK3	
CELLMARKERCAN_LUTEAL_CELLS																									
CELLMARKERCAN_MEGAKARYOCYTES	FLI1	PROX1	SYP	TSPAN9	RGS18	TREML1	GATA2	VWF	ALOX12	SELP	NFATC1	CIB1	PLK3	MAFG	IL2RA	PECAM1	PPARG	IL6	IL21R	LRP1	ACHE	VEGFA	TGFB1	GP9	
CELLMARKERCAN_MELANOCYTES	PRKN	KLC2	IL24	TPH2	PPARA	PPARD	PPARG	PRKCA																	
CELLMARKERCAN_MESANGIAL_CELLS	SH3BGRL	RASD1	SNCG	FILIP1L	FLNA	MEF2C	CSTB	SMARCA5	PDE3A	MAP3K20															
CELLMARKERCAN_MESOTHELIAL_CELLS																									
CELLMARKERCAN_MICROFOLD_CELLS	ONECUT2	FOSL2	IRF6	IRF2	NFIB	FOXP4																			
CELLMARKERCAN_MICROGLIA	S100A8	LTC4S	TGFBR1	S100A9	LPCAT2	ITGB5	SPHK1	COLEC12	ALDH1A2	CCR5	SLCO2B1	PTGS1	APBB1IP	CYTH4	MERTK	MAFB	HPGDS	CCL4	CCL3	CCRL2	UCP2	CLEC3B	MRC2	CLEC10A	PF4
CELLMARKERCAN_MONOCYTES	ITGAM	ACE	FUT4	NA	SELL	CD163	FCGR1A	FCGR2B	ACP5	MRC1	GBP1	OAS1	IRF7	PLSCR1	MX1	IL1RN	IFIT1	IDO1	IFIT3	CXCL10	S100A9	S100A8	S100A4	CLEC7A	CSF3R
CELLMARKERCAN_MOTOR_NEURONS																									
CELLMARKERCAN_MULLER_CELLS	GNAI2	GPR37	KCNJ2	KCNJ10	SPC25	ITM2B	ATG4B	CA2	ZBED4	PRDX6	SLC1A3	HES1	KIT	CRB1	ANXA4	CD44	GSTM5	SLC6A1	SFTPA1						
CELLMARKERCAN_MYELOID_DERIVED_SUPPRESSOR_…																									
CELLMARKERCAN_MYOBLASTS	MYD88	BRAF	EGFR	ZBTB16	SIX1	PDZRN3																			
CELLMARKERCAN_MYOEPITHELIAL_CELLS																									
CELLMARKERCAN_MYOFIBROBLASTS																									
CELLMARKERCAN_NEUROBLASTS	EFNA2	DCX	CCKAR	CCKBR	GRM5	EZH2	DLX2	MARK2	LRP8	VLDLR	DAB1	NCAN	EPHA4	TACC3	EGFR	TUBB3	SIRT1	NCAM1	NEUROD1	SCGN	KLK3	NES	WT1	ZBED4	PROS1
CELLMARKERCAN_NEUROENDOCRINE_CELLS																									
CELLMARKERCAN_NEURONS	PRPH	MECP2	RIN1	OMP	PTPN11	GHSR	NPY	AGRP	RESP18	STX1A	SCN2B	NA	B3GAT2	FMO1	FBLN2	SHISA2	LUZP2	SCRG1	IGF1	NRGN	CELF6	AVP	RORA	VGF	PCSK2
CELLMARKERCAN_NEUTROPHILS	LTF	LYZ	ELANE	S100A8	IL1B	CXCL2	HP	CCL3	HDC	MMP8	LRG1	OSM	MMP9	PILRA	CLEC4D	CLEC4E	ASPRV1	CCRL2	CCR1	NCF1	TREM1	SORL1	ARG2	BST1	IL1R2
CELLMARKERCAN_NK_CELLS	HMBOX1	AHR	PRF1	CCL4	SEMA6D	FHL2	CD2	CD7	CD3G	CD33	DPP4	LAT	PCDH15	CCL5	GZMA	GZMB	CMA1	DUSP2	TXK	DOK2	CST7	KLRC2	SAMD3	TBX21	CHSY1
CELLMARKERCAN_NORADRENERGIC_NEURONS																									
CELLMARKERCAN_NUOCYTES																									
CELLMARKERCAN_OLIGODENDROCYTES	FASN	OMG	ZDHHC9	PTGDS	ADAMTS4	MYRF	SEMA4D	TMEM125	KLHL2	DPY19L1	TSPAN15	HAPLN2	NKX6-2	GJB1	OPALIN	VLDLR	MCAM	TMEM163	ERMN	FA2H	CLDN11	ANLN	ELOVL7	PLLP	DDC
CELLMARKERCAN_OSTEOBLASTS	CCL11	SPP1	FAS	TNFRSF10A	WNT5A	CX3CL1	F2R	PTH1R	ITGA2	ITGA5	NOD1	NOD2	THY1	ESR1	CYP27B1	P2RX4	P2RX5	INSR	EFNB2	EPHB4	VCAM1	FGFR1	FGFR2	BGLAP	TLR5
CELLMARKERCAN_OSTEOCLAST_PRECURSOR_CELLS																									
CELLMARKERCAN_OSTEOCLASTS	SLC39A14	AQP9	ONECUT1	ITGB3	ITGB5	IRAK3	ITGA2	TAS1R3	SERPINF1	ITGAV	ITGB1	P2RY6	CCL3	EPHB1	CA2	HIF1A	CX3CR1	ESR1	EFNB2	VDR	CASR	CD68	GRM8	SMAD4	SPP1
CELLMARKERCAN_OSTEOCYTES																									
CELLMARKERCAN_PANETH_CELLS	TLR5	WNT3	KCNN4	RAP1A	ATG16L1	WNT9B	DLL4	IL4R	TLR9	CTNNB1	DLL1	TM4SF20	DEFA3	MPTX1	KLF15										
CELLMARKERCAN_PARATHYROID_CHIEF_CELLS																									
CELLMARKERCAN_PERICYTES	INPP4B	VIM	PTH1R	IFITM1	TBX18	NT5E	MFGE8	ALPL	COL1A1	MYO1B	COG7	P2RY14	HEYL	GNB4	MSX1	CTGF									
CELLMARKERCAN_PERITUBULAR_MYOID_CELLS																									
CELLMARKERCAN_PHOTORECEPTOR_CELLS																									
CELLMARKERCAN_PLASMA_CELLS	FCGR2B	CCR10	ABCB9	AMPD1	ANGPT4	ATXN8OS	C11ORF80	CD79A	DENND5B	EAF2	FCRL2	GNG7	GPR25	GUSBP11	HIST1H2AE	HIST1H2BG	HLA-DOB	IGHD	IGHE	IGHM	IGKC	IGLL3P	KCNA3	KCNG2	LIME1
CELLMARKERCAN_PLASMACYTOID_DENDRITIC_CELLS	IRF7	IRF8	IGLL1	CUX2	MCTP2	KLK1	BLNK	GRM8	PRKCA	CDH1	IL17RB	CARD6	CLEC4G	S100A8	JCHAIN	SERPINF1	ITM2C	TCF4	BCL11A	MZB1	DERL3	ZFAT	NRP1	SLC15A4	CCR9
CELLMARKERCAN_PODOCYTES	DPP4	ANGPTL2	ITGB5	RHPN1	BCAM	MAGI2	COL4A3	EFNB1	NPR1	PLOD2	SRGAP1	LAMB2	NPR3	ITGA3	PLCE1	PROS1	MERTK	MYO1D	TCF21	TNS2	ADM	OPTN	PARD3B	SEMA3E	MYO1E
CELLMARKERCAN_PROXIMAL_TUBULE_CELLS	NOX4	SLC17A1	SLC13A3	CYP2E1	SLC22A8	FOLR1	SLC7A13	AK4	SLC5A8	SLC22A1	GHR	UPB1	SLC17A3	GCDH	GSTZ1	ASL	FAH	TMEM106A	GSTA2	CRYL1	SLC5A12	AOAH	SLC13A1	ABCC2	SLC9A8
CELLMARKERCAN_PULMONARY_ALVEOLAR_TYPE_I_C…	PXDC1	AQP3	CRLF1	EGFL6	FSTL3	MEX3B	RADIL	SMARCA1	P2RX7	PDPN															
CELLMARKERCAN_PULMONARY_ALVEOLAR_TYPE_II_…	PGC	CXCR2	CXCL2	GRK2	RUNX3	CTNND1	PPP1R14C	DDX3Y	PIGR	LRG1	NRN1	PPBP	IL1B	INMT	CRLF1										
CELLMARKERCAN_PURKINJE_NEURONS																									
CELLMARKERCAN_RETICULOCYTES																									
CELLMARKERCAN_RETINAL_GANGLION_CELLS																									
CELLMARKERCAN_RETINAL_PROGENITOR_CELLS																									
CELLMARKERCAN_SATELLITE_CELLS	PEG3	MSTN	SCRIB	NFATC2	IRF2	BMI1	SIX1	HOXC10	NOTCH3	HEYL	GAL	NCAM1	CAV1												
CELLMARKERCAN_SCHWANN_CELLS	SCN7A	CD82	TAGLN	NOV	ALDOC	APOD	CRYAB	PLP1	PMP22	CADM4	KCNA1	CNP	GPR37L1	NRN1	STMN1										
CELLMARKERCAN_SEROTONERGIC_NEURONS																									
CELLMARKERCAN_SERTOLI_CELLS	MRO	HSD17B3	AMHR2	SCX	HSD17B1	STIM1	ANKRD7	PHF7	CXCL12	STAR	WFDC2	PRND	HSPBAP1	NXF3	GBA2	PADI2	ABCG8	DIAPH2	CTSA	PFKFB3	MSI1	NOTCH2	GATA6	CLDN11	OCLN
CELLMARKERCAN_SMOOTH_MUSCLE_CELLS	ACTG2	MUSTN1	SOD3	HSPB6	RRAD	LMOD1	ITGA1	SPEG	EHD2	RBPMS2	MAP3K7CL	SLC38A11	GJA4	LGR6	MSRB3	AKT2	RASL12	ZBTB44	WWP2	SSPN	OTUD1	FAS	VASN	PDGFD	SMOC1
CELLMARKERCAN_STROMAL_CELLS																									
CELLMARKERCAN_T_CELLS	CD163L1	ICOS	IL18R1	TNFRSF4	CCL20	CLEC2D	CD8A	CD6	S100A4	CCL5	LCK	CD81	THY1	LAT	SKAP1	TCF7	CCR7	MYB	CCL4L2	PYHIN1	GZMA	JUNB	DUSP2	IFNG	CD52
CELLMARKERCAN_T_CYTOTOXIC_CELLS																									
CELLMARKERCAN_T_FOLLICULAR_HELPER_CELLS																									
CELLMARKERCAN_T_MEMORY_CELLS																									
CELLMARKERCAN_TRANSIENT_CELLS																									
CELLMARKERCAN_TRICHOCYTES																									
CELLMARKERCAN_TRIGEMINAL_NEURONS																									
CELLMARKERCAN_TROPHOBLAST_CELLS																									
CELLMARKERCAN_TROPHOBLAST_PROGENITOR_CELLS																									
CELLMARKERCAN_TUFT_CELLS	RAC2	PTGS1	IRF7	FFAR3	ALOX5	TSLP	IL13RA1	IL17RB	PTPRC																
CELLMARKERCANENDOTHELIALCELLSAORTA																									
CELLMARKERCUR_N_ABNORMAL_MYELOID_CELL																									

CELLMARKERCAN_ACINAR_CELLS	PNLIPRP1	CTRC	REG3A	SERPINA3	PRSS3	REG1B	CFB	GDF15	MUC1	C15ORF48	DUOXA2	AKR1C3	OLFM4	GSTA1	LGALS2	PDZK1IP1	RARRES2	CXCL17	UBD	GSTA2	ANPEP	LYZ	ANGPTL4	ALDOB	
CELLMARKERCUR_N_ABNORMAL_PLASMA_CELL																									
CELLMARKERCUR_N_ACTIVATED_DENDRITIC_CELL																									
CELLMARKERCUR_N_ACTIVATED_EFFECTOR_CELL																									
CELLMARKERCUR_N_ACTIVATED_HEPATIC_STELLAT…																									
CELLMARKERCUR_N_ACTIVATED_LANGERHANS_CELL																									
CELLMARKERCUR_N_ACTIVATED_MACROPHAGE																									
CELLMARKERCUR_N_ACTIVATED_MEMORY_T_CELL																									
CELLMARKERCUR_N_ACTIVATED_NATURAL_KILLER_…																									
CELLMARKERCUR_N_ACTIVATED_REGULATORY_T_CE…																									
CELLMARKERCUR_N_ADIPOCYTE_PROGENITOR_CELL																									
CELLMARKERCUR_N_ADRENAL_MEDULLA_CELL																									
CELLMARKERCUR_N_ADULT_SPERMATOGONIA																									
CELLMARKERCUR_N_ADVENTITIAL_CELL																									
CELLMARKERCUR_N_AEROCYTE																									
CELLMARKERCUR_N_AIRWAY_SMOOTH_MUSCLE_CELL																									
CELLMARKERCUR_N_ALVEOLAR_CELL_TYPE_1																									
CELLMARKERCUR_N_ALVEOLAR_CELL_TYPE_2																									
CELLMARKERCUR_N_ALVEOLAR_EPITHELIAL_CELL_…																									
CELLMARKERCUR_N_ALVEOLAR_EPITHELIAL_CELL_…																									
CELLMARKERCUR_N_ALVEOLAR_PNEUMOCYTE_TYPE_I																									
CELLMARKERCUR_N_ALVEOLAR_PNEUMOCYTE_TYPE_…																									
CELLMARKERCUR_N_AMACRINE_CELL																									
CELLMARKERCUR_N_AMNIOTIC_FLUID_STEM_CELL																									
CELLMARKERCUR_N_ANTERIOR_CELL																									
CELLMARKERCUR_N_ANTERIOR_HINDBRAIN_CELL																									
CELLMARKERCUR_N_ANTI_TUMOR_IMMUNE_CELL																									
CELLMARKERCUR_N_ANTIGEN_PRESENTATION_CANC…																									
CELLMARKERCUR_N_ASTROCYTE	THBS1	BMPR1B	PEA15	GLUL	FABP7	ALDOC	PCDH15	OLIG1	NDRG2	PDGFRA	ALDH1A1	VIM	PTGDS	APC	IRAG1	STOX1	GASK1B	ITGA6	LRRC3B	RYR3	CD44	CST3	ITGA1	CD109	COL12A1
CELLMARKERCUR_N_ATRIAL_CELL																									
CELLMARKERCUR_N_ATRIOVENTRICULAR_CANAL_CE…																									
CELLMARKERCUR_N_ATYPICAL_MEMORY_B_CELL																									
CELLMARKERCUR_N_B_2_CELL																									
CELLMARKERCUR_N_B1_CELL																									
CELLMARKERCUR_N_B10_REGULATORY_B_CELL																									
CELLMARKERCUR_N_BASAL_CELL	RPE65	LGALS1	ITGA6	S100A2	PDPN	NGFR	ITGB4	TP63	NPPC	LAMB3	DST	BCAM	LAMA3	DAPL1	SHOC2	NT5E	SOX2	COL4A1	ARL13B	TEAD1	CASP3	TACSTD2	KRT2	TGM3	DSC1
CELLMARKERCUR_N_BASAL_EPITHELIAL_CELL																									
CELLMARKERCUR_N_BASAL_KERATINOCYTE																									
CELLMARKERCUR_N_BASAL_LIKE_CELL																									
CELLMARKERCUR_N_BASAL_PROGENITOR_CELL																									
CELLMARKERCUR_N_BASOPHIL	TLR4	ANPEP	C5AR1	CD40LG	FCGR3A	ICAM1	IGHE	IL2RA	IL5RA	LAMP1	SPN	TLR1	TLR2	TLR6	TLR9	CCR2									
CELLMARKERCUR_N_BILIARY_CELL																									
CELLMARKERCUR_N_BONE_MARROW_STEM_CELL																									
CELLMARKERCUR_N_BONE_SPECIFIC_CELL																									
CELLMARKERCUR_N_BRAIN_CELL																									
CELLMARKERCUR_N_CANCER_CELL	CD4	AFP	DIPK1C	TNFRSF1A	TSPAN7	MTX2	BCL2	MCL1	SAMHD1	ILRUN	UHRF1BP1	PLEKHA1	CEACAM5	CDKN2A	PIK3CA	TP53	MYC	FBXW7	CD163	BSG	CD68	FGF23	AHSA1	OLIG3	BARHL1
CELLMARKERCUR_N_CANCER_STEM_LIKE_CELL																									
CELLMARKERCUR_N_CAPILLARY_CELL																									
CELLMARKERCUR_N_CAR_T_CELL																									
CELLMARKERCUR_N_CARDIAC_CELL																									
CELLMARKERCUR_N_CARDIOMYOCYTE	PECAM1	WT1	CSF1R	CD3D	TPSAB1	COL1A1	SOX9	ACTN2	ACTC1	PCM1	MYH11	HADHA	PCDH7	TTN	MYBPC3	CORIN	POPDC2	HEPACAM	MB	MYL4	IRX4	SCN5A			
CELLMARKERCUR_N_CARDIOSPHERE_DERIVED_CELL																									
CELLMARKERCUR_N_CARDIOVASCULAR_PROGENITOR…																									
CELLMARKERCUR_N_CAUDAL_CORTEX_CELL																									
CELLMARKERCUR_N_CD14_MONOCYTE																									
CELLMARKERCUR_N_CD16_MONOCYTE																									
CELLMARKERCUR_N_CD4_CD28_T_CELL																									
CELLMARKERCUR_N_CD4_PERIPHERALLY_DERIVED_…																									
CELLMARKERCUR_N_CD4_T_CELL																									
CELLMARKERCUR_N_CD56BRIGHT_NATURAL_KILLER…																									
CELLMARKERCUR_N_CD56DIM_NATURAL_KILLER_CE…																									
CELLMARKERCUR_N_CD8_T_CELL	TSC22D3	APBA2	ZFP36L2	GZMM	PF4	MAPKAP…	THUMPD1	LIME1	ZNF91	FLT3LG	GADD45A	CDKN2AIP	CAMLG	SF1	KLF9	PRR5	TLE5								
CELLMARKERCUR_N_CENTRAL_MEMORY_T_CELL	CASP8	AQP3	KMT2A	STX16	CREBZF	TIMM8A	USP9Y	CLUAP1	CYLD	ATM	CEP68	NEFL	TXK	PCNX1	DOCK9	FYB1	TXLNGY	NFATC3	MAP3K1	PCM1	EOMES	TBX21			
CELLMARKERCUR_N_CENTRAL_MEMORY_T_HELPER_1…																									
CELLMARKERCUR_N_CENTRAL_NERVOUS_SYSTEM_ST…																									
CELLMARKERCUR_N_CHANDELIER_CELL																									
CELLMARKERCUR_N_CHIEF_CELL																									
CELLMARKERCUR_N_CHONDROCYTE																									
CELLMARKERCUR_N_CHROMAFFIN_CELL																									
CELLMARKERCUR_N_CIRCULATING_ANGIOGENIC_CE…																									
CELLMARKERCUR_N_CIRCULATING_FETAL_CELL	ACTB	PUM2	MMP9	CD9	SMC3	CRIP1	IL7R	OLIG2	GATA5	F11R	DLX5	FOXA2	HAMP	NCAM1	JUNB	COL1A2									
CELLMARKERCUR_N_CIRCULATING_NATURAL_KILLE…																									
CELLMARKERCUR_N_CIRCULATING_PRECURSOR_CELL																									
CELLMARKERCUR_N_CIRCULATING_PROGENITOR_CE…																									
CELLMARKERCUR_N_CIRCULATING_T_FOLLICULAR_…																									
CELLMARKERCUR_N_CIRCULATING_TUMOR_CELL																									
CELLMARKERCUR_N_CLASS_SWITCHED_PLASMABLAST																									
CELLMARKERCUR_N_CLASSICAL_B_CELL																									
CELLMARKERCUR_N_CLASSICAL_CANCER_ASSOCIAT…																									
CELLMARKERCUR_N_CLASSICAL_MONOCYTE	HLA-DRA	GPR183	ISG15	CD74	PLBD1	IFIT2	HLA-DPB1	IFI44L	ITGA4	MX1	CYP1B1	HLA-DPA1	PADI4	EPSTI1	HLA-DQB1	XAF1	CDKN1A	LYZ	FCN1	VCAN	CD68				
CELLMARKERCUR_N_CLAUDIN_LOW_CELL																									
CELLMARKERCUR_N_COLLECTING_DUCTAL_CELL																									
CELLMARKERCUR_N_COLONCYTE																									
CELLMARKERCUR_N_COLORECTAL_STEM_CELL																									
CELLMARKERCUR_N_COLUMNAR_EPITHELIAL_CELL																									
CELLMARKERCUR_N_COMMON_LYMPHOID_PROGENITO…																									
CELLMARKERCUR_N_COMMON_MYELOID_PROGENITOR…																									
CELLMARKERCUR_N_COMPLEMENT_SECRETING_CANC…																									
CELLMARKERCUR_N_CONE_PHOTORECEPTOR_CELL																									
CELLMARKERCUR_N_CONNECTING_TUBULAR_CELL																									
CELLMARKERCUR_N_CONVENTIONAL_CD4_T_CELL																									
CELLMARKERCUR_N_CORNEAL_ENDOTHELIAL_CELL																									
CELLMARKERCUR_N_CORNEAL_EPITHELIAL_CELL																									
CELLMARKERCUR_N_CORNEOCYTE_LIKE_CELL																									
CELLMARKERCUR_N_CORTICAL_CELL																									
CELLMARKERCUR_N_CORTICAL_STROMAL_CELL																									
CELLMARKERCUR_N_CORTICOTROPH_TUMOR_CELL																									
CELLMARKERCUR_N_CRANIAL_NEURAL_CREST_CELL																									
CELLMARKERCUR_N_CRYPT_CELL																									
CELLMARKERCUR_N_CRYPT_STEM_CELL																									
CELLMARKERCUR_N_CYCLING_B_CELL																									
CELLMARKERCUR_N_CYCLING_EPITHELIAL_CELL																									
CELLMARKERCUR_N_CYTOTOXIC_CD8_T_CELL																									
CELLMARKERCUR_N_CYTOTOXIC_DOUBLE_POSITIVE…																									
CELLMARKERCUR_N_CYTOTOXIC_T_CELL	CST7	GZMK	CD4	TCF7	TIA1	MAPKAP…	CRTAM	CD5	CD7	CD34	CD38	CD200	TNF	IL7R	MME	SELP									

Table S5: Cell state marker gene list

CELLMARKERCAN_ACINAR_CELLS	PNLIPRP1	CTRC	REG3A	SERPINA3	PRSS3	REG1B	CFB	GDF15	MUC1	C15ORF48	DUOXA2	AKR1C3	OLFM4	GSTA1	LGALS2	PDZK1IP1	RARRES2	CXCL17	UBD	GSTA2	ANPEP	LYZ	ANGPTL4	ALDOB	
CELLMARKERCUR_N_CYTOTOXIC_T_CELL_2																									
CELLMARKERCUR_N_DAMAGE_ASSOCIATED_TRANSIE…																									
CELLMARKERCUR_N_DARK_ZONE_CELL																									
CELLMARKERCUR_N_DEDIFFERENTIATION_ADIPOCY…																									
CELLMARKERCUR_N_DEEP_LAYER_NEURON																									
CELLMARKERCUR_N_DEFINITIVE_ENDODERM_CELL																									
CELLMARKERCUR_N_DELTA_CELL																									
CELLMARKERCUR_N_DEMYELINATING_MICROGLIAL_…																									
CELLMARKERCUR_N_DENDRITIC_CELL_2A																									
CELLMARKERCUR_N_DENDRITIC_CELL_LINEAGE																									
CELLMARKERCUR_N_DENTAL_PULP_CELL																									
CELLMARKERCUR_N_DERMAL_CELL																									
CELLMARKERCUR_N_DEUTEROSOMAL_CELL																									
CELLMARKERCUR_N_DIFFUSE_GASTRIC_CANCER_CE…																									
CELLMARKERCUR_N_DISTAL_CELL																									
CELLMARKERCUR_N_DISTAL_STEM_CELL																									
CELLMARKERCUR_N_DISTAL_TUBULAR_CELL																									
CELLMARKERCUR_N_DIVIDING_CELL																									
CELLMARKERCUR_N_DIVIDING_PLASMA_B_CELL																									
CELLMARKERCUR_N_DIVIDING_T_CELL																									
CELLMARKERCUR_N_DOPAMINERGIC_NEURON																									
CELLMARKERCUR_N_DORSAL_ANTERIOR_HINDBRAIN…																									
CELLMARKERCUR_N_DORSAL_LATERAL_GANGLIONIC…																									
CELLMARKERCUR_N_DORSAL_MEDIAL_GANGLIONIC_…																									
CELLMARKERCUR_N_DOUBLE_NEGATIVE_B_CELL																									
CELLMARKERCUR_N_DOUBLE_NEGATIVE_T_CELL																									
CELLMARKERCUR_N_DOUBLE_POSITIVE_T_CELL																									
CELLMARKERCUR_N_DOUBLE_POSITIVE_TREG_CELL																									
CELLMARKERCUR_N_EARLY_HEMANGIOBLAST																									
CELLMARKERCUR_N_EARLY_PROXIMAL_TUBULE_CELL																									
CELLMARKERCUR_N_EARLY_RESPONSE_SECRETORY_…																									
CELLMARKERCUR_N_EFFECTOR_CHONDROCYTE																									
CELLMARKERCUR_N_EFFECTOR_MEMORY_T_CELL	EOMES	CD44	CD28	GDPD5	TBCD	DDX17	LTK	AKT3	TBC1D5	CCR2	PRKY	NFATC4	MEFV	FLI1	TRA	EZR	EWSR1	SND1-IT1	RUNX3	CXCR3	TNFRSF25	NEAT1	SAR1A		
CELLMARKERCUR_N_EFFECTOR_NATURAL_KILLER_C…																									
CELLMARKERCUR_N_ELONGATING_CELL																									
CELLMARKERCUR_N_EMBRYONIC_CELL																									
CELLMARKERCUR_N_EMBRYONIC_STEM_CELL	CGA	KHDC1L	LGALS16	PDGFRA	CCR4	CD24	TNFRSF8	ITGB1	PROM1	ABCG2	CD59	KIT	PECAM1	THY1	L1TD1	PRDM14	MGA	ZNF281	PCGF6	ZFP42	TRIM28	FIP1L1	SMC1A	KLF2	MYC
CELLMARKERCUR_N_ENDOCARDIAL_CELL																									
CELLMARKERCUR_N_ENDOCRINE_PROGENITOR_CELL																									
CELLMARKERCUR_N_ENDODERM_PROGENITOR_CELL																									
CELLMARKERCUR_N_ENDODERMAL_CELL																									
CELLMARKERCUR_N_ENDOMETRIAL_STROMAL_CELL																									
CELLMARKERCUR_N_ENDOMETRIAL_STROMAL_STEM_…																									
CELLMARKERCUR_N_ENDOTHELIAL_PRECURSOR_CELL																									
CELLMARKERCUR_N_ENSHEATHING_GLIAL_CELL																									
CELLMARKERCUR_N_ENTERIC_NEURON																									
CELLMARKERCUR_N_EOSINOPHIL	ADGRE1	PTPRC	LYZ	RETN	S100A8	S100A9	CD68	SORL1	CORO1A	EPO	CCL13	CCL5	CCL11	ACACB	TRMO	PTGDR2	ABHD2	THBS1	HRH4	KBTBD11	LRP5L	THBS4	GALC	H1-2	CYSLTR2
CELLMARKERCUR_N_EPENDYMAL_CELL																									
CELLMARKERCUR_N_EPIBLAST_CELL																									
CELLMARKERCUR_N_EPICARDIAL_CELL																									
CELLMARKERCUR_N_EPICARDIUM_DERIVED_CELL																									
CELLMARKERCUR_N_EPIDERMAL_CELL																									
CELLMARKERCUR_N_EPIDERMAL_STEM_CELL																									
CELLMARKERCUR_N_EPITHELIAL_CELL	SPRR3	PECAM1	CDH1	CD14	PTPRC	LSAMP	ATP2A2	EZR	CLIC6	LAMA1	CD44	FREM2	TRPV6	ELF3	CLDN6	ERBB3	ESRP1	CLDN4	HTR2C	PIP	ALCAM	KIT	SMN1	CHKA	ZEB1
CELLMARKERCUR_N_EPITHELIAL_DUCTAL_CELL																									
CELLMARKERCUR_N_EPITHELIAL_MESENCHYMAL_CE…																									
CELLMARKERCUR_N_EPITHELIOID_MACROPHAGE																									
CELLMARKERCUR_N_EPSILON_CELL																									
CELLMARKERCUR_N_ERYTHROBLAST																									
CELLMARKERCUR_N_ERYTHROID_CELL																									
CELLMARKERCUR_N_ERYTHROID_PROGENITOR_CELL																									
CELLMARKERCUR_N_ESOPHAGEAL_CELL																									
CELLMARKERCUR_N_ESOPHAGEAL_PROGENITOR_CELL																									
CELLMARKERCUR_N_EXCITATORY_NEURON	RBFOX1	IGFBPL1	RORB	CAMK2A	SYT1	NCAM1	ATP1A3																		
CELLMARKERCUR_N_EXHAUSTED_DOUBLE_POSITIVE…																									
CELLMARKERCUR_N_EXTRAVILLOUS_TROPHOBLAST																									
CELLMARKERCUR_N_F_PERICYTE																									
CELLMARKERCUR_N_FETAL_MYOBLAST																									
CELLMARKERCUR_N_FIBROBLAST_LIKE_CELL																									
CELLMARKERCUR_N_FIBROCHONDROCYTE																									
CELLMARKERCUR_N_FIBROCHONDROCYTE_PROGENIT…																									
CELLMARKERCUR_N_FOAM_CELL																									
CELLMARKERCUR_N_FOLLICULAR_B_CELL	CD79B	BCL6	CD27	CD80	CD86	CD40	CD81	EBF1	FOXO1	PAX5	CD24	TNFRSF9	PDCD1	PTPRC	IKZF1	ICOSLG	IL21R								
CELLMARKERCUR_N_FOLLICULAR_CELL																									
CELLMARKERCUR_N_FOLLICULAR_DENDRITIC_CELL																									
CELLMARKERCUR_N_FOREBRAIN_CELL																									
CELLMARKERCUR_N_GABAERGIC_NEURON																									
CELLMARKERCUR_N_GANGLION_CELL																									
CELLMARKERCUR_N_GASTRIC_NEUROENDOCRINE_CE…																									
CELLMARKERCUR_N_GASTRIC_STEM_CELL																									
CELLMARKERCUR_N_GERM_CELL	ACRV1	PRM2	PRM1	DMRT1	EGFR	NR6A1	MEA1	PIWIL1	TDRKH	PIWIL2	TEX11	TDRD1	KLF17	PIWIL4	SCARA5	TEX19	FABP9	KIT	RBMY1A1						
CELLMARKERCUR_N_GERMINAL_CENTER_B_CELL	SUGCT	RFTN1	BCL7A	HMGN2	TCL1A	EZR	ACTG1	SERF2	LPP	GAPDH	HMGN1	CD19	CD38	CD9	MKI67	CD81	BCL6	IRF8	PAX5	BACH2	PTPRC	FOXO1	MEF2B	MME	
CELLMARKERCUR_N_GERMINAL_CENTER_CELL																									
CELLMARKERCUR_N_GERMINAL_CENTRE_B_CELL																									
CELLMARKERCUR_N_GLIAL_CELL	CDH5	SOX10	CCL11	ACKR1	RBP7	TAGLN	RGS5	CLEC9A	GFRA3	RXRG	APOE	PHOX2B	LAMP5	ZFP36	CCN2	ENTPD2	BCAN	FGL2	HMGA2	SPRY1	CD14	FGFR3	FABP7	PTPRC	CD44
CELLMARKERCUR_N_GLIOMA_STEM_CELL																									
CELLMARKERCUR_N_GLOBOSE_BASAL_CELL																									
CELLMARKERCUR_N_GLOMERULAR_PARIETAL_EPITH…																									
CELLMARKERCUR_N_GLUTAMATERGIC_NEURON																									
CELLMARKERCUR_N_GOBLET_CELL	CLCA1	MYC	PTGDR	PBK	MKI67	TRABD2A	ATOH1	UBE2C	CA1	FCGBP	MUC4	ITLN1	XBP1	PIGR	VMO1	HES6									
CELLMARKERCUR_N_GONADOTROPE																									
CELLMARKERCUR_N_GONADOTROPH_CELL																									
CELLMARKERCUR_N_GRANULOCYTE	CD24	S100A9	S100A8	ANPEP	FCGR2A	LILRA5																			
CELLMARKERCUR_N_GRANULOCYTE_MONOCYTE_PROG…																									
CELLMARKERCUR_N_GRANULOCYTIC_UNK																									
CELLMARKERCUR_N_GRANULOSA_CELL																									
CELLMARKERCUR_N_HAEMATOPOIETIC_CELL																									
CELLMARKERCUR_N_HAIR_FOLLICLE_CELL																									
CELLMARKERCUR_N_HEMANGIOBLAST																									
CELLMARKERCUR_N_HEMATOPOIETIC_CELL	SOX8	CD55	CD40	CD14	THY1	IL3RA	TFRC	CD109	PECAM1	CCL5	CD69	CST7													
CELLMARKERCUR_N_HEMATOPOIETIC_PRECURSOR_C…																									
CELLMARKERCUR_N_HEMATOPOIETIC_PROGENITOR_…	THY1	SPINK2	MPL	VWF	MMRN1	KDR	CASR																		
CELLMARKERCUR_N_HEPATIC_CELL																									
CELLMARKERCUR_N_HEPATIC_PROGENITOR_CELL																									

Table S5: Cell state marker gene list

CELLMARKERCAN_ACINAR_CELLS	PNLIPRP1	CTRC	REG3A	SERPINA3	PRSS3	REG1B	CFB	GDF15	MUC1	C15ORF48	DUOXA2	AKR1C3	OLFM4	GSTA1	LGALS2	PDZK1IP1	RARRES2	CXCL17	UBD	GSTA2	ANPEP	LYZ	ANGPTL4	ALDOB	
CELLMARKERCUR_N_HEPATIC_STELLATE_CELL																									
CELLMARKERCUR_N_HEPATOBLAST																									
CELLMARKERCUR_N_HEPATOCYTE	FXYD1	GJB1	PPARG	SMAD1	NR1H2	TGM2	FGB	GATA6	A2M	GATA4	HAVCR1	DUOX2	FOXA1												
CELLMARKERCUR_N_HISTIOCYTE																									
CELLMARKERCUR_N_HMSC_TREATED_B_CELL																									
CELLMARKERCUR_N_HOFBAUER_CELL																									
CELLMARKERCUR_N_HOMEOSTATIC_CHONDROCYTE	TOB1	ZNF395	PNRC1	ZFP36L2	ADM	WSB1	H2AX	MASTL	TRA2A	ISM1	WWP2	INTS6	RHOB	RSRP1	ID1	PPP1R10	PPP1R1…	SNHG1	MYLIP	SNHG12	C6orf62	RPPH1	CLK1	ARRDC3	BRD2
CELLMARKERCUR_N_HOMEOSTATIC_MICROGLIAL_CE…																									
CELLMARKERCUR_N_HORIZONTAL_BASAL_CELL																									
CELLMARKERCUR_N_HORIZONTAL_CELL																									
CELLMARKERCUR_N_HYPOBLAST_CELL																									
CELLMARKERCUR_N_IDIOPATHIC_PULMONARY_FIBR…																									
CELLMARKERCUR_N_IGA_MEMORY_B_CELL																									
CELLMARKERCUR_N_IGG_MEMORY_B_CELL																									
CELLMARKERCUR_N_IGM_MEMORY_B_CELL																									
CELLMARKERCUR_N_IGM_ONLY_B_CELL																									
CELLMARKERCUR_N_IL7R_T_HELPER_CELL																									
CELLMARKERCUR_N_IMMATURE_ERYTHROID_CELL																									
CELLMARKERCUR_N_IMMATURE_MYELOID_CELL																									
CELLMARKERCUR_N_IMMATURE_NEUTROPHIL																									
CELLMARKERCUR_N_IMMATURE_TRANSITIONAL_B_C…																									
CELLMARKERCUR_N_IMMUNE_CELL	KLRF1	S100A4	CD3E	MAPK14	IL6	IL27	TSLP	TNF	CCL24	CCL2	IL18	CCL5	PDCD1	JCHAIN	KIT	CD247	MSR1	GNLY	VCAN	PPARG	CD74	MKI67	MZB1	IRF8	IL7R
CELLMARKERCUR_N_INDUCED_PLURIPOTENT_STEM_…	FUT4	ITGA6	ITGB1	TACSTD2	POU5F1	LEFTY2	TPBG	VSIR	TRIM71																
CELLMARKERCUR_N_INFILTRATING_MACROPHAGE																									
CELLMARKERCUR_N_INFLAMMATORY_CANCER_ASSOC…																									
CELLMARKERCUR_N_INFLAMMATORY_DENDRITIC_CE…																									
CELLMARKERCUR_N_INFLAMMATORY_MONOCYTE																									
CELLMARKERCUR_N_INHIBITORY_NEURON	TH	TAC1	PDYN	SYT1	DLX6-A…	RSPO3	PCP4																		
CELLMARKERCUR_N_INNATE_LYMPHOID_CELL	GNLY	SOX4	ST2	CD27	PTPRC	NCR3																			
CELLMARKERCUR_N_INNER_CELL_MASS_CELL																									
CELLMARKERCUR_N_INNER_RADIAL_GLIAL_CELL																									
CELLMARKERCUR_N_INTER_MEDIATE_PROGENITOR_…																									
CELLMARKERCUR_N_INTERCALATED_CELL																									
CELLMARKERCUR_N_INTERFERON_RESPONSIVE_SEC…																									
CELLMARKERCUR_N_INTERMEDIATE_MONOCYTE																									
CELLMARKERCUR_N_INTERMEDIATE_MONOCYTE_CELL																									
CELLMARKERCUR_N_INTERSTITIAL_MACROPHAGE																									
CELLMARKERCUR_N_INTERSTITIAL_PROGENITOR_C…																									
CELLMARKERCUR_N_INTESTINAL_EPITHELIAL_STE…																									
CELLMARKERCUR_N_INTESTINAL_GASTRIC_CANCER…																									
CELLMARKERCUR_N_INTESTINAL_METAPLASIA_CELL																									
CELLMARKERCUR_N_INTRAEPITHELIAL_LYMPHOCYT…																									
CELLMARKERCUR_N_IONOCYTE																									
CELLMARKERCUR_N_IRIS_PUTTATIVE_STEM_CELL																									
CELLMARKERCUR_N_KUPFFER_CELL																									
CELLMARKERCUR_N_LACTOTROPH_CELL																									
CELLMARKERCUR_N_LANGERHANS_CELL																									
CELLMARKERCUR_N_LEUKOCYTE	CD8A	EOMES	IFNG	CD27	IL7R	CD28	CD96	CD2	CD40	CD86	CD53	LSP1	CORO1A	LST1	ALCAM	CD163	TREM1	CX3CR1	CD48	CD80	FCGR1A	TLR4	FCGR3B	CTSS	GNLY
CELLMARKERCUR_N_LIMBAL_EPITHELIAL_STEM_CE…																									
CELLMARKERCUR_N_LIMBAL_STEM_CELL																									
CELLMARKERCUR_N_LIPOFIBROBLAST																									
CELLMARKERCUR_N_LIVER_PROGENITOR_CELL																									
CELLMARKERCUR_N_LIVER_STEM_CELL																									
CELLMARKERCUR_N_LMMLEYDIG_CELL																									
CELLMARKERCUR_N_LONOCYTE																									
CELLMARKERCUR_N_LOOP_OF_HENLE_CELL																									
CELLMARKERCUR_N_LOOP_PROGENITOR_CELL																									
CELLMARKERCUR_N_LOW_DENSITY_NEUTROPHIL																									
CELLMARKERCUR_N_LUMINAL_PROGENITOR_CELL																									
CELLMARKERCUR_N_LUNG_CELL																									
CELLMARKERCUR_N_LYMPH_NODE_CELL																									
CELLMARKERCUR_N_LYMPHATIC_CELL																									
CELLMARKERCUR_N_LYMPHATIC_ENDOTHELIAL_CELL																									
CELLMARKERCUR_N_LYMPHOBLASTOID_CELL																									
CELLMARKERCUR_N_LYMPHOCYTE	IL32	CD3E	CD7	IL2RG	IL7R	CD96	CD274	LYVE1	TRAC	LAG3	CXCL13	TOX	CD52	HOPX	NCR3	ZBTB16	CD24	CD38	PDPN	PROX1	TEK	NR2F1	GPR182	NR2F2	KDR
CELLMARKERCUR_N_LYMPHOID_CELL	CD244	FASLG	CCR4	CCR6	FCGR3A	IL2RB	IL12RB1	ITGAE	KIT	KLRB1	KLRD1	LY9	MME	SELL	SPN	ST2	IL18R1	NCR2	NCR1	KLRK1	IL17RA	B3GAT1	SLAMF7	NCR3	
CELLMARKERCUR_N_LYMPHOID_PROGENITOR_CELL																									
CELLMARKERCUR_N_LYMPHOID_STRUCTURE_CELL																									
CELLMARKERCUR_N_M_PERICYTE																									
CELLMARKERCUR_N_M1_MACROPHAGE	NOS2	IL12A	IRF5	SOCS3	CXCL9	CD38	IL1B	NFKB1	CD83	VSIR	CXCL11	STAT1	CXCL12	CXCL8	FCGR1CP	IL6	HLA-DRA	IL12B	IL23A	IRF1	IL1A	IFI27	FOS	S100A8	BCL2A1
CELLMARKERCUR_N_M1_MICROGLIAL_CELL																									
CELLMARKERCUR_N_M2_MACROPHAGE	CLEC10A	CSF1R	VSIG4	CCL3	MARCO	NRP2	CD276	MERTK	CXCL10	STAB1	IL6	CD200R1	TLR1	TLR8	VSIR	SIGLEC1	TREM2	C1QB	FGL2	C1QC	AXL	C1QA	S100A9	CCL18	FCGR3A
CELLMARKERCUR_N_MACROGLIAL_CELL																									
CELLMARKERCUR_N_MALIGNANT_CELL																									
CELLMARKERCUR_N_MALIGNANT_PLASMA_CELL																									
CELLMARKERCUR_N_MARGINAL_ASSOCIATED_MACRO…																									
CELLMARKERCUR_N_MAST_CELL	ACE	LY75	CD36	CEACAM5	IL9R	CLEC12A	SUSD2	BST2	ALOX5	CD34	LTC4S	ALCAM	TNFRSF…	CD164	BTN3A2	FZD4	NECTIN1	ABCB1	CSF2RB	PTPRJ	CEACAM6	ITGA1	CSF1R	ALOX5AP	IL1RL1
CELLMARKERCUR_N_MATURE_ASTROCYTE																									
CELLMARKERCUR_N_MATURE_B_CELL																									
CELLMARKERCUR_N_MATURE_DENDRITIC_CELL																									
CELLMARKERCUR_N_MATURE_ENDOTHELIAL_CELL																									
CELLMARKERCUR_N_MATURE_LUMINAL_CELL																									
CELLMARKERCUR_N_MATURE_NEURON																									
CELLMARKERCUR_N_MATURE_NEURONAL																									
CELLMARKERCUR_N_MATURE_OLIGODENDROCYTE																									
CELLMARKERCUR_N_MATURE_OSTEOBLAST																									
CELLMARKERCUR_N_MATURE_PODOCYTE																									
CELLMARKERCUR_N_MEDIAL_AND_DISTAL_SEGMENT…																									
CELLMARKERCUR_N_MEDIAL_GANGLIONIC_EMINENC…																									
CELLMARKERCUR_N_MEGAKARYOCYTE	SRGN	FYB1	GMFG	PTPRC	OST4	CPA3	TPSB2	GP5	IL3RA	ITGA6	ITGAV	MPL	PECAM1	NECTIN2	POU5F1	SOX2	CXCR4	CST3							
CELLMARKERCUR_N_MEGAKARYOCYTE_ERYTHROID_C…	ANPEP	FASLG	CXCR5	CD8A	ENTPD1	CCR3	CCR4	CCR5	CCR6	CTLA4	FCER2	FCGR3A	IL2RA	IL2RB	IL6R	IL6ST	IL7R	IL9	IL12RB1	ITGAE	KLRB1	KLRD1	LY9	MME	NCAM1
CELLMARKERCUR_N_MEGAKARYOCYTE_ERYTHROID_P…																									
CELLMARKERCUR_N_MEGAKARYOCYTE_PROGENITOR_…																									
CELLMARKERCUR_N_MELANOMA_CELL																									
CELLMARKERCUR_N_MEMORY_B_CELL	KIR2DL1	CD22	ARID1A	FAS	QPCT	CD24	TSC2	SMAD4	JCHAIN	CEACAM21	CCR2	CD44	CCR6	CD72	RB1	TCL1A	CCR7	GPR183	SELL	TLR1	TLR2	TLR6	TLR7	TLR10	RBPJ
CELLMARKERCUR_N_MEMORY_B_CELL_PRECURSOR																									
CELLMARKERCUR_N_MEMORY_CD27_B_CELL																									
CELLMARKERCUR_N_MEMORY_CD4_T_CELL																									
CELLMARKERCUR_N_MEMORY_DOUBLE_POSITIVE_T_…																									
CELLMARKERCUR_N_MEMORY_REGULATORY_T_CELL																									
CELLMARKERCUR_N_MEMORY_T_CELL	CD274	CD28	CD3E	CXCR6	ENTPD1	GZMK																			
CELLMARKERCUR_N_MERKEL_CELL																									
CELLMARKERCUR_N_MESENCHYMAL_CELL	FBN1	PLAT	TGFBR2	SPARC	CTSC	FBLN5	MMP2	CD248	CD59	TWIST1	MSN	FAP	PDGFRB	MCAM	ECM1	CXCL12	DLK1	TWIST2	WT1	HAND1	DCN	COL3A1	PDGFRA	COL1A2	CCL21
CELLMARKERCUR_N_MESENCHYMAL_EPITHELIAL_TR…																									

Table S5: Cell state marker gene list

CELLMARKERCAN_ACINAR_CELLS	PNLIPRP1	CTRC	REG3A	SERPINA3	PRSS3	REG1B	CFB	GDF15	MUC1	C15ORF48	DUOXA2	AKR1C3	OLFM4	GSTA1	LGALS2	PDZK1IP1	RARRES2	CXCL17	UBD	GSTA2	ANPEP	LYZ	ANGPTL4	ALDOB	
CELLMARKERCUR_N_MESENCHYMAL_GLIOBLASTOMA_…																									
CELLMARKERCUR_N_MESENCHYMAL_STEM_CELL	POU5F1	MCAM	ITGA11	CMAHP	CASP3	SUSD2	CD4	ITGA6	CXCR4	ISLR	EPCAM	PDGFRA	CXCL12	PROM1	NOTCH4	NOTCH1	CD163	CDH5	CD74	CD53	ADGRE5	CD40	BSG	VIM	ALDH1A1
CELLMARKERCUR_N_MESENCHYMAL_STROMAL_CELL	LEPR	CXCL12	VCAM1	ITGAV	NES	RGS5	CD9	ICAM1	ADGRE5	ITGB2	FZD9	IL4R	ITGA1	CDH1	CD55	TFRC	TNFRSF…	ITGA5	NCAM1	ERBB2	ITGA6	CD58	ITGB4	ITGB3	CD99
CELLMARKERCUR_N_MESENDODERMAL_CELL																									
CELLMARKERCUR_N_MESODERMAL_EMBRYONIC_STEM…																									
CELLMARKERCUR_N_MESODERMAL_PROGENITOR_CELL																									
CELLMARKERCUR_N_METAPLASTIC_STEM_CELL																									
CELLMARKERCUR_N_MEYLOID_CELL																									
CELLMARKERCUR_N_MICROGLIAL_CELL	APBB1IP	CSF1R	TNF	DPP4	GFAP	TYROBP	C3	TLR2	IL1A	C1QA	LAPTM5	C1QB	HLA-DRA	MKI67	HLA-B	SLC2A5	P2RY13	MARCKS	CD40	NOS2	FCGR1A	CD14	CSF3R	PLXDC2	CYBB
CELLMARKERCUR_N_MICROVILLAR_CELL																									
CELLMARKERCUR_N_MIDBRAIN_DOPAMINERGIC_NEU…																									
CELLMARKERCUR_N_MONO_MACROPHAGE																									
CELLMARKERCUR_N_MONOCYTE_DERIVED_CELL																									
CELLMARKERCUR_N_MONOCYTE_DERIVED_MYELOID_…																									
CELLMARKERCUR_N_MONOCYTE_LINEAGE																									
CELLMARKERCUR_N_MONOCYTE_PRECURSOR																									
CELLMARKERCUR_N_MONOCYTE_PROGENITOR_CELL																									
CELLMARKERCUR_N_MUELLER_GLIA_CELL																									
CELLMARKERCUR_N_MULTIPOTENT_MESENCHYMAL_S…																									
CELLMARKERCUR_N_MULTIPOTENT_RETINAL_PROGE…																									
CELLMARKERCUR_N_MULTIPOTENT_STEM_CELL																									
CELLMARKERCUR_N_MULTIPOTENT_STROMAL_CELL																									
CELLMARKERCUR_N_MUSCLE_CELL																									
CELLMARKERCUR_N_MUSCLE_DERIVED_CELL																									
CELLMARKERCUR_N_MUSCLE_SATELLITE_CELL																									
CELLMARKERCUR_N_MYELOBLAST																									
CELLMARKERCUR_N_MYELOID_DENDRITIC_CELL	LAMP3	CD1E	CLEC9A	CLIC2	WFDC21P	CD1A	CD1B																		
CELLMARKERCUR_N_MYELOID_DENDRITIC_CELL_1																									
CELLMARKERCUR_N_MYELOID_DERIVED_CELL																									
CELLMARKERCUR_N_MYELOID_DERIVED_SUPPRESSO…	TREM1	S100A9	S100A8	FOLR2	CEACAM8	FLT1	FUT4	IL4R																	
CELLMARKERCUR_N_MYELOID_PROGENITOR_CELL																									
CELLMARKERCUR_N_MYO_EPITHELIAL_CELL																									
CELLMARKERCUR_N_MYOBLAST	MEF2D	ACTN1	SIX4	PITX3	FOXO4	MSTN																			
CELLMARKERCUR_N_MYOCYTE																									
CELLMARKERCUR_N_MYOFIBROBLAST	STX2	GATA6	PCLAF	GINS2	TYMS	ADAMDE…	GREM2	TOP2A	HPGD	ADAM28	SOX6	MKI67	MYH11	MFAP4	RBP1	MYO1D	MYO10	NEBL	RYR2	ITGA8	CD68	PDGFRA	ELN	RGS5	MMP14
CELLMARKERCUR_N_MYOGENIC_ENDOTHELIAL_CELL																									
CELLMARKERCUR_N_MYOGENIC_PROGENITOR_CELL																									
CELLMARKERCUR_N_MYOID_CELL																									
CELLMARKERCUR_N_NAIVE_B_CELL	PTPRC	CD37	CD74	MME	CD83	CD22	CD72	CCR7	SELL																
CELLMARKERCUR_N_NAIVE_CD8_T_CELL																									
CELLMARKERCUR_N_NAIVE_DOUBLE_POSITIVE_T_C…																									
CELLMARKERCUR_N_NAIVE_PROGENITOR_CELL																									
CELLMARKERCUR_N_NAIVE_T_CELL																									
CELLMARKERCUR_N_NASCENT_NEPHRON_CELL																									
CELLMARKERCUR_N_NATURAL_KILLER_CD56_BRIGH…																									
CELLMARKERCUR_N_NATURAL_KILLER_CD56_DIM_C…																									
CELLMARKERCUR_N_NEGATIVE_MESENCHYMAL_STEM…																									
CELLMARKERCUR_N_NEURAL_CELL																									
CELLMARKERCUR_N_NEURAL_CREST_STEM_CELL																									
CELLMARKERCUR_N_NEURAL_CREST_STEMLIKE_CELL																									
CELLMARKERCUR_N_NEURAL_PRECURSOR_CELL																									
CELLMARKERCUR_N_NEURAL_STEM_CELL	ID1	NES	LIFR	FABP7	IL6ST	HOPX	MAP2	SOX10	RUNX1	FGFR2	FGFR4	GATA2	NR6A1	GFAP	HOXB1	ID2	MSX1	NOTCH2	ROR2	OTX2	PAX3	SOX11	NR2F1	ZIC1	SOX21
CELLMARKERCUR_N_NEUROECTODERM_CELL																									
CELLMARKERCUR_N_NEURONAL_INTERMEDIATE_PRO…																									
CELLMARKERCUR_N_NEURONAL_PROGENITOR_CELL																									
CELLMARKERCUR_N_NEUTROPHIL_PROGENITOR_CELL																									
CELLMARKERCUR_N_NON_NEURON																									
CELLMARKERCUR_N_NON_PT_TUBULAR_CELL																									
CELLMARKERCUR_N_NON_RECIRCULATING_TISSUE_…																									
CELLMARKERCUR_N_NONMYELINATING_SCHWANN_CE…																									
CELLMARKERCUR_N_NOTOCHORD_CELL																									
CELLMARKERCUR_N_NUCLEATED_CELL																									
CELLMARKERCUR_N_NUCLEUS_PULPOSUS_PROGENIT…																									
CELLMARKERCUR_N_OLIGODENDROCYTE	VCAN	APC	GRIA1	GRIA2	MYT1	ZNF488	PRKCQ	GPR62	ENPP6	SGK2	MAL	GALNT6	PPP1R1…	GPR17	PLEKHH1	FA2H	EVI2A	ERBB3	RFFL	BCAS1	PDGFRA	DLL3	ERMN	EDIL3	CNDP1
CELLMARKERCUR_N_OSTEOBLAST	TOP2A	COL2A1	SOX9	ALPP	SPP1	BGLAP																			
CELLMARKERCUR_N_OUTER_RADIAL_GLIA																									
CELLMARKERCUR_N_OVALBUMIN_SPECIFIC_REGULA…																									
CELLMARKERCUR_N_OVARIAN_CELL																									
CELLMARKERCUR_N_OVARIAN_STROMAL_CELL																									
CELLMARKERCUR_N_P53_LIKE_CELL																									
CELLMARKERCUR_N_PAN_B_CELL																									
CELLMARKERCUR_N_PAN_CANCER_ASSOCIATED_FIB…																									
CELLMARKERCUR_N_PAN_MACROPHAGE																									
CELLMARKERCUR_N_PAN_MONOCYTE																									
CELLMARKERCUR_N_PAN_NEURONAL_CELL																									
CELLMARKERCUR_N_PAN_TROPHECTODERM_CELL																									
CELLMARKERCUR_N_PANCREATIC_ENDODERM_CELL																									
CELLMARKERCUR_N_PANCREATIC_POLYPEPTIDE_CE…																									
CELLMARKERCUR_N_PANCREATIC_PROGENITOR_CELL																									
CELLMARKERCUR_N_PANCREATIC_STEM_CELL																									
CELLMARKERCUR_N_PANETH_CELL																									
CELLMARKERCUR_N_PANNEURON																									
CELLMARKERCUR_N_PANVASCULAR_CELL																									
CELLMARKERCUR_N_PAPILLARY_FIBROBLAST																									
CELLMARKERCUR_N_PARIETAL_CELL																									
CELLMARKERCUR_N_PARVALBUMIN_INTERNEURON																									
CELLMARKERCUR_N_PATELET																									
CELLMARKERCUR_N_PATHOGENIC_T_PERIPHERAL_H…																									
CELLMARKERCUR_N_PELVIC_EPITHELIAL_CELL																									
CELLMARKERCUR_N_PERICYTE	CD19	KAT14	COL1A1	COL1A2	COL3A1	DCN	BMP4	FOXC2	TAGLN	MYH11	MYL9	THBS1	TYMS	THBS4	WNT6	NFKBIA	MKI67	TOP2A	CEBPD	MBP	CENPF	BMPER	GPAT2	CYGB	GINS2
CELLMARKERCUR_N_PERIODONTAL_LIGAMENT_STEM…																									
CELLMARKERCUR_N_PERIOSTEUM_DERIVED_PROGEN…																									
CELLMARKERCUR_N_PERITUBULAR_MYOID_CELL																									
CELLMARKERCUR_N_PERIVASCULAR_LIKE_CELL																									
CELLMARKERCUR_N_PHAGOCYTE																									
CELLMARKERCUR_N_PHOTORECEPTOR_CELL																									
CELLMARKERCUR_N_PIT_CELL																									
CELLMARKERCUR_N_PIT_MUCOUS_CELL																									
CELLMARKERCUR_N_PITUITARY_STEM_CELL																									
CELLMARKERCUR_N_PLACENTAL_TISSUE_CELL																									
CELLMARKERCUR_N_PLACODE_CELL																									
CELLMARKERCUR_N_PLASMABLAST_B_CELL																									
CELLMARKERCUR_N_PLATELET	LAMP2	PECAM1	SELP	SPN	VEGFA	TNFSF14	CD40LG	CD14	PPBP	CXCR1	GATA2	CD274	GP9	LAP	FCER2	GP5	LAMP1	MPL	SLC44A1						
CELLMARKERCUR_N_PLURIPOTENT_STEM_CELL	LIN28A	GPC3	NECTIN2	PECAM1	PRSS50	CDH11	CD9																		

CELLMARKERCAN_ACINAR_CELLS	PNLIPRP1	CTRC	REG3A	SERPINA3	PRSS3	REG1B	CFB	GDF15	MUC1	C15ORF48	DUOXA2	AKR1C3	OLFM4	GSTA1	LGALS2	PDZK1IP1	RARRES2	CXCL17	UBD	GSTA2	ANPEP	LYZ	ANGPTL4	ALDOB	
CELLMARKERCUR_N_PODOCYTE_PRECURSOR																									
CELLMARKERCUR_N_PORTAL_FIBROBLAST																									
CELLMARKERCUR_N_POSTERIOR_CELL																									
CELLMARKERCUR_N_PRE_B_CELL																									
CELLMARKERCUR_N_PRE_CONVENTIONAL_DENDRITI…																									
CELLMARKERCUR_N_PRE_DENDRITIC_CELL																									
CELLMARKERCUR_N_PRECURSOR_CELL																									
CELLMARKERCUR_N_PREHYPERTROPHIC_CHONDROCY…	ABI3BP	SLC7A2	COL15A1	PART1	STEAP1	DPT	NT5E	AKR1C2	COL3A1	S100A4	CAPS	AOC2	MMP1	COL11A1	COL9A3	SOX9									
CELLMARKERCUR_N_PRESOMITE_CELL																									
CELLMARKERCUR_N_PRETUBULAR_AGGREGATE_CELL																									
CELLMARKERCUR_N_PRIMITIVE_ENDODERM_CELL																									
CELLMARKERCUR_N_PRINCIPAL_CELL																									
CELLMARKERCUR_N_PRO_B_CELL																									
CELLMARKERCUR_N_PRO_INFLAMMATORY_MACROPHA…																									
CELLMARKERCUR_N_PRO_NEUTROPHIL																									
CELLMARKERCUR_N_PROLIFERATING_CELL																									
CELLMARKERCUR_N_PROLIFERATIVE_CHONDROCYTE	PDCD11	UPP1	RFLNB	SMOX	RYBP	SLC4A7	P3H2	MTHFD2	TGFB1	DDX21	TXNRD1	SNX9													
CELLMARKERCUR_N_PROLIFERATIVE_DUCTAL_CELL																									
CELLMARKERCUR_N_PROLIFERATIVE_NEURAL_PROG…																									
CELLMARKERCUR_N_PRONEURAL_GLIOBLASTOMA_CE…																									
CELLMARKERCUR_N_PROSPERMATOGONIA																									
CELLMARKERCUR_N_PROSTATE_CELL																									
CELLMARKERCUR_N_PROXIMAL_CELL																									
CELLMARKERCUR_N_PROXIMAL_SEGMENT_CELL																									
CELLMARKERCUR_N_PROXIMAL_STEM_CELL																									
CELLMARKERCUR_N_PROXIMAL_TUBULE_CELL																									
CELLMARKERCUR_N_PULMONARY_NEUROENDOCRINE_…																									
CELLMARKERCUR_N_PYRAMIDAL_CELL																									
CELLMARKERCUR_N_QUIESCENT_T_STEM_CELL																									
CELLMARKERCUR_N_REGULATORY_B_CELL																									
CELLMARKERCUR_N_REGULATORY_INNATE_LYMPHOI…																									
CELLMARKERCUR_N_RENAL_TUBULAR_CELL																									
CELLMARKERCUR_N_RESIDENT_MEMORY_T_CELL																									
CELLMARKERCUR_N_RESPIRATORY_HORIZONTAL_BA…																									
CELLMARKERCUR_N_RESPIRATORY_SECRETORY_CELL																									
CELLMARKERCUR_N_RESPONDER_T_CELL																									
CELLMARKERCUR_N_RESPONDING_CONVENTIONAL_T…																									
CELLMARKERCUR_N_RESTING_MEMORY_B_CELL																									
CELLMARKERCUR_N_RESTING_NAIVE_B_CELL																									
CELLMARKERCUR_N_RESTING_REGULATORY_T_CELL																									
CELLMARKERCUR_N_RETICULAR_FIBROBLAST																									
CELLMARKERCUR_N_RETINA_CELL																									
CELLMARKERCUR_N_ROD_CELL																									
CELLMARKERCUR_N_SATELLITE_CELL																									
CELLMARKERCUR_N_SEPTUM_TRANSVERSUM_MESENC…																									
CELLMARKERCUR_N_SEROUS_CELL																									
CELLMARKERCUR_N_SIDE_POPULATION_CELL																									
CELLMARKERCUR_N_SKELETAL_MUSCLE_CELL																									
CELLMARKERCUR_N_SKELETAL_MUSCLE_STEM_CELL																									
CELLMARKERCUR_N_SMOOTH_MUSCLE_CELL	COX4I2	GJA4	CRYAB	ADIRF	MT1A	CSRP2	SMN1	MMP2	SMTN	ACTN2	MYH2	MYL2	PRLR	SFRP4											
CELLMARKERCUR_N_SMOOTH_MUSCLE_LIKE_CELL																									
CELLMARKERCUR_N_SOMATIC_CELL																									
CELLMARKERCUR_N_SOMATIC_STEM_CELL																									
CELLMARKERCUR_N_SOMATOSTATIN_INTERNEURON																									
CELLMARKERCUR_N_SPECIALIST_ANTIGEN_PRESEN…	KIT	LY75	MME	MRC1	NT5E	SIGLEC1	THBD	TLR2	VCAM1	NRP1	LILRA4	TLR9	SLAMF7	CLEC4C	CCR2										
CELLMARKERCUR_N_SPERMATOGENIC_CELL																									
CELLMARKERCUR_N_SPERMATOGONIAL_CELL	KIT	PIWIL4	FGFR3	C19orf84	GFRA1	CD9	TSPAN33	ZBTB16																	
CELLMARKERCUR_N_SPERMATOGONIAL_STEM_CELL																									
CELLMARKERCUR_N_SQUAMOUS_DIFFERENTIATION_…																									
CELLMARKERCUR_N_STELLATE_CELL																									
CELLMARKERCUR_N_STEM_CELL_DERIVED_ALPHA_C…																									
CELLMARKERCUR_N_STEM_CELL_DERIVED_BETA_CE…																									
CELLMARKERCUR_N_STEM_CELL_DERIVED_ENDOTHE…																									
CELLMARKERCUR_N_STEM_LIKE_CD8_T_CELL																									
CELLMARKERCUR_N_STROMAL_CELL	VIM	COL1A2	DCN	FBLN1	COL1A1	GJA1	HES4	FOXC2	S100B	WNT5B	CHI3L1	BMP4	MADCAM1	CD68	CD36	RGS5	RBP7	SOX17	VWA1	CDH5	ACKR1	XCR1	WNT2B	CLEC9A	SOX10
CELLMARKERCUR_N_SUBPALLIUM_CELL																									
CELLMARKERCUR_N_SUBPLATE_CELL																									
CELLMARKERCUR_N_SUPPRESSIVE_MONOCYTE																									
CELLMARKERCUR_N_SUPPRESSOR_T_CELL																									
CELLMARKERCUR_N_SUPRABASAL_CELL																									
CELLMARKERCUR_N_SURFACE_ECTODERM_CELL																									
CELLMARKERCUR_N_SUSTENTACULAR_CELL																									
CELLMARKERCUR_N_SYMPATHOADRENAL_CELL																									
CELLMARKERCUR_N_SYNCYTIOTROPHOBLAST																									
CELLMARKERCUR_N_SYNTHETIC_SMOOTH_MUSCLE_C…																									
CELLMARKERCUR_N_T_CELL_LARGE_GRANULAR_LYM…																									
CELLMARKERCUR_N_T_CELL_LINEAGE																									
CELLMARKERCUR_N_T_HELPER_CELL	CD200	FASLG	ENTPD1	CCR3	CCR5	IL2RA	IL6R	IL9	IL12RB1	ITGAE	KLRB1	MME	NT5E	SELP	IL18R1	PTGDR2									
CELLMARKERCUR_N_T_MEMORY_STEM_CELL																									
CELLMARKERCUR_N_T2_MZP_REGULATORY_B_CELL																									
CELLMARKERCUR_N_TENDON_CELL																									
CELLMARKERCUR_N_TERMINAL_DIFFERENTIATED_B…																									
CELLMARKERCUR_N_THALAMIC_CELL																									
CELLMARKERCUR_N_THECA_INTERNA_CELL																									
CELLMARKERCUR_N_THYMIC_EPITHELIAL_CELL																									
CELLMARKERCUR_N_TISSUE_LIKE_MEMORY_B_CELL																									
CELLMARKERCUR_N_TISSUE_RESIDENT_CELL																									
CELLMARKERCUR_N_TISSUE_RESIDENT_EFFECTOR_…																									
CELLMARKERCUR_N_TISSUE_RESIDENT_MACROPHAGE																									
CELLMARKERCUR_N_TISSUE_RESIDENT_MEMORY_LI…																									
CELLMARKERCUR_N_TOLDC_INDUCED_REGULATORY_…																									
CELLMARKERCUR_N_TONSIL_CELL																									
CELLMARKERCUR_N_TRABECULAR_MESHWORK_CELL																									
CELLMARKERCUR_N_TRANSITIONAL_B_CELL																									
CELLMARKERCUR_N_TRANSITIONAL_DENDRITIC_CE…																									
CELLMARKERCUR_N_TRUNCATED_RADIAL_GLIAL_CE…																									
CELLMARKERCUR_N_TUBULAR_CELL																									
CELLMARKERCUR_N_TUMOR_ASSOCIATED_MICROGLI…																									
CELLMARKERCUR_N_TUMOR_ENDOTHELIAL_CELL																									
CELLMARKERCUR_N_TUMOR_EPITHELIAL_CELL																									
CELLMARKERCUR_N_TUMOR_INFILTRATING_REGULA…																									
CELLMARKERCUR_N_TUMOR_INFILTRATING_T_CELL																									
CELLMARKERCUR_N_TUMOR_INITIATING_CELL																									
CELLMARKERCUR_N_TUMOR_REGULATORY_T_CELL																									

Table S5: Cell state marker gene list

CELLMARKERCAN_ACINAR_CELLS	PNLIPRP1	CTRC	REG3A	SERPINA3	PRSS3	REG1B	CFB	GDF15	MUC1	C15ORF48	DUOXA2	AKR1C3	OLFM4	GSTA1	LGALS2	PDZK1IP1	RARRES2	CXCL17	UBD	GSTA2	ANPEP	LYZ	ANGPTL4	ALDOB	
CELLMARKERCUR_N_UMBRELLA_CELL																									
CELLMARKERCUR_N_UNRESTRICTED_SOMATIC_STEM…																									
CELLMARKERCUR_N_UNSWITCHED_PLASMABLAST																									
CELLMARKERCUR_N_URETERIC_BUD_CELL																									
CELLMARKERCUR_N_UROTHELIAL_CELL																									
CELLMARKERCUR_N_UROTHELIUM_CELL																									
CELLMARKERCUR_N_VASCULATURE_CELL																									
CELLMARKERCUR_N_VEIN_CELL																									
CELLMARKERCUR_N_VENOUS_CELL																									
CELLMARKERCUR_N_VENTRAL_LATERAL_GANGLIONI…																									
CELLMARKERCUR_N_VENTRAL_MEDIAL_GANGLIONIC…																									
CELLMARKERCUR_N_VENTRICULAR_CELL																									
CELLMARKERCUR_N_YOUNG_NEURON																									
CELLMARKERCUR_N_ZONA_FASCICULATA_CELL																									
CELLMARKERCUR_N_ZONA_GLOMERULOSA_CELL																									
CELLMARKERCUR_N_ZONA_RETICULARIS_CELL																									
CELLMARKERCUR_N_ZYGOTE																									
CELLMARKERCURNACTIVATEDEFFECTORMEMORYCD4T…																									
CELLMARKERCURNACTIVATEDTISSUERESIDENTMEMO…																									
CELLMARKERCURNACTIVATEDTISSUERESIDENTMEMO…																									
CELLMARKERCURNALPHACELLACELL																									
CELLMARKERCURNALVEOLARTYPEIIATIICELL																									
CELLMARKERCURNATYPICALBCELLABC																									
CELLMARKERCURNAXLCD11CPLASMACYTOIDDENDRIT…																									
CELLMARKERCURNAXLCD123PLASMACYTOIDDENDRIT…																									
CELLMARKERCURNBASALFOREBRAINCHOLINERGICNE…																									
CELLMARKERCURNBRUSHCELLTUFTCELL																									
CELLMARKERCURNCANCERASSOCIATEDFIBROBLASTC…																									
CELLMARKERCURNCAPSULARFIBROBLASTFB2																									
CELLMARKERCURNCCR7DENDRITICCELL																									
CELLMARKERCURNCD106MESENCHYMALSTEMCELL																									
CELLMARKERCURNCD14MACROPHAGE																									
CELLMARKERCURNCD16DENDRITICCELL																									
CELLMARKERCURNCD25LAG3TCELL																									
CELLMARKERCURNCD34GRANULOCYTICPROGENITORC…																									
CELLMARKERCURNCD34HEMATOPOIETICSTEMCELL																									
CELLMARKERCURNCD34PREBCELL																									
CELLMARKERCURNCD34PROGENITORCELL																									
CELLMARKERCURNCD34THYMOCYTE																									
CELLMARKERCURNCD40LGTHELPERCELL																									
CELLMARKERCURNCD4CD25REGULATORYTCELL																									
CELLMARKERCURNCD4CENTRALMEMORYLIKETTCMLIK…																									
CELLMARKERCURNCD4CYTOTOXICT1CELL																									
CELLMARKERCURNCD4CYTOTOXICT2CELL																									
CELLMARKERCURNCD4THELPERCELL																									
CELLMARKERCURNCHRONICALLYACTIVATEDCD4TCELL																									
CELLMARKERCURNCHRONICALLYACTIVATEDCD8TCELL																									
CELLMARKERCURNCLASSSWITCHEDMEMORYBIGGU03B…																									
CELLMARKERCURNCLEC10ADENDRITICCELL																									
CELLMARKERCURNCLEC9ADENDRITICCELL																									
CELLMARKERCURNCONVENTIONALDENDRITICCELL2B…																									
CELLMARKERCURNCONVENTIONALDENDRITICCELL2B…																									
CELLMARKERCURNCORTICALSOMATOSTATINSSTINTE…																									
CELLMARKERCURNCYTOTOXICCD4T2CELL																									
CELLMARKERCURNCYTOTOXICCD4TCELL																									
CELLMARKERCURND1MSNMEDIUMSPINYNEURON																									
CELLMARKERCURND2MSNMEDIUMSPINYNEURON																									
CELLMARKERCURNDISTALTUBULELOOPOFHENLECELL																									
CELLMARKERCURNEARLYCD34MEGAKARYOCYTEERYTH…																									
CELLMARKERCURNEARLYTCELLPROGENITORETP																									
CELLMARKERCURNEFFECTORMEMORYCD4TCELL	HOXA9	TRIM25	ZFP90	SLC2A13	MAML2	SPRED2																			
CELLMARKERCURNEFFECTORREGULATORYTETREGCELL																									
CELLMARKERCURNEFFECTORTTEFFCELL	IFNG	CD8A	TNFRSF9	EOMES	KLRG1	CCR7	FOXP3	CD82	CD14																
CELLMARKERCURNEXHAUSTEDCD4TCELL																									
CELLMARKERCURNEXHAUSTEDCD8TCELL	ISG15	CD82	IFIT3	IFITM3	BST2	GNLY	IFI44L	CCL3	MYO7A	WARS1	PHLDA1	TNFRSF9	CD27	LAYN	GZMK	NKG7	BTLA	HNF1A	EOMES	IRF4	HSPB1	GIMAP6	CXCR6	HSPH1	
CELLMARKERCURNEXHAUSTEDCD8TPROGENITORCELL																									
CELLMARKERCURNEXHAUSTEDTTEXCELL	TOX3	SIRT1	TOX	TOX4	KLRG1	VSIR	CERS6	CD274	CXCL13	LAYN	HMMR	PCLAF	TYMS	UBE2C	CD38	PI3	MKI67	TOP2A	CHI3L2	IGLC3	CD276	CD8A	PDCD1LG2	CD8B	KIR3DL1
CELLMARKERCURNEXTRATELENCEPHALICETCELL																									
CELLMARKERCURNFINALLYHIGHLYEFFECTORTEMRAM…																									
CELLMARKERCURNFOLLICULARREGULATORYTTFRCELL																									
CELLMARKERCURNFOXP3NATURALREGULATORYTTREG…																									
CELLMARKERCURNGAMMADELTAU03B3DTCELL	STMN2	FXYD2	NCR1	TRD	TRG	STYK1	CD160	FEZ1	MIR9-1HG	CD28	CD69														
CELLMARKERCURNGERMINALCENTREGCLIKECELL																									
CELLMARKERCURNGLOMERULARENDOTHELIALCELLECG																									
CELLMARKERCURNGRANULOCYTICMYELOIDDERIVEDS…																									
CELLMARKERCURNGRBREGULATORYBCELL																									
CELLMARKERCURNGZMKTCELL																									
CELLMARKERCURNHIGHLYDIFFERENTIATEDEFFECTO…																									
CELLMARKERCURNINDUCEDREGULATORYTTREGCELL																									
CELLMARKERCURNINFLAMMATORYCANCERASSOCIATE…																									
CELLMARKERCURNINNATELYMPHOIDCELLILCS																									
CELLMARKERCURNINTRATHYMICFIBROBLASTFB1																									
CELLMARKERCURNITOCELLHEPATICSTELLATECELL																									
CELLMARKERCURNLYMPHANGIOLEIOMYOMATOSISCEL…																									
CELLMARKERCURNMARGINALZONEMZLIKEBCELL																									
CELLMARKERCURNMEMORYSTEMCELLTSCM																									
CELLMARKERCURNMICROGLIADERIVEDTUMORASSOCI…																									
CELLMARKERCURNMKERYTHCELL																									
CELLMARKERCURNMYOFIBROBLASTICCANCERASSOCI…																									
CELLMARKERCURNNAIVEREGULATORYTTREGCELL																									
CELLMARKERCURNNATURALREGULATORYTTREGCELL																									
CELLMARKERCURNPERIPHERALBLOODMONONUCLEARC…																									
CELLMARKERCURNPITUITARYSTEMCELLPSC																									
CELLMARKERCURNPLASMACYTOIDDENDRITICCELLPDC	PTPRC	CD34	CD2AP	CLEC4A	TCL1A	TNFRSF…	BCL11A	VEGFB	PLAC8	CCDC50	CLIC3	C12orf75	LILRB4	SMPD3	SLC15A4	LAMP5	LRRC26	GAPT	NUCB2	PPP1R14B	CYB561A3	MAP1A	PTCRA	SCT	HIGD1A
CELLMARKERCURNPOLYMORPHONUCLEARMYELOIDDER…																									
CELLMARKERCURNPOLYMORPHONUCLEARNEUTROPHIL…																									
CELLMARKERCURNPOSTERIORPRESOMITICMESODERM…																									
CELLMARKERCURNPROLIFERATIVECD4TCELL																									
CELLMARKERCURNPROLIFERATIVECD8TCELL																									
CELLMARKERCURNREDBLOODCELLERYTHROCYTE	GYPC	HEMGN	SCN2A	ITGAV	ITGB3	MYC	KLF1	EPCAM																	
CELLMARKERCURNREGULATORYBBREGCELL																									
CELLMARKERCURNREGULATORYCD4TCELL																									
CELLMARKERCURNREGULATORYCD8TCELL																									
CELLMARKERCURNREGULATORYTTREGCELL	ICA1	IKZF2	S1PR4	IL10RA	CDC25B	CD3E	CD3D	CD27	BATF	BACH2	PRDM1	IL32	CD28	CD70	LTA	IL4I1	SOCS1	LGALS3	LGALS1	IL5RA	FCGR2B	NKG7	CD244	UTS2	LAG3

CELLMARKERCAN_ACINAR_CELLS	PNLIPRP1	CTRC	REG3A	SERPINA3	PRSS3	REG1B	CFB	GDF15	MUC1	C15ORF48	DUOXA2	AKR1C3	OLFM4	GSTA1	LGALS2	PDZK1IP1	RARRES2	CXCL17	UBD	GSTA2	ANPEP	LYZ	ANGPTL4	ALDOB	
CELLMARKERCURNREGULATORYTTREGCELL	TNFRSF4	TNFRSF9	CORO1B	ICA1	ARID5B	FANK1	RTKN2	GBP2	PDCD1	SIRPG	CD27	CD2	CD3E	CD3G	RNF19A	TNFRSF1B	SMAD3	LAP	ITGAE	SELL	STAT5A	IL1R2	EOS	ADA	DPP4
CELLMARKERCURNRENALVESICLECOMMASHAPEDBODY…																									
CELLMARKERCURNRESIDENTMEMORYCD8TCELL																									
CELLMARKERCURNREVERTANTMEMORYCD8TTEMRACELL																									
CELLMARKERCURNSCARASSOCIATEDMACROPHAGESAM																									
CELLMARKERCURNSENESCENTCD8TCELL																									
CELLMARKERCURNSTEMPROGENITORCELL																									
CELLMARKERCURNSWITCHEDMEMORYBCELLSWMB																									
CELLMARKERCURNT1TRANSITIONALBCELL																									
CELLMARKERCURNT2TRANSITIONALBCELL																									
CELLMARKERCURNTCELLNATURALKILLERCELL																									
CELLMARKERCURNTERMINALEFFECTORCD8TCELL																									
CELLMARKERCURNTERMINALLYDIFFERENTIATEDCD8…																									
CELLMARKERCURNTFOLLICULARHELPERTFHCELL	ZNF764	THADA	SH3TC1	PTPN13	TOX	POMT1	ELAPOR1	SIRPG	MRTFB	STK39	CHGB	LDLRAD4	H4C12	PASK	CHI3L2	B3GAT1	TSHR	PVALB	MAGEH1	SMAD1	CDK5R1	HEY1	ST8SIA1	MYO6	KCNK5
CELLMARKERCURNTHELPER1TH1CELL																									
CELLMARKERCURNTHELPER1TH1CELL	CD38	CD70	APOD	CSF2	DUSP5	APBB2	SYNGR3	BTG3	DOK5	LRRN3	DGKI	ATP9A	ZBTB32	IL22	CMAHP	EGFL6									
CELLMARKERCURNTHELPER2TH2CELL	NCAPG	ABO	LMAN1	RELN	COL5A1	MAF	GZMA	LIMA1	NEIL3	CXCR6	SLC39A14	WDHD1	ADCY1	BIRC5	PMCH	PHEX	CDC7	EVI5	ANK1	SNRPD1	AHI1	IL26	PTGIS	HELLS	DHFR
CELLMARKERCURNTHELPER2TH2CELL																									
CELLMARKERCURNTHELPER9TH9CELL																									
CELLMARKERCURNTHELPERTHCELL	KLRB1	KLF2	PLAC8	TOX2	CD200	CXCL13	MME	CXCL9	CXCL10	IFNG	CXCL11	IL12B	ITGAX	CCR4	SLC25A12	DDX50	FAM111A	CD28	UBE2L3	LRBA	PPP2R5C	FRYL	RPA1	NUP107	BORA
CELLMARKERCURNTHYMICEPITHELIALCELLTEC																									
CELLMARKERCURNTISSUERESIDENTMEMORYCD4TCELL																									
CELLMARKERCURNTISSUERESIDENTMEMORYCD8TCELL																									
CELLMARKERCURNTISSUERESIDENTMEMORYTTRMCELL	GZMB	CCL5	CCL4	CCR7	ITGB7	ITGAL	ITGB2	ITGB1																	
CELLMARKERCURNTPERIPHERALHELPERTPHCELL																									
CELLMARKERCURNTRANSIENTAMPLIFYINGTACELL																									
CELLMARKERCURNTRANSITIONALEXHAUSTEDCD8TCE…																									
CELLMARKERCURNTUMORINFILTRATINGLYMPHOCYTE…																									
CELLMARKERCURNUNSWITCHEDMEMORYBCELLUNSWMB																									
CELLMARKERCURNVASARECTAENDOTHELIALCELLECVR																									
CELLMARKERCURNVONECONOMONEURONVEN																									
CELLMARKERCURNYOLKSACDERIVEDMYELOIDBIASED…																									
CELLMARKEREXP_ACINAR_CELLS	PNLIPRP1	CTRC	REG3A	SERPINA3	PRSS3	REG1B	CFB	GDF15	MUC1	C15ORF48	DUOXA2	AKR1C3	OLFM4	GSTA1	LGALS2	PDZK1IP1	RARRES2	CXCL17	UBD	GSTA2	ANPEP	LYZ	ANGPTL4	ALDOB	
CELLMARKEREXP_ADRENERGIC_NEURONS																									
CELLMARKEREXP_AIRWAY_EPITHELIAL_CELLS																									
CELLMARKEREXP_AIRWAY_SMOOTH_MUSCLE_CELLS																									
CELLMARKEREXP_ALPHA_CELLS	SH3GL2	SCGB2A1	FAP	DPP4	GPR119	MAFB	PAX6	NEUROD1	LOXL4	PLCE1	GC	KLHL41	FEV	PTGER3	RFX6	SMARCA1	PGR	LDB2	IRX1	UCP2	FXYD5	RGS4	GLS		
CELLMARKEREXP_ANTERIOR_PITUITARY_GLAND_CE…	FGF10	FSHB	PCSK1	GNRHR	PITX1	GH1	TSHB	PAX7	LHB																
CELLMARKEREXP_ASTROCYTES	SLC16A2	SYNE1	DIO2	SLC7A10	ENTPD2	HSPB6	PLXNB1	LCAT	PLA2G7	CMTM5	S1PR1	SLC39A12	LUZP2	GSTA4	SLC25A28	SLC25A46	WNT3	FZD2	TEAD1	RLBP1	ALDOC	GJA1	APOE	LCN2	SOX9
CELLMARKEREXP_B_CELLS	HLA-DQA1	FLI1	CD14	SEMA6D	LAIR1	IFIT3	IGLL1	DNTT	MME	SPN	CD19	CD24	CD27	B3GAT1	CD72	MUM1	PAX5	JCHAIN	MZB1	LY6D	FCMR	BANK1	EDEM1	VPREB3	POU2AF1
CELLMARKEREXP_B_CELLS_MEMORY	CD79B	CLCA3P	CR2	CXCR5	DENND5B	FCGR2B	FCRL2	FRK	GNG7	GPR18	GUSBP11	HHEX	HLA-DOB	IFNA10	IGHD	IGHM	IGKC	IGLL3P	IL7	IRF8	LTB	LY86	MBL2	MS4A1	NMBR
CELLMARKEREXP_B_CELLS_NAIVE	FCGR2B	FCRL2	FRK	GPR18	GUSBP11	HHEX	HLA-DOB	IGHD	IGHM	IGKC	IGLL3P	IL4R	IRF8	LTB	LY86	MEP1A	MICAL3	MS4A1	NMBR	P2RX5	P2RY14	PNOC	PSG2	PTPRCAP	RALGPS2
CELLMARKEREXP_BASAL_CELLS	ALKBH7	DNPH1	KRT23	SLC16A3	GYPC	DUSP23	RARRES1	ITGA2	SLC25A37	GAPDHP1	ITGB6	ITGB4	SPHK1	ACTG2	IRF6	RAB13	LAMB3	ACADVL	MMP7	HEBP2	FBXO32	MT1E	MT2P1	SERINC2	NNMT
CELLMARKEREXP_BASOPHILS	DNASE2B	FADS2	GPC4	GRM6	ACSS2	ADORA2B	AQP9	BACE1	BMP4	CABLES1	CCL4	CEBPA	E2F8	FADS1	FAR2	FBXO9	GABBR1	GPR183	HGF	HIST1H1D	HIST1H2AC	IFITM1	INF2	JAZF1	L1CAM
CELLMARKEREXP_BERGMANN_GLIA	ZEB2	UFL1	NRXN3	ATP7A	BICD2	GJA1	METRN	P2RX7	EGFR	GRIA1	P2RY1	GRIN2B	GRIN3A	46269	DAO	ITGB1	KIF1BP								
CELLMARKEREXP_BETA_CELLS	GJD2	SCGN	IGF2	SYT13	FFAR2	NPTX2	PFKFB2	EDARADD	HOPX	SH3GL2	ADCYAP1	SCGB2A1	CASR	MAFB	PAX6	NEUROD1	ISL1	TGFBR3	SMAD9	SIX3	SIX2	BMP5	PIR	STXBP5	DLK1
CELLMARKEREXP_CAJAL_RETZIUS_CELLS	NSMCE3	DACH1	FAM69B	HOMER2	MAP9	DNM3	LZTFL1	FSD1	TCEAL1	RAB11FI…	BARHL2	TRIM44	SLC17A6	B3GALT1	SLITRK5	ERC2	NBEA	SLC25A36	ZDBF2	MAPK8	GDF5	ACHE	CCM2	WRAP73	NRG1
CELLMARKEREXP_CHOLANGIOCYTES	SOX9	EPCAM	CLDN4	MMP7	TFF2	SCGB3A1	FXYD2	DEFB1	CD24	LCN2	CXCL1	CXCL6	LGALS2	TACSTD2	ELF3	SPP1	MUC5B	LGALS4							
CELLMARKEREXP_CHOLINERGIC_NEURONS																									
CELLMARKEREXP_CHONDROCYTES	IGF2	MIA	SDC3	EMP1	COL6A1	CYTL1	LCN2	LUM	GADD45B	SCRG1	HAPLN1	COL11A1	S100A9	CHAD	COL9A3	DLK1	S100A8	CHODL	ATF4	SHOX	MGP	TCF4	VDR	ATRX	NOG
CELLMARKEREXP_DELTA_CELLS	PRG4	GHSR	FRZB	PCSK1	GABRG2	POU3F1	BHLHE41	EHF	LCORL	ETV1															
CELLMARKEREXP_DISTAL_TUBULE_CELLS	HOXD8	KL	EGF	TMEM213	TMEM52B	DEFB1	FXYD2	USMG5	TRHDE	KCNJ16	RHCG	SOSTDC1	SCNN1A	VDR	WNK1	SLC8A1	AQP2	AVPR2							
CELLMARKEREXP_DUCTAL_CELLS	PERP	PDLIM3	WFDC2	SLC3A1	AQP1	ALDH1A3	VTCN1	KRT19	TFF1	TFF2	KRT7	CLDN4	LAMB3	TACSTD2	CCL2	DCDC2	CXCL2	CTSH	S100A10						
CELLMARKEREXP_EMBRYONIC_STEM_CELLS	TDGF1	HOXB5	SUMO2	BNIP3	GJC1	TRIM6	PUM2	GJB4	SOX15	UTF1	GJB1	LMNA	FBXO15	PML	THY1	KIT	EPCAM	CD9	ITGB1	CD59	PROM1	PECAM1	ITGA6	FZD1	KITLG
CELLMARKEREXP_ENTERIC_NEURONS	ATF5	DMKN	CACNA1B	PIK3CA	ZDHHC2	MYL1	EML5	MFSD6	SCN7A	ARHGEF28	HAND2	VSTM2L	FAM19A5	EFNA5	HOXB4	TLX2	GOLGA7B	SLC18A3	TOX	CELF6	GPR22	PLCE1	CYB561	GFRA2	FLOT2
CELLMARKEREXP_ENTEROENDOCRINE_CELLS	NTS	SST	MLN	GPR119	GCG	CHGB	CPE	GAL	NUCB2	IAPP	GHRL	CHGA	TRPA1	ENPP2	AFP	VEGFA	FFAR4	MC4R	INSL5	SSTR2	ALCAM	TAS1R2	TAS1R3	GNAT3	
CELLMARKEREXP_EPENDYMAL_CELLS	MEIG1	HSPA2	CFAP65	LRRC23	TEKT4	CALML4	PIFO	CRYGN	HDC	PCP4L1	DYNLRB2	TMEM107	RIIAD1	TMEM212	RSPH1	CCDC153	S100B	AK7	RARRES2	PVALB	ANGPTL2	CFAP54	STOML3	PLTP	SSPO
CELLMARKEREXP_EPSILON_CELLS	HRH2	CALCR	SLC6A16	PCSK6	ADAMTS6	COL22A1	FAM124A	COL12A1	CD109	THSD4	ARX	NKX2-2	ACSL1	FRZB	VTN	APOH	SPINK1	VSTM2L	SPTSSB	S100A6	KRT8				
CELLMARKEREXP_ERYTHROID_LIKE_AND_ERYTHROI…	DCAF12	HBD	HEMGN	HMBS	NCOA4	RBM38	UCP2	UBB	TMCC2	SLC4A1	SLC25A39	SLC25A37	FOSB	HTATSF1	EPB41	RSAD2	COX6B2	APOA1	ASNS	ALAD	S100A9	AHSG	S100A8	ERMAP	KCNN4
CELLMARKEREXP_GAMMA_DELTA_T_CELLS	MKI67	UBE2C	TUBA1B	ASPM	CENPA	TOP2A	PCNA	AURKB	BIRC5	NUSAP1	TROAP	TUBB	H2AFX	CENPF	CCNB1	H2AFZ	TRGV2	TRGV10	TRGV11	TRGV1	TRGV3	TRGV4	TRGV5	TRGV8	TRGV9
CELLMARKEREXP_GOBLET_CELLS	GALNT12	ATOH1	REP15	PDIA5	PLA2G10	KLK1	SPDEF	LRRC26	BACE2	MANF	SPINK4														
CELLMARKEREXP_HEMANGIOBLASTS																									
CELLMARKEREXP_HEMATOPOIETIC_STEM_CELLS	HHEX	MEIS1	LYL1	ELF1	HLF	RGS18	MYCT1	ADGRG1	DNMT3B	MYCN	ARID3A	ITGA2B	GATA3	ROBO4	PITX2	TXNIP	RUNX1	PTPRS	PTPRC	CD44	GFI1	CD48	HMGB3	TLR2	APC
CELLMARKEREXP_HEPATIC_STELLATE_CELLS	SYP	VCL	TIMP1	COL1A1	TAGLN	COL1A2	COL3A1	SPARC	RBP1	DCN	MYL9	TPM2	MEG3	BGN	IGFBP7	IGFBP3	CYR61	OLFML3	IGFBP6	CCL2	COLEC11	HGF			
CELLMARKEREXP_HIS_BUNDLE_CELLS																									
CELLMARKEREXP_IMMATURE_NEURONS	NES	RBFOX3	CREB1	NEUROD1	ASCL1	NTRK1	SYN1	NF1	BCL2	SLC6A5	FOXD2	ICAM5	SSTR2	NEUROG2											
CELLMARKEREXP_JUXTAGLOMERULAR_CELLS																									
CELLMARKEREXP_KERATINOCYTES	IL13RA1	SGPP1	PKP3	PKP1	ANXA8	TRIM29	DSC3	FST	AQP3	KRT15	S100A14	DMKN	ENTPD2	FGF5	NGFR	TFAP2A	LGALS7	CXCL1	CCL2	SFN	KRT10	SBSN	KRT84	KRT36	
CELLMARKEREXP_KIDNEY_PROGENITOR_CELLS																									
CELLMARKEREXP_KUPFFER_CELLS	MNDA	SLC15A3	DNASE1…	MARCO	HFE	CD38	CD163	CCR5	CLEC1B	CLEC4G	GPIHBP1	FOLR2	PLTP	FTL	IRF7	SPIC	CSF1R	C1QC	C1QA	C1QB	CLEC4E				
CELLMARKEREXP_LEYDIG_CELLS	RLN3	TAC1	STAR	THBS2	PTGDS	LCN2	HSD17B3	ASS1	PRLR	CYP26B1	IGF2	IGFBP5	CFD	DLK1	IGFBP3	INHBA	VIT	ALDH1A3	NOS1	EDNRA	SMOC2	ADAM21	ESRRG	GPRC6A	GJA1
CELLMARKEREXP_LOOP_OF_HENLE_CELLS	NKAIN4	ADAMTS5	CXCL1	GEM	FGA	PLA2G7	THBS1	ENC1	SPSB1	ARID5A	CD40	SLC14A2	NRGN	RNF182	PCSK9	DRAM1	ARC	NEK6	CDKN1C	CALCRL	SMARCD3	KCNT1	PTGS2	IRX2	PPP1R1B
CELLMARKEREXP_LUMINAL_EPITHELIAL_CELLS	SERPINA1	PIP	ATP7B	HIF1A	FGFR4	DDR1	CEBPD	PGR	KRT8	UXT	PTH1R	CD9	AR	AQP3	ATP2C2	WNT5A	SLC12A2	ESR1	AQP5	RUNX1	CDH1	MUC1	ANPEP	KLK3	
CELLMARKEREXP_MAMMARY_EPITHELIAL_CELLS	CSN3	CSN1S1	RARRES1	KRT17	NNMT	ALDH1A3	SGMS2	PIEZO1	ANO1	WNT5B	NEDD9	FOXP4	LSR	LIF	KRT19	KRT7	STAT3	KRT8	KRT18						
CELLMARKEREXP_MEGAKARYOCYTES	FLI1	PROX1	SYP	TSPAN9	RGS18	TREML1	GATA2	VWF	ALOX12	SELP	NFATC1	CIB1	PLK3	MAFG	IL2RA	PECAM1	PPARG	IL6	IL21R	LRP1	ACHE	VEGFA	TGFB1	GP9	
CELLMARKEREXP_MELANOCYTES	MEN1	POMC	TFAP2A	RAB38	TP63	CRABP1	BMI1	PTN	PRKN	KLC2	IL24	TPH2	PPARA	PPARD	PPARG	PRKCA									
CELLMARKEREXP_MESANGIAL_CELLS	MIB1	NOSTRIN	ADAMTS…	EMILIN1	ITGA8	DYRK1A	ARID5B	CCR7	FN1	PAWR	PTN	SERPINE2	TBX18	TEK	THY1	MYL9	TPM2	CALD1	HOPX	NR4A2	ACTN1	AKAP12	ROCK1	SH3BGRL	RASD1
CELLMARKEREXP_MESOTHELIAL_CELLS	FZD1	RASL11A	CDON	BCL3	ACKR3	NPR1	PCSK6	DPP4	RND1	GBP2	MEDAG	AR	LGALS7	ANO1	GSTA3	OSR1	TWSG1	ADGRD1	CRLS1	FLNC	PTGS2	C2	FMO2	FMOD	FGF1
CELLMARKEREXP_MICROFOLD_CELLS	ONECUT2	FOSL2	IRF6	IRF2	NFIB	FOXP4																			
CELLMARKEREXP_MICROGLIA	TMEM173	TBXAS1	P2RY6	P2RY13	CEBPA	CD180	MYO1F	OLFML3	S100A8	LTC4S	TGFBR1	S100A9	LPCAT2	ITGB5	LAIR1	SPHK1	COLEC12	ALDH1A2	AEBP1	PTGDS	CCR5	SLCO2B1	PTGS1	APBB1IP	CYFIP1
CELLMARKEREXP_MONOCYTES	ITGAM	ACE	FUT4	ICAM1	SELL	CD163	FCGR1A	FCGR2B	ACP5	MRC1	TNF	PLAU	GBP1	OAS1	IRF7	PLSCR1	MX1	IL1RN	HLA-DRA	IFIT1	IDO1	IFIT3	TNFSF10	CXCL10	S100A9
CELLMARKEREXP_MULLER_CELLS	COL6A1	COL11A1	GLUL	CLU	S100A16	SOX2	APOE	DBI	GNAI2	GPR37	KCNJ2	KCNJ10	SPC25	ITM2B	ATG4B	CA2	ZBED4	PRDX6	SLC1A3	HES1	KIT	CRB1	ANXA4	CD44	GSTM5
CELLMARKEREXP_MYOBLASTS	TNFRSF1…	ARL4D	CCL2	MYD88	BRAF	EGFR	ZBTB16	SIX1	PDZRN3																
CELLMARKEREXP_MYOCYTES	MYH1	MYH4	MYH7	MYH8	NBR1	NEB	NID1	NID2	TMOD4	TPM2	TTN	MYF5	MYF6	MYOD1	MYOG	SIX1	SIX4	PAX3	PAX7	ADAM12	ARHGAP26	CAV3	CDH15	CMKLR1	CXCR4
CELLMARKEREXP_MYOFIBROBLASTS																									
CELLMARKEREXP_NEUROBLASTS	EFNA2	DCX	CCKAR	CCKBR	GRM5	EZH2	DLX2	MARK2	LRP8	VLDLR	DAB1	NCAN	EPHA4	TACC3	EGFR	TUBB3	SIRT1	NCAM1	NEUROD1	SCGN	KLK3	NES	WT1	ZBED4	PROS1
CELLMARKEREXP_NEUTROPHILS	DEFA3	LTF	LYZ	ELANE	S100A8	IL1B	CXCL2	HP	CCL3	HDC	MMP8	LRG1	OSM	MMP9	PILRA	CLEC4D	CLEC4E	ASPRV1	CCRL2	CCR1	NCF1	TREM1	SORL1	ARG2	BST1
CELLMARKEREXP_NK_CELLS	HMBOX1	AHR	PRF1	CCL4	SEMA6D	FHL2	CD2	CD7	CD3G	CD33	DPP4	LAT	PCDH15	CCL5	GZMA	GZMB	CMA1	DUSP2	TXK	DOK2	CST7	HSD11B1	KLRC2	SAMD3	TBX21
CELLMARKEREXP_NORADRENERGIC_NEURONS																									
CELLMARKEREXP_OLIGODENDROCYTES	FASN	OMG	ZDHHC9	PTGDS	ADAMTS4	MYRF	SEMA4D	TMEM125	KLHL2	DPY19L1	TSPAN15	HAPLN2	NKX6-2	GJB1	OPALIN	VLDLR	MCAM	TMEM163	ERMN	FA2H	CLDN11	ANLN	ELOVL7	PLLP	DDC
CELLMARKEREXP_OSTEOBLASTS	CCL11	SPP1	FAS	TNFRSF10A	WNT5A	CX3CL1	F2R	PTH1R	ITGA2	ITGA5	NOD1	NOD2	THY1	ESR1	CYP27B1	P2RX4	P2RX5	INSR	EFNB2	EPHB4	VCAM1	FGFR1	FGFR2	BGLAP	TLR5
CELLMARKEREXP_OSTEOCLAST_PRECURSOR_CELLS																									
CELLMARKEREXP_OSTEOCLASTS	SLC39A14	AQP9	ONECUT1	ITGB3	ITGB5	IRAK3	ITGA2	TAS1R3	SERPINF1	ITGAV	ITGB1	P2RY6	CCL3	EPHB1	CA2	HIF1A	CX3CR1	ESR1	EFNB2	VDR	CASR	CD68	GRM8	SMAD4	SPP1
CELLMARKEREXP_OSTEOCYTES																									
CELLMARKEREXP_PANETH_CELLS	NOD2	LGR4	RIPK2	TLR5	WNT3	KCNN4	RAP1A	ATG16L1	WNT9B	DLL4	IL4R	TLR9	CTNNB1	DLL1	TM4SF20	DEFA3	MPTX1	KLF15							
CELLMARKEREXP_PARATHYROID_CHIEF_CELLS																									
CELLMARKEREXP_PERICYTES	HSD11B1	MCAM	MXRA8	PDE5A	NR1H3	SERPIN…	EMID1	ECM1	COLEC11	RARRES2	REM1	ASPN	CYGB	FABP4	VTN	STEAP4	NDUFA4…	SLC38A11	ATP13A5	AOC3	ANGPT2	INPP4B	GPIHBP1	VIM	PTH1R
CELLMARKEREXP_PHOTORECEPTOR_CELLS	GLS	STX3	GRM6	STRIP2	CHGA	RNPC3	VSTM2B	CACNA2…	VSX2	TRNP1	NEUROD4	GNB3	OTX2	CACNA2…	LIN7A	CCDC136	NDNF	GNG13	PROX1	GPR179	CABP5	KCNMA1	LRTM1	PRKCA	QPCT
CELLMARKEREXP_PLASMA_CELLS	FCGR2B	CCR10	ABCB9	AMPD1	ANGPT4	ATXN8OS	C11ORF80	CD79A	DENND5B	EAF2	FCRL2	GNG7	GPR25	GUSBP11	HIST1H2AE	HIST1H2BG	HLA-DOB	IGHD	IGHE	IGHM	IGKC	IGLL3P	KCNA3	KCNG2	LIME1
CELLMARKEREXP_PLASMACYTOID_DENDRITIC_CELLS	CXCR4	PACSIN1	TLR7	TLR9	IRF7	IRF8	IGLL1	CUX2	MCTP2	KLK1	BLNK	GRM8	PRKCA	CDH1	IL17RB	CARD6	CLEC4G	S100A8	JCHAIN	SERPINF1	ITM2C	TCF4	BCL11A	MZB1	DERL3
CELLMARKEREXP_PODOCYTES	DPP4	ANGPTL2	ITGB5	RHPN1	BCAM	MAGI2	COL4A3	EFNB1	NPR1	PLOD2	SRGAP1	LAMB2	NPR3	ITGA3	PLCE1	PROS1	MERTK	MYO1D	TCF21	TNS2	ADM	OPTN	PARD3B	SEMA3E	MYO1E
CELLMARKEREXP_PRINCIPAL_CELLS	PHACTR1	CELSR2	FZD7	SLC2A9	RNF186	TRPV5	STC1	PELI2	KLK1	ACER2	AQP4	FZD1	GATA3	LGALS3	PTGES	TSPAN1	NR3C2	HES1							
CELLMARKEREXP_PROXIMAL_TUBULE_CELLS	NOX4	SLC17A1	SLC13A3	CYP2E1	SLC22A8	FOLR1	SLC7A13	AK4	SLC5A8	SLC22A1	GHR	UPB1	SLC17A3	GCDH	GSTZ1	ASL	FAH	TMEM106A	GSTA2	CRYL1	SLC5A12	AOAH	SLC13A1	ABCC2	PKHD1
CELLMARKEREXP_PULMONARY_ALVEOLAR_TYPE_I_C…	PXDC1	AQP3	CRLF1	EGFL6	FSTL3	MEX3B	RADIL	SMARCA1	P2RX7	PDPN															

Table S5: Cell state marker gene list

CELLMARKERCAN_ACINAR_CELLS	PNLIPRP1	CTRC	REG3A	SERPINA3	PRSS3	REG1B	CFB	GDF15	MUC1	C15ORF48	DUOXA2	AKR1C3	OLFM4	GSTA1	LGALS2	PDZK1IP1	RARRES2	CXCL17	UBD	GSTA2	ANPEP	LYZ	ANGPTL4	ALDOB	
CELLMARKEREXP_PULMONARY_ALVEOLAR_TYPE_II_…	PGC	CXCR2	CXCL2	GRK2	RUNX3	CTNND1	PPP1R14C	DDX3Y	PIGR	LRG1	NRN1	PPBP	IL1B	INMT	CRLF1										
CELLMARKEREXP_PURKINJE_FIBER_CELLS																									
CELLMARKEREXP_PURKINJE_NEURONS	SLC22A15	CACNG2	SYT2	CPLX1	PKP4	KCNMA1	ADGRB1	HCN1	SLC24A2	GABRA1	CLSTN3	SLC24A3	DLG4	CNPY1	SLC12A5	SLC32A1	GRIA3	CAMK2A	PENK	HIVEP2	NRIP3	TSHZ1	PCBP3	GPSM1	PPM1E
CELLMARKEREXP_PYRAMIDAL_CELLS	RTN4	PDE2A	KCNB1	KCNB2	KCNQ2	KCNQ3	SATB2	OXTR	CCK	KCND2	KCND3														
CELLMARKEREXP_RETICULOCYTES																									
CELLMARKEREXP_RETINAL_GANGLION_CELLS	DCTN1	GGT7	BTBD10	EFNA3	SLC29A4	STOML1	FBXO44	NELL1	RTN2	BBC3	EVC2	SYT16	CPNE5	TTLL1	GPHN	SIX6	ABLIM3	POU6F2	GRIP1	TOX2	NARF	BARHL2	DTX1	MAB21L2	KCTD8
CELLMARKEREXP_SALIVARY_MUCOUS_CELLS																									
CELLMARKEREXP_SATELLITE_CELLS	HMOX1	TNFRSF…	RUNX1	BARX2	TRIM32	VAMP2	PEG3	MSTN	SCRIB	NFATC2	IRF2	BMI1	SIX1	HOXC10	NOTCH3	HEYL	GAL	NCAM1	CAV1						
CELLMARKEREXP_SCHWANN_CELLS	RXRG	SOX10	CMTM5	BCHE	EGFL8	GFRA3	SEMA3B	MATN2	SCN7A	CD82	TAGLN	NOV	ALDOC	APOD	CRYAB	PLP1	PMP22	CADM4	KCNA1	CNP	GPR37L1	NRN1	STMN1		
CELLMARKEREXP_SEROTONERGIC_NEURONS																									
CELLMARKEREXP_SERTOLI_CELLS	MRO	HSD17B3	AMHR2	SEMA5B	SCX	HSD17B1	STIM1	ANKRD7	PHF7	CXCL12	STAR	WFDC2	PRND	HSPBAP1	NXF3	GBA2	PADI2	ABCG8	DIAPH2	CTSA	PFKFB3	MSI1	NOTCH2	GATA6	CLDN11
CELLMARKEREXP_SMOOTH_MUSCLE_CELLS	ACTG2	MUSTN1	SOD3	HSPB6	RRAD	LMOD1	ITGA1	SPEG	EHD2	RBPMS2	MAP3K7CL	SLC38A11	GJA4	KAT2B	LGR6	MSRB3	AKT2	RASL12	ZBTB44	WWP2	SSPN	OTUD1	FAS	VASN	PDGFD
CELLMARKEREXP_SPERMATOZOA																									
CELLMARKEREXP_STROMAL_CELLS	FAP	LAMC2	SNED1	TNC	LUM	COL15A1	GDF10	COL4A1	BMP4	WNT2	BMP5														
CELLMARKEREXP_T_CELLS	CD7	MAFF	TGIF1	RORA	TNFAIP3	CREM	PXDC1	NABP1	FAM110A	EEF1B2P3	TRAC	CD69	PFN1P1	IL32	CXCR4	46266	BCL2	CYTL1	CD2	CTSW	PTPN22	TXK	GDPD3	TRAF1	IL2RA
CELLMARKEREXP_T_CELLS_NAIVE																									
CELLMARKEREXP_T_CYTOTOXIC_CELLS																									
CELLMARKEREXP_T_HELPER_CELLS	CCR7	ICOS	PTGDR2	HAVCR1	IL1R2	IRF4	STAT6	IL17A	IL17F	IL21	IL22	RORA	RORC	CD38	KLRB1	AHR	CCR10	CD3D	CD4	CCR4	CCR6	IL9	CD2	CD28	CD5
CELLMARKEREXP_T_MEMORY_CELLS	CD28	BIN2	RAPGEF6	ARHGEF1	S100A9	ARHGAP…	CD3E	SKAP1	GIMAP4	CD2	PTPRC	LAT	TCF7	LEF1	46266	ARHGAP45	SATB1	PTPRCAP	S100A8	LTB	LCK	TRAC	CD3G	CD3D	TRBC2
CELLMARKEREXP_TRICHOCYTES																									
CELLMARKEREXP_TRIGEMINAL_NEURONS	ATL1	HSPB8	FKBP1B	TLN2	SYNM	GDAP1	DGKZ	PRUNE2	SLC17A6	FGF13	DGKH	CPNE3	CCDC92	SNCG	NICN1	NEFM	RGS7BP	ADD2	PIEZO2	VAMP1	SCN10A				
CELLMARKEREXP_TROPHOBLAST_CELLS	SERPINE1	PHLDA2	TFEB	PERP	PARP1	ERVFRD…	HLA-G																		
CELLMARKEREXP_TROPHOBLAST_PROGENITOR_CELLS																									
CELLMARKEREXP_TUFT_CELLS	RAC2	PTGS1	IRF7	FFAR3	ALOX5	TSLP	IL13RA1	IL17RB	PTPRC																
CELLMARKEREXP_VASCULAR_SMOOTH_MUSCLE_CELLS																									
CELLMARKEREXPENDOTHELIALCELLSAORTA	SYNPO	ATP2A3	GIMAP4	CMKLR1	ITGA9	SMAD6	EFNB2	LOX	FZD4	NTN1	NPR1	ARL4D	HEY2	HYAL2	FKBP7	NXN	PDIA5	APH1B	SOX18	WBP1L	LAMB2	CHST12	P3H3	KLK10	MALL
CELLMARKEREXPNEURALSTEMPRECURSORCELLS	IFI44	MGAT5	PBK	DLX2	CBX3	RND3	DLX5	SP9	POU3F4	BARHL2	LHX9	SHOX2	DBX1	DLL3	PITX2	OTX1	ARX	NXPH1	TOX3	ASCL2	HES5	HOPX	NEUROD1	PRSS56	SOX9
CELLMARKERMSIGDB_AIZARANI_LIVER_C12_NK_NK…	RUNX3	KLRF1	ALOX5AP	LCP1	IKZF3	EOMES	IFITM1	CD3E	IL2RG	CD247	BTN3A1	KLRB1	GZMA	PYHIN1	TXK	RAC2	SYTL3	GZMK	PTPRC	BTG2	TXNIP	CD69	DNAJA4	TRGC1	WIPF1
CELLMARKERMSIGDB_AIZARANI_LIVER_C22_RESID…	AMPD1	PTK2B	CD27	TSC22D3	HLA-DOB	SPCS2	BTG2	PLPP5	CRELD2																
CELLMARKERMSIGDB_AIZARANI_LIVER_C28_NK_NK…	SLA	STK17B	CYTIP	SLC38A1	ARHGDIB	PRDM1	PTPRC	FAM177…	RBPJ	SAMSN1	ETS1	PDE7A	PTGER4	RNF125	RUNX3	NFKB1	LCP1	PARP8	PPP1CB	SYTL3	ITK	KIT	WIPF1	KLRB1	SH2D2A
CELLMARKERMSIGDB_AIZARANI_LIVER_C29_MVECS…	EDN1	THBD	SRPX	PTPRB	IL6	MYADM	FBLN2	HEG1	IL33	PECAM1	DLC1	FKBP1A	TSC22D1	TGM2	POSTN	PLPP3	YBX3	TIMP1	CRIM1	TIMP3	PDLIM1	TGFBR3	CDKN1A	AQP1	LDB2
CELLMARKERMSIGDB_AIZARANI_LIVER_C30_HEPAT…	GADD45G	RN7SL5…	MAT1A	HAL	ADGRA3	F12	DST	H6PD	TENM1	ERRFI1	ZMAT3	C2orf68	SLC23A2	AFM	ACSL1	ITPR2	PROC	ERBB3	ZC3H13	CFH	CYP2C8	AR	LONP2	FAM20A	FTCD
CELLMARKERMSIGDB_AIZARANI_LIVER_C8_RESIDE…	MYDGF	LY9	H1-10	ERLEC1	SPCS2	ISG20	NUCB2	SPCS3	SDC1	TMEM258	PTK2B														
CELLMARKERMSIGDB_AIZARANI_LIVER_C9_LSECS_1	PDE2A	TIMP3	COL4A2	IL1RL1	PTPRB	LIFR	CCL15-C…	SHANK3	PPFIBP1	PXDN	UGCG	SPARC	STC1	COL4A1	CRHBP	MRO	SASH1	BTNL9	MCAM	ADGRF5	MRC1	NOTCH4	RELN	CLEC1B	MARCKS…
CELLMARKERMSIGDB_BUSSLINGER_DUODENAL_BCHE…	NOTCH2N…	PLAC8	GMNN	LGALS3	LPCAT4	FAM13A	S100A6	TMEM219	SRI	FKBP1A	ID1	GSTP1	AKR1C3	RBM47	TSPAN8	CDH17	TMSB10	CST3	LGALS4						
CELLMARKERMSIGDB_BUSSLINGER_DUODENAL_DIFF…	IDH2	HMGB1	HSP90A…	MT1F	TOP2A	RAN	TXN	OLFM4	CPS1	YBX1	RPS24	MLEC	AQP1	RPS6	EEF1B2	DUT	RPL4	RPL7A	RPS23	GSTP1	RPS3	RPS5	GAPDH	RPS3A	SERBP1
CELLMARKERMSIGDB_BUSSLINGER_DUODENAL_EC_C…	SCGN	CADPS	ADH4	CES1	MS4A8	SPINK1	ID1																		
CELLMARKERMSIGDB_BUSSLINGER_DUODENAL_IMMU…	CD69	RUNX3	IL7R	EVL	LCK	HLA-B	NKG7	IKZF3	IL2RB	HLA-E	IFITM2	FYB1	CELF2	ETS1	STK17B	CKLF	CD247	CD48	S100A4	HCST	FNBP1	ACTB	CYFIP2	PPP1R18	ACAP1
CELLMARKERMSIGDB_BUSSLINGER_DUODENAL_STEM…	PPP1R1B	HSPD1	ARID5B	RPS24	RPS23	RPL9	RPL13A	NRARP	ZFAS1	ZNF503	APP	HES1	GDF15	MYB	RPS3A	RPL10A	EGR1	IRF2BP2	EIF3E	RPL14	RPS27A	EEF1B2	PTMA	RNF43	BTG2
CELLMARKERMSIGDB_BUSSLINGER_DUODENAL_TUFT…	PBXIP1	PSTPIP2	KRT18	MYO1B	POU2F3	SKAP2	TMEM74B	KRT20	RASSF6	SPIB	ANKRD30…	SERPINA1	PIK3CG	FGFR4	TAMALIN	MARCKSL1	BCAM	CCSER1	TMEM17…	KCTD12	KLK13	IFI6	ZDHHC8BP	FURIN	CORO2B
CELLMARKERMSIGDB_BUSSLINGER_ESOPHAGEAL_EA…	TMPO	CENPF	CSRP2	CALM2	SMC4	RAD21	HNRNPA1	PRKDC	HNRNPR	HNRNPF	DSC3	HSPD1	VSNL1	FBL	HNRNPA2B1	TRA2B	HNRNPC	NFIC	YBX1	RAN	SET	HNRNPAB	DDX21	CCT5	NCL
CELLMARKERMSIGDB_BUSSLINGER_ESOPHAGEAL_PR…	GPX4	PRC1	MCM7	KRT19	TK1	SMC4	IFITM3	H2AZ1	HOPX	PTMA	SLC3A2	SYNE2	CXCL14	TKT	CD81	NAP1L1	SNRPB	ARL6IP1	NUCKS1	FBL	HMGB1	HNRNPA…	ANP32B	HNRNPA1	ATP1B3
CELLMARKERMSIGDB_BUSSLINGER_GASTRIC_CHIEF…	SLC12A2	CXCL17	NEDD4L	GLUL	LINC00…	GPRC5C	TSC22D1	ELL2	PRDX4	LEPR	DCXR	APLP1	HMGCS2	METTL7A	XBP1	RPLP1	SEC62	ENAH	TMEM97	SSR4	PABPC4	ELAPOR1	CA9	IGFBP2	MECOM
CELLMARKERMSIGDB_BUSSLINGER_GASTRIC_G_CEL…	PDK4	DIRAS3	TM4SF4	MLXIPL	SUCNR1	IDH1	SCG3	SCGN	C12orf75	MAN1A1	SSTR2	SCG2	ISL1	SYT7	CADPS	NEUROD1	PCLO	RAB3B	QPCT	SLC7A1	LRP4	PDE8B	DST	DEPP1	PEMT
CELLMARKERMSIGDB_BUSSLINGER_GASTRIC_ISTHM…	JPT1	GPX2	FOXQ1	GSTP1	KRT18	CES2	TRNP1	FABP5	ACTN4	CLIC1	BCAS1	HSPA5	TUBA1C	MGST3	MGLL	S100P	LMNA	LIMA1	REG4	TSPAN1	CAST	TUBA1B	KLF2	IL1RN	SULT1C2
CELLMARKERMSIGDB_BUSSLINGER_GASTRIC_NECK_…	RGMB	ERGIC1	ERN2	PIK3C2G	RRBP1	CST3	CREB3L1	NOP53	HDLBP	EGR1	PIGR	CD24													
CELLMARKERMSIGDB_BUSSLINGER_GASTRIC_OXYNT…	AZGP1	QDPR	RAB3B	ALDH1A1	KIF1A	ABCC8	CLPS	CAMK2B	C4orf48	PTPRN	PDZRN3	PROX1	BEX1	ERO1B	SERPINA1	ANK2	SMOC2	ACSL1	CCKBR	CPLX2	OR51E1	REV3L	DEPP1	GCNT2	ECE1
CELLMARKERMSIGDB_BUSSLINGER_GASTRIC_PREZY…	IL33	JUN	LINC00…	ARHGAP18	ADAM28	CDKN1C	MECOM	EPHB3	FOXP1	NFKBIZ	IER2	ZNF395	SLC4A4	PABPC4	GADD45B	ARSD	MAGI1	PLCB4	NBEAL1	FMOD	TCEA3	CXADR	GOLGB1	ERN2	PGC
CELLMARKERMSIGDB_BUSSLINGER_GASTRIC_REG3A…	STAT1	CXCL2	FAM3D	CXCL3	SOD2	IFI27	WARS1	SAT1	SULT1C2	HLA-B	PSME2	KCNE3	ALDH2	B2M	HLA-A	ID1	TMSB10	CD24	ANXA10	HSPA8					
CELLMARKERMSIGDB_CUI_DEVELOPING_HEART_5TH…	DGCR6	CYB5A	RGS3	GJA1	SLIT2	HEY2	OLFML3	DBI	EMCN	ISOC1	EIF2S3	NFIA	COX7A1	COL3A1	C7	H4C3	ESAM	CMTM7	CD24	ATP1A3					
CELLMARKERMSIGDB_CUI_DEVELOPING_HEART_5TH…	IGFBPL1	GATM	TUBB6	HSDL1	NR2F2-…	HEY1	AIF1L	SVIP	MARCHF3	SORD	EGR2	CPNE5	H2BC21	PREP	SYNPO2L	LINC00842	LINC00…	ACTA1	KLK6	B3GNT2	DUSP5	STAR	PJA1	CPE	ULK4
CELLMARKERMSIGDB_CUI_DEVELOPING_HEART_C1_…	REC8	DDIT3	MACROH…	TNFRSF12A	PDPN	COA1	HSPA5	RASD1	TXNDC17	CNN1	NME4	CXADR	FDFT1	KPNA2	CKB	EIF3M	PDHA1	GMNN	CRABP2	IDH2	ITGB1BP2	MDH1	MYRF	BZW2	CALB2
CELLMARKERMSIGDB_CUI_DEVELOPING_HEART_C2_…	NPPA	COX7A1	FITM1	HSPB3	MYBPC3	ANKRD1	MDH1	TTN	SH3BGR	MB	SORBS2	COX5A	ENO3	COX6A2	MYL3	PDLIM5	SYNPO2L	LINC008…	RBM24	PPP1R3C	SMYD1	TRIM55	HSPB2	CRIP2	FHL2
CELLMARKERMSIGDB_CUI_DEVELOPING_HEART_COM…	BANCR	LGALS3	COL12A1	UGDH-AS1	IL32	RBP7	PDK4	COL15A1	RNASE1	TIMP2	AHSP	SRGN	COL6A2	TCF21	ASPN	ALDOC	HLA-B	NEFL	LPP	C7	CASQ2	NIBAN1	CXCL8	ITM2A	COL5A2
CELLMARKERMSIGDB_CUI_DEVELOPING_HEART_COR…	ADGRL4	C1QTNF9	CA4	GIMAP7	ROBO4	CD74	MEOX1	MEF2C	MTSS1	CAVIN2	GIMAP4	APOD	FLT4	GMFG	RBP7	ARHGAP18	ITGA6								
CELLMARKERMSIGDB_CUI_DEVELOPING_HEART_END…	PLVAP	ST6GAL…	TMEM100	MYH6	QSOX1	CLEC11A	SMOC1	COL18A1	PGAM2	FOXC1	KLHL4	TMEM141	NT5E	ANGPTL4	SRPX	PLAC9	IRX3	IL33	ST6GAL1	EMILIN1	BMPER	ENPP1	EPHA4	DSG2	ATP1B1
CELLMARKERMSIGDB_CUI_DEVELOPING_HEART_LEF…	NPPC	PLK2	EMCN	ISOC1	GJA1	ANXA1	PHLDA1	SCUBE3	BRIP1	KCNJ2	CMC1	POSTN	S100A11	SOX11	TRAIP	SOX4									
CELLMARKERMSIGDB_CUI_DEVELOPING_HEART_RIG…	DES	MYLK3	BMAL2	RNF187	SPRYD3	DPP9	RASSF8	NES	GHRH	NR4A2	COL9A1	RTN4R	INPP1	LINC01…	MTMR9	POFUT2	DAGLB								
CELLMARKERMSIGDB_CUI_DEVELOPING_HEART_SMO…	NTRK2	OLFML2B	CTSK	LOX	BGN	CFH	CPE	C1QTNF3	F10	TSHZ2	S100A4	ID2	PPIC	MFAP5	COLEC12	DUSP1	RNU6-2	NUCB2	ACVR2A	RARRES2	OSR2	NPDC1	HEXIM1	EGFL6	
CELLMARKERMSIGDB_DESCARTES_FETAL_ADRENAL_…	SLC22A1	HMBOX1…	LINC02…	MYADM-AS1	RXYLT1	ZNF786	TNFRSF1…	H2AC17	PBDC1	RN7SL74…	GALNT9	VAV3-AS1	HSD3B2	CFAP47	KIRREL1-…	ZNF747	CADM1-…	AMIGO1	TYMSOS	RNU6-51…	LMNB1-DT	PTPN23…	CTF1	PNMA6F	SAXO1
CELLMARKERMSIGDB_DESCARTES_FETAL_CEREBELL…	PRPH	BARHL2	MUC19	DRD4	RPSAP43	MCHR2	HMCN2	RGS5-A…	LINC001…	GPC5-AS1	KLK4	NREP-AS1	ASB15	INSM2	RPS7P8	HMGN1P14	LINC00…	DYTN	RNA5SP…	LINC016…	KLKP1	RPL21P…	RNU4-70P	LINC02…	
CELLMARKERMSIGDB_DESCARTES_FETAL_CEREBELL…	ENSG000…	ENSG00…	FNDC1-…	TFAP2A-A…	LINC02…	ALKAL2	NNMT	LINC02…	LINC005…	LBX1	LINC013…	C1QL3	LARGE2	GOLGA6…	LINC02502	LINC01358	RN7SL8…	NPY2R	ENSG00…						
CELLMARKERMSIGDB_DESCARTES_FETAL_CEREBELL…	ADAM28	TFEC	C1QC	CYBB	CD53	BLNK	LY86	P2RY13	APBB1IP	MS4A7	C1QA	PLD4	IRF8	HPGDS	CD68	CSF3R	HMOX1	RBM47	C1QB	LPAR5	TYROBP	ITGB2	PIK3R5	LINC02…	SPI1
CELLMARKERMSIGDB_DESCARTES_FETAL_CEREBELL…	LINC002…	SEMA3E	ANKRD3…	CORIN	GPR39	LINC01…	ABCA17P	ARHGEF…	FAM153CP	LINC020…	FGF19	VIT	ENSG00…	TDRD5	COL6A5	MIR4527HG	PENK-A…	IFNG-AS1	RPS20P5	WNT16	ESRP1	ENPP7P8	RPS26P15	LINC01…	FOXP4-…
CELLMARKERMSIGDB_DESCARTES_FETAL_CEREBELL…	SCUBE1	LPA	DDX50P2	OPRD1	ANKRD2…	TLL2	GOLT1A	LINC01…	PHEX-AS1	LINC019…	LINC011…	ZNF860	PPP3R2	C6	ALPK2	ENSG0000…	MANCR	RNA5SP2…	ANAPC1…	RNU6-12…	PEX5L-AS2	RNA5SP…	FNDC9	IL31RA	PNMA6F
CELLMARKERMSIGDB_DESCARTES_FETAL_CEREBELL…	LECT2	ARSJ	HTR5A-…	ENSG0000…	PCARE	ENSG00…	LMO7DN-…	MARK2P…	SSBP3P1																
CELLMARKERMSIGDB_DESCARTES_FETAL_CEREBELL…	SULT1E1	FOXQ1	CLEC3B	CLDN5	APLN	FAM124B	PCAT19	ACVRL1	ADGRL4	ERG	LRRC32	DSP	KDR	FLT1	CAVIN2	CAVIN3	SLC6A12	FOXF2	IGFBP7	LSR	TBX2	DPEP1	CDH5	FOXS1	STRA6
CELLMARKERMSIGDB_DESCARTES_FETAL_CEREBRUM…	DRD3	SCGN	DLX2	SIM1	SLCO6A1	ENSG00…	DLX5	GPR6	CENPS-C…	TAC3	FNDC9	CLRN1-A…	BRS3	ISL1-DT	SLC6A5	SHOX	LHX8	NNMT	LINC02…	LRRC52-…	HMGB1P47	SP8	MME-AS1	LINC01…	
CELLMARKERMSIGDB_DESCARTES_FETAL_CEREBRUM…	SYNE4	LINC02…	RMEL3	LINC00868	TMEM215	LINC01…	LINC021…	RPL21P…	LINC011…	LINC016…															
CELLMARKERMSIGDB_DESCARTES_FETAL_CEREBRUM…	LYL1	RIPOR3	ANGPT1	TSC22D4	NEXN	ELOVL7	MPP7	TJP2	F11R	CCND3	KCNC3	CTSA	MYOM1	RGS18	GMPR	CD58	PTGIS	SH3TC2	TNFAIP8	IRS2	H2AC6	MFSD2B	PSTPIP2	OCIAD2	CD96
CELLMARKERMSIGDB_DESCARTES_FETAL_CEREBRUM…	PIK3R5	ITGAM	HMOX1	LINC02642	SLC11A1	KCNK13	GPR34	LINC02…	LINC002…	EGR3	PDK4	TMEM144	PIK3AP1	FGL1	TLR1	LAT2	RBM47	VAV1	RAB20	MYO1F	PLA2G7	LGMN	XACT	NPL	SOCS6
CELLMARKERMSIGDB_DESCARTES_FETAL_CEREBRUM…	MYT1	MMP16	SCRG1	NKX2-2	NOVA1-…	OLIG1	ASIC4	NT5E	KLRC4-K…	KLRC2	MYRF	LINC022…	BMP8B	GAL3ST1	KLRC3	KLRK1-AS1	H1-9P	CHAD	SSTR1	LINC030…	GDNF-AS1	MAG	MIR219A…	LINC00…	ENSG00…
CELLMARKERMSIGDB_DESCARTES_FETAL_CEREBRUM…	TRPC3	PDE1C	HS3ST2	LINGO3	CORIN	LINC01…	ARHGEF33	SYT2	ABCA17P	FAM153CP	ENSG000…	TDRD5	NGFR	CHRNA3	ENSG0000…	FGF19	COL6A5	CFTR	SKOR1	WNT16	FAM153A	SPINT1	LINC014…	SLC18A3	GNAT3
CELLMARKERMSIGDB_DESCARTES_FETAL_CEREBRUM…	ARHGEF15	CAVIN3	SEMA3G	CARMN	SOX7	DPEP1	FLT4	FAM124B	ENSG000…	HTRA3	SULT1E1	DSP	LAMC3	SLC6A12	VAMP5	TBX18	NOTCH4	HIC1	CCDC141	SLCO2A1	ITIH5	LINC02…	ADGRL4	GPER1	EDN1
CELLMARKERMSIGDB_DESCARTES_FETAL_EYE_ASTR…	GFAP	ALDH1L1	CTXND1	SCRG1	FXYD2	FMO1	PLAAT5	KCNJ16	MIDEAS-…	LINC029…	TNFRSF1…	NKX6-1	EHBP1-…	SOX3											
CELLMARKERMSIGDB_DESCARTES_FETAL_EYE_GANG…	TMEM74	B4GALN…	DDX50P2	RAB37	LINC01…	SYT2	TUBBP5	LGI3	FNDC9	SLC17A6…	IRX4	C4orf50	LINC02…	RPL31P…	ENSG0000…	GPR139	AKAIN1	LINC025…	RN7SL1…	IL1RL2	MTUS2-AS2	LRRC73	GRASLND	C1QTNF…	LINC02…
CELLMARKERMSIGDB_DESCARTES_FETAL_EYE_LENS…	COL4A6	LINC02…	CAMK2A	LIM2	MBOAT1	CRYGEP	DNMBP	ME1	MIR583HG	CAPRIN2	TIMELESS	BHMT	CERK	LCTL	LINC01170	GSS	CCDC169	GJA3	CRMA	LNPK	RSPO4	LINC02…	BIRC7	LINC00…	RAB39A
CELLMARKERMSIGDB_DESCARTES_FETAL_EYE_PHOT…	C7orf33	MTATP6…	SUSD2	NPTX1	LINC00…	SSTR5-…	HOTAIRM1	OLAH	GNAT1	MTCO1P15	MPPED2-…	ENSG000…	MTND1P…	MTCO2P…	SAMD7	LINC01915	GNAT2	PRICKLE…	RPL18P…	RPL30P7	TERT	LRIT2	HTR1F	LINC03…	DRGX
CELLMARKERMSIGDB_DESCARTES_FETAL_EYE_RETI…	TMEM98	SFRP5	TRPC4	SLC6A13	SLC6A20	MROH7	TTR	SLC39A…	HORMAD2	PRDM16-…	SLC26A7	CNDP1	RLBP1	RGR	INPP5K	PRKCQ	STRIP2	ELOVL7	PCAT1	LRAT	BCO1	SLC16A8	SLC22A8	PAFAH1…	TLCD4
CELLMARKERMSIGDB_DESCARTES_FETAL_EYE_SMOO…	HPSE2	C12orf…	GINS2	SEC16B	SLC12A4	GLMP	KCNAB2	PNMA3	MARF1	LRRC47	PRKG2	METTL9	SRRM2-…	CECR3	FOXRED2	DERL3	NUDT16…	FUZ	TCEA1	RAB13	UNC119B	TESK1	TRPM6	C11orf…	LINC01…
CELLMARKERMSIGDB_DESCARTES_FETAL_EYE_STRO…	LTBP4	ISLR	THBS1	DKK2	CXCL12	ADAMTS5	FNDC1	ASPN	COL6A3	THBS2	ADAMTS2	IGFBP6	DPT	EGFL6	SCARA5	LINC02511	IGSF10	SULT1E1	CREB3L1	C1R	CTHRC1	EMILIN1	ADAMTSL4	FMOD	TWIST1
CELLMARKERMSIGDB_DESCARTES_FETAL_EYE_VASC…	PKD1L1	CAVIN2	FGD5	ROBO4	DUSP6	VASH1	VWF	TEK	SMAD7	ABCG2	HAPLN1	ACVRL1	THSD1	SOX18	RAMP3	KANK3	PDE2A	LXN	SELP	LRRC32	ALPL	CLDN5	FLT4	CLEC14A	IHH
CELLMARKERMSIGDB_DESCARTES_FETAL_HEART_EL…	TACSTD2	CYP2W1	DEGS2	MS4A8	ENSG00…	TTC23L	ANKRD2	SIX1	SLC44A4	RSPH1	CLIC6	WIF1	MUC1	SAXO2	RPL10P9	EFCAB12	CAPS	DNAH5	RXYLT1…	CFAP95	ZBBX	LINC02…			
CELLMARKERMSIGDB_DESCARTES_FETAL_HEART_EN…	ST6GALN…	LRATD1	LYPD2	LINC01099	LINC01…	WNT9B	SOST	LINC01…	LINC022…	ANGPTL5	FAM3D	LINC013…	RASSF1…	PDE3A-…											
CELLMARKERMSIGDB_DESCARTES_FETAL_HEART_LY…	HOXD9	ADGRG3	MFSD4A	ABCA4	ADD3-A…	C6orf1…	C20orf2…	TSPAN8	LINC006…	P4HA3	LINC028…	GPR182	HOXD10	FZD10	ZNF24TR	LINC02840	TMEM25…	LINC011…	HOXD4	PGM5-AS1					
CELLMARKERMSIGDB_DESCARTES_FETAL_HEART_SC…	TFAP2A	ST6GAL…	METRN	ASPA	NR4A2	RXRG	TENM3-A…	CHST9	PLXNB3	COL20A1	GINS3	MIA	CTXND1	NLGN3	PMP2	ALDH1A3	ATP10B	LINC003…	LINC01…	KCNJ10	MYOT	LINC01…	GRAMD2A	LINC00…	RHEBL1
CELLMARKERMSIGDB_DESCARTES_FETAL_HEART_SM…	FOXS1	ACTG2	PDE4C	TBX2-AS1	HIGD1B	LMOD1	RGS16	ERRFI1…	LINC022…	ACTA2-A…	ADRA1D	TMEM74B	SEPTIN…	CCDC190	LGR6	GPR20	BCAS3-…	FOXL1	HCG22	ILDR1	CACNA1S	APOL5	PELO-AS1	RGS5-A…	
CELLMARKERMSIGDB_DESCARTES_FETAL_HEART_ST…	EMILIN3	CILP	AGTR2	ANGPTL1	LPAR4	ANGPTL6	NPTX2	C1QTNF…	RTL3	CCL11															
CELLMARKERMSIGDB_DESCARTES_FETAL_HEART_VI…	SLC5A7	PCBP3	FGF14-…	SLC7A14	TMEM130	CA10	GAL	ACHE	ISL1	EYA4	NECAB2	FAM163A	PLPP4	CHRNA7	OPRM1	SYT9	LINC02…	KCNK3	PIRT	KCNH1	PIANP	NEFH	SLC18A3	RGS9	PHYHIPL
CELLMARKERMSIGDB_DESCARTES_FETAL_INTESTIN…	RASGEF1C	TRIM9	ABCA8	CSMD2	LINC01…	SHC4	COL22A1	TTYH1	ATP1A2	FLRT1	FOXD3	LINC004…	LAMP5	SERPIN…	NPTX2	HEYL	SYNPR	FOXD3-A…	HCRTR1	ZNF788P	PAQR6	SOX8	GPR12	RLBP1	H1-9P
CELLMARKERMSIGDB_DESCARTES_FETAL_INTESTIN…	KLHL14	SLC30A3	LINC00…	ABCA4	LINC02…	ZNF24TR	LINC019…	DNAJC8…	APP-DT	ART5	RPL17P36	GALNT16…	PM20D1	GNAT3	ADRB2	DSCR9									
CELLMARKERMSIGDB_DESCARTES_FETAL_INTESTIN…	DSC3	LINC02…	MTUS1-…	RSPO1	EPS8L1	NEK5	SLPI	FGF18	TMEM191A	C21orf62	NAT8L	SPHKAP	KLK11	CRB2	KLF2-DT	FAM110C	ADAMTS…	NPHS1	SCEL	AOX3P	AOX1	COL8A2	TMEM255A	TMEM15…	SCNN1A
CELLMARKERMSIGDB_DESCARTES_FETAL_INTESTIN…	LINC005…	JPH2	FGF7	FST	OLFML2B	KCNMB1	LDB3	AOC3	SERTAD4	CHRDL2	ADRB3	SLITRK3	FBXL22	NOG	ACTA2-AS1	KCNJ8	IL17B	TACR2	CAPN3	KCNA1	TNMD	POPDC3	TTC23-A…	OVCH2	NIPAL4
CELLMARKERMSIGDB_DESCARTES_FETAL_INTESTIN…	EDAR	LPAR4	CCL19	F2RL2	NXPH1	FAM167B	GLI1	OR7E47P	CH25H	COL6A5	ADH1B	FNDC1-I…	ENSG000…	FZD10	FNDC1-…	MMP27	MSC								
CELLMARKERMSIGDB_DESCARTES_FETAL_INTESTIN…	DLL4	LINC01…	LHX6	DIPK1B	LINC00924	SPTBN5	ESM1	FABP4	APLN	SOX17	FABP5	SELP	MSX1	FAM110D	GPR4	RP1L1	VSIG2	NT5ELP	TNNT3						
CELLMARKERMSIGDB_DESCARTES_FETAL_KIDNEY_M…	PHACTR3	COL8A1	REN	LINC00924	INSYN2A	RARRES1	MYOCD	SLC7A14	PITPNM3	SEZ6L2	NKD2	SHC3	RMDN2-…	LINC02…	BDNF	FOXS1	TYRP1	IGLON5	TBX22	SLC6A1	LINC01914	PGM5P4	CGB7	PITX3	FGF19
CELLMARKERMSIGDB_DESCARTES_FETAL_KIDNEY_U…	FOXQ1	MACC1	L1CAM	LINC00645	CLDN4	MYO3B	KCTD8	SNTG1	UPK3A	PRDM16	ESRP1	ACER2	KRT8	SAMD12	BCAS1	SNCG	SPINK1	TFAP2A	ADGRF1	PDE7B	ALDH1A1	RDH10	C4orf19	MAL2	GRHL2
CELLMARKERMSIGDB_DESCARTES_FETAL_LIVER_ER…	UBAC1	GYPE	FHDC1	ALAD	EPOR	MINPP1	ART4	TRAK2	RHD	SLC22A4	RHAG	UROS	PTH2R	ALAS2	EPB42	KCNH2	KLF1	RFESD	ACSBG1	FLACC1	SLC38A5	CD46P1	ABCC13	DNAJA4	ACHE
CELLMARKERMSIGDB_DESCARTES_FETAL_LIVER_HE…	HOXA6	CDH9	HTR1F	HOXA7	CFAP92	LINC01…	HSPE1-M…	CPNE7	INPP5J	USP6	ICA1-AS1														
CELLMARKERMSIGDB_DESCARTES_FETAL_LIVER_HE…	PLG	AMBP	KLB	HNF4A	SHANK2	SERPIN…	APOA1	BHMT	MLXIPL	SLC13A5	AFP	KNG1	CYP4A11	CYP19A1	GPC3	LINC00470	GAS2	APOM	G6PC1	PRAP1	ITIH3	ASGR1	TF	GLS2	SLC38A4
CELLMARKERMSIGDB_DESCARTES_FETAL_LIVER_ME…	TFPI2	CGN	PODXL	ELN	CAVIN1	S100A10	LINC030…	NPNT	RASSF8	COL21A1	RALYL	MSLN	NEBL	LHX9	LMO4	PTPRQ	SLC4A4	EGR1	LURAP1L	OGN	MYRF	TRABD2B	GADL1	KRT19	GAS1
CELLMARKERMSIGDB_DESCARTES_FETAL_LUNG_BRO…	TPD52L1	UGT2B7	MAP3K21	ADCY8	DACT2	CLDN2	EDN3	LINC03…	ITGB6	FAM41C	CCDC198	SUSD2	SLC19A3	IL12A-…	PPP1R3A	FREM3	CDH16	KIF12	PTCSC3	LINC023…	LMO7DN	KCNJ5	FGF20	IRX6	PALM3
CELLMARKERMSIGDB_DESCARTES_FETAL_LUNG_CIL…	DNAH5	TEKT1	LINC02…	CFAP43	SPAG6	KIAA20…	LMNTD1	FHAD1	SERPINI2	RSPH1	CFAP77	ADGB	TMEM231	CFAP299	SPATA18	CFAP276	DNAAF1	DNAI3	NEK10	HYDIN	MROH9	ZMYND10	C9orf24	SPAG17	CCDC60
CELLMARKERMSIGDB_DESCARTES_FETAL_LUNG_LYM…	HOXD3	LRRN4CL	SLC27A3	GGT5	HAPLN3	C6orf1…	NFIA-AS2	ABCA4	P4HA3	ADD3-AS1	HOXD9	ZP2	UNC5A	C5orf64	LINC00636	ADAMTS4	FZD10	LINC026…	GPR182	ZNF24TR	HOXD-AS2	MSMP	LINC011…	LINC01…	FOXC2

Table S5: Cell state marker gene list

CELLMARKERCAN_ACINAR_CELLS	PNLIPRP1	CTRC	REG3A	SERPINA3	PRSS3	REG1B	CFB	GDF15	MUC1	C15ORF48	DUOXA2	AKR1C3	OLFM4	GSTA1	LGALS2	PDZK1IP1	RARRES2	CXCL17	UBD	GSTA2	ANPEP	LYZ	ANGPTL4	ALDOB	
CELLMARKERMSIGDB_DESCARTES_FETAL_LUNG_NEU…	GRIK1	KCNJ6	NPTX1	SV2B	COL22A1	RPRM	GHRLOS	GFRA3	KSR2	MAOB	SYT13	HTR2C	KLK12	KCNH2	MMP26	RASD1	SLC38A…	PCSK1	SLC18A1	AMIGO2	RGS4	NKX2-2	VWA5B2	NPSR1-…	DLL3
CELLMARKERMSIGDB_DESCARTES_FETAL_LUNG_STR…	PTH1R	C2CD6	PHEX	FGFR4	AGMO	VEGFD	CACNA1G	CYP27C1	LINC023…	FGF7	TRHDE	TEX15	PGR	TDO2	LURAP1L-…	SLIT2-IT1	KIF25	XPNPEP2	MUSK	CACNA1C…	HSD11B1	MTMR12…	TM6SF2	TULP1	ZNF385…
CELLMARKERMSIGDB_DESCARTES_FETAL_LUNG_VAS…	BTNL9	FCN3	MADCAM1	SHROOM1	TMEM100	PRX	APLN	BBOX1-…	DPEP1	MYOC	TCF15	ULBP1	ESM1	ENSG00…	COLEC10	ADORA2A	FOXS1	SLC22A25	ULBP2	RN7SL69P	SELE	LINC02…	CD300LG	ENSG00…	RN7SL5…
CELLMARKERMSIGDB_DESCARTES_FETAL_MUSCLE_L…	CYTL1	RASSF9	NALCN	GJC2	PARD6G	KBTBD11	EFCC1	RAPGEF4	TSPEAR	STAP2	P4HA3	SLC39A14	PLIN5	CXADR	UNC5A	MYL12-AS1	TPMT	LINC021…	C6orf1…	KLKB1	SLC35E4	DLK2	NFIA-AS2	CELSR1	ANKRD3…
CELLMARKERMSIGDB_DESCARTES_FETAL_MUSCLE_S…	LMF1-AS1	KRT40	ADGRA1	LINC02821	GALNT14	LINC01…	NEU4	USP44	CCER2	GCK	RNY4P34	LINC011…	RPS3AP…	MAGEC3	CELA2B	CHRDL2	AMH	CCDC162P	MAPK15	ANKRD18B	LINC02367	BNC1	SPATA13	MYEOV	LINC01…
CELLMARKERMSIGDB_DESCARTES_FETAL_MUSCLE_S…	RCAN2	DAAM2	LINGO1	GPR176	C1QTNF…	ANO1	TEX41	EEF1DP3	RASL12	STEAP4	OR51E1	SLC38A11	FAM162B	PLN	MAPT	RRAD	ACTG2	FRK	OR51E2	ADCY5	FHL5	COX4I2	LINC007…	FOXS1	ITGA11
CELLMARKERMSIGDB_DESCARTES_FETAL_MUSCLE_V…	DLL4	APLN	MADCAM1	CCM2L	VEGFC	TMEM88	GRIA2	ADAMTS…	C2CD4B	TAFA2	NOSTRIN	C1QTNF9	NRN1	C1orf1…	FAM124B	TMC1	SOX17	NOS3	CA4	SPNS2	FAM167B	PRSS51	MAPK11	GRIN2A	TMEM15…
CELLMARKERMSIGDB_DESCARTES_FETAL_PANCREAS…	CPB1	ERP27	SPINK1	DPEP1	PTF1A	KLK1	SLC39A5	MT1X	MT1E	AMBP	MT2A	SEL1L	GATM	SLC16A…	ALB	FBXO2	IL22RA1	POMC	CUZD1	RNF186	CELA2A	CBS	REG4	KLB	SPX
CELLMARKERMSIGDB_DESCARTES_FETAL_PANCREAS…	PRPS2	GRIN2B	PCDH11X	ALPK2	AARD	PJVK	COL6A5	OCA2	COL7A1	MYBPC2	NPFFR1	LINC016…	MINCR	TPBGL	LPAL2	FNDC1-AS1	CDX1	HOXC9	ADGRF1	EIF5-DT	PTGER1	DAZL	PCDH11Y	HS3ST6	RPL27P6
CELLMARKERMSIGDB_DESCARTES_FETAL_PANCREAS…	SHC4	FOXD3	TFAP2A	CNP	ASPA	HS3ST4	MEGF10	RASGEF…	SOX2	SYNPR	CMTM5	CRYM	INSC	MIA	FAM181B	ST3GAL6-…	BCAN	B3GALT2	LINC01…	GINS3	PAQR6	SLC16A4	ST8SIA5	NLGN3	TAC1
CELLMARKERMSIGDB_DESCARTES_FETAL_PANCREAS…	CEMIP	IRX3	TNFAIP…	FZD10	C20orf…	FOXC2	TNFRSF9	BMPER	INO80C	LINC012…	CXCL5	BMP6	TBX1	ELMOD1	LRRC69	ABCA4	ADD3-A…	GNA14-A…	DHODH	SLC30A3	PDF	TRIM54	KCNJ2-A…	LINC00…	GPR182
CELLMARKERMSIGDB_DESCARTES_FETAL_PANCREAS…	AOX1	PTPRQ	INMT	PPP2R2C	FGF1	SILC1	RPRM	SMTNL2	VTN	GRIN3A	BNC1	LINC023…	BDKRB1	CLDN15	TNNT1	LRRN4	BRINP2	LRP2	CLIC5	ATP7B	CFB	CHAC1	TGM1	C19orf…	CRB2
CELLMARKERMSIGDB_DESCARTES_FETAL_PANCREAS…	OR51E2	SGCA	PLN	CASQ2	TLX1	LINC02…	NTF3	LINC00…	LINC007…	MUSTN1	CIDEA	ACTG1P25	ANKRD2…	LINC03…	SFN	AOC4P									
CELLMARKERMSIGDB_DESCARTES_FETAL_PANCREAS…	TCIM	TMEM88	CD320	BCL6B	ALPL	IFI27	COL13A1	SOX17	LHX6	FAM167B	TCF15	BHLHE40…	APLN	EXOC3L1	SSUH2	FAM124B	UPB1	SLC9C1	ESM1	OIT3	AVPR2	ADAD2	MADCAM1…	ENSG00…	ODF3L2
CELLMARKERMSIGDB_DESCARTES_FETAL_PLACENTA…	ASAP3	COL17A1	SEMA4C	ASPSCR1	LAIR2	FAT2	CCNE1	CLDN19	GPR146	KCNK12	SYT8	PTGES	MYCN	PYCR1	LDLRAD2	EGFR-AS1	MYCNOS	STAB2	LY6K	KRT16P6	RTN4RL2	AADACL3	RNF223	PTPRQ	TNNI2
CELLMARKERMSIGDB_DESCARTES_FETAL_PLACENTA…	KCNK3	NOTCH3	ADAMTS…	WNT3A	PELO	ADARB2	POSTN	FAM162B	RGS20	BMPR1B	CARMN	KCNS3	COX4I2	GRID1	SCN11A	CCDC3	OLFM2	PDLIM3	CSPG4	LZTS1	ADAMTS20	GNAL	NDUFA4L2	CHRM2	CLTCL1
CELLMARKERMSIGDB_DESCARTES_FETAL_PLACENTA…	GXYLT2	GBX2-A…	OCA2	C2orf83	HSD3BP5	EEF1DP3	LIN7A	GLI2	COL16A1	COL8A2	SELENBP1	PITX1	SLC6A15	DES	ACKR1	GDF6	LINC02…	ZNF804B	PTGFR	CACNA2D4	LINC01931	LINC02…	ASB4	LINC02…	CYP2W1
CELLMARKERMSIGDB_DESCARTES_FETAL_PLACENTA…	FRMD6	AP1S3	L1TD1	ADCY2	PRSS12	ZNRF3-…	MAP7D2	DSC3	FAM118A	CRISPLD1	MCOLN3	LINC012…	MIR205…	EDARADD	TENM3-AS1	OFCC1	VSTM5	SIGLEC6	CYP2J2	LINC022…	TRPV4	ATP6V0…	TTPA	HSPD1P6	LRATD1
CELLMARKERMSIGDB_DESCARTES_FETAL_PLACENTA…	ALPP	CAPN6	CSHL1	HSD11B2	NECTIN3	CHODL	RYBP	DEPDC1B	CCSAP	TCL6	CGB3	MAN1C1	PSG11	CRH	LGALS14	LINC01483	BPGM	HSPB8	TRPV6	ZNF117	ENDOU	INSL4	PSG8	CCK	C4orf36
CELLMARKERMSIGDB_DESCARTES_FETAL_SPLEEN_E…	CALN1	EPB42	PRSS57	ERMAP	HDC	MINPP1	GYPE	EPOR	SLC22A16	SLC2A14	PCLAF	ART4	SLC22A4	ACSBG1	TRIM10	EIF5AP2	DHRS13	DMC1	SLC6A9	E2F2	IL1RL1	SLC14A1	ATP13A3…	SLC29A1	TESMIN
CELLMARKERMSIGDB_DESCARTES_FETAL_SPLEEN_S…	ST6GALN…	LINC00…	PHACTR3	LINC03092	OVCH1	DNM3OS	LRRC52-…	PRR16	LINC020…	ADRA1B	LRRC3B	LYPD6B	KCNIP1…	ZNF804B	MIR3659HG	TPH2	EYA1	P3H2-AS1	ENPP7P4	MKX	LINC02126	TACR3	RPL21P99	SCTR	NPM1P10
CELLMARKERMSIGDB_DESCARTES_FETAL_SPLEEN_V…	KIAA1671	DGKE	SNCAIP	GREB1L	NOSTRIN	KLHL4	PTCHD4	FAP	CHRNA7	HOXD3	ZNF503-…	RGS7BP	CALCR	SLC5A4	UNC80	LINC03002	GCOM1	TMEM233	PROX1-…	OR5G5P	LINC01594	ALG1L6P	LINC021…	LOC102…	LINC02…
CELLMARKERMSIGDB_DESCARTES_FETAL_STOMACH_…	TMEM190	CCDC170	TPPP3	CCDC190	DNAI1	ADGB	ERICH3	DNAH6	KCNE1	TTC29	ANKFN1	LMNTD1	CDC20B	RSPH1	CCDC30	CFAP73	DTHD1	CFAP70	FHAD1	DNAH10	SPAG6	SPATA17	CFAP221	SNTN	ZBBX
CELLMARKERMSIGDB_DESCARTES_FETAL_STOMACH_…	PRIMA1	SOX10	VGLL3	COL20A1	SLC15A3	EGFLAM	ENTPD2	CRYAB	ATP1A2	WDR86	TMEM71	LINC015…	AIF1L	LINC01…	CRYM	IGSF11	GABRA2	HEYL	ELOVL2	SNHG19	ADGRB1	CCN3	CALHM2	PPP1R1C	
CELLMARKERMSIGDB_DESCARTES_FETAL_STOMACH_…	FAM163A	PCBP3	ELAVL3	INA	LINC01…	EEF1A2	SOGA3	FHOD3	ATCAY	HOXB5	MIR137HG	TUBB2B	NOS1	MYT1L	SEZ6L	NPTXR	SLC5A7	LHFPL3	PRPH	KLF3-AS1	ANKRD29	STAC	MAPT	KCNQ3	SHF
CELLMARKERMSIGDB_DESCARTES_FETAL_STOMACH_…	CD28	STAT4	TRAF3I…	TXK	CD2	CCR7	SH2D1A	ICOS	CD5	SELL	PTPN7	CD27	LINC01…	GPSM3	SEPTIN1	TBC1D10C	PRKCQ	IL21R	IGHM	MFNG	RASGRP2	RASAL3	GATA3	FAM78A	GVINP1
CELLMARKERMSIGDB_DESCARTES_FETAL_STOMACH_…	SPHKAP	NXPH2	RSPO1	ENSG0000…	CBLN2	BST1	TGM1	HHIP-A…	TNNT1	CFB	BDNF	RN7SL18…	TMPRSS…												
CELLMARKERMSIGDB_DESCARTES_FETAL_STOMACH_…	KRT20	GUCY2C	LGALS4	ANXA13	GK	MEP1A	CES2	OLFM4	APOC3	ACSL5	PRSS3	MELTF	MGAM2	ACE2	MUC17	NR1H4	SCTR	SULT1E1	MT2A	CDHR2	HKDC1	EPS15-…	TM4SF20	HSD17B2	CLDN2
CELLMARKERMSIGDB_DESCARTES_FETAL_STOMACH_…	ADAP2	TBXAS1	RILPL2	CD14	MMP9	MPEG1	FGD2	RUBCNL	CD163L1	DNASE1L3	HLA-DMA	PLD4	HLA-DQ…	MERTK	PIK3AP1	HCK	CIITA	FCER1G	IL1B	C1QB	CLEC7A	ATP8B4	CD84	ITGAX	MS4A6A
CELLMARKERMSIGDB_DESCARTES_FETAL_STOMACH_…	DEPP1	FEV	FRMPD4	CRYBA2	LINC02…	NKX2-2	PTH2R	SLC18A1	DNAJC12	NPSR1-A…	ROS1	KCNH6	MBOAT4	CDK5R2	MEGF11	RFX4	RBPJL	PCSK1	PTF1A	AP3B2	RGS4	SYT13	GCK	LHX5-A…	HAP1
CELLMARKERMSIGDB_DESCARTES_FETAL_STOMACH_…	ANGPTL3	FGB	PNPLA1	CKMT2	EPN3	SULT2A1	AQP4	FRMD1	ODAM	MYBPC3	ASB11	MTCO3P12	SERPIN…	DHRS2	APOBEC2	ETNPPL	GLYATL1	CLEC3A							
CELLMARKERMSIGDB_DESCARTES_FETAL_STOMACH_…	A2ML1	PAX9	SPRR3	GBP6	KRT6B	NECTIN4	COL17A1	TMPRSS…	LY6D	NMU	TNS4	FAT2	SCNN1B	GJB3	TCN1	CAPNS2	CALML5	APOBEC3A	GALNT14	PLEKHN1	LINC01644	PCP4L1	KRT6C	TMPRSS…	FGFBP1
CELLMARKERMSIGDB_DESCARTES_FETAL_STOMACH_…	SCARA5	SRPX	TAGLN	TMEM119	BMP5	CAPN6	PLAC9	VSTM2A	SHISA3	FOXF2	DPT	ANGPTL6	OSR2	TWIST1	GLIS1	TWIST2	GLYATL2	ITGA11	CNN1	MDFI	ANGPTL1	INHBA	PTGER1	TRPA1	VSTM2A…
CELLMARKERMSIGDB_DESCARTES_FETAL_STOMACH_…	ESAM	BCL6B	TMEM233	TBXA2R	LINC01…	GALNT15																			
CELLMARKERMSIGDB_DESCARTES_FETAL_THYMUS_T…	LINC005…	LINC01…	ZFP1	TMSB15B	CD40LG	LTA																			
CELLMARKERMSIGDB_DESCARTES_MAIN_FETAL_ACI…	CEL	CELP	TPRN	PTF1A	SLC16A…	PNLIPR…	CUZD1	GPHA2	PLBD1	ERP27	PLA2G1B	FREM2-A…	SEL1L	DLK1	SCG5	MSRB1	NOMO1	MPV17L	GP2	AQP8	TBX6	MT1L	CTRL	TMED6	CTRB2
CELLMARKERMSIGDB_DESCARTES_MAIN_FETAL_ADR…	SPINK13	LINC01…	TNXA	CYP21A2	TNXB	GSTA12P	GSTA9P	GSTA4	LINC025…	RHBDD2	POR	STAR	LINC02…	GML	CYP11B1	DCAF10	WHRN	SVIL-AS1	SLC16A9	SNCG	PAPSS2	CYP17A1	DHCR7	AQP11	LINC02…
CELLMARKERMSIGDB_DESCARTES_MAIN_FETAL_AST…	PTCH1	PHYHIPL	FGFBP3	CYP26C1	CYP26A1	OTOG	HEPACAM	ST8SIA1	C2CD5-A…	DBX2	C1QL4	RFX4	FZD10	EEF1DP3	PCDH8	RNY1P5	LINC00…	LINC025…	LINC02…	ADGRG1	WIPF2	GFAP	WNT3	CYP4F2…	CKAP2L…
CELLMARKERMSIGDB_DESCARTES_MAIN_FETAL_BIP…	ENSG000…	LINC02…	MRPS30…	TRPC7	TRPC7-…	ENSG00…	GLRA1	LINC01…	NIPAL4-…	TDRD6	LRRC1	GUSBP4	GABRR1	MAP7	TMEM196	RN7SL83P	ZPBP	GNL2P1	ZNF804B	ENSG000…	TRMT9B	HSPA8P…	NRG1	NRG1-I…	NRG1-I…
CELLMARKERMSIGDB_DESCARTES_MAIN_FETAL_BRO…	SFTPA1	C10orf…	PRR13P2	WIF1	CPM	LMO7DN	LMO7DN-…	SLC10A2	ATP11A	ATP11A-…	PTCSC3	MBIP	SFTA3	NKX2-1	NKX2-1-A…	LINC02277	NPC2	GOLGA8B	DAPK2	CLDN6	HAS3	ENSG00…	CABLES1	CRLF1	KCNJ15
CELLMARKERMSIGDB_DESCARTES_MAIN_FETAL_CAR…	JMJD1C-…	CTNNA3	RBM20	RNA5SP327	NAV2-A…	MYBPC3	USP28	LINC02…	KBTBD13	HCN4	INCA1	MYOCD	MRPL58	FHOD3	ALPK2	KCNJ4	CPT1B	LINC011…							
CELLMARKERMSIGDB_DESCARTES_MAIN_FETAL_CIL…	FAM183A	CCDC24	ARMH1	DYNLT4	CCDC17	TSPAN1	KNCN	LDLRAD1	C1orf87	DYNLT5	DNAI4	C1orf141	LINC01…	ERICH3	ERICH3-A…	DNAI3	ODF2L	RWDD3-DT	LINC01…	CFAP276	PIFO	HIPK1	LINC017…	SPAG17	RIIAD1
CELLMARKERMSIGDB_DESCARTES_MAIN_FETAL_DUC…	CCN2	PRKAR1…	RABGEF…	CFTR	GSTK1	MNX1	MNX1-AS2	GS1-24…	HEY1	CALB1	PUF60	KIF12	TMEM141	TMEM72	OGDHL	FAM245A	HHEX	SFRP5	CALHM3	NEURL1-…	NEURL1	RASSF7	OR5G5P	FXYD2	FXYD6-…
CELLMARKERMSIGDB_DESCARTES_MAIN_FETAL_END…	SPARCL1	EMCN	DKK2	F2RL2	HAPLN1	ECSCR	LTC4S	FOXC1	NT5E	SLC35A1	CALHM5	GINM1	CCL26	BAALC-…	FZD6	CTHRC1	IL33	HRCT1	ENG	NPDC1	FUT11	MMRN2	CPXM2	PRCP	FZD4
CELLMARKERMSIGDB_DESCARTES_MAIN_FETAL_ENS…	HMX2	LINC02…	DLG2-A…	HTR3B	RPL10P…	NOS1	GPC6-AS2	HEATR5A	EML5	GPR176	LTK	ZNF843	CNGB1	HOXB4	HOXB5	DTD1-AS1	GRK3	PPEF1	FGF13						
CELLMARKERMSIGDB_DESCARTES_MAIN_FETAL_EPI…	SPATA48	CBLL1-…	CPA4	PHYHIP	TRPA1	NECAB1	CA9	FAM21FP	FAM133CP	UNC5B-A…	OLFML1	ARL14EP…	IL18	MKRN9P	MEDAG	KCTD4	TGM1	AQP9	SPATA41	MSLN	LINC00922	CALB2	CBLN2	ZNF676	ENSG00…
CELLMARKERMSIGDB_DESCARTES_MAIN_FETAL_GAN…	ENSG000…	POU4F1	ENSG00…	CRABP1	ENSG00…	TRARG1	LINC029…																		
CELLMARKERMSIGDB_DESCARTES_MAIN_FETAL_GOB…	AGR2	PSPHP1	SAMD12	TSPAN15	LIPF	MUC6	MUC5AC	FAR1	CREB3L1	B3GNT6	GPRC5A	LYZ	RFLNA	ERO1A	DUOX2	SLC30A4-…	RNU5B-1	PDPK2P	C16orf…	ERN2	IL17C	ENSG00…	TMEM238L	KRT18P…	CSF3
CELLMARKERMSIGDB_DESCARTES_MAIN_FETAL_HEM…	SPINK2	LINC02…	GALNT7…	LINC02100	S100Z	TRIM27	HCG25	KLC4-A…	ZP3	CDK6-AS1	ZNF789	LINC028…	NDUFAF6	UBR5-DT	BAALC	DENND3-A…	EIF2S2…	KCNIP2-…	ENSG00…	TRIM66	C1QTNF4	LINC00…	TM7SF3	BMAL2-…	MARS1
CELLMARKERMSIGDB_DESCARTES_MAIN_FETAL_HOR…	TLE4	MIR181…	TIGD3	DRD2	LOC574…	ERC1	ESRRB	ONECUT1	ENSG000…	MEGF11	CACNG3	PMFBP1	RAB11F…	LINC01…	LINC02565	ONECUT2	MALT1	MRPL37P1	ONECUT3	CACNG7	PAK5	ENSG00…	GRIA3		
CELLMARKERMSIGDB_DESCARTES_MAIN_FETAL_INH…	ROBO3	MGAT4C	ANO4	LHX5	SLC35F4	SKOR1	GLCE	CPLX4	CDH22																
CELLMARKERMSIGDB_DESCARTES_MAIN_FETAL_INH…																									
CELLMARKERMSIGDB_DESCARTES_MAIN_FETAL_INT…	GBA3	PRKG2	MTTP	PANCR	PRSS48	BTNL8	BTNL3	SLC17A4	TRIM15	TINAG	CYP2W1	MUC12	MOGAT3	SLC26A3	CLCN1	DEFA6	DEFA5	ANXA13	DGAT1	LCN15	SLC34A3	CUBN	MALRD1	ENTPD7	PDZD7
CELLMARKERMSIGDB_DESCARTES_MAIN_FETAL_ISL…	TBCC	SF3B5	SNX10-…	RASA4CP	GCK	STX1A	PAX4	SLC30A8	MAFA	CACFD1	NSMF	SUV39H2…	NEUROG3	ENSG00…	CALY	INS	KCNJ11	ABCC8	CNIH2	IAPP	PLUT	SYNDIG…	SSTR5-A…	SSTR5	RAB26
CELLMARKERMSIGDB_DESCARTES_MAIN_FETAL_LYM…	EFNA5	LINC02…	LOX	KLHL3	FLT4	CYP2AC…	GJA1	LINC01…	ENSG000…	RADIL	ARL4A	GNG11	TMEM140	GIMAP5	KBTBD11	GFUS	MSMP	CNTNAP3B	PGM5	PGM5-AS1	ENSG0000…	TCTN3	UBTD1	ADD3-A…	IRAG1-…
CELLMARKERMSIGDB_DESCARTES_MAIN_FETAL_MES…	KRTDAP	CA11	KLK10	HAS1	PLCB1	RPRD1B	NFATC2	MIR548…	RNU4-45P	C21orf62															
CELLMARKERMSIGDB_DESCARTES_MAIN_FETAL_MET…	KCNJ1	SINHCAF	DENND5B	ADAMTS20	HOTAIR	HOXC11	HOXC-AS3	HOXC10	HOXC8	WASF3	SLC12A1	LINC022…	UMOD	CLEC18B	MYHAS	SOST	LINC03…	SELENOV	SHISA8						
CELLMARKERMSIGDB_DESCARTES_MAIN_FETAL_MYE…	AIF1	HLA-DQ…	CALHM6…	ENSG0000…	AOAH-I…	NAMPT	WWP1	CCDC26	MTND5P14	MANCR	SRGN	RRP12	VENTX	SPI1	MS4A6A	MS4A4E	MS4A4A	MS4A7	CABP4	JAML	CD163L1	CD163	CLEC4A	CLEC7A	CMKLR1
CELLMARKERMSIGDB_DESCARTES_MAIN_FETAL_NEU…	PIP4K2C	ASCL1	LINC00…	SOX21-AS1	GAST	LINC02…	KCNH6	NPTX1	SEC11C	VMAC	ACSBG2	LINC017…	GPR42	FFAR2	CCNP	KLK12	KLK14	LINC002…	TFF3	USP41	HTR2C				
CELLMARKERMSIGDB_DESCARTES_MAIN_FETAL_OLI…	FYN	ENSG00…	LHFPL3	KCND2	RNU6-5…	CSMD1	PMP2	MMP16	ADGRB1	SHC3	MIR219A…	PCDH15	ENSG00…	ZNF365	GRID1	SOX6	LRRC4C	RNU6ATA…	OPCML	OPCML-I…	LINC02743	DBX2-A…	PCDH9-A…	NOVA1	NOVA1-…
CELLMARKERMSIGDB_DESCARTES_MAIN_FETAL_PDE…	MEGF10	PCDHA10	RN7SL6…	NSG2	AACSP1	RNU6-8…	POU6F2	C7orf25	PCLO	SEMA3A	SLC26A5…	RELN	FEZF1-…	LINC01…	KCNB2	ANKRD20A…	FAM88B	ANKRD20…	LINC01…	DIRAS2	CSGALNAC…	ERCC6	CHAT	HSPD1P…	RNU7-1…
CELLMARKERMSIGDB_DESCARTES_MAIN_FETAL_PDE…	BTG2-DT	HHAT	RPS6KC1	HHIPL2	TAF1A	AIDA	CNIH3	SDE2	STUM	PSEN2	RPL36P6	SNORA80B	DNAJC27	ZNF512	LINC01460	HNRNPLL	CHAC2	LINC018…	GCFC2	SH2D6	ANKRD33B…	CNNM3	APPAT	SULT1C4	TFCP2L1
CELLMARKERMSIGDB_DESCARTES_MAIN_FETAL_RET…	OSTM1-A…	NR2E1	NPVF	GRB10	POT1-A…	CLVS1	MYBL1	ZHX2	RNU2-47P	CNTLN	MOB3B	LINC015…	NOTCH1	RTKN2	SORCS1	LGR4	GLYATL2	FOXN4	RPS27P…	RFC3	TMTC4	NALCN-…	ABHD12B	DDHD1	C14orf…
CELLMARKERMSIGDB_DESCARTES_MAIN_FETAL_SAT…	MYMX	MICAL1	VGLL2	ZC2HC1B	PURB	RPS3AP…	MEST	MSC-AS1	LINC011…	MATN2	DMRT2	COL15A1	CSTF2T	SLF2	PITX3	PPP1R14B	HNRNPA…	CCND2	ITGA7	MYF5	LINC02397	GLT8D2	MIDEAS	SPG21	ADAMTS…
CELLMARKERMSIGDB_DESCARTES_MAIN_FETAL_SCH…	MRGPRF-…	GPR83	NANOGP1	TSPAN11	DHH	HSPD1P4	GAS2L3	C12orf…	LINC003…	STARD13	EDNRB	RHBDF1	CTF2P	ASPA	PMP22	CNP	NGFR	NGFR-AS1	ABCA8	MIA	CADM4	PRNP	KIAA1755	S100B	SOX10
CELLMARKERMSIGDB_DESCARTES_MAIN_FETAL_SLC…	GPR61	LRIG2	HSD3B2	LINC01719	ENTREP3	KIRREL…	DEDD	DUSP12	PBX1-AS1	C1orf220	LINC008…	LEMD1-A…	LINC00…	SNX25P1	GPATCH2	LYPLAL1-…	RPSAP21	SLC4A1AP	BABAM2	WDR43	AHSA2P	VPS54	MAT2A	PTCD3	ENSG00…
CELLMARKERMSIGDB_DESCARTES_MAIN_FETAL_SMO…	ABCC9	NDUFA4…	AVPR1A	RASL12	CSPG4	MYH11	CCR10	CNTNAP1	AOC3	GJC1	HIGD1B	SEPTIN4	TBX2	CYGB	ADGRA3P1	CCDC102B	NOTCH3	MEF2B	TMEM74B	COX4I2	APOL5				
CELLMARKERMSIGDB_DESCARTES_MAIN_FETAL_STE…	NEURL1B	ADAMTS2	ANKS1A	DAAM2	LINC02…	IGFBP3	ENSG000…	HGF	FBXO24	LAMB1	DENND2A	ARHGEF3…	OR2A1-…	HMBOX1	COLEC10	CER1	ADAMTS…	ADAMTS13	CCDC3	GRK5	IFITM10	INS-IG…	SLC17A8	NR1H4	LINC02…
CELLMARKERMSIGDB_DESCARTES_MAIN_FETAL_SYM…	DBH	CCDC172	PRLHR	ADGRA1	TH	NPAS4	C12orf29	KSR2	VPS33A	MICU2	FGF14-I…	FGF14-A…	GCH1	CHGA	NSMCE3	HACD3	NLRP1	PNMT	C1QL1	LINC019…	CYB561	PRCD	RPSAP57	GALR1	KLK4
CELLMARKERMSIGDB_DESCARTES_MAIN_FETAL_UNI…	KIRREL3	RPH3A	CCDC175	ENSG0000…	MPP3	ADCYAP1	DPY19L3	MORC2-…	CNKSR2	TRPC5															
CELLMARKERMSIGDB_DESCARTES_MAIN_FETAL_URE…	LINC029…	ELF5	MYEOV	UPK2	AQP2	ENSG00…	DHRS2	SCNN1G	SCNN1B	WNT9B	HOXB7	SEPTIN9…	SLC14A2	LOC112…	UPK1A	PVALB	UPK3A	DCAF12L1							
CELLMARKERMSIGDB_DESCARTES_MAIN_FETAL_VAS…	ABCB1	CAV1	SOX7	TCIM	FCN2	JCAD	NODAL	OIT3	APLNR	ROBO4	SLCO1A2	PTPRB	C1QTNF9	DLL4	RFLNB	ARHGEF15	CD300LG	DGKE	ODF3L2	RASIP1	TCF15	PRND	CYYR1	RFPL1	SHANK3
CELLMARKERMSIGDB_DESCARTES_MAIN_FETAL_VIS…	HSPD1P1	FST	ZBED8	TUBB2A	TOB2P1	TUBB	TMEM151B	BEND6	FBXO30	CDK13-DT	EEF1DP4	YWHAG	KBTBD1…	FAM167A	NEIL2	STMN2	ENHO	ASTN2-A…	NCS1	LINC026…	DNAJC9-A…	ELOVL3	LINC015…	NAV2-A…	ALKBH3…
CELLMARKERMSIGDB_DURANTE_ADULT_OLFACTORY_…	CD74	HLA-DMB	CORO1A	POU2F2	CD53	GPR183	LIMD2	TAGAP	NFKBID	BIRC3	REL														
CELLMARKERMSIGDB_DURANTE_ADULT_OLFACTORY_…	LCN2	GPX3	BPIFA1	PI3	BPIFB4	VMO1	LCN15	SCGB3A1	GSTA1																
CELLMARKERMSIGDB_DURANTE_ADULT_OLFACTORY_…	ARHGDIB	GMFG	STK4	TSC22D3	TNFAIP3	RORA																			
CELLMARKERMSIGDB_DURANTE_ADULT_OLFACTORY_…	CLEC2D	TRGC2	SAMSN1	CD8B	CST7	DUSP2	FYB1	CD48	ACAP1	LCK	ARHGDIB	LINC018…	TSC22D3	ZFP36L2	TAGAP	EVL	GZMM	STK4	CYTIP	CXCR6	CTSW	STK17A	LEPROTL1	HOPX	NKG7
CELLMARKERMSIGDB_DURANTE_ADULT_OLFACTORY_…	COL1A1	CLDN11	CALD1	BGN	FGF7	C1R	CCDC3	CCN1	TMEM100	MFAP4	GLIPR1	ITGBL1	CCL11	SELENOP	IGFBP4	PGRMC1	TWIST1	TAGLN	PLPP3	PRRX1	ECM1	COL6A2	LAPTM4A	IFITM3	TGM2
CELLMARKERMSIGDB_DURANTE_ADULT_OLFACTORY_…	RTN1	SOX4	TUBB	RBP1	TUBA1A	COTL1	PSIP1	H2AZ1	MAP1B	H4C3	HMGN2														
CELLMARKERMSIGDB_DURANTE_ADULT_OLFACTORY_…	MS4A7	GPR183	APOC1	HLA-DMB	SRGN	FGL2	LST1	MNDA	CD14	CST3	PHACTR1	PLAUR	RNASE6	NPC2	FTL	RGS10	LAPTM5	CD68	PLD4	GPR34	IGSF6	COTL1	RNASET2	HLA-DQ…	SPI1
CELLMARKERMSIGDB_DURANTE_ADULT_OLFACTORY_…	SRGN	GATA2	HPGD	FTH1	NFKBIA	CD37	CAPG	CD83	MAOB	PPP1R15A	RAC2	SELENOK	PTGS2	CKLF	RGS2	RAB27B	CSF1	PLAUR	LMO4	NR4A2	EMP3	BIRC3	LAPTM4A	ACSL4	STX3
CELLMARKERMSIGDB_DURANTE_ADULT_OLFACTORY_…	HLA-DPB1	HLA-DP…	HLA-DR…	HLA-DRB1	SFRP2	CCL4L2	SFRP1	HLA-DMA	RGS2	HLA-DRA	CD74	SGK1	SRGN	REL	HERPUD1										
CELLMARKERMSIGDB_DURANTE_ADULT_OLFACTORY_…	XCL2	FCER1G	PLEK	TRBC1	PYHIN1	MATK	CD160	DOK2	IL2RB	BIN2	CORO1A	RAC2	SPON2	HOPX	SPN	PTPRC	TBC1D1…	MYO1F	GPR65	CCL3	B2M	LCP1	CD69	HLA-C	TMSB4X
CELLMARKERMSIGDB_DURANTE_ADULT_OLFACTORY_…	SEMA3C	GNG13	BIK	BMX	GOLM1	CLDN3	FAM3B	ENC1	ATP6V0B	CD24	ATP6V1A	SC5D	RASSF6	ANXA4	IRAG2	AKR1B1	APLP2	KRT18	KRT8	TPD52	CD9	C15orf…	SMS	AVIL	KRT7
CELLMARKERMSIGDB_DURANTE_ADULT_OLFACTORY_…	VIM	KLF2	SOX18	TM4SF18	SLC9A3…	CAVIN2	APOLD1	ENG	RAMP3	EDN1	SOCS3	CCDC85B	HLA-E	PCAT19	ID3	LMCD1	GIMAP7	CLU	PALMD	PODXL	TSPAN7	CYYR1	TGFBR2	FLT1	ABCG2
CELLMARKERMSIGDB_DURANTE_ADULT_OLFACTORY_…	ANXA2	SERPIN…	PHLDA2	ATP1B3	ANXA1	S100A11	PRDX1	ELF3	CALML3																
CELLMARKERMSIGDB_DURANTE_ADULT_OLFACTORY_…	PRSS23	CXCL8	CLDN10	CYB5A	TMEM213	TSPAN1	KRT19	ELF3	S100A14	MUC1	MT3	VSIG2	FAM3D	LY6D	PIGR	NOS2	HPGD	CLDN4	AQP5	POR	MGST1	SCGB1A1	METTL7A		
CELLMARKERMSIGDB_DURANTE_ADULT_OLFACTORY_…	DYNLL1	ATP5IF1	NTS	CALM1	H2AZ1	S100A2	TUBA1B	TUBB	PPA1	CIB1	TUBA1A	CD24	H4C3												
CELLMARKERMSIGDB_DURANTE_ADULT_OLFACTORY_…	TINAGL1	MCAM	PDLIM3	RHOB	PPP1R1…	ADIRF	HIGD1B	NR2F2	FLNA	EGR1	KLF2	SOD3	PLN	NOTCH3	CSRP1	ACTG2	CRISPL…	ECRG4	DEPP1	ARID5B	SPARC	ATF3	LHFPL6	MAP1B	ID4
CELLMARKERMSIGDB_FAN_EMBRYONIC_CTX_ASTROC…	MLC1	DIO2	AGT	ADCYAP1R1	PLAGL1	RAMP3	SFXN5	GPX3	CXCR4	GFAP	LRP4	PAQR8	FOXG1	BTBD17	CBS	CXCL14	LYN	GABRB1	NOG	MMP28	ADORA2B	GALNT16	SIRPA	CHRDL1	TSKU
CELLMARKERMSIGDB_FAN_EMBRYONIC_CTX_BIG_GR…	KCNJ8	SPARC	MFSD2A	HIGD1B	LAMC3	IFI27	ARHGAP29	SRARP	STRA6	ITGA1	GJA4	CAVIN3	SLC38A5	TM4SF18	PLXDC1	GNG11	CLEC3B	TIE1	IFITM3	FLT1	FRZB	NID1	COL3A1	TMEM204	VWF
CELLMARKERMSIGDB_FAN_EMBRYONIC_CTX_BIG_GR…	PGF	LHX5-A…	CALB2	NHLH2	CABP7	GADD45A	LYNX1	CYGB	AIM2	EMX2OS	EBF3	LINC008…	ADAM12	SCGB1D2	LHX1	OLFM1	FCER1A	CNTNAP2	FSTL5	RASSF6	TUBA4A	NDRG4	PCOLCE2	SPINT2	SV2B
CELLMARKERMSIGDB_FAN_EMBRYONIC_CTX_BIG_GR…	PLXNA4	CACNG8	RBFOX1	PRRT2	MLLT3	ADCY1	EPHB6	LHX2	PLXNA2	ICA1L	PPP2R2B	ACTN2	R3HDM1	GOLGA8B	GRIA2	SCN2A	FBXW7	DYNC1I1	LINC00…	PANTR1	MAP6	SRCIN1	SYT5		
CELLMARKERMSIGDB_FAN_EMBRYONIC_CTX_BIG_GR…	ADAM28	LPAR5	IGSF6	FCGR1BP	FCGR1CP	LINC02…	HPGDS	SIGLEC…	SAMSN1	APBB1IP	MS4A7	CD68	TBXAS1	LAPTM5	ACY3	SFMBT2	ADORA3	PLD4	MRC1	MROCKI	LTC4S	CCR1	MERTK	LY86	RHBDF2
CELLMARKERMSIGDB_FAN_EMBRYONIC_CTX_BRAIN_…	ENG	CD320	VWF	ABCB1	TFRC	PON2	SLC3A2	SLC7A1	SLC38A3	FN1	SLC16A1	TIE1	CSRP2	SLC9A3…	SPARC	EPAS1	ECSCR	CLEC3B	DIPK2B	ID3	S100A16	CRIP2	CLEC14A	MMP28	ABLIM1
CELLMARKERMSIGDB_FAN_EMBRYONIC_CTX_BRAIN_…	EDNRA	LHFPL6	COL6A1	IGFBP7	FRZB	MCAM	CPE	AGRN	DLC1	FOXS1	TPM2	SEMA5A	NR2F2	FSTL1	HIGD1B	PLOD1	ZEB1	UACA	SPARCL1	LAMA4	DCN	MYO1B	COL3A1	PCDH18	ITGA1
CELLMARKERMSIGDB_FAN_EMBRYONIC_CTX_EX_1_E…																									
CELLMARKERMSIGDB_FAN_EMBRYONIC_CTX_IN_2_I…																									
CELLMARKERMSIGDB_FAN_EMBRYONIC_CTX_IN_3_I…																									
CELLMARKERMSIGDB_FAN_EMBRYONIC_CTX_IN_5_I…																									
CELLMARKERMSIGDB_FAN_EMBRYONIC_CTX_IN_6_I…																									
CELLMARKERMSIGDB_FAN_EMBRYONIC_CTX_MICROG…																									
CELLMARKERMSIGDB_FAN_EMBRYONIC_CTX_OLIG	ZDHHC9	SECISB…	GSN	PEX5L	SIRT2	NFASC	CARNS1	PLPP2	MAL	MYRF	TMEM125	CMTM5	PLEKHH1	SLAIN1	LGI3	BIN1	SOX2-OT	FEZ1	GPR37	MAP7	UGT8	ELOVL1	PLEKHA1	SLC24A2	PTK2
CELLMARKERMSIGDB_FAN_EMBRYONIC_CTX_OPC	SLC35F1	SEMA5A	SNAP25	KLRC2	PCSK1N	IL1RAP	SCN1A	SPRY4	SCG3	MPZL1	PDE4B	BRINP1	VXN	TAOK3	OLIG1	NXPH1	ARL2BP	TSPAN13	UCHL1	SLC1A1	ATCAY	C2orf80	CXADR	LRRK2	SNTG1

Table S5: Cell state marker gene list

CELLMARKERCAN_ACINAR_CELLS	PNLIPRP1	CTRC	REG3A	SERPINA3	PRSS3	REG1B	CFB	GDF15	MUC1	C15ORF48	DUOXA2	AKR1C3	OLFM4	GSTA1	LGALS2	PDZK1IP1	RARRES2	CXCL17	UBD	GSTA2	ANPEP	LYZ	ANGPTL4	ALDOB	
CELLMARKERMSIGDB_FAN_OVARY_CL1_GPRC5A_TNF…	CFH	SPSB1	STK17A	VIM	SERPIN…	CD63	MORF4L2	SERPIN…	AKAP12	ATF4	HNRNPH3	HSP90AA1	MAP1LC…	COL6A2	RAB7A	FILIP1L	NCL	HSPA8	MEG3	EIF4E	EIF1B	HNRNPA…	AMD1	MLF1	SGK1
CELLMARKERMSIGDB_FAN_OVARY_CL11_MURAL_GRA…	PLAAT3	UBE2D1	SERPIN…	HSD17B1	GSTA4	PMAIP1	SNAPC1	MARCKS	CHORDC1	DNAJB1	IRS1	LIPH	RBP1	SPINT2	PLAT	LAPTM4B	HSP90A…	MYO10	FSCN1	GNAI1	SFRP4	NUP214	PDLIM5	TSPAN6	ZFAND2A
CELLMARKERMSIGDB_FAN_OVARY_CL13_MONOCYTE_…	FABP5	SH3BGR…	TYMP	SDS	FCN1	CD68	CYBA	MS4A6A	LAPTM5	S100A11	SAT1	FTL	CXCL2	S100A9	SPI1	BCL2A1	FTH1	CSTB	IFI30	C1QA	G0S2	SERPIN…	COTL1	CD52	FCER1A
CELLMARKERMSIGDB_FAN_OVARY_CL14_MATURE_SM…	RGS16	CAVIN3	IGFBP7	NET1	MAP3K7…	IER2	ISYNA1	TPM1	PTN	CRIP2	S100A4	PTP4A3	TPPP3	DEPP1	SPARCL1	PHLDA2	HIGD1B	CAV1	PGF	CCDC3	CSRP1	MEF2C	LGI4	ZFP36	JUN
CELLMARKERMSIGDB_FAN_OVARY_CL15_SMALL_ANT…	MCL1	PMAIP1	HNRNPA…	MGARP	AMH	SRPX	POMP	HIF1A	MYL12B	EIF5	HES1	ARL4D	DNAJA1	JMJD6	HSPH1	AKIRIN1	DNAJB6	KLF10	EMX2	AMOTL2	DUSP14	DSP	MRPL18	LEPROT…	BEX1
CELLMARKERMSIGDB_FAN_OVARY_CL18_B_LYMPHOC…	HLA-DPB1	HLA-DP…	IRF7	CREM	C12orf…	CTSZ	BID	RNASET2	NR3C1	CD37	TSPAN13	SEC61B	TYROBP	HSPA1A	ITM2C	GNA15	SERPIN…	NOPCHAP1	HLA-DMA	PLAC8	STK4	LDLRAD4	HERPUD1	HLA-DR…	SRGN
CELLMARKERMSIGDB_FAN_OVARY_CL2_PUTATIVE_E…	HMGN3	GSTM3	COX6C	COL1A1	CYB5A	ZFP36L1	C7	S100A6	ITM2B	CEBPD	BST2	C1R	SELENOM	HES1	ZFP36	FOSB	RPL31	HMGN2	C12orf…	NBL1	SEPTIN7	H3-3B	RPL36	RPL12	COL6A2
CELLMARKERMSIGDB_FAN_OVARY_CL5_HEALTHY_SE…	LANCL2	CLEC11A	IRF8	FRZB	FSTL1	SERPIN…	ZFAND2A	DNAJA1	DNAJB4	PTCH1	SPON2	CACYBP	SNHG25	NSG1	COL5A1	F3	VCAM1	IFITM3	SNAI2	IFITM1	HTRA3	NID2	MCL1	CDH11	SUSD3
CELLMARKERMSIGDB_FAN_OVARY_CL6_PUTATIVE_E…	CLDN11	HSPA6	COL6A2	TAGLN	MYLK	FOSB	IGF1	OLFML3	DNAJB1	APOE	PMP22	TCEAL4	LHFPL6	SERPIN…	ACTA2	DUSP1	GADD45B	IGFBP5	ALDH1A1	MFAP4	CALD1	SPON2	COL6A1	AEBP1	TPM1
CELLMARKERMSIGDB_FAN_OVARY_CL8_MATURE_CUM…	MAGED2	FST	STMN1	INHA	BEX3	PRSS23	REEP6	MIR202…	CCND2	TMSB10	SRPX	AVPI1	TCEA3	TSPAN6	PLAAT3	HNRNPA1L2	IDH1	NDFIP1	LMBR1L	UQCRQ	DMAC1	MT1X	CHGB	GSTA4	TRAPPC4
CELLMARKERMSIGDB_FAN_OVARY_CL9_PUTATIVE_A…	SDCBP	CAV1	SELE	SQSTM1	PLIN2	MGP	ARHGAP29	SOCS2	CLIC2	PIM3	CTHRC1	SPARCL1	ANXA1	CLEC14A	GIMAP6	STOM	IFI16	CLU	IFIT1	PLEKHO1	HMGA1	ADGRL4	MX1	IFIT3	HHEX
CELLMARKERMSIGDB_GAO_ESOPHAGUS_25W_C1_CIL…	ARHGAP18	ARHGAP…	ARHGEF…	ARHGEF38	ARL13B	ARMC2	ARMC3	ARRDC1…	ASIC1	ASTN2	ATAT1	ATP2C2	ATP6V0…	ATP6V1…	ATXN2	B9D1	BAIAP3	BASP1	BBS1	BBS5	BCAS3	GPRASP3	BPIFB1	BTC	CABCOC…
CELLMARKERMSIGDB_GAO_ESOPHAGUS_25W_C2_KRT…	GK	GPR160	GPRC5A	GPT2	HDAC9	HOPX	HTRA2	IGF2BP…	ISG15	KRT78	KYNU	LINC015…	LIPH	MACC1	MANSC1	MBD6	MCPH1	MID2	MNX1	MUC21	MUC3A	NCCRP1	NTHL1	NTN4	OAS1
CELLMARKERMSIGDB_GAO_ESOPHAGUS_25W_C3_FGF…	L3MBTL3	LAPTM5	GATD1-…	LPXN	LTV1	MBNL3	MCUR1	MFSD3	NDRG4	NUP160	NUP35	PEX11A	PHTF2	PIP4K2A	PPP1R9B	PWWP2A	RALY-A…	RBSN	RNF166	RPL39L	SH3KBP1	SLC2A3	SPATC1L	TCF7	TET1
CELLMARKERMSIGDB_GAO_LARGE_INTESTINE_24W_…	COL5A2	COL6A6	COL8A1	COLEC12	CPXM1	CPZ	CSF1	CTHRC1	CYBRD1	DCN	DPT	HYCC1	FBLN1	FNDC1	FZD1	GBP1	GBP1P1	GFRA2	GPC6	GSTM5	HAPLN3	HTRA1	ICAM1	IFI6	IFIT2
CELLMARKERMSIGDB_GAO_LARGE_INTESTINE_24W_…	LRRC57	LYPD8	MALL	MAMDC4	MCF2L2	MLXIPL	MOGAT2	MSLN	MUC20	MYO1A	NBEAL1	NOXA1	NR1H4	NR1I2	PARP12	PLS1	PRAP1	PRSS3	RARRES1	RNPC3	SI	SLC13A1	SLC15A1	SLC35D2	SLC41A2
CELLMARKERMSIGDB_GAO_LARGE_INTESTINE_24W_…	C1QC	C2	C5AR1	CASP1	CCDC134	CCL3	CCL4	CCR1	CD14	CD163	CD163L1	CD180	CD209	CD300A	CD300E	CD33	CD37	CD4	CD40	CD48	CD53	CD68	CD74	CD83	CD84
CELLMARKERMSIGDB_GAO_LARGE_INTESTINE_24W_…																									
CELLMARKERMSIGDB_GAO_LARGE_INTESTINE_24W_…	GOLT1A	GPX2	HNF1A	HSD17B2	ITPKC	LEFTY1	LGR5	LHFPL3…	LIPG	LY6G6D	MGST1	MYOM3	NOX1	OLFM4	PCSK9	PDZD3	PKP2	PLAGL2	PLEKHH1	PPP1R1B	PTGDR	RGMB	RNF157	RNF186	SFN
CELLMARKERMSIGDB_GAO_LARGE_INTESTINE_24W_…	PLCD1	PLEKHA6	PTPRN2	RASEF	SBNO1	SCNN1A	SLC17A5	SLC37A1	SORL1	SPDEF	STXBP5-…	SYT7	SYTL2	TSPAN1	UGT2B7	ZG16	ZMYND8								
CELLMARKERMSIGDB_GAO_LARGE_INTESTINE_ADUL…	BCAS4	BEX1	BMPER	BMPR1B	BNIP3	ABTB3	CFAP300	BMERB1	C19orf44	C1orf127	C2CD4B	C3orf14	C7	CACNA1A	CACNA1C-…	CACNA1D	CACNA2…	CADPS	CALML3…	CAMK2B	CAMK2N2	DNAI7	CASR	CBLN1	CBLN2
CELLMARKERMSIGDB_GAO_LARGE_INTESTINE_ADUL…	ATP1A2	ATP6V0…	AUNIP	B4GALNT4	B4GALT6	BAG2	BARX2	BCAM	BCL6	BEGAIN	BLM	BNC2	C11orf…	C14orf…	C16orf95	C17orf97	CATSPE…	C1orf226	C2CD4C	C2orf74	C5AR1	C7	CACNA1C	CACNA1I	CACTIN
CELLMARKERMSIGDB_GAO_LARGE_INTESTINE_ADUL…	LINC020…	IGFBP5	IL10	KCNMB4	KDR	KLRD1	LEMD1-A…	LINC01…	LINC014…	ZNF606-…	LINC017…	LINC024…	LRRN1	MCMDC2	MNDA	MPDZ	MUC19	NOS1	PTGIS	PXDC1	RNF212	RYR2	SLC36A2	SMIM17	SPACA6…
CELLMARKERMSIGDB_GAO_LARGE_INTESTINE_ADUL…	ARHGAP4	ARHGAP6	ARHGAP9	ARHGDIB	ASB2	ATP8B2	BASP1	BLK	BLVRA	BMF	BNIP3	BRSK1	BST2	BTK	BTN2A2	C16orf54	C16orf…	RHEX	C1QC	CABLES1	CAV1	CCDC10…	CCDC69	CCDC88A	CCR10
CELLMARKERMSIGDB_GAO_LARGE_INTESTINE_ADUL…	AMOTL1	ANGPT1	ANGPTL2	ANKRD20A…	ANKRD29	ANTXR1	ANXA1	ANXA6	AOC3	APLNR	APOD	APOE	APOL4	ARHGAP…	ARHGAP6	ARHGEF15	ARHGEF…	ARL10	ARMCX1	ASAP1	AXL	BCL6B	BGN	BMP4	BMPER
CELLMARKERMSIGDB_GAO_SMALL_INTESTINE_24W_…	KIF26B	MATN1-…	MGST1	NR5A2	PCYOX1L	PEX14	PIGH	PLEK2	POF1B	PRR15	PRR15L	RNASE6	SCARNA2	SLC2A1	SLC35A4	SLC35C2	SLC51B	ST20	STARD1…	TATDN1	TSPAN8	TTR	VPS26B	ZNF586	
CELLMARKERMSIGDB_GAO_SMALL_INTESTINE_24W_…	DAB1	DDC	DOK7	DPEP1	DPP4	EMB	ENPP7	ETNK2	FABP2	FAH	FAM110C	FBP1	FBXO2	G0S2	GCHFR	GJB1	GLRX	GOLT1A	HHLA2	IFI30	IL22RA1	IL32	KEL	MYORG	MAMDC4
CELLMARKERMSIGDB_GAO_SMALL_INTESTINE_24W_…	MCM3AP-…	MSANTD2	MT1M	MUC2	MYO1F	NEURL1	PAX3	PLEKHG6	REG4	REP15	RNASE1	SPDEF	SPINK4	SRP72	ST6GALNA…	STARD10	STXBP5…	SYP	TFF1	TSPYL5	VAMP4	ZBTB24			
CELLMARKERMSIGDB_GAO_SMALL_INTESTINE_24W_…	HCN1	HTR1D	KCNQ2	KCNT2	CIP2A	KIF1A	LAMA1	LHX9	LINC002…	LINC003…	LINC006…	LINC012…	ENSG00…	MX1	OGFRL1	OR4N3P	POU2AF1	PTPN13	RASAL2…	SRPX2	SV2B	SYT16	TBC1D22…	TCEANC2	TMEM201
CELLMARKERMSIGDB_GAO_SMALL_INTESTINE_24W_…	KCNAB1-…	LINC00…	LINC00…	LINC01354	ENSG00…	PCDH10…	LOX	LSAMP-…	MADCAM1	MEI4	METTL1	MSC	NANOS1	NTF3	P4HA2-AS1	PCDH8	PLSCR5	POMGNT2	PPP1R18	PRDM14	RASA1	RFX3-DT	RTL5	RHBDD1	RPL23A…
CELLMARKERMSIGDB_GAO_STOMACH_24W_C1_PROCR…	POMT2	PPIH	PWWP2B	RPE	SCARF2	SGK1	SLC25A38	SLX4IP	SNX7	TRAF4	UBE2S	ZFAND1	ZFP41	ZNF143	ZNF354C	ZNF571									
CELLMARKERMSIGDB_GAO_STOMACH_24W_C2_TFF2P…																									
CELLMARKERMSIGDB_GAO_STOMACH_24W_C4_PARIE…																									
CELLMARKERMSIGDB_GAO_STOMACH_24W_C5_PUTAT…	LIMS2	LRP5	LRRFIP2	MICALL1	MVB12B	NFIX	NPB	NRXN3	OLR1	P2RX4	PIPOX	PPP1R14A	RASSF3	RTL5	RPRD1B	RSPO3	SBSPON	SETD1B	SGCA	SLC24A3	SLC2A4	SMG8	SRF	TIMM17A	TMEM47
CELLMARKERMSIGDB_GAUTAM_EYE_IRIS_CILIARY_…	ARHGAP4	FAM171B	HCG18	CPSF1	BCAT2	STEEP1	TRMT10C	BZW2	NDST1	TINF2	STK3	RPL10	GARS1	NDUFB3	MLLT6	PLCB1	UNC119	CRTC2	CISH	CSRNP3	OSTF1	WTIP	GNPAT	UBTD1	PPP2R3C
CELLMARKERMSIGDB_GAUTAM_EYE_IRIS_CILIARY_…	CTNNAL1	MAFK	TXNDC12	PBX1	ERICH5	CREG1	INHBB	DNAJC6	RPL21	NCAM1	ENY2	DIPK2A	SPOCK1	PMEPA1	MAST4	TGM2	CADM3	HSP90AB1	BCL2L13	EGR2	ZCRB1	COL4A6	EFNA5	GCSH	RPL6
CELLMARKERMSIGDB_GAUTAM_EYE_IRIS_CILIARY_…	BAIAP3	MITD1	SLC17A9	STK38	CIAO2A	MOB4	SYNGR1	SLC2A6	NPAT	UBE2E2	ANKZF1	TMEM19	ZNF138	GCA	LRBA	SNX10	LAX1	GATA2	WASHC2A	HMGB1	TBRG1	CDC40	EBP	MAP4K2	ADA2
CELLMARKERMSIGDB_GAUTAM_EYE_IRIS_CILIARY_…	PTMA	PPIB	SPTBN1	UGP2	PFKFB3	SELENOM	IFNAR1	CTTNBP2	SAT1	REV3L	RBMS3	PLXDC1	PNRC1	RHOBTB3	CCDC71L	OTULINL	CBLB	LTBP4	C7	S100A11	TGFBI	SEC61B	LARP4	FMO3	AKR1C2
CELLMARKERMSIGDB_GAUTAM_EYE_IRIS_CILIARY_…	GOLT1B	SFSWAP	KAT2B	CCDC12	UBE2D1	PPP1R1…	TCEAL1	VSIR	WASL	ARHGEF6	N4BP2L2	DCTN2	MAP2	GAB1	AJAP1	CDC42EP5	JOSD2	CFL2	ITPR3	EPN2	KLF15	CREB1	RRAD	ANKZF1	SYT11
CELLMARKERMSIGDB_HAY_BONE_MARROW_CD34_POS…																									
CELLMARKERMSIGDB_HAY_BONE_MARROW_CD34_POS…																									
CELLMARKERMSIGDB_HAY_BONE_MARROW_CD34_POS…																									
CELLMARKERMSIGDB_HAY_BONE_MARROW_CD34_POS…	ADRA2A	SLC18A2	CDH6	RBPMS2	ACTN1	ADCY6	ITGA2	DTD1	TRPC6	LTC4S	PTGER3	RAB38	EFNB2	STUM	RAC3	TPI1	OLFM3	TEAD4	RAB3C	HTR2A	MGLL	RYR2	PPIF	DKK1	TTC27
CELLMARKERMSIGDB_HAY_BONE_MARROW_CD8_T_CE…	CLEC2B	SYNE2	ZNF683	F2R	STK17A	A2M-AS1	MIAT	GUK1	TENT5C	RASAL3	SRSF7	IFNG	TMA7	ATG2A	CRTAM	UBC	PTPN22	MSC	TPRG1	PPDPF	TNFRSF9	TTC16	PARP8	LINC00…	RNF19A
CELLMARKERMSIGDB_HAY_BONE_MARROW_EARLY_ER…	MYB	AHCY	UQCRH	NPM3	PEBP1	SORD	PMP22	CASP3	GNL3	GSTM3	TUFM	MRPS25	PRMT1	MORF4L2	CASP6	PPA1	CHCHD2	COL6A5	TMEM97	CMSS1	NHP2	AK2	KCNK5	MRPL52	SLC27A2
CELLMARKERMSIGDB_HAY_BONE_MARROW_EOSINOPH…	KIF22	H1-4	MRPL33	KIAA0930	MZT2B	SERPIN…	TPM3	HNRNPD	ANKRD13D	SUB1	ASRGL1	NDUFS6	PRLR	ALYREF	MRPL23	NDUFB11	CEBPA	RECK	IQGAP3	PHPT1	HSPA5	RPN1	NDUFA3	MT-CYB	WDFY2
CELLMARKERMSIGDB_HAY_BONE_MARROW_NAIVE_T_…	TRAC	RPL9	LEPROT…	RPS6	RPS16	CD3D	RPS28	RPL19	RPS20	RPL3	PIK3IP1	RPL27A	RPL41	NOSIP	RPS3	RPL38	JUNB	RPS13	RPS10	MAL	RPL18	RPL27	RPL36	RPS18	NOP53
CELLMARKERMSIGDB_HAY_BONE_MARROW_NEUTROPH…	MS4A6A	CDA	APLP2	GRN	MCL1	METTL9	TNFSF13B	MGST1	CYBB	PLAUR	MPEG1	STXBP2	GAPDH	SH3BGR…	CSF3R	TREM1	OAZ1	KCTD12	RAB31	C4orf48	MT-CO1	CFP	CD302	FPR1	TKT
CELLMARKERMSIGDB_HAY_BONE_MARROW_NK_CELLS	B2M	APMAP	ITGB2	KLRC1	TTC38	SH2D1B	HLA-A	CD160	PFN1	IFITM1	NCR3	HCST	CHST12	ADGRG1	FCRL6	PRSS23	C1orf21	XCL2	MYO1F	JAK1	ZAP70	RAC2	HLA-E	SAMD3	PLAAT4
CELLMARKERMSIGDB_HAY_BONE_MARROW_PRE_DEND…																									
CELLMARKERMSIGDB_HAY_BONE_MARROW_PRO_B	H2AZ2	PIMREG	NUCKS1	ASF1B	VDAC1	CENPF	IGLC5	ZWINT	HMGB3	MAD2L1	TMSB15A	HNRNPA2…	CRNDE	H2AX	USP1	SPC25	CMTM8	DEK	RAD51A…	HMGN1	UBE2C	CD72	CDCA8	SLC25A5	E2F2
CELLMARKERMSIGDB_HAY_BONE_MARROW_STROMAL	FSTL1	NTRK2	CHL1	CALD1	COL1A2	LPL	GAS6	FRZB	FMO3	FBLN1	ALDH1A3	CCDC80	RBP1	EPAS1	BGN	TNC	DDR2	LTBP2	EBF3	PAPPA	IGFBP2	FMO2	PLAC9	ENPEP	ECM2
CELLMARKERMSIGDB_HE_LIM_SUN_FETAL_LUNG_C0…	RGMA	KCNMB1	PDLIM3	FBN2	ALDH1B1	FBXL22	HHIP	GBP2	WIF1	GREM2	TUBB2B	PRKAR2B	CDH3	SLC25A4	RBM38	SMOC2	TSPAN2	NET1	RALGPS2	HACD1	FENDRR	SPEG	FLNB	COL23A1	ASB2
CELLMARKERMSIGDB_HE_LIM_SUN_FETAL_LUNG_C0…	HTRA3	FZD4	LINC01…	PRRX1	ADAMTS…	C1QTNF2	FIBIN	MFAP5	DPYSL3	ASPN	DLK1	ITM2A	WNT2B	FLRT2	TRIO	ADAMTSL3	SCARA5	RGS10	CPZ	GFRA1	EMP1	RASSF2	CFH	OMD	PDIA5
CELLMARKERMSIGDB_HE_LIM_SUN_FETAL_LUNG_C0…	CENPU	MSMO1	PSAT1	PTTG1	CENPF	KPNA2	BAZ1A	RMI2	CCNB2	BIRC5	ZWINT	H1-3	CDT1	UHRF1	UBE2T	AIF1	KRT18	MCM4	CENPK	UCK2	CENPM	H1-2	H1-1	H2BC9	CLSPN
CELLMARKERMSIGDB_HE_LIM_SUN_FETAL_LUNG_C0…	CXADR	JUP	COL18A1	SVIL	PDPN	TMEM37	EFEMP1	OCIAD2	SH3BP5	TUBB2B	ARL4A	SULF1	SEMA3B	LAMA5	TSTD1	EPS8L1	COL2A1	HHIP	CFC1	NPDC1	APLP1	COL26A1	LEPROTL1	MEIS2	CFC1B
CELLMARKERMSIGDB_HE_LIM_SUN_FETAL_LUNG_C0…	FOXP2	TENM3	COL15A1	PSAT1	HHIP	AIF1L	TTYH1	BOC	SOX5	DHRS3	WFDC2	FGFRL1	SEMA6A	EPYC	CRYL1	DAAM2	MPPED2	RFLNA	CRISPL…	CDC42EP3	RGCC	SCARB1	LINC005…	NOTUM	PDGFC
CELLMARKERMSIGDB_HE_LIM_SUN_FETAL_LUNG_C0…																									
CELLMARKERMSIGDB_HE_LIM_SUN_FETAL_LUNG_C0…	CLDN15	PLLP	TMEM17…	SULF1	LINC01…	NPDC1	SEMA3B	VSIR	C1R	PDZK1IP1	COL18A1	SEMA3C	CYSTM1	ANXA8	GAS6	ALOX15	TNNT1	CRIP1	C19orf…	SPINT2	ANXA3	HTRA1	PRG4	WNT2B	CFB
CELLMARKERMSIGDB_HE_LIM_SUN_FETAL_LUNG_C0…	CRABP2	LINC02…	PRKAR2B	GPC4	EPS8	KPNA2	COLEC11	XIST	TWIST1	RARB	SH3BP5	LDB2	PAG1	RALGPS2	DPP10	CNTN4	TMTC2	SIPA1L2	NKX6-1	MIR99AHG	HNRNPA1L3	CD24	OAF	RHBDD2	RGS10
CELLMARKERMSIGDB_HE_LIM_SUN_FETAL_LUNG_C0…	ZEB1	CNN1	TYRP1	PLPPR4	PDGFC	FBXO32	ADAMTS6	HIPK2	KCNK3	BCL11A	LDLRAD4	IRAG1	SSC5D	WNT5A	ENTPD1-A…	ALKAL1	RASL11B	TRAF5	PCSK5	EYA4	LIMS2	DACH2	NGFR	MTARC1	PLXNA4
CELLMARKERMSIGDB_HE_LIM_SUN_FETAL_LUNG_C0…	NPDC1	RASSF7	DMKN	REC8	LINC01…	TMEM17…	PDPN	LGALS8	PLP2	CFC1	UCHL1	CXADR	ANXA3	CYSTM1	HHIP	CFH	ASS1	NET1	WT1	SH3BP5	CFI	PTPRF	CFC1B	GPM6A	TMEM17…
CELLMARKERMSIGDB_HE_LIM_SUN_FETAL_LUNG_C0…	IGF1	CT45A9	LEF1	DACH2	MYH11	ZEB1	CPED1	PAG1	ADAMTSL2	SYT1	MYOCD	ACTG2	PRAG1	WNT11	CT45A7	TMEM100	RHCG	RASL11B	CT45A6	MT1X	TAB2	PTGER2	CT45A5	MID1IP1	WNT5A
CELLMARKERMSIGDB_HE_LIM_SUN_FETAL_LUNG_C1…	RIN2	ITGB6	BANCR	PLLP	PHACTR1	CRYAB	IL18	SEMA5A	ABCA8	COL4A3	KRT4	FAM20A	COL8A1	TGFBI	NRP2	TUBB6	SLC39A8	HTRA1							
CELLMARKERMSIGDB_HE_LIM_SUN_FETAL_LUNG_C1…	AARD	BHLHE40	SLPI	ELAPOR1	NIBAN1	CAPS	SFTA1P	HS3ST1	MUC20	KRT7	PRSS12	EHF	GRHL1	S100P	TRIM22	SCGB3A1	CRTAC1	CTSW	SMIM22	SIX1	HLA-B	EPAS1	LIPH	TMC5	SPINK5
CELLMARKERMSIGDB_HE_LIM_SUN_FETAL_LUNG_C1…	GRHL1	NR2F2-…	SPTSSB	PHLDA1	ABCG1	SCD	GABRA1	LYPD6B	FSTL5	HSPA6	SRGAP1	H3-5	TOX3	CAMK1D	FRMD3	RP1									
CELLMARKERMSIGDB_HE_LIM_SUN_FETAL_LUNG_C1…	CENPH	TACC3	LDLR	PCDH17	TK1	MND1	TPM2	MKI67	RFC4	APOA1	INSIG1	MCM4	RAD51A…	TPX2	MCM6	GUCY1A2	VCAN	CENPK	ASF1B	IGDCC3	UQCRHL	DEPDC1B	RFC5	CDK1	CTXN1
CELLMARKERMSIGDB_HE_LIM_SUN_FETAL_LUNG_C1…	HES6	RIMBP2	ELAPOR1	RFX6	KCNH6	BTBD17	NEURL1	C21orf…	NCAM1	NKX2-2	NRXN1	PTPRN	ENO2	TTLL7	SEMA3A	RGS17	NAV1	ANK2	ACOT7	RAP1GAP2	GABRB3	SLC29A4	PTPRN2	ADGRG1	CA8
CELLMARKERMSIGDB_HE_LIM_SUN_FETAL_LUNG_C1…																									
CELLMARKERMSIGDB_HE_LIM_SUN_FETAL_LUNG_C1…	FGF20	SFTPA2	CEMIP	KCNJ5	ADORA1	FMN1	COL6A3																		
CELLMARKERMSIGDB_HE_LIM_SUN_FETAL_LUNG_C1…	CEACAM6	RASAL1	CHST9	USHBP1	DLL1	MFNG	CDC25B	TEX45	KIF19	SRGAP1	ACKR3	FSTL5	CRACDL	PHACTR3	TOX3	RNF186	IGFALS	ACTL10	CAMK1D	DRAIC	LFNG	IGSF1	FAM13C	CLDN10	PLLP
CELLMARKERMSIGDB_HE_LIM_SUN_FETAL_LUNG_C1…	FCGBP	CAPS	NXPH4	SLC15A2	NPNT	KRT4	DPYSL3	CXCL1	HS3ST1	ELAPOR1	RUNX1	SOD3	ARHGEF…	CFH	MUC4	STING1	TMEM15…	TNFSF10	CEACAM6	GLIPR2	CRYM	AGR3	CCNO	VTCN1	HCAR2
CELLMARKERMSIGDB_HE_LIM_SUN_FETAL_LUNG_C1…	CACNA1A	MEG3	KRT7	NRXN1	ANK2	CAMK1D	TUBB3	DNAJC12	LINC002…	QPCT	INSM1	CALCA	BIK	GCH1	SLC36A4	KIF1A	CRMP1	ARFGEF3	CHGB	SYP	SEZ6L2	CXXC4	TPBG	TMEM17…	TMEM17…
CELLMARKERMSIGDB_HE_LIM_SUN_FETAL_LUNG_C1…	NFE2L3	LGR6	TRIM29	PHLDA1	TP63	SERPIN…	SOX10	FGFR1	IL32	SYNPO2	EHF	FHL2	MIA	SOSTDC1	FRZB	PALLD	CAVIN1	SMOC2	IL27RA	SPARCL1	STXBP6	SEZ6L2	TNFAIP3	CLDN10	TGFA
CELLMARKERMSIGDB_HE_LIM_SUN_FETAL_LUNG_C1…	SIX1	DTX2	CLDN10	SLC15A2	CXCL2	WNK2	SEZ6L2	HTRA1	AQP5	ASS1	INSR	GLIPR2	CAPS	CHST9	NR4A2	HLA-B	SLC1A5	NECTIN1	TTYH1	SYCE1L	RASSF6	GRAMD2B	LURAP1L	PALLD	NPDC1
CELLMARKERMSIGDB_HE_LIM_SUN_FETAL_LUNG_C2…	SLC29A3	DST	CALU	SDC3	NR1H3	SLC38A6	CD151	SCD	MGLL	CREB3L2	SIGLEC7	SLC36A1	AMDHD2	STOM	VMO1	ACP5	FPR3	BCAM	GFRA2	FABP3	SIGLEC1	RPP25	TMIGD3	BOLA3	OTOA
CELLMARKERMSIGDB_HE_LIM_SUN_FETAL_LUNG_C2…	EGLN3	NET1	ITGB7	CALCRL	SLAMF8	NAPSA	PTK2	TACSTD2	CXCR3	SERPINF2	DENND1B	BTLA	LINC02…	ZNF366	LYRM4	XCR1	MYCL	LMNA	CCND1	RHOF	GNG7	PKIB	PDE4DIP	DCTPP1	SNX22
CELLMARKERMSIGDB_HE_LIM_SUN_FETAL_LUNG_C2…	TCTN3	IL18	CCDC86	HLA-DQB2	FLT3	SPINT1	SLC38A1	TMEM273	MSLN	ALCAM	ST14	SLAMF8	FILIP1L	FCGR2B	TMEM156	COL9A2	KCNMB1	TVP23A	IRF4	GEM	MOB3B	PTGDR2	BCL7A	FBLN2	SNAI3
CELLMARKERMSIGDB_HE_LIM_SUN_FETAL_LUNG_C2…																									
CELLMARKERMSIGDB_HE_LIM_SUN_FETAL_LUNG_C2…	MCM6	CST7	ERLIN1	SLC43A1	C16orf…	CITED4	RAB44	TSPOAP1	KCNE5	FKBP11	PCLAF	MEG3	ARMH1	HGF	MGST1	DDX11	TOP1MT								
CELLMARKERMSIGDB_HE_LIM_SUN_FETAL_LUNG_C2…	CD69	IGF2BP2	APP	CTSW	CHST12	CDCA7	BEX3	ISYNA1	RAB37	NUCB2	GUCY1A1	HMGA2	TFPI	MEST	ADA	BCL11A	OCIAD2	TBC1D10C	CXXC5	TRBC2	NPR3	ARMH1	PRKACB	B3GNT2	ANGPT1
CELLMARKERMSIGDB_HE_LIM_SUN_FETAL_LUNG_C2…	TPM1	LTBP1	MMRN1	CLU	GNG11	CDKN2D	CNST	NFE2	DMTN	NEXN	RDH11	P2RX1	NT5C3A	PRKAR2B	SELP	PTGS1	TSPAN32	GFI1B	RUFY1	GRAP2	MYL9	ITGB3	GP6	ATP2C1	LGALSL
CELLMARKERMSIGDB_HE_LIM_SUN_FETAL_LUNG_C2…	CLEC4E	PROK2	HP	ACSL1	AQP9	PGLYRP1	LTB4R	TUBA4A	CMTM2	THBS1	RGL4	TREM1	HK2	PADI2	RAB27A	FCAR	ORM2	CLEC4D	HK3	FPR2	ADM	CYFIP2	CPD	LINC00…	ADGRG3
CELLMARKERMSIGDB_HE_LIM_SUN_FETAL_LUNG_C2…	SLC39A8	TUBA4A	KIFAP3	NFE2	GADD45A	MOB1B	LTBP1	LRP12	MEIS1	GATA1	H2BC15	C12orf76	BCL2L1	H2AC15	H2BC11	H4C2	ECE1	PLEKHF2	XPNPEP1	DGKD	RIPOR2	NET1	TMEM40	EPB41	P2RY1
CELLMARKERMSIGDB_HE_LIM_SUN_FETAL_LUNG_C2…	MYL6B	PPM1J	SIGLEC6	RHEX	ST14	IGKC	JCHAIN	HAUS1	ACAP1	SLAMF7	CXXC5	LMNB1	IDH3A	CARHSP1	CDKN2D	CDCA7L	SEL1L3	FBLN1	SPNS3	OPN3	SH2D3C	UPF3B	RIPOR2	SIT1	CYB5B
CELLMARKERMSIGDB_HE_LIM_SUN_FETAL_LUNG_C2…	TYMS	SERPIN…	HELLS	MCM2	RFLNB	RRM1	ATAD2	PHGDH	CENPK	CENPF	GINS2	UBE2C	IGFBP2	KBTBD11	KCNE5	WEE1	PCLAF	TK1	TIMELE…	MCM4	NT5DC2	TOP2A	CIP2A	SLC2A4…	MLC1
CELLMARKERMSIGDB_HE_LIM_SUN_FETAL_LUNG_C2…	RAB27A	MTMR11	DYSF	G0S2	KCNE1	LTB4R	ADM	MGST1	VSTM1	VNN2	STEAP4	OLIG1													
CELLMARKERMSIGDB_HE_LIM_SUN_FETAL_LUNG_C2…	SLC7A8	BLVRA	NPL	CD209	ITSN1	ABCA1	PCDH12	MTUS1	RCN3	COLEC12	MERTK	NRP2	ABCG2	ABCC5	SCN9A	RAB3IL1	PLEKHG5	TMEM37	TNFRSF…	HOMER3	ITGB5	C2	FAM20A	SLC12A5	
CELLMARKERMSIGDB_HE_LIM_SUN_FETAL_LUNG_C3…	TBC1D20	KHDRBS2	MIER2	HOPX	CYB561	RNF24	BTBD3	NEDD4L	EVA1B	VASN	PHLDB1	SULT1A1	EHD3	ITGA3	B3GALNT1	RARG	ZNFX1	DPEP1	LRRC36	RILPL2	SYNM	ADAMTS1	PRICKLE2	CDH8	ADAMTS…
CELLMARKERMSIGDB_HE_LIM_SUN_FETAL_LUNG_C3…	CCNA2	SLC38A5	CCNB2	TOP2A	TPX2	SPTB	MINPP1	SPTA1	ICAM4	DNAJA4	H2AC14	ZWINT	DLGAP5	SMIM1	ERMAP	GFI1B	KEL	H2AC13	PTTG1	BIRC5	SLC1A5	HBZ	ADD2	RHCE	UBE2T
CELLMARKERMSIGDB_HE_LIM_SUN_FETAL_LUNG_C3…	STC1	CLEC11A	CYRIB	KPNA2	TRAF4	MADCAM1	PAICS	TMEM17…	NNAT	ADORA2A	LHX6	HNRNPA1…	SKA2	TCF15	UCHL1	H1-3	WIPI1	MLLT11	C19orf…	RAC3	FKBP1C	FEZ1	MYO10	WASF1	UQCRHL
CELLMARKERMSIGDB_HE_LIM_SUN_FETAL_LUNG_C3…	TSHZ2	SGCE	ADIRF	PDPN	TNFRSF…	STAB2	FLNC	NR2F1	GPM6A	GALNT1	ALDH3A2	PDLIM1	SBSPON	RASSF9	TBX1	IGF1	CCDC80	TCTN3	MFAP4	COLEC12	STON2	PMP22	DPP4	ENTREP1	RNASE4
CELLMARKERMSIGDB_HE_LIM_SUN_FETAL_LUNG_C3…	PTPRE	CRACR2B	FLNC	DSP	PDE2A	SIRPA	SCN3B	STON2	RAB11FI…	SAT2	SLC27A3	ITGA9	MPP7	NTS	GPM6A	LTBP4	SLC38A1	MTUS1	TIMP2	FAM102A	LAMB1	IL7	VPS35L	GNG12	ENOSF1
CELLMARKERMSIGDB_HE_LIM_SUN_FETAL_LUNG_C3…	CBX2	ADORA2A	GPIHBP1	EDIL3	TIAM1	PDE3B	NCKAP5	LHX6	MAOA	FMNL2	DPEP1	CA2	ARHGEF…	MIR181…	AFF3	MADCAM1	RASSF2	NETO2	KHDRBS2	ANGPTL4	CRYBG1	TLE2	CCL2	ANKRD44	MYOC
CELLMARKERMSIGDB_HE_LIM_SUN_FETAL_LUNG_C3…	HEY2	MMP16	TMEM12…	JAG1	QSOX1	SELENOM	ALDH1A1	MECOM	GATA6	CCN1	IFITM1	NOMO2	GASK1B	TSPAN2	CRIM1	BMX	SFRP4	FBN2	CXCL2	BMP4	CPAMD8	ID2	CRISPLD1	EXT1	SMAD6
CELLMARKERMSIGDB_HE_LIM_SUN_FETAL_LUNG_C3…	TTN	LTBP4	PTPN14	GJD3	MCUB	PDLIM1	SCG3	DAPK1	CCL21	UNC5B	PDPN	SULF2	ITGB4	CDC42E…	C17orf58	KANK2	GLRX	GUCY1A1	CEP85L	CCDC141	ADIRF	ITGAV	FAM3C	C20orf…	SGCE
CELLMARKERMSIGDB_HE_LIM_SUN_FETAL_LUNG_C3…	PLAC9	IL1R1	NPNT	PLXDC2	COL9A3	CHODL	ECRG4	CDC42E…	C7	CDH11	LYPD2	CD82	LRATD2	SORBS2	RARRES1	COL3A1	SEMA3F	ALCAM	MIR99A…	FBLN2	ITGB4	FILIP1L	DLL1	LFNG	LYST
CELLMARKERMSIGDB_HE_LIM_SUN_FETAL_LUNG_C4…	HAVCR2	FEZ1	SPATA13	FAM3C	NDFIP2	CD82	ICAM1	PNO1	SNX8	EGR2	CD72	DOT1L	RRS1	TNFRSF4	SFXN4	STX11	UCK2	PUS1	BCL2A1	BCL2L11	RRP9	TMEM12…	TIMM8A	NEK6	
CELLMARKERMSIGDB_HE_LIM_SUN_FETAL_LUNG_C4…	THEMIS	ICOS	CSGALN…	CTSL	PASK	RAB25	EPHX2	FKBP5	LINC015…																
CELLMARKERMSIGDB_HE_LIM_SUN_FETAL_LUNG_C4…																									
CELLMARKERMSIGDB_HE_LIM_SUN_FETAL_LUNG_C4…	TOB1	RCAN3	PRKAR1B	SERPINF1	SGK1	TMEM204	SERINC5	CD79A	NT5E	MAL	BACH2	JUP	IGFBP2	SREBF1	PAK1	MEOX1	LYPD3	PCNT	DBN1	GBP1	NPDC1	TRABD2A	LINC015…	CLEC11A	FAM117B
CELLMARKERMSIGDB_HE_LIM_SUN_FETAL_LUNG_C4…	H2AC14	ATAD2	PCNA	LMNB1	H1-5	PKMYT1	DNAJC9	SPC25	GMNN	RRM1	APOBEC3B	DYNC2I2	AURKB	SAC3D1	FEN1	CCNA2	MAD2L1	DHFR	EZH2	CDT1	H2AC13	LIG1	GTSE1	H3C2	PTTG1
CELLMARKERMSIGDB_HE_LIM_SUN_FETAL_LUNG_C4…	NUDT1	CENPU	CCNA2	CKAP2L	ZWINT	KIF22	UHRF1	FAM111A	SPC25	CDCA3	H1-5	NCAPD2	RRM2	CDCA8	CLSPN	H3C2	PKMYT1	H2AC13	DTYMK	CDCA5	KIF11	RRM1	USP1	KIF2C	PTTG1
CELLMARKERMSIGDB_HE_LIM_SUN_FETAL_LUNG_C4…	TOX2	SPATS2L	CDKN1A	NRIP1	NFATC1	AHR	LZTFL1	CPNE7	STK24	ARHGAP10	CXCR6	BLK	GGT1	TSPAN15	FYCO1	TIAM1	JMY	ROGDI	MATN2	APP	REC8	MGAT5	CSF1	ADAM19	SLC4A10

Table S5: Cell state marker gene list

CELLMARKERCAN_ACINAR_CELLS	PNLIPRP1	CTRC	REG3A	SERPINA3	PRSS3	REG1B	CFB	GDF15	MUC1	C15ORF48	DUOXA2	AKR1C3	OLFM4	GSTA1	LGALS2	PDZK1IP1	RARRES2	CXCL17	UBD	GSTA2	ANPEP	LYZ	ANGPTL4	ALDOB	
CELLMARKERMSIGDB_HE_LIM_SUN_FETAL_LUNG_C4…																									
CELLMARKERMSIGDB_HE_LIM_SUN_FETAL_LUNG_C4…	NREP	VOPP1	THEMIS	RANBP6	SLC29A1	GALNT7	RRAGB	BCOR	LETM1	DNASE1	RPTOR	APC	PTCRA	CBFA2T3	TNFRSF21	PRKCG	RORC	PPP1R1C	RASSF6	MAP1A	CYP2U1	PTPRM	CD2AP	GNA15	MCTP1
CELLMARKERMSIGDB_HE_LIM_SUN_FETAL_LUNG_C4…	HS3ST3B1	IKZF4	TSPAN5	TMEM154	EPHX2	HNRNPLL	LGALS9	FAS	GPA33	GK	FBLN7	SNX9	CYTOR	CD79A	CHRM3-AS2	BCL2L11									
CELLMARKERMSIGDB_HE_LIM_SUN_FETAL_LUNG_C5…	PLPP5	CD82	ACP5	PLEK	CCR6	ITGB7	IL27RA	CPNE5	EGR1	PNOC	RBKS	COL19A1	TRAF5	TENT5C	RIN3	BTLA	KLF4	CTSH	BIN2	ZNF331	GPR65	NCR3	IL10RA	NR4A3	DPEP2
CELLMARKERMSIGDB_HE_LIM_SUN_FETAL_LUNG_C5…	SRGN	TYMP	FCRL5	CTSC	MIR155…	TYW3	SMS	CLEC17A	MTERF4	CFLAR	S100A6	CXCR5	RASA4B	ATIC	PRKCD	SNX11	CTSZ	MCOLN2	EHD1	DOK2	PHPT1	PKIG	PLK3	HCST	IL10RA
CELLMARKERMSIGDB_HE_LIM_SUN_FETAL_LUNG_C5…																									
CELLMARKERMSIGDB_HE_LIM_SUN_FETAL_LUNG_C5…	PSD3	CD27	CKMT2	LILRA2	ITGA5	GSN																			
CELLMARKERMSIGDB_HE_LIM_SUN_FETAL_LUNG_C5…	FEN1	EGFL7	ATAD2	KIF22	CKS1B	CENPW	MDK	DHFR	SMS	PKMYT1	CDK1	CENPF	ETS2	ARL6IP6	EZH2	PCNA	SYNGR1	ERG	TPX2	HSPB11	SKA2	FAM111A	HMGB3	SCCPDH	GMNN
CELLMARKERMSIGDB_HE_LIM_SUN_FETAL_LUNG_C6…	PLAAT2	SLC2A10	CORO2B	WDR24	HBB	FGF14	SEPTIN11	CYP2W1	TRPV4	CP	CCDC190	MT3	DIAPH2	WFDC6	CES4A	KLHL6	APOD	ITGB4	LARP6	PZP	KLHL5	KRT7	AQP5-AS1	CD82	PAX9
CELLMARKERMSIGDB_HE_LIM_SUN_FETAL_LUNG_C7…	ANGPT1	RPS4Y1	NFIX	FRAS1	ALDH1A3	DPY19L3	GPR17	TGFBI	SMIM5	TFAP2C	CTNNA3	LMO3	DGKB	GRID2	ENG	GABRA2	PLA2G4A	MYO1C	MGAT4C	DYNLT2B	TMEM35B	ADAMTS…	CASP4	IL17RC	HSPB2
CELLMARKERMSIGDB_HE_LIM_SUN_FETAL_LUNG_C7…	FRZB	SYNM	ANKHD1	LAMA5	WFIKKN2	DNASE2	ARHGAP31	ZNF599	NKD1	PRR34-A…	MAP3K5	ARHGAP18	WDR27	PKP2	UNC5B	FRMD5	TMEM100	BLOC1S5	LINC02…	NDUFA4L2	KCNIP4	PSMB8	EGLN3	NPB	EGLN2
CELLMARKERMSIGDB_HE_LIM_SUN_FETAL_LUNG_C7…	IL11RA	BGN	SAMHD1	PLEKHA4	PRSS23	PON2	ITGA1	RAB20	FIBIN	MIA	RNASE1	WIPF1	MAL	SORBS1	FBLN5	USP53	SHFL	AQP1	MYL9	XKR4	CD44	BHLHE40	ASPA	COL16A1	TRPM3
CELLMARKERMSIGDB_HE_LIM_SUN_FETAL_LUNG_C7…	SV2C	SYT4	AKR1C2	KLHL5	NTNG1	FRY	ELMO1	HMGCLL1	ENTPD3	HOXD1	PLCB2	CNKSR2	DLGAP1	CELF5	B3GNT4	PNCK	CHRM3	SEPTIN3	SH3GL2	SLC18A2	HRH1	TRPV2	SPOCK3	SLC6A2	SCN2A
CELLMARKERMSIGDB_HE_LIM_SUN_FETAL_LUNG_C7…	ARL4C	SNCG	VAT1L	ICA1	ARHGDIG	TAC3	SLC2A3	GNAO1	SEZ6L	DUSP26	SLC4A8	PLPP4	RUFY2	REEP2	PIANP	ISLR2	ADAM22	TSPAN7	TRIM36	NRP1	FAM241B	ELAVL2	ALCAM	MTUS1	GATA3
CELLMARKERMSIGDB_HE_LIM_SUN_FETAL_LUNG_C7…	APC2	STX1A	SYNPO2	ARHGEF3	ARID3B	VSTM2A	TRIM36	RPS6KL1	CNGB1	ABCB1	HMX2	PLEKHA6	HS3ST5	CDK5R1	FBP1	HID1	REM2	MTMR7	SNX10	ST6GAL2	EPS8L1	SGIP1	BTBD8	FNDC5	CLCN4
CELLMARKERMSIGDB_HU_FETAL_RETINA_AMACRINE	DIRAS2	MIR124…	SCN3A	LINC00461	NNAT	PCBP3	MEIS1	RORB	SRGAP3	TRIB2	GRIA2	TAGLN3	ANK3	ELMOD1	PAX6	SYT1	RBFOX2	RTN1	STMN2	MAB21L1	MAP2	CCDC88A	SOX4	SCG3	INA
CELLMARKERMSIGDB_HU_FETAL_RETINA_BIPOLAR	MIR9-1HG	TF	BTBD8	FRMD3	GPX3	SV2B	STX3	SLC4A10	CABP2	NRXN3	ZNF385B	UNC119	PROX1	CAMK2B	GNG13	NEUROD1	CRYBG3	PDC	MAP7	MIR124-…	CRYM	TRNP1	OTX2	SNAP25	SAG
CELLMARKERMSIGDB_HU_FETAL_RETINA_FIBROBLA…	SHISA2	CD248	EDN3	LUM	ITM2A	HBZ	NR3C1	EMCN	RARB	LAMB1	PRRX1	NID1	FOXC2	CTSC	COL5A1	COL5A2	KCTD12	PIEZO2	FOXF2	PDGFRA	COL6A2	MIR99A…	OGN	PMP22	SERPIN…
CELLMARKERMSIGDB_HU_FETAL_RETINA_HORIZONT…	CRYGD	MIAT	MGARP	TAGLN3	SCN3A	CD24	MLLT11	SOX4	TUBB2B	RTN1	MEIS1	NNAT	PAX6	JPT1	TUBA1A	STMN1									
CELLMARKERMSIGDB_HU_FETAL_RETINA_MICROGLIA	IFI16	CYBB	S100A11	CCL3L3	PLD4	A2M	VAMP8	KCTD12	CTSS	ZFP36	VSIG4	VSIR	HLA-B	C3AR1	RNASE6	MS4A7	OLR1	TREM2	FCGR1A	LPAR6	GPR34	PTPRC	ADAM28	TMEM52B	SPP1
CELLMARKERMSIGDB_HU_FETAL_RETINA_MULLER	SAT1	RASSF4	TRIB2	CDC42EP4	RCVRN	GAD2	CLU	ZFP36L1	SIX6	NCAM1	RTN4	IRF4	PEA15	CKB	VIM	BEX1	NNAT	SNRPG							
CELLMARKERMSIGDB_HU_FETAL_RETINA_PHOTOREC…	MAK	PROM1	RBP3	MIR124-1…	FAM161A	PRDM1	CDHR1	IMPG2	EPB41L2	FABP7	NTM	PRPH2	ATP1B2	CCDC181	CAMK2B	NEUROD4	UNC119	AANAT	RIMS2	OTX2	PCBP4	SLC17A7	FSTL5	TULP1	PTPN13
CELLMARKERMSIGDB_HU_FETAL_RETINA_RGC	MLLT11	RGS10	KLF7	REEP1	CDKN2D	SMIM18	UCHL1	TAGLN3	CXCR4	CRMP1	STMN2	RBPMS	SCN9A	GLRX	L1CAM	ADCY1	EPS8L1	DPYSL3	TSPAN7	TRIM36	SLC17A6	CRIP2	ELAVL2	MAPT	SVBP
CELLMARKERMSIGDB_LAKE_ADULT_KIDNEY_C1_EPI…	ZBTB16	TLN2	PLCL1	SLC5A12	NPAS3	RAPGEF2																			
CELLMARKERMSIGDB_LAKE_ADULT_KIDNEY_C10_TH…	UMOD	NCOA7	TGM2	FTH1	SERPIN…	RASD1	IFITM2	CBR1	HNRNPA2…	ID2	HSP90B1	TFPI	GDF15	RIMS2	EPCAM	CEBPD	RPLP0	HSPD1	KRT7	KRT18	IDH2	CLDN10…	RPS2	GAPDH	LINC01…
CELLMARKERMSIGDB_LAKE_ADULT_KIDNEY_C12_TH…	PEBP1	CXCL14	ATP5F1B	SFRP1	SERPIN…	SELENB…	ATP1A1	RARRES2	TMEM37	WNK4	ESRRB	IDH2	SDC1	DNASE1	SLC25A4	LDHB	COX7B	SLC22A2	ATP5MC3	COX4I1	PRSS8	USP2	CASR	ACSS1	NDUFB9
CELLMARKERMSIGDB_LAKE_ADULT_KIDNEY_C18_CO…	FUCA1	PWRN1	KRT8	CD24	KITLG	EPCAM	NUPR1	GATA3	CDH16	ITM2B	ACTG1	SPTBN2	FYB2	TMBIM6	H3-3B	CD9	SLC16A5	ASAH1	RPL3	HSP90AA1	MYL12B	DUSP6	EEF1A1	CYB5A	TOX
CELLMARKERMSIGDB_LAKE_ADULT_KIDNEY_C19_CO…	SLC4A1	DHRS7	RHCG	EIF1	AKR1C1	HSP90A…	INSIG1	CD9	MSMO1	RPL3	TXNIP	RPL15	PTGER3	CD24	MAP1LC3B	RPS6	PRDX1	TMSB4X	RPS2	EPCAM	CA12	SCIN	SAT1	ATP6V0B	COX4I1
CELLMARKERMSIGDB_LAKE_ADULT_KIDNEY_C25_EN…	TCF12	SSH2	KMT2E	FCHSD2	TMEM16…	ZFC3H1	PPFIBP1	FOXP1	ZFHX3	ZFAND3	FOXP2	MYH9	SRPK2	CAST											
CELLMARKERMSIGDB_LAKE_ADULT_KIDNEY_C28_IN…	PRKG1	PTEN	SORBS1	LDB2	IGFBP7	ARHGAP…	NFIA	NAV2	TPM1	INPP4B	RERG	COL4A2	THSD4	KALRN	NR2F2-AS1	UTRN	MAP3K5	CBLB	COL4A4	ZBTB16	PDLIM5	EXT1	USP53	ROCK2	DST
CELLMARKERMSIGDB_LAKE_ADULT_KIDNEY_C29_UN…	NALF1	MAGI2	BNC2	BICD1	RHEX	MAML3	BCL2	ZBTB20	BICC1	OSBPL10	NEDD9	CRIM1	CNTN4	TMEM17…	PRUNE2	TPM1	PARD3	PALLD	UTRN	L3MBTL4	ZBTB16	DCDC2	ZDHHC14	THRB	WWOX
CELLMARKERMSIGDB_LAKE_ADULT_KIDNEY_C5_PRO…	EPHA8	LRFN2	DPP10	CAMK4	CLUAP1	PTMS	AFM	RASGEF…	R3HDML-…	FXR2	ZNF614	PDHX	C2orf2…	FSIP1	PARP10	PTH1R	MOGAT1	LINC013…	CCDC149	SLC22A11	MARK2	TTC23L	EOLA1-DT	PLG	LRRC4C
CELLMARKERMSIGDB_MANNO_MIDBRAIN_NEUROTYPE…	FAU	GABARAP	H3-3B	HNRNPA2B1	HNRNPDL	HNRNPK	HNRNPU	HSPA8	MARCKS	NACA2	NDUFA13	BEX3	PARK7	PCBP1	PFDN5	PSMB4	RBM39	RBM8A	RPL13	RPL14	RPL15	RPL28	RPL3	RPL31	RPL34
CELLMARKERMSIGDB_MANNO_MIDBRAIN_NEUROTYPE…	APP	ARG2	ARHGAP…	ARHGEF3	ARL8A	ASNS	ATCAY	ATP1A3	ATP2B2	ATP8A1	BASP1	BCL11A	BEX2	BEX5	BIN1	BIVM-ERC…	ARMH4	C1orf35	C1QL1	C22orf42	C2CD4C	COL8A1	CA1	CACNA1E	CACNG2
CELLMARKERMSIGDB_MANNO_MIDBRAIN_NEUROTYPE…	APLP1	APP	ARG2	ARHGAP23	ARHGEF3	ARL8A	ARMCX5-…	ARRB1	ASB4	ASIC2	ASXL3	ATCAY	ATP11C	ATP1A3	ATP1B1	ATP8A2	ATP9A	B3GALT2	BASP1	BCL11A	BDNF	BEND4	BEX5	BNC2	TSPOAP1
CELLMARKERMSIGDB_MANNO_MIDBRAIN_NEUROTYPE…	ASXL3	ATCAY	ATP1A3	ATP1B1	ATP8A2	ATP9A	B4GALNT1	B4GALT6	BAALC	BASP1	BDNF	BEGAIN	BEX1	BEX2	BEX5	BLCAP	BNC2	BRSK1	BSN	BTBD1	C12orf76	C19orf…	C1orf35	C1QL1	C22orf…
CELLMARKERMSIGDB_MANNO_MIDBRAIN_NEUROTYPE…	ADM	ADM5	ADORA2A	ADORA2A-…	AFAP1L1	AFAP1L2	AFF1	AHNAK	AJAP1	AKAP12	ALPL	AMOTL1	ANGPT2	ANGPTL4	ANKRD37	ANXA1	ANXA11	ANXA2R	ANXA3	ANXA4	ANXA5	AP1S2	APCDD1	APLN	APLNR
CELLMARKERMSIGDB_MANNO_MIDBRAIN_NEUROTYPE…	APOBEC3C	APOBEC…	APOBR	APOC1	APOC2	APOE	ARHGAP25	ARHGAP…	ARHGAP4	ARHGDIB	ARHGEF6	ARPC1B	ARRB2	ARRDC2	ASAH1	ATF3	PGGHG	ATP8B4	AXL	B2M	B3GNT5	B4GALT1	BAG3	BCAP29	BCL2A1
CELLMARKERMSIGDB_MANNO_MIDBRAIN_NEUROTYPE…	ANKRD12	ANKRD1…	ANKRD3…	ANKRD44	ANKRD9	ANO5	ANO8	APLP1	APOL6	CHN1	ARID4A	ARPP21	ASIC2	ASNS	ASXL3	ATCAY	ATP1A3	ATP1B1	ATP2B1	ATP2B2	ATP8A2	ATP9A	B4GALNT4	BAALC	BACH2
CELLMARKERMSIGDB_MANNO_MIDBRAIN_NEUROTYPE…	C1QL4	C2CD4C	CACNB3	CACNB4	CACNG7	CADPS	CBLN2	CCBE1	CCDC122	CCDC184	CD2	CD24	CDK5R1	ADA2	CELF3	CELF5	CELF6	CELSR3	CLCN4	CMIP	CNR1	CNTN2	CRMP1	CSMD3	CSRNP3
CELLMARKERMSIGDB_MANNO_MIDBRAIN_NEUROTYPE…	APC2	APLP1	APOL6	ARPP21	ASIC1	ASIC4	ASXL3	ATCAY	ATP1A3	ATP8A1	AUTS2	B3GALT2	ADGRB3	BASP1	BCL11A	BCL11B	BEND4	BHLHE22	BICD1	BSN	TSPOAP1	BZW2	C1orf35	C22orf…	CACNA1G
CELLMARKERMSIGDB_MANNO_MIDBRAIN_NEUROTYPE…	ATP1A3	ATP2B1	ATP2B2	ATP6V1A	B4GALN…	BACH2	ADGRB3	BASP1	BEX1	BEX2	BEX5	BRINP2	BSN	BZW2	C11orf87	C17orf75	C1orf35	C1QL2	C4orf48	CACNA1C	CACNB3	CACNG2	CADM1	CADM3	CAP2
CELLMARKERMSIGDB_MANNO_MIDBRAIN_NEUROTYPE…	ATCAY	ATP13A2	ATP13A4	ATP1B1	ATP1B2	ATP2B4	B4GALNT1	B4GALT6	BAALC	ADGRB1	BAMBI	BARD1	BCAN	BCHE	BIRC3	BIRC5	BIVM-E…	BLOC1S1	BOC	BRINP1	BRINP2	BRINP3	BUB1B	C1orf21	MIR9-1…
CELLMARKERMSIGDB_MANNO_MIDBRAIN_NEUROTYPE…	ALDH9A1	ALX4	JAML	AMOTL1	ANGPT2	ANGPTL4	ANKRD37	ANKRD44	ANPEP	ANTXR1	ANXA2	ANXA4	ANXA5	ANXA6	AP2S1	APOBEC3C	APOE	APOL6	APOLD1	ARAP3	ARHGAP1	ARHGAP…	ARHGAP24	ARHGAP…	ARHGAP…
CELLMARKERMSIGDB_MANNO_MIDBRAIN_NEUROTYPE…	C19orf48	C21orf…	KNL1	CCDC34	CCNA2	CCNB1	CCNB2	CCND1	CDK1	CDC25C	CDCA3	CDCA5	CDCA8	CDHR1	CDK2	CDKN3	CELSR2	CENPE	CENPF	CENPK	CENPN	CENPQ	CENPU	CENPW	CHEK1
CELLMARKERMSIGDB_MANNO_MIDBRAIN_NEUROTYPE…	BUB1	BUB1B	C21orf…	C22orf15	DIPK2A	CALML4	CAP2	KNL1	CCDC8	CCNA2	CCNB1	CCNB2	CCND1	CDK1	CDC25A	CDC25C	CDCA3	CDCA5	CDCA8	CDH2	CDK2	CDK6	CDKN3	CELSR1	CENPE
CELLMARKERMSIGDB_MANNO_MIDBRAIN_NEUROTYPE…	BRCA2	BUB1	BUB1B	C19orf48	C21orf…	C22orf…	DIPK2A	C6orf1…	CAP2	KNL1	CCDC34	CCDC8	CCNA2	CCNB1	CCNB2	CCND1	CDK1	CDC25A	CDC25B	CDC25C	CDCA3	CDCA5	CDCA8	CDKN3	CEBPA
CELLMARKERMSIGDB_MANNO_MIDBRAIN_NEUROTYPE…	BMP4	BMP7	BRCA1	BUB1	BUB1B	HOATZ	C1orf226	PRADC1	DIPK2A	C6orf118	TCIM	CAP2	CFAP210	CCDC8	CCNB2	CCND1	CD47	CDK1	CDC25A	CDCA3	CDCA5	CDCA8	CDKN3	CDO1	CDON
CELLMARKERMSIGDB_MANNO_MIDBRAIN_NEUROTYPE…	BRCA1	HOATZ	C1orf2…	C21orf62	DIPK2A	CFAP299	C5orf49	C6orf1…	C8orf34	CFAP77	C9orf24	CADM3	CAP2	CBLN4	CCDC146	ODAD3	CCDC17	CFAP210	CCDC181	CCDC39	CCND2-AS1	CD44	CDC25C	CDK6	CDO1
CELLMARKERMSIGDB_MANNO_MIDBRAIN_NEUROTYPE…	ARHGEF1	ARHGEF…	ARHGEF4	ARHGEF6	ARMC3	ARVCF	ASTN1	ATP13A4	ATP1A2	ATP1B2	ATP2B2	ATP2B4	ATXN1	B3GAT1	B3GNT2	ADGRB2	ADGRB3	BCAN	BCAP29	BCHE	BDH2	BMP7	BOC	HOATZ	C12orf…
CELLMARKERMSIGDB_MANNO_MIDBRAIN_NEUROTYPE…	ATP1B2	ATP2B2	ATP2B4	AURKB	ADGRB2	ADGRB3	BARD1	BCAN	BCAP29	BCL6	BIRC5	BOC	BRCA1	BTN3A3	BUB1B	C17orf67	MIR9-1…	C21orf58	C2orf72	C6orf118	C8orf34	C9orf24	CA12	CABIN1	ODAD1
CELLMARKERMSIGDB_MANNO_MIDBRAIN_NEUROTYPE…	BIRC5	HOATZ	C1orf21	MIR9-1HG	C21orf…	C21orf…	C22orf15	C2orf72	THUMPD2	C6orf118	VXN	C9orf24	CA12	CCDC39	CCDC8	CD44	CD82	CDCA5	CDH13	CENPF	CENPK	CENPN	CFAP44	CHKA	CHST3
CELLMARKERMSIGDB_MANNO_MIDBRAIN_NEUROTYPE…	ARAP2	ARHGAP…	ARHGAP5	ARHGEF26	ARHGEF…	ARHGEF…	ARL6IP5	ARMC3	ARX	ATF3	ATP1A2	ATP1B2	ATP2B2	ATXN1	B3GNT2	B4GALT6	ADGRB2	BCAN	BCHE	BCO2	BMP1	BNC2	BOC	TSPOAP1	HOATZ
CELLMARKERMSIGDB_MANNO_MIDBRAIN_NEUROTYPE…	B3GALT2	BAALC	BASP1	BCL11A	BEND5	BIVM-E…	BRINP2	BZW2	C1orf21	C1orf35	C1QL4	CACNB3	CACNG2	CADPS	CBLN2	CCDC184	HAUS1	CD2	CD24	CDC42EP3	CDH22	CDK5R1	ADA2	TMEM12…	CELF3
CELLMARKERMSIGDB_MANNO_MIDBRAIN_NEUROTYPE…	ATL1	ATP1A3	ATP2B2	ATP6V1A	B9D1	BAALC	ADGRB1	BASP1	BEX1	BEX2	BEX5	BOP1	BRINP1	BRINP3	BTBD1	BTBD6	CFAP276	FNDC10	C22orf…	CA1	CACNA1A	CACNA1B	CACNA1C	CACNA1E	CACNA1G
CELLMARKERMSIGDB_MENON_FETAL_KIDNEY_0_CAP…	SOX4	TUBA1A	PAX2	LUC7L3	HMGB1	PTMAP2	BAZ1B	RBM25	SEPTIN6	ILF3	RPL13AP…	ANP32B	BAZ2B	GOLGA4	TOP2A	MDK	SMC4	SYNE2	SF3B2	EIF3A	BST2	ANKRD11	H4C3	ZC3H13	MKI67
CELLMARKERMSIGDB_MENON_FETAL_KIDNEY_10_IM…	MEF2C	CST3	TMSB4X	HLA-C	FGL2	TPM3	MAF	ACTB	COTL1	GPX1	ACTR2	FTL	PFN1	RGS10	SH3BGRL3	FTLP3	HLA-E	ARPC2	KLF6	CNPY3	SAT1	PABPC1	TMSB4XP8	CCDC88A	CAP1
CELLMARKERMSIGDB_MENON_FETAL_KIDNEY_2_NEP…	RPSA	ODC1	HOXD8	SOX4	LAMB1	RAB3IP	ABLIM1	TRIB2	TUBB2B	JAG1	MAL	ANP32E	HOXA10	RPLP0P6	MARCKSL1	HIPK2	BTG1	DBI	AFDN	EIF3F					
CELLMARKERMSIGDB_MENON_FETAL_KIDNEY_3_STR…	MEG3	CTSC	NR2F2	CDH11	MYLK	TPM1	SFRP1	TPM4	IGFBP5	MEIS2	GPC3	COL4A1	MARCKS	XIST	UACA	SEPTIN11	TCF21	FSTL1	ALCAM	SPARC	PALLD	PXDN	RBP1	RAI14	DST
CELLMARKERMSIGDB_MENON_FETAL_KIDNEY_6_COL…	CYB5A	MAL2	NPNT	KRT18	RAB11F…	COBLL1	S100A11	AKR1B1	DMKN	IQGAP1	HOXB7	CD9	AFDN	EMX2OS	TMSB4XP8	ANXA2	FOS	PTBP3	CCDC198	ASAH1	MAL	KLF6	SCOC	JUN	SAT1
CELLMARKERMSIGDB_MURARO_PANCREAS_ACINAR_C…	GSTA1	OLFM4	PNLIPR…	PRSS3	BCAT1	CELA3B	LGALS2	KLK1	DUOX2	ANPEP	ADH1C	CFB	LYZ	FGL1	REG3G	MUC1	IL32	CXCL17	SERPIN…	AKR1C3	AKR1C2	SERPIN…	MXRA5	AKR1B10	AOX1
CELLMARKERMSIGDB_MURARO_PANCREAS_ALPHA_CELL	CALCOCO2	HECTD4	RAB11A	DNAJC12	VAMP2	PKD2	MYL6	IDI1	NOMO1	UBL5	AHI1	CMIP	TRNP1	ANXA6	CADPS	BOLA3	ABHD16A	AK2	ABI2	SRPK2	TMEM205	HMGN3	COX7A2	HIPK2	DYNLRB1
CELLMARKERMSIGDB_MURARO_PANCREAS_BETA_CELL	PCSK1	RRAGD	PCDH7	CASR	FTL	OTULINL	ENTPD3	TIMP2	ENO2	OLMALINC	PLCXD3	ROBO2	SHISAL…	MAPT	PRUNE2	SMAD9	ATP2A3	SYT7	ID1	STXBP1	LOC154761	SLC30A8	SURF4	ARG2	ITPR3
CELLMARKERMSIGDB_MURARO_PANCREAS_ENDOTHEL…	ADGRF5	F2RL3	NRP2	ERG	PRDM1	MMRN2	MCAM	GASK1B	TCF4	EMP1	BDKRB2	CXCR4	THBD	APLNR	ITGA5	CLEC14A	NOTCH4	SNAI1	IGFBP4	SGK1	FSCN1	MYCT1	EMCN	NKX2-3	SHANK3
CELLMARKERMSIGDB_MURARO_PANCREAS_EPSILON_…	VSTM2L	TRNP1	NUAK1	SPON2	KCNH2	CDKN2A	FFAR4	SAMD5	PROX1	PIGK	SYNJ2	SERPINB6	SPINK1	ASAH1	ARX	PCSK1	ZKSCAN1	CLU	TM4SF4	PEG10					
CELLMARKERMSIGDB_MURARO_PANCREAS_MESENCHY…	LTBP2	LOXL2	SNAI2	WNT5A	SPON2	DCN	IL11	INHBA	LGALS1	SERPINH1	CRLF1	TIMP1	NID1	NOTCH3	COL12A1	TIMP3	IGFBP4	FBN1	CALD1	MRC2	LAMA4	DKK3	PAPPA	FMOD	CRISPL…
CELLMARKERMSIGDB_RUBENSTEIN_SKELETAL_MUSC…	BCL2A1	FCGR1A	CYBB	JAML	CXCL8	TYROBP	RNASE6	RGS2	PILRA	APOBEC3A	CTSS	CALHM6	FCER1G	LY86	STXBP2	TNFSF13B	CD68	COTL1	CD48	CD83	MNDA	C1orf1…	PLAUR	TBXAS1	CD14
CELLMARKERMSIGDB_RUBENSTEIN_SKELETAL_MUSC…	RPS18	RPS3A	CADM2	RPL3	PON2	RPL6	RPL10A	PDLIM4	RASD1	RPL35A	RPL32	RPS6	RPL10	RPL26	RPL5	RPL39	PPP1R1…	RPS23	RPL12	RPS2	RPS27A	ALDH1A1	SGCA	RXRG	CNN3
CELLMARKERMSIGDB_RUBENSTEIN_SKELETAL_MUSC…	PPP1R12B	GUCY1A1	BGN	SLIT3	NET1	RGS16	SOD3	LMOD1	HACD1	COX4I2	EDNRA	GPRC5C	PIP5K1B	MYL9	KLHL23	ISYNA1	CKB	SNCG	MCAM	TBX2	RBPMS2	CRIP1	PRSS23	NTRK2	MAP3K20
CELLMARKERMSIGDB_TRAVAGLINI_LUNG_ALVEOLAR…	SEMA3B	ABCA1	MGLL	LRRN4	KLF6	SLC2A3	MYRF	MYL9	ROR1	SEMA5A	LAMA3	CLDN18	ANXA3	TNNC1	HEG1	CLIC3	PKDCC	MYL12A	KRT18	PHLDB2	ITLN2	CAV2	SLC39A8	GPM6A	PLLP
CELLMARKERMSIGDB_TRAVAGLINI_LUNG_BASOPHIL…	SLC18A2	SDCBP	SRGN	KIT	GLUL	CLU	MAOB	HPGD	TSC22D1	FOSB	CD69	CREM	LEO1	ID2	LTC4S	H3-3B	SIGLEC…	FCER1A	PLIN2	CD9	RGS1	GATA2	LMO4	SLC45A3	NR4A3
CELLMARKERMSIGDB_TRAVAGLINI_LUNG_CAPILLAR…	F3	GPX1	B3GALN…	RTN1	INMT	CD83	ADGRL2	TUBA1A	LGALS1	EXPH5	UBB	GPX3	VIPR1	EMP2	ITM2B	PRKCE	GNG11	RNF24							
CELLMARKERMSIGDB_TRAVAGLINI_LUNG_CAPILLAR…	NDRG2	FIS1	HNRNPA3	CCND1	COPS9	ATOX1	VAMP5	GMFG	MYZAP	IFI27	SAP18	RPS26	YWHAE	TRIR	NDUFB9	ATP5PF	COX7A1	TOMM7	ATP5MJ	NDUFB4	OST4	HMGB1	KIF5B	CCNI	NAP1L1
CELLMARKERMSIGDB_TRAVAGLINI_LUNG_CD4_MEMO…	LEF1	CNN2	TRAF3I…	CLEC2D	HLA-A	GSTK1	PBXIP1	SLAMF1	IFITM1	MIAT	BCL11B	SIT1	TMEM123	SARAF	RPL13A	ARHGAP15	CDC14A	SEPTIN1	SLFN5	GPR183	TRAT1	PRDM1	HSPA8	EVL	CTLA4
CELLMARKERMSIGDB_TRAVAGLINI_LUNG_EREG_DEN…	LYZ	CLEC10A	DSE	HLA-DPB1	TGFBI	TIMP1	HLA-DRA	CD14	ATP13A3	PPIF	NPC2	UPP1	CEBPB	CEBPD	ETS2	TLR2	HLA-DQ…	EMP1	CST3	CD300E	HLA-DRB1	FPR1	MRC1	IL1B	HLA-DQ…
CELLMARKERMSIGDB_TRAVAGLINI_LUNG_IGSF21_D…	HNMT	WLS	ATF3	MS4A7	CD83	PPIC	CPM	HSPA7	PDGFC	DAB2	FCGR2B	TGFBI	CD93	GGTA1	CSF1R	MAFB	LMNA	GATM	CXCL2	MS4A6A	CXCL16	ARHGAP…			
CELLMARKERMSIGDB_TRAVAGLINI_LUNG_MESOTHEL…	AADAC	ALDH1A3	PLA2G2A	WT1	DSC3	BNC1	MYRF	DAPK1	GALNT9	RPS12	DCBLD2	TIMP1	KRT18	LGALS2	HSD17B6	LHFPL2	RPL36	KLF5	MEIS2	RDH10	SLC39A8	SLC16A1	HK2	PRG4	HS2ST1
CELLMARKERMSIGDB_TRAVAGLINI_LUNG_MYOFIBRO…	ALDH2	PSTPIP1	COL12A1	CXCL14	LXN	LTBP2	ROBO1	WDR91	CCDC68	ANGPTL6	COL3A1	TGFBR3	DKK3	SLITRK6	CYSLTR1	MMP2	POSTN	ATP1B1	CREB5	ENC1	CCDC146	C16orf…	PCSK1N	PDGFC	GEM
CELLMARKERMSIGDB_TRAVAGLINI_LUNG_NATURAL_…	CRTAM	XIST	CXCR3	KRT81	ITGA1	MYL12A	GZMB	CD7	RGS1	GATA3															
CELLMARKERMSIGDB_TRAVAGLINI_LUNG_NEUTROPH…	CDA	NCF1C	CMTM2	MMP9	AQP9	C5AR1	SRGN	ABTB1	CEBPB	S100A12	FTH1P10	TREM1	MNDA	TRIB1	CLEC4E	ITGAX	FFAR2	GCA	RNF149	LRG1	FCGR2A	PCBP1-…	ALPL	ZDHHC18	PGLYRP1
CELLMARKERMSIGDB_TRAVAGLINI_LUNG_OLR1_CLA…	SLC25A37	NINJ1	CD300E	TNIP1	WTAP	SAT1	S100A9	TLR2	SRGN	SLC39A8	CD163	PNRC1	RIN2	DSE	VCAN	RNF19B	CEBPB	S100A8	FCN1	EMP1	CXCL8	PFKFB3	TNFRSF1B	ETS2	ANPEP
CELLMARKERMSIGDB_TRAVAGLINI_LUNG_PLATELET…	TUBA4A	MARCHF2	GP9	MAP3K7CL	CTTN	PTCRA	TPM4	TMEM40	TREML1	GAS2L1	GPX1	MYL9	CMTM5	CXCR2P1	C2orf88	ACTN1	DMTN	ESAM	PRKAR2B	PLA2G12A	BEX3	ITGA2B	RAB11A	SNCA	TLN1
CELLMARKERMSIGDB_TRAVAGLINI_LUNG_PROLIFER…	SGO1	CCNA2	PRC1	TPX2	DEPDC1	FOXM1	RAD51AP1	CDCA5	TYMS	ANLN	CCNB2	H1-5	CENPA	CDC20	CKS1B	TACC3	KIF20A	CENPU	HMGB1	SPC25	CEP55	RRM2	CKAP2L	NDC80	MAD2L1
CELLMARKERMSIGDB_TRAVAGLINI_LUNG_PROLIFER…	CDKN3	PRC1	CKAP2L	GRN	CENPK	TPX2	ASF1B	MND1	GTSE1	RRM2	NUF2	KIFC1	HP	CENPM	SHCBP1	CENPF	SPC25	CENPN	MELK	CLSPN	CKS1B	CCNA2	APOC1	VSIG4	C1QB
CELLMARKERMSIGDB_TRAVAGLINI_LUNG_PROXIMAL…	PERP	EEF1G	SERPIN…	ADH1C	SERPIN…	POSTN	PABPC1	RPSA	RPS2	LGALS7B	RPL3	RPL15	RPL4	SERPIN…	RPS7	RPL28	CHST9	GAS5	SLC25A6	RPLP0	RPL7A	RPLP1	GPR87	KRT17	RASSF6
CELLMARKERMSIGDB_TRAVAGLINI_LUNG_SEROUS_C…	PPP1R1B	TTYH1	ODAM	PHLDA1	BPIFB1	CCL28	PIGR	KCNN4	MARCKSL1	SSR4	GATM	PLTP	SERPIN…	PRB2	BPIFA1	PADI2	SLC13A2	DEPTOR	WFDC2	FAM3D	MUC7	BARX2	SMR3A	TESC	PKDCC
CELLMARKERMSIGDB_TRAVAGLINI_LUNG_SIGNALIN…	MBIP	FBP1	SFTPC	SLC34A2	LAMP3	MTUS1	FABP5	DLC1	GKN2	NINJ2	HOPX	CD74	DRAM1	SNX30	KCNJ15	ROS1	S100A14	ZNF385B	SELENB…	CRTAC1	CXCL17	RFTN1	ATP8A1	PCDH9	LMO3
CELLMARKERMSIGDB_TRAVAGLINI_LUNG_TREM2_DE…	C15orf48	CYP27A1	FBP1	TXN	PLA2G7	HLA-DQ…	FABP3	MRC1	GPX3	CTSH	C1QC	C1QB	SLAMF8	ALDH2	FN1	FPR3	FTL	KCNMA1	TMEM17…	MMP9	SERPINF1	TMEM17…	C1QA	HLA-DMA	PSAP
CELLMARKERMSIGDB_TRAVAGLINI_LUNG_VASCULAR…	EZR	ITGA1	TPM2	MYH9	PPP1R1…	IGFBP7	FHL5	LGI4	JAG1	SEMA5A	ARHGDIB	CAV1	CDH6	TPM1	ISYNA1	HSPA2	LMOD1	MYL6	ACTN1	SOD3	PARM1	LPP	INPP4B	RCAN2	PDLIM7
CELLMARKERMSIGDB_TRAVAGLINI_LUNG_VEIN_CELL	SELE	SLCO2A1	ABI3BP	PRCP	CADM3-…	ADGRG6	TIMP1	PTGDS	MMRN1	ADAMTS9	UPP1	CSF3	ICAM1	ADAMTS4	LYST	TNFAIP3									
CELLMARKERMSIGDB_VANGURP_PANCREATIC_ALPHA…	PALLD	PAPPA2	PCSK2	PEMT	PLCE1	PLIN3	RGS4	RNASEK	SLC7A2	SMIM24	SPINT2	TM4SF4	TMEM17…	TMEM17…	TTR	VGF									
CELLMARKERMSIGDB_VANGURP_PANCREATIC_DELTA…	ERBB4	ETV1	EYS	F5	FAM102A	FFAR4	FRZB	GABRB3	GPX3	HADH	HHEX	ISL1	LDHA	LEPR	LRFN5	MDK	MLPH	MS4A8	MTUS1	NCOA7	NDRG4	NLRP1	NPTX2	OPRD1	PAPPA2
CELLMARKERMSIGDB_VANGURP_PANCREATIC_GAMMA…	PAX6	PEG10	PPY	PTP4A3	PXK	S100A10	SCG2	SCGB2A1	SEMA3E	SERTM1	SLC4A4	SLC6A4	SLITRK6	SPINK1	SPOCK1	STMN2	THSD7A	TM4SF4	TMEM17…	TMEM47	TXNIP				
CELLMARKERMSIGDB_ZHENG_CORD_BLOOD_C1_PUTA…	CXCL3	DAAM1	DMTN	DNM3	EFNB2	ESAM	EXOC3L2	F13A1	FCGR2A	FERMT3	FYB1	GABRE	GFI1B	GNAZ	GP1BA	GP6	GP9	GRAP2	GSN	HBD	HEXIM1	HPSE	ITGA2B	ITGA6	KALRN
CELLMARKERMSIGDB_ZHENG_CORD_BLOOD_C10_MUL…	COL24A1	CORO1A	CXXC5	CYFIP2	CYTH1	CYTH4	DDX17	DSTN	EVL	FGD6	GFOD1	GSTP1	HCST	IGHM	JCHAIN	IL6R	IQGAP2	IRF8	ITGAL	ITM2C	KCNK17	KIAA00…	FAM30A	LAIR1	LINC00…
CELLMARKERMSIGDB_ZHENG_CORD_BLOOD_C2_PUTA…	FLNA	FOSB	GATA1	GATA2	GDE1	GLUL	GOLT1B	GRAP2	GSE1	GTF3C4	HDC	HMGCS1	HPGDS	HSP90A…	HSPA5	IFITM2	IKZF2	ING2	JUND	LAPTM5	LDB1	LGALS1	LMNA	LMO4	LYAR
CELLMARKERMSIGDB_ZHENG_CORD_BLOOD_C4_PUTA…	ETS2	EWSR1	RIPOR3	FOXO3	G3BP2	GALNT1	GATA2	GNAI1	HPGDS	HSP90AA1	HTR1F	IKZF2	IL1RL1	IMPA1	ITGA4	ITGAE	JUN	CRACD	KIT	LAPTM4A	LDLR	LEPROT	MAN2A2	MED12L	MLLT3
CELLMARKERMSIGDB_ZHENG_CORD_BLOOD_C5_SIMI…	H2AC8	RNU2-4…	HSPA5	H2BC21	RNU5D-1	JUN	TMEM107	RNU5A-…	H2BC7	AVP	JUNB	CD69	RNU5B-1	C12orf…	NEAT1	MEG3	H1-2	CLEC2B	H2BC4	TUBB4B	NFKBIA	NFKBIZ	SOCS2	MEF2C	H1-10
CELLMARKERMSIGDB_ZHENG_CORD_BLOOD_C6_HSC_…	HIF1A	HLA-DMB	HLA-DP…	HLA-DPB1	HLA-DQ…	HLA-DQ…	HLA-DRA	HLA-DR…	HLA-DRB5	HLA-E	HLF	HMGA2	ID2	IRAK3	IRF1	KAT6A	LIMCH1	LPAR6	LRRFIP1	MAFF	MAFG	MAP3K8	MECOM	MEF2C	MEG3
CELLMARKERMSIGDB_ZHENG_CORD_BLOOD_C7_PUTA…	HSP90B1	MSI2	ATG10	MACROH2A1	PTPRE	ANKRD3…	TTF1	RILPL2	LRRFIP1	SPINK2	MZB1	GBP4	BCL11A	ANKRD36	IFITM1	MT-ND5	C1QTNF4	KRR1	GLIPR1	ZC3HAV1	TTC3	RUNX1	GNB4	ZBTB8A	EIF2S3
CELLMARKERMSIGDB_ZHENG_CORD_BLOOD_C8_PUTA…	ACTG1	RILPL2	SELL	PTPRCAP	SNHG6	IGLL1	MT-TL1	HINT1	CD44	RCSD1	FAU	HLA-B	SNHG8	IFITM3	GAS5	PIK3R1	BTF3	CD34	GNG11	PFDN5	ARHGAP25	ENO1	MPG	S100Z	HSH2D
CELLMARKERMSIGDB_ZHONG_PFC_C1_ASTROCYTE	ZNF302	ADD3	DHCR7	HMGN3	S1PR1	SLC9A3…																			
CELLMARKERMSIGDB_ZHONG_PFC_C1_DLX5_POS_IN…																									
CELLMARKERMSIGDB_ZHONG_PFC_C2_ASCL1_POS_A…																									

Table S5: Cell state marker gene list

CELLMARKERCAN_ACINAR_CELLS	PNLIPRP1	CTRC	REG3A	SERPINA3	PRSS3	REG1B	CFB	GDF15	MUC1	C15ORF48	DUOXA2	AKR1C3	OLFM4	GSTA1	LGALS2	PDZK1IP1	RARRES2	CXCL17	UBD	GSTA2	ANPEP	LYZ	ANGPTL4	ALDOB	
CELLMARKERMSIGDB_ZHONG_PFC_C2_UNKNOWN_NPC	DEPDC1	DLGAP5	DNALI1	FAM83D	G2E3	GTSE1	HJURP	ITCH	KDM4A	KIF20A	KIF22	KIF2C	KIF4A	KNSTRN	KPNA2	LDHA	ENSG00…	MFAP2	MIS18B…	MKI67	MZT1	NCAPD2	NDC80	NDE1	NDST4
CELLMARKERMSIGDB_ZHONG_PFC_C3_ASTROCYTE	CELF6	PRKCB	SNAPC4	FGF9	C2CD4C	LIMS2	OSBP2	VSTM2B	ARFGEF3	ZNF316	PDE1C	SLCO3A1	COL18A1	DPYD	GNAL	CNTN4	SPHKAP	GCN1	ANKRD44	ACAP3	TCAF1	ABCC5	STXBP6	BIN1	SLC7A5…
CELLMARKERMSIGDB_ZHONG_PFC_C3_HOPX_POS_OPC																									
CELLMARKERMSIGDB_ZHONG_PFC_C3_MICROGLIA	ZBTB18	NCOA1	BAALC	FABP3	KIF21B	BOK	NPDC1	SHC2	SPTAN1	NEO1	TNKS2	OPCML	AK1	MAPRE3	FABP7	PTPRK	FAM110B	CTXN1	C12orf…	NBEA	PPP5C	ZSCAN29	KRT31	DCLK2	MAP6
CELLMARKERMSIGDB_ZHONG_PFC_C3_UNKNOWN_INP	NCAPH	NDC80	NEUROD4	NMU	NUF2	PENK	PPP1R17	PRC1	SGO1	SKA1	SPC25	TPX2	UBE2C	UBE2T											
CELLMARKERMSIGDB_ZHONG_PFC_C4_UNKNOWN_INP	EIF2S1	ELAVL4	ELP6	EOMES	EXOSC1	GADD45G	GMIP	GNL3	HSPA1A	HSPA1B	HSPA5	INO80C	KIN	KLHDC8A	LDB1	LINC02487	MEST	MFNG	MPHOSP…	MRPL42	MRTO4	MTHFD2	NELL2	NEUROG1	NFATC3
CELLMARKERMSIGDB_ZHONG_PFC_C6_PLXNA4_POS_…																									
CELLMARKERMSIGDB_ZHONG_PFC_C7_ORG_UNDERGO…	GLRA2	GRIA2	GUSBP11	HSPA12A	IQGAP2	KRT31	LEMD2	LHFPL4	MIR124-…	LNX1	LONRF2	LRRC8B	MAP6	MAPT	MEF2C	MEGF8	MIAT	MLLT3	AFDN-DT	MNT	MTURN	MYT1L	NDRG1	NEUROD2	NIN
CELLMARKERMSIGDB_ZHONG_PFC_C8_ORG_PROLIFE…	DEPDC1B	EMP3	GAS2L3	HSD17B11	ITGB1B…	KIF18A	KIF20A	KNSTRN	LGALS1	MT2A	MZT1	NEK2	NMU	NOTCH2…	NUSAP1	ODF2	OIP5	PLIN3	PLK1	PRC1	PRR11	PSRC1	PTTG1	RACGAP1	RP2
CELLMARKERMSIGDB_ZHONG_PFC_C8_UNKNOWN_NEU…	DNAJB2	DOCK9	DOK5	DTX1	DTX4	DUSP23	EEF1A2	EPHB6	FABP3	CYRIA	FASN	FBXW7	FOXP1	GAP43	HIVEP2	IGSF21	JAG2	KBTBD11	KHDRBS3	LHX2	LPL	PLPPR1	MAP6	MAPK11	MAPK8I…
CELLMARKERMSIGDB_ZHONG_PFC_C9_ORG_OTHER	GNG12	GPM6B	GPX3	HOPX	ID2	IFI27L2	ITGB8	ITM2C	MYORG	LGALS3	LIFR	LINC009…	LITAF	LRRC3B	MEST	MFGE8	MGST3	MLC1	MOXD1	MT3	NDN	NDP	NDRG2	NFE2L2	NOTCH2
CELLMARKERMSIGDB_ZHONG_PFC_HES1_POS_C1_NPC																									
CELLMARKERMSIGDB_ZHONG_PFC_MAJOR_TYPES_AS…	TSC22D4	RAMP1	BCAN	ID1	SLC1A3	EDNRB	PLPP3	PTN	ADORA2B	TTYH1	SPON1	F3	KCNE5	LYN	RAB31	FBLN2	PSAT1	DIO2	HES5	LINC009…	SLC4A4	SLC15A2	GDPD2	SLC1A2	CCDC80
CELLMARKERMSIGDB_ZHONG_PFC_MAJOR_TYPES_EX…																									
CELLMARKERMSIGDB_ZHONG_PFC_MAJOR_TYPES_MI…	TMEM52B	RGS10	FCGR1A	CH25H	FPR1	IGSF6	C1QC	ALOX5AP	SLCO2B1	LY86	VSIR	OLFML3	A2M	ADAP2	ARHGDIB	GPR34	SRGN	LST1	ITGB2	S100A11	PTAFR	CD68	LINC027…	ZFP36	APOC1
CELLMARKERMSIGDB_ZHONG_PFC_MAJOR_TYPES_OPC	VXN	SIRT2	CD9	NKAIN4	DUSP6	EDNRB	KANK1	SEMA5A	LIMA1	LUZP2	RAB31	EPN2	SOX8	SPRY4	BRINP3	CNP	SPATA6	PLEKHB1	LHFPL3	SLITRK2	GRID2	TSC22D4	C2orf80	EPB41L2	SCN1A

CELLMARKERCAN_ACINAR_CELLS																									
CELLMARKERCAN_ADIPOCYTE_PROGENITOR_CELLS																									
CELLMARKERCAN_ADRENERGIC_NEURONS																									
CELLMARKERCAN_AIRWAY_EPITHELIAL_CELLS																									
CELLMARKERCAN_ALPHA_CELLS																									
CELLMARKERCAN_ALVEOLAR_MACROPHAGES																									
CELLMARKERCAN_ASTROCYTES	VIM	CCR7	AANAT	FABP7	SOCS3	SYNM	HMG20A	NFATC3	SRR	SLC1A6	ETS1	SLIT1	SNPH	IL23A											
CELLMARKERCAN_B_CELLS																									
CELLMARKERCAN_B_CELLS_MEMORY	NPIPB15	P2RX5	PNOC	PTPRCAP	RALGPS2	RASGRP2	SIK1	SIT1	SLC12A1	SP140	STAP1	TMEM156	TNFRSF13B	TNFRSF17	TRAF4	VPREB3	ZBTB32								
CELLMARKERCAN_B_CELLS_NAIVE	SIK1	SLC12A1	SPIB	STAP1	TCL1A	UGT1A8	VPREB3	ZNF286A	TNFSF8	FOXP1	MITF	CD52	HLA-DQB1	TNFRSF13C	LINC00926	STAG3	BIRC3	CD74							
CELLMARKERCAN_BASOPHILS	LAMA5	MBOAT1	NFIL3	NPL	OSBPL5	OTUB2	P2RY1	PDK3	PDZD4	PERP	PIGY	PLEKHG5	PLG	RNF39	SLC26A3	SLC26A8	SNCAIP	ST6GALNAC5	SUPT3H	TULP3	TEC	UNC93A	WRN	ID1	
CELLMARKERCAN_BERGMANN_GLIA																									
CELLMARKERCAN_BETA_CELLS	MEG3	RGS16																							
CELLMARKERCAN_CAJAL_RETZIUS_CELLS																									
CELLMARKERCAN_CARDIAC_STEM_AND_PRECURSOR_…																									
CELLMARKERCAN_CARDIOMYOCYTES	MYOZ2	ENO3	TRDN	ACTN2	TNNI1	SMPX	MYL7	TBX20	TRIM63	MB	CTGF	MYL4	LOX	CTNNB1	CPT1A	SOX18	NOTCH1	SLN	CSRP3	TXLNB	ATP2A2	CKM	CKB	CXCR4	IL11RA
CELLMARKERCAN_CHOLANGIOCYTES																									
CELLMARKERCAN_CHOLINERGIC_NEURONS																									
CELLMARKERCAN_CHONDROCYTES																									
CELLMARKERCAN_CHOROID_PLEXUS_CELLS																									
CELLMARKERCAN_CILIATED_CELLS																									
CELLMARKERCAN_DELTA_CELLS																									
CELLMARKERCAN_DOPAMINERGIC_NEURONS																									
CELLMARKERCAN_DUCTAL_CELLS																									
CELLMARKERCAN_EMBRYONIC_STEM_CELLS	SMAD9	SMAD4	SMAD2	SMAD3	CTNNB1	HHEX	HES1	ESRRB	DNMT3B	TRIM28	PIWIL1	PIWIL2	PIWIL4	LIFR	IL6ST										
CELLMARKERCAN_ENTERIC_GLIA_CELLS																									
CELLMARKERCAN_ENTERIC_NEURONS																									
CELLMARKERCAN_ENTEROCYTES	MEP1A	FABP6	RBP2	CYP3A4	APOB	MTTP	SLC2A2	APOC3	UGT2B17	CD36	REG3A	ISX	AQP10	S100G	BEST4	SLC28A1	SLC28A2	SLC30A10	SLC51B	VNN1	SLC23A1	TREH	ABCC2	ABCG5	CYP3A7
CELLMARKERCAN_ENTEROENDOCRINE_CELLS																									
CELLMARKERCAN_EPENDYMAL_CELLS																									
CELLMARKERCAN_EPSILON_CELLS																									
CELLMARKERCAN_FOLLICULAR_CELLS																									
CELLMARKERCAN_FOVEOLAR_CELLS																									
CELLMARKERCAN_GAMMA_DELTA_T_CELLS																									
CELLMARKERCAN_GERM_CELLS																									
CELLMARKERCAN_GLUTAMINERGIC_NEURONS																									
CELLMARKERCAN_GRANULOSA_CELLS																									
CELLMARKERCAN_HEMATOPOIETIC_STEM_CELLS	GNL3	MGST1	G0S2	PRNP	CD34	BMI1	CD59	ITGA4	ANPEP	CD33	IL3RA	HLX	NKX3-1	THSD1	NCOR2	EGR1	FGD5	MECOM	CTNNAL1	VWF	PBX2	TAL1	ERG	NEO1	EGR3
CELLMARKERCAN_IMMATURE_NEURONS																									
CELLMARKERCAN_INTERNEURONS	NDNF																								
CELLMARKERCAN_IONOCYTES																									
CELLMARKERCAN_KERATINOCYTES																									
CELLMARKERCAN_KUPFFER_CELLS																									
CELLMARKERCAN_LEYDIG_CELLS																									
CELLMARKERCAN_LOOP_OF_HENLE_CELLS																									
CELLMARKERCAN_LUMINAL_EPITHELIAL_CELLS																									
CELLMARKERCAN_LUTEAL_CELLS																									
CELLMARKERCAN_MEGAKARYOCYTES																									
CELLMARKERCAN_MELANOCYTES																									
CELLMARKERCAN_MESANGIAL_CELLS																									
CELLMARKERCAN_MESOTHELIAL_CELLS																									
CELLMARKERCAN_MICROFOLD_CELLS																									
CELLMARKERCAN_MICROGLIA	CD53	FOS	EGR1	AIF1	CTSS	CSF1R	SKI	C1QB																	
CELLMARKERCAN_MONOCYTES	MNDA	MS4A6A	CLEC12A	CD48	PRTN3	FCGR3A	VCAN	IFITM3	FN1	ADGRE1	CD44	CSF1R	CX3CR1	ITGAL	PECAM1	PTPRC	SPN	PSAP	FCN1						
CELLMARKERCAN_MOTOR_NEURONS																									
CELLMARKERCAN_MULLER_CELLS																									
CELLMARKERCAN_MYELOID_DERIVED_SUPPRESSOR_…																									
CELLMARKERCAN_MYOBLASTS																									
CELLMARKERCAN_MYOEPITHELIAL_CELLS																									
CELLMARKERCAN_MYOFIBROBLASTS																									
CELLMARKERCAN_NEUROBLASTS	CNR1																								
CELLMARKERCAN_NEUROENDOCRINE_CELLS																									
CELLMARKERCAN_NEURONS	CHODL	NECAB2	KCNMA1	SLC17A7	PPP1R1B	PCP4	MEG3	BEX2	BEX1	NDN	CAMK2N1	PNISR	SNHG11	STMN2	PTK2B	DDN	ICAM5	GNG2	SNAP25	VSNL1	PACSIN1	CACNA2D1	SGIP1	ATP6V0A1	MYO5A
CELLMARKERCAN_NEUTROPHILS	ADAM8	CD177	PTGS2	PRTN3	CTSG	SERPINB1	SLC1A5	SNX20	ADPGK	PSTPIP1	LYST	S100A4	NLRP3	CSF3R											
CELLMARKERCAN_NK_CELLS	XCL2	CXCR4	IL18R1	SERPINB9	DOCK2	SH2D2A	S100A4	XCL1	GZMK	IFNG	CCL3	CSF2	IL2RG	TGFB1	KIR2DL1	KIR3DL1	LILRB1	KLRG1	NCR3	ADAMTS14	ITGA2	STYK1	CLEC2D	CD247	ZBTB16
CELLMARKERCAN_NORADRENERGIC_NEURONS																									
CELLMARKERCAN_NUOCYTES																									
CELLMARKERCAN_OLIGODENDROCYTES	POU3F1	NINJ2	SGK2	GJC3	GPR37	IL33	KLK6	NFE2L3	PEX5L	CLDN14	ITGB4	MYO1D	PDE8A	CNTF	EML1	KIF13B	KIF6	NIPAL4	SEC14L5	SP7	ST18	DUSP15	PRKCZ	ARSG	NPC1
CELLMARKERCAN_OSTEOBLASTS	CALB1	COL11A1	COL11A2	IBSP	POSTN	SFRP4	NA	COL12A1	WISP2	SP7	PENK	NPNT	OLFML1												
CELLMARKERCAN_OSTEOCLAST_PRECURSOR_CELLS																									
CELLMARKERCAN_OSTEOCLASTS																									
CELLMARKERCAN_OSTEOCYTES																									
CELLMARKERCAN_PANETH_CELLS																									
CELLMARKERCAN_PARATHYROID_CHIEF_CELLS																									
CELLMARKERCAN_PERICYTES																									
CELLMARKERCAN_PERITUBULAR_MYOID_CELLS																									
CELLMARKERCAN_PHOTORECEPTOR_CELLS																									
CELLMARKERCAN_PLASMA_CELLS	MAN1A1	MANEA	MAST1	MROH7	MZB1	P2RX5	PAX7	PDK1	PNOC	RASGRP3	REN	RGS13	RPL3P7	SIK1	SPAG4	ST6GALNAC4	TGM5	TMEM156	UGT2B17	ZBP1	ZNF165	IGLC2	IGHG1	IGHG2	IGHG3
CELLMARKERCAN_PLASMACYTOID_DENDRITIC_CELLS	IRF5	BST2																							
CELLMARKERCAN_PODOCYTES	NEBL	SH2D4A	TDRD5	PTH1R	IQGAP2	CTGF	FAT1	FOXD1	TRIB2	LOXL2	COL4A5	FOXC1	PLEKHH2	CTNNAL1	DACH1	SULF1	HSPB8	KANK1	ADCY1	DOCK5	CRB2	LRRC49	CORO2B	ARHGAP24	GPRC5A
CELLMARKERCAN_PROXIMAL_TUBULE_CELLS	SCIN	ALPL	G6PC	SLC22A13	SLC22A7	SLC16A9	AGXT2	SORD	SOD3	HNF4A	NME4	APOE	HGD	OSGIN1	HSD11B1	SERPINF2	HAAO	PPIC	TFF3	GNG13	SLC23A3	GC	CACNG5	TGFBI	RBP4
CELLMARKERCAN_PULMONARY_ALVEOLAR_TYPE_I_C…																									
CELLMARKERCAN_PULMONARY_ALVEOLAR_TYPE_II_…																									
CELLMARKERCAN_PURKINJE_NEURONS																									
CELLMARKERCAN_RETICULOCYTES																									
CELLMARKERCAN_RETINAL_GANGLION_CELLS																									
CELLMARKERCAN_RETINAL_PROGENITOR_CELLS																									
CELLMARKERCAN_SATELLITE_CELLS																									
CELLMARKERCAN_SCHWANN_CELLS																									
CELLMARKERCAN_SEROTONERGIC_NEURONS																									
CELLMARKERCAN_SERTOLI_CELLS	SERPINB9	SERPING1	CD55	CD46	CLU	FSHR	RXRB	PRLR	SLC16A3	EPS8	NR1H2	BCAR3	SOX9												
CELLMARKERCAN_SMOOTH_MUSCLE_CELLS	AAED1	AOC3	SEC24D	KCNAB1	MYLK4	KIF1C	MRVI1	JPH2	ITGA9	KCNMB1	NOX4	NOV	DMPK	SH3BGR	ITGA8	TCF21	ADAMDEC1	HHIP	MFAP5	SPON2	LAMB2	GJC1	ACKR3	OGN	SMTN
CELLMARKERCAN_STROMAL_CELLS																									
CELLMARKERCAN_T_CELLS	BRAF																								
CELLMARKERCAN_T_CYTOTOXIC_CELLS																									
CELLMARKERCAN_T_FOLLICULAR_HELPER_CELLS																									
CELLMARKERCAN_T_MEMORY_CELLS																									
CELLMARKERCAN_TRANSIENT_CELLS																									
CELLMARKERCAN_TRICHOCYTES																									
CELLMARKERCAN_TRIGEMINAL_NEURONS																									
CELLMARKERCAN_TROPHOBLAST_CELLS																									
CELLMARKERCAN_TROPHOBLAST_PROGENITOR_CELLS																									
CELLMARKERCAN_TUFT_CELLS																									
CELLMARKERCANENDOTHELIALCELLSAORTA																									
CELLMARKERCUR_N_ABNORMAL_MYELOID_CELL																									

Table S5: Cell state marker gene list

CELLMARKERCAN_ACINAR_CELLS																									
CELLMARKERCUR_N_ABNORMAL_PLASMA_CELL																									
CELLMARKERCUR_N_ACTIVATED_DENDRITIC_CELL																									
CELLMARKERCUR_N_ACTIVATED_EFFECTOR_CELL																									
CELLMARKERCUR_N_ACTIVATED_HEPATIC_STELLAT…																									
CELLMARKERCUR_N_ACTIVATED_LANGERHANS_CELL																									
CELLMARKERCUR_N_ACTIVATED_MACROPHAGE																									
CELLMARKERCUR_N_ACTIVATED_MEMORY_T_CELL																									
CELLMARKERCUR_N_ACTIVATED_NATURAL_KILLER_…																									
CELLMARKERCUR_N_ACTIVATED_REGULATORY_T_CE…																									
CELLMARKERCUR_N_ADIPOCYTE_PROGENITOR_CELL																									
CELLMARKERCUR_N_ADRENAL_MEDULLA_CELL																									
CELLMARKERCUR_N_ADULT_SPERMATOGONIA																									
CELLMARKERCUR_N_ADVENTITIAL_CELL																									
CELLMARKERCUR_N_AEROCYTE																									
CELLMARKERCUR_N_AIRWAY_SMOOTH_MUSCLE_CELL																									
CELLMARKERCUR_N_ALVEOLAR_CELL_TYPE_1																									
CELLMARKERCUR_N_ALVEOLAR_CELL_TYPE_2																									
CELLMARKERCUR_N_ALVEOLAR_EPITHELIAL_CELL_…																									
CELLMARKERCUR_N_ALVEOLAR_EPITHELIAL_CELL_…																									
CELLMARKERCUR_N_ALVEOLAR_PNEUMOCYTE_TYPE_I																									
CELLMARKERCUR_N_ALVEOLAR_PNEUMOCYTE_TYPE_…																									
CELLMARKERCUR_N_AMACRINE_CELL																									
CELLMARKERCUR_N_AMNIOTIC_FLUID_STEM_CELL																									
CELLMARKERCUR_N_ANTERIOR_CELL																									
CELLMARKERCUR_N_ANTERIOR_HINDBRAIN_CELL																									
CELLMARKERCUR_N_ANTI_TUMOR_IMMUNE_CELL																									
CELLMARKERCUR_N_ANTIGEN_PRESENTATION_CANC…																									
CELLMARKERCUR_N_ASTROCYTE	CLU	LAMB2	HSP90A…	PTN	GPC5	HSPB1	TNC	CADM2	NRXN1	MBP	ATP1A2	ATP1B2	MT3	PON2	HEPN1	HEPACAM	ACSBG1	SLC25A18	RFX4	CHRDL1	SLC39A…	PPP1R3G	F3	GJB6	SLC15…
CELLMARKERCUR_N_ATRIAL_CELL																									
CELLMARKERCUR_N_ATRIOVENTRICULAR_CANAL_CE…																									
CELLMARKERCUR_N_ATYPICAL_MEMORY_B_CELL																									
CELLMARKERCUR_N_B_2_CELL																									
CELLMARKERCUR_N_B1_CELL																									
CELLMARKERCUR_N_B10_REGULATORY_B_CELL																									
CELLMARKERCUR_N_BASAL_CELL	IVL																								
CELLMARKERCUR_N_BASAL_EPITHELIAL_CELL																									
CELLMARKERCUR_N_BASAL_KERATINOCYTE																									
CELLMARKERCUR_N_BASAL_LIKE_CELL																									
CELLMARKERCUR_N_BASAL_PROGENITOR_CELL																									
CELLMARKERCUR_N_BASOPHIL																									
CELLMARKERCUR_N_BILIARY_CELL																									
CELLMARKERCUR_N_BONE_MARROW_STEM_CELL																									
CELLMARKERCUR_N_BONE_SPECIFIC_CELL																									
CELLMARKERCUR_N_BRAIN_CELL																									
CELLMARKERCUR_N_CANCER_CELL	PCSK9	ZIC1	PDE1C	PAX6	IFITM1	CD5	HJURP	SLAMF7	TNFRSF…	CD38	SDC1	MBTPS2	PARP1	EGFR	BRAF	KRAS	NF2	TERT	IDH1	PIK3R1	NRAS	PTEN	DLX1	GFAP	VIM
CELLMARKERCUR_N_CANCER_STEM_LIKE_CELL																									
CELLMARKERCUR_N_CAPILLARY_CELL																									
CELLMARKERCUR_N_CAR_T_CELL																									
CELLMARKERCUR_N_CARDIAC_CELL																									
CELLMARKERCUR_N_CARDIOMYOCYTE																									
CELLMARKERCUR_N_CARDIOSPHERE_DERIVED_CELL																									
CELLMARKERCUR_N_CARDIOVASCULAR_PROGENITOR…																									
CELLMARKERCUR_N_CAUDAL_CORTEX_CELL																									
CELLMARKERCUR_N_CD14_MONOCYTE																									
CELLMARKERCUR_N_CD16_MONOCYTE																									
CELLMARKERCUR_N_CD4_CD28_T_CELL																									
CELLMARKERCUR_N_CD4_PERIPHERALLY_DERIVED_…																									
CELLMARKERCUR_N_CD4_T_CELL																									
CELLMARKERCUR_N_CD56BRIGHT_NATURAL_KILLER…																									
CELLMARKERCUR_N_CD56DIM_NATURAL_KILLER_CE…																									
CELLMARKERCUR_N_CD8_T_CELL																									
CELLMARKERCUR_N_CENTRAL_MEMORY_T_CELL																									
CELLMARKERCUR_N_CENTRAL_MEMORY_T_HELPER_1…																									
CELLMARKERCUR_N_CENTRAL_NERVOUS_SYSTEM_ST…																									
CELLMARKERCUR_N_CHANDELIER_CELL																									
CELLMARKERCUR_N_CHIEF_CELL																									
CELLMARKERCUR_N_CHONDROCYTE																									
CELLMARKERCUR_N_CHROMAFFIN_CELL																									
CELLMARKERCUR_N_CIRCULATING_ANGIOGENIC_CE…																									
CELLMARKERCUR_N_CIRCULATING_FETAL_CELL																									
CELLMARKERCUR_N_CIRCULATING_NATURAL_KILLE…																									
CELLMARKERCUR_N_CIRCULATING_PRECURSOR_CELL																									
CELLMARKERCUR_N_CIRCULATING_PROGENITOR_CE…																									
CELLMARKERCUR_N_CIRCULATING_T_FOLLICULAR_…																									
CELLMARKERCUR_N_CIRCULATING_TUMOR_CELL																									
CELLMARKERCUR_N_CLASS_SWITCHED_PLASMABLAST																									
CELLMARKERCUR_N_CLASSICAL_B_CELL																									
CELLMARKERCUR_N_CLASSICAL_CANCER_ASSOCIAT…																									
CELLMARKERCUR_N_CLASSICAL_MONOCYTE																									
CELLMARKERCUR_N_CLAUDIN_LOW_CELL																									
CELLMARKERCUR_N_COLLECTING_DUCTAL_CELL																									
CELLMARKERCUR_N_COLONCYTE																									
CELLMARKERCUR_N_COLORECTAL_STEM_CELL																									
CELLMARKERCUR_N_COLUMNAR_EPITHELIAL_CELL																									
CELLMARKERCUR_N_COMMON_LYMPHOID_PROGENITO…																									
CELLMARKERCUR_N_COMMON_MYELOID_PROGENITOR…																									
CELLMARKERCUR_N_COMPLEMENT_SECRETING_CANC…																									
CELLMARKERCUR_N_CONE_PHOTORECEPTOR_CELL																									
CELLMARKERCUR_N_CONNECTING_TUBULAR_CELL																									
CELLMARKERCUR_N_CONVENTIONAL_CD4_T_CELL																									
CELLMARKERCUR_N_CORNEAL_ENDOTHELIAL_CELL																									
CELLMARKERCUR_N_CORNEAL_EPITHELIAL_CELL																									
CELLMARKERCUR_N_CORNEOCYTE_LIKE_CELL																									
CELLMARKERCUR_N_CORTICAL_CELL																									
CELLMARKERCUR_N_CORTICAL_STROMAL_CELL																									
CELLMARKERCUR_N_CORTICOTROPH_TUMOR_CELL																									
CELLMARKERCUR_N_CRANIAL_NEURAL_CREST_CELL																									
CELLMARKERCUR_N_CRYPT_CELL																									
CELLMARKERCUR_N_CRYPT_STEM_CELL																									
CELLMARKERCUR_N_CYCLING_B_CELL																									
CELLMARKERCUR_N_CYCLING_EPITHELIAL_CELL																									
CELLMARKERCUR_N_CYTOTOXIC_CD8_T_CELL																									
CELLMARKERCUR_N_CYTOTOXIC_DOUBLE_POSITIVE…																									
CELLMARKERCUR_N_CYTOTOXIC_T_CELL																									

CELLMARKERCAN_ACINAR_CELLS																									
CELLMARKERCUR_N_CYTOTOXIC_T_CELL_2																									
CELLMARKERCUR_N_DAMAGE_ASSOCIATED_TRANSIE…																									
CELLMARKERCUR_N_DARK_ZONE_CELL																									
CELLMARKERCUR_N_DEDIFFERENTIATION_ADIPOCY…																									
CELLMARKERCUR_N_DEEP_LAYER_NEURON																									
CELLMARKERCUR_N_DEFINITIVE_ENDODERM_CELL																									
CELLMARKERCUR_N_DELTA_CELL																									
CELLMARKERCUR_N_DEMYELINATING_MICROGLIAL_…																									
CELLMARKERCUR_N_DENDRITIC_CELL_2A																									
CELLMARKERCUR_N_DENDRITIC_CELL_LINEAGE																									
CELLMARKERCUR_N_DENTAL_PULP_CELL																									
CELLMARKERCUR_N_DERMAL_CELL																									
CELLMARKERCUR_N_DEUTEROSOMAL_CELL																									
CELLMARKERCUR_N_DIFFUSE_GASTRIC_CANCER_CE…																									
CELLMARKERCUR_N_DISTAL_CELL																									
CELLMARKERCUR_N_DISTAL_STEM_CELL																									
CELLMARKERCUR_N_DISTAL_TUBULAR_CELL																									
CELLMARKERCUR_N_DIVIDING_CELL																									
CELLMARKERCUR_N_DIVIDING_PLASMA_B_CELL																									
CELLMARKERCUR_N_DIVIDING_T_CELL																									
CELLMARKERCUR_N_DOPAMINERGIC_NEURON																									
CELLMARKERCUR_N_DORSAL_ANTERIOR_HINDBRAIN…																									
CELLMARKERCUR_N_DORSAL_LATERAL_GANGLIONIC…																									
CELLMARKERCUR_N_DORSAL_MEDIAL_GANGLIONIC_…																									
CELLMARKERCUR_N_DOUBLE_NEGATIVE_B_CELL																									
CELLMARKERCUR_N_DOUBLE_NEGATIVE_T_CELL																									
CELLMARKERCUR_N_DOUBLE_POSITIVE_T_CELL																									
CELLMARKERCUR_N_DOUBLE_POSITIVE_TREG_CELL																									
CELLMARKERCUR_N_EARLY_HEMANGIOBLAST																									
CELLMARKERCUR_N_EARLY_PROXIMAL_TUBULE_CELL																									
CELLMARKERCUR_N_EARLY_RESPONSE_SECRETORY_…																									
CELLMARKERCUR_N_EFFECTOR_CHONDROCYTE																									
CELLMARKERCUR_N_EFFECTOR_MEMORY_T_CELL																									
CELLMARKERCUR_N_EFFECTOR_NATURAL_KILLER_C…																									
CELLMARKERCUR_N_ELONGATING_CELL																									
CELLMARKERCUR_N_EMBRYONIC_CELL																									
CELLMARKERCUR_N_EMBRYONIC_STEM_CELL	SMAD1	ESRRB	SMC2	PCID2	FOXD3	ENY2	STAT3	CNOT3	TFCP2L1	GDF3	TCL1A	TCF3	TBX3	NCL	KLF5	TET1	KLF4	SALL4	ZFX	NR0B1	LIF				
CELLMARKERCUR_N_ENDOCARDIAL_CELL																									
CELLMARKERCUR_N_ENDOCRINE_PROGENITOR_CELL																									
CELLMARKERCUR_N_ENDODERM_PROGENITOR_CELL																									
CELLMARKERCUR_N_ENDODERMAL_CELL																									
CELLMARKERCUR_N_ENDOMETRIAL_STROMAL_CELL																									
CELLMARKERCUR_N_ENDOMETRIAL_STROMAL_STEM_…																									
CELLMARKERCUR_N_ENDOTHELIAL_PRECURSOR_CELL																									
CELLMARKERCUR_N_ENSHEATHING_GLIAL_CELL																									
CELLMARKERCUR_N_ENTERIC_NEURON																									
CELLMARKERCUR_N_EOSINOPHIL	RCOR3	TKTL1	CLC	CD101	KCNH2	SIAH1	RRP12	TIPARP	CAT	SYNJ1	SMPD3	MYO15B	TGIF1	HES1	CD53	CD52	CD244	CD24	C3AR1	C5AR1	CCR1	CR1	FCER2	FCGR1A	CXCR3
CELLMARKERCUR_N_EPENDYMAL_CELL																									
CELLMARKERCUR_N_EPIBLAST_CELL																									
CELLMARKERCUR_N_EPICARDIAL_CELL																									
CELLMARKERCUR_N_EPICARDIUM_DERIVED_CELL																									
CELLMARKERCUR_N_EPIDERMAL_CELL																									
CELLMARKERCUR_N_EPIDERMAL_STEM_CELL																									
CELLMARKERCUR_N_EPITHELIAL_CELL	CXADR	CLDN12	TJP1	SDC1	OCLN	ALDH1A3	KRT14	ACTA2	ESR1	S100A9	MCAM	BST2	PIEZO2	COL13A1	TM4SF1	CDH5	CLEC14A	ICAM1	NRP2	CDX2	LRP5	GJA5	TMEM1…	CD99	KCNK17
CELLMARKERCUR_N_EPITHELIAL_DUCTAL_CELL																									
CELLMARKERCUR_N_EPITHELIAL_MESENCHYMAL_CE…																									
CELLMARKERCUR_N_EPITHELIOID_MACROPHAGE																									
CELLMARKERCUR_N_EPSILON_CELL																									
CELLMARKERCUR_N_ERYTHROBLAST																									
CELLMARKERCUR_N_ERYTHROID_CELL																									
CELLMARKERCUR_N_ERYTHROID_PROGENITOR_CELL																									
CELLMARKERCUR_N_ESOPHAGEAL_CELL																									
CELLMARKERCUR_N_ESOPHAGEAL_PROGENITOR_CELL																									
CELLMARKERCUR_N_EXCITATORY_NEURON																									
CELLMARKERCUR_N_EXHAUSTED_DOUBLE_POSITIVE…																									
CELLMARKERCUR_N_EXTRAVILLOUS_TROPHOBLAST																									
CELLMARKERCUR_N_F_PERICYTE																									
CELLMARKERCUR_N_FETAL_MYOBLAST																									
CELLMARKERCUR_N_FIBROBLAST_LIKE_CELL																									
CELLMARKERCUR_N_FIBROCHONDROCYTE																									
CELLMARKERCUR_N_FIBROCHONDROCYTE_PROGENIT…																									
CELLMARKERCUR_N_FOAM_CELL																									
CELLMARKERCUR_N_FOLLICULAR_B_CELL																									
CELLMARKERCUR_N_FOLLICULAR_CELL																									
CELLMARKERCUR_N_FOLLICULAR_DENDRITIC_CELL																									
CELLMARKERCUR_N_FOREBRAIN_CELL																									
CELLMARKERCUR_N_GABAERGIC_NEURON																									
CELLMARKERCUR_N_GANGLION_CELL																									
CELLMARKERCUR_N_GASTRIC_NEUROENDOCRINE_CE…																									
CELLMARKERCUR_N_GASTRIC_STEM_CELL																									
CELLMARKERCUR_N_GERM_CELL																									
CELLMARKERCUR_N_GERMINAL_CENTER_B_CELL																									
CELLMARKERCUR_N_GERMINAL_CENTER_CELL																									
CELLMARKERCUR_N_GERMINAL_CENTRE_B_CELL																									
CELLMARKERCUR_N_GLIAL_CELL	CXCR4																								
CELLMARKERCUR_N_GLIOMA_STEM_CELL																									
CELLMARKERCUR_N_GLOBOSE_BASAL_CELL																									
CELLMARKERCUR_N_GLOMERULAR_PARIETAL_EPITH…																									
CELLMARKERCUR_N_GLUTAMATERGIC_NEURON																									
CELLMARKERCUR_N_GOBLET_CELL																									
CELLMARKERCUR_N_GONADOTROPE																									
CELLMARKERCUR_N_GONADOTROPH_CELL																									
CELLMARKERCUR_N_GRANULOCYTE																									
CELLMARKERCUR_N_GRANULOCYTE_MONOCYTE_PROG…																									
CELLMARKERCUR_N_GRANULOCYTIC_UNK																									
CELLMARKERCUR_N_GRANULOSA_CELL																									
CELLMARKERCUR_N_HAEMATOPOIETIC_CELL																									
CELLMARKERCUR_N_HAIR_FOLLICLE_CELL																									
CELLMARKERCUR_N_HEMANGIOBLAST																									
CELLMARKERCUR_N_HEMATOPOIETIC_CELL																									
CELLMARKERCUR_N_HEMATOPOIETIC_PRECURSOR_C…																									
CELLMARKERCUR_N_HEMATOPOIETIC_PROGENITOR_…																									
CELLMARKERCUR_N_HEPATIC_CELL																									
CELLMARKERCUR_N_HEPATIC_PROGENITOR_CELL																									

Table S5: Cell state marker gene list

CELLMARKERCAN_ACINAR_CELLS																									
CELLMARKERCUR_N_HEPATIC_STELLATE_CELL																									
CELLMARKERCUR_N_HEPATOBLAST																									
CELLMARKERCUR_N_HEPATOCYTE																									
CELLMARKERCUR_N_HISTIOCYTE																									
CELLMARKERCUR_N_HMSC_TREATED_B_CELL																									
CELLMARKERCUR_N_HOFBAUER_CELL																									
CELLMARKERCUR_N_HOMEOSTATIC_CHONDROCYTE	RGS2	MCL1	PER1	TENT5C	NFKBIA	JUN	FIBIN	ATF3	HES1	MT1E	RBBP6	ARID4A	RGS16	NR4A2	OSR2	ZFP36L1	SGK1	SLC20A1	TRA2B	ID3	CSRNP1	TSPYL2	RSRC2	TIMP1	IFITM3
CELLMARKERCUR_N_HOMEOSTATIC_MICROGLIAL_CE…																									
CELLMARKERCUR_N_HORIZONTAL_BASAL_CELL																									
CELLMARKERCUR_N_HORIZONTAL_CELL																									
CELLMARKERCUR_N_HYPOBLAST_CELL																									
CELLMARKERCUR_N_IDIOPATHIC_PULMONARY_FIBR…																									
CELLMARKERCUR_N_IGA_MEMORY_B_CELL																									
CELLMARKERCUR_N_IGG_MEMORY_B_CELL																									
CELLMARKERCUR_N_IGM_MEMORY_B_CELL																									
CELLMARKERCUR_N_IGM_ONLY_B_CELL																									
CELLMARKERCUR_N_IL7R_T_HELPER_CELL																									
CELLMARKERCUR_N_IMMATURE_ERYTHROID_CELL																									
CELLMARKERCUR_N_IMMATURE_MYELOID_CELL																									
CELLMARKERCUR_N_IMMATURE_NEUTROPHIL																									
CELLMARKERCUR_N_IMMATURE_TRANSITIONAL_B_C…																									
CELLMARKERCUR_N_IMMUNE_CELL	BLK	CXCR4	CD53	CD79A	FCER1G	CD163	CTLA4	ENTPD1	ITGAM	CD8B	CD274	PDCD1L…	CD7	CD48	CD69	SPN	SLAMF7	CD28	SIGLEC5	ITGB2	ICAM3	LY9	CD5	CD52	CD2
CELLMARKERCUR_N_INDUCED_PLURIPOTENT_STEM_…																									
CELLMARKERCUR_N_INFILTRATING_MACROPHAGE																									
CELLMARKERCUR_N_INFLAMMATORY_CANCER_ASSOC…																									
CELLMARKERCUR_N_INFLAMMATORY_DENDRITIC_CE…																									
CELLMARKERCUR_N_INFLAMMATORY_MONOCYTE																									
CELLMARKERCUR_N_INHIBITORY_NEURON																									
CELLMARKERCUR_N_INNATE_LYMPHOID_CELL																									
CELLMARKERCUR_N_INNER_CELL_MASS_CELL																									
CELLMARKERCUR_N_INNER_RADIAL_GLIAL_CELL																									
CELLMARKERCUR_N_INTER_MEDIATE_PROGENITOR_…																									
CELLMARKERCUR_N_INTERCALATED_CELL																									
CELLMARKERCUR_N_INTERFERON_RESPONSIVE_SEC…																									
CELLMARKERCUR_N_INTERMEDIATE_MONOCYTE																									
CELLMARKERCUR_N_INTERMEDIATE_MONOCYTE_CELL																									
CELLMARKERCUR_N_INTERSTITIAL_MACROPHAGE																									
CELLMARKERCUR_N_INTERSTITIAL_PROGENITOR_C…																									
CELLMARKERCUR_N_INTESTINAL_EPITHELIAL_STE…																									
CELLMARKERCUR_N_INTESTINAL_GASTRIC_CANCER…																									
CELLMARKERCUR_N_INTESTINAL_METAPLASIA_CELL																									
CELLMARKERCUR_N_INTRAEPITHELIAL_LYMPHOCYT…																									
CELLMARKERCUR_N_IONOCYTE																									
CELLMARKERCUR_N_IRIS_PUTTATIVE_STEM_CELL																									
CELLMARKERCUR_N_KUPFFER_CELL																									
CELLMARKERCUR_N_LACTOTROPH_CELL																									
CELLMARKERCUR_N_LANGERHANS_CELL																									
CELLMARKERCUR_N_LEUKOCYTE	TRBC2																								
CELLMARKERCUR_N_LIMBAL_EPITHELIAL_STEM_CE…																									
CELLMARKERCUR_N_LIMBAL_STEM_CELL																									
CELLMARKERCUR_N_LIPOFIBROBLAST																									
CELLMARKERCUR_N_LIVER_PROGENITOR_CELL																									
CELLMARKERCUR_N_LIVER_STEM_CELL																									
CELLMARKERCUR_N_LMMLEYDIG_CELL																									
CELLMARKERCUR_N_LONOCYTE																									
CELLMARKERCUR_N_LOOP_OF_HENLE_CELL																									
CELLMARKERCUR_N_LOOP_PROGENITOR_CELL																									
CELLMARKERCUR_N_LOW_DENSITY_NEUTROPHIL																									
CELLMARKERCUR_N_LUMINAL_PROGENITOR_CELL																									
CELLMARKERCUR_N_LUNG_CELL																									
CELLMARKERCUR_N_LYMPH_NODE_CELL																									
CELLMARKERCUR_N_LYMPHATIC_CELL																									
CELLMARKERCUR_N_LYMPHATIC_ENDOTHELIAL_CELL																									
CELLMARKERCUR_N_LYMPHOBLASTOID_CELL																									
CELLMARKERCUR_N_LYMPHOCYTE	TBX1	FLT4	HOXD3	NUSAP1	ATP5F1C	LY6E	IRF4	GNLY	PRF1	NR4A2	GZMB	NR4A1	MKI67	PDCD1	CD3G	PECAM1	IL1RL1	CCR7	CD163						
CELLMARKERCUR_N_LYMPHOID_CELL																									
CELLMARKERCUR_N_LYMPHOID_PROGENITOR_CELL																									
CELLMARKERCUR_N_LYMPHOID_STRUCTURE_CELL																									
CELLMARKERCUR_N_M_PERICYTE																									
CELLMARKERCUR_N_M1_MACROPHAGE	IL2RA	CLEC7A	ROS1																						
CELLMARKERCUR_N_M1_MICROGLIAL_CELL																									
CELLMARKERCUR_N_M2_MACROPHAGE	MMP12	ADORA3	IRF4	STAT6	SOCS3	CCL22	MMP9	MMP19	MMP7	CCL4	IL4R	IL7R	FN1	VEGFA	CCL17	TGFB2	TGFB3	TGM2	PPARG	CCL13	ANXA1	DAB2	CCL23	IL4I1	CXCR1
CELLMARKERCUR_N_MACROGLIAL_CELL																									
CELLMARKERCUR_N_MALIGNANT_CELL																									
CELLMARKERCUR_N_MALIGNANT_PLASMA_CELL																									
CELLMARKERCUR_N_MARGINAL_ASSOCIATED_MACRO…																									
CELLMARKERCUR_N_MAST_CELL	MPO	PRG2	NR0B1	MAOB	ELANE	SLC24A3	PTGDS	HPGD	ABCC4	PPM1H	MLPH	PTGS1	SCG2	ADCYAP1	CEACAM8	TAL1	CALB2	SIGLEC6	RGS13	CD24	CR1	CSF2RA	FCER2	FCGR1A	FCGR2A
CELLMARKERCUR_N_MATURE_ASTROCYTE																									
CELLMARKERCUR_N_MATURE_B_CELL																									
CELLMARKERCUR_N_MATURE_DENDRITIC_CELL																									
CELLMARKERCUR_N_MATURE_ENDOTHELIAL_CELL																									
CELLMARKERCUR_N_MATURE_LUMINAL_CELL																									
CELLMARKERCUR_N_MATURE_NEURON																									
CELLMARKERCUR_N_MATURE_NEURONAL																									
CELLMARKERCUR_N_MATURE_OLIGODENDROCYTE																									
CELLMARKERCUR_N_MATURE_OSTEOBLAST																									
CELLMARKERCUR_N_MATURE_PODOCYTE																									
CELLMARKERCUR_N_MEDIAL_AND_DISTAL_SEGMENT…																									
CELLMARKERCUR_N_MEDIAL_GANGLIONIC_EMINENC…																									
CELLMARKERCUR_N_MEGAKARYOCYTE																									
CELLMARKERCUR_N_MEGAKARYOCYTE_ERYTHROID_C…	NT5E	PDCD1	PECAM1	PTPRC	SELL	SELP	SPN	ST2	IL18R1	NRP1	PTGDR2	KLRK1	IL17RA	ICOS	CD200R1	CCR2									
CELLMARKERCUR_N_MEGAKARYOCYTE_ERYTHROID_P…																									
CELLMARKERCUR_N_MEGAKARYOCYTE_PROGENITOR_…																									
CELLMARKERCUR_N_MELANOMA_CELL																									
CELLMARKERCUR_N_MEMORY_B_CELL																									
CELLMARKERCUR_N_MEMORY_B_CELL_PRECURSOR																									
CELLMARKERCUR_N_MEMORY_CD27_B_CELL																									
CELLMARKERCUR_N_MEMORY_CD4_T_CELL																									
CELLMARKERCUR_N_MEMORY_DOUBLE_POSITIVE_T_…																									
CELLMARKERCUR_N_MEMORY_REGULATORY_T_CELL																									
CELLMARKERCUR_N_MEMORY_T_CELL																									
CELLMARKERCUR_N_MERKEL_CELL																									
CELLMARKERCUR_N_MESENCHYMAL_CELL	CCL19	AXL	ENG	RUNX2	MMP9	TIMP2	TSC2	CHD7	MMP7	PITX2	LCN2	COL4A1	HAVCR1	ALCAM	TFRC	PTPRC	ACTA2	ILK	ITGB5	LEF1	S100A4	PECAM1	EPCAM	MFAP5	WNT2
CELLMARKERCUR_N_MESENCHYMAL_EPITHELIAL_TR…																									

Table S5: Cell state marker gene list

CELLMARKERCAN_ACINAR_CELLS																									
CELLMARKERCUR_N_MESENCHYMAL_GLIOBLASTOMA_…																									
CELLMARKERCUR_N_MESENCHYMAL_STEM_CELL	ABCG2	ITGA1	FZD9	CD24	CD81	DPP4	ALPL	BMPR1A	BMPR1B	BMPR2	ICAM1	IL6ST	CD63	GYPA	CD200	CDH1	ITGB2	CSPG4	CD276	CD99	SLC3A2	CD9	CD55	IL4R	CD59
CELLMARKERCUR_N_MESENCHYMAL_STROMAL_CELL	MME	NRP1	CD63																						
CELLMARKERCUR_N_MESENDODERMAL_CELL																									
CELLMARKERCUR_N_MESODERMAL_EMBRYONIC_STEM…																									
CELLMARKERCUR_N_MESODERMAL_PROGENITOR_CELL																									
CELLMARKERCUR_N_METAPLASTIC_STEM_CELL																									
CELLMARKERCUR_N_MEYLOID_CELL																									
CELLMARKERCUR_N_MICROGLIAL_CELL	ASPH	STAB1	CD163	TEK	B2M	BST2	HMOX1	MNDA	TUBA1A	C3AR1	GNG7	CCT3	HSPA8	CCND1	DOCK8	TREM2	CSF1	CD9	SPP1	APOE	ITGAX	LPL	CD33	CST7	AXL
CELLMARKERCUR_N_MICROVILLAR_CELL																									
CELLMARKERCUR_N_MIDBRAIN_DOPAMINERGIC_NEU…																									
CELLMARKERCUR_N_MONO_MACROPHAGE																									
CELLMARKERCUR_N_MONOCYTE_DERIVED_CELL																									
CELLMARKERCUR_N_MONOCYTE_DERIVED_MYELOID_…																									
CELLMARKERCUR_N_MONOCYTE_LINEAGE																									
CELLMARKERCUR_N_MONOCYTE_PRECURSOR																									
CELLMARKERCUR_N_MONOCYTE_PROGENITOR_CELL																									
CELLMARKERCUR_N_MUELLER_GLIA_CELL																									
CELLMARKERCUR_N_MULTIPOTENT_MESENCHYMAL_S…																									
CELLMARKERCUR_N_MULTIPOTENT_RETINAL_PROGE…																									
CELLMARKERCUR_N_MULTIPOTENT_STEM_CELL																									
CELLMARKERCUR_N_MULTIPOTENT_STROMAL_CELL																									
CELLMARKERCUR_N_MUSCLE_CELL																									
CELLMARKERCUR_N_MUSCLE_DERIVED_CELL																									
CELLMARKERCUR_N_MUSCLE_SATELLITE_CELL																									
CELLMARKERCUR_N_MYELOBLAST																									
CELLMARKERCUR_N_MYELOID_DENDRITIC_CELL																									
CELLMARKERCUR_N_MYELOID_DENDRITIC_CELL_1																									
CELLMARKERCUR_N_MYELOID_DERIVED_CELL																									
CELLMARKERCUR_N_MYELOID_DERIVED_SUPPRESSO…																									
CELLMARKERCUR_N_MYELOID_PROGENITOR_CELL																									
CELLMARKERCUR_N_MYO_EPITHELIAL_CELL																									
CELLMARKERCUR_N_MYOBLAST																									
CELLMARKERCUR_N_MYOCYTE																									
CELLMARKERCUR_N_MYOFIBROBLAST	MAFB	KAT14	NRXN1	KRT14	TYRP1	CCL21	ENG	PTPRC	SCGB1B…	PDPN	COL1A2	FAP	FNDC1	SFRP4	POSTN	WT1	MCAM	FN1	DES	TIMP1	PALLD				
CELLMARKERCUR_N_MYOGENIC_ENDOTHELIAL_CELL																									
CELLMARKERCUR_N_MYOGENIC_PROGENITOR_CELL																									
CELLMARKERCUR_N_MYOID_CELL																									
CELLMARKERCUR_N_NAIVE_B_CELL																									
CELLMARKERCUR_N_NAIVE_CD8_T_CELL																									
CELLMARKERCUR_N_NAIVE_DOUBLE_POSITIVE_T_C…																									
CELLMARKERCUR_N_NAIVE_PROGENITOR_CELL																									
CELLMARKERCUR_N_NAIVE_T_CELL																									
CELLMARKERCUR_N_NASCENT_NEPHRON_CELL																									
CELLMARKERCUR_N_NATURAL_KILLER_CD56_BRIGH…																									
CELLMARKERCUR_N_NATURAL_KILLER_CD56_DIM_C…																									
CELLMARKERCUR_N_NEGATIVE_MESENCHYMAL_STEM…																									
CELLMARKERCUR_N_NEURAL_CELL																									
CELLMARKERCUR_N_NEURAL_CREST_STEM_CELL																									
CELLMARKERCUR_N_NEURAL_CREST_STEMLIKE_CELL																									
CELLMARKERCUR_N_NEURAL_PRECURSOR_CELL																									
CELLMARKERCUR_N_NEURAL_STEM_CELL	FOXD3	LRTM1	CDCP1	SLAIN1	FUT4	GABPA	PMP2	RXRA	SLC2A1	SMARCA4	CD200														
CELLMARKERCUR_N_NEUROECTODERM_CELL																									
CELLMARKERCUR_N_NEURONAL_INTERMEDIATE_PRO…																									
CELLMARKERCUR_N_NEURONAL_PROGENITOR_CELL																									
CELLMARKERCUR_N_NEUTROPHIL_PROGENITOR_CELL																									
CELLMARKERCUR_N_NON_NEURON																									
CELLMARKERCUR_N_NON_PT_TUBULAR_CELL																									
CELLMARKERCUR_N_NON_RECIRCULATING_TISSUE_…																									
CELLMARKERCUR_N_NONMYELINATING_SCHWANN_CE…																									
CELLMARKERCUR_N_NOTOCHORD_CELL																									
CELLMARKERCUR_N_NUCLEATED_CELL																									
CELLMARKERCUR_N_NUCLEUS_PULPOSUS_PROGENIT…																									
CELLMARKERCUR_N_OLIGODENDROCYTE	SGSM3	PCDH15	DSCAM	SOX6	QKI	PCDH9																			
CELLMARKERCUR_N_OSTEOBLAST																									
CELLMARKERCUR_N_OUTER_RADIAL_GLIA																									
CELLMARKERCUR_N_OVALBUMIN_SPECIFIC_REGULA…																									
CELLMARKERCUR_N_OVARIAN_CELL																									
CELLMARKERCUR_N_OVARIAN_STROMAL_CELL																									
CELLMARKERCUR_N_P53_LIKE_CELL																									
CELLMARKERCUR_N_PAN_B_CELL																									
CELLMARKERCUR_N_PAN_CANCER_ASSOCIATED_FIB…																									
CELLMARKERCUR_N_PAN_MACROPHAGE																									
CELLMARKERCUR_N_PAN_MONOCYTE																									
CELLMARKERCUR_N_PAN_NEURONAL_CELL																									
CELLMARKERCUR_N_PAN_TROPHECTODERM_CELL																									
CELLMARKERCUR_N_PANCREATIC_ENDODERM_CELL																									
CELLMARKERCUR_N_PANCREATIC_POLYPEPTIDE_CE…																									
CELLMARKERCUR_N_PANCREATIC_PROGENITOR_CELL																									
CELLMARKERCUR_N_PANCREATIC_STEM_CELL																									
CELLMARKERCUR_N_PANETH_CELL																									
CELLMARKERCUR_N_PANNEURON																									
CELLMARKERCUR_N_PANVASCULAR_CELL																									
CELLMARKERCUR_N_PAPILLARY_FIBROBLAST																									
CELLMARKERCUR_N_PARIETAL_CELL																									
CELLMARKERCUR_N_PARVALBUMIN_INTERNEURON																									
CELLMARKERCUR_N_PATELET																									
CELLMARKERCUR_N_PATHOGENIC_T_PERIPHERAL_H…																									
CELLMARKERCUR_N_PELVIC_EPITHELIAL_CELL																									
CELLMARKERCUR_N_PERICYTE	TESC	PROCR	EBF2	PCLAF	SPON2	ADIRF	MEIS2	MEIS1	SYNM	COX4I2	TRPC6	NCAM1	ITGB1	SOX17	ENG	JAG1	CFB	SMN1	PHLDA1	NDUFA4L2	ALCAM	MME	PDGFA	DAPK3	
CELLMARKERCUR_N_PERIODONTAL_LIGAMENT_STEM…																									
CELLMARKERCUR_N_PERIOSTEUM_DERIVED_PROGEN…																									
CELLMARKERCUR_N_PERITUBULAR_MYOID_CELL																									
CELLMARKERCUR_N_PERIVASCULAR_LIKE_CELL																									
CELLMARKERCUR_N_PHAGOCYTE																									
CELLMARKERCUR_N_PHOTORECEPTOR_CELL																									
CELLMARKERCUR_N_PIT_CELL																									
CELLMARKERCUR_N_PIT_MUCOUS_CELL																									
CELLMARKERCUR_N_PITUITARY_STEM_CELL																									
CELLMARKERCUR_N_PLACENTAL_TISSUE_CELL																									
CELLMARKERCUR_N_PLACODE_CELL																									
CELLMARKERCUR_N_PLASMABLAST_B_CELL																									
CELLMARKERCUR_N_PLATELET																									
CELLMARKERCUR_N_PLURIPOTENT_STEM_CELL																									

CELLMARKERCAN_ACINAR_CELLS																									
CELLMARKERCUR_N_PODOCYTE_PRECURSOR																									
CELLMARKERCUR_N_PORTAL_FIBROBLAST																									
CELLMARKERCUR_N_POSTERIOR_CELL																									
CELLMARKERCUR_N_PRE_B_CELL																									
CELLMARKERCUR_N_PRE_CONVENTIONAL_DENDRITI…																									
CELLMARKERCUR_N_PRE_DENDRITIC_CELL																									
CELLMARKERCUR_N_PRECURSOR_CELL																									
CELLMARKERCUR_N_PREHYPERTROPHIC_CHONDROCY…																									
CELLMARKERCUR_N_PRESOMITE_CELL																									
CELLMARKERCUR_N_PRETUBULAR_AGGREGATE_CELL																									
CELLMARKERCUR_N_PRIMITIVE_ENDODERM_CELL																									
CELLMARKERCUR_N_PRINCIPAL_CELL																									
CELLMARKERCUR_N_PRO_B_CELL																									
CELLMARKERCUR_N_PRO_INFLAMMATORY_MACROPHA…																									
CELLMARKERCUR_N_PRO_NEUTROPHIL																									
CELLMARKERCUR_N_PROLIFERATING_CELL																									
CELLMARKERCUR_N_PROLIFERATIVE_CHONDROCYTE																									
CELLMARKERCUR_N_PROLIFERATIVE_DUCTAL_CELL																									
CELLMARKERCUR_N_PROLIFERATIVE_NEURAL_PROG…																									
CELLMARKERCUR_N_PRONEURAL_GLIOBLASTOMA_CE…																									
CELLMARKERCUR_N_PROSPERMATOGONIA																									
CELLMARKERCUR_N_PROSTATE_CELL																									
CELLMARKERCUR_N_PROXIMAL_CELL																									
CELLMARKERCUR_N_PROXIMAL_SEGMENT_CELL																									
CELLMARKERCUR_N_PROXIMAL_STEM_CELL																									
CELLMARKERCUR_N_PROXIMAL_TUBULE_CELL																									
CELLMARKERCUR_N_PULMONARY_NEUROENDOCRINE_…																									
CELLMARKERCUR_N_PYRAMIDAL_CELL																									
CELLMARKERCUR_N_QUIESCENT_T_STEM_CELL																									
CELLMARKERCUR_N_REGULATORY_B_CELL																									
CELLMARKERCUR_N_REGULATORY_INNATE_LYMPHOI…																									
CELLMARKERCUR_N_RENAL_TUBULAR_CELL																									
CELLMARKERCUR_N_RESIDENT_MEMORY_T_CELL																									
CELLMARKERCUR_N_RESPIRATORY_HORIZONTAL_BA…																									
CELLMARKERCUR_N_RESPIRATORY_SECRETORY_CELL																									
CELLMARKERCUR_N_RESPONDER_T_CELL																									
CELLMARKERCUR_N_RESPONDING_CONVENTIONAL_T…																									
CELLMARKERCUR_N_RESTING_MEMORY_B_CELL																									
CELLMARKERCUR_N_RESTING_NAIVE_B_CELL																									
CELLMARKERCUR_N_RESTING_REGULATORY_T_CELL																									
CELLMARKERCUR_N_RETICULAR_FIBROBLAST																									
CELLMARKERCUR_N_RETINA_CELL																									
CELLMARKERCUR_N_ROD_CELL																									
CELLMARKERCUR_N_SATELLITE_CELL																									
CELLMARKERCUR_N_SEPTUM_TRANSVERSUM_MESENC…																									
CELLMARKERCUR_N_SEROUS_CELL																									
CELLMARKERCUR_N_SIDE_POPULATION_CELL																									
CELLMARKERCUR_N_SKELETAL_MUSCLE_CELL																									
CELLMARKERCUR_N_SKELETAL_MUSCLE_STEM_CELL																									
CELLMARKERCUR_N_SMOOTH_MUSCLE_CELL																									
CELLMARKERCUR_N_SMOOTH_MUSCLE_LIKE_CELL																									
CELLMARKERCUR_N_SOMATIC_CELL																									
CELLMARKERCUR_N_SOMATIC_STEM_CELL																									
CELLMARKERCUR_N_SOMATOSTATIN_INTERNEURON																									
CELLMARKERCUR_N_SPECIALIST_ANTIGEN_PRESEN…																									
CELLMARKERCUR_N_SPERMATOGENIC_CELL																									
CELLMARKERCUR_N_SPERMATOGONIAL_CELL																									
CELLMARKERCUR_N_SPERMATOGONIAL_STEM_CELL																									
CELLMARKERCUR_N_SQUAMOUS_DIFFERENTIATION_…																									
CELLMARKERCUR_N_STELLATE_CELL																									
CELLMARKERCUR_N_STEM_CELL_DERIVED_ALPHA_C…																									
CELLMARKERCUR_N_STEM_CELL_DERIVED_BETA_CE…																									
CELLMARKERCUR_N_STEM_CELL_DERIVED_ENDOTHE…																									
CELLMARKERCUR_N_STEM_LIKE_CD8_T_CELL																									
CELLMARKERCUR_N_STROMAL_CELL	RSPO3	CCL11	PLVAP	SMN1	PRELP	MYL9	TNC	PDGFRB	LUM	PDGFRA	VWF	LIMCH1	POSTN	ABCA10	MYH11	COL3A1	WT1	CLEC11A	FGFR1	IGFBP5	TPM2	IVL	PSMB11	FOXN1	MYOD1
CELLMARKERCUR_N_SUBPALLIUM_CELL																									
CELLMARKERCUR_N_SUBPLATE_CELL																									
CELLMARKERCUR_N_SUPPRESSIVE_MONOCYTE																									
CELLMARKERCUR_N_SUPPRESSOR_T_CELL																									
CELLMARKERCUR_N_SUPRABASAL_CELL																									
CELLMARKERCUR_N_SURFACE_ECTODERM_CELL																									
CELLMARKERCUR_N_SUSTENTACULAR_CELL																									
CELLMARKERCUR_N_SYMPATHOADRENAL_CELL																									
CELLMARKERCUR_N_SYNCYTIOTROPHOBLAST																									
CELLMARKERCUR_N_SYNTHETIC_SMOOTH_MUSCLE_C…																									
CELLMARKERCUR_N_T_CELL_LARGE_GRANULAR_LYM…																									
CELLMARKERCUR_N_T_CELL_LINEAGE																									
CELLMARKERCUR_N_T_HELPER_CELL																									
CELLMARKERCUR_N_T_MEMORY_STEM_CELL																									
CELLMARKERCUR_N_T2_MZP_REGULATORY_B_CELL																									
CELLMARKERCUR_N_TENDON_CELL																									
CELLMARKERCUR_N_TERMINAL_DIFFERENTIATED_B…																									
CELLMARKERCUR_N_THALAMIC_CELL																									
CELLMARKERCUR_N_THECA_INTERNA_CELL																									
CELLMARKERCUR_N_THYMIC_EPITHELIAL_CELL																									
CELLMARKERCUR_N_TISSUE_LIKE_MEMORY_B_CELL																									
CELLMARKERCUR_N_TISSUE_RESIDENT_CELL																									
CELLMARKERCUR_N_TISSUE_RESIDENT_EFFECTOR_…																									
CELLMARKERCUR_N_TISSUE_RESIDENT_MACROPHAGE																									
CELLMARKERCUR_N_TISSUE_RESIDENT_MEMORY_LI…																									
CELLMARKERCUR_N_TOLDC_INDUCED_REGULATORY_…																									
CELLMARKERCUR_N_TONSIL_CELL																									
CELLMARKERCUR_N_TRABECULAR_MESHWORK_CELL																									
CELLMARKERCUR_N_TRANSITIONAL_B_CELL																									
CELLMARKERCUR_N_TRANSITIONAL_DENDRITIC_CE…																									
CELLMARKERCUR_N_TRUNCATED_RADIAL_GLIAL_CE…																									
CELLMARKERCUR_N_TUBULAR_CELL																									
CELLMARKERCUR_N_TUMOR_ASSOCIATED_MICROGLI…																									
CELLMARKERCUR_N_TUMOR_ENDOTHELIAL_CELL																									
CELLMARKERCUR_N_TUMOR_EPITHELIAL_CELL																									
CELLMARKERCUR_N_TUMOR_INFILTRATING_REGULA…																									
CELLMARKERCUR_N_TUMOR_INFILTRATING_T_CELL																									
CELLMARKERCUR_N_TUMOR_INITIATING_CELL																									
CELLMARKERCUR_N_TUMOR_REGULATORY_T_CELL																									

CELLMARKERCAN_ACINAR_CELLS																									
CELLMARKERCUR_N_UMBRELLA_CELL																									
CELLMARKERCUR_N_UNRESTRICTED_SOMATIC_STEM…																									
CELLMARKERCUR_N_UNSWITCHED_PLASMABLAST																									
CELLMARKERCUR_N_URETERIC_BUD_CELL																									
CELLMARKERCUR_N_UROTHELIAL_CELL																									
CELLMARKERCUR_N_UROTHELIUM_CELL																									
CELLMARKERCUR_N_VASCULATURE_CELL																									
CELLMARKERCUR_N_VEIN_CELL																									
CELLMARKERCUR_N_VENOUS_CELL																									
CELLMARKERCUR_N_VENTRAL_LATERAL_GANGLIONI…																									
CELLMARKERCUR_N_VENTRAL_MEDIAL_GANGLIONIC…																									
CELLMARKERCUR_N_VENTRICULAR_CELL																									
CELLMARKERCUR_N_YOUNG_NEURON																									
CELLMARKERCUR_N_ZONA_FASCICULATA_CELL																									
CELLMARKERCUR_N_ZONA_GLOMERULOSA_CELL																									
CELLMARKERCUR_N_ZONA_RETICULARIS_CELL																									
CELLMARKERCUR_N_ZYGOTE																									
CELLMARKERCURNACTIVATEDEFFECTORMEMORYCD4T…																									
CELLMARKERCURNACTIVATEDTISSUERESIDENTMEMO…																									
CELLMARKERCURNACTIVATEDTISSUERESIDENTMEMO…																									
CELLMARKERCURNALPHACELLACELL																									
CELLMARKERCURNALVEOLARTYPEIIATIICELL																									
CELLMARKERCURNATYPICALBCELLABC																									
CELLMARKERCURNAXLCD11CPLASMACYTOIDDENDRIT…																									
CELLMARKERCURNAXLCD123PLASMACYTOIDDENDRIT…																									
CELLMARKERCURNBASALFOREBRAINCHOLINERGICNE…																									
CELLMARKERCURNBRUSHCELLTUFTCELL																									
CELLMARKERCURNCANCERASSOCIATEDFIBROBLASTC…																									
CELLMARKERCURNCAPSULARFIBROBLASTFB2																									
CELLMARKERCURNCCR7DENDRITICCELL																									
CELLMARKERCURNCD106MESENCHYMALSTEMCELL																									
CELLMARKERCURNCD14MACROPHAGE																									
CELLMARKERCURNCD16DENDRITICCELL																									
CELLMARKERCURNCD25LAG3TCELL																									
CELLMARKERCURNCD34GRANULOCYTICPROGENITORC…																									
CELLMARKERCURNCD34HEMATOPOIETICSTEMCELL																									
CELLMARKERCURNCD34PREBCELL																									
CELLMARKERCURNCD34PROGENITORCELL																									
CELLMARKERCURNCD34THYMOCYTE																									
CELLMARKERCURNCD40LGTHELPERCELL																									
CELLMARKERCURNCD4CD25REGULATORYTCELL																									
CELLMARKERCURNCD4CENTRALMEMORYLIKETTCMLIK…																									
CELLMARKERCURNCD4CYTOTOXICT1CELL																									
CELLMARKERCURNCD4CYTOTOXICT2CELL																									
CELLMARKERCURNCD4THELPERCELL																									
CELLMARKERCURNCHRONICALLYACTIVATEDCD4TCELL																									
CELLMARKERCURNCHRONICALLYACTIVATEDCD8TCELL																									
CELLMARKERCURNCLASSSWITCHEDMEMORYBIGGU03B…																									
CELLMARKERCURNCLEC10ADENDRITICCELL																									
CELLMARKERCURNCLEC9ADENDRITICCELL																									
CELLMARKERCURNCONVENTIONALDENDRITICCELL2B…																									
CELLMARKERCURNCONVENTIONALDENDRITICCELL2B…																									
CELLMARKERCURNCORTICALSOMATOSTATINSSTINTE…																									
CELLMARKERCURNCYTOTOXICCD4T2CELL																									
CELLMARKERCURNCYTOTOXICCD4TCELL																									
CELLMARKERCURND1MSNMEDIUMSPINYNEURON																									
CELLMARKERCURND2MSNMEDIUMSPINYNEURON																									
CELLMARKERCURNDISTALTUBULELOOPOFHENLECELL																									
CELLMARKERCURNEARLYCD34MEGAKARYOCYTEERYTH…																									
CELLMARKERCURNEARLYTCELLPROGENITORETP																									
CELLMARKERCURNEFFECTORMEMORYCD4TCELL																									
CELLMARKERCURNEFFECTORREGULATORYTETREGCELL																									
CELLMARKERCURNEFFECTORTTEFFCELL																									
CELLMARKERCURNEXHAUSTEDCD4TCELL																									
CELLMARKERCURNEXHAUSTEDCD8TCELL																									
CELLMARKERCURNEXHAUSTEDCD8TPROGENITORCELL																									
CELLMARKERCURNEXHAUSTEDTTEXCELL	CD3E	HAVCR1	CD4	CD2	BTLA	ADORA2A	CD96	ICOS	IFIT3	ENTPD1	TNFRSF9														
CELLMARKERCURNEXTRATELENCEPHALICETCELL																									
CELLMARKERCURNFINALLYHIGHLYEFFECTORTEMRAM…																									
CELLMARKERCURNFOLLICULARREGULATORYTTFRCELL																									
CELLMARKERCURNFOXP3NATURALREGULATORYTTREG…																									
CELLMARKERCURNGAMMADELTAU03B3DTCELL																									
CELLMARKERCURNGERMINALCENTREGCLIKECELL																									
CELLMARKERCURNGLOMERULARENDOTHELIALCELLECG																									
CELLMARKERCURNGRANULOCYTICMYELOIDDERIVEDS…																									
CELLMARKERCURNGRBREGULATORYBCELL																									
CELLMARKERCURNGZMKTCELL																									
CELLMARKERCURNHIGHLYDIFFERENTIATEDEFFECTO…																									
CELLMARKERCURNINDUCEDREGULATORYTTREGCELL																									
CELLMARKERCURNINFLAMMATORYCANCERASSOCIATE…																									
CELLMARKERCURNINNATELYMPHOIDCELLILCS																									
CELLMARKERCURNINTRATHYMICFIBROBLASTFB1																									
CELLMARKERCURNITOCELLHEPATICSTELLATECELL																									
CELLMARKERCURNLYMPHANGIOLEIOMYOMATOSISCEL…																									
CELLMARKERCURNMARGINALZONEMZLIKEBCELL																									
CELLMARKERCURNMEMORYSTEMCELLTSCM																									
CELLMARKERCURNMICROGLIADERIVEDTUMORASSOCI…																									
CELLMARKERCURNMKERYTHCELL																									
CELLMARKERCURNMYOFIBROBLASTICCANCERASSOCI…																									
CELLMARKERCURNNAIVEREGULATORYTTREGCELL																									
CELLMARKERCURNNATURALREGULATORYTTREGCELL																									
CELLMARKERCURNPERIPHERALBLOODMONONUCLEARC…																									
CELLMARKERCURNPITUITARYSTEMCELLPSC																									
CELLMARKERCURNPLASMACYTOIDDENDRITICCELLPDC	TRAF4	PLD4	ALOX5AP	SPCS1	OFD1	LTB	IDH3A	APP	PTGDS	PTPRS	UGCG	SELL	TSPAN13	SEC61B	CCDC186	IRF4									
CELLMARKERCURNPOLYMORPHONUCLEARMYELOIDDER…																									
CELLMARKERCURNPOLYMORPHONUCLEARNEUTROPHIL…																									
CELLMARKERCURNPOSTERIORPRESOMITICMESODERM…																									
CELLMARKERCURNPROLIFERATIVECD4TCELL																									
CELLMARKERCURNPROLIFERATIVECD8TCELL																									
CELLMARKERCURNREDBLOODCELLERYTHROCYTE																									
CELLMARKERCURNREGULATORYBBREGCELL																									
CELLMARKERCURNREGULATORYCD4TCELL																									
CELLMARKERCURNREGULATORYCD8TCELL																									
CELLMARKERCURNREGULATORYTTREGCELL	ENC1	CCR8	TGFB1	STAT5B	CCR10	LAYN	MAGEH1	CD3G	IL10	CD40LG	MAF	IFNG	IL17A	IL23R	IL1R1	ID3	LTB	CNGB1	NT5E	CCR6	RORC	SELL	GPA33	KLRD1	CD8B

Table S5: Cell state marker gene list

CELLMARKERCAN_ACINAR_CELLS																									
CELLMARKERCURNREGULATORYTTREGCELL	CXCR6																								
CELLMARKERCURNRENALVESICLECOMMASHAPEDBODY…																									
CELLMARKERCURNRESIDENTMEMORYCD8TCELL																									
CELLMARKERCURNREVERTANTMEMORYCD8TTEMRACELL																									
CELLMARKERCURNSCARASSOCIATEDMACROPHAGESAM																									
CELLMARKERCURNSENESCENTCD8TCELL																									
CELLMARKERCURNSTEMPROGENITORCELL																									
CELLMARKERCURNSWITCHEDMEMORYBCELLSWMB																									
CELLMARKERCURNT1TRANSITIONALBCELL																									
CELLMARKERCURNT2TRANSITIONALBCELL																									
CELLMARKERCURNTCELLNATURALKILLERCELL																									
CELLMARKERCURNTERMINALEFFECTORCD8TCELL																									
CELLMARKERCURNTERMINALLYDIFFERENTIATEDCD8…																									
CELLMARKERCURNTFOLLICULARHELPERTFHCELL	CXCR6																								
CELLMARKERCURNTHELPER1TH1CELL																									
CELLMARKERCURNTHELPER1TH1CELL																									
CELLMARKERCURNTHELPER2TH2CELL	LAIR2	GSTA4	CENPF	SMAD2	MB	MICAL2	CDC25C	TIGIT	GEM	TNFRSF4	CD274	CXCL12	PECAM1	AKT1	EPHB2	CXCR4									
CELLMARKERCURNTHELPER2TH2CELL																									
CELLMARKERCURNTHELPER9TH9CELL																									
CELLMARKERCURNTHELPERTHCELL	ATF2	SRSF10	ICOS	ASF1A	PHF10	SEC24C	BATF	ANP32B	GOLGA8A	ITM2A	TRBV20…	ITGAL													
CELLMARKERCURNTHYMICEPITHELIALCELLTEC																									
CELLMARKERCURNTISSUERESIDENTMEMORYCD4TCELL																									
CELLMARKERCURNTISSUERESIDENTMEMORYCD8TCELL																									
CELLMARKERCURNTISSUERESIDENTMEMORYTTRMCELL																									
CELLMARKERCURNTPERIPHERALHELPERTPHCELL																									
CELLMARKERCURNTRANSIENTAMPLIFYINGTACELL																									
CELLMARKERCURNTRANSITIONALEXHAUSTEDCD8TCE…																									
CELLMARKERCURNTUMORINFILTRATINGLYMPHOCYTE…																									
CELLMARKERCURNUNSWITCHEDMEMORYBCELLUNSWMB																									
CELLMARKERCURNVASARECTAENDOTHELIALCELLECVR																									
CELLMARKERCURNVONECONOMONEURONVEN																									
CELLMARKERCURNYOLKSACDERIVEDMYELOIDBIASED…																									
CELLMARKEREXP_ACINAR_CELLS																									
CELLMARKEREXP_ADRENERGIC_NEURONS																									
CELLMARKEREXP_AIRWAY_EPITHELIAL_CELLS																									
CELLMARKEREXP_AIRWAY_SMOOTH_MUSCLE_CELLS																									
CELLMARKEREXP_ALPHA_CELLS																									
CELLMARKEREXP_ANTERIOR_PITUITARY_GLAND_CE…																									
CELLMARKEREXP_ASTROCYTES	VIM	CCR7	AANAT	FABP7	SOCS3	SYNM	HMG20A	NFATC3	SRR	SLC1A6	ETS1	SLIT1	SNPH	IL23A											
CELLMARKEREXP_B_CELLS	CRELD2	DERL3	RALGPS2	FCHSD2	POLD4	TNFRSF17	HVCN1	FCRLA	EDEM2	BLNK	TXNDC11	BTLA	SMAP2	FKBP11	SEC61A1	SPCS3	SPIB	EAF2	CXCR4	BIRC3	IGLC2	IGLC3	IGLC1	IL21R	IGKC
CELLMARKEREXP_B_CELLS_MEMORY	NPIPB15	P2RX5	PNOC	PTPRCAP	RALGPS2	RASGRP2	SIK1	SIT1	SLC12A1	SP140	STAP1	TMEM156	TNFRSF13B	TNFRSF17	TRAF4	VPREB3	ZBTB32								
CELLMARKEREXP_B_CELLS_NAIVE	RASGRP2	SELL	SIK1	SLC12A1	SPIB	STAP1	TCL1A	UGT1A8	VPREB3	ZNF286A	TNFSF8	FOXP1	MITF	CD52	HLA-DQB1	TNFRSF13C	LINC009…	STAG3	BIRC3	CD74					
CELLMARKEREXP_BASAL_CELLS	KRT15	PLP2	MT1X	S100A14	LAMC2	BMP7	MYLK																		
CELLMARKEREXP_BASOPHILS	LAMA5	MBOAT1	NFIL3	NPL	OSBPL5	OTUB2	P2RY1	PDK3	PDZD4	PERP	PIGY	PLEKHG5	PLG	RNF39	SLC26A3	SLC26A8	SNCAIP	ST6GALNAC5	SUPT3H	TULP3	TEC	UNC93A	WRN	ID1	
CELLMARKEREXP_BERGMANN_GLIA																									
CELLMARKEREXP_BETA_CELLS	MEG3	RGS16																							
CELLMARKEREXP_CAJAL_RETZIUS_CELLS	ATP13A2	CNTNAP2	PNOC	LHX1	LINGO2	FAT3	SRGAP3	CDH4	FLRT3	EDIL3	ZCCHC12	RALGPS2	FAM196A	SMAD1	TBR1	KCNH7	LHX5	CALB2	EMX2	ZIC2	WNT7B	AATF	NANOS1	TBCK	RGMB
CELLMARKEREXP_CHOLANGIOCYTES																									
CELLMARKEREXP_CHOLINERGIC_NEURONS																									
CELLMARKEREXP_CHONDROCYTES	AGER	IL10RA																							
CELLMARKEREXP_DELTA_CELLS																									
CELLMARKEREXP_DISTAL_TUBULE_CELLS																									
CELLMARKEREXP_DUCTAL_CELLS																									
CELLMARKEREXP_EMBRYONIC_STEM_CELLS	SOX2	KLF4	ZFP42	ZFX	TAF8	HMGA2	NACC1	NR6A1	STAT3	LEF1	L1TD1	KHDC3L	GDF3	DPPA5	DPPA4	DPPA2	DPPA3	SMAD1	SMAD5	SMAD9	SMAD4	SMAD2	SMAD3	CTNNB1	HHEX
CELLMARKEREXP_ENTERIC_NEURONS	SLC7A14	JPH3	CASZ1	TAC1	SCN3A	CHRNA3	PARVA	SLC10A4	TM4SF4	FAM19A1	SLC5A7	PHOX2B	PIRT	HOXB5	HOXA5	GAL	CARTPT	NMU	VIP	CCK					
CELLMARKEREXP_ENTEROENDOCRINE_CELLS																									
CELLMARKEREXP_EPENDYMAL_CELLS	EFNB3	MYB	RFX2	ZMYND10	AK8	CFAP44	TM4SF1																		
CELLMARKEREXP_EPSILON_CELLS																									
CELLMARKEREXP_ERYTHROID_LIKE_AND_ERYTHROI…	PAN3	PFAS	VAMP5	PNPO	TSPO2	RHD	KLF1	TSPAN33	EPB42	CD44	CD47	CD55	EDRF1	EPB41L2	GYPC	KEL	RHAG	THRB	UROD	UROS	SNCA				
CELLMARKEREXP_GAMMA_DELTA_T_CELLS	TRGJ1	TRGJ2	TRGJP1	TRGJP2	TRGJP	TRGC1	TRGC2	TRDD1	TRDD2	TRDD3	TRDJ1	TRDJ2	TRDJ3	TRDJ4	TRDV1	TRDV2	TRDV3								
CELLMARKEREXP_GOBLET_CELLS																									
CELLMARKEREXP_HEMANGIOBLASTS																									
CELLMARKEREXP_HEMATOPOIETIC_STEM_CELLS	GNL3	MGST1	G0S2	PRNP	CD34	BMI1	CD59	ITGA4	ANPEP	CD33	IL3RA	HLX	NKX3-1	THSD1	NCOR2	EGR1	FGD5	MECOM	CTNNAL1	VWF	PBX2	TAL1	ERG	NEO1	EGR3
CELLMARKEREXP_HEPATIC_STELLATE_CELLS																									
CELLMARKEREXP_HIS_BUNDLE_CELLS																									
CELLMARKEREXP_IMMATURE_NEURONS																									
CELLMARKEREXP_JUXTAGLOMERULAR_CELLS																									
CELLMARKEREXP_KERATINOCYTES																									
CELLMARKEREXP_KIDNEY_PROGENITOR_CELLS																									
CELLMARKEREXP_KUPFFER_CELLS																									
CELLMARKEREXP_LEYDIG_CELLS	GAS6	SULT1E1	CYP19A1	PLAUR	FABP9																				
CELLMARKEREXP_LOOP_OF_HENLE_CELLS	LIPG	TNNI1	LRRC66	IGF1																					
CELLMARKEREXP_LUMINAL_EPITHELIAL_CELLS																									
CELLMARKEREXP_MAMMARY_EPITHELIAL_CELLS																									
CELLMARKEREXP_MEGAKARYOCYTES																									
CELLMARKEREXP_MELANOCYTES																									
CELLMARKEREXP_MESANGIAL_CELLS	SNCG	FILIP1L	FLNA	MEF2C	CSTB	SMARCA5	PDE3A	MAP3K20																	
CELLMARKEREXP_MESOTHELIAL_CELLS	EFEMP1	FRAS1	RSPO1	RARRES2																					
CELLMARKEREXP_MICROFOLD_CELLS																									
CELLMARKEREXP_MICROGLIA	CYTH4	MERTK	MAFB	HPGDS	BIN2	CMTM6	CCL4	CCL3	CCRL2	UCP2	CLEC3B	MRC2	CLEC10A	PF4	CD53	FOS	EGR1	AIF1	CTSS	CSF1R	SKI	C1QB			
CELLMARKEREXP_MONOCYTES	S100A8	S100A4	CLEC7A	CSF3R	MNDA	MS4A6A	ZFP36…	LTA4H	CLEC12A	CD48	PRTN3	FCGR3A	VCAN	IFITM3	FN1	ADGRE1	CD44	CSF1R	CX3CR1	ITGAL	PECAM1	PTPRC	SPN	PSAP	FCN1
CELLMARKEREXP_MULLER_CELLS	SLC6A1	SFTPA1																							
CELLMARKEREXP_MYOBLASTS																									
CELLMARKEREXP_MYOCYTES	DLL1	DOCK1	DOCK5	FLNC	IQSEC1	ITGA3	MMP2	MMP9	MRC1	MYH14	NOTCH3	NPHS1	PLD1	PRKCQ	PTGIR	PTK2	PXN	SULF1	TRIO	WASHC1	WIPF1	NPPA	NPPB	EDN1	GATA4
CELLMARKEREXP_MYOFIBROBLASTS																									
CELLMARKEREXP_NEUROBLASTS	CNR1																								
CELLMARKEREXP_NEUTROPHILS	IL1R2	CFP	ADAM8	CD177	PTGS2	OAS3	PRTN3	AZU1	CTSG	SERPIN…	CAMP	SLC1A5	SNX20	ADPGK	PSTPIP1	LYST	DOCK8	S100A4	NLRP3	CSF3R					
CELLMARKEREXP_NK_CELLS	CHSY1	XCL2	TRDC	CXCR4	IL18R1	SERPINB9	DOCK2	SH2D2A	S100A4	XCL1	GZMK	IFNG	CCL3	CSF2	IL2RG	TGFB1	KIR2DL1	KIR3DL1	LILRB1	KLRG1	NCR3	ADAMTS14	ITGA2	STYK1	CLEC2D
CELLMARKEREXP_NORADRENERGIC_NEURONS																									
CELLMARKEREXP_OLIGODENDROCYTES	POU3F1	NINJ2	SGK2	GJC3	GPR37	IL33	KLK6	NFE2L3	PEX5L	CLDN14	ITGB4	MYO1D	PDE8A	CNTF	EML1	KIF13B	KIF6	NIPAL4	SEC14L5	SP7	ST18	DUSP15	PRKCZ	ARSG	NPC1
CELLMARKEREXP_OSTEOBLASTS	CALB1	COL11A1	COL11A2	IBSP	POSTN	SFRP4	BMP1	OLFML2B	COL12A1	WISP2	SP7	PENK	NPNT	OLFML1											
CELLMARKEREXP_OSTEOCLAST_PRECURSOR_CELLS																									
CELLMARKEREXP_OSTEOCLASTS																									
CELLMARKEREXP_OSTEOCYTES																									
CELLMARKEREXP_PANETH_CELLS																									
CELLMARKEREXP_PARATHYROID_CHIEF_CELLS																									
CELLMARKEREXP_PERICYTES	IFITM1	TBX18	NT5E	MFGE8	ALPL	COL1A1	MYO1B	COG7	P2RY14	HEYL	GNB4	MSX1	CTGF												
CELLMARKEREXP_PHOTORECEPTOR_CELLS	CPLX3	B3GALT2	ISL1	PCP2																					
CELLMARKEREXP_PLASMA_CELLS	MAN1A1	MANEA	MAST1	MROH7	MZB1	P2RX5	PAX7	PDK1	PNOC	RASGRP3	REN	RGS13	RPL3P7	SIK1	SPAG4	ST6GALNAC4	TGM5	TMEM156	UGT2B17	ZBP1	ZNF165	IGLC2	IGHG1	IGHG2	IGHG3
CELLMARKEREXP_PLASMACYTOID_DENDRITIC_CELLS	ZFAT	NRP1	SLC15A4	CCR9	IRF5	BST2																			
CELLMARKEREXP_PODOCYTES	NEBL	SH2D4A	TDRD5	PTH1R	IQGAP2	CTGF	FAT1	ILDR2	FOXD1	TRIB2	LOXL2	COL4A5	FOXC1	PLEKHH2	CTNNAL1	DACH1	SULF1	HSPB8	KANK1	ADCY1	DOCK5	CRB2	LRRC49	CORO2B	ARHGA…
CELLMARKEREXP_PRINCIPAL_CELLS																									
CELLMARKEREXP_PROXIMAL_TUBULE_CELLS	SLC9A8	SCIN	ALPL	G6PC	SLC22A…	SLC22A7	SLC16…	AGXT2	SORD	SOD3	HNF4A	NME4	APOE	HGD	OSGIN1	HSD11B1	SERPINF2	HAAO	PPIC	TFF3	GNG13	SLC23A3	GC	CACNG5	TGFBI
CELLMARKEREXP_PULMONARY_ALVEOLAR_TYPE_I_C…																									

Table S5: Cell state marker gene list

CELLMARKERCAN_ACINAR_CELLS																									
CELLMARKEREXP_PULMONARY_ALVEOLAR_TYPE_II_…																									
CELLMARKEREXP_PURKINJE_FIBER_CELLS																									
CELLMARKEREXP_PURKINJE_NEURONS	DLGAP1	GNG4	SHISA8	SYNPR	PRKCA	PCP4L1	PCP4	SLC1A6	GRID2	GAD2															
CELLMARKEREXP_PYRAMIDAL_CELLS																									
CELLMARKEREXP_RETICULOCYTES																									
CELLMARKEREXP_RETINAL_GANGLION_CELLS	CPNE4	KLHDC8A	IRX6	CHRNA6	CACNB3	CSDC2	AKAP7	HPCA	ISL1	SLC17A6	FGF12	DNER	TMEM163	RBPMS	POU4F1	RBPMS2	CDK14	FXYD7	NEFM	SNCG					
CELLMARKEREXP_SALIVARY_MUCOUS_CELLS																									
CELLMARKEREXP_SATELLITE_CELLS																									
CELLMARKEREXP_SCHWANN_CELLS																									
CELLMARKEREXP_SEROTONERGIC_NEURONS																									
CELLMARKEREXP_SERTOLI_CELLS	OCLN	SERPINB9	SERPIN…	CD55	CD46	CLU	FSHR	RXRB	PRLR	SLC16A3	EPS8	NR1H2	BCAR3	SOX9											
CELLMARKEREXP_SMOOTH_MUSCLE_CELLS	SMOC1	AAED1	AOC3	SEC24D	KCNAB1	MYLK4	KIF1C	MRVI1	JPH2	ITGA9	KCNMB1	NOX4	NOV	DMPK	SH3BGR	ITGA8	TCF21	ADAMDEC1	HHIP	MFAP5	SPON2	LAMB2	GJC1	ACKR3	OGN
CELLMARKEREXP_SPERMATOZOA																									
CELLMARKEREXP_STROMAL_CELLS																									
CELLMARKEREXP_T_CELLS	CD8B	BATF3	GZMH	LAG3	GZMK	GZMB	SH2D1A	MYO1G	FMNL1	S1PR4	CD247	GIMAP5	CD28	CD160	TRDC	RHOH	KLRB1	CCR2	IL2RB	CD163L1	MBD2	ICOS	IL18R1	TNFRSF4	CCL20
CELLMARKEREXP_T_CELLS_NAIVE																									
CELLMARKEREXP_T_CYTOTOXIC_CELLS																									
CELLMARKEREXP_T_HELPER_CELLS	IL1RL1	FOXP3	CD3G	TNFRSF4	CD40LG	IL17RB	HPGDS	NFATC2	NFKB1	NR3C1	PPARG														
CELLMARKEREXP_T_MEMORY_CELLS	CD7	CXCR6	ICOS	CCR6	ITGAE	ENPP1																			
CELLMARKEREXP_TRICHOCYTES																									
CELLMARKEREXP_TRIGEMINAL_NEURONS																									
CELLMARKEREXP_TROPHOBLAST_CELLS																									
CELLMARKEREXP_TROPHOBLAST_PROGENITOR_CELLS																									
CELLMARKEREXP_TUFT_CELLS																									
CELLMARKEREXP_VASCULAR_SMOOTH_MUSCLE_CELLS																									
CELLMARKEREXPENDOTHELIALCELLSAORTA	CLEC1A	MARVELD1	FAM198B	THBD	NOS3	TNS2	ATL2	ACKR3	COL4A4	CALCRL	COL4A3	PER3	STAB1	CYP1B1	EPHX2	KLK8	BACE2	BMP6	SULF1	CPQ	PI16	AMIGO2	PTPRJ	TRIM47	LMCD1
CELLMARKEREXPNEURALSTEMPRECURSORCELLS	ALDOC	APOE	ID4	SOX2	DCX	CCND2	DLX1	S100A6																	
CELLMARKERMSIGDB_AIZARANI_LIVER_C12_NK_NK…																									
CELLMARKERMSIGDB_AIZARANI_LIVER_C22_RESID…																									
CELLMARKERMSIGDB_AIZARANI_LIVER_C28_NK_NK…	CD53	FYN	SMCHD1	DUSP4	EVI2A	ANXA1	GATA3	AKNA	PBXIP1	TCF7	EML4	MYBL1	SMAP2	CDC42SE2	TENT4B	TENT5C	RPS27A	STK17A	MAF	CELF2	CD40LG	USP36	PPP2R…	JUNB	RGCC
CELLMARKERMSIGDB_AIZARANI_LIVER_C29_MVECS…	NDRG1	TUBB6	CAVIN1	CRIP2	ABI3BP	MMRN1	CD34	FNIP2	C1QTNF1	LIFR	ITPKC	TCF4	RFK	GJA1	SPRY1	JAM2	CCN1	ADAMTS9	PLPP1	DPYSL3	ADAMTS4	PNP	BMPR2	FILIP1	DKK3
CELLMARKERMSIGDB_AIZARANI_LIVER_C30_HEPAT…	SLC43A2	INSIG1	LINC00…	SLC39A14	SHROOM1	ITIH3	PNPLA3	ARHGEF…	ABCA6	ZFAND5	CYP3A43	LEAP2	SELENOO	C4BPB	CPS1	PLGLB1	DIS3	ZNF283	LINC01…	GPT2	MIRLET…	DDI2	MFSD4B	PTK6	CPEB4
CELLMARKERMSIGDB_AIZARANI_LIVER_C8_RESIDE…																									
CELLMARKERMSIGDB_AIZARANI_LIVER_C9_LSECS_1	CD4	IFITM10	USP13	ADGRL4	KDR	LDB2	RPGR	TINAGL1	ARHGAP…	F8	PDLIM1	DLC1	SEMA6A	RRBP1	MEG3	RAPGEF5	IL1R1	TFPI	MS4A6A	FAM107A	APOLD1	ENG	NR2F2	ZNF462	C1QTN…
CELLMARKERMSIGDB_BUSSLINGER_DUODENAL_BCHE…																									
CELLMARKERMSIGDB_BUSSLINGER_DUODENAL_DIFF…	ASPM	RPL18A	RPL37A	GPX2	HNRNPAB	RPL22	RPL7	RPL13A	RPL5	RRM2	RPL41	AGR2	NPM1	ARL6IP1	CENPF	ANP32B	RPS13	AHCY	CDC42E…	SNHG5	PIGR	RPS15	C1QBP	EMP2	RPS12
CELLMARKERMSIGDB_BUSSLINGER_DUODENAL_EC_C…																									
CELLMARKERMSIGDB_BUSSLINGER_DUODENAL_IMMU…	ARL4C	CD8B	RASSF5	MSN	CD96	ITGAE	AKNA	IKZF1	SPOCK2	FYN	ARHGAP9	IL32	PIP4K2A	ITGAL	ANXA1	LCP2	CD37	CORO1A	LAT	HCLS1	TPSB2	GIMAP7	BCL11B	STK17A	HLA-C
CELLMARKERMSIGDB_BUSSLINGER_DUODENAL_STEM…	RPL5	RPL12	PIK3R1	RPS3	GAS5	RPL30	RPS6	SNHG8	ETS2	RPL31	TMEM123	IER3	EPHB2	RPL34	SH3BP4	RPS4X	RPL41	AQP1	EEF1A1	SNHG6	GPR160	NECTIN3	MYO10	C9orf152	MLEC
CELLMARKERMSIGDB_BUSSLINGER_DUODENAL_TUFT…	CRIP1	ITPR2	GSN	ALOX5AP	ANO7	FARP2	CD24	SOX9	CLMN	SNRNP27	TMEM17…	LUC7L3	PIK3R5	MTSS1	RASSF7	VIPR1	ADH6	TMEM87A	SIGIRR	ADGRG6	ACTN1	BRD4	CHD9	TPM1	GNAI2
CELLMARKERMSIGDB_BUSSLINGER_ESOPHAGEAL_EA…	SRSF3	SLC7A1	KRT15	HES1	IMPA2	HNRNPD	SLC25…	SYNCRIP	TKT	SYNE2	FOS	NPM1	NUCKS1	HMGN1	RPL13A	B2M	ATP5MC3	RPS6	RPL4	HSPA1A	ENO1	RPL5	HSP90…	RPL15	HSPA8
CELLMARKERMSIGDB_BUSSLINGER_ESOPHAGEAL_PR…	RPL13A	NCL	NFIC	KRT5	HNRNPC	EIF4B	EEF2	B2M	ENO1	RPS5	YBX1	RPL4	RPL5	DSC3	RPL18A	RPL15	RPS3	RPL10A	EEF1B2	RPL10	EEF1A1				
CELLMARKERMSIGDB_BUSSLINGER_GASTRIC_CHIEF…	IER2	CST3	FTH1	FTL	JUN	P4HB	EEF2																		
CELLMARKERMSIGDB_BUSSLINGER_GASTRIC_G_CEL…	NKX2-2	PBX3	CXXC4	RFX6	TM4SF5	RAB26	MDK	SEC11C	SLC40A1	KIF1A	TIMP1	CD36	FEV	NOL4	KLB	PCSK1	CAMK2N1	DTNA	HIGD1A	ARFGEF3	MEIS2	PFN2	SAMD5	PLXNA2	ABCB10
CELLMARKERMSIGDB_BUSSLINGER_GASTRIC_ISTHM…	LDLR	MANF	RNU4AT…	SPINK5	ARL6IP1	CLTB	CLDN18	S100A16	TPM4	CENPE	ANO1	SNRPF	LGALS4	ENSG0000…	S100A10	GSN	RAC1	MYH14	HMGB1	DBI	TPM1	FLNB	TK1	SLC7A8	TMPRS…
CELLMARKERMSIGDB_BUSSLINGER_GASTRIC_NECK_…																									
CELLMARKERMSIGDB_BUSSLINGER_GASTRIC_OXYNT…	RIMS2	CAVIN3	APLP1	DPP6	SMOC1	HEPACAM2	ITGBL1	SCG5	DNASE1	PCSK1N	CMIP	TEAD1	LEFTY1	LINC00261	WFDC2	GPR160	FGF14	RUNX1T1	DNER	SEC11C	GC	ARRDC4	ARFGE…	PCLO	FKBP2
CELLMARKERMSIGDB_BUSSLINGER_GASTRIC_PREZY…	ASPH	TTC3	METTL7A	APP	AKAP9	SEC62	CA9	FOS	ATF4	HDLBP	SRRM2														
CELLMARKERMSIGDB_BUSSLINGER_GASTRIC_REG3A…																									
CELLMARKERMSIGDB_CUI_DEVELOPING_HEART_5TH…																									
CELLMARKERMSIGDB_CUI_DEVELOPING_HEART_5TH…	CNN1	SLC30A3	SMCO4	ELAPOR2	SHISA5	ITM2A	PPP2R…	ZFP36	RAB9B	INTS6L	RHOD	BVES	DES	FAM78A	VWDE	BAIAP2L1	DESI1	MYBPHL	DUSP2	ZNF385B	EPM2AI…	TMEM185A	WNK1	PDE8B	CREB3…
CELLMARKERMSIGDB_CUI_DEVELOPING_HEART_C1_…	ASB2	FDPS	CSRP1	CLDN6	TMEM141	CTSV	TM7SF2	H2BC21	BEX2	RCAN1	SNHG17	EMP2	LRRC49	CXCL14	CES1	KLK5	KLK6	TMEM98	ABRA	RSRP1	SCG5	IGFBPL1	MAP1L…	TRH	NEURL2
CELLMARKERMSIGDB_CUI_DEVELOPING_HEART_C2_…	NMRK2	PDE4DIP	MYOM1	MLF1	APOBEC2	NEXN	UNC45B	TECRL	BNIP3	PPP1R1…	NDUFS6	TRDN	ALDOC	POPDC2	GOT1	ATP5MC1	TCAP	BANCR	RRAD	MTUS2	CKMT2	MYH6	HACD1	MYLK3	EIF1B
CELLMARKERMSIGDB_CUI_DEVELOPING_HEART_COM…	FHL1	HAPLN1	MYL2	COL6A6	KLF4	SHISA9	RPS4Y1	GSTT1	PGAM2	MAB21L3	CD34	CNOT11	TSPO	DPT	COL6A1	SPARC									
CELLMARKERMSIGDB_CUI_DEVELOPING_HEART_COR…																									
CELLMARKERMSIGDB_CUI_DEVELOPING_HEART_END…	F2RL2	SCARA3	EPS8	PLA2G5																					
CELLMARKERMSIGDB_CUI_DEVELOPING_HEART_LEF…																									
CELLMARKERMSIGDB_CUI_DEVELOPING_HEART_RIG…																									
CELLMARKERMSIGDB_CUI_DEVELOPING_HEART_SMO…																									
CELLMARKERMSIGDB_DESCARTES_FETAL_ADRENAL_…	TUBBP5	ZNF296	SNORA1…	GTF2H2B	BAK1	VPS9D1-…	HOGA1	NFIA-A…	RNVU1-…	LINC00…	LYG1	MINAR2	C5orf64	CAV3	TTLL9	CBR1-AS1	BRWD1-I…	CLXN	EXOC3-…	ANO7L1	PHB1P19	MRGBP	PRICK…	LINC018…	HDHD5…
CELLMARKERMSIGDB_DESCARTES_FETAL_CEREBELL…																									
CELLMARKERMSIGDB_DESCARTES_FETAL_CEREBELL…																									
CELLMARKERMSIGDB_DESCARTES_FETAL_CEREBELL…	LCP1	NCKAP1L	CSF2RA	FPR1	VAV1	PIK3AP1	RGS10	ITGAM	TBXAS1	CD84	DOCK2	HCK	GPR34	FYB1	IRAG2	CSF1R	BIN2	TMEM144	FGL1	LINC010…	HPSE	IL6R	LAPTM5	SLC38A4	PLEK
CELLMARKERMSIGDB_DESCARTES_FETAL_CEREBELL…	SHH	ST13P20	LINC02…	KLF5	GNAT3	LINC023…	LINC0…	AP1M2	LINC03…	LINC01…	SLCO4C1	ARG1	LOXL1-AS1	DCDC2C	CYP4Z2P	ENSG00000…	LINC015…	ATP2B2-IT1	POLR2D…	ITPR1-DT	LINC00…	MED28P8	METTL…	AIPL1	LINC0…
CELLMARKERMSIGDB_DESCARTES_FETAL_CEREBELL…	ENSG000…	RNA5SP1…	LINC02…	RGS13	ENSG00…	LIPN	KLF2P2	CLC																	
CELLMARKERMSIGDB_DESCARTES_FETAL_CEREBELL…																									
CELLMARKERMSIGDB_DESCARTES_FETAL_CEREBELL…	CGNL1	CCDC141	ABCB1	ITIH5	ITM2A	FN1	RGS5	TM4SF1	SLC38A…	AFAP1L1	TBX3	SERPIN…	ENSG0000…	ECM1	LINC02147	TGM2	HIC1	ANXA1	MMP25	SLC7A5	SLCO1A2	CD248	KCNJ8	LAMC3	SOX7
CELLMARKERMSIGDB_DESCARTES_FETAL_CEREBRUM…																									
CELLMARKERMSIGDB_DESCARTES_FETAL_CEREBRUM…																									
CELLMARKERMSIGDB_DESCARTES_FETAL_CEREBRUM…	FERMT3	THOC2	LRP12	MFAP3L	F2RL3	DAPP1	MYO3B	ITGA2B	PKN3	SH3BGR…	TREML1	TMEM91	ATP2A3	CMTM5	DOCK5	GP9	LINC005…	DMTN	MEPCE	TGFBRAP1	TUBB1	MARCHF2	KIAA0…	UNC13D	SLC44…
CELLMARKERMSIGDB_DESCARTES_FETAL_CEREBRUM…	FMN1	ATP8B4	LRRK1	SLC7A7	CPVL	BHLHE41	B3GNT5	SPP1	CD4	GLIPR1	CD83	CSF1	P3H2	CTSS	RTTN	P2RY12	GAL3ST4	S100Z	AP1B1	WDFY4	RUNX2	APBB1IP	SYT6	HAVCR2	PPARG
CELLMARKERMSIGDB_DESCARTES_FETAL_CEREBRUM…	MOBP	MOG	LINC02…	CHRM3-A…	ASIC4-…	KLRC4	TMEM2…	HMX1	GDF6	TNR-IT1	GDNF	KLRK1	DMRT2	CXCL13	RN7SL795P	NR0B1	LINC015…	ATP13A5-A…	MATN1	TMEM235	VSX1	DCAF4L2	FCRLA	GJB1	RNU6-…
CELLMARKERMSIGDB_DESCARTES_FETAL_CEREBRUM…	FOXP4-A…	ZNF385D…	DNAJC5G	ST13P20	TRIM54	LINC023…	SUDS3…	DAB1-A…	ARG1	YBX2P1	LINC01…	FGF3	SPINT1-A…	ATP2B2-I…	POLR2DP2	ENSG00000…	RSU1P3	MED28P8	LINC00…	HAR1A	ENSG00…	TRIM60P…	ATP2B…	AIPL1	RNA5S…
CELLMARKERMSIGDB_DESCARTES_FETAL_CEREBRUM…	TGM2	THBD	PRELP	UNC5B	SPON2	KANK3	COL3A1	KCNJ8	OLFML2A	CAVIN1	KLF4	EFNA1	MECOM	ETS1	PCDH18	IGF2	HEYL	ARHGAP29	TEK	EDNRA	SLC9A3…	SLC7A5	SLCO1…	RGS5	MELTF
CELLMARKERMSIGDB_DESCARTES_FETAL_EYE_ASTR…																									
CELLMARKERMSIGDB_DESCARTES_FETAL_EYE_GANG…	IL12A																								
CELLMARKERMSIGDB_DESCARTES_FETAL_EYE_LENS…	MYO18A	LINC018…	UPP1	KMO	DRG1P2	SLC27A6	CRYGB	WNT7A	GJA8	SQOR	HIF1A-…	GACAT3	PITHD1	CARD14	TMEM114	LINC02763	ARSI	CHRNA2	MIR193…	P3H2-AS1	SORD	HSPA6	TRPM8	TDH	LINC0…
CELLMARKERMSIGDB_DESCARTES_FETAL_EYE_PHOT…	LINC010…	LINC020…	LGALS9…	UNCX	SNORA75	COX5BP2	LINC0…	EGFLAM…																	
CELLMARKERMSIGDB_DESCARTES_FETAL_EYE_RETI…	ATP6V1C2	LINC006…	ADTRP	MYOCD	SLC6A12	KCNQ4	SLC45…	PRODH	CCDC33	LMNTD1	ADRA2C	ENSG00…	OR51E2	PRKCQ-AS1	TMEM72	ENSG00000…	TMEM235	CLDN19	LINC00…	LINC012…	FAM83B	PGR	PFKFB2	ERMN	MFRP
CELLMARKERMSIGDB_DESCARTES_FETAL_EYE_SMOO…	DNAI3	PYY2	CDNF	BORCS7-…	CBLN1	SNHG8	VMA21	ANKRD33	DEDD	SPRING1	SPIN4	ETNK2	ZNF665	RPL17P9	NAMA	LAT	ZNF384	AP5Z1	SLC22A…	ASPA	COQ3	BMP5	PABIR1	SNHG1	ITGA2…
CELLMARKERMSIGDB_DESCARTES_FETAL_EYE_STRO…	ADAM33	ADGRG2	ITGBL1	NDNF	TNFAIP6	EDNRA	PDGFRL	C1QTNF7	OMD	PCDH18	PENK	TRABD2B	VASN	OLFML1	EMILIN3	FGF18	INHBA	MEDAG	CA12	ITGB3	PODN	IL11RA	TWIST2	PRRX2	KDELR3
CELLMARKERMSIGDB_DESCARTES_FETAL_EYE_VASC…	ZNF366	MYCT1	TAMALIN	ADORA2A…	GIMAP4	DIPK2B	CCM2L	S1PR1	NOTCH4	RASIP1	TCIM	RXFP1	ABCB1	CETP	CYP26B1	QRFPR	ATG9B	PCAT19	ADGRL4	BEND4	GIMAP1	TINAGL1	APOL3	GGT5	SOX7
CELLMARKERMSIGDB_DESCARTES_FETAL_HEART_EL…																									
CELLMARKERMSIGDB_DESCARTES_FETAL_HEART_EN…																									
CELLMARKERMSIGDB_DESCARTES_FETAL_HEART_LY…																									
CELLMARKERMSIGDB_DESCARTES_FETAL_HEART_SC…	BMP8B	LINC026…	GPR17	MMD2	CLDN14	ARSL	CFAP61	ST3GAL…	ANGPTL7	PP7080	COL22A1	MIR663…	ARTN	MAG	HSPB2-C1…	LINC01198	CNMD	CARMAL							
CELLMARKERMSIGDB_DESCARTES_FETAL_HEART_SM…																									
CELLMARKERMSIGDB_DESCARTES_FETAL_HEART_ST…																									
CELLMARKERMSIGDB_DESCARTES_FETAL_HEART_VI…	VGF	TMEM255A	TMC3	SLC10A4	ST8SIA3	DDC	GRM8	FAM167A	MAB21L1	DBH	PHOX2A	NPY2R	CAMK2N2	ISL1-DT	SLC6A2	PHOX2B-AS1	CHRNB4	LINC01561	TH	ZCCHC12	LGI3	MAB21L2	CHRM1	GALR1	SEMA4F
CELLMARKERMSIGDB_DESCARTES_FETAL_INTESTIN…	GGT8P	CMTM5	LINC01…	KCNA2	ENPP7P2	LMO1	CHST6	ARTN	NCAPGP1																
CELLMARKERMSIGDB_DESCARTES_FETAL_INTESTIN…																									
CELLMARKERMSIGDB_DESCARTES_FETAL_INTESTIN…	WNT10A	ADIRF	SILC1	TNS2-AS1	CARNS1	SMTNL2	ARL4D	PTGDS	KLK10	ANAPC2	MUC16	CERS3-…	NECAB1	QRFPR	LRRN4	WT1-AS	TGM1	SNRPA1-DT	WNT7B	ICA1-AS1	TM4SF1…	PCOLCE2	TNFRS…	TRIM6	CEMP1
CELLMARKERMSIGDB_DESCARTES_FETAL_INTESTIN…	RNU7-47P	PPIC-AS1																							
CELLMARKERMSIGDB_DESCARTES_FETAL_INTESTIN…																									
CELLMARKERMSIGDB_DESCARTES_FETAL_INTESTIN…																									
CELLMARKERMSIGDB_DESCARTES_FETAL_KIDNEY_M…	ELOVL3	NDP	LINC02…	GNG8	GOT1L1	LINC028…	RNU6-…																		
CELLMARKERMSIGDB_DESCARTES_FETAL_KIDNEY_U…	HPGD	SHROOM1	SPINT1	LYPD6B	LINC00…	MAP3K21	PPFIB…	MYH14	SDK1	KRT18	NIPAL1	COBLL1	IL1RAPL2	RAB11FIP2	RET	TMPRSS2	LOC2832…	PPL	ANXA3	SDC1	ACOXL	PERP	RASEF	CLIC6	SPTLC3
CELLMARKERMSIGDB_DESCARTES_FETAL_LIVER_ER…	ENSG000…	THEGL	TSPAN17	PPT2	ODAD4	LINC013…	ENSG0…	TDH	A4GALT	XG	ERFE	ABCB5	KRT13	HDC	HBBP1	ACKR1	LINC024…	SEC14L3	SHISA7	ERVE-1	ASIC4	ANKRD61	FKBPL	RNU6-87P	RPS12…
CELLMARKERMSIGDB_DESCARTES_FETAL_LIVER_HE…																									
CELLMARKERMSIGDB_DESCARTES_FETAL_LIVER_HE…	SERPINA1	PTP4A1	FGB	SLC39A14	RBP4	CYP3A7	CYP4F3	ERBB3	ITIH2	AHSG	MT1H	ITIH1	GC	AGT	CPB2	APOA2	SLC38A3	SLC7A2	SLC2A2	ALB	DUSP9	MTTP	APOB	APOC3	HPX
CELLMARKERMSIGDB_DESCARTES_FETAL_LIVER_ME…	ERBB4	WT1	SPOCK2	COL11A1	MYO5B	ITGA3	PPM1E	DPP4	UPK3B	BNC1	DISP1	NTNG1	SMPD3	CDH3	ZBTB7C	CCDC80	GAS6	GXYLT2	CRIP1	MIR100HG	LINC02…	C21orf62	PTPRT	BOC	MGP
CELLMARKERMSIGDB_DESCARTES_FETAL_LUNG_BRO…	DMBT1	LHFPL3-…	SP5	TEPP	SLC10A2	PGC	ODAM	SMIM43	RBBP8NL	LINC01…	LMO7DN…	LINC01…	ENSG0000…	RPS29P11	LHFPL3-A…	LINC01426	H1-9P	GGT2P	GPX6	PLCZ1	SLC34A3				
CELLMARKERMSIGDB_DESCARTES_FETAL_LUNG_CIL…	CFAP126	WDR49	CFAP251	ECT2L	AGR3	DNAH11	CFAP45	CIBAR2	LRRC46	LRRIQ1	ANKFN1	SAXO2	PIFO	C6orf118	PPP1R42	CFAP54	ODAD2	DLEC1	FOXJ1	ERICH3	CLXN	DRC3	FANK1	SLC44A4	ARMC3
CELLMARKERMSIGDB_DESCARTES_FETAL_LUNG_LYM…	D21S208…	KRT222	DAND5	GJD3	LINC02…	SLC5A1	PHF24	HOXD10	LRRC15	LINC02…	CASP12	CYP2AC…	LRCOL1	LOC12490…	CH25H	TM4SF18-A…	LRRC70	ENSG00000…	NETO1	POU4F1	ACSM2B	SLC30A3	HOXD4	GLRA4	LINC0…

CELLMARKERCAN_ACINAR_CELLS																									
CELLMARKERMSIGDB_DESCARTES_FETAL_LUNG_NEU…	VGF	BTBD17	CHRD	POTEJ	CHDH	QPCT	OR51E1	LINC02…	LMX1B	RIMKLA	A1CF	SLC16A…	PGAM2	PHGR1	TFF1	POTEI	NR0B2	SFRP4	FIBCD1	TMPRSS6	KCNIP3	TTR	ASCL2	RAB26	TMEM1…
CELLMARKERMSIGDB_DESCARTES_FETAL_LUNG_STR…	MIR1245A	ENSG000…	RGS13	LINC024…	MIR218…	LSAMP-A…	MMP23B																		
CELLMARKERMSIGDB_DESCARTES_FETAL_LUNG_VAS…	RNGTTP1	MUC19	RNU6-5…	S100A3																					
CELLMARKERMSIGDB_DESCARTES_FETAL_MUSCLE_L…	TMEM217	GSK3B-DT	TMEM63C	ASGR2	KLHL14	GCOM1	ABCA4	FAM120…	PEX16	RPL26L…	HAND2	CEP19	SFTA1P	LINC00920	KCTD17	GPRIN1	PIK3C2G	LINC02133	SEC14L2	MFSD4A	NHLRC1	ZNF436-…	LINC0…	CALHM6	SLCO5…
CELLMARKERMSIGDB_DESCARTES_FETAL_MUSCLE_S…	GDPD4	LINC012…	ADAMTS…	RNU6-13…	CNGA3	OPRD1	ENPP7…	MT1M	RN7SL3…																
CELLMARKERMSIGDB_DESCARTES_FETAL_MUSCLE_S…	CORIN	ADRA1D	KIAA15…	LINC022…	NRIP2	SLC6A1	CABP1	TMC4	TMEM74B	ADORA2B	RAB3B	LAMC3	SEPTIN4-…	KCNA5	CCN4	CACNA1C-I…	NKD2	TRPC4	ACTA2-…	SLC7A11	P2RX3	B4GALNT1	ENTPD3	PDE1B	WDR72
CELLMARKERMSIGDB_DESCARTES_FETAL_MUSCLE_V…	INMT	SLC5A4	PTPRR	CYYR1-A…	RAMP3	TMEM255B	ENSG0…	RAB3C	ACKR1	CD300LG	SELE	AVPR2	ENSG0000…	ESM1	LINC01697	PRR29	LIPE	GFRAL	GRASLND	NOS2	LY86-A…	RBP7	TPO	LIMASI	F2RL2
CELLMARKERMSIGDB_DESCARTES_FETAL_PANCREAS…	PRR35	PRSS16	MIOS-DT	CCKAR	GSTA1	FREM2-A…	KCNE5	CNPY1	CCKBR	HTR1E	TMED11P	NCCRP1	CLPSL1	MT1M	APOB	BCL2L15	ADAM7-A…	MEP1A	ABCC6P1	FABP1	FAM87A	MYMX	MT1HL1	AKR7L	LYPLA…
CELLMARKERMSIGDB_DESCARTES_FETAL_PANCREAS…	FMO6P	SPIC	LRRC37…	CILP2	TNFRSF…	C1GALT1…	KPNA2…	MIR367…	LINC02…	IGFN1	GAL3ST3	WDR97	ENSG0000…	OLA1P1	LOC10192…	SNORD116-…	HTD2	CYP51A1P2							
CELLMARKERMSIGDB_DESCARTES_FETAL_PANCREAS…	MMD2	PMP2	RBMS3-…	LINC020…	FOXD3-…	GPR83	GJC3	WNT16	DEPDC7	POLR3G	AZGP1	DCT	LINC02082	ZMYM4-AS1	PAPPA-AS1	LINC01198	VSTM2B-…	GPR12	GPR17	LINC006…	LINC02…	PLAC9P1	MYOT	HCRTR1	RPL13…
CELLMARKERMSIGDB_DESCARTES_FETAL_PANCREAS…	GABRP	RNU1-10…	MYCBPAP	CCL20	RPS7P4	RPAP3-DT	DDX59…	VPS26B…	RPS26P…	HOXD10	RPS15A…	KRT18P…	FLNC-AS1	CACNA2D1…	RSF1-IT1	P3H2-AS1	IL5RA								
CELLMARKERMSIGDB_DESCARTES_FETAL_PANCREAS…	KLK11	ALOX15	GSDME	MUC16	OTOGL	GNGT1	ROR1-…	CPA4	EXTL1	FAM13A…	NEK5	LINC02…	SERTAD4-…	SERTM1	LINC03092	CELF2-AS2	KLK5	LINC00922	SCEL	LINC012…	SGSM3-…	PLA2G10	RAET1E	SI	SPHKAP
CELLMARKERMSIGDB_DESCARTES_FETAL_PANCREAS…																									
CELLMARKERMSIGDB_DESCARTES_FETAL_PANCREAS…	SCN4A	VSIG2	FCN3	SPAAR	TM4SF1…	MIR126	AQP7	ADM5	C1QTNF9	RP1L1	HAPLN1	FOXI2	MALL	LINC01936	ACSM2B	CRYBB3									
CELLMARKERMSIGDB_DESCARTES_FETAL_PLACENTA…	LINC003…	SLC17A8	POMC	GKN1	CILP2	FAM43B	LY6D	KCNJ15	NOS2	RN7SL8…	MAG	SOWAHD	DAW1	LINC01819	DEPDC7	IGFL2-AS1	KISS1R	WFDC3	KLRG2	PDE6H	BEST3				
CELLMARKERMSIGDB_DESCARTES_FETAL_PLACENTA…	LINC028…	GFRA2	C9orf1…	MAPT	KCNK17	HEYL	BMPER	HAS2	ADRB3	NGFR	REN	CDH19	RASL12	HIGD1B	ANKRD30A	FAM182B	ABCC9	SEPTIN4	COLEC10	LINC020…	SEMA5B	PGM5	ADGRB1	HECW2-A…	ENSG0…
CELLMARKERMSIGDB_DESCARTES_FETAL_PLACENTA…	FMO1	CDH15	HS3ST6	LINC011…	SLC18A3	RNU7-11…	SYT15	GDNF-A…	RTL9	GDNF	FGF10-…	C1QTNF2	WSCD2	OACYLP	PKP1	PON3	LINC027…	ENSG00000…	LOC105…	NGF	NCKAP5…	SFTPD	ENSG0…	MMP23B	MYOD1
CELLMARKERMSIGDB_DESCARTES_FETAL_PLACENTA…	RFX6	LINC011…	KCNG1	SLC28A2	ZBED9	LINC012…	POF1B	TUBA5P	GPR87	OVCH2	GABRA4	NALCN-…	BFSP2-AS1	SLC52A3	ZYG11A	ANKRD65	POTEKP	CYP4F35P	LINC01…	APELA	GCNT4	LINC022…	FTLP14	MAST4-A…	FAM41C
CELLMARKERMSIGDB_DESCARTES_FETAL_PLACENTA…	PSG7	LGALS13	CRYBG2	LINC004…	TPRXL	ENSG000…	ADHFE1	ERV3-1	TREML2	LYPD5	LINC02…	TMEM54	CPXM2	OLAH	LINC00967	SLC26A7	PSCA	HS3ST3B1	ZNF468	SH2D5	LINC01…	LINC011…	TCHH	LGALS16	IL22R…
CELLMARKERMSIGDB_DESCARTES_FETAL_SPLEEN_E…	CDC25A	EPCAM	SLC5A4…	CDK15	C16orf…	ACHE	ST8SI…	SPAG17	PRG2	PLPP4	RWDD3-…	TSPO2	TYMSOS	LONRF3	EPS15-AS1	ENSG00000…	DNAJA4	SLC25A15							
CELLMARKERMSIGDB_DESCARTES_FETAL_SPLEEN_S…	KBTBD11…	MTMR12P1	NPY5R	CDH7	MIR124…	LINC019…	MYHAS																		
CELLMARKERMSIGDB_DESCARTES_FETAL_SPLEEN_V…	RFX8	MIR34AHG	ODF3L2	LINC015…	TACR1	TRPM6	EEF1A…	ETS2-A…	B3GNTL…	PRSS48															
CELLMARKERMSIGDB_DESCARTES_FETAL_STOMACH_…	RGS22	SPEF2	CFAP251	C8orf34	TEKT1	CCDC146	NEK11	ANKUB1	FOXJ1	CCDC60	SPATA6L	ODF2L	CFAP44	KIAA0825	DNAI7	FAM227A	CDHR4	DNAI4	CFAP45	VWA3B	MAP3K19	FABP6	ECT2L	PIFO	DRC1
CELLMARKERMSIGDB_DESCARTES_FETAL_STOMACH_…																									
CELLMARKERMSIGDB_DESCARTES_FETAL_STOMACH_…	NPY	SYT5	UCHL1	TUBB2A	ARPP21	PCDH15	OLFM3	NWD2	ACTL6B	LRFN2	ADCYAP…	CNGB1	RGS11	LINC02641	NKAIN1	CELF5	HCN1	ATP1A3	NDRG4	TCEAL5	WDR5B	TMEM255A	LINC0…	ZNF77	SCN11A
CELLMARKERMSIGDB_DESCARTES_FETAL_STOMACH_…	TRAT1	ZAP70	KLRB1	MATK	NKG7	TMIGD2	AGAP2	EPHA1-…	LAT	PAX5	PCED1B…	SIRPG	CD79B	MS4A1	CD79A	SASH3	SHISAL2A	LTA	CTSW	RAC2	GZMM	ICAM3	S1PR4	PYHIN1	MYO1G
CELLMARKERMSIGDB_DESCARTES_FETAL_STOMACH_…																									
CELLMARKERMSIGDB_DESCARTES_FETAL_STOMACH_…	EPS8L3	BAIAP2L2	ADGRG7	BTNL3	PIGR	VIPR1	MT1F	CDX1	LHFPL3…	SLC6A20	GPA33	TRPM6	ISX	SLC5A12	MOGAT2	SLC26A3	TRIM31	CHP2	FZD5	GUCA2B	CREB3L3	GCNT3	IYD	PDZD7	ENPP7
CELLMARKERMSIGDB_DESCARTES_FETAL_STOMACH_…	PLEK	CD300A	CCDC26	HLA-DMB	ADGRE2	MS4A7	CD86	NCF2	FLT3	IGSF6	HACD4	LTV1	CSF3R	PTAFR	TYROBP	LINC00996	IDO1	TRPM2	GCA	ALOX5	LY86	MS4A4E	S100A8	NAPSB	IFI30
CELLMARKERMSIGDB_DESCARTES_FETAL_STOMACH_…	DPEP1																								
CELLMARKERMSIGDB_DESCARTES_FETAL_STOMACH_…																									
CELLMARKERMSIGDB_DESCARTES_FETAL_STOMACH_…	KRT17	GJB6	SERPIN…	CARD14	GPR87	FOXN1	UPK1A	CLCA2	ADH7	DLK2	JMJD7	SERPIN…	S100A3	KRT16	CYP11A1	TMPRSS11B…	DSG1-AS1	FAM83C							
CELLMARKERMSIGDB_DESCARTES_FETAL_STOMACH_…	PI16																								
CELLMARKERMSIGDB_DESCARTES_FETAL_STOMACH_…																									
CELLMARKERMSIGDB_DESCARTES_FETAL_THYMUS_T…																									
CELLMARKERMSIGDB_DESCARTES_MAIN_FETAL_ACI…	CTRB1	LOC1005…	ZC3H18	DPEP1	HEXD	KIRREL2	SYCN	KLK1	MATN4	RBPJL	MID1IP1	SSR4													
CELLMARKERMSIGDB_DESCARTES_MAIN_FETAL_ADR…	FDX1	GRAMD1B	SLC2A14	LINC012…	GNS	SCARB1	SLC46…	CIDEB	RDH12	GALC	CYP11A1	OTOA	OGFOD1	BBS2	USB1	AGRP	CYB5B	OSGIN1	SLC47A1	SNORA59B	ALDH3A2	GRIN2C	FDXR	FOXK2	RNA5S…
CELLMARKERMSIGDB_DESCARTES_MAIN_FETAL_AST…	ENSG000…	RNGTTP1	GPM6B																						
CELLMARKERMSIGDB_DESCARTES_MAIN_FETAL_BIP…	NFIB	TMEM215	RN7SL7…	CYP2C8	CABP2	RPL31P46	RSF1-…	ENSG00…	CACNA2…	FAM234B	GSG1	LINC02…	WASF3-AS1	B3GLCT	SERTM1	ENSG00000…	VSX2	GABRB3	SV2B	C15orf32	YPEL3-…	TMEM132E	CA10	SMIM36	ENSG0…
CELLMARKERMSIGDB_DESCARTES_MAIN_FETAL_BRO…	SUSD2																								
CELLMARKERMSIGDB_DESCARTES_MAIN_FETAL_CAR…																									
CELLMARKERMSIGDB_DESCARTES_MAIN_FETAL_CIL…	CFAP141	THBS3-A…	LRRC71	VSIG8	CFAP45	KLHDC9	UFC1	CFAP126	CCDC190	CCDC181	MROH9	SLC9C2	ANKRD45	APOBEC4	SERTAD4-…	NEK2-DT	SPATA17	ACBD3-AS1	EFCAB2	MIR7515…	C2orf50	WDR35	DRC1	FAM166C	MAPRE3
CELLMARKERMSIGDB_DESCARTES_MAIN_FETAL_DUC…	PDE3A-A…	FBXO21	CLDN10	LINC012…	VASH1-…	LCIIAR	GREP1	CLDN9	HSD3B7	PPP1R1B	RNU6-2…	ACBD4	ROCR	LINC01152	H3-3B	CBX3P2	MPND	NRTN	ISYNA1	LZTS3	SALL4	LRRC75B			
CELLMARKERMSIGDB_DESCARTES_MAIN_FETAL_END…	MANSC1	SMCO3	PTHLH	LRIG3	POSTN	CGNL1	SMAD6	TLNRD1	CDH11	TNFSF12	TNFAIP1	SLC6A4	CPD	RAMP2	TMEM100	SMAD7	CALR	NUCB1	THBD	PROCR	NORAD	APCDD1L	MCHR1	CD99	BMX
CELLMARKERMSIGDB_DESCARTES_MAIN_FETAL_ENS…																									
CELLMARKERMSIGDB_DESCARTES_MAIN_FETAL_EPI…	KLK5	KLK7	LRRN4																						
CELLMARKERMSIGDB_DESCARTES_MAIN_FETAL_GAN…																									
CELLMARKERMSIGDB_DESCARTES_MAIN_FETAL_GOB…	TEX14	MYADM-A…	TFF2	VSIG1																					
CELLMARKERMSIGDB_DESCARTES_MAIN_FETAL_HEM…	IRAK3	FAM216A	TAOK3	ENSG000…	RPL21P…	LINC023…	TIPIN	LOC100…	ABCC1	ENSG00…	LOC105…	SPNS3	STAT5A	LRRC37A4P	MPO	HSF5	PRSS57	AZU1	PRTN3	ELANE	PRAM1	LINC029…	NRIP1	CLDN14-…	DDTL
CELLMARKERMSIGDB_DESCARTES_MAIN_FETAL_HOR…																									
CELLMARKERMSIGDB_DESCARTES_MAIN_FETAL_INH…																									
CELLMARKERMSIGDB_DESCARTES_MAIN_FETAL_INH…																									
CELLMARKERMSIGDB_DESCARTES_MAIN_FETAL_INT…	ACSL5	LHPP	NLRP6	MUC2	SLC5A12	RPLP0P2	ACY3	APOA4	PDZD3	FGF23	MYO1A	RNU6-3…	CDX2	URAD	MIR4307HG	LINC02323	STRC	WHAMM	SLC28A1	TPSG1	CA7	SLC13A2	TMIGD1	SPACA3	ANKRD…
CELLMARKERMSIGDB_DESCARTES_MAIN_FETAL_ISL…	LINC021…	PABPN1L	ACLY	PPY	BTBD17	TTR	PLD3	SYNGR4	NKX2-2	NOL4L-…	LINC01…	MAFB	GNAS	CCDC188	CABP7	C22orf42	ASB9	ARX	PCSK1N	RTL5					
CELLMARKERMSIGDB_DESCARTES_MAIN_FETAL_LYM…	LYVE1	PRR5L	RAB3IL1	MAP4K2	PDE2A	CASP12	PPFIB…	GPR182	RASSF9	NTS	ELK3	STAB2	LOC12490…	DTX1	TMEM255B	RHOJ	CYP46A1	TNFAIP8L3	PLEKHO2	ANKDD1A	ZP2	ADGRG3	PIEZO1	GRAP	GRAPL
CELLMARKERMSIGDB_DESCARTES_MAIN_FETAL_MES…																									
CELLMARKERMSIGDB_DESCARTES_MAIN_FETAL_MET…																									
CELLMARKERMSIGDB_DESCARTES_MAIN_FETAL_MYE…	SH2B3	SDS	RILPL2	GPR183	RNASE6	SNX6	LGMN	DMXL2	PYCARD	SCIMP	CLEC10A	ABI3	CD300LB	CD300C	LINC01478	MCOLN1	RETN	CD209	MAN2B1	IFI30	LRRC25	TYROBP	PLAUR	RN7SL36…	C5AR2
CELLMARKERMSIGDB_DESCARTES_MAIN_FETAL_NEU…																									
CELLMARKERMSIGDB_DESCARTES_MAIN_FETAL_OLI…	LINC022…	LINC022…	LINC00…	EPN2	CHAD	ENSG000…	NETO1…	DLL3	LINC00…	BCAS1	OLIG2	OLIG1	CASK	NLGN4Y											
CELLMARKERMSIGDB_DESCARTES_MAIN_FETAL_PDE…	IQSEC3	HMGB1P49	SCN8A	OSBPL9P4	PPFIA2	CABP1	CNMD	PCDH9	PCDH9-…	PCDH9-…	ARHGEF…	RNU6-8	DAAM1	LINC02259	RBFOX1	RNU7-99P	RNU6-45…	GRIN2A	ENSG00…	ADAMTS18	KRT17P1	HLF	KLHL14	SNAP25	PAK3
CELLMARKERMSIGDB_DESCARTES_MAIN_FETAL_PDE…	CCNT2	DARS1-A…	CCDC14…	INO80D	SLC23A3	TRNT1	THRB-…	RBMS3-…	RPL21P…	ZNF660	ZDHHC3	LIMD1-…	PTPN23	FBXW12	USP19	STIMATE-M…	SLMAP	FOXP1	FOXP1-…	C3orf52	ZBTB20…	RAB43	H1-10…	COL6A4P2	DBR1
CELLMARKERMSIGDB_DESCARTES_MAIN_FETAL_RET…	GABRG3	DUT-AS1	TOX3	ADORA2B	AOC2	CASC17	LINC0…	RPL30P…	PWWP3A	VSTM2B	APCDD1…	LINC00…	LINC01697	ADARB1	CECR3	PCYT1B									
CELLMARKERMSIGDB_DESCARTES_MAIN_FETAL_SAT…	LINC009…	LMF1-AS1	SOX8	CDH15	SLC22A…	CHRNB1	KRT40	KRT31	LMNB2	NTN5	CDC25B	EDN3	P2RX6	LINC01283	CLCN5	FGF16	LPAR4	LDOC1							
CELLMARKERMSIGDB_DESCARTES_MAIN_FETAL_SCH…	ITIH6	PLP1																							
CELLMARKERMSIGDB_DESCARTES_MAIN_FETAL_SLC…	LYG1	IL1A	NIFK	SMPD4BP	HIBCH	HECW2-A…	C2CD6	KANSL1…	GMPPA	EPHA4	UICLM	IL17RC	CIDECP1	ZNF385D-…	ACVR2B-A…	PTPN23-DT	SPINK8	CACNA1D	SELENOK	PDE12	GBE1	ZBTB11-…	DBTP1	WDR5B	TPRA1
CELLMARKERMSIGDB_DESCARTES_MAIN_FETAL_SMO…																									
CELLMARKERMSIGDB_DESCARTES_MAIN_FETAL_STE…	DIO3OS	DIO3	LINC01…	RPS2P44	HS3ST3…	SEPTIN4…	BCAS3…	ABCA10	ROCK1P1	CCDC68	CCBE1	ANGPTL6	UPB1	RPS6KA6											
CELLMARKERMSIGDB_DESCARTES_MAIN_FETAL_SYM…	CHGB	CFAP61-…	SMIM45	TSPYL2	SERTM2	IL13RA2	HTATS…	LINC00…																	
CELLMARKERMSIGDB_DESCARTES_MAIN_FETAL_UNI…																									
CELLMARKERMSIGDB_DESCARTES_MAIN_FETAL_URE…																									
CELLMARKERMSIGDB_DESCARTES_MAIN_FETAL_VAS…	APLN	EEF1A1P…																							
CELLMARKERMSIGDB_DESCARTES_MAIN_FETAL_VIS…	LINC027…	CHRM1	PPP2R5B	RNU6-67…	ENSG00…	HEPHL1	NCAM1…	SCN2B	CCDC184	PRPH	LINC03…	PCOTH	RNA5SP383	RAB15	YBX1P1	PERPP2	ARHGDIG	GNAO1	NECAB2	TUBB3	RN7SL7…	C17orf1…	PIRT	VAT1	ENSG0…
CELLMARKERMSIGDB_DURANTE_ADULT_OLFACTORY_…																									
CELLMARKERMSIGDB_DURANTE_ADULT_OLFACTORY_…																									
CELLMARKERMSIGDB_DURANTE_ADULT_OLFACTORY_…																									
CELLMARKERMSIGDB_DURANTE_ADULT_OLFACTORY_…	TNFAIP3	ALOX5AP	STK17B	SH3BGRL3	ID2	LSP1	RGS1	PTGER4	TMSB4X	GMFG	LCP1	SYTL3	RAC2	TRAF3IP3	RUNX3	LAPTM5	TBC1D10C	PTPN22	IL2RG	JAML	CD96	ARL4C	CKLF	RORA	
CELLMARKERMSIGDB_DURANTE_ADULT_OLFACTORY_…	THY1	CYP1B1	MXRA8	ACTA2	HGF	TIMP1	TGFBI	CCL2	AKAP12	TCIM	CH25H	TPM1	CDH11	TFAP2B	PAX9	SYNPO2	ABCA8	TIMP2	AEBP1	VIM	SPARC	GSN	ENPP2	TFAP2A	IGFBP5
CELLMARKERMSIGDB_DURANTE_ADULT_OLFACTORY_…																									
CELLMARKERMSIGDB_DURANTE_ADULT_OLFACTORY_…	FCGR2A	CSF1R	CXCR4	CLEC7A	CTSS	IFI30	CXCL16	ITGB2	CCL4L2	LY86	CYBA	FCGR3A	CX3CR1	CSF2RA	C3	MSR1	TREM2	CLEC10A	RAB32	P2RY13	C3AR1	GRN	HERPU…	C15orf48	TYMP
CELLMARKERMSIGDB_DURANTE_ADULT_OLFACTORY_…	PLIN2	HSPA1B	NFKBIZ	IER2	ELF1	DNAJB1	SOCS1	ARHGAP…	NR4A3	CAVIN2	SDCBP	ADRB2	CD44	EGR3	TNFAIP3	SGK1	AREG	CCL4	CREM	ZNF331	ANKRD28	DUSP6	DDIT4	DUSP2	CCL2
CELLMARKERMSIGDB_DURANTE_ADULT_OLFACTORY_…																									
CELLMARKERMSIGDB_DURANTE_ADULT_OLFACTORY_…	CD53	HLA-A	IL2RG	HLA-B	SRGN	PLAC8	ARL4C	IFITM2	IL32	CYBA	TRAF3I…	ARHGDIB	PFN1	SH3BGRL3	MYL12A	CD48	CXCR4	LIMD2	CD52	APOBEC3G	CCL4L2	LSP1	EMP3	EVL	RAP1B
CELLMARKERMSIGDB_DURANTE_ADULT_OLFACTORY_…	PBXIP1	MGST1	RARRES2	AZGP1																					
CELLMARKERMSIGDB_DURANTE_ADULT_OLFACTORY_…	PECAM1	PLAT	CRIP2	JAM2	SPTBN1	PLVAP	ESAM	FAM13C	TXNIP	RDX	EMP1	IGFBP4	FAM110D	NOSTRIN	LPAR6	C2CD4B	CXCL12	RUNDC3B	TIMP3	CALCRL	KCTD12	CD93	TMEM88	CD74	TCF4
CELLMARKERMSIGDB_DURANTE_ADULT_OLFACTORY_…																									
CELLMARKERMSIGDB_DURANTE_ADULT_OLFACTORY_…																									
CELLMARKERMSIGDB_DURANTE_ADULT_OLFACTORY_…																									
CELLMARKERMSIGDB_DURANTE_ADULT_OLFACTORY_…	ISYNA1	CAVIN3	IGFBP7	SEPTIN7	STEAP4	SOCS3	LMOD1	MT2A	RCAN2	MT1M	SYNPO2	IGFBP5	LPP	PPP1R12A	PLAC9	MAP3K20	FOS	CARMN	CNN1	SPARCL1	CAVIN1	ZFP36	HSPA1A	CRYAB	KANK2
CELLMARKERMSIGDB_FAN_EMBRYONIC_CTX_ASTROC…	PRSS35	IFI44L	EFHD2	FADS2	CPNE5	MRC2	ID3	ID1	SOAT1	ANOS1	C1QTNF3	LPCAT1	TCIM	LAMP5	DCN	TM7SF2	HES4	FRMD4A	PLEC	SYPL1	LSS	CDH4	HTR2A	OAF	LRRC1…
CELLMARKERMSIGDB_FAN_EMBRYONIC_CTX_BIG_GR…	KDR	LAMA4	ADGRL4	ECM1	DIPK2B	LXN	MCAM	DLC1	PCAT19	SLCO2A1	SERPIN…	ADA	CAVIN2	S1PR3	CDH5	ANPEP	CCDC3	LAMA2	CETP	CAV1	PLAC9	ENG	PCDH18	ROBO4	RAMP2
CELLMARKERMSIGDB_FAN_EMBRYONIC_CTX_BIG_GR…	LHX3	KLHL13	TP73	EMX2	PCBP3	CEND1	CLUL1	AGTR1	ECEL1	SUSD4	NRN1	RTBDN	TXN	DEGS1	CPNE9	PIDD1	RSPO2	MRAP2	MDH1	FAM167A	LINC00…	TMEM163	CLU	STS	PRKAG…
CELLMARKERMSIGDB_FAN_EMBRYONIC_CTX_BIG_GR…																									
CELLMARKERMSIGDB_FAN_EMBRYONIC_CTX_BIG_GR…	CCL3	OLR1	SELPLG	ADAP2	VSIR	C3AR1	TLR4	TMEM119	CRYBB1	SERPIN…	CSF3R	RNASET2	IL13RA1	CTSC	CD84	RGS10	FCGR3A	AIF1	FCGR2A	DHRS9	APOC1	FCGRT	OLFML3	CYBB	ALOX5…
CELLMARKERMSIGDB_FAN_EMBRYONIC_CTX_BRAIN_…	VIM	LAMA4	TM4SF18	FSTL1	S100A10	SERPINH1	CYYR1	ADGRF5	ID1	EMCN	JCAD	STRA6	TSC22D1	C1orf54	ARHGAP29	PRXL2A	CD93	HES1	COBLL1	RBP1	APLNR	TJP1	RELL1	IFITM3	TCN2
CELLMARKERMSIGDB_FAN_EMBRYONIC_CTX_BRAIN_…	LGALS1	PLAC9	COL6A2	CYTH3	NID1	LMNA	PLCE1	GNG11	SLC12A2	FERMT2	IFITM3	CSPG4	ARHGAP29	GPER1	SLC12A7	CD9	CDH6	PDE8B	ABCC9	MGLL	COL18A1	LGALS3BP	LAMB2	IFITM1	VCL
CELLMARKERMSIGDB_FAN_EMBRYONIC_CTX_EX_1_E…																									
CELLMARKERMSIGDB_FAN_EMBRYONIC_CTX_IN_2_I…																									
CELLMARKERMSIGDB_FAN_EMBRYONIC_CTX_IN_3_I…																									
CELLMARKERMSIGDB_FAN_EMBRYONIC_CTX_IN_5_I…																									
CELLMARKERMSIGDB_FAN_EMBRYONIC_CTX_IN_6_I…																									
CELLMARKERMSIGDB_FAN_EMBRYONIC_CTX_MICROG…																									
CELLMARKERMSIGDB_FAN_EMBRYONIC_CTX_OLIG	FA2H	CERS2	S100B	DYNC1I2	RNASE1	LINC030…	SEMA4D	ANKS1B	TPPP	GLTP	GJB1	TMEM15…	FAIM2	STMN4	SLC44A1	PACC1	FNTA	CLDND1	TECR	SH3GL3	ZNF488	NECTIN1	SPTLC2	TSPAN15	BCAS1
CELLMARKERMSIGDB_FAN_EMBRYONIC_CTX_OPC	CCND1	TM4SF1	FIP1L1	PCDH20	BRINP3	CNPY2																			

Table S5: Cell state marker gene list

CELLMARKERCAN_ACINAR_CELLS																									
CELLMARKERMSIGDB_FAN_OVARY_CL1_GPRC5A_TNF…	EIF4A1	ELOC	TIMP1	ATP1B1	TSPAN5	MARCHF3	MAFF	DDX21	NOP16	HSPA9	UBE2D1	HSPD1	HSP90AB1	MAT2A	RHOBTB3	NOLC1	FGFR1	GNL3	LRRC59	TSPYL1	CLTB	WDR43	DSE	PNO1	CRY1
CELLMARKERMSIGDB_FAN_OVARY_CL11_MURAL_GRA…	UBXN8	RANGAP1	MYL12A	HSPD1	MRPL18	LMBR1L	FGD4	SOX4	AFF4	HSPE1	ENC1	LEPROT…	TES	DSP-AS1	BAG3	F3	EPDR1	GPX3	TM7SF2	DNAJA1	LGR4	CSPP1	EXT1	AKIRIN1	SPRR2F
CELLMARKERMSIGDB_FAN_OVARY_CL13_MONOCYTE_…	NINJ1	MNDA	LSP1	CPVL	C1orf1…	TIMP1	CLEC1…	OLR1	KYNU	GPX1	PLAUR	MS4A7	PLEK	BID	CD83	MMP9	CD44	SERPINB1	CD48	BASP1	CTSZ	CTSB	CD1C	CXCL16	PPIF
CELLMARKERMSIGDB_FAN_OVARY_CL14_MATURE_CUM…	CALD1	FOS	LMOD1	BGN	CPE	NOTCH3	OAZ2	CAVIN1	S100A6	MCAM	JUNB	AOC3	SERPINI1	CD151	SORBS2	HEXIM1	TBX2-AS1	NTRK2	SH3BGRL	SEPTIN4	SELENOM	RCAN2	ATF3	MYLK	COX7A1
CELLMARKERMSIGDB_FAN_OVARY_CL15_SMALL_ANT…	CCNL1	PRDM1	PRKAR2B	DNAJB1	RSRC2	AHSA1	DDX5	ETF1	EGR3	KLF6	MIR202…	GSTA1	TRA2B	PLAT	SGK1	MYL12A	ARF4	WIPF3	WT1	SPINT2	REEP6	FILIP1L	CAMTA1	RBM22	FDXR
CELLMARKERMSIGDB_FAN_OVARY_CL18_B_LYMPHOC…	REL	ATP1B3	NINJ1	PLP2	EMP3	HLA-C	LILRB4	HLA-B	MIR155…	PHACTR1	SIDT1	GPR65	SLC15A4	SELL	THEMIS2	CLIC3	SYNGR2	FTH1	LPXN	MZB1	HSP90B1	CORO1A	BIRC3	DERL3	CXCR4
CELLMARKERMSIGDB_FAN_OVARY_CL2_PUTATIVE_E…	SNHG32	TOMM7	RPL36A	COMMD6	NUCKS1	DUSP1	N4BP2…	RPL34	RPL41	NBEAL1	OST4	ST13	RPL38	TMEM258	ID2	PEBP1	SNRPD2	RPS27	MDK	RPL26	CIRBP	RPL30	RPL35A	TCEAL9	RPL23A
CELLMARKERMSIGDB_FAN_OVARY_CL5_HEALTHY_SE…	RASD1	ANGPTL4	UBB	SGK1	FDX1	LAPTM4A	KLF10	HNRNPA…	S100A13	FHL1	FKBP10	CLEC2B	LAMA4	EMILIN1	NME2	RND3	PLAGL1	ATP2B1	RNASE1	NID1	HSPE1	MTUS1	LAMB1	CHORDC1	CPXM1
CELLMARKERMSIGDB_FAN_OVARY_CL6_PUTATIVE_E…	LGALS3BP	GPC3	COL6A3	CCDC80	MGP	HES1	FBLN1	PDK4	BGN	TPM2	TIMP1	MXRA8	CFH	GSTM3	QSOX1	MMP23B	PLTP	IGFBP3	PDGFRL	NR4A1	PRELP	CTSF	SFRP4	HSPA1A	SMOC2
CELLMARKERMSIGDB_FAN_OVARY_CL8_MATURE_CUM…	GRIK1	DBI	SLC16A1	SMIM1	MGST3	ANXA6	ELOB	NDP	NR5A2	UBE2C	IGFBP2	MICOS10	CKAP2	LAPTM4B	PHPT1	DAG1	TM7SF2	NDUFB9	S100B	MIF	MZT2B	PLA2G12A	RANBP1	ATP5ME	COX5B
CELLMARKERMSIGDB_FAN_OVARY_CL9_PUTATIVE_A…	ITPRID2	AQP1	CHIC2	VWF	WWTR1	RDX	SOX17	HLA-E	ERG	YPEL2	AP1S2	IGFBP7	GIMAP8	EMCN	A2M	CD55	NEAT1	TMEM70	IFI44L	DAB2	IFI27	TSC22D1	SWAP70	MGST2	HLX
CELLMARKERMSIGDB_GAO_ESOPHAGUS_25W_C1_CIL…	C11orf16	CFAP300	HOATZ	C12orf76	C16orf…	FYB2	CFAP2…	C1orf87	C20orf…	C20orf…	C2CD2L	ECRG4	FAM166C	C2orf81	CFAP20DC	CFAP299	C4orf47	C5AR1	CPLANE1	C5orf49	C6orf1…	C7orf57	C8orf…	CFAP95	C9orf…
CELLMARKERMSIGDB_GAO_ESOPHAGUS_25W_C2_KRT…	PDE9A	PDP1	PDZK1I…	PRSS22	PRSS27	PRSS8	RNF10…	S100A8	SECTM1	SLC15A1	SLC6A14	SPNS2	SPRR2A	ST3GAL4	STEAP4	TBC1D2	TGM2	TMPRSS11B	TMPRSS…	TMPRSS1…	TOR1B	USP6NL	VASN	VCPKMT	XDH
CELLMARKERMSIGDB_GAO_ESOPHAGUS_25W_C3_FGF…	TMEM183A	TRAF1	ZNF142	ZNF462																					
CELLMARKERMSIGDB_GAO_LARGE_INTESTINE_24W_…	IFIT3	ISLR	LAMA2	LOX	LPAR1	LRRC17	LUM	MAPK10	MATN2	MGP	MMP2	MRC2	MX1	MYC	NGFR	NNMT	OGN	OLFML1	PAMR1	PCOLCE	PDGFRL	PDPN	PHC1	PIEZO2	PLAGL1
CELLMARKERMSIGDB_GAO_LARGE_INTESTINE_24W_…	SLC6A19	SMIM24	SPIB	STEAP1	TMEM45B	TONSL	ZNF264																		
CELLMARKERMSIGDB_GAO_LARGE_INTESTINE_24W_…	CD86	CEBPB	ADA2	CFP	CHI3L1	CLEC10A	CLEC2B	CLEC4G	CLEC7A	CMKLR1	CNEP1R1	CORO7	COTL1	CPVL	CR1	CREG1	CSF1R	CSF2RB	CSF3R	CTSC	CTSL	CTSZ	CXCL10	CXCL16	CXCL2
CELLMARKERMSIGDB_GAO_LARGE_INTESTINE_24W_…																									
CELLMARKERMSIGDB_GAO_LARGE_INTESTINE_24W_…	SLC27A2	SLC38A4	SLPI	SOX9	SULT1B1	TAF4B	TMEM1…	TSPAN8	URB1-A…	ZBED9	ZNF296														
CELLMARKERMSIGDB_GAO_LARGE_INTESTINE_24W_…																									
CELLMARKERMSIGDB_GAO_LARGE_INTESTINE_ADUL…	CBX6	CCDC181	CCDC40	CCDC74A	CCDC74B	CDH22	CDHR3	CDK5R2	CDKN2A	CEP126	CEP170	CES1	CFC1	CHGB	CHRNB2	CNIH2	COL2A1	COL5A2	COL8A2	CPB1	CPE	CPLX2	CRYBA2	CTNND2	CXCL6
CELLMARKERMSIGDB_GAO_LARGE_INTESTINE_ADUL…	CAND2	CASQ2	CBX7	CCDC170	CD28	CD300E	CD36	CDC20B	CDC45	CDH6	CDKL2	CECR2	CENPE	CEP152	CGNL1	CHIC1	CHST13	CLEC16A	CLEC19A	CMPK2	CMYA5	CNBD2	CNNM1	CCNP	COL4A6
CELLMARKERMSIGDB_GAO_LARGE_INTESTINE_ADUL…	ST8SIA1	SULT1C4	SYCP2	SYNPO2	VIPR2	VWF	ZFP42	ZNF114																	
CELLMARKERMSIGDB_GAO_LARGE_INTESTINE_ADUL…	CD19	CD2	CD27	CD300A	CD37	CD3D	CD3E	CD48	CD52	CD53	CD69	CD7	CD79A	CD79B	CDK14	CDKN2A	ADA2	CEP128	CEP85L	CERCAM	CFAP54	CHST11	CHST15	CLEC2B	CLECL…
CELLMARKERMSIGDB_GAO_LARGE_INTESTINE_ADUL…	BST2	TMEM273	C11orf…	C14orf1…	C1R	C1S	EPIST	C8G	CALCRL	CALD1	CAV1	CAV2	CBX6	CCDC102B	CCDC8	CCDC80	CCL11	CCL2	CCM2L	CD34	CD36	CD4	CD93	CDH11	CDH13
CELLMARKERMSIGDB_GAO_SMALL_INTESTINE_24W_…																									
CELLMARKERMSIGDB_GAO_SMALL_INTESTINE_24W_…	MEP1A	METTL7B	MFSD2A	MS4A8	MST1	MUC13	MYO1A	NAGS	NPL	NR0B2	OCIAD2	OSGIN1	PLAGL2	PNCK	PNP	PON2	PP7080	PRSS3	RAB25	RARRES1	RBP2	RBP4	RGN	RNF128	RNF186
CELLMARKERMSIGDB_GAO_SMALL_INTESTINE_24W_…																									
CELLMARKERMSIGDB_GAO_SMALL_INTESTINE_24W_…	ZNF23	ZNF385B	ZNF662																						
CELLMARKERMSIGDB_GAO_SMALL_INTESTINE_24W_…	SACS	SH3GL1P1	SHISA3	SLC36A4	TOE1	TRIM62	TRIQK	TRPC3	TTLL3	WHAMMP3	ZBBX	ZNF503…	ZNF608												
CELLMARKERMSIGDB_GAO_STOMACH_24W_C1_PROCR…																									
CELLMARKERMSIGDB_GAO_STOMACH_24W_C2_TFF2P…																									
CELLMARKERMSIGDB_GAO_STOMACH_24W_C4_PARIE…																									
CELLMARKERMSIGDB_GAO_STOMACH_24W_C5_PUTAT…	TMEM51	TNFAIP8	VSNL1	WFDC1	WIF1	YBEY																			
CELLMARKERMSIGDB_GAUTAM_EYE_IRIS_CILIARY_…	PPP6C	TLNRD1	STX4	TREX1	PRR5L	CCDC86	MYO1E	TMSB4X	HMOX2	EPN1	SQOR	TRIM33	IFFO1	ZNF302	TSG101	ZNF575	SLC66A3	ARMCX2	SLC30A1	KMT5B	PRKD2	CCM2	SYNJ2	CDK12	SLC1A7
CELLMARKERMSIGDB_GAUTAM_EYE_IRIS_CILIARY_…	PLP2	CXADR	FAT1	PDZD2	BRD2	MYO1B	RAB28	EPHA2	SLC16A1	MXD1	RPS19	SQLE	ALDH1A3	MKNK2	HK2	GSTM5	CRIP2	FRMD4B	MIR443…	DGKG	PPP1R1…	GGA2	THY1	EFNA1	ODF3B
CELLMARKERMSIGDB_GAUTAM_EYE_IRIS_CILIARY_…	RNF168	CD86	LINC00…	RASSF5	SETDB2	TPT1	ELOF1	FGD3	NME1-N…	SEPTIN1	TRAPPC…	TIFA	USP48	H2AC18	NR2C2AP	HADH	SINHCAF	RPL35	PPP1CA	SS18L2	ARPC1B	ZNF107	DR1	KLHL6-A…	IDI1
CELLMARKERMSIGDB_GAUTAM_EYE_IRIS_CILIARY_…	PID1	BGN	HSPB6	RBM17	GGT5	CALCRL	ELOC	NR2F2	SRM	SOX4	COL1A1	FILIP1L	LIMA1	GLCE	LAPTM4A	IL15RA	APOL4	CREM	YBX3	PDGFRL	LARP6	RBMS1	PLSCR1	AKR1C1	TFPI
CELLMARKERMSIGDB_GAUTAM_EYE_IRIS_CILIARY_…	CCP110	FRMD3	PCDH20	HCFC2	RAB40B	RTKN	PLEKH…	IRF4	TAFA5	WDFY1	PHYHD1	TRMT13	ROBO3	CGRRF1	RBMXL1	MPG	RNASEH2C	NCBP3	ADGRG6	CD58	PNISR	HLA-C	WWP1	COMMD3	ZC2HC…
CELLMARKERMSIGDB_HAY_BONE_MARROW_CD34_POS…																									
CELLMARKERMSIGDB_HAY_BONE_MARROW_CD34_POS…																									
CELLMARKERMSIGDB_HAY_BONE_MARROW_CD34_POS…																									
CELLMARKERMSIGDB_HAY_BONE_MARROW_CD34_POS…	PDIA5	GSTT2B	KALRN	SLC37A1	EXOC3L4	PRKG1	PIEZO2	KAZN	MLEC	KLHL13	EFHC2	GLOD5	MYH7	STAC											
CELLMARKERMSIGDB_HAY_BONE_MARROW_CD8_T_CE…	ITGB1	JMJD6	TRG-AS1	SLA	LINC02…	GBP5	NR4A2	HERPUD2	MFSD10	KIF19	TNIP3	CAPN2	KIF21A	ATP8A1	SON	CCNH	MT1F	ARAP2	LINC03…	CXCR6	PRDM1	CHI3L2	ENC1	SLAMF6	
CELLMARKERMSIGDB_HAY_BONE_MARROW_EARLY_ER…	COQ8A	PGAP4	MGST2	GGCT	FBL	RPL23	PLTP	DCTPP1	AIG1	KIT	TRIP6	MUC1	SELENOH	MRPS23	CLNS1A	MICOS10	CYP2E1	HNRNPC	ECHS1	PHF6	HDAC7	SLC39A4	MRPS26	EIF1AX	TFAM
CELLMARKERMSIGDB_HAY_BONE_MARROW_EOSINOPH…	TMBIM6	TMEM160	MBD3	P2RY2	IL1RAP	ATP8B4	SNHG25	KBTBD11	TMX4	GNB1															
CELLMARKERMSIGDB_HAY_BONE_MARROW_NAIVE_T_…	MALAT1	RPL10A	TRBC2	ZFP36L2	RPL14	RPL4	TOMM7	RPSA	CD3G	RPS19	FLT3LG	RPL18A	EVL	RPL6	LEF1	RCAN3	TPT1	CCR7	TCF7	RPL5	LCK	RPS8	CD27	RPL37	SPOCK2
CELLMARKERMSIGDB_HAY_BONE_MARROW_NEUTROPH…	CAPG	ITGAM	VIM	PYCARD	BST1	S100A10	KLF4	GCA	ELOB	FGL2	AGTRAP	SLC11A1	ATP5F1E	CES1	LAMTOR4	CKAP4	IQGAP1	GSTP1	MCEMP1	TMEM107	PGD	TMEM176B	BASP1	TMEM170B	CLEC4E
CELLMARKERMSIGDB_HAY_BONE_MARROW_NK_CELLS	BIN2	PYHIN1	TBX21	KLRC2	LITAF	CALM1	IL2RG	PTGDR	LAIR2	DOK2	TPST2	TXK	SEPTIN7	NCR1	TRGC1	B3GNT7	APOBEC3G	MYL12A	SH2D2A	CHST2	CEP78	GNG2	MIB2	KIR3DL2	SUN2
CELLMARKERMSIGDB_HAY_BONE_MARROW_PRE_DEND…																									
CELLMARKERMSIGDB_HAY_BONE_MARROW_PRO_B	PARP1	MXD3	ACTG1	TPX2	CDCA5	SGO1	SSBP2	CLSPN	EZH2	EIF4A3	GTSE1	SAC3D1	LMNB1	CKS1B	ASPM	PHGDH	SRSF3	CENPM	POU2AF1	CCND3	KIFC1	RAD51	TCF3	ANP32E	MACRO…
CELLMARKERMSIGDB_HAY_BONE_MARROW_STROMAL	MGP	TNFAIP6	RBMS3	EPHX1	ALPL	TAGLN	TFPI	ADIRF	MDK	CDH11	COL14A1	ANGPTL4	MXRA8	FOXC1	COL3A1	SPARCL1	COL6A1	FGF7	GJA1	MDFI	C1QTNF1	EGR1	PDGFRA	CCN2	VGLL3
CELLMARKERMSIGDB_HE_LIM_SUN_FETAL_LUNG_C0…	CORO1C	C3orf70	SMYD3	MRGPRF	CHRM3	ANXA3	KLF7	MACIR	CDC42E…	GRB14	PDLIM4	HEPH	CD47	NKD1	CAV2	PPP1R12B	CBX6	ANGPTL2	SYNGR2	PSD	IRAG1	RABGAP1	IMPA2	NKX6-1	ISG15
CELLMARKERMSIGDB_HE_LIM_SUN_FETAL_LUNG_C0…	EPHB2	THBS3	MIR503…	DNER	NAV3	MXRA5	PTGIS	FJX1	SCG5	PROS1	NRK	TNXB	ADAM33	BST1	PI15	CLEC3B	SSC5D	FMO1	GXYLT2	ANGPTL6	CHRDL1	NTRK3			
CELLMARKERMSIGDB_HE_LIM_SUN_FETAL_LUNG_C0…	TOP2A	MND1	RASL11B	CDC20	SDC1	CDK1	AURKB	NUSAP1	CENPN	MKI67	NSG1	CTPS1	SCD	CCNA2	BCL11A	FZD2	GPC2	ORC6	TENM3	GPSM3	PIMREG	CDCA5	KIF20A	BID	CHAF1A
CELLMARKERMSIGDB_HE_LIM_SUN_FETAL_LUNG_C0…	BEX2	ASS1	NEO1	CD47	SYNGR2	CRABP2	GJA1	GCA	WASF1	BDNF	CD320	TNNI1	RAB11FIP1	NXPH2	HMGA1	GAS6	CDH3	LMNB1	PTGS1	LINC006…	RAB33A	GPM6A	RASSF7	SINHCAF	ITGA3
CELLMARKERMSIGDB_HE_LIM_SUN_FETAL_LUNG_C0…	PLEKHG1	FIBIN	PRELP	SLC2A1	DLK1	BEX1	CYP26…	NEDD9	SUSD5	PRRX1	CHST11	PEG3	TYRP1	AMOT	MAG	VSNL1	CTHRC1	PYGL	DEPTOR						
CELLMARKERMSIGDB_HE_LIM_SUN_FETAL_LUNG_C0…																									
CELLMARKERMSIGDB_HE_LIM_SUN_FETAL_LUNG_C0…	DMKN	CLDN1	CARD16	PTGS1	RASSF7	TSTD1	MT1E	AQP1	CAPG	PLP2	GPM6A	PTGIS	LGALS2	WT1	PDPN	GJA1	TGM1	VAMP8	MAP3K8	FLRT3	KLF2	PERP	REEP1	MMP28	ADAM15
CELLMARKERMSIGDB_HE_LIM_SUN_FETAL_LUNG_C0…	FOXF2	RGMA	TOX	LRIG3	LMNB1	SYNPO2	LEF1	C8orf34	EFEMP1	MID1IP1	DNAJC12	TRIOBP	MCM4	REEP3	SGK1	TUBB2B	FHL2	TRIB2	CLMP	ITPRID2	CTHRC1	PTTG1	IL17D	PXK	PIM1
CELLMARKERMSIGDB_HE_LIM_SUN_FETAL_LUNG_C0…	LMOD1	ISM1	COL23A1	C16orf89	KLHL4	MYL4	NAV2	SLC29A3	SLC16A3	CHRDL1	CDH6	MTCL1	C3orf70	NPB	ANO4	RELB	KIF26B	ZNF536	LTBP2	ENTPD1	EPCAM	LRRN1			
CELLMARKERMSIGDB_HE_LIM_SUN_FETAL_LUNG_C0…	TSTD1	CAVIN2	PDZK1I…	PLLP	ALCAM	TSPAN7	LAMA5	GJA1	SEMA3B	KLK10	SMTNL2	RAB11F…	MAG	SLPI	GCA	FKBP11	HSBP1L1	ARL4A	LGALS2	SERPINB9	RDH10	PLAAT4	DPP4	MYRF	CADPS2
CELLMARKERMSIGDB_HE_LIM_SUN_FETAL_LUNG_C0…	SNAI1	SLC26A2	PCSK5	JCAD	MSANTD4	RNF43	RAI2	MTUS1	REEP1	THBD	RGN	ZNF536	ANO4	SYT17	SLC9A6	SLFN12L	C1QTNF5	PROM1	MT1E	INTS6L					
CELLMARKERMSIGDB_HE_LIM_SUN_FETAL_LUNG_C1…																									
CELLMARKERMSIGDB_HE_LIM_SUN_FETAL_LUNG_C1…	PAPSS2	DPYD	MLPH	CYP2B6	SCNN1G	SLA	SULT2…	ISG20	CRIM1	NXPH4	SPINK1	NEGR1	CA8	SPNS2	RHOV	CLIC5	PAG1	ROBO2	NDUFA4…	FCGR2A	SERPIN…				
CELLMARKERMSIGDB_HE_LIM_SUN_FETAL_LUNG_C1…																									
CELLMARKERMSIGDB_HE_LIM_SUN_FETAL_LUNG_C1…	POC1A	KIFC1	CDT1	LAMA1	DIAPH3	ALPL	CDCA8	H2BC9	CCDC34	H1-1	ACOT7	AURKB	PMAIP1	TROAP	TIMELESS	NCAPH	APOC1	CKAP2L	NUF2	FEN1	GCH1	SGO1	TRIP13	GADD45G	CDCA3
CELLMARKERMSIGDB_HE_LIM_SUN_FETAL_LUNG_C1…	ATP2A3	STMN3	PGAM2	RAB26	GPR160	SCG5	CD200	PCLO	RGS4	CDHR3	GNAO1	SCGN	QPCT	TUBB3	CCDC28B	GPX3	LINC002…	SMIM6	STX1A	DST	SYP	STMN2	MAP2	CNTNAP2	CACNA…
CELLMARKERMSIGDB_HE_LIM_SUN_FETAL_LUNG_C1…																									
CELLMARKERMSIGDB_HE_LIM_SUN_FETAL_LUNG_C1…																									
CELLMARKERMSIGDB_HE_LIM_SUN_FETAL_LUNG_C1…	OXTR	SNCAIP	PIGW	LNX1																					
CELLMARKERMSIGDB_HE_LIM_SUN_FETAL_LUNG_C1…	GRAMD2B	NR4A2	RND3	SFTPA2	SPDEF	MT3	PLEKH…	TNNT3	MUC5B	MUC15	TSPAN1	DEGS2	MAFB	MYC	DEFB1	TMC5	MET	LCN2	SIX1	ATP2C2	TSPAN5	BHLHE40	LYPD6B	CAMK1D	SULT2…
CELLMARKERMSIGDB_HE_LIM_SUN_FETAL_LUNG_C1…	SYT1	TTLL7	JAM3	RGS17	TAGLN3	SMOC2	ROBO1	IZUMO4	NCALD	RAB3B	CACNA2…	SCGN	TMEM178A	SRRM4	TMEM181	RGS7	WSB2	PHGR1	SCN3A	PCLO	RHBDL1	STMN3	PTPRN2	ADAM28	CAMK2B
CELLMARKERMSIGDB_HE_LIM_SUN_FETAL_LUNG_C1…	STOM	SFRP1	CXCL3	CSPG4	PKP1	COL7A1	NPPC	KLK10	ARSJ	CHST9	HPRT1	SLC16A1	PRNP	PHLDA3	LPAR6	GPM6B	LAMA1	EFHD1	CD40	STRA6	DAPL1	UBD	APOD	CXCL2	ROBO1
CELLMARKERMSIGDB_HE_LIM_SUN_FETAL_LUNG_C1…	MEG3	PALMD	TPBG	TCN1	DTX4	GLYATL2	AZGP1	SLC5A1	SOX10	GABRP	CCL28	VTCN1	PLAAT3	ZNF750	MGP	LRRC26	KCNN4	TNFSF10	CX3CL1	CEACAM6	TSHZ2	TRIM29	CXCL1	C1orf115	AQP5-…
CELLMARKERMSIGDB_HE_LIM_SUN_FETAL_LUNG_C2…	MERTK	PDZD11	ITSN1	SCN1B	TREM2	TNS3	INF2	CD9	LACC1	PLOD3	HSD17B…	CD38	MX1	FCGR3A	NMRK1	VAMP5	TMEM176A	DNASE2	PLEC	BCL2L1	TMEM17…	IGF2R	TCTN1	ETV5	ME1
CELLMARKERMSIGDB_HE_LIM_SUN_FETAL_LUNG_C2…	KIF16B	P2RY14	AGPAT1	THEM4	APOL3	REC8	AIM2	SLC66A1	BCL6	FAH	SIGLEC…	CCSER1	TSTD1	RUBCNL	MIR4435-…	NCOA7	PLPP1	PDE4B	MAP4K1	ENPP1	VAV3	PDLIM1	PPM1J	SLC46A3	PLEKH…
CELLMARKERMSIGDB_HE_LIM_SUN_FETAL_LUNG_C2…	KIF17	TMEM8B	NCKAP5																						
CELLMARKERMSIGDB_HE_LIM_SUN_FETAL_LUNG_C2…																									
CELLMARKERMSIGDB_HE_LIM_SUN_FETAL_LUNG_C2…																									
CELLMARKERMSIGDB_HE_LIM_SUN_FETAL_LUNG_C2…	SPTBN1	MAP4K2	DNMT3A	ACAP1	EBPL	ODF2L	LAPTM…	SOCS2	RHOH	ACBD6	BAALC	GNAI1	ERGIC1	TM7SF3	EPB41L4A…	POLR2C	CDCA7L	CAMK2G	SEPTIN…	HDAC7	RNF220	ZCCHC7	DBN1	ACSS1	PBXIP1
CELLMARKERMSIGDB_HE_LIM_SUN_FETAL_LUNG_C2…	MFSD2B	C2orf88	F2R	SNCA	DAAM1	ALOX12	TAL1	CD9	TRIM58	BCL2L1	GMPR	ATP2A3	LRRFIP2	PBX1	CCL5	TREML1	KALRN	STIM1	RIPOR3	LMNA	TLK1	MAFG	C11or…	ABCC4	INKA1
CELLMARKERMSIGDB_HE_LIM_SUN_FETAL_LUNG_C2…	TNFRSF1…	MTARC1	STEAP4	IGF2R	MGAM	AP5B1	APOBE…	CLEC5A	F5	ECE1	ANXA3	DGAT2	VNN3P	LINC03078	TNFAIP6	VNN1	ADGRE3								
CELLMARKERMSIGDB_HE_LIM_SUN_FETAL_LUNG_C2…	FAM210B	E2F3	PPBP	RBM38	CREBRF	TNIK	GNG11	ZNF431	STIM1	BROX	TNFAIP…	BBC3	CA2	CLEC1B	LGALSL	GP1BA	F2R	MACO1	TRIM58	SDE2	GP9	CPEB4	H2AC11	KLHL24	PDLIM1
CELLMARKERMSIGDB_HE_LIM_SUN_FETAL_LUNG_C2…	NAPSA	ADK	CKS1B	RHOF	ADA	CBX5	GTF2E2	SULF2	CARD11	DAXX	CCDC69	ARL6IP6	ACSL3	TYMS	CTPS1	CCDC167	GNG7	MCRS1	S100B	DNAJC9	STX18	CYFIP2	TFDP1	NDRG1	ERG28
CELLMARKERMSIGDB_HE_LIM_SUN_FETAL_LUNG_C2…																									
CELLMARKERMSIGDB_HE_LIM_SUN_FETAL_LUNG_C2…																									
CELLMARKERMSIGDB_HE_LIM_SUN_FETAL_LUNG_C3…																									
CELLMARKERMSIGDB_HE_LIM_SUN_FETAL_LUNG_C3…	OAS2	NCKIPSD	TNS3	LAMA5	ULBP2	CD83	PLXNC1	NCALD	GBP1	GPR4	SPRED2	GNG7	PKIA	TBXA2R	ARC	PDE1C	CDKAL1	ARHGAP6	TBX2-A…	ATF3	KCNN2	TAGLN3	LRRC49	ZBTB16	SPRY4
CELLMARKERMSIGDB_HE_LIM_SUN_FETAL_LUNG_C3…	DNAJC9	CKS2	HBB	GCLM	RGS10	CDK1	KIF22	H2AC11	HBQ1	ITGA4	NCAPG	MFSD2B	CHPT1	CDKN3	ASPM	CR1L	UBE2C	UBE2S	RHD	GYPE	C17orf…	OSBP2	PRC1	CDC20	KIFC1
CELLMARKERMSIGDB_HE_LIM_SUN_FETAL_LUNG_C3…	UCK2	STC2	SLC25A4	KRT8	CENPV	TYMS	ID2	CCNB1I…	PTGR1	FADS1	P4HA1	ETV4	LONRF1	SLX9	FAM43A	PSMD14	YARS1	ANP32E	F2R	BOP1	RUVBL1	PHGDH	ICAM1	ALDH7A1	ZNF581
CELLMARKERMSIGDB_HE_LIM_SUN_FETAL_LUNG_C3…	KBTBD11	NR1H3	LAMB1	UNC5B	ST6GAL…	MAN1A1	SCN3B	PLAAT4	HOXD8	RAB11F…	PDE2A	REEP1	LETR1	CHRDL1	PKHD1L1	MAP3K1	SFXN3	PDE1A	SYNE1	A4GALT	LINC01…	PTPN14	SNCG	HS3ST1	ABI3BP
CELLMARKERMSIGDB_HE_LIM_SUN_FETAL_LUNG_C3…	CCL14	OAF	FLRT2	CD74	BCL7C	NIBAN1	PMP22	FAM107B	TSHZ2	ABI3BP	PIEZO2	PGM1	MAGED4B	PARD6G	LY96	PPIC	HTRA1	KBTBD11	DOCK5	MAGED4	C6orf1…	LAYN	REEP1	PLAAT4	PDGFA
CELLMARKERMSIGDB_HE_LIM_SUN_FETAL_LUNG_C3…	SOX11	DIXDC1	LIMD1	SPACDR	CX3CL1	GCOM1	RBP5	HOPX																	
CELLMARKERMSIGDB_HE_LIM_SUN_FETAL_LUNG_C3…	KAZALD1	SERPINE1	TMTC2	MPZL2	PDE3A	IFI6	MET	MRC2	THBS1	CPXM2	BCAT1	LOX	DPP7	CSRNP1	F2R	TANC2	CCN2	PTGS2	FGF18	ASS1	TMEM47	ITPR1	LPCAT2	IGFBP3	EPDR1
CELLMARKERMSIGDB_HE_LIM_SUN_FETAL_LUNG_C3…	PHLDB3	ZDHHC17	DDX10	LYRM1	SLC41A1	NR1H3	S100P	MEST	TSHZ2	CAMK1	SEMA3A	TMEM12…	MFAP4	FOXC2	PRKCZ	HOXD9	ABCA4	CAMK2D	CCDC80	CXXC5	CFI	HLA-F	NUPR1	HES4	CELSR1
CELLMARKERMSIGDB_HE_LIM_SUN_FETAL_LUNG_C3…	SPINK5	NALF1	CCDC69	VCAM1	ASRGL1	MAP2	HSPA2	ADAMTS6	CYP1B1	PTGIS	MYOF	HLA-DR…	RBP5	DHH	HMGCLL1	GFOD1	ATP2A3	EHBP1L1	SULT1E1	SCUBE3	ASAP3	SYT15	DNM3	HAS2	FAM12…
CELLMARKERMSIGDB_HE_LIM_SUN_FETAL_LUNG_C4…																									
CELLMARKERMSIGDB_HE_LIM_SUN_FETAL_LUNG_C4…																									
CELLMARKERMSIGDB_HE_LIM_SUN_FETAL_LUNG_C4…																									
CELLMARKERMSIGDB_HE_LIM_SUN_FETAL_LUNG_C4…																									
CELLMARKERMSIGDB_HE_LIM_SUN_FETAL_LUNG_C4…	UHRF1	SMC2	NCAPG	PHF19	CKAP2L	CLSPN	KPNA2	CENPK	FBXO5	CDCA8	NDC80	MCM5	NCAPH	CBX5	NUDT1	MAD2L2	ASPM	DTYMK	TACC3	HADH	SKA2	CDKN3	KIF2C	CDCA3	TCF19
CELLMARKERMSIGDB_HE_LIM_SUN_FETAL_LUNG_C4…	CENPW	DNAJC9	PHF19	CENPN	CENPH	ATAD2	KIF20B	FBXO5	H2AC11	FANCG	MAD2L1	MXD3	CDCA4	HJURP	HIRIP3	NCAPG	DLGAP5	ACTN1	PCNA	MCM7	NUF2	ESCO2	RACGA…	CDKN3	FOXM1
CELLMARKERMSIGDB_HE_LIM_SUN_FETAL_LUNG_C4…	FSD1	NR1D1	RARG	LDLRAD4	ATP10D	DAPK1	RUNX2	KLF4	ENPP1	RGS16	ARL3	PERP	WDR86	GPR35	MVB12B	ABHD15	ANKH	MPZL3	IL17RE	SVIL	MAN1A1	PLXND1	KRT86		

Table S5: Cell state marker gene list

CELLMARKERCAN_ACINAR_CELLS																									
CELLMARKERMSIGDB_HE_LIM_SUN_FETAL_LUNG_C4…																									
CELLMARKERMSIGDB_HE_LIM_SUN_FETAL_LUNG_C4…	MAFK	MOB1B	PELI2	PTK7	FOXK2	SH3TC1	BCL7A	EPHB6	PCSK5	KCNA3	ZNF518B	TBL2	TBC1D19	ADORA2A	ETV6	SCAI	MED13L	TLE3	PIKFYVE	ANKRD36	MSI2				
CELLMARKERMSIGDB_HE_LIM_SUN_FETAL_LUNG_C4…																									
CELLMARKERMSIGDB_HE_LIM_SUN_FETAL_LUNG_C5…	PARVB	NAPSA	SPOCK2																						
CELLMARKERMSIGDB_HE_LIM_SUN_FETAL_LUNG_C5…	PABPC4	GGCT	CCR7	IRF5	IGFLR1	TMEM154	CD58	CRYZ	ARHGAP9	RRAS2	PARVB	PTPN7	ACP5	WNT10A	NAMPT	ADAMTS6	CYBB	UGCG	TRAP1	MTHFD2	NAGK	GTF2H5	NAPSA	MCRIP2	ARL4C
CELLMARKERMSIGDB_HE_LIM_SUN_FETAL_LUNG_C5…																									
CELLMARKERMSIGDB_HE_LIM_SUN_FETAL_LUNG_C5…																									
CELLMARKERMSIGDB_HE_LIM_SUN_FETAL_LUNG_C5…	NCAPG	CDCA4	RAD51A…	HPRT1	CCNA2	AURKB	C19or…	CENPK	BIRC5	SMIM3	GBP4	TK1	UBE2C	LIG1	PCLAF	FBXO5	MND1	KIF15	RACGAP1	NINJ1	CLSPN	TPGS2	LINC0…	PHGDH	CHAF1A
CELLMARKERMSIGDB_HE_LIM_SUN_FETAL_LUNG_C6…	LINC015…	KIAA0513	SLIT1	PITX1	SMAD3	TNFRSF21	LGR4	TOX3	LINC01…	C14orf…	DPYSL3	CAPN9	SEC14L3												
CELLMARKERMSIGDB_HE_LIM_SUN_FETAL_LUNG_C7…	PLSCR3	SRPX	MAL2	AFAP1L1	CHST9	ITGA2	SP100	NTM	COL28A1	NMI	IL1RAP	ARHGAP…	HMCN1	SFXN3	GRIP2	SHROOM4	ELN	PSMB8	LYPD6	UTP15	CYP4V2	EGLN2	COL6A3		
CELLMARKERMSIGDB_HE_LIM_SUN_FETAL_LUNG_C7…	PLA2G6	GRIP2	TAPT1																						
CELLMARKERMSIGDB_HE_LIM_SUN_FETAL_LUNG_C7…	NIBAN1	FRMD3	IQGAP2	TPBG	GPNMB	ST6GALN…	CDC42…	COL28A1	FAM102A	LIMCH1	DOCK5	FSTL3	ART3	TGFBR3	SH3D19	LAMP5	RGS10	HTRA1	NTM	SFTPC	NFIX	MAL2	RBM43	LINC022…	RASGE…
CELLMARKERMSIGDB_HE_LIM_SUN_FETAL_LUNG_C7…	GRIA3	RBFOX1	PRUNE2	DHCR24	CPLX2																				
CELLMARKERMSIGDB_HE_LIM_SUN_FETAL_LUNG_C7…	AKR1C1	DPYS	PTGER3	PRKAR2B	HES4	PPP2R5B	PNMA2	SPINT2	CAMK2B	ACTL6B	SYNGR3	ARHGEF…	SCAMP5	DMTN	MCHR1	ARG2	MAP6	ATP6V1G2	ATP13A2	HHIP	MPP3	INPP1	FN3K	SYT5	CORO1A
CELLMARKERMSIGDB_HE_LIM_SUN_FETAL_LUNG_C7…	ATRNL1	YPEL4	NYAP2	TLE6																					
CELLMARKERMSIGDB_HU_FETAL_RETINA_AMACRINE	BEX1	RCVRN	GPM6A	MLLT11	CD24	CRABP1	TUBB2A	DPYSL2	TUBB2B	NREP	RERE	MARCKS…	STMN1	CFL1	LUC7L3										
CELLMARKERMSIGDB_HU_FETAL_RETINA_BIPOLAR	CRYAA	FAM171B	NRL	MIR124-…	ANK3	CACNA1F	NFIB	CRX	AGAP1	RP1	PLCD4	ABCA7	ATP2B1	TCF4	AIPL1	RD3	GNAT1	SYP	ATP1B2	RAX2	LINC00…	SRRM4	CDHR1	MPP4	NR2E3
CELLMARKERMSIGDB_HU_FETAL_RETINA_FIBROBLA…	COL6A3	VCAN	MXRA5	CTSK	TGM2	EPB41L2	SNAI2	COL4A1	CCN2	RSPO3	PDGFRB	EGR1	BAMBI	EGFL6	GUCY1A1	EMP3	IFITM3	CDH11	IL11RA	ID3	CYTL1	TXNIP	TPST1	IFITM2	ANGPT1
CELLMARKERMSIGDB_HU_FETAL_RETINA_HORIZONT…																									
CELLMARKERMSIGDB_HU_FETAL_RETINA_MICROGLIA	LCP1	FYB1	P2RY13	APBB1IP	LY86	GPR183	ADAP2	GMFG	BHLHE41	FPR1	MAF	HCLS1	SAMSN1	SAMHD1	DOCK8	IGSF6	PTAFR	FCGRT	B2M	HLA-E	FCGR3A	CCL4L2	CXCL8	HLA-DRB1	FCGR2A
CELLMARKERMSIGDB_HU_FETAL_RETINA_MULLER																									
CELLMARKERMSIGDB_HU_FETAL_RETINA_PHOTOREC…	SRRM4	BAZ2B	GRM6	NDRG1	UBXN4	PTPRZ1	AHI1	PLEKHB1	TUBB4B	DUSP1	CADPS	SNAP25	VAMP2	TERT	FOS	SYT1	CKB	AKAP9	NNAT	H4C3	SOX4	H3-3B	TPI1		
CELLMARKERMSIGDB_HU_FETAL_RETINA_RGC	DLL3	TPPP3	ACOT7	CALB2	ALCAM	DPYSL5	SCG3	GDAP1L1	SNCA	FXYD7	PGM2L1	BNIP3	IRX2	TFAP2D	HMGCS1	SH3BGRL3	FDFT1	ANK2	CHGB	HMGCR	CD24	CD200	AKR1C1	C14orf1…	HSPA1…
CELLMARKERMSIGDB_LAKE_ADULT_KIDNEY_C1_EPI…																									
CELLMARKERMSIGDB_LAKE_ADULT_KIDNEY_C10_TH…	SLC25A5	IER3	WFDC2	FTL	CPM	CD44	CD74	LRP10	TIMP1	LDHB	LAPTM4B	VMP1	EFNA5	MGLL	DNAH5	SNX10	ZFP36L2	NEK10	H3-3B	PLOD2	RPS16	UBB	S100A…	JTB	GSTP1
CELLMARKERMSIGDB_LAKE_ADULT_KIDNEY_C12_TH…	NDUFA4	ATP5MC1	PSAP	CNP	OBSL1	COX6B1	MST1L	PLCB1	NELL1	NTRK2	MAL	MPC1	DNAJC19	ATP5F1A	COX7C	IMPA2	UQCRB	TSPAN8	GSTM3	PDXK	BICDL1	CLCN5	ACSL1	PRDX3	OXCT1
CELLMARKERMSIGDB_LAKE_ADULT_KIDNEY_C18_CO…	GSTM3	HSP90B1	RPL7	ARG2	RPL19	RDH10	B2M	CYSTM1	BAG1	GDF15	RPL10	BMPR1B	DUSP1	RPLP0	GAPDH	SOD1	RPS2	MYL6	UBB	AKR1B1	SRP14	RPS6	RBBP8	PFKFB3	RPS4X
CELLMARKERMSIGDB_LAKE_ADULT_KIDNEY_C19_CO…	EEF1A1	RPS4X	RPLP0	ZDHHC3	ATP6V1A	RPL13	RTN4	PSMA7	RPL7	ITM2B	RPL10	CYB5A	ARHGAP18	ATP6AP2	CYSTM1	SLC16A5	ATP6V1G1	RPL19	RPS5	DYNLT3	WFDC2	CA2	H1-0	RPL6	LDHA
CELLMARKERMSIGDB_LAKE_ADULT_KIDNEY_C25_EN…																									
CELLMARKERMSIGDB_LAKE_ADULT_KIDNEY_C28_IN…	FNDC3B	ITSN1	TMSB4X	PLXDC2	RBMS1	FOXO3	LPP	DACH1	TTC28	IL6ST	MED13L	REV3L	UACA	AUTS2	SYNE1	SSBP2	SETBP1	DGKH	EPS8	TACC1	APBB2	UGGT2	PRKCA	VMP1	LINC-…
CELLMARKERMSIGDB_LAKE_ADULT_KIDNEY_C29_UN…	CCDC198	CEP112	AUTS2	EXT1	PAWR	DST	RBMS3	GALNT14	EHBP1	KANK1	ZEB1	GPHN	MACROD2	SHROOM3	ARHGAP29	PDE7B	SPIDR	NR2F2-AS1	DANT2	AKT3	MBD5	PARD3B	KLF6	PLEKHA7	AGBL4
CELLMARKERMSIGDB_LAKE_ADULT_KIDNEY_C5_PRO…	RUNX1	GRAMD1B	WDR19	LINC002…	SLC28A1	NFIX	CLTC	ASS1	CNTNAP2	TENM2	SMIM24	C11orf…	CSMD1	ABCC2	NRP1	AGMO	ENPP6	SLC5A12	DNM1	CYP2R1	RAB11F…	MMP15	CUBN	UPB1	DAB1
CELLMARKERMSIGDB_MANNO_MIDBRAIN_NEUROTYPE…	RPL38	RPL41	RPL6	RPL7	RPL7A	RPL8	RPL9	RPS27A	RPS4X	RPS8	RSRC2	SAP18	SEC61B	SEC61G	SERBP1	SF3B1	SKP1	SLC25A3	SON	SRP14	SRSF5	SUB1	SUMO2	ELOB	TSC22…
CELLMARKERMSIGDB_MANNO_MIDBRAIN_NEUROTYPE…	CADM1	CADPS	CALB2	CAMK2B	CAMK2N1	CASZ1	CBLN2	CCDC136	CCSER1	CD2	CD24	CD27-A…	CDC42EP3	CDH13	CDH4	CDK5R1	ADA2	TMEM121B	CELF3	CELF4	CELF5	CELSR3	CGN	CHD5	CHGA
CELLMARKERMSIGDB_MANNO_MIDBRAIN_NEUROTYPE…	ARMH4	C1orf35	C2CD4C	C2CD5	CA1	CACNA1B	CACNA…	CACNA1E	CACNG2	CADM1	CADPS	CADPS2	CALB1	CAMK2B	CAMK2N1	CASP2	CBLN2	CCBE1	CCDC136	CCDC184	CCDC85A	CCDC88A	CCSER1	CD2	CD24
CELLMARKERMSIGDB_MANNO_MIDBRAIN_NEUROTYPE…	C6orf118	CACNA1A	CACNA1B	CACNA1E	CACNG2	CACNG4	CACNG8	CADPS	CALB1	CALN1	CAMK2N1	CASKIN1	CBLN2	CBLN4	CCDC85A	CD2	CD24	CD27-AS1	CDH13	CDH4	CDH8	CDK14	CDK5R1	CDKL2	CDKN2D
CELLMARKERMSIGDB_MANNO_MIDBRAIN_NEUROTYPE…	APOL4	APOLD1	APRT	ARAP2	ARAP3	ARC	ARHGA…	ARHGAP…	ARHGEF1	ARHGEF…	ARHGEF…	ARPC1B	ASAH1	ASB9	PGGHG	ATP8B1	AXIN2	B2M	B4GALT1	BCAM	BCAP29	BCL3	BCL6B	BMP4	BNIP2
CELLMARKERMSIGDB_MANNO_MIDBRAIN_NEUROTYPE…	BCL3	BDNF	BHLHE41	BIN1	BIN2	BIRC3	BLNK	BLVRB	BMP2K	BRI3	BTK	C12orf…	C17orf67	PITHD1	C1orf54	C1QA	C1QB	C1QC	C3	C3AR1	CAPG	CAPZB	CARD11	CARD9	CASP1
CELLMARKERMSIGDB_MANNO_MIDBRAIN_NEUROTYPE…	ADGRB1	ADGRB3	BASP1	BCL11B	BCO2	BDNF	BDP1	BEND6	BLCAP	BNC2	BRINP1	BRINP2	BRINP3	BRSK1	BSN	C11orf87	C12orf76	C1orf35	C1QL4	C22orf42	C3orf62	C5orf49	CA1	CABLES2	CACNA…
CELLMARKERMSIGDB_MANNO_MIDBRAIN_NEUROTYPE…	CTNNA2	CXADR	CXXC4	DCC	DCX	GSDME	DOCK3	DOCK4	DOK6	DPYSL3	DRD2	DSCAM	DTD1	DUSP26	EBF1	EBF3	EDIL3	ELAVL2	ELAVL3	ELAVL4	EPB41	EPHA8	ETFB	NALF1	ENTRE…
CELLMARKERMSIGDB_MANNO_MIDBRAIN_NEUROTYPE…	CACNB3	CACNG2	CADPS	CASKIN1	CASZ1	CBLN4	CCDC1…	CCDC85A	CCDC85B	CD2	CD24	CDH13	CDH4	CDK5R1	TMEM121B	CELF3	CELF4	CELF5	CELSR3	CHGA	CHN2	CHST1	CLCN4	CLUL1	CMIP
CELLMARKERMSIGDB_MANNO_MIDBRAIN_NEUROTYPE…	CASZ1	CCDC112	CCDC184	CD2	CD24	TMEM121B	CELF3	CELF6	CELSR3	CEND1	CHGA	CHN2	CHRM2	CHRNA3	CHRNB4	CLCN4	CLUL1	CLVS1	CNST	CNTN2	CNTN4	CNTNAP2	CNTNA…	CORO2A	CPEB3
CELLMARKERMSIGDB_MANNO_MIDBRAIN_NEUROTYPE…	C1QL1	C21orf58	C2orf2…	C2orf72	THUMPD2	VXN	CA1	CACFD1	CACNA1G	CACNG2	CACNG7	CADM2	CADM4	CALCRL	CASK	CCDC175	CCDC39	CCND2	CDCA7L	CEND1	CEP83-…	CGREF1	CHKB-…	CHST11	CHTF8
CELLMARKERMSIGDB_MANNO_MIDBRAIN_NEUROTYPE…	ARHGAP42	ARHGAP6	ARHGDIB	ARHGEF12	ARHGEF…	ARHGEF4	ARHGE…	ARID5B	ARRDC2	ASAH1	ASB9	ATF3	PGGHG	ATP1A2	ATP1B2	ATP1B3	ATP8B1	AVPR1A	AXIN2	AXL	B2M	B4GALT3	BCAP29	BCHE	BCL3
CELLMARKERMSIGDB_MANNO_MIDBRAIN_NEUROTYPE…	CKAP2	CKAP2L	CKS1B	CKS2	CLSPN	CNPY1	COL2A1	CPSF3	CRB2	CROT	CRYM	CRYZ	CCN2	CTNNAL1	CCN1	DBF4	DEPDC1	DEPDC1B	DHFR	DLGAP5	DMBX1	DMRTA2	DNAJC9	DSC2	DSG2
CELLMARKERMSIGDB_MANNO_MIDBRAIN_NEUROTYPE…	CENPF	CENPN	CENPU	CENPV	CENPW	CEP152	CHAF1A	CHEK1	CHN2	CHRDL1	CKAP2	CKAP2L	CKS1B	CLSPN	CNN2	CNPY1	CNTN4	CNTN6	COL2A1	CRB2	CRYZ	CCN2	CTNNA…	CCN1	CYYR1
CELLMARKERMSIGDB_MANNO_MIDBRAIN_NEUROTYPE…	CENPE	CENPF	CENPK	CENPN	CENPQ	CENPU	CENPW	CEP152	CHAF1A	CHEK1	CHN2	CHRDL1	CHST9	CKAP2	CKAP2L	CKS1B	CKS2	CLSPN	CNN2	CNPY1	CNTN6	COL2A1	CPSF3	CRB2	CRNKL1
CELLMARKERMSIGDB_MANNO_MIDBRAIN_NEUROTYPE…	CELSR1	CENPE	CENPF	CENPQ	CENPU	CENPW	CEP152	CERKL	CFAP126	CFAP44	CHEK1	CHN2	CHRDL1	CKAP2L	CKS1B	CMTM8	CNPY1	CNTN4	CNTN6	COL27A1	COL2A1	CORIN	CRB2	CRISPLD1	CRYZ
CELLMARKERMSIGDB_MANNO_MIDBRAIN_NEUROTYPE…	CDON	CELSR1	CFAP126	CFAP44	CFAP45	CHN2	CHST3	CLU	COL18A1	COL21A1	COL23A1	COL4A5	CPNE2	CRB2	CREB5	CROT	CRYAB	CRYL1	CSPG5	CSPP1	CTDSP1	CCN2	CXCL2	CCN1	DACH1
CELLMARKERMSIGDB_MANNO_MIDBRAIN_NEUROTYPE…	C12orf76	OR11H2	NMNAT2	CFAP107	C1orf21	MIR9-1HG	C2orf…	COL8A1	CFAP299	C5orf49	IRF1-A…	C6orf1…	C8orf34	TCIM	C8orf48	CFAP77	C9orf24	CA12	CACNB2	CADM4	CAP2	CAST	ODAD1	CCDC146	CCDC17
CELLMARKERMSIGDB_MANNO_MIDBRAIN_NEUROTYPE…	CCDC144A	CCDC146	CCDC17	CFAP210	CCDC8	CCND2-A…	CDC25C	CDCA8	CDH13	CDK1	CDR1	CENPE	CENPN	CFAP44	CHD5	CHST3	CITED1	CMTM4	CMTM5	COL11A1	COL27A1	COL4A5	COLQ	CPNE2	CRB2
CELLMARKERMSIGDB_MANNO_MIDBRAIN_NEUROTYPE…	CHST6	COL27A1	COL2A1	COL9A3	CPNE5	CREB5	CRTAP	CSGALN…	CSMD1	CSMD2	CSMD3	CSPG5	CST3	CTNNA3	CTNND2	CYP26B1	DBI	DCLK1	DCLK2	DHFR	DHX32	DNAAF3	DNAH6	DNAH7	DNER
CELLMARKERMSIGDB_MANNO_MIDBRAIN_NEUROTYPE…	C12orf75	C12orf76	NMNAT2	CFAP107	CFAP276	C1orf226	C21or…	MEMO1	C2orf72	THUMPD2	DIPK2A	CFAP299	C5orf49	C6orf118	C6orf89	C8orf34	TCIM	CFAP77	C9orf24	CACNG4	CADM3	CALU	CAST	CATIP	ODAD1
CELLMARKERMSIGDB_MANNO_MIDBRAIN_NEUROTYPE…	CELF4	CELF5	CELSR3	CERS6	CHGA	CHML	CLVS1	CLVS2	CNR1	CNTN4	COMMD3…	COMTD1	CPEB3	CRMP1	CTNNA2	CXADR	DCLK1	DCX	DMRTA2	DNM3	DOCK3	DOK4	DOK6	DPYSL2	DPYSL3
CELLMARKERMSIGDB_MANNO_MIDBRAIN_NEUROTYPE…	CACNG8	CADM1	CALB1	CAMK2B	CAMK2N2	CASZ1	CBLN2	CCDC184	CCDC85A	CCSER1	CD2	CD47	CDH13	CDH8	CDK5R1	CDKL2	CDKN2D	CDRT4	TMEM12…	CELF3	CELF4	CELF5	CELF6	CGREF1	CHCHD1
CELLMARKERMSIGDB_MENON_FETAL_KIDNEY_0_CAP…	NBPF1	NKTR	RPS3AP6	ATRX	SET	RIF1	ZKSCA…	DNMT1	SUPT16H	CHD4	USP1	TPR	KAT6B	HNRNPA1L3	RSF1	BPTF	MCM7	SNRNP70	CUX1	TIA1	PRXL2A	CCDC14	RPS10	PEG10	NOP56
CELLMARKERMSIGDB_MENON_FETAL_KIDNEY_10_IM…	FOS	ZFP36L2	HLA-A	S100A11	TPT1	GRB2	TMSB10	DAB2	RPLP1	ARPC3	LRRFIP1	LGALS1	VAMP8	RPL27	CYBA	UBA52	OAZ1	EEF1B2P6	NEAT1	RPLP2	RBPJ	SNHG5	MYL6	CAPZB	PSAP
CELLMARKERMSIGDB_MENON_FETAL_KIDNEY_2_NEP…																									
CELLMARKERMSIGDB_MENON_FETAL_KIDNEY_3_STR…	VIM	KCNQ1OT1	ATP2B1	RRBP1	CD81	COL4A2	NR2F1	NCAM1	PBX1	MYL6	SOX4	SERPIN…	MAGED2	SEPTIN7	CCDC88A	TTC3	CHD3	FOS	GNAS	SELENOW	MACF1	C12orf57	MDK	VCAN	RPS4X…
CELLMARKERMSIGDB_MENON_FETAL_KIDNEY_6_COL…	S100A10	ARL4C	IER2	ENO1	PTPRF	SPATS2L	ZFP36…	S100A6	LHX1	CALM1	TSTD1	CD2AP	NEDD4L	MECOM	IVNS1ABP	EZR	EGR1	EFNB2	PPDPF	TSC22D1	LIMCH1	LRRFIP1	ID1	GSTP1	HSPB1
CELLMARKERMSIGDB_MURARO_PANCREAS_ACINAR_C…	CELA2B	MGST1	UBD	C3	C15orf…	CA12	GDF15	GATM	RNASE1	AKR1C1	NIBAN1	FGG	BNIP5	TC2N	MT1G	TMEM97	ATP10B	B3GNT7	CXCL2	ERP27	TCN1	PDZK1IP1	FKBP9…	UGT2B15	CTRL
CELLMARKERMSIGDB_MURARO_PANCREAS_ALPHA_CE…	FAM167A	ATXN2	COA3	YIPF6	PCNX1	SEC14L1	PRAF2	NDUFAF8	ITFG1	NDUFB2	ANKH	BEX2	ANAPC11	CRMP1	ATP6AP1	NDUFC1	STX16	ST8SIA3	TMEM258	TPD52	TMEM147	ITPK1	MANEAL	SGPL1	PROX1
CELLMARKERMSIGDB_MURARO_PANCREAS_BETA_CELL	GEM	CDKN1A	GNAS	SREBF1	GLIS3	HSP90AA1	DSP	EXPH5	GAD2	EDARADD	STX1A	MEIS2	EIF4A2	FTH1	PLCB4	SIPA1L2	PLEKHA6	ALCAM	C14orf…	SLC6A17	PTPN3	SUSD4	SYNE2	MNX1	FFAR4
CELLMARKERMSIGDB_MURARO_PANCREAS_ENDOTHEL…	PCAT19	ETS1	COL4A1	JAG1	PNP	PTPRB	COL4A2	TIE1	NQO1	ROBO4	GPR4	ARHGAP…	NRP1	ACE	TM4SF18	LAMA4	GIMAP7	ADAMTS4	A2M	CXCL12	HEY1	TP53I11	PXDN	ECSCR	SPARC
CELLMARKERMSIGDB_MURARO_PANCREAS_EPSILON_…																									
CELLMARKERMSIGDB_MURARO_PANCREAS_MESENCHY…	HEYL	MICAL2	CDH11	ISLR	TNFAIP6	ITGA1	PLAT	PRRX1	CTHRC1	C11orf…	HSD11B1	LAMC3	GREM1	BMP8A	HTRA1	THY1	EDNRA	FSTL1	COL18A1	ITGA11	LAMA2	BASP1	S1PR3	LTBP1	UACA
CELLMARKERMSIGDB_RUBENSTEIN_SKELETAL_MUSC…	CPVL	RNASET2	ITGB2	STX11	IL10RA	GLIPR1	AP1S2	PTPRC	PYCARD	SLC31A2	SAT1	CD52	PLEK	CD37	ARRB2	NFKBIZ	NR4A2	NAMPT	FYB1	GCA	CYBA	VAMP8	DUSP2	EIF4EBP1	CD53
CELLMARKERMSIGDB_RUBENSTEIN_SKELETAL_MUSC…	DAG1	RPS4X	DES	RPS3	RPL21	RPL41	TPD52…	RPL34	RPS13	RPS24	RPL9	ZFP36	DUSP1	SPRY1	HMGN2	RPL13	RPS9	RPL13A	NACA	CIRBP	RPL37A	PDLIM3	RPL11	RPS5	RPS12
CELLMARKERMSIGDB_RUBENSTEIN_SKELETAL_MUSC…	CDH6	DKK3	HCFC1R1	TMEM38B	DSTN	NDUFA4L2	C11or…	PPP1R1…	CALD1	GUCY1B1	NUDT4	OR51E1	FHL5	SVIL	SYNPO2	ADIRF	MSRB3	TPM1	HES4	CHCHD10	MAPRE2	OAZ2	NDUFA4	FILIP1	MRPS6
CELLMARKERMSIGDB_TRAVAGLINI_LUNG_ALVEOLAR…	RGCC	PDPN	PDGFA	COL4A3	CRYAB	FHL1	GPRC5A	CST6	CYP4B1	ST6GAL…	MS4A15	WNT7A	PCYOX1	PAPSS2	CCN1	DENND3	COL4A4	MYADM	SUSD2	AQP4	CD151	LMO7	TSPAN…	TAGLN	ICAM1
CELLMARKERMSIGDB_TRAVAGLINI_LUNG_BASOPHIL…	CALB2	BHLHE40	CD83	SAMSN1	KDM6B	TNFAIP3	PTGS2	SYAP1	CKLF	TIMP3	REL	MYADM	ELL2	NFKBIZ	EGR3	RAB27B	CAVIN2	STXBP6	ELF1	SLC2A3	PRDX6	ENPP3	BACE2	NDUFA4	ITM2C
CELLMARKERMSIGDB_TRAVAGLINI_LUNG_CAPILLAR…																									
CELLMARKERMSIGDB_TRAVAGLINI_LUNG_CAPILLAR…	CHMP2A	SERF2	MRFAP1	RBM17	ENSA	TLE5	RBX1	DYNC1L…	NEDD8	NDUFA13	LSM3	COX6C	LAMTOR1	NDUFB1	DBI	ARPC1A	HMGN2	PSMA6	SEPTIN7	COX7A2	CMPK1	SLIRP	NDUFA3	SELENOK	UQCRQ
CELLMARKERMSIGDB_TRAVAGLINI_LUNG_CD4_MEMO…	CRIP2	CD96	SPOCK2	TRADD	RPS3	CAPN2	ICAM3	SUSD3	PCED1B…	SKAP1	RORA	HINT1	ICOS	LINC00892	TXNIP	CCR6	ETS1	CORO1A	IL2RG	SLC38A1	HNRNPLL	ITK			
CELLMARKERMSIGDB_TRAVAGLINI_LUNG_EREG_DEN…	SDC2	AIF1	IL13RA1	SGK1	YBX3	HLA-DPA1	METRNL	TREM1	SDS	G0S2	LST1	RGS2	CXCL3	CXCL8	TYMP	AREG	C1orf162	HIF1A	CLEC7A	OSM	VCAN	HLA-DMB	CDKN1A	C15orf48	YWHAH
CELLMARKERMSIGDB_TRAVAGLINI_LUNG_IGSF21_D…																									
CELLMARKERMSIGDB_TRAVAGLINI_LUNG_MESOTHEL…	SLC12A8	SYCE1L	SMOX	DMKN	VCAM1	RGS4	RPLP0…	TFRC	CFI	IL18	SULF1	ABRACL	SULF2	DSP	ANKRD28	PDZK1IP1	HLA-F	CASP4	GJA1	S100A10	HAS1	PKP2	IL1RAP	RPL37	WFDC2
CELLMARKERMSIGDB_TRAVAGLINI_LUNG_MYOFIBRO…	S100A10	CTHRC1	PRELP	NEDD9	WNT5A	PDLIM3	USP18	COMP	VCAN	PKIG	PTGER1	VIM	CCN2	COL1A1	NTM	CNN3									
CELLMARKERMSIGDB_TRAVAGLINI_LUNG_NATURAL_…																									
CELLMARKERMSIGDB_TRAVAGLINI_LUNG_NEUTROPH…	FOS	YPEL3	PHC2	RGS2	VNN2	FTL	LRRK2	HLA-B	MIDN	IRS2	SIRPB1	MCL1	KDM6B	DYSF	S100A11	PADI2	MEGF9	VSIR	KCNJ15	BCL2A1	MSRB1	ABHD5	FTLP3	XPO6	VNN3P
CELLMARKERMSIGDB_TRAVAGLINI_LUNG_OLR1_CLA…	NFKB1	GRINA	CLEC4E	VEGFA	MXD1	TNIP3	S100A…	FPR1	FTH1	TNFAIP3	BACH1	CYTOR	PLAUR	KYNU	CCL20	OLR1	LITAF	RAB31	MIR394…	TYMP	LYZ	IVNS1ABP	TNFAI…	MAP3K20	GPR84
CELLMARKERMSIGDB_TRAVAGLINI_LUNG_PLATELET…	CLEC1B	GSTO1	KIF2A	OAZ1	PDGFA-…	GP1BA	TAGLN2	LCN2	RNF11	PDZK1I…	CLDN5	MYL12A	DAPP1	LAMTOR1	PDLIM1	ETFA	NAT8B	YWHAH	C19orf…	ABCC3	GMPR	SH3BGRL2	MPIG6B	SMOX	PTPN18
CELLMARKERMSIGDB_TRAVAGLINI_LUNG_PROLIFER…	ESCO2	MND1	NCAPH	UBE2T	HJURP	CENPW	KIF23	H4C3	CDK1	H1-1	TROAP	GAPDH	CCNB1	PIMREG	MELK	KIF2C	CENPK	KRT5	PKMYT1	FEN1	CENPH	PTMA	DLGAP5	CDCA3	ARHGA…
CELLMARKERMSIGDB_TRAVAGLINI_LUNG_PROLIFER…	AURKB	LGALS3	CDT1	FN1	LGALS3…	CCDC34	SGO1	NCAPG	NDC80	UBE2T	LPL	SMC2	MGST3	CENPU	TROAP	GPNMB	CENPA	MGST1	ALDH2	DYNC2I2	FAM111B	SERPING1	PHLDA3	TCF19	CYP27…
CELLMARKERMSIGDB_TRAVAGLINI_LUNG_PROXIMAL…	PTPRZ1	RPS4X	TSHZ2	DLK2	NPM1	AQP5	BEND5	HCAR2	NACA	NSG1	LDHB	SEMA5A	ISLR	SNHG29	EIF3F	RPL13A	VAV3	SLC1A5	RPS18	GYPC	SNX31	ALDH2	RPL8	IL33	HS3ST1
CELLMARKERMSIGDB_TRAVAGLINI_LUNG_SEROUS_C…	CXCL17	GGTA1	PRB4	SFRP1	TPD52L1	RTN1	SELEN…	ELF5	PAIP2B	RBP7	NPDC1	APIP	FDCSP	CYP4X1	CRISP3	SLC5A1	STK39	AQP5	FKBP11	AIF1L	GCNT3	ALDH1L1	CA2	GMDS	CLDN10
CELLMARKERMSIGDB_TRAVAGLINI_LUNG_SIGNALIN…	ABCA3	DUOX1	ETV5	PPP2R5A	CREB3L1	CYB5A	ATP11A	FNIP2	TXNIP	PARM1	CHIAP2	TMSB4X	CD44	C1orf21	TXNDC17	FASN	IRX2-DT	QSOX1	TMEM125	CD81	SCD	MALL			
CELLMARKERMSIGDB_TRAVAGLINI_LUNG_TREM2_DE…	MARCO	GSN	IL4I1	UBD	ACP2	FUOM	PTMS	IFI30	A2M	PLXDC2	NR1H3	ACE	SERPING1	C2	DAB2	LGALS3	CYBB	CD81	MMP14	HEXB	MSR1	MAFB	ENG	TYMP	CTSL
CELLMARKERMSIGDB_TRAVAGLINI_LUNG_VASCULAR…	BGN	CRIM1	SYTL2	CALD1	MYLK	TPM4	MSRB3	PTMA	SEPTIN7	CNN3	FRZB	RPS7													
CELLMARKERMSIGDB_TRAVAGLINI_LUNG_VEIN_CELL																									
CELLMARKERMSIGDB_VANGURP_PANCREATIC_ALPHA…																									
CELLMARKERMSIGDB_VANGURP_PANCREATIC_DELTA…	PCP4	PCSK1	PDLIM4	PKIB	PLEKHB1	PRG4	PSIP1	RASSF6	RBP4	RGS2	S100A6	SCD5	SEC11C	SERPINA1	SLC17A6	SLC38A1	SORL1	SST	SYNE2	TENM3	TIMP2	TMSB4X	TPPP3	UNC5B	VIM
CELLMARKERMSIGDB_VANGURP_PANCREATIC_GAMMA…																									
CELLMARKERMSIGDB_ZHENG_CORD_BLOOD_C1_PUTA…	KCNE3	LCN2	LGALSL	LIMS1	LRRC32	LTBP1	LY6G6F	LY6G6D	MFAP3L	MMRN1	MOB3C	MYL12A	MYLK	NEXN	NID1	NRGN	PBK	PDE3A	PDE5A	PDGFA	PDGFB	PDLIM1	PF4	PF4V1	PLEK
CELLMARKERMSIGDB_ZHENG_CORD_BLOOD_C10_MUL…	LINC004…	LINC008…	LPIN1	LST1	LTB	LUC7L3	LZTFL1	MALAT1	MAP1A	MED13L	MEF2A	MME	MZB1	NCOA7	NEGR1	NIN	OSBPL3	PACSIN1	PAG1	PIK3R1	PRAM1	PREX1	PRKCB	QPRT	RAB31
CELLMARKERMSIGDB_ZHENG_CORD_BLOOD_C2_PUTA…	MALAT1	MIR4435…	MLEC	MS4A2	MS4A3	MT-TL1	NDEL1	NEDD9	P2RX5	P4HB	PABPC4	PDIA3	PIM2	PLEK	PLGRKT	PLIN2	PNMT	PNN	POU2F2	PPP1R15A	PSME4	PTMA	RAB11…	S100A4	S100A6
CELLMARKERMSIGDB_ZHENG_CORD_BLOOD_C4_PUTA…	MOB1B	MPP1	MT-RNR2	MYB	MYCT1	NFIA	NFKBIZ	NLK	NOP58	PBX1	PDZD8	PHACTR4	PHTF1	PKIG	PLXDC2	PNN	PRKACB	PSTPIP2	RAPGEF2	RHOH	RNF130	RSBN1	SDCBP	SERPINB1	SESN3
CELLMARKERMSIGDB_ZHENG_CORD_BLOOD_C5_SIMI…	HLA-DQB1	MECOM	ZFP36L2	MAFF	H2AC20	CD52	LPAR6	HLA-DRA	CLEC9A	CXCL8	MLLT3	RIPK2	HLF	IER2	CAVIN2	TPT1	PNRC1	HMGA2	TRA2B	NR4A1	MYCT1	MDK	SKAP1	HLA-DRB1	DUSP2
CELLMARKERMSIGDB_ZHENG_CORD_BLOOD_C6_HSC_…	MEIS1	LINC-PI…	MLLT3	MMRN1	MT-ND2	MT-ND5	NAMPT	NEAT1	NFKBIA	NR4A1	NR4A2	PABPC1	PELI1	PIM3	PPP1CB	PREX2	PRKCH	PTPRC	RANBP2	RILPL2	RIPK2	RNF125	RNU2-…	SAMSN1	SAT1
CELLMARKERMSIGDB_ZHENG_CORD_BLOOD_C7_PUTA…	USP36	BCL7A	IFNGR1	MIS18BP1	SELL	TRIM24	IFI16	HNRNPH1	KRAS	TCF7L2	TAOK3	STARD4…	CYTIP	DEK	SPN	FAM161A	SPTBN1	SMIM24	CHD1	IFITM3	ANKRD26	SLK	NPDC1	WDR49	CALCO…
CELLMARKERMSIGDB_ZHENG_CORD_BLOOD_C8_PUTA…	EEF1B2	PADI4	HCST	ERGIC1	S100A4	BCL3	DYNLT1	VMP1	NACA	RASGRP2	MT-ND3	NUCB2	HOPX	MT-CO1	NPDC1	HLA-DRA	PRAM1	PROM1	COMMD6	GSTP1	SNHG5	UBA52	IFITM1	PSME1	MT-CYB
CELLMARKERMSIGDB_ZHONG_PFC_C1_ASTROCYTE																									
CELLMARKERMSIGDB_ZHONG_PFC_C1_DLX5_POS_IN…																									
CELLMARKERMSIGDB_ZHONG_PFC_C2_ASCL1_POS_A…																									

Table S5: Cell state marker gene list

CELLMARKERCAN_ACINAR_CELLS																									
CELLMARKERMSIGDB_ZHONG_PFC_C2_UNKNOWN_NPC	NEIL3	NEK2	NUSAP1	OIP5	PHF19	PIF1	PLK1	PRR11	PSRC1	PTTG1	RACGAP1	RANGAP1	REEP4	SGO2	SPAG5	SPDL1	TACC3	TOP2A	TPX2	TRIM59	TROAP	TTK	TUBA1C	TUBB4B	TUBB6
CELLMARKERMSIGDB_ZHONG_PFC_C3_ASTROCYTE	ADAP1	MAPK11	RIMBP2	FAM89B	ARPP21	SLC25A29	CLVS2	PDIA2	SYT13	SRCIN1	EPB41L1	CACNA1E	NEXMIF	PPFIA2	CARMIL3	SEMA3C	STX1A	SLIT1	PRDM2	RNF165	NEFM	SCG2	OBSCN	CDKL2	NFIX
CELLMARKERMSIGDB_ZHONG_PFC_C3_HOPX_POS_OPC																									
CELLMARKERMSIGDB_ZHONG_PFC_C3_MICROGLIA	NAV1	MVD	MOAP1	ATP6V0E2	AMER2	SUZ12P1	DAAM1	NGRN	CHRNA4	ATXN2	PCBP4	SDC3	AKT2	LSM14B	IMPDH1	LRRN3	GCSH	PFN2	ARL4C	STARD10	NTRK2	ANK2	PENK	MAP1A	TSC2
CELLMARKERMSIGDB_ZHONG_PFC_C3_UNKNOWN_INP																									
CELLMARKERMSIGDB_ZHONG_PFC_C4_UNKNOWN_INP	NHLH2	NKAIN3	NUMBL	OCIAD2	P2RX5-…	PCBP4	PHF21B	PHLDB2	POLR2H	POLR2L	PRMT9	PSMC6	PSMG1	RGS16	RNF138	RPAP2	RPAP3	RSBN1	S100PBP	SERINC2	SERPIN…	SLC4A7	SLC6A…	SNHG8	SOCS4
CELLMARKERMSIGDB_ZHONG_PFC_C6_PLXNA4_POS_…																									
CELLMARKERMSIGDB_ZHONG_PFC_C7_ORG_UNDERGO…	NTM	PTPRD	RBFOX1	RNF213	RUNX1T1	SATB2	SCD5	SEMA3C	SERP2	SH3PXD…	SHC2	SLA	SNAP91	SORL1	SRGAP1	TMEM132B	TNIK	TTC14							
CELLMARKERMSIGDB_ZHONG_PFC_C8_ORG_PROLIFE…	SFRP2	SGO2	SHCBP1	SMTN	TACC3	TMEM256	TPX2	TROAP	TUBA1C	TUBB4B	TUBB6	TXNDC12	UNC50												
CELLMARKERMSIGDB_ZHONG_PFC_C8_UNKNOWN_NEU…	MT3	MVD	NCALD	NECAB1	NELL2	NEUROD2	NEURO…	NPY1R	NTRK2	PALM	PDXP	PENK	PHYHIP	PIK3R1	PLXNA4	POU3F2	PPFIA3	PTPRK	R3HDM1	RBFOX1	RGS11	SCD	SEMA3C	SLA	SMARC…
CELLMARKERMSIGDB_ZHONG_PFC_C9_ORG_OTHER	NRG1	NSDHL	NTM	OAT	PDLIM3	PDPN	PEA15	PLPP3	PSAT1	PTN	PTPRN2	PTPRZ1	S100A13	S100A16	SAT1	SC5D	SCRG1	SEL1L3	SELENOW	SLC1A3	SLC9A3…	SLCO1C1	SNX5	SPARC	STOM
CELLMARKERMSIGDB_ZHONG_PFC_HES1_POS_C1_NPC																									
CELLMARKERMSIGDB_ZHONG_PFC_MAJOR_TYPES_AS…	LGALS3	EEPD1	LRRC17	GRM3	MT2A	TFPI	ANOS1	NHSL1-…	PMP22	GJA1	RAMP3	PAQR8	EFNA1	ACSBG1	PRSS35	CASTOR1	HAPLN1	HAPLN3	GFAP	FAM107A	PRODH	SLC25A18	GATM	CD82	NCAN
CELLMARKERMSIGDB_ZHONG_PFC_MAJOR_TYPES_EX…																									
CELLMARKERMSIGDB_ZHONG_PFC_MAJOR_TYPES_MI…	CD14	MNDA	SAMSN1	FYB1	CYBB	TLR4	ADAM28	APBB1IP	CD74	CD84	PDK4	PYCARD	STING1	SERPINF1	CTSC	CD36	RNASET2	PTPRC	MS4A7	HPGDS	HLA-E	GPR183	DAB2	HTRA1	VAMP8
CELLMARKERMSIGDB_ZHONG_PFC_MAJOR_TYPES_OPC	TRIB2	PDE4B	DBI	MMP2	LRRC4C	SPRY1	RHOC	MT3	SERPIN…	SOX6	CD82	CNTN1	ITGB8	ETV5	ETV1	CSPG5	SPARC	NOVA1	PON2	LMF1	CST3	RFTN2	ITM2C	PTPRZ1	CADM2

CELLMARKERCAN_ACINAR_CELLS																													
CELLMARKERCAN_ADIPOCYTE_PROGENITOR_CELLS																													
CELLMARKERCAN_ADRENERGIC_NEURONS																													
CELLMARKERCAN_AIRWAY_EPITHELIAL_CELLS																													
CELLMARKERCAN_ALPHA_CELLS																													
CELLMARKERCAN_ALVEOLAR_MACROPHAGES																													
CELLMARKERCAN_ASTROCYTES																													
CELLMARKERCAN_B_CELLS																													
CELLMARKERCAN_B_CELLS_MEMORY																													
CELLMARKERCAN_B_CELLS_NAIVE																													
CELLMARKERCAN_BASOPHILS																													
CELLMARKERCAN_BERGMANN_GLIA																													
CELLMARKERCAN_BETA_CELLS																													
CELLMARKERCAN_CAJAL_RETZIUS_CELLS																													
CELLMARKERCAN_CARDIAC_STEM_AND_PRECURSOR_…																													
CELLMARKERCAN_CARDIOMYOCYTES	MITF	ZFPM2	BDNF	GJA5	FMOD	MTTP	SLC5A1	ADIPOQ	TRPV1	HAMP	SGPL1	HMGB2	STC1	FGF23	STRN	HEY2	ADIPOR1	ADIPOR2	PDE1A	BMP4	CASQ2	TPM1							
CELLMARKERCAN_CHOLANGIOCYTES																													
CELLMARKERCAN_CHOLINERGIC_NEURONS																													
CELLMARKERCAN_CHONDROCYTES																													
CELLMARKERCAN_CHOROID_PLEXUS_CELLS																													
CELLMARKERCAN_CILIATED_CELLS																													
CELLMARKERCAN_DELTA_CELLS																													
CELLMARKERCAN_DOPAMINERGIC_NEURONS																													
CELLMARKERCAN_DUCTAL_CELLS																													
CELLMARKERCAN_EMBRYONIC_STEM_CELLS																													
CELLMARKERCAN_ENTERIC_GLIA_CELLS																													
CELLMARKERCAN_ENTERIC_NEURONS																													
CELLMARKERCAN_ENTEROCYTES	DMBT1	ABCG8	PLIN2	GSTA1	CYBRD1	ABCG2	NR1H4	DAB1	TM6SF2	NR5A2	PDZK1	ACSL5	AQP11	AQP7	AQP8	HMOX1	SLC39A5	SLC51A	TRPM6	MOGAT2	GLS	MAOA	DGAT1	DGAT2					
CELLMARKERCAN_ENTEROENDOCRINE_CELLS																													
CELLMARKERCAN_EPENDYMAL_CELLS																													
CELLMARKERCAN_EPSILON_CELLS																													
CELLMARKERCAN_FOLLICULAR_CELLS																													
CELLMARKERCAN_FOVEOLAR_CELLS																													
CELLMARKERCAN_GAMMA_DELTA_T_CELLS																													
CELLMARKERCAN_GERM_CELLS																													
CELLMARKERCAN_GLUTAMINERGIC_NEURONS																													
CELLMARKERCAN_GRANULOSA_CELLS																													
CELLMARKERCAN_HEMATOPOIETIC_STEM_CELLS	MAFF	ITGA6	GJB1	CDH2	ALDH1A1	GFI1B	NOTCH1	NOTCH2	PLXDC2	ESAM	ACE	TRPC6	HOXB5	TET2															
CELLMARKERCAN_IMMATURE_NEURONS																													
CELLMARKERCAN_INTERNEURONS																													
CELLMARKERCAN_IONOCYTES																													
CELLMARKERCAN_KERATINOCYTES																													
CELLMARKERCAN_KUPFFER_CELLS																													
CELLMARKERCAN_LEYDIG_CELLS																													
CELLMARKERCAN_LOOP_OF_HENLE_CELLS																													
CELLMARKERCAN_LUMINAL_EPITHELIAL_CELLS																													
CELLMARKERCAN_LUTEAL_CELLS																													
CELLMARKERCAN_MEGAKARYOCYTES																													
CELLMARKERCAN_MELANOCYTES																													
CELLMARKERCAN_MESANGIAL_CELLS																													
CELLMARKERCAN_MESOTHELIAL_CELLS																													
CELLMARKERCAN_MICROFOLD_CELLS																													
CELLMARKERCAN_MICROGLIA																													
CELLMARKERCAN_MONOCYTES																													
CELLMARKERCAN_MOTOR_NEURONS																													
CELLMARKERCAN_MULLER_CELLS																													
CELLMARKERCAN_MYELOID_DERIVED_SUPPRESSOR_…																													
CELLMARKERCAN_MYOBLASTS																													
CELLMARKERCAN_MYOEPITHELIAL_CELLS																													
CELLMARKERCAN_MYOFIBROBLASTS																													
CELLMARKERCAN_NEUROBLASTS																													
CELLMARKERCAN_NEUROENDOCRINE_CELLS																													
CELLMARKERCAN_NEURONS	DYNC1I1	PRUNE2	CACNA2D2	SLC17A6	TUBB3	ELAVL3																							
CELLMARKERCAN_NEUTROPHILS																													
CELLMARKERCAN_NK_CELLS	SPON2	LAIR2	HOPX	CD8A																									
CELLMARKERCAN_NORADRENERGIC_NEURONS																													
CELLMARKERCAN_NUOCYTES																													
CELLMARKERCAN_OLIGODENDROCYTES	MAG	SLC25A29	SLC25A19	PLEKHH1	TMEM88B	DBNDD2	EFNB3	SGK3	PNPLA2	ARRDC2	ENPP2	PLP1	ITPR2	MBP															
CELLMARKERCAN_OSTEOBLASTS																													
CELLMARKERCAN_OSTEOCLAST_PRECURSOR_CELLS																													
CELLMARKERCAN_OSTEOCLASTS																													
CELLMARKERCAN_OSTEOCYTES																													
CELLMARKERCAN_PANETH_CELLS																													
CELLMARKERCAN_PARATHYROID_CHIEF_CELLS																													
CELLMARKERCAN_PERICYTES																													
CELLMARKERCAN_PERITUBULAR_MYOID_CELLS																													
CELLMARKERCAN_PHOTORECEPTOR_CELLS																													
CELLMARKERCAN_PLASMA_CELLS	IGHGP	IGLC3	JCHAIN	IGHA1	IGHG4	IGHA2	IGLV3-1	IGLC7	SSR4	IL16	BHLHA15	PPIB																	
CELLMARKERCAN_PLASMACYTOID_DENDRITIC_CELLS																													
CELLMARKERCAN_PODOCYTES	COL4A4	AIF1L	MET	EPB41L5	DOCK1	AJUBA	CD2AP	GOLIM4	ZBTB8A	TOB1	WSB2	EMC2	EHD4	ZFP91	ETF1	EMC7	LUC7L2	MAPT	CDKN1C										
CELLMARKERCAN_PROXIMAL_TUBULE_CELLS	NAPSA																												
CELLMARKERCAN_PULMONARY_ALVEOLAR_TYPE_I_C…																													
CELLMARKERCAN_PULMONARY_ALVEOLAR_TYPE_II_…																													
CELLMARKERCAN_PURKINJE_NEURONS																													
CELLMARKERCAN_RETICULOCYTES																													
CELLMARKERCAN_RETINAL_GANGLION_CELLS																													
CELLMARKERCAN_RETINAL_PROGENITOR_CELLS																													
CELLMARKERCAN_SATELLITE_CELLS																													
CELLMARKERCAN_SCHWANN_CELLS																													
CELLMARKERCAN_SEROTONERGIC_NEURONS																													
CELLMARKERCAN_SERTOLI_CELLS																													
CELLMARKERCAN_SMOOTH_MUSCLE_CELLS	BGN	MYL9	MYLK	PCP4L1	SNCG	PLN	NRP2																						
CELLMARKERCAN_STROMAL_CELLS																													
CELLMARKERCAN_T_CELLS																													
CELLMARKERCAN_T_CYTOTOXIC_CELLS																													
CELLMARKERCAN_T_FOLLICULAR_HELPER_CELLS																													
CELLMARKERCAN_T_MEMORY_CELLS																													
CELLMARKERCAN_TRANSIENT_CELLS																													
CELLMARKERCAN_TRICHOCYTES																													
CELLMARKERCAN_TRIGEMINAL_NEURONS																													
CELLMARKERCAN_TROPHOBLAST_CELLS																													
CELLMARKERCAN_TROPHOBLAST_PROGENITOR_CELLS																													
CELLMARKERCAN_TUFT_CELLS																													
CELLMARKERCANENDOTHELIALCELLSAORTA																													
CELLMARKERCUR_N_ABNORMAL_MYELOID_CELL																													

Table S5: Cell state marker gene list

CELLMARKERCAN_ACINAR_CELLS																													
CELLMARKERCUR_N_ABNORMAL_PLASMA_CELL																													
CELLMARKERCUR_N_ACTIVATED_DENDRITIC_CELL																													
CELLMARKERCUR_N_ACTIVATED_EFFECTOR_CELL																													
CELLMARKERCUR_N_ACTIVATED_HEPATIC_STELLAT…																													
CELLMARKERCUR_N_ACTIVATED_LANGERHANS_CELL																													
CELLMARKERCUR_N_ACTIVATED_MACROPHAGE																													
CELLMARKERCUR_N_ACTIVATED_MEMORY_T_CELL																													
CELLMARKERCUR_N_ACTIVATED_NATURAL_KILLER_…																													
CELLMARKERCUR_N_ACTIVATED_REGULATORY_T_CE…																													
CELLMARKERCUR_N_ADIPOCYTE_PROGENITOR_CELL																													
CELLMARKERCUR_N_ADRENAL_MEDULLA_CELL																													
CELLMARKERCUR_N_ADULT_SPERMATOGONIA																													
CELLMARKERCUR_N_ADVENTITIAL_CELL																													
CELLMARKERCUR_N_AEROCYTE																													
CELLMARKERCUR_N_AIRWAY_SMOOTH_MUSCLE_CELL																													
CELLMARKERCUR_N_ALVEOLAR_CELL_TYPE_1																													
CELLMARKERCUR_N_ALVEOLAR_CELL_TYPE_2																													
CELLMARKERCUR_N_ALVEOLAR_EPITHELIAL_CELL_…																													
CELLMARKERCUR_N_ALVEOLAR_EPITHELIAL_CELL_…																													
CELLMARKERCUR_N_ALVEOLAR_PNEUMOCYTE_TYPE_I																													
CELLMARKERCUR_N_ALVEOLAR_PNEUMOCYTE_TYPE_…																													
CELLMARKERCUR_N_AMACRINE_CELL																													
CELLMARKERCUR_N_AMNIOTIC_FLUID_STEM_CELL																													
CELLMARKERCUR_N_ANTERIOR_CELL																													
CELLMARKERCUR_N_ANTERIOR_HINDBRAIN_CELL																													
CELLMARKERCUR_N_ANTI_TUMOR_IMMUNE_CELL																													
CELLMARKERCUR_N_ANTIGEN_PRESENTATION_CANC…																													
CELLMARKERCUR_N_ASTROCYTE	SLC4A4	TTPA	SYNE3	SOX2	TSPO	APOE	HOPX	FAM10…	IQCK	MEF2C	LRP1	PISD	MPG	IL17A	AGT	HES1	SPARCL1	CDH11	ID1										
CELLMARKERCUR_N_ATRIAL_CELL																													
CELLMARKERCUR_N_ATRIOVENTRICULAR_CANAL_CE…																													
CELLMARKERCUR_N_ATYPICAL_MEMORY_B_CELL																													
CELLMARKERCUR_N_B_2_CELL																													
CELLMARKERCUR_N_B1_CELL																													
CELLMARKERCUR_N_B10_REGULATORY_B_CELL																													
CELLMARKERCUR_N_BASAL_CELL																													
CELLMARKERCUR_N_BASAL_EPITHELIAL_CELL																													
CELLMARKERCUR_N_BASAL_KERATINOCYTE																													
CELLMARKERCUR_N_BASAL_LIKE_CELL																													
CELLMARKERCUR_N_BASAL_PROGENITOR_CELL																													
CELLMARKERCUR_N_BASOPHIL																													
CELLMARKERCUR_N_BILIARY_CELL																													
CELLMARKERCUR_N_BONE_MARROW_STEM_CELL																													
CELLMARKERCUR_N_BONE_SPECIFIC_CELL																													
CELLMARKERCUR_N_BRAIN_CELL																													
CELLMARKERCUR_N_CANCER_CELL	MKI67	DCX	ACYP2	LCN2	SLC22A…	RSPH1	CAPS	PMEL	CD276	ASPM	CHD5	TOP2A	PCDH9	EZH2	PBK	AQP4	SOX2	POU5F1	PROM1	PDGFRA	S100B	SAMD9	COL1A2	PTPRZ1	MARCK…	GPM6A	TUBA1A	MIR9-…	TUBB2B
CELLMARKERCUR_N_CANCER_STEM_LIKE_CELL																													
CELLMARKERCUR_N_CAPILLARY_CELL																													
CELLMARKERCUR_N_CAR_T_CELL																													
CELLMARKERCUR_N_CARDIAC_CELL																													
CELLMARKERCUR_N_CARDIOMYOCYTE																													
CELLMARKERCUR_N_CARDIOSPHERE_DERIVED_CELL																													
CELLMARKERCUR_N_CARDIOVASCULAR_PROGENITOR…																													
CELLMARKERCUR_N_CAUDAL_CORTEX_CELL																													
CELLMARKERCUR_N_CD14_MONOCYTE																													
CELLMARKERCUR_N_CD16_MONOCYTE																													
CELLMARKERCUR_N_CD4_CD28_T_CELL																													
CELLMARKERCUR_N_CD4_PERIPHERALLY_DERIVED_…																													
CELLMARKERCUR_N_CD4_T_CELL																													
CELLMARKERCUR_N_CD56BRIGHT_NATURAL_KILLER…																													
CELLMARKERCUR_N_CD56DIM_NATURAL_KILLER_CE…																													
CELLMARKERCUR_N_CD8_T_CELL																													
CELLMARKERCUR_N_CENTRAL_MEMORY_T_CELL																													
CELLMARKERCUR_N_CENTRAL_MEMORY_T_HELPER_1…																													
CELLMARKERCUR_N_CENTRAL_NERVOUS_SYSTEM_ST…																													
CELLMARKERCUR_N_CHANDELIER_CELL																													
CELLMARKERCUR_N_CHIEF_CELL																													
CELLMARKERCUR_N_CHONDROCYTE																													
CELLMARKERCUR_N_CHROMAFFIN_CELL																													
CELLMARKERCUR_N_CIRCULATING_ANGIOGENIC_CE…																													
CELLMARKERCUR_N_CIRCULATING_FETAL_CELL																													
CELLMARKERCUR_N_CIRCULATING_NATURAL_KILLE…																													
CELLMARKERCUR_N_CIRCULATING_PRECURSOR_CELL																													
CELLMARKERCUR_N_CIRCULATING_PROGENITOR_CE…																													
CELLMARKERCUR_N_CIRCULATING_T_FOLLICULAR_…																													
CELLMARKERCUR_N_CIRCULATING_TUMOR_CELL																													
CELLMARKERCUR_N_CLASS_SWITCHED_PLASMABLAST																													
CELLMARKERCUR_N_CLASSICAL_B_CELL																													
CELLMARKERCUR_N_CLASSICAL_CANCER_ASSOCIAT…																													
CELLMARKERCUR_N_CLASSICAL_MONOCYTE																													
CELLMARKERCUR_N_CLAUDIN_LOW_CELL																													
CELLMARKERCUR_N_COLLECTING_DUCTAL_CELL																													
CELLMARKERCUR_N_COLONCYTE																													
CELLMARKERCUR_N_COLORECTAL_STEM_CELL																													
CELLMARKERCUR_N_COLUMNAR_EPITHELIAL_CELL																													
CELLMARKERCUR_N_COMMON_LYMPHOID_PROGENITO…																													
CELLMARKERCUR_N_COMMON_MYELOID_PROGENITOR…																													
CELLMARKERCUR_N_COMPLEMENT_SECRETING_CANC…																													
CELLMARKERCUR_N_CONE_PHOTORECEPTOR_CELL																													
CELLMARKERCUR_N_CONNECTING_TUBULAR_CELL																													
CELLMARKERCUR_N_CONVENTIONAL_CD4_T_CELL																													
CELLMARKERCUR_N_CORNEAL_ENDOTHELIAL_CELL																													
CELLMARKERCUR_N_CORNEAL_EPITHELIAL_CELL																													
CELLMARKERCUR_N_CORNEOCYTE_LIKE_CELL																													
CELLMARKERCUR_N_CORTICAL_CELL																													
CELLMARKERCUR_N_CORTICAL_STROMAL_CELL																													
CELLMARKERCUR_N_CORTICOTROPH_TUMOR_CELL																													
CELLMARKERCUR_N_CRANIAL_NEURAL_CREST_CELL																													
CELLMARKERCUR_N_CRYPT_CELL																													
CELLMARKERCUR_N_CRYPT_STEM_CELL																													
CELLMARKERCUR_N_CYCLING_B_CELL																													
CELLMARKERCUR_N_CYCLING_EPITHELIAL_CELL																													
CELLMARKERCUR_N_CYTOTOXIC_CD8_T_CELL																													
CELLMARKERCUR_N_CYTOTOXIC_DOUBLE_POSITIVE…																													
CELLMARKERCUR_N_CYTOTOXIC_T_CELL																													

CELLMARKERCAN_ACINAR_CELLS																													
CELLMARKERCUR_N_CYTOTOXIC_T_CELL_2																													
CELLMARKERCUR_N_DAMAGE_ASSOCIATED_TRANSIE…																													
CELLMARKERCUR_N_DARK_ZONE_CELL																													
CELLMARKERCUR_N_DEDIFFERENTIATION_ADIPOCY…																													
CELLMARKERCUR_N_DEEP_LAYER_NEURON																													
CELLMARKERCUR_N_DEFINITIVE_ENDODERM_CELL																													
CELLMARKERCUR_N_DELTA_CELL																													
CELLMARKERCUR_N_DEMYELINATING_MICROGLIAL_…																													
CELLMARKERCUR_N_DENDRITIC_CELL_2A																													
CELLMARKERCUR_N_DENDRITIC_CELL_LINEAGE																													
CELLMARKERCUR_N_DENTAL_PULP_CELL																													
CELLMARKERCUR_N_DERMAL_CELL																													
CELLMARKERCUR_N_DEUTEROSOMAL_CELL																													
CELLMARKERCUR_N_DIFFUSE_GASTRIC_CANCER_CE…																													
CELLMARKERCUR_N_DISTAL_CELL																													
CELLMARKERCUR_N_DISTAL_STEM_CELL																													
CELLMARKERCUR_N_DISTAL_TUBULAR_CELL																													
CELLMARKERCUR_N_DIVIDING_CELL																													
CELLMARKERCUR_N_DIVIDING_PLASMA_B_CELL																													
CELLMARKERCUR_N_DIVIDING_T_CELL																													
CELLMARKERCUR_N_DOPAMINERGIC_NEURON																													
CELLMARKERCUR_N_DORSAL_ANTERIOR_HINDBRAIN…																													
CELLMARKERCUR_N_DORSAL_LATERAL_GANGLIONIC…																													
CELLMARKERCUR_N_DORSAL_MEDIAL_GANGLIONIC_…																													
CELLMARKERCUR_N_DOUBLE_NEGATIVE_B_CELL																													
CELLMARKERCUR_N_DOUBLE_NEGATIVE_T_CELL																													
CELLMARKERCUR_N_DOUBLE_POSITIVE_T_CELL																													
CELLMARKERCUR_N_DOUBLE_POSITIVE_TREG_CELL																													
CELLMARKERCUR_N_EARLY_HEMANGIOBLAST																													
CELLMARKERCUR_N_EARLY_PROXIMAL_TUBULE_CELL																													
CELLMARKERCUR_N_EARLY_RESPONSE_SECRETORY_…																													
CELLMARKERCUR_N_EFFECTOR_CHONDROCYTE																													
CELLMARKERCUR_N_EFFECTOR_MEMORY_T_CELL																													
CELLMARKERCUR_N_EFFECTOR_NATURAL_KILLER_C…																													
CELLMARKERCUR_N_ELONGATING_CELL																													
CELLMARKERCUR_N_EMBRYONIC_CELL																													
CELLMARKERCUR_N_EMBRYONIC_STEM_CELL																													
CELLMARKERCUR_N_ENDOCARDIAL_CELL																													
CELLMARKERCUR_N_ENDOCRINE_PROGENITOR_CELL																													
CELLMARKERCUR_N_ENDODERM_PROGENITOR_CELL																													
CELLMARKERCUR_N_ENDODERMAL_CELL																													
CELLMARKERCUR_N_ENDOMETRIAL_STROMAL_CELL																													
CELLMARKERCUR_N_ENDOMETRIAL_STROMAL_STEM_…																													
CELLMARKERCUR_N_ENDOTHELIAL_PRECURSOR_CELL																													
CELLMARKERCUR_N_ENSHEATHING_GLIAL_CELL																													
CELLMARKERCUR_N_ENTERIC_NEURON																													
CELLMARKERCUR_N_EOSINOPHIL	IL9R	LAIR1	SPN																										
CELLMARKERCUR_N_EPENDYMAL_CELL																													
CELLMARKERCUR_N_EPIBLAST_CELL																													
CELLMARKERCUR_N_EPICARDIAL_CELL																													
CELLMARKERCUR_N_EPICARDIUM_DERIVED_CELL																													
CELLMARKERCUR_N_EPIDERMAL_CELL																													
CELLMARKERCUR_N_EPIDERMAL_STEM_CELL																													
CELLMARKERCUR_N_EPITHELIAL_CELL	PCDH17	TSPAN7	APLNR	FLRT2	ITGA8	GJA4	ADGRG6	DLL4	PLVAP	RAMP2	UNC5B	GYPC	FUT4	CD24	ANPEP	IL6R	IL6ST	ITGAE	THBD	VCAM1	FLOT2	CD34	MUC1	PPL	JUP	TFF3	KLF6	MITF	RPE65
CELLMARKERCUR_N_EPITHELIAL_DUCTAL_CELL																													
CELLMARKERCUR_N_EPITHELIAL_MESENCHYMAL_CE…																													
CELLMARKERCUR_N_EPITHELIOID_MACROPHAGE																													
CELLMARKERCUR_N_EPSILON_CELL																													
CELLMARKERCUR_N_ERYTHROBLAST																													
CELLMARKERCUR_N_ERYTHROID_CELL																													
CELLMARKERCUR_N_ERYTHROID_PROGENITOR_CELL																													
CELLMARKERCUR_N_ESOPHAGEAL_CELL																													
CELLMARKERCUR_N_ESOPHAGEAL_PROGENITOR_CELL																													
CELLMARKERCUR_N_EXCITATORY_NEURON																													
CELLMARKERCUR_N_EXHAUSTED_DOUBLE_POSITIVE…																													
CELLMARKERCUR_N_EXTRAVILLOUS_TROPHOBLAST																													
CELLMARKERCUR_N_F_PERICYTE																													
CELLMARKERCUR_N_FETAL_MYOBLAST																													
CELLMARKERCUR_N_FIBROBLAST_LIKE_CELL																													
CELLMARKERCUR_N_FIBROCHONDROCYTE																													
CELLMARKERCUR_N_FIBROCHONDROCYTE_PROGENIT…																													
CELLMARKERCUR_N_FOAM_CELL																													
CELLMARKERCUR_N_FOLLICULAR_B_CELL																													
CELLMARKERCUR_N_FOLLICULAR_CELL																													
CELLMARKERCUR_N_FOLLICULAR_DENDRITIC_CELL																													
CELLMARKERCUR_N_FOREBRAIN_CELL																													
CELLMARKERCUR_N_GABAERGIC_NEURON																													
CELLMARKERCUR_N_GANGLION_CELL																													
CELLMARKERCUR_N_GASTRIC_NEUROENDOCRINE_CE…																													
CELLMARKERCUR_N_GASTRIC_STEM_CELL																													
CELLMARKERCUR_N_GERM_CELL																													
CELLMARKERCUR_N_GERMINAL_CENTER_B_CELL																													
CELLMARKERCUR_N_GERMINAL_CENTER_CELL																													
CELLMARKERCUR_N_GERMINAL_CENTRE_B_CELL																													
CELLMARKERCUR_N_GLIAL_CELL																													
CELLMARKERCUR_N_GLIOMA_STEM_CELL																													
CELLMARKERCUR_N_GLOBOSE_BASAL_CELL																													
CELLMARKERCUR_N_GLOMERULAR_PARIETAL_EPITH…																													
CELLMARKERCUR_N_GLUTAMATERGIC_NEURON																													
CELLMARKERCUR_N_GOBLET_CELL																													
CELLMARKERCUR_N_GONADOTROPE																													
CELLMARKERCUR_N_GONADOTROPH_CELL																													
CELLMARKERCUR_N_GRANULOCYTE																													
CELLMARKERCUR_N_GRANULOCYTE_MONOCYTE_PROG…																													
CELLMARKERCUR_N_GRANULOCYTIC_UNK																													
CELLMARKERCUR_N_GRANULOSA_CELL																													
CELLMARKERCUR_N_HAEMATOPOIETIC_CELL																													
CELLMARKERCUR_N_HAIR_FOLLICLE_CELL																													
CELLMARKERCUR_N_HEMANGIOBLAST																													
CELLMARKERCUR_N_HEMATOPOIETIC_CELL																													
CELLMARKERCUR_N_HEMATOPOIETIC_PRECURSOR_C…																													
CELLMARKERCUR_N_HEMATOPOIETIC_PROGENITOR_…																													
CELLMARKERCUR_N_HEPATIC_CELL																													
CELLMARKERCUR_N_HEPATIC_PROGENITOR_CELL																													

CELLMARKERCAN_ACINAR_CELLS																													
CELLMARKERCUR_N_HEPATIC_STELLATE_CELL																													
CELLMARKERCUR_N_HEPATOBLAST																													
CELLMARKERCUR_N_HEPATOCYTE																													
CELLMARKERCUR_N_HISTIOCYTE																													
CELLMARKERCUR_N_HMSC_TREATED_B_CELL																													
CELLMARKERCUR_N_HOFBAUER_CELL																													
CELLMARKERCUR_N_HOMEOSTATIC_CHONDROCYTE	TXNIP	GDF15																											
CELLMARKERCUR_N_HOMEOSTATIC_MICROGLIAL_CE…																													
CELLMARKERCUR_N_HORIZONTAL_BASAL_CELL																													
CELLMARKERCUR_N_HORIZONTAL_CELL																													
CELLMARKERCUR_N_HYPOBLAST_CELL																													
CELLMARKERCUR_N_IDIOPATHIC_PULMONARY_FIBR…																													
CELLMARKERCUR_N_IGA_MEMORY_B_CELL																													
CELLMARKERCUR_N_IGG_MEMORY_B_CELL																													
CELLMARKERCUR_N_IGM_MEMORY_B_CELL																													
CELLMARKERCUR_N_IGM_ONLY_B_CELL																													
CELLMARKERCUR_N_IL7R_T_HELPER_CELL																													
CELLMARKERCUR_N_IMMATURE_ERYTHROID_CELL																													
CELLMARKERCUR_N_IMMATURE_MYELOID_CELL																													
CELLMARKERCUR_N_IMMATURE_NEUTROPHIL																													
CELLMARKERCUR_N_IMMATURE_TRANSITIONAL_B_C…																													
CELLMARKERCUR_N_IMMUNE_CELL	CD96	SIRPG	LAIR1	CCR5	SLAMF6	FCGR2A	CD6	CD37	MYO1G																				
CELLMARKERCUR_N_INDUCED_PLURIPOTENT_STEM_…																													
CELLMARKERCUR_N_INFILTRATING_MACROPHAGE																													
CELLMARKERCUR_N_INFLAMMATORY_CANCER_ASSOC…																													
CELLMARKERCUR_N_INFLAMMATORY_DENDRITIC_CE…																													
CELLMARKERCUR_N_INFLAMMATORY_MONOCYTE																													
CELLMARKERCUR_N_INHIBITORY_NEURON																													
CELLMARKERCUR_N_INNATE_LYMPHOID_CELL																													
CELLMARKERCUR_N_INNER_CELL_MASS_CELL																													
CELLMARKERCUR_N_INNER_RADIAL_GLIAL_CELL																													
CELLMARKERCUR_N_INTER_MEDIATE_PROGENITOR_…																													
CELLMARKERCUR_N_INTERCALATED_CELL																													
CELLMARKERCUR_N_INTERFERON_RESPONSIVE_SEC…																													
CELLMARKERCUR_N_INTERMEDIATE_MONOCYTE																													
CELLMARKERCUR_N_INTERMEDIATE_MONOCYTE_CELL																													
CELLMARKERCUR_N_INTERSTITIAL_MACROPHAGE																													
CELLMARKERCUR_N_INTERSTITIAL_PROGENITOR_C…																													
CELLMARKERCUR_N_INTESTINAL_EPITHELIAL_STE…																													
CELLMARKERCUR_N_INTESTINAL_GASTRIC_CANCER…																													
CELLMARKERCUR_N_INTESTINAL_METAPLASIA_CELL																													
CELLMARKERCUR_N_INTRAEPITHELIAL_LYMPHOCYT…																													
CELLMARKERCUR_N_IONOCYTE																													
CELLMARKERCUR_N_IRIS_PUTTATIVE_STEM_CELL																													
CELLMARKERCUR_N_KUPFFER_CELL																													
CELLMARKERCUR_N_LACTOTROPH_CELL																													
CELLMARKERCUR_N_LANGERHANS_CELL																													
CELLMARKERCUR_N_LEUKOCYTE																													
CELLMARKERCUR_N_LIMBAL_EPITHELIAL_STEM_CE…																													
CELLMARKERCUR_N_LIMBAL_STEM_CELL																													
CELLMARKERCUR_N_LIPOFIBROBLAST																													
CELLMARKERCUR_N_LIVER_PROGENITOR_CELL																													
CELLMARKERCUR_N_LIVER_STEM_CELL																													
CELLMARKERCUR_N_LMMLEYDIG_CELL																													
CELLMARKERCUR_N_LONOCYTE																													
CELLMARKERCUR_N_LOOP_OF_HENLE_CELL																													
CELLMARKERCUR_N_LOOP_PROGENITOR_CELL																													
CELLMARKERCUR_N_LOW_DENSITY_NEUTROPHIL																													
CELLMARKERCUR_N_LUMINAL_PROGENITOR_CELL																													
CELLMARKERCUR_N_LUNG_CELL																													
CELLMARKERCUR_N_LYMPH_NODE_CELL																													
CELLMARKERCUR_N_LYMPHATIC_CELL																													
CELLMARKERCUR_N_LYMPHATIC_ENDOTHELIAL_CELL																													
CELLMARKERCUR_N_LYMPHOBLASTOID_CELL																													
CELLMARKERCUR_N_LYMPHOCYTE																													
CELLMARKERCUR_N_LYMPHOID_CELL																													
CELLMARKERCUR_N_LYMPHOID_PROGENITOR_CELL																													
CELLMARKERCUR_N_LYMPHOID_STRUCTURE_CELL																													
CELLMARKERCUR_N_M_PERICYTE																													
CELLMARKERCUR_N_M1_MACROPHAGE																													
CELLMARKERCUR_N_M1_MICROGLIAL_CELL																													
CELLMARKERCUR_N_M2_MACROPHAGE	CX3CR1	CCR2																											
CELLMARKERCUR_N_MACROGLIAL_CELL																													
CELLMARKERCUR_N_MALIGNANT_CELL																													
CELLMARKERCUR_N_MALIGNANT_PLASMA_CELL																													
CELLMARKERCUR_N_MARGINAL_ASSOCIATED_MACRO…																													
CELLMARKERCUR_N_MAST_CELL	FUT4	IL2	IL3RA	IL5RA	IL6R	PTPRC	SPN																						
CELLMARKERCUR_N_MATURE_ASTROCYTE																													
CELLMARKERCUR_N_MATURE_B_CELL																													
CELLMARKERCUR_N_MATURE_DENDRITIC_CELL																													
CELLMARKERCUR_N_MATURE_ENDOTHELIAL_CELL																													
CELLMARKERCUR_N_MATURE_LUMINAL_CELL																													
CELLMARKERCUR_N_MATURE_NEURON																													
CELLMARKERCUR_N_MATURE_NEURONAL																													
CELLMARKERCUR_N_MATURE_OLIGODENDROCYTE																													
CELLMARKERCUR_N_MATURE_OSTEOBLAST																													
CELLMARKERCUR_N_MATURE_PODOCYTE																													
CELLMARKERCUR_N_MEDIAL_AND_DISTAL_SEGMENT…																													
CELLMARKERCUR_N_MEDIAL_GANGLIONIC_EMINENC…																													
CELLMARKERCUR_N_MEGAKARYOCYTE																													
CELLMARKERCUR_N_MEGAKARYOCYTE_ERYTHROID_C…																													
CELLMARKERCUR_N_MEGAKARYOCYTE_ERYTHROID_P…																													
CELLMARKERCUR_N_MEGAKARYOCYTE_PROGENITOR_…																													
CELLMARKERCUR_N_MELANOMA_CELL																													
CELLMARKERCUR_N_MEMORY_B_CELL																													
CELLMARKERCUR_N_MEMORY_B_CELL_PRECURSOR																													
CELLMARKERCUR_N_MEMORY_CD27_B_CELL																													
CELLMARKERCUR_N_MEMORY_CD4_T_CELL																													
CELLMARKERCUR_N_MEMORY_DOUBLE_POSITIVE_T_…																													
CELLMARKERCUR_N_MEMORY_REGULATORY_T_CELL																													
CELLMARKERCUR_N_MEMORY_T_CELL																													
CELLMARKERCUR_N_MERKEL_CELL																													
CELLMARKERCUR_N_MESENCHYMAL_CELL	LGR6	CLDN7	TNC	ITGA5	WNT5A	KDELR3	GPC6	SERPI…	INHBA	CDH11	PRRX1	YAP1	COL6A3	COL12…	CYP11…	IGF1	LAMA2												
CELLMARKERCUR_N_MESENCHYMAL_EPITHELIAL_TR…																													

Table S5: Cell state marker gene list

CELLMARKERCAN_ACINAR_CELLS																													
CELLMARKERCUR_N_MESENCHYMAL_GLIOBLASTOMA_…																													
CELLMARKERCUR_N_MESENCHYMAL_STEM_CELL	NRP1	ITGB3	ITGB4	CD58	DAZL	STRA8	DDX4	ZBTB16	ZEB1	PDGFB	ENG	FN1	B4GAL…	NOTCH3	RGS5	GUCY1…													
CELLMARKERCUR_N_MESENCHYMAL_STROMAL_CELL																													
CELLMARKERCUR_N_MESENDODERMAL_CELL																													
CELLMARKERCUR_N_MESODERMAL_EMBRYONIC_STEM…																													
CELLMARKERCUR_N_MESODERMAL_PROGENITOR_CELL																													
CELLMARKERCUR_N_METAPLASTIC_STEM_CELL																													
CELLMARKERCUR_N_MEYLOID_CELL																													
CELLMARKERCUR_N_MICROGLIAL_CELL	C1QC	ADORA3	BIN1	SALL1	GPR34	SIGLE…	OLFML3	CCL3	SPI1	PDGFRA	IL1B	NLRP1	SOX2	CTSB	IFNGR1	TSPO	TMEM19	LYVE1	PILRA	MS4A6A	VSIG4	ADGRG1	MOBP	VCAN	IRF8	GUSB	TPD52	TMEM1…	GLB1
CELLMARKERCUR_N_MICROVILLAR_CELL																													
CELLMARKERCUR_N_MIDBRAIN_DOPAMINERGIC_NEU…																													
CELLMARKERCUR_N_MONO_MACROPHAGE																													
CELLMARKERCUR_N_MONOCYTE_DERIVED_CELL																													
CELLMARKERCUR_N_MONOCYTE_DERIVED_MYELOID_…																													
CELLMARKERCUR_N_MONOCYTE_LINEAGE																													
CELLMARKERCUR_N_MONOCYTE_PRECURSOR																													
CELLMARKERCUR_N_MONOCYTE_PROGENITOR_CELL																													
CELLMARKERCUR_N_MUELLER_GLIA_CELL																													
CELLMARKERCUR_N_MULTIPOTENT_MESENCHYMAL_S…																													
CELLMARKERCUR_N_MULTIPOTENT_RETINAL_PROGE…																													
CELLMARKERCUR_N_MULTIPOTENT_STEM_CELL																													
CELLMARKERCUR_N_MULTIPOTENT_STROMAL_CELL																													
CELLMARKERCUR_N_MUSCLE_CELL																													
CELLMARKERCUR_N_MUSCLE_DERIVED_CELL																													
CELLMARKERCUR_N_MUSCLE_SATELLITE_CELL																													
CELLMARKERCUR_N_MYELOBLAST																													
CELLMARKERCUR_N_MYELOID_DENDRITIC_CELL																													
CELLMARKERCUR_N_MYELOID_DENDRITIC_CELL_1																													
CELLMARKERCUR_N_MYELOID_DERIVED_CELL																													
CELLMARKERCUR_N_MYELOID_DERIVED_SUPPRESSO…																													
CELLMARKERCUR_N_MYELOID_PROGENITOR_CELL																													
CELLMARKERCUR_N_MYO_EPITHELIAL_CELL																													
CELLMARKERCUR_N_MYOBLAST																													
CELLMARKERCUR_N_MYOCYTE																													
CELLMARKERCUR_N_MYOFIBROBLAST																													
CELLMARKERCUR_N_MYOGENIC_ENDOTHELIAL_CELL																													
CELLMARKERCUR_N_MYOGENIC_PROGENITOR_CELL																													
CELLMARKERCUR_N_MYOID_CELL																													
CELLMARKERCUR_N_NAIVE_B_CELL																													
CELLMARKERCUR_N_NAIVE_CD8_T_CELL																													
CELLMARKERCUR_N_NAIVE_DOUBLE_POSITIVE_T_C…																													
CELLMARKERCUR_N_NAIVE_PROGENITOR_CELL																													
CELLMARKERCUR_N_NAIVE_T_CELL																													
CELLMARKERCUR_N_NASCENT_NEPHRON_CELL																													
CELLMARKERCUR_N_NATURAL_KILLER_CD56_BRIGH…																													
CELLMARKERCUR_N_NATURAL_KILLER_CD56_DIM_C…																													
CELLMARKERCUR_N_NEGATIVE_MESENCHYMAL_STEM…																													
CELLMARKERCUR_N_NEURAL_CELL																													
CELLMARKERCUR_N_NEURAL_CREST_STEM_CELL																													
CELLMARKERCUR_N_NEURAL_CREST_STEMLIKE_CELL																													
CELLMARKERCUR_N_NEURAL_PRECURSOR_CELL																													
CELLMARKERCUR_N_NEURAL_STEM_CELL																													
CELLMARKERCUR_N_NEUROECTODERM_CELL																													
CELLMARKERCUR_N_NEURONAL_INTERMEDIATE_PRO…																													
CELLMARKERCUR_N_NEURONAL_PROGENITOR_CELL																													
CELLMARKERCUR_N_NEUTROPHIL_PROGENITOR_CELL																													
CELLMARKERCUR_N_NON_NEURON																													
CELLMARKERCUR_N_NON_PT_TUBULAR_CELL																													
CELLMARKERCUR_N_NON_RECIRCULATING_TISSUE_…																													
CELLMARKERCUR_N_NONMYELINATING_SCHWANN_CE…																													
CELLMARKERCUR_N_NOTOCHORD_CELL																													
CELLMARKERCUR_N_NUCLEATED_CELL																													
CELLMARKERCUR_N_NUCLEUS_PULPOSUS_PROGENIT…																													
CELLMARKERCUR_N_OLIGODENDROCYTE																													
CELLMARKERCUR_N_OSTEOBLAST																													
CELLMARKERCUR_N_OUTER_RADIAL_GLIA																													
CELLMARKERCUR_N_OVALBUMIN_SPECIFIC_REGULA…																													
CELLMARKERCUR_N_OVARIAN_CELL																													
CELLMARKERCUR_N_OVARIAN_STROMAL_CELL																													
CELLMARKERCUR_N_P53_LIKE_CELL																													
CELLMARKERCUR_N_PAN_B_CELL																													
CELLMARKERCUR_N_PAN_CANCER_ASSOCIATED_FIB…																													
CELLMARKERCUR_N_PAN_MACROPHAGE																													
CELLMARKERCUR_N_PAN_MONOCYTE																													
CELLMARKERCUR_N_PAN_NEURONAL_CELL																													
CELLMARKERCUR_N_PAN_TROPHECTODERM_CELL																													
CELLMARKERCUR_N_PANCREATIC_ENDODERM_CELL																													
CELLMARKERCUR_N_PANCREATIC_POLYPEPTIDE_CE…																													
CELLMARKERCUR_N_PANCREATIC_PROGENITOR_CELL																													
CELLMARKERCUR_N_PANCREATIC_STEM_CELL																													
CELLMARKERCUR_N_PANETH_CELL																													
CELLMARKERCUR_N_PANNEURON																													
CELLMARKERCUR_N_PANVASCULAR_CELL																													
CELLMARKERCUR_N_PAPILLARY_FIBROBLAST																													
CELLMARKERCUR_N_PARIETAL_CELL																													
CELLMARKERCUR_N_PARVALBUMIN_INTERNEURON																													
CELLMARKERCUR_N_PATELET																													
CELLMARKERCUR_N_PATHOGENIC_T_PERIPHERAL_H…																													
CELLMARKERCUR_N_PELVIC_EPITHELIAL_CELL																													
CELLMARKERCUR_N_PERICYTE																													
CELLMARKERCUR_N_PERIODONTAL_LIGAMENT_STEM…																													
CELLMARKERCUR_N_PERIOSTEUM_DERIVED_PROGEN…																													
CELLMARKERCUR_N_PERITUBULAR_MYOID_CELL																													
CELLMARKERCUR_N_PERIVASCULAR_LIKE_CELL																													
CELLMARKERCUR_N_PHAGOCYTE																													
CELLMARKERCUR_N_PHOTORECEPTOR_CELL																													
CELLMARKERCUR_N_PIT_CELL																													
CELLMARKERCUR_N_PIT_MUCOUS_CELL																													
CELLMARKERCUR_N_PITUITARY_STEM_CELL																													
CELLMARKERCUR_N_PLACENTAL_TISSUE_CELL																													
CELLMARKERCUR_N_PLACODE_CELL																													
CELLMARKERCUR_N_PLASMABLAST_B_CELL																													
CELLMARKERCUR_N_PLATELET																													
CELLMARKERCUR_N_PLURIPOTENT_STEM_CELL																													

CELLMARKERCAN_ACINAR_CELLS																													
CELLMARKERCUR_N_PODOCYTE_PRECURSOR																													
CELLMARKERCUR_N_PORTAL_FIBROBLAST																													
CELLMARKERCUR_N_POSTERIOR_CELL																													
CELLMARKERCUR_N_PRE_B_CELL																													
CELLMARKERCUR_N_PRE_CONVENTIONAL_DENDRITI…																													
CELLMARKERCUR_N_PRE_DENDRITIC_CELL																													
CELLMARKERCUR_N_PRECURSOR_CELL																													
CELLMARKERCUR_N_PREHYPERTROPHIC_CHONDROCY…																													
CELLMARKERCUR_N_PRESOMITE_CELL																													
CELLMARKERCUR_N_PRETUBULAR_AGGREGATE_CELL																													
CELLMARKERCUR_N_PRIMITIVE_ENDODERM_CELL																													
CELLMARKERCUR_N_PRINCIPAL_CELL																													
CELLMARKERCUR_N_PRO_B_CELL																													
CELLMARKERCUR_N_PRO_INFLAMMATORY_MACROPHA…																													
CELLMARKERCUR_N_PRO_NEUTROPHIL																													
CELLMARKERCUR_N_PROLIFERATING_CELL																													
CELLMARKERCUR_N_PROLIFERATIVE_CHONDROCYTE																													
CELLMARKERCUR_N_PROLIFERATIVE_DUCTAL_CELL																													
CELLMARKERCUR_N_PROLIFERATIVE_NEURAL_PROG…																													
CELLMARKERCUR_N_PRONEURAL_GLIOBLASTOMA_CE…																													
CELLMARKERCUR_N_PROSPERMATOGONIA																													
CELLMARKERCUR_N_PROSTATE_CELL																													
CELLMARKERCUR_N_PROXIMAL_CELL																													
CELLMARKERCUR_N_PROXIMAL_SEGMENT_CELL																													
CELLMARKERCUR_N_PROXIMAL_STEM_CELL																													
CELLMARKERCUR_N_PROXIMAL_TUBULE_CELL																													
CELLMARKERCUR_N_PULMONARY_NEUROENDOCRINE_…																													
CELLMARKERCUR_N_PYRAMIDAL_CELL																													
CELLMARKERCUR_N_QUIESCENT_T_STEM_CELL																													
CELLMARKERCUR_N_REGULATORY_B_CELL																													
CELLMARKERCUR_N_REGULATORY_INNATE_LYMPHOI…																													
CELLMARKERCUR_N_RENAL_TUBULAR_CELL																													
CELLMARKERCUR_N_RESIDENT_MEMORY_T_CELL																													
CELLMARKERCUR_N_RESPIRATORY_HORIZONTAL_BA…																													
CELLMARKERCUR_N_RESPIRATORY_SECRETORY_CELL																													
CELLMARKERCUR_N_RESPONDER_T_CELL																													
CELLMARKERCUR_N_RESPONDING_CONVENTIONAL_T…																													
CELLMARKERCUR_N_RESTING_MEMORY_B_CELL																													
CELLMARKERCUR_N_RESTING_NAIVE_B_CELL																													
CELLMARKERCUR_N_RESTING_REGULATORY_T_CELL																													
CELLMARKERCUR_N_RETICULAR_FIBROBLAST																													
CELLMARKERCUR_N_RETINA_CELL																													
CELLMARKERCUR_N_ROD_CELL																													
CELLMARKERCUR_N_SATELLITE_CELL																													
CELLMARKERCUR_N_SEPTUM_TRANSVERSUM_MESENC…																													
CELLMARKERCUR_N_SEROUS_CELL																													
CELLMARKERCUR_N_SIDE_POPULATION_CELL																													
CELLMARKERCUR_N_SKELETAL_MUSCLE_CELL																													
CELLMARKERCUR_N_SKELETAL_MUSCLE_STEM_CELL																													
CELLMARKERCUR_N_SMOOTH_MUSCLE_CELL																													
CELLMARKERCUR_N_SMOOTH_MUSCLE_LIKE_CELL																													
CELLMARKERCUR_N_SOMATIC_CELL																													
CELLMARKERCUR_N_SOMATIC_STEM_CELL																													
CELLMARKERCUR_N_SOMATOSTATIN_INTERNEURON																													
CELLMARKERCUR_N_SPECIALIST_ANTIGEN_PRESEN…																													
CELLMARKERCUR_N_SPERMATOGENIC_CELL																													
CELLMARKERCUR_N_SPERMATOGONIAL_CELL																													
CELLMARKERCUR_N_SPERMATOGONIAL_STEM_CELL																													
CELLMARKERCUR_N_SQUAMOUS_DIFFERENTIATION_…																													
CELLMARKERCUR_N_STELLATE_CELL																													
CELLMARKERCUR_N_STEM_CELL_DERIVED_ALPHA_C…																													
CELLMARKERCUR_N_STEM_CELL_DERIVED_BETA_CE…																													
CELLMARKERCUR_N_STEM_CELL_DERIVED_ENDOTHE…																													
CELLMARKERCUR_N_STEM_LIKE_CD8_T_CELL																													
CELLMARKERCUR_N_STROMAL_CELL	AIRE	CLDN4	NEUROD1	LY75	MMP1	MMP2	MMP9	TIMP1	TIMP2	TLR1	TLR2	TLR3	TLR4	BST1	ICAM1	ICAM2	ICAM3	ITGAV	ITGB3	KIT	VCAM1	PROM1							
CELLMARKERCUR_N_SUBPALLIUM_CELL																													
CELLMARKERCUR_N_SUBPLATE_CELL																													
CELLMARKERCUR_N_SUPPRESSIVE_MONOCYTE																													
CELLMARKERCUR_N_SUPPRESSOR_T_CELL																													
CELLMARKERCUR_N_SUPRABASAL_CELL																													
CELLMARKERCUR_N_SURFACE_ECTODERM_CELL																													
CELLMARKERCUR_N_SUSTENTACULAR_CELL																													
CELLMARKERCUR_N_SYMPATHOADRENAL_CELL																													
CELLMARKERCUR_N_SYNCYTIOTROPHOBLAST																													
CELLMARKERCUR_N_SYNTHETIC_SMOOTH_MUSCLE_C…																													
CELLMARKERCUR_N_T_CELL_LARGE_GRANULAR_LYM…																													
CELLMARKERCUR_N_T_CELL_LINEAGE																													
CELLMARKERCUR_N_T_HELPER_CELL																													
CELLMARKERCUR_N_T_MEMORY_STEM_CELL																													
CELLMARKERCUR_N_T2_MZP_REGULATORY_B_CELL																													
CELLMARKERCUR_N_TENDON_CELL																													
CELLMARKERCUR_N_TERMINAL_DIFFERENTIATED_B…																													
CELLMARKERCUR_N_THALAMIC_CELL																													
CELLMARKERCUR_N_THECA_INTERNA_CELL																													
CELLMARKERCUR_N_THYMIC_EPITHELIAL_CELL																													
CELLMARKERCUR_N_TISSUE_LIKE_MEMORY_B_CELL																													
CELLMARKERCUR_N_TISSUE_RESIDENT_CELL																													
CELLMARKERCUR_N_TISSUE_RESIDENT_EFFECTOR_…																													
CELLMARKERCUR_N_TISSUE_RESIDENT_MACROPHAGE																													
CELLMARKERCUR_N_TISSUE_RESIDENT_MEMORY_LI…																													
CELLMARKERCUR_N_TOLDC_INDUCED_REGULATORY_…																													
CELLMARKERCUR_N_TONSIL_CELL																													
CELLMARKERCUR_N_TRABECULAR_MESHWORK_CELL																													
CELLMARKERCUR_N_TRANSITIONAL_B_CELL																													
CELLMARKERCUR_N_TRANSITIONAL_DENDRITIC_CE…																													
CELLMARKERCUR_N_TRUNCATED_RADIAL_GLIAL_CE…																													
CELLMARKERCUR_N_TUBULAR_CELL																													
CELLMARKERCUR_N_TUMOR_ASSOCIATED_MICROGLI…																													
CELLMARKERCUR_N_TUMOR_ENDOTHELIAL_CELL																													
CELLMARKERCUR_N_TUMOR_EPITHELIAL_CELL																													
CELLMARKERCUR_N_TUMOR_INFILTRATING_REGULA…																													
CELLMARKERCUR_N_TUMOR_INFILTRATING_T_CELL																													
CELLMARKERCUR_N_TUMOR_INITIATING_CELL																													
CELLMARKERCUR_N_TUMOR_REGULATORY_T_CELL																													

CELLMARKERCAN_ACINAR_CELLS																													
CELLMARKERCUR_N_UMBRELLA_CELL																													
CELLMARKERCUR_N_UNRESTRICTED_SOMATIC_STEM…																													
CELLMARKERCUR_N_UNSWITCHED_PLASMABLAST																													
CELLMARKERCUR_N_URETERIC_BUD_CELL																													
CELLMARKERCUR_N_UROTHELIAL_CELL																													
CELLMARKERCUR_N_UROTHELIUM_CELL																													
CELLMARKERCUR_N_VASCULATURE_CELL																													
CELLMARKERCUR_N_VEIN_CELL																													
CELLMARKERCUR_N_VENOUS_CELL																													
CELLMARKERCUR_N_VENTRAL_LATERAL_GANGLIONI…																													
CELLMARKERCUR_N_VENTRAL_MEDIAL_GANGLIONIC…																													
CELLMARKERCUR_N_VENTRICULAR_CELL																													
CELLMARKERCUR_N_YOUNG_NEURON																													
CELLMARKERCUR_N_ZONA_FASCICULATA_CELL																													
CELLMARKERCUR_N_ZONA_GLOMERULOSA_CELL																													
CELLMARKERCUR_N_ZONA_RETICULARIS_CELL																													
CELLMARKERCUR_N_ZYGOTE																													
CELLMARKERCURNACTIVATEDEFFECTORMEMORYCD4T…																													
CELLMARKERCURNACTIVATEDTISSUERESIDENTMEMO…																													
CELLMARKERCURNACTIVATEDTISSUERESIDENTMEMO…																													
CELLMARKERCURNALPHACELLACELL																													
CELLMARKERCURNALVEOLARTYPEIIATIICELL																													
CELLMARKERCURNATYPICALBCELLABC																													
CELLMARKERCURNAXLCD11CPLASMACYTOIDDENDRIT…																													
CELLMARKERCURNAXLCD123PLASMACYTOIDDENDRIT…																													
CELLMARKERCURNBASALFOREBRAINCHOLINERGICNE…																													
CELLMARKERCURNBRUSHCELLTUFTCELL																													
CELLMARKERCURNCANCERASSOCIATEDFIBROBLASTC…																													
CELLMARKERCURNCAPSULARFIBROBLASTFB2																													
CELLMARKERCURNCCR7DENDRITICCELL																													
CELLMARKERCURNCD106MESENCHYMALSTEMCELL																													
CELLMARKERCURNCD14MACROPHAGE																													
CELLMARKERCURNCD16DENDRITICCELL																													
CELLMARKERCURNCD25LAG3TCELL																													
CELLMARKERCURNCD34GRANULOCYTICPROGENITORC…																													
CELLMARKERCURNCD34HEMATOPOIETICSTEMCELL																													
CELLMARKERCURNCD34PREBCELL																													
CELLMARKERCURNCD34PROGENITORCELL																													
CELLMARKERCURNCD34THYMOCYTE																													
CELLMARKERCURNCD40LGTHELPERCELL																													
CELLMARKERCURNCD4CD25REGULATORYTCELL																													
CELLMARKERCURNCD4CENTRALMEMORYLIKETTCMLIK…																													
CELLMARKERCURNCD4CYTOTOXICT1CELL																													
CELLMARKERCURNCD4CYTOTOXICT2CELL																													
CELLMARKERCURNCD4THELPERCELL																													
CELLMARKERCURNCHRONICALLYACTIVATEDCD4TCELL																													
CELLMARKERCURNCHRONICALLYACTIVATEDCD8TCELL																													
CELLMARKERCURNCLASSSWITCHEDMEMORYBIGGU03B…																													
CELLMARKERCURNCLEC10ADENDRITICCELL																													
CELLMARKERCURNCLEC9ADENDRITICCELL																													
CELLMARKERCURNCONVENTIONALDENDRITICCELL2B…																													
CELLMARKERCURNCONVENTIONALDENDRITICCELL2B…																													
CELLMARKERCURNCORTICALSOMATOSTATINSSTINTE…																													
CELLMARKERCURNCYTOTOXICCD4T2CELL																													
CELLMARKERCURNCYTOTOXICCD4TCELL																													
CELLMARKERCURND1MSNMEDIUMSPINYNEURON																													
CELLMARKERCURND2MSNMEDIUMSPINYNEURON																													
CELLMARKERCURNDISTALTUBULELOOPOFHENLECELL																													
CELLMARKERCURNEARLYCD34MEGAKARYOCYTEERYTH…																													
CELLMARKERCURNEARLYTCELLPROGENITORETP																													
CELLMARKERCURNEFFECTORMEMORYCD4TCELL																													
CELLMARKERCURNEFFECTORREGULATORYTETREGCELL																													
CELLMARKERCURNEFFECTORTTEFFCELL																													
CELLMARKERCURNEXHAUSTEDCD4TCELL																													
CELLMARKERCURNEXHAUSTEDCD8TCELL																													
CELLMARKERCURNEXHAUSTEDCD8TPROGENITORCELL																													
CELLMARKERCURNEXHAUSTEDTTEXCELL																													
CELLMARKERCURNEXTRATELENCEPHALICETCELL																													
CELLMARKERCURNFINALLYHIGHLYEFFECTORTEMRAM…																													
CELLMARKERCURNFOLLICULARREGULATORYTTFRCELL																													
CELLMARKERCURNFOXP3NATURALREGULATORYTTREG…																													
CELLMARKERCURNGAMMADELTAU03B3DTCELL																													
CELLMARKERCURNGERMINALCENTREGCLIKECELL																													
CELLMARKERCURNGLOMERULARENDOTHELIALCELLECG																													
CELLMARKERCURNGRANULOCYTICMYELOIDDERIVEDS…																													
CELLMARKERCURNGRBREGULATORYBCELL																													
CELLMARKERCURNGZMKTCELL																													
CELLMARKERCURNHIGHLYDIFFERENTIATEDEFFECTO…																													
CELLMARKERCURNINDUCEDREGULATORYTTREGCELL																													
CELLMARKERCURNINFLAMMATORYCANCERASSOCIATE…																													
CELLMARKERCURNINNATELYMPHOIDCELLILCS																													
CELLMARKERCURNINTRATHYMICFIBROBLASTFB1																													
CELLMARKERCURNITOCELLHEPATICSTELLATECELL																													
CELLMARKERCURNLYMPHANGIOLEIOMYOMATOSISCEL…																													
CELLMARKERCURNMARGINALZONEMZLIKEBCELL																													
CELLMARKERCURNMEMORYSTEMCELLTSCM																													
CELLMARKERCURNMICROGLIADERIVEDTUMORASSOCI…																													
CELLMARKERCURNMKERYTHCELL																													
CELLMARKERCURNMYOFIBROBLASTICCANCERASSOCI…																													
CELLMARKERCURNNAIVEREGULATORYTTREGCELL																													
CELLMARKERCURNNATURALREGULATORYTTREGCELL																													
CELLMARKERCURNPERIPHERALBLOODMONONUCLEARC…																													
CELLMARKERCURNPITUITARYSTEMCELLPSC																													
CELLMARKERCURNPLASMACYTOIDDENDRITICCELLPDC																													
CELLMARKERCURNPOLYMORPHONUCLEARMYELOIDDER…																													
CELLMARKERCURNPOLYMORPHONUCLEARNEUTROPHIL…																													
CELLMARKERCURNPOSTERIORPRESOMITICMESODERM…																													
CELLMARKERCURNPROLIFERATIVECD4TCELL																													
CELLMARKERCURNPROLIFERATIVECD8TCELL																													
CELLMARKERCURNREDBLOODCELLERYTHROCYTE																													
CELLMARKERCURNREGULATORYBBREGCELL																													
CELLMARKERCURNREGULATORYCD4TCELL																													
CELLMARKERCURNREGULATORYCD8TCELL																													
CELLMARKERCURNREGULATORYTTREGCELL	TRAF3	EZH2	FCRL3	LGMN	IKZF4	GATA3	TGFB3	MIR15…	PTGIR	GNG8	PLN	NRP1	ITGAE	REL	CXCR6														

CELLMARKERCAN_ACINAR_CELLS																													
CELLMARKERCURNREGULATORYTTREGCELL																													
CELLMARKERCURNRENALVESICLECOMMASHAPEDBODY…																													
CELLMARKERCURNRESIDENTMEMORYCD8TCELL																													
CELLMARKERCURNREVERTANTMEMORYCD8TTEMRACELL																													
CELLMARKERCURNSCARASSOCIATEDMACROPHAGESAM																													
CELLMARKERCURNSENESCENTCD8TCELL																													
CELLMARKERCURNSTEMPROGENITORCELL																													
CELLMARKERCURNSWITCHEDMEMORYBCELLSWMB																													
CELLMARKERCURNT1TRANSITIONALBCELL																													
CELLMARKERCURNT2TRANSITIONALBCELL																													
CELLMARKERCURNTCELLNATURALKILLERCELL																													
CELLMARKERCURNTERMINALEFFECTORCD8TCELL																													
CELLMARKERCURNTERMINALLYDIFFERENTIATEDCD8…																													
CELLMARKERCURNTFOLLICULARHELPERTFHCELL																													
CELLMARKERCURNTHELPER1TH1CELL																													
CELLMARKERCURNTHELPER1TH1CELL																													
CELLMARKERCURNTHELPER2TH2CELL																													
CELLMARKERCURNTHELPER2TH2CELL																													
CELLMARKERCURNTHELPER9TH9CELL																													
CELLMARKERCURNTHELPERTHCELL																													
CELLMARKERCURNTHYMICEPITHELIALCELLTEC																													
CELLMARKERCURNTISSUERESIDENTMEMORYCD4TCELL																													
CELLMARKERCURNTISSUERESIDENTMEMORYCD8TCELL																													
CELLMARKERCURNTISSUERESIDENTMEMORYTTRMCELL																													
CELLMARKERCURNTPERIPHERALHELPERTPHCELL																													
CELLMARKERCURNTRANSIENTAMPLIFYINGTACELL																													
CELLMARKERCURNTRANSITIONALEXHAUSTEDCD8TCE…																													
CELLMARKERCURNTUMORINFILTRATINGLYMPHOCYTE…																													
CELLMARKERCURNUNSWITCHEDMEMORYBCELLUNSWMB																													
CELLMARKERCURNVASARECTAENDOTHELIALCELLECVR																													
CELLMARKERCURNVONECONOMONEURONVEN																													
CELLMARKERCURNYOLKSACDERIVEDMYELOIDBIASED…																													
CELLMARKEREXP_ACINAR_CELLS																													
CELLMARKEREXP_ADRENERGIC_NEURONS																													
CELLMARKEREXP_AIRWAY_EPITHELIAL_CELLS																													
CELLMARKEREXP_AIRWAY_SMOOTH_MUSCLE_CELLS																													
CELLMARKEREXP_ALPHA_CELLS																													
CELLMARKEREXP_ANTERIOR_PITUITARY_GLAND_CE…																													
CELLMARKEREXP_ASTROCYTES																													
CELLMARKEREXP_B_CELLS	VPREB1	LRMP	KLHL6	SLAMF6	FAM129C	BST1	MSH5	DOK3	BACH2	PXK	IGHG1	IGHG3	IGHG4	CD38	PTPRC	EBF1	BCL11A	CCR7	CD55	CD74	CD52	TLR9	SWAP70	HMGA1					
CELLMARKEREXP_B_CELLS_MEMORY																													
CELLMARKEREXP_B_CELLS_NAIVE																													
CELLMARKEREXP_BASAL_CELLS																													
CELLMARKEREXP_BASOPHILS																													
CELLMARKEREXP_BERGMANN_GLIA																													
CELLMARKEREXP_BETA_CELLS																													
CELLMARKEREXP_CAJAL_RETZIUS_CELLS	DACT1	EOMES	CNR1	HAR1A																									
CELLMARKEREXP_CHOLANGIOCYTES																													
CELLMARKEREXP_CHOLINERGIC_NEURONS																													
CELLMARKEREXP_CHONDROCYTES																													
CELLMARKEREXP_DELTA_CELLS																													
CELLMARKEREXP_DISTAL_TUBULE_CELLS																													
CELLMARKEREXP_DUCTAL_CELLS																													
CELLMARKEREXP_EMBRYONIC_STEM_CELLS	HES1	ESRRB	DNMT3B	TRIM28	PIWIL1	PIWIL2	PIWIL4	LIFR	IL6ST																				
CELLMARKEREXP_ENTERIC_NEURONS																													
CELLMARKEREXP_ENTEROENDOCRINE_CELLS																													
CELLMARKEREXP_EPENDYMAL_CELLS																													
CELLMARKEREXP_EPSILON_CELLS																													
CELLMARKEREXP_ERYTHROID_LIKE_AND_ERYTHROI…																													
CELLMARKEREXP_GAMMA_DELTA_T_CELLS																													
CELLMARKEREXP_GOBLET_CELLS																													
CELLMARKEREXP_HEMANGIOBLASTS																													
CELLMARKEREXP_HEMATOPOIETIC_STEM_CELLS	MAFF	ITGA6	GJB1	CDH2	ALDH1A1	GFI1B	NOTCH1	NOTCH2	PLXDC2	ESAM	ACE	TRPC6	HOXB5	TET2															
CELLMARKEREXP_HEPATIC_STELLATE_CELLS																													
CELLMARKEREXP_HIS_BUNDLE_CELLS																													
CELLMARKEREXP_IMMATURE_NEURONS																													
CELLMARKEREXP_JUXTAGLOMERULAR_CELLS																													
CELLMARKEREXP_KERATINOCYTES																													
CELLMARKEREXP_KIDNEY_PROGENITOR_CELLS																													
CELLMARKEREXP_KUPFFER_CELLS																													
CELLMARKEREXP_LEYDIG_CELLS																													
CELLMARKEREXP_LOOP_OF_HENLE_CELLS																													
CELLMARKEREXP_LUMINAL_EPITHELIAL_CELLS																													
CELLMARKEREXP_MAMMARY_EPITHELIAL_CELLS																													
CELLMARKEREXP_MEGAKARYOCYTES																													
CELLMARKEREXP_MELANOCYTES																													
CELLMARKEREXP_MESANGIAL_CELLS																													
CELLMARKEREXP_MESOTHELIAL_CELLS																													
CELLMARKEREXP_MICROFOLD_CELLS																													
CELLMARKEREXP_MICROGLIA																													
CELLMARKEREXP_MONOCYTES	LYZ	RHOC	PILRA	NFKBIZ	NAAA	LY6E	LYN	MS4A7	CEBPB	CD68	IFI30	S100A…	SERPI…	RGS2	LST1	SPI1	TYMP	CSTA	FGL2	PYCARD	LYST	CCL3	IL1B	CFP	CD36				
CELLMARKEREXP_MULLER_CELLS																													
CELLMARKEREXP_MYOBLASTS																													
CELLMARKEREXP_MYOCYTES	GATA5	MEF2A	MEF2B	MEF2D	POPDC2	CHODL	MYLPF	MYL1	CKM	TCAP	MYOZ1	PYGM	ENO3	THBS4															
CELLMARKEREXP_MYOFIBROBLASTS																													
CELLMARKEREXP_NEUROBLASTS																													
CELLMARKEREXP_NEUTROPHILS																													
CELLMARKEREXP_NK_CELLS	CD247	ZBTB16	SPON2	LAIR2	HOPX	CD8A																							
CELLMARKEREXP_NORADRENERGIC_NEURONS																													
CELLMARKEREXP_OLIGODENDROCYTES	MAG	SLC25A29	SLC25A19	PLEKHH1	TMEM88B	DBNDD2	EFNB3	SGK3	PNPLA2	ARRDC2	ENPP2	PLP1	ITPR2	MBP															
CELLMARKEREXP_OSTEOBLASTS																													
CELLMARKEREXP_OSTEOCLAST_PRECURSOR_CELLS																													
CELLMARKEREXP_OSTEOCLASTS																													
CELLMARKEREXP_OSTEOCYTES																													
CELLMARKEREXP_PANETH_CELLS																													
CELLMARKEREXP_PARATHYROID_CHIEF_CELLS																													
CELLMARKEREXP_PERICYTES																													
CELLMARKEREXP_PHOTORECEPTOR_CELLS																													
CELLMARKEREXP_PLASMA_CELLS	IGHGP	IGLC3	JCHAIN	IGHA1	IGHG4	IGHA2	IGLV3-1	IGLC7	SSR4	IL16	BHLHA15	PPIB																	
CELLMARKEREXP_PLASMACYTOID_DENDRITIC_CELLS																													
CELLMARKEREXP_PODOCYTES	GPRC5A	COL4A4	AIF1L	MET	EPB41L5	DOCK1	AJUBA	CD2AP	GOLIM4	ZBTB8A	TOB1	WSB2	EMC2	EHD4	ZFP91	ETF1	EMC7	LUC7L2	MAPT	CDKN1C									
CELLMARKEREXP_PRINCIPAL_CELLS																													
CELLMARKEREXP_PROXIMAL_TUBULE_CELLS	RBP4	NAPSA																											
CELLMARKEREXP_PULMONARY_ALVEOLAR_TYPE_I_C…																													

Table S5: Cell state marker gene list

CELLMARKERCAN_ACINAR_CELLS																													
CELLMARKEREXP_PULMONARY_ALVEOLAR_TYPE_II_…																													
CELLMARKEREXP_PURKINJE_FIBER_CELLS																													
CELLMARKEREXP_PURKINJE_NEURONS																													
CELLMARKEREXP_PYRAMIDAL_CELLS																													
CELLMARKEREXP_RETICULOCYTES																													
CELLMARKEREXP_RETINAL_GANGLION_CELLS																													
CELLMARKEREXP_SALIVARY_MUCOUS_CELLS																													
CELLMARKEREXP_SATELLITE_CELLS																													
CELLMARKEREXP_SCHWANN_CELLS																													
CELLMARKEREXP_SEROTONERGIC_NEURONS																													
CELLMARKEREXP_SERTOLI_CELLS																													
CELLMARKEREXP_SMOOTH_MUSCLE_CELLS	SMTN	BGN	MYL9	MYLK	PCP4L1	SNCG	PLN	NRP2																					
CELLMARKEREXP_SPERMATOZOA																													
CELLMARKEREXP_STROMAL_CELLS																													
CELLMARKEREXP_T_CELLS	CLEC2D	CD8A	CD6	S100A4	CCL5	LCK	CD81	THY1	LAT	SKAP1	TCF7	CCR7	MYB	CCL4L2	PYHIN1	GZMA	JUNB	DUSP2	IFNG	CD52	BRAF								
CELLMARKEREXP_T_CELLS_NAIVE																													
CELLMARKEREXP_T_CYTOTOXIC_CELLS																													
CELLMARKEREXP_T_HELPER_CELLS																													
CELLMARKEREXP_T_MEMORY_CELLS																													
CELLMARKEREXP_TRICHOCYTES																													
CELLMARKEREXP_TRIGEMINAL_NEURONS																													
CELLMARKEREXP_TROPHOBLAST_CELLS																													
CELLMARKEREXP_TROPHOBLAST_PROGENITOR_CELLS																													
CELLMARKEREXP_TUFT_CELLS																													
CELLMARKEREXP_VASCULAR_SMOOTH_MUSCLE_CELLS																													
CELLMARKEREXPENDOTHELIALCELLSAORTA	PDE3A	BMX	PROCR	SMOC1	CRACR2B	GJA5	BMP4	SOD3	ADH7	PCOLC…	PTGIS	ACE	GXYLT2	CYTL1	EHD3														
CELLMARKEREXPNEURALSTEMPRECURSORCELLS																													
CELLMARKERMSIGDB_AIZARANI_LIVER_C12_NK_NK…																													
CELLMARKERMSIGDB_AIZARANI_LIVER_C22_RESID…																													
CELLMARKERMSIGDB_AIZARANI_LIVER_C28_NK_NK…	AUTS2	BCL11B	LEPROTL1	PIM2	SATB1	FNBP1	RPL36A	RPS29	RCAN3	TRAC	CD52	PDE4D	PPP1R2	ZC3HA…	GPRIN3	RPS27	PIK3R1	IL2RG	PDCD4	CXCL13	RASSF5	LEF1	TRBC2	TXNIP	CXCR6				
CELLMARKERMSIGDB_AIZARANI_LIVER_C29_MVECS…	PALMD	MYCT1	TPM4	FLNB	CD93	CD59	TIE1	ITPRIP	RAMP2	IL1RL1	CALCRL	FOSL1	EFNB2	COL3A1	ESAM	APOLD1	EMCN	IFI27	ATN1	IFITM3	ENG	TSPAN7	NFATC1	NFATC2	PTGS1	COL8A1	ARHGA…	SLCO2…	ID1
CELLMARKERMSIGDB_AIZARANI_LIVER_C30_HEPAT…	ZNF605	PCDH11Y	CHRD	C8A	AGXT	HLF	C17orf…	PCDHB1	FST	BCDIN…	CYP2C9	RAD54…	TRIM72	FBXO48	COLEC…	INTS6	BIVM	C5	SELEN…	ABCC2	CA5A	PROZ	KREME…	ST3GA…	RN7SL…	ZNF454	RPS6K…	TJP2	CLMN
CELLMARKERMSIGDB_AIZARANI_LIVER_C8_RESIDE…																													
CELLMARKERMSIGDB_AIZARANI_LIVER_C9_LSECS_1	SPRY4	SEC14L1	HYAL2	IGFBP7	CDH5	MARCKS	TIE1	INSR	S100A…	PLTP	SLC2A3	SMAD6	TGFBR3	FLT4	NRP1	JMJD1C	PRCP	PIEZO2	CAVIN1	TOP1	CXCL16	APP	LIMCH1	CEP170	EPAS1	PLPP3	YES1	IL33	IFI27
CELLMARKERMSIGDB_BUSSLINGER_DUODENAL_BCHE…																													
CELLMARKERMSIGDB_BUSSLINGER_DUODENAL_DIFF…	RACK1	RPS7	IMPDH2	ATP5MC1	RPL35	RPL8	PCLAF	RPL32	RPS18	SNHG29	HNRNPR	PPIF	RPL14	HNRNP…	LRPPRC	RPL6	RPS16	TKT	GOLM1	RPS14	ADH1C	PLP2	RPL9	HINT1	NDUFA…	RPL12	ENO1	RPL15	FBL
CELLMARKERMSIGDB_BUSSLINGER_DUODENAL_EC_C…																													
CELLMARKERMSIGDB_BUSSLINGER_DUODENAL_IMMU…	CD160	B2M	ITGA1	IL2RG	TMC8	WIPF1	NLRC3	GNAI2	ALOX5…	TBC1D…	ARHGAP…	HOPX	LPXN	PREX1	SEPTI…	COTL1	JUNB	SEMA4D	SEPTI…	CISH	RASAL3	MBNL1	MYL12A	CPA3	PRKCH	ARL6I…	GPR171	FMNL1	SASH3
CELLMARKERMSIGDB_BUSSLINGER_DUODENAL_STEM…	CA9	RCN1	RPL7	RPL35A	RPS18	RPL15	MECOM	RPS12	ZFP36…	RPL7A	CLU	SET	IMPDH2	RPS13	NCL	TKT	RPS14	SMARCC1	TRIM24	RPS8	SNHG29	DSG2	CD9	HBEGF	RPL37A	BTF3	RPL11	ENC1	ADD3
CELLMARKERMSIGDB_BUSSLINGER_DUODENAL_TUFT…	SEPTIN2	KRT8	ID2	SOX4	PHIP	MT2A	MT1G																						
CELLMARKERMSIGDB_BUSSLINGER_ESOPHAGEAL_EA…	RPL14	RPL10A	EEF1A1	RPS3A	RPLP1																								
CELLMARKERMSIGDB_BUSSLINGER_ESOPHAGEAL_PR…																													
CELLMARKERMSIGDB_BUSSLINGER_GASTRIC_CHIEF…																													
CELLMARKERMSIGDB_BUSSLINGER_GASTRIC_G_CEL…	SORL1	NAV1	PRKACB	ANK3	SORBS2	MAST4	TMEM17…	SELEN…	GNAI2	PDLIM5	ABCA5	CMIP	BTG2	HLA-A	TPD52	VAMP2	DSP	BPTF	FKBP2	SSR4	FXYD3	MARCKS	SPINT2	EGR1					
CELLMARKERMSIGDB_BUSSLINGER_GASTRIC_ISTHM…	PDIA4	EFNB2	LINC011…	CALM2	ZBTB7C	H2AZ1	JUP	CYP3A5	AHNAK	TKT	LGALS3	SMC4	NQO1	AKR1C3	VSIG1	PLP2	CALR	ALDH3A1	TSPAN…	ASPM	CYP2S1	HSP90…	CYP2C…	TSPAN3	FCGBP	TXNRD1	CYP1B1	PHLDA2	PRDX1
CELLMARKERMSIGDB_BUSSLINGER_GASTRIC_NECK_…																													
CELLMARKERMSIGDB_BUSSLINGER_GASTRIC_OXYNT…	BEX3	IFI6	SEZ6L2	SSR4	VAMP2	SERPI…	YWHAQ	SOX4	EPCAM	SEC62	CALM1																		
CELLMARKERMSIGDB_BUSSLINGER_GASTRIC_PREZY…																													
CELLMARKERMSIGDB_BUSSLINGER_GASTRIC_REG3A…																													
CELLMARKERMSIGDB_CUI_DEVELOPING_HEART_5TH…																													
CELLMARKERMSIGDB_CUI_DEVELOPING_HEART_5TH…	MCEMP1	EHD4	NPPB	FEZ1	DUSP14	NTF4	HAND2	ADM																					
CELLMARKERMSIGDB_CUI_DEVELOPING_HEART_C1_…	RHPN2	AMHR2	UCHL1	VSNL1	FXYD7	NCCRP1	SLC9A3…	MGARP	LIX1																				
CELLMARKERMSIGDB_CUI_DEVELOPING_HEART_C2_…	SNTA1	LRRC10	CHCHD10	MYZAP	PPP1R1…	MAP3K…	CFL2	PFKP	POPDC3	SLC8A1	SH3RF2	CISD1	PPP1R…	PTP4A3	ADPRH…	MYH7	IDH2												
CELLMARKERMSIGDB_CUI_DEVELOPING_HEART_COM…																													
CELLMARKERMSIGDB_CUI_DEVELOPING_HEART_COR…																													
CELLMARKERMSIGDB_CUI_DEVELOPING_HEART_END…																													
CELLMARKERMSIGDB_CUI_DEVELOPING_HEART_LEF…																													
CELLMARKERMSIGDB_CUI_DEVELOPING_HEART_RIG…																													
CELLMARKERMSIGDB_CUI_DEVELOPING_HEART_SMO…																													
CELLMARKERMSIGDB_DESCARTES_FETAL_ADRENAL_…	SNX25P1	ULK4P1	ZNF683	NOMO2	FRMPD3…	CCT6B	ZNF845	AMD1P3	SPINK8	ZNF597	F2RL1	GTF2I…	ATP2A1	ELAC1	LINC0…														
CELLMARKERMSIGDB_DESCARTES_FETAL_CEREBELL…																													
CELLMARKERMSIGDB_DESCARTES_FETAL_CEREBELL…																													
CELLMARKERMSIGDB_DESCARTES_FETAL_CEREBELL…	SYK	KCNK13	NPL	LINC00…	PTAFR	MERTK	APOC1	TMC8	S100Z	RAB20	TLR10	IPCEF1	CD36	FMN1	SOCS6	LGMN	PLA2G7	ITGAL	GPNMB	PDK4	SALL1	NRROS	LRRC39	CTSC	RNASE…	CD74	RYR1	GLIPR1	RUNX1
CELLMARKERMSIGDB_DESCARTES_FETAL_CEREBELL…	FGF3	EGOT	LINC023…	RPL3P13	ATP2B2…	TRIM6…	PPP1R2…	LINC0…	ENSG0…	LINC0…	API5P2	RN7SL…																	
CELLMARKERMSIGDB_DESCARTES_FETAL_CEREBELL…																													
CELLMARKERMSIGDB_DESCARTES_FETAL_CEREBELL…																													
CELLMARKERMSIGDB_DESCARTES_FETAL_CEREBELL…	MFSD2A	NDUFA4L2	CD93	CA2	TMEM204	COL3A1	LEF1	THBD	NID1	CFH	ADM	OLFML…	ICAM2	NOTCH4	PRELP	GPER1	MECOM	RAMP2	ADGRA2	EDN1	NOSTR…	SLC9A…	CCN2	TBX18	KANK3	EHD2	SLC2A1	DLL4	IFITM3
CELLMARKERMSIGDB_DESCARTES_FETAL_CEREBRUM…																													
CELLMARKERMSIGDB_DESCARTES_FETAL_CEREBRUM…																													
CELLMARKERMSIGDB_DESCARTES_FETAL_CEREBRUM…	ST3GAL4	ABRACL	EGF	CALHM5	PDCL	DCAF12	NLRC5	MAX	ABCA2	MTURN	SENCR	TAP1	ARL1	TENT5C	PIM1	PPP4R…	SRF	CD55	F13A1	TMEM2…	NBEAL2	TSPOA…	IER5L	AHR	UROD	XK	EHD3	LINC0…	SYTL4
CELLMARKERMSIGDB_DESCARTES_FETAL_CEREBRUM…	IPCEF1	CTSC	CYTH4	SFMBT2	IL17RA	BIN1	RIN3	LPCAT2	LPIN2	DOCK4	LINC-P…	B4GAL…	HRH1	ALPK1	ADAP2	GNB4	STEAP1B	SMAP2	MERTK	CX3CR1	AOAH	TMC8	DOCK8	TREM2	MAF	BNC2	GAB3	ARHGA…	RYR1
CELLMARKERMSIGDB_DESCARTES_FETAL_CEREBRUM…	LINC02…	KRT18P10	LINC012…	TENT5B	RNU6-5…	RNU6-…																							
CELLMARKERMSIGDB_DESCARTES_FETAL_CEREBRUM…	API5P2	PPP1R2P5	RORA-AS2	SKOR1-…	TFAP2A…	BSPH1	LINC00…	WDR95P	RNU6-…	LIN28…	LINC01…	PROP1	RNU6-…	PDE11…	LINC0…	KCTD9…													
CELLMARKERMSIGDB_DESCARTES_FETAL_CEREBRUM…	MMRN2	VWA1	HBB	EVA1B	EPAS1	ID1	PGF	ACE	LSR	SLC2A1	NKD2	IGFBP7	ANGPT2	ADM	ITM2A	IFI27	FGD5	CGNL1	CCDC8…	DLC1	FN1	ITGA11	LINC0…	RBPMS	ID3	PDGFRB	FILIP1	FAM43A	SERPI…
CELLMARKERMSIGDB_DESCARTES_FETAL_EYE_ASTR…																													
CELLMARKERMSIGDB_DESCARTES_FETAL_EYE_GANG…																													
CELLMARKERMSIGDB_DESCARTES_FETAL_EYE_LENS…	PIP5KL1	NREP-AS1	HMSD	LINC01…	LINC02…	ADRA1B	FBXL21P	EFCAB8	NOCT	LINC0…	PITX3	PLEKH…	LINC0…	RNU5E…	F12	SOX1	HTR1E	ZNF778…	NEURL2										
CELLMARKERMSIGDB_DESCARTES_FETAL_EYE_PHOT…																													
CELLMARKERMSIGDB_DESCARTES_FETAL_EYE_RETI…	RASSF6	LIN28B-…	ADAD2	PLGLA	SMG1P6																								
CELLMARKERMSIGDB_DESCARTES_FETAL_EYE_SMOO…	ABCB5	TEX21P	DLGAP1-…																										
CELLMARKERMSIGDB_DESCARTES_FETAL_EYE_STRO…	MIR124…	SELENOM	LMX1B	RNASE4	EMX2OS	CHRD	SOCS1	PTHLH	EMX2	SLC6A2	ADAMTS4	MATN4	MMP19	ALX3	TNFSF9	EVA1C…	TMEM20…												
CELLMARKERMSIGDB_DESCARTES_FETAL_EYE_VASC…	MLKL	LINC030…	NOS3	USHBP1	ECSCR	ADCY4	ARHGEF…	FAM12…	PPP1R…	HAPLN3	SEMA3G	GOLGA…	MPL	STARD8	ICAM2	BTNL8	CLEC1A												
CELLMARKERMSIGDB_DESCARTES_FETAL_HEART_EL…																													
CELLMARKERMSIGDB_DESCARTES_FETAL_HEART_EN…																													
CELLMARKERMSIGDB_DESCARTES_FETAL_HEART_LY…																													
CELLMARKERMSIGDB_DESCARTES_FETAL_HEART_SC…																													
CELLMARKERMSIGDB_DESCARTES_FETAL_HEART_SM…																													
CELLMARKERMSIGDB_DESCARTES_FETAL_HEART_ST…																													
CELLMARKERMSIGDB_DESCARTES_FETAL_HEART_VI…	CPEB1	SLC18A1	MYT1	RAB39B	NPM2	ECEL1	FNDC9	ENSG0…	KCNK12	LINC0…	HCN2	GATA3…	C14or…																
CELLMARKERMSIGDB_DESCARTES_FETAL_INTESTIN…																													
CELLMARKERMSIGDB_DESCARTES_FETAL_INTESTIN…																													
CELLMARKERMSIGDB_DESCARTES_FETAL_INTESTIN…	RAET1E	PHYHIP	PDE7B-A…	ACTRT3	SULT1D…	CPA4	MTND6P…	SFTPD	FGF11	RPL34…	KLHL26	EGOT	LINC0…	AGAP11															
CELLMARKERMSIGDB_DESCARTES_FETAL_INTESTIN…																													
CELLMARKERMSIGDB_DESCARTES_FETAL_INTESTIN…																													
CELLMARKERMSIGDB_DESCARTES_FETAL_INTESTIN…																													
CELLMARKERMSIGDB_DESCARTES_FETAL_KIDNEY_M…																													
CELLMARKERMSIGDB_DESCARTES_FETAL_KIDNEY_U…	MUC1	FER1L4	AOC1	CLDN7	FBP1	UPK1B	MIR31HG	LINC0…	KSR2	WNT7B	SLC38A…	CFI	C6orf…	VTCN1	LAD1	ST14	CNGA1	ARHGEF…	RNF43	SLC9A2	GRHL3	BICDL2	PROM2	RASSF6	NECTI…	EVPL	VSIG2	TMC4	GRHL1
CELLMARKERMSIGDB_DESCARTES_FETAL_LIVER_ER…	ADAM7	RNU6-67…	GRASLND	RNU6-1…																									
CELLMARKERMSIGDB_DESCARTES_FETAL_LIVER_HE…																													
CELLMARKERMSIGDB_DESCARTES_FETAL_LIVER_HE…	BHMT2	FGG	HMGCS1	FGA	MT1G	APOH	SULT1E1	VTN	PROX1	TTR	HPN	HGD	LINC0…	FOXP2	HMGCS2	MT1E	A1CF	ANGPTL3	SUGCT	MT1F	LIPC	SLC22…	ADH1A	ALDOB	APOC1	PBLD	GPC5	AMN	SERPI…
CELLMARKERMSIGDB_DESCARTES_FETAL_LIVER_ME…	DPP6	LGALS3	NXPH2	MGAT4C	EFEMP1	GSG1L	SPOCK1	SAMD11	ARHGA…	HSPA1B	LAMA5	FGF9	GFPT2	SLC16…	TLL1	GRIK2	ARHGEF…	RSPO1	EMP2	TNNT1	SLC34…	COL8A1	NBL1	ELFN1	TENM3	COL9A3	HS6ST3	MUC16	GJA1
CELLMARKERMSIGDB_DESCARTES_FETAL_LUNG_BRO…																													
CELLMARKERMSIGDB_DESCARTES_FETAL_LUNG_CIL…	TMC5	SPATA17	CFAP70	DCDC1	DNAI4	TMEM2…	CFAP210	DNAH10	DNAI7	ODF3B	CFAP61	CFAP91	PACRG	PPIL6	CFAP44	CCDC1…	CETN2	EFCAB6	CASC2	CCDC30	DNAH7	KCNE1	DNAH6	RGS22	LRGUK	STK33	ENKUR	SPAG16	IFT57
CELLMARKERMSIGDB_DESCARTES_FETAL_LUNG_LYM…	LINC02…																												

Table S5: Cell state marker gene list

CELLMARKERCAN_ACINAR_CELLS																													
CELLMARKERMSIGDB_DESCARTES_FETAL_LUNG_NEU…	RHBDL1	SMIM32	MBOAT4	ENSG00…	SSTR5-…	PTGFR	NPC1L1	PDCD6…	OPRD1	GPX2	PKD2L1	ETS2-…	SLC6A…	GKN2	TRPC7…	NDUFB…	NREP-A…												
CELLMARKERMSIGDB_DESCARTES_FETAL_LUNG_STR…																													
CELLMARKERMSIGDB_DESCARTES_FETAL_LUNG_VAS…																													
CELLMARKERMSIGDB_DESCARTES_FETAL_MUSCLE_L…																													
CELLMARKERMSIGDB_DESCARTES_FETAL_MUSCLE_S…																													
CELLMARKERMSIGDB_DESCARTES_FETAL_MUSCLE_S…	BCAS3-…	RBPMS2	LEFTY2	NMUR2	MYO1B-…	ANKRD…																							
CELLMARKERMSIGDB_DESCARTES_FETAL_MUSCLE_V…	TM4SF1…	LINC013…																											
CELLMARKERMSIGDB_DESCARTES_FETAL_PANCREAS…	RPL7AP…	LHB	STEAP1	AQP12A	PTCHD3	PNLIP	PRSS3P1	ENSG0…	PRR15	RTL1	LINC02…	REG1B	IL17C	C12or…	OPN4	LINC0…													
CELLMARKERMSIGDB_DESCARTES_FETAL_PANCREAS…																													
CELLMARKERMSIGDB_DESCARTES_FETAL_PANCREAS…																													
CELLMARKERMSIGDB_DESCARTES_FETAL_PANCREAS…																													
CELLMARKERMSIGDB_DESCARTES_FETAL_PANCREAS…	RPL21P…	RSPO1	MT1A	KLK10	LINC01…	LRP8-…																							
CELLMARKERMSIGDB_DESCARTES_FETAL_PANCREAS…																													
CELLMARKERMSIGDB_DESCARTES_FETAL_PANCREAS…																													
CELLMARKERMSIGDB_DESCARTES_FETAL_PLACENTA…																													
CELLMARKERMSIGDB_DESCARTES_FETAL_PLACENTA…	DAAM2-…	PLCB1-I…	CIB4	VASH2	TRPC3	COL13…	CACNA1…	GPR20	SCN4B	ABCC8	ANGPTL6	ERFE	LINC0…	PAQR5	FAM22…	FOXF2													
CELLMARKERMSIGDB_DESCARTES_FETAL_PLACENTA…	ENSG00…	FLJ12825	KCNJ12	MIR218…	KCTD19	LINC0…	RPL31P…	LINC0…	HSD3B…	CCNYL2	GATA6-…	ASB18	LINC0…	ARHGA…	RPL17…	LINC0…	SFTPD-…	LINC01…	SLC22…	ASZ1	MATN1	LINC0…	PLAAT5						
CELLMARKERMSIGDB_DESCARTES_FETAL_PLACENTA…	OR8G1	ZNF723	CLCA2	CTBP2P8	CDH8-A…	LINC0…																							
CELLMARKERMSIGDB_DESCARTES_FETAL_PLACENTA…	SCNN1B	GH1	NCF4-AS1	MISP3	PSG8-A…	LINC0…	ANKRD33	LY6G6C	ENSG0…	TREML…	HSPD1P…	RRS1-…	POU2F3	SLC7A4	XKRX	PLCE1…	PSG10P	PRSS41	LINC0…										
CELLMARKERMSIGDB_DESCARTES_FETAL_SPLEEN_E…																													
CELLMARKERMSIGDB_DESCARTES_FETAL_SPLEEN_S…																													
CELLMARKERMSIGDB_DESCARTES_FETAL_SPLEEN_V…																													
CELLMARKERMSIGDB_DESCARTES_FETAL_STOMACH_…	EFCAB6	C4orf47	CFAP52	EFHC1	DNAI2	C20or…	FANK1	ODAD2	ABCA13	ALDH3…	DNAH5	DNAI3	DLEC1	AK9	BBOF1	ROPN1L	NEK5	AK8	TOGAR…	CFAP91	C9orf…	SPATA…	CFAP46	DNAH2	ZMYND…	C1orf…	CFAP2…	CSPP1	LRRC46
CELLMARKERMSIGDB_DESCARTES_FETAL_STOMACH_…																													
CELLMARKERMSIGDB_DESCARTES_FETAL_STOMACH_…																													
CELLMARKERMSIGDB_DESCARTES_FETAL_STOMACH_…	CHI3L2	OAS2	IL2RB	ZNF831	CORO1A	LINC0…	GZMA	TAP2																					
CELLMARKERMSIGDB_DESCARTES_FETAL_STOMACH_…																													
CELLMARKERMSIGDB_DESCARTES_FETAL_STOMACH_…	SLC5A9	CCL25	KHK	ADRA2A	NR1I2	GPD1	SLC17A4	OTC	HNF4A…	MUC3A	MIR194…	GDPD2	ANKS4B	NMUR2	CHST13	LRRC66	PCK1	ANKRD4…	CYP2B…	ABCC6…	MGC32…	PLA2G…	LINC0…						
CELLMARKERMSIGDB_DESCARTES_FETAL_STOMACH_…	IDO2	NFAM1																											
CELLMARKERMSIGDB_DESCARTES_FETAL_STOMACH_…																													
CELLMARKERMSIGDB_DESCARTES_FETAL_STOMACH_…																													
CELLMARKERMSIGDB_DESCARTES_FETAL_STOMACH_…																													
CELLMARKERMSIGDB_DESCARTES_FETAL_STOMACH_…																													
CELLMARKERMSIGDB_DESCARTES_FETAL_STOMACH_…																													
CELLMARKERMSIGDB_DESCARTES_FETAL_THYMUS_T…																													
CELLMARKERMSIGDB_DESCARTES_MAIN_FETAL_ACI…																													
CELLMARKERMSIGDB_DESCARTES_MAIN_FETAL_ADR…	MC2R	NPC1	CLEC4GP1	LDLR	OR7A5	ZNF98	LIPE	SULT2…	PPP2R…	MRAP	NR0B1	ENSG0…	TBC1D…	ZNF275															
CELLMARKERMSIGDB_DESCARTES_MAIN_FETAL_AST…																													
CELLMARKERMSIGDB_DESCARTES_MAIN_FETAL_BIP…	LINC00…	CDH20	RNU6-11…	LINC01…	HCN2	ENSG0…	ZNF223	BSPH1	ELSPB…	MACRO…	AIMP1P1	MACRO…	MACRO…	VSX1	GRIK1	GRIK1…	DSCAM	DSCAM-…	ARSH	ENSG0…									
CELLMARKERMSIGDB_DESCARTES_MAIN_FETAL_BRO…																													
CELLMARKERMSIGDB_DESCARTES_MAIN_FETAL_CAR…																													
CELLMARKERMSIGDB_DESCARTES_MAIN_FETAL_CIL…	IFT172	TOGARAM2	DPY30	MORN2	BCYRN1	C2orf…	CLHC1	CFAP36	SANBR	DNAAF…	WDR54	DNAH6	TEKT4	VWA3B	CRACDL	TSGA10	TBC1D8	CCDC138	NPHP1	RABL2A	CFAP2…	CCDC7…	CCDC7…	MAP3K…	STAM2	TTC21B	BBS5	CFAP2…	ERICH…
CELLMARKERMSIGDB_DESCARTES_MAIN_FETAL_DUC…																													
CELLMARKERMSIGDB_DESCARTES_MAIN_FETAL_END…	TPT1P15	ARMCX2	PWWP3B	AVPR2	F8	CLIC2																							
CELLMARKERMSIGDB_DESCARTES_MAIN_FETAL_ENS…																													
CELLMARKERMSIGDB_DESCARTES_MAIN_FETAL_EPI…																													
CELLMARKERMSIGDB_DESCARTES_MAIN_FETAL_GAN…																													
CELLMARKERMSIGDB_DESCARTES_MAIN_FETAL_GOB…																													
CELLMARKERMSIGDB_DESCARTES_MAIN_FETAL_HEM…	MIR366…	MTND5P26	FIRRE	EOLA2																									
CELLMARKERMSIGDB_DESCARTES_MAIN_FETAL_HOR…																													
CELLMARKERMSIGDB_DESCARTES_MAIN_FETAL_INH…																													
CELLMARKERMSIGDB_DESCARTES_MAIN_FETAL_INH…																													
CELLMARKERMSIGDB_DESCARTES_MAIN_FETAL_INT…	FADS6	ENPP7	PVALEF	REEP6	PGPEP1	TM6SF2	ERICH4	IGSF23	CEACA…	CBLC	CEACAM…	POFUT1	R3HDML	RBBP8…	DNAJB7	XPNPE…													
CELLMARKERMSIGDB_DESCARTES_MAIN_FETAL_ISL…																													
CELLMARKERMSIGDB_DESCARTES_MAIN_FETAL_LYM…	GJD3	SPHK1	ZNF24TR	INO80C	TNFRSF…	NFATC1	PARD6G	HOMER3	ZNF34…	D21S2…	TSPEAR	TBX1	SLC35…	DIPK2B	KLHL4														
CELLMARKERMSIGDB_DESCARTES_MAIN_FETAL_MES…																													
CELLMARKERMSIGDB_DESCARTES_MAIN_FETAL_MET…																													
CELLMARKERMSIGDB_DESCARTES_MAIN_FETAL_MYE…	SIGLEC…	SIGLEC9	SIGLEC7	CD33	SIGLEC…	SIGLE…	FPR2	FPR3	LILRB3	LILRA6	LILRB5	LILRB2	LILRA5	LILRB1	LILRA1	FCAR	SIGLEC1	SAMHD1	PELAT…	IFNAR2	LINC0…	HMOX1	NCF4	TLR8					
CELLMARKERMSIGDB_DESCARTES_MAIN_FETAL_NEU…																													
CELLMARKERMSIGDB_DESCARTES_MAIN_FETAL_OLI…																													
CELLMARKERMSIGDB_DESCARTES_MAIN_FETAL_PDE…	FRMD7																												
CELLMARKERMSIGDB_DESCARTES_MAIN_FETAL_PDE…	PHC3	RPL22L1	KLHL24	TRA2B	ENSG00…	LSG1	MUC20-…	ZNF87…	FAM53A	CLNK	LINC02…	RASSF6	PARM1	HELQ	STPG2	LSM6	C4orf45	ZDHHC1…	BRD9	TERLR1	ZNF622	LINC0…	MTMR12	ZFR	AMACR	DDX4	RMEL3	TRAPP…	EEF1B…
CELLMARKERMSIGDB_DESCARTES_MAIN_FETAL_RET…																													
CELLMARKERMSIGDB_DESCARTES_MAIN_FETAL_SAT…																													
CELLMARKERMSIGDB_DESCARTES_MAIN_FETAL_SCH…																													
CELLMARKERMSIGDB_DESCARTES_MAIN_FETAL_SLC…	RNF7	LINC020…	RPL38P1	SIAH2	RNU6-6…	ACTL6A	LINC02…	LINC0…	SDHAP1	MAPK1…	RNU6-1…	MAD2L1	C4orf…	LINC0…	DCTD	EXOC3…	NUP155	TMEM267	AMD1P3	DHX29	SREK1…	GTF2H…	ANKRD…	POLK	TBCA	ATP6A…	LUCAT1	GIN1	SRP19
CELLMARKERMSIGDB_DESCARTES_MAIN_FETAL_SMO…																													
CELLMARKERMSIGDB_DESCARTES_MAIN_FETAL_STE…																													
CELLMARKERMSIGDB_DESCARTES_MAIN_FETAL_SYM…																													
CELLMARKERMSIGDB_DESCARTES_MAIN_FETAL_UNI…																													
CELLMARKERMSIGDB_DESCARTES_MAIN_FETAL_URE…																													
CELLMARKERMSIGDB_DESCARTES_MAIN_FETAL_VAS…																													
CELLMARKERMSIGDB_DESCARTES_MAIN_FETAL_VIS…	FSD1	RNA5SP4…	TMEM59L	SNPH	VN1R10…	ENSG0…	CPMER	NEFH	YWHAH	SULT4…	MKRN5P	MAGEE1	KRT18…	BCORP1															
CELLMARKERMSIGDB_DURANTE_ADULT_OLFACTORY_…																													
CELLMARKERMSIGDB_DURANTE_ADULT_OLFACTORY_…																													
CELLMARKERMSIGDB_DURANTE_ADULT_OLFACTORY_…																													
CELLMARKERMSIGDB_DURANTE_ADULT_OLFACTORY_…																													
CELLMARKERMSIGDB_DURANTE_ADULT_OLFACTORY_…	NR2F2	CLEC11A	GJA1	TPM2	SSPN	KCNE4	GATA2	SELEN…																					
CELLMARKERMSIGDB_DURANTE_ADULT_OLFACTORY_…																													
CELLMARKERMSIGDB_DURANTE_ADULT_OLFACTORY_…	APOE	ZNF331	NR4A2	DUSP1	YWHAH	CEBPD	SLC40A1																						
CELLMARKERMSIGDB_DURANTE_ADULT_OLFACTORY_…																													
CELLMARKERMSIGDB_DURANTE_ADULT_OLFACTORY_…																													
CELLMARKERMSIGDB_DURANTE_ADULT_OLFACTORY_…	UCP2	CMC1	DUSP2	EFHD2	PLAAT4	CCND3	CYRIB	PSMB9	PPP2R…	PTGDS																			
CELLMARKERMSIGDB_DURANTE_ADULT_OLFACTORY_…																													
CELLMARKERMSIGDB_DURANTE_ADULT_OLFACTORY_…	PRCP	ABCB1	CAVIN1	CDKN1A	FXYD5																								
CELLMARKERMSIGDB_DURANTE_ADULT_OLFACTORY_…																													
CELLMARKERMSIGDB_DURANTE_ADULT_OLFACTORY_…																													
CELLMARKERMSIGDB_DURANTE_ADULT_OLFACTORY_…																													
CELLMARKERMSIGDB_DURANTE_ADULT_OLFACTORY_…	GGT5	EBF1	IRAG1	PPP1R1…	PDGFRB	PALLD	EFHD1	JUN	COX4I2	COL14…	MYL6	PTP4A3	JUNB	TNS1	TGFB1…	CCL2	GADD45B	HSPA1B	TCIM										
CELLMARKERMSIGDB_FAN_EMBRYONIC_CTX_ASTROC…	EMID1	NPNT	CSDC2	ANGPTL1	CYB5A																								
CELLMARKERMSIGDB_FAN_EMBRYONIC_CTX_BIG_GR…	SLC2A1	ABCB1	LAMB2	CALD1	ADGRF5	ID1	SLC7A5	C1orf…	FSTL1	APCDD1	COL5A2	MYL9	FOXC1	EPAS1	TFPI	CLEC1…	APLNR	FOXF2	HES1	CSPG4	LAMC1	IFI6	VAMP5	BSG	CD34	TFRC	COL6A2	EMCN	MSRB3
CELLMARKERMSIGDB_FAN_EMBRYONIC_CTX_BIG_GR…	DDAH1	TMEM238L	SCN4B	RCAN2	VAT1	INSM2	SLC22A…	FBXO2	PLPP2	SNCB	LINGO2	CCDC8…	ANXA6	GDF5	SLC27…	THSD7B	ZIC2	OPCML	GPR37	IGSF9	ANKRD…	GNG3	GOT1	CYCS	IRF6	MGST3	LHFPL6	RAB37	TNFRS…
CELLMARKERMSIGDB_FAN_EMBRYONIC_CTX_BIG_GR…																													
CELLMARKERMSIGDB_FAN_EMBRYONIC_CTX_BIG_GR…	SLCO2B1	IFNGR1	TYROBP	TLR7	PLXDC2	C2	IL6R	LPAR6	CD86	CD53	MNDA	PTGS1	A2M	TLR10	SIGLE…	GPR183	ADRB2	IRF8	LGMN	DOCK8	PYCARD	HTRA1	SLC9A9	NINJ1	PLVAP	ITGB2	GAL3S…	LAIR1	GGTA1
CELLMARKERMSIGDB_FAN_EMBRYONIC_CTX_BRAIN_…	MYCT1	RGCC	ECM1	CLIC4	SLCO2A1	HOPX	RUNDC3B	LY6E	CAVIN3	SLC39…	IFI6	MYO10	SAMM50	DPEP1	PALMD	RASGR…	TM4SF1	LXN	VWA1	DLC1	CGNL1	QPCT	THSD1	APOLD1	TMEM2…	VAMP5	CD82	NOTCH4	NAMPT
CELLMARKERMSIGDB_FAN_EMBRYONIC_CTX_BRAIN_…	LAMB1	SEPTIN11	ECE1	COL9A1	TPM4	LAMC1	MYL9	SEPTI…	TGFB1…	COLEC…	ISYNA1	RBP1	SPRY1	CLEC1…	ITGB1	LAMA2	VAMP5	SLC20A2	COX7A1	GUCY1…	THY1	PTK2	SERPI…	SERPI…	CDH11	CNN3	MAGED2	CRIP2	MFGE8
CELLMARKERMSIGDB_FAN_EMBRYONIC_CTX_EX_1_E…																													
CELLMARKERMSIGDB_FAN_EMBRYONIC_CTX_IN_2_I…																													
CELLMARKERMSIGDB_FAN_EMBRYONIC_CTX_IN_3_I…																													
CELLMARKERMSIGDB_FAN_EMBRYONIC_CTX_IN_5_I…																													
CELLMARKERMSIGDB_FAN_EMBRYONIC_CTX_IN_6_I…																													
CELLMARKERMSIGDB_FAN_EMBRYONIC_CTX_MICROG…																													
CELLMARKERMSIGDB_FAN_EMBRYONIC_CTX_OLIG	NKX6-2	KIF13B	NCOA7	TUBB4A	REEP3	CSRP1	SLC12A2	ENDOD1	EDIL3	ANO4	STRN	ABCA2	ELAPO…	LPAR1	NKAIN2	BZW2	SYNGR2	GAL3ST1	ERBB3	RNF13	YPEL2	BACE1	SOX10	FAR1	SELEN…	LINC0…	PLAAT3	PPP1R…	TSC22…
CELLMARKERMSIGDB_FAN_EMBRYONIC_CTX_OPC																													

Table S5: Cell state marker gene list

CELLMARKERCAN_ACINAR_CELLS																													
CELLMARKERMSIGDB_FAN_OVARY_CL1_GPRC5A_TNF…	EMP1	MRPL32	EMP2	MFAP4	GPC3	PLTP	LGALS3	STIP1	BAG3	EEF2	TENT5A	KPNA2	PDLIM3	CYTOR	SFPQ	RRS1	KLF4	AEBP1	TUBA1C	CCDC80	PNRC1	NPM1	RPL7A	SNRPB	ILF2	DUSP14	NBL1	SERBP1	CTSL
CELLMARKERMSIGDB_FAN_OVARY_CL11_MURAL_GRA…	TEAD1	PRDM1	SQLE	STK17B	TMEM97	JPT1	ZFAND5	VMP1	SARAF	CDH2	HMGCS1	CCND2	HEY2	CTNNA…	CD55	TCEAL9	TCP1	ACSL4	DSE	NDFIP1	SFPQ	PRKAR…	CRHBP	ARHGA…	GRB14	DNAJB4	TXNRD1	G0S2	TCEA3
CELLMARKERMSIGDB_FAN_OVARY_CL13_MONOCYTE_…	INSIG1	SERPINA1	ARPC1B	HLA-DMB	RGS1	AP1S2	CD86	RNASE…	ITGB2	FCGR2A	S100A6	PFN1	GRN	PHACT…	RNASE6	GPAT3	CFP	TNFSF1…	GMFG	PSAP	STX11	SAMSN1	PKM	HMOX1	ATP1B3	CAPG	VAMP8	VMO1	ANXA2
CELLMARKERMSIGDB_FAN_OVARY_CL14_MATURE_SM…	FXYD1	SELENOW	CRYAB	ARPC1A	IFITM3	ID2	COL18A1	PPP1R…	HCFC1…	TGFB1…	GUCY1A1	MGST3	GSN	RHOB	EFHD1	TSC22…	PPP1R1…	EGR1	FOSB	IGFBP5	ESAM	MAP1B	PLS3	SEPTI…	MSRB3	RASD1	MYL6	RERG	HSPA2
CELLMARKERMSIGDB_FAN_OVARY_CL15_SMALL_ANT…	CRNDE	CACYBP	ING1	TAX1BP1	PTGER2	DYNLL1	PPA1	BUD31	RAB5IF	ATF3	ENC1	CSPP1	PPP1R…	LSR	PLAAT3	TCP1	SERTAD1	SOWAHC	PLIN3	FAM21…	STMN1	RPS26	IVNS1…	GNAI1	MIF	TRMT1…	MAGED2	C4orf…	FSCN1
CELLMARKERMSIGDB_FAN_OVARY_CL18_B_LYMPHOC…	TRAF4	SH3TC1	HSP90AA1	VAMP8	CLECL1P	SLC7A…	FCER1G	LSP1	CTSS	HVCN1	RANBP2	SSR4	MS4A6A	CYB56…	P2RY6	GRN	LILRA4	RUBCN	GUK1	SLC7A5	MAP1A	HLA-A	NCF1	TYMP	PMAIP1	IL4I1	OPN3	SPIB	DNASE…
CELLMARKERMSIGDB_FAN_OVARY_CL2_PUTATIVE_E…	RPS15A	UQCRB	IFITM3	RPS23	RPL17	RPL7	RPS18	TPM2	RPL24	RPLP1	RPS8	RPL32	PFDN5	RPL39	BEX3	RPS13	TMA7	RPL37	LAPTM…	RPS24	RPS15	RPL21	RPL11	RPS20					
CELLMARKERMSIGDB_FAN_OVARY_CL5_HEALTHY_SE…	ISOC1	GADD45G	LAMP5	COL18A1	RBBP6	CTSC	SDC2	DSEL	DDX5	KLHDC…	SEPTIN9	PMP22	CCNL1	RGS2	SPG21	MMP2	BAG3	ARF4	RCN1	MGARP	PPP1CC	PHLDA1	DYNLL1	CFAP20	TMEM4…	NR4A1	VGLL3	GLRX	RPS7
CELLMARKERMSIGDB_FAN_OVARY_CL6_PUTATIVE_E…	TCF21	RNASE4	EMILIN1	TCEAL1	RARRES2	LRRC17	THY1	CCN5	INTS6	ZFP36…	ALDH7A1	LGALS1	IER2	MYL9	SELEN…	PDGFRA	ITM2B	PLPP3	PSAP	SSPN	NFIA	C20or…	CTSK	COLEC…	KRT19	TMEM98	NDRG2	ESD	GADD4…
CELLMARKERMSIGDB_FAN_OVARY_CL8_MATURE_CUM…	GSTP1	LSM5	TBCA	H4C3	CKB	NDUFA7	CDH2	TMEM97	GTF2H5	EMX2	MT1F	MACRO…	CTSV	DNPH1	UQCR10	CLIC1	HUWE1	LGALS3…	ELK1	S100A…	PRDX3	HMGB2	TMEM1…	SRSF9	ATP5M…	NDUFA3	CDH3	ATP5MF	ZNF358
CELLMARKERMSIGDB_FAN_OVARY_CL9_PUTATIVE_A…	NCOA7	IER3	EHD4	SMAD1	CAV2	MBNL1	LAMA4	BCAM	TUBB6	PRSS23	LPAR6	BACE2	EPAS1	CNKSR3	GBP1	PCDH17	TMEM25…	SAT1	ESAM	ARRDC3	TFPI	EFNA1	SP100	VMP1	PLPP3	LAP3	KLF6	PTPRB	UGCG
CELLMARKERMSIGDB_GAO_ESOPHAGUS_25W_C1_CIL…	CACNG6	CAPS2	CAPSL	DNAI7	CCDC103	ODAD1	CCDC13	CCDC1…	CCDC1…	CCDC1…	CFAP210	CCDC1…	CCDC24	CCDC33	CCDC39	CCDC60	CCDC65	CCDC74A	CCDC7…	CCDC78	CCDC81	CCDC8…	CD164…	CD38	CDHR3	CDHR4	CDS1	CEBPA	CEP162
CELLMARKERMSIGDB_GAO_ESOPHAGUS_25W_C2_KRT…	ZFAND2A	ZNF503-…	ZNF710																										
CELLMARKERMSIGDB_GAO_ESOPHAGUS_25W_C3_FGF…																													
CELLMARKERMSIGDB_GAO_LARGE_INTESTINE_24W_…	PRICKL…	QSER1	RCN3	RERG	SCN7A	SCUBE2	SERPIN…	SERPI…	SFRP1	SFRP2	SPARC	SPON1	SPON2	SPTLC3	SRPX	SVEP1	THBS2	TMEM59L	TNFAI…	TPST1	TTYH2	VASN	VCAM1	VCAN	ZNF469				
CELLMARKERMSIGDB_GAO_LARGE_INTESTINE_24W_…																													
CELLMARKERMSIGDB_GAO_LARGE_INTESTINE_24W_…	CXCL3	CXCL8	CXCL9	CXCR4	EOLA1	CYBB	CYTH4	DAB2	DNAJA4	DNASE…	DOCK2	DOK2	DUSP2	DUSP6	EBI3	EDEM1	EIF4A3	EVI2B	EYA2	F13A1	FAM11…	FAM16…	ABRAX…	CALHM6	CYRIA	RIPOR1	FCER1G	FCGR1A	FCGR2A
CELLMARKERMSIGDB_GAO_LARGE_INTESTINE_24W_…																													
CELLMARKERMSIGDB_GAO_LARGE_INTESTINE_24W_…																													
CELLMARKERMSIGDB_GAO_LARGE_INTESTINE_24W_…																													
CELLMARKERMSIGDB_GAO_LARGE_INTESTINE_ADUL…	CXXC4	DIRAS3	DRICH1	DUSP26	DUSP4	DZIP3	EFCC1	ELAVL4	ENKD1	ENPP2	ETV1	F5	ALKAL1	TAFA5	FAP	FAR2P2	FBLL1	FBXL16	FEV	FFAR2	FGFBP3	FGFR1	FMN2	FRMD6	FRY	FSTL4	FTX	GALR1	CASTO…
CELLMARKERMSIGDB_GAO_LARGE_INTESTINE_ADUL…	COL5A1	COL6A4P2	CPB2-AS1	CPED1	CPT1B	CTBP1…	CTIF	CTSZ	CTU1	CX3CL1	CXCL5	CYP2B6	DAND5	DCBLD1	DCDC2	DGAT2	DGKG	DHX57	DLST	DNAH5	DNAJC…	DUSP16	EFR3B	ELOVL5	ENTPD…	EPG5	EPGN	EYS	FAM18…
CELLMARKERMSIGDB_GAO_LARGE_INTESTINE_ADUL…																													
CELLMARKERMSIGDB_GAO_LARGE_INTESTINE_ADUL…	CLIP4	COL18A1	COL24A1	COL4A4	COL9A3	COQ10A	CORO1A	CPNE5	CREB3…	CSF2RB	CSGALN…	CST7	CTSW	TASL	CYBB	CYP4F…	CYTH4	CYTIP	DAPP1	DENND…	DENND…	DERL3	DOCK2	DOCK8	DOK2	DOK3	DTNB	DUSP5	EAF2
CELLMARKERMSIGDB_GAO_LARGE_INTESTINE_ADUL…	CDH5	CDH6	CEP112	CFH	CHL1	CHST11	CHST7	CLDN5	CLEC1…	CLEC1…	CLEC3B	CLIC2	CLMP	CNRIP1	CNTLN	COL12…	COL13A1	COL14A1	COL15…	COL18…	COL1A1	COL1A2	COL27…	COL3A1	COL4A1	COL4A2	COL4A5	COL4A6	COL5A1
CELLMARKERMSIGDB_GAO_SMALL_INTESTINE_24W_…																													
CELLMARKERMSIGDB_GAO_SMALL_INTESTINE_24W_…	S100A9	S100G	SEC11C	SERPIN…	SLC34A3	SLC46…	SLC5A9	SLC6A…	SLC7A7	SMIM1	SMIM24	STARD5	SULT1…	TGM2	TM4SF…	TMEM1…	TMEM86B	TREH	TSPAN…	UGT3A1	VTN	XPNPE…	ZDHHC…						
CELLMARKERMSIGDB_GAO_SMALL_INTESTINE_24W_…																													
CELLMARKERMSIGDB_GAO_SMALL_INTESTINE_24W_…																													
CELLMARKERMSIGDB_GAO_SMALL_INTESTINE_24W_…																													
CELLMARKERMSIGDB_GAO_STOMACH_24W_C1_PROCR…																													
CELLMARKERMSIGDB_GAO_STOMACH_24W_C2_TFF2P…																													
CELLMARKERMSIGDB_GAO_STOMACH_24W_C4_PARIE…																													
CELLMARKERMSIGDB_GAO_STOMACH_24W_C5_PUTAT…																													
CELLMARKERMSIGDB_GAUTAM_EYE_IRIS_CILIARY_…	CEP112	ARSB	KXD1	PLEKHG1	SLC38A…	SLC25…	RHOBTB1	PDSS2	RELL1	PSMD14	SLC19A3	REXO4	PPAN	METAP1	ZC3HA…	PKN3	SCYL3	PDCD6IP	MCUR1	PLAA	IFI44	KIF3B	LINC0…	KHSRP	UBR1	ATXN2L	SNN	MTX2	NEMF
CELLMARKERMSIGDB_GAUTAM_EYE_IRIS_CILIARY_…	S100A4	HSPG2	LYPD6B	CKS2	TPMT	SERPI…	SIX3	EPB41…																					
CELLMARKERMSIGDB_GAUTAM_EYE_IRIS_CILIARY_…	TMEM63A	RAB5IF	LINC009…	PEA15	APOBEC…	MCOLN2	HDAC10	COMMD6	TMEM2…	CENPM	LINC01…	SSNA1	HSP90…																
CELLMARKERMSIGDB_GAUTAM_EYE_IRIS_CILIARY_…	AK3	ELL2	BMP1	GEM	PITPNC1	LAMA4	PDPN	CP	CALD1	ITM2B	MTHFD2																		
CELLMARKERMSIGDB_GAUTAM_EYE_IRIS_CILIARY_…	FNIP2	WDR47	PPP2R2B	CLMN	CRK	ATP6V…	ADGRB3	RIN2	NTM	RLBP1	MAP3K11	IFNAR2	ZNF532	COL5A3	CAT	OPN3	LYSMD2												
CELLMARKERMSIGDB_HAY_BONE_MARROW_CD34_POS…																													
CELLMARKERMSIGDB_HAY_BONE_MARROW_CD34_POS…																													
CELLMARKERMSIGDB_HAY_BONE_MARROW_CD34_POS…																													
CELLMARKERMSIGDB_HAY_BONE_MARROW_CD34_POS…																													
CELLMARKERMSIGDB_HAY_BONE_MARROW_CD8_T_CE…																													
CELLMARKERMSIGDB_HAY_BONE_MARROW_EARLY_ER…	FAM136A	WDR43	NDUFAF2	PNPO	PABPC4	DNAH14	DMAC1	RRP9	SLC12…	OLA1	SDHAF3	RPP40	ATP5MK	INTS13	NDUFC2	GTF3A	ABO	GATA2-…	NMI	MDH2	EXTL2								
CELLMARKERMSIGDB_HAY_BONE_MARROW_EOSINOPH…																													
CELLMARKERMSIGDB_HAY_BONE_MARROW_NAIVE_T_…	RPS3A	CD2	CD48	RPS4X	CAMK4	CD6	BCL11B	RPS7	LAT	RPL22	RPL7	ACAP1	TRBC1	CLEC2D	TLE5	TRAT1	SH3YL1	MGAT4A	RPL39	PASK	RPL17	NELL2	BTG1	RPL28	TNFRS…	RPS5	AQP3	GIMAP7	OXNAD1
CELLMARKERMSIGDB_HAY_BONE_MARROW_NEUTROPH…	SERF2	RGS2	ANXA1	CD93	NCF1	NCF2	NUP214	MXD1	JUND	FOSL2	LY96	KLF6	MEGF9	BRI3	TMEM1…	RNF130	IRS2	TMSB10	ZFAND5	SLC2A3	CTSZ	ASGR1	STX11	LRRK2	GLIPR1	RHOB	ACTB	LCP1	ANPEP
CELLMARKERMSIGDB_HAY_BONE_MARROW_NK_CELLS	KIR2DL3	CD300A	NCAM1	ABHD17A	IRF1-A…	IL18R…	ID2	PRKCH	MMP23B	ARPC2	SYTL3	GPR65	ANXA6	CCL4	LGALS…	SKAP1	ARPC5L	PAXX	TMIGD2	PPP1R…	RUNX3	CD244	CYRIB	WIPF1	PLEKH…	IRF1	CFL1	OSTF1	HSH2D
CELLMARKERMSIGDB_HAY_BONE_MARROW_PRE_DEND…																													
CELLMARKERMSIGDB_HAY_BONE_MARROW_PRO_B	NUF2	PPP1CA	KIF15	C21orf…	RNASEH…	SRP9	HNRNPA3	RAD21	CKAP2L	E2F1	YWHAQ	TMPO	CENPN	HNRNP…	BUB3	TIFA	CDCA7	CDKN2A	KIF11	NDC80	CEP55	KNL1	TACC3	SAE1	FAIM	MIS18…	PPIA	CENPA	FBXO5
CELLMARKERMSIGDB_HAY_BONE_MARROW_STROMAL	PGF	SOCS3	MMP2	KITLG	ANGPTL2	OSMR	C11orf…	PPIC	AMOTL2	DENND…	FBN1	LAMA4	CFD	ITIH5	SLC7A…	IGFBP7	ID4	CYP1B1	GPX8	EFEMP1	ISLR	ABCA8	LIFR	ERRFI1	PCDH18	RAI14	DEPP1	BMPER	SVEP1
CELLMARKERMSIGDB_HE_LIM_SUN_FETAL_LUNG_C0…	PLPP2	KLHL23	RBPMS2	MEIS2	ITGA5	FBXO22	NRG1	JPH2	BACE2	FGF7	KIAA12…	MTCL1	ATL2	CXXC4	FOXF2	KRT18	PRICKL…	NEDD4L	ROGDI	CRABP2	SULF2	GFRA1	SLC38…	HHIP-…	NCAM1	PHLDB2	ST6GA…	CYRIB	CDH2
CELLMARKERMSIGDB_HE_LIM_SUN_FETAL_LUNG_C0…																													
CELLMARKERMSIGDB_HE_LIM_SUN_FETAL_LUNG_C0…	HNRNPA…	H2AC14	FBXO5	ZNF93	TMEFF1	SPC25	CCNB1																						
CELLMARKERMSIGDB_HE_LIM_SUN_FETAL_LUNG_C0…	SCG5	PAWR	SERPINB9	GRB7	TMEM10…	RSPO3	HOXA4	MIEN1	AFDN-…	FGF9	CXXC5	PLEKH…	LGALS8	RICTOR	HMGA2	BEX1	PRKAR1B	HSBP1L1	HACD1	NINJ1	TMEM1…	FLNC	C16or…	LRRN4	SIGIRR	PDLIM4	HVCN1	DHCR7	SPOCK2
CELLMARKERMSIGDB_HE_LIM_SUN_FETAL_LUNG_C0…																													
CELLMARKERMSIGDB_HE_LIM_SUN_FETAL_LUNG_C0…																													
CELLMARKERMSIGDB_HE_LIM_SUN_FETAL_LUNG_C0…	PLAAT4	GFPT2	CXADR	MEDAG	DPP4	LGALS8	UCHL1	FRAS1	LRRN4	REC8	ASS1	IGFBP2	CFI	FKBP11	SLC16…	RSPO1	BICDL1	COBLL1	SCG5	LTC4S	CDON	PDGFRL	PLAAT3	GCA	COL9A3	NPR1	AGRN	LINC0…	MEGF6
CELLMARKERMSIGDB_HE_LIM_SUN_FETAL_LUNG_C0…	TRIO	UBE2C	OSR2	B3GALT6	GINS2	PEG3	HELLS	TNFAI…	FBXO5	PDLIM4	PTCH2	MID1	RHNO1	ZFHX4	AEBP2	SERIN…	VOPP1	DBF4	GPSM3	TK1	PDE1A	BMP7	KIF26B	PHLDB2	UBE2T	ITGA4	IQGAP2	TOP2A	MSC-A…
CELLMARKERMSIGDB_HE_LIM_SUN_FETAL_LUNG_C0…																													
CELLMARKERMSIGDB_HE_LIM_SUN_FETAL_LUNG_C0…	CLDN15	CDH3	ADAM15	UNC5B-…	LRRN4	C4A	ENTPD2	PDGFRL	C4B	REEP1	BICDL1	OCIAD2	CAPG	CD9	EFNA1	BEX2	TCEAL2	SEZ6L2	RGS10	APLP1	LTC4S	LINC0…	C19or…	C1R	SPRR2F	VSIR	ADIRF	RSPO1	AQP1
CELLMARKERMSIGDB_HE_LIM_SUN_FETAL_LUNG_C0…																													
CELLMARKERMSIGDB_HE_LIM_SUN_FETAL_LUNG_C1…																													
CELLMARKERMSIGDB_HE_LIM_SUN_FETAL_LUNG_C1…																													
CELLMARKERMSIGDB_HE_LIM_SUN_FETAL_LUNG_C1…																													
CELLMARKERMSIGDB_HE_LIM_SUN_FETAL_LUNG_C1…	ALYREF	NUP205	CTPS1	SRSF12	GTSE1	GYPC	FOXM1	MSANT…	CENPM	ASPM	FGD6	COL26…	CDKN3	BUB1	PKMYT1	CADM4	TTLL5	PIMREG	SGO2	NCAPG	PDE7A	CBS	PHLDA1	LTBP1	KIF11	KIF15	DHFR	KIF20A	GEMIN2
CELLMARKERMSIGDB_HE_LIM_SUN_FETAL_LUNG_C1…	DEF8	RTN1	MATN2	DPP10	BACE1	CACNA…	MS4A8	CLDN5	CNIH2	TOX	ADGRL1	SYT5	ITPR1	GPRIN3	CELF3	SYT11	ABCA5	KCNJ6	STXBP1	VGF	PCSK2	MCF2L	DUSP26	C14or…	RUNX1…	RASD1	RIC3	SRRM4	PCSK1
CELLMARKERMSIGDB_HE_LIM_SUN_FETAL_LUNG_C1…																													
CELLMARKERMSIGDB_HE_LIM_SUN_FETAL_LUNG_C1…																													
CELLMARKERMSIGDB_HE_LIM_SUN_FETAL_LUNG_C1…																													
CELLMARKERMSIGDB_HE_LIM_SUN_FETAL_LUNG_C1…	STEAP4	NECTIN4	ISL1	CXCL2	UPK1B	ATP12A	BMPR1B	PLAAT4	NPDC1	DUSP4	DTX2	CH25H	GRHL1	ABO	SAMHD1	NIBAN1	WNK2	TRPM4	TOX3	ATP13…	SLC44…	SLC1A5	SH2D3A	CAPN8	CFLAR	STEAP1	BICDL2	CPAMD8	ANKRD…
CELLMARKERMSIGDB_HE_LIM_SUN_FETAL_LUNG_C1…	GNB5	SYT11	SCNN1A	CCDC28B	STX1A	GABRB3	NCAM1	SMPD3	PRUNE2	GNG4	ELAPOR1	RIC3	OTULI…	VWA5B2	DLL4	ADGRG1	ELAVL4	SMIM22	KIF19	GALNT…	RAP1G…	LINC0…	TACC2	NBEA	DOP1B	NR0B2	SOBP	MAPK8…	ATP2A3
CELLMARKERMSIGDB_HE_LIM_SUN_FETAL_LUNG_C1…	FAS	GAS6	MYB	AEBP1	CXCR4	TSHZ2	FBXO32	GFRA1	NPDC1	KRT14	GABRG3	IFITM1	SPHK1	SGK1	CD82	NDE1	SEMA6A	COL5A1	SELEN…	GYPC	SIX1	NTN1	PLPP2	CD70	C1QTN…	TMEM1…	LHFPL6	MT1E	CYTH1
CELLMARKERMSIGDB_HE_LIM_SUN_FETAL_LUNG_C1…	NCK2	RHOV	IQGAP2	MYC	FBXO32	SOSTD…	IL17RC	RGCC	ATL1	VCAN	CXCL3	SMIM22	ITGB4	CP	SERTA…	SH3BP5	PRKAR2B	DUSP4	MLYCD	PER2	C4orf…	ADGRG1	LRIG1	PACS1	CFH	PART1	MT1E	MAFB	ZNF217
CELLMARKERMSIGDB_HE_LIM_SUN_FETAL_LUNG_C2…	UPP1	CETP	ABCC5	PPIC	TMEM9	IFI27	APOL6	SUCNR1	FAM78A	TTC38	EPB41L2	ENPP2	SGSH	TIMD4	IFIT1	TGM2	GBP2	PPARG	SIGLE…	TRIM47	PELAT…	PLXNA1	SIL1	VCAM1	FEZ1	IFIT3	TNFRS…	NTMT1	UCKL1
CELLMARKERMSIGDB_HE_LIM_SUN_FETAL_LUNG_C2…	CBFA2T3	LDLRAD4	DIPK2A	LIMA1	FNIP2	RAB29	CYP2S1	INPP5F	ACSL5	CCDC1…	DPP4	AP1G2	PNMA1	TOX	PPP1R…	TOMM34	DLGAP4	TLR10	DGAT2	PLCD1	C1orf…	CD40	HMOX2	BEND5	USP1	SNRNP…	TIFAB	GLTP	OXSR1
CELLMARKERMSIGDB_HE_LIM_SUN_FETAL_LUNG_C2…																													
CELLMARKERMSIGDB_HE_LIM_SUN_FETAL_LUNG_C2…																													
CELLMARKERMSIGDB_HE_LIM_SUN_FETAL_LUNG_C2…																													
CELLMARKERMSIGDB_HE_LIM_SUN_FETAL_LUNG_C2…	ARID5B	LTB	PRSS2	FCMR	FAM30A	CNST	CDK6	TMEM3…	OXLD1	FARSB	MLLT3	VEZT	MYB	AP1G2	SATB1	RPRD1A	LRRC61	ACY3	RCN2	H2BC12	SS18	CD7	CCNB1…	TASP1	KLHDC2	MSH2	SCMH1	MRPL45	SERPI…
CELLMARKERMSIGDB_HE_LIM_SUN_FETAL_LUNG_C2…	F2RL3	RAB37	KIFC3	SLC37A1	MEIS1	PDLIM7	BEX3	UNC13D	CENPT	PTGIR	CD226	TGFB1…	ENDOD1	MAST4	TNIK	DNM3	SPARC	SLA2	MYCT1	PDE5A	RASA3	FHL2	SSX2IP	TIMP3	PKHD1…	PTCRA	EHD3	XPNPE…	CMAS
CELLMARKERMSIGDB_HE_LIM_SUN_FETAL_LUNG_C2…																													
CELLMARKERMSIGDB_HE_LIM_SUN_FETAL_LUNG_C2…	YOD1	ESAM	CMAS	PPP4R3B	H2BC7	LARP4	SORBS1	XK	PELI2	TBPL1	PDE5A	KIFC3	SELP	ABCC4	SPRY1	PBX1	RCHY1	GMPR	H2BC21	GATA2	REXO2	CLK4	SSX2IP	TIMP3	GADD4…	ING5	PCGF5	TRAPP…	CASP3
CELLMARKERMSIGDB_HE_LIM_SUN_FETAL_LUNG_C2…	ZNF296	RDH14	HMGB3	DPF2	SMN2	UHRF1	THEM4	FLT3	PPP1R…	GALNT3	TUBB6	BANK1	SLC20…	PTTG1	SMC6	LIMA1	NIBAN1	LIME1	CMSS1	URI1	MYO1E	EXOSC3	PDLIM1	EBNA1…	TPGS2	NCBP2…	MAP4K1	RRAGC	NOP14
CELLMARKERMSIGDB_HE_LIM_SUN_FETAL_LUNG_C2…																													
CELLMARKERMSIGDB_HE_LIM_SUN_FETAL_LUNG_C2…																													
CELLMARKERMSIGDB_HE_LIM_SUN_FETAL_LUNG_C2…																													
CELLMARKERMSIGDB_HE_LIM_SUN_FETAL_LUNG_C3…	TRPV2	GRID1	SEMA7A	HOXB5	XKR6	IL12A	PSEN2	MATK	MPPED2	DYRK2	TMEM108	PRDM1	F2RL3																
CELLMARKERMSIGDB_HE_LIM_SUN_FETAL_LUNG_C3…	TYMS	ABCB10	HMMR	DHFR	AMMECR1	H1-5	SLC43A1	CDCA3	VRK1	C21or…	KIF20B	NUDT1	TFR2	TRAK2	FBXO5	CKAP2L	HJURP	TRIM10	CENPE	MZT1	FEN1	NUF2	RRM1	AURKA	STEAP3	CENPU	MYBL2	SKA2	DYNC2…
CELLMARKERMSIGDB_HE_LIM_SUN_FETAL_LUNG_C3…	PPIL1	PYCR1	CDC42EP3	STRA6	MRPL10	ITGAE	TRABD	MRRF	TMC6	SDHAF3	RGS19	CYP2S1	RTCA	IER5L	CHN1	CENPW	NTMT1	TMEM17…	GPSM3	PDCL3	TMEM69	GPX8	FAM16…	HDHD5	MYC	TMED3	TMEM51	NDUFA…	CDCA7
CELLMARKERMSIGDB_HE_LIM_SUN_FETAL_LUNG_C3…	OLFML1	GNG12	SLC38A1	MCUB	SLC43A3	FAM17…	IER3	LYRM1	F8	SLC45…	DAPK1	CFI	LAPTM…	HAPLN3	GLS	GPR182	THBS3	OAF	RELN	FZD6	TLE1	AKR1C2	GUCY1…	FOXC2	NTN1	EDNRB	PDIA5	CDC42…	MAGED…
CELLMARKERMSIGDB_HE_LIM_SUN_FETAL_LUNG_C3…	GLIPR2	SGCE	CXADR	RASSF9	CALHM6	PRR5L	RAB11F…	LETR1	PGAP4	ZNF521	PLSCR4	POLE4	NR1H3	NFIA-…	LINC0…	SMYD2	SYNE1	TNFRSF…	SLC9A9	EFNA5	SLC45…	GLT8D2	CASP4	SEMA3A	FAM16…	CLU	MAN1A1	SULF2	IPO11
CELLMARKERMSIGDB_HE_LIM_SUN_FETAL_LUNG_C3…																													
CELLMARKERMSIGDB_HE_LIM_SUN_FETAL_LUNG_C3…	PDGFD	FILIP1L	DIPK2A	SKI	ID4	PCSK5	ABI3BP	NFKBIZ	TAGLN	SGCE	COL9A3	NOMO3	GOLGA3	SEMA3F	CCDC80	LINC0…	GIPC2	ATXN1	MLLT1	SULT1…	CDIP1	PTPN14	PMEPA1	COL5A2	KANK1	MRAP2	MEDAG	DHH	NOMO1
CELLMARKERMSIGDB_HE_LIM_SUN_FETAL_LUNG_C3…	GAB1	TBC1D4	THBS3	CPT1A	EPS8L1	VKORC…	SLC38A1	LAPTM…	RAB11…	LTBP1	PGM5	ST6GA…	PLCD1	NEO1	HSD3B7	SFXN3	IGF1	ASGR1	PPP1R…	TMOD2	DUSP5	CCDC3	RAB3I…	LYSMD2	PEG10	TBX1	HOXD8	SV2C	GALNT2
CELLMARKERMSIGDB_HE_LIM_SUN_FETAL_LUNG_C3…	WFDC1	APOL1	ASS1	TMEM273	IL11RA	EVA1C	XAF1	S1PR4	MIR50…	PALLD	TSLP																		
CELLMARKERMSIGDB_HE_LIM_SUN_FETAL_LUNG_C4…																													
CELLMARKERMSIGDB_HE_LIM_SUN_FETAL_LUNG_C4…																													
CELLMARKERMSIGDB_HE_LIM_SUN_FETAL_LUNG_C4…																													
CELLMARKERMSIGDB_HE_LIM_SUN_FETAL_LUNG_C4…																													
CELLMARKERMSIGDB_HE_LIM_SUN_FETAL_LUNG_C4…	MCM3	FAM111A	NUF2	FANCI	CENPN	TUBG1	CDCA4	HIRIP3	H2AC11	RACGA…	RFC4	PCLAF	H2AC16	HJURP	CMC2	CENPW	RNASEH…	KIF23	ACYP1	CCNB2	MND1	ACOT7	CEP78	SPC24	XPO1	KNL1	KIF11	HELLS	GGH
CELLMARKERMSIGDB_HE_LIM_SUN_FETAL_LUNG_C4…	DHFR	PLK1	CCDC34	HMMR	LIG1	FEN1	NSD2	SAC3D1	CCNB2	CKAP2	KIF15	PHGDH	TMEM1…	SGO1	SGO2	CNTRL	CDCA7	CENPE	SKA2	PCLAF	MIS18…	TK1	RAD51C	HADH	UBE2S	ANXA5	NDC80	TFDP1	CMC2
CELLMARKERMSIGDB_HE_LIM_SUN_FETAL_LUNG_C4…																													

Table S5: Cell state marker gene list

CELLMARKERCAN_ACINAR_CELLS																													
CELLMARKERMSIGDB_HE_LIM_SUN_FETAL_LUNG_C4…																													
CELLMARKERMSIGDB_HE_LIM_SUN_FETAL_LUNG_C4…																													
CELLMARKERMSIGDB_HE_LIM_SUN_FETAL_LUNG_C4…																													
CELLMARKERMSIGDB_HE_LIM_SUN_FETAL_LUNG_C5…																													
CELLMARKERMSIGDB_HE_LIM_SUN_FETAL_LUNG_C5…	BIK	NFATC1	EIF4G1	GM2A	SYNE2	IGHG2	ENTPD1	GPX7	DCPS	NEDD9	NT5DC1	TENT5C	CCL3	CCDC2…	RELB	SEMA7A	GNL3	TNFRSF…	ADAM8	VARS1	UQCC2	SUPT5H	PAICS	SH2B2	HMOX2	ZYX	NOPCH…	IL27RA	MDFIC
CELLMARKERMSIGDB_HE_LIM_SUN_FETAL_LUNG_C5…																													
CELLMARKERMSIGDB_HE_LIM_SUN_FETAL_LUNG_C5…																													
CELLMARKERMSIGDB_HE_LIM_SUN_FETAL_LUNG_C5…	CKAP2	GINS2	C1QTNF4	RRM2	CKAP2L	CCDC8…	MCM2	NCAPH	CCDC1…	FAM11…	DTYMK	RNASE…	NCAPD2	PTTG1	HELLS	TACC3	CCNB2	YEATS4	RMI2	SGO1	LST1	HAUS4	MCM4	FHIT	PAFAH…	CDCA5	KNL1	RASD1	SAC3D1
CELLMARKERMSIGDB_HE_LIM_SUN_FETAL_LUNG_C6…																													
CELLMARKERMSIGDB_HE_LIM_SUN_FETAL_LUNG_C7…																													
CELLMARKERMSIGDB_HE_LIM_SUN_FETAL_LUNG_C7…																													
CELLMARKERMSIGDB_HE_LIM_SUN_FETAL_LUNG_C7…	MARCHF3	SMIM5	FADS3	FGL2	TMEM71	GPR17	SLC16A4	VAMP5	SOCS3	NAALA…	GJC3	AATK	BMF	PTX3	COL15…	RNASE4	DNASE2	PLSCR4	CCL2	CYBRD1	DEPDC7	SEMA3G	PDGFC						
CELLMARKERMSIGDB_HE_LIM_SUN_FETAL_LUNG_C7…																													
CELLMARKERMSIGDB_HE_LIM_SUN_FETAL_LUNG_C7…	CACNB3	SULT4A1	FSTL5	RFTN1	PLPPR3	TBX20	RGS9	EPB41…	LIX1	SLC10…	PEG3	SYT17	CLSTN3	SLC18…	PHYHI…	POU2F2	BBC3	CXXC4	PRUNE2	GRK5	CEND1	RAB3B	TMEM1…	PSD3	DCLK1	APBB3	GPR22	PAK5	ZCCHC…
CELLMARKERMSIGDB_HE_LIM_SUN_FETAL_LUNG_C7…																													
CELLMARKERMSIGDB_HU_FETAL_RETINA_AMACRINE																													
CELLMARKERMSIGDB_HU_FETAL_RETINA_BIPOLAR	MAP4	PCBP3	PCBP4	SYT1	KCNV2	PROM1	MEG3	CCDC8…	NNAT	ROM1	CKB	GPM6A	TFAP2B																
CELLMARKERMSIGDB_HU_FETAL_RETINA_FIBROBLA…	CD99	NDNF	LY6E	SLC26A7	MYLIP	CALD1	PITX1	EPHA3	SYNPO2	HBA1	IRF1	FBLN1	SIX1	IFI16	IGF2	CYP26…	LAMA1	KANK2	H19	RRBP1	GLT8D2	ALDH1…	HBA2	HBG2	DLC1	APOD	FCGRT	CCDC80	ZFP36
CELLMARKERMSIGDB_HU_FETAL_RETINA_HORIZONT…																													
CELLMARKERMSIGDB_HU_FETAL_RETINA_MICROGLIA	HPGDS	GSN	ACY3	ARPC1B	CD83	IRF8	RGS10	HLA-D…	IFNGR1	PLXDC2	CSF3R	CEBPD	CD36	GATM	CD14	SYK	OGFRL1	GAL3ST4	PLEK	TBXAS1	PARVG	LYN	FOLR2	CD300A	HCK	ADORA3	IL6R	OTULI…	SKAP2
CELLMARKERMSIGDB_HU_FETAL_RETINA_MULLER																													
CELLMARKERMSIGDB_HU_FETAL_RETINA_PHOTOREC…																													
CELLMARKERMSIGDB_HU_FETAL_RETINA_RGC	NAGK	PCSK2	SCG5	PPP1R1C	PPP1R1A	SLIT1	DCDC1	RAB33A	RIT2	ATOH7	DCC	CEND1	NKAIN1	DYNC1…	CXADR	JPT1	ACSL4	RBPMS2	IRX3	EDIL3	CCDC1…	MIAT	C3orf…	DYNLT1	ATP6V…	BMERB1	TUBB2B	GNG2	PBX3
CELLMARKERMSIGDB_LAKE_ADULT_KIDNEY_C1_EPI…																													
CELLMARKERMSIGDB_LAKE_ADULT_KIDNEY_C10_TH…	CPD	SLC16A7	CD63	SRP14	TSC22D1	TPT1	PTTG1IP	CRYAB	RPS6	ARPC1B	GHITM	CCT6A	ARFGA…	IVNS1…	DBI	ATP1A1	KNG1	RPS19	CD24	PFKFB3	TMBIM6	PKM	SIM2	RACK1	EIF5	CLDN10	PRDX1	RARRE…	RPLP1
CELLMARKERMSIGDB_LAKE_ADULT_KIDNEY_C12_TH…	GPC3	NUDT4	COX5A	SIM2	SLC25A5	METTL…	NDUFB10	KCTD16	CYC1	NPC2	CYS1	RPL9	FGFR3	GOLM1	GAS6	UQCRQ	EEF1A1	RPS3A	UQCRH	PRDX5	COX7A2	EPHX2	SCD5	NDUFA1	PTGER3	COA3	NDUFS6	ENO1	RPS5
CELLMARKERMSIGDB_LAKE_ADULT_KIDNEY_C18_CO…	ATP6V0…	PEBP1	RPL13	HACD3	KRT18	CA2	AIF1L	CLU	LDHB	MYL12A	RPS3A	EIF1	CD164	PLEKH…	PTBP3	MAL2	RPL11	CD63	RPS14	TACST…	TPM4	RPS18	TPT1	PIK3C…	DYNLT1	TXNIP	S100A…	RPL15	IGFBP7
CELLMARKERMSIGDB_LAKE_ADULT_KIDNEY_C19_CO…	MPP7	RACK1	RPS18	FYB2	GPX1	RPS3A	HLA-B	SLC25…	RPL11	MYL12B	SPTBN2	TXNRD1	S100A…	SOD1	RPL5	ISCU	TMBIM6	HSPB1	RPL31	RPS14	EPS8	FTH1	HSPH1	HSPA4L	CNBP	RPS8	TSPAN1	DSP	SRP14
CELLMARKERMSIGDB_LAKE_ADULT_KIDNEY_C25_EN…																													
CELLMARKERMSIGDB_LAKE_ADULT_KIDNEY_C28_IN…	SCMH1	RPLP1	TCF12	KIAA12…	AKT3	STK3	RASAL2	SBF2	DPYD	UBE2E2	MIR99A…	ROCK1																	
CELLMARKERMSIGDB_LAKE_ADULT_KIDNEY_C29_UN…	FER	ARGLU1	DLG2	TRAPPC9	TTC28	RORA	MYO9A	MACF1	SVIL	GULP1	DPYD	NOX4																	
CELLMARKERMSIGDB_LAKE_ADULT_KIDNEY_C5_PRO…	CNTN1	GUSBP11	NRSN2-A…	INSR	SLC7A7	MIR10…	NSMF	SORCS1	PCDH15	SLC22…	URGCP	SAFB2	RNF220	PAPPA2	AFMID	KMT2B	SLC22A…	ASPSCR1	SLC7A8	MIOX	MACRO…	LINC0…	SLC13…	TENT5A	SMIM2…	CFAP46	RNF21…	LENG8	BRD9
CELLMARKERMSIGDB_MANNO_MIDBRAIN_NEUROTYPE…	TUBA1B	UBB	UBL5	UQCRB	UQCRQ																								
CELLMARKERMSIGDB_MANNO_MIDBRAIN_NEUROTYPE…	CHL1	CHRNA4	CHRNA7	CHST1	CLCN4	CLSTN2	CLUL1	CLVS1	CLVS2	CNGB1	CNTN1	COBL	COLQ	CPEB4	CPNE4	CRHBP	CRMP1	CSMD1	CSRNP3	CTNNA2	CTTNB…	CTXN2	CXADR	DAAM1	DACT1	DCLK1	DCX	DDC	DGKE
CELLMARKERMSIGDB_MANNO_MIDBRAIN_NEUROTYPE…	CDH13	CDH22	CDH3	CDH4	CDK14	CDK5R1	CDKL2	CDKN2D	TMEM1…	CELF3	CELF4	CELF5	CERS6	CHD3	CHD5	CHGA	CHL1	CHN2	CHRNA4	CHRNA7	CHST1	CHSY3	CLASP2	CLCN4	CLVS1	CMIP	CNR1	CNTN1	COMTD1
CELLMARKERMSIGDB_MANNO_MIDBRAIN_NEUROTYPE…	CDON	TMEM121B	CELF3	CELF4	CELF5	CGN	CHD5	CHL1	CHMP2B	CHRNA4	CHRNA6	CHRNA7	CHRNB3	CHST1	CLDN1	CMIP	CNR1	CNTN1	CNTN4	CNTN5	CNTNA…	COL11…	CORO2A	CPEB3	CREG2	CRMP1	CSMD1	CSMD2	CSRNP3
CELLMARKERMSIGDB_MANNO_MIDBRAIN_NEUROTYPE…	BOK	BSG	BTNL9	TOMM40	ERC2-I…	C1orf…	C1orf54	SRARP	C2CD4B	TCIM	CA2	CA5A	CALCRL	CARD8	CASP1	CASP12	CASP3	CASP4	CAST	CAV1	CAV2	CCDC1…	CCDC1…	CCDC68	CCDC8…	CCM2L	CCNL1	CCNY	CD164
CELLMARKERMSIGDB_MANNO_MIDBRAIN_NEUROTYPE…	CASP4	CASP9	CCDC17	CCDC175	CCL2	CCL3	CCL4	CCR1	CCRL2	CD14	CD163	CD274	CD36	CD37	CD4	CD44	CD48	CD53	CD5L	CD68	CD69	CD74	CD83	CD84	CD86	CDH17	CDKN1A	CEBPA	CEBPD
CELLMARKERMSIGDB_MANNO_MIDBRAIN_NEUROTYPE…	CACNA1B	CACNA1C	CACNA1E	CACNA1G	CACNG2	CACNG4	CACNG8	CADM1	CADM2	CALN1	CAMK2B	CAMK2…	CAMSA…	CASZ1	CBLN2	CBLN4	CBX4	CCDC136	CCDC1…	CCDC1…	CCDC39	CCDC8…	CCDC8…	CCSER1	CD2	CD24	CD27-…	CDH4	CDH8
CELLMARKERMSIGDB_MANNO_MIDBRAIN_NEUROTYPE…	TAFA2	CYRIA	TLCD3B	RIPOR2	FGF12	FGF14	FGF9	FHOD3	FMN2	FNIP2	FOXA1	FOXA2	FOXD2	FOXD2…	FOXP2	GABRB3	GABRG2	GAP43	GDAP1…	GLRA2	GNAL	GNG2	GNG3	GP1BB	GPC2	GPM6A	GPR12	GPRIN3	GRIA2
CELLMARKERMSIGDB_MANNO_MIDBRAIN_NEUROTYPE…	CNGB1	CNR1	CNTNAP2	COMTD1	CORO2A	CPEB2	CPEB3	CPEB4	CRMP1	CSRNP3	CXADR	DACT1	DACT3	DCC	DCX	DGKE	DICER1	DLG3	DMTN	DNAJC…	DNAJC6	DNER	DNM3	DOK6	DOT1L	CTSC	DPYSL3	DSCAM	DUSP26
CELLMARKERMSIGDB_MANNO_MIDBRAIN_NEUROTYPE…	CPNE8	CRMP1	CRNDE	CSMD3	CTNNA2	CTXN2	CTXN3	CXADR	CYP4X1	DACH1	DBH	DCX	DGCR6	DMTN	DNAJC6	DNM1	DNM3	DOCK4	DOK4	DOK5	DOK6	DPYSL3	DTD1	DUSP26	ECEL1	EDIL3	EEF1A2	ELAVL2	ELAVL3
CELLMARKERMSIGDB_MANNO_MIDBRAIN_NEUROTYPE…	CMYA5	CNTN1	COL2A1	COL9A1	COL9A3	CPEB1	CPNE2	CREB5	CRTAC1	CRYL1	CSGALN…	CSMD3	CSPG5	CTHRC1	CTNND2	BCLAF3	DBI	DCLK2	DHX32	DNAAF3	DNAH7	DND1	DNER	DNM3	DOCK1	DPP6	DSEL	EDNRB	EFCAB…
CELLMARKERMSIGDB_MANNO_MIDBRAIN_NEUROTYPE…	BCL6	BGN	BTN3A3	C11orf…	C16orf…	EML2-…	C1QTNF1	C1S	SLC66…	C9orf…	CA2	CA4	CACNA…	CACNB4	CADM4	CALCRL	CALD1	CALU	CAMK2D	CASP4	CAST	CAV2	CCDC17	CCDC3	CCDC8	CCL2	CCL8	CCM2L	CD163…
CELLMARKERMSIGDB_MANNO_MIDBRAIN_NEUROTYPE…	DSN1	DTX4	ECT2	EEF1D	ENOSF1	EPHB2	ESCO2	PIMREG	DIS3L2	FAM72A	FAM83D	FANCB	FANCI	FAT3	FBLN2	FBXO5	FGFR3	FNDC3B	FOXA2	FOXM1	FZD1	FZD2	GBX2	GINS1	GINS2	GMNN	GNG5	GPSM2	GSTP1
CELLMARKERMSIGDB_MANNO_MIDBRAIN_NEUROTYPE…	DBF4	DDC	DEK	DEPDC1	DEPDC1B	DHFR	DLGAP5	DMRTA2	DOK1	DSC2	DSG2	ECT2	EEF1D	EFNA5	EFS	ELOVL2	EN2	ENKUR	EPHB2	ESCO2	EZH2	GASK1B	PIMREG	DIS3L2	FAM72A	FAM83D	LRATD2	FANCB	FANCI
CELLMARKERMSIGDB_MANNO_MIDBRAIN_NEUROTYPE…	CRYZ	CSRP2	CSTF3-DT	CTNNAL1	CTNNBL1	CCN1	DANCR	DBF4	DCC	DDC	DEK	DEPDC1	DEPDC…	DHFR	DLGAP5	DMRTA2	DSC2	DSG2	DSN1	ECT2	EEF1D	EFS	EN2	EPHB2	EPHB4	ERF	ESCO2	EXOSC8	GASK1B
CELLMARKERMSIGDB_MANNO_MIDBRAIN_NEUROTYPE…	CCN2	AGAP12P	CTHRC1	CCN1	CYTL1	CYYR1	DCC	DDC	DDIT4	DEPDC…	DMRTA2	DNAH7	DSC2	DSG2	CLXN	EFCC1	EFEMP2	EFNA5	EN2	ENO1	ENPP2	ERF	FAM13A	FAM18…	GASK1B	CIBAR2	FANCI	FAT1	FAT3
CELLMARKERMSIGDB_MANNO_MIDBRAIN_NEUROTYPE…	DCDC1	DCN	DDAH1	DEPTOR	DMRTA2	DNAAF1	DNAH6	DNAH7	DNAJB4	DOK5	DSG2	DTX4	ECEL1	ECHDC2	EEPD1	CLXN	EFHC2	EFHD2	ELN	ELOVL1	ENKUR	EPHB4	ERBB4	ERF	ERMN	EVI5	FABP7	FAM18…	GASK1B
CELLMARKERMSIGDB_MANNO_MIDBRAIN_NEUROTYPE…	CFAP210	CCDC175	CCDC39	CFAP73	CCL2	CCND2…	CD44	CD81	CDC42…	CDH13	CDHR1	CDR1	CELSR1	CETN2	CFAP1…	CFAP44	CFAP45	CFAP52	CHST3	CHST6	CHST9	CLCN3	CLU	CMTM5	CNN2	COL11…	COL27…	COL4A5	COLQ
CELLMARKERMSIGDB_MANNO_MIDBRAIN_NEUROTYPE…	CREB5	CRYAB	CSGALNA…	CSMD2	CSMD3	CSPG5	CSPP1	CST3	CTDSP1	CTNND2	BCLAF3	CYP26…	CCN1	DBI	DBX2	DCLK2	DDAH1	DEPDC1	DNAAF1	DNAH6	DNAH7	DNAJB1	DPF3	DPP6	DSC3	DSEL	DTX4	EDNRB	EEPD1
CELLMARKERMSIGDB_MANNO_MIDBRAIN_NEUROTYPE…	DOCK1	DPF3	DPP6	DPP7	DTNA	EDNRB	EEPD1	EFHD2	EGFR	EGLN3	EGR1	ELN	EPHA3	ERBB3	ERF	ETV1	ETV4	ETV5	F3	FABP5	FABP7	FAM18…	INKA2	FAM81A	LRATD2	FAM89A	FANCB	FANCI	FBLN2
CELLMARKERMSIGDB_MANNO_MIDBRAIN_NEUROTYPE…	CCDC146	CCDC17	CFAP210	CCDC39	CCDC8	CD19	CD36	CD44	CD47	CDH13	CDH4	CDK2A…	CDO1	CDR1	CELSR1	CERKL	CETN2	CFAP126	CFAP44	CFAP45	CFAP52	CFC1	CHPF	CHST3	CHST6	CLU	CMTM4	CMTM8	CNPY4
CELLMARKERMSIGDB_MANNO_MIDBRAIN_NEUROTYPE…	DUSP26	DUSP4	EBF3	EDIL3	ELAVL2	ELAVL3	ELAVL4	ELOVL4	EMX2	EMX2OS	ENC1	ENOX1	EPB41	EPB41…	ESRRG	ETFB	FAM117B	TMEM18…	TAFA2	TLCD3B	FAXC	FBLL1	FBXL16	FGF12	FGF14	FHOD3	FNIP2	FOXA2	FOXP2
CELLMARKERMSIGDB_MANNO_MIDBRAIN_NEUROTYPE…	CHD5	CHGA	CHGB	CHODL	CHRNA4	CHST1	CLCN4	CLVS1	CNGB1	CNIH2	CNTN1	CNTN4	CNTNA…	COL5A2	COMTD1	CPEB3	CPNE4	CPNE5	CPNE7	CREG2	CRMP1	CRTAC1	CSRNP3	CTHRC1	CTXN2	CYP4X1	DACT3	DCX	DDC
CELLMARKERMSIGDB_MENON_FETAL_KIDNEY_0_CAP…	DACH1	RPS19P1	ENAH	RPS4XP6	PTMS	SPEN	GABPB1…	BDP1	TOP2B	SMC1A	BRD3	SMC3	NUDT3	HMGB2	PPM1G	DDX24													
CELLMARKERMSIGDB_MENON_FETAL_KIDNEY_10_IM…	TOP1	ATP5F1E	IQGAP1	RPS15A	S100A6	CFL1	RPL27A	TUBA1B	RPL12																				
CELLMARKERMSIGDB_MENON_FETAL_KIDNEY_2_NEP…																													
CELLMARKERMSIGDB_MENON_FETAL_KIDNEY_3_STR…	RPL28	NKTR	TUBA1A	ITGB1	RPL24P8	PPP1R…	TOP2B																						
CELLMARKERMSIGDB_MENON_FETAL_KIDNEY_6_COL…	B2M	SPINT2	PTPN13	MYL12A	PDHA1	MGST3	EIF1	HDAC1	CHD3	PAX8	MAP4	COA3	METAP2	CLTC	LRPAP1	ECHDC2	ARPC2	RTN4	TCEAL9	MRPL33									
CELLMARKERMSIGDB_MURARO_PANCREAS_ACINAR_C…	SDC4	GPX2	BIRC3	TPST2	TMC5	SGK1	STEAP4	SAT1	CBS	GATA4	SOD2	KCNQ1	MECOM	TRIB2	CPA4	CYB5A	NR5A2	LCN2	MTUS1	UGDH	CD24	ZFP36…	TACST…	LDHB	YBX3	IMPA2	DUSP4	NTN4	CXCL3
CELLMARKERMSIGDB_MURARO_PANCREAS_ALPHA_CE…	PRPS1	SESN2	SELENOI	NAXE	SNAP25	ECH1	NRCAM	SGSM2	KCNK3	MYEF2	CD164	VPS41	H1-10	PKM	NDUFA1	PTS	FKBP2	SMDT1	UBB	EPHX2	NAV1	ZBTB41	FBXW7	VPS26B	SLC29…	NAA38	ASPH	TMEM1…	SEM1
CELLMARKERMSIGDB_MURARO_PANCREAS_BETA_CELL	WNT4	GNPDA1	WSCD2	NECTIN3	MAP1B	RASD1	RASGRF1	CYP2U1	RAPH1	RAP1G…	PRSS23	ATRNL1	KIF5C	DHRS7	ASAP1	TFCP2…	SLC2A13	CADM1	FAM22…	TBC1D4	SNHG7	DHRS2	GSN	CCNI	RAB15	CHL1	UBE2Q…	MXRA7	INSM1
CELLMARKERMSIGDB_MURARO_PANCREAS_ENDOTHEL…	RHOJ	CYP1A1	GNG11	GMFG	BCL6B	SPRY4	PDE2A	DOCK4	TEK	SHE	CD36	STING1	TMEM2…	PLEKH…	GIMAP4	FAM43A	SOCS3	FZD4	STC1	CDH5	ADAMT…	MEF2C	ARHGD…	RGS5	SEMA3G	CLIC2	SERPI…	WWTR1	LRRC32
CELLMARKERMSIGDB_MURARO_PANCREAS_EPSILON_…																													
CELLMARKERMSIGDB_MURARO_PANCREAS_MESENCHY…	TGFBI	NREP	HTRA3	AEBP1	SPOCK1	TPM2	ANTXR1	CYP1B1	GFPT2	LIF	EMP1	UNC5B	PDGFRA	CCDC80	F2R	STC1	SLC1A5	PAG1	VIM	SLC7A…	TBX3	CTSC	GPX8	POSTN	SPON1	ADAMT…	LHFPL6	ANO1	MMP11
CELLMARKERMSIGDB_RUBENSTEIN_SKELETAL_MUSC…	NUP214	C4orf48	HCST	SMAP2	PLAC8	TYMP	BID	FGL2	NPC2	CORO1A	FTL	LIMD2	LCP1	LAPTM5	RGS10	IER3	FTH1	FCGR3A	SRGN	HCLS1	TKT	LY96	SMCO4	NFKBIA	SOD2	CEBPB	CYRIB	FOS	NR4A1
CELLMARKERMSIGDB_RUBENSTEIN_SKELETAL_MUSC…	RACK1	MYC	RPL24	CD63	HSP90A…	RPL15	RPS27	RPL37	RSL1D1	CCN1	EIF3L	CLU	RBP1	RPL4	RPL18	RARRE…	ZFAS1	SNRPE	RPL31	RPL30	RPL8	SIRT2	RPL7A	RPS19	S100A4	LAMP2	RPL14	RPS15	SBDS
CELLMARKERMSIGDB_RUBENSTEIN_SKELETAL_MUSC…	FLNA	PTP4A3	PPP1R12A	PALLD	ARID5A	FAM16…	CSRP1	UBA2	NOTCH3	ARHGE…	MIR145	LPP	ACTN1	IGFBP5	MT-CO1	GADD4…	IRAG1	ILK	RBPMS	MGST3	ARPC1A	MFGE8	ACTB	MYL6	PPP1R…	COX7A2	S100A4	CALM2	RGS5
CELLMARKERMSIGDB_TRAVAGLINI_LUNG_ALVEOLAR…	LIMS2	CADM1	EHD2	PLEKHO1	NDNF	SAMD4A	CD55	TNFRS…	CFL2	PDLIM2	VEGFA	ACTB	CCBE1	HPCAL1	MAP2	KLK7	SLC6A4	MATN3	EFNA1	DST	GRK5	HOPX	LIMCH1	RAB11…	GAS6	PRSS8	FN1	ABCA8	EPB41…
CELLMARKERMSIGDB_TRAVAGLINI_LUNG_BASOPHIL…	DUSP10	PTGS1	NTM	AREG	FTH1	METTL…	CNRIP1	MIR23…	DDX5	CD63	AP1S3	JUND	SKIL	ICAM1	DUSP6	SLC26…	ACSL4	MITF	RHBDD2	RHOH	CAPG	BATF	GCSAML	VIM	SQSTM1	RENBP	FTH1P3	TPM4	SOCS1
CELLMARKERMSIGDB_TRAVAGLINI_LUNG_CAPILLAR…																													
CELLMARKERMSIGDB_TRAVAGLINI_LUNG_CAPILLAR…	POLR2F	SERINC1	PSME2	SH3BGRL	CHMP5	ATP5PD	SEC61G	BUD31	BBLN	PEA15	UBE2L3	CSTB	GPX4	RAP1A	ARL2	HPCAL1	HSBP1	PRDX5	SYF2	VAMP8	EIF5B	UQCRB	TPM3	OPTN	COX6B1	CARD16	NDUFA…	MRPL33	RPS27L
CELLMARKERMSIGDB_TRAVAGLINI_LUNG_CD4_MEMO…																													
CELLMARKERMSIGDB_TRAVAGLINI_LUNG_EREG_DEN…	MPHOSP…	IFI30	FCN1	ATP5F1E	MCL1	CSF2RA	IFNGR2	FGD4	SOCS3	FCER1G	MAFB	SLC25…	MAP2K1	CPVL	USP53	STAB1	SEMA6B	NECTIN2	HLA-D…	TNFRS…	CHMP1B	GK	SOD2	CSF1R	CD74	SERPI…	SRGN	PLSCR1	LITAF
CELLMARKERMSIGDB_TRAVAGLINI_LUNG_IGSF21_D…																													
CELLMARKERMSIGDB_TRAVAGLINI_LUNG_MESOTHEL…	TMEM91	KCNK1	CPXM1	TSTD1	MFSD2A	EIF1AY	DKK1	ABCA1	C3	CCNYL1	GOLM1	SGK1	CD200	TMEM1…	NSG1	ACSL4	PLOD2	ST3GAL5	NQO1	CCDC80	SLC20…	CYRIB	TIMM1…	TRAPP…	IFI27	NOP16	ARPC5L	KLK8	CRB2
CELLMARKERMSIGDB_TRAVAGLINI_LUNG_MYOFIBRO…																													
CELLMARKERMSIGDB_TRAVAGLINI_LUNG_NATURAL_…																													
CELLMARKERMSIGDB_TRAVAGLINI_LUNG_NEUTROPH…	FTH1	SPI1	TNFRSF1…	DUSP1	ACTN1	EMP3	DGAT2	ANPEP	R3HDM4	SLC2A3	RNF24	CHI3L1	CATIP	IL2RG	MZT2B	ARRB2	TMSB4X…	ATG2A	NEAT1	IGF2R	PELI1	SAT1	NDUFA1	STEAP4	NCF4	VASP	SOD2	SMAP2	CEBPD
CELLMARKERMSIGDB_TRAVAGLINI_LUNG_OLR1_CLA…	FCAR	MIR4435…	TREM1	PNP	MMP19	LUCAT1	THBS1	CLEC5A	FPR2	LCP2	TPM4	SERPI…	IRAK2	EHD1	MCEMP1	LHFPL2	FCER1G	APOBEC…	NFKBIA	RBM47	LILRB2	SLC7A5	ANXA5	EIF1	ADAM19	VASP	SEC61G	POMP	EREG
CELLMARKERMSIGDB_TRAVAGLINI_LUNG_PLATELET…	H2BC12	ENKUR	CALM3	MYLK	ARHGAP6	VIM-A…	PF4V1	TLK1	TUBA8	VCL	OST4	SNAP23	GFI1B	NAP1L1	TRIM58	ILK	EIF2AK1	HEMGN	LY6G6F	CAPZA2	ALOX12	PKM	R3HDM4	TPM1	INF2	CARD19	H2BC21	HEXIM2	TSC22…
CELLMARKERMSIGDB_TRAVAGLINI_LUNG_PROLIFER…	CENPN	HMGN2	SMC2	NRM	CENPE	NUDT1	PLK1	CDT1	PCNA	NMU	TTK	CDC45	AURKA	MCM7	DIAPH3	CKAP2	KRT15	SGO2	NCAPG	RAD51	RMI2	KIF20B	RPL39L	DUT	GINS2	BRCA2	RACGA…	TCF19	H2AX
CELLMARKERMSIGDB_TRAVAGLINI_LUNG_PROLIFER…	ANLN	HMGB3	TMEM106C	HMGB1	C1QA	FABP5	DLGAP5	DHFR	CDCA3	CDCA5	ASPM	MRC1	PBK	FANCI	TUBB4B	KIF11	SGMS2	CCL23	KIF2C	DTYMK	C1QC	BRCA2	TUBA1C	CDC20	PPARG	SKA3	RNASE…	OLR1	CCNB1
CELLMARKERMSIGDB_TRAVAGLINI_LUNG_PROXIMAL…	ALOX15	RPL29	INKA1	SNHG5	TPM2	FMO2	EEF1D	IGFBP2	PTN	LARP6	UQCRH	SULT2…	PITX1	RPS15	ATP5F…	RPL12	OAF	IMPDH2	TRIM29	TNFSF…	AVPI1	RPS5	RPL26	EYA2	KCTD1	EEF1A1	EIF3E	ZNF703	RPL13
CELLMARKERMSIGDB_TRAVAGLINI_LUNG_SEROUS_C…	WFDC21P	PHGDH	SH3BGRL2	CDC42E…	PYCR1	NUCB2	SMCO4	HSD17…	MANSC1	GPT2	PPP1R1…	FUOM	ELAPO…	CAPN12	PHB1	ALDH1…	ATP2A3	BAZ2B-…	XBP1	AK4	PDCD4	FAM10…							
CELLMARKERMSIGDB_TRAVAGLINI_LUNG_SIGNALIN…																													
CELLMARKERMSIGDB_TRAVAGLINI_LUNG_TREM2_DE…	SLCO2B1	CPM	HLA-DRA	RARRES1	MS4A7	HLA-D…	GPX4	FABP5	LYZ	RAB13	HLA-DR…	HLA-D…	S100A…	HSD11…	RAB31	LAMP2	NCEH1	HLA-DOA	CTSK	LGALS…	MS4A4A	NPL	ADAMD…	AGPAT2	ITGAM	PILRA	HNMT	LHFPL2	AXL
CELLMARKERMSIGDB_TRAVAGLINI_LUNG_VASCULAR…																													
CELLMARKERMSIGDB_TRAVAGLINI_LUNG_VEIN_CELL																													
CELLMARKERMSIGDB_VANGURP_PANCREATIC_ALPHA…																													
CELLMARKERMSIGDB_VANGURP_PANCREATIC_DELTA…	VMP1																												
CELLMARKERMSIGDB_VANGURP_PANCREATIC_GAMMA…																													
CELLMARKERMSIGDB_ZHENG_CORD_BLOOD_C1_PUTA…	PPBP	PPIF	PRKAR2B	PROS1	RAB27B	RBPMS2	RGS18	RUFY1	CAVIN2	SELP	SLC44A1	SLFN14	SPTB	STOM	TESC	THBS1	TMEM40	TMSB4X	TPM1	TREML1	TSPAN…	TUBB1	VCL	VWF					
CELLMARKERMSIGDB_ZHENG_CORD_BLOOD_C10_MUL…	RBM17	RBM38	RERE	RNASET2	S100Z	SASH3	SATB1	SCN3A	SETBP1	SH2D3C	SH3TC1	SLC2A5	SLC9A…	SPINK2	SPNS3	STARD9	SYK	TNFAIP2	TRAF3…	TRBC2	TTC3	TYROBP	USP36						
CELLMARKERMSIGDB_ZHENG_CORD_BLOOD_C2_PUTA…	SDF2L1	SEC24D	SLC25A3	SLC2A1	SLC40A1	SLC6A6	SMIM1	SOX4	SPTY2…	STAM	STAT5A	TES	TFR2	TFRC	TIMM1…	TMSB10	TOB2	TPSAB1	TRIB2	TSPYL2	VASP	VIM							
CELLMARKERMSIGDB_ZHENG_CORD_BLOOD_C4_PUTA…	SKIL	SLA	SLC38A2	SLC39A8	SLC40A1	SNX5	SPTY2D1	ITPRI…	STK4	TAF1D	TEC	TESPA1	TNIK	SMIM27	TSEN54	TSPYL2	YARS1	ZBTB16	ZBTB20	ZEB2	ZFAS1	ZMYND8	ZNF711						
CELLMARKERMSIGDB_ZHENG_CORD_BLOOD_C5_SIMI…	FOS	CD74	HOPX	H4C5	AREG	GADD4…	H1-3	ID2	ATF3	SEZ6L2	CRHBP	SELEN…	APOL3	JAML	LRBA	EIF4A2	BTG2	INSIG1	GADD4…	GNG11	SERTA…	OXT	SPINK2						
CELLMARKERMSIGDB_ZHENG_CORD_BLOOD_C6_HSC_…	CAVIN2	SELENOK	SLC2A3	SMCHD1	SNX9	SOCS2	SPP1	SRGN	STAT1	TACC1	TCF4	TFPI	THEMI…	TNFRS…	TPM2	TSC22…	TSC22D3	TSPYL2	TUBA4A	USP36	WDR45B	ZFP36…	ZNF331						
CELLMARKERMSIGDB_ZHENG_CORD_BLOOD_C7_PUTA…	KPNB1	BRCA2	MT-RNR2	VPS35	BTF3	DYNLT1	HSPA5	FLT3	TUBA1B	ZRANB2	CCDC14	RFLNB	HIF1A	PSMA6	MT-AT…	GTF3A	TCERG1	CASP4	SET	ENO1	CSGAL…	ARID4A							
CELLMARKERMSIGDB_ZHENG_CORD_BLOOD_C8_PUTA…	LDLRAD2	SPNS3	ATP5MC2	CD48	IGHM	EGFL7	FLT3	ATP5F…	ADA	IGFBP7	MACROH…	HLA-C	NAP1L1	RUNX1	CALHM6	ERLIN1	EIF3K	HNRNPA1	LMNTD1	RNASE…	SNRPD2	ARHGA…	MT-ND4						
CELLMARKERMSIGDB_ZHONG_PFC_C1_ASTROCYTE																													
CELLMARKERMSIGDB_ZHONG_PFC_C1_DLX5_POS_IN…																													
CELLMARKERMSIGDB_ZHONG_PFC_C2_ASCL1_POS_A…																													

Table S5: Cell state marker gene list

CELLMARKERCAN_ACINAR_CELLS																													
CELLMARKERMSIGDB_ZHONG_PFC_C2_UNKNOWN_NPC	UBE2C	ZNF649																											
CELLMARKERMSIGDB_ZHONG_PFC_C3_ASTROCYTE	PLXNA3	SCN3A	LINC029…	SRRM4	ZFTA	DLX1	NGEF	DNM3	TPTE2…	RNF112	VSTM2B…	MYO16	CCBE1	ZFP90	TMEM1…	RGS11	SYBU	ZNF37A	THRB	MATCA…	CXADR	JAG2	CDC42…	GAB2	ZNF78…	SETX	UNC79	FBXO41	MGRN1
CELLMARKERMSIGDB_ZHONG_PFC_C3_HOPX_POS_OPC																													
CELLMARKERMSIGDB_ZHONG_PFC_C3_MICROGLIA	DLX2	TUBA1A	WASF1	CYP27C1	PHACTR3	GDA	ENHO	CAMKV	PKD1	LRRC4C	IVNS1A…	GPR12	MT3	LMO4	DTX1	BHLHE…	RAB9B	MYT1L	CADM1	GUCY1…	WDR59	RBM4B	ARL8A	OPA1	DLG3	ATCAY	CLVS2	PTPN5	TCF4
CELLMARKERMSIGDB_ZHONG_PFC_C3_UNKNOWN_INP																													
CELLMARKERMSIGDB_ZHONG_PFC_C4_UNKNOWN_INP	SUPT16H	SVIL	SYP	TAGLN3	TCP10L	TIMM50	TTF1	UBAP2	UBE2I	ZBED8	ZFP82	ZIC2	ZNF254	ZNF43	ZNF605														
CELLMARKERMSIGDB_ZHONG_PFC_C6_PLXNA4_POS_…																													
CELLMARKERMSIGDB_ZHONG_PFC_C7_ORG_UNDERGO…																													
CELLMARKERMSIGDB_ZHONG_PFC_C8_ORG_PROLIFE…																													
CELLMARKERMSIGDB_ZHONG_PFC_C8_UNKNOWN_NEU…	SPOCK1	SRM	STK32B	SYNGR3	SYT4	SYT7	UNC13A	USP32…	USP32…	VSTM2L																			
CELLMARKERMSIGDB_ZHONG_PFC_C9_ORG_OTHER	TAGLN2	TKTL1	TMEM132B	TMEM9B	TNC	TSPAN7	TSPO	TTYH1	TUBB2A																				
CELLMARKERMSIGDB_ZHONG_PFC_HES1_POS_C1_NPC																													
CELLMARKERMSIGDB_ZHONG_PFC_MAJOR_TYPES_AS…	GLUD1	PLTP	ADGRG1	SLC9A3…	SLC39A…	TRIM47	PEA15	LRRC3B	DTNA	LRIG1	NKAIN4	VEPH1	PTPRZ1	BMP7	LITAF	ITM2C	DBI	SFXN5	DBX2	CSPG5	CPNE5	GPM6B	TIMP3	SLC38…	GPR37…	KCNJ10	EFHD2	TCIM	LGI1
CELLMARKERMSIGDB_ZHONG_PFC_MAJOR_TYPES_EX…																													
CELLMARKERMSIGDB_ZHONG_PFC_MAJOR_TYPES_MI…	CRYBB1	NINJ1	LCP1	LILRB4	TMIGD3	CPVL	HCK	HCST	FCGR2A	ADORA3	MIR23A…	IFNGR1	IRF8	LPAR6	LPAR5	ACY3	CEBPD	SFMBT2	OLR1	SIGLE…	ABCG2	FCGR1…	BTG2	CSF3R	CREG1	IL6R	CD37	MKNK1	GADD4…
CELLMARKERMSIGDB_ZHONG_PFC_MAJOR_TYPES_OPC	ANXA5	TSPAN7	GATM	B2M	TTYH1	PTN	PLPP3	GPM6B	NXPH1	GNG7	ALCAM	PSAT1	SLC35…	CD63	SEZ6L	SCD5	LSAMP												

CELLMARKERCAN_ACINAR_CELLS																														
CELLMARKERCAN_ADIPOCYTE_PROGENITOR_CELLS																														
CELLMARKERCAN_ADRENERGIC_NEURONS																														
CELLMARKERCAN_AIRWAY_EPITHELIAL_CELLS																														
CELLMARKERCAN_ALPHA_CELLS																														
CELLMARKERCAN_ALVEOLAR_MACROPHAGES																														
CELLMARKERCAN_ASTROCYTES																														
CELLMARKERCAN_B_CELLS																														
CELLMARKERCAN_B_CELLS_MEMORY																														
CELLMARKERCAN_B_CELLS_NAIVE																														
CELLMARKERCAN_BASOPHILS																														
CELLMARKERCAN_BERGMANN_GLIA																														
CELLMARKERCAN_BETA_CELLS																														
CELLMARKERCAN_CAJAL_RETZIUS_CELLS																														
CELLMARKERCAN_CARDIAC_STEM_AND_PRECURSOR_…																														
CELLMARKERCAN_CARDIOMYOCYTES																														
CELLMARKERCAN_CHOLANGIOCYTES																														
CELLMARKERCAN_CHOLINERGIC_NEURONS																														
CELLMARKERCAN_CHONDROCYTES																														
CELLMARKERCAN_CHOROID_PLEXUS_CELLS																														
CELLMARKERCAN_CILIATED_CELLS																														
CELLMARKERCAN_DELTA_CELLS																														
CELLMARKERCAN_DOPAMINERGIC_NEURONS																														
CELLMARKERCAN_DUCTAL_CELLS																														
CELLMARKERCAN_EMBRYONIC_STEM_CELLS																														
CELLMARKERCAN_ENTERIC_GLIA_CELLS																														
CELLMARKERCAN_ENTERIC_NEURONS																														
CELLMARKERCAN_ENTEROCYTES																														
CELLMARKERCAN_ENTEROENDOCRINE_CELLS																														
CELLMARKERCAN_EPENDYMAL_CELLS																														
CELLMARKERCAN_EPSILON_CELLS																														
CELLMARKERCAN_FOLLICULAR_CELLS																														
CELLMARKERCAN_FOVEOLAR_CELLS																														
CELLMARKERCAN_GAMMA_DELTA_T_CELLS																														
CELLMARKERCAN_GERM_CELLS																														
CELLMARKERCAN_GLUTAMINERGIC_NEURONS																														
CELLMARKERCAN_GRANULOSA_CELLS																														
CELLMARKERCAN_HEMATOPOIETIC_STEM_CELLS																														
CELLMARKERCAN_IMMATURE_NEURONS																														
CELLMARKERCAN_INTERNEURONS																														
CELLMARKERCAN_IONOCYTES																														
CELLMARKERCAN_KERATINOCYTES																														
CELLMARKERCAN_KUPFFER_CELLS																														
CELLMARKERCAN_LEYDIG_CELLS																														
CELLMARKERCAN_LOOP_OF_HENLE_CELLS																														
CELLMARKERCAN_LUMINAL_EPITHELIAL_CELLS																														
CELLMARKERCAN_LUTEAL_CELLS																														
CELLMARKERCAN_MEGAKARYOCYTES																														
CELLMARKERCAN_MELANOCYTES																														
CELLMARKERCAN_MESANGIAL_CELLS																														
CELLMARKERCAN_MESOTHELIAL_CELLS																														
CELLMARKERCAN_MICROFOLD_CELLS																														
CELLMARKERCAN_MICROGLIA																														
CELLMARKERCAN_MONOCYTES																														
CELLMARKERCAN_MOTOR_NEURONS																														
CELLMARKERCAN_MULLER_CELLS																														
CELLMARKERCAN_MYELOID_DERIVED_SUPPRESSOR_…																														
CELLMARKERCAN_MYOBLASTS																														
CELLMARKERCAN_MYOEPITHELIAL_CELLS																														
CELLMARKERCAN_MYOFIBROBLASTS																														
CELLMARKERCAN_NEUROBLASTS																														
CELLMARKERCAN_NEUROENDOCRINE_CELLS																														
CELLMARKERCAN_NEURONS																														
CELLMARKERCAN_NEUTROPHILS																														
CELLMARKERCAN_NK_CELLS																														
CELLMARKERCAN_NORADRENERGIC_NEURONS																														
CELLMARKERCAN_NUOCYTES																														
CELLMARKERCAN_OLIGODENDROCYTES																														
CELLMARKERCAN_OSTEOBLASTS																														
CELLMARKERCAN_OSTEOCLAST_PRECURSOR_CELLS																														
CELLMARKERCAN_OSTEOCLASTS																														
CELLMARKERCAN_OSTEOCYTES																														
CELLMARKERCAN_PANETH_CELLS																														
CELLMARKERCAN_PARATHYROID_CHIEF_CELLS																														
CELLMARKERCAN_PERICYTES																														
CELLMARKERCAN_PERITUBULAR_MYOID_CELLS																														
CELLMARKERCAN_PHOTORECEPTOR_CELLS																														
CELLMARKERCAN_PLASMA_CELLS																														
CELLMARKERCAN_PLASMACYTOID_DENDRITIC_CELLS																														
CELLMARKERCAN_PODOCYTES																														
CELLMARKERCAN_PROXIMAL_TUBULE_CELLS																														
CELLMARKERCAN_PULMONARY_ALVEOLAR_TYPE_I_C…																														
CELLMARKERCAN_PULMONARY_ALVEOLAR_TYPE_II_…																														
CELLMARKERCAN_PURKINJE_NEURONS																														
CELLMARKERCAN_RETICULOCYTES																														
CELLMARKERCAN_RETINAL_GANGLION_CELLS																														
CELLMARKERCAN_RETINAL_PROGENITOR_CELLS																														
CELLMARKERCAN_SATELLITE_CELLS																														
CELLMARKERCAN_SCHWANN_CELLS																														
CELLMARKERCAN_SEROTONERGIC_NEURONS																														
CELLMARKERCAN_SERTOLI_CELLS																														
CELLMARKERCAN_SMOOTH_MUSCLE_CELLS																														
CELLMARKERCAN_STROMAL_CELLS																														
CELLMARKERCAN_T_CELLS																														
CELLMARKERCAN_T_CYTOTOXIC_CELLS																														
CELLMARKERCAN_T_FOLLICULAR_HELPER_CELLS																														
CELLMARKERCAN_T_MEMORY_CELLS																														
CELLMARKERCAN_TRANSIENT_CELLS																														
CELLMARKERCAN_TRICHOCYTES																														
CELLMARKERCAN_TRIGEMINAL_NEURONS																														
CELLMARKERCAN_TROPHOBLAST_CELLS																														
CELLMARKERCAN_TROPHOBLAST_PROGENITOR_CELLS																														
CELLMARKERCAN_TUFT_CELLS																														
CELLMARKERCANENDOTHELIALCELLSAORTA																														
CELLMARKERCUR_N_ABNORMAL_MYELOID_CELL																														

CELLMARKERCAN_ACINAR_CELLS																														
CELLMARKERCUR_N_ABNORMAL_PLASMA_CELL																														
CELLMARKERCUR_N_ACTIVATED_DENDRITIC_CELL																														
CELLMARKERCUR_N_ACTIVATED_EFFECTOR_CELL																														
CELLMARKERCUR_N_ACTIVATED_HEPATIC_STELLAT…																														
CELLMARKERCUR_N_ACTIVATED_LANGERHANS_CELL																														
CELLMARKERCUR_N_ACTIVATED_MACROPHAGE																														
CELLMARKERCUR_N_ACTIVATED_MEMORY_T_CELL																														
CELLMARKERCUR_N_ACTIVATED_NATURAL_KILLER_…																														
CELLMARKERCUR_N_ACTIVATED_REGULATORY_T_CE…																														
CELLMARKERCUR_N_ADIPOCYTE_PROGENITOR_CELL																														
CELLMARKERCUR_N_ADRENAL_MEDULLA_CELL																														
CELLMARKERCUR_N_ADULT_SPERMATOGONIA																														
CELLMARKERCUR_N_ADVENTITIAL_CELL																														
CELLMARKERCUR_N_AEROCYTE																														
CELLMARKERCUR_N_AIRWAY_SMOOTH_MUSCLE_CELL																														
CELLMARKERCUR_N_ALVEOLAR_CELL_TYPE_1																														
CELLMARKERCUR_N_ALVEOLAR_CELL_TYPE_2																														
CELLMARKERCUR_N_ALVEOLAR_EPITHELIAL_CELL_…																														
CELLMARKERCUR_N_ALVEOLAR_EPITHELIAL_CELL_…																														
CELLMARKERCUR_N_ALVEOLAR_PNEUMOCYTE_TYPE_I																														
CELLMARKERCUR_N_ALVEOLAR_PNEUMOCYTE_TYPE_…																														
CELLMARKERCUR_N_AMACRINE_CELL																														
CELLMARKERCUR_N_AMNIOTIC_FLUID_STEM_CELL																														
CELLMARKERCUR_N_ANTERIOR_CELL																														
CELLMARKERCUR_N_ANTERIOR_HINDBRAIN_CELL																														
CELLMARKERCUR_N_ANTI_TUMOR_IMMUNE_CELL																														
CELLMARKERCUR_N_ANTIGEN_PRESENTATION_CANC…																														
CELLMARKERCUR_N_ASTROCYTE																														
CELLMARKERCUR_N_ATRIAL_CELL																														
CELLMARKERCUR_N_ATRIOVENTRICULAR_CANAL_CE…																														
CELLMARKERCUR_N_ATYPICAL_MEMORY_B_CELL																														
CELLMARKERCUR_N_B_2_CELL																														
CELLMARKERCUR_N_B1_CELL																														
CELLMARKERCUR_N_B10_REGULATORY_B_CELL																														
CELLMARKERCUR_N_BASAL_CELL																														
CELLMARKERCUR_N_BASAL_EPITHELIAL_CELL																														
CELLMARKERCUR_N_BASAL_KERATINOCYTE																														
CELLMARKERCUR_N_BASAL_LIKE_CELL																														
CELLMARKERCUR_N_BASAL_PROGENITOR_CELL																														
CELLMARKERCUR_N_BASOPHIL																														
CELLMARKERCUR_N_BILIARY_CELL																														
CELLMARKERCUR_N_BONE_MARROW_STEM_CELL																														
CELLMARKERCUR_N_BONE_SPECIFIC_CELL																														
CELLMARKERCUR_N_BRAIN_CELL																														
CELLMARKERCUR_N_CANCER_CELL	BCAN	PTN	KLRB1	TIMP1	ITK	ARHGA…	EDNRA	SELP	TLL1	SEMA6A	CDH5	CPNE1	TPST2	CRYBB2	SULF2	KLRF1	SELE	ALPI	FAM17…	ADH1B	AKR1A1	CAMK1D	CHST15	GOLM1	ISLR2	CD36	PRDM1	B3GNT2	TMPRS…	STOM
CELLMARKERCUR_N_CANCER_STEM_LIKE_CELL																														
CELLMARKERCUR_N_CAPILLARY_CELL																														
CELLMARKERCUR_N_CAR_T_CELL																														
CELLMARKERCUR_N_CARDIAC_CELL																														
CELLMARKERCUR_N_CARDIOMYOCYTE																														
CELLMARKERCUR_N_CARDIOSPHERE_DERIVED_CELL																														
CELLMARKERCUR_N_CARDIOVASCULAR_PROGENITOR…																														
CELLMARKERCUR_N_CAUDAL_CORTEX_CELL																														
CELLMARKERCUR_N_CD14_MONOCYTE																														
CELLMARKERCUR_N_CD16_MONOCYTE																														
CELLMARKERCUR_N_CD4_CD28_T_CELL																														
CELLMARKERCUR_N_CD4_PERIPHERALLY_DERIVED_…																														
CELLMARKERCUR_N_CD4_T_CELL																														
CELLMARKERCUR_N_CD56BRIGHT_NATURAL_KILLER…																														
CELLMARKERCUR_N_CD56DIM_NATURAL_KILLER_CE…																														
CELLMARKERCUR_N_CD8_T_CELL																														
CELLMARKERCUR_N_CENTRAL_MEMORY_T_CELL																														
CELLMARKERCUR_N_CENTRAL_MEMORY_T_HELPER_1…																														
CELLMARKERCUR_N_CENTRAL_NERVOUS_SYSTEM_ST…																														
CELLMARKERCUR_N_CHANDELIER_CELL																														
CELLMARKERCUR_N_CHIEF_CELL																														
CELLMARKERCUR_N_CHONDROCYTE																														
CELLMARKERCUR_N_CHROMAFFIN_CELL																														
CELLMARKERCUR_N_CIRCULATING_ANGIOGENIC_CE…																														
CELLMARKERCUR_N_CIRCULATING_FETAL_CELL																														
CELLMARKERCUR_N_CIRCULATING_NATURAL_KILLE…																														
CELLMARKERCUR_N_CIRCULATING_PRECURSOR_CELL																														
CELLMARKERCUR_N_CIRCULATING_PROGENITOR_CE…																														
CELLMARKERCUR_N_CIRCULATING_T_FOLLICULAR_…																														
CELLMARKERCUR_N_CIRCULATING_TUMOR_CELL																														
CELLMARKERCUR_N_CLASS_SWITCHED_PLASMABLAST																														
CELLMARKERCUR_N_CLASSICAL_B_CELL																														
CELLMARKERCUR_N_CLASSICAL_CANCER_ASSOCIAT…																														
CELLMARKERCUR_N_CLASSICAL_MONOCYTE																														
CELLMARKERCUR_N_CLAUDIN_LOW_CELL																														
CELLMARKERCUR_N_COLLECTING_DUCTAL_CELL																														
CELLMARKERCUR_N_COLONCYTE																														
CELLMARKERCUR_N_COLORECTAL_STEM_CELL																														
CELLMARKERCUR_N_COLUMNAR_EPITHELIAL_CELL																														
CELLMARKERCUR_N_COMMON_LYMPHOID_PROGENITO…																														
CELLMARKERCUR_N_COMMON_MYELOID_PROGENITOR…																														
CELLMARKERCUR_N_COMPLEMENT_SECRETING_CANC…																														
CELLMARKERCUR_N_CONE_PHOTORECEPTOR_CELL																														
CELLMARKERCUR_N_CONNECTING_TUBULAR_CELL																														
CELLMARKERCUR_N_CONVENTIONAL_CD4_T_CELL																														
CELLMARKERCUR_N_CORNEAL_ENDOTHELIAL_CELL																														
CELLMARKERCUR_N_CORNEAL_EPITHELIAL_CELL																														
CELLMARKERCUR_N_CORNEOCYTE_LIKE_CELL																														
CELLMARKERCUR_N_CORTICAL_CELL																														
CELLMARKERCUR_N_CORTICAL_STROMAL_CELL																														
CELLMARKERCUR_N_CORTICOTROPH_TUMOR_CELL																														
CELLMARKERCUR_N_CRANIAL_NEURAL_CREST_CELL																														
CELLMARKERCUR_N_CRYPT_CELL																														
CELLMARKERCUR_N_CRYPT_STEM_CELL																														
CELLMARKERCUR_N_CYCLING_B_CELL																														
CELLMARKERCUR_N_CYCLING_EPITHELIAL_CELL																														
CELLMARKERCUR_N_CYTOTOXIC_CD8_T_CELL																														
CELLMARKERCUR_N_CYTOTOXIC_DOUBLE_POSITIVE…																														
CELLMARKERCUR_N_CYTOTOXIC_T_CELL																														

CELLMARKERCAN_ACINAR_CELLS																														
CELLMARKERCUR_N_CYTOTOXIC_T_CELL_2																														
CELLMARKERCUR_N_DAMAGE_ASSOCIATED_TRANSIE…																														
CELLMARKERCUR_N_DARK_ZONE_CELL																														
CELLMARKERCUR_N_DEDIFFERENTIATION_ADIPOCY…																														
CELLMARKERCUR_N_DEEP_LAYER_NEURON																														
CELLMARKERCUR_N_DEFINITIVE_ENDODERM_CELL																														
CELLMARKERCUR_N_DELTA_CELL																														
CELLMARKERCUR_N_DEMYELINATING_MICROGLIAL_…																														
CELLMARKERCUR_N_DENDRITIC_CELL_2A																														
CELLMARKERCUR_N_DENDRITIC_CELL_LINEAGE																														
CELLMARKERCUR_N_DENTAL_PULP_CELL																														
CELLMARKERCUR_N_DERMAL_CELL																														
CELLMARKERCUR_N_DEUTEROSOMAL_CELL																														
CELLMARKERCUR_N_DIFFUSE_GASTRIC_CANCER_CE…																														
CELLMARKERCUR_N_DISTAL_CELL																														
CELLMARKERCUR_N_DISTAL_STEM_CELL																														
CELLMARKERCUR_N_DISTAL_TUBULAR_CELL																														
CELLMARKERCUR_N_DIVIDING_CELL																														
CELLMARKERCUR_N_DIVIDING_PLASMA_B_CELL																														
CELLMARKERCUR_N_DIVIDING_T_CELL																														
CELLMARKERCUR_N_DOPAMINERGIC_NEURON																														
CELLMARKERCUR_N_DORSAL_ANTERIOR_HINDBRAIN…																														
CELLMARKERCUR_N_DORSAL_LATERAL_GANGLIONIC…																														
CELLMARKERCUR_N_DORSAL_MEDIAL_GANGLIONIC_…																														
CELLMARKERCUR_N_DOUBLE_NEGATIVE_B_CELL																														
CELLMARKERCUR_N_DOUBLE_NEGATIVE_T_CELL																														
CELLMARKERCUR_N_DOUBLE_POSITIVE_T_CELL																														
CELLMARKERCUR_N_DOUBLE_POSITIVE_TREG_CELL																														
CELLMARKERCUR_N_EARLY_HEMANGIOBLAST																														
CELLMARKERCUR_N_EARLY_PROXIMAL_TUBULE_CELL																														
CELLMARKERCUR_N_EARLY_RESPONSE_SECRETORY_…																														
CELLMARKERCUR_N_EFFECTOR_CHONDROCYTE																														
CELLMARKERCUR_N_EFFECTOR_MEMORY_T_CELL																														
CELLMARKERCUR_N_EFFECTOR_NATURAL_KILLER_C…																														
CELLMARKERCUR_N_ELONGATING_CELL																														
CELLMARKERCUR_N_EMBRYONIC_CELL																														
CELLMARKERCUR_N_EMBRYONIC_STEM_CELL																														
CELLMARKERCUR_N_ENDOCARDIAL_CELL																														
CELLMARKERCUR_N_ENDOCRINE_PROGENITOR_CELL																														
CELLMARKERCUR_N_ENDODERM_PROGENITOR_CELL																														
CELLMARKERCUR_N_ENDODERMAL_CELL																														
CELLMARKERCUR_N_ENDOMETRIAL_STROMAL_CELL																														
CELLMARKERCUR_N_ENDOMETRIAL_STROMAL_STEM_…																														
CELLMARKERCUR_N_ENDOTHELIAL_PRECURSOR_CELL																														
CELLMARKERCUR_N_ENSHEATHING_GLIAL_CELL																														
CELLMARKERCUR_N_ENTERIC_NEURON																														
CELLMARKERCUR_N_EOSINOPHIL																														
CELLMARKERCUR_N_EPENDYMAL_CELL																														
CELLMARKERCUR_N_EPIBLAST_CELL																														
CELLMARKERCUR_N_EPICARDIAL_CELL																														
CELLMARKERCUR_N_EPICARDIUM_DERIVED_CELL																														
CELLMARKERCUR_N_EPIDERMAL_CELL																														
CELLMARKERCUR_N_EPIDERMAL_STEM_CELL																														
CELLMARKERCUR_N_EPITHELIAL_CELL	TYR	FOXC1	AQP5	PAX6	BEST1	RLBP1	CRX	RAX	MUC5AC	TFF2	TFF1	PGC	MUC6	SFN	SMARC…	BEST4	LGR5	CCL7	RBP2	SLC26…	CCL20	TNFRS…	CCL13	MUC2	FABP1	HCK	ASCL2	SLC26…	CDC25C	LEFTY1
CELLMARKERCUR_N_EPITHELIAL_DUCTAL_CELL																														
CELLMARKERCUR_N_EPITHELIAL_MESENCHYMAL_CE…																														
CELLMARKERCUR_N_EPITHELIOID_MACROPHAGE																														
CELLMARKERCUR_N_EPSILON_CELL																														
CELLMARKERCUR_N_ERYTHROBLAST																														
CELLMARKERCUR_N_ERYTHROID_CELL																														
CELLMARKERCUR_N_ERYTHROID_PROGENITOR_CELL																														
CELLMARKERCUR_N_ESOPHAGEAL_CELL																														
CELLMARKERCUR_N_ESOPHAGEAL_PROGENITOR_CELL																														
CELLMARKERCUR_N_EXCITATORY_NEURON																														
CELLMARKERCUR_N_EXHAUSTED_DOUBLE_POSITIVE…																														
CELLMARKERCUR_N_EXTRAVILLOUS_TROPHOBLAST																														
CELLMARKERCUR_N_F_PERICYTE																														
CELLMARKERCUR_N_FETAL_MYOBLAST																														
CELLMARKERCUR_N_FIBROBLAST_LIKE_CELL																														
CELLMARKERCUR_N_FIBROCHONDROCYTE																														
CELLMARKERCUR_N_FIBROCHONDROCYTE_PROGENIT…																														
CELLMARKERCUR_N_FOAM_CELL																														
CELLMARKERCUR_N_FOLLICULAR_B_CELL																														
CELLMARKERCUR_N_FOLLICULAR_CELL																														
CELLMARKERCUR_N_FOLLICULAR_DENDRITIC_CELL																														
CELLMARKERCUR_N_FOREBRAIN_CELL																														
CELLMARKERCUR_N_GABAERGIC_NEURON																														
CELLMARKERCUR_N_GANGLION_CELL																														
CELLMARKERCUR_N_GASTRIC_NEUROENDOCRINE_CE…																														
CELLMARKERCUR_N_GASTRIC_STEM_CELL																														
CELLMARKERCUR_N_GERM_CELL																														
CELLMARKERCUR_N_GERMINAL_CENTER_B_CELL																														
CELLMARKERCUR_N_GERMINAL_CENTER_CELL																														
CELLMARKERCUR_N_GERMINAL_CENTRE_B_CELL																														
CELLMARKERCUR_N_GLIAL_CELL																														
CELLMARKERCUR_N_GLIOMA_STEM_CELL																														
CELLMARKERCUR_N_GLOBOSE_BASAL_CELL																														
CELLMARKERCUR_N_GLOMERULAR_PARIETAL_EPITH…																														
CELLMARKERCUR_N_GLUTAMATERGIC_NEURON																														
CELLMARKERCUR_N_GOBLET_CELL																														
CELLMARKERCUR_N_GONADOTROPE																														
CELLMARKERCUR_N_GONADOTROPH_CELL																														
CELLMARKERCUR_N_GRANULOCYTE																														
CELLMARKERCUR_N_GRANULOCYTE_MONOCYTE_PROG…																														
CELLMARKERCUR_N_GRANULOCYTIC_UNK																														
CELLMARKERCUR_N_GRANULOSA_CELL																														
CELLMARKERCUR_N_HAEMATOPOIETIC_CELL																														
CELLMARKERCUR_N_HAIR_FOLLICLE_CELL																														
CELLMARKERCUR_N_HEMANGIOBLAST																														
CELLMARKERCUR_N_HEMATOPOIETIC_CELL																														
CELLMARKERCUR_N_HEMATOPOIETIC_PRECURSOR_C…																														
CELLMARKERCUR_N_HEMATOPOIETIC_PROGENITOR_…																														
CELLMARKERCUR_N_HEPATIC_CELL																														
CELLMARKERCUR_N_HEPATIC_PROGENITOR_CELL																														

CELLMARKERCAN_ACINAR_CELLS																														
CELLMARKERCUR_N_HEPATIC_STELLATE_CELL																														
CELLMARKERCUR_N_HEPATOBLAST																														
CELLMARKERCUR_N_HEPATOCYTE																														
CELLMARKERCUR_N_HISTIOCYTE																														
CELLMARKERCUR_N_HMSC_TREATED_B_CELL																														
CELLMARKERCUR_N_HOFBAUER_CELL																														
CELLMARKERCUR_N_HOMEOSTATIC_CHONDROCYTE																														
CELLMARKERCUR_N_HOMEOSTATIC_MICROGLIAL_CE…																														
CELLMARKERCUR_N_HORIZONTAL_BASAL_CELL																														
CELLMARKERCUR_N_HORIZONTAL_CELL																														
CELLMARKERCUR_N_HYPOBLAST_CELL																														
CELLMARKERCUR_N_IDIOPATHIC_PULMONARY_FIBR…																														
CELLMARKERCUR_N_IGA_MEMORY_B_CELL																														
CELLMARKERCUR_N_IGG_MEMORY_B_CELL																														
CELLMARKERCUR_N_IGM_MEMORY_B_CELL																														
CELLMARKERCUR_N_IGM_ONLY_B_CELL																														
CELLMARKERCUR_N_IL7R_T_HELPER_CELL																														
CELLMARKERCUR_N_IMMATURE_ERYTHROID_CELL																														
CELLMARKERCUR_N_IMMATURE_MYELOID_CELL																														
CELLMARKERCUR_N_IMMATURE_NEUTROPHIL																														
CELLMARKERCUR_N_IMMATURE_TRANSITIONAL_B_C…																														
CELLMARKERCUR_N_IMMUNE_CELL																														
CELLMARKERCUR_N_INDUCED_PLURIPOTENT_STEM_…																														
CELLMARKERCUR_N_INFILTRATING_MACROPHAGE																														
CELLMARKERCUR_N_INFLAMMATORY_CANCER_ASSOC…																														
CELLMARKERCUR_N_INFLAMMATORY_DENDRITIC_CE…																														
CELLMARKERCUR_N_INFLAMMATORY_MONOCYTE																														
CELLMARKERCUR_N_INHIBITORY_NEURON																														
CELLMARKERCUR_N_INNATE_LYMPHOID_CELL																														
CELLMARKERCUR_N_INNER_CELL_MASS_CELL																														
CELLMARKERCUR_N_INNER_RADIAL_GLIAL_CELL																														
CELLMARKERCUR_N_INTER_MEDIATE_PROGENITOR_…																														
CELLMARKERCUR_N_INTERCALATED_CELL																														
CELLMARKERCUR_N_INTERFERON_RESPONSIVE_SEC…																														
CELLMARKERCUR_N_INTERMEDIATE_MONOCYTE																														
CELLMARKERCUR_N_INTERMEDIATE_MONOCYTE_CELL																														
CELLMARKERCUR_N_INTERSTITIAL_MACROPHAGE																														
CELLMARKERCUR_N_INTERSTITIAL_PROGENITOR_C…																														
CELLMARKERCUR_N_INTESTINAL_EPITHELIAL_STE…																														
CELLMARKERCUR_N_INTESTINAL_GASTRIC_CANCER…																														
CELLMARKERCUR_N_INTESTINAL_METAPLASIA_CELL																														
CELLMARKERCUR_N_INTRAEPITHELIAL_LYMPHOCYT…																														
CELLMARKERCUR_N_IONOCYTE																														
CELLMARKERCUR_N_IRIS_PUTTATIVE_STEM_CELL																														
CELLMARKERCUR_N_KUPFFER_CELL																														
CELLMARKERCUR_N_LACTOTROPH_CELL																														
CELLMARKERCUR_N_LANGERHANS_CELL																														
CELLMARKERCUR_N_LEUKOCYTE																														
CELLMARKERCUR_N_LIMBAL_EPITHELIAL_STEM_CE…																														
CELLMARKERCUR_N_LIMBAL_STEM_CELL																														
CELLMARKERCUR_N_LIPOFIBROBLAST																														
CELLMARKERCUR_N_LIVER_PROGENITOR_CELL																														
CELLMARKERCUR_N_LIVER_STEM_CELL																														
CELLMARKERCUR_N_LMMLEYDIG_CELL																														
CELLMARKERCUR_N_LONOCYTE																														
CELLMARKERCUR_N_LOOP_OF_HENLE_CELL																														
CELLMARKERCUR_N_LOOP_PROGENITOR_CELL																														
CELLMARKERCUR_N_LOW_DENSITY_NEUTROPHIL																														
CELLMARKERCUR_N_LUMINAL_PROGENITOR_CELL																														
CELLMARKERCUR_N_LUNG_CELL																														
CELLMARKERCUR_N_LYMPH_NODE_CELL																														
CELLMARKERCUR_N_LYMPHATIC_CELL																														
CELLMARKERCUR_N_LYMPHATIC_ENDOTHELIAL_CELL																														
CELLMARKERCUR_N_LYMPHOBLASTOID_CELL																														
CELLMARKERCUR_N_LYMPHOCYTE																														
CELLMARKERCUR_N_LYMPHOID_CELL																														
CELLMARKERCUR_N_LYMPHOID_PROGENITOR_CELL																														
CELLMARKERCUR_N_LYMPHOID_STRUCTURE_CELL																														
CELLMARKERCUR_N_M_PERICYTE																														
CELLMARKERCUR_N_M1_MACROPHAGE																														
CELLMARKERCUR_N_M1_MICROGLIAL_CELL																														
CELLMARKERCUR_N_M2_MACROPHAGE																														
CELLMARKERCUR_N_MACROGLIAL_CELL																														
CELLMARKERCUR_N_MALIGNANT_CELL																														
CELLMARKERCUR_N_MALIGNANT_PLASMA_CELL																														
CELLMARKERCUR_N_MARGINAL_ASSOCIATED_MACRO…																														
CELLMARKERCUR_N_MAST_CELL																														
CELLMARKERCUR_N_MATURE_ASTROCYTE																														
CELLMARKERCUR_N_MATURE_B_CELL																														
CELLMARKERCUR_N_MATURE_DENDRITIC_CELL																														
CELLMARKERCUR_N_MATURE_ENDOTHELIAL_CELL																														
CELLMARKERCUR_N_MATURE_LUMINAL_CELL																														
CELLMARKERCUR_N_MATURE_NEURON																														
CELLMARKERCUR_N_MATURE_NEURONAL																														
CELLMARKERCUR_N_MATURE_OLIGODENDROCYTE																														
CELLMARKERCUR_N_MATURE_OSTEOBLAST																														
CELLMARKERCUR_N_MATURE_PODOCYTE																														
CELLMARKERCUR_N_MEDIAL_AND_DISTAL_SEGMENT…																														
CELLMARKERCUR_N_MEDIAL_GANGLIONIC_EMINENC…																														
CELLMARKERCUR_N_MEGAKARYOCYTE																														
CELLMARKERCUR_N_MEGAKARYOCYTE_ERYTHROID_C…																														
CELLMARKERCUR_N_MEGAKARYOCYTE_ERYTHROID_P…																														
CELLMARKERCUR_N_MEGAKARYOCYTE_PROGENITOR_…																														
CELLMARKERCUR_N_MELANOMA_CELL																														
CELLMARKERCUR_N_MEMORY_B_CELL																														
CELLMARKERCUR_N_MEMORY_B_CELL_PRECURSOR																														
CELLMARKERCUR_N_MEMORY_CD27_B_CELL																														
CELLMARKERCUR_N_MEMORY_CD4_T_CELL																														
CELLMARKERCUR_N_MEMORY_DOUBLE_POSITIVE_T_…																														
CELLMARKERCUR_N_MEMORY_REGULATORY_T_CELL																														
CELLMARKERCUR_N_MEMORY_T_CELL																														
CELLMARKERCUR_N_MERKEL_CELL																														
CELLMARKERCUR_N_MESENCHYMAL_CELL																														
CELLMARKERCUR_N_MESENCHYMAL_EPITHELIAL_TR…																														

Table S5: Cell state marker gene list

CELLMARKERCAN_ACINAR_CELLS																														
CELLMARKERCUR_N_MESENCHYMAL_GLIOBLASTOMA_…																														
CELLMARKERCUR_N_MESENCHYMAL_STEM_CELL																														
CELLMARKERCUR_N_MESENCHYMAL_STROMAL_CELL																														
CELLMARKERCUR_N_MESENDODERMAL_CELL																														
CELLMARKERCUR_N_MESODERMAL_EMBRYONIC_STEM…																														
CELLMARKERCUR_N_MESODERMAL_PROGENITOR_CELL																														
CELLMARKERCUR_N_METAPLASTIC_STEM_CELL																														
CELLMARKERCUR_N_MEYLOID_CELL																														
CELLMARKERCUR_N_MICROGLIAL_CELL	GALK1	BHLHE…	GRIA2	CREG1	GRN	GAS7	GM2A	ATOH8	ST14	COPS9	TPI1	TCAP	TOX2	SSR4	RPS19	CXCR4	GYPC	GBA	RPS25	SLAMF9	CHCHD5	ZNF696	GPR65	HIF1A	SULF2	PLIN2	ACACA	TIMP2	RPS10	RPS18
CELLMARKERCUR_N_MICROVILLAR_CELL																														
CELLMARKERCUR_N_MIDBRAIN_DOPAMINERGIC_NEU…																														
CELLMARKERCUR_N_MONO_MACROPHAGE																														
CELLMARKERCUR_N_MONOCYTE_DERIVED_CELL																														
CELLMARKERCUR_N_MONOCYTE_DERIVED_MYELOID_…																														
CELLMARKERCUR_N_MONOCYTE_LINEAGE																														
CELLMARKERCUR_N_MONOCYTE_PRECURSOR																														
CELLMARKERCUR_N_MONOCYTE_PROGENITOR_CELL																														
CELLMARKERCUR_N_MUELLER_GLIA_CELL																														
CELLMARKERCUR_N_MULTIPOTENT_MESENCHYMAL_S…																														
CELLMARKERCUR_N_MULTIPOTENT_RETINAL_PROGE…																														
CELLMARKERCUR_N_MULTIPOTENT_STEM_CELL																														
CELLMARKERCUR_N_MULTIPOTENT_STROMAL_CELL																														
CELLMARKERCUR_N_MUSCLE_CELL																														
CELLMARKERCUR_N_MUSCLE_DERIVED_CELL																														
CELLMARKERCUR_N_MUSCLE_SATELLITE_CELL																														
CELLMARKERCUR_N_MYELOBLAST																														
CELLMARKERCUR_N_MYELOID_DENDRITIC_CELL																														
CELLMARKERCUR_N_MYELOID_DENDRITIC_CELL_1																														
CELLMARKERCUR_N_MYELOID_DERIVED_CELL																														
CELLMARKERCUR_N_MYELOID_DERIVED_SUPPRESSO…																														
CELLMARKERCUR_N_MYELOID_PROGENITOR_CELL																														
CELLMARKERCUR_N_MYO_EPITHELIAL_CELL																														
CELLMARKERCUR_N_MYOBLAST																														
CELLMARKERCUR_N_MYOCYTE																														
CELLMARKERCUR_N_MYOFIBROBLAST																														
CELLMARKERCUR_N_MYOGENIC_ENDOTHELIAL_CELL																														
CELLMARKERCUR_N_MYOGENIC_PROGENITOR_CELL																														
CELLMARKERCUR_N_MYOID_CELL																														
CELLMARKERCUR_N_NAIVE_B_CELL																														
CELLMARKERCUR_N_NAIVE_CD8_T_CELL																														
CELLMARKERCUR_N_NAIVE_DOUBLE_POSITIVE_T_C…																														
CELLMARKERCUR_N_NAIVE_PROGENITOR_CELL																														
CELLMARKERCUR_N_NAIVE_T_CELL																														
CELLMARKERCUR_N_NASCENT_NEPHRON_CELL																														
CELLMARKERCUR_N_NATURAL_KILLER_CD56_BRIGH…																														
CELLMARKERCUR_N_NATURAL_KILLER_CD56_DIM_C…																														
CELLMARKERCUR_N_NEGATIVE_MESENCHYMAL_STEM…																														
CELLMARKERCUR_N_NEURAL_CELL																														
CELLMARKERCUR_N_NEURAL_CREST_STEM_CELL																														
CELLMARKERCUR_N_NEURAL_CREST_STEMLIKE_CELL																														
CELLMARKERCUR_N_NEURAL_PRECURSOR_CELL																														
CELLMARKERCUR_N_NEURAL_STEM_CELL																														
CELLMARKERCUR_N_NEUROECTODERM_CELL																														
CELLMARKERCUR_N_NEURONAL_INTERMEDIATE_PRO…																														
CELLMARKERCUR_N_NEURONAL_PROGENITOR_CELL																														
CELLMARKERCUR_N_NEUTROPHIL_PROGENITOR_CELL																														
CELLMARKERCUR_N_NON_NEURON																														
CELLMARKERCUR_N_NON_PT_TUBULAR_CELL																														
CELLMARKERCUR_N_NON_RECIRCULATING_TISSUE_…																														
CELLMARKERCUR_N_NONMYELINATING_SCHWANN_CE…																														
CELLMARKERCUR_N_NOTOCHORD_CELL																														
CELLMARKERCUR_N_NUCLEATED_CELL																														
CELLMARKERCUR_N_NUCLEUS_PULPOSUS_PROGENIT…																														
CELLMARKERCUR_N_OLIGODENDROCYTE																														
CELLMARKERCUR_N_OSTEOBLAST																														
CELLMARKERCUR_N_OUTER_RADIAL_GLIA																														
CELLMARKERCUR_N_OVALBUMIN_SPECIFIC_REGULA…																														
CELLMARKERCUR_N_OVARIAN_CELL																														
CELLMARKERCUR_N_OVARIAN_STROMAL_CELL																														
CELLMARKERCUR_N_P53_LIKE_CELL																														
CELLMARKERCUR_N_PAN_B_CELL																														
CELLMARKERCUR_N_PAN_CANCER_ASSOCIATED_FIB…																														
CELLMARKERCUR_N_PAN_MACROPHAGE																														
CELLMARKERCUR_N_PAN_MONOCYTE																														
CELLMARKERCUR_N_PAN_NEURONAL_CELL																														
CELLMARKERCUR_N_PAN_TROPHECTODERM_CELL																														
CELLMARKERCUR_N_PANCREATIC_ENDODERM_CELL																														
CELLMARKERCUR_N_PANCREATIC_POLYPEPTIDE_CE…																														
CELLMARKERCUR_N_PANCREATIC_PROGENITOR_CELL																														
CELLMARKERCUR_N_PANCREATIC_STEM_CELL																														
CELLMARKERCUR_N_PANETH_CELL																														
CELLMARKERCUR_N_PANNEURON																														
CELLMARKERCUR_N_PANVASCULAR_CELL																														
CELLMARKERCUR_N_PAPILLARY_FIBROBLAST																														
CELLMARKERCUR_N_PARIETAL_CELL																														
CELLMARKERCUR_N_PARVALBUMIN_INTERNEURON																														
CELLMARKERCUR_N_PATELET																														
CELLMARKERCUR_N_PATHOGENIC_T_PERIPHERAL_H…																														
CELLMARKERCUR_N_PELVIC_EPITHELIAL_CELL																														
CELLMARKERCUR_N_PERICYTE																														
CELLMARKERCUR_N_PERIODONTAL_LIGAMENT_STEM…																														
CELLMARKERCUR_N_PERIOSTEUM_DERIVED_PROGEN…																														
CELLMARKERCUR_N_PERITUBULAR_MYOID_CELL																														
CELLMARKERCUR_N_PERIVASCULAR_LIKE_CELL																														
CELLMARKERCUR_N_PHAGOCYTE																														
CELLMARKERCUR_N_PHOTORECEPTOR_CELL																														
CELLMARKERCUR_N_PIT_CELL																														
CELLMARKERCUR_N_PIT_MUCOUS_CELL																														
CELLMARKERCUR_N_PITUITARY_STEM_CELL																														
CELLMARKERCUR_N_PLACENTAL_TISSUE_CELL																														
CELLMARKERCUR_N_PLACODE_CELL																														
CELLMARKERCUR_N_PLASMABLAST_B_CELL																														
CELLMARKERCUR_N_PLATELET																														
CELLMARKERCUR_N_PLURIPOTENT_STEM_CELL																														

CELLMARKERCAN_ACINAR_CELLS																														
CELLMARKERCUR_N_PODOCYTE_PRECURSOR																														
CELLMARKERCUR_N_PORTAL_FIBROBLAST																														
CELLMARKERCUR_N_POSTERIOR_CELL																														
CELLMARKERCUR_N_PRE_B_CELL																														
CELLMARKERCUR_N_PRE_CONVENTIONAL_DENDRITI…																														
CELLMARKERCUR_N_PRE_DENDRITIC_CELL																														
CELLMARKERCUR_N_PRECURSOR_CELL																														
CELLMARKERCUR_N_PREHYPERTROPHIC_CHONDROCY…																														
CELLMARKERCUR_N_PRESOMITE_CELL																														
CELLMARKERCUR_N_PRETUBULAR_AGGREGATE_CELL																														
CELLMARKERCUR_N_PRIMITIVE_ENDODERM_CELL																														
CELLMARKERCUR_N_PRINCIPAL_CELL																														
CELLMARKERCUR_N_PRO_B_CELL																														
CELLMARKERCUR_N_PRO_INFLAMMATORY_MACROPHA…																														
CELLMARKERCUR_N_PRO_NEUTROPHIL																														
CELLMARKERCUR_N_PROLIFERATING_CELL																														
CELLMARKERCUR_N_PROLIFERATIVE_CHONDROCYTE																														
CELLMARKERCUR_N_PROLIFERATIVE_DUCTAL_CELL																														
CELLMARKERCUR_N_PROLIFERATIVE_NEURAL_PROG…																														
CELLMARKERCUR_N_PRONEURAL_GLIOBLASTOMA_CE…																														
CELLMARKERCUR_N_PROSPERMATOGONIA																														
CELLMARKERCUR_N_PROSTATE_CELL																														
CELLMARKERCUR_N_PROXIMAL_CELL																														
CELLMARKERCUR_N_PROXIMAL_SEGMENT_CELL																														
CELLMARKERCUR_N_PROXIMAL_STEM_CELL																														
CELLMARKERCUR_N_PROXIMAL_TUBULE_CELL																														
CELLMARKERCUR_N_PULMONARY_NEUROENDOCRINE_…																														
CELLMARKERCUR_N_PYRAMIDAL_CELL																														
CELLMARKERCUR_N_QUIESCENT_T_STEM_CELL																														
CELLMARKERCUR_N_REGULATORY_B_CELL																														
CELLMARKERCUR_N_REGULATORY_INNATE_LYMPHOI…																														
CELLMARKERCUR_N_RENAL_TUBULAR_CELL																														
CELLMARKERCUR_N_RESIDENT_MEMORY_T_CELL																														
CELLMARKERCUR_N_RESPIRATORY_HORIZONTAL_BA…																														
CELLMARKERCUR_N_RESPIRATORY_SECRETORY_CELL																														
CELLMARKERCUR_N_RESPONDER_T_CELL																														
CELLMARKERCUR_N_RESPONDING_CONVENTIONAL_T…																														
CELLMARKERCUR_N_RESTING_MEMORY_B_CELL																														
CELLMARKERCUR_N_RESTING_NAIVE_B_CELL																														
CELLMARKERCUR_N_RESTING_REGULATORY_T_CELL																														
CELLMARKERCUR_N_RETICULAR_FIBROBLAST																														
CELLMARKERCUR_N_RETINA_CELL																														
CELLMARKERCUR_N_ROD_CELL																														
CELLMARKERCUR_N_SATELLITE_CELL																														
CELLMARKERCUR_N_SEPTUM_TRANSVERSUM_MESENC…																														
CELLMARKERCUR_N_SEROUS_CELL																														
CELLMARKERCUR_N_SIDE_POPULATION_CELL																														
CELLMARKERCUR_N_SKELETAL_MUSCLE_CELL																														
CELLMARKERCUR_N_SKELETAL_MUSCLE_STEM_CELL																														
CELLMARKERCUR_N_SMOOTH_MUSCLE_CELL																														
CELLMARKERCUR_N_SMOOTH_MUSCLE_LIKE_CELL																														
CELLMARKERCUR_N_SOMATIC_CELL																														
CELLMARKERCUR_N_SOMATIC_STEM_CELL																														
CELLMARKERCUR_N_SOMATOSTATIN_INTERNEURON																														
CELLMARKERCUR_N_SPECIALIST_ANTIGEN_PRESEN…																														
CELLMARKERCUR_N_SPERMATOGENIC_CELL																														
CELLMARKERCUR_N_SPERMATOGONIAL_CELL																														
CELLMARKERCUR_N_SPERMATOGONIAL_STEM_CELL																														
CELLMARKERCUR_N_SQUAMOUS_DIFFERENTIATION_…																														
CELLMARKERCUR_N_STELLATE_CELL																														
CELLMARKERCUR_N_STEM_CELL_DERIVED_ALPHA_C…																														
CELLMARKERCUR_N_STEM_CELL_DERIVED_BETA_CE…																														
CELLMARKERCUR_N_STEM_CELL_DERIVED_ENDOTHE…																														
CELLMARKERCUR_N_STEM_LIKE_CD8_T_CELL																														
CELLMARKERCUR_N_STROMAL_CELL																														
CELLMARKERCUR_N_SUBPALLIUM_CELL																														
CELLMARKERCUR_N_SUBPLATE_CELL																														
CELLMARKERCUR_N_SUPPRESSIVE_MONOCYTE																														
CELLMARKERCUR_N_SUPPRESSOR_T_CELL																														
CELLMARKERCUR_N_SUPRABASAL_CELL																														
CELLMARKERCUR_N_SURFACE_ECTODERM_CELL																														
CELLMARKERCUR_N_SUSTENTACULAR_CELL																														
CELLMARKERCUR_N_SYMPATHOADRENAL_CELL																														
CELLMARKERCUR_N_SYNCYTIOTROPHOBLAST																														
CELLMARKERCUR_N_SYNTHETIC_SMOOTH_MUSCLE_C…																														
CELLMARKERCUR_N_T_CELL_LARGE_GRANULAR_LYM…																														
CELLMARKERCUR_N_T_CELL_LINEAGE																														
CELLMARKERCUR_N_T_HELPER_CELL																														
CELLMARKERCUR_N_T_MEMORY_STEM_CELL																														
CELLMARKERCUR_N_T2_MZP_REGULATORY_B_CELL																														
CELLMARKERCUR_N_TENDON_CELL																														
CELLMARKERCUR_N_TERMINAL_DIFFERENTIATED_B…																														
CELLMARKERCUR_N_THALAMIC_CELL																														
CELLMARKERCUR_N_THECA_INTERNA_CELL																														
CELLMARKERCUR_N_THYMIC_EPITHELIAL_CELL																														
CELLMARKERCUR_N_TISSUE_LIKE_MEMORY_B_CELL																														
CELLMARKERCUR_N_TISSUE_RESIDENT_CELL																														
CELLMARKERCUR_N_TISSUE_RESIDENT_EFFECTOR_…																														
CELLMARKERCUR_N_TISSUE_RESIDENT_MACROPHAGE																														
CELLMARKERCUR_N_TISSUE_RESIDENT_MEMORY_LI…																														
CELLMARKERCUR_N_TOLDC_INDUCED_REGULATORY_…																														
CELLMARKERCUR_N_TONSIL_CELL																														
CELLMARKERCUR_N_TRABECULAR_MESHWORK_CELL																														
CELLMARKERCUR_N_TRANSITIONAL_B_CELL																														
CELLMARKERCUR_N_TRANSITIONAL_DENDRITIC_CE…																														
CELLMARKERCUR_N_TRUNCATED_RADIAL_GLIAL_CE…																														
CELLMARKERCUR_N_TUBULAR_CELL																														
CELLMARKERCUR_N_TUMOR_ASSOCIATED_MICROGLI…																														
CELLMARKERCUR_N_TUMOR_ENDOTHELIAL_CELL																														
CELLMARKERCUR_N_TUMOR_EPITHELIAL_CELL																														
CELLMARKERCUR_N_TUMOR_INFILTRATING_REGULA…																														
CELLMARKERCUR_N_TUMOR_INFILTRATING_T_CELL																														
CELLMARKERCUR_N_TUMOR_INITIATING_CELL																														
CELLMARKERCUR_N_TUMOR_REGULATORY_T_CELL																														

CELLMARKERCAN_ACINAR_CELLS																														
CELLMARKERCUR_N_UMBRELLA_CELL																														
CELLMARKERCUR_N_UNRESTRICTED_SOMATIC_STEM…																														
CELLMARKERCUR_N_UNSWITCHED_PLASMABLAST																														
CELLMARKERCUR_N_URETERIC_BUD_CELL																														
CELLMARKERCUR_N_UROTHELIAL_CELL																														
CELLMARKERCUR_N_UROTHELIUM_CELL																														
CELLMARKERCUR_N_VASCULATURE_CELL																														
CELLMARKERCUR_N_VEIN_CELL																														
CELLMARKERCUR_N_VENOUS_CELL																														
CELLMARKERCUR_N_VENTRAL_LATERAL_GANGLIONI…																														
CELLMARKERCUR_N_VENTRAL_MEDIAL_GANGLIONIC…																														
CELLMARKERCUR_N_VENTRICULAR_CELL																														
CELLMARKERCUR_N_YOUNG_NEURON																														
CELLMARKERCUR_N_ZONA_FASCICULATA_CELL																														
CELLMARKERCUR_N_ZONA_GLOMERULOSA_CELL																														
CELLMARKERCUR_N_ZONA_RETICULARIS_CELL																														
CELLMARKERCUR_N_ZYGOTE																														
CELLMARKERCURNACTIVATEDEFFECTORMEMORYCD4T…																														
CELLMARKERCURNACTIVATEDTISSUERESIDENTMEMO…																														
CELLMARKERCURNACTIVATEDTISSUERESIDENTMEMO…																														
CELLMARKERCURNALPHACELLACELL																														
CELLMARKERCURNALVEOLARTYPEIIATIICELL																														
CELLMARKERCURNATYPICALBCELLABC																														
CELLMARKERCURNAXLCD11CPLASMACYTOIDDENDRIT…																														
CELLMARKERCURNAXLCD123PLASMACYTOIDDENDRIT…																														
CELLMARKERCURNBASALFOREBRAINCHOLINERGICNE…																														
CELLMARKERCURNBRUSHCELLTUFTCELL																														
CELLMARKERCURNCANCERASSOCIATEDFIBROBLASTC…																														
CELLMARKERCURNCAPSULARFIBROBLASTFB2																														
CELLMARKERCURNCCR7DENDRITICCELL																														
CELLMARKERCURNCD106MESENCHYMALSTEMCELL																														
CELLMARKERCURNCD14MACROPHAGE																														
CELLMARKERCURNCD16DENDRITICCELL																														
CELLMARKERCURNCD25LAG3TCELL																														
CELLMARKERCURNCD34GRANULOCYTICPROGENITORC…																														
CELLMARKERCURNCD34HEMATOPOIETICSTEMCELL																														
CELLMARKERCURNCD34PREBCELL																														
CELLMARKERCURNCD34PROGENITORCELL																														
CELLMARKERCURNCD34THYMOCYTE																														
CELLMARKERCURNCD40LGTHELPERCELL																														
CELLMARKERCURNCD4CD25REGULATORYTCELL																														
CELLMARKERCURNCD4CENTRALMEMORYLIKETTCMLIK…																														
CELLMARKERCURNCD4CYTOTOXICT1CELL																														
CELLMARKERCURNCD4CYTOTOXICT2CELL																														
CELLMARKERCURNCD4THELPERCELL																														
CELLMARKERCURNCHRONICALLYACTIVATEDCD4TCELL																														
CELLMARKERCURNCHRONICALLYACTIVATEDCD8TCELL																														
CELLMARKERCURNCLASSSWITCHEDMEMORYBIGGU03B…																														
CELLMARKERCURNCLEC10ADENDRITICCELL																														
CELLMARKERCURNCLEC9ADENDRITICCELL																														
CELLMARKERCURNCONVENTIONALDENDRITICCELL2B…																														
CELLMARKERCURNCONVENTIONALDENDRITICCELL2B…																														
CELLMARKERCURNCORTICALSOMATOSTATINSSTINTE…																														
CELLMARKERCURNCYTOTOXICCD4T2CELL																														
CELLMARKERCURNCYTOTOXICCD4TCELL																														
CELLMARKERCURND1MSNMEDIUMSPINYNEURON																														
CELLMARKERCURND2MSNMEDIUMSPINYNEURON																														
CELLMARKERCURNDISTALTUBULELOOPOFHENLECELL																														
CELLMARKERCURNEARLYCD34MEGAKARYOCYTEERYTH…																														
CELLMARKERCURNEARLYTCELLPROGENITORETP																														
CELLMARKERCURNEFFECTORMEMORYCD4TCELL																														
CELLMARKERCURNEFFECTORREGULATORYTETREGCELL																														
CELLMARKERCURNEFFECTORTTEFFCELL																														
CELLMARKERCURNEXHAUSTEDCD4TCELL																														
CELLMARKERCURNEXHAUSTEDCD8TCELL																														
CELLMARKERCURNEXHAUSTEDCD8TPROGENITORCELL																														
CELLMARKERCURNEXHAUSTEDTTEXCELL																														
CELLMARKERCURNEXTRATELENCEPHALICETCELL																														
CELLMARKERCURNFINALLYHIGHLYEFFECTORTEMRAM…																														
CELLMARKERCURNFOLLICULARREGULATORYTTFRCELL																														
CELLMARKERCURNFOXP3NATURALREGULATORYTTREG…																														
CELLMARKERCURNGAMMADELTAU03B3DTCELL																														
CELLMARKERCURNGERMINALCENTREGCLIKECELL																														
CELLMARKERCURNGLOMERULARENDOTHELIALCELLECG																														
CELLMARKERCURNGRANULOCYTICMYELOIDDERIVEDS…																														
CELLMARKERCURNGRBREGULATORYBCELL																														
CELLMARKERCURNGZMKTCELL																														
CELLMARKERCURNHIGHLYDIFFERENTIATEDEFFECTO…																														
CELLMARKERCURNINDUCEDREGULATORYTTREGCELL																														
CELLMARKERCURNINFLAMMATORYCANCERASSOCIATE…																														
CELLMARKERCURNINNATELYMPHOIDCELLILCS																														
CELLMARKERCURNINTRATHYMICFIBROBLASTFB1																														
CELLMARKERCURNITOCELLHEPATICSTELLATECELL																														
CELLMARKERCURNLYMPHANGIOLEIOMYOMATOSISCEL…																														
CELLMARKERCURNMARGINALZONEMZLIKEBCELL																														
CELLMARKERCURNMEMORYSTEMCELLTSCM																														
CELLMARKERCURNMICROGLIADERIVEDTUMORASSOCI…																														
CELLMARKERCURNMKERYTHCELL																														
CELLMARKERCURNMYOFIBROBLASTICCANCERASSOCI…																														
CELLMARKERCURNNAIVEREGULATORYTTREGCELL																														
CELLMARKERCURNNATURALREGULATORYTTREGCELL																														
CELLMARKERCURNPERIPHERALBLOODMONONUCLEARC…																														
CELLMARKERCURNPITUITARYSTEMCELLPSC																														
CELLMARKERCURNPLASMACYTOIDDENDRITICCELLPDC																														
CELLMARKERCURNPOLYMORPHONUCLEARMYELOIDDER…																														
CELLMARKERCURNPOLYMORPHONUCLEARNEUTROPHIL…																														
CELLMARKERCURNPOSTERIORPRESOMITICMESODERM…																														
CELLMARKERCURNPROLIFERATIVECD4TCELL																														
CELLMARKERCURNPROLIFERATIVECD8TCELL																														
CELLMARKERCURNREDBLOODCELLERYTHROCYTE																														
CELLMARKERCURNREGULATORYBBREGCELL																														
CELLMARKERCURNREGULATORYCD4TCELL																														
CELLMARKERCURNREGULATORYCD8TCELL																														
CELLMARKERCURNREGULATORYTTREGCELL																														

Table S5: Cell state marker gene list

CELLMARKERCAN_ACINAR_CELLS																														
CELLMARKERCURNREGULATORYTTREGCELL																														
CELLMARKERCURNRENALVESICLECOMMASHAPEDBODY…																														
CELLMARKERCURNRESIDENTMEMORYCD8TCELL																														
CELLMARKERCURNREVERTANTMEMORYCD8TTEMRACELL																														
CELLMARKERCURNSCARASSOCIATEDMACROPHAGESAM																														
CELLMARKERCURNSENESCENTCD8TCELL																														
CELLMARKERCURNSTEMPROGENITORCELL																														
CELLMARKERCURNSWITCHEDMEMORYBCELLSWMB																														
CELLMARKERCURNT1TRANSITIONALBCELL																														
CELLMARKERCURNT2TRANSITIONALBCELL																														
CELLMARKERCURNTCELLNATURALKILLERCELL																														
CELLMARKERCURNTERMINALEFFECTORCD8TCELL																														
CELLMARKERCURNTERMINALLYDIFFERENTIATEDCD8…																														
CELLMARKERCURNTFOLLICULARHELPERTFHCELL																														
CELLMARKERCURNTHELPER1TH1CELL																														
CELLMARKERCURNTHELPER1TH1CELL																														
CELLMARKERCURNTHELPER2TH2CELL																														
CELLMARKERCURNTHELPER2TH2CELL																														
CELLMARKERCURNTHELPER9TH9CELL																														
CELLMARKERCURNTHELPERTHCELL																														
CELLMARKERCURNTHYMICEPITHELIALCELLTEC																														
CELLMARKERCURNTISSUERESIDENTMEMORYCD4TCELL																														
CELLMARKERCURNTISSUERESIDENTMEMORYCD8TCELL																														
CELLMARKERCURNTISSUERESIDENTMEMORYTTRMCELL																														
CELLMARKERCURNTPERIPHERALHELPERTPHCELL																														
CELLMARKERCURNTRANSIENTAMPLIFYINGTACELL																														
CELLMARKERCURNTRANSITIONALEXHAUSTEDCD8TCE…																														
CELLMARKERCURNTUMORINFILTRATINGLYMPHOCYTE…																														
CELLMARKERCURNUNSWITCHEDMEMORYBCELLUNSWMB																														
CELLMARKERCURNVASARECTAENDOTHELIALCELLECVR																														
CELLMARKERCURNVONECONOMONEURONVEN																														
CELLMARKERCURNYOLKSACDERIVEDMYELOIDBIASED…																														
CELLMARKEREXP_ACINAR_CELLS																														
CELLMARKEREXP_ADRENERGIC_NEURONS																														
CELLMARKEREXP_AIRWAY_EPITHELIAL_CELLS																														
CELLMARKEREXP_AIRWAY_SMOOTH_MUSCLE_CELLS																														
CELLMARKEREXP_ALPHA_CELLS																														
CELLMARKEREXP_ANTERIOR_PITUITARY_GLAND_CE…																														
CELLMARKEREXP_ASTROCYTES																														
CELLMARKEREXP_B_CELLS																														
CELLMARKEREXP_B_CELLS_MEMORY																														
CELLMARKEREXP_B_CELLS_NAIVE																														
CELLMARKEREXP_BASAL_CELLS																														
CELLMARKEREXP_BASOPHILS																														
CELLMARKEREXP_BERGMANN_GLIA																														
CELLMARKEREXP_BETA_CELLS																														
CELLMARKEREXP_CAJAL_RETZIUS_CELLS																														
CELLMARKEREXP_CHOLANGIOCYTES																														
CELLMARKEREXP_CHOLINERGIC_NEURONS																														
CELLMARKEREXP_CHONDROCYTES																														
CELLMARKEREXP_DELTA_CELLS																														
CELLMARKEREXP_DISTAL_TUBULE_CELLS																														
CELLMARKEREXP_DUCTAL_CELLS																														
CELLMARKEREXP_EMBRYONIC_STEM_CELLS																														
CELLMARKEREXP_ENTERIC_NEURONS																														
CELLMARKEREXP_ENTEROENDOCRINE_CELLS																														
CELLMARKEREXP_EPENDYMAL_CELLS																														
CELLMARKEREXP_EPSILON_CELLS																														
CELLMARKEREXP_ERYTHROID_LIKE_AND_ERYTHROI…																														
CELLMARKEREXP_GAMMA_DELTA_T_CELLS																														
CELLMARKEREXP_GOBLET_CELLS																														
CELLMARKEREXP_HEMANGIOBLASTS																														
CELLMARKEREXP_HEMATOPOIETIC_STEM_CELLS																														
CELLMARKEREXP_HEPATIC_STELLATE_CELLS																														
CELLMARKEREXP_HIS_BUNDLE_CELLS																														
CELLMARKEREXP_IMMATURE_NEURONS																														
CELLMARKEREXP_JUXTAGLOMERULAR_CELLS																														
CELLMARKEREXP_KERATINOCYTES																														
CELLMARKEREXP_KIDNEY_PROGENITOR_CELLS																														
CELLMARKEREXP_KUPFFER_CELLS																														
CELLMARKEREXP_LEYDIG_CELLS																														
CELLMARKEREXP_LOOP_OF_HENLE_CELLS																														
CELLMARKEREXP_LUMINAL_EPITHELIAL_CELLS																														
CELLMARKEREXP_MAMMARY_EPITHELIAL_CELLS																														
CELLMARKEREXP_MEGAKARYOCYTES																														
CELLMARKEREXP_MELANOCYTES																														
CELLMARKEREXP_MESANGIAL_CELLS																														
CELLMARKEREXP_MESOTHELIAL_CELLS																														
CELLMARKEREXP_MICROFOLD_CELLS																														
CELLMARKEREXP_MICROGLIA																														
CELLMARKEREXP_MONOCYTES																														
CELLMARKEREXP_MULLER_CELLS																														
CELLMARKEREXP_MYOBLASTS																														
CELLMARKEREXP_MYOCYTES																														
CELLMARKEREXP_MYOFIBROBLASTS																														
CELLMARKEREXP_NEUROBLASTS																														
CELLMARKEREXP_NEUTROPHILS																														
CELLMARKEREXP_NK_CELLS																														
CELLMARKEREXP_NORADRENERGIC_NEURONS																														
CELLMARKEREXP_OLIGODENDROCYTES																														
CELLMARKEREXP_OSTEOBLASTS																														
CELLMARKEREXP_OSTEOCLAST_PRECURSOR_CELLS																														
CELLMARKEREXP_OSTEOCLASTS																														
CELLMARKEREXP_OSTEOCYTES																														
CELLMARKEREXP_PANETH_CELLS																														
CELLMARKEREXP_PARATHYROID_CHIEF_CELLS																														
CELLMARKEREXP_PERICYTES																														
CELLMARKEREXP_PHOTORECEPTOR_CELLS																														
CELLMARKEREXP_PLASMA_CELLS																														
CELLMARKEREXP_PLASMACYTOID_DENDRITIC_CELLS																														
CELLMARKEREXP_PODOCYTES																														
CELLMARKEREXP_PRINCIPAL_CELLS																														
CELLMARKEREXP_PROXIMAL_TUBULE_CELLS																														
CELLMARKEREXP_PULMONARY_ALVEOLAR_TYPE_I_C…																														

Table S5: Cell state marker gene list

CELLMARKERCAN_ACINAR_CELLS																														
CELLMARKEREXP_PULMONARY_ALVEOLAR_TYPE_II_…																														
CELLMARKEREXP_PURKINJE_FIBER_CELLS																														
CELLMARKEREXP_PURKINJE_NEURONS																														
CELLMARKEREXP_PYRAMIDAL_CELLS																														
CELLMARKEREXP_RETICULOCYTES																														
CELLMARKEREXP_RETINAL_GANGLION_CELLS																														
CELLMARKEREXP_SALIVARY_MUCOUS_CELLS																														
CELLMARKEREXP_SATELLITE_CELLS																														
CELLMARKEREXP_SCHWANN_CELLS																														
CELLMARKEREXP_SEROTONERGIC_NEURONS																														
CELLMARKEREXP_SERTOLI_CELLS																														
CELLMARKEREXP_SMOOTH_MUSCLE_CELLS																														
CELLMARKEREXP_SPERMATOZOA																														
CELLMARKEREXP_STROMAL_CELLS																														
CELLMARKEREXP_T_CELLS																														
CELLMARKEREXP_T_CELLS_NAIVE																														
CELLMARKEREXP_T_CYTOTOXIC_CELLS																														
CELLMARKEREXP_T_HELPER_CELLS																														
CELLMARKEREXP_T_MEMORY_CELLS																														
CELLMARKEREXP_TRICHOCYTES																														
CELLMARKEREXP_TRIGEMINAL_NEURONS																														
CELLMARKEREXP_TROPHOBLAST_CELLS																														
CELLMARKEREXP_TROPHOBLAST_PROGENITOR_CELLS																														
CELLMARKEREXP_TUFT_CELLS																														
CELLMARKEREXP_VASCULAR_SMOOTH_MUSCLE_CELLS																														
CELLMARKEREXPENDOTHELIALCELLSAORTA																														
CELLMARKEREXPNEURALSTEMPRECURSORCELLS																														
CELLMARKERMSIGDB_AIZARANI_LIVER_C12_NK_NK…																														
CELLMARKERMSIGDB_AIZARANI_LIVER_C22_RESID…																														
CELLMARKERMSIGDB_AIZARANI_LIVER_C28_NK_NK…																														
CELLMARKERMSIGDB_AIZARANI_LIVER_C29_MVECS…	STC1	SOX7	CAV2	TUBB4B	PTGIS	HSPB1	WARS1	BMP4	A2M	LMO2	TAGLN2	TUBA1A	F8	FOSB	JMJD1C	TIMP2	ANXA2	ANXA1	ATP1A1	PLTP	DOCK9	MDK	SELP	GNG11	PALM	GPX3	RAMP3	NFIA	GALNT…	DUSP6
CELLMARKERMSIGDB_AIZARANI_LIVER_C30_HEPAT…	NTRK3	RN7SL…	GABPB2	TSHZ2	MTMR9	HEMK1	VSTM4	DSG3	PPP2R…	ARMC9	NUGGC	ABLIM3	HAO2	PTPN3	MEG8	ACADVL	ASL	CLNK	GCKR	IDH2	NLRP12	SEMA3E	AASS	HGFAC	LMOD3	PPARA	PCK1	SH3PX…	ENPP1	CYB56…
CELLMARKERMSIGDB_AIZARANI_LIVER_C8_RESIDE…																														
CELLMARKERMSIGDB_AIZARANI_LIVER_C9_LSECS_1	NRP2	RASIP1	TACC1	HIF1A	NOSTR…	MAP1B	SLC7A8	DUSP5	CUL4B	EFNB2	EGFL7	PREX2	ERG	ACTN1	HIC1	FZD4	SLC27…	HERC1	UACA	CD36	TP53I…	ARHGE…	DAAM1	FSCN1	CALU	MYO10	DUSP6	CEMIP2	PLEKH…	GATA4
CELLMARKERMSIGDB_BUSSLINGER_DUODENAL_BCHE…																														
CELLMARKERMSIGDB_BUSSLINGER_DUODENAL_DIFF…	CDCA7	MT1X	COX5A	MT1E	NCL	EEF1G	VDR	KRT19	RPL13	RPS4X	CKS2	PAICS	RPL3	RPL31	CCND2	TOMM40	RPL37	KLF5	LSM4	DBI	FAM12…	SET	TUFM	RPL26	EEF1A1	RPL36	RPL34	ILF3	MTHFD1	CD9
CELLMARKERMSIGDB_BUSSLINGER_DUODENAL_EC_C…																														
CELLMARKERMSIGDB_BUSSLINGER_DUODENAL_IMMU…	STK10	THEMIS	NAP1L1	PDE7A	ID2	ZYX	HLA-A	ABI3	ITM2A	PRKACB	LTB	CLEC2D	TSC22…	CXCR4	APBB1…	ANXA6	PGC	GNG2	SEPTI…	EVI2B	GNLY	RPS27	FLNA	STOM	PRF1	SARAF	PFN1	MDFIC	CCR5	CYTIP
CELLMARKERMSIGDB_BUSSLINGER_DUODENAL_STEM…	CCND2	CERS6	FERMT1	AGR2	RPL26	HSPE1	RPL22…	DACH1	RPS5	ACTN1	FBL	EIF1AX	HNRNP…	RPL32	EDN1	RPL4	NCOA7	RPL35	LAMB1	CD44	HSP90…	CES1	BMP4	TXN	TM4SF1	DUT	FOS	RPS7	MLXIP	TSC22…
CELLMARKERMSIGDB_BUSSLINGER_DUODENAL_TUFT…																														
CELLMARKERMSIGDB_BUSSLINGER_ESOPHAGEAL_EA…																														
CELLMARKERMSIGDB_BUSSLINGER_ESOPHAGEAL_PR…																														
CELLMARKERMSIGDB_BUSSLINGER_GASTRIC_CHIEF…																														
CELLMARKERMSIGDB_BUSSLINGER_GASTRIC_G_CEL…																														
CELLMARKERMSIGDB_BUSSLINGER_GASTRIC_ISTHM…	DHFR	YWHAH	AGR3	PLA2G…	LAD1	TENT5A	TPX2	MYO5C	RASSF6	AKAP1	BLVRB	ITGB4	FAM17…	RRBP1	KLF4	RASEF	TLN2	EIF3J	HYOU1	CTNNA1	UGDH	MAPK3	LMO7	REP15	HNRNP…	PTMA	MCU	TLCD2	S100A…	GALE
CELLMARKERMSIGDB_BUSSLINGER_GASTRIC_NECK_…																														
CELLMARKERMSIGDB_BUSSLINGER_GASTRIC_OXYNT…																														
CELLMARKERMSIGDB_BUSSLINGER_GASTRIC_PREZY…																														
CELLMARKERMSIGDB_BUSSLINGER_GASTRIC_REG3A…																														
CELLMARKERMSIGDB_CUI_DEVELOPING_HEART_5TH…																														
CELLMARKERMSIGDB_CUI_DEVELOPING_HEART_5TH…																														
CELLMARKERMSIGDB_CUI_DEVELOPING_HEART_C1_…																														
CELLMARKERMSIGDB_CUI_DEVELOPING_HEART_C2_…																														
CELLMARKERMSIGDB_CUI_DEVELOPING_HEART_COM…																														
CELLMARKERMSIGDB_CUI_DEVELOPING_HEART_COR…																														
CELLMARKERMSIGDB_CUI_DEVELOPING_HEART_END…																														
CELLMARKERMSIGDB_CUI_DEVELOPING_HEART_LEF…																														
CELLMARKERMSIGDB_CUI_DEVELOPING_HEART_RIG…																														
CELLMARKERMSIGDB_CUI_DEVELOPING_HEART_SMO…																														
CELLMARKERMSIGDB_DESCARTES_FETAL_ADRENAL_…																														
CELLMARKERMSIGDB_DESCARTES_FETAL_CEREBELL…																														
CELLMARKERMSIGDB_DESCARTES_FETAL_CEREBELL…																														
CELLMARKERMSIGDB_DESCARTES_FETAL_CEREBELL…	MYO1F	SLC7A7	CPVL	P3H2	ATP8B4	MKNK1	AOAH	TLR1	CSF1	WDFY4	RHBDF2	GLDN	MAF	ARHGA…	B3GNT5	GAL3S…	CD83	RIN3	ALPK1	CTSS	RTTN	CD226	PPARG	ABCC4	IFNGR1	LCP2	ICAM1	IGF1	LRRK1	HAVCR2
CELLMARKERMSIGDB_DESCARTES_FETAL_CEREBELL…																														
CELLMARKERMSIGDB_DESCARTES_FETAL_CEREBELL…																														
CELLMARKERMSIGDB_DESCARTES_FETAL_CEREBELL…																														
CELLMARKERMSIGDB_DESCARTES_FETAL_CEREBELL…	PCDH18	TTN	NKD2	MMRN2	ETS1	C1orf…	IGF2	VAMP5	GNG11	SLC7A1	CNN2	SLC7A…	PODXL	PTPRB	TGFB1…	FLT4	RRAS	VWA1	CLIC5	TFRC	ISM1	RBPMS	TAGLN2	SLCO2…	EPAS1	ABCC9	TRPC6	ZNF503	RASL12	PELO
CELLMARKERMSIGDB_DESCARTES_FETAL_CEREBRUM…																														
CELLMARKERMSIGDB_DESCARTES_FETAL_CEREBRUM…																														
CELLMARKERMSIGDB_DESCARTES_FETAL_CEREBRUM…	CAVIN…	CNST	LCA5	CCRL2	CCNJL	AK9	OR7E1…	GP6	ADCYA…	TSPAN…	PRR29	DIAPH1	RNF141	GAS2L1	IL27RA	CDHR1	NRGN	DGKD	C2orf…	SLC10…	NT5C3A	LINC0…	MREG	H1-2	HEXIM1	RAB37	GABRE	SLC50…	NENF	MPIG6B
CELLMARKERMSIGDB_DESCARTES_FETAL_CEREBRUM…	DOCK2	NFATC2	LRMDA	ADAM28	LRRC39	SYNDI…	TRPM2	PLVAP	OTULI…	CTSB	CSF1R	RNF149	LY86	PLCG2	GCNT2	ARHGA…	TLR4	IL13R…	MGAT4A	ELL2	MANBA	SNX29	P2RX4	RPS10…	ATG7	DIAPH2	VSIG4	ZNF710	PLD1	GNG7
CELLMARKERMSIGDB_DESCARTES_FETAL_CEREBRUM…																														
CELLMARKERMSIGDB_DESCARTES_FETAL_CEREBRUM…																														
CELLMARKERMSIGDB_DESCARTES_FETAL_CEREBRUM…	PHLDB2	CLEC3B	CCM2L	APLN	COL4A1	COL1A2	DLL4	TMEM2…	PLXDC1	EHD2	ABCB1	S1PR3	ITGA1	CLIC5	ATP8B1	IFITM3	FRZB	RHOC	SLC39…	ARAP3	ANXA1	C1orf…	LGALS1	LMO2	SLC12…	CLEC1…	RRAS	APLNR	SPARC	CYYR1
CELLMARKERMSIGDB_DESCARTES_FETAL_EYE_ASTR…																														
CELLMARKERMSIGDB_DESCARTES_FETAL_EYE_GANG…																														
CELLMARKERMSIGDB_DESCARTES_FETAL_EYE_LENS…																														
CELLMARKERMSIGDB_DESCARTES_FETAL_EYE_PHOT…																														
CELLMARKERMSIGDB_DESCARTES_FETAL_EYE_RETI…																														
CELLMARKERMSIGDB_DESCARTES_FETAL_EYE_SMOO…																														
CELLMARKERMSIGDB_DESCARTES_FETAL_EYE_STRO…																														
CELLMARKERMSIGDB_DESCARTES_FETAL_EYE_VASC…																														
CELLMARKERMSIGDB_DESCARTES_FETAL_HEART_EL…																														
CELLMARKERMSIGDB_DESCARTES_FETAL_HEART_EN…																														
CELLMARKERMSIGDB_DESCARTES_FETAL_HEART_LY…																														
CELLMARKERMSIGDB_DESCARTES_FETAL_HEART_SC…																														
CELLMARKERMSIGDB_DESCARTES_FETAL_HEART_SM…																														
CELLMARKERMSIGDB_DESCARTES_FETAL_HEART_ST…																														
CELLMARKERMSIGDB_DESCARTES_FETAL_HEART_VI…																														
CELLMARKERMSIGDB_DESCARTES_FETAL_INTESTIN…																														
CELLMARKERMSIGDB_DESCARTES_FETAL_INTESTIN…																														
CELLMARKERMSIGDB_DESCARTES_FETAL_INTESTIN…																														
CELLMARKERMSIGDB_DESCARTES_FETAL_INTESTIN…																														
CELLMARKERMSIGDB_DESCARTES_FETAL_INTESTIN…																														
CELLMARKERMSIGDB_DESCARTES_FETAL_INTESTIN…																														
CELLMARKERMSIGDB_DESCARTES_FETAL_KIDNEY_M…																														
CELLMARKERMSIGDB_DESCARTES_FETAL_KIDNEY_U…	ITGB4	TSPAN1	CALB1	ATP10B	CPXM2	MYEOV	CTXND1	GPX2	ITGB6	WNT9B	FAM3B	SLC38…	MAOA	SMIM22	OVOL1	RNF128	TTC6	FOXA1	CLDN8	FA2H	IRF6	PRDM1…	LINC0…	NUDT1…	FRK	CBLC	TMEM1…	VWA5B1	KRT15	FAM83B
CELLMARKERMSIGDB_DESCARTES_FETAL_LIVER_ER…																														
CELLMARKERMSIGDB_DESCARTES_FETAL_LIVER_HE…																														
CELLMARKERMSIGDB_DESCARTES_FETAL_LIVER_HE…	C5	ADH6	ARG1	SERPI…	ATF5	DCDC2	GPAM	MT2A	MSMO1	FABP1	SCD	PEG10	ASS1	AGXT	ACADSB	FMO5	ASGR2	KIF6	FGL1	AK4	MT1X	TENM1	PLPPR1	ACSL4	ERRFI1	BDH1	TM7SF2	COL2A1	PEBP1	HISLA
CELLMARKERMSIGDB_DESCARTES_FETAL_LIVER_ME…	SEMA3B	COL4A6	RBFOX1	CYP4X1	ADAMT…	CBLN2	SEZ6L2	BASP1…	CPAMD8	CARNS1	PRDM6	RAB34	TMEM1…	DSC3	KLK11	LAMA3	CYSTM1	PROCR	LRP2	CNGB3	PTGFRN	HTRA1	PPL	CA9	MEOX2	ID4	ITGB8	HAS1	FZD7	KLF5
CELLMARKERMSIGDB_DESCARTES_FETAL_LUNG_BRO…																														
CELLMARKERMSIGDB_DESCARTES_FETAL_LUNG_CIL…	CCDC1…	CHST9	SPEF2	EFHC1	C4orf…	CFAP2…	ESRRG	LINC0…	ULK4	NEK11	SPATA…	C8orf…	PTPRT	TPPP3	CEP126	DRC1	TMEM1…	KIF6	TOGAR…	PIERC…	LRRC23	ALDH3…	PPP1R…	CFAP2…	CFAP53	IQCH	CAPSL	BBOF1	CFAP46	CCDC78
CELLMARKERMSIGDB_DESCARTES_FETAL_LUNG_LYM…																														

Table S5: Cell state marker gene list

CELLMARKERCAN_ACINAR_CELLS																														
CELLMARKERMSIGDB_DESCARTES_FETAL_LUNG_NEU…																														
CELLMARKERMSIGDB_DESCARTES_FETAL_LUNG_STR…																														
CELLMARKERMSIGDB_DESCARTES_FETAL_LUNG_VAS…																														
CELLMARKERMSIGDB_DESCARTES_FETAL_MUSCLE_L…																														
CELLMARKERMSIGDB_DESCARTES_FETAL_MUSCLE_S…																														
CELLMARKERMSIGDB_DESCARTES_FETAL_MUSCLE_S…																														
CELLMARKERMSIGDB_DESCARTES_FETAL_MUSCLE_V…																														
CELLMARKERMSIGDB_DESCARTES_FETAL_PANCREAS…																														
CELLMARKERMSIGDB_DESCARTES_FETAL_PANCREAS…																														
CELLMARKERMSIGDB_DESCARTES_FETAL_PANCREAS…																														
CELLMARKERMSIGDB_DESCARTES_FETAL_PANCREAS…																														
CELLMARKERMSIGDB_DESCARTES_FETAL_PANCREAS…																														
CELLMARKERMSIGDB_DESCARTES_FETAL_PANCREAS…																														
CELLMARKERMSIGDB_DESCARTES_FETAL_PANCREAS…																														
CELLMARKERMSIGDB_DESCARTES_FETAL_PLACENTA…																														
CELLMARKERMSIGDB_DESCARTES_FETAL_PLACENTA…																														
CELLMARKERMSIGDB_DESCARTES_FETAL_PLACENTA…																														
CELLMARKERMSIGDB_DESCARTES_FETAL_PLACENTA…																														
CELLMARKERMSIGDB_DESCARTES_FETAL_PLACENTA…																														
CELLMARKERMSIGDB_DESCARTES_FETAL_SPLEEN_E…																														
CELLMARKERMSIGDB_DESCARTES_FETAL_SPLEEN_S…																														
CELLMARKERMSIGDB_DESCARTES_FETAL_SPLEEN_V…																														
CELLMARKERMSIGDB_DESCARTES_FETAL_STOMACH_…	CFAP1…	FAM18…	DNAAF…	PPP1R…	FRMPD2	LRRC23	PPIL6	IQCH	C5orf…	ODF3B	MARCH…	TRAF3…	DRC3	CCDC40	ZNF474	NWD1	AK7	TTLL9	NRAD1	FAM21…	HOATZ	C6orf…	DYNLR…	VNN3P	ARHGA…	CCDC1…	ODAD4	CFAP69	CC2D2A	TTC21A
CELLMARKERMSIGDB_DESCARTES_FETAL_STOMACH_…																														
CELLMARKERMSIGDB_DESCARTES_FETAL_STOMACH_…																														
CELLMARKERMSIGDB_DESCARTES_FETAL_STOMACH_…																														
CELLMARKERMSIGDB_DESCARTES_FETAL_STOMACH_…																														
CELLMARKERMSIGDB_DESCARTES_FETAL_STOMACH_…																														
CELLMARKERMSIGDB_DESCARTES_FETAL_STOMACH_…																														
CELLMARKERMSIGDB_DESCARTES_FETAL_STOMACH_…																														
CELLMARKERMSIGDB_DESCARTES_FETAL_STOMACH_…																														
CELLMARKERMSIGDB_DESCARTES_FETAL_STOMACH_…																														
CELLMARKERMSIGDB_DESCARTES_FETAL_STOMACH_…																														
CELLMARKERMSIGDB_DESCARTES_FETAL_STOMACH_…																														
CELLMARKERMSIGDB_DESCARTES_FETAL_THYMUS_T…																														
CELLMARKERMSIGDB_DESCARTES_MAIN_FETAL_ACI…																														
CELLMARKERMSIGDB_DESCARTES_MAIN_FETAL_ADR…																														
CELLMARKERMSIGDB_DESCARTES_MAIN_FETAL_AST…																														
CELLMARKERMSIGDB_DESCARTES_MAIN_FETAL_BIP…																														
CELLMARKERMSIGDB_DESCARTES_MAIN_FETAL_BRO…																														
CELLMARKERMSIGDB_DESCARTES_MAIN_FETAL_CAR…																														
CELLMARKERMSIGDB_DESCARTES_MAIN_FETAL_CIL…	TTC30B	TTC30A	OSBPL6	DUSP19	DNAH7	E2F3P2	ANKRD…	KIAA2…	MDH1B	SPAG16	CATIP	CFAP65	GLB1L	TUBA4B	DAW1	FBXO36	IQCA1	TRAF3…	ANKMY1	CROCC2	CNTN4…	SRGAP…	EFHB	NEK10	DLEC1	TTC21A	ULK4	CCDC13	KIF9	P4HTM
CELLMARKERMSIGDB_DESCARTES_MAIN_FETAL_DUC…																														
CELLMARKERMSIGDB_DESCARTES_MAIN_FETAL_END…																														
CELLMARKERMSIGDB_DESCARTES_MAIN_FETAL_ENS…																														
CELLMARKERMSIGDB_DESCARTES_MAIN_FETAL_EPI…																														
CELLMARKERMSIGDB_DESCARTES_MAIN_FETAL_GAN…																														
CELLMARKERMSIGDB_DESCARTES_MAIN_FETAL_GOB…																														
CELLMARKERMSIGDB_DESCARTES_MAIN_FETAL_HEM…																														
CELLMARKERMSIGDB_DESCARTES_MAIN_FETAL_HOR…																														
CELLMARKERMSIGDB_DESCARTES_MAIN_FETAL_INH…																														
CELLMARKERMSIGDB_DESCARTES_MAIN_FETAL_INH…																														
CELLMARKERMSIGDB_DESCARTES_MAIN_FETAL_INT…																														
CELLMARKERMSIGDB_DESCARTES_MAIN_FETAL_ISL…																														
CELLMARKERMSIGDB_DESCARTES_MAIN_FETAL_LYM…																														
CELLMARKERMSIGDB_DESCARTES_MAIN_FETAL_MES…																														
CELLMARKERMSIGDB_DESCARTES_MAIN_FETAL_MET…																														
CELLMARKERMSIGDB_DESCARTES_MAIN_FETAL_MYE…																														
CELLMARKERMSIGDB_DESCARTES_MAIN_FETAL_NEU…																														
CELLMARKERMSIGDB_DESCARTES_MAIN_FETAL_OLI…																														
CELLMARKERMSIGDB_DESCARTES_MAIN_FETAL_PDE…																														
CELLMARKERMSIGDB_DESCARTES_MAIN_FETAL_PDE…	GUSBP3	THBS4…	SERIN…	ARSK	LINC0…	PTMAP2	ANKHD1	SRA1	RARS1	FOXI1	ZNF34…	DUSP22	SOX4	H1-4	ZNF165	ZNF311	ILRUN	SRSF3	TAF8	CDC5L	CD2AP	MANEA…	PNISR…	USP45	PDSS2	HSF2	TRMT11	L3MBT…	PEX7	KATNA1
CELLMARKERMSIGDB_DESCARTES_MAIN_FETAL_RET…																														
CELLMARKERMSIGDB_DESCARTES_MAIN_FETAL_SAT…																														
CELLMARKERMSIGDB_DESCARTES_MAIN_FETAL_SCH…																														
CELLMARKERMSIGDB_DESCARTES_MAIN_FETAL_SLC…	SRFBP1	TH2LC…	PKD2L…	PKD2L2	SNORD…	HARS2	PCDHG…	LINC0…	SIMC1	LYRM4	CAGE1	ABT1	H2AC17	ZSCAN…	ZKSCA…	CCHCR1	FKBPL	BAK1	BRPF3	SAYSD1	SDHAF4	GPRC6A	MED23	RMND1	SLC22…	BRAT1	RP9P	GTF2I…	TYW1	PMS2P4
CELLMARKERMSIGDB_DESCARTES_MAIN_FETAL_SMO…																														
CELLMARKERMSIGDB_DESCARTES_MAIN_FETAL_STE…																														
CELLMARKERMSIGDB_DESCARTES_MAIN_FETAL_SYM…																														
CELLMARKERMSIGDB_DESCARTES_MAIN_FETAL_UNI…																														
CELLMARKERMSIGDB_DESCARTES_MAIN_FETAL_URE…																														
CELLMARKERMSIGDB_DESCARTES_MAIN_FETAL_VAS…																														
CELLMARKERMSIGDB_DESCARTES_MAIN_FETAL_VIS…																														
CELLMARKERMSIGDB_DURANTE_ADULT_OLFACTORY_…																														
CELLMARKERMSIGDB_DURANTE_ADULT_OLFACTORY_…																														
CELLMARKERMSIGDB_DURANTE_ADULT_OLFACTORY_…																														
CELLMARKERMSIGDB_DURANTE_ADULT_OLFACTORY_…																														
CELLMARKERMSIGDB_DURANTE_ADULT_OLFACTORY_…																														
CELLMARKERMSIGDB_DURANTE_ADULT_OLFACTORY_…																														
CELLMARKERMSIGDB_DURANTE_ADULT_OLFACTORY_…																														
CELLMARKERMSIGDB_DURANTE_ADULT_OLFACTORY_…																														
CELLMARKERMSIGDB_DURANTE_ADULT_OLFACTORY_…																														
CELLMARKERMSIGDB_DURANTE_ADULT_OLFACTORY_…																														
CELLMARKERMSIGDB_DURANTE_ADULT_OLFACTORY_…																														
CELLMARKERMSIGDB_DURANTE_ADULT_OLFACTORY_…																														
CELLMARKERMSIGDB_DURANTE_ADULT_OLFACTORY_…																														
CELLMARKERMSIGDB_DURANTE_ADULT_OLFACTORY_…																														
CELLMARKERMSIGDB_DURANTE_ADULT_OLFACTORY_…																														
CELLMARKERMSIGDB_DURANTE_ADULT_OLFACTORY_…																														
CELLMARKERMSIGDB_FAN_EMBRYONIC_CTX_ASTROC…																														
CELLMARKERMSIGDB_FAN_EMBRYONIC_CTX_BIG_GR…	COBLL1	FOXQ1	PECAM1	EBF1	SLC12…	CYTOR	TGM2	PPP1R…	NOSTR…	SLC38…	MMP28	SLC16…	SLC3A2	PLCE1	CA2	CD93	TM4SF1	MYCT1	RRAS	CYYR1	UNC5B	SLC9A…	ATP1A2	ZNF366	TESC	DCN	ECE1	DOCK6	IFITM1	NID2
CELLMARKERMSIGDB_FAN_EMBRYONIC_CTX_BIG_GR…	ARHGD…	ATP1A3	CHST2	EPDR1	ATP5M…	EPPK1	B4GAT1	DEFB1	ALDOA	CPM	SNAP25	NUPR1	ANTKMT	COX5B	CLSTN1	CYP3A5	NEFL	CKMT1A	PCSK1N	CNGA3	SLC35…	FDPS	KIAA1…	ATF5	HPCAL1	TSTD1	MAP1L…	CD164…	SCARA3	TUSC3
CELLMARKERMSIGDB_FAN_EMBRYONIC_CTX_BIG_GR…																														
CELLMARKERMSIGDB_FAN_EMBRYONIC_CTX_BIG_GR…	CXCL16	CREG1	RNASE6	LAT2	TFEC	LY96	GADD4…	HAVCR2	IER3	MKNK1	LST1	FCER1G	GSN	LINC0…	CD4	CPED1	INPP5D	NCF4	CYTH4	SLC7A7	HCK	CTSB	NPL	HMOX1	STAB1	MILR1	BIN2	ATP8B4	CCL4	CPVL
CELLMARKERMSIGDB_FAN_EMBRYONIC_CTX_BRAIN_…	CNN3	HAPLN3	LEF1	IFITM1	TSPAN5	PAM	EFNA1	SMAD1	MYLK2	CAV2	IGFBP4	HYAL2	RHOC	SLC39…	INSR	NECTI…	LIMS2	NPDC1	ECE1	MYO6	YES1	CAPN2	SCARB1	SPTBN1	SPOCK2	ARHGA…	LAMB1	CAVIN1	LAMC1	TAGLN2
CELLMARKERMSIGDB_FAN_EMBRYONIC_CTX_BRAIN_…	GJC1	TJP1	PTGDR2	SMOC2	EPS8	HES1	S1PR3	F2R	TBX2	ANPEP	SPON2	ADORA…	EBF1	SYNE2	SEPTI…	ADA	PGF	ARHGE…	CFAP20	RBMS3	FLNA	SLC38…	PDE7B	PCOLCE	PTEN	PAPSS2	COL5A2	PPP1R…	PARVA	DMD
CELLMARKERMSIGDB_FAN_EMBRYONIC_CTX_EX_1_E…																														
CELLMARKERMSIGDB_FAN_EMBRYONIC_CTX_IN_2_I…																														
CELLMARKERMSIGDB_FAN_EMBRYONIC_CTX_IN_3_I…																														
CELLMARKERMSIGDB_FAN_EMBRYONIC_CTX_IN_5_I…																														
CELLMARKERMSIGDB_FAN_EMBRYONIC_CTX_IN_6_I…																														
CELLMARKERMSIGDB_FAN_EMBRYONIC_CTX_MICROG…																														
CELLMARKERMSIGDB_FAN_EMBRYONIC_CTX_OLIG	APLN	PLLP	C1orf…	PRKCSH	SEPTI…	GRIN2A	CFAP95	SERPI…	CD9	ABLIM2	ABHD1…	TRIM2	LINC0…	LARP6	CA14	GNAI1	MTUS1	KIAA0…	ST6GA…	EPB41…	PRR18	ATP1B1	DHCR24	MAN2A1	FAM10…	SH3TC2	FAM10…	NIPAL3	MAPK8…	VSTM2B
CELLMARKERMSIGDB_FAN_EMBRYONIC_CTX_OPC																														

Table S5: Cell state marker gene list

CELLMARKERCAN_ACINAR_CELLS																														
CELLMARKERMSIGDB_FAN_OVARY_CL1_GPRC5A_TNF…	EPS8	NOP56	HNRNP…	DDX5	GADD4…	CPE	MRTO4	STAR	CTNNA…	TCP1	NUDT4	CCT2	IGFBP5	MRFAP1	SERTA…	PRNP	ACTG1	TNFRS…	PLOD2	RAN	HNRNPF	GPATC…	CCT5	CCDC47	MAP1B	LDHA	UBAP1	H2AZ1	UBC	NXT1
CELLMARKERMSIGDB_FAN_OVARY_CL11_MURAL_GRA…	HMGCR	IGF1R	GRIK1	HSPA4	CENAT…	NR5A2	SLC25…	LRRC8C	AHSA1	EPS8	IVNS1…	DST	KDM5A	TNNI3	CKS2	MT-ND2	RHOBT…	GASK1B	EIF5	SLC16…	AMOTL2	CACYBP	HUWE1	DNAJB6	VIM	SLC5A3	DNAJA4	YPEL2	BMPR2	RHOB
CELLMARKERMSIGDB_FAN_OVARY_CL13_MONOCYTE_…	CFLAR	HCST	CXCR4	NPC2	GSTO1	ATP6V…	LCP1	IGSF6	CD14	ANXA5	ASAH1	CARD16	GLIPR1	MGAT1	NFKB1	VSIG4	NFKBIA	PDE4D…	ADA	GNA15	FNIP2	FGL2	RILPL2	CD37	PLIN2	FXYD5	ATP6V…	TNFRS…	CLEC7A	CD53
CELLMARKERMSIGDB_FAN_OVARY_CL14_MATURE_SM…	HES1	LGALS1	EHD2	KANK2	CYB5R3	SOCS3	HES4	A2M	SPARC	CAV2	MTHFD2	LHFPL6	RASL12	CCN1	PKIG	NDUFA4	CD44	ILK	ACTB	EBF1	PTK2	PDLIM7	CSRP2	MAFB	ASAH1	FXYD6	NDRG2	CBX6	INTS6	GPX3
CELLMARKERMSIGDB_FAN_OVARY_CL15_SMALL_ANT…	ATG101	LAPTM…	MYO10	PPP2CA	IGF2B…	ZNF814	TCEA3	CLK1	KPNA2	CKB	PTS	HSPA1B	CTNNA…	CFAP20	CSRP2	SAT2	ZFAND5	MAFF	SRSF9	METAP2	CCND2	GRIK1	HMGCS1	TNPO3	HNRNP…	ZNF711	CCN2	CD59	HSPA1A	CTSV
CELLMARKERMSIGDB_FAN_OVARY_CL18_B_LYMPHOC…	GPX1	CTSB	HSPA1B	JAML	TGFB1	IRF4	ARL6I…	PPP1R…	FYTTD1	GPSM3	SCT	ATF5	CD4	CD68	SH3BP5	IL2RG	FABP5	EGLN3	CD53	PTCRA	SPCS1	GRB2	HES4	LCP1	MYBL2	SQSTM1	NOP58	SMIM14	POLB	TOP1
CELLMARKERMSIGDB_FAN_OVARY_CL2_PUTATIVE_E…																														
CELLMARKERMSIGDB_FAN_OVARY_CL5_HEALTHY_SE…	TRA2B	IER2	CKS2	PXDN	TMEM98	SAT2	PDGFRB	UBC	OAF	ANKRD…	ISG15	NR2F2	PLOD1	SULF2	NECTI…	RCN2	OSTC	TSHZ2	RHOBT…	C7	LDHB	SNHG19	MRPL18	EXTL2	PTK7	HSPH1	PEG3	LAMC1	CITED2	ADM
CELLMARKERMSIGDB_FAN_OVARY_CL6_PUTATIVE_E…	EFEMP2	SNAI2	SGCE	FSTL1	TXNIP	HEXIM1	SLC16…	PPM1K	RGS2	CLEC1…	CSRP1	CRYAB	LTBP4	EID1	ADH5	TIMP2	GYPC	TMEM1…	MMP14	GSN	SH3BG…	KLF10	CPXM1	GSTM5	DDIT4	NDN	PLD3	TGFB1…	FN1	FXYD1
CELLMARKERMSIGDB_FAN_OVARY_CL8_MATURE_CUM…	PTRHD1	MDK	RPS25	MT-ND…	APOBE…	MGARP	CBX3	LSM7	H1-0	PHF6	BEX5	ITGB1	NDUFS5	HDHD3	EPDR1	TSPAN7	PRR15	ST6GA…	UQCR11	SCD	SYNE2	PHEX	SEM1	PRDX2	DSP	NDUFB7	GREM1	RIMS4	ARPC2	HS6ST1
CELLMARKERMSIGDB_FAN_OVARY_CL9_PUTATIVE_A…	ICAM1	LRRC8C	PHACT…	PNP	NFKBIA	YBX3	GIMAP4	NFIB	LMNA	ABL2	GBP2	B2M	ARID5A	HLA-B	SASH1	ITM2A	CCDC50	PARP14	DEPP1	NCK1	CRIM1	CD74	LIMS1	ELF1	NPDC1	HLA-C	UBC	CDC42…	DNAJA1	CHMP4B
CELLMARKERMSIGDB_GAO_ESOPHAGUS_25W_C1_CIL…	CEP97	CES1	CES4A	CFAP1…	CFAP2…	CFAP43	CFAP44	CFAP46	CFAP47	CFAP52	CFAP53	CFAP54	CFAP57	CFAP61	CFAP69	CFP	CHST11	CHST9	CLDN16	COL4A5	CP	CPEB1	CROCC	CSMD1	CSPP1	CST6	CCN2	CXXC4	DALRD3	DCDC1
CELLMARKERMSIGDB_GAO_ESOPHAGUS_25W_C2_KRT…																														
CELLMARKERMSIGDB_GAO_ESOPHAGUS_25W_C3_FGF…																														
CELLMARKERMSIGDB_GAO_LARGE_INTESTINE_24W_…																														
CELLMARKERMSIGDB_GAO_LARGE_INTESTINE_24W_…																														
CELLMARKERMSIGDB_GAO_LARGE_INTESTINE_24W_…	FCGR3A	FERMT3	FES	FGD2	FGL2	FLI1	FMNL1	FPR1	FYB1	GATM	GBP5	GFRA2	GGTA1	GLA	GM2A	GMFG	GNL3	GNPDA1	GPR132	GPR13…	GPR34	GPR65	GPSM3	HAVCR2	HCAR2	HCAR3	HCK	HCLS1	HCST	HLA-D…
CELLMARKERMSIGDB_GAO_LARGE_INTESTINE_24W_…																														
CELLMARKERMSIGDB_GAO_LARGE_INTESTINE_24W_…																														
CELLMARKERMSIGDB_GAO_LARGE_INTESTINE_24W_…																														
CELLMARKERMSIGDB_GAO_LARGE_INTESTINE_ADUL…	GDF11	GFRA3	GJA4	GLIS3	GLYAT…	GNAO1	GNAZ	GNG2	GOLGA…	GPR173	GPRAS…	GPRIN1	TAMAL…	GSG1	GULP1	HDAC9	HOPX	IGFBP3	INSL5	INSM1	ISYNA1	JAZF1	KCNC1	KCNH2	KCNH6	KCNH8	KCNJ3	KCNK3	KCNMB2	KIAA0…
CELLMARKERMSIGDB_GAO_LARGE_INTESTINE_ADUL…	INKA2…	RIPOR2	GARIN…	FAM86…	FAM89A	FFAR2	FILIP…	IL6ST…	FOXL2…	FOXP2	FPR1	GAS6-…	GATA2	GBGT1	GFI1	GINS1	GJC1	GNA12	GOLGA…	GPC6	CMKLR…	GREM1	GRIP2	GSDMA	GTF2I…	GUCY1…	HACE1	HAR1A	HAUS8	HCAR2
CELLMARKERMSIGDB_GAO_LARGE_INTESTINE_ADUL…																														
CELLMARKERMSIGDB_GAO_LARGE_INTESTINE_ADUL…	ELMO1	EMB	EMP3	ENO2	ERN1	EVI2A	EVI2B	RFLNB	FAM11…	HYCC1	NIBAN1	CYRIA	CIBAR2	FCGR2B	FCMR	FCRL2	FCRL5	FER1L4	FGD2	FGL2	FICD	FLI1	FLT3LG	FRMD6	FXYD5	FYB1	FYN	GABRR2	GAMT	GIMAP4
CELLMARKERMSIGDB_GAO_LARGE_INTESTINE_ADUL…	COL5A2	COL5A3	COL6A1	COL6A2	COL6A3	COLEC…	COX7A1	CPAMD8	CPED1	CRIP2	CRISP…	CSF1	CSF3R	CSMD3	CSPG4	CTSF	CXCL12	CXCL14	CYBRD1	CYGB	CYP27…	CYYR1	DACT1	DCHS1	DCN	DKK3	DLC1	DMD	DNM3OS	DPYSL3
CELLMARKERMSIGDB_GAO_SMALL_INTESTINE_24W_…																														
CELLMARKERMSIGDB_GAO_SMALL_INTESTINE_24W_…																														
CELLMARKERMSIGDB_GAO_SMALL_INTESTINE_24W_…																														
CELLMARKERMSIGDB_GAO_SMALL_INTESTINE_24W_…																														
CELLMARKERMSIGDB_GAO_SMALL_INTESTINE_24W_…																														
CELLMARKERMSIGDB_GAO_STOMACH_24W_C1_PROCR…																														
CELLMARKERMSIGDB_GAO_STOMACH_24W_C2_TFF2P…																														
CELLMARKERMSIGDB_GAO_STOMACH_24W_C4_PARIE…																														
CELLMARKERMSIGDB_GAO_STOMACH_24W_C5_PUTAT…																														
CELLMARKERMSIGDB_GAUTAM_EYE_IRIS_CILIARY_…	EIF2A…	PRDX4	ATP2A2	FRMD4B	MNAT1	NFIA	PTPN11	DNAJA2	THUMP…	PPP1R…	ATP11C	DNAJC…	CSNK2…	PTBP3	PINX1	TUBA4A	ANKRD…	FAM12…	CTNND1	MRPL42	PKN1	EIF6	VPS26A	COPS4	ORMDL2	SETX	BLOC1…	C1orf…	RPS15A	TAP1
CELLMARKERMSIGDB_GAUTAM_EYE_IRIS_CILIARY_…																														
CELLMARKERMSIGDB_GAUTAM_EYE_IRIS_CILIARY_…																														
CELLMARKERMSIGDB_GAUTAM_EYE_IRIS_CILIARY_…																														
CELLMARKERMSIGDB_GAUTAM_EYE_IRIS_CILIARY_…																														
CELLMARKERMSIGDB_HAY_BONE_MARROW_CD34_POS…																														
CELLMARKERMSIGDB_HAY_BONE_MARROW_CD34_POS…																														
CELLMARKERMSIGDB_HAY_BONE_MARROW_CD34_POS…																														
CELLMARKERMSIGDB_HAY_BONE_MARROW_CD34_POS…																														
CELLMARKERMSIGDB_HAY_BONE_MARROW_CD8_T_CE…																														
CELLMARKERMSIGDB_HAY_BONE_MARROW_EARLY_ER…																														
CELLMARKERMSIGDB_HAY_BONE_MARROW_EOSINOPH…																														
CELLMARKERMSIGDB_HAY_BONE_MARROW_NAIVE_T_…	RORA	EIF3E	DNAJB1	RPS21	TSC22…	TMEM1…	TNFAI…	RPL24	KLF2	ITM2A	AP3M2	TRABD…	NCMAP…	CD5	RPS15	LDHB	STMN3	FYB1	RPS17	COMMD6	ICOS	RHOH	TRAF3…	RPS2	FXYD5	ETS1	CD28	ARHGA…	TSHZ2	VAMP2
CELLMARKERMSIGDB_HAY_BONE_MARROW_NEUTROPH…	ANXA5	SCPEP1	TIMP2	ATP6V…	ANXA2	LY86	MIR22…	OSCAR	PRAM1	TMEM1…	CLEC1…	DAZAP2	HNMT	MSRB1	PADI4	IGSF6	MTMR11	AP2S1	ALDOA	SERPI…	IVNS1…	GNAI2	SDCBP	LTA4H	FOLR3	PGAM1	GLIPR2	TRIB1	CTSH	OTUD1
CELLMARKERMSIGDB_HAY_BONE_MARROW_NK_CELLS	PTPN4	LINC0…	XCL1	SYTL1	KIR2D…	DHRS7	PIP4K…	CD53	EFHD2	LPCAT1	STAT4	ARL4C	PCSK7	TGFBR3	ADAM8	NMUR1	PDGFD	TSEN54	EIF3G	CX3CR1	SYNE1	ARHGA…	ASCL2	SPTSSB	CAPN12	TBC1D…	ZBP1	ZBTB16	ARHGD…	HLA-F
CELLMARKERMSIGDB_HAY_BONE_MARROW_PRE_DEND…																														
CELLMARKERMSIGDB_HAY_BONE_MARROW_PRO_B	LAT2	RACGA…	HELLS	RBMX	DDX39A	SUMO2	SERHL2	HNRNP…	STRBP	SKA3	HJURP	CDCA7L	FBXO43	RMI2	MSI2	HMGN3	PAQR4	MND1	CDKN2C	EAF2	PXMP2	TCF19	TFDP2	ITGB3…	SGO2	SLC7A3	PSIP1	SMARC…	SIVA1	TMEM2…
CELLMARKERMSIGDB_HAY_BONE_MARROW_STROMAL	FRMD6	VEGFC	OLFML…	NUPR1	GFRA1	NOTCH3	TMEM1…	FOSB	ANTXR1	RND3	ANGPT1	ADAMT…	SNAI2	GHR	TMEM37	NAV2	CFH	ITGBL1	COX7A1	SLC22…	TMEM1…	RSPO1	EGFLAM	LGI4	TIMP4	SRPX	FN1	ANK2	SEMA6D	GLDN
CELLMARKERMSIGDB_HE_LIM_SUN_FETAL_LUNG_C0…	RAI14	TRAF5	CTXN1	PODXL2	KITLG	PGM5	RIMS1	SLC27…	CAP2	NTF3	SINHC…	VWA1	PDCL3	RAMP1	BNC2	ANKRD…	TACC2	AHNAK2	SNX27	PRUNE2	WFDC1	NXN	ENTPD1	ANOS1	HAND2	ETHE1	ECHDC2	AKIP1	CD24	FLNC
CELLMARKERMSIGDB_HE_LIM_SUN_FETAL_LUNG_C0…																														
CELLMARKERMSIGDB_HE_LIM_SUN_FETAL_LUNG_C0…																														
CELLMARKERMSIGDB_HE_LIM_SUN_FETAL_LUNG_C0…	HHIP-…	DHRS3	PLCL1	LSR	PLEKH…	SLITR…	C10or…	BNC1	EPS8L2	BNC2	RNASE1	PNPLA4	TCEAL2	AGRN	AFDN	SLC39…	MMP11	POMGN…	C11or…	STK26	AMOTL2	TM7SF2	RCHY1	LY6H	KLK5	GALNT9	NSG1	HNRNP…	HRH2	CSDC2
CELLMARKERMSIGDB_HE_LIM_SUN_FETAL_LUNG_C0…																														
CELLMARKERMSIGDB_HE_LIM_SUN_FETAL_LUNG_C0…																														
CELLMARKERMSIGDB_HE_LIM_SUN_FETAL_LUNG_C0…	SMPD3	CAVIN2	GSDME	TNXB	EMP1	UPK1B	IL18	RERG	PHYHIP	BAIAP2	TGFB3	ITLN1	CDH3	KLK10	TMEM1…	NDRG1	PROCR	MYRF	CA9	PKHD1…	HEG1	SMTNL2	MST1	EFNA1	MBP	CD74	TCEAL2	MVP	NMU	HHIP
CELLMARKERMSIGDB_HE_LIM_SUN_FETAL_LUNG_C0…	MKI67	TNFRS…	FZD2	CDK1	TPX2	CENPF	ZWINT	CLSPN	RMI2	EPHA4	CENPM	LMNB2	EZH2	BIRC5	UBE2S	METTL…	SHISA3	H1-1	CENPU	THNSL2	SYK	CCNA2	CENPN	NTM	NCAPH	MND1	HSPA1…	NKAIN3	NUSAP1	DIAPH3
CELLMARKERMSIGDB_HE_LIM_SUN_FETAL_LUNG_C0…																														
CELLMARKERMSIGDB_HE_LIM_SUN_FETAL_LUNG_C0…	GALNT9	RNASE1	GSDME	JUP	FLRT3	MMP28	COL2A1	TMEM1…	PLAAT3	RHOU	CRIM1	CA9	ITGA3	SLC7A7	LEPRO…	NINJ1	STK26	LMNB1	BNC2	LINC0…	GADL1	RSPO3	HPGD	SVIL	FGF9	LY6H	SYNGR2	SFRP5	ANXA8…	MUS81
CELLMARKERMSIGDB_HE_LIM_SUN_FETAL_LUNG_C0…																														
CELLMARKERMSIGDB_HE_LIM_SUN_FETAL_LUNG_C1…																														
CELLMARKERMSIGDB_HE_LIM_SUN_FETAL_LUNG_C1…																														
CELLMARKERMSIGDB_HE_LIM_SUN_FETAL_LUNG_C1…																														
CELLMARKERMSIGDB_HE_LIM_SUN_FETAL_LUNG_C1…	MAN1A1	CHAF1A	CDC6	PSRC1	TTI2	MELK	HMMR	CHEK1	XXYLT1	KIF2C	CAPN6	TESMIN	CDCA5	RRS1	KIF23	WDHD1	PRIM1	MAP7D1	DLGAP5	YKT6	DDC	CCNQ	PRKCA	KCMF1	RANGA…	C14or…	KNSTRN	GPC2	SLF1	NES
CELLMARKERMSIGDB_HE_LIM_SUN_FETAL_LUNG_C1…	SGPL1	INSR	COL12…	MVB12B	SLC7A5	TMEM51	GPBAR1	HSD11…	EPHA4	TTR	SHISA…	INHBA	SRGAP3	CALY	DIRAS2	ASCL1	SLC12…	FAM17…	JAM3	SCRN1	RASA4B	LSS	DOP1B	AP1AR	DPYSL3	AAK1	ARHGE…	NRCAM	CERS6	GRP
CELLMARKERMSIGDB_HE_LIM_SUN_FETAL_LUNG_C1…																														
CELLMARKERMSIGDB_HE_LIM_SUN_FETAL_LUNG_C1…																														
CELLMARKERMSIGDB_HE_LIM_SUN_FETAL_LUNG_C1…																														
CELLMARKERMSIGDB_HE_LIM_SUN_FETAL_LUNG_C1…	ST6GA…	S100A9	CYP2F1	KDR	CXCL8	NDUFA…	RIMS1	VCAN	TLR5	DDI2	IRAG2	ISG20	MLPH	SPINK1	IFITM1	FNBP1	GSTA1	BPIFB1	TPCN1	WIPI1	KRT15	PTGFR	LSP1	MT1E	LUZP2	ATP2A3	KCNK6	PLAAT3	SMIM22	SFTA1P
CELLMARKERMSIGDB_HE_LIM_SUN_FETAL_LUNG_C1…	SLITR…	DMPK	CHRD	SLC35…	TOX	VGF	MAPRE3	CPLX2	TCERG…	DUSP26	DACH2	DST	RASSF6	MARK1	SYT4	SYT13	MAOB	ADCY2	NEBL	RUNX1…	SLC18…	ST18	CACNA…	FBXW7	INA	RIMBP2	ABCA5	SLC29…	RALYL	SYT5
CELLMARKERMSIGDB_HE_LIM_SUN_FETAL_LUNG_C1…	ISLR	RCBTB2	PLAT	LUZP1	PAK3	SHISA2	CLDN1	STMN3	UACA	LY75	CYP4X1	PTGER4	RGS10	BHLHE…	ZHX2	CXCL1	NCALD	NRP2	MAP1B	TNFAI…	DUSP5	FNBP1	C6orf…	BCL2	ST3GA…	SSPN	FRMD4A	TTC39C	SCARB1	FBLN2
CELLMARKERMSIGDB_HE_LIM_SUN_FETAL_LUNG_C1…	B3GNT5	F3	MARCH…	FKBP11	DGKA	TCEA3	HS3ST1	SAMHD1	CYB5R2	DOCK9	PIM2	FRAT2	LTC4S	METTL…	DST	CLCF1	SOCS6	TRAK1	RILPL2	TMEM6…	RUNX1	SH3PX…	PIP	CALML5	PRSS21	LINC0…	ZG16B	TM4SF…	KIF13B	TSPAN8
CELLMARKERMSIGDB_HE_LIM_SUN_FETAL_LUNG_C2…	PATL2	GAL3S…	MYO1E	TDP2	DNAJC…	ZMAT5	SLC35…	VPS37C	TCN2	CXCL12	TNS1	APPL2	ARHGA…	CD58	MFSD12	CARM1	C1orf…	PGM2L1	FARP1	TFPT	NCEH1	GFER	CCDC1…	RNF14	TMEM94	CMKLR1	CBFB	H4C16	FMNL2	EPSTI1
CELLMARKERMSIGDB_HE_LIM_SUN_FETAL_LUNG_C2…	WDR41	SULF2	ACAP1	SERPI…	HPS5	PTPN22	CYYR1	ZEB1	CLEC1A	TSPAN2	TPMT	PMM1	PARM1	LRRK2	C15or…	PDE6D	IDH3A	GPR13…	DST	RCN2	ADAM19	HMGN4	TRPV2	TSPAN…	CAMK2G	DBN1	RFTN1	PON2	KATNA1	NCALD
CELLMARKERMSIGDB_HE_LIM_SUN_FETAL_LUNG_C2…																														
CELLMARKERMSIGDB_HE_LIM_SUN_FETAL_LUNG_C2…																														
CELLMARKERMSIGDB_HE_LIM_SUN_FETAL_LUNG_C2…																														
CELLMARKERMSIGDB_HE_LIM_SUN_FETAL_LUNG_C2…	MYL6B	BEX2	USP11	DPPA4	SYNGR1	ERG	TOX	TMIGD2	CPXM1	NFE2	CHD1L	INTS12	STK26	MFAP4	KAT6B	LRBA	SYTL1	JCHAIN	TFDP2	PLCB1	HCG18	TEX30	MRPS27	GIMAP2	SNRPN	MATK	ZNF100	AK3	RDH14	GSTM2
CELLMARKERMSIGDB_HE_LIM_SUN_FETAL_LUNG_C2…	UBL4A	MEG3	ABHD1…	DGKD	CALD1	ANO6	TUBA8	ABCC3	MIR44…	HPSE	ISCA1	FAXDC2	PF4V1	RNF24	CTSW	GNB5	RBPMS2	CTTN	FHOD1	GADD4…	HEXIM1	LRP12	P2RY1	MGLL	TRIM13	LINC0…	SRC	CLIC4	TMEM91	CXCL3
CELLMARKERMSIGDB_HE_LIM_SUN_FETAL_LUNG_C2…																														
CELLMARKERMSIGDB_HE_LIM_SUN_FETAL_LUNG_C2…	CHAMP1	KLHL42	ACRBP	ZMYM5	ZBTB11	H4C15	H4C14	NECAB3	PHF1	XRN1	ARHGA…	BET1	KDM5B	STOM	MPL	KLF1	H4C11	PEAR1	KALRN	COL24…	B3GNT2	MFSD2B	C1orf…	HBG1	DMTN	TSPAN…	BSDC1	RAB27B	EGLN3	PF4
CELLMARKERMSIGDB_HE_LIM_SUN_FETAL_LUNG_C2…	ASF1A	COQ4	MTA1	ATAD2	MAGOHB	WDR43	GNG11	SUZ12	MRPL39	IRF4	UBE2S	ST6GA…	ADGRG5	ZNF326	GTF3C5	RUVBL1	RYBP	KCTD5	NIPSN…	DTYMK	MCM3	GEMIN7	GPX7	IARS1	DHFR	RNASE…	TOPBP1	MZB1	MRPS10	GMPS
CELLMARKERMSIGDB_HE_LIM_SUN_FETAL_LUNG_C2…																														
CELLMARKERMSIGDB_HE_LIM_SUN_FETAL_LUNG_C2…																														
CELLMARKERMSIGDB_HE_LIM_SUN_FETAL_LUNG_C2…																														
CELLMARKERMSIGDB_HE_LIM_SUN_FETAL_LUNG_C3…																														
CELLMARKERMSIGDB_HE_LIM_SUN_FETAL_LUNG_C3…	PCLAF	NDC80	TSPAN…	C20or…	CDCA8	DBF4	PLEK2	ANKRD9	CCDC34	CYBRD1	BTG3	KCNH2	TACC3	CDCA2	SMIM5	RRM2	CKAP2	ATAD2	SLC6A9	ATP1B2	PKMYT1	CENPA	SLC7A5	CCNB1	PLK1	PIP5K…	LRR1	CTSE	SMC2	SNRNP…
CELLMARKERMSIGDB_HE_LIM_SUN_FETAL_LUNG_C3…	NR2F6	NEK6	CDC42…	SPATC…	SNAI1	LMNB1	GNL3	CISD3	HOXB2	EIF2B2	PUM3	HAUS4	NQO1	UTP18	SPOCK2	TIMM9	BCL2L…	GTPBP4	USP18	STMN3	IFT22	FAM13A	FKBP5	CCNB2	MXI1	ARRDC3	C1QTN…	BIRC5	EXOSC7	NAMPT
CELLMARKERMSIGDB_HE_LIM_SUN_FETAL_LUNG_C3…	ZDHHC…	ITPK1	LY96	AKR1C3	PDLIM4	RAB6B	COL9A3	MAGED4	GIMAP5	PLEKH…	MEST	PTX3	ADAMT…	FAM10…	PRKCZ	SRGAP3	ITGB4	SCARA3	ASRGL1	PPARG	STOX2	TMEM1…	GSTM3	LRRC70	DNPH1	SFT2D2	RAB3I…	TRIM47	SIRPA	C20or…
CELLMARKERMSIGDB_HE_LIM_SUN_FETAL_LUNG_C3…	BZW2	ASGR1	RAB3I…	GPR182	CDC42…	ODF2L	SCN9A	SFXN3	TM4SF…	CMBL	GGT5	SLC43…	CISD1	HSDL2	ANGPT2	PDIA5	CFI	SPHK1	ARID5B	FILIP1	CHCHD…	PRKCZ	SFRP1	CHST15	GALNT1	IGFBP5	LIMCH1	PCLO	C4orf…	HAPLN3
CELLMARKERMSIGDB_HE_LIM_SUN_FETAL_LUNG_C3…																														
CELLMARKERMSIGDB_HE_LIM_SUN_FETAL_LUNG_C3…	MIR15…	CYTL1	TENT5A	BMP6	SLC22…	RUNX1…	GFOD1	TP53I…	EPB41…	MYORG	NNMT	TNFRS…	TMTC1	THBS3	PLCG2	HEXA	CKB	GCHFR	SKIL	NR4A2	GDF7	SPOCK1	RRAS	IL13R…	RCN3	KCNMB1	CD58	METRNL	C1R	RBMS3
CELLMARKERMSIGDB_HE_LIM_SUN_FETAL_LUNG_C3…	MPP7	ADAMT…	MID1	CHRDL1	PIK3C…	EBLN3P	A4GALT	FILIP1	MVP	SLC43…	TPBG	FLNC	SWAP70	KBTBD…	AOPEP	SRD5A3	SOCS2	P2RX4	TNFRS…	IFT122	PRICK…	TMEM3…	ENTRE…	FZD6	FBXO34	PARD6G	LYPD6	PIGP	KLHL4	FHIT
CELLMARKERMSIGDB_HE_LIM_SUN_FETAL_LUNG_C3…																														
CELLMARKERMSIGDB_HE_LIM_SUN_FETAL_LUNG_C4…																														
CELLMARKERMSIGDB_HE_LIM_SUN_FETAL_LUNG_C4…																														
CELLMARKERMSIGDB_HE_LIM_SUN_FETAL_LUNG_C4…																														
CELLMARKERMSIGDB_HE_LIM_SUN_FETAL_LUNG_C4…																														
CELLMARKERMSIGDB_HE_LIM_SUN_FETAL_LUNG_C4…	ORC6	PRPSA…	PLK1	H2BC9	UBE2S	HMMR	C19or…	FOXM1	NCAPH2	MIS18…	CENPH	RFC2	KIF20B	CDC20	MXD3	CASP8…	H3C15	ARL6I…	RPL39L	CIP2A	LMNB2	MELK	C21or…	EBP	H3C14	MCM2	CCDC2…	DLGAP5	FAM11…	H3C3
CELLMARKERMSIGDB_HE_LIM_SUN_FETAL_LUNG_C4…	SYNE2	KPNA2	ZDHHC…	FANCI	CD5	CENPA	MND1	PRC1	CDKN2C	CKAP5	CCNB1	NEIL3	YEATS4	LRRN3	CDCA2	MELK	RAD51…	HMGB3	ACYP1	C19or…	H2BC9	STMN3	CDC20	CD6	TMEM1…	FAM11…	PRR11	MYBL2	HELLS	RECQL
CELLMARKERMSIGDB_HE_LIM_SUN_FETAL_LUNG_C4…																														

Table S5: Cell state marker gene list

CELLMARKERCAN_ACINAR_CELLS																														
CELLMARKERMSIGDB_HE_LIM_SUN_FETAL_LUNG_C4…																														
CELLMARKERMSIGDB_HE_LIM_SUN_FETAL_LUNG_C4…																														
CELLMARKERMSIGDB_HE_LIM_SUN_FETAL_LUNG_C4…																														
CELLMARKERMSIGDB_HE_LIM_SUN_FETAL_LUNG_C5…																														
CELLMARKERMSIGDB_HE_LIM_SUN_FETAL_LUNG_C5…	RARA	SNX10	LINC0…	SLX9	MRPS25	RFTN1	PDLIM2	POLD2	TLR10	KCNN4	MRPS12	FAM16…	LTBP3	SEL1L3	MRPL27	SNRNP…	PDCL3	ZCRB1	MIF4GD	AFTPH	EBNA1…	MRPL11	CD5	POLR3K	NFKB2	FH	PAK1I…	NFKBIZ	WARS1	S100A…
CELLMARKERMSIGDB_HE_LIM_SUN_FETAL_LUNG_C5…																														
CELLMARKERMSIGDB_HE_LIM_SUN_FETAL_LUNG_C5…																														
CELLMARKERMSIGDB_HE_LIM_SUN_FETAL_LUNG_C5…	PXMP2	MCM6	GMPS	HACD3	CCDC34	CNTLN	VRK1	H3C2	E2F2	KIF20B	CDC20	MAP3K…	BARD1	H2AC13	TFDP1	TIMEL…	SCMH1	ELK3	LRR1	CENPN	ASPM	UBE2S	VAMP5	PLSCR1	CMC2	KIF11	ATAD5	CDCA3	H1-5	NDC80
CELLMARKERMSIGDB_HE_LIM_SUN_FETAL_LUNG_C6…																														
CELLMARKERMSIGDB_HE_LIM_SUN_FETAL_LUNG_C7…																														
CELLMARKERMSIGDB_HE_LIM_SUN_FETAL_LUNG_C7…																														
CELLMARKERMSIGDB_HE_LIM_SUN_FETAL_LUNG_C7…																														
CELLMARKERMSIGDB_HE_LIM_SUN_FETAL_LUNG_C7…																														
CELLMARKERMSIGDB_HE_LIM_SUN_FETAL_LUNG_C7…	DPF1	FAM21…	SH2D3C	CYGB	TG	SRRM3	NACAD	PTPRR	NMNAT2	PRKCZ	KLHL5	CACNB1	DNM3	DOC2B	JPH4	DNER	TMEFF2	TMEM1…	MAP7	DLGAP1	FECH	ACBD5	PLPPR2	LIN7B	CHST1	KIF5A	CACNA…	NYAP1	ATP7A	PRSS3
CELLMARKERMSIGDB_HE_LIM_SUN_FETAL_LUNG_C7…																														
CELLMARKERMSIGDB_HU_FETAL_RETINA_AMACRINE																														
CELLMARKERMSIGDB_HU_FETAL_RETINA_BIPOLAR																														
CELLMARKERMSIGDB_HU_FETAL_RETINA_FIBROBLA…	TFPI	FOSB	NFIA	CLMP	NRP2	FRMD6	LGI1	DKK2	RAB34	MRC2	NR2F2	TIMP1	GPX8	SVIL	TGFB1…	ECE1	NFKBIZ	RBMS3	PLVAP	CCND2	CREB5	THY1	NOTCH3	MMP2	C1orf…	LIMA1	EMX2	FSTL1	ETS1	LRRC17
CELLMARKERMSIGDB_HU_FETAL_RETINA_HORIZONT…																														
CELLMARKERMSIGDB_HU_FETAL_RETINA_MICROGLIA	INPP5D	TMIGD3	LAT2	DOCK2	IL13R…	MPEG1	HAVCR2	MKNK1	CTSB	CD84	PYCARD	OLFML3	LGMN	NINJ1	LILRB4	NCKAP…	CORO1A	GNB4	NCF4	IKZF1	ITPR2	LIPA	HLA-D…	SAT1	RHOG	EVI2A	CD86	TAGAP	HLA-C	ELF1
CELLMARKERMSIGDB_HU_FETAL_RETINA_MULLER																														
CELLMARKERMSIGDB_HU_FETAL_RETINA_PHOTOREC…																														
CELLMARKERMSIGDB_HU_FETAL_RETINA_RGC	ETFB	LRFN5	CTNNA2	FABP3	FNDC5	FGF13	XPR1	PAFAH…	DPYSL2	KIF5A	TMEM1…	CALM3	AKAP6	FABP5	VSTM2L	ROBO2	VDAC3	DTD1	TAC1	OPTN	TXN	KIFAP3	CRABP2	GET1	ZC2HC…	AP3S1	AFAP1	SEZ6L	TMOD2	MAP2
CELLMARKERMSIGDB_LAKE_ADULT_KIDNEY_C1_EPI…																														
CELLMARKERMSIGDB_LAKE_ADULT_KIDNEY_C10_TH…	B2M	BCL6	RPL19	RAN	ANXA5	GPX1	CTSB	GNAS	HSBP1	MCL1	RPS23	PPA1	NAMPT	UQCRF…	ALDOA	MAOA	EIF1	RPS27A	RPL15	CACNA…	YBX1	MYL12B	RPL11	PCBP1	PANTR1	HINT1	ERRFI1	ITM2B	PEBP1	SERF2
CELLMARKERMSIGDB_LAKE_ADULT_KIDNEY_C12_TH…	CHCHD…	CBR1	RPS9	RPS11	ALDOA	GNAS	HADHB	DNAJC…	SCHLA…	BEX3	CADM1	UQCRF…	HS6ST2	CD59	RPS12	ATP5PF	EIF3K	RPS14	CD24	SLC25…	HSBP1	NDUFV1	ATP5PD	RPL18	GAPDH	PRXL2A	RPL3	HERPU…	UQCRC1	UXS1
CELLMARKERMSIGDB_LAKE_ADULT_KIDNEY_C18_CO…	SLPI	SARAF	RTN4	RACK1	HSPD1	S100A6	BEX3	IFITM3	ITM2C	RPS5	RPS27A	STIM2	PDIA6	LAMTO…	GPRC5C	CALM1	RPL7A	FTH1	NACA	PRKAR…	COX4I1	ADGRG1	MAN1A1	LDHA	EID1	PSMA7	RPL31	RPL5	RPS23	MECOM
CELLMARKERMSIGDB_LAKE_ADULT_KIDNEY_C19_CO…	COX5A	H3-3B	ARL6I…	CD164	SNHG29	KRT18	RPS16	AQP2	CYCS	FDFT1	RPL13A	TMEM59	RPL7A	UQCRF…	RPL18	ARF1	SOD2	GAPDH	PTGES3	RPL4	TPT1	CD63	CRYBG3	MTR	NACA	CIAO2B	MYL12A	HSPA4	CAPN2	CHCHD…
CELLMARKERMSIGDB_LAKE_ADULT_KIDNEY_C25_EN…																														
CELLMARKERMSIGDB_LAKE_ADULT_KIDNEY_C28_IN…																														
CELLMARKERMSIGDB_LAKE_ADULT_KIDNEY_C29_UN…																														
CELLMARKERMSIGDB_LAKE_ADULT_KIDNEY_C5_PRO…	HPN	GPX3	ACSM2B	PLEKH…	MAPT	MFSD4B	TNS1	TMEM6…	OTUD7A	BCOR	PPARA	ABAT	DHTKD1	ZBED3…	EYA2	MLXIPL	SRRM2	SIPA1…	C19or…	ACSM2A	TENM4	PCK1	USH1C	ANKS1B	SHISA6	LUC7L3	LARGE1	SLC2A9	PDZD2	DHRS3
CELLMARKERMSIGDB_MANNO_MIDBRAIN_NEUROTYPE…																														
CELLMARKERMSIGDB_MANNO_MIDBRAIN_NEUROTYPE…	DIRAS3	DLG2	DLG4	DLK1	DMTN	DNASE…	DNER	DNM3	DOCK4	DOK4	DOK6	DPP6	DPYSL3	DSCAM	EBF1	EBF2	EBF3	EDIL3	EEF1A2	EEPD1	EHBP1	EID2B	ELAVL2	ELAVL3	ELAVL4	ELOVL3	ELOVL4	EN1	EN2	ENC1
CELLMARKERMSIGDB_MANNO_MIDBRAIN_NEUROTYPE…	CORO2A	CPEB3	CPEB4	CRMP1	CSMD1	CSMD2	CSMD3	CSRNP3	CTNNA2	CTXN2	CXADR	CYP4X1	DAAM1	DAPK1	DCC	DCLK1	DCX	DDC	GSDME	DLK1	DMTN	DNAJB5	DNAJC3	DNAJC6	DNER	DNM1	DNM3	DOCK3	DOCK4	DOK4
CELLMARKERMSIGDB_MANNO_MIDBRAIN_NEUROTYPE…	CTNNA2	CXADR	CYP4X1	DCC	DCX	DDC	DEAF1	DGKE	DIRAS2	DIRAS3	DKK3	DLG2	DLK1	DMTN	DNAH2	DNAJC…	DNAJC6	DNM3	DOCK3	DOK4	DOK6	DOP1A	DPP6	DPP7	DPYSL3	DRD2	DSCAM	DUSP4	DYNC1…	EEF1A2
CELLMARKERMSIGDB_MANNO_MIDBRAIN_NEUROTYPE…	CD19	CD27	FCGR2A	CD34	CD4	CD55	CD59	CD81	CD82	CD93	CDA	CDC42…	CDH11	CDH5	CDKN1A	CDKN2B	CEACA…	CERS2	CETP	CFLAR	CGNL1	CHST15	CHST3	CHST7	CKMT2	CLDN5	CLEC1…	CLEC1A	CLEC2B	CLEC3B
CELLMARKERMSIGDB_MANNO_MIDBRAIN_NEUROTYPE…	CHCHD1	CLEC7A	CMKLR1	CNPY3	COLGA…	CORO1B	CPED1	CPVL	CREG1	CREM	CRYBB1	CSF1	CSF1R	CSF3R	CSRNP1	CTDSP1	CTSB	CTSC	CTSD	CTSH	CTSL	CTSS	CX3CR1	CXCL2	CXCL8	TASL	CYBA	CYBB	CYFIP1	CYTH4
CELLMARKERMSIGDB_MANNO_MIDBRAIN_NEUROTYPE…	CDK5R1	CDKL2	CDKL5	CDKN2D	CDRT4	TMEM1…	CELF2	CELF3	CELF4	CELF5	CEND1	CERS6	CHCHD1	CHD5	CHERP	CHGA	CHIC1	CHL1	CHRNA4	CHRNA7	CHST1	CLVS1	CLVS2	CMIP	CMTM4	CNGB1	CNR1	CNTN4	CNTNA…	CNTNA…
CELLMARKERMSIGDB_MANNO_MIDBRAIN_NEUROTYPE…	GRIN3A	GRM4	GSE1	MACRO…	HS6ST3	IGSF9	INA	INSM1	ISLR2	ITGA6	JPH4	JUP	KCNA3	KCNH8	KCNQ3	KCTD4	ARFGE…	KIF21A	KIF21B	KIF26A	KIF3C	KIF5C	KLC1	KLHL32	KLHL35	LCOR	LHX1	LINGO1	ENSG0…	ASPA
CELLMARKERMSIGDB_MANNO_MIDBRAIN_NEUROTYPE…	DUSP4	EFCAB7	ELAVL2	ELAVL3	ELAVL4	EMX2	EMX2OS	ENC1	ENO2	EPB41	EPHA3	ERC2	ESRRG	EYA1	F11R	FABP3	TCAF1	FAM11…	NALF1	TAFA2	ATOSA	TLCD3B	RIPOR2	FAM81A	LRATD1	FBXL12	FGF14	FGF9	FICD	FMN2
CELLMARKERMSIGDB_MANNO_MIDBRAIN_NEUROTYPE…	ELAVL4	ELOVL4	ENO2	EPB41…	EPHA3	ESRRB	ESRRG	ETFB	EYA1	FABP6	FADS3	FAIM2	FAM13…	NALF1	INSYN…	TLCD3B	FAR2P2	FAXC	FGF1	FGF13	FGF9	FNBP1L	FNDC5	FNDC9	FRY	FSD1	FSTL5	GABRA5	GABRG2	GAP43
CELLMARKERMSIGDB_MANNO_MIDBRAIN_NEUROTYPE…	EGFR	EGR1	EID2B	ELF1	ELFN2	EPN2	ERBB3	ERF	ETV1	ETV5	EVI2A	EYA2	FABP5	FABP7	FALEC	NALF1	FAM18…	FAM18…	TAFA2	TAFA5	FAM89A	FGFBP3	FICD	FXYD6	FYN	GAB1	GABBR2	GAL3S…	GAREM2	GATM
CELLMARKERMSIGDB_MANNO_MIDBRAIN_NEUROTYPE…	CD164	CD19	CD248	CD4	CD44	CD63	CD81	CD9	CDC42…	CDC42…	CDH11	CDH6	CDKN1A	CEBPB…	CEBPD	CETP	CFH	CFHR1	CHCHD1	CHMP1B	CHST2	CHSY3	CISD1	CISH	CLDN7	CLEC1…	CMKLR1	CMTM6	COBLL1	COL18…
CELLMARKERMSIGDB_MANNO_MIDBRAIN_NEUROTYPE…	GTSE1	GULP1	H2AX	H2AZ1	HAT1	HELLS	HERC2…	HES1	HES5	H2BC9	H3C2	H3C3	H4C9	H4C12	HJURP	HK2	HMGA1	HMGA2	HMGB2	HMGN2	HMMR	RIDA	IGDCC3	IGF2B…	IGFBP2	ILDR2	IRX2	IRX3	ITGB3…	ITGB8
CELLMARKERMSIGDB_MANNO_MIDBRAIN_NEUROTYPE…	FBLN1	FBN1	FBXL7	FBXO5	FNDC3B	FOXA1	FOXA2	FOXM1	FREM1	FREM2	FZD1	FZD2	FZD3	GBX2	GINS1	GINS2	GMNN	GNG5	GPC4	GPRC5C	GPSM2	GPX8	GTSE1	GULP1	H2AX	MACRO…	HELLS	HES1	H2BC9	H3C2
CELLMARKERMSIGDB_MANNO_MIDBRAIN_NEUROTYPE…	PIMREG	DIS3L2	FAM72A	FAM83D	LRATD2	FANCB	FANCI	FBLN1	FLRT2	FNDC1	FOXA1	FOXA2	FOXM1	FREM1	FREM2	GALNT7	GAS2	GBX2	GINS1	GINS2	GMNN	GNG5	GPRC5C	GPX8	GSTP1	GTF2H2	GTSE1	GULP1	H2AZ1	HADH
CELLMARKERMSIGDB_MANNO_MIDBRAIN_NEUROTYPE…	FNDC1	FNDC3B	FOXA1	FOXA2	FOXJ1	FOXM1	FOXP2	FREM2	FRMD3	FZD1	FZD2	GBX2	GDPD2	GINS2	GNG5	GPD1L	ADGRV1	GRM8	GTF2F2	GTSE1	GULP1	HEATR…	HES1	HILPDA	H2BC9	H3C2	H4C12	HJURP	HK2	HMGA1
CELLMARKERMSIGDB_MANNO_MIDBRAIN_NEUROTYPE…	FAM21…	IQANK1	LRATD2	FAT1	FAT3	FGFR3	FJX1	FLNC	FLVCR2	FNDC1	FOXA1	FOXA2	FOXJ1	FOXM1	FOXP2	FREM2	FRMD3	FRMPD2	FRZB	FSTL5	FZD1	FZD6	GABPB2	GCA	GDPD2	GLIS3	GNG12	GPC4	GPM6B	ADGRV1
CELLMARKERMSIGDB_MANNO_MIDBRAIN_NEUROTYPE…	COMT	CPNE2	CPNE5	CPXM2	CRB1	CRB2	CREB5	CRISP…	CRYAB	CRYM	CSGAL…	CSMD3	CSPG5	CST3	CCN2	CTNND2	CXCL2	CXCR4	CFAP47	THOC2	CYP26…	CCN1	DAG1	DBX2	DCDC1	DCLK1	DCLK2	DDAH1	DHCR7	DHX32
CELLMARKERMSIGDB_MANNO_MIDBRAIN_NEUROTYPE…	EFHC2	EFHD2	EGFR	EGR1	EGR2	ELN	ELOVL2	ENHO	ENPP2	EPB41…	EPHA3	ERF	ERVK1…	ESCO2	ETV1	F3	FABP5	FAM16…	FAM18…	INKA2	RIPOR2	LRATD2	FANCI	FASN	FAT1	FAT3	FGF1	FGFBP3	FGFR3	FHL1
CELLMARKERMSIGDB_MANNO_MIDBRAIN_NEUROTYPE…	FGFBP3	FGFR3	FOXL2	FOXM1	FUT9	GABBR2	GATM	GDPD2	GINS1	GLTP	GMPR	GNG12	GNG7	GPM6A	GPM6B	ADGRG1	GRAMD…	GRIA1	GRID2	GRIP2	GRM3	GRM5	HAPLN3	HAS2	HBA1	HBA2	HBG1	HBG2	HEPAC…	HEPN1
CELLMARKERMSIGDB_MANNO_MIDBRAIN_NEUROTYPE…	CNTN4	COBL	COL18…	COL27…	COL4A5	COL6A1	COL8A1	COLEC…	CORIN	CPEB2	CPNE8	CPQ	CPXM2	CRB1	CRB2	CREB5	CRIM1	CRIP1	CRISP…	CRYAB	CRYZ	CSGAL…	CSPG5	CCN2	CTNNA3	CFAP47	CCN1	DAG1	DAPK1	DCDC1
CELLMARKERMSIGDB_MANNO_MIDBRAIN_NEUROTYPE…	GABRB3	GABRG2	GAP43	GDAP1…	GLRA2	BICRAL	GNG2	GNG3	GOLGA…	GOLGA…	GPC2	GPM6A	GPR155	GPRIN1	GREM2	GRIA2	GRIK2	GRIN3A	GRIP1	HIVEP3	NSG2	HOOK1	HUNK	INA	IQCJ-…	ISLR2	JAKMI…	JPH4	KCNA3	KCNC1
CELLMARKERMSIGDB_MANNO_MIDBRAIN_NEUROTYPE…	DIRAS2	DMTN	DNAJC…	DNAJC5	DNAJC6	DNM3	DOK6	DPF1	DPYSL3	DYNC1…	EEF1A2	EID2B	ELAVL2	EMID1	ENO2	EPB41…	ERC1	ERC2	RETRE…	NALF1	TAFA1	TAFA2	TAFA5	RIPOR2	LRATD1	FAR2	FBLL1	FBXL16	FEV	FGF13
CELLMARKERMSIGDB_MENON_FETAL_KIDNEY_0_CAP…																														
CELLMARKERMSIGDB_MENON_FETAL_KIDNEY_10_IM…																														
CELLMARKERMSIGDB_MENON_FETAL_KIDNEY_2_NEP…																														
CELLMARKERMSIGDB_MENON_FETAL_KIDNEY_3_STR…																														
CELLMARKERMSIGDB_MENON_FETAL_KIDNEY_6_COL…																														
CELLMARKERMSIGDB_MURARO_PANCREAS_ACINAR_C…	PTGR1	SLC25…	F3	CEBPD	CPM	PSAT1	CREB3…	GJB1	TMPRS…	AKR7A3	XBP1	LGR4	RETRE…	LTB	GULP1	NUPR1	PDGFA	CYP3A5	BACE2	CCL20	IGFBP2	TCIM	HHLA2	TUBB2A	INSR	TM4SF1	FAM3B	CD44	KRTCA…	AADAC
CELLMARKERMSIGDB_MURARO_PANCREAS_ALPHA_CE…	MAGED2	CST3	CERS6	SDK1	PCSK1N	PFN2	RASSF5	GNE	TMEM80	NDUFB8	SH3RF1	SLC35…	CAMK2B	KCNK17	ROMO1	VKORC1	FAM10…	MBD4	ZBED5…	CD46	IRF6	TMEM17	GNAS	GK5	NT5C3A	QSOX2	MTSS2	FLNB	HMGN2	CD200
CELLMARKERMSIGDB_MURARO_PANCREAS_BETA_CELL	PKIB	PPM1E	KLHDC…	NR0B1	PIR	BTG3	NEXMIF	ROBO1	CSDE1	CDC42…	PAPSS2	SIX3	SQSTM1	TAGLN3	VWDE	TTC7B	OSBPL8	PEBP1	DNAJB9	ELAVL4	MGAT4A	IQSEC1	CABP7	RNF187	TPM3	CPD	NR3C1	SCG3	RIMKLA	HSPA1B
CELLMARKERMSIGDB_MURARO_PANCREAS_ENDOTHEL…	OLFML…	HEG1	MYLK	RAMP2	GIMAP8	DAB2	RAPGE…	ANGPT…	FCN3	LDB2	MSN	CYYR1	ITGA1	COL15…	ABI3	CLIC4	FLT4	DLC1	IFI27	VWF	PLEKH…	AFAP1…	EPAS1	LRRC8C	STOM	TGFBR2	RFLNB	MYCN	EFNA1	GBP4
CELLMARKERMSIGDB_MURARO_PANCREAS_EPSILON_…																														
CELLMARKERMSIGDB_MURARO_PANCREAS_MESENCHY…	CAV1	SULF1	PLAU	ITGA5	ZCCHC…	ACTA2	PLXDC1	KLF9	CCDC1…	FOXF2	ZNF469	MMP9	TGFB3	CAVIN1	MMP2	SRPX2	PLIN2	LAMP5	DACT1	MYLK	CCN4	LOX	CAVIN3	C1S	COL8A1	TBX2	MFGE8	ZEB2	NID2	TGFB1…
CELLMARKERMSIGDB_RUBENSTEIN_SKELETAL_MUSC…	CD44	UBE2D1	NAAA	CDKN1A	EFHD2	GPSM3	CARD16	GMFG	CCL3	TMSB4X	SH3BG…	PABPC1	ARPC1B	HLA-D…	AP2S1	LTA4H	CKLF	GLRX	OAZ1	ARPC3	GPX1	CASP1	SERP1	SAMHD1	PRELI…	LYST	DUSP1	UCP2	RAB5IF	ABRACL
CELLMARKERMSIGDB_RUBENSTEIN_SKELETAL_MUSC…	FGL2	RPL29	RPL22	HNRNP…	RPS8	LDHB	BTG2	WDR74	IMPDH2	RPL19	RBM3	SLC25…	SPG21	RPL22…	TSC22…	RPL18A	RSL24…	EEF1A1	NDRG2	BTF3	RPL17	TUBA1A	RPLP0	RPS15A	HDDC2	VDAC3	RPL36A	PEBP1	HEY1	CHCHD…
CELLMARKERMSIGDB_RUBENSTEIN_SKELETAL_MUSC…	COX8A	SELEN…	ITM2C	ZFHX3	PKIG	FILIP…	ENTPD1	SH3BG…	CAMTA1	NR2F2	NDUFB7	ATF3	PDLIM7	EFHD1	GBP2	PDLIM3	LDHB	C7orf…	MAP1B	COX7B	MOB2	SELEN…	PTK2	ARID5B	CSRP2	ATP5M…	CPE	UQCRB	CD9	VDAC2
CELLMARKERMSIGDB_TRAVAGLINI_LUNG_ALVEOLAR…	C1orf…	ANOS1	GGTLC1	EDN1	TIMP2	B2M	CLDN7	DAPK2	RRAS	HYAL1	KRT7	ATF3	ZBED2	SBSPON	DPYSL2	APLP2	LAMC2	NDST1	S100A4	ABLIM1	MYL6	OTUD1	TMEM1…	GGT1	CD9	RAB32	PIEZO1	TES	CDKN2B	IL18
CELLMARKERMSIGDB_TRAVAGLINI_LUNG_BASOPHIL…	ACOT7	TUBA1A	DDIT4	B4GAL…	RASGE…	SOCS3	PRKAR…	PTMA	DDX3X	PPP1R…	PEBP1	SRSF5	MAPK6	MLPH	MCL1	CSRNP1	SRSF3	STMN1	NDEL1	UBB	JUNB	GALC	MIR20…	SGK1	AHR	RNF145	FER	TRIM63	CTSG	ARHGE…
CELLMARKERMSIGDB_TRAVAGLINI_LUNG_CAPILLAR…																														
CELLMARKERMSIGDB_TRAVAGLINI_LUNG_CAPILLAR…	COX17	PDAP1	ITGB1	JCAD	RPL36…	UQCR10	S100A…	TMEM2…	RNF181	BLOC1…	CLTB	NDUFS5	GNG5	VAMP3	NDUFA4	RPS19…	NDUFS6	SRP9	MYH9	RWDD1	FKBP8	PSME1	GABAR…	HNRNPM	CALM3	CAPN2	NGRN	ATP5I…	IFI27…	NUCB2
CELLMARKERMSIGDB_TRAVAGLINI_LUNG_CD4_MEMO…																														
CELLMARKERMSIGDB_TRAVAGLINI_LUNG_EREG_DEN…	CTSL	MMP19	GLUL	MS4A7	S100A…	IVNS1…	RNASE6	FCAR	F13A1	LILRB2	PID1	TMSB10	ATP6V…	FTH1	OLR1	JARID2	FGL2	IL1RAP	GPR84	HLA-D…	FCGR2B	LIMS1	PHLDA2	SLC43…	TYROBP	SH3PX…	B3GNT5	RAC1	POMP	CSTA
CELLMARKERMSIGDB_TRAVAGLINI_LUNG_IGSF21_D…																														
CELLMARKERMSIGDB_TRAVAGLINI_LUNG_MESOTHEL…	HEG1	RPS18	DSG2	TPT1	RBP4	TMEM1…	GFPT2	RPL12	RPS2	EIF4E…	RPL17	ZNF330	SQOR	PROCR	ALOX5…	CRLS1	RPS15A	GCLM	PLAAT4	SERPI…	GMFB	PTPRF	DUSP4	HSPB11	LSR	RPS4Y1	MAP4K4	DPYSL3	MELTF	MST1
CELLMARKERMSIGDB_TRAVAGLINI_LUNG_MYOFIBRO…																														
CELLMARKERMSIGDB_TRAVAGLINI_LUNG_NATURAL_…																														
CELLMARKERMSIGDB_TRAVAGLINI_LUNG_NEUTROPH…	PHOSP…	COX7A2	PLAUR	CA4	IL17RA	PLIN5	CEACA…	IL18R…	CRISP…	PYGL	BEST1	UBALD2	HCLS1	PFKFB3	BBLN	ATP5F…	DAPK2	H3-3A	TMEM91	LIMK2	VAMP2	NDUFB1	IRAK3	NBEAL2	RPL7A…	ATG16…	PROK2	RASSF3	CTSS	COX5B
CELLMARKERMSIGDB_TRAVAGLINI_LUNG_OLR1_CLA…	TNFSF8	CTSB	PIK3A…	PELAT…	SIRPA	FNDC3B	ATP6V…	CSTA	HMGA1	RAC1	CCL3	SDC2	IL1A	AIF1	C5AR1	PTAFR	S100A…	SLC11…	MAP3K8	ITGA5	CCRL2	BID	PLSCR1	CFD	GNA15	NLRP3	CKAP4	CXCL2	C1orf…	JARID2
CELLMARKERMSIGDB_TRAVAGLINI_LUNG_PLATELET…	SCN1B	TNFSF4	RBX1	TPST2	XPNPE…	UBL4A	PTGS1	NEXN	AMD1	RSU1	FRMD3	TBXA2R	GNAZ	CLU	H2AC6	SMIM5	STON2	SCGB1…	STOM	ITGB3	EMC3	ARF1	F13A1	SPINT2	GPX4	NFE2	GRHL1	SNN	HGD	DAB2
CELLMARKERMSIGDB_TRAVAGLINI_LUNG_PROLIFER…	KNL1	NEK2	SLC25…	FANCI	DTYMK	PHF19	BUB1	ORC6	RNASE…	KIF11	ANP32B	CDCA2	HMGA1	CHEK2	GMNN	POC1A	CLSPN	CHEK1	DEK	ATAD5	PHGDH	CSTA	EZH2	BUB1B	RANBP1	GGH	MXD3	H1-3	REEP4	CDCA4
CELLMARKERMSIGDB_TRAVAGLINI_LUNG_PROLIFER…	FAM11…	POC1A	PKMYT1	MCEMP1	MS4A4A	SGO2	PTTG1	RAD51	ESCO2	MME	CKS2	TREM1	RETN	RRM1	PHGDH	TEX30	DEFB1	GMNN	TUBB6	FBP1	BUB1	PDLIM1	PHF19	FOXM1	KIF23	RACGA…	CTSD	EPHX1	PCOLC…	ARL6I…
CELLMARKERMSIGDB_TRAVAGLINI_LUNG_PROXIMAL…	FHL2	EIF3K	A4GALT	RPS3	LIME1	ORAI3	CHL1	SOX15	RPL18	SLITR…	LAYN	FBLN1	CAPNS2	RPS6	RPS9	ISYNA1	SYTL1	SOX2	EPAS1	RPL5	CTSD	MT1X	ARL4D	RPL7	TP63	SNHG32	RPL18A	RPL19	RAB38	HNRNP…
CELLMARKERMSIGDB_TRAVAGLINI_LUNG_SEROUS_C…																														
CELLMARKERMSIGDB_TRAVAGLINI_LUNG_SIGNALIN…																														
CELLMARKERMSIGDB_TRAVAGLINI_LUNG_TREM2_DE…	IGSF6	C5AR1	SLC1A3	AVPI1	TMEM51	CD9	CD74	PHLDA3	VSIG4	SNX10	VAT1	HSPB1	PTAFR	ABCC3	ATP6V…	HLA-D…	CXCL9	SLC7A7	CLEC4E	CD63	GM2A	C1orf…	CSF1R	DNASE2	GGTA1	SLC15…	CAVIN3	AKR1A1	FTH1	SCPEP1
CELLMARKERMSIGDB_TRAVAGLINI_LUNG_VASCULAR…																														
CELLMARKERMSIGDB_TRAVAGLINI_LUNG_VEIN_CELL																														
CELLMARKERMSIGDB_VANGURP_PANCREATIC_ALPHA…																														
CELLMARKERMSIGDB_VANGURP_PANCREATIC_DELTA…																														
CELLMARKERMSIGDB_VANGURP_PANCREATIC_GAMMA…																														
CELLMARKERMSIGDB_ZHENG_CORD_BLOOD_C1_PUTA…																														
CELLMARKERMSIGDB_ZHENG_CORD_BLOOD_C10_MUL…																														
CELLMARKERMSIGDB_ZHENG_CORD_BLOOD_C2_PUTA…																														
CELLMARKERMSIGDB_ZHENG_CORD_BLOOD_C4_PUTA…																														
CELLMARKERMSIGDB_ZHENG_CORD_BLOOD_C5_SIMI…																														
CELLMARKERMSIGDB_ZHENG_CORD_BLOOD_C6_HSC_…																														
CELLMARKERMSIGDB_ZHENG_CORD_BLOOD_C7_PUTA…																														
CELLMARKERMSIGDB_ZHENG_CORD_BLOOD_C8_PUTA…																														
CELLMARKERMSIGDB_ZHONG_PFC_C1_ASTROCYTE																														
CELLMARKERMSIGDB_ZHONG_PFC_C1_DLX5_POS_IN…																														
CELLMARKERMSIGDB_ZHONG_PFC_C2_ASCL1_POS_A…																														

Table S5: Cell state marker gene list

CELLMARKERCAN_ACINAR_CELLS																														
CELLMARKERMSIGDB_ZHONG_PFC_C2_UNKNOWN_NPC																														
CELLMARKERMSIGDB_ZHONG_PFC_C3_ASTROCYTE	PCLO	ENTRE…	VASH2	RPS6K…	PTPRU	ZNF445	PDP1	CACNA…	EXD3	AGAP2	CLDN5	CAMKV	STK11	RIMKLA	PSD	TMEM1…	TRIM46	SVOP	HMGA1	KCNT2	RESF1	SLC38…	PDCD7	ZNF324	MDN1	TMEM44	RFLNB	MEF2C	SLC4A3	TSPAN…
CELLMARKERMSIGDB_ZHONG_PFC_C3_HOPX_POS_OPC																														
CELLMARKERMSIGDB_ZHONG_PFC_C3_MICROGLIA	NCAM1	NETO2	PTPRO	DPYSL2	ZNF324	LPAR2	RNF40	ADCY1	BEX1	CDH10	NSG2	FADS3	MAP4	KCNA6	SGSM2	FCHO1	TUBB2B	FBXW7	TARS3	RAMP3	AMPH	SYP	CELSR2	B4GAT1	EPHA5	KIF1B	GNAL	NUDCD3	DPYSL4	MATK
CELLMARKERMSIGDB_ZHONG_PFC_C3_UNKNOWN_INP																														
CELLMARKERMSIGDB_ZHONG_PFC_C4_UNKNOWN_INP																														
CELLMARKERMSIGDB_ZHONG_PFC_C6_PLXNA4_POS_…																														
CELLMARKERMSIGDB_ZHONG_PFC_C7_ORG_UNDERGO…																														
CELLMARKERMSIGDB_ZHONG_PFC_C8_ORG_PROLIFE…																														
CELLMARKERMSIGDB_ZHONG_PFC_C8_UNKNOWN_NEU…																														
CELLMARKERMSIGDB_ZHONG_PFC_C9_ORG_OTHER																														
CELLMARKERMSIGDB_ZHONG_PFC_HES1_POS_C1_NPC																														
CELLMARKERMSIGDB_ZHONG_PFC_MAJOR_TYPES_AS…	SLC13…	NPNT	RIT2	SLC1A4	SPRY1	VCAM1	GLUL	ADCYA…	CDC42…	DCN	RFX4	GNG7	SIRPA	PDGFRB	LRRC1…	LRP4	PLEKH…	RGCC	ADD3	ZFP36…	LGALS1	METTL…	RGS20	LIX1	RHOC	AK4	NT5E	MMD2	LGALS…	PDLIM5
CELLMARKERMSIGDB_ZHONG_PFC_MAJOR_TYPES_EX…																														
CELLMARKERMSIGDB_ZHONG_PFC_MAJOR_TYPES_MI…	CTSS	LGMN	PLEK	MERTK	IER3	SELPLG	PLXDC2	JUNB	LAT2	LY96	GMFG	NEAT1	TMEM1…	GAL3S…	IFI30	DOCK8	CTSB	TBXAS1	IKZF1	C2	ARPC1B	GSN	CYBA	C3AR1	BIN2	NPL	SLC7A8	MGST2	STAB1	APOE
CELLMARKERMSIGDB_ZHONG_PFC_MAJOR_TYPES_OPC																														

CELLMARKERCAN_ACINAR_CELLS																														
CELLMARKERCAN_ADIPOCYTE_PROGENITOR_CELLS																														
CELLMARKERCAN_ADRENERGIC_NEURONS																														
CELLMARKERCAN_AIRWAY_EPITHELIAL_CELLS																														
CELLMARKERCAN_ALPHA_CELLS																														
CELLMARKERCAN_ALVEOLAR_MACROPHAGES																														
CELLMARKERCAN_ASTROCYTES																														
CELLMARKERCAN_B_CELLS																														
CELLMARKERCAN_B_CELLS_MEMORY																														
CELLMARKERCAN_B_CELLS_NAIVE																														
CELLMARKERCAN_BASOPHILS																														
CELLMARKERCAN_BERGMANN_GLIA																														
CELLMARKERCAN_BETA_CELLS																														
CELLMARKERCAN_CAJAL_RETZIUS_CELLS																														
CELLMARKERCAN_CARDIAC_STEM_AND_PRECURSOR_…																														
CELLMARKERCAN_CARDIOMYOCYTES																														
CELLMARKERCAN_CHOLANGIOCYTES																														
CELLMARKERCAN_CHOLINERGIC_NEURONS																														
CELLMARKERCAN_CHONDROCYTES																														
CELLMARKERCAN_CHOROID_PLEXUS_CELLS																														
CELLMARKERCAN_CILIATED_CELLS																														
CELLMARKERCAN_DELTA_CELLS																														
CELLMARKERCAN_DOPAMINERGIC_NEURONS																														
CELLMARKERCAN_DUCTAL_CELLS																														
CELLMARKERCAN_EMBRYONIC_STEM_CELLS																														
CELLMARKERCAN_ENTERIC_GLIA_CELLS																														
CELLMARKERCAN_ENTERIC_NEURONS																														
CELLMARKERCAN_ENTEROCYTES																														
CELLMARKERCAN_ENTEROENDOCRINE_CELLS																														
CELLMARKERCAN_EPENDYMAL_CELLS																														
CELLMARKERCAN_EPSILON_CELLS																														
CELLMARKERCAN_FOLLICULAR_CELLS																														
CELLMARKERCAN_FOVEOLAR_CELLS																														
CELLMARKERCAN_GAMMA_DELTA_T_CELLS																														
CELLMARKERCAN_GERM_CELLS																														
CELLMARKERCAN_GLUTAMINERGIC_NEURONS																														
CELLMARKERCAN_GRANULOSA_CELLS																														
CELLMARKERCAN_HEMATOPOIETIC_STEM_CELLS																														
CELLMARKERCAN_IMMATURE_NEURONS																														
CELLMARKERCAN_INTERNEURONS																														
CELLMARKERCAN_IONOCYTES																														
CELLMARKERCAN_KERATINOCYTES																														
CELLMARKERCAN_KUPFFER_CELLS																														
CELLMARKERCAN_LEYDIG_CELLS																														
CELLMARKERCAN_LOOP_OF_HENLE_CELLS																														
CELLMARKERCAN_LUMINAL_EPITHELIAL_CELLS																														
CELLMARKERCAN_LUTEAL_CELLS																														
CELLMARKERCAN_MEGAKARYOCYTES																														
CELLMARKERCAN_MELANOCYTES																														
CELLMARKERCAN_MESANGIAL_CELLS																														
CELLMARKERCAN_MESOTHELIAL_CELLS																														
CELLMARKERCAN_MICROFOLD_CELLS																														
CELLMARKERCAN_MICROGLIA																														
CELLMARKERCAN_MONOCYTES																														
CELLMARKERCAN_MOTOR_NEURONS																														
CELLMARKERCAN_MULLER_CELLS																														
CELLMARKERCAN_MYELOID_DERIVED_SUPPRESSOR_…																														
CELLMARKERCAN_MYOBLASTS																														
CELLMARKERCAN_MYOEPITHELIAL_CELLS																														
CELLMARKERCAN_MYOFIBROBLASTS																														
CELLMARKERCAN_NEUROBLASTS																														
CELLMARKERCAN_NEUROENDOCRINE_CELLS																														
CELLMARKERCAN_NEURONS																														
CELLMARKERCAN_NEUTROPHILS																														
CELLMARKERCAN_NK_CELLS																														
CELLMARKERCAN_NORADRENERGIC_NEURONS																														
CELLMARKERCAN_NUOCYTES																														
CELLMARKERCAN_OLIGODENDROCYTES																														
CELLMARKERCAN_OSTEOBLASTS																														
CELLMARKERCAN_OSTEOCLAST_PRECURSOR_CELLS																														
CELLMARKERCAN_OSTEOCLASTS																														
CELLMARKERCAN_OSTEOCYTES																														
CELLMARKERCAN_PANETH_CELLS																														
CELLMARKERCAN_PARATHYROID_CHIEF_CELLS																														
CELLMARKERCAN_PERICYTES																														
CELLMARKERCAN_PERITUBULAR_MYOID_CELLS																														
CELLMARKERCAN_PHOTORECEPTOR_CELLS																														
CELLMARKERCAN_PLASMA_CELLS																														
CELLMARKERCAN_PLASMACYTOID_DENDRITIC_CELLS																														
CELLMARKERCAN_PODOCYTES																														
CELLMARKERCAN_PROXIMAL_TUBULE_CELLS																														
CELLMARKERCAN_PULMONARY_ALVEOLAR_TYPE_I_C…																														
CELLMARKERCAN_PULMONARY_ALVEOLAR_TYPE_II_…																														
CELLMARKERCAN_PURKINJE_NEURONS																														
CELLMARKERCAN_RETICULOCYTES																														
CELLMARKERCAN_RETINAL_GANGLION_CELLS																														
CELLMARKERCAN_RETINAL_PROGENITOR_CELLS																														
CELLMARKERCAN_SATELLITE_CELLS																														
CELLMARKERCAN_SCHWANN_CELLS																														
CELLMARKERCAN_SEROTONERGIC_NEURONS																														
CELLMARKERCAN_SERTOLI_CELLS																														
CELLMARKERCAN_SMOOTH_MUSCLE_CELLS																														
CELLMARKERCAN_STROMAL_CELLS																														
CELLMARKERCAN_T_CELLS																														
CELLMARKERCAN_T_CYTOTOXIC_CELLS																														
CELLMARKERCAN_T_FOLLICULAR_HELPER_CELLS																														
CELLMARKERCAN_T_MEMORY_CELLS																														
CELLMARKERCAN_TRANSIENT_CELLS																														
CELLMARKERCAN_TRICHOCYTES																														
CELLMARKERCAN_TRIGEMINAL_NEURONS																														
CELLMARKERCAN_TROPHOBLAST_CELLS																														
CELLMARKERCAN_TROPHOBLAST_PROGENITOR_CELLS																														
CELLMARKERCAN_TUFT_CELLS																														
CELLMARKERCANENDOTHELIALCELLSAORTA																														
CELLMARKERCUR_N_ABNORMAL_MYELOID_CELL																														

CELLMARKERCAN_ACINAR_CELLS																														
CELLMARKERCUR_N_ABNORMAL_PLASMA_CELL																														
CELLMARKERCUR_N_ACTIVATED_DENDRITIC_CELL																														
CELLMARKERCUR_N_ACTIVATED_EFFECTOR_CELL																														
CELLMARKERCUR_N_ACTIVATED_HEPATIC_STELLAT…																														
CELLMARKERCUR_N_ACTIVATED_LANGERHANS_CELL																														
CELLMARKERCUR_N_ACTIVATED_MACROPHAGE																														
CELLMARKERCUR_N_ACTIVATED_MEMORY_T_CELL																														
CELLMARKERCUR_N_ACTIVATED_NATURAL_KILLER_…																														
CELLMARKERCUR_N_ACTIVATED_REGULATORY_T_CE…																														
CELLMARKERCUR_N_ADIPOCYTE_PROGENITOR_CELL																														
CELLMARKERCUR_N_ADRENAL_MEDULLA_CELL																														
CELLMARKERCUR_N_ADULT_SPERMATOGONIA																														
CELLMARKERCUR_N_ADVENTITIAL_CELL																														
CELLMARKERCUR_N_AEROCYTE																														
CELLMARKERCUR_N_AIRWAY_SMOOTH_MUSCLE_CELL																														
CELLMARKERCUR_N_ALVEOLAR_CELL_TYPE_1																														
CELLMARKERCUR_N_ALVEOLAR_CELL_TYPE_2																														
CELLMARKERCUR_N_ALVEOLAR_EPITHELIAL_CELL_…																														
CELLMARKERCUR_N_ALVEOLAR_EPITHELIAL_CELL_…																														
CELLMARKERCUR_N_ALVEOLAR_PNEUMOCYTE_TYPE_I																														
CELLMARKERCUR_N_ALVEOLAR_PNEUMOCYTE_TYPE_…																														
CELLMARKERCUR_N_AMACRINE_CELL																														
CELLMARKERCUR_N_AMNIOTIC_FLUID_STEM_CELL																														
CELLMARKERCUR_N_ANTERIOR_CELL																														
CELLMARKERCUR_N_ANTERIOR_HINDBRAIN_CELL																														
CELLMARKERCUR_N_ANTI_TUMOR_IMMUNE_CELL																														
CELLMARKERCUR_N_ANTIGEN_PRESENTATION_CANC…																														
CELLMARKERCUR_N_ASTROCYTE																														
CELLMARKERCUR_N_ATRIAL_CELL																														
CELLMARKERCUR_N_ATRIOVENTRICULAR_CANAL_CE…																														
CELLMARKERCUR_N_ATYPICAL_MEMORY_B_CELL																														
CELLMARKERCUR_N_B_2_CELL																														
CELLMARKERCUR_N_B1_CELL																														
CELLMARKERCUR_N_B10_REGULATORY_B_CELL																														
CELLMARKERCUR_N_BASAL_CELL																														
CELLMARKERCUR_N_BASAL_EPITHELIAL_CELL																														
CELLMARKERCUR_N_BASAL_KERATINOCYTE																														
CELLMARKERCUR_N_BASAL_LIKE_CELL																														
CELLMARKERCUR_N_BASAL_PROGENITOR_CELL																														
CELLMARKERCUR_N_BASOPHIL																														
CELLMARKERCUR_N_BILIARY_CELL																														
CELLMARKERCUR_N_BONE_MARROW_STEM_CELL																														
CELLMARKERCUR_N_BONE_SPECIFIC_CELL																														
CELLMARKERCUR_N_BRAIN_CELL																														
CELLMARKERCUR_N_CANCER_CELL	TNS2	MET	VCAM1	JAG1	THSD1	PSD	KIN	BCAM	C1GAL…	ENG	RSPO3	DOCK9	NOTCH1	KRT7	ESR1	BRCA1	BRCA2	PDCD1	TRPS1	PIP	NKX2-1	GATA3	EREG	CYP3A4	CYP3A5	CYP3A7	STAT1	RIC8A	IRF9	ISG15
CELLMARKERCUR_N_CANCER_STEM_LIKE_CELL																														
CELLMARKERCUR_N_CAPILLARY_CELL																														
CELLMARKERCUR_N_CAR_T_CELL																														
CELLMARKERCUR_N_CARDIAC_CELL																														
CELLMARKERCUR_N_CARDIOMYOCYTE																														
CELLMARKERCUR_N_CARDIOSPHERE_DERIVED_CELL																														
CELLMARKERCUR_N_CARDIOVASCULAR_PROGENITOR…																														
CELLMARKERCUR_N_CAUDAL_CORTEX_CELL																														
CELLMARKERCUR_N_CD14_MONOCYTE																														
CELLMARKERCUR_N_CD16_MONOCYTE																														
CELLMARKERCUR_N_CD4_CD28_T_CELL																														
CELLMARKERCUR_N_CD4_PERIPHERALLY_DERIVED_…																														
CELLMARKERCUR_N_CD4_T_CELL																														
CELLMARKERCUR_N_CD56BRIGHT_NATURAL_KILLER…																														
CELLMARKERCUR_N_CD56DIM_NATURAL_KILLER_CE…																														
CELLMARKERCUR_N_CD8_T_CELL																														
CELLMARKERCUR_N_CENTRAL_MEMORY_T_CELL																														
CELLMARKERCUR_N_CENTRAL_MEMORY_T_HELPER_1…																														
CELLMARKERCUR_N_CENTRAL_NERVOUS_SYSTEM_ST…																														
CELLMARKERCUR_N_CHANDELIER_CELL																														
CELLMARKERCUR_N_CHIEF_CELL																														
CELLMARKERCUR_N_CHONDROCYTE																														
CELLMARKERCUR_N_CHROMAFFIN_CELL																														
CELLMARKERCUR_N_CIRCULATING_ANGIOGENIC_CE…																														
CELLMARKERCUR_N_CIRCULATING_FETAL_CELL																														
CELLMARKERCUR_N_CIRCULATING_NATURAL_KILLE…																														
CELLMARKERCUR_N_CIRCULATING_PRECURSOR_CELL																														
CELLMARKERCUR_N_CIRCULATING_PROGENITOR_CE…																														
CELLMARKERCUR_N_CIRCULATING_T_FOLLICULAR_…																														
CELLMARKERCUR_N_CIRCULATING_TUMOR_CELL																														
CELLMARKERCUR_N_CLASS_SWITCHED_PLASMABLAST																														
CELLMARKERCUR_N_CLASSICAL_B_CELL																														
CELLMARKERCUR_N_CLASSICAL_CANCER_ASSOCIAT…																														
CELLMARKERCUR_N_CLASSICAL_MONOCYTE																														
CELLMARKERCUR_N_CLAUDIN_LOW_CELL																														
CELLMARKERCUR_N_COLLECTING_DUCTAL_CELL																														
CELLMARKERCUR_N_COLONCYTE																														
CELLMARKERCUR_N_COLORECTAL_STEM_CELL																														
CELLMARKERCUR_N_COLUMNAR_EPITHELIAL_CELL																														
CELLMARKERCUR_N_COMMON_LYMPHOID_PROGENITO…																														
CELLMARKERCUR_N_COMMON_MYELOID_PROGENITOR…																														
CELLMARKERCUR_N_COMPLEMENT_SECRETING_CANC…																														
CELLMARKERCUR_N_CONE_PHOTORECEPTOR_CELL																														
CELLMARKERCUR_N_CONNECTING_TUBULAR_CELL																														
CELLMARKERCUR_N_CONVENTIONAL_CD4_T_CELL																														
CELLMARKERCUR_N_CORNEAL_ENDOTHELIAL_CELL																														
CELLMARKERCUR_N_CORNEAL_EPITHELIAL_CELL																														
CELLMARKERCUR_N_CORNEOCYTE_LIKE_CELL																														
CELLMARKERCUR_N_CORTICAL_CELL																														
CELLMARKERCUR_N_CORTICAL_STROMAL_CELL																														
CELLMARKERCUR_N_CORTICOTROPH_TUMOR_CELL																														
CELLMARKERCUR_N_CRANIAL_NEURAL_CREST_CELL																														
CELLMARKERCUR_N_CRYPT_CELL																														
CELLMARKERCUR_N_CRYPT_STEM_CELL																														
CELLMARKERCUR_N_CYCLING_B_CELL																														
CELLMARKERCUR_N_CYCLING_EPITHELIAL_CELL																														
CELLMARKERCUR_N_CYTOTOXIC_CD8_T_CELL																														
CELLMARKERCUR_N_CYTOTOXIC_DOUBLE_POSITIVE…																														
CELLMARKERCUR_N_CYTOTOXIC_T_CELL																														

Table S5: Cell state marker gene list

CELLMARKERCAN_ACINAR_CELLS																														
CELLMARKERCUR_N_CYTOTOXIC_T_CELL_2																														
CELLMARKERCUR_N_DAMAGE_ASSOCIATED_TRANSIE…																														
CELLMARKERCUR_N_DARK_ZONE_CELL																														
CELLMARKERCUR_N_DEDIFFERENTIATION_ADIPOCY…																														
CELLMARKERCUR_N_DEEP_LAYER_NEURON																														
CELLMARKERCUR_N_DEFINITIVE_ENDODERM_CELL																														
CELLMARKERCUR_N_DELTA_CELL																														
CELLMARKERCUR_N_DEMYELINATING_MICROGLIAL_…																														
CELLMARKERCUR_N_DENDRITIC_CELL_2A																														
CELLMARKERCUR_N_DENDRITIC_CELL_LINEAGE																														
CELLMARKERCUR_N_DENTAL_PULP_CELL																														
CELLMARKERCUR_N_DERMAL_CELL																														
CELLMARKERCUR_N_DEUTEROSOMAL_CELL																														
CELLMARKERCUR_N_DIFFUSE_GASTRIC_CANCER_CE…																														
CELLMARKERCUR_N_DISTAL_CELL																														
CELLMARKERCUR_N_DISTAL_STEM_CELL																														
CELLMARKERCUR_N_DISTAL_TUBULAR_CELL																														
CELLMARKERCUR_N_DIVIDING_CELL																														
CELLMARKERCUR_N_DIVIDING_PLASMA_B_CELL																														
CELLMARKERCUR_N_DIVIDING_T_CELL																														
CELLMARKERCUR_N_DOPAMINERGIC_NEURON																														
CELLMARKERCUR_N_DORSAL_ANTERIOR_HINDBRAIN…																														
CELLMARKERCUR_N_DORSAL_LATERAL_GANGLIONIC…																														
CELLMARKERCUR_N_DORSAL_MEDIAL_GANGLIONIC_…																														
CELLMARKERCUR_N_DOUBLE_NEGATIVE_B_CELL																														
CELLMARKERCUR_N_DOUBLE_NEGATIVE_T_CELL																														
CELLMARKERCUR_N_DOUBLE_POSITIVE_T_CELL																														
CELLMARKERCUR_N_DOUBLE_POSITIVE_TREG_CELL																														
CELLMARKERCUR_N_EARLY_HEMANGIOBLAST																														
CELLMARKERCUR_N_EARLY_PROXIMAL_TUBULE_CELL																														
CELLMARKERCUR_N_EARLY_RESPONSE_SECRETORY_…																														
CELLMARKERCUR_N_EFFECTOR_CHONDROCYTE																														
CELLMARKERCUR_N_EFFECTOR_MEMORY_T_CELL																														
CELLMARKERCUR_N_EFFECTOR_NATURAL_KILLER_C…																														
CELLMARKERCUR_N_ELONGATING_CELL																														
CELLMARKERCUR_N_EMBRYONIC_CELL																														
CELLMARKERCUR_N_EMBRYONIC_STEM_CELL																														
CELLMARKERCUR_N_ENDOCARDIAL_CELL																														
CELLMARKERCUR_N_ENDOCRINE_PROGENITOR_CELL																														
CELLMARKERCUR_N_ENDODERM_PROGENITOR_CELL																														
CELLMARKERCUR_N_ENDODERMAL_CELL																														
CELLMARKERCUR_N_ENDOMETRIAL_STROMAL_CELL																														
CELLMARKERCUR_N_ENDOMETRIAL_STROMAL_STEM_…																														
CELLMARKERCUR_N_ENDOTHELIAL_PRECURSOR_CELL																														
CELLMARKERCUR_N_ENSHEATHING_GLIAL_CELL																														
CELLMARKERCUR_N_ENTERIC_NEURON																														
CELLMARKERCUR_N_EOSINOPHIL																														
CELLMARKERCUR_N_EPENDYMAL_CELL																														
CELLMARKERCUR_N_EPIBLAST_CELL																														
CELLMARKERCUR_N_EPICARDIAL_CELL																														
CELLMARKERCUR_N_EPICARDIUM_DERIVED_CELL																														
CELLMARKERCUR_N_EPIDERMAL_CELL																														
CELLMARKERCUR_N_EPIDERMAL_STEM_CELL																														
CELLMARKERCUR_N_EPITHELIAL_CELL	TRPM5	SCGN	CHGA	CA1	KRT20	ENPEP	SI	FABP7	APOA1	NEURO…	TPH1	C8G	UBD	TRIM31	NCOA7	CD3D	ATG5	SKAP2	PPM1G	TAGLN2	PRDX5	TEK	PAX2	MME	L1CAM	SMAD4	NCAM1	BAP1	S100P	CPED1
CELLMARKERCUR_N_EPITHELIAL_DUCTAL_CELL																														
CELLMARKERCUR_N_EPITHELIAL_MESENCHYMAL_CE…																														
CELLMARKERCUR_N_EPITHELIOID_MACROPHAGE																														
CELLMARKERCUR_N_EPSILON_CELL																														
CELLMARKERCUR_N_ERYTHROBLAST																														
CELLMARKERCUR_N_ERYTHROID_CELL																														
CELLMARKERCUR_N_ERYTHROID_PROGENITOR_CELL																														
CELLMARKERCUR_N_ESOPHAGEAL_CELL																														
CELLMARKERCUR_N_ESOPHAGEAL_PROGENITOR_CELL																														
CELLMARKERCUR_N_EXCITATORY_NEURON																														
CELLMARKERCUR_N_EXHAUSTED_DOUBLE_POSITIVE…																														
CELLMARKERCUR_N_EXTRAVILLOUS_TROPHOBLAST																														
CELLMARKERCUR_N_F_PERICYTE																														
CELLMARKERCUR_N_FETAL_MYOBLAST																														
CELLMARKERCUR_N_FIBROBLAST_LIKE_CELL																														
CELLMARKERCUR_N_FIBROCHONDROCYTE																														
CELLMARKERCUR_N_FIBROCHONDROCYTE_PROGENIT…																														
CELLMARKERCUR_N_FOAM_CELL																														
CELLMARKERCUR_N_FOLLICULAR_B_CELL																														
CELLMARKERCUR_N_FOLLICULAR_CELL																														
CELLMARKERCUR_N_FOLLICULAR_DENDRITIC_CELL																														
CELLMARKERCUR_N_FOREBRAIN_CELL																														
CELLMARKERCUR_N_GABAERGIC_NEURON																														
CELLMARKERCUR_N_GANGLION_CELL																														
CELLMARKERCUR_N_GASTRIC_NEUROENDOCRINE_CE…																														
CELLMARKERCUR_N_GASTRIC_STEM_CELL																														
CELLMARKERCUR_N_GERM_CELL																														
CELLMARKERCUR_N_GERMINAL_CENTER_B_CELL																														
CELLMARKERCUR_N_GERMINAL_CENTER_CELL																														
CELLMARKERCUR_N_GERMINAL_CENTRE_B_CELL																														
CELLMARKERCUR_N_GLIAL_CELL																														
CELLMARKERCUR_N_GLIOMA_STEM_CELL																														
CELLMARKERCUR_N_GLOBOSE_BASAL_CELL																														
CELLMARKERCUR_N_GLOMERULAR_PARIETAL_EPITH…																														
CELLMARKERCUR_N_GLUTAMATERGIC_NEURON																														
CELLMARKERCUR_N_GOBLET_CELL																														
CELLMARKERCUR_N_GONADOTROPE																														
CELLMARKERCUR_N_GONADOTROPH_CELL																														
CELLMARKERCUR_N_GRANULOCYTE																														
CELLMARKERCUR_N_GRANULOCYTE_MONOCYTE_PROG…																														
CELLMARKERCUR_N_GRANULOCYTIC_UNK																														
CELLMARKERCUR_N_GRANULOSA_CELL																														
CELLMARKERCUR_N_HAEMATOPOIETIC_CELL																														
CELLMARKERCUR_N_HAIR_FOLLICLE_CELL																														
CELLMARKERCUR_N_HEMANGIOBLAST																														
CELLMARKERCUR_N_HEMATOPOIETIC_CELL																														
CELLMARKERCUR_N_HEMATOPOIETIC_PRECURSOR_C…																														
CELLMARKERCUR_N_HEMATOPOIETIC_PROGENITOR_…																														
CELLMARKERCUR_N_HEPATIC_CELL																														
CELLMARKERCUR_N_HEPATIC_PROGENITOR_CELL																														

CELLMARKERCAN_ACINAR_CELLS																														
CELLMARKERCUR_N_HEPATIC_STELLATE_CELL																														
CELLMARKERCUR_N_HEPATOBLAST																														
CELLMARKERCUR_N_HEPATOCYTE																														
CELLMARKERCUR_N_HISTIOCYTE																														
CELLMARKERCUR_N_HMSC_TREATED_B_CELL																														
CELLMARKERCUR_N_HOFBAUER_CELL																														
CELLMARKERCUR_N_HOMEOSTATIC_CHONDROCYTE																														
CELLMARKERCUR_N_HOMEOSTATIC_MICROGLIAL_CE…																														
CELLMARKERCUR_N_HORIZONTAL_BASAL_CELL																														
CELLMARKERCUR_N_HORIZONTAL_CELL																														
CELLMARKERCUR_N_HYPOBLAST_CELL																														
CELLMARKERCUR_N_IDIOPATHIC_PULMONARY_FIBR…																														
CELLMARKERCUR_N_IGA_MEMORY_B_CELL																														
CELLMARKERCUR_N_IGG_MEMORY_B_CELL																														
CELLMARKERCUR_N_IGM_MEMORY_B_CELL																														
CELLMARKERCUR_N_IGM_ONLY_B_CELL																														
CELLMARKERCUR_N_IL7R_T_HELPER_CELL																														
CELLMARKERCUR_N_IMMATURE_ERYTHROID_CELL																														
CELLMARKERCUR_N_IMMATURE_MYELOID_CELL																														
CELLMARKERCUR_N_IMMATURE_NEUTROPHIL																														
CELLMARKERCUR_N_IMMATURE_TRANSITIONAL_B_C…																														
CELLMARKERCUR_N_IMMUNE_CELL																														
CELLMARKERCUR_N_INDUCED_PLURIPOTENT_STEM_…																														
CELLMARKERCUR_N_INFILTRATING_MACROPHAGE																														
CELLMARKERCUR_N_INFLAMMATORY_CANCER_ASSOC…																														
CELLMARKERCUR_N_INFLAMMATORY_DENDRITIC_CE…																														
CELLMARKERCUR_N_INFLAMMATORY_MONOCYTE																														
CELLMARKERCUR_N_INHIBITORY_NEURON																														
CELLMARKERCUR_N_INNATE_LYMPHOID_CELL																														
CELLMARKERCUR_N_INNER_CELL_MASS_CELL																														
CELLMARKERCUR_N_INNER_RADIAL_GLIAL_CELL																														
CELLMARKERCUR_N_INTER_MEDIATE_PROGENITOR_…																														
CELLMARKERCUR_N_INTERCALATED_CELL																														
CELLMARKERCUR_N_INTERFERON_RESPONSIVE_SEC…																														
CELLMARKERCUR_N_INTERMEDIATE_MONOCYTE																														
CELLMARKERCUR_N_INTERMEDIATE_MONOCYTE_CELL																														
CELLMARKERCUR_N_INTERSTITIAL_MACROPHAGE																														
CELLMARKERCUR_N_INTERSTITIAL_PROGENITOR_C…																														
CELLMARKERCUR_N_INTESTINAL_EPITHELIAL_STE…																														
CELLMARKERCUR_N_INTESTINAL_GASTRIC_CANCER…																														
CELLMARKERCUR_N_INTESTINAL_METAPLASIA_CELL																														
CELLMARKERCUR_N_INTRAEPITHELIAL_LYMPHOCYT…																														
CELLMARKERCUR_N_IONOCYTE																														
CELLMARKERCUR_N_IRIS_PUTTATIVE_STEM_CELL																														
CELLMARKERCUR_N_KUPFFER_CELL																														
CELLMARKERCUR_N_LACTOTROPH_CELL																														
CELLMARKERCUR_N_LANGERHANS_CELL																														
CELLMARKERCUR_N_LEUKOCYTE																														
CELLMARKERCUR_N_LIMBAL_EPITHELIAL_STEM_CE…																														
CELLMARKERCUR_N_LIMBAL_STEM_CELL																														
CELLMARKERCUR_N_LIPOFIBROBLAST																														
CELLMARKERCUR_N_LIVER_PROGENITOR_CELL																														
CELLMARKERCUR_N_LIVER_STEM_CELL																														
CELLMARKERCUR_N_LMMLEYDIG_CELL																														
CELLMARKERCUR_N_LONOCYTE																														
CELLMARKERCUR_N_LOOP_OF_HENLE_CELL																														
CELLMARKERCUR_N_LOOP_PROGENITOR_CELL																														
CELLMARKERCUR_N_LOW_DENSITY_NEUTROPHIL																														
CELLMARKERCUR_N_LUMINAL_PROGENITOR_CELL																														
CELLMARKERCUR_N_LUNG_CELL																														
CELLMARKERCUR_N_LYMPH_NODE_CELL																														
CELLMARKERCUR_N_LYMPHATIC_CELL																														
CELLMARKERCUR_N_LYMPHATIC_ENDOTHELIAL_CELL																														
CELLMARKERCUR_N_LYMPHOBLASTOID_CELL																														
CELLMARKERCUR_N_LYMPHOCYTE																														
CELLMARKERCUR_N_LYMPHOID_CELL																														
CELLMARKERCUR_N_LYMPHOID_PROGENITOR_CELL																														
CELLMARKERCUR_N_LYMPHOID_STRUCTURE_CELL																														
CELLMARKERCUR_N_M_PERICYTE																														
CELLMARKERCUR_N_M1_MACROPHAGE																														
CELLMARKERCUR_N_M1_MICROGLIAL_CELL																														
CELLMARKERCUR_N_M2_MACROPHAGE																														
CELLMARKERCUR_N_MACROGLIAL_CELL																														
CELLMARKERCUR_N_MALIGNANT_CELL																														
CELLMARKERCUR_N_MALIGNANT_PLASMA_CELL																														
CELLMARKERCUR_N_MARGINAL_ASSOCIATED_MACRO…																														
CELLMARKERCUR_N_MAST_CELL																														
CELLMARKERCUR_N_MATURE_ASTROCYTE																														
CELLMARKERCUR_N_MATURE_B_CELL																														
CELLMARKERCUR_N_MATURE_DENDRITIC_CELL																														
CELLMARKERCUR_N_MATURE_ENDOTHELIAL_CELL																														
CELLMARKERCUR_N_MATURE_LUMINAL_CELL																														
CELLMARKERCUR_N_MATURE_NEURON																														
CELLMARKERCUR_N_MATURE_NEURONAL																														
CELLMARKERCUR_N_MATURE_OLIGODENDROCYTE																														
CELLMARKERCUR_N_MATURE_OSTEOBLAST																														
CELLMARKERCUR_N_MATURE_PODOCYTE																														
CELLMARKERCUR_N_MEDIAL_AND_DISTAL_SEGMENT…																														
CELLMARKERCUR_N_MEDIAL_GANGLIONIC_EMINENC…																														
CELLMARKERCUR_N_MEGAKARYOCYTE																														
CELLMARKERCUR_N_MEGAKARYOCYTE_ERYTHROID_C…																														
CELLMARKERCUR_N_MEGAKARYOCYTE_ERYTHROID_P…																														
CELLMARKERCUR_N_MEGAKARYOCYTE_PROGENITOR_…																														
CELLMARKERCUR_N_MELANOMA_CELL																														
CELLMARKERCUR_N_MEMORY_B_CELL																														
CELLMARKERCUR_N_MEMORY_B_CELL_PRECURSOR																														
CELLMARKERCUR_N_MEMORY_CD27_B_CELL																														
CELLMARKERCUR_N_MEMORY_CD4_T_CELL																														
CELLMARKERCUR_N_MEMORY_DOUBLE_POSITIVE_T_…																														
CELLMARKERCUR_N_MEMORY_REGULATORY_T_CELL																														
CELLMARKERCUR_N_MEMORY_T_CELL																														
CELLMARKERCUR_N_MERKEL_CELL																														
CELLMARKERCUR_N_MESENCHYMAL_CELL																														
CELLMARKERCUR_N_MESENCHYMAL_EPITHELIAL_TR…																														

CELLMARKERCAN_ACINAR_CELLS																														
CELLMARKERCUR_N_MESENCHYMAL_GLIOBLASTOMA_…																														
CELLMARKERCUR_N_MESENCHYMAL_STEM_CELL																														
CELLMARKERCUR_N_MESENCHYMAL_STROMAL_CELL																														
CELLMARKERCUR_N_MESENDODERMAL_CELL																														
CELLMARKERCUR_N_MESODERMAL_EMBRYONIC_STEM…																														
CELLMARKERCUR_N_MESODERMAL_PROGENITOR_CELL																														
CELLMARKERCUR_N_METAPLASTIC_STEM_CELL																														
CELLMARKERCUR_N_MEYLOID_CELL																														
CELLMARKERCUR_N_MICROGLIAL_CELL	GSTM5	COLEC…	TPT1	ACD	RPL37	DPYS	SMAD7	RPS20	LRPAP1	CBX6	RPLP2	ZNF703	PDCD1	PLAUR	A1BG	ADAM8	TLN2	RPS16	NCEH1	EEF1A1	LGALS…	ETL4	RPL23	ATP1A3	LOX	CTBP1…	AQP6	TTYH3	RPS15	RPS12
CELLMARKERCUR_N_MICROVILLAR_CELL																														
CELLMARKERCUR_N_MIDBRAIN_DOPAMINERGIC_NEU…																														
CELLMARKERCUR_N_MONO_MACROPHAGE																														
CELLMARKERCUR_N_MONOCYTE_DERIVED_CELL																														
CELLMARKERCUR_N_MONOCYTE_DERIVED_MYELOID_…																														
CELLMARKERCUR_N_MONOCYTE_LINEAGE																														
CELLMARKERCUR_N_MONOCYTE_PRECURSOR																														
CELLMARKERCUR_N_MONOCYTE_PROGENITOR_CELL																														
CELLMARKERCUR_N_MUELLER_GLIA_CELL																														
CELLMARKERCUR_N_MULTIPOTENT_MESENCHYMAL_S…																														
CELLMARKERCUR_N_MULTIPOTENT_RETINAL_PROGE…																														
CELLMARKERCUR_N_MULTIPOTENT_STEM_CELL																														
CELLMARKERCUR_N_MULTIPOTENT_STROMAL_CELL																														
CELLMARKERCUR_N_MUSCLE_CELL																														
CELLMARKERCUR_N_MUSCLE_DERIVED_CELL																														
CELLMARKERCUR_N_MUSCLE_SATELLITE_CELL																														
CELLMARKERCUR_N_MYELOBLAST																														
CELLMARKERCUR_N_MYELOID_DENDRITIC_CELL																														
CELLMARKERCUR_N_MYELOID_DENDRITIC_CELL_1																														
CELLMARKERCUR_N_MYELOID_DERIVED_CELL																														
CELLMARKERCUR_N_MYELOID_DERIVED_SUPPRESSO…																														
CELLMARKERCUR_N_MYELOID_PROGENITOR_CELL																														
CELLMARKERCUR_N_MYO_EPITHELIAL_CELL																														
CELLMARKERCUR_N_MYOBLAST																														
CELLMARKERCUR_N_MYOCYTE																														
CELLMARKERCUR_N_MYOFIBROBLAST																														
CELLMARKERCUR_N_MYOGENIC_ENDOTHELIAL_CELL																														
CELLMARKERCUR_N_MYOGENIC_PROGENITOR_CELL																														
CELLMARKERCUR_N_MYOID_CELL																														
CELLMARKERCUR_N_NAIVE_B_CELL																														
CELLMARKERCUR_N_NAIVE_CD8_T_CELL																														
CELLMARKERCUR_N_NAIVE_DOUBLE_POSITIVE_T_C…																														
CELLMARKERCUR_N_NAIVE_PROGENITOR_CELL																														
CELLMARKERCUR_N_NAIVE_T_CELL																														
CELLMARKERCUR_N_NASCENT_NEPHRON_CELL																														
CELLMARKERCUR_N_NATURAL_KILLER_CD56_BRIGH…																														
CELLMARKERCUR_N_NATURAL_KILLER_CD56_DIM_C…																														
CELLMARKERCUR_N_NEGATIVE_MESENCHYMAL_STEM…																														
CELLMARKERCUR_N_NEURAL_CELL																														
CELLMARKERCUR_N_NEURAL_CREST_STEM_CELL																														
CELLMARKERCUR_N_NEURAL_CREST_STEMLIKE_CELL																														
CELLMARKERCUR_N_NEURAL_PRECURSOR_CELL																														
CELLMARKERCUR_N_NEURAL_STEM_CELL																														
CELLMARKERCUR_N_NEUROECTODERM_CELL																														
CELLMARKERCUR_N_NEURONAL_INTERMEDIATE_PRO…																														
CELLMARKERCUR_N_NEURONAL_PROGENITOR_CELL																														
CELLMARKERCUR_N_NEUTROPHIL_PROGENITOR_CELL																														
CELLMARKERCUR_N_NON_NEURON																														
CELLMARKERCUR_N_NON_PT_TUBULAR_CELL																														
CELLMARKERCUR_N_NON_RECIRCULATING_TISSUE_…																														
CELLMARKERCUR_N_NONMYELINATING_SCHWANN_CE…																														
CELLMARKERCUR_N_NOTOCHORD_CELL																														
CELLMARKERCUR_N_NUCLEATED_CELL																														
CELLMARKERCUR_N_NUCLEUS_PULPOSUS_PROGENIT…																														
CELLMARKERCUR_N_OLIGODENDROCYTE																														
CELLMARKERCUR_N_OSTEOBLAST																														
CELLMARKERCUR_N_OUTER_RADIAL_GLIA																														
CELLMARKERCUR_N_OVALBUMIN_SPECIFIC_REGULA…																														
CELLMARKERCUR_N_OVARIAN_CELL																														
CELLMARKERCUR_N_OVARIAN_STROMAL_CELL																														
CELLMARKERCUR_N_P53_LIKE_CELL																														
CELLMARKERCUR_N_PAN_B_CELL																														
CELLMARKERCUR_N_PAN_CANCER_ASSOCIATED_FIB…																														
CELLMARKERCUR_N_PAN_MACROPHAGE																														
CELLMARKERCUR_N_PAN_MONOCYTE																														
CELLMARKERCUR_N_PAN_NEURONAL_CELL																														
CELLMARKERCUR_N_PAN_TROPHECTODERM_CELL																														
CELLMARKERCUR_N_PANCREATIC_ENDODERM_CELL																														
CELLMARKERCUR_N_PANCREATIC_POLYPEPTIDE_CE…																														
CELLMARKERCUR_N_PANCREATIC_PROGENITOR_CELL																														
CELLMARKERCUR_N_PANCREATIC_STEM_CELL																														
CELLMARKERCUR_N_PANETH_CELL																														
CELLMARKERCUR_N_PANNEURON																														
CELLMARKERCUR_N_PANVASCULAR_CELL																														
CELLMARKERCUR_N_PAPILLARY_FIBROBLAST																														
CELLMARKERCUR_N_PARIETAL_CELL																														
CELLMARKERCUR_N_PARVALBUMIN_INTERNEURON																														
CELLMARKERCUR_N_PATELET																														
CELLMARKERCUR_N_PATHOGENIC_T_PERIPHERAL_H…																														
CELLMARKERCUR_N_PELVIC_EPITHELIAL_CELL																														
CELLMARKERCUR_N_PERICYTE																														
CELLMARKERCUR_N_PERIODONTAL_LIGAMENT_STEM…																														
CELLMARKERCUR_N_PERIOSTEUM_DERIVED_PROGEN…																														
CELLMARKERCUR_N_PERITUBULAR_MYOID_CELL																														
CELLMARKERCUR_N_PERIVASCULAR_LIKE_CELL																														
CELLMARKERCUR_N_PHAGOCYTE																														
CELLMARKERCUR_N_PHOTORECEPTOR_CELL																														
CELLMARKERCUR_N_PIT_CELL																														
CELLMARKERCUR_N_PIT_MUCOUS_CELL																														
CELLMARKERCUR_N_PITUITARY_STEM_CELL																														
CELLMARKERCUR_N_PLACENTAL_TISSUE_CELL																														
CELLMARKERCUR_N_PLACODE_CELL																														
CELLMARKERCUR_N_PLASMABLAST_B_CELL																														
CELLMARKERCUR_N_PLATELET																														
CELLMARKERCUR_N_PLURIPOTENT_STEM_CELL																														

CELLMARKERCAN_ACINAR_CELLS																														
CELLMARKERCUR_N_PODOCYTE_PRECURSOR																														
CELLMARKERCUR_N_PORTAL_FIBROBLAST																														
CELLMARKERCUR_N_POSTERIOR_CELL																														
CELLMARKERCUR_N_PRE_B_CELL																														
CELLMARKERCUR_N_PRE_CONVENTIONAL_DENDRITI…																														
CELLMARKERCUR_N_PRE_DENDRITIC_CELL																														
CELLMARKERCUR_N_PRECURSOR_CELL																														
CELLMARKERCUR_N_PREHYPERTROPHIC_CHONDROCY…																														
CELLMARKERCUR_N_PRESOMITE_CELL																														
CELLMARKERCUR_N_PRETUBULAR_AGGREGATE_CELL																														
CELLMARKERCUR_N_PRIMITIVE_ENDODERM_CELL																														
CELLMARKERCUR_N_PRINCIPAL_CELL																														
CELLMARKERCUR_N_PRO_B_CELL																														
CELLMARKERCUR_N_PRO_INFLAMMATORY_MACROPHA…																														
CELLMARKERCUR_N_PRO_NEUTROPHIL																														
CELLMARKERCUR_N_PROLIFERATING_CELL																														
CELLMARKERCUR_N_PROLIFERATIVE_CHONDROCYTE																														
CELLMARKERCUR_N_PROLIFERATIVE_DUCTAL_CELL																														
CELLMARKERCUR_N_PROLIFERATIVE_NEURAL_PROG…																														
CELLMARKERCUR_N_PRONEURAL_GLIOBLASTOMA_CE…																														
CELLMARKERCUR_N_PROSPERMATOGONIA																														
CELLMARKERCUR_N_PROSTATE_CELL																														
CELLMARKERCUR_N_PROXIMAL_CELL																														
CELLMARKERCUR_N_PROXIMAL_SEGMENT_CELL																														
CELLMARKERCUR_N_PROXIMAL_STEM_CELL																														
CELLMARKERCUR_N_PROXIMAL_TUBULE_CELL																														
CELLMARKERCUR_N_PULMONARY_NEUROENDOCRINE_…																														
CELLMARKERCUR_N_PYRAMIDAL_CELL																														
CELLMARKERCUR_N_QUIESCENT_T_STEM_CELL																														
CELLMARKERCUR_N_REGULATORY_B_CELL																														
CELLMARKERCUR_N_REGULATORY_INNATE_LYMPHOI…																														
CELLMARKERCUR_N_RENAL_TUBULAR_CELL																														
CELLMARKERCUR_N_RESIDENT_MEMORY_T_CELL																														
CELLMARKERCUR_N_RESPIRATORY_HORIZONTAL_BA…																														
CELLMARKERCUR_N_RESPIRATORY_SECRETORY_CELL																														
CELLMARKERCUR_N_RESPONDER_T_CELL																														
CELLMARKERCUR_N_RESPONDING_CONVENTIONAL_T…																														
CELLMARKERCUR_N_RESTING_MEMORY_B_CELL																														
CELLMARKERCUR_N_RESTING_NAIVE_B_CELL																														
CELLMARKERCUR_N_RESTING_REGULATORY_T_CELL																														
CELLMARKERCUR_N_RETICULAR_FIBROBLAST																														
CELLMARKERCUR_N_RETINA_CELL																														
CELLMARKERCUR_N_ROD_CELL																														
CELLMARKERCUR_N_SATELLITE_CELL																														
CELLMARKERCUR_N_SEPTUM_TRANSVERSUM_MESENC…																														
CELLMARKERCUR_N_SEROUS_CELL																														
CELLMARKERCUR_N_SIDE_POPULATION_CELL																														
CELLMARKERCUR_N_SKELETAL_MUSCLE_CELL																														
CELLMARKERCUR_N_SKELETAL_MUSCLE_STEM_CELL																														
CELLMARKERCUR_N_SMOOTH_MUSCLE_CELL																														
CELLMARKERCUR_N_SMOOTH_MUSCLE_LIKE_CELL																														
CELLMARKERCUR_N_SOMATIC_CELL																														
CELLMARKERCUR_N_SOMATIC_STEM_CELL																														
CELLMARKERCUR_N_SOMATOSTATIN_INTERNEURON																														
CELLMARKERCUR_N_SPECIALIST_ANTIGEN_PRESEN…																														
CELLMARKERCUR_N_SPERMATOGENIC_CELL																														
CELLMARKERCUR_N_SPERMATOGONIAL_CELL																														
CELLMARKERCUR_N_SPERMATOGONIAL_STEM_CELL																														
CELLMARKERCUR_N_SQUAMOUS_DIFFERENTIATION_…																														
CELLMARKERCUR_N_STELLATE_CELL																														
CELLMARKERCUR_N_STEM_CELL_DERIVED_ALPHA_C…																														
CELLMARKERCUR_N_STEM_CELL_DERIVED_BETA_CE…																														
CELLMARKERCUR_N_STEM_CELL_DERIVED_ENDOTHE…																														
CELLMARKERCUR_N_STEM_LIKE_CD8_T_CELL																														
CELLMARKERCUR_N_STROMAL_CELL																														
CELLMARKERCUR_N_SUBPALLIUM_CELL																														
CELLMARKERCUR_N_SUBPLATE_CELL																														
CELLMARKERCUR_N_SUPPRESSIVE_MONOCYTE																														
CELLMARKERCUR_N_SUPPRESSOR_T_CELL																														
CELLMARKERCUR_N_SUPRABASAL_CELL																														
CELLMARKERCUR_N_SURFACE_ECTODERM_CELL																														
CELLMARKERCUR_N_SUSTENTACULAR_CELL																														
CELLMARKERCUR_N_SYMPATHOADRENAL_CELL																														
CELLMARKERCUR_N_SYNCYTIOTROPHOBLAST																														
CELLMARKERCUR_N_SYNTHETIC_SMOOTH_MUSCLE_C…																														
CELLMARKERCUR_N_T_CELL_LARGE_GRANULAR_LYM…																														
CELLMARKERCUR_N_T_CELL_LINEAGE																														
CELLMARKERCUR_N_T_HELPER_CELL																														
CELLMARKERCUR_N_T_MEMORY_STEM_CELL																														
CELLMARKERCUR_N_T2_MZP_REGULATORY_B_CELL																														
CELLMARKERCUR_N_TENDON_CELL																														
CELLMARKERCUR_N_TERMINAL_DIFFERENTIATED_B…																														
CELLMARKERCUR_N_THALAMIC_CELL																														
CELLMARKERCUR_N_THECA_INTERNA_CELL																														
CELLMARKERCUR_N_THYMIC_EPITHELIAL_CELL																														
CELLMARKERCUR_N_TISSUE_LIKE_MEMORY_B_CELL																														
CELLMARKERCUR_N_TISSUE_RESIDENT_CELL																														
CELLMARKERCUR_N_TISSUE_RESIDENT_EFFECTOR_…																														
CELLMARKERCUR_N_TISSUE_RESIDENT_MACROPHAGE																														
CELLMARKERCUR_N_TISSUE_RESIDENT_MEMORY_LI…																														
CELLMARKERCUR_N_TOLDC_INDUCED_REGULATORY_…																														
CELLMARKERCUR_N_TONSIL_CELL																														
CELLMARKERCUR_N_TRABECULAR_MESHWORK_CELL																														
CELLMARKERCUR_N_TRANSITIONAL_B_CELL																														
CELLMARKERCUR_N_TRANSITIONAL_DENDRITIC_CE…																														
CELLMARKERCUR_N_TRUNCATED_RADIAL_GLIAL_CE…																														
CELLMARKERCUR_N_TUBULAR_CELL																														
CELLMARKERCUR_N_TUMOR_ASSOCIATED_MICROGLI…																														
CELLMARKERCUR_N_TUMOR_ENDOTHELIAL_CELL																														
CELLMARKERCUR_N_TUMOR_EPITHELIAL_CELL																														
CELLMARKERCUR_N_TUMOR_INFILTRATING_REGULA…																														
CELLMARKERCUR_N_TUMOR_INFILTRATING_T_CELL																														
CELLMARKERCUR_N_TUMOR_INITIATING_CELL																														
CELLMARKERCUR_N_TUMOR_REGULATORY_T_CELL																														

Table S5: Cell state marker gene list

CELLMARKERCAN_ACINAR_CELLS																														
CELLMARKERCUR_N_UMBRELLA_CELL																														
CELLMARKERCUR_N_UNRESTRICTED_SOMATIC_STEM…																														
CELLMARKERCUR_N_UNSWITCHED_PLASMABLAST																														
CELLMARKERCUR_N_URETERIC_BUD_CELL																														
CELLMARKERCUR_N_UROTHELIAL_CELL																														
CELLMARKERCUR_N_UROTHELIUM_CELL																														
CELLMARKERCUR_N_VASCULATURE_CELL																														
CELLMARKERCUR_N_VEIN_CELL																														
CELLMARKERCUR_N_VENOUS_CELL																														
CELLMARKERCUR_N_VENTRAL_LATERAL_GANGLIONI…																														
CELLMARKERCUR_N_VENTRAL_MEDIAL_GANGLIONIC…																														
CELLMARKERCUR_N_VENTRICULAR_CELL																														
CELLMARKERCUR_N_YOUNG_NEURON																														
CELLMARKERCUR_N_ZONA_FASCICULATA_CELL																														
CELLMARKERCUR_N_ZONA_GLOMERULOSA_CELL																														
CELLMARKERCUR_N_ZONA_RETICULARIS_CELL																														
CELLMARKERCUR_N_ZYGOTE																														
CELLMARKERCURNACTIVATEDEFFECTORMEMORYCD4T…																														
CELLMARKERCURNACTIVATEDTISSUERESIDENTMEMO…																														
CELLMARKERCURNACTIVATEDTISSUERESIDENTMEMO…																														
CELLMARKERCURNALPHACELLACELL																														
CELLMARKERCURNALVEOLARTYPEIIATIICELL																														
CELLMARKERCURNATYPICALBCELLABC																														
CELLMARKERCURNAXLCD11CPLASMACYTOIDDENDRIT…																														
CELLMARKERCURNAXLCD123PLASMACYTOIDDENDRIT…																														
CELLMARKERCURNBASALFOREBRAINCHOLINERGICNE…																														
CELLMARKERCURNBRUSHCELLTUFTCELL																														
CELLMARKERCURNCANCERASSOCIATEDFIBROBLASTC…																														
CELLMARKERCURNCAPSULARFIBROBLASTFB2																														
CELLMARKERCURNCCR7DENDRITICCELL																														
CELLMARKERCURNCD106MESENCHYMALSTEMCELL																														
CELLMARKERCURNCD14MACROPHAGE																														
CELLMARKERCURNCD16DENDRITICCELL																														
CELLMARKERCURNCD25LAG3TCELL																														
CELLMARKERCURNCD34GRANULOCYTICPROGENITORC…																														
CELLMARKERCURNCD34HEMATOPOIETICSTEMCELL																														
CELLMARKERCURNCD34PREBCELL																														
CELLMARKERCURNCD34PROGENITORCELL																														
CELLMARKERCURNCD34THYMOCYTE																														
CELLMARKERCURNCD40LGTHELPERCELL																														
CELLMARKERCURNCD4CD25REGULATORYTCELL																														
CELLMARKERCURNCD4CENTRALMEMORYLIKETTCMLIK…																														
CELLMARKERCURNCD4CYTOTOXICT1CELL																														
CELLMARKERCURNCD4CYTOTOXICT2CELL																														
CELLMARKERCURNCD4THELPERCELL																														
CELLMARKERCURNCHRONICALLYACTIVATEDCD4TCELL																														
CELLMARKERCURNCHRONICALLYACTIVATEDCD8TCELL																														
CELLMARKERCURNCLASSSWITCHEDMEMORYBIGGU03B…																														
CELLMARKERCURNCLEC10ADENDRITICCELL																														
CELLMARKERCURNCLEC9ADENDRITICCELL																														
CELLMARKERCURNCONVENTIONALDENDRITICCELL2B…																														
CELLMARKERCURNCONVENTIONALDENDRITICCELL2B…																														
CELLMARKERCURNCORTICALSOMATOSTATINSSTINTE…																														
CELLMARKERCURNCYTOTOXICCD4T2CELL																														
CELLMARKERCURNCYTOTOXICCD4TCELL																														
CELLMARKERCURND1MSNMEDIUMSPINYNEURON																														
CELLMARKERCURND2MSNMEDIUMSPINYNEURON																														
CELLMARKERCURNDISTALTUBULELOOPOFHENLECELL																														
CELLMARKERCURNEARLYCD34MEGAKARYOCYTEERYTH…																														
CELLMARKERCURNEARLYTCELLPROGENITORETP																														
CELLMARKERCURNEFFECTORMEMORYCD4TCELL																														
CELLMARKERCURNEFFECTORREGULATORYTETREGCELL																														
CELLMARKERCURNEFFECTORTTEFFCELL																														
CELLMARKERCURNEXHAUSTEDCD4TCELL																														
CELLMARKERCURNEXHAUSTEDCD8TCELL																														
CELLMARKERCURNEXHAUSTEDCD8TPROGENITORCELL																														
CELLMARKERCURNEXHAUSTEDTTEXCELL																														
CELLMARKERCURNEXTRATELENCEPHALICETCELL																														
CELLMARKERCURNFINALLYHIGHLYEFFECTORTEMRAM…																														
CELLMARKERCURNFOLLICULARREGULATORYTTFRCELL																														
CELLMARKERCURNFOXP3NATURALREGULATORYTTREG…																														
CELLMARKERCURNGAMMADELTAU03B3DTCELL																														
CELLMARKERCURNGERMINALCENTREGCLIKECELL																														
CELLMARKERCURNGLOMERULARENDOTHELIALCELLECG																														
CELLMARKERCURNGRANULOCYTICMYELOIDDERIVEDS…																														
CELLMARKERCURNGRBREGULATORYBCELL																														
CELLMARKERCURNGZMKTCELL																														
CELLMARKERCURNHIGHLYDIFFERENTIATEDEFFECTO…																														
CELLMARKERCURNINDUCEDREGULATORYTTREGCELL																														
CELLMARKERCURNINFLAMMATORYCANCERASSOCIATE…																														
CELLMARKERCURNINNATELYMPHOIDCELLILCS																														
CELLMARKERCURNINTRATHYMICFIBROBLASTFB1																														
CELLMARKERCURNITOCELLHEPATICSTELLATECELL																														
CELLMARKERCURNLYMPHANGIOLEIOMYOMATOSISCEL…																														
CELLMARKERCURNMARGINALZONEMZLIKEBCELL																														
CELLMARKERCURNMEMORYSTEMCELLTSCM																														
CELLMARKERCURNMICROGLIADERIVEDTUMORASSOCI…																														
CELLMARKERCURNMKERYTHCELL																														
CELLMARKERCURNMYOFIBROBLASTICCANCERASSOCI…																														
CELLMARKERCURNNAIVEREGULATORYTTREGCELL																														
CELLMARKERCURNNATURALREGULATORYTTREGCELL																														
CELLMARKERCURNPERIPHERALBLOODMONONUCLEARC…																														
CELLMARKERCURNPITUITARYSTEMCELLPSC																														
CELLMARKERCURNPLASMACYTOIDDENDRITICCELLPDC																														
CELLMARKERCURNPOLYMORPHONUCLEARMYELOIDDER…																														
CELLMARKERCURNPOLYMORPHONUCLEARNEUTROPHIL…																														
CELLMARKERCURNPOSTERIORPRESOMITICMESODERM…																														
CELLMARKERCURNPROLIFERATIVECD4TCELL																														
CELLMARKERCURNPROLIFERATIVECD8TCELL																														
CELLMARKERCURNREDBLOODCELLERYTHROCYTE																														
CELLMARKERCURNREGULATORYBBREGCELL																														
CELLMARKERCURNREGULATORYCD4TCELL																														
CELLMARKERCURNREGULATORYCD8TCELL																														
CELLMARKERCURNREGULATORYTTREGCELL																														

CELLMARKERCAN_ACINAR_CELLS																														
CELLMARKERCURNREGULATORYTTREGCELL																														
CELLMARKERCURNRENALVESICLECOMMASHAPEDBODY…																														
CELLMARKERCURNRESIDENTMEMORYCD8TCELL																														
CELLMARKERCURNREVERTANTMEMORYCD8TTEMRACELL																														
CELLMARKERCURNSCARASSOCIATEDMACROPHAGESAM																														
CELLMARKERCURNSENESCENTCD8TCELL																														
CELLMARKERCURNSTEMPROGENITORCELL																														
CELLMARKERCURNSWITCHEDMEMORYBCELLSWMB																														
CELLMARKERCURNT1TRANSITIONALBCELL																														
CELLMARKERCURNT2TRANSITIONALBCELL																														
CELLMARKERCURNTCELLNATURALKILLERCELL																														
CELLMARKERCURNTERMINALEFFECTORCD8TCELL																														
CELLMARKERCURNTERMINALLYDIFFERENTIATEDCD8…																														
CELLMARKERCURNTFOLLICULARHELPERTFHCELL																														
CELLMARKERCURNTHELPER1TH1CELL																														
CELLMARKERCURNTHELPER1TH1CELL																														
CELLMARKERCURNTHELPER2TH2CELL																														
CELLMARKERCURNTHELPER2TH2CELL																														
CELLMARKERCURNTHELPER9TH9CELL																														
CELLMARKERCURNTHELPERTHCELL																														
CELLMARKERCURNTHYMICEPITHELIALCELLTEC																														
CELLMARKERCURNTISSUERESIDENTMEMORYCD4TCELL																														
CELLMARKERCURNTISSUERESIDENTMEMORYCD8TCELL																														
CELLMARKERCURNTISSUERESIDENTMEMORYTTRMCELL																														
CELLMARKERCURNTPERIPHERALHELPERTPHCELL																														
CELLMARKERCURNTRANSIENTAMPLIFYINGTACELL																														
CELLMARKERCURNTRANSITIONALEXHAUSTEDCD8TCE…																														
CELLMARKERCURNTUMORINFILTRATINGLYMPHOCYTE…																														
CELLMARKERCURNUNSWITCHEDMEMORYBCELLUNSWMB																														
CELLMARKERCURNVASARECTAENDOTHELIALCELLECVR																														
CELLMARKERCURNVONECONOMONEURONVEN																														
CELLMARKERCURNYOLKSACDERIVEDMYELOIDBIASED…																														
CELLMARKEREXP_ACINAR_CELLS																														
CELLMARKEREXP_ADRENERGIC_NEURONS																														
CELLMARKEREXP_AIRWAY_EPITHELIAL_CELLS																														
CELLMARKEREXP_AIRWAY_SMOOTH_MUSCLE_CELLS																														
CELLMARKEREXP_ALPHA_CELLS																														
CELLMARKEREXP_ANTERIOR_PITUITARY_GLAND_CE…																														
CELLMARKEREXP_ASTROCYTES																														
CELLMARKEREXP_B_CELLS																														
CELLMARKEREXP_B_CELLS_MEMORY																														
CELLMARKEREXP_B_CELLS_NAIVE																														
CELLMARKEREXP_BASAL_CELLS																														
CELLMARKEREXP_BASOPHILS																														
CELLMARKEREXP_BERGMANN_GLIA																														
CELLMARKEREXP_BETA_CELLS																														
CELLMARKEREXP_CAJAL_RETZIUS_CELLS																														
CELLMARKEREXP_CHOLANGIOCYTES																														
CELLMARKEREXP_CHOLINERGIC_NEURONS																														
CELLMARKEREXP_CHONDROCYTES																														
CELLMARKEREXP_DELTA_CELLS																														
CELLMARKEREXP_DISTAL_TUBULE_CELLS																														
CELLMARKEREXP_DUCTAL_CELLS																														
CELLMARKEREXP_EMBRYONIC_STEM_CELLS																														
CELLMARKEREXP_ENTERIC_NEURONS																														
CELLMARKEREXP_ENTEROENDOCRINE_CELLS																														
CELLMARKEREXP_EPENDYMAL_CELLS																														
CELLMARKEREXP_EPSILON_CELLS																														
CELLMARKEREXP_ERYTHROID_LIKE_AND_ERYTHROI…																														
CELLMARKEREXP_GAMMA_DELTA_T_CELLS																														
CELLMARKEREXP_GOBLET_CELLS																														
CELLMARKEREXP_HEMANGIOBLASTS																														
CELLMARKEREXP_HEMATOPOIETIC_STEM_CELLS																														
CELLMARKEREXP_HEPATIC_STELLATE_CELLS																														
CELLMARKEREXP_HIS_BUNDLE_CELLS																														
CELLMARKEREXP_IMMATURE_NEURONS																														
CELLMARKEREXP_JUXTAGLOMERULAR_CELLS																														
CELLMARKEREXP_KERATINOCYTES																														
CELLMARKEREXP_KIDNEY_PROGENITOR_CELLS																														
CELLMARKEREXP_KUPFFER_CELLS																														
CELLMARKEREXP_LEYDIG_CELLS																														
CELLMARKEREXP_LOOP_OF_HENLE_CELLS																														
CELLMARKEREXP_LUMINAL_EPITHELIAL_CELLS																														
CELLMARKEREXP_MAMMARY_EPITHELIAL_CELLS																														
CELLMARKEREXP_MEGAKARYOCYTES																														
CELLMARKEREXP_MELANOCYTES																														
CELLMARKEREXP_MESANGIAL_CELLS																														
CELLMARKEREXP_MESOTHELIAL_CELLS																														
CELLMARKEREXP_MICROFOLD_CELLS																														
CELLMARKEREXP_MICROGLIA																														
CELLMARKEREXP_MONOCYTES																														
CELLMARKEREXP_MULLER_CELLS																														
CELLMARKEREXP_MYOBLASTS																														
CELLMARKEREXP_MYOCYTES																														
CELLMARKEREXP_MYOFIBROBLASTS																														
CELLMARKEREXP_NEUROBLASTS																														
CELLMARKEREXP_NEUTROPHILS																														
CELLMARKEREXP_NK_CELLS																														
CELLMARKEREXP_NORADRENERGIC_NEURONS																														
CELLMARKEREXP_OLIGODENDROCYTES																														
CELLMARKEREXP_OSTEOBLASTS																														
CELLMARKEREXP_OSTEOCLAST_PRECURSOR_CELLS																														
CELLMARKEREXP_OSTEOCLASTS																														
CELLMARKEREXP_OSTEOCYTES																														
CELLMARKEREXP_PANETH_CELLS																														
CELLMARKEREXP_PARATHYROID_CHIEF_CELLS																														
CELLMARKEREXP_PERICYTES																														
CELLMARKEREXP_PHOTORECEPTOR_CELLS																														
CELLMARKEREXP_PLASMA_CELLS																														
CELLMARKEREXP_PLASMACYTOID_DENDRITIC_CELLS																														
CELLMARKEREXP_PODOCYTES																														
CELLMARKEREXP_PRINCIPAL_CELLS																														
CELLMARKEREXP_PROXIMAL_TUBULE_CELLS																														
CELLMARKEREXP_PULMONARY_ALVEOLAR_TYPE_I_C…																														

Table S5: Cell state marker gene list

CELLMARKERCAN_ACINAR_CELLS																														
CELLMARKEREXP_PULMONARY_ALVEOLAR_TYPE_II_…																														
CELLMARKEREXP_PURKINJE_FIBER_CELLS																														
CELLMARKEREXP_PURKINJE_NEURONS																														
CELLMARKEREXP_PYRAMIDAL_CELLS																														
CELLMARKEREXP_RETICULOCYTES																														
CELLMARKEREXP_RETINAL_GANGLION_CELLS																														
CELLMARKEREXP_SALIVARY_MUCOUS_CELLS																														
CELLMARKEREXP_SATELLITE_CELLS																														
CELLMARKEREXP_SCHWANN_CELLS																														
CELLMARKEREXP_SEROTONERGIC_NEURONS																														
CELLMARKEREXP_SERTOLI_CELLS																														
CELLMARKEREXP_SMOOTH_MUSCLE_CELLS																														
CELLMARKEREXP_SPERMATOZOA																														
CELLMARKEREXP_STROMAL_CELLS																														
CELLMARKEREXP_T_CELLS																														
CELLMARKEREXP_T_CELLS_NAIVE																														
CELLMARKEREXP_T_CYTOTOXIC_CELLS																														
CELLMARKEREXP_T_HELPER_CELLS																														
CELLMARKEREXP_T_MEMORY_CELLS																														
CELLMARKEREXP_TRICHOCYTES																														
CELLMARKEREXP_TRIGEMINAL_NEURONS																														
CELLMARKEREXP_TROPHOBLAST_CELLS																														
CELLMARKEREXP_TROPHOBLAST_PROGENITOR_CELLS																														
CELLMARKEREXP_TUFT_CELLS																														
CELLMARKEREXP_VASCULAR_SMOOTH_MUSCLE_CELLS																														
CELLMARKEREXPENDOTHELIALCELLSAORTA																														
CELLMARKEREXPNEURALSTEMPRECURSORCELLS																														
CELLMARKERMSIGDB_AIZARANI_LIVER_C12_NK_NK…																														
CELLMARKERMSIGDB_AIZARANI_LIVER_C22_RESID…																														
CELLMARKERMSIGDB_AIZARANI_LIVER_C28_NK_NK…																														
CELLMARKERMSIGDB_AIZARANI_LIVER_C29_MVECS…	PEA15	CDC42…	S1PR1	ERG	INSR	ITM2B	CHSY1	FXYD6	BMX	PLEC	TFPI	PTHLH	NR2F2	MYC	TACC1	SERPI…	LAMB2	TINAG…	SOCS3	SEC14…	WWTR1	PKIG	TEK	PGM5	CYB5R3	NDRG4	DOC2B	SPTAN1	LIMCH1	F2R
CELLMARKERMSIGDB_AIZARANI_LIVER_C30_HEPAT…	TLCD2	C1R	NR1I2	TLCD4	DNAJC…	LINC0…	GPAM	VEGFA	ARIH2…	PCYT1A	SLC35…	GPR82	A2M	SLC17…	EPHX2	PCAT7	CCBE1	LINC0…	COL27…	SNORD…	GGPS1	CFHR3	GRB14	FKBP5	DDX10	C6	ALDH1…	COMMD2	CAMK1D	DGKH
CELLMARKERMSIGDB_AIZARANI_LIVER_C8_RESIDE…																														
CELLMARKERMSIGDB_AIZARANI_LIVER_C9_LSECS_1	ANGPT2	BHLHE…	LGMN	SOX7	CAPZA2	EDN1	NUAK1	TGM2	WWTR1	HES1	FILIP1	ANKS1A	GNG11	TAL1	DENND…	ADAMT…	GALNT1	CD93	VASH1	GBP4	SERPI…	FOSL2	UPP1	ACKR3	PTPN1	ROBO4	ITGA5	EMP1	ZNF160	FAM43A
CELLMARKERMSIGDB_BUSSLINGER_DUODENAL_BCHE…																														
CELLMARKERMSIGDB_BUSSLINGER_DUODENAL_DIFF…	SNHG16	LBR	RPS27A	RPL11	SRSF2	BHLHE…	ATP5F…	GAS5	RCC2	LAD1	SNRPB	NDUFS5	SNRPE	RPS11	EPHB2	KCNE3	SMC4	ADD3	NASP	RPLP1	RPS29	YWHAQ	RPS19	RPL35A	RPL39	SNHG8	BANF1	CLU	NRARP	HNRNPD
CELLMARKERMSIGDB_BUSSLINGER_DUODENAL_EC_C…																														
CELLMARKERMSIGDB_BUSSLINGER_DUODENAL_IMMU…	SUN2	STAT4	TYROBP	LPIN1	YPEL5	LSP1	DOCK8	RFLNB	NR4A2	IL16	JAML	CNN2	MIAT	ADGRE5	TXNIP	CAP1	FLT3LG	DENND…	DDX5	SLA	RAB27A	CCR9	EIF1	NIN	CMTM3	ARHGE…	FXYD5	PSD4	IQGAP1	CD81
CELLMARKERMSIGDB_BUSSLINGER_DUODENAL_STEM…	NR4A1	CPS1	TXNIP	CDC42…	RHOBT…	ZFP36…	ANP32B	EMP2	TCF12	CCL25	RPL22	ADRA2A	RPS28	BCL11A	SESN1	RPL24	BEX3	CDKN1B	ZBTB38	FOSB	MARCKS	SH3BG…	LRIG1	CEMIP2	GOLM1	CRLS1	LRPPRC	NFIA	PRKDC	OLA1
CELLMARKERMSIGDB_BUSSLINGER_DUODENAL_TUFT…																														
CELLMARKERMSIGDB_BUSSLINGER_ESOPHAGEAL_EA…																														
CELLMARKERMSIGDB_BUSSLINGER_ESOPHAGEAL_PR…																														
CELLMARKERMSIGDB_BUSSLINGER_GASTRIC_CHIEF…																														
CELLMARKERMSIGDB_BUSSLINGER_GASTRIC_G_CEL…																														
CELLMARKERMSIGDB_BUSSLINGER_GASTRIC_ISTHM…	PLEK2	LARP1B	PHACT…	ID1	CYP51…	FAM12…	KDELR1	LAMB3	PDIA6	SLC9A1	TACC3	EIF2S2	PERP	UBE2C	ANXA10	KCNE3	CAPN5	ABCC3	LAMA3	TTC39A	EZR	TST	MYO1A	TFCP2…	TOP1	HDGF	SLC44…	TXN	CRELD2	IBTK
CELLMARKERMSIGDB_BUSSLINGER_GASTRIC_NECK_…																														
CELLMARKERMSIGDB_BUSSLINGER_GASTRIC_OXYNT…																														
CELLMARKERMSIGDB_BUSSLINGER_GASTRIC_PREZY…																														
CELLMARKERMSIGDB_BUSSLINGER_GASTRIC_REG3A…																														
CELLMARKERMSIGDB_CUI_DEVELOPING_HEART_5TH…																														
CELLMARKERMSIGDB_CUI_DEVELOPING_HEART_5TH…																														
CELLMARKERMSIGDB_CUI_DEVELOPING_HEART_C1_…																														
CELLMARKERMSIGDB_CUI_DEVELOPING_HEART_C2_…																														
CELLMARKERMSIGDB_CUI_DEVELOPING_HEART_COM…																														
CELLMARKERMSIGDB_CUI_DEVELOPING_HEART_COR…																														
CELLMARKERMSIGDB_CUI_DEVELOPING_HEART_END…																														
CELLMARKERMSIGDB_CUI_DEVELOPING_HEART_LEF…																														
CELLMARKERMSIGDB_CUI_DEVELOPING_HEART_RIG…																														
CELLMARKERMSIGDB_CUI_DEVELOPING_HEART_SMO…																														
CELLMARKERMSIGDB_DESCARTES_FETAL_ADRENAL_…																														
CELLMARKERMSIGDB_DESCARTES_FETAL_CEREBELL…																														
CELLMARKERMSIGDB_DESCARTES_FETAL_CEREBELL…																														
CELLMARKERMSIGDB_DESCARTES_FETAL_CEREBELL…	IL17RA	LRMDA	BIN1	XACT	BHLHE…	AP1B1	NFATC2	HRH1	GNB4	CD4	MANBA	ELL2	SLC9A9	STEAP…	ADAP2	BNC2	RUBCNL	SFMBT2	DAGLB	MAP3K5	DNASE2	B4GAL…	GAB3	PLD1	LINC-…	ANKH	HTRA1	ZNF710	CTSB	ARHGA…
CELLMARKERMSIGDB_DESCARTES_FETAL_CEREBELL…																														
CELLMARKERMSIGDB_DESCARTES_FETAL_CEREBELL…																														
CELLMARKERMSIGDB_DESCARTES_FETAL_CEREBELL…																														
CELLMARKERMSIGDB_DESCARTES_FETAL_CEREBELL…	ID3	PDGFRB	ANGPT2	S1PR3	JCAD	ARAP3	ACE	LXN	EDNRA	WWTR1	MYO1B	PHLDB2	IFI27	PALMD	IFITM2	NPAS2	RASIP1	SLC38…	SLC39…	ENG	CAV2	CAV1	IFI6	THSD1	HSPG2	S100A…	ACTN4	SLC16…	COL4A1	UNC5B
CELLMARKERMSIGDB_DESCARTES_FETAL_CEREBRUM…																														
CELLMARKERMSIGDB_DESCARTES_FETAL_CEREBRUM…																														
CELLMARKERMSIGDB_DESCARTES_FETAL_CEREBRUM…	CTNNA…	SCTR	NUDT16	LANCL3	FAM16…	PDCD6…	TRIM58	CTR9	CD69	BTN3A2	COL6A3	P2RX1	SDE2	SMIM12	MDM1	SNHG6	ZNF770	PKHD1…	SMIM15	TSPAN…	RASSF7	RITA1	RADIL	GP1BA	MAB21…	RUFY1	LEFTY1	RSPH14	IRF2B…	DPYD-…
CELLMARKERMSIGDB_DESCARTES_FETAL_CEREBRUM…	MKNK1	ENTPD1	CD74	RAP1A	CREG1	TBC1D…	ARHGA…	HK2	ITGAL	SP140	SPTLC3	LINC0…	C12or…	GLDN	SELPLG	TMEM1…	CLEC7A	CARD11	LILRB4	TMIGD3	CD68	GPNMB	CYSLT…	FCGR1A	RGS1	LY96	ARRB2	SLC38…	NFKBID	IL1B
CELLMARKERMSIGDB_DESCARTES_FETAL_CEREBRUM…																														
CELLMARKERMSIGDB_DESCARTES_FETAL_CEREBRUM…																														
CELLMARKERMSIGDB_DESCARTES_FETAL_CEREBRUM…	KCNE4	HSPB1	TM4SF1	COL6A2	HYAL2	DUSP6	IFI6	HMCN1	TFRC	ABCA9	ABCC9	CAV2	IGFBP4	SLC38…	THSD1	DCN	BCAM	GRAP	KLF2	ACTN4	KLF10	RASL12	LAMC1	S100A…	PCDH12	COL18…	C1orf…	SLC39…	ROBO4	PROCR
CELLMARKERMSIGDB_DESCARTES_FETAL_EYE_ASTR…																														
CELLMARKERMSIGDB_DESCARTES_FETAL_EYE_GANG…																														
CELLMARKERMSIGDB_DESCARTES_FETAL_EYE_LENS…																														
CELLMARKERMSIGDB_DESCARTES_FETAL_EYE_PHOT…																														
CELLMARKERMSIGDB_DESCARTES_FETAL_EYE_RETI…																														
CELLMARKERMSIGDB_DESCARTES_FETAL_EYE_SMOO…																														
CELLMARKERMSIGDB_DESCARTES_FETAL_EYE_STRO…																														
CELLMARKERMSIGDB_DESCARTES_FETAL_EYE_VASC…																														
CELLMARKERMSIGDB_DESCARTES_FETAL_HEART_EL…																														
CELLMARKERMSIGDB_DESCARTES_FETAL_HEART_EN…																														
CELLMARKERMSIGDB_DESCARTES_FETAL_HEART_LY…																														
CELLMARKERMSIGDB_DESCARTES_FETAL_HEART_SC…																														
CELLMARKERMSIGDB_DESCARTES_FETAL_HEART_SM…																														
CELLMARKERMSIGDB_DESCARTES_FETAL_HEART_ST…																														
CELLMARKERMSIGDB_DESCARTES_FETAL_HEART_VI…																														
CELLMARKERMSIGDB_DESCARTES_FETAL_INTESTIN…																														
CELLMARKERMSIGDB_DESCARTES_FETAL_INTESTIN…																														
CELLMARKERMSIGDB_DESCARTES_FETAL_INTESTIN…																														
CELLMARKERMSIGDB_DESCARTES_FETAL_INTESTIN…																														
CELLMARKERMSIGDB_DESCARTES_FETAL_INTESTIN…																														
CELLMARKERMSIGDB_DESCARTES_FETAL_INTESTIN…																														
CELLMARKERMSIGDB_DESCARTES_FETAL_KIDNEY_M…																														
CELLMARKERMSIGDB_DESCARTES_FETAL_KIDNEY_U…	EPB41…	CGN	B3GNT3	MIR20…	GATA3…	DEGS2	S100A2	TRIM29	SLC9A4	LEAP2	FAM18…	KCNS3	VGLL1	S100A…	CDCP1	SEPTI…	RNF223	FSTL4	PLEKH…	FAM11…	MLPH	MMP7	POF1B	SYT16	PRR35	TTC39A	PVALB	CCNO-…	SSH3	GALNT…
CELLMARKERMSIGDB_DESCARTES_FETAL_LIVER_ER…																														
CELLMARKERMSIGDB_DESCARTES_FETAL_LIVER_HE…																														
CELLMARKERMSIGDB_DESCARTES_FETAL_LIVER_HE…	SLC2A9	PLCXD2	ALDH1…	H19	THRB	SPTLC3	FN1	APOE	GLUD1	MAT1A	PECR	MGST1	DHCR7	CDHR3	PNPLA3	PALMD	PPP2R…	C8A	SERPI…	ALDH6…	CA5A	SERPI…	SLC39…	HNF4G	F12	FBP1	HSD17…	ALDH1…	CYP3A5	ELOVL2
CELLMARKERMSIGDB_DESCARTES_FETAL_LIVER_ME…	SFRP5	PDPN	DDR1	FOXC1	CDH20	TGFB3	EPHB6	CLIC3	CRB2	CFAP54	KNDC1	CTXN1	EPS8L1	STK31	GRID2	SILC1	WT1-AS	ADAMT…	KLF2-…	PARM1	FLNC	CAVIN3	COL8A2	FBN3	LRRN1	KRT7	NKD1	CLIC5	NEK5	MTMR7
CELLMARKERMSIGDB_DESCARTES_FETAL_LUNG_BRO…																														
CELLMARKERMSIGDB_DESCARTES_FETAL_LUNG_CIL…	CABCO…	CCDC17	OSCP1	MARCH…	DNAH2	WDR38	C21or…	LRRC1…	AK8	MORN5	DNAI2	IQCG	ZNF474	C11or…	SPACA9	RSPH9	CDHR4	NEK5	C1orf…	MORN2	CFAP69	FAM81B	HHLA2	ST6GA…	TMEM67	C1orf…	CC2D2A	FAM22…	DNAAF…	MAPK15
CELLMARKERMSIGDB_DESCARTES_FETAL_LUNG_LYM…																														

CELLMARKERCAN_ACINAR_CELLS																														
CELLMARKERMSIGDB_DESCARTES_FETAL_LUNG_NEU…																														
CELLMARKERMSIGDB_DESCARTES_FETAL_LUNG_STR…																														
CELLMARKERMSIGDB_DESCARTES_FETAL_LUNG_VAS…																														
CELLMARKERMSIGDB_DESCARTES_FETAL_MUSCLE_L…																														
CELLMARKERMSIGDB_DESCARTES_FETAL_MUSCLE_S…																														
CELLMARKERMSIGDB_DESCARTES_FETAL_MUSCLE_S…																														
CELLMARKERMSIGDB_DESCARTES_FETAL_MUSCLE_V…																														
CELLMARKERMSIGDB_DESCARTES_FETAL_PANCREAS…																														
CELLMARKERMSIGDB_DESCARTES_FETAL_PANCREAS…																														
CELLMARKERMSIGDB_DESCARTES_FETAL_PANCREAS…																														
CELLMARKERMSIGDB_DESCARTES_FETAL_PANCREAS…																														
CELLMARKERMSIGDB_DESCARTES_FETAL_PANCREAS…																														
CELLMARKERMSIGDB_DESCARTES_FETAL_PANCREAS…																														
CELLMARKERMSIGDB_DESCARTES_FETAL_PANCREAS…																														
CELLMARKERMSIGDB_DESCARTES_FETAL_PLACENTA…																														
CELLMARKERMSIGDB_DESCARTES_FETAL_PLACENTA…																														
CELLMARKERMSIGDB_DESCARTES_FETAL_PLACENTA…																														
CELLMARKERMSIGDB_DESCARTES_FETAL_PLACENTA…																														
CELLMARKERMSIGDB_DESCARTES_FETAL_PLACENTA…																														
CELLMARKERMSIGDB_DESCARTES_FETAL_SPLEEN_E…																														
CELLMARKERMSIGDB_DESCARTES_FETAL_SPLEEN_S…																														
CELLMARKERMSIGDB_DESCARTES_FETAL_SPLEEN_V…																														
CELLMARKERMSIGDB_DESCARTES_FETAL_STOMACH_…	MNS1	CFAP77	STOML3	CFAP61	CFAP1…	CLXN	TMEM67	DRC7	TMEM2…	CROCC2	FAM81B	CFAP2…	CCDC81	ANKMY1	SAXO2	TMEM2…	CLUAP1	STMND1	RSPH9	CFAP74	CFAP2…	NME5	KIF6	CABCO…	PSENEN	LEKR1	SPA17	EFCAB…	CFAP53	DCDC2B
CELLMARKERMSIGDB_DESCARTES_FETAL_STOMACH_…																														
CELLMARKERMSIGDB_DESCARTES_FETAL_STOMACH_…																														
CELLMARKERMSIGDB_DESCARTES_FETAL_STOMACH_…																														
CELLMARKERMSIGDB_DESCARTES_FETAL_STOMACH_…																														
CELLMARKERMSIGDB_DESCARTES_FETAL_STOMACH_…																														
CELLMARKERMSIGDB_DESCARTES_FETAL_STOMACH_…																														
CELLMARKERMSIGDB_DESCARTES_FETAL_STOMACH_…																														
CELLMARKERMSIGDB_DESCARTES_FETAL_STOMACH_…																														
CELLMARKERMSIGDB_DESCARTES_FETAL_STOMACH_…																														
CELLMARKERMSIGDB_DESCARTES_FETAL_STOMACH_…																														
CELLMARKERMSIGDB_DESCARTES_FETAL_STOMACH_…																														
CELLMARKERMSIGDB_DESCARTES_FETAL_THYMUS_T…																														
CELLMARKERMSIGDB_DESCARTES_MAIN_FETAL_ACI…																														
CELLMARKERMSIGDB_DESCARTES_MAIN_FETAL_ADR…																														
CELLMARKERMSIGDB_DESCARTES_MAIN_FETAL_AST…																														
CELLMARKERMSIGDB_DESCARTES_MAIN_FETAL_BIP…																														
CELLMARKERMSIGDB_DESCARTES_MAIN_FETAL_BRO…																														
CELLMARKERMSIGDB_DESCARTES_MAIN_FETAL_CAR…																														
CELLMARKERMSIGDB_DESCARTES_MAIN_FETAL_CIL…	DALRD3	CDHR4	ZMYND…	DNAH1	NEK4	ASB14	DNAH12	RPS8P5	CFAP2…	SYNPR…	SNTN	CDHR1…	IFT57	HHLA2	DZIP3	CFAP44	CFAP4…	CCDC1…	CFAP91	CFAP1…	CFAP92	RPL32…	EFCAB…	IFT122	NEK11	DZIP1L	NME9	ANKUB1	ERICH…	LEKR1
CELLMARKERMSIGDB_DESCARTES_MAIN_FETAL_DUC…																														
CELLMARKERMSIGDB_DESCARTES_MAIN_FETAL_END…																														
CELLMARKERMSIGDB_DESCARTES_MAIN_FETAL_ENS…																														
CELLMARKERMSIGDB_DESCARTES_MAIN_FETAL_EPI…																														
CELLMARKERMSIGDB_DESCARTES_MAIN_FETAL_GAN…																														
CELLMARKERMSIGDB_DESCARTES_MAIN_FETAL_GOB…																														
CELLMARKERMSIGDB_DESCARTES_MAIN_FETAL_HEM…																														
CELLMARKERMSIGDB_DESCARTES_MAIN_FETAL_HOR…																														
CELLMARKERMSIGDB_DESCARTES_MAIN_FETAL_INH…																														
CELLMARKERMSIGDB_DESCARTES_MAIN_FETAL_INH…																														
CELLMARKERMSIGDB_DESCARTES_MAIN_FETAL_INT…																														
CELLMARKERMSIGDB_DESCARTES_MAIN_FETAL_ISL…																														
CELLMARKERMSIGDB_DESCARTES_MAIN_FETAL_LYM…																														
CELLMARKERMSIGDB_DESCARTES_MAIN_FETAL_MES…																														
CELLMARKERMSIGDB_DESCARTES_MAIN_FETAL_MET…																														
CELLMARKERMSIGDB_DESCARTES_MAIN_FETAL_MYE…																														
CELLMARKERMSIGDB_DESCARTES_MAIN_FETAL_NEU…																														
CELLMARKERMSIGDB_DESCARTES_MAIN_FETAL_OLI…																														
CELLMARKERMSIGDB_DESCARTES_MAIN_FETAL_PDE…																														
CELLMARKERMSIGDB_DESCARTES_MAIN_FETAL_PDE…	HSPA8…	CLDN20	USP42	MIOS-…	STK31	SPDYE1	SUN3	TVP23…	LMTK2	RPS3A…	ZSCAN…	SLC12…	ORC5	RINT1	TNPO3	DGKI	ATP6V…	TMEM2…	RAB19	ARHGE…	ZNF212	PTPRN…	ADAM7	ELP3	ADAM2	TERF1	LINC0…	ATP6V…	VIRMA	MED30
CELLMARKERMSIGDB_DESCARTES_MAIN_FETAL_RET…																														
CELLMARKERMSIGDB_DESCARTES_MAIN_FETAL_SAT…																														
CELLMARKERMSIGDB_DESCARTES_MAIN_FETAL_SCH…																														
CELLMARKERMSIGDB_DESCARTES_MAIN_FETAL_SLC…	STAG3…	STAG3…	PMS2P3	SNORA…	TMEM1…	LINC0…	PPP1R…	STAG3…	IRS3P	SLC26…	LSM8	LINC0…	ZBED1…	TNFRS…	HMBOX…	POMK	MRPS28	E2F5	RMDN1	CIBAR1	CCN3	PVT1	RNVU1…	JRK	CYP11…	LY6E-…	SPATC1	LRRC14	SAXO1	FANCG
CELLMARKERMSIGDB_DESCARTES_MAIN_FETAL_SMO…																														
CELLMARKERMSIGDB_DESCARTES_MAIN_FETAL_STE…																														
CELLMARKERMSIGDB_DESCARTES_MAIN_FETAL_SYM…																														
CELLMARKERMSIGDB_DESCARTES_MAIN_FETAL_UNI…																														
CELLMARKERMSIGDB_DESCARTES_MAIN_FETAL_URE…																														
CELLMARKERMSIGDB_DESCARTES_MAIN_FETAL_VAS…																														
CELLMARKERMSIGDB_DESCARTES_MAIN_FETAL_VIS…																														
CELLMARKERMSIGDB_DURANTE_ADULT_OLFACTORY_…																														
CELLMARKERMSIGDB_DURANTE_ADULT_OLFACTORY_…																														
CELLMARKERMSIGDB_DURANTE_ADULT_OLFACTORY_…																														
CELLMARKERMSIGDB_DURANTE_ADULT_OLFACTORY_…																														
CELLMARKERMSIGDB_DURANTE_ADULT_OLFACTORY_…																														
CELLMARKERMSIGDB_DURANTE_ADULT_OLFACTORY_…																														
CELLMARKERMSIGDB_DURANTE_ADULT_OLFACTORY_…																														
CELLMARKERMSIGDB_DURANTE_ADULT_OLFACTORY_…																														
CELLMARKERMSIGDB_DURANTE_ADULT_OLFACTORY_…																														
CELLMARKERMSIGDB_DURANTE_ADULT_OLFACTORY_…																														
CELLMARKERMSIGDB_DURANTE_ADULT_OLFACTORY_…																														
CELLMARKERMSIGDB_DURANTE_ADULT_OLFACTORY_…																														
CELLMARKERMSIGDB_DURANTE_ADULT_OLFACTORY_…																														
CELLMARKERMSIGDB_DURANTE_ADULT_OLFACTORY_…																														
CELLMARKERMSIGDB_DURANTE_ADULT_OLFACTORY_…																														
CELLMARKERMSIGDB_DURANTE_ADULT_OLFACTORY_…																														
CELLMARKERMSIGDB_FAN_EMBRYONIC_CTX_ASTROC…																														
CELLMARKERMSIGDB_FAN_EMBRYONIC_CTX_BIG_GR…	COL18…	PGF	PDE7B	SEMA5A	ID3	TGFB1…	PLOD1	QPCT	MYLK2	ISG15	NKD2	TMEM88	LHFPL6	BHLHE…	S100A…	TPM2	JCAD	EFNA1	CAV2	RELL1	TJP1	CGNL1	ADCY4	COLEC…	EMP2	LAMB1	EVA1B	ACVRL1	TCN2	RASGR…
CELLMARKERMSIGDB_FAN_EMBRYONIC_CTX_BIG_GR…	TUBB4B	TMEM5…	PCSK1	TANK	DIRAS2	FST	ADRA2C	CCDC13	PKM	CBLN4	STMN3	PRDX5	ATP6V…	PPARG…	SPINK5	NALF1	CNTNA…	GCHFR	SCG2	PTPRU	NDUFB3	ZNF503	CACNA…	MAOA	LSAMP	GABRG1	GPR6	THSD7A	DUSP23	EPHA8
CELLMARKERMSIGDB_FAN_EMBRYONIC_CTX_BIG_GR…																														
CELLMARKERMSIGDB_FAN_EMBRYONIC_CTX_BIG_GR…	BTG2	TAL1	RUBCNL	VAMP8	STING1	IRAG2	SAT1	SLC29…	SYK	FYB1	CD36	FOSB	P2RX4	DAB2	LPCAT2	FGD2	OAS1	SUSD3	EVI2B	IL17RA	ZFP36…	ITGAM	GM2A	ITGAX	CD300A	IKZF1	OTULI…	SERPI…	NFKBIZ	DNAJB1
CELLMARKERMSIGDB_FAN_EMBRYONIC_CTX_BRAIN_…	S100A…	CA2	CA4	CD200	GALNT…	DNAJC1	EGFL7	HEG1	DOCK9	LMO2	GIMAP7	MYO1B	BHLHE…	CPD	TEK	ACVRL1	EMP2	JAM2	TTN	WWTR1	CCDC1…	HID1	NID1	IL32	NOSTR…	LRP8	BEX3	S1PR1	PLK2	PTPRB
CELLMARKERMSIGDB_FAN_EMBRYONIC_CTX_BRAIN_…	PDLIM7	NTM	MAP1L…	CTNNB…	CRIP1	TIMP1	TAGLN2	C11or…	SGCE	GUCY1…	EHD2	CYTOR	MAP3K…	NID2	ACTN1	PDZD2	OLFML…	TMEM2…	CAVIN3	FXYD6	GPRC5C	FAM20A	MYLK	ITPR1	PTPRK	TESC	PXDN	SNAI2	EPAS1	FOXF2
CELLMARKERMSIGDB_FAN_EMBRYONIC_CTX_EX_1_E…																														
CELLMARKERMSIGDB_FAN_EMBRYONIC_CTX_IN_2_I…																														
CELLMARKERMSIGDB_FAN_EMBRYONIC_CTX_IN_3_I…																														
CELLMARKERMSIGDB_FAN_EMBRYONIC_CTX_IN_5_I…																														
CELLMARKERMSIGDB_FAN_EMBRYONIC_CTX_IN_6_I…																														
CELLMARKERMSIGDB_FAN_EMBRYONIC_CTX_MICROG…																														
CELLMARKERMSIGDB_FAN_EMBRYONIC_CTX_OLIG	SYNJ2	MAP6D1	GALNT…	CD22	CPD	GJC2	KCNMB4	RAP1A	ANK3	CD47	CDKN1C	PFDN2	SCARB2	MARCK…	RASGR…	LANCL1	PTPDC1	BACE2	PIP4K…	C21or…	GOT1	UBL3	ST3GA…	APOD	ADAMT…	UNC5C	FRMD4B	CNTN2	FIS1	ANLN
CELLMARKERMSIGDB_FAN_EMBRYONIC_CTX_OPC																														

Table S5: Cell state marker gene list

CELLMARKERCAN_ACINAR_CELLS																														
CELLMARKERMSIGDB_FAN_OVARY_CL1_GPRC5A_TNF…	ENO1	PSMG1	CYCS	CHMP4B	PHLDA1	HNRNP…	EIF3A	RPL5	BTG3	SLC1A5	YBX1	SNHG15	HNRNPU	GLA	CCT6A	GTPBP4	CNN3	ARF4	EEF1A1	WDR74	SGCE	MMP2	CSNK1…	CHD1	TOMM40	CAVIN1	ETF1	TUBB4B	UGDH	LTBP4
CELLMARKERMSIGDB_FAN_OVARY_CL11_MURAL_GRA…	THBS1	CKB	CHIC2	ELK1	TXN2	SLC46…	VCAN	RDX	CCN1	PHEX	PTTG1…	MAP1L…	CDK6	CD47	NOP58	RNF19A	IRS2	BCAT1	GMNN	PARP11	PGM1	SMAD3	COL4A1	S100B	SMS	BEX1	AVPI1	RAB31	TAF1D	CMTM8
CELLMARKERMSIGDB_FAN_OVARY_CL13_MONOCYTE_…	CASP1	ANXA1	DOK2	TNFAI…	ARPC3	TSPO	ATP5F…	CMTM6	LGALS2	ARL8B	REL	FCGR2B	SERF2	CYTIP	PTPRE	IER3	CSTA	HAVCR2	LPXN	IL4I1	PPT1	LY96	LITAF	RNF130	CEBPB	HLA-C	B2M	ISG20	RGS2	ACTB
CELLMARKERMSIGDB_FAN_OVARY_CL14_MATURE_SM…	HSPB1	ROCK1	ZFP36…	FLNA	MFAP4	PDLIM1	ARPC5	RSU1	NUDT4	COX7A2	MGP	CHCHD…	IFITM2	IER3	TSC22…	CHURC1	NENF	IFITM1	EPAS1	UBA2	ITGB1	BTG2	EID1	ESD	NME3	MT-ND5	NDUFB7	C4orf3	C12or…	BRD2
CELLMARKERMSIGDB_FAN_OVARY_CL15_SMALL_ANT…	EIF4A1	HSPA8	GADD4…	BANF1	HSPD1	MATCA…	IP6K2	MTRF1L	DEDD2	RIMKLB	ACTB	RBM8A	AKIRI…	GJA1	H2AZ1	PTP4A1	ARF5	TXN2	SLC25…	U2AF1	BEX3	P3H2	TRA2A	DNAJB…	ACSM3	TXNDC…	SRSF3	SNRPG	SUB1	IQCG
CELLMARKERMSIGDB_FAN_OVARY_CL18_B_LYMPHOC…	AKAP13	TNFRS…	LGMN	RAP1A	CMTM6	UGCG	HLA-D…	PFN1	FMNL1	DDX24	CNPY3	MAD1L1	CALR	FERMT3	CDC37	SH2B3	M6PR	HSPH1	ARFGA…	TNIP1	B2M	PTPN1	CCDC50	HSPA8	TCF4	NFKB1	NPC2	RELB	FTL	HCST
CELLMARKERMSIGDB_FAN_OVARY_CL2_PUTATIVE_E…																														
CELLMARKERMSIGDB_FAN_OVARY_CL5_HEALTHY_SE…	PPP3CA	RBM22	EFEMP2	HMGN2	KPNA2	MAGED1	LIMA1	TCP1	C1orf…	HES1	MORN2	H2AC6	DLC1	PLTP	PDGFRA	GBP4	GBP1	GLT8D2	PTMS	PTGER2	AKAP1…	ZNF106	GCSH	NRP1	IP6K2	RRBP1	JUN	TXNL1	P4HB	C4BPB
CELLMARKERMSIGDB_FAN_OVARY_CL6_PUTATIVE_E…	PPP1R…	PEBP1	BTG2	IGFBP4	SPARC…	FCGRT	HNMT	TCEAL3	TSPAN4	BNIP3L	TENT5A	GLUL	VIM	KCNQ1…	CYBRD1	NR2F2	SCD5	KLF4	RCN1	BST2	SOCS3	MATN2	ATP6A…	NFIC	APP	SH3BP5	ARL4D	COL18…	CDH11	RWDD4
CELLMARKERMSIGDB_FAN_OVARY_CL8_MATURE_CUM…	ATP5MJ	TMSB4X	NME4	IRF4	MT-CO2	NDUFS8	BANF1	BAMBI	MFAP2	HIF1A	CAMTA1	S100A…	GRB14	MEX3B	CHCHD…	HOPX	ARF5	ST3GA…	UQCC2	MYO1B	TMEM2…	SIGLE…	NME2	COX7C	PCBP2	MRPL23	CRHBP	PPARG	HS3ST1	COX8A
CELLMARKERMSIGDB_FAN_OVARY_CL9_PUTATIVE_A…	NDRG1	MAST4	MT2A	NOP10	TAGLN2	FXYD5	CAPZA2	MSN	CRIP2	LIFR	HLA-D…	HLA-A	CYTOR	TXN	IRF1	NANS	S100A…	FKBP1A	MAP1L…	HNRNPF	GSN	GNG12	DNAJB4	STING1	CCDC8…	ATP1A1	CDC37	HLA-D…	LUZP1	IFNGR1
CELLMARKERMSIGDB_GAO_ESOPHAGUS_25W_C1_CIL…	DCDC2	DCP1B	DGCR6	DHRS9	DLEC1	DMPK	DNAAF1	DNAH1	DNAH10	DNAH11	DNAH12	DNAH5	DNAH6	DNAH7	DNAH9	DNAI1	DNAI2	DNAL1	DPCD	DPY19…	DPY19…	DPY19…	DRC1	DTHD1	DTNA	DYDC2	DYNC2…	DNAAF4	DZIP1L	DZIP3
CELLMARKERMSIGDB_GAO_ESOPHAGUS_25W_C2_KRT…																														
CELLMARKERMSIGDB_GAO_ESOPHAGUS_25W_C3_FGF…																														
CELLMARKERMSIGDB_GAO_LARGE_INTESTINE_24W_…																														
CELLMARKERMSIGDB_GAO_LARGE_INTESTINE_24W_…																														
CELLMARKERMSIGDB_GAO_LARGE_INTESTINE_24W_…	HLA-D…	HLA-D…	HLA-D…	HLA-D…	HLA-D…	HLA-D…	HLA-D…	HMOX1	HPGDS	HSPA6	ICAM1	ICAM4	IFI30	IFNGR1	IGSF6	IKZF1	IL10	IL1A	IL1B	IL1R2	IL1RN	IL23A	IL4I1	INHBA	INPP5D	IPMK	IRF8	IRS2	ITGAM	ITGAX
CELLMARKERMSIGDB_GAO_LARGE_INTESTINE_24W_…																														
CELLMARKERMSIGDB_GAO_LARGE_INTESTINE_24W_…																														
CELLMARKERMSIGDB_GAO_LARGE_INTESTINE_24W_…																														
CELLMARKERMSIGDB_GAO_LARGE_INTESTINE_ADUL…	MATCA…	KIF1A	KIF26A	KIF5C	KL	KLRC2	KRT40	LARP6	LCN15	SERTA…	LINC0…	LINC0…	LINC0…	LMX1A	RANBP…	LINC0…	LINC0…	PENK-…	LINC0…	ISL1-…	ENSG0…	LRRC36	MAFA	MAMSTR	MAP1B	MAP2	MAP6	MAP7D2	MAP9	MAPK8…
CELLMARKERMSIGDB_GAO_LARGE_INTESTINE_ADUL…	HCN3	HEG1	HIVEP1	HLA-D…	HLA-D…	HMCN2	HNRNP…	HOXD4	HSD17…	HSPA4L	HTR4	IFIT2	IFIT3	IKZF4	IL10R…	IL23A	IL4I1	INTS2	IRAK2	KCNA5	KCNA7	KCNH8	KCNIP4	KCNQ4	BTBD8	KIF14	KIF21B	KLF7	KLHL25	KLHL32
CELLMARKERMSIGDB_GAO_LARGE_INTESTINE_ADUL…																														
CELLMARKERMSIGDB_GAO_LARGE_INTESTINE_ADUL…	GLCCI1	GLDC	GLIPR1	GMFG	GNG2	GNG7	GOLGA…	GPLD1	GPR15	GPR183	GPR65	GPSM3	GRK5	GSTM2	GYPC	HCLS1	HCST	HERC5	HHEX	HLA-D…	HLA-D…	HOMER3	HVCN1	ICAM1	ICAM4	IFFO2	IFNAR2	IGF1	IGFLR1	IGLL5
CELLMARKERMSIGDB_GAO_LARGE_INTESTINE_ADUL…	DYNC2…	DZIP1	ECM1	ECSCR	EDNRA	EDNRB	EFEMP1	EFEMP2	EGFL7	EGFLAM	EHD2	ELMO1	EMCN	EMID1	EMILI…	ENG	ENHO	ENPP2	ESAM	ETS1	ETV1	EVA1B	F10	F2RL3	FABP4	FADS1	RFLNB	FAM10…	FAM13C	ALKAL2
CELLMARKERMSIGDB_GAO_SMALL_INTESTINE_24W_…																														
CELLMARKERMSIGDB_GAO_SMALL_INTESTINE_24W_…																														
CELLMARKERMSIGDB_GAO_SMALL_INTESTINE_24W_…																														
CELLMARKERMSIGDB_GAO_SMALL_INTESTINE_24W_…																														
CELLMARKERMSIGDB_GAO_SMALL_INTESTINE_24W_…																														
CELLMARKERMSIGDB_GAO_STOMACH_24W_C1_PROCR…																														
CELLMARKERMSIGDB_GAO_STOMACH_24W_C2_TFF2P…																														
CELLMARKERMSIGDB_GAO_STOMACH_24W_C4_PARIE…																														
CELLMARKERMSIGDB_GAO_STOMACH_24W_C5_PUTAT…																														
CELLMARKERMSIGDB_GAUTAM_EYE_IRIS_CILIARY_…	CDKN1B	ARAP3	PSMD2	RPS6K…	RAB32	CCT5	TASOR	ATG3	PEX5	RPL36	COA4	TSPAN…	AGPS	ANGEL1	CROCC…	ARHGE…	ECH1	SRA1	DTX3L	ZFP14	RALGA…	ARHGA…	AP2A2	PMAIP1	CTCF	CMC1	MRPS1…	C17or…	CDC42…	ELK4
CELLMARKERMSIGDB_GAUTAM_EYE_IRIS_CILIARY_…																														
CELLMARKERMSIGDB_GAUTAM_EYE_IRIS_CILIARY_…																														
CELLMARKERMSIGDB_GAUTAM_EYE_IRIS_CILIARY_…																														
CELLMARKERMSIGDB_GAUTAM_EYE_IRIS_CILIARY_…																														
CELLMARKERMSIGDB_HAY_BONE_MARROW_CD34_POS…																														
CELLMARKERMSIGDB_HAY_BONE_MARROW_CD34_POS…																														
CELLMARKERMSIGDB_HAY_BONE_MARROW_CD34_POS…																														
CELLMARKERMSIGDB_HAY_BONE_MARROW_CD34_POS…																														
CELLMARKERMSIGDB_HAY_BONE_MARROW_CD8_T_CE…																														
CELLMARKERMSIGDB_HAY_BONE_MARROW_EARLY_ER…																														
CELLMARKERMSIGDB_HAY_BONE_MARROW_EOSINOPH…																														
CELLMARKERMSIGDB_HAY_BONE_MARROW_NAIVE_T_…	ISG20	EEF1D	SEPTI…	CD69	CORO1B	AAK1	LDLRA…	RPL36A	CHRM3…	STK4	CD40LG	SNHG32	RNF125	FAU	RPL12	CMPK1	RPL35	LINC0…	PIK3R1	INPP4B	TCEA3	PBXIP1	RPL29	EIF4A2	FOXP1	ABLIM1	OCIAD2	RPS23	EML4	LINC0…
CELLMARKERMSIGDB_HAY_BONE_MARROW_NEUTROPH…	SULT1…	THBS1	TALDO1	NDUFB1	C1orf…	ADA2	RTN3	FBXL5	MT-ND3	CXCL8	ATP5M…	SIRPB1	QKI	CARD16	CRISP…	CD163	VNN2	CNPY3	CATSP…	CREB5	IRAK3	ENTPD1	NAIP	CAPNS1	STAB1	CARS2	OGFRL1	CHP1	PKM	DMXL2
CELLMARKERMSIGDB_HAY_BONE_MARROW_NK_CELLS	MYOM2	RNF165	PSME1	NCALD	CORO1A	TSPAN…	AKNA	FEZ1	YPEL1	PSMB9	TNFRS…	CDC42…	UBE2F	CDK2A…	LINC0…	MAFF	RNF213	CTSC	MYBL1	RASSF1	NDUFB2	ITGB7	DTHD1	PDIA3	PILRB	FASLG	MCTP2	BOK	LLGL2	PLAAT3
CELLMARKERMSIGDB_HAY_BONE_MARROW_PRE_DEND…																														
CELLMARKERMSIGDB_HAY_BONE_MARROW_PRO_B	DLEU2	RBBP7	NONO	GLO1	GGH	MYL6B	KHDRB…	SMARC…	HNRNPR	DDAH2	CBX5	SHCBP1	MYO3A	NRM	TMEM1…	MARCK…	RPL39L	SRSF10	APOLD1	BARD1	KPNA2	CCNI	HMCES	HAUS1	ANP32A	SKA2	RALY	H1-5	H2AC12	LIG1
CELLMARKERMSIGDB_HAY_BONE_MARROW_STROMAL	LBP	TWIST2	PDE1A	CRYAB	IGFBP4	COL28…	MXRA5	LINC0…	EMILI…	MT1M	CCN1	SIX1	TRABD…	TUSC3	GPM6B	CAVIN1	RASD1	ADGRL2	PDGFRL	WASF3	EMP2	APOD	IGFBP3	IL34	TNS2	FMOD	CAV2	PRELP	THY1	EMP1
CELLMARKERMSIGDB_HE_LIM_SUN_FETAL_LUNG_C0…	CYP39…	GLIPR1	PRKACB	SLC8A1	ZEB1	STK38L	BTN2A1	THRB	PVALB	CDC34	AAK1	ZFHX4	MDFIC	ANKS1A	VASP	PPP1R…	ACOT1	FSD1	HMGA1	KPNA3	PAWR	KLHL42	P2RY1	IGSF3	FREM2	SYTL2	OCIAD2	SYNC	NTM	HNRNP…
CELLMARKERMSIGDB_HE_LIM_SUN_FETAL_LUNG_C0…																														
CELLMARKERMSIGDB_HE_LIM_SUN_FETAL_LUNG_C0…																														
CELLMARKERMSIGDB_HE_LIM_SUN_FETAL_LUNG_C0…	TMUB1	HMCES	WWC2	RNF8	FZD4	SH3RF1	DDR1	PIM3	CDON	SLC7A7	ARHGA…	GATA6…	SLC25…	ECHDC2	TSEN15	LRATD2	PTPRF	PPP1R…	TPD52…	IQCA1	AATF	CRLS1	METTL…	COL11…	NPW	PRKCI	NMU	CADM2	CD24	UNC5B…
CELLMARKERMSIGDB_HE_LIM_SUN_FETAL_LUNG_C0…																														
CELLMARKERMSIGDB_HE_LIM_SUN_FETAL_LUNG_C0…																														
CELLMARKERMSIGDB_HE_LIM_SUN_FETAL_LUNG_C0…	ITGA3	PDZRN4	GCHFR	HAS1	OBSL1	SYNGR2	PROS1	PDLIM4	FNDC1	NINJ1	C1QTN…	MCUB	BEX2	CRIM1	CTSH	GALNT9	HSPB8	SMOC2	ENTPD2	TMTC2	PODN	RAB11…	FLRT2	PSMB8	CDK2A…	TOB1	PDXK	CD47	LINC0…	LSR
CELLMARKERMSIGDB_HE_LIM_SUN_FETAL_LUNG_C0…	GRIK2	GTF2I…	C12or…	EPB41	EMC9	CCNB1	EFNB2	CCNB2	NETO2	CNTNA…	KIF23	CDCA5	CCDC7…	KIF22	GPR161	ANPEP	SPIN2A	CDC20	DSN1	INPP5F	DEPDC…	TRIP13	PBK	UHRF1	JAZF1	CENPE	HMGN5	PCDH17	ISL2	GTSE1
CELLMARKERMSIGDB_HE_LIM_SUN_FETAL_LUNG_C0…																														
CELLMARKERMSIGDB_HE_LIM_SUN_FETAL_LUNG_C0…	TMCO3	CD47	RPRD1B	SLC16…	TNXB	PROS1	PROCR	CADM2	PTAFR	NPW	TMEM37	CACNB3	SDC4	PDLIM4	HVCN1	BAIAP2	CA11	SMPD3	PDZRN4	AGRN	ANXA8	ARPC1B	VAMP8	PERP	CAV2	CCN4	FLNC	TGFB3	ITM2A	HACD1
CELLMARKERMSIGDB_HE_LIM_SUN_FETAL_LUNG_C0…																														
CELLMARKERMSIGDB_HE_LIM_SUN_FETAL_LUNG_C1…																														
CELLMARKERMSIGDB_HE_LIM_SUN_FETAL_LUNG_C1…																														
CELLMARKERMSIGDB_HE_LIM_SUN_FETAL_LUNG_C1…																														
CELLMARKERMSIGDB_HE_LIM_SUN_FETAL_LUNG_C1…	CENPE	PBK	HIC2	RACGA…	SLC29…	KIF4A	ATAD2	RTKN2	PLK1	SFR1	C6orf…	PIM1	FANCI	HSD17…	NCAPD2	CENPA	BYSL	MYBL2	MSI1	PRIM2	PRTG	GINS4	TRMT6	RNF14…	ADM	CDCA2	WASF1	TOPBP1	CBX2	ZWILCH
CELLMARKERMSIGDB_HE_LIM_SUN_FETAL_LUNG_C1…	DNAJC5	AMPH	CLIP3	TOM1L2	MSI1	MAPK8…	GDAP1	HIC2	CACNA…	FSTL5	PRDM1…	PKD1	TMEM65	SYNJ2…	CTXN1	MAPRE3	GCH1	THSD4	APPAT	LY6H	FAM13…	SYNJ2	TRNP1	TBL1X	CASZ1	MFSD6	PRXL2C	TCEAL7	RFX2	ASCL2
CELLMARKERMSIGDB_HE_LIM_SUN_FETAL_LUNG_C1…																														
CELLMARKERMSIGDB_HE_LIM_SUN_FETAL_LUNG_C1…																														
CELLMARKERMSIGDB_HE_LIM_SUN_FETAL_LUNG_C1…																														
CELLMARKERMSIGDB_HE_LIM_SUN_FETAL_LUNG_C1…	PRRG4	FAM18…	CYTL1	EFHD1	MCTP2	EMP1	CYP2J2	BCL6	FKBP11	LYPD3	COL6A2	S100A2	SCNN1A	ARHGA…	GRHL3	ZNF750	SMIM31	CSTA	TGM2	CYBRD1	DNAH5	GALNT…	LRIG1	TMPRS…	PAPSS2	TNFAI…	ENTPD5	CX3CL1	B4GAL…	EBF4
CELLMARKERMSIGDB_HE_LIM_SUN_FETAL_LUNG_C1…	MCF2L	NDUFA…	RETRE…	AMIGO2	MGAT4C	NOL4	ANKH	MFSD6	CADM2	MAP2	USP20	AKT3	KCNH2	ESPN	MAP6	PLCB4	RGS4	ASNS	DGKZ	JAKMI…	SH3BP4	PDZRN3	PCSK2	CASZ1	GDAP1	CLIP3	CDH2	PLPPR2	DRAIC	A4GALT
CELLMARKERMSIGDB_HE_LIM_SUN_FETAL_LUNG_C1…	MEIS1	CFH	STK38L	HTRA1	FZD7	MARCH…	TIAM1	FABP7	PGAP1	SAA1	LAMA4	CCL20	SRGAP3	CFLAR	ELAPO…	ITGB4	S100B	IFI16	CNFN	MAMDC2	SNAI2	CDKAL1	BDH1	EVA1C	ANGPT2	TNFSF…	PDE4B	CD200	C17or…	ST6GA…
CELLMARKERMSIGDB_HE_LIM_SUN_FETAL_LUNG_C1…	DCUN1…	SFRP1	LINC0…	FCGBP	BARX2	PRR4	DEFB1	WNT5B	C9orf…	BHLHE…	MGAT2	SLIT3	C14or…	HEY1	SULF2	EYA2	LENG9	LCN2	PKP1	MLPH	RAB3D	FOSL1	SH3RF1	STEAP1	GCLM	NMRK1	AACS	SLC66…	A1BG	UTP20
CELLMARKERMSIGDB_HE_LIM_SUN_FETAL_LUNG_C2…	HLA-F	LXN	SLC22…	TMEM2…	JKAMP	GIMAP6	ADORA3	GRK3	PCK2	HPSE	CLIC2	LINC0…	MFAP1	DAPK1	DERL1	CPNE2	CCDC1…	ACBD5	LHFPL2	ABCG1	SIKE1	FECH	ARHGA…	SGPL1	NAGLU	IL10	PLVAP	DTX3L	CD40	CREB3
CELLMARKERMSIGDB_HE_LIM_SUN_FETAL_LUNG_C2…	CLIC2	NIBAN1	SPINT1	ZNF296	NUBPL	IGFBP7	PIK3CB	REEP3	PLEKH…	GPR160	RHEX	MAP2K6	ALOX15	FARS2	ERAP2	HLA-F	CYSTM1	NAV1	TAP2	CLEC2B	ST14	TRERF1	SEPTI…	NEGR1	GRAMD…	EPB41	FAM13…	FBXO27	HACD2	RUSC1
CELLMARKERMSIGDB_HE_LIM_SUN_FETAL_LUNG_C2…																														
CELLMARKERMSIGDB_HE_LIM_SUN_FETAL_LUNG_C2…																														
CELLMARKERMSIGDB_HE_LIM_SUN_FETAL_LUNG_C2…																														
CELLMARKERMSIGDB_HE_LIM_SUN_FETAL_LUNG_C2…	PMS2	IGHD	PLAAT4	BDH2	MSRB3	RHOBT…	SEPTI…	STAP1	NUP107	LPIN1	KRI1	ZSCAN…	TEC	CD109	BCL2	GATA2	MAST4	ANKRD…	BET1	MAGED1	CUTC	PTPN7	TMEM1…	QTRT1	GABPB…	NKG7	PDE7A	ABCD4	CAVIN2	SUPV3…
CELLMARKERMSIGDB_HE_LIM_SUN_FETAL_LUNG_C2…	LRBA	SLC44…	EGLN3	MARCH…	CLCN3	GAS2L1	GATA2	PDE3A	HIPK2	ECE1	COL24…	MTURN	CD151	ANK1	FADS2	RGS3	ANXA3	CDIP1	DBN1	DGKG	ACRBP	TC2N	TBXA2R	MFAP3L	C19or…	SMTN	HNRNP…	EPOR	ARMC8	TBPL1
CELLMARKERMSIGDB_HE_LIM_SUN_FETAL_LUNG_C2…																														
CELLMARKERMSIGDB_HE_LIM_SUN_FETAL_LUNG_C2…	ANXA3	TENT5C	ZNF175	MSI2	H2BC18	NEXN	ICAM2	DEPP1	PTGS1	PTK2	MACIR	TFPI	P2RX1	AEN	MFSD1…	MARCH…	MITF	FAXDC2	LXN	NFATC1	UIMC1	CLCN7	CD226	CD58	STAM	RAB3IP	MAP4K5	PITPNB	MGME1	AGO3
CELLMARKERMSIGDB_HE_LIM_SUN_FETAL_LUNG_C2…	RCN2	USP11	KEAP1	GAPT	CPSF3	DUS3L	C19or…	NIPA2	ST3GA…	CDT1	MDFIC	AP3M1	PAICS	MAGED1	TSEN54	CCNB1…	GEMIN2	DKC1	CHAF1A	CDCA7	MNAT1	CD2	ELP6	DTX2	DHTKD1	NIBAN3	ACAA2	CTSW	MCOLN2	MCM6
CELLMARKERMSIGDB_HE_LIM_SUN_FETAL_LUNG_C2…																														
CELLMARKERMSIGDB_HE_LIM_SUN_FETAL_LUNG_C2…																														
CELLMARKERMSIGDB_HE_LIM_SUN_FETAL_LUNG_C2…																														
CELLMARKERMSIGDB_HE_LIM_SUN_FETAL_LUNG_C3…																														
CELLMARKERMSIGDB_HE_LIM_SUN_FETAL_LUNG_C3…	RNF123	TSPO2	TROAP	CENPW	PIGQ	CENPM	GTSE1	SSX2IP	GMNN	SNX22	RMDN3	KIF23	NCAPH	CKS1B	FAM11…	H3C2	CLSPN	LIG1	ABHD5	KIF2C	CA8	IKZF1	ACHE	CDT1	LPCAT3	FANCI	ACKR1	EZH2	TK1	ELL2
CELLMARKERMSIGDB_HE_LIM_SUN_FETAL_LUNG_C3…	SNHG32	EXOSC5	RNF2	DUSP5	SNRNP…	FH	AKIP1	TK1	NOL4L	NIPSN…	RARG	RPUSD3	CLDN7	NEURL…	USP5	IFITM1	POLR3D	COL15…	FASN	PPP1R…	RHOBT…	NDUFA…	CCDC86	ACOT9	ANAPC…	DOK4	PTGES2	C8orf…	ALG3	JMJD6
CELLMARKERMSIGDB_HE_LIM_SUN_FETAL_LUNG_C3…	IFT57	LRRN4…	SMYD2	HSPA2	IL7	SDC3	ACKR2	FILIP1	FBN1	HSD3B7	CEP85L	PELI2	MVP	ITGAV	MET	NNMT	PDGFA	DDX10	PPP1R…	EFEMP1	SGK3	ITGA9	SLC24…	NMNAT2	ELK4	SLC38…	PDGFC	AK4	C1QTN…	THEMI…
CELLMARKERMSIGDB_HE_LIM_SUN_FETAL_LUNG_C3…	THEMI…	LRRN4…	MAGI1	STAB1	NBL1	KCTD17	UNC5B	EPS15	SYNM	HOXD9	FHL3	CYP46…	UGP2	COLEC…	SNX10	TSPAN…	CMKLR2	OLFML1	CARD19	WDFY2	SLC24…	BMP2K	TMEM1…	MICAL2	OLFML…	LRRC70	KLHL4	KLHL3	SNCG	SH3TC1
CELLMARKERMSIGDB_HE_LIM_SUN_FETAL_LUNG_C3…																														
CELLMARKERMSIGDB_HE_LIM_SUN_FETAL_LUNG_C3…	SMAD7	SEZ6L2	GOLIM4	GUCY1…	MAP3K8	ERAP2	CDC42…	GBP1	SELP	IER3	CYBRD1	RGMB	SOCS2	CTSC	CLIC3	PGRMC2	GCA	TRAM2	ALCAM	CBLN2	SEMA3G	SYT11	GALNT2	ENTPD2	NUAK1	PTGS1	EFNA5	SOX5	KISS1	SSTR1
CELLMARKERMSIGDB_HE_LIM_SUN_FETAL_LUNG_C3…	TNFRS…	TMEM1…	TM7SF2	BCR	HVCN1	PBXIP1	EBF1	PCCA	KCNN3	UBE2E2	EDNRB	VPS39	DTX1	HS3ST1	NFATC2	CABIN1	NBAS	ICE2	SLC5A3	HSDL2	FLRT2	OAF	LRRN4…	IFT57	LETR1	C1orf…	ZNF38…	CLIC2	SDC3	GLS
CELLMARKERMSIGDB_HE_LIM_SUN_FETAL_LUNG_C3…																														
CELLMARKERMSIGDB_HE_LIM_SUN_FETAL_LUNG_C4…																														
CELLMARKERMSIGDB_HE_LIM_SUN_FETAL_LUNG_C4…																														
CELLMARKERMSIGDB_HE_LIM_SUN_FETAL_LUNG_C4…																														
CELLMARKERMSIGDB_HE_LIM_SUN_FETAL_LUNG_C4…																														
CELLMARKERMSIGDB_HE_LIM_SUN_FETAL_LUNG_C4…	FABP5	H2AC12	SUZ12	YEATS4	EXOSC8	CDC45	SGO1	KIF15	NCAPD2	CDKN2C	TFDP1	H2AC17	RBBP8	BARD1	DPM2	CDCA7	DSCC1	SKA3	HCFC1…	CKAP2	ESCO2	GMPS	MCM6	MTHFD2	CENPE	NCAPG2	POLD1	NSD2	PXMP2	DIAPH3
CELLMARKERMSIGDB_HE_LIM_SUN_FETAL_LUNG_C4…	MTRFR	H2AC16	KIF23	KIF14	ARHGA…	CBX5	DDX11	NCAPH	GMNN	NCAPG2	GGH	MCM10	RNASE…	FANCD2	NCAPD3	VRK1	TPRKB	ARMH1	ANAPC…	TROAP	RHNO1	CDC45	TCF19	HAT1	ANLN	PBK	XPO1	WDR76	HAUS1	KIF20A
CELLMARKERMSIGDB_HE_LIM_SUN_FETAL_LUNG_C4…																														

Table S5: Cell state marker gene list

CELLMARKERCAN_ACINAR_CELLS																														
CELLMARKERMSIGDB_HE_LIM_SUN_FETAL_LUNG_C4…																														
CELLMARKERMSIGDB_HE_LIM_SUN_FETAL_LUNG_C4…																														
CELLMARKERMSIGDB_HE_LIM_SUN_FETAL_LUNG_C4…																														
CELLMARKERMSIGDB_HE_LIM_SUN_FETAL_LUNG_C5…																														
CELLMARKERMSIGDB_HE_LIM_SUN_FETAL_LUNG_C5…	IFNGR1	PMPCA	CBFA2…	LINC0…	RAB29	C4orf…	ANXA7	CMTM3	DENND…	AKNA	METTL8	GCA	POLR1C	MRPL15	NOLC1	RPA1	S100A…	PARN	MRPL24	TFRC	MTFP1	PTGES2	SEC11C	DEGS1	EHD4	MRPL1	ENOSF1	ABCE1	BID	QTRT1
CELLMARKERMSIGDB_HE_LIM_SUN_FETAL_LUNG_C5…																														
CELLMARKERMSIGDB_HE_LIM_SUN_FETAL_LUNG_C5…																														
CELLMARKERMSIGDB_HE_LIM_SUN_FETAL_LUNG_C5…	FANCI	CDK2	HYI	BRD7	HJURP	H2AC14	TNFRS…	CTNNA…	ACYP1	MXD3	GTSE1	NUF2	NRIP1	RFC2	H2AC11	AARS1	TCF19	SHMT1	ESCO2	CENPH	LINC0…	HIRIP3	CDCA8	CDCA2	H2AC16	CDKN3	RBBP8	CDC45	NAE1	H2BC11
CELLMARKERMSIGDB_HE_LIM_SUN_FETAL_LUNG_C6…																														
CELLMARKERMSIGDB_HE_LIM_SUN_FETAL_LUNG_C7…																														
CELLMARKERMSIGDB_HE_LIM_SUN_FETAL_LUNG_C7…																														
CELLMARKERMSIGDB_HE_LIM_SUN_FETAL_LUNG_C7…																														
CELLMARKERMSIGDB_HE_LIM_SUN_FETAL_LUNG_C7…	GNB5	STK32C	HRK	CNR1	FRY	OSTM1	ST8SI…	CNIH2	EML5	DPP6	SNAP91	TTC39C	CALY	SMIM18	ANK3	LINC0…	SNX10	CDK5R2	KCNMA1	HSD11…	ELMO1	ABCB1	MEIS3	MAPK8…	REEP1	TMEM2…	PITRM1	B3GNT4	SVOP	CPLX1
CELLMARKERMSIGDB_HE_LIM_SUN_FETAL_LUNG_C7…																														
CELLMARKERMSIGDB_HU_FETAL_RETINA_AMACRINE																														
CELLMARKERMSIGDB_HU_FETAL_RETINA_BIPOLAR																														
CELLMARKERMSIGDB_HU_FETAL_RETINA_FIBROBLA…	LAMC1	IGFBP6	MFAP4	EGFL7	S1PR3	TMEM1…	MEF2C	FGFR2	COL21…	FOS	HIF3A	JUNB	FLRT3	TMEM2…	IGFBP5	TPM2	MCAM	IGFBP7	FBN1	SCARA5	SERTA…	MXRA8	IER2	PPP1R…	HSPA5	FOXP1	JAG1	CALU	PTN	SOCS3
CELLMARKERMSIGDB_HU_FETAL_RETINA_HORIZONT…																														
CELLMARKERMSIGDB_HU_FETAL_RETINA_MICROGLIA	PCED1…	RGS2	RNA5S…	KLF2	GIMAP4	LSP1	RB1	FGL2	ITGAX	MERTK	BLNK	MNDA	CREG1	LAIR1	STING1	CDKN1A	B3GNT5	CLEC7A	TXNIP	SORL1	CYBA	CTSC	RPS6K…	ENTPD1	RHBDF2	LINC0…	TLR10	SH2B3	NR4A2	JUNB
CELLMARKERMSIGDB_HU_FETAL_RETINA_MULLER																														
CELLMARKERMSIGDB_HU_FETAL_RETINA_PHOTOREC…																														
CELLMARKERMSIGDB_HU_FETAL_RETINA_RGC	PFDN4	FAR2	COMTD1	FSD1	FNBP1L	SRGAP1	TBCB	PCSK1N	PNMA2	TMEM97	PRUNE2	ADD2	ATAT1	SQLE	PCMT1	RAB31	ACAT2	RBFOX3	RXRG	KLC1	ELAVL3	MAP1L…	MSMO1	HCFC1…	RAB3A	MGST3	VAT1	STMN4	NDRG4	CDS2
CELLMARKERMSIGDB_LAKE_ADULT_KIDNEY_C1_EPI…																														
CELLMARKERMSIGDB_LAKE_ADULT_KIDNEY_C10_TH…	SOD1	BTF3	TPI1	XRCC5	SLC38…	PON2	CYCS	ACADVL	YWHAB	TMEM59	SDCBP	RPL13A	NDUFA4	RPS18	RPS5	ITM2C	PSMA7	PDIA6	HSP90…	TNFSF…	RPS20	GNAI1	SPPL2A	CDH16	SEC14…	CCT3	APLP2	PNRC1	PPIA	RPL13
CELLMARKERMSIGDB_LAKE_ADULT_KIDNEY_C12_TH…	RPL7A	GOT1	PRDM16	COX6C	CYCS	RPS20	MPC2	NDUFB2	RANBP…	RPL7	ITM2B	KIF12	PKM	RPS16	CIRBP	UQCR10	ESRRG	ATP5ME	RPL11	BACE2	GPX4	PLXNB1	LINC0…	CA12	CD81	RPS28	PCDH9	RPS17	CDH16	RPLP1
CELLMARKERMSIGDB_LAKE_ADULT_KIDNEY_C18_CO…	CRYAB	BTF3	ATP6V…	HSP90…	ZFP36…	RPS19	ALDOA	NPM1	RPL10A	EPRS1	PPIA	SOD2	CHCHD2	RPSA	C11or…	METAP2	RPL13A	MYH10	PLSCR1	THSD7A	RPL6	REEP5	TMEM2…	PPIB	PDIA3	SGPP1	RPS8	CREM	SLC44…	YWHAB
CELLMARKERMSIGDB_LAKE_ADULT_KIDNEY_C19_CO…	SLC25…	LIMA1	TMEM1…	PRDX6	SARAF	TMPRS…	EEF2	TACST…	ATP6V…	HINT1	GSTP1	RPS27A	MDH1	RPS11	ATP5F…	LAMTO…	CUL5	RPLP1	NDUFA4	RPL30	RPL10A	ATP5F…	ATP5M…	CHCHD2	GRB14	MBOAT2	SNTB1	EIF4A2	PTBP3	ALDH1…
CELLMARKERMSIGDB_LAKE_ADULT_KIDNEY_C25_EN…																														
CELLMARKERMSIGDB_LAKE_ADULT_KIDNEY_C28_IN…																														
CELLMARKERMSIGDB_LAKE_ADULT_KIDNEY_C29_UN…																														
CELLMARKERMSIGDB_LAKE_ADULT_KIDNEY_C5_PRO…	MCTP1	NSUN6	LINC0…	SLC16…	METTL…	AGXT2	ANPEP	KCNK5	CYP4A…	LINC0…	PDE4D	PGPEP1	BBS9	AP3D1	KIF12	EPHX2	CYP3A5	GLIS1	COL27…	TCEA3	TIMM2…	RASSF4	ACSF2	RBM25	ZBTB16	RGN	DNM2	PRODH2	DCC	COL4A1
CELLMARKERMSIGDB_MANNO_MIDBRAIN_NEUROTYPE…																														
CELLMARKERMSIGDB_MANNO_MIDBRAIN_NEUROTYPE…	ENO2	EPB41…	EPHA5	ERBB4	ERC2	TMEM1…	RETRE…	FAM13C	NALF1	TAFA1	FAM21…	CYRIA	RIPOR2	FAT3	FAXC	FBLL1	FBXL16	FGF13	FGF9	FJX1	FNBP1	FNDC5	FNDC9	FOXA1	FOXA2	FOXP2	FRMD3	FSD1	FSTL4	FUT9
CELLMARKERMSIGDB_MANNO_MIDBRAIN_NEUROTYPE…	DOK6	DPP6	DPYSL3	DSCAM	DUSP26	DUSP4	EBF3	EHBP1	EID2B	ELAVL2	ELAVL3	ELAVL4	ELFN2	ELOVL3	ELOVL4	EN1	EN2	ENC1	ENO2	ENPP4	EPB41	EPB41…	EPB41…	EPHA5	ERBB4	ERC2	FADS3	FAIM2	TMEM1…	RETRE…
CELLMARKERMSIGDB_MANNO_MIDBRAIN_NEUROTYPE…	EID2B	ELAVL2	ELAVL4	ELOVL3	ELOVL4	EN1	EN2	ENHO	ENO2	ENOX1	ENPP4	EPB41…	EPB41…	EPHA5	ERBB4	ERC2	FADS3	FAIM2	RETRE…	NALF1	FAM18…	ENTRE…	CALHM6	TLCD3B	FBLL1	FBXL16	FGF13	FGF9	FHOD3	FKBP1B
CELLMARKERMSIGDB_MANNO_MIDBRAIN_NEUROTYPE…	CLIC2	CLIC4	CLIC5	CNN2	COBLL1	COX7A1	CPD	CPNE2	CPNE5	CPT1A	CREB5	CREM	CRIM1	CRTAP	CSF3	CSGAL…	CSRNP1	CSRP1	CSRP2	CTDSP1	CCN2	CTHRC1	CXCL1	CXCL12	CXCL2	CXCL8	DIPK2B	CYB5A	CYBA	CCN1
CELLMARKERMSIGDB_MANNO_MIDBRAIN_NEUROTYPE…	CYTIP	DAB2	INTS6L	DENND3	DENND…	DEPTOR	DERL3	DHRS3	DHRS9	DNAJA4	DNAJB1	DNAJB4	DOCK1	DOCK4	DOCK8	DOK2	DPF3	DPP7	DRAM1	DRAM2	DUSP1	DUSP2	EDEM1	EFHD2	EGR1	EGR2	EGR3	ELF1	ELL2	ELOVL1
CELLMARKERMSIGDB_MANNO_MIDBRAIN_NEUROTYPE…	CNTRL	COMTD1	CPEB2	CPEB3	CPEB4	CREG2	CRMP1	CSAD	CSGAL…	CSMD1	CSMD2	CSMD3	CSRNP3	CTNNA3	CTTNB…	CXADR	CYFIP2	DAAM1	DACT1	DCLK1	DCX	DENND3	DGKE	DICER1	DIRAS3	DLG2	DMTN	DNAJC3	DNAJC6	DND1
CELLMARKERMSIGDB_MANNO_MIDBRAIN_NEUROTYPE…	LPAR2	LPCAT4	PLPPR1	PLPPR3	LRRC49	LRRC55	LRRTM4	MAML3	MAP1B	MAP2	MAP6	MAP7D2	MAPK6	MAPK8	MAPK8…	MAPRE3	MAST1	MCF2	MCF2L	MIAT	MICAL1	MIR12…	MIR7-3	MLLT11	MPP3	MSANT…	MSANT…	MSI1	MYO5A	MYT1
CELLMARKERMSIGDB_MANNO_MIDBRAIN_NEUROTYPE…	FNDC9	FOXA2	FRMD5	FRY	FSCN1	FSD2	FTO	FUT9	GABRA1	GABRB2	GABRG2	GAD1	GAD2	GATA2	GATA3	GATA3…	GNAO1	GNG2	GNG3	GNPTAB	GOLGA…	GPC2	GPM6A	GPR12	GPR13…	GPR26	GRIA1	GRIA2	GRIA4	GRIP1
CELLMARKERMSIGDB_MANNO_MIDBRAIN_NEUROTYPE…	GARNL3	GDAP1	GDAP1…	GLRA2	GLRX	GNAL	GNG2	GNG3	GPC2	ADGRA…	GPR155	GPR176	GPR85	GPRIN1	GREM2	GRIA4	GRIK1	GRM1	GSG1L	HCN3	HMGCL…	HSPA1…	HTR3A	INA	INHBA…	IRX3	ISL1	ISL2	JPH3	JPH4
CELLMARKERMSIGDB_MANNO_MIDBRAIN_NEUROTYPE…	GFOD1	GNAL	GNG4	GNG7	GPM6A	GPM6B	GPR17	ADGRG1	GRAMD…	TAMAL…	GRB14	GRIA3	GRIA4	GRID1	GRID2	GRM5	GTSE1	HAGH	HBA1	HBA2	HBB	HBG1	HBG2	HEPAC…	HEPN1	HES5	HSH2D	IDH1	IFI27…	IGF1
CELLMARKERMSIGDB_MANNO_MIDBRAIN_NEUROTYPE…	COL1A2	COL21…	COL3A1	COL4A1	COL4A2	COL5A2	COL5A3	COL6A1	COL9A1	COLEC…	COLEC…	COX4I2	COX7A1	CPED1	CPQ	CPT1A	CREM	CRIP1	CRISP…	CRTAP	CSAG1	CSPG4	CSRNP1	CTDSP1	CTSK	CXCL2	CYBA	CYP4X1	CCN1	CYSLT…
CELLMARKERMSIGDB_MANNO_MIDBRAIN_NEUROTYPE…	JAM2	KCNQ2	CIP2A	KIF11	KIF14	KIF15	KIF18A	KIF22	KIF23	KIF2A	KIF2B	KIF2C	KIF4A	KIRRE…	KNSTRN	LAMA1	LBR	LGR5	LHFPL6	LIMCH1	LITAF	LMNB1	LMNB2	LRP2	LRRN1	MAD2L1	MAT2B	SRF	MCM5	MCM6
CELLMARKERMSIGDB_MANNO_MIDBRAIN_NEUROTYPE…	H3C3	H4C6	H4C11	HJURP	HK2	HMGA1	HMGA2	HMGB2	HMGN2	HMMR	JPT2	IGDCC3	IGF2B…	IGF2B…	IGFBP2	ITGB3…	JAM2	JAM3	KCNQ2	DENND…	KIF11	KIF14	KIF15	KIF18A	KIF22	KIF23	KIF2A	KIF2C	KIF4A	KLHDC…
CELLMARKERMSIGDB_MANNO_MIDBRAIN_NEUROTYPE…	HELLS	HES1	HILPDA	H2BC9	H2BC11	H3C2	H3C3	H3C7	H4C6	H4C9	H4C11	H4C12	H2AC25	HJURP	HK2	HMGA1	HMGA2	HMGB1	HMGB2	HMGB3	HMGN2	HMGN5	HMMR	HNRNP…	HSH2D	HUNK	IGDCC3	IGDCC4	IGF2B…	IGFBP2
CELLMARKERMSIGDB_MANNO_MIDBRAIN_NEUROTYPE…	HMGA2	HMGB2	HMMR	HYDIN	IFT88	IGDCC3	IGF2B…	IGFBP2	IQGAP2	IRS1	ITGA6	ITGB3…	ITGB8	JADE1	JAM2	JAM3	KCNG1	KCNMA1	KIF11	KIF15	KIF2A	KITLG	KLHDC…	KLHL32	L3HYP…	LGI1	LIPG	LMX1A	LMX1B	ENSG0…
CELLMARKERMSIGDB_MANNO_MIDBRAIN_NEUROTYPE…	GPRC5C	GRIK1	GULP1	HBA1	HBA2	HBG1	HBG2	HEATR…	HEPAC…	HES1	HES5	HEY1	HK2	RIDA	HYDIN	ID3	ID4	IFI6	IGDCC3	IGDCC4	IGFBP2	IGFBP5	IGSF1	IL18	IL33	ILDR2	INHBB	INTU	IQCA1	IQCG
CELLMARKERMSIGDB_MANNO_MIDBRAIN_NEUROTYPE…	DIO3	DKK3	DLEC1	DNAAF1	DNAAF3	DNAH6	DNAH7	DNAI2	DNAJB1	DNAJB…	DNAJB4	DNALI1	DOK5	DPP6	DTNA	DTX4	DNAAF…	EDNRB	EEPD1	CLXN	EFEMP1	EFEMP2	EFHC2	EGFR	EGR1	EGR2	ELN	ELOVL2	EMP1	ENHO
CELLMARKERMSIGDB_MANNO_MIDBRAIN_NEUROTYPE…	FLJ16…	FOS	FOXJ1	FZD2	GAB1	GABBR2	GABRG1	GAREM2	GATM	GBX2	GDPD2	GFAP	GINS1	GJA1	GLIS3	GNG12	GNG7	GOLGA…	GPC1	GPC4	GPM6B	GPR13…	ADGRG1	GPX8	GRIA1	GRID2	GRM3	H2AX	HAS2	HBA1
CELLMARKERMSIGDB_MANNO_MIDBRAIN_NEUROTYPE…	HES1	HES5	HRH1	HTRA1	IER3I…	IFI44L	IGFBP2	ILDR2	IQCA1	IRX1	ITGAV	ITGB5	ITGB8	ITPKB	ITPR2	JPH3	ANOS1	KANK1	KAT2B	KCND2	KCNE5	KCNIP1	KCNJ1	KCNQ2	MYORG	CEP126	KLRC2	KRT17	LAMA1	LAMB2
CELLMARKERMSIGDB_MANNO_MIDBRAIN_NEUROTYPE…	DCDC2	DCLK1	DCN	DDAH1	DHRS3	DIRAS2	DKK3	DLG5-…	DLGAP…	DNAAF3	DNAH6	DNAH7	DNAJB…	DNAJB4	DNALI1	CTSC	DPP6	DPP7	DSC2	DSC3	DSG2	DST	DTHD1	DTNA	DTX4	DNAAF…	ECHDC2	EDNRB	EEPD1	CLXN
CELLMARKERMSIGDB_MANNO_MIDBRAIN_NEUROTYPE…	KCND2	KCNH8	KCNQ3	KHDRB…	ARFGE…	KIF21B	KIF26A	KIF3A	KIF3C	KIF5C	KLC1	KLHL1	KLHL35	KMO	L1CAM	LETMD1	LHFPL4	LHX1	LMTK3	ENSG0…	LONRF2	LONRF3	LPAR2	LPGAT1	LPIN1	PLPPR2	PLPPR4	LRFN5	LRRC49	LRRTM4
CELLMARKERMSIGDB_MANNO_MIDBRAIN_NEUROTYPE…	FGF14	FKBP1B	FNDC9	FRMPD4	FRY	FSD1	GABBR2	GABRB1	GABRB2	GABRG1	GABRG2	GAP43	GARNL3	GATA2	GATA3	GATA3…	GCH1	GCHFR	GDAP1…	GFOD1	GLRA2	GLT8D2	GNAL	GNAO1	GNG2	GNG3	GOLGA…	GOT1	GPC3	GPM6A
CELLMARKERMSIGDB_MENON_FETAL_KIDNEY_0_CAP…																														
CELLMARKERMSIGDB_MENON_FETAL_KIDNEY_10_IM…																														
CELLMARKERMSIGDB_MENON_FETAL_KIDNEY_2_NEP…																														
CELLMARKERMSIGDB_MENON_FETAL_KIDNEY_3_STR…																														
CELLMARKERMSIGDB_MENON_FETAL_KIDNEY_6_COL…																														
CELLMARKERMSIGDB_MURARO_PANCREAS_ACINAR_C…	FBP1	NFIB	GPR160	EDN1	FAM13A	AMY2B	C4orf…	LRG1	IFITM3	REG1CP	RASEF	LYN	GPRC5A	ATP8B1	ERBB3	CLDN4	PLTP	SH3BP4	CCL28	GALNT7	OCLN	MET	NFKBIZ	CYP17…	ABCC3	SERPI…	CXCL1	PHLDA1	RASSF6	PACSI…
CELLMARKERMSIGDB_MURARO_PANCREAS_ALPHA_CE…	GUSBP…	CNPY2	PPP3CB	OGT	FNDC3A	COPE	PDXDC…	PSEN2	IDS	CUTA	ZKSCA…	INA	RAB29	TRAK1	GDI1	SLC36…	SPCS1	NDUFA…	BIVM	FBXL16	CNKSR3	AIG1	ST18	EPB41…	SH3KB…	PPP1R…	LIMCH1	CALM1	MAGI2	BTG2
CELLMARKERMSIGDB_MURARO_PANCREAS_BETA_CELL	KIF1A	HSPA1A	DDIT3	ELMO1	CAMK2…	PCLO	SIX2	ENO1	BTBD3	OLFM1	GSE1	SERIN…	MAN1C1	AKT3	MLXIPL	DIP2C	PFN2	CEBPG	ACLY	HSP90…	TMEM37	FAM10…	FMN2	CYYR1	HS6ST1	TSPAN…	EIF4B	VAT1L	TMOD1	PSD3
CELLMARKERMSIGDB_MURARO_PANCREAS_ENDOTHEL…	NOVA2	FRMD4A	BMPR2	ST8SI…	GATA2	RAB31	SPARC…	ID3	PMEPA1	PRSS23	NOTCH1	ZEB1	P2RY6	LIFR	RAI14	EFNB2	IFITM1	DUSP6	SLC7A…	LRRC8A	NRARP	SERPI…	CD9	NLRC3	IGFBP7	PPP1R…	SH2D3C	RASGR…	FHOD1	SEC14…
CELLMARKERMSIGDB_MURARO_PANCREAS_EPSILON_…																														
CELLMARKERMSIGDB_MURARO_PANCREAS_MESENCHY…	LAMB1	BMP1	MMP1	KCNE4	SERPI…	CYTOR	KANK4	PIEZO2	SNAI1	F2RL2	KANK2	FKBP10	PHGDH	GNG11	FHL2	SULF2	RUNX1	PHLDB1	SCARA3	CALU	GPNMB	OLFML…	NOTCH1	CHSY1	SMIM3	PPP1R…	FLNA	CARMN	ARHGE…	IGFBP7
CELLMARKERMSIGDB_RUBENSTEIN_SKELETAL_MUSC…	MCL1	SUPT4…	PLBD1	ACTB	PRR13	WDR74	VMA21	ATP6V…	CYRIA	SERPI…	CTSZ	DNAJB1	PPT1	RHOG	HLA-D…	ZFP36	PFDN5	TMEM1…	RNF130	FOSB	ATP6V…	IFNGR2	CTSH	RPL39	SLC25…	PTP4A2	S100A4	PGLS	RPS24	CD55
CELLMARKERMSIGDB_RUBENSTEIN_SKELETAL_MUSC…	EEF2	EEF1D	RPS10	RPLP2	RPL23A	CD9	NPM1	MIF	RPL36	AK1	RPSA	NPC2	NFIA	IER2	RPS26	NDUFS5	RPS28	FXYD1	EIF3E	RPS21	RPS14	EIF3H	RPS25	ESD	TLE4	EIF1	GAPDH	RPL38	TPT1	RPL27
CELLMARKERMSIGDB_RUBENSTEIN_SKELETAL_MUSC…	SLC25…	SLC25…	CCL2	ROCK1	KANK2	CAPZA2	MT-CO2	PLAC9	COX7C	COX6C	TSC22…	CCND1	COX5B	CCNI	CNN3	AIG1	ECRG4	HES1	JAG1	TGFB1…	CRYAB	FOS	COX14	COX7A1	TNS1	ARPC5	WDR1	PDCL3	H1-0	CYSTM1
CELLMARKERMSIGDB_TRAVAGLINI_LUNG_ALVEOLAR…	BDNF	CD47	ITGA3	EYA4	CEACA…	NEXN	GJA1	PDLIM3	RDX	RBP1	C10or…	TTLL7	RNASE1	GPRIN2	ALS2CL	DLC1	ANO6	ATP2B4	ATP1B3	CPM	HSPG2	ANKRD1	SPRYD7	MYO1B	AHNAK	SPRIN…	P4HA2	TACC1	FOLR1	FBLN5
CELLMARKERMSIGDB_TRAVAGLINI_LUNG_BASOPHIL…	FDX1	ZNF331	GABPB1	NFE2L2	PRNP	BTK	GNA15	LIF	PLAUR	MYO5C	PBX1	TMEM2…	SVOPL	CAMLG	PLPP1	RBM39	TENT5A	GMPR	CTNNB…	NFKB1	RPL36…	ALG13	SRSF7	HSP90…	PALLD	PRG2	ABHD1…	ZBTB32	CDK15	NSMCE…
CELLMARKERMSIGDB_TRAVAGLINI_LUNG_CAPILLAR…																														
CELLMARKERMSIGDB_TRAVAGLINI_LUNG_CAPILLAR…	PSMB9	ATP5F…	SEPTI…	PRMT1	SH3BG…	ZEB1	IK	AP3S1	PCNP	SNRPD2	ANP32B	ROMO1	PET100	NDUFA2	MORF4…	EI24	DYNLT1	MRPS1…	CALR	MAP4	COX7C	DAZAP2	TMEM2…	RTF2	HNRNP…	ARL2BP	SUMO3	SSU72	COX4I1	GPX1
CELLMARKERMSIGDB_TRAVAGLINI_LUNG_CD4_MEMO…																														
CELLMARKERMSIGDB_TRAVAGLINI_LUNG_EREG_DEN…	RILPL2	S100A…	ZFP36…	PFKFB3	LCP2	HLA-D…	NEAT1	PTPN1	VSIG4	MAT2A	LGALS1	SCO2	CXCL16	TPM4	PEA15	BCAT1	CREM	MT2A	ADGRE2	NLRP3	CTSH	LUCAT1	GSN	CTSZ	PLAU	PDE4B	VDR	PSAP	EPB41…	FOS
CELLMARKERMSIGDB_TRAVAGLINI_LUNG_IGSF21_D…																														
CELLMARKERMSIGDB_TRAVAGLINI_LUNG_MESOTHEL…	DHCR24	TNFSF…	JPT1	PHLDA2	PHGDH	PAFAH…	UPK1B	RAB31	CTNNA…	GGCT	C4orf…	CLTC	TIMM9	ATP13…	PPP1R…	FAM17…	TAF10	RPS17	RBBP8	CNKSR3	BICC1	UROS	PYCARD	IL20	RAB7B	FAM20A	METRN	TNFSF…	CISD1	PTGIS
CELLMARKERMSIGDB_TRAVAGLINI_LUNG_MYOFIBRO…																														
CELLMARKERMSIGDB_TRAVAGLINI_LUNG_NATURAL_…																														
CELLMARKERMSIGDB_TRAVAGLINI_LUNG_NEUTROPH…	TYROBP	RNF145	CPD	CIRBP	CR1	FTH1P3	C16or…	TAPBP	KLF2	MX2	PGGHG	CICP27	JTB	TXNL4A	COX8A	SOCS3	MARCH…	B4GAL…	DHX34	H3P6	PER1	BCL6	BRI3	RNF213	CKAP4	CLEC4D	SELL	WAS	FGD3	FPR2
CELLMARKERMSIGDB_TRAVAGLINI_LUNG_OLR1_CLA…	CCL2	HCK	LST1	AK4	CD14	SLC2A3	DMXL2	EIF1B	DRAM1	CTSS	YBX3	SIGLE…	CEP170	ATP2B1	AGFG1	SERPI…	YWHAZ	IL1R1	SOCS3	SEMA6B	SPAG9	TMEM1…	CSF3R	LIMS1	FLT1	IRAK3	FTH1P3	ZMIZ1	NECTI…	RUNX1
CELLMARKERMSIGDB_TRAVAGLINI_LUNG_PLATELET…	TACC3	CDKN2D	PRDX6	CCDC8…	LGALSL	SPOCD1	PDE5A	FAM11…	DNAJB6	LYPLA…	RAB32	GUCY1…	LTBP1	VDAC3	SLPI	CAPN1	STX11	PIP4P2	MGLL	WDR1	MINDY1	ARPC1B	GADD4…	LAT	MIR68…	SPNS1	MTURN	DYNLL1	P2RY12	MFSD1
CELLMARKERMSIGDB_TRAVAGLINI_LUNG_PROLIFER…	H2AZ2	CHTF18	FAM83D	CDC6	YBX1	KPNA2	FAM11…	RFC4	RPSA	NCAPD2	KIF4A	TYMSOS	SERPI…	NUCKS1	LMNB1	CENPM	SAE1	PKM	PLP2	TMPO	CLN6	CKS2	UHRF1	TMEM1…	LGALS…	DAPL1	MTFR2	IMPDH2	DSC3	SNCA
CELLMARKERMSIGDB_TRAVAGLINI_LUNG_PROLIFER…	HADH	HJURP	CES1	CENPS	AKR1C3	PRIM1	PARAL1	SERPI…	DIAPH3	INHBA	BRCA1	S100A…	ITGB3…	UHRF1	RHEB	OASL	FAM89A	ACP5	APOE	VMO1	ATAD2	ORC6	OIP5	NUPR1	SKA2	CDKN2C	TACC3	LRPAP1	CTSC	SCCPDH
CELLMARKERMSIGDB_TRAVAGLINI_LUNG_PROXIMAL…	RPL6	EGFR	RPS3A	NFIA	CAPN1	ITGA2	AKR1A1	IRAG2	F3	PLEKH…	CDKN2C	IFI16	RPL10	C16or…	TNFRS…	HMGB3	EIF3L	FOXN3	FAM3B	NOP53	IFITM…	LDLRA…	FAM3D	MDK	RPS8	SLC9A…	TSC22…	CHCHD…	DSC3	PAX9
CELLMARKERMSIGDB_TRAVAGLINI_LUNG_SEROUS_C…																														
CELLMARKERMSIGDB_TRAVAGLINI_LUNG_SIGNALIN…																														
CELLMARKERMSIGDB_TRAVAGLINI_LUNG_TREM2_DE…	SGK1	CORO1C	HLA-D…	CD151	HLA-D…	FCGR2A	CREG1	LTA4H	ATOX1	NCF2	GAA	UBE2E2	RBM47	OLR1	FILIP…	RAB20	LILRB5	IFI27	BHLHE…	ABHD12	SIRPA	RGCC	TGFBI	LAIR1	GNS	ALDH1…	PDXK	HCK	HLA-D…	LAMP1
CELLMARKERMSIGDB_TRAVAGLINI_LUNG_VASCULAR…																														
CELLMARKERMSIGDB_TRAVAGLINI_LUNG_VEIN_CELL																														
CELLMARKERMSIGDB_VANGURP_PANCREATIC_ALPHA…																														
CELLMARKERMSIGDB_VANGURP_PANCREATIC_DELTA…																														
CELLMARKERMSIGDB_VANGURP_PANCREATIC_GAMMA…																														
CELLMARKERMSIGDB_ZHENG_CORD_BLOOD_C1_PUTA…																														
CELLMARKERMSIGDB_ZHENG_CORD_BLOOD_C10_MUL…																														
CELLMARKERMSIGDB_ZHENG_CORD_BLOOD_C2_PUTA…																														
CELLMARKERMSIGDB_ZHENG_CORD_BLOOD_C4_PUTA…																														
CELLMARKERMSIGDB_ZHENG_CORD_BLOOD_C5_SIMI…																														
CELLMARKERMSIGDB_ZHENG_CORD_BLOOD_C6_HSC_…																														
CELLMARKERMSIGDB_ZHENG_CORD_BLOOD_C7_PUTA…																														
CELLMARKERMSIGDB_ZHENG_CORD_BLOOD_C8_PUTA…																														
CELLMARKERMSIGDB_ZHONG_PFC_C1_ASTROCYTE																														
CELLMARKERMSIGDB_ZHONG_PFC_C1_DLX5_POS_IN…																														
CELLMARKERMSIGDB_ZHONG_PFC_C2_ASCL1_POS_A…																														

Table S5: Cell state marker gene list

CELLMARKERCAN_ACINAR_CELLS																														
CELLMARKERMSIGDB_ZHONG_PFC_C2_UNKNOWN_NPC																														
CELLMARKERMSIGDB_ZHONG_PFC_C3_ASTROCYTE	GRK3	PLXNA4	MMP17	EVL	MMD	NBEA	HRAS	FLRT2	ZEB2	SSX2IP	MICAL1	ZNF527	SIN3B	MYCN	CALY	KCTD12	CHST15	CNIH2	CACNB3	AFAP1	KCTD3	TUBGC…	SYT1	GAS7	ZNF573	LMO7	NKAIN1	POU2F2	VSTM2L	PNPLA6
CELLMARKERMSIGDB_ZHONG_PFC_C3_HOPX_POS_OPC																														
CELLMARKERMSIGDB_ZHONG_PFC_C3_MICROGLIA	SLIT1	CDC42…	GNG4	PDIA2	RAP1G…	PRKAR…	FXR2	MAST1	KNDC1	ACAP3	TMEM1…	DMWD	CYRIA	CELF5	NCAN	NDRG4	CHGB	PRKAR…	TRIM2	RAB15	KRT19	GNAZ	ATP1B2	SYNGA…	DNM3	ANK3	CSRP2	PTP4A3	ENO2	SCD5
CELLMARKERMSIGDB_ZHONG_PFC_C3_UNKNOWN_INP																														
CELLMARKERMSIGDB_ZHONG_PFC_C4_UNKNOWN_INP																														
CELLMARKERMSIGDB_ZHONG_PFC_C6_PLXNA4_POS_…																														
CELLMARKERMSIGDB_ZHONG_PFC_C7_ORG_UNDERGO…																														
CELLMARKERMSIGDB_ZHONG_PFC_C8_ORG_PROLIFE…																														
CELLMARKERMSIGDB_ZHONG_PFC_C8_UNKNOWN_NEU…																														
CELLMARKERMSIGDB_ZHONG_PFC_C9_ORG_OTHER																														
CELLMARKERMSIGDB_ZHONG_PFC_HES1_POS_C1_NPC																														
CELLMARKERMSIGDB_ZHONG_PFC_MAJOR_TYPES_AS…	HEY1	SASH1	SOX9	BDH2	CBR1	SEZ6L	SELEN…	STON2	AIF1L	PHACT…	ID4	SLITR…	PDPN	SERPI…	TM7SF2	LAMB2	GNG12	BMAL1	MYORG	GNG11	B2M	HES4	TIMP1	LRATD2	WSCD1	LRRTM3	RGMA	DAG1	PDLIM3	DDAH1
CELLMARKERMSIGDB_ZHONG_PFC_MAJOR_TYPES_EX…																														
CELLMARKERMSIGDB_ZHONG_PFC_MAJOR_TYPES_MI…	ADRB2	RNASE6	SIGLE…	INPP5D	TNFRS…	SGK1	BIN1	DHRS3	FCGR1…	CEBPA	DHRS9	RHBDF2	ABI3	IL13R…	GRN	TMEM3…	OTULI…	GIMAP4	RGS19	PDGFB	CD300A	CXCL16	EGR2	MRC1	CD86	PLVAP	CD4	TPP1	IFITM2	SYNGR2
CELLMARKERMSIGDB_ZHONG_PFC_MAJOR_TYPES_OPC																														

Table S5: Cell state marker gene list

CELLMARKERCAN_ACINAR_CELLS																														
CELLMARKERCAN_ADIPOCYTE_PROGENITOR_CELLS																														
CELLMARKERCAN_ADRENERGIC_NEURONS																														
CELLMARKERCAN_AIRWAY_EPITHELIAL_CELLS																														
CELLMARKERCAN_ALPHA_CELLS																														
CELLMARKERCAN_ALVEOLAR_MACROPHAGES																														
CELLMARKERCAN_ASTROCYTES																														
CELLMARKERCAN_B_CELLS																														
CELLMARKERCAN_B_CELLS_MEMORY																														
CELLMARKERCAN_B_CELLS_NAIVE																														
CELLMARKERCAN_BASOPHILS																														
CELLMARKERCAN_BERGMANN_GLIA																														
CELLMARKERCAN_BETA_CELLS																														
CELLMARKERCAN_CAJAL_RETZIUS_CELLS																														
CELLMARKERCAN_CARDIAC_STEM_AND_PRECURSOR_…																														
CELLMARKERCAN_CARDIOMYOCYTES																														
CELLMARKERCAN_CHOLANGIOCYTES																														
CELLMARKERCAN_CHOLINERGIC_NEURONS																														
CELLMARKERCAN_CHONDROCYTES																														
CELLMARKERCAN_CHOROID_PLEXUS_CELLS																														
CELLMARKERCAN_CILIATED_CELLS																														
CELLMARKERCAN_DELTA_CELLS																														
CELLMARKERCAN_DOPAMINERGIC_NEURONS																														
CELLMARKERCAN_DUCTAL_CELLS																														
CELLMARKERCAN_EMBRYONIC_STEM_CELLS																														
CELLMARKERCAN_ENTERIC_GLIA_CELLS																														
CELLMARKERCAN_ENTERIC_NEURONS																														
CELLMARKERCAN_ENTEROCYTES																														
CELLMARKERCAN_ENTEROENDOCRINE_CELLS																														
CELLMARKERCAN_EPENDYMAL_CELLS																														
CELLMARKERCAN_EPSILON_CELLS																														
CELLMARKERCAN_FOLLICULAR_CELLS																														
CELLMARKERCAN_FOVEOLAR_CELLS																														
CELLMARKERCAN_GAMMA_DELTA_T_CELLS																														
CELLMARKERCAN_GERM_CELLS																														
CELLMARKERCAN_GLUTAMINERGIC_NEURONS																														
CELLMARKERCAN_GRANULOSA_CELLS																														
CELLMARKERCAN_HEMATOPOIETIC_STEM_CELLS																														
CELLMARKERCAN_IMMATURE_NEURONS																														
CELLMARKERCAN_INTERNEURONS																														
CELLMARKERCAN_IONOCYTES																														
CELLMARKERCAN_KERATINOCYTES																														
CELLMARKERCAN_KUPFFER_CELLS																														
CELLMARKERCAN_LEYDIG_CELLS																														
CELLMARKERCAN_LOOP_OF_HENLE_CELLS																														
CELLMARKERCAN_LUMINAL_EPITHELIAL_CELLS																														
CELLMARKERCAN_LUTEAL_CELLS																														
CELLMARKERCAN_MEGAKARYOCYTES																														
CELLMARKERCAN_MELANOCYTES																														
CELLMARKERCAN_MESANGIAL_CELLS																														
CELLMARKERCAN_MESOTHELIAL_CELLS																														
CELLMARKERCAN_MICROFOLD_CELLS																														
CELLMARKERCAN_MICROGLIA																														
CELLMARKERCAN_MONOCYTES																														
CELLMARKERCAN_MOTOR_NEURONS																														
CELLMARKERCAN_MULLER_CELLS																														
CELLMARKERCAN_MYELOID_DERIVED_SUPPRESSOR_…																														
CELLMARKERCAN_MYOBLASTS																														
CELLMARKERCAN_MYOEPITHELIAL_CELLS																														
CELLMARKERCAN_MYOFIBROBLASTS																														
CELLMARKERCAN_NEUROBLASTS																														
CELLMARKERCAN_NEUROENDOCRINE_CELLS																														
CELLMARKERCAN_NEURONS																														
CELLMARKERCAN_NEUTROPHILS																														
CELLMARKERCAN_NK_CELLS																														
CELLMARKERCAN_NORADRENERGIC_NEURONS																														
CELLMARKERCAN_NUOCYTES																														
CELLMARKERCAN_OLIGODENDROCYTES																														
CELLMARKERCAN_OSTEOBLASTS																														
CELLMARKERCAN_OSTEOCLAST_PRECURSOR_CELLS																														
CELLMARKERCAN_OSTEOCLASTS																														
CELLMARKERCAN_OSTEOCYTES																														
CELLMARKERCAN_PANETH_CELLS																														
CELLMARKERCAN_PARATHYROID_CHIEF_CELLS																														
CELLMARKERCAN_PERICYTES																														
CELLMARKERCAN_PERITUBULAR_MYOID_CELLS																														
CELLMARKERCAN_PHOTORECEPTOR_CELLS																														
CELLMARKERCAN_PLASMA_CELLS																														
CELLMARKERCAN_PLASMACYTOID_DENDRITIC_CELLS																														
CELLMARKERCAN_PODOCYTES																														
CELLMARKERCAN_PROXIMAL_TUBULE_CELLS																														
CELLMARKERCAN_PULMONARY_ALVEOLAR_TYPE_I_C…																														
CELLMARKERCAN_PULMONARY_ALVEOLAR_TYPE_II_…																														
CELLMARKERCAN_PURKINJE_NEURONS																														
CELLMARKERCAN_RETICULOCYTES																														
CELLMARKERCAN_RETINAL_GANGLION_CELLS																														
CELLMARKERCAN_RETINAL_PROGENITOR_CELLS																														
CELLMARKERCAN_SATELLITE_CELLS																														
CELLMARKERCAN_SCHWANN_CELLS																														
CELLMARKERCAN_SEROTONERGIC_NEURONS																														
CELLMARKERCAN_SERTOLI_CELLS																														
CELLMARKERCAN_SMOOTH_MUSCLE_CELLS																														
CELLMARKERCAN_STROMAL_CELLS																														
CELLMARKERCAN_T_CELLS																														
CELLMARKERCAN_T_CYTOTOXIC_CELLS																														
CELLMARKERCAN_T_FOLLICULAR_HELPER_CELLS																														
CELLMARKERCAN_T_MEMORY_CELLS																														
CELLMARKERCAN_TRANSIENT_CELLS																														
CELLMARKERCAN_TRICHOCYTES																														
CELLMARKERCAN_TRIGEMINAL_NEURONS																														
CELLMARKERCAN_TROPHOBLAST_CELLS																														
CELLMARKERCAN_TROPHOBLAST_PROGENITOR_CELLS																														
CELLMARKERCAN_TUFT_CELLS																														
CELLMARKERCANENDOTHELIALCELLSAORTA																														
CELLMARKERCUR_N_ABNORMAL_MYELOID_CELL																														

Table S5: Cell state marker gene list

CELLMARKERCAN_ACINAR_CELLS																														
CELLMARKERCUR_N_ABNORMAL_PLASMA_CELL																														
CELLMARKERCUR_N_ACTIVATED_DENDRITIC_CELL																														
CELLMARKERCUR_N_ACTIVATED_EFFECTOR_CELL																														
CELLMARKERCUR_N_ACTIVATED_HEPATIC_STELLAT…																														
CELLMARKERCUR_N_ACTIVATED_LANGERHANS_CELL																														
CELLMARKERCUR_N_ACTIVATED_MACROPHAGE																														
CELLMARKERCUR_N_ACTIVATED_MEMORY_T_CELL																														
CELLMARKERCUR_N_ACTIVATED_NATURAL_KILLER_…																														
CELLMARKERCUR_N_ACTIVATED_REGULATORY_T_CE…																														
CELLMARKERCUR_N_ADIPOCYTE_PROGENITOR_CELL																														
CELLMARKERCUR_N_ADRENAL_MEDULLA_CELL																														
CELLMARKERCUR_N_ADULT_SPERMATOGONIA																														
CELLMARKERCUR_N_ADVENTITIAL_CELL																														
CELLMARKERCUR_N_AEROCYTE																														
CELLMARKERCUR_N_AIRWAY_SMOOTH_MUSCLE_CELL																														
CELLMARKERCUR_N_ALVEOLAR_CELL_TYPE_1																														
CELLMARKERCUR_N_ALVEOLAR_CELL_TYPE_2																														
CELLMARKERCUR_N_ALVEOLAR_EPITHELIAL_CELL_…																														
CELLMARKERCUR_N_ALVEOLAR_EPITHELIAL_CELL_…																														
CELLMARKERCUR_N_ALVEOLAR_PNEUMOCYTE_TYPE_I																														
CELLMARKERCUR_N_ALVEOLAR_PNEUMOCYTE_TYPE_…																														
CELLMARKERCUR_N_AMACRINE_CELL																														
CELLMARKERCUR_N_AMNIOTIC_FLUID_STEM_CELL																														
CELLMARKERCUR_N_ANTERIOR_CELL																														
CELLMARKERCUR_N_ANTERIOR_HINDBRAIN_CELL																														
CELLMARKERCUR_N_ANTI_TUMOR_IMMUNE_CELL																														
CELLMARKERCUR_N_ANTIGEN_PRESENTATION_CANC…																														
CELLMARKERCUR_N_ASTROCYTE																														
CELLMARKERCUR_N_ATRIAL_CELL																														
CELLMARKERCUR_N_ATRIOVENTRICULAR_CANAL_CE…																														
CELLMARKERCUR_N_ATYPICAL_MEMORY_B_CELL																														
CELLMARKERCUR_N_B_2_CELL																														
CELLMARKERCUR_N_B1_CELL																														
CELLMARKERCUR_N_B10_REGULATORY_B_CELL																														
CELLMARKERCUR_N_BASAL_CELL																														
CELLMARKERCUR_N_BASAL_EPITHELIAL_CELL																														
CELLMARKERCUR_N_BASAL_KERATINOCYTE																														
CELLMARKERCUR_N_BASAL_LIKE_CELL																														
CELLMARKERCUR_N_BASAL_PROGENITOR_CELL																														
CELLMARKERCUR_N_BASOPHIL																														
CELLMARKERCUR_N_BILIARY_CELL																														
CELLMARKERCUR_N_BONE_MARROW_STEM_CELL																														
CELLMARKERCUR_N_BONE_SPECIFIC_CELL																														
CELLMARKERCUR_N_BRAIN_CELL																														
CELLMARKERCUR_N_CANCER_CELL	STAT2	JAK1	OAS1	TGM2	CD19	CD1C	KLRD1	IGHG1	CD3G	VCAN	KRT5	CD274	AREG	UBXN10	SYNE3	CYFIP2	HDAC11	HOXC10	APOBE…	ANO1	KIT	SATB2	OLFM4	ATP1B3	CX3CR1	SLC31…	PODXL	SLC17…	PTPRO	NDUFA…
CELLMARKERCUR_N_CANCER_STEM_LIKE_CELL																														
CELLMARKERCUR_N_CAPILLARY_CELL																														
CELLMARKERCUR_N_CAR_T_CELL																														
CELLMARKERCUR_N_CARDIAC_CELL																														
CELLMARKERCUR_N_CARDIOMYOCYTE																														
CELLMARKERCUR_N_CARDIOSPHERE_DERIVED_CELL																														
CELLMARKERCUR_N_CARDIOVASCULAR_PROGENITOR…																														
CELLMARKERCUR_N_CAUDAL_CORTEX_CELL																														
CELLMARKERCUR_N_CD14_MONOCYTE																														
CELLMARKERCUR_N_CD16_MONOCYTE																														
CELLMARKERCUR_N_CD4_CD28_T_CELL																														
CELLMARKERCUR_N_CD4_PERIPHERALLY_DERIVED_…																														
CELLMARKERCUR_N_CD4_T_CELL																														
CELLMARKERCUR_N_CD56BRIGHT_NATURAL_KILLER…																														
CELLMARKERCUR_N_CD56DIM_NATURAL_KILLER_CE…																														
CELLMARKERCUR_N_CD8_T_CELL																														
CELLMARKERCUR_N_CENTRAL_MEMORY_T_CELL																														
CELLMARKERCUR_N_CENTRAL_MEMORY_T_HELPER_1…																														
CELLMARKERCUR_N_CENTRAL_NERVOUS_SYSTEM_ST…																														
CELLMARKERCUR_N_CHANDELIER_CELL																														
CELLMARKERCUR_N_CHIEF_CELL																														
CELLMARKERCUR_N_CHONDROCYTE																														
CELLMARKERCUR_N_CHROMAFFIN_CELL																														
CELLMARKERCUR_N_CIRCULATING_ANGIOGENIC_CE…																														
CELLMARKERCUR_N_CIRCULATING_FETAL_CELL																														
CELLMARKERCUR_N_CIRCULATING_NATURAL_KILLE…																														
CELLMARKERCUR_N_CIRCULATING_PRECURSOR_CELL																														
CELLMARKERCUR_N_CIRCULATING_PROGENITOR_CE…																														
CELLMARKERCUR_N_CIRCULATING_T_FOLLICULAR_…																														
CELLMARKERCUR_N_CIRCULATING_TUMOR_CELL																														
CELLMARKERCUR_N_CLASS_SWITCHED_PLASMABLAST																														
CELLMARKERCUR_N_CLASSICAL_B_CELL																														
CELLMARKERCUR_N_CLASSICAL_CANCER_ASSOCIAT…																														
CELLMARKERCUR_N_CLASSICAL_MONOCYTE																														
CELLMARKERCUR_N_CLAUDIN_LOW_CELL																														
CELLMARKERCUR_N_COLLECTING_DUCTAL_CELL																														
CELLMARKERCUR_N_COLONCYTE																														
CELLMARKERCUR_N_COLORECTAL_STEM_CELL																														
CELLMARKERCUR_N_COLUMNAR_EPITHELIAL_CELL																														
CELLMARKERCUR_N_COMMON_LYMPHOID_PROGENITO…																														
CELLMARKERCUR_N_COMMON_MYELOID_PROGENITOR…																														
CELLMARKERCUR_N_COMPLEMENT_SECRETING_CANC…																														
CELLMARKERCUR_N_CONE_PHOTORECEPTOR_CELL																														
CELLMARKERCUR_N_CONNECTING_TUBULAR_CELL																														
CELLMARKERCUR_N_CONVENTIONAL_CD4_T_CELL																														
CELLMARKERCUR_N_CORNEAL_ENDOTHELIAL_CELL																														
CELLMARKERCUR_N_CORNEAL_EPITHELIAL_CELL																														
CELLMARKERCUR_N_CORNEOCYTE_LIKE_CELL																														
CELLMARKERCUR_N_CORTICAL_CELL																														
CELLMARKERCUR_N_CORTICAL_STROMAL_CELL																														
CELLMARKERCUR_N_CORTICOTROPH_TUMOR_CELL																														
CELLMARKERCUR_N_CRANIAL_NEURAL_CREST_CELL																														
CELLMARKERCUR_N_CRYPT_CELL																														
CELLMARKERCUR_N_CRYPT_STEM_CELL																														
CELLMARKERCUR_N_CYCLING_B_CELL																														
CELLMARKERCUR_N_CYCLING_EPITHELIAL_CELL																														
CELLMARKERCUR_N_CYTOTOXIC_CD8_T_CELL																														
CELLMARKERCUR_N_CYTOTOXIC_DOUBLE_POSITIVE…																														
CELLMARKERCUR_N_CYTOTOXIC_T_CELL																														

Table S5: Cell state marker gene list

CELLMARKERCAN_ACINAR_CELLS																														
CELLMARKERCUR_N_CYTOTOXIC_T_CELL_2																														
CELLMARKERCUR_N_DAMAGE_ASSOCIATED_TRANSIE…																														
CELLMARKERCUR_N_DARK_ZONE_CELL																														
CELLMARKERCUR_N_DEDIFFERENTIATION_ADIPOCY…																														
CELLMARKERCUR_N_DEEP_LAYER_NEURON																														
CELLMARKERCUR_N_DEFINITIVE_ENDODERM_CELL																														
CELLMARKERCUR_N_DELTA_CELL																														
CELLMARKERCUR_N_DEMYELINATING_MICROGLIAL_…																														
CELLMARKERCUR_N_DENDRITIC_CELL_2A																														
CELLMARKERCUR_N_DENDRITIC_CELL_LINEAGE																														
CELLMARKERCUR_N_DENTAL_PULP_CELL																														
CELLMARKERCUR_N_DERMAL_CELL																														
CELLMARKERCUR_N_DEUTEROSOMAL_CELL																														
CELLMARKERCUR_N_DIFFUSE_GASTRIC_CANCER_CE…																														
CELLMARKERCUR_N_DISTAL_CELL																														
CELLMARKERCUR_N_DISTAL_STEM_CELL																														
CELLMARKERCUR_N_DISTAL_TUBULAR_CELL																														
CELLMARKERCUR_N_DIVIDING_CELL																														
CELLMARKERCUR_N_DIVIDING_PLASMA_B_CELL																														
CELLMARKERCUR_N_DIVIDING_T_CELL																														
CELLMARKERCUR_N_DOPAMINERGIC_NEURON																														
CELLMARKERCUR_N_DORSAL_ANTERIOR_HINDBRAIN…																														
CELLMARKERCUR_N_DORSAL_LATERAL_GANGLIONIC…																														
CELLMARKERCUR_N_DORSAL_MEDIAL_GANGLIONIC_…																														
CELLMARKERCUR_N_DOUBLE_NEGATIVE_B_CELL																														
CELLMARKERCUR_N_DOUBLE_NEGATIVE_T_CELL																														
CELLMARKERCUR_N_DOUBLE_POSITIVE_T_CELL																														
CELLMARKERCUR_N_DOUBLE_POSITIVE_TREG_CELL																														
CELLMARKERCUR_N_EARLY_HEMANGIOBLAST																														
CELLMARKERCUR_N_EARLY_PROXIMAL_TUBULE_CELL																														
CELLMARKERCUR_N_EARLY_RESPONSE_SECRETORY_…																														
CELLMARKERCUR_N_EFFECTOR_CHONDROCYTE																														
CELLMARKERCUR_N_EFFECTOR_MEMORY_T_CELL																														
CELLMARKERCUR_N_EFFECTOR_NATURAL_KILLER_C…																														
CELLMARKERCUR_N_ELONGATING_CELL																														
CELLMARKERCUR_N_EMBRYONIC_CELL																														
CELLMARKERCUR_N_EMBRYONIC_STEM_CELL																														
CELLMARKERCUR_N_ENDOCARDIAL_CELL																														
CELLMARKERCUR_N_ENDOCRINE_PROGENITOR_CELL																														
CELLMARKERCUR_N_ENDODERM_PROGENITOR_CELL																														
CELLMARKERCUR_N_ENDODERMAL_CELL																														
CELLMARKERCUR_N_ENDOMETRIAL_STROMAL_CELL																														
CELLMARKERCUR_N_ENDOMETRIAL_STROMAL_STEM_…																														
CELLMARKERCUR_N_ENDOTHELIAL_PRECURSOR_CELL																														
CELLMARKERCUR_N_ENSHEATHING_GLIAL_CELL																														
CELLMARKERCUR_N_ENTERIC_NEURON																														
CELLMARKERCUR_N_EOSINOPHIL																														
CELLMARKERCUR_N_EPENDYMAL_CELL																														
CELLMARKERCUR_N_EPIBLAST_CELL																														
CELLMARKERCUR_N_EPICARDIAL_CELL																														
CELLMARKERCUR_N_EPICARDIUM_DERIVED_CELL																														
CELLMARKERCUR_N_EPIDERMAL_CELL																														
CELLMARKERCUR_N_EPIDERMAL_STEM_CELL																														
CELLMARKERCUR_N_EPITHELIAL_CELL	KRT12…	ALB	FOXJ1	SFTPC	CLDN18	SFTPB	NKX2-1	DAPL1	PRR4	LTF	TUBB1	CCDC78	TP73	ASCL3	SPDEF	CALCA	PDPN	TP63	SCGB3…	SFTPD	DCLK1	ASCL1	CFTR	CLIC5	AGER	FOXI1	CCKAR	ETV5	LPO	AGTR1
CELLMARKERCUR_N_EPITHELIAL_DUCTAL_CELL																														
CELLMARKERCUR_N_EPITHELIAL_MESENCHYMAL_CE…																														
CELLMARKERCUR_N_EPITHELIOID_MACROPHAGE																														
CELLMARKERCUR_N_EPSILON_CELL																														
CELLMARKERCUR_N_ERYTHROBLAST																														
CELLMARKERCUR_N_ERYTHROID_CELL																														
CELLMARKERCUR_N_ERYTHROID_PROGENITOR_CELL																														
CELLMARKERCUR_N_ESOPHAGEAL_CELL																														
CELLMARKERCUR_N_ESOPHAGEAL_PROGENITOR_CELL																														
CELLMARKERCUR_N_EXCITATORY_NEURON																														
CELLMARKERCUR_N_EXHAUSTED_DOUBLE_POSITIVE…																														
CELLMARKERCUR_N_EXTRAVILLOUS_TROPHOBLAST																														
CELLMARKERCUR_N_F_PERICYTE																														
CELLMARKERCUR_N_FETAL_MYOBLAST																														
CELLMARKERCUR_N_FIBROBLAST_LIKE_CELL																														
CELLMARKERCUR_N_FIBROCHONDROCYTE																														
CELLMARKERCUR_N_FIBROCHONDROCYTE_PROGENIT…																														
CELLMARKERCUR_N_FOAM_CELL																														
CELLMARKERCUR_N_FOLLICULAR_B_CELL																														
CELLMARKERCUR_N_FOLLICULAR_CELL																														
CELLMARKERCUR_N_FOLLICULAR_DENDRITIC_CELL																														
CELLMARKERCUR_N_FOREBRAIN_CELL																														
CELLMARKERCUR_N_GABAERGIC_NEURON																														
CELLMARKERCUR_N_GANGLION_CELL																														
CELLMARKERCUR_N_GASTRIC_NEUROENDOCRINE_CE…																														
CELLMARKERCUR_N_GASTRIC_STEM_CELL																														
CELLMARKERCUR_N_GERM_CELL																														
CELLMARKERCUR_N_GERMINAL_CENTER_B_CELL																														
CELLMARKERCUR_N_GERMINAL_CENTER_CELL																														
CELLMARKERCUR_N_GERMINAL_CENTRE_B_CELL																														
CELLMARKERCUR_N_GLIAL_CELL																														
CELLMARKERCUR_N_GLIOMA_STEM_CELL																														
CELLMARKERCUR_N_GLOBOSE_BASAL_CELL																														
CELLMARKERCUR_N_GLOMERULAR_PARIETAL_EPITH…																														
CELLMARKERCUR_N_GLUTAMATERGIC_NEURON																														
CELLMARKERCUR_N_GOBLET_CELL																														
CELLMARKERCUR_N_GONADOTROPE																														
CELLMARKERCUR_N_GONADOTROPH_CELL																														
CELLMARKERCUR_N_GRANULOCYTE																														
CELLMARKERCUR_N_GRANULOCYTE_MONOCYTE_PROG…																														
CELLMARKERCUR_N_GRANULOCYTIC_UNK																														
CELLMARKERCUR_N_GRANULOSA_CELL																														
CELLMARKERCUR_N_HAEMATOPOIETIC_CELL																														
CELLMARKERCUR_N_HAIR_FOLLICLE_CELL																														
CELLMARKERCUR_N_HEMANGIOBLAST																														
CELLMARKERCUR_N_HEMATOPOIETIC_CELL																														
CELLMARKERCUR_N_HEMATOPOIETIC_PRECURSOR_C…																														
CELLMARKERCUR_N_HEMATOPOIETIC_PROGENITOR_…																														
CELLMARKERCUR_N_HEPATIC_CELL																														
CELLMARKERCUR_N_HEPATIC_PROGENITOR_CELL																														

CELLMARKERCAN_ACINAR_CELLS																														
CELLMARKERCUR_N_HEPATIC_STELLATE_CELL																														
CELLMARKERCUR_N_HEPATOBLAST																														
CELLMARKERCUR_N_HEPATOCYTE																														
CELLMARKERCUR_N_HISTIOCYTE																														
CELLMARKERCUR_N_HMSC_TREATED_B_CELL																														
CELLMARKERCUR_N_HOFBAUER_CELL																														
CELLMARKERCUR_N_HOMEOSTATIC_CHONDROCYTE																														
CELLMARKERCUR_N_HOMEOSTATIC_MICROGLIAL_CE…																														
CELLMARKERCUR_N_HORIZONTAL_BASAL_CELL																														
CELLMARKERCUR_N_HORIZONTAL_CELL																														
CELLMARKERCUR_N_HYPOBLAST_CELL																														
CELLMARKERCUR_N_IDIOPATHIC_PULMONARY_FIBR…																														
CELLMARKERCUR_N_IGA_MEMORY_B_CELL																														
CELLMARKERCUR_N_IGG_MEMORY_B_CELL																														
CELLMARKERCUR_N_IGM_MEMORY_B_CELL																														
CELLMARKERCUR_N_IGM_ONLY_B_CELL																														
CELLMARKERCUR_N_IL7R_T_HELPER_CELL																														
CELLMARKERCUR_N_IMMATURE_ERYTHROID_CELL																														
CELLMARKERCUR_N_IMMATURE_MYELOID_CELL																														
CELLMARKERCUR_N_IMMATURE_NEUTROPHIL																														
CELLMARKERCUR_N_IMMATURE_TRANSITIONAL_B_C…																														
CELLMARKERCUR_N_IMMUNE_CELL																														
CELLMARKERCUR_N_INDUCED_PLURIPOTENT_STEM_…																														
CELLMARKERCUR_N_INFILTRATING_MACROPHAGE																														
CELLMARKERCUR_N_INFLAMMATORY_CANCER_ASSOC…																														
CELLMARKERCUR_N_INFLAMMATORY_DENDRITIC_CE…																														
CELLMARKERCUR_N_INFLAMMATORY_MONOCYTE																														
CELLMARKERCUR_N_INHIBITORY_NEURON																														
CELLMARKERCUR_N_INNATE_LYMPHOID_CELL																														
CELLMARKERCUR_N_INNER_CELL_MASS_CELL																														
CELLMARKERCUR_N_INNER_RADIAL_GLIAL_CELL																														
CELLMARKERCUR_N_INTER_MEDIATE_PROGENITOR_…																														
CELLMARKERCUR_N_INTERCALATED_CELL																														
CELLMARKERCUR_N_INTERFERON_RESPONSIVE_SEC…																														
CELLMARKERCUR_N_INTERMEDIATE_MONOCYTE																														
CELLMARKERCUR_N_INTERMEDIATE_MONOCYTE_CELL																														
CELLMARKERCUR_N_INTERSTITIAL_MACROPHAGE																														
CELLMARKERCUR_N_INTERSTITIAL_PROGENITOR_C…																														
CELLMARKERCUR_N_INTESTINAL_EPITHELIAL_STE…																														
CELLMARKERCUR_N_INTESTINAL_GASTRIC_CANCER…																														
CELLMARKERCUR_N_INTESTINAL_METAPLASIA_CELL																														
CELLMARKERCUR_N_INTRAEPITHELIAL_LYMPHOCYT…																														
CELLMARKERCUR_N_IONOCYTE																														
CELLMARKERCUR_N_IRIS_PUTTATIVE_STEM_CELL																														
CELLMARKERCUR_N_KUPFFER_CELL																														
CELLMARKERCUR_N_LACTOTROPH_CELL																														
CELLMARKERCUR_N_LANGERHANS_CELL																														
CELLMARKERCUR_N_LEUKOCYTE																														
CELLMARKERCUR_N_LIMBAL_EPITHELIAL_STEM_CE…																														
CELLMARKERCUR_N_LIMBAL_STEM_CELL																														
CELLMARKERCUR_N_LIPOFIBROBLAST																														
CELLMARKERCUR_N_LIVER_PROGENITOR_CELL																														
CELLMARKERCUR_N_LIVER_STEM_CELL																														
CELLMARKERCUR_N_LMMLEYDIG_CELL																														
CELLMARKERCUR_N_LONOCYTE																														
CELLMARKERCUR_N_LOOP_OF_HENLE_CELL																														
CELLMARKERCUR_N_LOOP_PROGENITOR_CELL																														
CELLMARKERCUR_N_LOW_DENSITY_NEUTROPHIL																														
CELLMARKERCUR_N_LUMINAL_PROGENITOR_CELL																														
CELLMARKERCUR_N_LUNG_CELL																														
CELLMARKERCUR_N_LYMPH_NODE_CELL																														
CELLMARKERCUR_N_LYMPHATIC_CELL																														
CELLMARKERCUR_N_LYMPHATIC_ENDOTHELIAL_CELL																														
CELLMARKERCUR_N_LYMPHOBLASTOID_CELL																														
CELLMARKERCUR_N_LYMPHOCYTE																														
CELLMARKERCUR_N_LYMPHOID_CELL																														
CELLMARKERCUR_N_LYMPHOID_PROGENITOR_CELL																														
CELLMARKERCUR_N_LYMPHOID_STRUCTURE_CELL																														
CELLMARKERCUR_N_M_PERICYTE																														
CELLMARKERCUR_N_M1_MACROPHAGE																														
CELLMARKERCUR_N_M1_MICROGLIAL_CELL																														
CELLMARKERCUR_N_M2_MACROPHAGE																														
CELLMARKERCUR_N_MACROGLIAL_CELL																														
CELLMARKERCUR_N_MALIGNANT_CELL																														
CELLMARKERCUR_N_MALIGNANT_PLASMA_CELL																														
CELLMARKERCUR_N_MARGINAL_ASSOCIATED_MACRO…																														
CELLMARKERCUR_N_MAST_CELL																														
CELLMARKERCUR_N_MATURE_ASTROCYTE																														
CELLMARKERCUR_N_MATURE_B_CELL																														
CELLMARKERCUR_N_MATURE_DENDRITIC_CELL																														
CELLMARKERCUR_N_MATURE_ENDOTHELIAL_CELL																														
CELLMARKERCUR_N_MATURE_LUMINAL_CELL																														
CELLMARKERCUR_N_MATURE_NEURON																														
CELLMARKERCUR_N_MATURE_NEURONAL																														
CELLMARKERCUR_N_MATURE_OLIGODENDROCYTE																														
CELLMARKERCUR_N_MATURE_OSTEOBLAST																														
CELLMARKERCUR_N_MATURE_PODOCYTE																														
CELLMARKERCUR_N_MEDIAL_AND_DISTAL_SEGMENT…																														
CELLMARKERCUR_N_MEDIAL_GANGLIONIC_EMINENC…																														
CELLMARKERCUR_N_MEGAKARYOCYTE																														
CELLMARKERCUR_N_MEGAKARYOCYTE_ERYTHROID_C…																														
CELLMARKERCUR_N_MEGAKARYOCYTE_ERYTHROID_P…																														
CELLMARKERCUR_N_MEGAKARYOCYTE_PROGENITOR_…																														
CELLMARKERCUR_N_MELANOMA_CELL																														
CELLMARKERCUR_N_MEMORY_B_CELL																														
CELLMARKERCUR_N_MEMORY_B_CELL_PRECURSOR																														
CELLMARKERCUR_N_MEMORY_CD27_B_CELL																														
CELLMARKERCUR_N_MEMORY_CD4_T_CELL																														
CELLMARKERCUR_N_MEMORY_DOUBLE_POSITIVE_T_…																														
CELLMARKERCUR_N_MEMORY_REGULATORY_T_CELL																														
CELLMARKERCUR_N_MEMORY_T_CELL																														
CELLMARKERCUR_N_MERKEL_CELL																														
CELLMARKERCUR_N_MESENCHYMAL_CELL																														
CELLMARKERCUR_N_MESENCHYMAL_EPITHELIAL_TR…																														

Table S5: Cell state marker gene list

CELLMARKERCAN_ACINAR_CELLS																														
CELLMARKERCUR_N_MESENCHYMAL_GLIOBLASTOMA_…																														
CELLMARKERCUR_N_MESENCHYMAL_STEM_CELL																														
CELLMARKERCUR_N_MESENCHYMAL_STROMAL_CELL																														
CELLMARKERCUR_N_MESENDODERMAL_CELL																														
CELLMARKERCUR_N_MESODERMAL_EMBRYONIC_STEM…																														
CELLMARKERCUR_N_MESODERMAL_PROGENITOR_CELL																														
CELLMARKERCUR_N_METAPLASTIC_STEM_CELL																														
CELLMARKERCUR_N_MEYLOID_CELL																														
CELLMARKERCUR_N_MICROGLIAL_CELL	RPS9	CTSZ	RPS21	CD34	RFTN1	RPS26	RPS27A	CH25H	S100A4	EMP2	SECTM1	ASTN1	ARSA	ATP6V…	PKM	PHETA1	SYNDI…	H2BP1	PGK1	HEXA	RPL13A	RPS28	ECM1	ATP1B3	CHST2	RPL37A	MAMDC2	MPEG1	SLC11…	CXCL14
CELLMARKERCUR_N_MICROVILLAR_CELL																														
CELLMARKERCUR_N_MIDBRAIN_DOPAMINERGIC_NEU…																														
CELLMARKERCUR_N_MONO_MACROPHAGE																														
CELLMARKERCUR_N_MONOCYTE_DERIVED_CELL																														
CELLMARKERCUR_N_MONOCYTE_DERIVED_MYELOID_…																														
CELLMARKERCUR_N_MONOCYTE_LINEAGE																														
CELLMARKERCUR_N_MONOCYTE_PRECURSOR																														
CELLMARKERCUR_N_MONOCYTE_PROGENITOR_CELL																														
CELLMARKERCUR_N_MUELLER_GLIA_CELL																														
CELLMARKERCUR_N_MULTIPOTENT_MESENCHYMAL_S…																														
CELLMARKERCUR_N_MULTIPOTENT_RETINAL_PROGE…																														
CELLMARKERCUR_N_MULTIPOTENT_STEM_CELL																														
CELLMARKERCUR_N_MULTIPOTENT_STROMAL_CELL																														
CELLMARKERCUR_N_MUSCLE_CELL																														
CELLMARKERCUR_N_MUSCLE_DERIVED_CELL																														
CELLMARKERCUR_N_MUSCLE_SATELLITE_CELL																														
CELLMARKERCUR_N_MYELOBLAST																														
CELLMARKERCUR_N_MYELOID_DENDRITIC_CELL																														
CELLMARKERCUR_N_MYELOID_DENDRITIC_CELL_1																														
CELLMARKERCUR_N_MYELOID_DERIVED_CELL																														
CELLMARKERCUR_N_MYELOID_DERIVED_SUPPRESSO…																														
CELLMARKERCUR_N_MYELOID_PROGENITOR_CELL																														
CELLMARKERCUR_N_MYO_EPITHELIAL_CELL																														
CELLMARKERCUR_N_MYOBLAST																														
CELLMARKERCUR_N_MYOCYTE																														
CELLMARKERCUR_N_MYOFIBROBLAST																														
CELLMARKERCUR_N_MYOGENIC_ENDOTHELIAL_CELL																														
CELLMARKERCUR_N_MYOGENIC_PROGENITOR_CELL																														
CELLMARKERCUR_N_MYOID_CELL																														
CELLMARKERCUR_N_NAIVE_B_CELL																														
CELLMARKERCUR_N_NAIVE_CD8_T_CELL																														
CELLMARKERCUR_N_NAIVE_DOUBLE_POSITIVE_T_C…																														
CELLMARKERCUR_N_NAIVE_PROGENITOR_CELL																														
CELLMARKERCUR_N_NAIVE_T_CELL																														
CELLMARKERCUR_N_NASCENT_NEPHRON_CELL																														
CELLMARKERCUR_N_NATURAL_KILLER_CD56_BRIGH…																														
CELLMARKERCUR_N_NATURAL_KILLER_CD56_DIM_C…																														
CELLMARKERCUR_N_NEGATIVE_MESENCHYMAL_STEM…																														
CELLMARKERCUR_N_NEURAL_CELL																														
CELLMARKERCUR_N_NEURAL_CREST_STEM_CELL																														
CELLMARKERCUR_N_NEURAL_CREST_STEMLIKE_CELL																														
CELLMARKERCUR_N_NEURAL_PRECURSOR_CELL																														
CELLMARKERCUR_N_NEURAL_STEM_CELL																														
CELLMARKERCUR_N_NEUROECTODERM_CELL																														
CELLMARKERCUR_N_NEURONAL_INTERMEDIATE_PRO…																														
CELLMARKERCUR_N_NEURONAL_PROGENITOR_CELL																														
CELLMARKERCUR_N_NEUTROPHIL_PROGENITOR_CELL																														
CELLMARKERCUR_N_NON_NEURON																														
CELLMARKERCUR_N_NON_PT_TUBULAR_CELL																														
CELLMARKERCUR_N_NON_RECIRCULATING_TISSUE_…																														
CELLMARKERCUR_N_NONMYELINATING_SCHWANN_CE…																														
CELLMARKERCUR_N_NOTOCHORD_CELL																														
CELLMARKERCUR_N_NUCLEATED_CELL																														
CELLMARKERCUR_N_NUCLEUS_PULPOSUS_PROGENIT…																														
CELLMARKERCUR_N_OLIGODENDROCYTE																														
CELLMARKERCUR_N_OSTEOBLAST																														
CELLMARKERCUR_N_OUTER_RADIAL_GLIA																														
CELLMARKERCUR_N_OVALBUMIN_SPECIFIC_REGULA…																														
CELLMARKERCUR_N_OVARIAN_CELL																														
CELLMARKERCUR_N_OVARIAN_STROMAL_CELL																														
CELLMARKERCUR_N_P53_LIKE_CELL																														
CELLMARKERCUR_N_PAN_B_CELL																														
CELLMARKERCUR_N_PAN_CANCER_ASSOCIATED_FIB…																														
CELLMARKERCUR_N_PAN_MACROPHAGE																														
CELLMARKERCUR_N_PAN_MONOCYTE																														
CELLMARKERCUR_N_PAN_NEURONAL_CELL																														
CELLMARKERCUR_N_PAN_TROPHECTODERM_CELL																														
CELLMARKERCUR_N_PANCREATIC_ENDODERM_CELL																														
CELLMARKERCUR_N_PANCREATIC_POLYPEPTIDE_CE…																														
CELLMARKERCUR_N_PANCREATIC_PROGENITOR_CELL																														
CELLMARKERCUR_N_PANCREATIC_STEM_CELL																														
CELLMARKERCUR_N_PANETH_CELL																														
CELLMARKERCUR_N_PANNEURON																														
CELLMARKERCUR_N_PANVASCULAR_CELL																														
CELLMARKERCUR_N_PAPILLARY_FIBROBLAST																														
CELLMARKERCUR_N_PARIETAL_CELL																														
CELLMARKERCUR_N_PARVALBUMIN_INTERNEURON																														
CELLMARKERCUR_N_PATELET																														
CELLMARKERCUR_N_PATHOGENIC_T_PERIPHERAL_H…																														
CELLMARKERCUR_N_PELVIC_EPITHELIAL_CELL																														
CELLMARKERCUR_N_PERICYTE																														
CELLMARKERCUR_N_PERIODONTAL_LIGAMENT_STEM…																														
CELLMARKERCUR_N_PERIOSTEUM_DERIVED_PROGEN…																														
CELLMARKERCUR_N_PERITUBULAR_MYOID_CELL																														
CELLMARKERCUR_N_PERIVASCULAR_LIKE_CELL																														
CELLMARKERCUR_N_PHAGOCYTE																														
CELLMARKERCUR_N_PHOTORECEPTOR_CELL																														
CELLMARKERCUR_N_PIT_CELL																														
CELLMARKERCUR_N_PIT_MUCOUS_CELL																														
CELLMARKERCUR_N_PITUITARY_STEM_CELL																														
CELLMARKERCUR_N_PLACENTAL_TISSUE_CELL																														
CELLMARKERCUR_N_PLACODE_CELL																														
CELLMARKERCUR_N_PLASMABLAST_B_CELL																														
CELLMARKERCUR_N_PLATELET																														
CELLMARKERCUR_N_PLURIPOTENT_STEM_CELL																														

Table S5: Cell state marker gene list

CELLMARKERCAN_ACINAR_CELLS																														
CELLMARKERCUR_N_PODOCYTE_PRECURSOR																														
CELLMARKERCUR_N_PORTAL_FIBROBLAST																														
CELLMARKERCUR_N_POSTERIOR_CELL																														
CELLMARKERCUR_N_PRE_B_CELL																														
CELLMARKERCUR_N_PRE_CONVENTIONAL_DENDRITI…																														
CELLMARKERCUR_N_PRE_DENDRITIC_CELL																														
CELLMARKERCUR_N_PRECURSOR_CELL																														
CELLMARKERCUR_N_PREHYPERTROPHIC_CHONDROCY…																														
CELLMARKERCUR_N_PRESOMITE_CELL																														
CELLMARKERCUR_N_PRETUBULAR_AGGREGATE_CELL																														
CELLMARKERCUR_N_PRIMITIVE_ENDODERM_CELL																														
CELLMARKERCUR_N_PRINCIPAL_CELL																														
CELLMARKERCUR_N_PRO_B_CELL																														
CELLMARKERCUR_N_PRO_INFLAMMATORY_MACROPHA…																														
CELLMARKERCUR_N_PRO_NEUTROPHIL																														
CELLMARKERCUR_N_PROLIFERATING_CELL																														
CELLMARKERCUR_N_PROLIFERATIVE_CHONDROCYTE																														
CELLMARKERCUR_N_PROLIFERATIVE_DUCTAL_CELL																														
CELLMARKERCUR_N_PROLIFERATIVE_NEURAL_PROG…																														
CELLMARKERCUR_N_PRONEURAL_GLIOBLASTOMA_CE…																														
CELLMARKERCUR_N_PROSPERMATOGONIA																														
CELLMARKERCUR_N_PROSTATE_CELL																														
CELLMARKERCUR_N_PROXIMAL_CELL																														
CELLMARKERCUR_N_PROXIMAL_SEGMENT_CELL																														
CELLMARKERCUR_N_PROXIMAL_STEM_CELL																														
CELLMARKERCUR_N_PROXIMAL_TUBULE_CELL																														
CELLMARKERCUR_N_PULMONARY_NEUROENDOCRINE_…																														
CELLMARKERCUR_N_PYRAMIDAL_CELL																														
CELLMARKERCUR_N_QUIESCENT_T_STEM_CELL																														
CELLMARKERCUR_N_REGULATORY_B_CELL																														
CELLMARKERCUR_N_REGULATORY_INNATE_LYMPHOI…																														
CELLMARKERCUR_N_RENAL_TUBULAR_CELL																														
CELLMARKERCUR_N_RESIDENT_MEMORY_T_CELL																														
CELLMARKERCUR_N_RESPIRATORY_HORIZONTAL_BA…																														
CELLMARKERCUR_N_RESPIRATORY_SECRETORY_CELL																														
CELLMARKERCUR_N_RESPONDER_T_CELL																														
CELLMARKERCUR_N_RESPONDING_CONVENTIONAL_T…																														
CELLMARKERCUR_N_RESTING_MEMORY_B_CELL																														
CELLMARKERCUR_N_RESTING_NAIVE_B_CELL																														
CELLMARKERCUR_N_RESTING_REGULATORY_T_CELL																														
CELLMARKERCUR_N_RETICULAR_FIBROBLAST																														
CELLMARKERCUR_N_RETINA_CELL																														
CELLMARKERCUR_N_ROD_CELL																														
CELLMARKERCUR_N_SATELLITE_CELL																														
CELLMARKERCUR_N_SEPTUM_TRANSVERSUM_MESENC…																														
CELLMARKERCUR_N_SEROUS_CELL																														
CELLMARKERCUR_N_SIDE_POPULATION_CELL																														
CELLMARKERCUR_N_SKELETAL_MUSCLE_CELL																														
CELLMARKERCUR_N_SKELETAL_MUSCLE_STEM_CELL																														
CELLMARKERCUR_N_SMOOTH_MUSCLE_CELL																														
CELLMARKERCUR_N_SMOOTH_MUSCLE_LIKE_CELL																														
CELLMARKERCUR_N_SOMATIC_CELL																														
CELLMARKERCUR_N_SOMATIC_STEM_CELL																														
CELLMARKERCUR_N_SOMATOSTATIN_INTERNEURON																														
CELLMARKERCUR_N_SPECIALIST_ANTIGEN_PRESEN…																														
CELLMARKERCUR_N_SPERMATOGENIC_CELL																														
CELLMARKERCUR_N_SPERMATOGONIAL_CELL																														
CELLMARKERCUR_N_SPERMATOGONIAL_STEM_CELL																														
CELLMARKERCUR_N_SQUAMOUS_DIFFERENTIATION_…																														
CELLMARKERCUR_N_STELLATE_CELL																														
CELLMARKERCUR_N_STEM_CELL_DERIVED_ALPHA_C…																														
CELLMARKERCUR_N_STEM_CELL_DERIVED_BETA_CE…																														
CELLMARKERCUR_N_STEM_CELL_DERIVED_ENDOTHE…																														
CELLMARKERCUR_N_STEM_LIKE_CD8_T_CELL																														
CELLMARKERCUR_N_STROMAL_CELL																														
CELLMARKERCUR_N_SUBPALLIUM_CELL																														
CELLMARKERCUR_N_SUBPLATE_CELL																														
CELLMARKERCUR_N_SUPPRESSIVE_MONOCYTE																														
CELLMARKERCUR_N_SUPPRESSOR_T_CELL																														
CELLMARKERCUR_N_SUPRABASAL_CELL																														
CELLMARKERCUR_N_SURFACE_ECTODERM_CELL																														
CELLMARKERCUR_N_SUSTENTACULAR_CELL																														
CELLMARKERCUR_N_SYMPATHOADRENAL_CELL																														
CELLMARKERCUR_N_SYNCYTIOTROPHOBLAST																														
CELLMARKERCUR_N_SYNTHETIC_SMOOTH_MUSCLE_C…																														
CELLMARKERCUR_N_T_CELL_LARGE_GRANULAR_LYM…																														
CELLMARKERCUR_N_T_CELL_LINEAGE																														
CELLMARKERCUR_N_T_HELPER_CELL																														
CELLMARKERCUR_N_T_MEMORY_STEM_CELL																														
CELLMARKERCUR_N_T2_MZP_REGULATORY_B_CELL																														
CELLMARKERCUR_N_TENDON_CELL																														
CELLMARKERCUR_N_TERMINAL_DIFFERENTIATED_B…																														
CELLMARKERCUR_N_THALAMIC_CELL																														
CELLMARKERCUR_N_THECA_INTERNA_CELL																														
CELLMARKERCUR_N_THYMIC_EPITHELIAL_CELL																														
CELLMARKERCUR_N_TISSUE_LIKE_MEMORY_B_CELL																														
CELLMARKERCUR_N_TISSUE_RESIDENT_CELL																														
CELLMARKERCUR_N_TISSUE_RESIDENT_EFFECTOR_…																														
CELLMARKERCUR_N_TISSUE_RESIDENT_MACROPHAGE																														
CELLMARKERCUR_N_TISSUE_RESIDENT_MEMORY_LI…																														
CELLMARKERCUR_N_TOLDC_INDUCED_REGULATORY_…																														
CELLMARKERCUR_N_TONSIL_CELL																														
CELLMARKERCUR_N_TRABECULAR_MESHWORK_CELL																														
CELLMARKERCUR_N_TRANSITIONAL_B_CELL																														
CELLMARKERCUR_N_TRANSITIONAL_DENDRITIC_CE…																														
CELLMARKERCUR_N_TRUNCATED_RADIAL_GLIAL_CE…																														
CELLMARKERCUR_N_TUBULAR_CELL																														
CELLMARKERCUR_N_TUMOR_ASSOCIATED_MICROGLI…																														
CELLMARKERCUR_N_TUMOR_ENDOTHELIAL_CELL																														
CELLMARKERCUR_N_TUMOR_EPITHELIAL_CELL																														
CELLMARKERCUR_N_TUMOR_INFILTRATING_REGULA…																														
CELLMARKERCUR_N_TUMOR_INFILTRATING_T_CELL																														
CELLMARKERCUR_N_TUMOR_INITIATING_CELL																														
CELLMARKERCUR_N_TUMOR_REGULATORY_T_CELL																														

Table S5: Cell state marker gene list

CELLMARKERCAN_ACINAR_CELLS																														
CELLMARKERCUR_N_UMBRELLA_CELL																														
CELLMARKERCUR_N_UNRESTRICTED_SOMATIC_STEM…																														
CELLMARKERCUR_N_UNSWITCHED_PLASMABLAST																														
CELLMARKERCUR_N_URETERIC_BUD_CELL																														
CELLMARKERCUR_N_UROTHELIAL_CELL																														
CELLMARKERCUR_N_UROTHELIUM_CELL																														
CELLMARKERCUR_N_VASCULATURE_CELL																														
CELLMARKERCUR_N_VEIN_CELL																														
CELLMARKERCUR_N_VENOUS_CELL																														
CELLMARKERCUR_N_VENTRAL_LATERAL_GANGLIONI…																														
CELLMARKERCUR_N_VENTRAL_MEDIAL_GANGLIONIC…																														
CELLMARKERCUR_N_VENTRICULAR_CELL																														
CELLMARKERCUR_N_YOUNG_NEURON																														
CELLMARKERCUR_N_ZONA_FASCICULATA_CELL																														
CELLMARKERCUR_N_ZONA_GLOMERULOSA_CELL																														
CELLMARKERCUR_N_ZONA_RETICULARIS_CELL																														
CELLMARKERCUR_N_ZYGOTE																														
CELLMARKERCURNACTIVATEDEFFECTORMEMORYCD4T…																														
CELLMARKERCURNACTIVATEDTISSUERESIDENTMEMO…																														
CELLMARKERCURNACTIVATEDTISSUERESIDENTMEMO…																														
CELLMARKERCURNALPHACELLACELL																														
CELLMARKERCURNALVEOLARTYPEIIATIICELL																														
CELLMARKERCURNATYPICALBCELLABC																														
CELLMARKERCURNAXLCD11CPLASMACYTOIDDENDRIT…																														
CELLMARKERCURNAXLCD123PLASMACYTOIDDENDRIT…																														
CELLMARKERCURNBASALFOREBRAINCHOLINERGICNE…																														
CELLMARKERCURNBRUSHCELLTUFTCELL																														
CELLMARKERCURNCANCERASSOCIATEDFIBROBLASTC…																														
CELLMARKERCURNCAPSULARFIBROBLASTFB2																														
CELLMARKERCURNCCR7DENDRITICCELL																														
CELLMARKERCURNCD106MESENCHYMALSTEMCELL																														
CELLMARKERCURNCD14MACROPHAGE																														
CELLMARKERCURNCD16DENDRITICCELL																														
CELLMARKERCURNCD25LAG3TCELL																														
CELLMARKERCURNCD34GRANULOCYTICPROGENITORC…																														
CELLMARKERCURNCD34HEMATOPOIETICSTEMCELL																														
CELLMARKERCURNCD34PREBCELL																														
CELLMARKERCURNCD34PROGENITORCELL																														
CELLMARKERCURNCD34THYMOCYTE																														
CELLMARKERCURNCD40LGTHELPERCELL																														
CELLMARKERCURNCD4CD25REGULATORYTCELL																														
CELLMARKERCURNCD4CENTRALMEMORYLIKETTCMLIK…																														
CELLMARKERCURNCD4CYTOTOXICT1CELL																														
CELLMARKERCURNCD4CYTOTOXICT2CELL																														
CELLMARKERCURNCD4THELPERCELL																														
CELLMARKERCURNCHRONICALLYACTIVATEDCD4TCELL																														
CELLMARKERCURNCHRONICALLYACTIVATEDCD8TCELL																														
CELLMARKERCURNCLASSSWITCHEDMEMORYBIGGU03B…																														
CELLMARKERCURNCLEC10ADENDRITICCELL																														
CELLMARKERCURNCLEC9ADENDRITICCELL																														
CELLMARKERCURNCONVENTIONALDENDRITICCELL2B…																														
CELLMARKERCURNCONVENTIONALDENDRITICCELL2B…																														
CELLMARKERCURNCORTICALSOMATOSTATINSSTINTE…																														
CELLMARKERCURNCYTOTOXICCD4T2CELL																														
CELLMARKERCURNCYTOTOXICCD4TCELL																														
CELLMARKERCURND1MSNMEDIUMSPINYNEURON																														
CELLMARKERCURND2MSNMEDIUMSPINYNEURON																														
CELLMARKERCURNDISTALTUBULELOOPOFHENLECELL																														
CELLMARKERCURNEARLYCD34MEGAKARYOCYTEERYTH…																														
CELLMARKERCURNEARLYTCELLPROGENITORETP																														
CELLMARKERCURNEFFECTORMEMORYCD4TCELL																														
CELLMARKERCURNEFFECTORREGULATORYTETREGCELL																														
CELLMARKERCURNEFFECTORTTEFFCELL																														
CELLMARKERCURNEXHAUSTEDCD4TCELL																														
CELLMARKERCURNEXHAUSTEDCD8TCELL																														
CELLMARKERCURNEXHAUSTEDCD8TPROGENITORCELL																														
CELLMARKERCURNEXHAUSTEDTTEXCELL																														
CELLMARKERCURNEXTRATELENCEPHALICETCELL																														
CELLMARKERCURNFINALLYHIGHLYEFFECTORTEMRAM…																														
CELLMARKERCURNFOLLICULARREGULATORYTTFRCELL																														
CELLMARKERCURNFOXP3NATURALREGULATORYTTREG…																														
CELLMARKERCURNGAMMADELTAU03B3DTCELL																														
CELLMARKERCURNGERMINALCENTREGCLIKECELL																														
CELLMARKERCURNGLOMERULARENDOTHELIALCELLECG																														
CELLMARKERCURNGRANULOCYTICMYELOIDDERIVEDS…																														
CELLMARKERCURNGRBREGULATORYBCELL																														
CELLMARKERCURNGZMKTCELL																														
CELLMARKERCURNHIGHLYDIFFERENTIATEDEFFECTO…																														
CELLMARKERCURNINDUCEDREGULATORYTTREGCELL																														
CELLMARKERCURNINFLAMMATORYCANCERASSOCIATE…																														
CELLMARKERCURNINNATELYMPHOIDCELLILCS																														
CELLMARKERCURNINTRATHYMICFIBROBLASTFB1																														
CELLMARKERCURNITOCELLHEPATICSTELLATECELL																														
CELLMARKERCURNLYMPHANGIOLEIOMYOMATOSISCEL…																														
CELLMARKERCURNMARGINALZONEMZLIKEBCELL																														
CELLMARKERCURNMEMORYSTEMCELLTSCM																														
CELLMARKERCURNMICROGLIADERIVEDTUMORASSOCI…																														
CELLMARKERCURNMKERYTHCELL																														
CELLMARKERCURNMYOFIBROBLASTICCANCERASSOCI…																														
CELLMARKERCURNNAIVEREGULATORYTTREGCELL																														
CELLMARKERCURNNATURALREGULATORYTTREGCELL																														
CELLMARKERCURNPERIPHERALBLOODMONONUCLEARC…																														
CELLMARKERCURNPITUITARYSTEMCELLPSC																														
CELLMARKERCURNPLASMACYTOIDDENDRITICCELLPDC																														
CELLMARKERCURNPOLYMORPHONUCLEARMYELOIDDER…																														
CELLMARKERCURNPOLYMORPHONUCLEARNEUTROPHIL…																														
CELLMARKERCURNPOSTERIORPRESOMITICMESODERM…																														
CELLMARKERCURNPROLIFERATIVECD4TCELL																														
CELLMARKERCURNPROLIFERATIVECD8TCELL																														
CELLMARKERCURNREDBLOODCELLERYTHROCYTE																														
CELLMARKERCURNREGULATORYBBREGCELL																														
CELLMARKERCURNREGULATORYCD4TCELL																														
CELLMARKERCURNREGULATORYCD8TCELL																														
CELLMARKERCURNREGULATORYTTREGCELL																														

CELLMARKERCAN_ACINAR_CELLS																														
CELLMARKERCURNREGULATORYTTREGCELL																														
CELLMARKERCURNRENALVESICLECOMMASHAPEDBODY…																														
CELLMARKERCURNRESIDENTMEMORYCD8TCELL																														
CELLMARKERCURNREVERTANTMEMORYCD8TTEMRACELL																														
CELLMARKERCURNSCARASSOCIATEDMACROPHAGESAM																														
CELLMARKERCURNSENESCENTCD8TCELL																														
CELLMARKERCURNSTEMPROGENITORCELL																														
CELLMARKERCURNSWITCHEDMEMORYBCELLSWMB																														
CELLMARKERCURNT1TRANSITIONALBCELL																														
CELLMARKERCURNT2TRANSITIONALBCELL																														
CELLMARKERCURNTCELLNATURALKILLERCELL																														
CELLMARKERCURNTERMINALEFFECTORCD8TCELL																														
CELLMARKERCURNTERMINALLYDIFFERENTIATEDCD8…																														
CELLMARKERCURNTFOLLICULARHELPERTFHCELL																														
CELLMARKERCURNTHELPER1TH1CELL																														
CELLMARKERCURNTHELPER1TH1CELL																														
CELLMARKERCURNTHELPER2TH2CELL																														
CELLMARKERCURNTHELPER2TH2CELL																														
CELLMARKERCURNTHELPER9TH9CELL																														
CELLMARKERCURNTHELPERTHCELL																														
CELLMARKERCURNTHYMICEPITHELIALCELLTEC																														
CELLMARKERCURNTISSUERESIDENTMEMORYCD4TCELL																														
CELLMARKERCURNTISSUERESIDENTMEMORYCD8TCELL																														
CELLMARKERCURNTISSUERESIDENTMEMORYTTRMCELL																														
CELLMARKERCURNTPERIPHERALHELPERTPHCELL																														
CELLMARKERCURNTRANSIENTAMPLIFYINGTACELL																														
CELLMARKERCURNTRANSITIONALEXHAUSTEDCD8TCE…																														
CELLMARKERCURNTUMORINFILTRATINGLYMPHOCYTE…																														
CELLMARKERCURNUNSWITCHEDMEMORYBCELLUNSWMB																														
CELLMARKERCURNVASARECTAENDOTHELIALCELLECVR																														
CELLMARKERCURNVONECONOMONEURONVEN																														
CELLMARKERCURNYOLKSACDERIVEDMYELOIDBIASED…																														
CELLMARKEREXP_ACINAR_CELLS																														
CELLMARKEREXP_ADRENERGIC_NEURONS																														
CELLMARKEREXP_AIRWAY_EPITHELIAL_CELLS																														
CELLMARKEREXP_AIRWAY_SMOOTH_MUSCLE_CELLS																														
CELLMARKEREXP_ALPHA_CELLS																														
CELLMARKEREXP_ANTERIOR_PITUITARY_GLAND_CE…																														
CELLMARKEREXP_ASTROCYTES																														
CELLMARKEREXP_B_CELLS																														
CELLMARKEREXP_B_CELLS_MEMORY																														
CELLMARKEREXP_B_CELLS_NAIVE																														
CELLMARKEREXP_BASAL_CELLS																														
CELLMARKEREXP_BASOPHILS																														
CELLMARKEREXP_BERGMANN_GLIA																														
CELLMARKEREXP_BETA_CELLS																														
CELLMARKEREXP_CAJAL_RETZIUS_CELLS																														
CELLMARKEREXP_CHOLANGIOCYTES																														
CELLMARKEREXP_CHOLINERGIC_NEURONS																														
CELLMARKEREXP_CHONDROCYTES																														
CELLMARKEREXP_DELTA_CELLS																														
CELLMARKEREXP_DISTAL_TUBULE_CELLS																														
CELLMARKEREXP_DUCTAL_CELLS																														
CELLMARKEREXP_EMBRYONIC_STEM_CELLS																														
CELLMARKEREXP_ENTERIC_NEURONS																														
CELLMARKEREXP_ENTEROENDOCRINE_CELLS																														
CELLMARKEREXP_EPENDYMAL_CELLS																														
CELLMARKEREXP_EPSILON_CELLS																														
CELLMARKEREXP_ERYTHROID_LIKE_AND_ERYTHROI…																														
CELLMARKEREXP_GAMMA_DELTA_T_CELLS																														
CELLMARKEREXP_GOBLET_CELLS																														
CELLMARKEREXP_HEMANGIOBLASTS																														
CELLMARKEREXP_HEMATOPOIETIC_STEM_CELLS																														
CELLMARKEREXP_HEPATIC_STELLATE_CELLS																														
CELLMARKEREXP_HIS_BUNDLE_CELLS																														
CELLMARKEREXP_IMMATURE_NEURONS																														
CELLMARKEREXP_JUXTAGLOMERULAR_CELLS																														
CELLMARKEREXP_KERATINOCYTES																														
CELLMARKEREXP_KIDNEY_PROGENITOR_CELLS																														
CELLMARKEREXP_KUPFFER_CELLS																														
CELLMARKEREXP_LEYDIG_CELLS																														
CELLMARKEREXP_LOOP_OF_HENLE_CELLS																														
CELLMARKEREXP_LUMINAL_EPITHELIAL_CELLS																														
CELLMARKEREXP_MAMMARY_EPITHELIAL_CELLS																														
CELLMARKEREXP_MEGAKARYOCYTES																														
CELLMARKEREXP_MELANOCYTES																														
CELLMARKEREXP_MESANGIAL_CELLS																														
CELLMARKEREXP_MESOTHELIAL_CELLS																														
CELLMARKEREXP_MICROFOLD_CELLS																														
CELLMARKEREXP_MICROGLIA																														
CELLMARKEREXP_MONOCYTES																														
CELLMARKEREXP_MULLER_CELLS																														
CELLMARKEREXP_MYOBLASTS																														
CELLMARKEREXP_MYOCYTES																														
CELLMARKEREXP_MYOFIBROBLASTS																														
CELLMARKEREXP_NEUROBLASTS																														
CELLMARKEREXP_NEUTROPHILS																														
CELLMARKEREXP_NK_CELLS																														
CELLMARKEREXP_NORADRENERGIC_NEURONS																														
CELLMARKEREXP_OLIGODENDROCYTES																														
CELLMARKEREXP_OSTEOBLASTS																														
CELLMARKEREXP_OSTEOCLAST_PRECURSOR_CELLS																														
CELLMARKEREXP_OSTEOCLASTS																														
CELLMARKEREXP_OSTEOCYTES																														
CELLMARKEREXP_PANETH_CELLS																														
CELLMARKEREXP_PARATHYROID_CHIEF_CELLS																														
CELLMARKEREXP_PERICYTES																														
CELLMARKEREXP_PHOTORECEPTOR_CELLS																														
CELLMARKEREXP_PLASMA_CELLS																														
CELLMARKEREXP_PLASMACYTOID_DENDRITIC_CELLS																														
CELLMARKEREXP_PODOCYTES																														
CELLMARKEREXP_PRINCIPAL_CELLS																														
CELLMARKEREXP_PROXIMAL_TUBULE_CELLS																														
CELLMARKEREXP_PULMONARY_ALVEOLAR_TYPE_I_C…																														

CELLMARKERCAN_ACINAR_CELLS																														
CELLMARKEREXP_PULMONARY_ALVEOLAR_TYPE_II_…																														
CELLMARKEREXP_PURKINJE_FIBER_CELLS																														
CELLMARKEREXP_PURKINJE_NEURONS																														
CELLMARKEREXP_PYRAMIDAL_CELLS																														
CELLMARKEREXP_RETICULOCYTES																														
CELLMARKEREXP_RETINAL_GANGLION_CELLS																														
CELLMARKEREXP_SALIVARY_MUCOUS_CELLS																														
CELLMARKEREXP_SATELLITE_CELLS																														
CELLMARKEREXP_SCHWANN_CELLS																														
CELLMARKEREXP_SEROTONERGIC_NEURONS																														
CELLMARKEREXP_SERTOLI_CELLS																														
CELLMARKEREXP_SMOOTH_MUSCLE_CELLS																														
CELLMARKEREXP_SPERMATOZOA																														
CELLMARKEREXP_STROMAL_CELLS																														
CELLMARKEREXP_T_CELLS																														
CELLMARKEREXP_T_CELLS_NAIVE																														
CELLMARKEREXP_T_CYTOTOXIC_CELLS																														
CELLMARKEREXP_T_HELPER_CELLS																														
CELLMARKEREXP_T_MEMORY_CELLS																														
CELLMARKEREXP_TRICHOCYTES																														
CELLMARKEREXP_TRIGEMINAL_NEURONS																														
CELLMARKEREXP_TROPHOBLAST_CELLS																														
CELLMARKEREXP_TROPHOBLAST_PROGENITOR_CELLS																														
CELLMARKEREXP_TUFT_CELLS																														
CELLMARKEREXP_VASCULAR_SMOOTH_MUSCLE_CELLS																														
CELLMARKEREXPENDOTHELIALCELLSAORTA																														
CELLMARKEREXPNEURALSTEMPRECURSORCELLS																														
CELLMARKERMSIGDB_AIZARANI_LIVER_C12_NK_NK…																														
CELLMARKERMSIGDB_AIZARANI_LIVER_C22_RESID…																														
CELLMARKERMSIGDB_AIZARANI_LIVER_C28_NK_NK…																														
CELLMARKERMSIGDB_AIZARANI_LIVER_C29_MVECS…	RPGR	ABLIM1	JUND	RIPOR1	HBEGF	KLF2	CD151	SWAP70	GPM6A	APP	SELE	ARID5B	GPRC5A	STOM	NRN1	B4GAL…	MIDN	IFITM2	LRRC32	BGN	FGF2	GNAI2	FLOT1	HYAL2	RFX2	MIR22…	SYNPO	ADGRL4	NOLC1	MAFF
CELLMARKERMSIGDB_AIZARANI_LIVER_C30_HEPAT…	LRCH3	XPR1	POR	CYP3A4	ADPRH…	MIGA1	CEP89	GMEB1	FYB2	TMEM1…	ZBTB25	METTL…	SLC25…	TAT	SLC25…	TMEM8…	CMC2	LACTB…	FMO5	FOXO3	ABCA1	MAVS	AIG1	HELB	SGSM1	INE2	ASPG	NBPF11	FBXO31	KIZ-A…
CELLMARKERMSIGDB_AIZARANI_LIVER_C8_RESIDE…																														
CELLMARKERMSIGDB_AIZARANI_LIVER_C9_LSECS_1	EHD3	HIPK2	TSPAN7	MMRN1	IL4R	YBX3	MYCT1	TCIM	KCTD20	TIMP2	PRSS23	MEF2C	MAP7D3	PXN	CCL14	TMEM44	PHACT…	HPCAL1	NPR1	SMTN	CDH13	IL6ST	F2R	MID1	ASAP1	FXYD6	ZNF451	FGF23	CDC42…	ECM1
CELLMARKERMSIGDB_BUSSLINGER_DUODENAL_BCHE…																														
CELLMARKERMSIGDB_BUSSLINGER_DUODENAL_DIFF…	RPS28	NOP56	ELAPO…	NOP58	PCNA	PRKDC	TRIM28	ATP5PB	DHFR	NUCKS1	COX6C	ATP5M…	SNRPF	EIF3L	HSP90…	TMPO	RPL18	RPL27	CCT2	CCT5	RPS15A	DDAH1	APP	C9orf…	CCT6A	TFRC	SLIRP	HNRNP…	MDH2	CANX
CELLMARKERMSIGDB_BUSSLINGER_DUODENAL_EC_C…																														
CELLMARKERMSIGDB_BUSSLINGER_DUODENAL_IMMU…	RGS10	PITPN…	RPL17	SIRPG	IDS	UBASH…	ARPC2	SELPLG	INPP4A	LGALS1	LIMD2	SLAMF7	SAMSN1	MYH9	SSBP4	MAP4K1	ARHGE…	RPS6K…	ZAP70	DOCK10	RHOH	PTPN22	ANKRD…	FOSB	BIN2	PPP1R…	NFATC2	ST3GA…	ITK	SYNRG
CELLMARKERMSIGDB_BUSSLINGER_DUODENAL_STEM…	COQ8A	CCND1	CHDH	RPS19	EPCAM	NOP58	SNRPB	GNL3	IVNS1…	HMGCS2	RPL3	NOP56	DMBT1	DDX21	HMGN1	RCC2	SELEN…	REPIN1	SLC38…	RPS25	RPL37	DARS1	CD46	RPL10	DSTN	RACK1	NPM1	EIF2S3	SERBP1	DKC1
CELLMARKERMSIGDB_BUSSLINGER_DUODENAL_TUFT…																														
CELLMARKERMSIGDB_BUSSLINGER_ESOPHAGEAL_EA…																														
CELLMARKERMSIGDB_BUSSLINGER_ESOPHAGEAL_PR…																														
CELLMARKERMSIGDB_BUSSLINGER_GASTRIC_CHIEF…																														
CELLMARKERMSIGDB_BUSSLINGER_GASTRIC_G_CEL…																														
CELLMARKERMSIGDB_BUSSLINGER_GASTRIC_ISTHM…	CTSE	ABLIM1	ASAP2	CFL1	SEC61G	HSP90…	SCD	MAL2	SERPI…	CYP4F…	ELOVL6	EIF4G1	ALDH3…	ENO1	ME1	CMBL	SLC16…	SEC61…	HPGD	EBP	PALLD	CKS2	PRC1	ARPC1A	GKN2	HNRNPM	SDC1	EIF4E2	MTDH	MLEC
CELLMARKERMSIGDB_BUSSLINGER_GASTRIC_NECK_…																														
CELLMARKERMSIGDB_BUSSLINGER_GASTRIC_OXYNT…																														
CELLMARKERMSIGDB_BUSSLINGER_GASTRIC_PREZY…																														
CELLMARKERMSIGDB_BUSSLINGER_GASTRIC_REG3A…																														
CELLMARKERMSIGDB_CUI_DEVELOPING_HEART_5TH…																														
CELLMARKERMSIGDB_CUI_DEVELOPING_HEART_5TH…																														
CELLMARKERMSIGDB_CUI_DEVELOPING_HEART_C1_…																														
CELLMARKERMSIGDB_CUI_DEVELOPING_HEART_C2_…																														
CELLMARKERMSIGDB_CUI_DEVELOPING_HEART_COM…																														
CELLMARKERMSIGDB_CUI_DEVELOPING_HEART_COR…																														
CELLMARKERMSIGDB_CUI_DEVELOPING_HEART_END…																														
CELLMARKERMSIGDB_CUI_DEVELOPING_HEART_LEF…																														
CELLMARKERMSIGDB_CUI_DEVELOPING_HEART_RIG…																														
CELLMARKERMSIGDB_CUI_DEVELOPING_HEART_SMO…																														
CELLMARKERMSIGDB_DESCARTES_FETAL_ADRENAL_…																														
CELLMARKERMSIGDB_DESCARTES_FETAL_CEREBELL…																														
CELLMARKERMSIGDB_DESCARTES_FETAL_CEREBELL…																														
CELLMARKERMSIGDB_DESCARTES_FETAL_CEREBELL…	MITF	DOCK4	PIK3CD	LYN	FTL	CREG1	SMAP2	GADD4…	PLVAP	PLEKH…	ANXA4	TAL1	ARHGA…	BMP2K	CAPZB	APOE	GM2A	ATG7	SNX29	ENTPD1	PLXDC2	TM6SF1	SKAP2	DIAPH2	LPCAT2	PLCG2	BTG2	ADAMT…	TBC1D…	FAM10…
CELLMARKERMSIGDB_DESCARTES_FETAL_CEREBELL…																														
CELLMARKERMSIGDB_DESCARTES_FETAL_CEREBELL…																														
CELLMARKERMSIGDB_DESCARTES_FETAL_CEREBELL…																														
CELLMARKERMSIGDB_DESCARTES_FETAL_CEREBELL…	RHOC	PCDH12	SLC3A2	HOPX	EVA1B	BCAM	ARHGA…	MELTF	DLC1	ABCG2	SERPI…	GIMAP6	SPARC	SLC39…	GRAP	HBG2	RGCC	HYAL2	SHC1	DENND…	ATP8B1	HBA2	CDH6	TSC22…	FAM43A	EGFL7	COL18…	MYO10	HBB	SMG1P3
CELLMARKERMSIGDB_DESCARTES_FETAL_CEREBRUM…																														
CELLMARKERMSIGDB_DESCARTES_FETAL_CEREBRUM…																														
CELLMARKERMSIGDB_DESCARTES_FETAL_CEREBRUM…	BANK1	MAP1A	FAM21…	SEC14…	SASS6	QKILA	STX11	NUP62…	OR2W3	LINC0…	PLPP7	RAP2B	LINC0…	E2F1	LY6G6F	CENPT	KEL	LGALSL	TFAP2E	MPST	CDKN2…	ZNF346	CFLAR…	SYS1-…	TUBA4A	CLEC1…	STK11	SELP	HACD4	SFT2D3
CELLMARKERMSIGDB_DESCARTES_FETAL_CEREBRUM…	CEP29…	GGTA1	LINC0…	SCIN	ENSG0…	ARHGA…	SIGLE…	SLC15…	TMEM1…	HCLS1	TASL	CEACA…	RAB39A	OSBPL…	CCR1	PPT1	SERPI…	NINJ1	TRAF3…	LINC0…	CD37	EXPH5	LINC0…	GPR183	LINC0…	GYPC	C5AR1	PDE4D…	TAGAP	LINC0…
CELLMARKERMSIGDB_DESCARTES_FETAL_CEREBRUM…																														
CELLMARKERMSIGDB_DESCARTES_FETAL_CEREBRUM…																														
CELLMARKERMSIGDB_DESCARTES_FETAL_CEREBRUM…	RHOB	KCNJ2	CDH5	LRRC55	VEGFC	SH2D3C	NIBAN2	MCAM	UACA	LMCD1	CD109	ZNF503	GIMAP4	SLC16…	CD93	CCDC3	AHNAK	SLFN5	PRKCH…	SMG1P3	HBA2	TTN	RGS3	PRKCH	CD320	NOTCH3	KDR	GIMAP1	PLOD1	SAMM50
CELLMARKERMSIGDB_DESCARTES_FETAL_EYE_ASTR…																														
CELLMARKERMSIGDB_DESCARTES_FETAL_EYE_GANG…																														
CELLMARKERMSIGDB_DESCARTES_FETAL_EYE_LENS…																														
CELLMARKERMSIGDB_DESCARTES_FETAL_EYE_PHOT…																														
CELLMARKERMSIGDB_DESCARTES_FETAL_EYE_RETI…																														
CELLMARKERMSIGDB_DESCARTES_FETAL_EYE_SMOO…																														
CELLMARKERMSIGDB_DESCARTES_FETAL_EYE_STRO…																														
CELLMARKERMSIGDB_DESCARTES_FETAL_EYE_VASC…																														
CELLMARKERMSIGDB_DESCARTES_FETAL_HEART_EL…																														
CELLMARKERMSIGDB_DESCARTES_FETAL_HEART_EN…																														
CELLMARKERMSIGDB_DESCARTES_FETAL_HEART_LY…																														
CELLMARKERMSIGDB_DESCARTES_FETAL_HEART_SC…																														
CELLMARKERMSIGDB_DESCARTES_FETAL_HEART_SM…																														
CELLMARKERMSIGDB_DESCARTES_FETAL_HEART_ST…																														
CELLMARKERMSIGDB_DESCARTES_FETAL_HEART_VI…																														
CELLMARKERMSIGDB_DESCARTES_FETAL_INTESTIN…																														
CELLMARKERMSIGDB_DESCARTES_FETAL_INTESTIN…																														
CELLMARKERMSIGDB_DESCARTES_FETAL_INTESTIN…																														
CELLMARKERMSIGDB_DESCARTES_FETAL_INTESTIN…																														
CELLMARKERMSIGDB_DESCARTES_FETAL_INTESTIN…																														
CELLMARKERMSIGDB_DESCARTES_FETAL_INTESTIN…																														
CELLMARKERMSIGDB_DESCARTES_FETAL_KIDNEY_M…																														
CELLMARKERMSIGDB_DESCARTES_FETAL_KIDNEY_U…	EPN3	EMX1	TMEM3…	ATP12A	PLS3-…	KRT5	LAMB3	OVCH2	CYP4B1	PLA2G…	PTGER1	COLCA1	SHH	FAM3D	TRPV6	POU5F1	ESRP2	TMEM2…	ALDH3…	ENSG0…	BSPRY	RHBG	LINC0…	PKP3	ACSM6	POU3F4	CLPSL1	AKR1C2	DCAF1…	CAPN13
CELLMARKERMSIGDB_DESCARTES_FETAL_LIVER_ER…																														
CELLMARKERMSIGDB_DESCARTES_FETAL_LIVER_HE…																														
CELLMARKERMSIGDB_DESCARTES_FETAL_LIVER_HE…	ANPEP	ABCC2	PRODH2	RGN	TCEA3	VSNL1	CNGA1	GPC5-…	ADORA…	C2orf…	CAPN12	RIDA	MAOB	AMDHD1	ABCG5	HRG	SPP2	FXYD2	AKR1D1	TDO2	C8B	ENPP1	TMPRS…	SGIP1	SLC22…	ACOT12	AKR1C3	SERPI…	SLC15…	SLC22…
CELLMARKERMSIGDB_DESCARTES_FETAL_LIVER_ME…	FXYD7	GALNT9	SPEG	CRYAB	CRACDL	AXIN2	CDH22	RASSF7	TMEM1…	PDZK1…	LY6H	ARL4D	IL17RE	GABRB2	ENTPD2	GSDME	FNDC1	MEDAG	FREM2	POF1B	PLLP	ANKRD…	MAP3K…	NTRK3	MMP24	EDIL3…	CA11	LINC0…	B4GAL…	POU6F2
CELLMARKERMSIGDB_DESCARTES_FETAL_LUNG_BRO…																														
CELLMARKERMSIGDB_DESCARTES_FETAL_LUNG_CIL…	C22or…	AK9	CFAP1…	FRMPD2	C6	CCDC40	AK7	CFAP47	MNS1	STMND1	STOML3	TSPAN…	SMIM6	ARMC2	SLC22…	DNAAF4	CFAP65	NPHP1	CSPP1	TRAF3…	TTC21A	DRC7	DNALI1	CROCC2	RP1	DYNLR…	LRRC51	NME5	RAB36	TTLL9
CELLMARKERMSIGDB_DESCARTES_FETAL_LUNG_LYM…																														

Table S5: Cell state marker gene list

CELLMARKERCAN_ACINAR_CELLS																														
CELLMARKERMSIGDB_DESCARTES_FETAL_LUNG_NEU…																														
CELLMARKERMSIGDB_DESCARTES_FETAL_LUNG_STR…																														
CELLMARKERMSIGDB_DESCARTES_FETAL_LUNG_VAS…																														
CELLMARKERMSIGDB_DESCARTES_FETAL_MUSCLE_L…																														
CELLMARKERMSIGDB_DESCARTES_FETAL_MUSCLE_S…																														
CELLMARKERMSIGDB_DESCARTES_FETAL_MUSCLE_S…																														
CELLMARKERMSIGDB_DESCARTES_FETAL_MUSCLE_V…																														
CELLMARKERMSIGDB_DESCARTES_FETAL_PANCREAS…																														
CELLMARKERMSIGDB_DESCARTES_FETAL_PANCREAS…																														
CELLMARKERMSIGDB_DESCARTES_FETAL_PANCREAS…																														
CELLMARKERMSIGDB_DESCARTES_FETAL_PANCREAS…																														
CELLMARKERMSIGDB_DESCARTES_FETAL_PANCREAS…																														
CELLMARKERMSIGDB_DESCARTES_FETAL_PANCREAS…																														
CELLMARKERMSIGDB_DESCARTES_FETAL_PANCREAS…																														
CELLMARKERMSIGDB_DESCARTES_FETAL_PLACENTA…																														
CELLMARKERMSIGDB_DESCARTES_FETAL_PLACENTA…																														
CELLMARKERMSIGDB_DESCARTES_FETAL_PLACENTA…																														
CELLMARKERMSIGDB_DESCARTES_FETAL_PLACENTA…																														
CELLMARKERMSIGDB_DESCARTES_FETAL_PLACENTA…																														
CELLMARKERMSIGDB_DESCARTES_FETAL_SPLEEN_E…																														
CELLMARKERMSIGDB_DESCARTES_FETAL_SPLEEN_S…																														
CELLMARKERMSIGDB_DESCARTES_FETAL_SPLEEN_V…																														
CELLMARKERMSIGDB_DESCARTES_FETAL_STOMACH_…	RPGR	CFAP2…	CIBAR2	CAPSL	RP1	LDLRA…	IQUB	ODAD1	MAPK15	DNAAF3	AGBL2	ENSG0…	CCDC1…	DNAJB…	CERKL	CSRP3…	MORN5	GRM5	LINC0…	SLC9C2	CFAP95	CES4A	CFAP65	CFAP57	CFAP4…	CFAP47	CTXN1	CFAP58	CCDC17	TUBA4B
CELLMARKERMSIGDB_DESCARTES_FETAL_STOMACH_…																														
CELLMARKERMSIGDB_DESCARTES_FETAL_STOMACH_…																														
CELLMARKERMSIGDB_DESCARTES_FETAL_STOMACH_…																														
CELLMARKERMSIGDB_DESCARTES_FETAL_STOMACH_…																														
CELLMARKERMSIGDB_DESCARTES_FETAL_STOMACH_…																														
CELLMARKERMSIGDB_DESCARTES_FETAL_STOMACH_…																														
CELLMARKERMSIGDB_DESCARTES_FETAL_STOMACH_…																														
CELLMARKERMSIGDB_DESCARTES_FETAL_STOMACH_…																														
CELLMARKERMSIGDB_DESCARTES_FETAL_STOMACH_…																														
CELLMARKERMSIGDB_DESCARTES_FETAL_STOMACH_…																														
CELLMARKERMSIGDB_DESCARTES_FETAL_STOMACH_…																														
CELLMARKERMSIGDB_DESCARTES_FETAL_THYMUS_T…																														
CELLMARKERMSIGDB_DESCARTES_MAIN_FETAL_ACI…																														
CELLMARKERMSIGDB_DESCARTES_MAIN_FETAL_ADR…																														
CELLMARKERMSIGDB_DESCARTES_MAIN_FETAL_AST…																														
CELLMARKERMSIGDB_DESCARTES_MAIN_FETAL_BIP…																														
CELLMARKERMSIGDB_DESCARTES_MAIN_FETAL_BRO…																														
CELLMARKERMSIGDB_DESCARTES_MAIN_FETAL_CAR…																														
CELLMARKERMSIGDB_DESCARTES_MAIN_FETAL_CIL…	ZBBX	WDR49	LRRC34	TMEM2…	CCDC3…	LINC0…	CLDN16	DYNLT…	TM4SF…	IQCG	LMLN	CFAP99	CCDC96	CC2D2A	PACRGL	DTHD1	WDR19	LINC0…	CHRNA9	NSUN7	SPATA…	UGT2B…	FAM47E	CFAP2…	CDS1	RN7SK…	ARHGE…	SGMS2	PRSS12	CEP17…
CELLMARKERMSIGDB_DESCARTES_MAIN_FETAL_DUC…																														
CELLMARKERMSIGDB_DESCARTES_MAIN_FETAL_END…																														
CELLMARKERMSIGDB_DESCARTES_MAIN_FETAL_ENS…																														
CELLMARKERMSIGDB_DESCARTES_MAIN_FETAL_EPI…																														
CELLMARKERMSIGDB_DESCARTES_MAIN_FETAL_GAN…																														
CELLMARKERMSIGDB_DESCARTES_MAIN_FETAL_GOB…																														
CELLMARKERMSIGDB_DESCARTES_MAIN_FETAL_HEM…																														
CELLMARKERMSIGDB_DESCARTES_MAIN_FETAL_HOR…																														
CELLMARKERMSIGDB_DESCARTES_MAIN_FETAL_INH…																														
CELLMARKERMSIGDB_DESCARTES_MAIN_FETAL_INH…																														
CELLMARKERMSIGDB_DESCARTES_MAIN_FETAL_INT…																														
CELLMARKERMSIGDB_DESCARTES_MAIN_FETAL_ISL…																														
CELLMARKERMSIGDB_DESCARTES_MAIN_FETAL_LYM…																														
CELLMARKERMSIGDB_DESCARTES_MAIN_FETAL_MES…																														
CELLMARKERMSIGDB_DESCARTES_MAIN_FETAL_MET…																														
CELLMARKERMSIGDB_DESCARTES_MAIN_FETAL_MYE…																														
CELLMARKERMSIGDB_DESCARTES_MAIN_FETAL_NEU…																														
CELLMARKERMSIGDB_DESCARTES_MAIN_FETAL_OLI…																														
CELLMARKERMSIGDB_DESCARTES_MAIN_FETAL_PDE…																														
CELLMARKERMSIGDB_DESCARTES_MAIN_FETAL_PDE…	SNTB1	EXOSC4	MFSD3	COMMD5	RNU6-…	TTC39B	EXOSC3	FRG1JP	MAMDC2	RPL24…	CARNM…	UBQLN…	TUT7	ZNF484	STX17	ZBTB26	GOLGA1	SLC25…	ADAMT…	ITGB1…	FAM21…	ECD	PPP3C…	ZNF50…	ADIRF…	LINC0…	CCNJ	RPS15…	LINC0…	WDR11
CELLMARKERMSIGDB_DESCARTES_MAIN_FETAL_RET…																														
CELLMARKERMSIGDB_DESCARTES_MAIN_FETAL_SAT…																														
CELLMARKERMSIGDB_DESCARTES_MAIN_FETAL_SCH…																														
CELLMARKERMSIGDB_DESCARTES_MAIN_FETAL_SLC…	TRMT1…	SPTLC1	LINC0…	INVS	SHOC1	TMEM2…	TPT1P9	PSMB7	ZBTB43	ASB6	TOR1A	SURF6	SURF2	TUBBP5	CDC123	CACNB2	PRTFD…	CREM	TMEM26	RN7SL…	LINC0…	CEP55	SLC25…	BORCS…	RN7SL…	NSMCE…	SCART1	PNPLA2	RPL21…	TMEM9…
CELLMARKERMSIGDB_DESCARTES_MAIN_FETAL_SMO…																														
CELLMARKERMSIGDB_DESCARTES_MAIN_FETAL_STE…																														
CELLMARKERMSIGDB_DESCARTES_MAIN_FETAL_SYM…																														
CELLMARKERMSIGDB_DESCARTES_MAIN_FETAL_UNI…																														
CELLMARKERMSIGDB_DESCARTES_MAIN_FETAL_URE…																														
CELLMARKERMSIGDB_DESCARTES_MAIN_FETAL_VAS…																														
CELLMARKERMSIGDB_DESCARTES_MAIN_FETAL_VIS…																														
CELLMARKERMSIGDB_DURANTE_ADULT_OLFACTORY_…																														
CELLMARKERMSIGDB_DURANTE_ADULT_OLFACTORY_…																														
CELLMARKERMSIGDB_DURANTE_ADULT_OLFACTORY_…																														
CELLMARKERMSIGDB_DURANTE_ADULT_OLFACTORY_…																														
CELLMARKERMSIGDB_DURANTE_ADULT_OLFACTORY_…																														
CELLMARKERMSIGDB_DURANTE_ADULT_OLFACTORY_…																														
CELLMARKERMSIGDB_DURANTE_ADULT_OLFACTORY_…																														
CELLMARKERMSIGDB_DURANTE_ADULT_OLFACTORY_…																														
CELLMARKERMSIGDB_DURANTE_ADULT_OLFACTORY_…																														
CELLMARKERMSIGDB_DURANTE_ADULT_OLFACTORY_…																														
CELLMARKERMSIGDB_DURANTE_ADULT_OLFACTORY_…																														
CELLMARKERMSIGDB_DURANTE_ADULT_OLFACTORY_…																														
CELLMARKERMSIGDB_DURANTE_ADULT_OLFACTORY_…																														
CELLMARKERMSIGDB_DURANTE_ADULT_OLFACTORY_…																														
CELLMARKERMSIGDB_DURANTE_ADULT_OLFACTORY_…																														
CELLMARKERMSIGDB_DURANTE_ADULT_OLFACTORY_…																														
CELLMARKERMSIGDB_FAN_EMBRYONIC_CTX_ASTROC…																														
CELLMARKERMSIGDB_FAN_EMBRYONIC_CTX_BIG_GR…	PCOLCE	HAPLN3	CDH6	TIMP1	EGFL7	VWA1	SH2D3C	CYTH3	UACA	LGALS1	WWTR1	PODXL	PELO	PPIC	RAPGE…	SLC39…	GMDS	RGCC	SLC7A1	HSPA1…	VCL	PAPSS2	ANXA1	COX7A1	UTRN	GALNT…	MYO1B	RHOC	LEF1	IFI44L
CELLMARKERMSIGDB_FAN_EMBRYONIC_CTX_BIG_GR…	CLSTN2	COX6A1	LY6H	FBLN1	PDE4B	CDH18	NDUFB8	CDH4	CD47	COX7A1	DMRTA2	EGFLAM	ACOT7	GAP43	VSTM4	WNT4	NOL3	CLCF1	SYP	SEZ6L	ATP5F…	ITGA11	ACSL4	DIABLO	MXRA5	ATP5MK	GPI	CDIP1	TXNL4A	NPB
CELLMARKERMSIGDB_FAN_EMBRYONIC_CTX_BIG_GR…																														
CELLMARKERMSIGDB_FAN_EMBRYONIC_CTX_BIG_GR…	ITM2B	SYNGR2	DOCK2	CASP1	MPEG1	PARVG	SKAP2	IER2	LIPA	IL6ST	CEBPD	DHRS3	TAGAP	HSPB1	MED12L	GRN	CMTM7	CNPY3	IL10RA	BLNK	JUN	DNASE2	CD83	LHFPL2	NPC2	IFI30	ZFP36…	CTSS	S100A…	HHEX
CELLMARKERMSIGDB_FAN_EMBRYONIC_CTX_BRAIN_…	ESYT2	PLLP	ACTN4	PROCR	CDH2	ZDHHC9	ZNF366	FKBP9	PRKCH	ERG	JUP	PPFIB…	MAGED2	MAP4K2	POMP	MT2A	NPIPB3	GPCPD1	TUSC3	MYH10	CD151	COX7A1	MT1F	OCLN	PTP4A3	BCL6B	HSPA1…	TPM4	WFS1	DSP
CELLMARKERMSIGDB_FAN_EMBRYONIC_CTX_BRAIN_…	ANXA2	TCEAL9	TNKS1…	LDHB	ATP1B2	TMEM7…	AK1	GNG12	SNRK	HES4	SH2D3C	SYTL2	AEBP1	GPM6B	VIM	FBLN1	PLS3	EFEMP2	GUCY1…	ZIC1	CCDC1…	KANK2	TAGLN	CFH	ADGRD1	UNC5B	TSPAN…	P2RY14	FARP1	PELO
CELLMARKERMSIGDB_FAN_EMBRYONIC_CTX_EX_1_E…																														
CELLMARKERMSIGDB_FAN_EMBRYONIC_CTX_IN_2_I…																														
CELLMARKERMSIGDB_FAN_EMBRYONIC_CTX_IN_3_I…																														
CELLMARKERMSIGDB_FAN_EMBRYONIC_CTX_IN_5_I…																														
CELLMARKERMSIGDB_FAN_EMBRYONIC_CTX_IN_6_I…																														
CELLMARKERMSIGDB_FAN_EMBRYONIC_CTX_MICROG…																														
CELLMARKERMSIGDB_FAN_EMBRYONIC_CTX_OLIG	TPD52	ROGDI	CLN8	GAB1	LIPA	EVI2A	LINC0…	FNBP1	MEGF9	CRYAB	CHADL	RHOU	ST18	ATG4C	PLOD3	BMP8A	GAS7	FGFR2	SERIN…	PLXNB3	H2BC21	THEMI…	HHIP	ZNF365	CDR2L	DNM2	SLC22…	RTN4	ELL2	FBXO32
CELLMARKERMSIGDB_FAN_EMBRYONIC_CTX_OPC																														

CELLMARKERCAN_ACINAR_CELLS																														
CELLMARKERMSIGDB_FAN_OVARY_CL1_GPRC5A_TNF…	NOP53	OGN	IARS1	RSL1D1	TBC1D…	OAT	HSPE1	DIMT1	PA2G4	CACYBP	RNF149	EIF1AX	NME1	COL1A1	PAWR	RARRE…	TCEAL1	IMPDH2	PLSCR1	RPSA	KDM6B	RPL9	UBB	CHMP2B	FKBP4	TUBB6	MAP2K3	UBE2D3	RBM25	HNRNP…
CELLMARKERMSIGDB_FAN_OVARY_CL11_MURAL_GRA…	JMJD6	WIPF3	TBC1D4	HOPX	CCT2	USO1	PPARG	GREB1	CRNDE	TJP1	MGARP	CHSY1	FILIP…	ITGAV	ZBTB10	MT-ND1	PPA1	DIPK2A	VAPA	ARIH1	HOMER1	BAMBI	VDAC2	FDFT1	RASL1…	PPHLN1	SERPI…	GATM	ITSN1	YAP1
CELLMARKERMSIGDB_FAN_OVARY_CL13_MONOCYTE_…	HLA-B	NAMPT	PKIB	JAML	TXN	PRR13	WTAP	ATP2B1	PLEKH…	NEAT1	STK4	LRRFI…	ARRB2	LGALS3	CLIC2	RALA	ARFGA…	GRB2	CTSH	BRI3	PSME2	ARHGD…	EVI2B	HCLS1	BTG1	BLVRB	CORO1A	FGR	PYCARD	TMSB4X
CELLMARKERMSIGDB_FAN_OVARY_CL14_MATURE_SM…	RHOA	PALLD	ACTN1	COX6A1	ISG15	AXL	ANAPC…	CAP1	UGP2	CKB	TSPO	PFN1	PLP2	PLK2	PPDPF	UQCR11	CLIC4	PSME1	NR2F2	ACTN4	FIS1	GEM	RABAC1	COX17	PTMS	NDUFB1	SYPL1	VAMP2	IRF1	TXNIP
CELLMARKERMSIGDB_FAN_OVARY_CL15_SMALL_ANT…	NME2	SRSF2	STIP1	CHIC2	CD164	TEAD1	CCT4	SYNE2	ZFAND6	MAOA	MAPRE1	HMGCR	UBE2C	PHLDA1	PHF6	NXF1	ARHGA…	RCN2	SLC16…	UBE2D1	NDFIP1	ARPC2	IRS1	FGD4	BCAT1	RAD23B	RAB5A	RHEB	LRAT	SCYL2
CELLMARKERMSIGDB_FAN_OVARY_CL18_B_LYMPHOC…	BZW1	CHORD…	TAGLN2	EVI2B	PAG1	ATP5F…	HCLS1	RHOF	CMTM7	MALT1	CAPG	CYTIP	RGS2	LAGE3	YWHAZ	SPCS3	UCP2	FXYD5	ELOA	HSPE1	SEC61G	RALA	CHCHD2	EIF5A	MOB1A	CD44	GNA13	CLEC2D	PTPN2	ALG2
CELLMARKERMSIGDB_FAN_OVARY_CL2_PUTATIVE_E…																														
CELLMARKERMSIGDB_FAN_OVARY_CL5_HEALTHY_SE…	MPG	TPM4	SEPTI…	PBX1	RWDD4	STAT1	ATP5F…	PGRMC1	COL4A1	EGR1	WLS	RSRC2	PPP1R…	C19or…	RAD23B	PLIN3	CHIC2	NEXN	CPED1	SGCE	MYL12B	RPLP0	LMAN1	ADH5	ALDH1…	ATRX	EIF3M	TMEM9	CAND1	DNAJB6
CELLMARKERMSIGDB_FAN_OVARY_CL6_PUTATIVE_E…	SNHG32	ID3	SEPTI…	VKORC1	CPQ	KDELR1	CELF2	SMARC…	TMEM59	ADAMT…	SVIL	ST13	DSTN	LAMP1	P4HB	LEPROT	TAX1B…	FKBP7	OS9	AKR7A2	ATRAID	ING2	ST3GA…	PPIC	C12or…	MYADM	HMGN3	SELEN…	EIF4A2	TMEM2…
CELLMARKERMSIGDB_FAN_OVARY_CL8_MATURE_CUM…	NEDD8	PPP1R…	ME2	PGM1	DUSP23	BCAT1	ATP5PF	SRI	DYNLT1	EPHX1	ATP5MK	SOWAHC	NDUFA1	RALB	UBL5	EPB41…	ARL2BP	VPS35	TCEAL2	ERG28	ATP5M…	MYO10	WIPF3	MT-CO1	OSGIN2	AP1S1	MT-ND3	NSG1	SRP9	COX7A2
CELLMARKERMSIGDB_FAN_OVARY_CL9_PUTATIVE_A…	IRF7	MTUS1	PPP1R…	ADAR	IFITM3	NUPR1	ELOC	IFI6	PLS3	SLC3A2	ISG15	PICALM	ACTN4	SLC2A3	BAZ1A	PLEKH…	LDB2	TAP1	SH3BP5	MEF2C	TNFRS…	RAB5C	HSPB1	BTG1	UBE2J1	MIR44…	COX17	CREBRF	MRPL33	NAMPT
CELLMARKERMSIGDB_GAO_ESOPHAGUS_25W_C1_CIL…	ECT2L	CLXN	EFCAB…	EFHB	EFHC1	EFHC2	ENDOG	ENPP4	ENPP5	EP300…	ERBB4	ERICH2	FABP6	FAM17…	TOGAR…	FAM18…	FAM22…	FAM81B	CIBAR2	FBXO15	FBXO31	FBXO36	FGF14	CEP43	FHAD1	FRMPD2	GALC	GALNS	GAS2L2	GAS8
CELLMARKERMSIGDB_GAO_ESOPHAGUS_25W_C2_KRT…																														
CELLMARKERMSIGDB_GAO_ESOPHAGUS_25W_C3_FGF…																														
CELLMARKERMSIGDB_GAO_LARGE_INTESTINE_24W_…																														
CELLMARKERMSIGDB_GAO_LARGE_INTESTINE_24W_…																														
CELLMARKERMSIGDB_GAO_LARGE_INTESTINE_24W_…	ITGB2	ITGB2…	JMY	RUBCNL	KIAA0…	KLHDC…	KYNU	LAPTM5	LAT2	LCP1	LCP2	LGMN	LILRB1	LILRB2	LILRB4	LILRB5	LINC0…	LIPA	LMO2	ENSG0…	WAKMA…	ENSG0…	LPAR6	LPCAT1	LSP1	LST1	LY86	LYN	LYPD3	LYVE1
CELLMARKERMSIGDB_GAO_LARGE_INTESTINE_24W_…																														
CELLMARKERMSIGDB_GAO_LARGE_INTESTINE_24W_…																														
CELLMARKERMSIGDB_GAO_LARGE_INTESTINE_24W_…																														
CELLMARKERMSIGDB_GAO_LARGE_INTESTINE_ADUL…	MAPK8…	MAPRE3	MARCH…	MARK1	MCOLN3	MLXIPL	MMP2	MS4A8	MSI1	MTCL1	MTURN	NAB2	NBEA	NCALD	NCKAP5	NEFL	NEURO…	NFASC	NINL	NKX2-2	NOL4	NPY1R	NRP1	OGDHL	OR51E2	OSCP1	PALMD	PAM	PAPPA2	PAX4
CELLMARKERMSIGDB_GAO_LARGE_INTESTINE_ADUL…	KRBOX4	KRT18…	LATS2	CEP290	LIG4	LIMS2	LINC0…	LINC0…	LINC0…	LINC0…	LINC0…	LINC0…	LINC0…	LINC0…	LINC0…	LINC0…	LINC0…	CCDC80	LINC0…	LINC0…	LINC0…	LINC0…	LINC0…	LIPE	LMNB1	TMEM3…	VPS33…	LINC0…	ENSG0…	LINC0…
CELLMARKERMSIGDB_GAO_LARGE_INTESTINE_ADUL…																														
CELLMARKERMSIGDB_GAO_LARGE_INTESTINE_ADUL…	IKZF1	IL10RA	IL10R…	IL12R…	IL16	IL23A	IL27RA	IL7R	INPP5D	IRF4	IRF5	ISG20	ITGA4	ITGA8	ITGAL	ITGB2	ITGB7	JSRP1	KCNA3	KCNAB2	KCNN3	KIF19	KLHL5	KLHL6	LAMA5	LAMP3	LAPTM5	LAX1	LCK	LCP1
CELLMARKERMSIGDB_GAO_LARGE_INTESTINE_ADUL…	GASK1B	FAM20C	FAM24B	RIPOR1	FBLN1	FBLN5	FBN1	FBXL7	FENDRR	FERMT2	FGD5	FGFR1	FHL1	FILIP…	FKBP10	FLNA	FLT1	FOXF1	FOXF2	FOXQ1	FRMD6	FRY	FST	FSTL1	FXYD1	FXYD6	GALNT…	GBP4	GDNF	GGT5
CELLMARKERMSIGDB_GAO_SMALL_INTESTINE_24W_…																														
CELLMARKERMSIGDB_GAO_SMALL_INTESTINE_24W_…																														
CELLMARKERMSIGDB_GAO_SMALL_INTESTINE_24W_…																														
CELLMARKERMSIGDB_GAO_SMALL_INTESTINE_24W_…																														
CELLMARKERMSIGDB_GAO_SMALL_INTESTINE_24W_…																														
CELLMARKERMSIGDB_GAO_STOMACH_24W_C1_PROCR…																														
CELLMARKERMSIGDB_GAO_STOMACH_24W_C2_TFF2P…																														
CELLMARKERMSIGDB_GAO_STOMACH_24W_C4_PARIE…																														
CELLMARKERMSIGDB_GAO_STOMACH_24W_C5_PUTAT…																														
CELLMARKERMSIGDB_GAUTAM_EYE_IRIS_CILIARY_…	CCDC1…	FAM24…	MRTO4	CSRP1	RPIA	SMYD5	EIF2S1	BPNT2	ACVR2B	LYL1	TRIM47	RARS1	RILPL2	NAT9	STAG1	CLDN10	POLR3H	NTRAS	MAGEH1	HMGN4	MIR15…	MESD	PRXL2C	APEX1	RBBP5	MBOAT7	ATP6V…	NAPG	MED12	SUPT5H
CELLMARKERMSIGDB_GAUTAM_EYE_IRIS_CILIARY_…																														
CELLMARKERMSIGDB_GAUTAM_EYE_IRIS_CILIARY_…																														
CELLMARKERMSIGDB_GAUTAM_EYE_IRIS_CILIARY_…																														
CELLMARKERMSIGDB_GAUTAM_EYE_IRIS_CILIARY_…																														
CELLMARKERMSIGDB_HAY_BONE_MARROW_CD34_POS…																														
CELLMARKERMSIGDB_HAY_BONE_MARROW_CD34_POS…																														
CELLMARKERMSIGDB_HAY_BONE_MARROW_CD34_POS…																														
CELLMARKERMSIGDB_HAY_BONE_MARROW_CD34_POS…																														
CELLMARKERMSIGDB_HAY_BONE_MARROW_CD8_T_CE…																														
CELLMARKERMSIGDB_HAY_BONE_MARROW_EARLY_ER…																														
CELLMARKERMSIGDB_HAY_BONE_MARROW_EOSINOPH…																														
CELLMARKERMSIGDB_HAY_BONE_MARROW_NAIVE_T_…	RPL36…	EEF1B2	CDC42…	TESPA1	CD96	TNFAI…	LINC0…	GSTK1	ANKRD…	GATA3	BIN1	ZNF331	ITK	SIRPG	LINC0…	TTC39C	SERIN…	PARP1…	SNHG8	STAT3	SUSD3	GPR171	DGKA	SFXN1	ATP6V…	RGCC	TC2N	SNRPN	ARHGE…	SVIP
CELLMARKERMSIGDB_HAY_BONE_MARROW_NEUTROPH…	CASP1	CLEC4D	TPP1	LRP1	PLXDC2	CD44	VSTM1	PTPRE	EREG	IFI27…	UQCR11	RAB3D	RBM47	HACD4	POLR2L	CREG1	AHNAK	FLNA	MIDN	HEXB	GASK1B	CD55	MYH9	PLEC	NFIL3	UBE2D1	ATP6V…	EAF1	ZYX	ATP6V…
CELLMARKERMSIGDB_HAY_BONE_MARROW_NK_CELLS	AOAH	TFAP2…	IER2	MBP	LGALS…	OSBPL5	MRPL10	DENND…	ITGAL	SIGIRR	ARPC4	LIM2	DDIT4	CEMIP2	CISD3	SCLT1	CD320	MVD	RHOF	GNPTAB	HMOX2	SCP2	BNC2	MYL12B	GTF3C1	KIR2D…	IL12R…	OPTN	HAVCR2	FHL3
CELLMARKERMSIGDB_HAY_BONE_MARROW_PRE_DEND…																														
CELLMARKERMSIGDB_HAY_BONE_MARROW_PRO_B	CCNE2	FUS	SPTBN1	WDR76	ARL6I…	E2F8	CIP2A	SMC1A	HNRNPM	ATAD2	SYVN1	HNRNPK	MED30	UBE2I	E2F7	AKR1B1	MZT1	ITGAE	CBX1	DYNLT…	UBE2E3	UBE2S	GCSAM	ZNF704	UGP2	NDUFA4	ENSG0…	FAM11…	NSD2	KIF14
CELLMARKERMSIGDB_HAY_BONE_MARROW_STROMAL	TDO2	WWTR1	CSF1	EDNRB	SLC7A2	SFRP1	SERTA…	C7	OSR2	EFEMP2	MARCKS	COL1A1	CCL2	TSKU	SYNPO2	IRX2-…	PARVA	ZFP36	FERMT2	ENAH	EFNA1	LMNA	GNG12	YAP1	TEAD1	TPBG	INHBB	FAM13C	VWA1	COL6A2
CELLMARKERMSIGDB_HE_LIM_SUN_FETAL_LUNG_C0…	TAGAP	PRKAG2	KCND3	LRRC3B	HPSE2	RPRD1B	PNCK	GIPC1	C1orf…	LRIG1	EPB41…	XIST	INKA2	SH3PX…	OLFML…	FBXO32	TMTC2	REC8	NTN1	TLE4	GPR161	AKIRI…	BCAR1	CRTC3	PACSI…	LDB2	KRT17	RGMB	RHOJ	RPS6K…
CELLMARKERMSIGDB_HE_LIM_SUN_FETAL_LUNG_C0…																														
CELLMARKERMSIGDB_HE_LIM_SUN_FETAL_LUNG_C0…																														
CELLMARKERMSIGDB_HE_LIM_SUN_FETAL_LUNG_C0…	XIST	HOXC5	CCND3	PARD6B	GABAR…	BAIAP2	SLC4A2	SPRR2F	MAG	LRRN1	ST6GA…	CSRP2	AGPAT2	CTXN1	HOOK1	ASPG	RNF141	ARHGA…	ZMIZ1	RHOU	DPP4	KIAA1…	RSPO1	CTSH	CADPS2	TOLLIP	OLFML…	ASH2L	CA11	C15or…
CELLMARKERMSIGDB_HE_LIM_SUN_FETAL_LUNG_C0…																														
CELLMARKERMSIGDB_HE_LIM_SUN_FETAL_LUNG_C0…																														
CELLMARKERMSIGDB_HE_LIM_SUN_FETAL_LUNG_C0…	LAMA5	HSBP1…	PRELP	PODXL	PLCB1	PTAFR	P4HTM	HDAC7	CGN	TAPBP	MAPKA…	LAMC2	PRR15L	PLEC	SFRP5	UNC5B…	EFNB2	PPP1R…	CFC1	CARNS1	MAMSTR	RPRD1B	SH3BP5	NQO1	CST6	FGF9	PLA2G…	RNASE4	NET1	CCNDB…
CELLMARKERMSIGDB_HE_LIM_SUN_FETAL_LUNG_C0…	AURKB	H2AC25	HAPLN1	C21or…	SPC25	NDC80	ATAD5	RAD51…	MAD2L1	KLHL5	SLC2A…	LATS2	CIP2A	FZR1	NEIL3	H2AC14	ADAMT…	KNL1	MELK	SLC8A1	CKAP2L	SGO2	SLC25…	KIFC1	FLRT3	DCLRE…	FGF13	DTNB	TACC3	PLK1
CELLMARKERMSIGDB_HE_LIM_SUN_FETAL_LUNG_C0…																														
CELLMARKERMSIGDB_HE_LIM_SUN_FETAL_LUNG_C0…	CLDN1	MET	HEG1	SEMA3C	PRSS33	KLF5	CAMK1D	ARHGA…	DHCR7	CARD16	EPS8L1	FRY	TMEM1…	ANXA1	PRKCI	TM7SF2	SCG5	ECHDC2	CDON	PYCARD	HSPB8	TFPI2	LAMC2	MIEN1	SORBS2	PPP1R…	MEIS2	RHOD	COBLL1	SLC16…
CELLMARKERMSIGDB_HE_LIM_SUN_FETAL_LUNG_C0…																														
CELLMARKERMSIGDB_HE_LIM_SUN_FETAL_LUNG_C1…																														
CELLMARKERMSIGDB_HE_LIM_SUN_FETAL_LUNG_C1…																														
CELLMARKERMSIGDB_HE_LIM_SUN_FETAL_LUNG_C1…																														
CELLMARKERMSIGDB_HE_LIM_SUN_FETAL_LUNG_C1…	POLA2	PARPBP	KHDRB…	FANCD2	NEK2	RIPPL…	ECT2	LOXL3	CIP2A	MYCN	TUBB4A	ST8SI…	RND3	AGPAT4	HBEGF	DDX11	NDE1	CITED4	TCEAL7	ARID3B	MDC1	WDR76	CRABP1	SPAG5	HES6	F2R	CDC25C	DSN1	SPC25	CEP55
CELLMARKERMSIGDB_HE_LIM_SUN_FETAL_LUNG_C1…	LRP11	NSG2	ABCA2	MMRN1	RASA4	CEP126	DDC	KLC2	MAP6	SCAMP5	FAM11…	EYA4	TNFRS…	PSD	SOBP	ZNF79…	BAIAP3	KCNH2	DLG5	MADD	CADPS	JAKMI…	RCOR2	SNAP25	SLCO3…	RTL5	NCALD	UNC80	FLVCR1	DGKZ
CELLMARKERMSIGDB_HE_LIM_SUN_FETAL_LUNG_C1…																														
CELLMARKERMSIGDB_HE_LIM_SUN_FETAL_LUNG_C1…																														
CELLMARKERMSIGDB_HE_LIM_SUN_FETAL_LUNG_C1…																														
CELLMARKERMSIGDB_HE_LIM_SUN_FETAL_LUNG_C1…	PLXND1	VSIR	SFN	CDKN1A	TNFRS…	DGKH	ALDH3…	AQP5	SLITR…	RAB27B	KIAA1…	NEBL	LHFPL6	ADGRF1	BIK	MEIS1	CHRM3	LMO3	TMEM40	ITGB4	PHLDB3	CREB3…	HLA-B	ADRA2A	KLK13	EPS8L1	COMTD1	C4orf…	MYB	PPM1L
CELLMARKERMSIGDB_HE_LIM_SUN_FETAL_LUNG_C1…	SPTBN2	EFHD2	PGAM2	RIMKLA	FNBP1	NAV1	RASA4B	DNER	SST	OPTN	KCNMA1	NRCAM	PSD	BAALC	GPC6	BAIAP3	INPP1	RUNDC…	ESPL1	SLCO3…	AP3B2	ADCY1	KCNT2	TNFRS…	SNAP25	FSTL5	NDRG4	SMIM32	CELF3	DPP10
CELLMARKERMSIGDB_HE_LIM_SUN_FETAL_LUNG_C1…	RARB	ZNF490	SFN	MARVE…	TFAP2C	MCUB	CCDC1…	TUBB6	PRLR	EMP3	MCC	SOX15	ACTA2	TNFRS…	S100A2	IRX2-…	USP31	ISL1	EGR3	PART1	PPP1R…	PTPRE	MAP2	GLCE	CPXM1	TRNP1	CRYBG1	CDKN1A	KLF2	PRKAR…
CELLMARKERMSIGDB_HE_LIM_SUN_FETAL_LUNG_C1…	PAX9	PDE4B	ESYT2	SAYSD1	RORA	MIA	SPDEF	SLC66…	SH2D3A	ITPR2	S100A2	DAGLB	OGFRL1	CRYAB	EFHD1	CASP4	SLITR…	CRIM1	MCC	GSTA1	SLC39…	FRMD4A	FBLN2	NEBL	METRN	MIF4GD	HDHD5…	ARSJ	GATM	RIC3
CELLMARKERMSIGDB_HE_LIM_SUN_FETAL_LUNG_C2…	IL4I1	SREBF1	GRAMD4	SPIC	C1orf…	FAM20C	PAPSS1	ZFYVE…	SH3YL1	RAB3G…	PRXL2A	EMP1	MRPL1	PIP5K…	NUPR1	PICK1	LPCAT3	CDCP1	ZNF480	KIFC3	VEGFB	PHYH	SERPI…	NRP2	RAB35	ABCC3	ACOX1	IFT74	CUEDC1	SLC39…
CELLMARKERMSIGDB_HE_LIM_SUN_FETAL_LUNG_C2…	TMEM97	PTGER2	LRRCC1	NUP210	MCOLN2	CYP2E1	HTR3A	TP53RK	BCAT2	H2AC14	H2AC13	HSH2D	CYFIP2	FHIP1B	COMTD1	FRY	FMNL2	OAT	PTPRC…	ACSS2	LMNB1	ARHGA…	HSPBP1	ECE1	DAPK2	MAP3K4	FLNB	PAWR	LONRF1	UPK3A
CELLMARKERMSIGDB_HE_LIM_SUN_FETAL_LUNG_C2…																														
CELLMARKERMSIGDB_HE_LIM_SUN_FETAL_LUNG_C2…																														
CELLMARKERMSIGDB_HE_LIM_SUN_FETAL_LUNG_C2…																														
CELLMARKERMSIGDB_HE_LIM_SUN_FETAL_LUNG_C2…	MRM3	TRUB1	TSPAN2	MPHOS…	MUL1	CMTR2	RGP1	TMEM2…	SCN3A	MTRFR	GATA3	NOG	PROM1	LRRC70	ICA1	SLC2A5	CYFIP2	ABHD4	RAB13	SCRN2	GBP4	TNFSF4	BCAS4	FAM21…	STIM1	ZNF302	STMN3	SULT1…	H3C10	KCNN4
CELLMARKERMSIGDB_HE_LIM_SUN_FETAL_LUNG_C2…	MAD2L…	PROS1	SYTL4	GET1	MPL	PPP1R…	MOB3C	HEMGN	DCLRE…	WIPI1	MAVS	FUT8	MOB1B	CHST12	SWI5	PLA2G…	SAMD14	SH3BG…	TSPAN…	PARD3	GNAZ	CD82	GJA4	ZFAND…	MYOM1	PRKCQ	PLEKH…	RHOF	YES1	GCLM
CELLMARKERMSIGDB_HE_LIM_SUN_FETAL_LUNG_C2…																														
CELLMARKERMSIGDB_HE_LIM_SUN_FETAL_LUNG_C2…	KIAA1…	TMEM91	MFSD11	MYLK	CDK17	KIAA0…	CCNT2	PRIMP…	VAPB	MYLK3	TXNL4B	PTP4A3	CTNS	SNCA	SIRT3	TBXA2R	CTNNA…	RCL1	CREBZF	MAPK6	CMTM5	ARRDC4	YES1	TCEAL9	WASF1	CDC16	DCLRE…	ANKRD…	PPM1A	RIMOC1
CELLMARKERMSIGDB_HE_LIM_SUN_FETAL_LUNG_C2…	NGLY1	PPM1K	CENPW	UTP23	FAM10…	RAB29	NCLN	LAS1L	NUP54	YARS1	DMAC2	ZMYM5	TUBG1	DCTPP1	SUN2	PMS1	MAG	TBCC	CMAS	HAMP	CEP95	CSF2RB	EIF1AY	ALCAM	RPIA	MAP3K7	RAB7B	PLAAT3	HMGCS1	LY9
CELLMARKERMSIGDB_HE_LIM_SUN_FETAL_LUNG_C2…																														
CELLMARKERMSIGDB_HE_LIM_SUN_FETAL_LUNG_C2…																														
CELLMARKERMSIGDB_HE_LIM_SUN_FETAL_LUNG_C2…																														
CELLMARKERMSIGDB_HE_LIM_SUN_FETAL_LUNG_C3…																														
CELLMARKERMSIGDB_HE_LIM_SUN_FETAL_LUNG_C3…	DIAPH3	SLC25…	SGO1	ATG4D	DEPDC1	FERMT3	SOX6	MAD2L1	NCEH1	PPME1	CENPK	SPC24	IQGAP2	CDCA5	SPINT2	CENPN	PARPBP	CIP2A	H1-3	KPNA2	H2AC16	CISH	BUB1	KNL1	SLC22…	RAB3I…	BLVRA	SHCBP1	TMOD1	AGO2
CELLMARKERMSIGDB_HE_LIM_SUN_FETAL_LUNG_C3…	CCN1	BCAT2	PFKP	ARHGA…	RBM18	MRPS27	FBXO17	RANGRF	MID1I…	NUP93	GIPC1	PTTG1	C12or…	ACOT13	TMEM2…	SELEN…	PDLIM4	TARBP2	SNAI2	ERRFI1	DEF8	CXXC5	EDIL3	TUBB3	DHCR7	MIR36…	HPRT1	ABCA1	HADH	CKS2
CELLMARKERMSIGDB_HE_LIM_SUN_FETAL_LUNG_C3…	TRMT9B	MXRA8	PGAP4	CAMK1	ALDH1…	MRC1	MID1	C6orf…	SEMA3A	FMOD	ZNF38…	COQ8A	TNFAI…	TRIP6	CASK	NEXN	RCAN1	FOXP2	PEG10	SLC30…	PGM1	BMX	PDLIM3	COL4A5	SSH1	GLRX	ROBO3	MGST1	CXADR	CNST
CELLMARKERMSIGDB_HE_LIM_SUN_FETAL_LUNG_C3…	MEGF6	PXMP2	PCSK6	CCDC1…	ITSN1	ASPH	THSD7A	PLIN5	MAP3K1	CXXC5	ARRDC2	LAPTM…	GIMAP5	TRPC6	C1QTN…	PGF	P4HA3	MMP17	DNPH1	SMOC1	TLE1	IFT57	ADAMT…	BCHE	DKK3	TNFAI…	TMEM1…	TRIP6	LINC0…	TRIL
CELLMARKERMSIGDB_HE_LIM_SUN_FETAL_LUNG_C3…																														
CELLMARKERMSIGDB_HE_LIM_SUN_FETAL_LUNG_C3…	ARHGA…	HLA-D…	PDLIM3	NOX4	PELI1	NPIPA7	C19or…	PXDC1	FZD8	PLXNA2	TOX	IRF6	HAPLN3	ATL2	ITGB5	FAM10…	PTGFRN	GHR	MIR99…	PLK3	KLHL13	TFPI2	XAF1	CAMK4	GRB14	ARID5A	MFAP4	AIF1L	SLC6A4	AIG1
CELLMARKERMSIGDB_HE_LIM_SUN_FETAL_LUNG_C3…	ZIC2	VANGL1	CASK	IGSF8	COLEC…	MAGED4	SYNE1	ACKR2	PLEKH…	ANGPT2	CRAT	PLSCR3	MXRA8	ADAM9	SGK3	CRNDE	PTPRK	PLPP5	SOCS6	CLDN11	TMEM4…	PDLIM4	B9D1	BHMT	LGALS…	SRPK1	TPD52	CTTNB…	C15or…	LRP11
CELLMARKERMSIGDB_HE_LIM_SUN_FETAL_LUNG_C3…																														
CELLMARKERMSIGDB_HE_LIM_SUN_FETAL_LUNG_C4…																														
CELLMARKERMSIGDB_HE_LIM_SUN_FETAL_LUNG_C4…																														
CELLMARKERMSIGDB_HE_LIM_SUN_FETAL_LUNG_C4…																														
CELLMARKERMSIGDB_HE_LIM_SUN_FETAL_LUNG_C4…																														
CELLMARKERMSIGDB_HE_LIM_SUN_FETAL_LUNG_C4…	SPAG5	WDR76	MAGOHB	SNRNP…	CDC6	CDCA2	DONSON	H3C7	DSN1	CDK2	CCDC34	PMVK	LRR1	RHNO1	PSMD14	PANK2	ZDHHC…	FANCG	ECT2	COMMD4	RCC1	CENPA	POP7	OXCT1	CHEK2	HAT1	H3C8	ACAT2	PPIF	DLEU2
CELLMARKERMSIGDB_HE_LIM_SUN_FETAL_LUNG_C4…	WDR54	ACAT2	DSN1	RFC4	AHCY	CBX1	RCC1	POLD1	BCL2L…	BARD1	CHI3L2	TUBG1	CHAF1A	CFAP20	MCM3	ARHGA…	DEPDC…	KNL1	BUB1	POC1A	RMI2	DYNC2…	BRCA2	MCM5	FBXL5	PSAT1	TRIM28	EBP	TTF2	TPGS2
CELLMARKERMSIGDB_HE_LIM_SUN_FETAL_LUNG_C4…																														

CELLMARKERCAN_ACINAR_CELLS																														
CELLMARKERMSIGDB_HE_LIM_SUN_FETAL_LUNG_C4…																														
CELLMARKERMSIGDB_HE_LIM_SUN_FETAL_LUNG_C4…																														
CELLMARKERMSIGDB_HE_LIM_SUN_FETAL_LUNG_C4…																														
CELLMARKERMSIGDB_HE_LIM_SUN_FETAL_LUNG_C5…																														
CELLMARKERMSIGDB_HE_LIM_SUN_FETAL_LUNG_C5…	ATG4C	KLF13	PELI1	CEPT1	U2AF1…	DCTD	RNF126	ADAM19	RASSF5	RILPL2	RPIA	PFKP	CCL22	PES1	APOBE…	MCCC2	ALDH3…	MRTO4	PAPSS1	QPCT	HDAC3	HMBS	NDUFA…	NUP37	DLD	NPM3	HSD17…	EBI3	C15or…	RASSF2
CELLMARKERMSIGDB_HE_LIM_SUN_FETAL_LUNG_C5…																														
CELLMARKERMSIGDB_HE_LIM_SUN_FETAL_LUNG_C5…																														
CELLMARKERMSIGDB_HE_LIM_SUN_FETAL_LUNG_C5…	ORC6	GGH	CKAP5	SYNE2	FANCG	RHNO1	PDS5B	RFC4	NSD2	PAQR4	BCL2L…	SPC25	FHL1	DSCC1	H3C3	SLC43…	NCAPG2	WDR76	CFAP20	DLEU2	TOPBP1	RDX	DIAPH3	DTL	ENG	CKLF	CTDSP…	BUB1	BRCA1	MCM10
CELLMARKERMSIGDB_HE_LIM_SUN_FETAL_LUNG_C6…																														
CELLMARKERMSIGDB_HE_LIM_SUN_FETAL_LUNG_C7…																														
CELLMARKERMSIGDB_HE_LIM_SUN_FETAL_LUNG_C7…																														
CELLMARKERMSIGDB_HE_LIM_SUN_FETAL_LUNG_C7…																														
CELLMARKERMSIGDB_HE_LIM_SUN_FETAL_LUNG_C7…																														
CELLMARKERMSIGDB_HE_LIM_SUN_FETAL_LUNG_C7…	LRRC8B	HOXB5	RGS4	FAXC	SCN2A	COX7A1	ZNF80…	NRG1	MTMR6	HHIP-…	RET	VSTM2A	KLC2	LAYN	RASGE…	EPB41…	RXYLT1	SLC38…	C1orf…	SRGAP1	FBXW8	SEMA4D	PNCK	ARFGE…	RGS7	TPPP3	AMPH	RUNDC…	PIRT	AKR1C2
CELLMARKERMSIGDB_HE_LIM_SUN_FETAL_LUNG_C7…																														
CELLMARKERMSIGDB_HU_FETAL_RETINA_AMACRINE																														
CELLMARKERMSIGDB_HU_FETAL_RETINA_BIPOLAR																														
CELLMARKERMSIGDB_HU_FETAL_RETINA_FIBROBLA…	TCF4	COL4A2	SPATS…	PLPP3	SPARC	RN7SL…	LTBP4	EMILI…	CCN1	ITGB1	EFEMP2	FST	LAPTM…	TBX18	SPON1	SNED1	JUN	ARHGA…	B2M	GNG12	RHOC	FLNA	IL6ST	NCOA7	CYTH3	PLEKH…	ANXA2	ADD3	TWIST1	SCIN
CELLMARKERMSIGDB_HU_FETAL_RETINA_HORIZONT…																														
CELLMARKERMSIGDB_HU_FETAL_RETINA_MICROGLIA	BAG3	RGS19	IL10RA	BTG2	SELPLG	SLA	AOAH	PTPRE	IPCEF1	TYMP	STAB1	GNAI2	U1	BIN1	TLR4	P2RY12	MYO1F	GRN	CYFIP1	AP1B1	EVI2B	TNFRS…	ARHGA…	SIGLE…	CAP1	SH3KB…	PLCB2	FOSB	TRIM38	HMOX1
CELLMARKERMSIGDB_HU_FETAL_RETINA_MULLER																														
CELLMARKERMSIGDB_HU_FETAL_RETINA_PHOTOREC…																														
CELLMARKERMSIGDB_HU_FETAL_RETINA_RGC	MVD	ADAM11	CCDC1…	CORO2A	CETN2	STMN3	DPYSL4	IDI1	CACNB3	PPP2R…	KIF3A	MAP1B	HEY1	DNER	DBNDD1	RAB3B	CADM1	STARD…	OCIAD2	STX1A	RUNDC…	KLHL35	EBP	CRABP1	TMSB4…	BTBD10	YWHAH	RAB6B	SYT13	SBK1
CELLMARKERMSIGDB_LAKE_ADULT_KIDNEY_C1_EPI…																														
CELLMARKERMSIGDB_LAKE_ADULT_KIDNEY_C10_TH…	FAU	EIF4G2	MTCH1	MYL6	COX5B	RPS29	RPL3	CANX	RPL10	ELF3	ZFAND5	DNAJA1	SELEN…	PLEKH…	RPL14	RPSA	RPS4X	EEF1A1	CYSTM1	RPL6	SASH1	COX4I1	PRDM1…	ARF1	DDX5	NPM1	HSPA9	ATP5M…	RPL4	MRFAP1
CELLMARKERMSIGDB_LAKE_ADULT_KIDNEY_C12_TH…	UGT8	RPL13	SERF2	TMEM59	NNT	ATP5M…	ATP5F…	MYL6	RPL13A	ANK2	EDF1	RPS27A	FAU	ATP5MG	RPS29	ACADVL	PANTR1	RNF150	SCNN1A	ACACB	ATP6V…	FKBP2	RPS18	CUTA	FAM17…	SGK2	RPL8	RPL32	SLC3A1	SSR4
CELLMARKERMSIGDB_LAKE_ADULT_KIDNEY_C18_CO…	HLA-B	NDRG1	LITAF	GPX1	PFDN5	ATP5F…	ISCU	CANX	MAL	TGOLN2	MYO1B	RPL30	CNBP	RPS12	RPL18	EIF4A2	RTN3	RPL8	GABAR…	RPS9	SCIN	SAT1	RPL9	IFITM2	KCNJ16	RPS20	PRDX6	CIB1	HINT1	CD81
CELLMARKERMSIGDB_LAKE_ADULT_KIDNEY_C19_CO…	HADHB	DYNLT1	RPS19	GABAR…	LDHB	SNX3	GHITM	ATP5PB	SELEN…	RPL32	PDIA6	PDIA3	REEP5	ATP1B1	PCBP1	HADHA	TBC1D…	EID1	EIF3K	RPS24	CBR1	SPTBN1	RPS13	RPS7	RPL21	RPS20	NDUFA5	RCAN2	CD74	PPIA
CELLMARKERMSIGDB_LAKE_ADULT_KIDNEY_C25_EN…																														
CELLMARKERMSIGDB_LAKE_ADULT_KIDNEY_C28_IN…																														
CELLMARKERMSIGDB_LAKE_ADULT_KIDNEY_C29_UN…																														
CELLMARKERMSIGDB_LAKE_ADULT_KIDNEY_C5_PRO…	CTNNA3	SLC39…	LARP1	LINC0…	VDR	LRP2	ASPA	KIFC3	TRPM3	UPP2	HOOK2	PRUNE2	C9orf…	HNF1A…	PPP1R…	SUGCT	TOB2	IL1RA…	AQP4-…	AK4	GALM	BNIP3L	OPHN1	ZC4H2	HECW1	BRD4	NFIC	KIAA1…	CACNA…	THOC2
CELLMARKERMSIGDB_MANNO_MIDBRAIN_NEUROTYPE…																														
CELLMARKERMSIGDB_MANNO_MIDBRAIN_NEUROTYPE…	GABBR2	GABRA2	GABRB1	GABRB3	GABRG2	GAP43	GARNL3	GDAP1…	GFRA1	GLCE	GLRA2	BICRAL	GNAL	GNAO1	GNAZ	GNG2	GNG3	GOLGA…	GPC2	GPM6A	GPR68	GPR85	GPRAS…	GPRIN3	GRIA1	GRIA3	GRIA4	GRIK2	GRIK3	GRIN3A
CELLMARKERMSIGDB_MANNO_MIDBRAIN_NEUROTYPE…	NALF1	FAM16…	TLCD3B	GARIN…	FAT3	FAT4	FAXC	FBLL1	FBXL16	FBXL3	FBXO11	FGD1	FGF13	FGF9	FKBP1B	FNBP1L	FNDC9	FOXA1	FOXA2	FOXP2	FRMD5	FRMD8	FRMPD4	FRS3	FRY	FUT9	GAB2	GABBR2	GABRA2	GABRB1
CELLMARKERMSIGDB_MANNO_MIDBRAIN_NEUROTYPE…	FLRT3	FNBP1L	FOXA1	FRMPD3	FRMPD4	FRY	FUT9	FXYD6…	GABAR…	GABRA2	GABRB1	GABRB3	GABRG2	GAP43	GARNL3	GFRA1	BICRAL	GNAL	GNAO1	GNG3	GNPTAB	GOLGA3	GOLGA…	GPC2	GPM6A	GPR12	GPR26	GPR85	GPRAS…	GPRIN3
CELLMARKERMSIGDB_MANNO_MIDBRAIN_NEUROTYPE…	CYYR1	DAB2	DAP	DCBLD1	DDIT3	RIGI	DEDD2	DEGS2	DENND…	DENND3	DGKE	DHRS3	DLL4	DNASE…	DNASE…	DOCK6	DOCK9	DOK4	DPEP1	DSP	DTX3L	DUSP1	DUSP5	DUSP6	DYSF	ECE1	ECM1	ECSCR	EDN1	EDN3
CELLMARKERMSIGDB_MANNO_MIDBRAIN_NEUROTYPE…	ENTPD1	ERF	ERMAP	ETS2	EVI2A	EVI2B	EXOSC5	FABP3	NIBAN1	TENT5A	FAR2	FCER1G	FCGR1A	FCGR1…	FCGR2A	FCGR3A	FCGRT	FERMT3	FGD2	FGL1	FGL2	FHL3	FKBP15	FKBP4	FKBP5	FLI1	FOLR2	FOSB	FOXN3	FOXO3
CELLMARKERMSIGDB_MANNO_MIDBRAIN_NEUROTYPE…	DNER	DNM3	DOCK3	DOCK4	DOK4	DOP1A	DPP6	DPYSL3	DSC3	DSCAM	DYNC1…	EDIL3	EEA1	EEF1A2	EID2B	ELAVL2	ELAVL3	ELAVL4	ELFN2	ELOVL4	EML5	ENC1	ENPP4	EPB41…	EPHA5	EPHB1	ERAP2	ERC1	ERC2	EYA4
CELLMARKERMSIGDB_MANNO_MIDBRAIN_NEUROTYPE…	NAP1L3	NAPB	NAV1	NCAM1	NDRG4	NDST3	NEGR1	NELL2	NFASC	NHLH2	NKAIN2	NKX6-2	NMNAT2	NNAT	NREP	NRN1	NSG1	NUAK1	NXPH4	NYAP1	OCIAD2	OLFM1	OLFM3	ONECU…	ONECU…	PAFAH…	PAK3	PAK5	PALB2	PCBP4
CELLMARKERMSIGDB_MANNO_MIDBRAIN_NEUROTYPE…	GRIP2	GRM7	GSE1	MACRO…	HBA1	HBB	HCN3	HDAC1	HERC1	HIVEP3	NSG2	HOOK1	HS3ST4	HS6ST3	IGBP1	IGFBP…	INA	INSM1	IRF2B…	IRGQ	IRX2	JAKMI…	JPH4	KALRN	KCNC1	KCNC2	KCNH8	KCNMA1	KCNMB2	KDM5B
CELLMARKERMSIGDB_MANNO_MIDBRAIN_NEUROTYPE…	JUP	KCNH8	KCNMA1	KHDRB…	ARFGE…	SHTN1	KIF21A	KIF26A	KIF3A	KIF5C	KIFAP3	KIFC2	KLC1	L1CAM	LCOR	LHX4	LINGO1	ENSG0…	ISL1-…	PLPPR2	PLPPR4	LRFN5	LRRN3	LRRTM2	LYST	LZTS1	LZTS3	MAB21…	MAP1B	MAP1L…
CELLMARKERMSIGDB_MANNO_MIDBRAIN_NEUROTYPE…	IGFBP2	INHBB	INTS1	ITGAV	ITGB5	ITGB8	ITM2C	ITPKB	ITPR2	JPH3	KANK1	KCND2	KCNE5	KCNIP1	KCNJ1	KCNQ2	KIF13A	KIF14	KLRC2	KRT14	KRT17	CNMD	LGR5	LHFPL2	LHFPL3	LIMA1	LINGO1	LMOD3	LPAR4	PLPPR1
CELLMARKERMSIGDB_MANNO_MIDBRAIN_NEUROTYPE…	CYSTM1	CYTH3	DAAM2	DACT1	DACT3	DBH	DBNDD2	DCBLD1	DCLK3	DCN	DDIT3	DGKA	DGKH	DNAJB4	DNAJC…	DNM3OS	DOCK1	DOCK6	DRAM1	DSP	DUSP5	DYNLT3	EBF1	ECE1	ECH1	ECM2	EDNRA	EFCAB…	EFCAB5	EFEMP2
CELLMARKERMSIGDB_MANNO_MIDBRAIN_NEUROTYPE…	MCM7	MDK	FAT2	MELK	MID1	MIR221	MIR454	MIR99…	MIS18…	MKI67	MND1	MSI1	MTTP	MXRA8	MYBL2	MYLK	NCAPD2	NCAPG	NCAPG2	NCAPH	NEK2	NELL2	NES	NOTCH1	NPTX2	NRARP	NUF2	NUSAP1	OTX2	OTX2-…
CELLMARKERMSIGDB_MANNO_MIDBRAIN_NEUROTYPE…	KNSTRN	LGI1	LIPG	LMNB1	LMNB2	LMX1A	LMX1B	LOXL4	LRP2	LSM4	LTBP1	MAD2L1	MASTL	MAT2B	SRF	MCM2	MCM3	MCM6	MCM7	MDK	MELK	MEST	MID1	MIR99…	MIS18…	MKI67	MMRN1	MND1	MSI1	MT1X
CELLMARKERMSIGDB_MANNO_MIDBRAIN_NEUROTYPE…	IMMP1L	INPPL1	IRS1	ITGB3…	JAM2	KCNQ2	KDM4A…	CIP2A	KIF11	KIF14	KIF15	KIF18A	KIF22	KIF23	KIF2A	KIF2B	KIF2C	KIF4A	KLHDC…	KNSTRN	KPNA2	KRT19	LDHA	LGI1	LGR5	LIX1	LMNB1	LMX1A	LMX1B	ENSG0…
CELLMARKERMSIGDB_MANNO_MIDBRAIN_NEUROTYPE…	LRP2	MAD2L1	MAGOH	MAGT1	MAML2	MAP6	SRF	MCM2	MCM3	MCM5	MDK	MELK	MEST	MID1	MIR36…	MIR99…	MLF1	MMP11	MMP2	MMRN1	MND1	MNS1	MSI1	NCAPG	NCAPG2	NCAPH	NEK11	NES	NOX4	NPY
CELLMARKERMSIGDB_MANNO_MIDBRAIN_NEUROTYPE…	IRS1	ITGB8	ITPKB	JAM2	KCNG1	KCNN3	CEP126	KIAA1…	SHISA…	KLF15	KLHDC…	KLHDC…	KMT2E…	LAMB2	LGI1	LHFPL6	LHX3	LIPG	LMCD1	LMX1A	LRP2BP	LRP8	LRRC17	LRRC3…	LRRIQ1	LRRN2	LRRTM3	LTBP1	LYPD1	MAML2
CELLMARKERMSIGDB_MANNO_MIDBRAIN_NEUROTYPE…	ENKUR	ENPP2	EPB41…	EPHA3	EPHB1	ERF	ERMARD	EVI5	EZR	F3	FABP5	FABP7	FADS2	FAM16…	FAM18…	FAM18…	GASK1B	TAFA5	FAM21…	FAM3C	RIPOR2	FAM66D	LRATD2	CIBAR2	FAT1	FAT3	FGF1	FGFBP3	FGFR3	FHL1
CELLMARKERMSIGDB_MANNO_MIDBRAIN_NEUROTYPE…	HBA2	HBB	HBG1	HBG2	HDAC4	HEATR…	HEPAC…	HEPN1	HES1	HES5	HEY2	RIDA	HS6ST2	HSDL2	HSPA6	HSPA7	HSPB1	HYDIN	ID4	IFI27…	IFI44	IFI44L	IFI6	IGFBP2	ILDR2	INHBB	INPPL1	IQCA1	IQGAP2	IRX1
CELLMARKERMSIGDB_MANNO_MIDBRAIN_NEUROTYPE…	LFNG	LHFPL3	LIFR	LIMA1	LITAF	LIX1	LMCD1	LRFN5	LRIG1	LRP1	LRP1B	LRRC17	LRRC4B	LUZP2	LYPD1	MAP3K1	MAPK14	MCM5	MDGA2	METRN	METTL…	MGST1	IGSF9B	MLC1	MNS1	MORN5	MPC1	MRC2	MT1X	MT2A
CELLMARKERMSIGDB_MANNO_MIDBRAIN_NEUROTYPE…	EFCAB…	EFCC1	EFEMP1	EFEMP2	EFHC2	EFNB3	EGLN3	EGR1	EID2B	ENKUR	ENPP2	EPB41…	EPHA1…	EPHA3	ERF	EVA1C	EVI5	EZR	F5	FADS2	FAH	FAM18…	GASK1B	ATOSA	FAT1	FAT3	FBLN5	FBXL7	FGF1	FGFR3
CELLMARKERMSIGDB_MANNO_MIDBRAIN_NEUROTYPE…	LZTS1	MAP1B	MAP2	MAP6	MAP7D2	MAPK6	MAPK8	MAPK8…	MAPRE2	MARCH…	MCF2	MCF2L	MIAT	MIR12…	MIR22…	MLLT11	MMP24	MSANT…	MTUS2	MYO5A	MYT1	NAP1L5	NAPB	NCAM1	NCAN	NCDN	NEDD4L	NFASC	NHLH2	NKAIN1
CELLMARKERMSIGDB_MANNO_MIDBRAIN_NEUROTYPE…	GPR12	GPR149	GPR176	GPR26	GPRAS…	GREM2	GRIA1	GRIA2	GRIA4	GRIK2	GRIN3A	GRIP2	GRM5	GRM7	GRM8	HBA1	NSG2	HPCAL4	HS3ST4	HS6ST3	HSPA1…	ICA1	IL27RA	INA	INHBA…	JAKMI…	JPH3	JPH4	KANK4	KCNC1
CELLMARKERMSIGDB_MENON_FETAL_KIDNEY_0_CAP…																														
CELLMARKERMSIGDB_MENON_FETAL_KIDNEY_10_IM…																														
CELLMARKERMSIGDB_MENON_FETAL_KIDNEY_2_NEP…																														
CELLMARKERMSIGDB_MENON_FETAL_KIDNEY_3_STR…																														
CELLMARKERMSIGDB_MENON_FETAL_KIDNEY_6_COL…																														
CELLMARKERMSIGDB_MURARO_PANCREAS_ACINAR_C…	FAM10…	LGALS3	ADAMT…	ZC3H1…	SLC39…	ELF3	KLF5	B3GNT2	PLIN5	IQGAP2	PTF1A	CXCL8	DHRS3	MYC	SH2D4A	FOXC1	KRT7	TCEA3	MAP3K5	PARD6B	TMSB4X	BAIAP…	SLC12…	OSMR	RAB27B	ATF3	SERIN…	ITGA6	PBLD	SORD
CELLMARKERMSIGDB_MURARO_PANCREAS_ALPHA_CE…	UQCR11	RHBDD2	PAPSS2	FADS1	SLC38…	KCNK16	DNPH1	NDUFB…	LBH	PGAP1	SYT4	RGS17	NBDY	TCEAL2	CERK	TUNAR	NFASC	CSTF3	HLA-A	TCEAL3	BCKDHB	GRSF1	SMAP2	RUNDC…	ITM2B	TUBA1B	JADE1	PITRM1	SLC9A7	IVNS1…
CELLMARKERMSIGDB_MURARO_PANCREAS_BETA_CELL	TOMM20	BHLHE…	AACS	BMP5	GNG4	MBP	BNIP3L	SOBP	RPL3	TCAF2	PER3	SRD5A1	TRIM2	SYNM	KCNJ11	MTUS2	HOOK1	DLGAP4	HOPX	VPS37A	BAG3	ACVR1B	ZNF395	IGF1R	KCNK17	MAPRE1	SPIRE1	RNF130	PARVB	CD200
CELLMARKERMSIGDB_MURARO_PANCREAS_ENDOTHEL…	MMP1	TCIM	ITPRIP	ZNF532	CD34	UNC5B	RBMS1	HLX	SH2B3	HOXB6	RILPL2	ARHGA…	SMAD7	NID1	MADCA…	HTRA3	DAB2IP	GRB10	PKP4	SLC12…	IFI16	HIP1	LPAR6	HDAC7	QKI	PEA15	ELK3	IFITM2	IL4R	CYP1B1
CELLMARKERMSIGDB_MURARO_PANCREAS_EPSILON_…																														
CELLMARKERMSIGDB_MURARO_PANCREAS_MESENCHY…	RAI14	PRKG1	PLAUR	MYOF	ALDH1…	FSCN1	LRRC32	MXRA8	FGF2	IL6	FRMD6	EBF1	ITPRIP	LHFPL2	CLIC4	ITPRI…	ZEB1	PHLDA1	CCN1	RAB31	HIC1	TPM4	STEAP1	NRP1	AXL	TNS1	ADAM19	DCBLD2	GUCY1…	RBMS1
CELLMARKERMSIGDB_RUBENSTEIN_SKELETAL_MUSC…	CASP4	ATP5F…	TALDO1	PSAP	HNMT	JUNB	HLA-D…	EIF1	LAMTO…	ARHGD…	PFN1	RPL26	TSPO	EIF4E2	CREG1	SLC25…	CRIP1	H3-3A	RPL28	CST3	S100A…	RPS13	OSTF1	LSM6	ZFAS1	STMP1	FXYD5	VCAN	PLP2	ATP5MG
CELLMARKERMSIGDB_RUBENSTEIN_SKELETAL_MUSC…	RPS16	RPS4Y1	CEBPD	ATP5F…	EEF1B2	SLC25…	RPLP1	NOP53	TCEAL9	FAU	EIF4A2	S100A6	NDUFA4	RHOBT…	RND3	H1-0	VDAC1	ALDOA	ATP5PO	EPB41…	COPS9	RPL23	HNRNP…	PPP1R…	EIF4A1	EIF2A	ZBTB20	MT-CO3	PRKX	RHOA
CELLMARKERMSIGDB_RUBENSTEIN_SKELETAL_MUSC…	MT-CO3	TINAG…	MAGED2	UQCRH	BCAM	SERPI…	HIGD1B	BAG1	ZNG1A	CAVIN3	PTMS	FXYD1	MPRIP	VIM	SNRK	AXL	MEF2C	YBX1	MPC1	GAPDH	NDUFB…	CPM	ITIH5	CYCS	ATP5F…	TPI1	PPP1CB	TAX1B…	RAP1A	CRIM1
CELLMARKERMSIGDB_TRAVAGLINI_LUNG_ALVEOLAR…	TMOD3	ENTRE…	ST3GA…	TRIM16	CGN	MGAT3	DIPK2A	ACKR4	ADRB2	NEDD9	ABI3BP	LAMB3	ANXA2	EFEMP1	MAP7D1	HSPB8	ARAP2	SULT1…	NTM	MYO1C	MYL12B	SULT1…	EHD1	PARD6B	PPFIB…	ANKS1A	IGFBP7	CAPN2	GADD4…	KRT8
CELLMARKERMSIGDB_TRAVAGLINI_LUNG_BASOPHIL…	TPSG1	COL18…	MORF4…	DUSP14	HIF1A	LINC0…	ZFP36	GRAP2	CPEB4	C17or…	SEPTI…	DHRS9	SIGLE…	NFKB2	IL13	AP3S1	VPS37B	CLCN3	VEGFA	TPM1	STX3	BTN2A2	EHD1	HEY1	SMYD3	DLC1	VMP1	P2RX1	LAT	STK17B
CELLMARKERMSIGDB_TRAVAGLINI_LUNG_CAPILLAR…																														
CELLMARKERMSIGDB_TRAVAGLINI_LUNG_CAPILLAR…	RHOA	MAPRE1	SELEN…	UACA	TRAPP…	ARPC2	TPR	SET	SPARC	ATRX	POLR2L	SRSF4	CFDP1	MYL6	DPYSL2	COMMD6	MED28	PFDN5	DDAH2	RALA	MT1L	CAPZA2	MYLK	SUMO2	EPAS1	UBE2E1	NAA38	PPP1R…	RPL38	GIMAP4
CELLMARKERMSIGDB_TRAVAGLINI_LUNG_CD4_MEMO…																														
CELLMARKERMSIGDB_TRAVAGLINI_LUNG_EREG_DEN…	PICALM	PRKAG…	PNP	CLEC4E	ANXA5	SLC16…	ARHGA…	NIBAN2	CCL17	RGCC	CDCP1	TOP1	SLAMF8	PHC2	ALCAM	RNF149	IL1R2	APLP2	CMTM6	ATP1B3	RGL1	COTL1	GNA15	CD86	FCGRT	RBM47	AP2S1	SPI1	MB21D2	MOB3B
CELLMARKERMSIGDB_TRAVAGLINI_LUNG_IGSF21_D…																														
CELLMARKERMSIGDB_TRAVAGLINI_LUNG_MESOTHEL…	DAB2	MRPL11	STEAP1	RPL41	MMD	PGM2	RGS17	EFNA1	PAPPA	SLC7A8	FCER1G	B4GAL…	IL1R1	MCUB	BACH1	CCDC25	BNC2	CCNG1	RGS10	MYL6B	LXN	TP53I3	RPS28	FAAP20	LINC0…	TMED3	EBPL	CHI3L1	LRRC59	PRKCI
CELLMARKERMSIGDB_TRAVAGLINI_LUNG_MYOFIBRO…																														
CELLMARKERMSIGDB_TRAVAGLINI_LUNG_NATURAL_…																														
CELLMARKERMSIGDB_TRAVAGLINI_LUNG_NEUTROPH…	PTGES…	PDLIM7	TLR4	S100A6	NCF2	RPL3P7	PAN3	NFAM1	SEC14…	JAML	HLX	COX6B1	CDK2A…	ARPC4	SORL1	RNA5S…	SLA	TLE3	CLEC7A	ARHGE…	NTNG2	CALM3	ANKRD…	DENND3	NIBAN1	MAP1L…	ELAPO…	R3HDM2	STX3	ATP6V…
CELLMARKERMSIGDB_TRAVAGLINI_LUNG_OLR1_CLA…	PRELI…	EMP3	IFI30	ATP5F…	KDM6B	MPP1	C3AR1	GSTO1	MCL1	FCGR2A	SRC	CHMP4B	PIM3	CCR1	SPI1	ITGAV	DENND…	GADD4…	TNFAI…	ANGPT…	ABCA1	GAPDH	SAMSN1	TYROBP	B3GNT5	TPT1	PID1	PSMA6	ICAM1	ADGRE2
CELLMARKERMSIGDB_TRAVAGLINI_LUNG_PLATELET…	TNNC2	LDLRA…	CCND3	NUTF2	PTGIR	WBP2	TDRP	SWI5	PRDX5	PARD3	PPM1A	H3P6	SMIM3	SELP	LYL1	PDCD10	UQCRH	SENCR	ZNF185	PYGL	PARVB	HACD4	BEND2	GGTA1	ICAM2	PBX1	TGFB1	AKIRI…	TMCC2	RAB27B
CELLMARKERMSIGDB_TRAVAGLINI_LUNG_PROLIFER…	NSG1	RRM1	ATP5F…	NCAPG2	DNAJC9	PITX1	NPM1	RPS2	FANCD2	FBLN1	S100A2	MCM5	SOX15	SMC4	UQCRH	ATP1B3	CLCA2	CDKN2C	IGFBP6	TPM2	SERPI…	UBE2S	E2F8	PRIM1	PAFAH…	RFC5	AHCY	SAC3D1	MCM3	CSE1L
CELLMARKERMSIGDB_TRAVAGLINI_LUNG_PROLIFER…	FEN1	ACTN1	ARHGA…	FHL1	ABCG1	PCNA	SMC4	DEPDC1	CENPH	GSTO1	SCD	MSR1	PLK1	DAB2	COPRS	IFI6	AMIGO2	CDCA2	TREM2	HEXB	EZH2	HNMT	VRK1	CRNDE	SAC3D1	ADAMT…	CIP2A	KNL1	ALDH3…	ACO1
CELLMARKERMSIGDB_TRAVAGLINI_LUNG_PROXIMAL…	ATP5F…	CYP2S1	RPS24	CLDN1	LRATD1	OAT	EIF3D	KLK11	FBXO32	CLEC2B	GAMT	ZNF38…	LRIG3	KLF5	CKMT1A	CALML3	RPL14	EEF1B2	LSP1	YBX1	RPL30	FAF1	THOC3	TNS4	TCF4	TNC	ATP1B3	TSKU	RPL32	HDAC1
CELLMARKERMSIGDB_TRAVAGLINI_LUNG_SEROUS_C…																														
CELLMARKERMSIGDB_TRAVAGLINI_LUNG_SIGNALIN…																														
CELLMARKERMSIGDB_TRAVAGLINI_LUNG_TREM2_DE…	SLC31…	COL6A1	CD86	PEA15	OSCAR	LAP3	TUBA1C	SMCO4	KCTD12	CLEC1…	SMIM30	GRINA	TNS1	ALCAM	PLBD1	C3AR1	AIF1	CLEC7A	GSTO1	ACOT7	RNASE6	PDLIM1	TIMP2	KLF4	IDH1	PLSCR1	CTNNA1	CTSS	SCARB2	TUBB6
CELLMARKERMSIGDB_TRAVAGLINI_LUNG_VASCULAR…																														
CELLMARKERMSIGDB_TRAVAGLINI_LUNG_VEIN_CELL																														
CELLMARKERMSIGDB_VANGURP_PANCREATIC_ALPHA…																														
CELLMARKERMSIGDB_VANGURP_PANCREATIC_DELTA…																														
CELLMARKERMSIGDB_VANGURP_PANCREATIC_GAMMA…																														
CELLMARKERMSIGDB_ZHENG_CORD_BLOOD_C1_PUTA…																														
CELLMARKERMSIGDB_ZHENG_CORD_BLOOD_C10_MUL…																														
CELLMARKERMSIGDB_ZHENG_CORD_BLOOD_C2_PUTA…																														
CELLMARKERMSIGDB_ZHENG_CORD_BLOOD_C4_PUTA…																														
CELLMARKERMSIGDB_ZHENG_CORD_BLOOD_C5_SIMI…																														
CELLMARKERMSIGDB_ZHENG_CORD_BLOOD_C6_HSC_…																														
CELLMARKERMSIGDB_ZHENG_CORD_BLOOD_C7_PUTA…																														
CELLMARKERMSIGDB_ZHENG_CORD_BLOOD_C8_PUTA…																														
CELLMARKERMSIGDB_ZHONG_PFC_C1_ASTROCYTE																														
CELLMARKERMSIGDB_ZHONG_PFC_C1_DLX5_POS_IN…																														
CELLMARKERMSIGDB_ZHONG_PFC_C2_ASCL1_POS_A…																														

CELLMARKERCAN_ACINAR_CELLS																														
CELLMARKERMSIGDB_ZHONG_PFC_C2_UNKNOWN_NPC																														
CELLMARKERMSIGDB_ZHONG_PFC_C3_ASTROCYTE	XKR4	PCDH19	KIAA1…	GNAZ	NRXN2	FADS3	LINC0…	EGFL7	CYP27…	CDK5R1	MAP6	PDE7A	MIR12…	NSG2	BMPR2	USP7	CEP164	IRF7	TOM1L2	GNB3	SPTBN2	PACS1	STXBP1	EEF1A2	ZNF320	IGHMB…	PALMD	SHANK2	RABL6	ASIC1
CELLMARKERMSIGDB_ZHONG_PFC_C3_HOPX_POS_OPC																														
CELLMARKERMSIGDB_ZHONG_PFC_C3_MICROGLIA	WDR47	SOBP	PI4KA…	LSAMP	PAFAH…	SEMA3C	ADAM22	SEPTI…	REEP2	ELAVL3	KDM4B	APBB1	NCDN	DDX51	CES2	RSPRY1	GNAO1	LRRC49	PCDH1…	ANKRD…	SEZ6L2	DAB2IP	MAPT	SNAP91	SV2A	PRRT2	SH3PX…	KIAA1…	SRM	PRXL2B
CELLMARKERMSIGDB_ZHONG_PFC_C3_UNKNOWN_INP																														
CELLMARKERMSIGDB_ZHONG_PFC_C4_UNKNOWN_INP																														
CELLMARKERMSIGDB_ZHONG_PFC_C6_PLXNA4_POS_…																														
CELLMARKERMSIGDB_ZHONG_PFC_C7_ORG_UNDERGO…																														
CELLMARKERMSIGDB_ZHONG_PFC_C8_ORG_PROLIFE…																														
CELLMARKERMSIGDB_ZHONG_PFC_C8_UNKNOWN_NEU…																														
CELLMARKERMSIGDB_ZHONG_PFC_C9_ORG_OTHER																														
CELLMARKERMSIGDB_ZHONG_PFC_HES1_POS_C1_NPC																														
CELLMARKERMSIGDB_ZHONG_PFC_MAJOR_TYPES_AS…	HADHB	TRIL	FABP7	FAM18…	GPX3	TBC1D…	PPP1R…	HNMT	OAT	BRINP…	ALPL	MYO10	ARHGE…	RASSF4	LUZP2	ELOVL2	BMPR1B	ELOVL5	IL33	C2orf…	P4HA1	ETV5	ATP13…	ROBO3	PHGDH	FERMT2	SYPL1	MAGT1	MRC2	ANXA5
CELLMARKERMSIGDB_ZHONG_PFC_MAJOR_TYPES_EX…																														
CELLMARKERMSIGDB_ZHONG_PFC_MAJOR_TYPES_MI…	ITGAX	PLA2G7	ZFP36…	IFI16	HAVCR2	SYK	MILR1	CTSD	GGTA1	MPEG1	ITGAM	SAT1	CCR1	SLC7A7	KLF2	FOSB	B2M	POLD4	RPH3A…	RUBCNL	P2RX4	LINC0…	CMTM7	SERPI…	EVI2A	EGR3	AOAH	GYPC	TLR10	OAS1
CELLMARKERMSIGDB_ZHONG_PFC_MAJOR_TYPES_OPC																														

CELLMARKERCAN_ACINAR_CELLS																														
CELLMARKERCAN_ADIPOCYTE_PROGENITOR_CELLS																														
CELLMARKERCAN_ADRENERGIC_NEURONS																														
CELLMARKERCAN_AIRWAY_EPITHELIAL_CELLS																														
CELLMARKERCAN_ALPHA_CELLS																														
CELLMARKERCAN_ALVEOLAR_MACROPHAGES																														
CELLMARKERCAN_ASTROCYTES																														
CELLMARKERCAN_B_CELLS																														
CELLMARKERCAN_B_CELLS_MEMORY																														
CELLMARKERCAN_B_CELLS_NAIVE																														
CELLMARKERCAN_BASOPHILS																														
CELLMARKERCAN_BERGMANN_GLIA																														
CELLMARKERCAN_BETA_CELLS																														
CELLMARKERCAN_CAJAL_RETZIUS_CELLS																														
CELLMARKERCAN_CARDIAC_STEM_AND_PRECURSOR_…																														
CELLMARKERCAN_CARDIOMYOCYTES																														
CELLMARKERCAN_CHOLANGIOCYTES																														
CELLMARKERCAN_CHOLINERGIC_NEURONS																														
CELLMARKERCAN_CHONDROCYTES																														
CELLMARKERCAN_CHOROID_PLEXUS_CELLS																														
CELLMARKERCAN_CILIATED_CELLS																														
CELLMARKERCAN_DELTA_CELLS																														
CELLMARKERCAN_DOPAMINERGIC_NEURONS																														
CELLMARKERCAN_DUCTAL_CELLS																														
CELLMARKERCAN_EMBRYONIC_STEM_CELLS																														
CELLMARKERCAN_ENTERIC_GLIA_CELLS																														
CELLMARKERCAN_ENTERIC_NEURONS																														
CELLMARKERCAN_ENTEROCYTES																														
CELLMARKERCAN_ENTEROENDOCRINE_CELLS																														
CELLMARKERCAN_EPENDYMAL_CELLS																														
CELLMARKERCAN_EPSILON_CELLS																														
CELLMARKERCAN_FOLLICULAR_CELLS																														
CELLMARKERCAN_FOVEOLAR_CELLS																														
CELLMARKERCAN_GAMMA_DELTA_T_CELLS																														
CELLMARKERCAN_GERM_CELLS																														
CELLMARKERCAN_GLUTAMINERGIC_NEURONS																														
CELLMARKERCAN_GRANULOSA_CELLS																														
CELLMARKERCAN_HEMATOPOIETIC_STEM_CELLS																														
CELLMARKERCAN_IMMATURE_NEURONS																														
CELLMARKERCAN_INTERNEURONS																														
CELLMARKERCAN_IONOCYTES																														
CELLMARKERCAN_KERATINOCYTES																														
CELLMARKERCAN_KUPFFER_CELLS																														
CELLMARKERCAN_LEYDIG_CELLS																														
CELLMARKERCAN_LOOP_OF_HENLE_CELLS																														
CELLMARKERCAN_LUMINAL_EPITHELIAL_CELLS																														
CELLMARKERCAN_LUTEAL_CELLS																														
CELLMARKERCAN_MEGAKARYOCYTES																														
CELLMARKERCAN_MELANOCYTES																														
CELLMARKERCAN_MESANGIAL_CELLS																														
CELLMARKERCAN_MESOTHELIAL_CELLS																														
CELLMARKERCAN_MICROFOLD_CELLS																														
CELLMARKERCAN_MICROGLIA																														
CELLMARKERCAN_MONOCYTES																														
CELLMARKERCAN_MOTOR_NEURONS																														
CELLMARKERCAN_MULLER_CELLS																														
CELLMARKERCAN_MYELOID_DERIVED_SUPPRESSOR_…																														
CELLMARKERCAN_MYOBLASTS																														
CELLMARKERCAN_MYOEPITHELIAL_CELLS																														
CELLMARKERCAN_MYOFIBROBLASTS																														
CELLMARKERCAN_NEUROBLASTS																														
CELLMARKERCAN_NEUROENDOCRINE_CELLS																														
CELLMARKERCAN_NEURONS																														
CELLMARKERCAN_NEUTROPHILS																														
CELLMARKERCAN_NK_CELLS																														
CELLMARKERCAN_NORADRENERGIC_NEURONS																														
CELLMARKERCAN_NUOCYTES																														
CELLMARKERCAN_OLIGODENDROCYTES																														
CELLMARKERCAN_OSTEOBLASTS																														
CELLMARKERCAN_OSTEOCLAST_PRECURSOR_CELLS																														
CELLMARKERCAN_OSTEOCLASTS																														
CELLMARKERCAN_OSTEOCYTES																														
CELLMARKERCAN_PANETH_CELLS																														
CELLMARKERCAN_PARATHYROID_CHIEF_CELLS																														
CELLMARKERCAN_PERICYTES																														
CELLMARKERCAN_PERITUBULAR_MYOID_CELLS																														
CELLMARKERCAN_PHOTORECEPTOR_CELLS																														
CELLMARKERCAN_PLASMA_CELLS																														
CELLMARKERCAN_PLASMACYTOID_DENDRITIC_CELLS																														
CELLMARKERCAN_PODOCYTES																														
CELLMARKERCAN_PROXIMAL_TUBULE_CELLS																														
CELLMARKERCAN_PULMONARY_ALVEOLAR_TYPE_I_C…																														
CELLMARKERCAN_PULMONARY_ALVEOLAR_TYPE_II_…																														
CELLMARKERCAN_PURKINJE_NEURONS																														
CELLMARKERCAN_RETICULOCYTES																														
CELLMARKERCAN_RETINAL_GANGLION_CELLS																														
CELLMARKERCAN_RETINAL_PROGENITOR_CELLS																														
CELLMARKERCAN_SATELLITE_CELLS																														
CELLMARKERCAN_SCHWANN_CELLS																														
CELLMARKERCAN_SEROTONERGIC_NEURONS																														
CELLMARKERCAN_SERTOLI_CELLS																														
CELLMARKERCAN_SMOOTH_MUSCLE_CELLS																														
CELLMARKERCAN_STROMAL_CELLS																														
CELLMARKERCAN_T_CELLS																														
CELLMARKERCAN_T_CYTOTOXIC_CELLS																														
CELLMARKERCAN_T_FOLLICULAR_HELPER_CELLS																														
CELLMARKERCAN_T_MEMORY_CELLS																														
CELLMARKERCAN_TRANSIENT_CELLS																														
CELLMARKERCAN_TRICHOCYTES																														
CELLMARKERCAN_TRIGEMINAL_NEURONS																														
CELLMARKERCAN_TROPHOBLAST_CELLS																														
CELLMARKERCAN_TROPHOBLAST_PROGENITOR_CELLS																														
CELLMARKERCAN_TUFT_CELLS																														
CELLMARKERCANENDOTHELIALCELLSAORTA																														
CELLMARKERCUR_N_ABNORMAL_MYELOID_CELL																														

CELLMARKERCAN_ACINAR_CELLS																														
CELLMARKERCUR_N_ABNORMAL_PLASMA_CELL																														
CELLMARKERCUR_N_ACTIVATED_DENDRITIC_CELL																														
CELLMARKERCUR_N_ACTIVATED_EFFECTOR_CELL																														
CELLMARKERCUR_N_ACTIVATED_HEPATIC_STELLAT…																														
CELLMARKERCUR_N_ACTIVATED_LANGERHANS_CELL																														
CELLMARKERCUR_N_ACTIVATED_MACROPHAGE																														
CELLMARKERCUR_N_ACTIVATED_MEMORY_T_CELL																														
CELLMARKERCUR_N_ACTIVATED_NATURAL_KILLER_…																														
CELLMARKERCUR_N_ACTIVATED_REGULATORY_T_CE…																														
CELLMARKERCUR_N_ADIPOCYTE_PROGENITOR_CELL																														
CELLMARKERCUR_N_ADRENAL_MEDULLA_CELL																														
CELLMARKERCUR_N_ADULT_SPERMATOGONIA																														
CELLMARKERCUR_N_ADVENTITIAL_CELL																														
CELLMARKERCUR_N_AEROCYTE																														
CELLMARKERCUR_N_AIRWAY_SMOOTH_MUSCLE_CELL																														
CELLMARKERCUR_N_ALVEOLAR_CELL_TYPE_1																														
CELLMARKERCUR_N_ALVEOLAR_CELL_TYPE_2																														
CELLMARKERCUR_N_ALVEOLAR_EPITHELIAL_CELL_…																														
CELLMARKERCUR_N_ALVEOLAR_EPITHELIAL_CELL_…																														
CELLMARKERCUR_N_ALVEOLAR_PNEUMOCYTE_TYPE_I																														
CELLMARKERCUR_N_ALVEOLAR_PNEUMOCYTE_TYPE_…																														
CELLMARKERCUR_N_AMACRINE_CELL																														
CELLMARKERCUR_N_AMNIOTIC_FLUID_STEM_CELL																														
CELLMARKERCUR_N_ANTERIOR_CELL																														
CELLMARKERCUR_N_ANTERIOR_HINDBRAIN_CELL																														
CELLMARKERCUR_N_ANTI_TUMOR_IMMUNE_CELL																														
CELLMARKERCUR_N_ANTIGEN_PRESENTATION_CANC…																														
CELLMARKERCUR_N_ASTROCYTE																														
CELLMARKERCUR_N_ATRIAL_CELL																														
CELLMARKERCUR_N_ATRIOVENTRICULAR_CANAL_CE…																														
CELLMARKERCUR_N_ATYPICAL_MEMORY_B_CELL																														
CELLMARKERCUR_N_B_2_CELL																														
CELLMARKERCUR_N_B1_CELL																														
CELLMARKERCUR_N_B10_REGULATORY_B_CELL																														
CELLMARKERCUR_N_BASAL_CELL																														
CELLMARKERCUR_N_BASAL_EPITHELIAL_CELL																														
CELLMARKERCUR_N_BASAL_KERATINOCYTE																														
CELLMARKERCUR_N_BASAL_LIKE_CELL																														
CELLMARKERCUR_N_BASAL_PROGENITOR_CELL																														
CELLMARKERCUR_N_BASOPHIL																														
CELLMARKERCUR_N_BILIARY_CELL																														
CELLMARKERCUR_N_BONE_MARROW_STEM_CELL																														
CELLMARKERCUR_N_BONE_SPECIFIC_CELL																														
CELLMARKERCUR_N_BRAIN_CELL																														
CELLMARKERCUR_N_CANCER_CELL	ELF3	CA9	WT1	HNF1B	EPS8L2	CHMP2A	CNDP2	PDCD6…	CEACA…	ZAR1	SYP	PHOX2B	TRIM63	PAX8	PAX2	FKBP4	POMC	NUF2	CHD1L	ACSL4	CTNNB1	MSRA	RUNX3	AMD1	ERBB4	ANGPT…	ROBO1	SLIT1	ROS1	RET
CELLMARKERCUR_N_CANCER_STEM_LIKE_CELL																														
CELLMARKERCUR_N_CAPILLARY_CELL																														
CELLMARKERCUR_N_CAR_T_CELL																														
CELLMARKERCUR_N_CARDIAC_CELL																														
CELLMARKERCUR_N_CARDIOMYOCYTE																														
CELLMARKERCUR_N_CARDIOSPHERE_DERIVED_CELL																														
CELLMARKERCUR_N_CARDIOVASCULAR_PROGENITOR…																														
CELLMARKERCUR_N_CAUDAL_CORTEX_CELL																														
CELLMARKERCUR_N_CD14_MONOCYTE																														
CELLMARKERCUR_N_CD16_MONOCYTE																														
CELLMARKERCUR_N_CD4_CD28_T_CELL																														
CELLMARKERCUR_N_CD4_PERIPHERALLY_DERIVED_…																														
CELLMARKERCUR_N_CD4_T_CELL																														
CELLMARKERCUR_N_CD56BRIGHT_NATURAL_KILLER…																														
CELLMARKERCUR_N_CD56DIM_NATURAL_KILLER_CE…																														
CELLMARKERCUR_N_CD8_T_CELL																														
CELLMARKERCUR_N_CENTRAL_MEMORY_T_CELL																														
CELLMARKERCUR_N_CENTRAL_MEMORY_T_HELPER_1…																														
CELLMARKERCUR_N_CENTRAL_NERVOUS_SYSTEM_ST…																														
CELLMARKERCUR_N_CHANDELIER_CELL																														
CELLMARKERCUR_N_CHIEF_CELL																														
CELLMARKERCUR_N_CHONDROCYTE																														
CELLMARKERCUR_N_CHROMAFFIN_CELL																														
CELLMARKERCUR_N_CIRCULATING_ANGIOGENIC_CE…																														
CELLMARKERCUR_N_CIRCULATING_FETAL_CELL																														
CELLMARKERCUR_N_CIRCULATING_NATURAL_KILLE…																														
CELLMARKERCUR_N_CIRCULATING_PRECURSOR_CELL																														
CELLMARKERCUR_N_CIRCULATING_PROGENITOR_CE…																														
CELLMARKERCUR_N_CIRCULATING_T_FOLLICULAR_…																														
CELLMARKERCUR_N_CIRCULATING_TUMOR_CELL																														
CELLMARKERCUR_N_CLASS_SWITCHED_PLASMABLAST																														
CELLMARKERCUR_N_CLASSICAL_B_CELL																														
CELLMARKERCUR_N_CLASSICAL_CANCER_ASSOCIAT…																														
CELLMARKERCUR_N_CLASSICAL_MONOCYTE																														
CELLMARKERCUR_N_CLAUDIN_LOW_CELL																														
CELLMARKERCUR_N_COLLECTING_DUCTAL_CELL																														
CELLMARKERCUR_N_COLONCYTE																														
CELLMARKERCUR_N_COLORECTAL_STEM_CELL																														
CELLMARKERCUR_N_COLUMNAR_EPITHELIAL_CELL																														
CELLMARKERCUR_N_COMMON_LYMPHOID_PROGENITO…																														
CELLMARKERCUR_N_COMMON_MYELOID_PROGENITOR…																														
CELLMARKERCUR_N_COMPLEMENT_SECRETING_CANC…																														
CELLMARKERCUR_N_CONE_PHOTORECEPTOR_CELL																														
CELLMARKERCUR_N_CONNECTING_TUBULAR_CELL																														
CELLMARKERCUR_N_CONVENTIONAL_CD4_T_CELL																														
CELLMARKERCUR_N_CORNEAL_ENDOTHELIAL_CELL																														
CELLMARKERCUR_N_CORNEAL_EPITHELIAL_CELL																														
CELLMARKERCUR_N_CORNEOCYTE_LIKE_CELL																														
CELLMARKERCUR_N_CORTICAL_CELL																														
CELLMARKERCUR_N_CORTICAL_STROMAL_CELL																														
CELLMARKERCUR_N_CORTICOTROPH_TUMOR_CELL																														
CELLMARKERCUR_N_CRANIAL_NEURAL_CREST_CELL																														
CELLMARKERCUR_N_CRYPT_CELL																														
CELLMARKERCUR_N_CRYPT_STEM_CELL																														
CELLMARKERCUR_N_CYCLING_B_CELL																														
CELLMARKERCUR_N_CYCLING_EPITHELIAL_CELL																														
CELLMARKERCUR_N_CYTOTOXIC_CD8_T_CELL																														
CELLMARKERCUR_N_CYTOTOXIC_DOUBLE_POSITIVE…																														
CELLMARKERCUR_N_CYTOTOXIC_T_CELL																														

CELLMARKERCAN_ACINAR_CELLS																														
CELLMARKERCUR_N_CYTOTOXIC_T_CELL_2																														
CELLMARKERCUR_N_DAMAGE_ASSOCIATED_TRANSIE…																														
CELLMARKERCUR_N_DARK_ZONE_CELL																														
CELLMARKERCUR_N_DEDIFFERENTIATION_ADIPOCY…																														
CELLMARKERCUR_N_DEEP_LAYER_NEURON																														
CELLMARKERCUR_N_DEFINITIVE_ENDODERM_CELL																														
CELLMARKERCUR_N_DELTA_CELL																														
CELLMARKERCUR_N_DEMYELINATING_MICROGLIAL_…																														
CELLMARKERCUR_N_DENDRITIC_CELL_2A																														
CELLMARKERCUR_N_DENDRITIC_CELL_LINEAGE																														
CELLMARKERCUR_N_DENTAL_PULP_CELL																														
CELLMARKERCUR_N_DERMAL_CELL																														
CELLMARKERCUR_N_DEUTEROSOMAL_CELL																														
CELLMARKERCUR_N_DIFFUSE_GASTRIC_CANCER_CE…																														
CELLMARKERCUR_N_DISTAL_CELL																														
CELLMARKERCUR_N_DISTAL_STEM_CELL																														
CELLMARKERCUR_N_DISTAL_TUBULAR_CELL																														
CELLMARKERCUR_N_DIVIDING_CELL																														
CELLMARKERCUR_N_DIVIDING_PLASMA_B_CELL																														
CELLMARKERCUR_N_DIVIDING_T_CELL																														
CELLMARKERCUR_N_DOPAMINERGIC_NEURON																														
CELLMARKERCUR_N_DORSAL_ANTERIOR_HINDBRAIN…																														
CELLMARKERCUR_N_DORSAL_LATERAL_GANGLIONIC…																														
CELLMARKERCUR_N_DORSAL_MEDIAL_GANGLIONIC_…																														
CELLMARKERCUR_N_DOUBLE_NEGATIVE_B_CELL																														
CELLMARKERCUR_N_DOUBLE_NEGATIVE_T_CELL																														
CELLMARKERCUR_N_DOUBLE_POSITIVE_T_CELL																														
CELLMARKERCUR_N_DOUBLE_POSITIVE_TREG_CELL																														
CELLMARKERCUR_N_EARLY_HEMANGIOBLAST																														
CELLMARKERCUR_N_EARLY_PROXIMAL_TUBULE_CELL																														
CELLMARKERCUR_N_EARLY_RESPONSE_SECRETORY_…																														
CELLMARKERCUR_N_EFFECTOR_CHONDROCYTE																														
CELLMARKERCUR_N_EFFECTOR_MEMORY_T_CELL																														
CELLMARKERCUR_N_EFFECTOR_NATURAL_KILLER_C…																														
CELLMARKERCUR_N_ELONGATING_CELL																														
CELLMARKERCUR_N_EMBRYONIC_CELL																														
CELLMARKERCUR_N_EMBRYONIC_STEM_CELL																														
CELLMARKERCUR_N_ENDOCARDIAL_CELL																														
CELLMARKERCUR_N_ENDOCRINE_PROGENITOR_CELL																														
CELLMARKERCUR_N_ENDODERM_PROGENITOR_CELL																														
CELLMARKERCUR_N_ENDODERMAL_CELL																														
CELLMARKERCUR_N_ENDOMETRIAL_STROMAL_CELL																														
CELLMARKERCUR_N_ENDOMETRIAL_STROMAL_STEM_…																														
CELLMARKERCUR_N_ENDOTHELIAL_PRECURSOR_CELL																														
CELLMARKERCUR_N_ENSHEATHING_GLIAL_CELL																														
CELLMARKERCUR_N_ENTERIC_NEURON																														
CELLMARKERCUR_N_EOSINOPHIL																														
CELLMARKERCUR_N_EPENDYMAL_CELL																														
CELLMARKERCUR_N_EPIBLAST_CELL																														
CELLMARKERCUR_N_EPICARDIAL_CELL																														
CELLMARKERCUR_N_EPICARDIUM_DERIVED_CELL																														
CELLMARKERCUR_N_EPIDERMAL_CELL																														
CELLMARKERCUR_N_EPIDERMAL_STEM_CELL																														
CELLMARKERCUR_N_EPITHELIAL_CELL	AGTR2	CCDC1…	TPPP3	AQP4	CYP2F1	SFTPA1	ACE2	EFHC1	FOXN4	CDC20B	SPRR2A	VMO1	SERPI…	IFITM3	IFIT1	FABP5	XBP1	TMSB4X	MLF1	TMEM1…	CAPS	TMC5	CHST9	TNIP3	MUC4	CP	ABCA3	CAV1	PRSS2	CLU
CELLMARKERCUR_N_EPITHELIAL_DUCTAL_CELL																														
CELLMARKERCUR_N_EPITHELIAL_MESENCHYMAL_CE…																														
CELLMARKERCUR_N_EPITHELIOID_MACROPHAGE																														
CELLMARKERCUR_N_EPSILON_CELL																														
CELLMARKERCUR_N_ERYTHROBLAST																														
CELLMARKERCUR_N_ERYTHROID_CELL																														
CELLMARKERCUR_N_ERYTHROID_PROGENITOR_CELL																														
CELLMARKERCUR_N_ESOPHAGEAL_CELL																														
CELLMARKERCUR_N_ESOPHAGEAL_PROGENITOR_CELL																														
CELLMARKERCUR_N_EXCITATORY_NEURON																														
CELLMARKERCUR_N_EXHAUSTED_DOUBLE_POSITIVE…																														
CELLMARKERCUR_N_EXTRAVILLOUS_TROPHOBLAST																														
CELLMARKERCUR_N_F_PERICYTE																														
CELLMARKERCUR_N_FETAL_MYOBLAST																														
CELLMARKERCUR_N_FIBROBLAST_LIKE_CELL																														
CELLMARKERCUR_N_FIBROCHONDROCYTE																														
CELLMARKERCUR_N_FIBROCHONDROCYTE_PROGENIT…																														
CELLMARKERCUR_N_FOAM_CELL																														
CELLMARKERCUR_N_FOLLICULAR_B_CELL																														
CELLMARKERCUR_N_FOLLICULAR_CELL																														
CELLMARKERCUR_N_FOLLICULAR_DENDRITIC_CELL																														
CELLMARKERCUR_N_FOREBRAIN_CELL																														
CELLMARKERCUR_N_GABAERGIC_NEURON																														
CELLMARKERCUR_N_GANGLION_CELL																														
CELLMARKERCUR_N_GASTRIC_NEUROENDOCRINE_CE…																														
CELLMARKERCUR_N_GASTRIC_STEM_CELL																														
CELLMARKERCUR_N_GERM_CELL																														
CELLMARKERCUR_N_GERMINAL_CENTER_B_CELL																														
CELLMARKERCUR_N_GERMINAL_CENTER_CELL																														
CELLMARKERCUR_N_GERMINAL_CENTRE_B_CELL																														
CELLMARKERCUR_N_GLIAL_CELL																														
CELLMARKERCUR_N_GLIOMA_STEM_CELL																														
CELLMARKERCUR_N_GLOBOSE_BASAL_CELL																														
CELLMARKERCUR_N_GLOMERULAR_PARIETAL_EPITH…																														
CELLMARKERCUR_N_GLUTAMATERGIC_NEURON																														
CELLMARKERCUR_N_GOBLET_CELL																														
CELLMARKERCUR_N_GONADOTROPE																														
CELLMARKERCUR_N_GONADOTROPH_CELL																														
CELLMARKERCUR_N_GRANULOCYTE																														
CELLMARKERCUR_N_GRANULOCYTE_MONOCYTE_PROG…																														
CELLMARKERCUR_N_GRANULOCYTIC_UNK																														
CELLMARKERCUR_N_GRANULOSA_CELL																														
CELLMARKERCUR_N_HAEMATOPOIETIC_CELL																														
CELLMARKERCUR_N_HAIR_FOLLICLE_CELL																														
CELLMARKERCUR_N_HEMANGIOBLAST																														
CELLMARKERCUR_N_HEMATOPOIETIC_CELL																														
CELLMARKERCUR_N_HEMATOPOIETIC_PRECURSOR_C…																														
CELLMARKERCUR_N_HEMATOPOIETIC_PROGENITOR_…																														
CELLMARKERCUR_N_HEPATIC_CELL																														
CELLMARKERCUR_N_HEPATIC_PROGENITOR_CELL																														

CELLMARKERCAN_ACINAR_CELLS																														
CELLMARKERCUR_N_HEPATIC_STELLATE_CELL																														
CELLMARKERCUR_N_HEPATOBLAST																														
CELLMARKERCUR_N_HEPATOCYTE																														
CELLMARKERCUR_N_HISTIOCYTE																														
CELLMARKERCUR_N_HMSC_TREATED_B_CELL																														
CELLMARKERCUR_N_HOFBAUER_CELL																														
CELLMARKERCUR_N_HOMEOSTATIC_CHONDROCYTE																														
CELLMARKERCUR_N_HOMEOSTATIC_MICROGLIAL_CE…																														
CELLMARKERCUR_N_HORIZONTAL_BASAL_CELL																														
CELLMARKERCUR_N_HORIZONTAL_CELL																														
CELLMARKERCUR_N_HYPOBLAST_CELL																														
CELLMARKERCUR_N_IDIOPATHIC_PULMONARY_FIBR…																														
CELLMARKERCUR_N_IGA_MEMORY_B_CELL																														
CELLMARKERCUR_N_IGG_MEMORY_B_CELL																														
CELLMARKERCUR_N_IGM_MEMORY_B_CELL																														
CELLMARKERCUR_N_IGM_ONLY_B_CELL																														
CELLMARKERCUR_N_IL7R_T_HELPER_CELL																														
CELLMARKERCUR_N_IMMATURE_ERYTHROID_CELL																														
CELLMARKERCUR_N_IMMATURE_MYELOID_CELL																														
CELLMARKERCUR_N_IMMATURE_NEUTROPHIL																														
CELLMARKERCUR_N_IMMATURE_TRANSITIONAL_B_C…																														
CELLMARKERCUR_N_IMMUNE_CELL																														
CELLMARKERCUR_N_INDUCED_PLURIPOTENT_STEM_…																														
CELLMARKERCUR_N_INFILTRATING_MACROPHAGE																														
CELLMARKERCUR_N_INFLAMMATORY_CANCER_ASSOC…																														
CELLMARKERCUR_N_INFLAMMATORY_DENDRITIC_CE…																														
CELLMARKERCUR_N_INFLAMMATORY_MONOCYTE																														
CELLMARKERCUR_N_INHIBITORY_NEURON																														
CELLMARKERCUR_N_INNATE_LYMPHOID_CELL																														
CELLMARKERCUR_N_INNER_CELL_MASS_CELL																														
CELLMARKERCUR_N_INNER_RADIAL_GLIAL_CELL																														
CELLMARKERCUR_N_INTER_MEDIATE_PROGENITOR_…																														
CELLMARKERCUR_N_INTERCALATED_CELL																														
CELLMARKERCUR_N_INTERFERON_RESPONSIVE_SEC…																														
CELLMARKERCUR_N_INTERMEDIATE_MONOCYTE																														
CELLMARKERCUR_N_INTERMEDIATE_MONOCYTE_CELL																														
CELLMARKERCUR_N_INTERSTITIAL_MACROPHAGE																														
CELLMARKERCUR_N_INTERSTITIAL_PROGENITOR_C…																														
CELLMARKERCUR_N_INTESTINAL_EPITHELIAL_STE…																														
CELLMARKERCUR_N_INTESTINAL_GASTRIC_CANCER…																														
CELLMARKERCUR_N_INTESTINAL_METAPLASIA_CELL																														
CELLMARKERCUR_N_INTRAEPITHELIAL_LYMPHOCYT…																														
CELLMARKERCUR_N_IONOCYTE																														
CELLMARKERCUR_N_IRIS_PUTTATIVE_STEM_CELL																														
CELLMARKERCUR_N_KUPFFER_CELL																														
CELLMARKERCUR_N_LACTOTROPH_CELL																														
CELLMARKERCUR_N_LANGERHANS_CELL																														
CELLMARKERCUR_N_LEUKOCYTE																														
CELLMARKERCUR_N_LIMBAL_EPITHELIAL_STEM_CE…																														
CELLMARKERCUR_N_LIMBAL_STEM_CELL																														
CELLMARKERCUR_N_LIPOFIBROBLAST																														
CELLMARKERCUR_N_LIVER_PROGENITOR_CELL																														
CELLMARKERCUR_N_LIVER_STEM_CELL																														
CELLMARKERCUR_N_LMMLEYDIG_CELL																														
CELLMARKERCUR_N_LONOCYTE																														
CELLMARKERCUR_N_LOOP_OF_HENLE_CELL																														
CELLMARKERCUR_N_LOOP_PROGENITOR_CELL																														
CELLMARKERCUR_N_LOW_DENSITY_NEUTROPHIL																														
CELLMARKERCUR_N_LUMINAL_PROGENITOR_CELL																														
CELLMARKERCUR_N_LUNG_CELL																														
CELLMARKERCUR_N_LYMPH_NODE_CELL																														
CELLMARKERCUR_N_LYMPHATIC_CELL																														
CELLMARKERCUR_N_LYMPHATIC_ENDOTHELIAL_CELL																														
CELLMARKERCUR_N_LYMPHOBLASTOID_CELL																														
CELLMARKERCUR_N_LYMPHOCYTE																														
CELLMARKERCUR_N_LYMPHOID_CELL																														
CELLMARKERCUR_N_LYMPHOID_PROGENITOR_CELL																														
CELLMARKERCUR_N_LYMPHOID_STRUCTURE_CELL																														
CELLMARKERCUR_N_M_PERICYTE																														
CELLMARKERCUR_N_M1_MACROPHAGE																														
CELLMARKERCUR_N_M1_MICROGLIAL_CELL																														
CELLMARKERCUR_N_M2_MACROPHAGE																														
CELLMARKERCUR_N_MACROGLIAL_CELL																														
CELLMARKERCUR_N_MALIGNANT_CELL																														
CELLMARKERCUR_N_MALIGNANT_PLASMA_CELL																														
CELLMARKERCUR_N_MARGINAL_ASSOCIATED_MACRO…																														
CELLMARKERCUR_N_MAST_CELL																														
CELLMARKERCUR_N_MATURE_ASTROCYTE																														
CELLMARKERCUR_N_MATURE_B_CELL																														
CELLMARKERCUR_N_MATURE_DENDRITIC_CELL																														
CELLMARKERCUR_N_MATURE_ENDOTHELIAL_CELL																														
CELLMARKERCUR_N_MATURE_LUMINAL_CELL																														
CELLMARKERCUR_N_MATURE_NEURON																														
CELLMARKERCUR_N_MATURE_NEURONAL																														
CELLMARKERCUR_N_MATURE_OLIGODENDROCYTE																														
CELLMARKERCUR_N_MATURE_OSTEOBLAST																														
CELLMARKERCUR_N_MATURE_PODOCYTE																														
CELLMARKERCUR_N_MEDIAL_AND_DISTAL_SEGMENT…																														
CELLMARKERCUR_N_MEDIAL_GANGLIONIC_EMINENC…																														
CELLMARKERCUR_N_MEGAKARYOCYTE																														
CELLMARKERCUR_N_MEGAKARYOCYTE_ERYTHROID_C…																														
CELLMARKERCUR_N_MEGAKARYOCYTE_ERYTHROID_P…																														
CELLMARKERCUR_N_MEGAKARYOCYTE_PROGENITOR_…																														
CELLMARKERCUR_N_MELANOMA_CELL																														
CELLMARKERCUR_N_MEMORY_B_CELL																														
CELLMARKERCUR_N_MEMORY_B_CELL_PRECURSOR																														
CELLMARKERCUR_N_MEMORY_CD27_B_CELL																														
CELLMARKERCUR_N_MEMORY_CD4_T_CELL																														
CELLMARKERCUR_N_MEMORY_DOUBLE_POSITIVE_T_…																														
CELLMARKERCUR_N_MEMORY_REGULATORY_T_CELL																														
CELLMARKERCUR_N_MEMORY_T_CELL																														
CELLMARKERCUR_N_MERKEL_CELL																														
CELLMARKERCUR_N_MESENCHYMAL_CELL																														
CELLMARKERCUR_N_MESENCHYMAL_EPITHELIAL_TR…																														

CELLMARKERCAN_ACINAR_CELLS																														
CELLMARKERCUR_N_MESENCHYMAL_GLIOBLASTOMA_…																														
CELLMARKERCUR_N_MESENCHYMAL_STEM_CELL																														
CELLMARKERCUR_N_MESENCHYMAL_STROMAL_CELL																														
CELLMARKERCUR_N_MESENDODERMAL_CELL																														
CELLMARKERCUR_N_MESODERMAL_EMBRYONIC_STEM…																														
CELLMARKERCUR_N_MESODERMAL_PROGENITOR_CELL																														
CELLMARKERCUR_N_METAPLASTIC_STEM_CELL																														
CELLMARKERCUR_N_MEYLOID_CELL																														
CELLMARKERCUR_N_MICROGLIAL_CELL	BAIAP…	MYO5A	NDUFA1	PGAM1	SERPI…	RPS24	TNFSF8	SMIM3	PTPRG	SCPEP1	DPYD	EEF1B2	KCNJ5	ATOX1	EPHX1	RPL32	SH3PX…	RPL39	MYO1E	PDCD6…	RPS14	FABP3	RPL18A	IL4I1	FABP5	SDF4	TGFBI	HCAR2	GAS2L1	ANKRD…
CELLMARKERCUR_N_MICROVILLAR_CELL																														
CELLMARKERCUR_N_MIDBRAIN_DOPAMINERGIC_NEU…																														
CELLMARKERCUR_N_MONO_MACROPHAGE																														
CELLMARKERCUR_N_MONOCYTE_DERIVED_CELL																														
CELLMARKERCUR_N_MONOCYTE_DERIVED_MYELOID_…																														
CELLMARKERCUR_N_MONOCYTE_LINEAGE																														
CELLMARKERCUR_N_MONOCYTE_PRECURSOR																														
CELLMARKERCUR_N_MONOCYTE_PROGENITOR_CELL																														
CELLMARKERCUR_N_MUELLER_GLIA_CELL																														
CELLMARKERCUR_N_MULTIPOTENT_MESENCHYMAL_S…																														
CELLMARKERCUR_N_MULTIPOTENT_RETINAL_PROGE…																														
CELLMARKERCUR_N_MULTIPOTENT_STEM_CELL																														
CELLMARKERCUR_N_MULTIPOTENT_STROMAL_CELL																														
CELLMARKERCUR_N_MUSCLE_CELL																														
CELLMARKERCUR_N_MUSCLE_DERIVED_CELL																														
CELLMARKERCUR_N_MUSCLE_SATELLITE_CELL																														
CELLMARKERCUR_N_MYELOBLAST																														
CELLMARKERCUR_N_MYELOID_DENDRITIC_CELL																														
CELLMARKERCUR_N_MYELOID_DENDRITIC_CELL_1																														
CELLMARKERCUR_N_MYELOID_DERIVED_CELL																														
CELLMARKERCUR_N_MYELOID_DERIVED_SUPPRESSO…																														
CELLMARKERCUR_N_MYELOID_PROGENITOR_CELL																														
CELLMARKERCUR_N_MYO_EPITHELIAL_CELL																														
CELLMARKERCUR_N_MYOBLAST																														
CELLMARKERCUR_N_MYOCYTE																														
CELLMARKERCUR_N_MYOFIBROBLAST																														
CELLMARKERCUR_N_MYOGENIC_ENDOTHELIAL_CELL																														
CELLMARKERCUR_N_MYOGENIC_PROGENITOR_CELL																														
CELLMARKERCUR_N_MYOID_CELL																														
CELLMARKERCUR_N_NAIVE_B_CELL																														
CELLMARKERCUR_N_NAIVE_CD8_T_CELL																														
CELLMARKERCUR_N_NAIVE_DOUBLE_POSITIVE_T_C…																														
CELLMARKERCUR_N_NAIVE_PROGENITOR_CELL																														
CELLMARKERCUR_N_NAIVE_T_CELL																														
CELLMARKERCUR_N_NASCENT_NEPHRON_CELL																														
CELLMARKERCUR_N_NATURAL_KILLER_CD56_BRIGH…																														
CELLMARKERCUR_N_NATURAL_KILLER_CD56_DIM_C…																														
CELLMARKERCUR_N_NEGATIVE_MESENCHYMAL_STEM…																														
CELLMARKERCUR_N_NEURAL_CELL																														
CELLMARKERCUR_N_NEURAL_CREST_STEM_CELL																														
CELLMARKERCUR_N_NEURAL_CREST_STEMLIKE_CELL																														
CELLMARKERCUR_N_NEURAL_PRECURSOR_CELL																														
CELLMARKERCUR_N_NEURAL_STEM_CELL																														
CELLMARKERCUR_N_NEUROECTODERM_CELL																														
CELLMARKERCUR_N_NEURONAL_INTERMEDIATE_PRO…																														
CELLMARKERCUR_N_NEURONAL_PROGENITOR_CELL																														
CELLMARKERCUR_N_NEUTROPHIL_PROGENITOR_CELL																														
CELLMARKERCUR_N_NON_NEURON																														
CELLMARKERCUR_N_NON_PT_TUBULAR_CELL																														
CELLMARKERCUR_N_NON_RECIRCULATING_TISSUE_…																														
CELLMARKERCUR_N_NONMYELINATING_SCHWANN_CE…																														
CELLMARKERCUR_N_NOTOCHORD_CELL																														
CELLMARKERCUR_N_NUCLEATED_CELL																														
CELLMARKERCUR_N_NUCLEUS_PULPOSUS_PROGENIT…																														
CELLMARKERCUR_N_OLIGODENDROCYTE																														
CELLMARKERCUR_N_OSTEOBLAST																														
CELLMARKERCUR_N_OUTER_RADIAL_GLIA																														
CELLMARKERCUR_N_OVALBUMIN_SPECIFIC_REGULA…																														
CELLMARKERCUR_N_OVARIAN_CELL																														
CELLMARKERCUR_N_OVARIAN_STROMAL_CELL																														
CELLMARKERCUR_N_P53_LIKE_CELL																														
CELLMARKERCUR_N_PAN_B_CELL																														
CELLMARKERCUR_N_PAN_CANCER_ASSOCIATED_FIB…																														
CELLMARKERCUR_N_PAN_MACROPHAGE																														
CELLMARKERCUR_N_PAN_MONOCYTE																														
CELLMARKERCUR_N_PAN_NEURONAL_CELL																														
CELLMARKERCUR_N_PAN_TROPHECTODERM_CELL																														
CELLMARKERCUR_N_PANCREATIC_ENDODERM_CELL																														
CELLMARKERCUR_N_PANCREATIC_POLYPEPTIDE_CE…																														
CELLMARKERCUR_N_PANCREATIC_PROGENITOR_CELL																														
CELLMARKERCUR_N_PANCREATIC_STEM_CELL																														
CELLMARKERCUR_N_PANETH_CELL																														
CELLMARKERCUR_N_PANNEURON																														
CELLMARKERCUR_N_PANVASCULAR_CELL																														
CELLMARKERCUR_N_PAPILLARY_FIBROBLAST																														
CELLMARKERCUR_N_PARIETAL_CELL																														
CELLMARKERCUR_N_PARVALBUMIN_INTERNEURON																														
CELLMARKERCUR_N_PATELET																														
CELLMARKERCUR_N_PATHOGENIC_T_PERIPHERAL_H…																														
CELLMARKERCUR_N_PELVIC_EPITHELIAL_CELL																														
CELLMARKERCUR_N_PERICYTE																														
CELLMARKERCUR_N_PERIODONTAL_LIGAMENT_STEM…																														
CELLMARKERCUR_N_PERIOSTEUM_DERIVED_PROGEN…																														
CELLMARKERCUR_N_PERITUBULAR_MYOID_CELL																														
CELLMARKERCUR_N_PERIVASCULAR_LIKE_CELL																														
CELLMARKERCUR_N_PHAGOCYTE																														
CELLMARKERCUR_N_PHOTORECEPTOR_CELL																														
CELLMARKERCUR_N_PIT_CELL																														
CELLMARKERCUR_N_PIT_MUCOUS_CELL																														
CELLMARKERCUR_N_PITUITARY_STEM_CELL																														
CELLMARKERCUR_N_PLACENTAL_TISSUE_CELL																														
CELLMARKERCUR_N_PLACODE_CELL																														
CELLMARKERCUR_N_PLASMABLAST_B_CELL																														
CELLMARKERCUR_N_PLATELET																														
CELLMARKERCUR_N_PLURIPOTENT_STEM_CELL																														

Table S5: Cell state marker gene list

CELLMARKERCAN_ACINAR_CELLS																														
CELLMARKERCUR_N_PODOCYTE_PRECURSOR																														
CELLMARKERCUR_N_PORTAL_FIBROBLAST																														
CELLMARKERCUR_N_POSTERIOR_CELL																														
CELLMARKERCUR_N_PRE_B_CELL																														
CELLMARKERCUR_N_PRE_CONVENTIONAL_DENDRITI…																														
CELLMARKERCUR_N_PRE_DENDRITIC_CELL																														
CELLMARKERCUR_N_PRECURSOR_CELL																														
CELLMARKERCUR_N_PREHYPERTROPHIC_CHONDROCY…																														
CELLMARKERCUR_N_PRESOMITE_CELL																														
CELLMARKERCUR_N_PRETUBULAR_AGGREGATE_CELL																														
CELLMARKERCUR_N_PRIMITIVE_ENDODERM_CELL																														
CELLMARKERCUR_N_PRINCIPAL_CELL																														
CELLMARKERCUR_N_PRO_B_CELL																														
CELLMARKERCUR_N_PRO_INFLAMMATORY_MACROPHA…																														
CELLMARKERCUR_N_PRO_NEUTROPHIL																														
CELLMARKERCUR_N_PROLIFERATING_CELL																														
CELLMARKERCUR_N_PROLIFERATIVE_CHONDROCYTE																														
CELLMARKERCUR_N_PROLIFERATIVE_DUCTAL_CELL																														
CELLMARKERCUR_N_PROLIFERATIVE_NEURAL_PROG…																														
CELLMARKERCUR_N_PRONEURAL_GLIOBLASTOMA_CE…																														
CELLMARKERCUR_N_PROSPERMATOGONIA																														
CELLMARKERCUR_N_PROSTATE_CELL																														
CELLMARKERCUR_N_PROXIMAL_CELL																														
CELLMARKERCUR_N_PROXIMAL_SEGMENT_CELL																														
CELLMARKERCUR_N_PROXIMAL_STEM_CELL																														
CELLMARKERCUR_N_PROXIMAL_TUBULE_CELL																														
CELLMARKERCUR_N_PULMONARY_NEUROENDOCRINE_…																														
CELLMARKERCUR_N_PYRAMIDAL_CELL																														
CELLMARKERCUR_N_QUIESCENT_T_STEM_CELL																														
CELLMARKERCUR_N_REGULATORY_B_CELL																														
CELLMARKERCUR_N_REGULATORY_INNATE_LYMPHOI…																														
CELLMARKERCUR_N_RENAL_TUBULAR_CELL																														
CELLMARKERCUR_N_RESIDENT_MEMORY_T_CELL																														
CELLMARKERCUR_N_RESPIRATORY_HORIZONTAL_BA…																														
CELLMARKERCUR_N_RESPIRATORY_SECRETORY_CELL																														
CELLMARKERCUR_N_RESPONDER_T_CELL																														
CELLMARKERCUR_N_RESPONDING_CONVENTIONAL_T…																														
CELLMARKERCUR_N_RESTING_MEMORY_B_CELL																														
CELLMARKERCUR_N_RESTING_NAIVE_B_CELL																														
CELLMARKERCUR_N_RESTING_REGULATORY_T_CELL																														
CELLMARKERCUR_N_RETICULAR_FIBROBLAST																														
CELLMARKERCUR_N_RETINA_CELL																														
CELLMARKERCUR_N_ROD_CELL																														
CELLMARKERCUR_N_SATELLITE_CELL																														
CELLMARKERCUR_N_SEPTUM_TRANSVERSUM_MESENC…																														
CELLMARKERCUR_N_SEROUS_CELL																														
CELLMARKERCUR_N_SIDE_POPULATION_CELL																														
CELLMARKERCUR_N_SKELETAL_MUSCLE_CELL																														
CELLMARKERCUR_N_SKELETAL_MUSCLE_STEM_CELL																														
CELLMARKERCUR_N_SMOOTH_MUSCLE_CELL																														
CELLMARKERCUR_N_SMOOTH_MUSCLE_LIKE_CELL																														
CELLMARKERCUR_N_SOMATIC_CELL																														
CELLMARKERCUR_N_SOMATIC_STEM_CELL																														
CELLMARKERCUR_N_SOMATOSTATIN_INTERNEURON																														
CELLMARKERCUR_N_SPECIALIST_ANTIGEN_PRESEN…																														
CELLMARKERCUR_N_SPERMATOGENIC_CELL																														
CELLMARKERCUR_N_SPERMATOGONIAL_CELL																														
CELLMARKERCUR_N_SPERMATOGONIAL_STEM_CELL																														
CELLMARKERCUR_N_SQUAMOUS_DIFFERENTIATION_…																														
CELLMARKERCUR_N_STELLATE_CELL																														
CELLMARKERCUR_N_STEM_CELL_DERIVED_ALPHA_C…																														
CELLMARKERCUR_N_STEM_CELL_DERIVED_BETA_CE…																														
CELLMARKERCUR_N_STEM_CELL_DERIVED_ENDOTHE…																														
CELLMARKERCUR_N_STEM_LIKE_CD8_T_CELL																														
CELLMARKERCUR_N_STROMAL_CELL																														
CELLMARKERCUR_N_SUBPALLIUM_CELL																														
CELLMARKERCUR_N_SUBPLATE_CELL																														
CELLMARKERCUR_N_SUPPRESSIVE_MONOCYTE																														
CELLMARKERCUR_N_SUPPRESSOR_T_CELL																														
CELLMARKERCUR_N_SUPRABASAL_CELL																														
CELLMARKERCUR_N_SURFACE_ECTODERM_CELL																														
CELLMARKERCUR_N_SUSTENTACULAR_CELL																														
CELLMARKERCUR_N_SYMPATHOADRENAL_CELL																														
CELLMARKERCUR_N_SYNCYTIOTROPHOBLAST																														
CELLMARKERCUR_N_SYNTHETIC_SMOOTH_MUSCLE_C…																														
CELLMARKERCUR_N_T_CELL_LARGE_GRANULAR_LYM…																														
CELLMARKERCUR_N_T_CELL_LINEAGE																														
CELLMARKERCUR_N_T_HELPER_CELL																														
CELLMARKERCUR_N_T_MEMORY_STEM_CELL																														
CELLMARKERCUR_N_T2_MZP_REGULATORY_B_CELL																														
CELLMARKERCUR_N_TENDON_CELL																														
CELLMARKERCUR_N_TERMINAL_DIFFERENTIATED_B…																														
CELLMARKERCUR_N_THALAMIC_CELL																														
CELLMARKERCUR_N_THECA_INTERNA_CELL																														
CELLMARKERCUR_N_THYMIC_EPITHELIAL_CELL																														
CELLMARKERCUR_N_TISSUE_LIKE_MEMORY_B_CELL																														
CELLMARKERCUR_N_TISSUE_RESIDENT_CELL																														
CELLMARKERCUR_N_TISSUE_RESIDENT_EFFECTOR_…																														
CELLMARKERCUR_N_TISSUE_RESIDENT_MACROPHAGE																														
CELLMARKERCUR_N_TISSUE_RESIDENT_MEMORY_LI…																														
CELLMARKERCUR_N_TOLDC_INDUCED_REGULATORY_…																														
CELLMARKERCUR_N_TONSIL_CELL																														
CELLMARKERCUR_N_TRABECULAR_MESHWORK_CELL																														
CELLMARKERCUR_N_TRANSITIONAL_B_CELL																														
CELLMARKERCUR_N_TRANSITIONAL_DENDRITIC_CE…																														
CELLMARKERCUR_N_TRUNCATED_RADIAL_GLIAL_CE…																														
CELLMARKERCUR_N_TUBULAR_CELL																														
CELLMARKERCUR_N_TUMOR_ASSOCIATED_MICROGLI…																														
CELLMARKERCUR_N_TUMOR_ENDOTHELIAL_CELL																														
CELLMARKERCUR_N_TUMOR_EPITHELIAL_CELL																														
CELLMARKERCUR_N_TUMOR_INFILTRATING_REGULA…																														
CELLMARKERCUR_N_TUMOR_INFILTRATING_T_CELL																														
CELLMARKERCUR_N_TUMOR_INITIATING_CELL																														
CELLMARKERCUR_N_TUMOR_REGULATORY_T_CELL																														

CELLMARKERCAN_ACINAR_CELLS																														
CELLMARKERCUR_N_UMBRELLA_CELL																														
CELLMARKERCUR_N_UNRESTRICTED_SOMATIC_STEM…																														
CELLMARKERCUR_N_UNSWITCHED_PLASMABLAST																														
CELLMARKERCUR_N_URETERIC_BUD_CELL																														
CELLMARKERCUR_N_UROTHELIAL_CELL																														
CELLMARKERCUR_N_UROTHELIUM_CELL																														
CELLMARKERCUR_N_VASCULATURE_CELL																														
CELLMARKERCUR_N_VEIN_CELL																														
CELLMARKERCUR_N_VENOUS_CELL																														
CELLMARKERCUR_N_VENTRAL_LATERAL_GANGLIONI…																														
CELLMARKERCUR_N_VENTRAL_MEDIAL_GANGLIONIC…																														
CELLMARKERCUR_N_VENTRICULAR_CELL																														
CELLMARKERCUR_N_YOUNG_NEURON																														
CELLMARKERCUR_N_ZONA_FASCICULATA_CELL																														
CELLMARKERCUR_N_ZONA_GLOMERULOSA_CELL																														
CELLMARKERCUR_N_ZONA_RETICULARIS_CELL																														
CELLMARKERCUR_N_ZYGOTE																														
CELLMARKERCURNACTIVATEDEFFECTORMEMORYCD4T…																														
CELLMARKERCURNACTIVATEDTISSUERESIDENTMEMO…																														
CELLMARKERCURNACTIVATEDTISSUERESIDENTMEMO…																														
CELLMARKERCURNALPHACELLACELL																														
CELLMARKERCURNALVEOLARTYPEIIATIICELL																														
CELLMARKERCURNATYPICALBCELLABC																														
CELLMARKERCURNAXLCD11CPLASMACYTOIDDENDRIT…																														
CELLMARKERCURNAXLCD123PLASMACYTOIDDENDRIT…																														
CELLMARKERCURNBASALFOREBRAINCHOLINERGICNE…																														
CELLMARKERCURNBRUSHCELLTUFTCELL																														
CELLMARKERCURNCANCERASSOCIATEDFIBROBLASTC…																														
CELLMARKERCURNCAPSULARFIBROBLASTFB2																														
CELLMARKERCURNCCR7DENDRITICCELL																														
CELLMARKERCURNCD106MESENCHYMALSTEMCELL																														
CELLMARKERCURNCD14MACROPHAGE																														
CELLMARKERCURNCD16DENDRITICCELL																														
CELLMARKERCURNCD25LAG3TCELL																														
CELLMARKERCURNCD34GRANULOCYTICPROGENITORC…																														
CELLMARKERCURNCD34HEMATOPOIETICSTEMCELL																														
CELLMARKERCURNCD34PREBCELL																														
CELLMARKERCURNCD34PROGENITORCELL																														
CELLMARKERCURNCD34THYMOCYTE																														
CELLMARKERCURNCD40LGTHELPERCELL																														
CELLMARKERCURNCD4CD25REGULATORYTCELL																														
CELLMARKERCURNCD4CENTRALMEMORYLIKETTCMLIK…																														
CELLMARKERCURNCD4CYTOTOXICT1CELL																														
CELLMARKERCURNCD4CYTOTOXICT2CELL																														
CELLMARKERCURNCD4THELPERCELL																														
CELLMARKERCURNCHRONICALLYACTIVATEDCD4TCELL																														
CELLMARKERCURNCHRONICALLYACTIVATEDCD8TCELL																														
CELLMARKERCURNCLASSSWITCHEDMEMORYBIGGU03B…																														
CELLMARKERCURNCLEC10ADENDRITICCELL																														
CELLMARKERCURNCLEC9ADENDRITICCELL																														
CELLMARKERCURNCONVENTIONALDENDRITICCELL2B…																														
CELLMARKERCURNCONVENTIONALDENDRITICCELL2B…																														
CELLMARKERCURNCORTICALSOMATOSTATINSSTINTE…																														
CELLMARKERCURNCYTOTOXICCD4T2CELL																														
CELLMARKERCURNCYTOTOXICCD4TCELL																														
CELLMARKERCURND1MSNMEDIUMSPINYNEURON																														
CELLMARKERCURND2MSNMEDIUMSPINYNEURON																														
CELLMARKERCURNDISTALTUBULELOOPOFHENLECELL																														
CELLMARKERCURNEARLYCD34MEGAKARYOCYTEERYTH…																														
CELLMARKERCURNEARLYTCELLPROGENITORETP																														
CELLMARKERCURNEFFECTORMEMORYCD4TCELL																														
CELLMARKERCURNEFFECTORREGULATORYTETREGCELL																														
CELLMARKERCURNEFFECTORTTEFFCELL																														
CELLMARKERCURNEXHAUSTEDCD4TCELL																														
CELLMARKERCURNEXHAUSTEDCD8TCELL																														
CELLMARKERCURNEXHAUSTEDCD8TPROGENITORCELL																														
CELLMARKERCURNEXHAUSTEDTTEXCELL																														
CELLMARKERCURNEXTRATELENCEPHALICETCELL																														
CELLMARKERCURNFINALLYHIGHLYEFFECTORTEMRAM…																														
CELLMARKERCURNFOLLICULARREGULATORYTTFRCELL																														
CELLMARKERCURNFOXP3NATURALREGULATORYTTREG…																														
CELLMARKERCURNGAMMADELTAU03B3DTCELL																														
CELLMARKERCURNGERMINALCENTREGCLIKECELL																														
CELLMARKERCURNGLOMERULARENDOTHELIALCELLECG																														
CELLMARKERCURNGRANULOCYTICMYELOIDDERIVEDS…																														
CELLMARKERCURNGRBREGULATORYBCELL																														
CELLMARKERCURNGZMKTCELL																														
CELLMARKERCURNHIGHLYDIFFERENTIATEDEFFECTO…																														
CELLMARKERCURNINDUCEDREGULATORYTTREGCELL																														
CELLMARKERCURNINFLAMMATORYCANCERASSOCIATE…																														
CELLMARKERCURNINNATELYMPHOIDCELLILCS																														
CELLMARKERCURNINTRATHYMICFIBROBLASTFB1																														
CELLMARKERCURNITOCELLHEPATICSTELLATECELL																														
CELLMARKERCURNLYMPHANGIOLEIOMYOMATOSISCEL…																														
CELLMARKERCURNMARGINALZONEMZLIKEBCELL																														
CELLMARKERCURNMEMORYSTEMCELLTSCM																														
CELLMARKERCURNMICROGLIADERIVEDTUMORASSOCI…																														
CELLMARKERCURNMKERYTHCELL																														
CELLMARKERCURNMYOFIBROBLASTICCANCERASSOCI…																														
CELLMARKERCURNNAIVEREGULATORYTTREGCELL																														
CELLMARKERCURNNATURALREGULATORYTTREGCELL																														
CELLMARKERCURNPERIPHERALBLOODMONONUCLEARC…																														
CELLMARKERCURNPITUITARYSTEMCELLPSC																														
CELLMARKERCURNPLASMACYTOIDDENDRITICCELLPDC																														
CELLMARKERCURNPOLYMORPHONUCLEARMYELOIDDER…																														
CELLMARKERCURNPOLYMORPHONUCLEARNEUTROPHIL…																														
CELLMARKERCURNPOSTERIORPRESOMITICMESODERM…																														
CELLMARKERCURNPROLIFERATIVECD4TCELL																														
CELLMARKERCURNPROLIFERATIVECD8TCELL																														
CELLMARKERCURNREDBLOODCELLERYTHROCYTE																														
CELLMARKERCURNREGULATORYBBREGCELL																														
CELLMARKERCURNREGULATORYCD4TCELL																														
CELLMARKERCURNREGULATORYCD8TCELL																														
CELLMARKERCURNREGULATORYTTREGCELL																														

Table S5: Cell state marker gene list

CELLMARKERCAN_ACINAR_CELLS																														
CELLMARKERCURNREGULATORYTTREGCELL																														
CELLMARKERCURNRENALVESICLECOMMASHAPEDBODY…																														
CELLMARKERCURNRESIDENTMEMORYCD8TCELL																														
CELLMARKERCURNREVERTANTMEMORYCD8TTEMRACELL																														
CELLMARKERCURNSCARASSOCIATEDMACROPHAGESAM																														
CELLMARKERCURNSENESCENTCD8TCELL																														
CELLMARKERCURNSTEMPROGENITORCELL																														
CELLMARKERCURNSWITCHEDMEMORYBCELLSWMB																														
CELLMARKERCURNT1TRANSITIONALBCELL																														
CELLMARKERCURNT2TRANSITIONALBCELL																														
CELLMARKERCURNTCELLNATURALKILLERCELL																														
CELLMARKERCURNTERMINALEFFECTORCD8TCELL																														
CELLMARKERCURNTERMINALLYDIFFERENTIATEDCD8…																														
CELLMARKERCURNTFOLLICULARHELPERTFHCELL																														
CELLMARKERCURNTHELPER1TH1CELL																														
CELLMARKERCURNTHELPER1TH1CELL																														
CELLMARKERCURNTHELPER2TH2CELL																														
CELLMARKERCURNTHELPER2TH2CELL																														
CELLMARKERCURNTHELPER9TH9CELL																														
CELLMARKERCURNTHELPERTHCELL																														
CELLMARKERCURNTHYMICEPITHELIALCELLTEC																														
CELLMARKERCURNTISSUERESIDENTMEMORYCD4TCELL																														
CELLMARKERCURNTISSUERESIDENTMEMORYCD8TCELL																														
CELLMARKERCURNTISSUERESIDENTMEMORYTTRMCELL																														
CELLMARKERCURNTPERIPHERALHELPERTPHCELL																														
CELLMARKERCURNTRANSIENTAMPLIFYINGTACELL																														
CELLMARKERCURNTRANSITIONALEXHAUSTEDCD8TCE…																														
CELLMARKERCURNTUMORINFILTRATINGLYMPHOCYTE…																														
CELLMARKERCURNUNSWITCHEDMEMORYBCELLUNSWMB																														
CELLMARKERCURNVASARECTAENDOTHELIALCELLECVR																														
CELLMARKERCURNVONECONOMONEURONVEN																														
CELLMARKERCURNYOLKSACDERIVEDMYELOIDBIASED…																														
CELLMARKEREXP_ACINAR_CELLS																														
CELLMARKEREXP_ADRENERGIC_NEURONS																														
CELLMARKEREXP_AIRWAY_EPITHELIAL_CELLS																														
CELLMARKEREXP_AIRWAY_SMOOTH_MUSCLE_CELLS																														
CELLMARKEREXP_ALPHA_CELLS																														
CELLMARKEREXP_ANTERIOR_PITUITARY_GLAND_CE…																														
CELLMARKEREXP_ASTROCYTES																														
CELLMARKEREXP_B_CELLS																														
CELLMARKEREXP_B_CELLS_MEMORY																														
CELLMARKEREXP_B_CELLS_NAIVE																														
CELLMARKEREXP_BASAL_CELLS																														
CELLMARKEREXP_BASOPHILS																														
CELLMARKEREXP_BERGMANN_GLIA																														
CELLMARKEREXP_BETA_CELLS																														
CELLMARKEREXP_CAJAL_RETZIUS_CELLS																														
CELLMARKEREXP_CHOLANGIOCYTES																														
CELLMARKEREXP_CHOLINERGIC_NEURONS																														
CELLMARKEREXP_CHONDROCYTES																														
CELLMARKEREXP_DELTA_CELLS																														
CELLMARKEREXP_DISTAL_TUBULE_CELLS																														
CELLMARKEREXP_DUCTAL_CELLS																														
CELLMARKEREXP_EMBRYONIC_STEM_CELLS																														
CELLMARKEREXP_ENTERIC_NEURONS																														
CELLMARKEREXP_ENTEROENDOCRINE_CELLS																														
CELLMARKEREXP_EPENDYMAL_CELLS																														
CELLMARKEREXP_EPSILON_CELLS																														
CELLMARKEREXP_ERYTHROID_LIKE_AND_ERYTHROI…																														
CELLMARKEREXP_GAMMA_DELTA_T_CELLS																														
CELLMARKEREXP_GOBLET_CELLS																														
CELLMARKEREXP_HEMANGIOBLASTS																														
CELLMARKEREXP_HEMATOPOIETIC_STEM_CELLS																														
CELLMARKEREXP_HEPATIC_STELLATE_CELLS																														
CELLMARKEREXP_HIS_BUNDLE_CELLS																														
CELLMARKEREXP_IMMATURE_NEURONS																														
CELLMARKEREXP_JUXTAGLOMERULAR_CELLS																														
CELLMARKEREXP_KERATINOCYTES																														
CELLMARKEREXP_KIDNEY_PROGENITOR_CELLS																														
CELLMARKEREXP_KUPFFER_CELLS																														
CELLMARKEREXP_LEYDIG_CELLS																														
CELLMARKEREXP_LOOP_OF_HENLE_CELLS																														
CELLMARKEREXP_LUMINAL_EPITHELIAL_CELLS																														
CELLMARKEREXP_MAMMARY_EPITHELIAL_CELLS																														
CELLMARKEREXP_MEGAKARYOCYTES																														
CELLMARKEREXP_MELANOCYTES																														
CELLMARKEREXP_MESANGIAL_CELLS																														
CELLMARKEREXP_MESOTHELIAL_CELLS																														
CELLMARKEREXP_MICROFOLD_CELLS																														
CELLMARKEREXP_MICROGLIA																														
CELLMARKEREXP_MONOCYTES																														
CELLMARKEREXP_MULLER_CELLS																														
CELLMARKEREXP_MYOBLASTS																														
CELLMARKEREXP_MYOCYTES																														
CELLMARKEREXP_MYOFIBROBLASTS																														
CELLMARKEREXP_NEUROBLASTS																														
CELLMARKEREXP_NEUTROPHILS																														
CELLMARKEREXP_NK_CELLS																														
CELLMARKEREXP_NORADRENERGIC_NEURONS																														
CELLMARKEREXP_OLIGODENDROCYTES																														
CELLMARKEREXP_OSTEOBLASTS																														
CELLMARKEREXP_OSTEOCLAST_PRECURSOR_CELLS																														
CELLMARKEREXP_OSTEOCLASTS																														
CELLMARKEREXP_OSTEOCYTES																														
CELLMARKEREXP_PANETH_CELLS																														
CELLMARKEREXP_PARATHYROID_CHIEF_CELLS																														
CELLMARKEREXP_PERICYTES																														
CELLMARKEREXP_PHOTORECEPTOR_CELLS																														
CELLMARKEREXP_PLASMA_CELLS																														
CELLMARKEREXP_PLASMACYTOID_DENDRITIC_CELLS																														
CELLMARKEREXP_PODOCYTES																														
CELLMARKEREXP_PRINCIPAL_CELLS																														
CELLMARKEREXP_PROXIMAL_TUBULE_CELLS																														
CELLMARKEREXP_PULMONARY_ALVEOLAR_TYPE_I_C…																														

CELLMARKERCAN_ACINAR_CELLS																														
CELLMARKEREXP_PULMONARY_ALVEOLAR_TYPE_II_…																														
CELLMARKEREXP_PURKINJE_FIBER_CELLS																														
CELLMARKEREXP_PURKINJE_NEURONS																														
CELLMARKEREXP_PYRAMIDAL_CELLS																														
CELLMARKEREXP_RETICULOCYTES																														
CELLMARKEREXP_RETINAL_GANGLION_CELLS																														
CELLMARKEREXP_SALIVARY_MUCOUS_CELLS																														
CELLMARKEREXP_SATELLITE_CELLS																														
CELLMARKEREXP_SCHWANN_CELLS																														
CELLMARKEREXP_SEROTONERGIC_NEURONS																														
CELLMARKEREXP_SERTOLI_CELLS																														
CELLMARKEREXP_SMOOTH_MUSCLE_CELLS																														
CELLMARKEREXP_SPERMATOZOA																														
CELLMARKEREXP_STROMAL_CELLS																														
CELLMARKEREXP_T_CELLS																														
CELLMARKEREXP_T_CELLS_NAIVE																														
CELLMARKEREXP_T_CYTOTOXIC_CELLS																														
CELLMARKEREXP_T_HELPER_CELLS																														
CELLMARKEREXP_T_MEMORY_CELLS																														
CELLMARKEREXP_TRICHOCYTES																														
CELLMARKEREXP_TRIGEMINAL_NEURONS																														
CELLMARKEREXP_TROPHOBLAST_CELLS																														
CELLMARKEREXP_TROPHOBLAST_PROGENITOR_CELLS																														
CELLMARKEREXP_TUFT_CELLS																														
CELLMARKEREXP_VASCULAR_SMOOTH_MUSCLE_CELLS																														
CELLMARKEREXPENDOTHELIALCELLSAORTA																														
CELLMARKEREXPNEURALSTEMPRECURSORCELLS																														
CELLMARKERMSIGDB_AIZARANI_LIVER_C12_NK_NK…																														
CELLMARKERMSIGDB_AIZARANI_LIVER_C22_RESID…																														
CELLMARKERMSIGDB_AIZARANI_LIVER_C28_NK_NK…																														
CELLMARKERMSIGDB_AIZARANI_LIVER_C29_MVECS…	IL1R1	NR2F1	RSPO3	TMTC1	IGFBP3	KDM6B	AHNAK	MATN2	S100A…	CDH5	GNAS	NASP	EHD2	SERPI…	NTS	FLT1	CSRP1	DDX21	HLA-E	MECOM	AIF1L	GARRE1	HIF1A	CRTAP	GJA5	SPTBN1	NFIB	PLVAP	MYOF	KLF4
CELLMARKERMSIGDB_AIZARANI_LIVER_C30_HEPAT…	FMO3	IKBIP	EXOC3…	LSINC…	HSPA4L	SLCO1…	PIK3C3	RALGA…	ITGA9…	ARHGE…	DHTKD1	MREG	KPNA6	TRMT9B	LUADT1	IGFBP1	ALAS1	QSOX1	NOS1AP	NBPF12	MAP3K…	LINC0…	KCNMB1	MACC1	COBLL1	IL1RAP	MAS1	SRSF11	ANGPT…	ID2
CELLMARKERMSIGDB_AIZARANI_LIVER_C8_RESIDE…																														
CELLMARKERMSIGDB_AIZARANI_LIVER_C9_LSECS_1	IFI44L	ME2	TOX2	NCOA7	PPP1R…	LIMA1	FKBP1A	PHC2	SLC25…	NR5A2	GALNT…	NISCH	ESAM	MAF	JUND	DYNC2…	RBMS1	TPM4	S1PR1	WARS1	SRPX	TFPI2	PCGF2	RASAL2	EXOC3…	MYH10	PLXNA2	MCF2L	ECE1	ARHGA…
CELLMARKERMSIGDB_BUSSLINGER_DUODENAL_BCHE…																														
CELLMARKERMSIGDB_BUSSLINGER_DUODENAL_DIFF…	PDLIM1	HNRNPU	RPL24	COX7A2	ESD	KPNB1	DEK	ANXA2	CHCHD2	PRDX3	RPL27A	ACTG1	PTGES3	CS	ATP5MF	DDX21	SRP9	COX8A	SF3B5	CYC1	SNHG6	MARCKS	HSP90…	NONO	PHB2	SPINK1	CBX3	ATP5F…	SOX4	COX6A1
CELLMARKERMSIGDB_BUSSLINGER_DUODENAL_EC_C…																														
CELLMARKERMSIGDB_BUSSLINGER_DUODENAL_IMMU…	FCER1G	RPS20	GPSM3	BCL2	LINC0…	ZC3HA…	INPP5D	DEF6	MCL1	ARHGA…	RBMS1	CD101	GMFG	RPL19	PLAAT4	SKAP1	CST7	ITM2C	TMC6	SH3KB…	GIMAP6	EMP3	CCL4	CAPG	XCL1	ITGB7	CDV3	CD44	ARHGA…	JUND
CELLMARKERMSIGDB_BUSSLINGER_DUODENAL_STEM…	EEF1G	SH3D19	EIF4B	YBX1	ATP5M…	ESRP1	EIF3L	KLF5	CRYZL…	CCT6A	RPS15A	ERGIC3	SNX5	XRCC5	TRIM28	APEX1	HMGA1	CFTR	HNRNPC	YWHAQ	RPL6	RPS29	MCM7	RAN	U2AF1	TMEM2…	PTGES3	RPL8	SNRPE	EIF3H
CELLMARKERMSIGDB_BUSSLINGER_DUODENAL_TUFT…																														
CELLMARKERMSIGDB_BUSSLINGER_ESOPHAGEAL_EA…																														
CELLMARKERMSIGDB_BUSSLINGER_ESOPHAGEAL_PR…																														
CELLMARKERMSIGDB_BUSSLINGER_GASTRIC_CHIEF…																														
CELLMARKERMSIGDB_BUSSLINGER_GASTRIC_G_CEL…																														
CELLMARKERMSIGDB_BUSSLINGER_GASTRIC_ISTHM…	EDEM3	TMEM54	LPCAT4	GOLGA4	MISP	PLEC	CCNB1	MRPL14	ANXA2	NR2F6	TSPO	ITGA6	SYK	KLF3	NDUFS6	CBR1	PLLP	EI24	GIPC1	NUSAP1	MLPH	SMPD3	MGST2	EPS8L3	PTTG1	DDX21	TESC	CDS1	ARHGD…	PPFIB…
CELLMARKERMSIGDB_BUSSLINGER_GASTRIC_NECK_…																														
CELLMARKERMSIGDB_BUSSLINGER_GASTRIC_OXYNT…																														
CELLMARKERMSIGDB_BUSSLINGER_GASTRIC_PREZY…																														
CELLMARKERMSIGDB_BUSSLINGER_GASTRIC_REG3A…																														
CELLMARKERMSIGDB_CUI_DEVELOPING_HEART_5TH…																														
CELLMARKERMSIGDB_CUI_DEVELOPING_HEART_5TH…																														
CELLMARKERMSIGDB_CUI_DEVELOPING_HEART_C1_…																														
CELLMARKERMSIGDB_CUI_DEVELOPING_HEART_C2_…																														
CELLMARKERMSIGDB_CUI_DEVELOPING_HEART_COM…																														
CELLMARKERMSIGDB_CUI_DEVELOPING_HEART_COR…																														
CELLMARKERMSIGDB_CUI_DEVELOPING_HEART_END…																														
CELLMARKERMSIGDB_CUI_DEVELOPING_HEART_LEF…																														
CELLMARKERMSIGDB_CUI_DEVELOPING_HEART_RIG…																														
CELLMARKERMSIGDB_CUI_DEVELOPING_HEART_SMO…																														
CELLMARKERMSIGDB_DESCARTES_FETAL_ADRENAL_…																														
CELLMARKERMSIGDB_DESCARTES_FETAL_CEREBELL…																														
CELLMARKERMSIGDB_DESCARTES_FETAL_CEREBELL…																														
CELLMARKERMSIGDB_DESCARTES_FETAL_CEREBELL…	RABEP1	GAB2	RPS10…	TLR4	SGK3	ADAM9	TRPM2	SORL1	TBC1D…	IRAK2	SLC11…	CTSZ	CYSLT…	MPEG1	SLC15…	SERPI…	IL1B	LINC0…	SELPLG	EGR3	TMEM1…	TLR6	IGSF6	CARD11	LILRB4	MNDA	NINJ1	GYPC	TEC	C2
CELLMARKERMSIGDB_DESCARTES_FETAL_CEREBELL…																														
CELLMARKERMSIGDB_DESCARTES_FETAL_CEREBELL…																														
CELLMARKERMSIGDB_DESCARTES_FETAL_CEREBELL…																														
CELLMARKERMSIGDB_DESCARTES_FETAL_CEREBELL…	DAAM2	IGFBP4	FKBP1A	EMCN	UACA	COL6A2	LMO2	MIR44…	RHOB	FSTL1	HBA1	KLF2	ANO2	CCDC8…	PLS3	BSG	PDGFB	LAMC1	ATP10A	CD82	TEK	LMCD1	DUSP6	SMG1P2	MYL12A	SMTN	ECE1	PRSS12	SLC12…	NIBAN2
CELLMARKERMSIGDB_DESCARTES_FETAL_CEREBRUM…																														
CELLMARKERMSIGDB_DESCARTES_FETAL_CEREBRUM…																														
CELLMARKERMSIGDB_DESCARTES_FETAL_CEREBRUM…	LINC0…	CEP85	TALDO1	FHL2	SNPH	RFK	ZNF792	MYO1H	HAGHL	SIAE	NEK8	RBM38	KCNA3	ACRBP	TAF6	HMGB1…	DUSP23	ENTPD5	FAM11…	HCP5	ABCC3	CHCHD7	GPR137	MRPL27	MIR54…	LARP6	PTCRA	EFCAB…	DNM3OS	ZBTB22
CELLMARKERMSIGDB_DESCARTES_FETAL_CEREBRUM…	SLC2A5	RGL3	MAST3	ADPGK	MPEG1	GCLC	CH25H	NFAM1	CD163	ADA2	MNDA	RPS6K…	LYVE1	CEP25…	LILRB1	AMPD3	MROCKI	IL18	CCDC8…	TYMP	APOC1	C2	DHRS9	LINC0…	ARHGA…	FCGR1…	TMEM2…	LINC0…	MYOZ2	PARVG
CELLMARKERMSIGDB_DESCARTES_FETAL_CEREBRUM…																														
CELLMARKERMSIGDB_DESCARTES_FETAL_CEREBRUM…																														
CELLMARKERMSIGDB_DESCARTES_FETAL_CEREBRUM…	COBLL1	LURAP…	SLC7A1	MYO10	JCAD	LAMB2	RPS28	WWTR1	RBP1	C4orf3	PREX2	COL4A2	SLC40…	SOX18	ERG	SERPI…	CAV1	HEG1	DOCK6	PTPRB	SLC30…	ATP10A	EMCN	CDH6	NRARP	LEF1	AFAP1…	CRIP2	SMG1P2	NOSTR…
CELLMARKERMSIGDB_DESCARTES_FETAL_EYE_ASTR…																														
CELLMARKERMSIGDB_DESCARTES_FETAL_EYE_GANG…																														
CELLMARKERMSIGDB_DESCARTES_FETAL_EYE_LENS…																														
CELLMARKERMSIGDB_DESCARTES_FETAL_EYE_PHOT…																														
CELLMARKERMSIGDB_DESCARTES_FETAL_EYE_RETI…																														
CELLMARKERMSIGDB_DESCARTES_FETAL_EYE_SMOO…																														
CELLMARKERMSIGDB_DESCARTES_FETAL_EYE_STRO…																														
CELLMARKERMSIGDB_DESCARTES_FETAL_EYE_VASC…																														
CELLMARKERMSIGDB_DESCARTES_FETAL_HEART_EL…																														
CELLMARKERMSIGDB_DESCARTES_FETAL_HEART_EN…																														
CELLMARKERMSIGDB_DESCARTES_FETAL_HEART_LY…																														
CELLMARKERMSIGDB_DESCARTES_FETAL_HEART_SC…																														
CELLMARKERMSIGDB_DESCARTES_FETAL_HEART_SM…																														
CELLMARKERMSIGDB_DESCARTES_FETAL_HEART_ST…																														
CELLMARKERMSIGDB_DESCARTES_FETAL_HEART_VI…																														
CELLMARKERMSIGDB_DESCARTES_FETAL_INTESTIN…																														
CELLMARKERMSIGDB_DESCARTES_FETAL_INTESTIN…																														
CELLMARKERMSIGDB_DESCARTES_FETAL_INTESTIN…																														
CELLMARKERMSIGDB_DESCARTES_FETAL_INTESTIN…																														
CELLMARKERMSIGDB_DESCARTES_FETAL_INTESTIN…																														
CELLMARKERMSIGDB_DESCARTES_FETAL_INTESTIN…																														
CELLMARKERMSIGDB_DESCARTES_FETAL_KIDNEY_M…																														
CELLMARKERMSIGDB_DESCARTES_FETAL_KIDNEY_U…	LCN12	TAC3	IQANK1	PLA2G…	LINC0…	PRSS22	DUOXA1	LINC0…	RAB17	CWH43	ERP27	CCNO	RNF183	GPR15…	LINC0…	MGC32…	PLEKH…	RBBP8…	CYP24…	DIRAS2	PTK6	KRT20	CYP2J2	RDH10…	NIPAL4	BNIPL	GOLT1A	SLC52…	SPRR3	SIGLE…
CELLMARKERMSIGDB_DESCARTES_FETAL_LIVER_ER…																														
CELLMARKERMSIGDB_DESCARTES_FETAL_LIVER_HE…																														
CELLMARKERMSIGDB_DESCARTES_FETAL_LIVER_HE…	CTNND2	HOGA1	SMOC1	CPN1	IGSF1	SDC1	PHYH	SLC6A1	CFB	KLKB1	F10	BAAT	F11	TOX3	AGXT2	CTXND1	SULT1…	SEMA4G	CP	HAMP	GJB1	ACMSD	UGT2B4	CHDH	F13B	F7	ADH4	LINC0…	DDC	WDR72
CELLMARKERMSIGDB_DESCARTES_FETAL_LIVER_ME…	ZBTB47	AOX1	SLPI	FAM11…	PLAAT5	HCFC1…	GSTM3	SLITR…	SNAP91	GNGT1	STON1…	IGSF9	TGM1	LINC0…	C1QTN…	ARFGE…	PTPRU	SMTNL2	PLA2G…	ADTRP	ALOX15	C6orf…	LINC0…	CFAP46	NPHS1	PIWIL…	SPINT1	COL12…	DNAH7	BRINP2
CELLMARKERMSIGDB_DESCARTES_FETAL_LUNG_BRO…																														
CELLMARKERMSIGDB_DESCARTES_FETAL_LUNG_CIL…	FBXL13	RSPH4A	VNN3P	DNAJB…	CFAP57	CCDC81	CFAP3…	CD164…	CFAP1…	KATNIP	CFAP4…	CFAP58	UBXN10	LEKR1	GOLGA…	AGBL2	ABCA13	OMG	DYNLT5	WDR54	IQUB	TTC26	CFAP2…	IL5RA	LDLRA…	B9D1	LINC0…	SRGAP…	NWD1	CAPS2
CELLMARKERMSIGDB_DESCARTES_FETAL_LUNG_LYM…																														

CELLMARKERCAN_ACINAR_CELLS																														
CELLMARKERMSIGDB_DESCARTES_FETAL_LUNG_NEU…																														
CELLMARKERMSIGDB_DESCARTES_FETAL_LUNG_STR…																														
CELLMARKERMSIGDB_DESCARTES_FETAL_LUNG_VAS…																														
CELLMARKERMSIGDB_DESCARTES_FETAL_MUSCLE_L…																														
CELLMARKERMSIGDB_DESCARTES_FETAL_MUSCLE_S…																														
CELLMARKERMSIGDB_DESCARTES_FETAL_MUSCLE_S…																														
CELLMARKERMSIGDB_DESCARTES_FETAL_MUSCLE_V…																														
CELLMARKERMSIGDB_DESCARTES_FETAL_PANCREAS…																														
CELLMARKERMSIGDB_DESCARTES_FETAL_PANCREAS…																														
CELLMARKERMSIGDB_DESCARTES_FETAL_PANCREAS…																														
CELLMARKERMSIGDB_DESCARTES_FETAL_PANCREAS…																														
CELLMARKERMSIGDB_DESCARTES_FETAL_PANCREAS…																														
CELLMARKERMSIGDB_DESCARTES_FETAL_PANCREAS…																														
CELLMARKERMSIGDB_DESCARTES_FETAL_PANCREAS…																														
CELLMARKERMSIGDB_DESCARTES_FETAL_PLACENTA…																														
CELLMARKERMSIGDB_DESCARTES_FETAL_PLACENTA…																														
CELLMARKERMSIGDB_DESCARTES_FETAL_PLACENTA…																														
CELLMARKERMSIGDB_DESCARTES_FETAL_PLACENTA…																														
CELLMARKERMSIGDB_DESCARTES_FETAL_PLACENTA…																														
CELLMARKERMSIGDB_DESCARTES_FETAL_SPLEEN_E…																														
CELLMARKERMSIGDB_DESCARTES_FETAL_SPLEEN_S…																														
CELLMARKERMSIGDB_DESCARTES_FETAL_SPLEEN_V…																														
CELLMARKERMSIGDB_DESCARTES_FETAL_STOMACH_…	AKAP14	EFHC2	KIF9	CCNO-…	CCDC1…	SYNPR…	PRH2	WDR54	DEUP1	OSCP1	LINC0…	TCTN1	ENKUR	AHI1-…	CFAP1…	BCYRN1	TTLL10	B9D1	FBXO36	CCDC33	KIF19	ENSG0…	MORN2	FAM22…	CCDC1…	MYCBP…	ANKRD…	DYDC2	TTC23L	LINC0…
CELLMARKERMSIGDB_DESCARTES_FETAL_STOMACH_…																														
CELLMARKERMSIGDB_DESCARTES_FETAL_STOMACH_…																														
CELLMARKERMSIGDB_DESCARTES_FETAL_STOMACH_…																														
CELLMARKERMSIGDB_DESCARTES_FETAL_STOMACH_…																														
CELLMARKERMSIGDB_DESCARTES_FETAL_STOMACH_…																														
CELLMARKERMSIGDB_DESCARTES_FETAL_STOMACH_…																														
CELLMARKERMSIGDB_DESCARTES_FETAL_STOMACH_…																														
CELLMARKERMSIGDB_DESCARTES_FETAL_STOMACH_…																														
CELLMARKERMSIGDB_DESCARTES_FETAL_STOMACH_…																														
CELLMARKERMSIGDB_DESCARTES_FETAL_STOMACH_…																														
CELLMARKERMSIGDB_DESCARTES_FETAL_STOMACH_…																														
CELLMARKERMSIGDB_DESCARTES_FETAL_THYMUS_T…																														
CELLMARKERMSIGDB_DESCARTES_MAIN_FETAL_ACI…																														
CELLMARKERMSIGDB_DESCARTES_MAIN_FETAL_ADR…																														
CELLMARKERMSIGDB_DESCARTES_MAIN_FETAL_AST…																														
CELLMARKERMSIGDB_DESCARTES_MAIN_FETAL_BIP…																														
CELLMARKERMSIGDB_DESCARTES_MAIN_FETAL_BRO…																														
CELLMARKERMSIGDB_DESCARTES_MAIN_FETAL_CAR…																														
CELLMARKERMSIGDB_DESCARTES_MAIN_FETAL_CIL…	BBS12	MGAT4D	TTC29	MAP9	SPATA4	LRP2BP	C4orf…	ZDHHC…	C5orf…	MTRR	ROPN1L	LINC0…	DNAH5	GOLPH…	TARS1…	TTC23L	RPL21…	SPEF2	CAPSL	CDC20B	CCNO	CCNO-…	LINC0…	LINC0…	WDR41	FAM15…	KIAA0…	FAM81B	FAM17…	TMEM2…
CELLMARKERMSIGDB_DESCARTES_MAIN_FETAL_DUC…																														
CELLMARKERMSIGDB_DESCARTES_MAIN_FETAL_END…																														
CELLMARKERMSIGDB_DESCARTES_MAIN_FETAL_ENS…																														
CELLMARKERMSIGDB_DESCARTES_MAIN_FETAL_EPI…																														
CELLMARKERMSIGDB_DESCARTES_MAIN_FETAL_GAN…																														
CELLMARKERMSIGDB_DESCARTES_MAIN_FETAL_GOB…																														
CELLMARKERMSIGDB_DESCARTES_MAIN_FETAL_HEM…																														
CELLMARKERMSIGDB_DESCARTES_MAIN_FETAL_HOR…																														
CELLMARKERMSIGDB_DESCARTES_MAIN_FETAL_INH…																														
CELLMARKERMSIGDB_DESCARTES_MAIN_FETAL_INH…																														
CELLMARKERMSIGDB_DESCARTES_MAIN_FETAL_INT…																														
CELLMARKERMSIGDB_DESCARTES_MAIN_FETAL_ISL…																														
CELLMARKERMSIGDB_DESCARTES_MAIN_FETAL_LYM…																														
CELLMARKERMSIGDB_DESCARTES_MAIN_FETAL_MES…																														
CELLMARKERMSIGDB_DESCARTES_MAIN_FETAL_MET…																														
CELLMARKERMSIGDB_DESCARTES_MAIN_FETAL_MYE…																														
CELLMARKERMSIGDB_DESCARTES_MAIN_FETAL_NEU…																														
CELLMARKERMSIGDB_DESCARTES_MAIN_FETAL_OLI…																														
CELLMARKERMSIGDB_DESCARTES_MAIN_FETAL_PDE…																														
CELLMARKERMSIGDB_DESCARTES_MAIN_FETAL_PDE…	FAM53B	CYP2E1	SIRT3	KCNQ1…	ASCL3	ALKBH3	CPSF7	RN7SL…	CSKMT	VPS51	AP5B1	FZD4-…	NOX4	SCARN…	MED17	TMEM1…	BUD13	FXYD6…	MPZL3	ZNF202	ST14	B3GAT…	RPS27…	MIR20…	CLEC4C	TAS2R…	GPRC5…	KANSL2	SMUG1	FGD6
CELLMARKERMSIGDB_DESCARTES_MAIN_FETAL_RET…																														
CELLMARKERMSIGDB_DESCARTES_MAIN_FETAL_SAT…																														
CELLMARKERMSIGDB_DESCARTES_MAIN_FETAL_SCH…																														
CELLMARKERMSIGDB_DESCARTES_MAIN_FETAL_SLC…	SBF2-…	FAR1-…	CALCB	TMEM8…	NAT10	APIP	TRAF6	LINC0…	ARFGA…	SELEN…	CCDC86	SDHAF2	TMEM1…	LTO1	FADD	RPS29…	CADM1…	TLCD5	CD27-…	FAM90…	PLEKH…	PYROX…	MUC19	DDN-A…	ATF1	PHB1P…	AMHR2	PAN2	DTX3	RXYLT1
CELLMARKERMSIGDB_DESCARTES_MAIN_FETAL_SMO…																														
CELLMARKERMSIGDB_DESCARTES_MAIN_FETAL_STE…																														
CELLMARKERMSIGDB_DESCARTES_MAIN_FETAL_SYM…																														
CELLMARKERMSIGDB_DESCARTES_MAIN_FETAL_UNI…																														
CELLMARKERMSIGDB_DESCARTES_MAIN_FETAL_URE…																														
CELLMARKERMSIGDB_DESCARTES_MAIN_FETAL_VAS…																														
CELLMARKERMSIGDB_DESCARTES_MAIN_FETAL_VIS…																														
CELLMARKERMSIGDB_DURANTE_ADULT_OLFACTORY_…																														
CELLMARKERMSIGDB_DURANTE_ADULT_OLFACTORY_…																														
CELLMARKERMSIGDB_DURANTE_ADULT_OLFACTORY_…																														
CELLMARKERMSIGDB_DURANTE_ADULT_OLFACTORY_…																														
CELLMARKERMSIGDB_DURANTE_ADULT_OLFACTORY_…																														
CELLMARKERMSIGDB_DURANTE_ADULT_OLFACTORY_…																														
CELLMARKERMSIGDB_DURANTE_ADULT_OLFACTORY_…																														
CELLMARKERMSIGDB_DURANTE_ADULT_OLFACTORY_…																														
CELLMARKERMSIGDB_DURANTE_ADULT_OLFACTORY_…																														
CELLMARKERMSIGDB_DURANTE_ADULT_OLFACTORY_…																														
CELLMARKERMSIGDB_DURANTE_ADULT_OLFACTORY_…																														
CELLMARKERMSIGDB_DURANTE_ADULT_OLFACTORY_…																														
CELLMARKERMSIGDB_DURANTE_ADULT_OLFACTORY_…																														
CELLMARKERMSIGDB_DURANTE_ADULT_OLFACTORY_…																														
CELLMARKERMSIGDB_DURANTE_ADULT_OLFACTORY_…																														
CELLMARKERMSIGDB_DURANTE_ADULT_OLFACTORY_…																														
CELLMARKERMSIGDB_FAN_EMBRYONIC_CTX_ASTROC…																														
CELLMARKERMSIGDB_FAN_EMBRYONIC_CTX_BIG_GR…	KANK2	PROCR	SLC2A3	ERG	XAF1	GIMAP7	PDE8B	SLC20…	PRKCH	SERPI…	AGRN	ITGB1	MECOM	HEG1	RHOJ	MGLL	STARD…	FKBP9	RGS3	CAVIN1	TTYH2	SLC12…	CLIC1	CD320	IFI35	MYH9	CRIP1	NECTI…	GPCPD1	ETS1
CELLMARKERMSIGDB_FAN_EMBRYONIC_CTX_BIG_GR…	TM7SF2	GARIN…	CHGB	HIGD1A	HYAL3	PERP	NACC2	PHLDA3	F12	ABLIM1	CKMT1B	CYB5A	CDH1	ATP6V…	PKP1	DNER	RHEB	COX5A	BAIAP2	QPCT	KRT222	ITM2C	COX7B	ENTPD3	DEGS2	ATP5M…	STK32C	MFSD4A	ATP6V…	CA2
CELLMARKERMSIGDB_FAN_EMBRYONIC_CTX_BIG_GR…																														
CELLMARKERMSIGDB_FAN_EMBRYONIC_CTX_BIG_GR…	TNFRS…	NAGA	RAB3I…	REEP4	ZFP36	AOAH	PPT1	PDPN	CYBA	NAIP	GLIPR1	PLEK	BIN1	ARPC1B	CTSH	DUSP1	SRGN	CTSD	OTUD1	RGS19	HCLS1	ABI3	PDGFB	XBP1	SIRPA	EGR1	LYN	LCP1	ABCG2	MGAT4A
CELLMARKERMSIGDB_FAN_EMBRYONIC_CTX_BRAIN_…	LAPTM…	CAVIN2	RAPGE…	SLC16…	SEMA6A	DSTN	SLC5A6	MT1E	ITGA6	CYB5R3	FKBP1A	ANXA1	SPINK8	CETP	FOXQ1	RPS6K…	PLS3	CDH6	COL6A2	AFAP1…	PPM1H	SLC7A…	NID2	LAMB2	FXYD5	FLT4	NKD2	OCIAD2	TGM2	CDH11
CELLMARKERMSIGDB_FAN_EMBRYONIC_CTX_BRAIN_…	ITGA11	PKIG	CTTN	TIMP3	HEYL	GRM3	RASL12	RERGL	SMTN	SLC9A…	SELEN…	ZYX	ATP5F…	TRPC6	KCNE4	PTH1R	HEY2	RFTN1	CDC42…	ADAM12	HTRA3	RHOC	PDLIM1	TRIB2	ECM1	PPP1R…	ADAM33	MYOF	AMOTL2	CD151
CELLMARKERMSIGDB_FAN_EMBRYONIC_CTX_EX_1_E…																														
CELLMARKERMSIGDB_FAN_EMBRYONIC_CTX_IN_2_I…																														
CELLMARKERMSIGDB_FAN_EMBRYONIC_CTX_IN_3_I…																														
CELLMARKERMSIGDB_FAN_EMBRYONIC_CTX_IN_5_I…																														
CELLMARKERMSIGDB_FAN_EMBRYONIC_CTX_IN_6_I…																														
CELLMARKERMSIGDB_FAN_EMBRYONIC_CTX_MICROG…																														
CELLMARKERMSIGDB_FAN_EMBRYONIC_CTX_OLIG	FUT8	KTN1	ZNF536	FRY	FOLH1	GOLGA7	LDB3	CLIC4	PLEKH…	ATP8A1	COLGA…	FAM12…	JAM3	SEPTI…	MOB3B	VWA1	TMCC3	NPC1	MVB12B	TMEFF2	DIXDC1	TTLL7	AATK	PRRG1	ARHGE…	DIP2B	NXPE3	TTYH2	DOCK10	KLF13
CELLMARKERMSIGDB_FAN_EMBRYONIC_CTX_OPC																														

Table S5: Cell state marker gene list

CELLMARKERCAN_ACINAR_CELLS																														
CELLMARKERMSIGDB_FAN_OVARY_CL1_GPRC5A_TNF…	ANKRD…	GCLM	MYADM	ATP1A1	ST13	NACA	CNBP	YTHDF2	HNRNP…	PRDX1	DEGS1	CALD1	KIF5B	CAV1	BRIX1	EIF3I	IFI16	BZW1	AXL	DNAJC2	MAP4K5	COL1A2	PEA15	CCNI	RPL4	TMEM1…	CCT3	TOP1	RACK1	LRRFI…
CELLMARKERMSIGDB_FAN_OVARY_CL11_MURAL_GRA…	NEXN	FNDC3B	WWC2	PRAME	DAAM1	PPP1R…	MTMR2	MYL12B	SRPX	RBM22	PNN	AKAP9	ETF1	ACTG1	TMEM4…	RPS25	ST3GA…	EML5	CCT4	EZH2	SEPTI…	FHL2	ARL5B	KIF5B	TOR1A…	LIMS1	SLCO3…	MIR22…	TAX1B…
CELLMARKERMSIGDB_FAN_OVARY_CL13_MONOCYTE_…	IFNGR2	ALOX5…	CYRIB	LGALS1	CSF2RA	LY86	CTSD	ARL4C	SQSTM1	ARPC5	UPP1	TAGLN2	RBM47	HMGA1	CYRIA	GPSM3	HSPA1A	CD58	TALDO1	GPR13…	SEPTI…	ATF5	TPM3	MAP3K8	SPHK1	CLIC1	ATP6V…	LACTB	MAFB	OAZ1
CELLMARKERMSIGDB_FAN_OVARY_CL14_MATURE_SM…	CALM2	MYADM	DYNC1…	ATP5PD	MCRIP1	ARPC1B	HMGN3	NDUFB…	RAB13	TMEM1…	CALM1	COX6C	NDN	PIN1	NDUFA…	MT-ND1	ARID5A	DYNLR…	ATP5MK	COX8A	PPP1R…	ATP5ME	ATP5F…	ELOB	YWHAH	FKBP8	ATP5M…	KLF10	RNH1	S100A…
CELLMARKERMSIGDB_FAN_OVARY_CL15_SMALL_ANT…	HSD17…	VDAC2	EXT1	CDH3	EIF1AD	LMBR1L	FXR1	H4C3	SMARC…	SFPQ	TMEM4…	PLA2G…	CD55	OSGIN2	BZW1	CD47	PNRC2	TCEAL9	RBBP6	PCBP1	IDI1	TBC1D…	G3BP2	WSB1	AFF4	SINHC…	RIN2	YTHDC1	MLF2	USPL1
CELLMARKERMSIGDB_FAN_OVARY_CL18_B_LYMPHOC…	PKM	CRIP1	ADRM1	SELEN…	MAP2K3	MKNK2	CST3	RILPL2	ZNF706	POLR2…	PARK7	FKBP4	ANXA11	ELF1	LIMD2	SRSF2	ALG13	MRPL18	ARPC3	NFKB2	HINT1	RRBP1	CORO1C	SEPTI…	H1-2	TAPBP	G3BP2	CBX6	LITAF	MANF
CELLMARKERMSIGDB_FAN_OVARY_CL2_PUTATIVE_E…																														
CELLMARKERMSIGDB_FAN_OVARY_CL5_HEALTHY_SE…	PPIC	TPM2	ALDH9…	TRIP6	SEC63	SERPI…	COL4A2	TTC3	ECE1	CCN2	CALD1	CALU	ATP5F…	CTR9	OSR2	APEX1	GLRX5	MIF	TMOD3	HSDL2	ARID4B	KLF4	U2AF1	ARF5	AHSA1	PPIA	ATG101	PFN2	PPP2CB	SCPEP1
CELLMARKERMSIGDB_FAN_OVARY_CL6_PUTATIVE_E…	IFITM3	IFI6	DEPP1	ECHDC2	CTSD	FKBP10	NUCB1	CEBPD	CPE	RAB34	PEG3	PFN2	PALLD	MAOB	IER3	S100A6	SCP2	AP3S1	NENF	ABCA8	ZFYVE…	CIRBP	FAM16…	BDH2	ATF3	NUCKS1	AKAP12	CCNI	TUBA1A	SMIM7
CELLMARKERMSIGDB_FAN_OVARY_CL8_MATURE_CUM…	HMGB3	UROS	CALM3	LSM4	ABRACL	TIMM8B	MSRB2	ZNF428	COL4A2	TUBA1B	PDGFC	CKS2	MT-ND4	IFI35	MT-CO3	NDUFA…	STMN3	TIMM13	USP48	PSMA4	GATA4	HINT1	HSBP1	NDUFC1	MRPL16	CLNS1A	RNASE…	SPRR2F	CSGAL…	PLEKH…
CELLMARKERMSIGDB_FAN_OVARY_CL9_PUTATIVE_A…	CDKN1A	VGLL4	PPP3CA	PLSCR1	ANXA2	GABAR…	MTHFD2	BMPR2	RAC1	PSMB8	SPTBN1	CFLAR	SOCS3	SLC25…	ATF4	EIF2A…	HLA-D…	DDX21	HIF1A	NECTI…	ENG	TUBB4B	SERBP1	RBMS1	ANKRD…	ACTN1	KRT18	TXNRD1	WTAP	PDLIM5
CELLMARKERMSIGDB_GAO_ESOPHAGUS_25W_C1_CIL…	GCA	GJA4	GLB1L	GLIS3…	GOLGA…	GPC5-…	GYG2P1	HHLA2	PLAAT2	HYDIN	IFT46	IFT57	IFT80	IFT81	IFT88	IKZF4	IL20RA	IL5RA	INPP5B	IQCA1	IQCD	IQCH	IQUB	ITGAL	ITGBL1	KATNB1	KCNB1	KCNE1	KCNMB2	KCNRG
CELLMARKERMSIGDB_GAO_ESOPHAGUS_25W_C2_KRT…																														
CELLMARKERMSIGDB_GAO_ESOPHAGUS_25W_C3_FGF…																														
CELLMARKERMSIGDB_GAO_LARGE_INTESTINE_24W_…																														
CELLMARKERMSIGDB_GAO_LARGE_INTESTINE_24W_…																														
CELLMARKERMSIGDB_GAO_LARGE_INTESTINE_24W_…	LYZ	MAF	MAFB	MANBA	MARCH…	MCOLN1	MERTK	METAP…	METRNL	MFSD1	MMP9	MPEG1	MRC1	MS4A4A	MS4A6A	MS4A7	MTHFD2	NAIP	NAMPT	NCF1	NCF1B	NCF2	NCF4	NCKAP…	NDST2	NFAM1	NFKBIE	NINJ1	NINJ2	NLRP3
CELLMARKERMSIGDB_GAO_LARGE_INTESTINE_24W_…																														
CELLMARKERMSIGDB_GAO_LARGE_INTESTINE_24W_…																														
CELLMARKERMSIGDB_GAO_LARGE_INTESTINE_24W_…																														
CELLMARKERMSIGDB_GAO_LARGE_INTESTINE_ADUL…	PAX6	PCBP4	PCDH9	PCLO	PCSK1	PCSK1N	PDE2A	PDX1	PEG10	PF4V1	PGAM2	PITX3	PLCXD3	PLXND1	PNPLA3	PPFIA2	PRAF2	PRKD1	PRPH	PSCA	PTK7	PTPRN	QPCT	RAB3C	RAI2	RAMP1	RAPGE…	RASA4	REG4	RFX3
CELLMARKERMSIGDB_GAO_LARGE_INTESTINE_ADUL…	LINC0…	ENSG0…	LINC0…	ENSG0…	CD109…	ENSG0…	LINC0…	ENSG0…	ENSG0…	BASP1…	LINC0…	ENSG0…	LINC0…	LOXL1	LRRC20	LRRC73	LST1	LTB	LYRM9	MAMLD1	MAP2	MAPK11	MEIS3…	MEOX2	MEX3B	MEX3D	MLXIPL	MPL	MPP7	MS4A10
CELLMARKERMSIGDB_GAO_LARGE_INTESTINE_ADUL…																														
CELLMARKERMSIGDB_GAO_LARGE_INTESTINE_ADUL…	LGALS1	LILRB1	LIMD2	LINC-…	CYTOR	LINC0…	SYNE3	LINC0…	LINC0…	LINC0…	WAKMA…	LINC0…	LINC0…	IRAG2	LRRC8C	LSP1	LY6E	LY9	LY96	MANEA	MAP4K1	MEF2C	MEI1	MIAT	MICB	MSN	MX2	MYL2	MYO1G	MYO5A
CELLMARKERMSIGDB_GAO_LARGE_INTESTINE_ADUL…	GIMAP6	GIMAP7	GIMAP8	GJA1	GJA4	GKN1	GLI3	GLIS2	GLP2R	GNG11	GPC6	GPX3	GRK5	GUCY1…	GULP1	HECW2	HEG1	HIGD1B	HOXB2	HS6ST1	HTR3B	HTRA1	IFI44L	IFIT5	IGDCC4	IGFBP3	IGFBP5	IGFBP6	IGFBP7	IL1R1
CELLMARKERMSIGDB_GAO_SMALL_INTESTINE_24W_…																														
CELLMARKERMSIGDB_GAO_SMALL_INTESTINE_24W_…																														
CELLMARKERMSIGDB_GAO_SMALL_INTESTINE_24W_…																														
CELLMARKERMSIGDB_GAO_SMALL_INTESTINE_24W_…																														
CELLMARKERMSIGDB_GAO_SMALL_INTESTINE_24W_…																														
CELLMARKERMSIGDB_GAO_STOMACH_24W_C1_PROCR…																														
CELLMARKERMSIGDB_GAO_STOMACH_24W_C2_TFF2P…																														
CELLMARKERMSIGDB_GAO_STOMACH_24W_C4_PARIE…																														
CELLMARKERMSIGDB_GAO_STOMACH_24W_C5_PUTAT…																														
CELLMARKERMSIGDB_GAUTAM_EYE_IRIS_CILIARY_…	CD2AP	PPIL1	CENPB	EPB41…	PSMB8…	GLRX	MED13	TMEM44	SLC35…	ZDHHC…	PDS5A	ARRDC1	LLPH	ARHGE…	RBM42	EWSR1	CCDC8…	IL18BP	SKI	PPID	ZNF595	PDGFC	NSMCE1	XRN2	MORC2	FAM89A	HIP1R	BEND7	WASL	C1orf…
CELLMARKERMSIGDB_GAUTAM_EYE_IRIS_CILIARY_…																														
CELLMARKERMSIGDB_GAUTAM_EYE_IRIS_CILIARY_…																														
CELLMARKERMSIGDB_GAUTAM_EYE_IRIS_CILIARY_…																														
CELLMARKERMSIGDB_GAUTAM_EYE_IRIS_CILIARY_…																														
CELLMARKERMSIGDB_HAY_BONE_MARROW_CD34_POS…																														
CELLMARKERMSIGDB_HAY_BONE_MARROW_CD34_POS…																														
CELLMARKERMSIGDB_HAY_BONE_MARROW_CD34_POS…																														
CELLMARKERMSIGDB_HAY_BONE_MARROW_CD34_POS…																														
CELLMARKERMSIGDB_HAY_BONE_MARROW_CD8_T_CE…																														
CELLMARKERMSIGDB_HAY_BONE_MARROW_EARLY_ER…																														
CELLMARKERMSIGDB_HAY_BONE_MARROW_EOSINOPH…																														
CELLMARKERMSIGDB_HAY_BONE_MARROW_NAIVE_T_…	RGL4	TNFRS…	PDE3B	ATM	EPHX2	ANKRD…	SLFN5	PRMT2	PHF1	CDKN1B	PCSK1N	ANXA2R	DDX24	CPA5	GPSM3	PLEKH…	ARID5B	DPP4	APBA2	GCC2	FAM10…	PRKCA	GIMAP1	ITPKB	TSTD1	SC5D	PABPC1	FAM10…	EIF1	TRADD
CELLMARKERMSIGDB_HAY_BONE_MARROW_NEUTROPH…	SELL	SLC16…	PGLS	KCNE3	SIRPA	NFAM1	TLR2	ZNF467	GNS	ATP5ME	AGFG1	SAMSN1	DPYD	GMFG	RHOU	RXRA	SERPI…	F5	PDXK	CKLF	MTARC1	COX6B1	RASSF2	PLA2G7	IFNGR1	LILRB3	NRG1	CD33	LINC0…	CCNY
CELLMARKERMSIGDB_HAY_BONE_MARROW_NK_CELLS	TRGV9	CLIC1	EOMES	SIGLE…	RAMP1	MMP25…	DDX5	BTN3A2	TOX	SIRT2	KIR3D…	ADGRE5	MLC1	UCP2	GK5	LINC0…	CIRBP	GNGT2	RNF167	RAB29	G6PD	CCNJL	ERBB2	RNF126	RNF166	TBCB	PLEKH…	CCDC12	POLR2G	IGF2R
CELLMARKERMSIGDB_HAY_BONE_MARROW_PRE_DEND…																														
CELLMARKERMSIGDB_HAY_BONE_MARROW_PRO_B	TIMEL…	H3C8	HSPB11	BIK	H2BC15	TIAM1…	HASPIN	EPB41…	SRP14	CTNNB…	GALNT…	PRPSA…	H2AC14	FOXM1	GDI2	CDC25C	PSMC3…	H2BC9	MFGE8	HERC5	SMARC…	CCDC1…	DCK	TMPO-…	MDH1	PAIP2	PHF19	N4BP3	FANCG	C12or…
CELLMARKERMSIGDB_HAY_BONE_MARROW_STROMAL	EPB41…	SEPTI…	MEIS2	CHST3	BCAR1	CXCL2	S100A…	ZFHX4	ADAMT…	PODN	ENPP2	HP	SLPI	PTX3	NCAM2	NECAB1	ABCA6	TMEM47	ITGB5	SCARA3	GPX3	GLT8D2	VASN	PPARG	ABCA9	SMOC1	BICC1	MRGPRF	SOX9	NFIB
CELLMARKERMSIGDB_HE_LIM_SUN_FETAL_LUNG_C0…	REEP1	WFS1	EFNB3	SVIL	SPSB1	ANAPC…	UBALD1	JAG1	RSPO3	CAVIN2	TMEM65	MIEN1	DIXDC1	FILIP…	CPD	ADAMT…	MYL7	AIF1L	PREB	CNTNA…	BOK	LAMA5	SIGIRR	ANO10	DMXL2	DRD2	SIAH2	AFMID	C11or…	ACOT7
CELLMARKERMSIGDB_HE_LIM_SUN_FETAL_LUNG_C0…																														
CELLMARKERMSIGDB_HE_LIM_SUN_FETAL_LUNG_C0…																														
CELLMARKERMSIGDB_HE_LIM_SUN_FETAL_LUNG_C0…	GPRC5C	OLFM2	FENDRR	CACNB3	SLC2A1	YARS1	STRBP	LRRC61	TRAF4	CDK2A…	FKBP11	TNNI3	TMX4	PALS1	DLG2	ADAMT…	H2AC25	RND1	ASPHD1	LGALS2	ZNF579	EPHB2	MID1	ADAM15	ATF7I…	CCDC1…	CDC42…	FRAS1	DISP1	UBE2H
CELLMARKERMSIGDB_HE_LIM_SUN_FETAL_LUNG_C0…																														
CELLMARKERMSIGDB_HE_LIM_SUN_FETAL_LUNG_C0…																														
CELLMARKERMSIGDB_HE_LIM_SUN_FETAL_LUNG_C0…	KLF5	IFI35	EPHB6	NPNT	ARL4A	TST	DDR1	CAV2	BST1	HHIP-…	ADAMT…	SEZ6L2	ARPC1B	CYP4B1	CYP4X1	C1GAL…	CFC1B	SDC4	SEC11C	BNC1	CRLS1	IGFBP6	EPS8L1	CCDC7…	CD55	GABAR…	TCEA3	GPRC5A	PAWR	UAP1
CELLMARKERMSIGDB_HE_LIM_SUN_FETAL_LUNG_C0…	CYTH3	NTF3	POC1A	DLGAP5	STARD4	ARHGA…	DEPDC1	LDLR	FLNB	KIF4A	NCAPG	TMEFF1	CHST9	ZNF80…	HJURP	H1-5	CDCA8	CENPA	PKMYT1	CIB2	RACGA…	EYA2	KIAA1…	ANKS1A	CCDC7…	H2AC16	EVA1A	PRC1	HSD17…	KIF15
CELLMARKERMSIGDB_HE_LIM_SUN_FETAL_LUNG_C0…																														
CELLMARKERMSIGDB_HE_LIM_SUN_FETAL_LUNG_C0…	SLC25…	HHIP-…	CDK2A…	MAMSTR	TMEM1…	CGN	ITLN1	IGFBP6	RABGG…	TMUB1	DHRS3	ARF3	PAWR	WARS1	GOLGA…	LSR	EPS8L2	MT1E	EML3	NPNT	PDXK	MEDAG	COL9A2	FRAS1	LRATD2	OPTN	CCNDB…	PSMB8	SLC34…	TST
CELLMARKERMSIGDB_HE_LIM_SUN_FETAL_LUNG_C0…																														
CELLMARKERMSIGDB_HE_LIM_SUN_FETAL_LUNG_C1…																														
CELLMARKERMSIGDB_HE_LIM_SUN_FETAL_LUNG_C1…																														
CELLMARKERMSIGDB_HE_LIM_SUN_FETAL_LUNG_C1…																														
CELLMARKERMSIGDB_HE_LIM_SUN_FETAL_LUNG_C1…	CDC45	NPM3	SPA17	SH3PX…	ADAMT…	NDC80	TTF2	TIPIN	FANCG	DEPDC1	TMEM37	SLC27…	TEDC2	LOXL1	LIN52	DDB2	GNPNA…	SSX2IP	BRCA1	PCLO	CENPJ	FAT3	ERI1	E2F1	KDM2B	CDC25A	PAIP2B	AURKA	PCYT2	BLM
CELLMARKERMSIGDB_HE_LIM_SUN_FETAL_LUNG_C1…	MAB21…	ADCY2	LIN7B	PAPSS2	ABLIM2	DEPP1	RALGDS	DUSP4	STX2	FBXO44	TMEFF1	CACNB2	PCDH17	TTC39A	ARX	IRS1	KLF12	SCG2	SPR	GALNS	ARHGA…	NBEA	GIPR	TMEM1…	NPIPA1	NACAD	PLPPR2	FFAR4	FOXA3	NDRG4
CELLMARKERMSIGDB_HE_LIM_SUN_FETAL_LUNG_C1…																														
CELLMARKERMSIGDB_HE_LIM_SUN_FETAL_LUNG_C1…																														
CELLMARKERMSIGDB_HE_LIM_SUN_FETAL_LUNG_C1…																														
CELLMARKERMSIGDB_HE_LIM_SUN_FETAL_LUNG_C1…	IL1R1	HPGD	MFSD6	EPAS1	ADAM28	LIPH	DNAJA4	CXCL3	KRT5	SYBU	SEMA3A	ANG	PAG1	IQGAP2	PAX9	C3	PSCA	CRACR…	TF	PAX1	LRG1	TMEM1…	ALPL	GGT6	DAPP1	B3GAL…	NTN1	LGALS9	ALOX15	PER2
CELLMARKERMSIGDB_HE_LIM_SUN_FETAL_LUNG_C1…	ASCL2	TBX1	MTMR7	FGF14	NAAA	IQSEC1	LFNG	TCEAL2	REXO1	TTC39A	NXPH4	TRIM46	PCDH17	MISP3	LRATD1	DCX	FAM13C	PLCL1	RGS11	SLC16…	CBFA2…	HOXB5	LHFPL6	SIX1	CLDN5	NPTX1	TOX3	EPB41…	NEGR1	SRGAP1
CELLMARKERMSIGDB_HE_LIM_SUN_FETAL_LUNG_C1…	MMP16	NAMPT…	FERMT1	ACVR1B	VCAM1	TRMT12	ANO1	TSLP	NGFR	UBAC1	PLAU	KRT23	TMEM2…	PITX1	NPL	RAC2	RFLNB	MOXD1	NFATC1	ACTG2	SLC6A8	LRP4	SULF1	ITGA9	ABTB2	RAB30	EDAR	RELL1	BOC	PAPSS2
CELLMARKERMSIGDB_HE_LIM_SUN_FETAL_LUNG_C1…	F8A1	AGFG2	C17or…	NEIL1	NPNT	SUSD6	CA8	SHLD2	MIR20…	PADI2	FGGY	PTPN18	SRF	ZNF12	SELEN…	ADIPO…	FOXO1	PTRH1	RAB27B	SEC24A	PIK3I…	SRGAP1	PHLDA1	PPFIB…	SLC33…	KRT15	TENT5B	PSME4	TFAP2C	PTPN23
CELLMARKERMSIGDB_HE_LIM_SUN_FETAL_LUNG_C2…	EPAS1	ACOT13	HIVEP3	LRRK1	OGFOD2	HK3	PCBD2	COPS8	TPD52	DDX60L	CD276	PPME1	RAB42	SPECC…	SLFN5	PYROX…	CXCL3	ACVRL1	MFSD5	TMEM1…	ZNF654	SLC35…	TMEM2…	DNASE…	FCGR3B	NFAM1	FAM12…	PGPEP1	KCMF1	IGSF21
CELLMARKERMSIGDB_HE_LIM_SUN_FETAL_LUNG_C2…	AP3M2	LACC1	PDLIM7	P3H2	THBD	SLC38…	KLHL22	CCDC1…	P2RY10	ATP2A3	CASP9	CD226	MAP3K…	ROBO1	CRYBG3	CLCN4	QPCT	MAP4K5	CHN2	CHST2	RAB30	REPS1	QRSL1	OXCT1	POLA2	FARP2	MTERF2	ST3GA…	INF2	PDP1
CELLMARKERMSIGDB_HE_LIM_SUN_FETAL_LUNG_C2…																														
CELLMARKERMSIGDB_HE_LIM_SUN_FETAL_LUNG_C2…																														
CELLMARKERMSIGDB_HE_LIM_SUN_FETAL_LUNG_C2…																														
CELLMARKERMSIGDB_HE_LIM_SUN_FETAL_LUNG_C2…	KLRG1	ACOT13	SLC9B2	MLC1	C16or…	RHPN1	CSTPP1	C11or…	S1PR4	FHL1	GPR146	HSH2D	DVL2	ELP4	NIPSN…	IPO11	RAMP1	AUTS2	DCP1B	MDK	CYYR1	NDN	HLF	CA8	TBCC	HTR1F	TP53I3	VPREB1	SPOCK2	CEP290
CELLMARKERMSIGDB_HE_LIM_SUN_FETAL_LUNG_C2…	SLC10…	RBM38	CTDSPL	SMIM1	ORAI2	BANK1	ZMYND8	ASAP2	SSBP2	BET1	SPX	UBASH…	PTPN12	PDGFA	CASP3	EFHC2	PTK2	GFOD1	MINDY2	MIGA2	MBOAT2	CA2	SERPI…	SERPI…	TSPOA…	CCDC2…	GSAP	GOLGA2	STK17A	FNBP1L
CELLMARKERMSIGDB_HE_LIM_SUN_FETAL_LUNG_C2…																														
CELLMARKERMSIGDB_HE_LIM_SUN_FETAL_LUNG_C2…	INKA2	CISH	CARMI…	E2F1	ZMYND8	RAB37	MIGA2	SLC66…	TNS1	SIAH1	PRKAR…	THBS1	TBC1D…	ADORA…	BLTP3A	NT5C2	H4C12	INKA1	STON2	PHTF2	AGPAT5	PLCXD1	GUCY1…	LAPTM…	INPP5K	KLHL5	FBXO30	ATG9A	SLFN14	LINC0…
CELLMARKERMSIGDB_HE_LIM_SUN_FETAL_LUNG_C2…	VRK1	NOP16	LRRC47	ST3GA…	CENPH	ELAC2	RAD51…	EXOSC9	UPK3A	PARP10	ATP6V…	MATK	DIDO1	ARMC6	TIFA	KRI1	SNCA	TIFAB	NUCB2	GMCL1	MCM2	PTPN7	NTAN1	MX1	TRAF4	ACTL6A	CD320	FMC1	MAD2L1	SLC7A5
CELLMARKERMSIGDB_HE_LIM_SUN_FETAL_LUNG_C2…																														
CELLMARKERMSIGDB_HE_LIM_SUN_FETAL_LUNG_C2…																														
CELLMARKERMSIGDB_HE_LIM_SUN_FETAL_LUNG_C2…																														
CELLMARKERMSIGDB_HE_LIM_SUN_FETAL_LUNG_C3…																														
CELLMARKERMSIGDB_HE_LIM_SUN_FETAL_LUNG_C3…	PKLR	H3C10	NAA50	SPECC1	FAM83D	CCNF	H2BC9	CDCA4	DEPDC…	KIF15	GCLC	KIAA1…	ESCO2	KCNN4	KREME…	PRR5	GINS2	SGO2	MID1I…	PAK1	PPOX	MCM2	RIPOR3	NCAPD3	ATAD5	RNASE…	ART4	DLEU2	SLC43…	OIP5
CELLMARKERMSIGDB_HE_LIM_SUN_FETAL_LUNG_C3…	HDGFL2	USP39	AAAS	CKS1B	BCOR	BCL3	PCBP4	DCBLD1	SGTA	SPATS…	KDM1A	COQ5	FLYWC…	KIF22	MRPL39	FAM22…	C12or…	UBALD2	NDFIP2	HOXA4	GRK5	SMYD3	MAPK11	PHF19	SNHG12	MRPL49	NELFA	ZNF593	PDLIM2	RNF14…
CELLMARKERMSIGDB_HE_LIM_SUN_FETAL_LUNG_C3…	ASPH	SLC7A1	NFATC2	LINC0…	HOXD3	TPD52	ARL15	BCR	ZDHHC…	CCN1	CYP27…	MEDAG	TTN	AKR1C1	BCHE	GLRB	CTBS	CAMK2D	TRADD	DTX1	FILIP…	ATP9A	IGFBP5	EFNA5	FBLN2	TMTC1	BHMT2	NBL1	CCDC1…	GLT8D2
CELLMARKERMSIGDB_HE_LIM_SUN_FETAL_LUNG_C3…	CAMKK2	CHRDL1	DTX1	CTDSPL	GPRC5C	CCDC80	WASF3	GLS	FHL2	NR2F1…	PLEKH…	ZP2	IMPDH1	DOCK8	HOXD8	ACADS	KCNIP1	SLCO2…	MATN2	TMEM3…	VAV3	OAS1	LGALS…	CNTNA…	CHKA	ABLIM3	EDNRB	CEP85L	ADIRF	ARFGA…
CELLMARKERMSIGDB_HE_LIM_SUN_FETAL_LUNG_C3…																														
CELLMARKERMSIGDB_HE_LIM_SUN_FETAL_LUNG_C3…	RPS6K…	IFI44L	CXCL3	DDAH1	MATN2	AGA	CADM1	FOXC1	S1PR4	SH3BP4	ABR	SAMD4A	CAPG	PRSS35	SLIT3	LTBP2	MCC	HIP1R	PTPRR	PLOD2	S100B	PCSK2	PER1	IFNAR2	BCL9L	COL23…	BST1	PTN	HMCN2	PI16
CELLMARKERMSIGDB_HE_LIM_SUN_FETAL_LUNG_C3…	PCLO	SLC9A…	ALDH3…	DSEL	MRAS	LRRC70	LINC0…	PODXL2	FLRT3	AGAP2…	ACYP2	TMTC1	TARS3	ZMYND8	S100A4	LDOC1	MAGED…	PIP5K…	REEP1	PDGFD	EXOSC7	COL4A5	PGAP4	GALNT1	APOE	SLPI	FAM17…	ORAI3	SESTD1	FAM22…
CELLMARKERMSIGDB_HE_LIM_SUN_FETAL_LUNG_C3…																														
CELLMARKERMSIGDB_HE_LIM_SUN_FETAL_LUNG_C4…																														
CELLMARKERMSIGDB_HE_LIM_SUN_FETAL_LUNG_C4…																														
CELLMARKERMSIGDB_HE_LIM_SUN_FETAL_LUNG_C4…																														
CELLMARKERMSIGDB_HE_LIM_SUN_FETAL_LUNG_C4…																														
CELLMARKERMSIGDB_HE_LIM_SUN_FETAL_LUNG_C4…	CLN6	RUVBL2	ATP1B3	GUSB	RAD51C	SCCPDH	NRM	LRRC59	HAUS4	RFC5	PRIM1	CFAP20	TIMEL…	GINS2	SKA1	GLO1	TROAP	MCM4	MYBL2	CTBP2	SYNE2	GNL1	ATAD5	CHEK1	POC1A	LMF2	CTNNA…	BAZ1B	PTPN18	SAR1A
CELLMARKERMSIGDB_HE_LIM_SUN_FETAL_LUNG_C4…	MRPL48	NRM	H2AC17	FCGRT	GGCT	ITGA6	TTK	DIAPH3	SPAG5	PRPF19	MTHFD2	SIT1	ODF2	INCENP	H3C3	CCNF	GNL1	FAF1	ARL6I…	BACH2	H2AC21	THEMIS	CD28	RFC2	PXMP2	RUVBL2	ELP5	KIF4A	C1orf…	HAUS4
CELLMARKERMSIGDB_HE_LIM_SUN_FETAL_LUNG_C4…																														

CELLMARKERCAN_ACINAR_CELLS																														
CELLMARKERMSIGDB_HE_LIM_SUN_FETAL_LUNG_C4…																														
CELLMARKERMSIGDB_HE_LIM_SUN_FETAL_LUNG_C4…																														
CELLMARKERMSIGDB_HE_LIM_SUN_FETAL_LUNG_C4…																														
CELLMARKERMSIGDB_HE_LIM_SUN_FETAL_LUNG_C5…																														
CELLMARKERMSIGDB_HE_LIM_SUN_FETAL_LUNG_C5…	OGT	ISOC2	PASK	STAT1	KBTBD8	ETF1	TGFB1	LASP1	TESPA1	TIMMD…	KCNAB2	TP53I…	C20or…	ARHGA…	CNR2	TCTN3	FAM13…	SIGLE…	PLEKH…	CFAP97	NOP16	HDAC9	MAP3K8	CCL4	RSU1	ETV6	PUM3	FKBP5	NUP210	PCCB
CELLMARKERMSIGDB_HE_LIM_SUN_FETAL_LUNG_C5…																														
CELLMARKERMSIGDB_HE_LIM_SUN_FETAL_LUNG_C5…																														
CELLMARKERMSIGDB_HE_LIM_SUN_FETAL_LUNG_C5…	SGO2	E2F8	PDZD11	CMTM3	TUBGC…	H2BC9	SPC24	DLGAP5	RIF1	CCN2	PRC1	MLLT11	TUBG1	CRTAP	KIF2C	CERS2	MYBL2	CHEK1	CDKN2C	SPAG5	CENPL	CDC6	FOXM1	NENF	H2AC17	ARL6I…	CEP78	N4BP2	ALDH5…	SLC20…
CELLMARKERMSIGDB_HE_LIM_SUN_FETAL_LUNG_C6…																														
CELLMARKERMSIGDB_HE_LIM_SUN_FETAL_LUNG_C7…																														
CELLMARKERMSIGDB_HE_LIM_SUN_FETAL_LUNG_C7…																														
CELLMARKERMSIGDB_HE_LIM_SUN_FETAL_LUNG_C7…																														
CELLMARKERMSIGDB_HE_LIM_SUN_FETAL_LUNG_C7…																														
CELLMARKERMSIGDB_HE_LIM_SUN_FETAL_LUNG_C7…	SYNPO2	SCN3A	CADPS	LTK	FAM89A	SEMA4F	PNMA6A	MAPK8…	NANOS1	MXRA7	TOX2	MAP7D2	ERC2	FBXO31	NCS1	MAST1	PSD	DIPK1A	HMGCL…	LRRC24	DENND…	NYAP2	SORL1	MPP2	BOK	ZFR2	GPRIN1	PPP1R…	HID1	STAMB…
CELLMARKERMSIGDB_HE_LIM_SUN_FETAL_LUNG_C7…																														
CELLMARKERMSIGDB_HU_FETAL_RETINA_AMACRINE																														
CELLMARKERMSIGDB_HU_FETAL_RETINA_BIPOLAR																														
CELLMARKERMSIGDB_HU_FETAL_RETINA_FIBROBLA…	PEAR1	ELK3	RNU4-2	MYH10	PLK2	ENG	RAB13	TPM1	NUDT4	CDC42…	SMTN	UACA	CCNL1	DSE	CLIC1	OLFML1	MYL12A	GOLIM4	TJP1	HES1	STOM	IQGAP1	HAS2	TUBB6	EGFR	P3H4	CPXM1	EVA1B	CEBPD	OAF
CELLMARKERMSIGDB_HU_FETAL_RETINA_HORIZONT…																														
CELLMARKERMSIGDB_HU_FETAL_RETINA_MICROGLIA	IER5	BIN2	CYRIB	SCAMP2	NPL	CD4	GADD4…	NFKBIA	HCST	LIMS1	GM2A	TLN1	RAP1A	LPAR5	RUNX1	CD37	SUSD3	ATP8B4	KLF6	ZFP36…	LPCAT2	RNF213	PPP1R…	SFMBT2	UCP2	LY96	ARHGA…	SLC2A3	MYH9	SP100
CELLMARKERMSIGDB_HU_FETAL_RETINA_MULLER																														
CELLMARKERMSIGDB_HU_FETAL_RETINA_PHOTOREC…																														
CELLMARKERMSIGDB_HU_FETAL_RETINA_RGC	NAV2	NMNAT2	TMSB1…	GDI1	NIPSN…	RALYL	CHRNA3	NKAIN4	PAK5	INSIG1	CPNE4	ADGRG1	SRI	KCTD16	GFRA2	ACLY	NAV1	AKR1C2	ITGB1…	RBFOX2	SLC38…	MEIS3	GNAO1	ATCAY	TOX2	NELL2	UBE2L6	FKBP1B	C4orf…	TCEAL7
CELLMARKERMSIGDB_LAKE_ADULT_KIDNEY_C1_EPI…																														
CELLMARKERMSIGDB_LAKE_ADULT_KIDNEY_C10_TH…	RPL7	RPS11	ATP5F…	ATP6V…	RHOA	NRXN3	RPS12	HSP90…	MYL12A	RPS15	RPS3A	ATP5F…	RPLP2	CD46	RRBP1	LRRFI…	TACST…	PABPC1	HSPA8	LITAF	PSAP	PTPN11	RPL7A	UBXN4	STK24	ITPR2	FAM13…	RPL8	PGK1	PLSCR1
CELLMARKERMSIGDB_LAKE_ADULT_KIDNEY_C12_TH…	RPS23	APLP2	PTPN4	ALDH1…	RPL37A	RPL10	TPI1	S100A6	RPL19	RPL24	SLC16…	CAT	RPS21	PRKX	PDE1A	RPS8	PFKL	SMS	RPS19	PKP4	ATP5PB	SNHG29	PDHA1	MRFAP1	RPS15	STK32B	SORL1	UBA52	PLCL1	CACNA…
CELLMARKERMSIGDB_LAKE_ADULT_KIDNEY_C18_CO…	FAU	IQGAP1	DDX5	RPL4	FTL	PDE1A	RPLP1	GUK1	RHOA	MGST3	RPS11	HLA-C	ATP5F…	GNAS	ANXA2	HADHA	ARF1	PRKAG2	ARPC2	SEC62	PSAP	RPS13	PTGES3	RPS15	TPI1	CXXC5	C12or…	HNRNP…	RPL41	ATP5M…
CELLMARKERMSIGDB_LAKE_ADULT_KIDNEY_C19_CO…	RPS27	PLOD2	SKP1	RPS12	PGGHG	NRDC	CD59	HSP90…	ATP5F…	CLCNKB	TERF2…	RPS25	RPS23	RPL24	RPL35A	RPL37A	CD46	PNPLA8	CDH16	PFDN5	RPS9	NPM1	ITGA6	LAPTM…	RPSA	RPL8	TPI1	CIB1	HLA-C	CLU
CELLMARKERMSIGDB_LAKE_ADULT_KIDNEY_C25_EN…																														
CELLMARKERMSIGDB_LAKE_ADULT_KIDNEY_C28_IN…																														
CELLMARKERMSIGDB_LAKE_ADULT_KIDNEY_C29_UN…																														
CELLMARKERMSIGDB_LAKE_ADULT_KIDNEY_C5_PRO…	SLC22…	MME	CCND3	ROBO2	EOLA2…	STX8	SLC16…	FGGY	CRYL1	UNC13B	MYRIP	MAOB	TNRC6C	ZC3H13	ATXN7…	CCDC1…	NFE2L1	GON4L	MYO6	SLC16…	FKBP5	BAZ2A	LINC0…	MAL2	PFKL	SLC36…	NCKAP5	NRG1	NELL1	SREK1
CELLMARKERMSIGDB_MANNO_MIDBRAIN_NEUROTYPE…																														
CELLMARKERMSIGDB_MANNO_MIDBRAIN_NEUROTYPE…	GRIP1	GRIP2	GRM8	GSE1	HBA1	HBA2	HBG1	HBG2	HERC2…	HIVEP3	NSG2	HOOK1	HPCAL4	HPGD	HS3ST4	ICA1L	IGF1	IGSF9	INA	INHBA…	INSM1	IRF2B…	IRGQ	JPH4	JUP	KCNAB1	KCNIP4	KCNMA1	KCNN3	KCNQ3
CELLMARKERMSIGDB_MANNO_MIDBRAIN_NEUROTYPE…	GABRB3	GABRG2	GAP43	GDAP1	GDPD5	GFRA1	GLRA2	BICRAL	GNAL	GNAO1	GNAQ	GNG2	GNG3	GNPTAB	GOLGA…	GPC2	GPM6A	GPR12	GPR176	GPR26	GPR85	GPRAS…	PIK3R1	GRIA3	GRIK3	GRIN3A	GRM8	GRP	MACRO…	HBA1
CELLMARKERMSIGDB_MANNO_MIDBRAIN_NEUROTYPE…	PIK3R1	GRIA2	GRIA3	GRIK3	GRIP2	GRM5	GRP	HBA1	HBA2	HBB	HBG1	HBG2	HERC2…	HMGCL…	NSG2	JPT1	HOOK1	HPCAL4	HPRT1	IGF1	IKZF4	INA	INSM1	IPCEF1	JAKMI…	JPH3	JPH4	JUP	KCNC2	KCNIP4
CELLMARKERMSIGDB_MANNO_MIDBRAIN_NEUROTYPE…	EEA1	EFCAB…	EFNA1	EFNB2	EGFL7	EGR1	EGR2	EGR3	EHD4	ELF1	ELL2	ELN	ELOVL1	ADGRL4	EMCN	EMP1	EMP2	ENG	ENTPD1	EOGT	EPAS1	EPHA2	EPHB4	EPHX4	ERF	ERG	ERVME…	ESAM	ESM1	ESYT2
CELLMARKERMSIGDB_MANNO_MIDBRAIN_NEUROTYPE…	FPR1	FTL	FYB1	GAB3	GADD4…	GAL3S…	GAS6	GATM	GIMAP4	GIMAP5	GLIPR1	GLRX	GM2A	GMFG	GNB4	GNG7	GNGT2	GPNMB	GPR13…	GPR183	GPR34	GPR65	GPRIN3	GPX1	TAMAL…	GRN	GSN	GYPC	HAUS4	HAVCR2
CELLMARKERMSIGDB_MANNO_MIDBRAIN_NEUROTYPE…	TMEM1…	FAM13…	RETRE…	NALF1	NALF1…	FAM16…	FAM17…	FAM18…	TAFA1	TAFA2	TAFA5	RIPOR2	FAR2	FAR2P2	FBLL1	FBXL16	FGF13	FGF14	FGF9	FHOD3	FLJ16…	FMN2	FNBP1	FNBP1L	FNDC5	FRMD3	FRMD4A	FRMD4B	FRMPD4	FRY
CELLMARKERMSIGDB_MANNO_MIDBRAIN_NEUROTYPE…	PCDH1…	PCDH9	PCSK1	PCSK1N	PDE1A	PDZD7	PER1	PEX5L	PGM2L1	PHACT…	PHF21B	PHLDA1	PHYHI…	PITX2	PKP2	PLCL1	PLCXD3	PLEKH…	POU2F2	POU3F1	POU3F2	PRKACB	PRKAR…	PRRT2	PSD2	PTPN3	PTPN5	RAB3A	RAB3C	RAB9B
CELLMARKERMSIGDB_MANNO_MIDBRAIN_NEUROTYPE…	CRACD	ARFGE…	CEP126	SHISA…	KIF21A	KIF21B	KIF5C	KIRRE…	KLC1	KLF7	KLHL14	KLHL35	KSR2	L1CAM	LARP1B	LHFPL4	LHX1	LHX5-…	LINGO1	LMO1	LMOD3	LMTK3	LINC0…	LONRF2	LPIN1	PLPPR1	PLPPR2	LRCH2	LRRTM2	MAP1B
CELLMARKERMSIGDB_MANNO_MIDBRAIN_NEUROTYPE…	MAP2	MAP3K9	MAP4	MAP6	MAP7D2	MAPK6	MAPRE3	MAST1	MCF2	MCF2L	MDGA2	MEG3	MGAT5	MIR21…	MIR7-3	MLLT11	MMP24	MPP2	MPP3	MSANT…	MT1F	MYO5A	MYT1	N4BP3	NAPB	NEFL	NEFM	NEIL1	NELL1	NELL2
CELLMARKERMSIGDB_MANNO_MIDBRAIN_NEUROTYPE…	PLPPR2	LRP1	LRRC4C	LRRK2	LRRTM3	LRRTM4	LUZP2	MAML2	MAP3K1	MCTP1	MDGA2	METRN	METTL…	IGSF9B	MMP16	MMP2	MT2A	MT3	MYT1	NCAM2	NCAPH	NDRG2	NEIL1	NEK6	NELL1	NETO1	NFIX	NKAIN4	NKX2-2	NLGN1
CELLMARKERMSIGDB_MANNO_MIDBRAIN_NEUROTYPE…	EFHD2	EGR1	EGR2	EGR3	EHBP1…	EHD2	ELL2	ELOVL1	EMCN	EMILI…	EML3	EMP2	ENPEP	ENTPD1	EPAS1	EPB41…	EPB41…	EPHA2	EPHB4	EPHX1	EPS8	EPSTI1	ERF	ETS1	EVA1B	EVA1C	F2R	F2RL2	F3	FABP4
CELLMARKERMSIGDB_MANNO_MIDBRAIN_NEUROTYPE…	PAICS	PAPPA	PARPBP	PBK	PCDH8	PCNA	PDE1A	PDPN	PDZRN3	PHGDH	PIEZO2	PIF1	PLIN3	PLK1	PLP1	PLSCR1	PMAIP1	POU3F1	POU3F2	PRC1	PRR11	PRTG	PTTG1	PTX3	RACGA…	RAD51…	RCOR2	RFC3	RFC4	RFTN2
CELLMARKERMSIGDB_MANNO_MIDBRAIN_NEUROTYPE…	MYBL2	NCAPD2	NCAPG	NCAPH	NEK2	NELL2	NES	NKD1	NPY	NRCAM	NUDT4	NUF2	NUSAP1	OTX2	OTX2-…	PARPBP	PBK	PDGFC	PDZRN3	PHGDH	PIF1	PIP5K…	PLEKH…	PLK1	PLOD2	PMAIP1	POU3F1	PRC1	PROM1	PRR11
CELLMARKERMSIGDB_MANNO_MIDBRAIN_NEUROTYPE…	LOXL2	LRP2	LTBP1	MAD2L1	MAGOHB	MAGT1	MARCO	MAT2B	MBD4	MBNL2	SRF	MCM2	MDK	MELK	MEST	MID1	MIR99…	MIS18…	MKI67	MLLT3	MMP2	MND1	MRPL22	MRPS15	MSI1	MT1X	N4BP3	NAP1L1	NCAPD2	NCAPG
CELLMARKERMSIGDB_MANNO_MIDBRAIN_NEUROTYPE…	NRCAM	NUF2	NUSAP1	NXPH1	OTX2	OTX2-…	P4HA1	PAM	PBK	PBXIP1	PDE11A	PDE3A	PDGFC	PDLIM5	PDZRN3	PHGDH	PIFO	PLCL1	PLEKH…	PLK1	PLOD2	PLTP	PLXDC2	PON2	PPIL6	PPP1R…	PRC1	PROM1	PTGR1	PTPRO
CELLMARKERMSIGDB_MANNO_MIDBRAIN_NEUROTYPE…	MED12L	FAT2	MGST1	MIR99…	MLF1	MNS1	MOK	MORN2	MORN5	PALS1	MPPED2	MSI1	MSX1	NACC2	NCKAP5	NDNF	NEBL	NEK11	NES	NFATC2	NFIA	NFIB	NINJ1	NKAIN4	NKD1	NOTCH1	NOTCH2	NPNT	NPR3	NPTX2
CELLMARKERMSIGDB_MANNO_MIDBRAIN_NEUROTYPE…	FLNC	FLRT3	FNDC3B	FOLH1B	FOS	FOSB	FOXJ1	FRMPD2	FSTL5	FUT9	FZD2	GABBR2	GALC	GAREM2	GATM	GCA	GDPD2	GFAP	GJA1	GLIS3	GLUD1	GNG12	GNG7	GOLM1	GPC1	GPC4	GPM6A	GPM6B	GPR13…	GPR155
CELLMARKERMSIGDB_MANNO_MIDBRAIN_NEUROTYPE…	IRX2	IRX4	ITGA6	ITGB5	ITGB8	ITPKB	ITPR2	JAM2	JUN	ANOS1	KANK1	KCNE5	KCNJ1	KCNN3	KCNQ2	MYORG	CEP126	KIF11	KIF14	KIF15	KIF1A	KIF23	KIF2C	KIRRE…	KLF15	KLF9	KRT14	KRT17	KRT5	LAMA5
CELLMARKERMSIGDB_MANNO_MIDBRAIN_NEUROTYPE…	MT3	MYBL2	MYLK	NAV1	NCAN	NCDN	NDP	NDRG2	NEK11	NEK6	NES	NFATC2	NFIA	NFIX	NKAIN4	NKX2-2	NLGN4Y	NOL7	NOTCH1	NPNT	NRXN1	NSRP1	NUSAP1	OLIG2	OMG	OPCML	OPRM1	OSBPL6	PACRG	PBK
CELLMARKERMSIGDB_MANNO_MIDBRAIN_NEUROTYPE…	FIBIN	FJX1	FLJ16…	FLNC	FLRT3	FMNL2	FNDC3B	FOS	FOXA1	FOXJ1	FOXP2	FREM2	FSTL1	FZD1	FZD3	GALC	GALNT…	GALNT3	GAREM2	GATM	GCA	GDPD2	GJA1	GLB1	GLIS3	GNA12	GNG12	GPC4	GPM6B	GPR155
CELLMARKERMSIGDB_MANNO_MIDBRAIN_NEUROTYPE…	NKAIN2	NLGN4X	NMNAT2	NNAT	NOL4	NOVA1	NREP	NRN1	NRSN1	NRXN1	NSG1	NXPH4	OCIAD2	OPN3	OSBPL6	PAFAH…	PAK3	PAK5	PAPPA	PBX3	PCDHG…	PCDH9	PCDHA7	PCSK2	PDE11A	PDE4D…	PEX5L	PGM2L1	PHACT…	PHF21B
CELLMARKERMSIGDB_MANNO_MIDBRAIN_NEUROTYPE…	KCNIP4	KCNK3	KCNQ3	KCTD2	ARFGE…	CEP126	KIF5C	KIFC2	KIRRE…	KIT	KLC1	KLHL29	KLHL32	KRT17	KSR2	L1CAM	LGI1	LHFPL3	LHFPL4	LINGO1	LMTK3	LMX1B	LONRF2	LPCAT4	LPIN2	PLPPR2	PLPPR3	PLPPR4	PLPPR5	LRFN5
CELLMARKERMSIGDB_MENON_FETAL_KIDNEY_0_CAP…																														
CELLMARKERMSIGDB_MENON_FETAL_KIDNEY_10_IM…																														
CELLMARKERMSIGDB_MENON_FETAL_KIDNEY_2_NEP…																														
CELLMARKERMSIGDB_MENON_FETAL_KIDNEY_3_STR…																														
CELLMARKERMSIGDB_MENON_FETAL_KIDNEY_6_COL…																														
CELLMARKERMSIGDB_MURARO_PANCREAS_ACINAR_C…	SOX4	NFKBIA	B4GAL…	CHAC1	ADAM9	KIAA1…	SLC16…	BCL3	HLA-F	SYTL2	KLF6	SLC22…	TGIF1	IL22R…	OAF	RPL10	CYP1A1	CCND2	SLFN5	TNFAI…	ZNF117	TMEM51	P4HB	TPM1	ZNF114	RBPMS	RPS6K…	ETS2	TM7SF2	MYL12A
CELLMARKERMSIGDB_MURARO_PANCREAS_ALPHA_CE…	PVR	THEM6	TCTN1	NIPSN…	ISL1	SDF2L1	NPTN	FAM21…	MIA2	CCDC1…	SIK3	DNAJA4	STC2	MAN1A1	SH3GL2	MYO10	CITED2	PCBD1	TMX4	RAB27A	ATP9A	TBC1D9	RCAN2	EMB	TOX3	ARHGA…	HMGN5	CERCAM	NUCB2	MAP7D3
CELLMARKERMSIGDB_MURARO_PANCREAS_BETA_CELL	NEBL	KLHL42	MPP1	DPP6	PDZD2	FBXO7	MARK1	ATP8A1	HSPH1	RGL1	KIF21A	MYO1D	AKAP11	TTC3	MYH14	SH3GL2	MAP2	GREM1	CBX7	BEX2	PKP2	RUFY3	ATP6V…	ARHGE…	ST3GA…	SNHG32	TAB2	TSPAN1	MAP1A	TCEA1
CELLMARKERMSIGDB_MURARO_PANCREAS_ENDOTHEL…	DPYSL3	EHD4	MPRIP	CSF2RB	CAVIN1	SASH1	PGM2	KCNN3	SPRED1	PREX1	VWA1	TUBB6	ARHGA…	SLC38…	FMNL3	MMP2	LUZP1	SNRK	TSPAN…	UPP1	SPON2	LAMB1	SWAP70	EFNB3	CHSY1	DKK3	F2R	FKBP1A	MSX1	INSR
CELLMARKERMSIGDB_MURARO_PANCREAS_EPSILON_…																														
CELLMARKERMSIGDB_MURARO_PANCREAS_MESENCHY…	SH2B3	KITLG	PSAT1	CREB3…	SPHK1	PTPRE	NQO1	HEG1	MSX1	GLI2	CYBRD1	LAMC1	TUBB6	WIPI1	CBLB	NT5E	HK1	ANXA1	COL16…	CEBPB	MIR44…	EPS8	IFI16	HOXB2	CYTH3	ARHGA…	DDIT4	SYDE1	RFTN1	MSC-A…
CELLMARKERMSIGDB_RUBENSTEIN_SKELETAL_MUSC…	RPS19	GABAR…	UBA52	ASAH1	PPP1R…	HLA-D…	HIGD2A	EMP3	CAPZA1	TMEM1…	ATP5PB	CMTM6	ARPC2	CDC42…	RPS29	CDC42	PDCL3	NACA	LAMTO…	RPS9	ATP6V…	MACRO…	RPS2	MOB1A	LAMTO…	NAP1L1	RPLP1	PPP1R…	SFT2D1	BLOC1…
CELLMARKERMSIGDB_RUBENSTEIN_SKELETAL_MUSC…	GLUL	TOMM7	SNRPD2	MT1E	COMT	MT-CO1	RHOB	LSP1	UXT	COX4I1	NAA20	COMMD6	BEX3	PPIA	EIF3D	RPS29	AKR1B1	RPS7	MICOS…	CST3	SARAF	SNHG8	EIF3M	MORF4…	RBMX	UQCRH	PTMA	ANXA5	HSPD1	EDF1
CELLMARKERMSIGDB_RUBENSTEIN_SKELETAL_MUSC…	CYC1	HMGN2	IGFBP7	CAP1	JUNB	FTH1	NDUFA5	TCP1	SPARC…	PPDPF	YWHAQ	ATP5MG	ACTN4	UBE2V2	GHITM	CHURC1	STRAP	ATP5PO	SMIM30	ZYX	PCNP	MT-ND1	VCL	RAB13	UQCRQ	PEBP1	RPL5	NDUFA6	NTN4	LMO4
CELLMARKERMSIGDB_TRAVAGLINI_LUNG_ALVEOLAR…	TACST…	PTPRE	NGF	ARL6I…	SNAP23	ANGPT…	WASF3	CLIC2	KHDRB…	EPB41…	GALNT…	FERMT2	KRT19	TNFAI…	PROS1	CCN2	NHSL1	ISG20	EPCAM	PLAC8	SNX22	GKN2	TOR1A…	INF2	TNS1	SPINT1	VSIG2	EFR3B	ATP11A	ARHGE…
CELLMARKERMSIGDB_TRAVAGLINI_LUNG_BASOPHIL…	NFKBID	NDFIP2	ITM2A	ALOX5	RARRE…	FAM10…	GATA1	PNPLA8	RPS4X	NR4A2	FERMT2	MSRA	RBBP8	RPL34	LAT2	NTRK1	PAK1	CD44	TDRD3	TSPYL1	SLC44…	DDAH2	VAPA	TSPAN4	PLAT	KRT19	ANKRD…	HNRNP…	CLIC1	NMT2
CELLMARKERMSIGDB_TRAVAGLINI_LUNG_CAPILLAR…																														
CELLMARKERMSIGDB_TRAVAGLINI_LUNG_CAPILLAR…	PIN4	SH3BP5	APOL3	UBE2L6	RNF11	FAM20…	BAG1	SERPI…	MRPS1…	YBX1	RPS29	SNHG32	POLR3…	GIMAP6	CALM1	KLHL5	OAZ1	PKIG	CARHS…	FTL	CCDC69	PPIA	ARRDC2	C1orf…	MPG	PHACT…	CYB5A	SPOP	TNIP1	DYNLT3
CELLMARKERMSIGDB_TRAVAGLINI_LUNG_CD4_MEMO…																														
CELLMARKERMSIGDB_TRAVAGLINI_LUNG_EREG_DEN…	CCL20	IL10	SERP1	FOSB	PNRC1	SAMSN1	SNCA	SMIM3	SLC2A3	SLC11…	JUND	ANPEP	PTPRE	RNF19B	LAT2	GABAR…	KCTD12	ACTR2	RPS26	SLC16…	IFITM3	HMOX1	C1QC	C3AR1	ACSL3	ELK3	HRH2	LY96	IL1R1	MSR1
CELLMARKERMSIGDB_TRAVAGLINI_LUNG_IGSF21_D…																														
CELLMARKERMSIGDB_TRAVAGLINI_LUNG_MESOTHEL…	MIR44…	MAF	GALNT1	RPL34	MT1E	OLA1	PSMB9	SLC18…	RAMP2	IER5	RPLP0	TMEM1…	ELOVL5	DUSP23	HRH1	SNHG29	PLSCR1	LRP2	PLIN2	UBE2S	TRAF4	RRP1	ZFAS1	MGST1	EPHB6	MEDAG	CITED4	FBXO2	CCDC7…	CHP1
CELLMARKERMSIGDB_TRAVAGLINI_LUNG_MYOFIBRO…																														
CELLMARKERMSIGDB_TRAVAGLINI_LUNG_NATURAL_…																														
CELLMARKERMSIGDB_TRAVAGLINI_LUNG_NEUTROPH…	WASHC1	MGRN1	KMT2E…	PLXNC1	ARHGA…	WASH3P	RALB	ALOX5…	PTGS2	MYO1F	RAB5C	LAMTO…	GIGYF1	JUNB	LPAR2	MRTFA	RGS18	MTND5…	CSNK1D	LAMTO…	TMA7	NPIPB…	TMEM1…	AMPD2	TNFRS…	DOK3	SAMSN1	RASGR…	G6PD	IFRD1
CELLMARKERMSIGDB_TRAVAGLINI_LUNG_OLR1_CLA…	PTPN1	S100A…	CLEC4D	EFHD2	ARFGA…	PTPN12	ACTN1	FURIN	TNFRS…	SLCO4…	NUMB	SLAMF1	TPST1	ATP2B…	IL1RAP	FLNA	RNF14…	GLIPR2	GRB2	SLC16…	PLEK	GNG5	GBP1	BAZ1A	ELK3	RILPL2	APLP2	DGAT2	BCAT1	DDX60L
CELLMARKERMSIGDB_TRAVAGLINI_LUNG_PLATELET…	FAM81B	AHCTF1	CORO1C	MISP3	SLC40…	MEIS1	TMSB4X	F11R	TMBIM1	CNST	UBAC2	APP	SCGB1…	ABLIM3	GTPBP2	TALDO1	PPDPF	PEAR1	TRAPP…	ATP9A	CCNG1	SH3BG…	C1orf…	TRAPP…	INKA2	HMG20B	ITGB1	MIR44…	CMIP	H2AC8
CELLMARKERMSIGDB_TRAVAGLINI_LUNG_PROLIFER…	C19or…	GPX2	ZNF703	GJB3	RAN	ATAD2	EEF1B2	TRIP13	NUP37	MZT2B	MCM6	MTHFD1	GPSM2	PPP1CA	DHFR	IKBIP	HNRNP…	EEF1G	MIR20…	CALD1	PKP1	PIK3R1	CDCA7L	GSTM4	SNAI2	HMGN5	ADH7	RPS5	POSTN	KRT6A
CELLMARKERMSIGDB_TRAVAGLINI_LUNG_PROLIFER…	CPE	ABCG2	UBASH…	SPATS…	SPAG5	CENPE	POGLU…	CCL18	AGRP	ALDH1…	PRR11	PPIC	HYI	PLBD1	E2F1	BARD1	BST1	FCGR1A	ECSCR	ACVRL1	HERC5	TUBG1	KIF20B	FOLR3	H2AX	RBP4	HAUS4	PTGR1	MTFR2	DNAJC9
CELLMARKERMSIGDB_TRAVAGLINI_LUNG_PROXIMAL…	SSBP2	UQCRC1	BCL11A	EIF3H	RPL11	TSPAN8	HLF	PHGDH	EPHX1	CCND1	SPOCK3	RPS23	FERMT1	CLDN10	ALDH7…	ITGB4	COX4I1	BAG1	MTSS1	GLTP	PIK3I…	CKMT1B	DPYSL3	AHCY	PCDH7	HES4	TACST…	EPB41…	RPS15A	LPAR6
CELLMARKERMSIGDB_TRAVAGLINI_LUNG_SEROUS_C…																														
CELLMARKERMSIGDB_TRAVAGLINI_LUNG_SIGNALIN…																														
CELLMARKERMSIGDB_TRAVAGLINI_LUNG_TREM2_DE…	HLA-D…	PCBD1	GMPR	RAB11…	TSPO	OSBPL…	KYNU	YWHAH	PTTG1…	FCGRT	GPX1	RENBP	TFEC	ARHGE…	LY96	PTPMT1	LRP1	CD14	BCAT1	TCN2	ANXA4	RDX	LILRB3	MAN2B1	FCHO2	RNH1	MGAT1	RNASE1	TPP1	SLC31…
CELLMARKERMSIGDB_TRAVAGLINI_LUNG_VASCULAR…																														
CELLMARKERMSIGDB_TRAVAGLINI_LUNG_VEIN_CELL																														
CELLMARKERMSIGDB_VANGURP_PANCREATIC_ALPHA…																														
CELLMARKERMSIGDB_VANGURP_PANCREATIC_DELTA…																														
CELLMARKERMSIGDB_VANGURP_PANCREATIC_GAMMA…																														
CELLMARKERMSIGDB_ZHENG_CORD_BLOOD_C1_PUTA…																														
CELLMARKERMSIGDB_ZHENG_CORD_BLOOD_C10_MUL…																														
CELLMARKERMSIGDB_ZHENG_CORD_BLOOD_C2_PUTA…																														
CELLMARKERMSIGDB_ZHENG_CORD_BLOOD_C4_PUTA…																														
CELLMARKERMSIGDB_ZHENG_CORD_BLOOD_C5_SIMI…																														
CELLMARKERMSIGDB_ZHENG_CORD_BLOOD_C6_HSC_…																														
CELLMARKERMSIGDB_ZHENG_CORD_BLOOD_C7_PUTA…																														
CELLMARKERMSIGDB_ZHENG_CORD_BLOOD_C8_PUTA…																														
CELLMARKERMSIGDB_ZHONG_PFC_C1_ASTROCYTE																														
CELLMARKERMSIGDB_ZHONG_PFC_C1_DLX5_POS_IN…																														
CELLMARKERMSIGDB_ZHONG_PFC_C2_ASCL1_POS_A…																														

Table S5: Cell state marker gene list

CELLMARKERCAN_ACINAR_CELLS																														
CELLMARKERMSIGDB_ZHONG_PFC_C2_UNKNOWN_NPC																														
CELLMARKERMSIGDB_ZHONG_PFC_C3_ASTROCYTE	CCDC1…	RFPL1S	MEGF6	L1CAM	ANKRD…	SS18L1	MAP3K9	BRSK2	SEZ6L2	EDEM3	PDZD4	SLC1A6	CLCN7	RUNX1…	CNOT6	DUSP4	SCN9A	SCN3B	TAF6L	VARS2	ARHGA…	ADCY1	SYNGR3	KCNQ1…	CELSR2	ILRUN	RECQL5	B4GAL…	ZNF721	ELAVL2
CELLMARKERMSIGDB_ZHONG_PFC_C3_HOPX_POS_OPC																														
CELLMARKERMSIGDB_ZHONG_PFC_C3_MICROGLIA	FGF13	TSPOA…	USP32…	LIMS2	USP46	PPP2R…	NLK	ARHGA…	TAB1	BCYRN1	RUNX1…	TPPP	SMARC…	CCDC1…	KIFAP3	ATXN7…	INA	GAD1	ARPP21	UBXN7	PNMA2	TCEA2	C2orf…	ZC2HC…	DCX	ACOT7	TDRP	DNM1	SRRM4	GOLGA…
CELLMARKERMSIGDB_ZHONG_PFC_C3_UNKNOWN_INP																														
CELLMARKERMSIGDB_ZHONG_PFC_C4_UNKNOWN_INP																														
CELLMARKERMSIGDB_ZHONG_PFC_C6_PLXNA4_POS_…																														
CELLMARKERMSIGDB_ZHONG_PFC_C7_ORG_UNDERGO…																														
CELLMARKERMSIGDB_ZHONG_PFC_C8_ORG_PROLIFE…																														
CELLMARKERMSIGDB_ZHONG_PFC_C8_UNKNOWN_NEU…																														
CELLMARKERMSIGDB_ZHONG_PFC_C9_ORG_OTHER																														
CELLMARKERMSIGDB_ZHONG_PFC_HES1_POS_C1_NPC																														
CELLMARKERMSIGDB_ZHONG_PFC_MAJOR_TYPES_AS…	CDC42…	ETV1	MIR9-…	NDRG2	PLCD1	DHCR7	PREX1	CYP51…	IFITM3	ABHD3	IGFBP4	SCD	PNPLA3	HLA-C	OLFM2	PHYHI…	MSMO1	NOTCH2	MEIS1	PBXIP1	ENHO	TRIB2	CA12	HLA-A	FAT1	ALDH6…	PRCP	RAB34	SNX5	GPR13…
CELLMARKERMSIGDB_ZHONG_PFC_MAJOR_TYPES_EX…																														
CELLMARKERMSIGDB_ZHONG_PFC_MAJOR_TYPES_MI…	LHFPL2	BST2	MYO1F	CYTH4	TMIGD2	TLR7	IL6ST	ITM2B	DOCK2	NPC2	PTPN6	ASAH1	LAIR1	C12or…	CTSL	PTGS1	TAL1	NCF4	TNFAI…	RHOG	GM2A	DUSP1	TMC6	LIPA	IL10RA	SP100	SLC15…	TMEM2…	XBP1	UCP2
CELLMARKERMSIGDB_ZHONG_PFC_MAJOR_TYPES_OPC																														

Table S5: Cell state marker gene list

CELLMARKERCAN_ACINAR_CELLS																														
CELLMARKERCAN_ADIPOCYTE_PROGENITOR_CELLS																														
CELLMARKERCAN_ADRENERGIC_NEURONS																														
CELLMARKERCAN_AIRWAY_EPITHELIAL_CELLS																														
CELLMARKERCAN_ALPHA_CELLS																														
CELLMARKERCAN_ALVEOLAR_MACROPHAGES																														
CELLMARKERCAN_ASTROCYTES																														
CELLMARKERCAN_B_CELLS																														
CELLMARKERCAN_B_CELLS_MEMORY																														
CELLMARKERCAN_B_CELLS_NAIVE																														
CELLMARKERCAN_BASOPHILS																														
CELLMARKERCAN_BERGMANN_GLIA																														
CELLMARKERCAN_BETA_CELLS																														
CELLMARKERCAN_CAJAL_RETZIUS_CELLS																														
CELLMARKERCAN_CARDIAC_STEM_AND_PRECURSOR_…																														
CELLMARKERCAN_CARDIOMYOCYTES																														
CELLMARKERCAN_CHOLANGIOCYTES																														
CELLMARKERCAN_CHOLINERGIC_NEURONS																														
CELLMARKERCAN_CHONDROCYTES																														
CELLMARKERCAN_CHOROID_PLEXUS_CELLS																														
CELLMARKERCAN_CILIATED_CELLS																														
CELLMARKERCAN_DELTA_CELLS																														
CELLMARKERCAN_DOPAMINERGIC_NEURONS																														
CELLMARKERCAN_DUCTAL_CELLS																														
CELLMARKERCAN_EMBRYONIC_STEM_CELLS																														
CELLMARKERCAN_ENTERIC_GLIA_CELLS																														
CELLMARKERCAN_ENTERIC_NEURONS																														
CELLMARKERCAN_ENTEROCYTES																														
CELLMARKERCAN_ENTEROENDOCRINE_CELLS																														
CELLMARKERCAN_EPENDYMAL_CELLS																														
CELLMARKERCAN_EPSILON_CELLS																														
CELLMARKERCAN_FOLLICULAR_CELLS																														
CELLMARKERCAN_FOVEOLAR_CELLS																														
CELLMARKERCAN_GAMMA_DELTA_T_CELLS																														
CELLMARKERCAN_GERM_CELLS																														
CELLMARKERCAN_GLUTAMINERGIC_NEURONS																														
CELLMARKERCAN_GRANULOSA_CELLS																														
CELLMARKERCAN_HEMATOPOIETIC_STEM_CELLS																														
CELLMARKERCAN_IMMATURE_NEURONS																														
CELLMARKERCAN_INTERNEURONS																														
CELLMARKERCAN_IONOCYTES																														
CELLMARKERCAN_KERATINOCYTES																														
CELLMARKERCAN_KUPFFER_CELLS																														
CELLMARKERCAN_LEYDIG_CELLS																														
CELLMARKERCAN_LOOP_OF_HENLE_CELLS																														
CELLMARKERCAN_LUMINAL_EPITHELIAL_CELLS																														
CELLMARKERCAN_LUTEAL_CELLS																														
CELLMARKERCAN_MEGAKARYOCYTES																														
CELLMARKERCAN_MELANOCYTES																														
CELLMARKERCAN_MESANGIAL_CELLS																														
CELLMARKERCAN_MESOTHELIAL_CELLS																														
CELLMARKERCAN_MICROFOLD_CELLS																														
CELLMARKERCAN_MICROGLIA																														
CELLMARKERCAN_MONOCYTES																														
CELLMARKERCAN_MOTOR_NEURONS																														
CELLMARKERCAN_MULLER_CELLS																														
CELLMARKERCAN_MYELOID_DERIVED_SUPPRESSOR_…																														
CELLMARKERCAN_MYOBLASTS																														
CELLMARKERCAN_MYOEPITHELIAL_CELLS																														
CELLMARKERCAN_MYOFIBROBLASTS																														
CELLMARKERCAN_NEUROBLASTS																														
CELLMARKERCAN_NEUROENDOCRINE_CELLS																														
CELLMARKERCAN_NEURONS																														
CELLMARKERCAN_NEUTROPHILS																														
CELLMARKERCAN_NK_CELLS																														
CELLMARKERCAN_NORADRENERGIC_NEURONS																														
CELLMARKERCAN_NUOCYTES																														
CELLMARKERCAN_OLIGODENDROCYTES																														
CELLMARKERCAN_OSTEOBLASTS																														
CELLMARKERCAN_OSTEOCLAST_PRECURSOR_CELLS																														
CELLMARKERCAN_OSTEOCLASTS																														
CELLMARKERCAN_OSTEOCYTES																														
CELLMARKERCAN_PANETH_CELLS																														
CELLMARKERCAN_PARATHYROID_CHIEF_CELLS																														
CELLMARKERCAN_PERICYTES																														
CELLMARKERCAN_PERITUBULAR_MYOID_CELLS																														
CELLMARKERCAN_PHOTORECEPTOR_CELLS																														
CELLMARKERCAN_PLASMA_CELLS																														
CELLMARKERCAN_PLASMACYTOID_DENDRITIC_CELLS																														
CELLMARKERCAN_PODOCYTES																														
CELLMARKERCAN_PROXIMAL_TUBULE_CELLS																														
CELLMARKERCAN_PULMONARY_ALVEOLAR_TYPE_I_C…																														
CELLMARKERCAN_PULMONARY_ALVEOLAR_TYPE_II_…																														
CELLMARKERCAN_PURKINJE_NEURONS																														
CELLMARKERCAN_RETICULOCYTES																														
CELLMARKERCAN_RETINAL_GANGLION_CELLS																														
CELLMARKERCAN_RETINAL_PROGENITOR_CELLS																														
CELLMARKERCAN_SATELLITE_CELLS																														
CELLMARKERCAN_SCHWANN_CELLS																														
CELLMARKERCAN_SEROTONERGIC_NEURONS																														
CELLMARKERCAN_SERTOLI_CELLS																														
CELLMARKERCAN_SMOOTH_MUSCLE_CELLS																														
CELLMARKERCAN_STROMAL_CELLS																														
CELLMARKERCAN_T_CELLS																														
CELLMARKERCAN_T_CYTOTOXIC_CELLS																														
CELLMARKERCAN_T_FOLLICULAR_HELPER_CELLS																														
CELLMARKERCAN_T_MEMORY_CELLS																														
CELLMARKERCAN_TRANSIENT_CELLS																														
CELLMARKERCAN_TRICHOCYTES																														
CELLMARKERCAN_TRIGEMINAL_NEURONS																														
CELLMARKERCAN_TROPHOBLAST_CELLS																														
CELLMARKERCAN_TROPHOBLAST_PROGENITOR_CELLS																														
CELLMARKERCAN_TUFT_CELLS																														
CELLMARKERCANENDOTHELIALCELLSAORTA																														
CELLMARKERCUR_N_ABNORMAL_MYELOID_CELL																														

CELLMARKERCAN_ACINAR_CELLS																														
CELLMARKERCUR_N_ABNORMAL_PLASMA_CELL																														
CELLMARKERCUR_N_ACTIVATED_DENDRITIC_CELL																														
CELLMARKERCUR_N_ACTIVATED_EFFECTOR_CELL																														
CELLMARKERCUR_N_ACTIVATED_HEPATIC_STELLAT…																														
CELLMARKERCUR_N_ACTIVATED_LANGERHANS_CELL																														
CELLMARKERCUR_N_ACTIVATED_MACROPHAGE																														
CELLMARKERCUR_N_ACTIVATED_MEMORY_T_CELL																														
CELLMARKERCUR_N_ACTIVATED_NATURAL_KILLER_…																														
CELLMARKERCUR_N_ACTIVATED_REGULATORY_T_CE…																														
CELLMARKERCUR_N_ADIPOCYTE_PROGENITOR_CELL																														
CELLMARKERCUR_N_ADRENAL_MEDULLA_CELL																														
CELLMARKERCUR_N_ADULT_SPERMATOGONIA																														
CELLMARKERCUR_N_ADVENTITIAL_CELL																														
CELLMARKERCUR_N_AEROCYTE																														
CELLMARKERCUR_N_AIRWAY_SMOOTH_MUSCLE_CELL																														
CELLMARKERCUR_N_ALVEOLAR_CELL_TYPE_1																														
CELLMARKERCUR_N_ALVEOLAR_CELL_TYPE_2																														
CELLMARKERCUR_N_ALVEOLAR_EPITHELIAL_CELL_…																														
CELLMARKERCUR_N_ALVEOLAR_EPITHELIAL_CELL_…																														
CELLMARKERCUR_N_ALVEOLAR_PNEUMOCYTE_TYPE_I																														
CELLMARKERCUR_N_ALVEOLAR_PNEUMOCYTE_TYPE_…																														
CELLMARKERCUR_N_AMACRINE_CELL																														
CELLMARKERCUR_N_AMNIOTIC_FLUID_STEM_CELL																														
CELLMARKERCUR_N_ANTERIOR_CELL																														
CELLMARKERCUR_N_ANTERIOR_HINDBRAIN_CELL																														
CELLMARKERCUR_N_ANTI_TUMOR_IMMUNE_CELL																														
CELLMARKERCUR_N_ANTIGEN_PRESENTATION_CANC…																														
CELLMARKERCUR_N_ASTROCYTE																														
CELLMARKERCUR_N_ATRIAL_CELL																														
CELLMARKERCUR_N_ATRIOVENTRICULAR_CANAL_CE…																														
CELLMARKERCUR_N_ATYPICAL_MEMORY_B_CELL																														
CELLMARKERCUR_N_B_2_CELL																														
CELLMARKERCUR_N_B1_CELL																														
CELLMARKERCUR_N_B10_REGULATORY_B_CELL																														
CELLMARKERCUR_N_BASAL_CELL																														
CELLMARKERCUR_N_BASAL_EPITHELIAL_CELL																														
CELLMARKERCUR_N_BASAL_KERATINOCYTE																														
CELLMARKERCUR_N_BASAL_LIKE_CELL																														
CELLMARKERCUR_N_BASAL_PROGENITOR_CELL																														
CELLMARKERCUR_N_BASOPHIL																														
CELLMARKERCUR_N_BILIARY_CELL																														
CELLMARKERCUR_N_BONE_MARROW_STEM_CELL																														
CELLMARKERCUR_N_BONE_SPECIFIC_CELL																														
CELLMARKERCUR_N_BRAIN_CELL																														
CELLMARKERCUR_N_CANCER_CELL	ALK	UGT1A9	SULT2…	PLIN2	MUC16	SMARC…	INSM1	TP63	PTK7	NEK6	MYBL1	TRAF1	TFRC	FCER2	CYB5R2	STAT6	MAML3	LIMD1	MAL	S1PR2	IL4I1	SH3BP5	FAS	TNFRS…	PDCD1…	CREB3…	LMO2	IRF4	JAK2	ASB13
CELLMARKERCUR_N_CANCER_STEM_LIKE_CELL																														
CELLMARKERCUR_N_CAPILLARY_CELL																														
CELLMARKERCUR_N_CAR_T_CELL																														
CELLMARKERCUR_N_CARDIAC_CELL																														
CELLMARKERCUR_N_CARDIOMYOCYTE																														
CELLMARKERCUR_N_CARDIOSPHERE_DERIVED_CELL																														
CELLMARKERCUR_N_CARDIOVASCULAR_PROGENITOR…																														
CELLMARKERCUR_N_CAUDAL_CORTEX_CELL																														
CELLMARKERCUR_N_CD14_MONOCYTE																														
CELLMARKERCUR_N_CD16_MONOCYTE																														
CELLMARKERCUR_N_CD4_CD28_T_CELL																														
CELLMARKERCUR_N_CD4_PERIPHERALLY_DERIVED_…																														
CELLMARKERCUR_N_CD4_T_CELL																														
CELLMARKERCUR_N_CD56BRIGHT_NATURAL_KILLER…																														
CELLMARKERCUR_N_CD56DIM_NATURAL_KILLER_CE…																														
CELLMARKERCUR_N_CD8_T_CELL																														
CELLMARKERCUR_N_CENTRAL_MEMORY_T_CELL																														
CELLMARKERCUR_N_CENTRAL_MEMORY_T_HELPER_1…																														
CELLMARKERCUR_N_CENTRAL_NERVOUS_SYSTEM_ST…																														
CELLMARKERCUR_N_CHANDELIER_CELL																														
CELLMARKERCUR_N_CHIEF_CELL																														
CELLMARKERCUR_N_CHONDROCYTE																														
CELLMARKERCUR_N_CHROMAFFIN_CELL																														
CELLMARKERCUR_N_CIRCULATING_ANGIOGENIC_CE…																														
CELLMARKERCUR_N_CIRCULATING_FETAL_CELL																														
CELLMARKERCUR_N_CIRCULATING_NATURAL_KILLE…																														
CELLMARKERCUR_N_CIRCULATING_PRECURSOR_CELL																														
CELLMARKERCUR_N_CIRCULATING_PROGENITOR_CE…																														
CELLMARKERCUR_N_CIRCULATING_T_FOLLICULAR_…																														
CELLMARKERCUR_N_CIRCULATING_TUMOR_CELL																														
CELLMARKERCUR_N_CLASS_SWITCHED_PLASMABLAST																														
CELLMARKERCUR_N_CLASSICAL_B_CELL																														
CELLMARKERCUR_N_CLASSICAL_CANCER_ASSOCIAT…																														
CELLMARKERCUR_N_CLASSICAL_MONOCYTE																														
CELLMARKERCUR_N_CLAUDIN_LOW_CELL																														
CELLMARKERCUR_N_COLLECTING_DUCTAL_CELL																														
CELLMARKERCUR_N_COLONCYTE																														
CELLMARKERCUR_N_COLORECTAL_STEM_CELL																														
CELLMARKERCUR_N_COLUMNAR_EPITHELIAL_CELL																														
CELLMARKERCUR_N_COMMON_LYMPHOID_PROGENITO…																														
CELLMARKERCUR_N_COMMON_MYELOID_PROGENITOR…																														
CELLMARKERCUR_N_COMPLEMENT_SECRETING_CANC…																														
CELLMARKERCUR_N_CONE_PHOTORECEPTOR_CELL																														
CELLMARKERCUR_N_CONNECTING_TUBULAR_CELL																														
CELLMARKERCUR_N_CONVENTIONAL_CD4_T_CELL																														
CELLMARKERCUR_N_CORNEAL_ENDOTHELIAL_CELL																														
CELLMARKERCUR_N_CORNEAL_EPITHELIAL_CELL																														
CELLMARKERCUR_N_CORNEOCYTE_LIKE_CELL																														
CELLMARKERCUR_N_CORTICAL_CELL																														
CELLMARKERCUR_N_CORTICAL_STROMAL_CELL																														
CELLMARKERCUR_N_CORTICOTROPH_TUMOR_CELL																														
CELLMARKERCUR_N_CRANIAL_NEURAL_CREST_CELL																														
CELLMARKERCUR_N_CRYPT_CELL																														
CELLMARKERCUR_N_CRYPT_STEM_CELL																														
CELLMARKERCUR_N_CYCLING_B_CELL																														
CELLMARKERCUR_N_CYCLING_EPITHELIAL_CELL																														
CELLMARKERCUR_N_CYTOTOXIC_CD8_T_CELL																														
CELLMARKERCUR_N_CYTOTOXIC_DOUBLE_POSITIVE…																														
CELLMARKERCUR_N_CYTOTOXIC_T_CELL																														

CELLMARKERCAN_ACINAR_CELLS																														
CELLMARKERCUR_N_CYTOTOXIC_T_CELL_2																														
CELLMARKERCUR_N_DAMAGE_ASSOCIATED_TRANSIE…																														
CELLMARKERCUR_N_DARK_ZONE_CELL																														
CELLMARKERCUR_N_DEDIFFERENTIATION_ADIPOCY…																														
CELLMARKERCUR_N_DEEP_LAYER_NEURON																														
CELLMARKERCUR_N_DEFINITIVE_ENDODERM_CELL																														
CELLMARKERCUR_N_DELTA_CELL																														
CELLMARKERCUR_N_DEMYELINATING_MICROGLIAL_…																														
CELLMARKERCUR_N_DENDRITIC_CELL_2A																														
CELLMARKERCUR_N_DENDRITIC_CELL_LINEAGE																														
CELLMARKERCUR_N_DENTAL_PULP_CELL																														
CELLMARKERCUR_N_DERMAL_CELL																														
CELLMARKERCUR_N_DEUTEROSOMAL_CELL																														
CELLMARKERCUR_N_DIFFUSE_GASTRIC_CANCER_CE…																														
CELLMARKERCUR_N_DISTAL_CELL																														
CELLMARKERCUR_N_DISTAL_STEM_CELL																														
CELLMARKERCUR_N_DISTAL_TUBULAR_CELL																														
CELLMARKERCUR_N_DIVIDING_CELL																														
CELLMARKERCUR_N_DIVIDING_PLASMA_B_CELL																														
CELLMARKERCUR_N_DIVIDING_T_CELL																														
CELLMARKERCUR_N_DOPAMINERGIC_NEURON																														
CELLMARKERCUR_N_DORSAL_ANTERIOR_HINDBRAIN…																														
CELLMARKERCUR_N_DORSAL_LATERAL_GANGLIONIC…																														
CELLMARKERCUR_N_DORSAL_MEDIAL_GANGLIONIC_…																														
CELLMARKERCUR_N_DOUBLE_NEGATIVE_B_CELL																														
CELLMARKERCUR_N_DOUBLE_NEGATIVE_T_CELL																														
CELLMARKERCUR_N_DOUBLE_POSITIVE_T_CELL																														
CELLMARKERCUR_N_DOUBLE_POSITIVE_TREG_CELL																														
CELLMARKERCUR_N_EARLY_HEMANGIOBLAST																														
CELLMARKERCUR_N_EARLY_PROXIMAL_TUBULE_CELL																														
CELLMARKERCUR_N_EARLY_RESPONSE_SECRETORY_…																														
CELLMARKERCUR_N_EFFECTOR_CHONDROCYTE																														
CELLMARKERCUR_N_EFFECTOR_MEMORY_T_CELL																														
CELLMARKERCUR_N_EFFECTOR_NATURAL_KILLER_C…																														
CELLMARKERCUR_N_ELONGATING_CELL																														
CELLMARKERCUR_N_EMBRYONIC_CELL																														
CELLMARKERCUR_N_EMBRYONIC_STEM_CELL																														
CELLMARKERCUR_N_ENDOCARDIAL_CELL																														
CELLMARKERCUR_N_ENDOCRINE_PROGENITOR_CELL																														
CELLMARKERCUR_N_ENDODERM_PROGENITOR_CELL																														
CELLMARKERCUR_N_ENDODERMAL_CELL																														
CELLMARKERCUR_N_ENDOMETRIAL_STROMAL_CELL																														
CELLMARKERCUR_N_ENDOMETRIAL_STROMAL_STEM_…																														
CELLMARKERCUR_N_ENDOTHELIAL_PRECURSOR_CELL																														
CELLMARKERCUR_N_ENSHEATHING_GLIAL_CELL																														
CELLMARKERCUR_N_ENTERIC_NEURON																														
CELLMARKERCUR_N_EOSINOPHIL																														
CELLMARKERCUR_N_EPENDYMAL_CELL																														
CELLMARKERCUR_N_EPIBLAST_CELL																														
CELLMARKERCUR_N_EPICARDIAL_CELL																														
CELLMARKERCUR_N_EPICARDIUM_DERIVED_CELL																														
CELLMARKERCUR_N_EPIDERMAL_CELL																														
CELLMARKERCUR_N_EPIDERMAL_STEM_CELL																														
CELLMARKERCUR_N_EPITHELIAL_CELL	SERPI…	KRT15	FOLR1	PDCD1	TIGIT	PIGR	GDF15	KRT10	SOX17	WFDC2	MMP7	FOXA1	ESRP2	MACC1	SLC15…	BACE2	CA2	DSC2	TJP2	KRT6A	AMACR	FRMD6	AR	TMPRS…	NKX3-1	KRT1	SCGB2…	DCD	MUCL1	IVL
CELLMARKERCUR_N_EPITHELIAL_DUCTAL_CELL																														
CELLMARKERCUR_N_EPITHELIAL_MESENCHYMAL_CE…																														
CELLMARKERCUR_N_EPITHELIOID_MACROPHAGE																														
CELLMARKERCUR_N_EPSILON_CELL																														
CELLMARKERCUR_N_ERYTHROBLAST																														
CELLMARKERCUR_N_ERYTHROID_CELL																														
CELLMARKERCUR_N_ERYTHROID_PROGENITOR_CELL																														
CELLMARKERCUR_N_ESOPHAGEAL_CELL																														
CELLMARKERCUR_N_ESOPHAGEAL_PROGENITOR_CELL																														
CELLMARKERCUR_N_EXCITATORY_NEURON																														
CELLMARKERCUR_N_EXHAUSTED_DOUBLE_POSITIVE…																														
CELLMARKERCUR_N_EXTRAVILLOUS_TROPHOBLAST																														
CELLMARKERCUR_N_F_PERICYTE																														
CELLMARKERCUR_N_FETAL_MYOBLAST																														
CELLMARKERCUR_N_FIBROBLAST_LIKE_CELL																														
CELLMARKERCUR_N_FIBROCHONDROCYTE																														
CELLMARKERCUR_N_FIBROCHONDROCYTE_PROGENIT…																														
CELLMARKERCUR_N_FOAM_CELL																														
CELLMARKERCUR_N_FOLLICULAR_B_CELL																														
CELLMARKERCUR_N_FOLLICULAR_CELL																														
CELLMARKERCUR_N_FOLLICULAR_DENDRITIC_CELL																														
CELLMARKERCUR_N_FOREBRAIN_CELL																														
CELLMARKERCUR_N_GABAERGIC_NEURON																														
CELLMARKERCUR_N_GANGLION_CELL																														
CELLMARKERCUR_N_GASTRIC_NEUROENDOCRINE_CE…																														
CELLMARKERCUR_N_GASTRIC_STEM_CELL																														
CELLMARKERCUR_N_GERM_CELL																														
CELLMARKERCUR_N_GERMINAL_CENTER_B_CELL																														
CELLMARKERCUR_N_GERMINAL_CENTER_CELL																														
CELLMARKERCUR_N_GERMINAL_CENTRE_B_CELL																														
CELLMARKERCUR_N_GLIAL_CELL																														
CELLMARKERCUR_N_GLIOMA_STEM_CELL																														
CELLMARKERCUR_N_GLOBOSE_BASAL_CELL																														
CELLMARKERCUR_N_GLOMERULAR_PARIETAL_EPITH…																														
CELLMARKERCUR_N_GLUTAMATERGIC_NEURON																														
CELLMARKERCUR_N_GOBLET_CELL																														
CELLMARKERCUR_N_GONADOTROPE																														
CELLMARKERCUR_N_GONADOTROPH_CELL																														
CELLMARKERCUR_N_GRANULOCYTE																														
CELLMARKERCUR_N_GRANULOCYTE_MONOCYTE_PROG…																														
CELLMARKERCUR_N_GRANULOCYTIC_UNK																														
CELLMARKERCUR_N_GRANULOSA_CELL																														
CELLMARKERCUR_N_HAEMATOPOIETIC_CELL																														
CELLMARKERCUR_N_HAIR_FOLLICLE_CELL																														
CELLMARKERCUR_N_HEMANGIOBLAST																														
CELLMARKERCUR_N_HEMATOPOIETIC_CELL																														
CELLMARKERCUR_N_HEMATOPOIETIC_PRECURSOR_C…																														
CELLMARKERCUR_N_HEMATOPOIETIC_PROGENITOR_…																														
CELLMARKERCUR_N_HEPATIC_CELL																														
CELLMARKERCUR_N_HEPATIC_PROGENITOR_CELL																														

Table S5: Cell state marker gene list

CELLMARKERCAN_ACINAR_CELLS																														
CELLMARKERCUR_N_HEPATIC_STELLATE_CELL																														
CELLMARKERCUR_N_HEPATOBLAST																														
CELLMARKERCUR_N_HEPATOCYTE																														
CELLMARKERCUR_N_HISTIOCYTE																														
CELLMARKERCUR_N_HMSC_TREATED_B_CELL																														
CELLMARKERCUR_N_HOFBAUER_CELL																														
CELLMARKERCUR_N_HOMEOSTATIC_CHONDROCYTE																														
CELLMARKERCUR_N_HOMEOSTATIC_MICROGLIAL_CE…																														
CELLMARKERCUR_N_HORIZONTAL_BASAL_CELL																														
CELLMARKERCUR_N_HORIZONTAL_CELL																														
CELLMARKERCUR_N_HYPOBLAST_CELL																														
CELLMARKERCUR_N_IDIOPATHIC_PULMONARY_FIBR…																														
CELLMARKERCUR_N_IGA_MEMORY_B_CELL																														
CELLMARKERCUR_N_IGG_MEMORY_B_CELL																														
CELLMARKERCUR_N_IGM_MEMORY_B_CELL																														
CELLMARKERCUR_N_IGM_ONLY_B_CELL																														
CELLMARKERCUR_N_IL7R_T_HELPER_CELL																														
CELLMARKERCUR_N_IMMATURE_ERYTHROID_CELL																														
CELLMARKERCUR_N_IMMATURE_MYELOID_CELL																														
CELLMARKERCUR_N_IMMATURE_NEUTROPHIL																														
CELLMARKERCUR_N_IMMATURE_TRANSITIONAL_B_C…																														
CELLMARKERCUR_N_IMMUNE_CELL																														
CELLMARKERCUR_N_INDUCED_PLURIPOTENT_STEM_…																														
CELLMARKERCUR_N_INFILTRATING_MACROPHAGE																														
CELLMARKERCUR_N_INFLAMMATORY_CANCER_ASSOC…																														
CELLMARKERCUR_N_INFLAMMATORY_DENDRITIC_CE…																														
CELLMARKERCUR_N_INFLAMMATORY_MONOCYTE																														
CELLMARKERCUR_N_INHIBITORY_NEURON																														
CELLMARKERCUR_N_INNATE_LYMPHOID_CELL																														
CELLMARKERCUR_N_INNER_CELL_MASS_CELL																														
CELLMARKERCUR_N_INNER_RADIAL_GLIAL_CELL																														
CELLMARKERCUR_N_INTER_MEDIATE_PROGENITOR_…																														
CELLMARKERCUR_N_INTERCALATED_CELL																														
CELLMARKERCUR_N_INTERFERON_RESPONSIVE_SEC…																														
CELLMARKERCUR_N_INTERMEDIATE_MONOCYTE																														
CELLMARKERCUR_N_INTERMEDIATE_MONOCYTE_CELL																														
CELLMARKERCUR_N_INTERSTITIAL_MACROPHAGE																														
CELLMARKERCUR_N_INTERSTITIAL_PROGENITOR_C…																														
CELLMARKERCUR_N_INTESTINAL_EPITHELIAL_STE…																														
CELLMARKERCUR_N_INTESTINAL_GASTRIC_CANCER…																														
CELLMARKERCUR_N_INTESTINAL_METAPLASIA_CELL																														
CELLMARKERCUR_N_INTRAEPITHELIAL_LYMPHOCYT…																														
CELLMARKERCUR_N_IONOCYTE																														
CELLMARKERCUR_N_IRIS_PUTTATIVE_STEM_CELL																														
CELLMARKERCUR_N_KUPFFER_CELL																														
CELLMARKERCUR_N_LACTOTROPH_CELL																														
CELLMARKERCUR_N_LANGERHANS_CELL																														
CELLMARKERCUR_N_LEUKOCYTE																														
CELLMARKERCUR_N_LIMBAL_EPITHELIAL_STEM_CE…																														
CELLMARKERCUR_N_LIMBAL_STEM_CELL																														
CELLMARKERCUR_N_LIPOFIBROBLAST																														
CELLMARKERCUR_N_LIVER_PROGENITOR_CELL																														
CELLMARKERCUR_N_LIVER_STEM_CELL																														
CELLMARKERCUR_N_LMMLEYDIG_CELL																														
CELLMARKERCUR_N_LONOCYTE																														
CELLMARKERCUR_N_LOOP_OF_HENLE_CELL																														
CELLMARKERCUR_N_LOOP_PROGENITOR_CELL																														
CELLMARKERCUR_N_LOW_DENSITY_NEUTROPHIL																														
CELLMARKERCUR_N_LUMINAL_PROGENITOR_CELL																														
CELLMARKERCUR_N_LUNG_CELL																														
CELLMARKERCUR_N_LYMPH_NODE_CELL																														
CELLMARKERCUR_N_LYMPHATIC_CELL																														
CELLMARKERCUR_N_LYMPHATIC_ENDOTHELIAL_CELL																														
CELLMARKERCUR_N_LYMPHOBLASTOID_CELL																														
CELLMARKERCUR_N_LYMPHOCYTE																														
CELLMARKERCUR_N_LYMPHOID_CELL																														
CELLMARKERCUR_N_LYMPHOID_PROGENITOR_CELL																														
CELLMARKERCUR_N_LYMPHOID_STRUCTURE_CELL																														
CELLMARKERCUR_N_M_PERICYTE																														
CELLMARKERCUR_N_M1_MACROPHAGE																														
CELLMARKERCUR_N_M1_MICROGLIAL_CELL																														
CELLMARKERCUR_N_M2_MACROPHAGE																														
CELLMARKERCUR_N_MACROGLIAL_CELL																														
CELLMARKERCUR_N_MALIGNANT_CELL																														
CELLMARKERCUR_N_MALIGNANT_PLASMA_CELL																														
CELLMARKERCUR_N_MARGINAL_ASSOCIATED_MACRO…																														
CELLMARKERCUR_N_MAST_CELL																														
CELLMARKERCUR_N_MATURE_ASTROCYTE																														
CELLMARKERCUR_N_MATURE_B_CELL																														
CELLMARKERCUR_N_MATURE_DENDRITIC_CELL																														
CELLMARKERCUR_N_MATURE_ENDOTHELIAL_CELL																														
CELLMARKERCUR_N_MATURE_LUMINAL_CELL																														
CELLMARKERCUR_N_MATURE_NEURON																														
CELLMARKERCUR_N_MATURE_NEURONAL																														
CELLMARKERCUR_N_MATURE_OLIGODENDROCYTE																														
CELLMARKERCUR_N_MATURE_OSTEOBLAST																														
CELLMARKERCUR_N_MATURE_PODOCYTE																														
CELLMARKERCUR_N_MEDIAL_AND_DISTAL_SEGMENT…																														
CELLMARKERCUR_N_MEDIAL_GANGLIONIC_EMINENC…																														
CELLMARKERCUR_N_MEGAKARYOCYTE																														
CELLMARKERCUR_N_MEGAKARYOCYTE_ERYTHROID_C…																														
CELLMARKERCUR_N_MEGAKARYOCYTE_ERYTHROID_P…																														
CELLMARKERCUR_N_MEGAKARYOCYTE_PROGENITOR_…																														
CELLMARKERCUR_N_MELANOMA_CELL																														
CELLMARKERCUR_N_MEMORY_B_CELL																														
CELLMARKERCUR_N_MEMORY_B_CELL_PRECURSOR																														
CELLMARKERCUR_N_MEMORY_CD27_B_CELL																														
CELLMARKERCUR_N_MEMORY_CD4_T_CELL																														
CELLMARKERCUR_N_MEMORY_DOUBLE_POSITIVE_T_…																														
CELLMARKERCUR_N_MEMORY_REGULATORY_T_CELL																														
CELLMARKERCUR_N_MEMORY_T_CELL																														
CELLMARKERCUR_N_MERKEL_CELL																														
CELLMARKERCUR_N_MESENCHYMAL_CELL																														
CELLMARKERCUR_N_MESENCHYMAL_EPITHELIAL_TR…																														

CELLMARKERCAN_ACINAR_CELLS																														
CELLMARKERCUR_N_MESENCHYMAL_GLIOBLASTOMA_…																														
CELLMARKERCUR_N_MESENCHYMAL_STEM_CELL																														
CELLMARKERCUR_N_MESENCHYMAL_STROMAL_CELL																														
CELLMARKERCUR_N_MESENDODERMAL_CELL																														
CELLMARKERCUR_N_MESODERMAL_EMBRYONIC_STEM…																														
CELLMARKERCUR_N_MESODERMAL_PROGENITOR_CELL																														
CELLMARKERCUR_N_METAPLASTIC_STEM_CELL																														
CELLMARKERCUR_N_MEYLOID_CELL																														
CELLMARKERCUR_N_MICROGLIAL_CELL	ZBTB8B	RPS15A	ADAMT…	PTPRZ1	CTSA	FBRSL1	TSHZ3	PSTPI…	KCNJ2	LDHA	KCNMA1	INF2	ZNF843	GLT1D1	RPLP0	RPS3	NDUFA6	TM9SF1	RPS5	CTSL	RPS4X	SERPI…	ZNF532	SLC38…	IGF1	SYNGR1	PTCHD1	CCL4	IGSF10	RPL35A
CELLMARKERCUR_N_MICROVILLAR_CELL																														
CELLMARKERCUR_N_MIDBRAIN_DOPAMINERGIC_NEU…																														
CELLMARKERCUR_N_MONO_MACROPHAGE																														
CELLMARKERCUR_N_MONOCYTE_DERIVED_CELL																														
CELLMARKERCUR_N_MONOCYTE_DERIVED_MYELOID_…																														
CELLMARKERCUR_N_MONOCYTE_LINEAGE																														
CELLMARKERCUR_N_MONOCYTE_PRECURSOR																														
CELLMARKERCUR_N_MONOCYTE_PROGENITOR_CELL																														
CELLMARKERCUR_N_MUELLER_GLIA_CELL																														
CELLMARKERCUR_N_MULTIPOTENT_MESENCHYMAL_S…																														
CELLMARKERCUR_N_MULTIPOTENT_RETINAL_PROGE…																														
CELLMARKERCUR_N_MULTIPOTENT_STEM_CELL																														
CELLMARKERCUR_N_MULTIPOTENT_STROMAL_CELL																														
CELLMARKERCUR_N_MUSCLE_CELL																														
CELLMARKERCUR_N_MUSCLE_DERIVED_CELL																														
CELLMARKERCUR_N_MUSCLE_SATELLITE_CELL																														
CELLMARKERCUR_N_MYELOBLAST																														
CELLMARKERCUR_N_MYELOID_DENDRITIC_CELL																														
CELLMARKERCUR_N_MYELOID_DENDRITIC_CELL_1																														
CELLMARKERCUR_N_MYELOID_DERIVED_CELL																														
CELLMARKERCUR_N_MYELOID_DERIVED_SUPPRESSO…																														
CELLMARKERCUR_N_MYELOID_PROGENITOR_CELL																														
CELLMARKERCUR_N_MYO_EPITHELIAL_CELL																														
CELLMARKERCUR_N_MYOBLAST																														
CELLMARKERCUR_N_MYOCYTE																														
CELLMARKERCUR_N_MYOFIBROBLAST																														
CELLMARKERCUR_N_MYOGENIC_ENDOTHELIAL_CELL																														
CELLMARKERCUR_N_MYOGENIC_PROGENITOR_CELL																														
CELLMARKERCUR_N_MYOID_CELL																														
CELLMARKERCUR_N_NAIVE_B_CELL																														
CELLMARKERCUR_N_NAIVE_CD8_T_CELL																														
CELLMARKERCUR_N_NAIVE_DOUBLE_POSITIVE_T_C…																														
CELLMARKERCUR_N_NAIVE_PROGENITOR_CELL																														
CELLMARKERCUR_N_NAIVE_T_CELL																														
CELLMARKERCUR_N_NASCENT_NEPHRON_CELL																														
CELLMARKERCUR_N_NATURAL_KILLER_CD56_BRIGH…																														
CELLMARKERCUR_N_NATURAL_KILLER_CD56_DIM_C…																														
CELLMARKERCUR_N_NEGATIVE_MESENCHYMAL_STEM…																														
CELLMARKERCUR_N_NEURAL_CELL																														
CELLMARKERCUR_N_NEURAL_CREST_STEM_CELL																														
CELLMARKERCUR_N_NEURAL_CREST_STEMLIKE_CELL																														
CELLMARKERCUR_N_NEURAL_PRECURSOR_CELL																														
CELLMARKERCUR_N_NEURAL_STEM_CELL																														
CELLMARKERCUR_N_NEUROECTODERM_CELL																														
CELLMARKERCUR_N_NEURONAL_INTERMEDIATE_PRO…																														
CELLMARKERCUR_N_NEURONAL_PROGENITOR_CELL																														
CELLMARKERCUR_N_NEUTROPHIL_PROGENITOR_CELL																														
CELLMARKERCUR_N_NON_NEURON																														
CELLMARKERCUR_N_NON_PT_TUBULAR_CELL																														
CELLMARKERCUR_N_NON_RECIRCULATING_TISSUE_…																														
CELLMARKERCUR_N_NONMYELINATING_SCHWANN_CE…																														
CELLMARKERCUR_N_NOTOCHORD_CELL																														
CELLMARKERCUR_N_NUCLEATED_CELL																														
CELLMARKERCUR_N_NUCLEUS_PULPOSUS_PROGENIT…																														
CELLMARKERCUR_N_OLIGODENDROCYTE																														
CELLMARKERCUR_N_OSTEOBLAST																														
CELLMARKERCUR_N_OUTER_RADIAL_GLIA																														
CELLMARKERCUR_N_OVALBUMIN_SPECIFIC_REGULA…																														
CELLMARKERCUR_N_OVARIAN_CELL																														
CELLMARKERCUR_N_OVARIAN_STROMAL_CELL																														
CELLMARKERCUR_N_P53_LIKE_CELL																														
CELLMARKERCUR_N_PAN_B_CELL																														
CELLMARKERCUR_N_PAN_CANCER_ASSOCIATED_FIB…																														
CELLMARKERCUR_N_PAN_MACROPHAGE																														
CELLMARKERCUR_N_PAN_MONOCYTE																														
CELLMARKERCUR_N_PAN_NEURONAL_CELL																														
CELLMARKERCUR_N_PAN_TROPHECTODERM_CELL																														
CELLMARKERCUR_N_PANCREATIC_ENDODERM_CELL																														
CELLMARKERCUR_N_PANCREATIC_POLYPEPTIDE_CE…																														
CELLMARKERCUR_N_PANCREATIC_PROGENITOR_CELL																														
CELLMARKERCUR_N_PANCREATIC_STEM_CELL																														
CELLMARKERCUR_N_PANETH_CELL																														
CELLMARKERCUR_N_PANNEURON																														
CELLMARKERCUR_N_PANVASCULAR_CELL																														
CELLMARKERCUR_N_PAPILLARY_FIBROBLAST																														
CELLMARKERCUR_N_PARIETAL_CELL																														
CELLMARKERCUR_N_PARVALBUMIN_INTERNEURON																														
CELLMARKERCUR_N_PATELET																														
CELLMARKERCUR_N_PATHOGENIC_T_PERIPHERAL_H…																														
CELLMARKERCUR_N_PELVIC_EPITHELIAL_CELL																														
CELLMARKERCUR_N_PERICYTE																														
CELLMARKERCUR_N_PERIODONTAL_LIGAMENT_STEM…																														
CELLMARKERCUR_N_PERIOSTEUM_DERIVED_PROGEN…																														
CELLMARKERCUR_N_PERITUBULAR_MYOID_CELL																														
CELLMARKERCUR_N_PERIVASCULAR_LIKE_CELL																														
CELLMARKERCUR_N_PHAGOCYTE																														
CELLMARKERCUR_N_PHOTORECEPTOR_CELL																														
CELLMARKERCUR_N_PIT_CELL																														
CELLMARKERCUR_N_PIT_MUCOUS_CELL																														
CELLMARKERCUR_N_PITUITARY_STEM_CELL																														
CELLMARKERCUR_N_PLACENTAL_TISSUE_CELL																														
CELLMARKERCUR_N_PLACODE_CELL																														
CELLMARKERCUR_N_PLASMABLAST_B_CELL																														
CELLMARKERCUR_N_PLATELET																														
CELLMARKERCUR_N_PLURIPOTENT_STEM_CELL																														

CELLMARKERCAN_ACINAR_CELLS																														
CELLMARKERCUR_N_PODOCYTE_PRECURSOR																														
CELLMARKERCUR_N_PORTAL_FIBROBLAST																														
CELLMARKERCUR_N_POSTERIOR_CELL																														
CELLMARKERCUR_N_PRE_B_CELL																														
CELLMARKERCUR_N_PRE_CONVENTIONAL_DENDRITI…																														
CELLMARKERCUR_N_PRE_DENDRITIC_CELL																														
CELLMARKERCUR_N_PRECURSOR_CELL																														
CELLMARKERCUR_N_PREHYPERTROPHIC_CHONDROCY…																														
CELLMARKERCUR_N_PRESOMITE_CELL																														
CELLMARKERCUR_N_PRETUBULAR_AGGREGATE_CELL																														
CELLMARKERCUR_N_PRIMITIVE_ENDODERM_CELL																														
CELLMARKERCUR_N_PRINCIPAL_CELL																														
CELLMARKERCUR_N_PRO_B_CELL																														
CELLMARKERCUR_N_PRO_INFLAMMATORY_MACROPHA…																														
CELLMARKERCUR_N_PRO_NEUTROPHIL																														
CELLMARKERCUR_N_PROLIFERATING_CELL																														
CELLMARKERCUR_N_PROLIFERATIVE_CHONDROCYTE																														
CELLMARKERCUR_N_PROLIFERATIVE_DUCTAL_CELL																														
CELLMARKERCUR_N_PROLIFERATIVE_NEURAL_PROG…																														
CELLMARKERCUR_N_PRONEURAL_GLIOBLASTOMA_CE…																														
CELLMARKERCUR_N_PROSPERMATOGONIA																														
CELLMARKERCUR_N_PROSTATE_CELL																														
CELLMARKERCUR_N_PROXIMAL_CELL																														
CELLMARKERCUR_N_PROXIMAL_SEGMENT_CELL																														
CELLMARKERCUR_N_PROXIMAL_STEM_CELL																														
CELLMARKERCUR_N_PROXIMAL_TUBULE_CELL																														
CELLMARKERCUR_N_PULMONARY_NEUROENDOCRINE_…																														
CELLMARKERCUR_N_PYRAMIDAL_CELL																														
CELLMARKERCUR_N_QUIESCENT_T_STEM_CELL																														
CELLMARKERCUR_N_REGULATORY_B_CELL																														
CELLMARKERCUR_N_REGULATORY_INNATE_LYMPHOI…																														
CELLMARKERCUR_N_RENAL_TUBULAR_CELL																														
CELLMARKERCUR_N_RESIDENT_MEMORY_T_CELL																														
CELLMARKERCUR_N_RESPIRATORY_HORIZONTAL_BA…																														
CELLMARKERCUR_N_RESPIRATORY_SECRETORY_CELL																														
CELLMARKERCUR_N_RESPONDER_T_CELL																														
CELLMARKERCUR_N_RESPONDING_CONVENTIONAL_T…																														
CELLMARKERCUR_N_RESTING_MEMORY_B_CELL																														
CELLMARKERCUR_N_RESTING_NAIVE_B_CELL																														
CELLMARKERCUR_N_RESTING_REGULATORY_T_CELL																														
CELLMARKERCUR_N_RETICULAR_FIBROBLAST																														
CELLMARKERCUR_N_RETINA_CELL																														
CELLMARKERCUR_N_ROD_CELL																														
CELLMARKERCUR_N_SATELLITE_CELL																														
CELLMARKERCUR_N_SEPTUM_TRANSVERSUM_MESENC…																														
CELLMARKERCUR_N_SEROUS_CELL																														
CELLMARKERCUR_N_SIDE_POPULATION_CELL																														
CELLMARKERCUR_N_SKELETAL_MUSCLE_CELL																														
CELLMARKERCUR_N_SKELETAL_MUSCLE_STEM_CELL																														
CELLMARKERCUR_N_SMOOTH_MUSCLE_CELL																														
CELLMARKERCUR_N_SMOOTH_MUSCLE_LIKE_CELL																														
CELLMARKERCUR_N_SOMATIC_CELL																														
CELLMARKERCUR_N_SOMATIC_STEM_CELL																														
CELLMARKERCUR_N_SOMATOSTATIN_INTERNEURON																														
CELLMARKERCUR_N_SPECIALIST_ANTIGEN_PRESEN…																														
CELLMARKERCUR_N_SPERMATOGENIC_CELL																														
CELLMARKERCUR_N_SPERMATOGONIAL_CELL																														
CELLMARKERCUR_N_SPERMATOGONIAL_STEM_CELL																														
CELLMARKERCUR_N_SQUAMOUS_DIFFERENTIATION_…																														
CELLMARKERCUR_N_STELLATE_CELL																														
CELLMARKERCUR_N_STEM_CELL_DERIVED_ALPHA_C…																														
CELLMARKERCUR_N_STEM_CELL_DERIVED_BETA_CE…																														
CELLMARKERCUR_N_STEM_CELL_DERIVED_ENDOTHE…																														
CELLMARKERCUR_N_STEM_LIKE_CD8_T_CELL																														
CELLMARKERCUR_N_STROMAL_CELL																														
CELLMARKERCUR_N_SUBPALLIUM_CELL																														
CELLMARKERCUR_N_SUBPLATE_CELL																														
CELLMARKERCUR_N_SUPPRESSIVE_MONOCYTE																														
CELLMARKERCUR_N_SUPPRESSOR_T_CELL																														
CELLMARKERCUR_N_SUPRABASAL_CELL																														
CELLMARKERCUR_N_SURFACE_ECTODERM_CELL																														
CELLMARKERCUR_N_SUSTENTACULAR_CELL																														
CELLMARKERCUR_N_SYMPATHOADRENAL_CELL																														
CELLMARKERCUR_N_SYNCYTIOTROPHOBLAST																														
CELLMARKERCUR_N_SYNTHETIC_SMOOTH_MUSCLE_C…																														
CELLMARKERCUR_N_T_CELL_LARGE_GRANULAR_LYM…																														
CELLMARKERCUR_N_T_CELL_LINEAGE																														
CELLMARKERCUR_N_T_HELPER_CELL																														
CELLMARKERCUR_N_T_MEMORY_STEM_CELL																														
CELLMARKERCUR_N_T2_MZP_REGULATORY_B_CELL																														
CELLMARKERCUR_N_TENDON_CELL																														
CELLMARKERCUR_N_TERMINAL_DIFFERENTIATED_B…																														
CELLMARKERCUR_N_THALAMIC_CELL																														
CELLMARKERCUR_N_THECA_INTERNA_CELL																														
CELLMARKERCUR_N_THYMIC_EPITHELIAL_CELL																														
CELLMARKERCUR_N_TISSUE_LIKE_MEMORY_B_CELL																														
CELLMARKERCUR_N_TISSUE_RESIDENT_CELL																														
CELLMARKERCUR_N_TISSUE_RESIDENT_EFFECTOR_…																														
CELLMARKERCUR_N_TISSUE_RESIDENT_MACROPHAGE																														
CELLMARKERCUR_N_TISSUE_RESIDENT_MEMORY_LI…																														
CELLMARKERCUR_N_TOLDC_INDUCED_REGULATORY_…																														
CELLMARKERCUR_N_TONSIL_CELL																														
CELLMARKERCUR_N_TRABECULAR_MESHWORK_CELL																														
CELLMARKERCUR_N_TRANSITIONAL_B_CELL																														
CELLMARKERCUR_N_TRANSITIONAL_DENDRITIC_CE…																														
CELLMARKERCUR_N_TRUNCATED_RADIAL_GLIAL_CE…																														
CELLMARKERCUR_N_TUBULAR_CELL																														
CELLMARKERCUR_N_TUMOR_ASSOCIATED_MICROGLI…																														
CELLMARKERCUR_N_TUMOR_ENDOTHELIAL_CELL																														
CELLMARKERCUR_N_TUMOR_EPITHELIAL_CELL																														
CELLMARKERCUR_N_TUMOR_INFILTRATING_REGULA…																														
CELLMARKERCUR_N_TUMOR_INFILTRATING_T_CELL																														
CELLMARKERCUR_N_TUMOR_INITIATING_CELL																														
CELLMARKERCUR_N_TUMOR_REGULATORY_T_CELL																														

Table S5: Cell state marker gene list

CELLMARKERCAN_ACINAR_CELLS																														
CELLMARKERCUR_N_UMBRELLA_CELL																														
CELLMARKERCUR_N_UNRESTRICTED_SOMATIC_STEM…																														
CELLMARKERCUR_N_UNSWITCHED_PLASMABLAST																														
CELLMARKERCUR_N_URETERIC_BUD_CELL																														
CELLMARKERCUR_N_UROTHELIAL_CELL																														
CELLMARKERCUR_N_UROTHELIUM_CELL																														
CELLMARKERCUR_N_VASCULATURE_CELL																														
CELLMARKERCUR_N_VEIN_CELL																														
CELLMARKERCUR_N_VENOUS_CELL																														
CELLMARKERCUR_N_VENTRAL_LATERAL_GANGLIONI…																														
CELLMARKERCUR_N_VENTRAL_MEDIAL_GANGLIONIC…																														
CELLMARKERCUR_N_VENTRICULAR_CELL																														
CELLMARKERCUR_N_YOUNG_NEURON																														
CELLMARKERCUR_N_ZONA_FASCICULATA_CELL																														
CELLMARKERCUR_N_ZONA_GLOMERULOSA_CELL																														
CELLMARKERCUR_N_ZONA_RETICULARIS_CELL																														
CELLMARKERCUR_N_ZYGOTE																														
CELLMARKERCURNACTIVATEDEFFECTORMEMORYCD4T…																														
CELLMARKERCURNACTIVATEDTISSUERESIDENTMEMO…																														
CELLMARKERCURNACTIVATEDTISSUERESIDENTMEMO…																														
CELLMARKERCURNALPHACELLACELL																														
CELLMARKERCURNALVEOLARTYPEIIATIICELL																														
CELLMARKERCURNATYPICALBCELLABC																														
CELLMARKERCURNAXLCD11CPLASMACYTOIDDENDRIT…																														
CELLMARKERCURNAXLCD123PLASMACYTOIDDENDRIT…																														
CELLMARKERCURNBASALFOREBRAINCHOLINERGICNE…																														
CELLMARKERCURNBRUSHCELLTUFTCELL																														
CELLMARKERCURNCANCERASSOCIATEDFIBROBLASTC…																														
CELLMARKERCURNCAPSULARFIBROBLASTFB2																														
CELLMARKERCURNCCR7DENDRITICCELL																														
CELLMARKERCURNCD106MESENCHYMALSTEMCELL																														
CELLMARKERCURNCD14MACROPHAGE																														
CELLMARKERCURNCD16DENDRITICCELL																														
CELLMARKERCURNCD25LAG3TCELL																														
CELLMARKERCURNCD34GRANULOCYTICPROGENITORC…																														
CELLMARKERCURNCD34HEMATOPOIETICSTEMCELL																														
CELLMARKERCURNCD34PREBCELL																														
CELLMARKERCURNCD34PROGENITORCELL																														
CELLMARKERCURNCD34THYMOCYTE																														
CELLMARKERCURNCD40LGTHELPERCELL																														
CELLMARKERCURNCD4CD25REGULATORYTCELL																														
CELLMARKERCURNCD4CENTRALMEMORYLIKETTCMLIK…																														
CELLMARKERCURNCD4CYTOTOXICT1CELL																														
CELLMARKERCURNCD4CYTOTOXICT2CELL																														
CELLMARKERCURNCD4THELPERCELL																														
CELLMARKERCURNCHRONICALLYACTIVATEDCD4TCELL																														
CELLMARKERCURNCHRONICALLYACTIVATEDCD8TCELL																														
CELLMARKERCURNCLASSSWITCHEDMEMORYBIGGU03B…																														
CELLMARKERCURNCLEC10ADENDRITICCELL																														
CELLMARKERCURNCLEC9ADENDRITICCELL																														
CELLMARKERCURNCONVENTIONALDENDRITICCELL2B…																														
CELLMARKERCURNCONVENTIONALDENDRITICCELL2B…																														
CELLMARKERCURNCORTICALSOMATOSTATINSSTINTE…																														
CELLMARKERCURNCYTOTOXICCD4T2CELL																														
CELLMARKERCURNCYTOTOXICCD4TCELL																														
CELLMARKERCURND1MSNMEDIUMSPINYNEURON																														
CELLMARKERCURND2MSNMEDIUMSPINYNEURON																														
CELLMARKERCURNDISTALTUBULELOOPOFHENLECELL																														
CELLMARKERCURNEARLYCD34MEGAKARYOCYTEERYTH…																														
CELLMARKERCURNEARLYTCELLPROGENITORETP																														
CELLMARKERCURNEFFECTORMEMORYCD4TCELL																														
CELLMARKERCURNEFFECTORREGULATORYTETREGCELL																														
CELLMARKERCURNEFFECTORTTEFFCELL																														
CELLMARKERCURNEXHAUSTEDCD4TCELL																														
CELLMARKERCURNEXHAUSTEDCD8TCELL																														
CELLMARKERCURNEXHAUSTEDCD8TPROGENITORCELL																														
CELLMARKERCURNEXHAUSTEDTTEXCELL																														
CELLMARKERCURNEXTRATELENCEPHALICETCELL																														
CELLMARKERCURNFINALLYHIGHLYEFFECTORTEMRAM…																														
CELLMARKERCURNFOLLICULARREGULATORYTTFRCELL																														
CELLMARKERCURNFOXP3NATURALREGULATORYTTREG…																														
CELLMARKERCURNGAMMADELTAU03B3DTCELL																														
CELLMARKERCURNGERMINALCENTREGCLIKECELL																														
CELLMARKERCURNGLOMERULARENDOTHELIALCELLECG																														
CELLMARKERCURNGRANULOCYTICMYELOIDDERIVEDS…																														
CELLMARKERCURNGRBREGULATORYBCELL																														
CELLMARKERCURNGZMKTCELL																														
CELLMARKERCURNHIGHLYDIFFERENTIATEDEFFECTO…																														
CELLMARKERCURNINDUCEDREGULATORYTTREGCELL																														
CELLMARKERCURNINFLAMMATORYCANCERASSOCIATE…																														
CELLMARKERCURNINNATELYMPHOIDCELLILCS																														
CELLMARKERCURNINTRATHYMICFIBROBLASTFB1																														
CELLMARKERCURNITOCELLHEPATICSTELLATECELL																														
CELLMARKERCURNLYMPHANGIOLEIOMYOMATOSISCEL…																														
CELLMARKERCURNMARGINALZONEMZLIKEBCELL																														
CELLMARKERCURNMEMORYSTEMCELLTSCM																														
CELLMARKERCURNMICROGLIADERIVEDTUMORASSOCI…																														
CELLMARKERCURNMKERYTHCELL																														
CELLMARKERCURNMYOFIBROBLASTICCANCERASSOCI…																														
CELLMARKERCURNNAIVEREGULATORYTTREGCELL																														
CELLMARKERCURNNATURALREGULATORYTTREGCELL																														
CELLMARKERCURNPERIPHERALBLOODMONONUCLEARC…																														
CELLMARKERCURNPITUITARYSTEMCELLPSC																														
CELLMARKERCURNPLASMACYTOIDDENDRITICCELLPDC																														
CELLMARKERCURNPOLYMORPHONUCLEARMYELOIDDER…																														
CELLMARKERCURNPOLYMORPHONUCLEARNEUTROPHIL…																														
CELLMARKERCURNPOSTERIORPRESOMITICMESODERM…																														
CELLMARKERCURNPROLIFERATIVECD4TCELL																														
CELLMARKERCURNPROLIFERATIVECD8TCELL																														
CELLMARKERCURNREDBLOODCELLERYTHROCYTE																														
CELLMARKERCURNREGULATORYBBREGCELL																														
CELLMARKERCURNREGULATORYCD4TCELL																														
CELLMARKERCURNREGULATORYCD8TCELL																														
CELLMARKERCURNREGULATORYTTREGCELL																														

CELLMARKERCAN_ACINAR_CELLS																														
CELLMARKERCURNREGULATORYTTREGCELL																														
CELLMARKERCURNRENALVESICLECOMMASHAPEDBODY…																														
CELLMARKERCURNRESIDENTMEMORYCD8TCELL																														
CELLMARKERCURNREVERTANTMEMORYCD8TTEMRACELL																														
CELLMARKERCURNSCARASSOCIATEDMACROPHAGESAM																														
CELLMARKERCURNSENESCENTCD8TCELL																														
CELLMARKERCURNSTEMPROGENITORCELL																														
CELLMARKERCURNSWITCHEDMEMORYBCELLSWMB																														
CELLMARKERCURNT1TRANSITIONALBCELL																														
CELLMARKERCURNT2TRANSITIONALBCELL																														
CELLMARKERCURNTCELLNATURALKILLERCELL																														
CELLMARKERCURNTERMINALEFFECTORCD8TCELL																														
CELLMARKERCURNTERMINALLYDIFFERENTIATEDCD8…																														
CELLMARKERCURNTFOLLICULARHELPERTFHCELL																														
CELLMARKERCURNTHELPER1TH1CELL																														
CELLMARKERCURNTHELPER1TH1CELL																														
CELLMARKERCURNTHELPER2TH2CELL																														
CELLMARKERCURNTHELPER2TH2CELL																														
CELLMARKERCURNTHELPER9TH9CELL																														
CELLMARKERCURNTHELPERTHCELL																														
CELLMARKERCURNTHYMICEPITHELIALCELLTEC																														
CELLMARKERCURNTISSUERESIDENTMEMORYCD4TCELL																														
CELLMARKERCURNTISSUERESIDENTMEMORYCD8TCELL																														
CELLMARKERCURNTISSUERESIDENTMEMORYTTRMCELL																														
CELLMARKERCURNTPERIPHERALHELPERTPHCELL																														
CELLMARKERCURNTRANSIENTAMPLIFYINGTACELL																														
CELLMARKERCURNTRANSITIONALEXHAUSTEDCD8TCE…																														
CELLMARKERCURNTUMORINFILTRATINGLYMPHOCYTE…																														
CELLMARKERCURNUNSWITCHEDMEMORYBCELLUNSWMB																														
CELLMARKERCURNVASARECTAENDOTHELIALCELLECVR																														
CELLMARKERCURNVONECONOMONEURONVEN																														
CELLMARKERCURNYOLKSACDERIVEDMYELOIDBIASED…																														
CELLMARKEREXP_ACINAR_CELLS																														
CELLMARKEREXP_ADRENERGIC_NEURONS																														
CELLMARKEREXP_AIRWAY_EPITHELIAL_CELLS																														
CELLMARKEREXP_AIRWAY_SMOOTH_MUSCLE_CELLS																														
CELLMARKEREXP_ALPHA_CELLS																														
CELLMARKEREXP_ANTERIOR_PITUITARY_GLAND_CE…																														
CELLMARKEREXP_ASTROCYTES																														
CELLMARKEREXP_B_CELLS																														
CELLMARKEREXP_B_CELLS_MEMORY																														
CELLMARKEREXP_B_CELLS_NAIVE																														
CELLMARKEREXP_BASAL_CELLS																														
CELLMARKEREXP_BASOPHILS																														
CELLMARKEREXP_BERGMANN_GLIA																														
CELLMARKEREXP_BETA_CELLS																														
CELLMARKEREXP_CAJAL_RETZIUS_CELLS																														
CELLMARKEREXP_CHOLANGIOCYTES																														
CELLMARKEREXP_CHOLINERGIC_NEURONS																														
CELLMARKEREXP_CHONDROCYTES																														
CELLMARKEREXP_DELTA_CELLS																														
CELLMARKEREXP_DISTAL_TUBULE_CELLS																														
CELLMARKEREXP_DUCTAL_CELLS																														
CELLMARKEREXP_EMBRYONIC_STEM_CELLS																														
CELLMARKEREXP_ENTERIC_NEURONS																														
CELLMARKEREXP_ENTEROENDOCRINE_CELLS																														
CELLMARKEREXP_EPENDYMAL_CELLS																														
CELLMARKEREXP_EPSILON_CELLS																														
CELLMARKEREXP_ERYTHROID_LIKE_AND_ERYTHROI…																														
CELLMARKEREXP_GAMMA_DELTA_T_CELLS																														
CELLMARKEREXP_GOBLET_CELLS																														
CELLMARKEREXP_HEMANGIOBLASTS																														
CELLMARKEREXP_HEMATOPOIETIC_STEM_CELLS																														
CELLMARKEREXP_HEPATIC_STELLATE_CELLS																														
CELLMARKEREXP_HIS_BUNDLE_CELLS																														
CELLMARKEREXP_IMMATURE_NEURONS																														
CELLMARKEREXP_JUXTAGLOMERULAR_CELLS																														
CELLMARKEREXP_KERATINOCYTES																														
CELLMARKEREXP_KIDNEY_PROGENITOR_CELLS																														
CELLMARKEREXP_KUPFFER_CELLS																														
CELLMARKEREXP_LEYDIG_CELLS																														
CELLMARKEREXP_LOOP_OF_HENLE_CELLS																														
CELLMARKEREXP_LUMINAL_EPITHELIAL_CELLS																														
CELLMARKEREXP_MAMMARY_EPITHELIAL_CELLS																														
CELLMARKEREXP_MEGAKARYOCYTES																														
CELLMARKEREXP_MELANOCYTES																														
CELLMARKEREXP_MESANGIAL_CELLS																														
CELLMARKEREXP_MESOTHELIAL_CELLS																														
CELLMARKEREXP_MICROFOLD_CELLS																														
CELLMARKEREXP_MICROGLIA																														
CELLMARKEREXP_MONOCYTES																														
CELLMARKEREXP_MULLER_CELLS																														
CELLMARKEREXP_MYOBLASTS																														
CELLMARKEREXP_MYOCYTES																														
CELLMARKEREXP_MYOFIBROBLASTS																														
CELLMARKEREXP_NEUROBLASTS																														
CELLMARKEREXP_NEUTROPHILS																														
CELLMARKEREXP_NK_CELLS																														
CELLMARKEREXP_NORADRENERGIC_NEURONS																														
CELLMARKEREXP_OLIGODENDROCYTES																														
CELLMARKEREXP_OSTEOBLASTS																														
CELLMARKEREXP_OSTEOCLAST_PRECURSOR_CELLS																														
CELLMARKEREXP_OSTEOCLASTS																														
CELLMARKEREXP_OSTEOCYTES																														
CELLMARKEREXP_PANETH_CELLS																														
CELLMARKEREXP_PARATHYROID_CHIEF_CELLS																														
CELLMARKEREXP_PERICYTES																														
CELLMARKEREXP_PHOTORECEPTOR_CELLS																														
CELLMARKEREXP_PLASMA_CELLS																														
CELLMARKEREXP_PLASMACYTOID_DENDRITIC_CELLS																														
CELLMARKEREXP_PODOCYTES																														
CELLMARKEREXP_PRINCIPAL_CELLS																														
CELLMARKEREXP_PROXIMAL_TUBULE_CELLS																														
CELLMARKEREXP_PULMONARY_ALVEOLAR_TYPE_I_C…																														

CELLMARKERCAN_ACINAR_CELLS																														
CELLMARKEREXP_PULMONARY_ALVEOLAR_TYPE_II_…																														
CELLMARKEREXP_PURKINJE_FIBER_CELLS																														
CELLMARKEREXP_PURKINJE_NEURONS																														
CELLMARKEREXP_PYRAMIDAL_CELLS																														
CELLMARKEREXP_RETICULOCYTES																														
CELLMARKEREXP_RETINAL_GANGLION_CELLS																														
CELLMARKEREXP_SALIVARY_MUCOUS_CELLS																														
CELLMARKEREXP_SATELLITE_CELLS																														
CELLMARKEREXP_SCHWANN_CELLS																														
CELLMARKEREXP_SEROTONERGIC_NEURONS																														
CELLMARKEREXP_SERTOLI_CELLS																														
CELLMARKEREXP_SMOOTH_MUSCLE_CELLS																														
CELLMARKEREXP_SPERMATOZOA																														
CELLMARKEREXP_STROMAL_CELLS																														
CELLMARKEREXP_T_CELLS																														
CELLMARKEREXP_T_CELLS_NAIVE																														
CELLMARKEREXP_T_CYTOTOXIC_CELLS																														
CELLMARKEREXP_T_HELPER_CELLS																														
CELLMARKEREXP_T_MEMORY_CELLS																														
CELLMARKEREXP_TRICHOCYTES																														
CELLMARKEREXP_TRIGEMINAL_NEURONS																														
CELLMARKEREXP_TROPHOBLAST_CELLS																														
CELLMARKEREXP_TROPHOBLAST_PROGENITOR_CELLS																														
CELLMARKEREXP_TUFT_CELLS																														
CELLMARKEREXP_VASCULAR_SMOOTH_MUSCLE_CELLS																														
CELLMARKEREXPENDOTHELIALCELLSAORTA																														
CELLMARKEREXPNEURALSTEMPRECURSORCELLS																														
CELLMARKERMSIGDB_AIZARANI_LIVER_C12_NK_NK…																														
CELLMARKERMSIGDB_AIZARANI_LIVER_C22_RESID…																														
CELLMARKERMSIGDB_AIZARANI_LIVER_C28_NK_NK…																														
CELLMARKERMSIGDB_AIZARANI_LIVER_C29_MVECS…	ELK3	FGD5	UPP1	INPP1	FILIP…	LIMA1	PTPRG	CCL15…	PARVA	TLR4	B4GAL…	ID3	OMD	TTC28	NUDT10	NEDD9	CMIP	LTBP4	SULF2	CD320	SERPI…	EHD4	NIBAN2	TP53I…	CLDN5	ARL4A	TNS2	SNTB2	INMT	CLEC3B
CELLMARKERMSIGDB_AIZARANI_LIVER_C30_HEPAT…	GNL3L	SERAC1	MOGAT3	GSDMB	ARL10	CHDH	VSIG1	ACACB	ZNF397	SEC14…	GTDC1	SLC25…	HPN	PHF6	LINC0…	A1CF	ACER3	FAM16…	DSG2	AIPL1	PROX1	FBXO21	LINC0…	PNISR	ZNF426	SIK2	GCGR	ARG1	SVOP	ACAD11
CELLMARKERMSIGDB_AIZARANI_LIVER_C8_RESIDE…																														
CELLMARKERMSIGDB_AIZARANI_LIVER_C9_LSECS_1	PRKAR…	RHOJ	MYO6	COL6A2	FLNB	MACF1	SPRY1	TUBB6	SGK1	DOCK9	PCDH17	KLF7	FHL1	NOVA2	CNN3	BST2	HSP90…	BSDC1	ANGPT…	BAIAP2	GRB10	PDGFB	NTN4	FCGRT	TMOD3	CTSD	ST6GA…	NOS3	PARP14	TNFRS…
CELLMARKERMSIGDB_BUSSLINGER_DUODENAL_BCHE…																														
CELLMARKERMSIGDB_BUSSLINGER_DUODENAL_DIFF…	RPL38	RPL10	RPL30	RPLP2	EI24	APEX1	HNRNPC	RPS20	KRT8	HMGN1	COX7C	ATP5PD	RPL23	SRSF1	EIF3A	EIF2S3	ECH1	RPS25	HNRNPM	VDAC1	RBM3	SRSF3	XRCC5	BTF3	HADH	EIF3E	RPS9	UQCRF…	EPCAM	MACRO…
CELLMARKERMSIGDB_BUSSLINGER_DUODENAL_EC_C…																														
CELLMARKERMSIGDB_BUSSLINGER_DUODENAL_IMMU…	KCNAB2	CD6	UBA52	DRAP1	ARPC1B	MYO1F	MYBL1	GRK2	MACF1	TNRC6C	MOB3A	SLA2	LAIR1	DDX3Y	TACC1	GIMAP1	RORA	SIT1	DENND…	AUTS2	WNK1	PLEKH…	PRKAR…	SYTL3	ACTR3	RPL28	KIT	HLA-D…	H1-10	LBH
CELLMARKERMSIGDB_BUSSLINGER_DUODENAL_STEM…	ADH1C	HNRNPD	TM9SF3	CBX3	DDAH1	SYNCR…	AHCY	ATF3	EIF3A	BANF1	RPL36	DSP	RPL13	SFPQ	HNRNPU	PDLIM1	IPO7	TOP2B	HNRNPM	CNBP	RPL39	RPS15	SRP9	PAPOLA	SYNE2	HDAC2	RPL27A	RPL18	KHDRB…	SRSF6
CELLMARKERMSIGDB_BUSSLINGER_DUODENAL_TUFT…																														
CELLMARKERMSIGDB_BUSSLINGER_ESOPHAGEAL_EA…																														
CELLMARKERMSIGDB_BUSSLINGER_ESOPHAGEAL_PR…																														
CELLMARKERMSIGDB_BUSSLINGER_GASTRIC_CHIEF…																														
CELLMARKERMSIGDB_BUSSLINGER_GASTRIC_G_CEL…																														
CELLMARKERMSIGDB_BUSSLINGER_GASTRIC_ISTHM…	VMP1	YWHAB	NANS	MYDGF	HMGB2	EFHD2	HMGCS1	FBLIM1	GALNT6	SNRPG	DSP	PSMA4	SNRPD1	ETNK1	MYOF	GARS1	SPINK1	STMN1	PTTG1…	RAB27B	VPS35	ALDH2	MARCKS	MICU1	RETSAT	GNA11	HES1	MVD	U2AF1	KIF1C
CELLMARKERMSIGDB_BUSSLINGER_GASTRIC_NECK_…																														
CELLMARKERMSIGDB_BUSSLINGER_GASTRIC_OXYNT…																														
CELLMARKERMSIGDB_BUSSLINGER_GASTRIC_PREZY…																														
CELLMARKERMSIGDB_BUSSLINGER_GASTRIC_REG3A…																														
CELLMARKERMSIGDB_CUI_DEVELOPING_HEART_5TH…																														
CELLMARKERMSIGDB_CUI_DEVELOPING_HEART_5TH…																														
CELLMARKERMSIGDB_CUI_DEVELOPING_HEART_C1_…																														
CELLMARKERMSIGDB_CUI_DEVELOPING_HEART_C2_…																														
CELLMARKERMSIGDB_CUI_DEVELOPING_HEART_COM…																														
CELLMARKERMSIGDB_CUI_DEVELOPING_HEART_COR…																														
CELLMARKERMSIGDB_CUI_DEVELOPING_HEART_END…																														
CELLMARKERMSIGDB_CUI_DEVELOPING_HEART_LEF…																														
CELLMARKERMSIGDB_CUI_DEVELOPING_HEART_RIG…																														
CELLMARKERMSIGDB_CUI_DEVELOPING_HEART_SMO…																														
CELLMARKERMSIGDB_DESCARTES_FETAL_ADRENAL_…																														
CELLMARKERMSIGDB_DESCARTES_FETAL_CEREBELL…																														
CELLMARKERMSIGDB_DESCARTES_FETAL_CEREBELL…																														
CELLMARKERMSIGDB_DESCARTES_FETAL_CEREBELL…	CEP29…	ENSG0…	HK2	CCR1	TMIGD3	ALOX5	NFKBID	CD37	SPTLC3	MMRN1	LINC0…	AZIN2	ARHGA…	FAM14…	SIGLE…	LY96	SP140	GPR183	TNFSF…	NMRK1	CH25H	FCER1G	PDE4D…	TASL	ARHGA…	AIF1	TMEM1…	GGTA1	UNC93…	MFSD1
CELLMARKERMSIGDB_DESCARTES_FETAL_CEREBELL…																														
CELLMARKERMSIGDB_DESCARTES_FETAL_CEREBELL…																														
CELLMARKERMSIGDB_DESCARTES_FETAL_CEREBELL…																														
CELLMARKERMSIGDB_DESCARTES_FETAL_CEREBELL…	CD109	HBG1	CD320	ID1	PRKCH	GIMAP1	SLC26…	SLFN5	CALD1	SH2D3C	KCNJ2	TIMP1	DCBLD1	SLC38…	LAMB2	LRRC55	SAMM50	C1orf…	CCM2L	FGD5	MAP4K2	CCDC3	SLC1A4	PLXDC1	NOTCH3	MYL12B	IQGAP1	FLVCR2	TNFAI…	CAPNS1
CELLMARKERMSIGDB_DESCARTES_FETAL_CEREBRUM…																														
CELLMARKERMSIGDB_DESCARTES_FETAL_CEREBRUM…																														
CELLMARKERMSIGDB_DESCARTES_FETAL_CEREBRUM…	LINC0…	RPUSD3	CD200…	PNMA8B	NAP1L5	RNF215	RPL37…	ZNG1C	AKIRI…	GTPBP6	TMEM40	CCDC61	SYDE2	CMAHP	KCNJ4	ENSG0…	CYP1B…	PIP4P2	THOC6	CISH	RTL5	ZFP28	PTGIR	RAD9A	PPIF	C19or…	CDC6	RASL1…	ENDOV	SLC39…
CELLMARKERMSIGDB_DESCARTES_FETAL_CEREBRUM…	TLR5	LAIR1	UNC93…	LINC0…	SLC29…	SASH3	FMNL1	DENND…	SULT1…	ERMAP	SUCNR1	MILR1	PRAM1	CHEK2	LY75	STYXL2	ENSG0…	RCN3	SPNS3	NCF2	CXCL16	CD180	COL8A2	IL7R	EVI2B	IGSF10	AIF1	SLFN11	MR1	SLC37…
CELLMARKERMSIGDB_DESCARTES_FETAL_CEREBRUM…																														
CELLMARKERMSIGDB_DESCARTES_FETAL_CEREBRUM…																														
CELLMARKERMSIGDB_DESCARTES_FETAL_CEREBRUM…	LIMS2	PPM1H	FSTL1	LRRC32	RGCC	FLVCR2	SHROO…	NODAL	ITGA4	STARD8	C11or…	TNFAI…	GIMAP6	CCN2	TPT1P…	CDC42…	HIGD1B	NPIPB4	GIMAP7	PCOLCE	CSTB	EML3	OSMR	SNAI2	NOS3	FHL3	MEGF6	GPR4	CLEC1…	AGRN
CELLMARKERMSIGDB_DESCARTES_FETAL_EYE_ASTR…																														
CELLMARKERMSIGDB_DESCARTES_FETAL_EYE_GANG…																														
CELLMARKERMSIGDB_DESCARTES_FETAL_EYE_LENS…																														
CELLMARKERMSIGDB_DESCARTES_FETAL_EYE_PHOT…																														
CELLMARKERMSIGDB_DESCARTES_FETAL_EYE_RETI…																														
CELLMARKERMSIGDB_DESCARTES_FETAL_EYE_SMOO…																														
CELLMARKERMSIGDB_DESCARTES_FETAL_EYE_STRO…																														
CELLMARKERMSIGDB_DESCARTES_FETAL_EYE_VASC…																														
CELLMARKERMSIGDB_DESCARTES_FETAL_HEART_EL…																														
CELLMARKERMSIGDB_DESCARTES_FETAL_HEART_EN…																														
CELLMARKERMSIGDB_DESCARTES_FETAL_HEART_LY…																														
CELLMARKERMSIGDB_DESCARTES_FETAL_HEART_SC…																														
CELLMARKERMSIGDB_DESCARTES_FETAL_HEART_SM…																														
CELLMARKERMSIGDB_DESCARTES_FETAL_HEART_ST…																														
CELLMARKERMSIGDB_DESCARTES_FETAL_HEART_VI…																														
CELLMARKERMSIGDB_DESCARTES_FETAL_INTESTIN…																														
CELLMARKERMSIGDB_DESCARTES_FETAL_INTESTIN…																														
CELLMARKERMSIGDB_DESCARTES_FETAL_INTESTIN…																														
CELLMARKERMSIGDB_DESCARTES_FETAL_INTESTIN…																														
CELLMARKERMSIGDB_DESCARTES_FETAL_INTESTIN…																														
CELLMARKERMSIGDB_DESCARTES_FETAL_INTESTIN…																														
CELLMARKERMSIGDB_DESCARTES_FETAL_KIDNEY_M…																														
CELLMARKERMSIGDB_DESCARTES_FETAL_KIDNEY_U…	CFAP1…	IVL	ELMO3	TFAP2…	TNS4	PKP1	GPC1-…	MUC15	PSORS…	PM20D1	ENSG0…	LINC0…	WNT6	PAK6	FMO9P	RPL21…	KRT80	ARHGE…	MFSD6L	CYP4F3	MSX2	TFAP2C	SYT8	GJB3	LINC0…	PRR15	LDHD	CCDC9B	GJB6	AGAP11
CELLMARKERMSIGDB_DESCARTES_FETAL_LIVER_ER…																														
CELLMARKERMSIGDB_DESCARTES_FETAL_LIVER_HE…																														
CELLMARKERMSIGDB_DESCARTES_FETAL_LIVER_HE…	CFHR1	NGEF	MST1P2	SOAT2	EHHADH	HABP2	AZGP1	TRIM55	PROC	IGFBP1	ALDH4…	KIF12	ACSM2B	MOGAT3	CCDC1…	LINC0…	SMLR1	CREB3…	HKDC1	RNF128	STEAP…	ACSM2A	C4BPB	PPARG…	ARSL	FETUB	GCGR	CAMK2…	LINC0…	MST1L
CELLMARKERMSIGDB_DESCARTES_FETAL_LIVER_ME…	CSDC2	CFTR	MDGA1	ITGB4	CYP2W1	DLGAP2	TPBG	GRIN2A	MEIS1…	GPC4	RGMA	CFAP2…	RTL8A	NETO1	CNTN3	PRR36	MPP2	LAMC2	OSR1	EFNA4	PDLIM4	ABCG4	LOX	C1QL1	SDR42…	EVPL	LINC0…	JPH4	CFAP2…	ATF3
CELLMARKERMSIGDB_DESCARTES_FETAL_LUNG_BRO…																														
CELLMARKERMSIGDB_DESCARTES_FETAL_LUNG_CIL…	DAW1	TTC23L	IK	ANKRD…	C7orf…	EFCAB…	FAM22…	CFAP95	EFCAB…	CCDC1…	C11or…	DZANK1	RPGR	ZDHHC1	EFHB	TTLL10	ODAD4	TEKT4	WDR93	ODAD1	RPGRI…	TTC34	C2orf…	KIF27	LCA5	USP2	GRIN3B	LINC0…	ENO4	CERKL
CELLMARKERMSIGDB_DESCARTES_FETAL_LUNG_LYM…																														

CELLMARKERCAN_ACINAR_CELLS																														
CELLMARKERMSIGDB_DESCARTES_FETAL_LUNG_NEU…																														
CELLMARKERMSIGDB_DESCARTES_FETAL_LUNG_STR…																														
CELLMARKERMSIGDB_DESCARTES_FETAL_LUNG_VAS…																														
CELLMARKERMSIGDB_DESCARTES_FETAL_MUSCLE_L…																														
CELLMARKERMSIGDB_DESCARTES_FETAL_MUSCLE_S…																														
CELLMARKERMSIGDB_DESCARTES_FETAL_MUSCLE_S…																														
CELLMARKERMSIGDB_DESCARTES_FETAL_MUSCLE_V…																														
CELLMARKERMSIGDB_DESCARTES_FETAL_PANCREAS…																														
CELLMARKERMSIGDB_DESCARTES_FETAL_PANCREAS…																														
CELLMARKERMSIGDB_DESCARTES_FETAL_PANCREAS…																														
CELLMARKERMSIGDB_DESCARTES_FETAL_PANCREAS…																														
CELLMARKERMSIGDB_DESCARTES_FETAL_PANCREAS…																														
CELLMARKERMSIGDB_DESCARTES_FETAL_PANCREAS…																														
CELLMARKERMSIGDB_DESCARTES_FETAL_PANCREAS…																														
CELLMARKERMSIGDB_DESCARTES_FETAL_PLACENTA…																														
CELLMARKERMSIGDB_DESCARTES_FETAL_PLACENTA…																														
CELLMARKERMSIGDB_DESCARTES_FETAL_PLACENTA…																														
CELLMARKERMSIGDB_DESCARTES_FETAL_PLACENTA…																														
CELLMARKERMSIGDB_DESCARTES_FETAL_PLACENTA…																														
CELLMARKERMSIGDB_DESCARTES_FETAL_SPLEEN_E…																														
CELLMARKERMSIGDB_DESCARTES_FETAL_SPLEEN_S…																														
CELLMARKERMSIGDB_DESCARTES_FETAL_SPLEEN_V…																														
CELLMARKERMSIGDB_DESCARTES_FETAL_STOMACH_…	PIERC…	ZDHHC1	WDR93	NME9	CFAP3…	TSPAN…	IL5RA	FBXW9	DOC2A	C7orf…	DNALI1	SPEF1	CCDC65	FUZ	SPACA9	LCA5L	TROAP…	CCDC13	SLFN13	EFHB	DNAAF8	RSPH14	OXTR	MB	LINC0…	CCNO	LRRC1…	FAM81A	CCDC78	WDR38
CELLMARKERMSIGDB_DESCARTES_FETAL_STOMACH_…																														
CELLMARKERMSIGDB_DESCARTES_FETAL_STOMACH_…																														
CELLMARKERMSIGDB_DESCARTES_FETAL_STOMACH_…																														
CELLMARKERMSIGDB_DESCARTES_FETAL_STOMACH_…																														
CELLMARKERMSIGDB_DESCARTES_FETAL_STOMACH_…																														
CELLMARKERMSIGDB_DESCARTES_FETAL_STOMACH_…																														
CELLMARKERMSIGDB_DESCARTES_FETAL_STOMACH_…																														
CELLMARKERMSIGDB_DESCARTES_FETAL_STOMACH_…																														
CELLMARKERMSIGDB_DESCARTES_FETAL_STOMACH_…																														
CELLMARKERMSIGDB_DESCARTES_FETAL_STOMACH_…																														
CELLMARKERMSIGDB_DESCARTES_FETAL_STOMACH_…																														
CELLMARKERMSIGDB_DESCARTES_FETAL_THYMUS_T…																														
CELLMARKERMSIGDB_DESCARTES_MAIN_FETAL_ACI…																														
CELLMARKERMSIGDB_DESCARTES_MAIN_FETAL_ADR…																														
CELLMARKERMSIGDB_DESCARTES_MAIN_FETAL_AST…																														
CELLMARKERMSIGDB_DESCARTES_MAIN_FETAL_BIP…																														
CELLMARKERMSIGDB_DESCARTES_MAIN_FETAL_BRO…																														
CELLMARKERMSIGDB_DESCARTES_MAIN_FETAL_CAR…																														
CELLMARKERMSIGDB_DESCARTES_MAIN_FETAL_CIL…	ZNF474	ENSG0…	C5orf…	NME5	IK	SCGB3…	C5orf…	SOX30	FABP6	FABP6…	TEX56P	C6orf…	STMND1	LNC-L…	ARMH2	ZNF20…	ZNF391	HCG14	SAPCD…	SLC44…	TCP11	TREM1	LRRC73	RSPH9	TCTE1	SPATS1	ANKRD…	CRISP2	EFHC1	EEF1B…
CELLMARKERMSIGDB_DESCARTES_MAIN_FETAL_DUC…																														
CELLMARKERMSIGDB_DESCARTES_MAIN_FETAL_END…																														
CELLMARKERMSIGDB_DESCARTES_MAIN_FETAL_ENS…																														
CELLMARKERMSIGDB_DESCARTES_MAIN_FETAL_EPI…																														
CELLMARKERMSIGDB_DESCARTES_MAIN_FETAL_GAN…																														
CELLMARKERMSIGDB_DESCARTES_MAIN_FETAL_GOB…																														
CELLMARKERMSIGDB_DESCARTES_MAIN_FETAL_HEM…																														
CELLMARKERMSIGDB_DESCARTES_MAIN_FETAL_HOR…																														
CELLMARKERMSIGDB_DESCARTES_MAIN_FETAL_INH…																														
CELLMARKERMSIGDB_DESCARTES_MAIN_FETAL_INH…																														
CELLMARKERMSIGDB_DESCARTES_MAIN_FETAL_INT…																														
CELLMARKERMSIGDB_DESCARTES_MAIN_FETAL_ISL…																														
CELLMARKERMSIGDB_DESCARTES_MAIN_FETAL_LYM…																														
CELLMARKERMSIGDB_DESCARTES_MAIN_FETAL_MES…																														
CELLMARKERMSIGDB_DESCARTES_MAIN_FETAL_MET…																														
CELLMARKERMSIGDB_DESCARTES_MAIN_FETAL_MYE…																														
CELLMARKERMSIGDB_DESCARTES_MAIN_FETAL_NEU…																														
CELLMARKERMSIGDB_DESCARTES_MAIN_FETAL_OLI…																														
CELLMARKERMSIGDB_DESCARTES_MAIN_FETAL_PDE…																														
CELLMARKERMSIGDB_DESCARTES_MAIN_FETAL_PDE…	CASC18	SART3	ALKBH2	MYO1H	TRPV4	MAPKA…	DHX37	MTIF3	STARD…	ELF1	DIS3	GGACT	PIP4P1	RIPK3	ENSG0…	PSMC6	ESR2	COX16	RPL22…	SLIRP	OTUB2	CCNK	WDR25	NDUFB…	BRF1	HERC2…	LINC0…	RTF1	VPS39…	LRRC57
CELLMARKERMSIGDB_DESCARTES_MAIN_FETAL_RET…																														
CELLMARKERMSIGDB_DESCARTES_MAIN_FETAL_SAT…																														
CELLMARKERMSIGDB_DESCARTES_MAIN_FETAL_SCH…																														
CELLMARKERMSIGDB_DESCARTES_MAIN_FETAL_SLC…	PHLDA…	LINC0…	POLR3B	MTERF2	PWP1	UNG	RAD9B	GTF2H3	NUFIP1	PPP1R…	PHF11	EMC9	DHRS1	CBLN3	PRPF39	RPS15…	KLHDC2	FNTB	COQ6	VRTN	LINC0…	PDCD6…	ULK4P1	DPH6	KNSTRN	THAP10	UNC45A	LINC0…	EME2	ZNF597
CELLMARKERMSIGDB_DESCARTES_MAIN_FETAL_SMO…																														
CELLMARKERMSIGDB_DESCARTES_MAIN_FETAL_STE…																														
CELLMARKERMSIGDB_DESCARTES_MAIN_FETAL_SYM…																														
CELLMARKERMSIGDB_DESCARTES_MAIN_FETAL_UNI…																														
CELLMARKERMSIGDB_DESCARTES_MAIN_FETAL_URE…																														
CELLMARKERMSIGDB_DESCARTES_MAIN_FETAL_VAS…																														
CELLMARKERMSIGDB_DESCARTES_MAIN_FETAL_VIS…																														
CELLMARKERMSIGDB_DURANTE_ADULT_OLFACTORY_…																														
CELLMARKERMSIGDB_DURANTE_ADULT_OLFACTORY_…																														
CELLMARKERMSIGDB_DURANTE_ADULT_OLFACTORY_…																														
CELLMARKERMSIGDB_DURANTE_ADULT_OLFACTORY_…																														
CELLMARKERMSIGDB_DURANTE_ADULT_OLFACTORY_…																														
CELLMARKERMSIGDB_DURANTE_ADULT_OLFACTORY_…																														
CELLMARKERMSIGDB_DURANTE_ADULT_OLFACTORY_…																														
CELLMARKERMSIGDB_DURANTE_ADULT_OLFACTORY_…																														
CELLMARKERMSIGDB_DURANTE_ADULT_OLFACTORY_…																														
CELLMARKERMSIGDB_DURANTE_ADULT_OLFACTORY_…																														
CELLMARKERMSIGDB_DURANTE_ADULT_OLFACTORY_…																														
CELLMARKERMSIGDB_DURANTE_ADULT_OLFACTORY_…																														
CELLMARKERMSIGDB_DURANTE_ADULT_OLFACTORY_…																														
CELLMARKERMSIGDB_DURANTE_ADULT_OLFACTORY_…																														
CELLMARKERMSIGDB_DURANTE_ADULT_OLFACTORY_…																														
CELLMARKERMSIGDB_DURANTE_ADULT_OLFACTORY_…																														
CELLMARKERMSIGDB_FAN_EMBRYONIC_CTX_ASTROC…																														
CELLMARKERMSIGDB_FAN_EMBRYONIC_CTX_BIG_GR…	MAP4K2	BST2	PARVA	LY6E	KLF4	HYAL2	TEK	TNS1	MGST2	COL6A1	IL32	LMO2	PTPRB	ZIC2	AEBP1	ZEB1	AFAP1…	HLA-H	PDLIM2	SLC7A8	MYL12A	WFS1	S100A…	ACTN1	FXYD5	LMNA	CLEC1…	SLC43…	SMTN	THSD1
CELLMARKERMSIGDB_FAN_EMBRYONIC_CTX_BIG_GR…	ME1	SLC18…	ATP5ME	NDUFA1	MAP1A	TMEM2…	BCAR3	WRAP73	PYGB	RCAN1	ATP5PD	SLC17…	MARCK…	TCTA	ZIC5	GSTT1	TOLLIP	PPP2R…	GHRH	GRM2	ACKR1	HSD17…	PTPRN	NCDN	NTRK1	MAST4	FAIM2	DMRT3	LINC0…	VAT1L
CELLMARKERMSIGDB_FAN_EMBRYONIC_CTX_BIG_GR…																														
CELLMARKERMSIGDB_FAN_EMBRYONIC_CTX_BIG_GR…	LGALS9	OSTF1	IFI16	PLA2G…	RHOG	MYO1F	HSPA1A	SP100	ARHGD…	MFSD1	PPP1R…	TPP1	UCP2	KLF2	IPCEF1	RCSD1	TGFBR2	FOS	CTSL	MAN2B1	TMEM3…	JUNB	H1-2	ASAH1	ARHGA…	TCIRG1	SLC7A8	B3GNT5	PTPRC	SORL1
CELLMARKERMSIGDB_FAN_EMBRYONIC_CTX_BRAIN_…	PPIC	GPD1L	BMPR2	PFKL	UTRN	GGT5	RGS3	SLC16…	FOXF2	SORBS3	AFDN	ADGRL2	LMCD1	ARAP3	STX3	IFI44	NES	SH2D3C	STXBP1	RRAGA	LAPTM…	PRSS12	SOX7	TTYH2	CARHS…	MMRN2	PDXK	PXN	TSPAN6	PTPRG
CELLMARKERMSIGDB_FAN_EMBRYONIC_CTX_BRAIN_…	PDE8A	NODAL	INPP4B	PLEKH…	EPB41…	TNFRS…	BCHE	DGKH	SLC43…	CSAG1	APOD	AMOTL1	TBX18	FAM16…	SASH1	C12or…	PDE5A	CCR10	MDK	PMP22	COX7B	GGT1	LUM	NR2F1	STARD…	TBXA2R	ITSN1	CCDC80	PDLIM2	RAPGE…
CELLMARKERMSIGDB_FAN_EMBRYONIC_CTX_EX_1_E…																														
CELLMARKERMSIGDB_FAN_EMBRYONIC_CTX_IN_2_I…																														
CELLMARKERMSIGDB_FAN_EMBRYONIC_CTX_IN_3_I…																														
CELLMARKERMSIGDB_FAN_EMBRYONIC_CTX_IN_5_I…																														
CELLMARKERMSIGDB_FAN_EMBRYONIC_CTX_IN_6_I…																														
CELLMARKERMSIGDB_FAN_EMBRYONIC_CTX_MICROG…																														
CELLMARKERMSIGDB_FAN_EMBRYONIC_CTX_OLIG	SHTN1	STX4	OSBPL…	CNTN1	TMEM1…	MAPRE2	C1orf…	RRAS2	TBC1D…	RAB33A	SVIP	ZNF189	APCDD1	ATP10B	NENF	SLC27…	H2AJ	BEST1	PEDS1	AMOTL2	NIPA1	ACSBG1	NACAD	SPATA2	DEGS1	PCBP4	MBNL2	CA2	FAM22…	SORT1
CELLMARKERMSIGDB_FAN_EMBRYONIC_CTX_OPC																														

Table S5: Cell state marker gene list

CELLMARKERCAN_ACINAR_CELLS																														
CELLMARKERMSIGDB_FAN_OVARY_CL1_GPRC5A_TNF…	UBA2	RPLP0	DCAF13	TRMT1…	SPART	TPT1	GEM	ANXA5	RPL18	FHL2	EIF5	RHEB	SNHG32	VAPA	NAA50	DNAJB6	APP	OSER1	PSMC4	BAG2	SBDS	AK6	TFAM	LHFPL6	G3BP1	SDC2	STAT3	PNPLA8	ATG101	GSTM3
CELLMARKERMSIGDB_FAN_OVARY_CL11_MURAL_GRA…	SCD	TSHZ2	RAB5A	STIP1	RAB2A	BUD31	MDK	SYNE2	DSEL	PTP4A1	RTN4	PLIN3	OTUD7B	FOSL2	MARCH…	FAM17…	FKBP4	MT-ND4	ZNF281	YWHAG	VIT	NFE2L2	KTN1	ATF4	METAP2	HMOX1	SINHC…	ATXN1	ARF4	MYO6
CELLMARKERMSIGDB_FAN_OVARY_CL13_MONOCYTE_…	ARL6I…	CALHM6	FPR3	BLOC1…	RHOG	ACTR2	LGALS9	SNN	ATP6V…	LGMN	RIT1	ATP13…	JARID2	WAS	AHR	ZEB2	NOP10	S100A…	MACRO…	EFHD2	ABRACL	CFL1	CREM	CTSL	GPX4	MFSD1	MPP1	FERMT3	SLC16…	M6PR
CELLMARKERMSIGDB_FAN_OVARY_CL14_MATURE_SM…	RHOC	GSTK1	CD164	MRPL33	CTSD	SDHD	BRK1	GNAS	BSG	DUSP1	NUCKS1	TMEM2…	ADI1	MT-ND…	WDR1	CCNI	COPS6	SRSF7	ECH1	VKORC1	ACADVL	WASF2	EIF5A	DCTN3	ADAMT…	CYSTM1	NDUFA3	MDH1	LEPROT	SLIRP
CELLMARKERMSIGDB_FAN_OVARY_CL15_SMALL_ANT…	ATP1B1	UQCRHL	AMHR2	SERPI…	SNHG15	DCUN1…	PIP5K…	AZIN1	PCBP2	MAP3K…	AJUBA	PPP1R…	RIOK3	DSEL	C1orf…	YAF2	INHBB	DUOX1	GNAI3	GRB14	IFRD1	BCAR3	MIR22…	DIPK2A	CSRNP1	RASL1…	NDUFS5	MAGOH	WASF1	PRAME
CELLMARKERMSIGDB_FAN_OVARY_CL18_B_LYMPHOC…	DBI	PPP1R2	SUB1	CFLAR	RAN	DNAJB…	PMEPA1	TGFBR1	POLE3	USP8	TALDO1	KPNA2	LSM1	SEC11C	BLOC1…	MBP	SIVA1	NUDC	ELOF1	BAZ1A	CDC42…	GNG5	AKIRI…	ANKRD…	LRRFI…	EMC3	MRPS6	OFD1	UBA52	RPL27
CELLMARKERMSIGDB_FAN_OVARY_CL2_PUTATIVE_E…																														
CELLMARKERMSIGDB_FAN_OVARY_CL5_HEALTHY_SE…	OSER1	ARL4D	CLK4	HSPA8	FAM21…	SEPTI…	ZNF90	NME3	TWSG1	ACADVL	TSPAN5	RPL22…	MLF2	ZFAND5	FLYWC…	ARHGA…	NOLC1	FILIP…	DARS1	IFI6	RBMS1	TCAF1	ECH1	ARL5B	ATP5MJ	ING1	SLC25…	NONO	AKIRI…	MTDH
CELLMARKERMSIGDB_FAN_OVARY_CL6_PUTATIVE_E…	HS3ST1	EEF1A1	BNIP3	PDCD4	NME3	DDAH2	CAPZB	CYB5A	FXYD6	NPC2	TSC22…	MDK	ENG	PBX1	MPST	SLC40…	KLHDC…	ACTR10	RABAC1	LRPAP1	LAPTM…	ARL1	LGALS3	NUCB2	EPHX1	C4orf3	SELEN…	TPM4	TCEAL8	TALDO1
CELLMARKERMSIGDB_FAN_OVARY_CL8_MATURE_CUM…	SFRP4	FOXL2	CCDC1…	AP2S1	SMS	TMEM1…	PRRT1	PFN1	COA3	PLP1	HMGB1	ECH1	CCDC69	GJA1	IFI27…	VDAC1	NDUFB3	CD47	PRDX5	YAP1	EIF4E…	ATP5M…	SLIRP	NDUFS4	FAM78A	ACTB	PPP1R…	SNRPE	TMEM1…	SEMA7A
CELLMARKERMSIGDB_FAN_OVARY_CL9_PUTATIVE_A…	NASP	HSPA2	PRDX1	HSPE1	POLR2L	UBE2D3	DDX39A	MGAT1	GRPEL1	SERTA…	JAK1	ANKRD…	THBS1	GADD4…	NDUFV2	TGIF1	MT-ND1	CAST	PSME2	CHMP5	FLOT1	SFPQ	DDX3X	GNG5	SINHC…	FOXP1	RBM8A	SYNE2	IFITM2	TOP1
CELLMARKERMSIGDB_GAO_ESOPHAGUS_25W_C1_CIL…	MACF1	CFAP92	TRMT9B	KIAA2…	KIF17	KIF19	KIF27	KIF3A	KIF6	KIF9	KLF3-…	KLHDC9	KLHL32	KNDC1	KSR1	LDLRA…	LGR4	LINC0…	LKAAE…	LMLN	ENSG0…	WAKMA…	LINC0…	GIHCG	LINC0…	LINC0…	LINC0…	LINC0…	LINC0…	ANXA2…
CELLMARKERMSIGDB_GAO_ESOPHAGUS_25W_C2_KRT…																														
CELLMARKERMSIGDB_GAO_ESOPHAGUS_25W_C3_FGF…																														
CELLMARKERMSIGDB_GAO_LARGE_INTESTINE_24W_…																														
CELLMARKERMSIGDB_GAO_LARGE_INTESTINE_24W_…																														
CELLMARKERMSIGDB_GAO_LARGE_INTESTINE_24W_…	NPL	NR4A2	NR4A3	NSMAF	NUFIP1	ODF3B	OGFRL1	OSM	PDE4B	PELI1	PFKFB3	PGD	PIK3A…	PLA2G7	PLAU	PLAUR	PLCB2	PLCG2	PLEK	PLIN2	PLXNC1	PPIF	PRKCB	PRMT9	PTAFR	PTGS2	PTPRC	PTPRE	RAC2	RAP2B
CELLMARKERMSIGDB_GAO_LARGE_INTESTINE_24W_…																														
CELLMARKERMSIGDB_GAO_LARGE_INTESTINE_24W_…																														
CELLMARKERMSIGDB_GAO_LARGE_INTESTINE_24W_…																														
CELLMARKERMSIGDB_GAO_LARGE_INTESTINE_ADUL…	RFX6	RGS4	RGS9	RIIAD1	RIMBP2	RIMS2	RNF183	RTN1	RUNDC…	SAMD3	SCAMP5	SCG2	SCG3	SCG5	SCGN	SCN3A	SCRN1	SCT	SDK2	SEMA3G	SETBP1	SEZ6	SLAIN1	SLC15…	SLC16…	SLC16…	SLC18…	SLC25…	SLC26…	SLC26…
CELLMARKERMSIGDB_GAO_LARGE_INTESTINE_ADUL…	MS4A7	MSANT…	MSLN	MSX1	MTUS2	MUC5AC	MYBPC3	MYH11	MYT1	NANOS1	NBR2	NFATC1	NFATC4	NIN	NKX3-1	NPHP4	NRGN	NRIP2	OBSCN	OGFR	OR1N2	OR5M9	OR7D2	OR7E1…	OVCH1	OXER1	OXGR1	PAXBP…	PCED1B	PDGFRB
CELLMARKERMSIGDB_GAO_LARGE_INTESTINE_ADUL…																														
CELLMARKERMSIGDB_GAO_LARGE_INTESTINE_ADUL…	MZB1	NCF1	NCF1B	NCF1C	NCF4	NCKAP…	NFATC1	NKG7	NLRP1	NR3C1	NR4A3	NUGGC	NXPE3	OAS2	ODF3B	OSBPL3	OSR2	P2RX1	P2RX5	P2RY10	PAIP2B	PAQR6	PARVB	PARVG	PCBP1…	PCED1…	PDE4B	PDK1	PECAM1	PGLYR…
CELLMARKERMSIGDB_GAO_LARGE_INTESTINE_ADUL…	IL33	INHBB	ISLR	ISYNA1	ITGA1	ITGA11	ITGA5	ITGA7	ITIH5	ITPR1	JAK2	JAM2	JAM3	KANK2	KANK3	KCNJ8	JCAD	KIAA1…	KIFC3	KIRRE…	KLHL5	LAMA4	LAMA5	LAMB2	LAMC1	LAMC3	LBH	LCA5L	LCAT	LDB2
CELLMARKERMSIGDB_GAO_SMALL_INTESTINE_24W_…																														
CELLMARKERMSIGDB_GAO_SMALL_INTESTINE_24W_…																														
CELLMARKERMSIGDB_GAO_SMALL_INTESTINE_24W_…																														
CELLMARKERMSIGDB_GAO_SMALL_INTESTINE_24W_…																														
CELLMARKERMSIGDB_GAO_SMALL_INTESTINE_24W_…																														
CELLMARKERMSIGDB_GAO_STOMACH_24W_C1_PROCR…																														
CELLMARKERMSIGDB_GAO_STOMACH_24W_C2_TFF2P…																														
CELLMARKERMSIGDB_GAO_STOMACH_24W_C4_PARIE…																														
CELLMARKERMSIGDB_GAO_STOMACH_24W_C5_PUTAT…																														
CELLMARKERMSIGDB_GAUTAM_EYE_IRIS_CILIARY_…	USP14	ADA2	CTBS	SDCBP	ELMO1	FAT4	TMEM1…	UHRF2	RPLP2	SHE	FAM17…	RIPK3	KMT2C	ALKBH3	ICA1	DYRK1A	PLOD3	CUL2	JAGN1	KDM1A	SEMA6B	MGAT2	DUSP26	RUVBL1	VPS13C	SMG7	SPTLC2	GCA	PRR14	CASP3
CELLMARKERMSIGDB_GAUTAM_EYE_IRIS_CILIARY_…																														
CELLMARKERMSIGDB_GAUTAM_EYE_IRIS_CILIARY_…																														
CELLMARKERMSIGDB_GAUTAM_EYE_IRIS_CILIARY_…																														
CELLMARKERMSIGDB_GAUTAM_EYE_IRIS_CILIARY_…																														
CELLMARKERMSIGDB_HAY_BONE_MARROW_CD34_POS…																														
CELLMARKERMSIGDB_HAY_BONE_MARROW_CD34_POS…																														
CELLMARKERMSIGDB_HAY_BONE_MARROW_CD34_POS…																														
CELLMARKERMSIGDB_HAY_BONE_MARROW_CD34_POS…																														
CELLMARKERMSIGDB_HAY_BONE_MARROW_CD8_T_CE…																														
CELLMARKERMSIGDB_HAY_BONE_MARROW_EARLY_ER…																														
CELLMARKERMSIGDB_HAY_BONE_MARROW_EOSINOPH…																														
CELLMARKERMSIGDB_HAY_BONE_MARROW_NAIVE_T_…	HAPLN3	THEM4	CRIP2	BCL2	THEMIS	CREM	NDFIP1	LRRN3	COX7C	RASGR…	RIC3	TOB1	GIMAP4	SOCS1	FBLN5	GCNT4	PNRC1	P2RY8	SF1	DCTN6…	SELEN…	IL23A	NSG1	ADTRP	RPS26	SUPT3H	TSPYL2	N4BP2…	SH2D3A	CCSER2
CELLMARKERMSIGDB_HAY_BONE_MARROW_NEUTROPH…	ACSL1	STK17B	MSN	POLE4	QSOX1	ACTR2	MBD2	RAB11…	SPG21	ATP6A…	PYGL	DYSF	TMEM2…	CCNL1	PROK2	GM2A	EMILI…	EIF4E3	KYNU	MFSD1	BCL6	FGD4	EHBP1…	SNX10	SMCO4	SKAP2	MIR23…	TLR4	PTGS2	IL17RA
CELLMARKERMSIGDB_HAY_BONE_MARROW_NK_CELLS	GFOD1	PDE6G	ADGRG5	PXN	TSPAN2	TRAPP…	KRT86	STARD…	SLA2	SDF4	BPGM	CFLAR	PRDX5	PITPN…	HDDC2	NDUFB7	RASSF5	C1orf…	SIPA1	AK5	DDX6	TKTL1	MLLT6	TADA3	SLC9A…	TAPBP	CCL3	SHFL	FBXO6	HENMT1
CELLMARKERMSIGDB_HAY_BONE_MARROW_PRE_DEND…																														
CELLMARKERMSIGDB_HAY_BONE_MARROW_PRO_B	CCNF	PIF1	ATP5F…	APBB1…	NCAPD2	BAHCC1	ACADM	PIN1	DCTN3	H2AC20	KPNB1	TXNDC…	INCENP	WDR62	CDK5R…	HNRNP…	PSMB8	HNRNPU	MID1I…	CTPS1	SFPQ	AMOTL1	CEP70	ALDH7…	SKP2	PSRC1	DRAXIN	NUDT21	PRR11	ACP1
CELLMARKERMSIGDB_HAY_BONE_MARROW_STROMAL	PDGFRB	EGFR	SOD3	SERPI…	GEM	S100A…	IL1R1	COL16…	EYA1	SSPN	GMDS	LGALS…	CYP4X1	COL5A2	LAPTM…	GSN	MATN3	NR2F2	ACSM5	EFS	LARP6	ADIPOQ	CCBE1	JUN	FKBP10	FNDC1	PDLIM4	TNFRS…	TIMP3	ID3
CELLMARKERMSIGDB_HE_LIM_SUN_FETAL_LUNG_C0…	ETV6	PTBP2	MXRA7	IQGAP2	PITX2	SLC4A7	CHD1L	PIM1	KCNMA1	AKAP1	WDR77	PCSK7	TINAG…	CLMP	TMEM1…	CYTH1	APOO	CHCHD7	EDN3	MID1I…	MRPS9	CCDC8…	IL33	INTS13	PTS	STARD…	UBE2H	ESYT2	PHLDA3	HOXA4
CELLMARKERMSIGDB_HE_LIM_SUN_FETAL_LUNG_C0…																														
CELLMARKERMSIGDB_HE_LIM_SUN_FETAL_LUNG_C0…																														
CELLMARKERMSIGDB_HE_LIM_SUN_FETAL_LUNG_C0…	KPNA2	TDRP	TRNP1	TOMM34	PRKCZ	CAVIN2	DIPK1A	SNCAIP	SETD9	TGFB2	GET4	GAL3S…	TP53RK	MAP3K1	B9D1	PKP2	ABCC1	PATJ	DIRAS3	NUDT14	TMEM88	MAGI1	MPPED2	KDM6A	SLC25…	MTHFD…	TES	PDHX	DUSP23	RHOD
CELLMARKERMSIGDB_HE_LIM_SUN_FETAL_LUNG_C0…																														
CELLMARKERMSIGDB_HE_LIM_SUN_FETAL_LUNG_C0…																														
CELLMARKERMSIGDB_HE_LIM_SUN_FETAL_LUNG_C0…	MRGPRF	BNC2	CA11	UBXN6	PDGFC	CD320	RGS10	TMEM88	TMEM1…	MAG	LEPRO…	MN1	PTPRQ	ADGRD1	SSH3	SERPI…	SIRPA	EFNB3	STK26	CSRNP1	ASPHD1	SCARB1	CASP4	ECHDC2	GLIS2	CADPS2	ARHGA…	NAV2	PRR15	SLC7A7
CELLMARKERMSIGDB_HE_LIM_SUN_FETAL_LUNG_C0…	DHX57	NEURL…	H3C2	ASPM	MSRA	KIF20A	PIMREG	OLFML…	TMEM65	ITGB8	ASF1B	H2AC11	CDCA2	KIF18B	NUF2	FANCI	CKAP2	FAM83D	NUDT14	PDXP	ZNF93	H4C12	TRAIP	MYBL2	CDCA3	TMEM1…	METTL…	KIF2C	TMEM1…	ARHGA…
CELLMARKERMSIGDB_HE_LIM_SUN_FETAL_LUNG_C0…																														
CELLMARKERMSIGDB_HE_LIM_SUN_FETAL_LUNG_C0…	WWC2	IGSF9	FSTL3	PARM1	COL11…	MAP3K8	IQCA1	METTL…	P4HA2	PAK4	CA2	NAV2	C3	COL26…	FENDRR	CD320	ADAM33	DDR1	BST1	HDAC7	GSDMD	NPR1	MVP	MEGF6	IFI35	AP1G2	HMCES	PLEC	OBSL1	FLNB
CELLMARKERMSIGDB_HE_LIM_SUN_FETAL_LUNG_C0…																														
CELLMARKERMSIGDB_HE_LIM_SUN_FETAL_LUNG_C1…																														
CELLMARKERMSIGDB_HE_LIM_SUN_FETAL_LUNG_C1…																														
CELLMARKERMSIGDB_HE_LIM_SUN_FETAL_LUNG_C1…																														
CELLMARKERMSIGDB_HE_LIM_SUN_FETAL_LUNG_C1…	PSME4	PKN3	MVK	EEF2K…	KIF18A	MCM10	JAM3	MAP1B	RRM2	FAM83D	GLCE	ATAD5	ANLN	SLC5A3	SANBR	FUOM	NCAPG2	TENT5B	SYNJ2	SKA1	SUV39…	ALDH1…	SYT11	LRR1	FGFR1	CD200	ATAD3A	ARG2	TSPAN5	SLC12…
CELLMARKERMSIGDB_HE_LIM_SUN_FETAL_LUNG_C1…	PPP6R1	PNMA2	GAL3S…	BEX5	ISL1	FGF14	MAP4K2	KIF19	DNAJC…	CDCP1	DLL4	MARK1	ZFTA	LYSMD2	DDN-A…	RAB3B	TAGLN3	PDE8B	ZBTB18	NSMF	CDKN2D	PROC	TMED8	NLRP1	EXTL3	RAPGE…	ANKH	GPC6	SPTSSB	SPECC1
CELLMARKERMSIGDB_HE_LIM_SUN_FETAL_LUNG_C1…																														
CELLMARKERMSIGDB_HE_LIM_SUN_FETAL_LUNG_C1…																														
CELLMARKERMSIGDB_HE_LIM_SUN_FETAL_LUNG_C1…																														
CELLMARKERMSIGDB_HE_LIM_SUN_FETAL_LUNG_C1…	NUPR1	PLLP	HEY1	SOX21	SYTL2	MAP1B	FAM10…	FXYD5	ARHGD…	RGS10	ARHGA…	UPK2	TFAP2A	GLIS3	CNGA1	NRXN3	MB	FYB2	ERN2	DOCK5	GABRP	CCDC80	POF1B	NTM	JAKMI…	CA10	TSPAN8	WNT5A	VIPR1	TFPI
CELLMARKERMSIGDB_HE_LIM_SUN_FETAL_LUNG_C1…	DENND…	LMBRD2	DACH1	CEP126	NEURO…	INHBB	CFC1	PPP1R…	GCNT1	GAB2	CADPS	KCNJ6	RINL	CFAP65	PKIB	FREM1	SPECC1	TMPRS…	ST8SI…	CACNB2	GRIA2	CDKN2D	KCNIP3	RIMS2	SERP2	BCL2	BMERB1	CNIH2	SOX21	TRMT9B
CELLMARKERMSIGDB_HE_LIM_SUN_FETAL_LUNG_C1…	ITGB6	PMAIP1	PDZRN3	AK4	GNG2	CCDC2…	CX3CL1	ODF3B	GNG12	NID1	PROM1	GCLC	MCAM	ADM	IGFBP3	PAQR4	VWA2	LEF1	FAM21…	DCLK1	TRPV4	COL16…	ETV4	FMNL3	ADAMT…	LAYN	COL1A2	QPCT	SLC1A1	IL17RD
CELLMARKERMSIGDB_HE_LIM_SUN_FETAL_LUNG_C1…	USP43	BIK	BICDL2	NR2F2…	TMEM1…	PINK1	NPHP3	C8orf…	TNFSF…	MUC20	PPP1R…	EPS8L1	ATL3	CDKN1A	LRATD1	KHDRB…	TMEM1…	THBS3	RNF14…	SLC43…	NTN1	COMTD1	RRP36	LPGAT1	ST3GA…	NSMF	TMCO4	NPEPL1	FAN1	TACC2
CELLMARKERMSIGDB_HE_LIM_SUN_FETAL_LUNG_C2…	CENPB	DHRS7B	PDE6G	OAS2	PALLD	BSCL2	SULT1…	DENND…	SASH1	RALGDS	GALM	GPX3	SNX24	CDK18	SLC26…	CEACA…	PANK3	CD72	TWF1	CADM1	FXYD6	WDR81	CYSTM1	ADAM9	HS1BP3	C17or…	FLVCR2	DENND…	RMDN3	PGM3
CELLMARKERMSIGDB_HE_LIM_SUN_FETAL_LUNG_C2…	SLC25…	SLC25…	MVB12B	ERICH5	TRIP6	CSKMT	SLC9A7	NT5C3B	ATP6V…	SLC24…	CLECL…	GPT2	PPY	PAQR8	RAB11…	RFPL4…	MCM7	NID1	H1-5	ISYNA1	CNFN	ECHDC2	PTTG1	DUSP18	BTN2A2	MIR18…	HLA-D…	SH3RF2	RFPL4A	STX3
CELLMARKERMSIGDB_HE_LIM_SUN_FETAL_LUNG_C2…																														
CELLMARKERMSIGDB_HE_LIM_SUN_FETAL_LUNG_C2…																														
CELLMARKERMSIGDB_HE_LIM_SUN_FETAL_LUNG_C2…																														
CELLMARKERMSIGDB_HE_LIM_SUN_FETAL_LUNG_C2…	H2BC7	SMYD3	PTGDR2	CLNK	MED17	ZNF738	IFT57	LEF1	TRH	NDUFA…	SCAI	DNMT3B	CEP70	BEND5	MTMR6	ERMP1	MED21	MFAP2	ZNF711	SUPT3H	KIT	MEIS1	HOXB4	SNHG21	HPGD	EIF3J…	SLC17…	KRBOX4	CLDN15	CD79B
CELLMARKERMSIGDB_HE_LIM_SUN_FETAL_LUNG_C2…	MICU1	MCU	ARHGE…	SLC50…	INF2	TSC22…	LRRC8B	GP5	RGS6	EGF	ELOVL7	ACAD8	TSPAN9	STRN4	KCTD20	RHAG	KIAA0…	PEAR1	IGF2B…	STON2	H3C10	ITGA6	ABHD4	PGRMC1	VPS37B	UXS1	ROCK2	HGD	B3GNT2	RTN2
CELLMARKERMSIGDB_HE_LIM_SUN_FETAL_LUNG_C2…																														
CELLMARKERMSIGDB_HE_LIM_SUN_FETAL_LUNG_C2…	PRKAR…	WASL	STX17	CBLB	TFR2	RHAG	RYBP	PLAG1	CAV2	ANKRD…	KBTBD2	PTDSS2	UBL4A	THRA	NT5M	IKZF5	HEMGN	MBTD1	ACAD8	ACSM3	IKZF4	ZNF140	ZNF714	ZNF51…	GFI1B	TRAF6	ANKRA2	B4GAL…	SSBP2	TRIM13
CELLMARKERMSIGDB_HE_LIM_SUN_FETAL_LUNG_C2…	RPL39L	POLR3K	MCM7	COQ5	LTK	SHQ1	LTV1	RASSF7	BRI3BP	AAR2	AREG	PPP3CC	C11or…	IMMP1L	TMEM1…	GPR146	YEATS4	EPHB1	PIDD1	NPM3	PCCB	CD2AP	PRPS1	GMPPB	GLRX5	EOLA2	SMN1	XXYLT1	CEP78	SLC2A1
CELLMARKERMSIGDB_HE_LIM_SUN_FETAL_LUNG_C2…																														
CELLMARKERMSIGDB_HE_LIM_SUN_FETAL_LUNG_C2…																														
CELLMARKERMSIGDB_HE_LIM_SUN_FETAL_LUNG_C2…																														
CELLMARKERMSIGDB_HE_LIM_SUN_FETAL_LUNG_C3…																														
CELLMARKERMSIGDB_HE_LIM_SUN_FETAL_LUNG_C3…	SLC25…	GCDH	BRCA2	AQP3	C2orf…	PCNA	SPAG5	ORC6	HES6	XK	FN3K	DSN1	ANKLE1	PRKAR…	APEH	CMPK2	SLC22…	NSD2	RABGA…	SLC37…	RFC4	MIGA2	ABCA7	UHRF1	ASF1B	H2AC12	MCM5	YEATS4	H2AC17	MST1
CELLMARKERMSIGDB_HE_LIM_SUN_FETAL_LUNG_C3…	EGFLAM	PPP1R…	ZNF622	OIT3	SNAPC1	HIPK2	QPCT	XRCC1	HRAS	ASB9	UBE2C	PCDH1	HSD17…	NSMCE2	AIMP2	LYAR	IER3	NAA15	C20or…	LDLRA…	TBRG4	GNL2	GPATC…	RNASE…	FAM21…	YARS2	MTFP1	FAM13…	HILPDA	CDCA7L
CELLMARKERMSIGDB_HE_LIM_SUN_FETAL_LUNG_C3…	SLC22…	CCDC1…	CTDSPL	C17or…	SYNM	MATN2	GNB5	CYB56…	FHL2	TANC2	TPBG	GALNT2	LRP11	DNASE2	HES4	NR2F1…	PTGS1	TAC3	TGFA	OSBPL…	SCN3A	RHOD	PLIN5	ACSL5	OXCT1	CELSR1	HOXA7	CRTAC1	EBF1	TLR4
CELLMARKERMSIGDB_HE_LIM_SUN_FETAL_LUNG_C3…	WIPF1	CAPG	INPP4B	ARRB2	GPRIN3	C20or…	NR2F2…	NKAIN4	DNASE2	SEMA3D	SLC35…	RSPH4A	DTX4	PKHD1…	PRKG1	SLC43…	P2RX4	DOC2B	LGMN	KALRN	APOD	MYO7A	FRMD6	SGK3	SPRY1	RPP25	TRMT9B	SNCAIP	SLC7A7	COL4A5
CELLMARKERMSIGDB_HE_LIM_SUN_FETAL_LUNG_C3…																														
CELLMARKERMSIGDB_HE_LIM_SUN_FETAL_LUNG_C3…	CRIP1	P4HTM	SLC16…	CASP1	TNRC6C	DSE	ABCA8	CBX6	APLP1	ADAMT…	ATP2A3	HOXA3	PLXNA4	HTRA3	FNIP2	ANKRD…	FMOD	NPIPA1	ROM1	PTGDS	SYT15	APOL1	PI15	CSF1	AMPH	LDLRA…	SLC25…	PKP2	USP35	PTGER4
CELLMARKERMSIGDB_HE_LIM_SUN_FETAL_LUNG_C3…	BIK	OSBPL…	IL1R1	IFI6	LAMA5	ANKH	ZDHHC…	SLC7A1	PSEN2	TUBB3	BEND5	SYNM	FOXO1	LY96	RAB6B	ASPH	PITPN…	ANO10	BBIP1	BHMT2	FBXO17	C16or…	ADAMT…	NBL1	PPP3CC	TRIM47	NEXN	SUN2	ARID5A	ZNF195
CELLMARKERMSIGDB_HE_LIM_SUN_FETAL_LUNG_C3…																														
CELLMARKERMSIGDB_HE_LIM_SUN_FETAL_LUNG_C4…																														
CELLMARKERMSIGDB_HE_LIM_SUN_FETAL_LUNG_C4…																														
CELLMARKERMSIGDB_HE_LIM_SUN_FETAL_LUNG_C4…																														
CELLMARKERMSIGDB_HE_LIM_SUN_FETAL_LUNG_C4…																														
CELLMARKERMSIGDB_HE_LIM_SUN_FETAL_LUNG_C4…	CCNB1	STIL	BRCA2	BCL2L…	PIDD1	BTG3	NEIL3	SHCBP1	H2BC15	DTL	TRNAU…	HAUS1	ACTL6A	PIMREG	TIMM10	NCAPD3	TRIM28	MCMBP	XRCC1	H2BC11	RAD54L	PPP5C	AURKA	MZT1	PBK	BRCA1	MPST	DDB2	CENPO	TSPAN4
CELLMARKERMSIGDB_HE_LIM_SUN_FETAL_LUNG_C4…	SPDL1	KATNB…	H2AC20	DBF4	RCAN3	PARPBP	PSMD14	TRIP13	PRPS1	MCM6	BRD8	CIP2A	SIRPG	HDGF	PRIM2	BAG3	BRCA1	PAFAH…	MAP4K4	HPGD	BLM	STAT1	CDC25C	PRIM1	SUZ12	MAD2L2	N4BP2	GINS2	HCFC1…	SKA3
CELLMARKERMSIGDB_HE_LIM_SUN_FETAL_LUNG_C4…																														

Table S5: Cell state marker gene list

CELLMARKERCAN_ACINAR_CELLS																														
CELLMARKERMSIGDB_HE_LIM_SUN_FETAL_LUNG_C4…																														
CELLMARKERMSIGDB_HE_LIM_SUN_FETAL_LUNG_C4…																														
CELLMARKERMSIGDB_HE_LIM_SUN_FETAL_LUNG_C4…																														
CELLMARKERMSIGDB_HE_LIM_SUN_FETAL_LUNG_C5…																														
CELLMARKERMSIGDB_HE_LIM_SUN_FETAL_LUNG_C5…	ATP1A1	ARPC4	C17or…	COMMD1	BRIX1	RPP25	WDR43	NUDT4	ARMCX6	HSD17…	SUMF2	RGCC	CASP1	CDC42…	INSIG1	BCAR3	FMNL3	GSTZ1	SRM	CHMP7	STAP1	GNPTAB	SLAMF7	ZNF593	ARF3	LCMT1	CASP3	DIPK1A	CMSS1	ERG28
CELLMARKERMSIGDB_HE_LIM_SUN_FETAL_LUNG_C5…																														
CELLMARKERMSIGDB_HE_LIM_SUN_FETAL_LUNG_C5…																														
CELLMARKERMSIGDB_HE_LIM_SUN_FETAL_LUNG_C5…	BRCA2	POC1A	H2AC8	CIP2A	ABHD3	KIF20A	CD34	ARHGA…	ZNF367	ACAT2	NCAPH2	IGFBP7	NEIL3	FAF1	ARMH1	GAMT	MIS18A	SHCBP1	INCENP	CEP55	DEPDC…	CSE1L	UBR7	DDB2	STMN3	HMMR	CCP110	PHF19	ZMIZ1	NIPSN…
CELLMARKERMSIGDB_HE_LIM_SUN_FETAL_LUNG_C6…																														
CELLMARKERMSIGDB_HE_LIM_SUN_FETAL_LUNG_C7…																														
CELLMARKERMSIGDB_HE_LIM_SUN_FETAL_LUNG_C7…																														
CELLMARKERMSIGDB_HE_LIM_SUN_FETAL_LUNG_C7…																														
CELLMARKERMSIGDB_HE_LIM_SUN_FETAL_LUNG_C7…																														
CELLMARKERMSIGDB_HE_LIM_SUN_FETAL_LUNG_C7…	TRPV2	NAPG	RAB15	HTR7	RIMBP2	SAMD14	CIB2	VWA5A	TMEM2…	PGAM2	GRIA3	MANEAL	EFNB3	SMPD3	VSTM2L	RCAN2	MARK1	FAM11…	RIMS2	RIC3	CXCR4	SH2B2	PLD5	SYBU	KLHDC9	MYT1L	NCDN	HTR3A	KCNK10	SYN3
CELLMARKERMSIGDB_HE_LIM_SUN_FETAL_LUNG_C7…																														
CELLMARKERMSIGDB_HU_FETAL_RETINA_AMACRINE																														
CELLMARKERMSIGDB_HU_FETAL_RETINA_BIPOLAR																														
CELLMARKERMSIGDB_HU_FETAL_RETINA_FIBROBLA…	WWTR1	BMP1	PRSS23	HIC1	COL4A5	EPHA7	FHL3	TSPYL2	CAST	SNORD…	GADD4…	DOCK1	SMARC…	FXYD5	RHOJ	TPBG	RARG	C1R	FRMD4A	EFNB1	LAMC3	STING1	TPM4	HLA-E	TNS2	TMEM2…	ALX4	AHNAK	EML4	ESAM
CELLMARKERMSIGDB_HU_FETAL_RETINA_HORIZONT…																														
CELLMARKERMSIGDB_HU_FETAL_RETINA_MICROGLIA	SYNGR2	GLRX	FTLP2	TMSB4…	OSTF1	CPVL	DUSP1	ST6GA…	CNPY3	MSN	IL6ST	HLA-D…	DOCK10	ARHGA…	TGFBR2	SWAP70	MIS18…	STK10	PPP1R…	PIP4K…	ETS2	MFSD1	CCR1	PLSCR1	GPX1	LAP3	PALD1	DOCK4	MGAT4A	PARP14
CELLMARKERMSIGDB_HU_FETAL_RETINA_MULLER																														
CELLMARKERMSIGDB_HU_FETAL_RETINA_PHOTOREC…																														
CELLMARKERMSIGDB_HU_FETAL_RETINA_RGC	UBE2D1	KIF3C	WDR47	ATL1	PGK1	MAPRE2	SCD	FAM24…	EPHA5	DYNC1…	MAP6	LDHA	FKBP3	EPHB1	NCAN	SMAD9	YWHAQ	PKIA	PDE6D	TENM3	TXNDC…	SMS	GABBR2	ACTR10	TTC9B	TUBA1A	CYCS	TMEFF2	NPDC1	PFKFB3
CELLMARKERMSIGDB_LAKE_ADULT_KIDNEY_C1_EPI…																														
CELLMARKERMSIGDB_LAKE_ADULT_KIDNEY_C10_TH…	DEK	TPST1	RPS14	SARAF	RPL10A	YBX3	C6orf…	GCC2	HIF1A	ITGA2	UQCRB	RPS13	RPS7	RPS8	PSMB1	HSPH1	EIF4A2	CAPN2	CNN3	CNBP	RPS17	SRSF3	NORAD	KIF5B	BACE2	PRKD1	CXXC5	RPL31	CNDP2	ACTR2
CELLMARKERMSIGDB_LAKE_ADULT_KIDNEY_C12_TH…	RPL27A	CPT1A	SUCLG1	RPL5	RPL34	CRIP2	ITGB5	GPC5	SOD1	MYO3B	HIBADH	MGAT5	RPS4X	FTH1	RPS3	EFHD1	MDH1	TRIM2	PFDN5	CHCHD2	CPVL	AK3	SELEN…	RPS6	CLDN1…	RPS27	RPS7	NRCAM	PPIA	RPL30
CELLMARKERMSIGDB_LAKE_ADULT_KIDNEY_C18_CO…	RPS27	SERF2	RPL14	RPLP2	PTPN13	KIF5B	LAPTM…	UBA52	RPL35	DSP	ACTR2	TMEM59	XRCC5	RPS16	ENO1	ATP6V…	PABPC1	EIF4G2	NDUFA4	PKM	RPL24	SKIL	PGK1	RPL21	RANBP2	TAX1B…	CA12	PLCL2	PTMA	RPS7
CELLMARKERMSIGDB_LAKE_ADULT_KIDNEY_C19_CO…	RPL9	NAMPT	RPS3	RPL23	COX7C	PTTG1…	RPS15	SLC25…	BTF3	RANBP2	GAS5	CCT6A	CANX	XRCC5	HNRNPR	KIF5B	IDH2	DDX5	RPL14	MTATP…	RBBP8	ALDOA	TAF7	IGFBP7	PEBP1	TMEM1…	MT2A	RPLP2	RPL35	PRKAR…
CELLMARKERMSIGDB_LAKE_ADULT_KIDNEY_C25_EN…																														
CELLMARKERMSIGDB_LAKE_ADULT_KIDNEY_C28_IN…																														
CELLMARKERMSIGDB_LAKE_ADULT_KIDNEY_C29_UN…																														
CELLMARKERMSIGDB_LAKE_ADULT_KIDNEY_C5_PRO…	TPCN1	ZNF80…	NHS	PPM1L	DPYS	TRABD…	UPF2	SHANK2	CDR2	LINC0…	PIGL	UBN1	ZMAT1	TNRC6A	RBM6	CMBL	SLC4A4	UPF3B	CSPP1	MAP3K…	ALDOB	DZIP1	SLIT3	SLC13…	POR	SRRT	GADD4…	RAPGE…	LIN52	GTF2I…
CELLMARKERMSIGDB_MANNO_MIDBRAIN_NEUROTYPE…																														
CELLMARKERMSIGDB_MANNO_MIDBRAIN_NEUROTYPE…	KCTD8	CEP126	SHTN1	SHISA…	KIF21B	KIF3A	KIF5C	KIFC2	KLF7	KLHL1	KLHL14	KLHL29	KLHL32	KLHL35	L1CAM	LAMC2	LDB2	LHFPL4	LMTK3	LMX1A	DRAIC	LPCAT4	PLPPR2	PLPPR4	LRRC3B	LRRC55	LRRC7	LSAMP	LY6H	LYST
CELLMARKERMSIGDB_MANNO_MIDBRAIN_NEUROTYPE…	HBA2	HCN3	HIVEP3	HMGCL…	NSG2	HMX2	HOOK1	HPCAL4	HS3ST4	HSPA1…	IGDCC4	IGF1	INA	INSM1	IQCJ-…	IRGQ	JAKMI…	JAKMI…	JPH3	JPH4	JUP	KALRN	KCNAB1	KCND2	KCNIP4	KCNJ6	KCNMA1	KCNN3	KCNQ3	KCTD7
CELLMARKERMSIGDB_MANNO_MIDBRAIN_NEUROTYPE…	KCNJ6	KCNN3	KCTD4	CEP126	KIAA1…	SHTN1	SHISA…	KIF21B	KIFAP3	KIFC2	KLC1	KLHL1	KLHL13	KLHL29	KLHL32	KRT17	KRT222	KRT5	L1CAM	LGI1	LGI2	LHFPL4	LMO3	LMO4	DRAIC	LONRF2	PLPPR2	PLPPR4	LRFN5	LRRC3…
CELLMARKERMSIGDB_MANNO_MIDBRAIN_NEUROTYPE…	ETS1	ETS2	EVA1B	F11R	F2RL1	FABP4	FAM12…	NIBAN1	ENTRE…	PRXL2A	FAM43A	FAM78A	FAM89A	FAR2	FBLN2	FCGRT	FCHO2	FES	FGD5	FGR	FHL3	FKBP1A	FKBP5	FLI1	FLNB	FLT1	FLT4	FLVCR2	FMNL3	FN1
CELLMARKERMSIGDB_MANNO_MIDBRAIN_NEUROTYPE…	HBA1	HBA2	HBG1	HBG2	HCLS1	HCST	HERPU…	HHEX	ARHGA…	HMOX1	HPGDS	HPSE	HRH1	HS3ST1	HSD17…	HSPA6	HSPA7	HSPB1	HSPD1	HSPH1	HTRA1	ICA1	ICAM1	IER2	IER5	IFI16	IFNGR1	IGF1	IGFBP3	IGSF6
CELLMARKERMSIGDB_MANNO_MIDBRAIN_NEUROTYPE…	GABBR2	GABPB…	GABRA1	GABRB1	GABRB2	GABRB3	GABRG1	GABRG2	GAD2	GAP43	GATA3	GDPD1	GFOD1	GK5	GLRA2	GLS	GNAL	GNAO1	GNG2	GNPTAB	GOLGA…	GOLGA…	GOLGA…	GP1BB	GPM6A	GPR12	GPR13…	GPR155	GPR176	GPRAS…
CELLMARKERMSIGDB_MANNO_MIDBRAIN_NEUROTYPE…	RAP1G…	RAPGE…	RASGE…	RBFOX2	RHOU	RNF112	RNF165	RNF175	ROBO1	ROBO2	RPL13…	RPL23A	RPS19	RPS6K…	RTN1	RUFY3	RUNDC…	RUSC1	SAMD5	SBK1	SCAMP5	SCG3	SCG5	SCN3A	SCN8A	SCUBE1	SEMA3A	SEMA3E	SEZ6L	SEZ6L2
CELLMARKERMSIGDB_MANNO_MIDBRAIN_NEUROTYPE…	MAP2	MAP3K9	MAP6	MAP7D2	MAPK8	MAPRE3	MARK1	MAST1	MCF2	MDGA1	MDGA2	MED12L	MEG3	MFSD6	MIAT	MICU3	MIR12…	MLLT11	MMP24	MSANT…	MTUS2	MUC19	MVB12B	MYT1	MYT1L	NALCN	NAP1L3	NAV1	NBEA	NCAM1
CELLMARKERMSIGDB_MANNO_MIDBRAIN_NEUROTYPE…	NETO1	NFASC	NIPA1	NLN	NMNAT2	NRP2	NSG1	NTRK1	NYAP1	OCIAD2	OGDHL	OLFM1	OSBPL6	P2RX3	PAFAH…	PCBP4	PCDH7	PCDH9	PCDHA7	PDE1B	PDP1	PEX5L	PFKM	PHACT…	PHF21B	PHOX2A	PHOX2B	PIANP	PID1	PLCB1
CELLMARKERMSIGDB_MANNO_MIDBRAIN_NEUROTYPE…	NLGN2	NLGN3	NLGN4Y	NPAS3	NRXN1	NTM	NTRK3	NXPH1	OLIG1	OLIG2	OMG	OPCML	OPHN1	OSBPL6	PBK	PCDH1…	PCDH15	PCDH17	PCDHG…	PCDHA7	PCDHG…	PDE1C	PDGFRA	PEA15	PHACT…	PHGDH	PHLDA1	PHYHI…	PID1	PIF1
CELLMARKERMSIGDB_MANNO_MIDBRAIN_NEUROTYPE…	NIBAN1	FAM13A	FAM16…	SERF1A	FAM43B	TENT5A	FAR2	FARP1	FBN1	FCGRT	FCHSD1	FERMT2	FES	FGD5	FHL5	FILIP…	FKBP1A	FKBP5	FLNA	FMNL3	FMO1	FN1	FNDC3B	FOS	FOSB	FOXC1	FOXD1	FOXF2	FOXL1	FOXQ1
CELLMARKERMSIGDB_MANNO_MIDBRAIN_NEUROTYPE…	RFX4	RNASE…	RNF13	RPA3	RPL13…	RPL18	RPL23A	RPS14	RPS19	RRM2	RTKN2	SALL1	SALL4	SAPCD2	SCUBE1	SFRP1	SFRP2	SGO1	SGO2	SHISA2	SHISA3	SHROO…	SKA3	SLC39…	SLC6A8	SMC4	SNRPF	SOX1	SOX2	SOX9
CELLMARKERMSIGDB_MANNO_MIDBRAIN_NEUROTYPE…	PRTG	PSRC1	PTGR1	PTPRK	PTPRO	PTTG1	PTX3	NECTI…	RAD51…	RAI14	RANBP1	RBM24	RCN1	RCOR2	REST	RFC3	RFX4	RNASE…	RPA3	RPL13…	RPL23A	RPS14	RPS19	RRM2	RTKN2	SALL1	SALL4	SDC1	SDC2	SFRP2
CELLMARKERMSIGDB_MANNO_MIDBRAIN_NEUROTYPE…	NCAPG2	NCAPH	NEK2	NES	NPM1	NPY	NR2F6	NUF2	NUSAP1	NXPH2	OTX2	OTX2-…	PAICS	PARPBP	PBK	PDGFC	PDPN	PHGDH	PIEZO2	PIF1	PIH1D1	PKIB	PLEKH…	PLIN2	PLIN3	PLK1	PMAIP1	POU3F1	PRC1	PRDX2
CELLMARKERMSIGDB_MANNO_MIDBRAIN_NEUROTYPE…	PTX3	NECTI…	RAB13	RAI14	RALGP…	RBM24	RBMS1	RBP1	RCN1	RCOR2	RERG	RFC3	RFX4	RIPPL…	RNASE…	RPA3	RPGR	RPL13…	RPL18	RPL23A	RPS14	RPS19	RSPH4A	RSPH9	RSPO3	SALL1	SALL4	SCN7A	SDC2	SDC4
CELLMARKERMSIGDB_MANNO_MIDBRAIN_NEUROTYPE…	NRBP2	NTS	NUAK2	NUDT4	NXPH1	OPHN1	OTX2	PARD3B	PBXIP1	PDE11A	PDE1A	PDE3A	PDGFC	PDLIM3	PDLIM5	PDPN	PEA15	PIFO	PIK3I…	PLEKH…	PLIN3	PLOD2	PLP1	PLSCR4	PLTP	PMP22	PON2	PPIL6	PREX1	PRTG
CELLMARKERMSIGDB_MANNO_MIDBRAIN_NEUROTYPE…	ADGRG1	GPRC5B	GRIA1	GRID2	GRM3	RAB27A	GULP1	HAPLN3	HAS2	HBA1	HBA2	HBG1	HBG2	HDAC4	HEATR…	HEPAC…	HEPN1	HES1	HES4	HES5	H1-2	H2AC6	H2BC12	HMGCS1	RIDA	HSDL2	HSPA6	HSPA7	HSPB1	HSPB8
CELLMARKERMSIGDB_MANNO_MIDBRAIN_NEUROTYPE…	CNMD	LFNG	LIFR	LIMCH1	LIPG	LITAF	LIX1	LRIG1	LRP1	LRP2	LRRC17	LRRC3…	LRRC4	LRRC4C	LRRN1	LTBP1	LUZP2	LYPD1	MAML2	MASP1	MCC	FAT2	METRN	METTL…	MFGE8	MGST1	IGSF9B	MKI67	MLC1	MMD2
CELLMARKERMSIGDB_MANNO_MIDBRAIN_NEUROTYPE…	PCDH17	PCDH7	PCDHG…	PDE1C	PDE7B	PDLIM3	PDPN	PEA15	PHGDH	PI15	PIEZO2	PIK3CB	PKMYT1	PLAT	PLEKH…	PLLP	PLTP	PMP2	PMP22	PNOC	PON2	PLPP3	PREX1	PRKCA	PSAT1	PTCH1	PTN	PTPRE	PTPRT	PTPRZ1
CELLMARKERMSIGDB_MANNO_MIDBRAIN_NEUROTYPE…	ADGRV1	GPRC5C	GRIA1	GRIN2A	HAPLN1	HAS2	HBA1	HBA2	HBG1	HBG2	HDAC4	HEATR…	HEPAC…	HEPN1	HES1	HES4	HES5	HEY1	H2AC6	HK2	HNRNP…	RIDA	HS3ST1	HS3ST4	HSDL2	HSPB1	HTRA1	HYDIN	ID3	ID4
CELLMARKERMSIGDB_MANNO_MIDBRAIN_NEUROTYPE…	PHYHI…	PITX2	PLEKH…	PLXNA3	POU2F2	POU3F1	POU4F1	PPFIA2	PPP3R1	PRICK…	PRKAR…	PRSS12	PTPN5	PTPRO	RAB3A	RAB3C	RAB3IP	RAB9B	RALYL	RAPGE…	RBFOX1	RBFOX2	RBM18	RELN	RIMS4	RND3	RNF112	RNF182	ROBO2	ROBO3
CELLMARKERMSIGDB_MANNO_MIDBRAIN_NEUROTYPE…	LRP1B	LY6H	MAOA	MAP1B	MAP1L…	MAP2K4	MAP7D2	MAPK8…	MAPRE2	MAPRE3	MARCH…	MAST1	MCF2	MCF2L	MEG3	MFSD6	MGAT4C	MICOS…	MLLT11	MMP24	MSANT…	MTURN	MTUS2	MYCBP2	MYO5A	NALCN	NAP1L3	NAP1L5	NAPB	NCAM1
CELLMARKERMSIGDB_MENON_FETAL_KIDNEY_0_CAP…																														
CELLMARKERMSIGDB_MENON_FETAL_KIDNEY_10_IM…																														
CELLMARKERMSIGDB_MENON_FETAL_KIDNEY_2_NEP…																														
CELLMARKERMSIGDB_MENON_FETAL_KIDNEY_3_STR…																														
CELLMARKERMSIGDB_MENON_FETAL_KIDNEY_6_COL…																														
CELLMARKERMSIGDB_MURARO_PANCREAS_ACINAR_C…	CASP4	FGA	FTH1P3	SEL1L	TPD52…	SOX9	HLA-D…	PMAIP1	MLPH	NCOA7	PIGR	RAB11…	TCIRG1	GALNT2	CCL8	LYPD1	RASGR…	TMBIM1	RHOC	DUSP23	SERPI…	TNFAI…	ANGPT…	RSU1	ITPRI…	ALDH2	RPL10L	TUBB3	CLMN	GALNT3
CELLMARKERMSIGDB_MURARO_PANCREAS_ALPHA_CE…	SCG3	IFI6	CTNND2	COX17	ATP5F…	LSAMP	CPNE8	TCTA	USP25	CRELD1	CBX6	RADX	P4HA2	MALAT1	CYSTM1	NDUFA7	G6PC2	PLP2	SCAMP1	MAGI2…	ANXA7	RGS9	GPAA1	NOVA1	EDIL3	H1-2	TENT5A	FAM47E	SLC37…	KIAA0…
CELLMARKERMSIGDB_MURARO_PANCREAS_BETA_CELL	MCUB	NGRN	RNF6	PIPOX	TNKS2	C1orf…	NCKAP1	LINC0…	GNAO1	MAN1A2	MYO3A	TMEM64	KCNK16	SVIP	TSHZ1	LRRTM3	PJA2	SYBU	TMCC3	LONRF2	UBL3	CDK5R1	RGS16	NBEA	CACNA…	MAPRE2	TSHZ3	MYCBP2	GPM6A	SLC18…
CELLMARKERMSIGDB_MURARO_PANCREAS_ENDOTHEL…	SRGN	TBC1D1	HK1	ITGA2	DIPK2B	RELL1	GARRE1	VIM	PLXNA2	JAK1	GAB1	AHR	UXS1	ITPRI…	REST	LDHB	PTPRE	ID1	S100A…	CAV1	LMO2	NOL4L	SLC44…	ICAM1	CCND1	LATS2	ANKRD…	SLC2A3	MCF2L	JUP
CELLMARKERMSIGDB_MURARO_PANCREAS_EPSILON_…																														
CELLMARKERMSIGDB_MURARO_PANCREAS_MESENCHY…	RND3	FIBIN	CMTM3	ADAMT…	RGS10	HCLS1	EHD2	SH3PX…	RFLNB	ITGB5	FERMT2	AHNAK	CRTAP	C3orf…	CEMIP	DAB2	PMP22	TPBG	ENC1	CCDC7…	FBXO32	STON1	PPFIB…	TMEM47	SH3PX…	LIMS1	STK17B	IFITM3	MTHFD2	ANGPT…
CELLMARKERMSIGDB_RUBENSTEIN_SKELETAL_MUSC…	RPS3A	SELEN…	UQCRH	RPL6	RPL11	PCBP2	GDI2	SERF2	RPL41	CTSB	RPL37	CITED2	ID2	RPL4	VMP1	LRRFI…	EIF3K	ATP5M…	RPS14	YBX1	RPS23	HSD17…	ARPC5	ATP6V…	RPL30	HLA-D…	CAPZB	RPL21	RPL32	JUND
CELLMARKERMSIGDB_RUBENSTEIN_SKELETAL_MUSC…	DARS1	COX7A…	JUND	EIF3F	BANF1	NDUFB9	VIM	ATP5MG	SNRPD1	PRDX6	HSPA8	VAPA	HNRNP…	HSPE1	HDAC2	PABPC1	UQCRB	HMGN1	ATP5M…	UBXN1	RPL7L1	CCT8	APRT	ATP5F…	VDAC2	VAMP2	TTC3	FUNDC2	UBA52	PSMB7
CELLMARKERMSIGDB_RUBENSTEIN_SKELETAL_MUSC…	MT-ND4	NDUFS5	ATP5F…	NIPSN…	SDHD	HIGD2A	UQCRF…	COX6B1	UQCRC1	RPL14	MRPS36	RPLP1	SLIRP	MAP3K…	COX4I1	OPTN	RBBP7	NDUFV2	MT-AT…	NDUFA1	ANAPC…	CNBP	PLP2	ALDOA	SELEN…	ANP32B	PGAM1	SKP1	ANXA6	CRIP2
CELLMARKERMSIGDB_TRAVAGLINI_LUNG_ALVEOLAR…	TM4SF1	ITPRIP	UBC	RAB31	RHOF	PMP22	CELF2	RASAL2	TBC1D2	PTPN1	N4BP1	SGCE	TMEM1…	STX11	HACD1	SFN	SERIN…	HSPB1	WWC2	SCD5	PDZD2	TXNDC…	LAPTM…	SMAD7	PNPLA2	SPTAN1	PLCE1	EPS8L2	B3GNT7	CLDN4
CELLMARKERMSIGDB_TRAVAGLINI_LUNG_BASOPHIL…	LINC0…	BZW1	LRP4	CD82	HS6ST1	C21or…	PRKX	LRRC70	PLK3	ATP6V…	TGIF1	IL18R1	PDCD4	TSEN54	BEX4	ANKRD…	SFT2D1	ARHGA…	SCIN	TESPA1	CLEC4…	COL13…	EGR1	PIK3R6	LXN	CRLF2	ANXA4	MAST4	TMEM2…	SNX5
CELLMARKERMSIGDB_TRAVAGLINI_LUNG_CAPILLAR…																														
CELLMARKERMSIGDB_TRAVAGLINI_LUNG_CAPILLAR…	RNF115	SYNPO	CCND3	PRX	HSPA1…	RPLP2	WBP11	POMP	PAK2	MAFF	NSRP1	GOLGA4	LARP7	SEC14…	RPS4Y1	CDC26	BTN3A3	TPM4	C7orf…	PTMS	WDR45B	TSR2	ANAPC…	PSIP1	TPM1	EPB41…	ARHGE…	TMEM1…	LHFPL6	H2AZ2
CELLMARKERMSIGDB_TRAVAGLINI_LUNG_CD4_MEMO…																														
CELLMARKERMSIGDB_TRAVAGLINI_LUNG_EREG_DEN…	SH3BP5	SLC7A5	MARCKS	FPR2	LHFPL2	HSBP1	TNFAI…	PRMT9	CD36	ACTG1	SPRED1	ASPH	PELAT…	FURIN	CEP170	NR4A2	GLIPR2	DUSP1	SIRPA	PTAFR	MFSD1	FTL	TPT1	ICAM1	RAB20	KYNU	PLXDC2	BACH1	KDM6B	NINJ1
CELLMARKERMSIGDB_TRAVAGLINI_LUNG_IGSF21_D…																														
CELLMARKERMSIGDB_TRAVAGLINI_LUNG_MESOTHEL…	IGFL2	MFAP2	EFNB2	POLD4	TRAPP…	CNIH4	MRPL41	NDUFA7	FKBP11	HPCAL1	UAP1	TMSB4X	LY6E	POLR2I	AP1S1	ARF6	SLPI	RPL35	MRPL23	PTGS1	EVA1C	RPS29	WARS1	ATP2B1	CLNS1A	ANP32B	NMI	CD74	PTRHD1	ODC1
CELLMARKERMSIGDB_TRAVAGLINI_LUNG_MYOFIBRO…																														
CELLMARKERMSIGDB_TRAVAGLINI_LUNG_NATURAL_…																														
CELLMARKERMSIGDB_TRAVAGLINI_LUNG_NEUTROPH…	IFITM3	DDX17	POM121	ADAM8	NCF1	CORO1A	JUND	C4orf3	PREX1	CXCL8	SMCHD1	CTSD	NAMPT…	RNA5S…	RPS19…	TMCC3	EIF4E…	SPTAN1	TWF2	RPSAP…	B4GAL…	SUPT1…	CCNI	IL1RAP	TP53I…	KY	GLT1D1	F8A1	ORAI2	MAPKA…
CELLMARKERMSIGDB_TRAVAGLINI_LUNG_OLR1_CLA…	CXCL16	CLEC7A	RPS26	LRG1	LCP1	B4GAL…	ITGAX	OLIG1	GPX1	SEC61B	PDSS1	NIBAN2	DNAAF1	LILRA5	ATP1B3	MARCK…	H3-3B	LILRB3	ELL2	TLR4	ACOD1	NEAT1	KCNN4	PSTPI…	ZEB2	GCH1	PDE4B	VIM	LY96	ACSL4
CELLMARKERMSIGDB_TRAVAGLINI_LUNG_PLATELET…	H2AJ	ITM2B	AIG1	SYMPK	FSTL1	GP6	H1-2	FKBP8	GNAS	TMEM91	TMEM1…	ANKRD9	JAM3	TCEAL9	ARRB1	RDH11	BIN2	ARPC5	PSTPI…	PCSK6	GSTA1	FHL1	RBBP6	FKBP1A	H1-0	PPP1R…	FAXDC2	TUBA1C	INKA1	IGF2B…
CELLMARKERMSIGDB_TRAVAGLINI_LUNG_PROLIFER…	SLC25…	CYC1	TMEM2…	YEATS4	MIS18…	DDX39A	KRT17	RPLP0	CDKN2D	TUBG1	LSM4	VRK1	DLK2	C16or…	FAM10…	ANP32E	CENPS	DDB2	HELLS	ATP5M…	ARL6I…	PTN	NPM3	HAUS1	UQCRC1	TPI1	RCC1	ITGB3…	PPP1R…	PPIH
CELLMARKERMSIGDB_TRAVAGLINI_LUNG_PROLIFER…	GAA	POLD3	LIG1	SIRPA	RFC4	TCF7L2	FCGR1…	PELAT…	UBE2S	APIP	VMA21	PRRG4	VAT1	BHLHE…	OSBPL…	GPSM2	CD163	ANXA5	RAB13	HIRIP3	CKAP2	SNX10	LRR1	REEP3	TRIOBP	ANXA2	IFI27…	MYBL2	MCOLN1	PTMS
CELLMARKERMSIGDB_TRAVAGLINI_LUNG_PROXIMAL…	RPS14	RPS19	GRTP1	DDB2	DUT	NDRG2	SYNGR1	FGFR3	PHLDA3	SPRY1	RPS17	UACA	ECE1	MMP10	TRIP6	SLC25…	PRR15	ZNF428	RIPK4	MRTFB	RPL9	RPS27A	EIF2A	CBR1	ITM2B	PYCARD	NUDT8	CHP2	POLD4	TMEM40
CELLMARKERMSIGDB_TRAVAGLINI_LUNG_SEROUS_C…																														
CELLMARKERMSIGDB_TRAVAGLINI_LUNG_SIGNALIN…																														
CELLMARKERMSIGDB_TRAVAGLINI_LUNG_TREM2_DE…	SPI1	IL13R…	IFI6	MS4A6A	PLA2G…	PLEKH…	NANS	NPC2	DBI	MYOF	PALLD	PAPSS1	CD58	SHTN1	TMEM70	TBC1D2	PRCP	SPARC	SPG21	PRDX3	TNFSF…	CXCL16	SLC38…	EPHX1	PRXL2A	SERPI…	COMT	ZNF38…	CHCHD…	VKORC1
CELLMARKERMSIGDB_TRAVAGLINI_LUNG_VASCULAR…																														
CELLMARKERMSIGDB_TRAVAGLINI_LUNG_VEIN_CELL																														
CELLMARKERMSIGDB_VANGURP_PANCREATIC_ALPHA…																														
CELLMARKERMSIGDB_VANGURP_PANCREATIC_DELTA…																														
CELLMARKERMSIGDB_VANGURP_PANCREATIC_GAMMA…																														
CELLMARKERMSIGDB_ZHENG_CORD_BLOOD_C1_PUTA…																														
CELLMARKERMSIGDB_ZHENG_CORD_BLOOD_C10_MUL…																														
CELLMARKERMSIGDB_ZHENG_CORD_BLOOD_C2_PUTA…																														
CELLMARKERMSIGDB_ZHENG_CORD_BLOOD_C3_PUTA…																														
CELLMARKERMSIGDB_ZHENG_CORD_BLOOD_C4_PUTA…																														
CELLMARKERMSIGDB_ZHENG_CORD_BLOOD_C5_SIMI…																														
CELLMARKERMSIGDB_ZHENG_CORD_BLOOD_C6_HSC_…																														
CELLMARKERMSIGDB_ZHENG_CORD_BLOOD_C7_PUTA…																														
CELLMARKERMSIGDB_ZHENG_CORD_BLOOD_C8_PUTA…																														
CELLMARKERMSIGDB_ZHONG_PFC_C1_ASTROCYTE																														
CELLMARKERMSIGDB_ZHONG_PFC_C1_DLX5_POS_IN…																														
CELLMARKERMSIGDB_ZHONG_PFC_C2_ASCL1_POS_A…																														

Table S5: Cell state marker gene list

CELLMARKERCAN_ACINAR_CELLS																														
CELLMARKERMSIGDB_ZHONG_PFC_C2_UNKNOWN_NPC																														
CELLMARKERMSIGDB_ZHONG_PFC_C3_ASTROCYTE	XPR1	SPTBN4	DENND…	FAM13…	SEMA6C	BICDL1	GRP	ZNF611	GOLGA…	STRBP	LETM1	DIP2C	MAPK8…	GAD2	BPTF	ZCCHC…	RPS6K…	CACNB1	RBFOX3	PPP1R…	SMPD3	TSPOA…	NFATC…	AKT3	KDM4B	KCTD7	NCAM1	ZNF566	GPR161	FMR1
CELLMARKERMSIGDB_ZHONG_PFC_C3_HOPX_POS_OPC																														
CELLMARKERMSIGDB_ZHONG_PFC_C3_MICROGLIA	NCS1	RNF220	FAT3	C8orf…	RIC3	UBE2Q…	NT5C3B	PJA1	NLN	TXNL4A	NEFM	POLR3A	MAP1B	PREPL	KIF3A	SYT4	CARM1	CACNA…	PHPT1	PPHLN1	ZNF614	MIR12…	FKBP1B	TRIM44	LINC0…	B4GAL…	CAP2	CACNA…	CRMP1	GAREM2
CELLMARKERMSIGDB_ZHONG_PFC_C3_UNKNOWN_INP																														
CELLMARKERMSIGDB_ZHONG_PFC_C4_UNKNOWN_INP																														
CELLMARKERMSIGDB_ZHONG_PFC_C6_PLXNA4_POS_…																														
CELLMARKERMSIGDB_ZHONG_PFC_C7_ORG_UNDERGO…																														
CELLMARKERMSIGDB_ZHONG_PFC_C8_ORG_PROLIFE…																														
CELLMARKERMSIGDB_ZHONG_PFC_C8_UNKNOWN_NEU…																														
CELLMARKERMSIGDB_ZHONG_PFC_C9_ORG_OTHER																														
CELLMARKERMSIGDB_ZHONG_PFC_HES1_POS_C1_NPC																														
CELLMARKERMSIGDB_ZHONG_PFC_MAJOR_TYPES_AS…	C3orf…	PLAGL1	CD63	HLA-B	ADGRB1	CNN3	HTRA1	NOTCH3	DNER	IFI27…	TEX264	HSD17…	ACSL6	TTYH2	TECPR2	SOX8	ID2	SPATA6	TCF7L2	NTRK2	GPRC5B	SDC3	YIF1A	GULP1	ITPR2	TP53I3	IDI1	COMT	PLIN3	TSPAN7
CELLMARKERMSIGDB_ZHONG_PFC_MAJOR_TYPES_EX…																														
CELLMARKERMSIGDB_ZHONG_PFC_MAJOR_TYPES_MI…	LINC0…	ATP2B…	ELF1	RBMS1	GAS6	TNFRS…	ZFP36…	SLC29…	RUNX1	MGAT4A	CD83	HLA-B	IL16	PARVG	ATP8B4	RIN2	GPSM3	HCLS1	OSTF1	NAGA	PALD1	IGF1	IL17RA	MAN2B1	KCTD12	TFEC	GLIPR1	H1-2	RAB32	SASH3
CELLMARKERMSIGDB_ZHONG_PFC_MAJOR_TYPES_OPC																														

CELLMARKERCAN_ACINAR_CELLS																														
CELLMARKERCAN_ADIPOCYTE_PROGENITOR_CELLS																														
CELLMARKERCAN_ADRENERGIC_NEURONS																														
CELLMARKERCAN_AIRWAY_EPITHELIAL_CELLS																														
CELLMARKERCAN_ALPHA_CELLS																														
CELLMARKERCAN_ALVEOLAR_MACROPHAGES																														
CELLMARKERCAN_ASTROCYTES																														
CELLMARKERCAN_B_CELLS																														
CELLMARKERCAN_B_CELLS_MEMORY																														
CELLMARKERCAN_B_CELLS_NAIVE																														
CELLMARKERCAN_BASOPHILS																														
CELLMARKERCAN_BERGMANN_GLIA																														
CELLMARKERCAN_BETA_CELLS																														
CELLMARKERCAN_CAJAL_RETZIUS_CELLS																														
CELLMARKERCAN_CARDIAC_STEM_AND_PRECURSOR_…																														
CELLMARKERCAN_CARDIOMYOCYTES																														
CELLMARKERCAN_CHOLANGIOCYTES																														
CELLMARKERCAN_CHOLINERGIC_NEURONS																														
CELLMARKERCAN_CHONDROCYTES																														
CELLMARKERCAN_CHOROID_PLEXUS_CELLS																														
CELLMARKERCAN_CILIATED_CELLS																														
CELLMARKERCAN_DELTA_CELLS																														
CELLMARKERCAN_DOPAMINERGIC_NEURONS																														
CELLMARKERCAN_DUCTAL_CELLS																														
CELLMARKERCAN_EMBRYONIC_STEM_CELLS																														
CELLMARKERCAN_ENTERIC_GLIA_CELLS																														
CELLMARKERCAN_ENTERIC_NEURONS																														
CELLMARKERCAN_ENTEROCYTES																														
CELLMARKERCAN_ENTEROENDOCRINE_CELLS																														
CELLMARKERCAN_EPENDYMAL_CELLS																														
CELLMARKERCAN_EPSILON_CELLS																														
CELLMARKERCAN_FOLLICULAR_CELLS																														
CELLMARKERCAN_FOVEOLAR_CELLS																														
CELLMARKERCAN_GAMMA_DELTA_T_CELLS																														
CELLMARKERCAN_GERM_CELLS																														
CELLMARKERCAN_GLUTAMINERGIC_NEURONS																														
CELLMARKERCAN_GRANULOSA_CELLS																														
CELLMARKERCAN_HEMATOPOIETIC_STEM_CELLS																														
CELLMARKERCAN_IMMATURE_NEURONS																														
CELLMARKERCAN_INTERNEURONS																														
CELLMARKERCAN_IONOCYTES																														
CELLMARKERCAN_KERATINOCYTES																														
CELLMARKERCAN_KUPFFER_CELLS																														
CELLMARKERCAN_LEYDIG_CELLS																														
CELLMARKERCAN_LOOP_OF_HENLE_CELLS																														
CELLMARKERCAN_LUMINAL_EPITHELIAL_CELLS																														
CELLMARKERCAN_LUTEAL_CELLS																														
CELLMARKERCAN_MEGAKARYOCYTES																														
CELLMARKERCAN_MELANOCYTES																														
CELLMARKERCAN_MESANGIAL_CELLS																														
CELLMARKERCAN_MESOTHELIAL_CELLS																														
CELLMARKERCAN_MICROFOLD_CELLS																														
CELLMARKERCAN_MICROGLIA																														
CELLMARKERCAN_MONOCYTES																														
CELLMARKERCAN_MOTOR_NEURONS																														
CELLMARKERCAN_MULLER_CELLS																														
CELLMARKERCAN_MYELOID_DERIVED_SUPPRESSOR_…																														
CELLMARKERCAN_MYOBLASTS																														
CELLMARKERCAN_MYOEPITHELIAL_CELLS																														
CELLMARKERCAN_MYOFIBROBLASTS																														
CELLMARKERCAN_NEUROBLASTS																														
CELLMARKERCAN_NEUROENDOCRINE_CELLS																														
CELLMARKERCAN_NEURONS																														
CELLMARKERCAN_NEUTROPHILS																														
CELLMARKERCAN_NK_CELLS																														
CELLMARKERCAN_NORADRENERGIC_NEURONS																														
CELLMARKERCAN_NUOCYTES																														
CELLMARKERCAN_OLIGODENDROCYTES																														
CELLMARKERCAN_OSTEOBLASTS																														
CELLMARKERCAN_OSTEOCLAST_PRECURSOR_CELLS																														
CELLMARKERCAN_OSTEOCLASTS																														
CELLMARKERCAN_OSTEOCYTES																														
CELLMARKERCAN_PANETH_CELLS																														
CELLMARKERCAN_PARATHYROID_CHIEF_CELLS																														
CELLMARKERCAN_PERICYTES																														
CELLMARKERCAN_PERITUBULAR_MYOID_CELLS																														
CELLMARKERCAN_PHOTORECEPTOR_CELLS																														
CELLMARKERCAN_PLASMA_CELLS																														
CELLMARKERCAN_PLASMACYTOID_DENDRITIC_CELLS																														
CELLMARKERCAN_PODOCYTES																														
CELLMARKERCAN_PROXIMAL_TUBULE_CELLS																														
CELLMARKERCAN_PULMONARY_ALVEOLAR_TYPE_I_C…																														
CELLMARKERCAN_PULMONARY_ALVEOLAR_TYPE_II_…																														
CELLMARKERCAN_PURKINJE_NEURONS																														
CELLMARKERCAN_RETICULOCYTES																														
CELLMARKERCAN_RETINAL_GANGLION_CELLS																														
CELLMARKERCAN_RETINAL_PROGENITOR_CELLS																														
CELLMARKERCAN_SATELLITE_CELLS																														
CELLMARKERCAN_SCHWANN_CELLS																														
CELLMARKERCAN_SEROTONERGIC_NEURONS																														
CELLMARKERCAN_SERTOLI_CELLS																														
CELLMARKERCAN_SMOOTH_MUSCLE_CELLS																														
CELLMARKERCAN_STROMAL_CELLS																														
CELLMARKERCAN_T_CELLS																														
CELLMARKERCAN_T_CYTOTOXIC_CELLS																														
CELLMARKERCAN_T_FOLLICULAR_HELPER_CELLS																														
CELLMARKERCAN_T_MEMORY_CELLS																														
CELLMARKERCAN_TRANSIENT_CELLS																														
CELLMARKERCAN_TRICHOCYTES																														
CELLMARKERCAN_TRIGEMINAL_NEURONS																														
CELLMARKERCAN_TROPHOBLAST_CELLS																														
CELLMARKERCAN_TROPHOBLAST_PROGENITOR_CELLS																														
CELLMARKERCAN_TUFT_CELLS																														
CELLMARKERCANENDOTHELIALCELLSAORTA																														
CELLMARKERCUR_N_ABNORMAL_MYELOID_CELL																														

Table S5: Cell state marker gene list

CELLMARKERCAN_ACINAR_CELLS																														
CELLMARKERCUR_N_ABNORMAL_PLASMA_CELL																														
CELLMARKERCUR_N_ACTIVATED_DENDRITIC_CELL																														
CELLMARKERCUR_N_ACTIVATED_EFFECTOR_CELL																														
CELLMARKERCUR_N_ACTIVATED_HEPATIC_STELLAT…																														
CELLMARKERCUR_N_ACTIVATED_LANGERHANS_CELL																														
CELLMARKERCUR_N_ACTIVATED_MACROPHAGE																														
CELLMARKERCUR_N_ACTIVATED_MEMORY_T_CELL																														
CELLMARKERCUR_N_ACTIVATED_NATURAL_KILLER_…																														
CELLMARKERCUR_N_ACTIVATED_REGULATORY_T_CE…																														
CELLMARKERCUR_N_ADIPOCYTE_PROGENITOR_CELL																														
CELLMARKERCUR_N_ADRENAL_MEDULLA_CELL																														
CELLMARKERCUR_N_ADULT_SPERMATOGONIA																														
CELLMARKERCUR_N_ADVENTITIAL_CELL																														
CELLMARKERCUR_N_AEROCYTE																														
CELLMARKERCUR_N_AIRWAY_SMOOTH_MUSCLE_CELL																														
CELLMARKERCUR_N_ALVEOLAR_CELL_TYPE_1																														
CELLMARKERCUR_N_ALVEOLAR_CELL_TYPE_2																														
CELLMARKERCUR_N_ALVEOLAR_EPITHELIAL_CELL_…																														
CELLMARKERCUR_N_ALVEOLAR_EPITHELIAL_CELL_…																														
CELLMARKERCUR_N_ALVEOLAR_PNEUMOCYTE_TYPE_I																														
CELLMARKERCUR_N_ALVEOLAR_PNEUMOCYTE_TYPE_…																														
CELLMARKERCUR_N_AMACRINE_CELL																														
CELLMARKERCUR_N_AMNIOTIC_FLUID_STEM_CELL																														
CELLMARKERCUR_N_ANTERIOR_CELL																														
CELLMARKERCUR_N_ANTERIOR_HINDBRAIN_CELL																														
CELLMARKERCUR_N_ANTI_TUMOR_IMMUNE_CELL																														
CELLMARKERCUR_N_ANTIGEN_PRESENTATION_CANC…																														
CELLMARKERCUR_N_ASTROCYTE																														
CELLMARKERCUR_N_ATRIAL_CELL																														
CELLMARKERCUR_N_ATRIOVENTRICULAR_CANAL_CE…																														
CELLMARKERCUR_N_ATYPICAL_MEMORY_B_CELL																														
CELLMARKERCUR_N_B_2_CELL																														
CELLMARKERCUR_N_B1_CELL																														
CELLMARKERCUR_N_B10_REGULATORY_B_CELL																														
CELLMARKERCUR_N_BASAL_CELL																														
CELLMARKERCUR_N_BASAL_EPITHELIAL_CELL																														
CELLMARKERCUR_N_BASAL_KERATINOCYTE																														
CELLMARKERCUR_N_BASAL_LIKE_CELL																														
CELLMARKERCUR_N_BASAL_PROGENITOR_CELL																														
CELLMARKERCUR_N_BASOPHIL																														
CELLMARKERCUR_N_BILIARY_CELL																														
CELLMARKERCUR_N_BONE_MARROW_STEM_CELL																														
CELLMARKERCUR_N_BONE_SPECIFIC_CELL																														
CELLMARKERCUR_N_BRAIN_CELL																														
CELLMARKERCUR_N_CANCER_CELL	MME	RAB29	PIM2	ITPKB	TNFRS…	FGFR1	CCDC50	FOXP1	NCAM1	FUT4	PDLIM7	SLC22…	CD79A	CR2	POU2A…	B3GAT1	STAT3	SARM1	SOX18	WFDC2	GPC1	WNT2	KARS1	AMACR	ATAD2	PFKFB4	PIAS2	UBE2T	SOX10	DCT
CELLMARKERCUR_N_CANCER_STEM_LIKE_CELL																														
CELLMARKERCUR_N_CAPILLARY_CELL																														
CELLMARKERCUR_N_CAR_T_CELL																														
CELLMARKERCUR_N_CARDIAC_CELL																														
CELLMARKERCUR_N_CARDIOMYOCYTE																														
CELLMARKERCUR_N_CARDIOSPHERE_DERIVED_CELL																														
CELLMARKERCUR_N_CARDIOVASCULAR_PROGENITOR…																														
CELLMARKERCUR_N_CAUDAL_CORTEX_CELL																														
CELLMARKERCUR_N_CD14_MONOCYTE																														
CELLMARKERCUR_N_CD16_MONOCYTE																														
CELLMARKERCUR_N_CD4_CD28_T_CELL																														
CELLMARKERCUR_N_CD4_PERIPHERALLY_DERIVED_…																														
CELLMARKERCUR_N_CD4_T_CELL																														
CELLMARKERCUR_N_CD56BRIGHT_NATURAL_KILLER…																														
CELLMARKERCUR_N_CD56DIM_NATURAL_KILLER_CE…																														
CELLMARKERCUR_N_CD8_T_CELL																														
CELLMARKERCUR_N_CENTRAL_MEMORY_T_CELL																														
CELLMARKERCUR_N_CENTRAL_MEMORY_T_HELPER_1…																														
CELLMARKERCUR_N_CENTRAL_NERVOUS_SYSTEM_ST…																														
CELLMARKERCUR_N_CHANDELIER_CELL																														
CELLMARKERCUR_N_CHIEF_CELL																														
CELLMARKERCUR_N_CHONDROCYTE																														
CELLMARKERCUR_N_CHROMAFFIN_CELL																														
CELLMARKERCUR_N_CIRCULATING_ANGIOGENIC_CE…																														
CELLMARKERCUR_N_CIRCULATING_FETAL_CELL																														
CELLMARKERCUR_N_CIRCULATING_NATURAL_KILLE…																														
CELLMARKERCUR_N_CIRCULATING_PRECURSOR_CELL																														
CELLMARKERCUR_N_CIRCULATING_PROGENITOR_CE…																														
CELLMARKERCUR_N_CIRCULATING_T_FOLLICULAR_…																														
CELLMARKERCUR_N_CIRCULATING_TUMOR_CELL																														
CELLMARKERCUR_N_CLASS_SWITCHED_PLASMABLAST																														
CELLMARKERCUR_N_CLASSICAL_B_CELL																														
CELLMARKERCUR_N_CLASSICAL_CANCER_ASSOCIAT…																														
CELLMARKERCUR_N_CLASSICAL_MONOCYTE																														
CELLMARKERCUR_N_CLAUDIN_LOW_CELL																														
CELLMARKERCUR_N_COLLECTING_DUCTAL_CELL																														
CELLMARKERCUR_N_COLONCYTE																														
CELLMARKERCUR_N_COLORECTAL_STEM_CELL																														
CELLMARKERCUR_N_COLUMNAR_EPITHELIAL_CELL																														
CELLMARKERCUR_N_COMMON_LYMPHOID_PROGENITO…																														
CELLMARKERCUR_N_COMMON_MYELOID_PROGENITOR…																														
CELLMARKERCUR_N_COMPLEMENT_SECRETING_CANC…																														
CELLMARKERCUR_N_CONE_PHOTORECEPTOR_CELL																														
CELLMARKERCUR_N_CONNECTING_TUBULAR_CELL																														
CELLMARKERCUR_N_CONVENTIONAL_CD4_T_CELL																														
CELLMARKERCUR_N_CORNEAL_ENDOTHELIAL_CELL																														
CELLMARKERCUR_N_CORNEAL_EPITHELIAL_CELL																														
CELLMARKERCUR_N_CORNEOCYTE_LIKE_CELL																														
CELLMARKERCUR_N_CORTICAL_CELL																														
CELLMARKERCUR_N_CORTICAL_STROMAL_CELL																														
CELLMARKERCUR_N_CORTICOTROPH_TUMOR_CELL																														
CELLMARKERCUR_N_CRANIAL_NEURAL_CREST_CELL																														
CELLMARKERCUR_N_CRYPT_CELL																														
CELLMARKERCUR_N_CRYPT_STEM_CELL																														
CELLMARKERCUR_N_CYCLING_B_CELL																														
CELLMARKERCUR_N_CYCLING_EPITHELIAL_CELL																														
CELLMARKERCUR_N_CYTOTOXIC_CD8_T_CELL																														
CELLMARKERCUR_N_CYTOTOXIC_DOUBLE_POSITIVE…																														
CELLMARKERCUR_N_CYTOTOXIC_T_CELL																														

CELLMARKERCAN_ACINAR_CELLS																														
CELLMARKERCUR_N_CYTOTOXIC_T_CELL_2																														
CELLMARKERCUR_N_DAMAGE_ASSOCIATED_TRANSIE…																														
CELLMARKERCUR_N_DARK_ZONE_CELL																														
CELLMARKERCUR_N_DEDIFFERENTIATION_ADIPOCY…																														
CELLMARKERCUR_N_DEEP_LAYER_NEURON																														
CELLMARKERCUR_N_DEFINITIVE_ENDODERM_CELL																														
CELLMARKERCUR_N_DELTA_CELL																														
CELLMARKERCUR_N_DEMYELINATING_MICROGLIAL_…																														
CELLMARKERCUR_N_DENDRITIC_CELL_2A																														
CELLMARKERCUR_N_DENDRITIC_CELL_LINEAGE																														
CELLMARKERCUR_N_DENTAL_PULP_CELL																														
CELLMARKERCUR_N_DERMAL_CELL																														
CELLMARKERCUR_N_DEUTEROSOMAL_CELL																														
CELLMARKERCUR_N_DIFFUSE_GASTRIC_CANCER_CE…																														
CELLMARKERCUR_N_DISTAL_CELL																														
CELLMARKERCUR_N_DISTAL_STEM_CELL																														
CELLMARKERCUR_N_DISTAL_TUBULAR_CELL																														
CELLMARKERCUR_N_DIVIDING_CELL																														
CELLMARKERCUR_N_DIVIDING_PLASMA_B_CELL																														
CELLMARKERCUR_N_DIVIDING_T_CELL																														
CELLMARKERCUR_N_DOPAMINERGIC_NEURON																														
CELLMARKERCUR_N_DORSAL_ANTERIOR_HINDBRAIN…																														
CELLMARKERCUR_N_DORSAL_LATERAL_GANGLIONIC…																														
CELLMARKERCUR_N_DORSAL_MEDIAL_GANGLIONIC_…																														
CELLMARKERCUR_N_DOUBLE_NEGATIVE_B_CELL																														
CELLMARKERCUR_N_DOUBLE_NEGATIVE_T_CELL																														
CELLMARKERCUR_N_DOUBLE_POSITIVE_T_CELL																														
CELLMARKERCUR_N_DOUBLE_POSITIVE_TREG_CELL																														
CELLMARKERCUR_N_EARLY_HEMANGIOBLAST																														
CELLMARKERCUR_N_EARLY_PROXIMAL_TUBULE_CELL																														
CELLMARKERCUR_N_EARLY_RESPONSE_SECRETORY_…																														
CELLMARKERCUR_N_EFFECTOR_CHONDROCYTE																														
CELLMARKERCUR_N_EFFECTOR_MEMORY_T_CELL																														
CELLMARKERCUR_N_EFFECTOR_NATURAL_KILLER_C…																														
CELLMARKERCUR_N_ELONGATING_CELL																														
CELLMARKERCUR_N_EMBRYONIC_CELL																														
CELLMARKERCUR_N_EMBRYONIC_STEM_CELL																														
CELLMARKERCUR_N_ENDOCARDIAL_CELL																														
CELLMARKERCUR_N_ENDOCRINE_PROGENITOR_CELL																														
CELLMARKERCUR_N_ENDODERM_PROGENITOR_CELL																														
CELLMARKERCUR_N_ENDODERMAL_CELL																														
CELLMARKERCUR_N_ENDOMETRIAL_STROMAL_CELL																														
CELLMARKERCUR_N_ENDOMETRIAL_STROMAL_STEM_…																														
CELLMARKERCUR_N_ENDOTHELIAL_PRECURSOR_CELL																														
CELLMARKERCUR_N_ENSHEATHING_GLIAL_CELL																														
CELLMARKERCUR_N_ENTERIC_NEURON																														
CELLMARKERCUR_N_EOSINOPHIL																														
CELLMARKERCUR_N_EPENDYMAL_CELL																														
CELLMARKERCUR_N_EPIBLAST_CELL																														
CELLMARKERCUR_N_EPICARDIAL_CELL																														
CELLMARKERCUR_N_EPICARDIUM_DERIVED_CELL																														
CELLMARKERCUR_N_EPIDERMAL_CELL																														
CELLMARKERCUR_N_EPIDERMAL_STEM_CELL																														
CELLMARKERCUR_N_EPITHELIAL_CELL	ANLN	FOXM1	TGM1	ITGB1	CKAP2L	HMGB2	EZH2	BRD4	SCEL	KRT80	VIL1	CLDN7	YAP1	TNFRS…	WNT4	HOXB2	DLK2	FOXN1	PSMB11	AIRE	MYOD1	ITGB4	LY75	FEZF2	ID4	S100A…	TPO	IYD	MGST1	FN1
CELLMARKERCUR_N_EPITHELIAL_DUCTAL_CELL																														
CELLMARKERCUR_N_EPITHELIAL_MESENCHYMAL_CE…																														
CELLMARKERCUR_N_EPITHELIOID_MACROPHAGE																														
CELLMARKERCUR_N_EPSILON_CELL																														
CELLMARKERCUR_N_ERYTHROBLAST																														
CELLMARKERCUR_N_ERYTHROID_CELL																														
CELLMARKERCUR_N_ERYTHROID_PROGENITOR_CELL																														
CELLMARKERCUR_N_ESOPHAGEAL_CELL																														
CELLMARKERCUR_N_ESOPHAGEAL_PROGENITOR_CELL																														
CELLMARKERCUR_N_EXCITATORY_NEURON																														
CELLMARKERCUR_N_EXHAUSTED_DOUBLE_POSITIVE…																														
CELLMARKERCUR_N_EXTRAVILLOUS_TROPHOBLAST																														
CELLMARKERCUR_N_F_PERICYTE																														
CELLMARKERCUR_N_FETAL_MYOBLAST																														
CELLMARKERCUR_N_FIBROBLAST_LIKE_CELL																														
CELLMARKERCUR_N_FIBROCHONDROCYTE																														
CELLMARKERCUR_N_FIBROCHONDROCYTE_PROGENIT…																														
CELLMARKERCUR_N_FOAM_CELL																														
CELLMARKERCUR_N_FOLLICULAR_B_CELL																														
CELLMARKERCUR_N_FOLLICULAR_CELL																														
CELLMARKERCUR_N_FOLLICULAR_DENDRITIC_CELL																														
CELLMARKERCUR_N_FOREBRAIN_CELL																														
CELLMARKERCUR_N_GABAERGIC_NEURON																														
CELLMARKERCUR_N_GANGLION_CELL																														
CELLMARKERCUR_N_GASTRIC_NEUROENDOCRINE_CE…																														
CELLMARKERCUR_N_GASTRIC_STEM_CELL																														
CELLMARKERCUR_N_GERM_CELL																														
CELLMARKERCUR_N_GERMINAL_CENTER_B_CELL																														
CELLMARKERCUR_N_GERMINAL_CENTER_CELL																														
CELLMARKERCUR_N_GERMINAL_CENTRE_B_CELL																														
CELLMARKERCUR_N_GLIAL_CELL																														
CELLMARKERCUR_N_GLIOMA_STEM_CELL																														
CELLMARKERCUR_N_GLOBOSE_BASAL_CELL																														
CELLMARKERCUR_N_GLOMERULAR_PARIETAL_EPITH…																														
CELLMARKERCUR_N_GLUTAMATERGIC_NEURON																														
CELLMARKERCUR_N_GOBLET_CELL																														
CELLMARKERCUR_N_GONADOTROPE																														
CELLMARKERCUR_N_GONADOTROPH_CELL																														
CELLMARKERCUR_N_GRANULOCYTE																														
CELLMARKERCUR_N_GRANULOCYTE_MONOCYTE_PROG…																														
CELLMARKERCUR_N_GRANULOCYTIC_UNK																														
CELLMARKERCUR_N_GRANULOSA_CELL																														
CELLMARKERCUR_N_HAEMATOPOIETIC_CELL																														
CELLMARKERCUR_N_HAIR_FOLLICLE_CELL																														
CELLMARKERCUR_N_HEMANGIOBLAST																														
CELLMARKERCUR_N_HEMATOPOIETIC_CELL																														
CELLMARKERCUR_N_HEMATOPOIETIC_PRECURSOR_C…																														
CELLMARKERCUR_N_HEMATOPOIETIC_PROGENITOR_…																														
CELLMARKERCUR_N_HEPATIC_CELL																														
CELLMARKERCUR_N_HEPATIC_PROGENITOR_CELL																														

CELLMARKERCAN_ACINAR_CELLS																														
CELLMARKERCUR_N_HEPATIC_STELLATE_CELL																														
CELLMARKERCUR_N_HEPATOBLAST																														
CELLMARKERCUR_N_HEPATOCYTE																														
CELLMARKERCUR_N_HISTIOCYTE																														
CELLMARKERCUR_N_HMSC_TREATED_B_CELL																														
CELLMARKERCUR_N_HOFBAUER_CELL																														
CELLMARKERCUR_N_HOMEOSTATIC_CHONDROCYTE																														
CELLMARKERCUR_N_HOMEOSTATIC_MICROGLIAL_CE…																														
CELLMARKERCUR_N_HORIZONTAL_BASAL_CELL																														
CELLMARKERCUR_N_HORIZONTAL_CELL																														
CELLMARKERCUR_N_HYPOBLAST_CELL																														
CELLMARKERCUR_N_IDIOPATHIC_PULMONARY_FIBR…																														
CELLMARKERCUR_N_IGA_MEMORY_B_CELL																														
CELLMARKERCUR_N_IGG_MEMORY_B_CELL																														
CELLMARKERCUR_N_IGM_MEMORY_B_CELL																														
CELLMARKERCUR_N_IGM_ONLY_B_CELL																														
CELLMARKERCUR_N_IL7R_T_HELPER_CELL																														
CELLMARKERCUR_N_IMMATURE_ERYTHROID_CELL																														
CELLMARKERCUR_N_IMMATURE_MYELOID_CELL																														
CELLMARKERCUR_N_IMMATURE_NEUTROPHIL																														
CELLMARKERCUR_N_IMMATURE_TRANSITIONAL_B_C…																														
CELLMARKERCUR_N_IMMUNE_CELL																														
CELLMARKERCUR_N_INDUCED_PLURIPOTENT_STEM_…																														
CELLMARKERCUR_N_INFILTRATING_MACROPHAGE																														
CELLMARKERCUR_N_INFLAMMATORY_CANCER_ASSOC…																														
CELLMARKERCUR_N_INFLAMMATORY_DENDRITIC_CE…																														
CELLMARKERCUR_N_INFLAMMATORY_MONOCYTE																														
CELLMARKERCUR_N_INHIBITORY_NEURON																														
CELLMARKERCUR_N_INNATE_LYMPHOID_CELL																														
CELLMARKERCUR_N_INNER_CELL_MASS_CELL																														
CELLMARKERCUR_N_INNER_RADIAL_GLIAL_CELL																														
CELLMARKERCUR_N_INTER_MEDIATE_PROGENITOR_…																														
CELLMARKERCUR_N_INTERCALATED_CELL																														
CELLMARKERCUR_N_INTERFERON_RESPONSIVE_SEC…																														
CELLMARKERCUR_N_INTERMEDIATE_MONOCYTE																														
CELLMARKERCUR_N_INTERMEDIATE_MONOCYTE_CELL																														
CELLMARKERCUR_N_INTERSTITIAL_MACROPHAGE																														
CELLMARKERCUR_N_INTERSTITIAL_PROGENITOR_C…																														
CELLMARKERCUR_N_INTESTINAL_EPITHELIAL_STE…																														
CELLMARKERCUR_N_INTESTINAL_GASTRIC_CANCER…																														
CELLMARKERCUR_N_INTESTINAL_METAPLASIA_CELL																														
CELLMARKERCUR_N_INTRAEPITHELIAL_LYMPHOCYT…																														
CELLMARKERCUR_N_IONOCYTE																														
CELLMARKERCUR_N_IRIS_PUTTATIVE_STEM_CELL																														
CELLMARKERCUR_N_KUPFFER_CELL																														
CELLMARKERCUR_N_LACTOTROPH_CELL																														
CELLMARKERCUR_N_LANGERHANS_CELL																														
CELLMARKERCUR_N_LEUKOCYTE																														
CELLMARKERCUR_N_LIMBAL_EPITHELIAL_STEM_CE…																														
CELLMARKERCUR_N_LIMBAL_STEM_CELL																														
CELLMARKERCUR_N_LIPOFIBROBLAST																														
CELLMARKERCUR_N_LIVER_PROGENITOR_CELL																														
CELLMARKERCUR_N_LIVER_STEM_CELL																														
CELLMARKERCUR_N_LMMLEYDIG_CELL																														
CELLMARKERCUR_N_LONOCYTE																														
CELLMARKERCUR_N_LOOP_OF_HENLE_CELL																														
CELLMARKERCUR_N_LOOP_PROGENITOR_CELL																														
CELLMARKERCUR_N_LOW_DENSITY_NEUTROPHIL																														
CELLMARKERCUR_N_LUMINAL_PROGENITOR_CELL																														
CELLMARKERCUR_N_LUNG_CELL																														
CELLMARKERCUR_N_LYMPH_NODE_CELL																														
CELLMARKERCUR_N_LYMPHATIC_CELL																														
CELLMARKERCUR_N_LYMPHATIC_ENDOTHELIAL_CELL																														
CELLMARKERCUR_N_LYMPHOBLASTOID_CELL																														
CELLMARKERCUR_N_LYMPHOCYTE																														
CELLMARKERCUR_N_LYMPHOID_CELL																														
CELLMARKERCUR_N_LYMPHOID_PROGENITOR_CELL																														
CELLMARKERCUR_N_LYMPHOID_STRUCTURE_CELL																														
CELLMARKERCUR_N_M_PERICYTE																														
CELLMARKERCUR_N_M1_MACROPHAGE																														
CELLMARKERCUR_N_M1_MICROGLIAL_CELL																														
CELLMARKERCUR_N_M2_MACROPHAGE																														
CELLMARKERCUR_N_MACROGLIAL_CELL																														
CELLMARKERCUR_N_MALIGNANT_CELL																														
CELLMARKERCUR_N_MALIGNANT_PLASMA_CELL																														
CELLMARKERCUR_N_MARGINAL_ASSOCIATED_MACRO…																														
CELLMARKERCUR_N_MAST_CELL																														
CELLMARKERCUR_N_MATURE_ASTROCYTE																														
CELLMARKERCUR_N_MATURE_B_CELL																														
CELLMARKERCUR_N_MATURE_DENDRITIC_CELL																														
CELLMARKERCUR_N_MATURE_ENDOTHELIAL_CELL																														
CELLMARKERCUR_N_MATURE_LUMINAL_CELL																														
CELLMARKERCUR_N_MATURE_NEURON																														
CELLMARKERCUR_N_MATURE_NEURONAL																														
CELLMARKERCUR_N_MATURE_OLIGODENDROCYTE																														
CELLMARKERCUR_N_MATURE_OSTEOBLAST																														
CELLMARKERCUR_N_MATURE_PODOCYTE																														
CELLMARKERCUR_N_MEDIAL_AND_DISTAL_SEGMENT…																														
CELLMARKERCUR_N_MEDIAL_GANGLIONIC_EMINENC…																														
CELLMARKERCUR_N_MEGAKARYOCYTE																														
CELLMARKERCUR_N_MEGAKARYOCYTE_ERYTHROID_C…																														
CELLMARKERCUR_N_MEGAKARYOCYTE_ERYTHROID_P…																														
CELLMARKERCUR_N_MEGAKARYOCYTE_PROGENITOR_…																														
CELLMARKERCUR_N_MELANOMA_CELL																														
CELLMARKERCUR_N_MEMORY_B_CELL																														
CELLMARKERCUR_N_MEMORY_B_CELL_PRECURSOR																														
CELLMARKERCUR_N_MEMORY_CD27_B_CELL																														
CELLMARKERCUR_N_MEMORY_CD4_T_CELL																														
CELLMARKERCUR_N_MEMORY_DOUBLE_POSITIVE_T_…																														
CELLMARKERCUR_N_MEMORY_REGULATORY_T_CELL																														
CELLMARKERCUR_N_MEMORY_T_CELL																														
CELLMARKERCUR_N_MERKEL_CELL																														
CELLMARKERCUR_N_MESENCHYMAL_CELL																														
CELLMARKERCUR_N_MESENCHYMAL_EPITHELIAL_TR…																														

CELLMARKERCAN_ACINAR_CELLS																														
CELLMARKERCUR_N_MESENCHYMAL_GLIOBLASTOMA_…																														
CELLMARKERCUR_N_MESENCHYMAL_STEM_CELL																														
CELLMARKERCUR_N_MESENCHYMAL_STROMAL_CELL																														
CELLMARKERCUR_N_MESENDODERMAL_CELL																														
CELLMARKERCUR_N_MESODERMAL_EMBRYONIC_STEM…																														
CELLMARKERCUR_N_MESODERMAL_PROGENITOR_CELL																														
CELLMARKERCUR_N_METAPLASTIC_STEM_CELL																														
CELLMARKERCUR_N_MEYLOID_CELL																														
CELLMARKERCUR_N_MICROGLIAL_CELL	NIN	FTH1	GPR84	FLT1	CSF2RA	RUNX3	CCK	CAPG	PLD3	TENT5C	IL15	RPSA	CD83	COX8A	CLEC7A	RPLP1	ADSS1	PEBP1	MOV10…	CTSD	STEAP3	TMED3	APBB2	FAM20C	DKK2	PERP	GAS2L3	ANK1	USP12	ATP6V…
CELLMARKERCUR_N_MICROVILLAR_CELL																														
CELLMARKERCUR_N_MIDBRAIN_DOPAMINERGIC_NEU…																														
CELLMARKERCUR_N_MONO_MACROPHAGE																														
CELLMARKERCUR_N_MONOCYTE_DERIVED_CELL																														
CELLMARKERCUR_N_MONOCYTE_DERIVED_MYELOID_…																														
CELLMARKERCUR_N_MONOCYTE_LINEAGE																														
CELLMARKERCUR_N_MONOCYTE_PRECURSOR																														
CELLMARKERCUR_N_MONOCYTE_PROGENITOR_CELL																														
CELLMARKERCUR_N_MUELLER_GLIA_CELL																														
CELLMARKERCUR_N_MULTIPOTENT_MESENCHYMAL_S…																														
CELLMARKERCUR_N_MULTIPOTENT_RETINAL_PROGE…																														
CELLMARKERCUR_N_MULTIPOTENT_STEM_CELL																														
CELLMARKERCUR_N_MULTIPOTENT_STROMAL_CELL																														
CELLMARKERCUR_N_MUSCLE_CELL																														
CELLMARKERCUR_N_MUSCLE_DERIVED_CELL																														
CELLMARKERCUR_N_MUSCLE_SATELLITE_CELL																														
CELLMARKERCUR_N_MYELOBLAST																														
CELLMARKERCUR_N_MYELOID_DENDRITIC_CELL																														
CELLMARKERCUR_N_MYELOID_DENDRITIC_CELL_1																														
CELLMARKERCUR_N_MYELOID_DERIVED_CELL																														
CELLMARKERCUR_N_MYELOID_DERIVED_SUPPRESSO…																														
CELLMARKERCUR_N_MYELOID_PROGENITOR_CELL																														
CELLMARKERCUR_N_MYO_EPITHELIAL_CELL																														
CELLMARKERCUR_N_MYOBLAST																														
CELLMARKERCUR_N_MYOCYTE																														
CELLMARKERCUR_N_MYOFIBROBLAST																														
CELLMARKERCUR_N_MYOGENIC_ENDOTHELIAL_CELL																														
CELLMARKERCUR_N_MYOGENIC_PROGENITOR_CELL																														
CELLMARKERCUR_N_MYOID_CELL																														
CELLMARKERCUR_N_NAIVE_B_CELL																														
CELLMARKERCUR_N_NAIVE_CD8_T_CELL																														
CELLMARKERCUR_N_NAIVE_DOUBLE_POSITIVE_T_C…																														
CELLMARKERCUR_N_NAIVE_PROGENITOR_CELL																														
CELLMARKERCUR_N_NAIVE_T_CELL																														
CELLMARKERCUR_N_NASCENT_NEPHRON_CELL																														
CELLMARKERCUR_N_NATURAL_KILLER_CD56_BRIGH…																														
CELLMARKERCUR_N_NATURAL_KILLER_CD56_DIM_C…																														
CELLMARKERCUR_N_NEGATIVE_MESENCHYMAL_STEM…																														
CELLMARKERCUR_N_NEURAL_CELL																														
CELLMARKERCUR_N_NEURAL_CREST_STEM_CELL																														
CELLMARKERCUR_N_NEURAL_CREST_STEMLIKE_CELL																														
CELLMARKERCUR_N_NEURAL_PRECURSOR_CELL																														
CELLMARKERCUR_N_NEURAL_STEM_CELL																														
CELLMARKERCUR_N_NEUROECTODERM_CELL																														
CELLMARKERCUR_N_NEURONAL_INTERMEDIATE_PRO…																														
CELLMARKERCUR_N_NEURONAL_PROGENITOR_CELL																														
CELLMARKERCUR_N_NEUTROPHIL_PROGENITOR_CELL																														
CELLMARKERCUR_N_NON_NEURON																														
CELLMARKERCUR_N_NON_PT_TUBULAR_CELL																														
CELLMARKERCUR_N_NON_RECIRCULATING_TISSUE_…																														
CELLMARKERCUR_N_NONMYELINATING_SCHWANN_CE…																														
CELLMARKERCUR_N_NOTOCHORD_CELL																														
CELLMARKERCUR_N_NUCLEATED_CELL																														
CELLMARKERCUR_N_NUCLEUS_PULPOSUS_PROGENIT…																														
CELLMARKERCUR_N_OLIGODENDROCYTE																														
CELLMARKERCUR_N_OSTEOBLAST																														
CELLMARKERCUR_N_OUTER_RADIAL_GLIA																														
CELLMARKERCUR_N_OVALBUMIN_SPECIFIC_REGULA…																														
CELLMARKERCUR_N_OVARIAN_CELL																														
CELLMARKERCUR_N_OVARIAN_STROMAL_CELL																														
CELLMARKERCUR_N_P53_LIKE_CELL																														
CELLMARKERCUR_N_PAN_B_CELL																														
CELLMARKERCUR_N_PAN_CANCER_ASSOCIATED_FIB…																														
CELLMARKERCUR_N_PAN_MACROPHAGE																														
CELLMARKERCUR_N_PAN_MONOCYTE																														
CELLMARKERCUR_N_PAN_NEURONAL_CELL																														
CELLMARKERCUR_N_PAN_TROPHECTODERM_CELL																														
CELLMARKERCUR_N_PANCREATIC_ENDODERM_CELL																														
CELLMARKERCUR_N_PANCREATIC_POLYPEPTIDE_CE…																														
CELLMARKERCUR_N_PANCREATIC_PROGENITOR_CELL																														
CELLMARKERCUR_N_PANCREATIC_STEM_CELL																														
CELLMARKERCUR_N_PANETH_CELL																														
CELLMARKERCUR_N_PANNEURON																														
CELLMARKERCUR_N_PANVASCULAR_CELL																														
CELLMARKERCUR_N_PAPILLARY_FIBROBLAST																														
CELLMARKERCUR_N_PARIETAL_CELL																														
CELLMARKERCUR_N_PARVALBUMIN_INTERNEURON																														
CELLMARKERCUR_N_PATELET																														
CELLMARKERCUR_N_PATHOGENIC_T_PERIPHERAL_H…																														
CELLMARKERCUR_N_PELVIC_EPITHELIAL_CELL																														
CELLMARKERCUR_N_PERICYTE																														
CELLMARKERCUR_N_PERIODONTAL_LIGAMENT_STEM…																														
CELLMARKERCUR_N_PERIOSTEUM_DERIVED_PROGEN…																														
CELLMARKERCUR_N_PERITUBULAR_MYOID_CELL																														
CELLMARKERCUR_N_PERIVASCULAR_LIKE_CELL																														
CELLMARKERCUR_N_PHAGOCYTE																														
CELLMARKERCUR_N_PHOTORECEPTOR_CELL																														
CELLMARKERCUR_N_PIT_CELL																														
CELLMARKERCUR_N_PIT_MUCOUS_CELL																														
CELLMARKERCUR_N_PITUITARY_STEM_CELL																														
CELLMARKERCUR_N_PLACENTAL_TISSUE_CELL																														
CELLMARKERCUR_N_PLACODE_CELL																														
CELLMARKERCUR_N_PLASMABLAST_B_CELL																														
CELLMARKERCUR_N_PLATELET																														
CELLMARKERCUR_N_PLURIPOTENT_STEM_CELL																														

Table S5: Cell state marker gene list

CELLMARKERCAN_ACINAR_CELLS																														
CELLMARKERCUR_N_PODOCYTE_PRECURSOR																														
CELLMARKERCUR_N_PORTAL_FIBROBLAST																														
CELLMARKERCUR_N_POSTERIOR_CELL																														
CELLMARKERCUR_N_PRE_B_CELL																														
CELLMARKERCUR_N_PRE_CONVENTIONAL_DENDRITI…																														
CELLMARKERCUR_N_PRE_DENDRITIC_CELL																														
CELLMARKERCUR_N_PRECURSOR_CELL																														
CELLMARKERCUR_N_PREHYPERTROPHIC_CHONDROCY…																														
CELLMARKERCUR_N_PRESOMITE_CELL																														
CELLMARKERCUR_N_PRETUBULAR_AGGREGATE_CELL																														
CELLMARKERCUR_N_PRIMITIVE_ENDODERM_CELL																														
CELLMARKERCUR_N_PRINCIPAL_CELL																														
CELLMARKERCUR_N_PRO_B_CELL																														
CELLMARKERCUR_N_PRO_INFLAMMATORY_MACROPHA…																														
CELLMARKERCUR_N_PRO_NEUTROPHIL																														
CELLMARKERCUR_N_PROLIFERATING_CELL																														
CELLMARKERCUR_N_PROLIFERATIVE_CHONDROCYTE																														
CELLMARKERCUR_N_PROLIFERATIVE_DUCTAL_CELL																														
CELLMARKERCUR_N_PROLIFERATIVE_NEURAL_PROG…																														
CELLMARKERCUR_N_PRONEURAL_GLIOBLASTOMA_CE…																														
CELLMARKERCUR_N_PROSPERMATOGONIA																														
CELLMARKERCUR_N_PROSTATE_CELL																														
CELLMARKERCUR_N_PROXIMAL_CELL																														
CELLMARKERCUR_N_PROXIMAL_SEGMENT_CELL																														
CELLMARKERCUR_N_PROXIMAL_STEM_CELL																														
CELLMARKERCUR_N_PROXIMAL_TUBULE_CELL																														
CELLMARKERCUR_N_PULMONARY_NEUROENDOCRINE_…																														
CELLMARKERCUR_N_PYRAMIDAL_CELL																														
CELLMARKERCUR_N_QUIESCENT_T_STEM_CELL																														
CELLMARKERCUR_N_REGULATORY_B_CELL																														
CELLMARKERCUR_N_REGULATORY_INNATE_LYMPHOI…																														
CELLMARKERCUR_N_RENAL_TUBULAR_CELL																														
CELLMARKERCUR_N_RESIDENT_MEMORY_T_CELL																														
CELLMARKERCUR_N_RESPIRATORY_HORIZONTAL_BA…																														
CELLMARKERCUR_N_RESPIRATORY_SECRETORY_CELL																														
CELLMARKERCUR_N_RESPONDER_T_CELL																														
CELLMARKERCUR_N_RESPONDING_CONVENTIONAL_T…																														
CELLMARKERCUR_N_RESTING_MEMORY_B_CELL																														
CELLMARKERCUR_N_RESTING_NAIVE_B_CELL																														
CELLMARKERCUR_N_RESTING_REGULATORY_T_CELL																														
CELLMARKERCUR_N_RETICULAR_FIBROBLAST																														
CELLMARKERCUR_N_RETINA_CELL																														
CELLMARKERCUR_N_ROD_CELL																														
CELLMARKERCUR_N_SATELLITE_CELL																														
CELLMARKERCUR_N_SEPTUM_TRANSVERSUM_MESENC…																														
CELLMARKERCUR_N_SEROUS_CELL																														
CELLMARKERCUR_N_SIDE_POPULATION_CELL																														
CELLMARKERCUR_N_SKELETAL_MUSCLE_CELL																														
CELLMARKERCUR_N_SKELETAL_MUSCLE_STEM_CELL																														
CELLMARKERCUR_N_SMOOTH_MUSCLE_CELL																														
CELLMARKERCUR_N_SMOOTH_MUSCLE_LIKE_CELL																														
CELLMARKERCUR_N_SOMATIC_CELL																														
CELLMARKERCUR_N_SOMATIC_STEM_CELL																														
CELLMARKERCUR_N_SOMATOSTATIN_INTERNEURON																														
CELLMARKERCUR_N_SPECIALIST_ANTIGEN_PRESEN…																														
CELLMARKERCUR_N_SPERMATOGENIC_CELL																														
CELLMARKERCUR_N_SPERMATOGONIAL_CELL																														
CELLMARKERCUR_N_SPERMATOGONIAL_STEM_CELL																														
CELLMARKERCUR_N_SQUAMOUS_DIFFERENTIATION_…																														
CELLMARKERCUR_N_STELLATE_CELL																														
CELLMARKERCUR_N_STEM_CELL_DERIVED_ALPHA_C…																														
CELLMARKERCUR_N_STEM_CELL_DERIVED_BETA_CE…																														
CELLMARKERCUR_N_STEM_CELL_DERIVED_ENDOTHE…																														
CELLMARKERCUR_N_STEM_LIKE_CD8_T_CELL																														
CELLMARKERCUR_N_STROMAL_CELL																														
CELLMARKERCUR_N_SUBPALLIUM_CELL																														
CELLMARKERCUR_N_SUBPLATE_CELL																														
CELLMARKERCUR_N_SUPPRESSIVE_MONOCYTE																														
CELLMARKERCUR_N_SUPPRESSOR_T_CELL																														
CELLMARKERCUR_N_SUPRABASAL_CELL																														
CELLMARKERCUR_N_SURFACE_ECTODERM_CELL																														
CELLMARKERCUR_N_SUSTENTACULAR_CELL																														
CELLMARKERCUR_N_SYMPATHOADRENAL_CELL																														
CELLMARKERCUR_N_SYNCYTIOTROPHOBLAST																														
CELLMARKERCUR_N_SYNTHETIC_SMOOTH_MUSCLE_C…																														
CELLMARKERCUR_N_T_CELL_LARGE_GRANULAR_LYM…																														
CELLMARKERCUR_N_T_CELL_LINEAGE																														
CELLMARKERCUR_N_T_HELPER_CELL																														
CELLMARKERCUR_N_T_MEMORY_STEM_CELL																														
CELLMARKERCUR_N_T2_MZP_REGULATORY_B_CELL																														
CELLMARKERCUR_N_TENDON_CELL																														
CELLMARKERCUR_N_TERMINAL_DIFFERENTIATED_B…																														
CELLMARKERCUR_N_THALAMIC_CELL																														
CELLMARKERCUR_N_THECA_INTERNA_CELL																														
CELLMARKERCUR_N_THYMIC_EPITHELIAL_CELL																														
CELLMARKERCUR_N_TISSUE_LIKE_MEMORY_B_CELL																														
CELLMARKERCUR_N_TISSUE_RESIDENT_CELL																														
CELLMARKERCUR_N_TISSUE_RESIDENT_EFFECTOR_…																														
CELLMARKERCUR_N_TISSUE_RESIDENT_MACROPHAGE																														
CELLMARKERCUR_N_TISSUE_RESIDENT_MEMORY_LI…																														
CELLMARKERCUR_N_TOLDC_INDUCED_REGULATORY_…																														
CELLMARKERCUR_N_TONSIL_CELL																														
CELLMARKERCUR_N_TRABECULAR_MESHWORK_CELL																														
CELLMARKERCUR_N_TRANSITIONAL_B_CELL																														
CELLMARKERCUR_N_TRANSITIONAL_DENDRITIC_CE…																														
CELLMARKERCUR_N_TRUNCATED_RADIAL_GLIAL_CE…																														
CELLMARKERCUR_N_TUBULAR_CELL																														
CELLMARKERCUR_N_TUMOR_ASSOCIATED_MICROGLI…																														
CELLMARKERCUR_N_TUMOR_ENDOTHELIAL_CELL																														
CELLMARKERCUR_N_TUMOR_EPITHELIAL_CELL																														
CELLMARKERCUR_N_TUMOR_INFILTRATING_REGULA…																														
CELLMARKERCUR_N_TUMOR_INFILTRATING_T_CELL																														
CELLMARKERCUR_N_TUMOR_INITIATING_CELL																														
CELLMARKERCUR_N_TUMOR_REGULATORY_T_CELL																														

CELLMARKERCAN_ACINAR_CELLS																														
CELLMARKERCUR_N_UMBRELLA_CELL																														
CELLMARKERCUR_N_UNRESTRICTED_SOMATIC_STEM…																														
CELLMARKERCUR_N_UNSWITCHED_PLASMABLAST																														
CELLMARKERCUR_N_URETERIC_BUD_CELL																														
CELLMARKERCUR_N_UROTHELIAL_CELL																														
CELLMARKERCUR_N_UROTHELIUM_CELL																														
CELLMARKERCUR_N_VASCULATURE_CELL																														
CELLMARKERCUR_N_VEIN_CELL																														
CELLMARKERCUR_N_VENOUS_CELL																														
CELLMARKERCUR_N_VENTRAL_LATERAL_GANGLIONI…																														
CELLMARKERCUR_N_VENTRAL_MEDIAL_GANGLIONIC…																														
CELLMARKERCUR_N_VENTRICULAR_CELL																														
CELLMARKERCUR_N_YOUNG_NEURON																														
CELLMARKERCUR_N_ZONA_FASCICULATA_CELL																														
CELLMARKERCUR_N_ZONA_GLOMERULOSA_CELL																														
CELLMARKERCUR_N_ZONA_RETICULARIS_CELL																														
CELLMARKERCUR_N_ZYGOTE																														
CELLMARKERCURNACTIVATEDEFFECTORMEMORYCD4T…																														
CELLMARKERCURNACTIVATEDTISSUERESIDENTMEMO…																														
CELLMARKERCURNACTIVATEDTISSUERESIDENTMEMO…																														
CELLMARKERCURNALPHACELLACELL																														
CELLMARKERCURNALVEOLARTYPEIIATIICELL																														
CELLMARKERCURNATYPICALBCELLABC																														
CELLMARKERCURNAXLCD11CPLASMACYTOIDDENDRIT…																														
CELLMARKERCURNAXLCD123PLASMACYTOIDDENDRIT…																														
CELLMARKERCURNBASALFOREBRAINCHOLINERGICNE…																														
CELLMARKERCURNBRUSHCELLTUFTCELL																														
CELLMARKERCURNCANCERASSOCIATEDFIBROBLASTC…																														
CELLMARKERCURNCAPSULARFIBROBLASTFB2																														
CELLMARKERCURNCCR7DENDRITICCELL																														
CELLMARKERCURNCD106MESENCHYMALSTEMCELL																														
CELLMARKERCURNCD14MACROPHAGE																														
CELLMARKERCURNCD16DENDRITICCELL																														
CELLMARKERCURNCD25LAG3TCELL																														
CELLMARKERCURNCD34GRANULOCYTICPROGENITORC…																														
CELLMARKERCURNCD34HEMATOPOIETICSTEMCELL																														
CELLMARKERCURNCD34PREBCELL																														
CELLMARKERCURNCD34PROGENITORCELL																														
CELLMARKERCURNCD34THYMOCYTE																														
CELLMARKERCURNCD40LGTHELPERCELL																														
CELLMARKERCURNCD4CD25REGULATORYTCELL																														
CELLMARKERCURNCD4CENTRALMEMORYLIKETTCMLIK…																														
CELLMARKERCURNCD4CYTOTOXICT1CELL																														
CELLMARKERCURNCD4CYTOTOXICT2CELL																														
CELLMARKERCURNCD4THELPERCELL																														
CELLMARKERCURNCHRONICALLYACTIVATEDCD4TCELL																														
CELLMARKERCURNCHRONICALLYACTIVATEDCD8TCELL																														
CELLMARKERCURNCLASSSWITCHEDMEMORYBIGGU03B…																														
CELLMARKERCURNCLEC10ADENDRITICCELL																														
CELLMARKERCURNCLEC9ADENDRITICCELL																														
CELLMARKERCURNCONVENTIONALDENDRITICCELL2B…																														
CELLMARKERCURNCONVENTIONALDENDRITICCELL2B…																														
CELLMARKERCURNCORTICALSOMATOSTATINSSTINTE…																														
CELLMARKERCURNCYTOTOXICCD4T2CELL																														
CELLMARKERCURNCYTOTOXICCD4TCELL																														
CELLMARKERCURND1MSNMEDIUMSPINYNEURON																														
CELLMARKERCURND2MSNMEDIUMSPINYNEURON																														
CELLMARKERCURNDISTALTUBULELOOPOFHENLECELL																														
CELLMARKERCURNEARLYCD34MEGAKARYOCYTEERYTH…																														
CELLMARKERCURNEARLYTCELLPROGENITORETP																														
CELLMARKERCURNEFFECTORMEMORYCD4TCELL																														
CELLMARKERCURNEFFECTORREGULATORYTETREGCELL																														
CELLMARKERCURNEFFECTORTTEFFCELL																														
CELLMARKERCURNEXHAUSTEDCD4TCELL																														
CELLMARKERCURNEXHAUSTEDCD8TCELL																														
CELLMARKERCURNEXHAUSTEDCD8TPROGENITORCELL																														
CELLMARKERCURNEXHAUSTEDTTEXCELL																														
CELLMARKERCURNEXTRATELENCEPHALICETCELL																														
CELLMARKERCURNFINALLYHIGHLYEFFECTORTEMRAM…																														
CELLMARKERCURNFOLLICULARREGULATORYTTFRCELL																														
CELLMARKERCURNFOXP3NATURALREGULATORYTTREG…																														
CELLMARKERCURNGAMMADELTAU03B3DTCELL																														
CELLMARKERCURNGERMINALCENTREGCLIKECELL																														
CELLMARKERCURNGLOMERULARENDOTHELIALCELLECG																														
CELLMARKERCURNGRANULOCYTICMYELOIDDERIVEDS…																														
CELLMARKERCURNGRBREGULATORYBCELL																														
CELLMARKERCURNGZMKTCELL																														
CELLMARKERCURNHIGHLYDIFFERENTIATEDEFFECTO…																														
CELLMARKERCURNINDUCEDREGULATORYTTREGCELL																														
CELLMARKERCURNINFLAMMATORYCANCERASSOCIATE…																														
CELLMARKERCURNINNATELYMPHOIDCELLILCS																														
CELLMARKERCURNINTRATHYMICFIBROBLASTFB1																														
CELLMARKERCURNITOCELLHEPATICSTELLATECELL																														
CELLMARKERCURNLYMPHANGIOLEIOMYOMATOSISCEL…																														
CELLMARKERCURNMARGINALZONEMZLIKEBCELL																														
CELLMARKERCURNMEMORYSTEMCELLTSCM																														
CELLMARKERCURNMICROGLIADERIVEDTUMORASSOCI…																														
CELLMARKERCURNMKERYTHCELL																														
CELLMARKERCURNMYOFIBROBLASTICCANCERASSOCI…																														
CELLMARKERCURNNAIVEREGULATORYTTREGCELL																														
CELLMARKERCURNNATURALREGULATORYTTREGCELL																														
CELLMARKERCURNPERIPHERALBLOODMONONUCLEARC…																														
CELLMARKERCURNPITUITARYSTEMCELLPSC																														
CELLMARKERCURNPLASMACYTOIDDENDRITICCELLPDC																														
CELLMARKERCURNPOLYMORPHONUCLEARMYELOIDDER…																														
CELLMARKERCURNPOLYMORPHONUCLEARNEUTROPHIL…																														
CELLMARKERCURNPOSTERIORPRESOMITICMESODERM…																														
CELLMARKERCURNPROLIFERATIVECD4TCELL																														
CELLMARKERCURNPROLIFERATIVECD8TCELL																														
CELLMARKERCURNREDBLOODCELLERYTHROCYTE																														
CELLMARKERCURNREGULATORYBBREGCELL																														
CELLMARKERCURNREGULATORYCD4TCELL																														
CELLMARKERCURNREGULATORYCD8TCELL																														
CELLMARKERCURNREGULATORYTTREGCELL																														

CELLMARKERCAN_ACINAR_CELLS																														
CELLMARKERCURNREGULATORYTTREGCELL																														
CELLMARKERCURNRENALVESICLECOMMASHAPEDBODY…																														
CELLMARKERCURNRESIDENTMEMORYCD8TCELL																														
CELLMARKERCURNREVERTANTMEMORYCD8TTEMRACELL																														
CELLMARKERCURNSCARASSOCIATEDMACROPHAGESAM																														
CELLMARKERCURNSENESCENTCD8TCELL																														
CELLMARKERCURNSTEMPROGENITORCELL																														
CELLMARKERCURNSWITCHEDMEMORYBCELLSWMB																														
CELLMARKERCURNT1TRANSITIONALBCELL																														
CELLMARKERCURNT2TRANSITIONALBCELL																														
CELLMARKERCURNTCELLNATURALKILLERCELL																														
CELLMARKERCURNTERMINALEFFECTORCD8TCELL																														
CELLMARKERCURNTERMINALLYDIFFERENTIATEDCD8…																														
CELLMARKERCURNTFOLLICULARHELPERTFHCELL																														
CELLMARKERCURNTHELPER1TH1CELL																														
CELLMARKERCURNTHELPER1TH1CELL																														
CELLMARKERCURNTHELPER2TH2CELL																														
CELLMARKERCURNTHELPER2TH2CELL																														
CELLMARKERCURNTHELPER9TH9CELL																														
CELLMARKERCURNTHELPERTHCELL																														
CELLMARKERCURNTHYMICEPITHELIALCELLTEC																														
CELLMARKERCURNTISSUERESIDENTMEMORYCD4TCELL																														
CELLMARKERCURNTISSUERESIDENTMEMORYCD8TCELL																														
CELLMARKERCURNTISSUERESIDENTMEMORYTTRMCELL																														
CELLMARKERCURNTPERIPHERALHELPERTPHCELL																														
CELLMARKERCURNTRANSIENTAMPLIFYINGTACELL																														
CELLMARKERCURNTRANSITIONALEXHAUSTEDCD8TCE…																														
CELLMARKERCURNTUMORINFILTRATINGLYMPHOCYTE…																														
CELLMARKERCURNUNSWITCHEDMEMORYBCELLUNSWMB																														
CELLMARKERCURNVASARECTAENDOTHELIALCELLECVR																														
CELLMARKERCURNVONECONOMONEURONVEN																														
CELLMARKERCURNYOLKSACDERIVEDMYELOIDBIASED…																														
CELLMARKEREXP_ACINAR_CELLS																														
CELLMARKEREXP_ADRENERGIC_NEURONS																														
CELLMARKEREXP_AIRWAY_EPITHELIAL_CELLS																														
CELLMARKEREXP_AIRWAY_SMOOTH_MUSCLE_CELLS																														
CELLMARKEREXP_ALPHA_CELLS																														
CELLMARKEREXP_ANTERIOR_PITUITARY_GLAND_CE…																														
CELLMARKEREXP_ASTROCYTES																														
CELLMARKEREXP_B_CELLS																														
CELLMARKEREXP_B_CELLS_MEMORY																														
CELLMARKEREXP_B_CELLS_NAIVE																														
CELLMARKEREXP_BASAL_CELLS																														
CELLMARKEREXP_BASOPHILS																														
CELLMARKEREXP_BERGMANN_GLIA																														
CELLMARKEREXP_BETA_CELLS																														
CELLMARKEREXP_CAJAL_RETZIUS_CELLS																														
CELLMARKEREXP_CHOLANGIOCYTES																														
CELLMARKEREXP_CHOLINERGIC_NEURONS																														
CELLMARKEREXP_CHONDROCYTES																														
CELLMARKEREXP_DELTA_CELLS																														
CELLMARKEREXP_DISTAL_TUBULE_CELLS																														
CELLMARKEREXP_DUCTAL_CELLS																														
CELLMARKEREXP_EMBRYONIC_STEM_CELLS																														
CELLMARKEREXP_ENTERIC_NEURONS																														
CELLMARKEREXP_ENTEROENDOCRINE_CELLS																														
CELLMARKEREXP_EPENDYMAL_CELLS																														
CELLMARKEREXP_EPSILON_CELLS																														
CELLMARKEREXP_ERYTHROID_LIKE_AND_ERYTHROI…																														
CELLMARKEREXP_GAMMA_DELTA_T_CELLS																														
CELLMARKEREXP_GOBLET_CELLS																														
CELLMARKEREXP_HEMANGIOBLASTS																														
CELLMARKEREXP_HEMATOPOIETIC_STEM_CELLS																														
CELLMARKEREXP_HEPATIC_STELLATE_CELLS																														
CELLMARKEREXP_HIS_BUNDLE_CELLS																														
CELLMARKEREXP_IMMATURE_NEURONS																														
CELLMARKEREXP_JUXTAGLOMERULAR_CELLS																														
CELLMARKEREXP_KERATINOCYTES																														
CELLMARKEREXP_KIDNEY_PROGENITOR_CELLS																														
CELLMARKEREXP_KUPFFER_CELLS																														
CELLMARKEREXP_LEYDIG_CELLS																														
CELLMARKEREXP_LOOP_OF_HENLE_CELLS																														
CELLMARKEREXP_LUMINAL_EPITHELIAL_CELLS																														
CELLMARKEREXP_MAMMARY_EPITHELIAL_CELLS																														
CELLMARKEREXP_MEGAKARYOCYTES																														
CELLMARKEREXP_MELANOCYTES																														
CELLMARKEREXP_MESANGIAL_CELLS																														
CELLMARKEREXP_MESOTHELIAL_CELLS																														
CELLMARKEREXP_MICROFOLD_CELLS																														
CELLMARKEREXP_MICROGLIA																														
CELLMARKEREXP_MONOCYTES																														
CELLMARKEREXP_MULLER_CELLS																														
CELLMARKEREXP_MYOBLASTS																														
CELLMARKEREXP_MYOCYTES																														
CELLMARKEREXP_MYOFIBROBLASTS																														
CELLMARKEREXP_NEUROBLASTS																														
CELLMARKEREXP_NEUTROPHILS																														
CELLMARKEREXP_NK_CELLS																														
CELLMARKEREXP_NORADRENERGIC_NEURONS																														
CELLMARKEREXP_OLIGODENDROCYTES																														
CELLMARKEREXP_OSTEOBLASTS																														
CELLMARKEREXP_OSTEOCLAST_PRECURSOR_CELLS																														
CELLMARKEREXP_OSTEOCLASTS																														
CELLMARKEREXP_OSTEOCYTES																														
CELLMARKEREXP_PANETH_CELLS																														
CELLMARKEREXP_PARATHYROID_CHIEF_CELLS																														
CELLMARKEREXP_PERICYTES																														
CELLMARKEREXP_PHOTORECEPTOR_CELLS																														
CELLMARKEREXP_PLASMA_CELLS																														
CELLMARKEREXP_PLASMACYTOID_DENDRITIC_CELLS																														
CELLMARKEREXP_PODOCYTES																														
CELLMARKEREXP_PRINCIPAL_CELLS																														
CELLMARKEREXP_PROXIMAL_TUBULE_CELLS																														
CELLMARKEREXP_PULMONARY_ALVEOLAR_TYPE_I_C…																														

CELLMARKERCAN_ACINAR_CELLS																														
CELLMARKEREXP_PULMONARY_ALVEOLAR_TYPE_II_…																														
CELLMARKEREXP_PURKINJE_FIBER_CELLS																														
CELLMARKEREXP_PURKINJE_NEURONS																														
CELLMARKEREXP_PYRAMIDAL_CELLS																														
CELLMARKEREXP_RETICULOCYTES																														
CELLMARKEREXP_RETINAL_GANGLION_CELLS																														
CELLMARKEREXP_SALIVARY_MUCOUS_CELLS																														
CELLMARKEREXP_SATELLITE_CELLS																														
CELLMARKEREXP_SCHWANN_CELLS																														
CELLMARKEREXP_SEROTONERGIC_NEURONS																														
CELLMARKEREXP_SERTOLI_CELLS																														
CELLMARKEREXP_SMOOTH_MUSCLE_CELLS																														
CELLMARKEREXP_SPERMATOZOA																														
CELLMARKEREXP_STROMAL_CELLS																														
CELLMARKEREXP_T_CELLS																														
CELLMARKEREXP_T_CELLS_NAIVE																														
CELLMARKEREXP_T_CYTOTOXIC_CELLS																														
CELLMARKEREXP_T_HELPER_CELLS																														
CELLMARKEREXP_T_MEMORY_CELLS																														
CELLMARKEREXP_TRICHOCYTES																														
CELLMARKEREXP_TRIGEMINAL_NEURONS																														
CELLMARKEREXP_TROPHOBLAST_CELLS																														
CELLMARKEREXP_TROPHOBLAST_PROGENITOR_CELLS																														
CELLMARKEREXP_TUFT_CELLS																														
CELLMARKEREXP_VASCULAR_SMOOTH_MUSCLE_CELLS																														
CELLMARKEREXPENDOTHELIALCELLSAORTA																														
CELLMARKEREXPNEURALSTEMPRECURSORCELLS																														
CELLMARKERMSIGDB_AIZARANI_LIVER_C12_NK_NK…																														
CELLMARKERMSIGDB_AIZARANI_LIVER_C22_RESID…																														
CELLMARKERMSIGDB_AIZARANI_LIVER_C28_NK_NK…																														
CELLMARKERMSIGDB_AIZARANI_LIVER_C29_MVECS…	ST6GA…	GSN	ECE1	KCNN3	IFI6	NCOA7	CYYR1	DSTN	MAST4	TSPAN…	AFF1	TMEM47	IGFBP4	PLSCR4	SASH1	ACKR3	OAZ2	CTNNA…	SKI	PTGDS	RHOJ	AVPI1	TSC22…	TCF7L1	ADGRG1	ARHGA…	ADGRF5	TRIOBP	UACA	PTPN14
CELLMARKERMSIGDB_AIZARANI_LIVER_C30_HEPAT…	CCNL2	PABPC…	NEDD9	FBXO17	PCLAF	CCT8	RUNDC1	BAAT	SLC25…	PTAFR	FAM98B	GRTP1	PPP6R2	C5AR2	SDS	FAXC	SEC23A	RNF216	MKLN1…	CAPN3	ONECU…	PDE4D…	ABCA5	SCML1	RN7SL…	IDS	TMEM1…	SGMS1…	RAMP2…	BDH1
CELLMARKERMSIGDB_AIZARANI_LIVER_C8_RESIDE…																														
CELLMARKERMSIGDB_AIZARANI_LIVER_C9_LSECS_1	GARRE1	CDC37	SNRK	SREK1	C5AR2	WSB1	FRMD8	ZEB1	CCDC50	TRIOBP	DDX21	RDX	NASP	PELI1	ARGLU1	LUZP1	AFF4	PEA15	BNIP3L	ABCG1	TCF7L2	MAP3K6	OSMR	TCF7L1	EPOR	SOGA1	NPDC1	PRICK…	AHCTF1	CNTNA…
CELLMARKERMSIGDB_BUSSLINGER_DUODENAL_BCHE…																														
CELLMARKERMSIGDB_BUSSLINGER_DUODENAL_DIFF…	SLC25…	EIF3K	COX5B	RPL29	ATP5M…	MGST1	CYCS	RPL28	ONECU…	NFIB	TM9SF3	YWHAZ	COX7B	LGALS4	NDUFB9	ZFAS1	CLDN3	ATP5I…	ATP5PO	RPL17	HNRNPK	SERF2	KRT18	PABPC1	ALDH2	SOD1	EIF4B	RPS27	SFPQ	EIF1AX
CELLMARKERMSIGDB_BUSSLINGER_DUODENAL_EC_C…																														
CELLMARKERMSIGDB_BUSSLINGER_DUODENAL_IMMU…	ESYT1	RAP1B	STK4	CD84	MLLT6	PTK2B	RNF166	FAM89B	GATA3	RPLP1	PVRIG	RGS19	XPO6	RBL2	JAK1	RPLP2	SH2D2A	KLF13	MYO9B	GNPTAB	CXCR3	SMAP2	EVI2A	GZMM	GPR82	SLC2A3	RPS16	TRAF3…	RASGR…	RPL4
CELLMARKERMSIGDB_BUSSLINGER_DUODENAL_STEM…	TOMM7	EML4	RPS27	RPL18A	GCNT1	SCARN…	RPS2	ATRX	RPS16	RPLP1	HNRNPK	RAD21	NONO	NFIB	PPIA	NSA2	FAM12…	TTC3	MT1G	MATR3	JUN	SERP1	YWHAE	ARF4	CLDN3	CANX	BCLAF1	EIF4A2	EEF2	RPS20
CELLMARKERMSIGDB_BUSSLINGER_DUODENAL_TUFT…																														
CELLMARKERMSIGDB_BUSSLINGER_ESOPHAGEAL_EA…																														
CELLMARKERMSIGDB_BUSSLINGER_ESOPHAGEAL_PR…																														
CELLMARKERMSIGDB_BUSSLINGER_GASTRIC_CHIEF…																														
CELLMARKERMSIGDB_BUSSLINGER_GASTRIC_G_CEL…																														
CELLMARKERMSIGDB_BUSSLINGER_GASTRIC_ISTHM…	PSMA7	ELF3	SLTM	OSBPL7	TACST…	CENPV	H2AJ	FMN1	INF2	ATP2A3	ACTG1	PSMD7	ADAM15	NIBAN2	CAPNS1	H4C3	SERPI…	CAPG	TUBB4B	CSTB	VILL	PYCR1	LFNG	PAQR8	ATP5I…	GMDS	PFN1	SF3B5	FASN	SGSM3
CELLMARKERMSIGDB_BUSSLINGER_GASTRIC_NECK_…																														
CELLMARKERMSIGDB_BUSSLINGER_GASTRIC_OXYNT…																														
CELLMARKERMSIGDB_BUSSLINGER_GASTRIC_PREZY…																														
CELLMARKERMSIGDB_BUSSLINGER_GASTRIC_REG3A…																														
CELLMARKERMSIGDB_CUI_DEVELOPING_HEART_5TH…																														
CELLMARKERMSIGDB_CUI_DEVELOPING_HEART_5TH…																														
CELLMARKERMSIGDB_CUI_DEVELOPING_HEART_C1_…																														
CELLMARKERMSIGDB_CUI_DEVELOPING_HEART_C2_…																														
CELLMARKERMSIGDB_CUI_DEVELOPING_HEART_COM…																														
CELLMARKERMSIGDB_CUI_DEVELOPING_HEART_COR…																														
CELLMARKERMSIGDB_CUI_DEVELOPING_HEART_END…																														
CELLMARKERMSIGDB_CUI_DEVELOPING_HEART_LEF…																														
CELLMARKERMSIGDB_CUI_DEVELOPING_HEART_RIG…																														
CELLMARKERMSIGDB_CUI_DEVELOPING_HEART_SMO…																														
CELLMARKERMSIGDB_DESCARTES_FETAL_ADRENAL_…																														
CELLMARKERMSIGDB_DESCARTES_FETAL_CEREBELL…																														
CELLMARKERMSIGDB_DESCARTES_FETAL_CEREBELL…																														
CELLMARKERMSIGDB_DESCARTES_FETAL_CEREBELL…	LGALS3	ARRB2	PRKCD	RENBP	ENSG0…	TAGAP	LINC0…	PLCB2	CEACA…	TMEM2…	ARHGA…	PLA2G…	ATP6V…	OSBPL…	RGL3	NFKBIZ	SUCNR1	ADA2	SMIM35	ARID5A	HSD17…	LINC0…	C3AR1	DHRS9	HCLS1	IL18	RASAL3	C5AR1	MAN2B1	DPP7
CELLMARKERMSIGDB_DESCARTES_FETAL_CEREBELL…																														
CELLMARKERMSIGDB_DESCARTES_FETAL_CEREBELL…																														
CELLMARKERMSIGDB_DESCARTES_FETAL_CEREBELL…																														
CELLMARKERMSIGDB_DESCARTES_FETAL_CEREBELL…	APCDD1	C4orf3	INF2	MCAM	CRIP2	HES1	KLF13	MYO1C	IQGAP2	COL4A2	ARHGA…	COBLL1	HEG1	LATS2	SHANK3	RGS3	PLOD1	AHNAK	GPD1L	PINK1	FILIP1	CDC42…	LINC0…	LINC0…	FHL3	CARMN	PROCR	GBP4	ARHGE…	KLF4
CELLMARKERMSIGDB_DESCARTES_FETAL_CEREBRUM…																														
CELLMARKERMSIGDB_DESCARTES_FETAL_CEREBRUM…																														
CELLMARKERMSIGDB_DESCARTES_FETAL_CEREBRUM…	LIG4	MSH2-…	TECR	DHRS12	PELO-…	LINC0…	DLG4	LRRC71	HHLA1	MRTO4	GTPBP2	WNT11	HAUS7	PPP1R…	FUNDC1	C1orf…	HYAL3	FOLR1	CALHM6	TEPSIN	ABCB6	L3MBT…	CAPN1…	CCER2	ADAD2	LIPH	OXTR	GRAP2	CENPH	H3C4
CELLMARKERMSIGDB_DESCARTES_FETAL_CEREBRUM…	RN7SL…	CRYBB1	TNFSF…	ADRB2	LGALS3	GBGT1	OAS1	ARRDC5	SIGLE…	PCED1…	CLEC1…	RASGR…	FGF20	BLVRB	EGR2	TRIM22	ACSM5	LNCAR…	ACY3	TMCO4	IRF5	KPNA2…	RN7SL…	NAIP	GPN3	SHISA…	CD33	DPRXP4	ENSG0…	LINC0…
CELLMARKERMSIGDB_DESCARTES_FETAL_CEREBRUM…																														
CELLMARKERMSIGDB_DESCARTES_FETAL_CEREBRUM…																														
CELLMARKERMSIGDB_DESCARTES_FETAL_CEREBRUM…	CXCL12	ITPR3	QPCT	SYDE1	SPINK8	CLEC1A	JAG2	MYLK2	NHSL2	SYNM	ISM1	PRND	FZD6	GATA2	ZNF703	FZD4	MXRA5	EIF3I	XRCC6…	EOGT	RRAGA	SNRPB	PTGDR2	DYSF	FOXF1	ENSG0…	TNKS1…	BTNL9	S100A…	VWA2
CELLMARKERMSIGDB_DESCARTES_FETAL_EYE_ASTR…																														
CELLMARKERMSIGDB_DESCARTES_FETAL_EYE_GANG…																														
CELLMARKERMSIGDB_DESCARTES_FETAL_EYE_LENS…																														
CELLMARKERMSIGDB_DESCARTES_FETAL_EYE_PHOT…																														
CELLMARKERMSIGDB_DESCARTES_FETAL_EYE_RETI…																														
CELLMARKERMSIGDB_DESCARTES_FETAL_EYE_SMOO…																														
CELLMARKERMSIGDB_DESCARTES_FETAL_EYE_STRO…																														
CELLMARKERMSIGDB_DESCARTES_FETAL_EYE_VASC…																														
CELLMARKERMSIGDB_DESCARTES_FETAL_HEART_EL…																														
CELLMARKERMSIGDB_DESCARTES_FETAL_HEART_EN…																														
CELLMARKERMSIGDB_DESCARTES_FETAL_HEART_LY…																														
CELLMARKERMSIGDB_DESCARTES_FETAL_HEART_SC…																														
CELLMARKERMSIGDB_DESCARTES_FETAL_HEART_SM…																														
CELLMARKERMSIGDB_DESCARTES_FETAL_HEART_ST…																														
CELLMARKERMSIGDB_DESCARTES_FETAL_HEART_VI…																														
CELLMARKERMSIGDB_DESCARTES_FETAL_INTESTIN…																														
CELLMARKERMSIGDB_DESCARTES_FETAL_INTESTIN…																														
CELLMARKERMSIGDB_DESCARTES_FETAL_INTESTIN…																														
CELLMARKERMSIGDB_DESCARTES_FETAL_INTESTIN…																														
CELLMARKERMSIGDB_DESCARTES_FETAL_INTESTIN…																														
CELLMARKERMSIGDB_DESCARTES_FETAL_INTESTIN…																														
CELLMARKERMSIGDB_DESCARTES_FETAL_KIDNEY_M…																														
CELLMARKERMSIGDB_DESCARTES_FETAL_KIDNEY_U…	SLC30…	ENSG0…	MAB21…	TEPP	LY6K	TMEM1…	GCNT4	SPR	CYP4F…	UGT1A6	MIR20…	CYP4Z…	HR	RAB17…	EDN2	LINC0…	EVPLL	SMIM6	FAM16…	CAPNS2	AVPR1A	ANKRD…	COSMOC	LY6D	MIR20…	ENSG0…	LEP	COL17…	RNF20…	MYO3B…
CELLMARKERMSIGDB_DESCARTES_FETAL_LIVER_ER…																														
CELLMARKERMSIGDB_DESCARTES_FETAL_LIVER_HE…																														
CELLMARKERMSIGDB_DESCARTES_FETAL_LIVER_HE…	GLT1D1	SC5D	HGFAC	TTC6	DBI	DGAT2	DIO1	ONECU…	CDO1	SLC17…	CLDN14	ABCC6…	UGT2B…	MIR12…	HJV	STEAP2	PPP1R…	ANG	LINC0…	GLYAT…	APOC4	PROX1…	SLC16…	TMEM1…	ACOX2	CRYZ	AFM	AADAC	SLC47…	ALDH8…
CELLMARKERMSIGDB_DESCARTES_FETAL_LIVER_ME…	PEX5L	NXNL2	LRRTM3	PNMA8A	PDGFRL	LINC0…	FRMD6…	LONRF2	VSTM2A	LINC0…	SMOC2	FAHD2B	GRIA4	FGF18	LRRIQ1	TTLL9	AMIGO1	CASC20	GRIN3A	PHLDA3	EPHA6	HSPB2	PRR15…	ADGRB2	INHBA	CNGA4	DCT	NCR3L…	TMEM1…	LINC0…
CELLMARKERMSIGDB_DESCARTES_FETAL_LUNG_BRO…																														
CELLMARKERMSIGDB_DESCARTES_FETAL_LUNG_CIL…	SPEF1	PRR29	NME9	EYA1	SYNPR…	DOC2A	RUVBL2	TCTN2	NEK2-…	TUBA4B	CCDC65	ASB14	HSPBP1	ODAD3	DNAL1	DYDC2	LRRC9	CCDC13	LRRC71	RABL2A	CLUAP1	IFT22	DCDC2B	RIBC1	USP2-…	THBS3…	FLACC1	FAM15…	MDH1B	PIH1D2
CELLMARKERMSIGDB_DESCARTES_FETAL_LUNG_LYM…																														

CELLMARKERCAN_ACINAR_CELLS																														
CELLMARKERMSIGDB_DESCARTES_FETAL_LUNG_NEU…																														
CELLMARKERMSIGDB_DESCARTES_FETAL_LUNG_STR…																														
CELLMARKERMSIGDB_DESCARTES_FETAL_LUNG_VAS…																														
CELLMARKERMSIGDB_DESCARTES_FETAL_MUSCLE_L…																														
CELLMARKERMSIGDB_DESCARTES_FETAL_MUSCLE_S…																														
CELLMARKERMSIGDB_DESCARTES_FETAL_MUSCLE_S…																														
CELLMARKERMSIGDB_DESCARTES_FETAL_MUSCLE_V…																														
CELLMARKERMSIGDB_DESCARTES_FETAL_PANCREAS…																														
CELLMARKERMSIGDB_DESCARTES_FETAL_PANCREAS…																														
CELLMARKERMSIGDB_DESCARTES_FETAL_PANCREAS…																														
CELLMARKERMSIGDB_DESCARTES_FETAL_PANCREAS…																														
CELLMARKERMSIGDB_DESCARTES_FETAL_PANCREAS…																														
CELLMARKERMSIGDB_DESCARTES_FETAL_PANCREAS…																														
CELLMARKERMSIGDB_DESCARTES_FETAL_PANCREAS…																														
CELLMARKERMSIGDB_DESCARTES_FETAL_PLACENTA…																														
CELLMARKERMSIGDB_DESCARTES_FETAL_PLACENTA…																														
CELLMARKERMSIGDB_DESCARTES_FETAL_PLACENTA…																														
CELLMARKERMSIGDB_DESCARTES_FETAL_PLACENTA…																														
CELLMARKERMSIGDB_DESCARTES_FETAL_PLACENTA…																														
CELLMARKERMSIGDB_DESCARTES_FETAL_SPLEEN_E…																														
CELLMARKERMSIGDB_DESCARTES_FETAL_SPLEEN_S…																														
CELLMARKERMSIGDB_DESCARTES_FETAL_SPLEEN_V…																														
CELLMARKERMSIGDB_DESCARTES_FETAL_STOMACH_…	MAT1A	LRRC73	BBS5	ODAD3	FAM17…	ATG9B	CCDC96	CATIP	PPP1R…	RRAD	THBS3…	MDH1B	LRRC51	PPP1R…	CFAP1…	PIH1D2	RAB36	KIF23…	NEK2-…	CCDC1…	TEKT2	DDX3I…	C10or…	C17or…	C22or…	SVOPL	RWDD3…	PLEKH…	DYNLT4	CDKN2…
CELLMARKERMSIGDB_DESCARTES_FETAL_STOMACH_…																														
CELLMARKERMSIGDB_DESCARTES_FETAL_STOMACH_…																														
CELLMARKERMSIGDB_DESCARTES_FETAL_STOMACH_…																														
CELLMARKERMSIGDB_DESCARTES_FETAL_STOMACH_…																														
CELLMARKERMSIGDB_DESCARTES_FETAL_STOMACH_…																														
CELLMARKERMSIGDB_DESCARTES_FETAL_STOMACH_…																														
CELLMARKERMSIGDB_DESCARTES_FETAL_STOMACH_…																														
CELLMARKERMSIGDB_DESCARTES_FETAL_STOMACH_…																														
CELLMARKERMSIGDB_DESCARTES_FETAL_STOMACH_…																														
CELLMARKERMSIGDB_DESCARTES_FETAL_STOMACH_…																														
CELLMARKERMSIGDB_DESCARTES_FETAL_STOMACH_…																														
CELLMARKERMSIGDB_DESCARTES_FETAL_STOMACH_…																														
CELLMARKERMSIGDB_DESCARTES_FETAL_THYMUS_T…																														
CELLMARKERMSIGDB_DESCARTES_MAIN_FETAL_ACI…																														
CELLMARKERMSIGDB_DESCARTES_MAIN_FETAL_ADR…																														
CELLMARKERMSIGDB_DESCARTES_MAIN_FETAL_AST…																														
CELLMARKERMSIGDB_DESCARTES_MAIN_FETAL_BIP…																														
CELLMARKERMSIGDB_DESCARTES_MAIN_FETAL_BRO…																														
CELLMARKERMSIGDB_DESCARTES_MAIN_FETAL_CAR…																														
CELLMARKERMSIGDB_DESCARTES_MAIN_FETAL_CIL…	HMGN3	LCA5	GJB7	CFAP2…	ARMC2	PPIL6	SMPD2	AK9	FAM22…	RSPH4A	VNN3P	AHI1-…	ECT2L	ADGB	CCDC1…	DYNLT1	RSPH3	PACRG	PACRG…	C6orf…	PRR18	IQCE	RSPH1…	RSPH1…	ENSG0…	AGR3	RAD17…	ITGB8…	DNAH11	MINDY4
CELLMARKERMSIGDB_DESCARTES_MAIN_FETAL_DUC…																														
CELLMARKERMSIGDB_DESCARTES_MAIN_FETAL_END…																														
CELLMARKERMSIGDB_DESCARTES_MAIN_FETAL_ENS…																														
CELLMARKERMSIGDB_DESCARTES_MAIN_FETAL_EPI…																														
CELLMARKERMSIGDB_DESCARTES_MAIN_FETAL_GAN…																														
CELLMARKERMSIGDB_DESCARTES_MAIN_FETAL_GOB…																														
CELLMARKERMSIGDB_DESCARTES_MAIN_FETAL_HEM…																														
CELLMARKERMSIGDB_DESCARTES_MAIN_FETAL_HOR…																														
CELLMARKERMSIGDB_DESCARTES_MAIN_FETAL_INH…																														
CELLMARKERMSIGDB_DESCARTES_MAIN_FETAL_INH…																														
CELLMARKERMSIGDB_DESCARTES_MAIN_FETAL_INT…																														
CELLMARKERMSIGDB_DESCARTES_MAIN_FETAL_ISL…																														
CELLMARKERMSIGDB_DESCARTES_MAIN_FETAL_LYM…																														
CELLMARKERMSIGDB_DESCARTES_MAIN_FETAL_MES…																														
CELLMARKERMSIGDB_DESCARTES_MAIN_FETAL_MET…																														
CELLMARKERMSIGDB_DESCARTES_MAIN_FETAL_MYE…																														
CELLMARKERMSIGDB_DESCARTES_MAIN_FETAL_NEU…																														
CELLMARKERMSIGDB_DESCARTES_MAIN_FETAL_OLI…																														
CELLMARKERMSIGDB_DESCARTES_MAIN_FETAL_PDE…																														
CELLMARKERMSIGDB_DESCARTES_MAIN_FETAL_PDE…	CDAN1	LACTB	SLC24…	ARIH1	MAN2C1	ST20-…	CEMIP	NMB	HMGN1…	CHD2	CERS3	JMJD8	PKD1-…	BRICD5	MMP25…	ZNF205	TIGD7	CREBBP	ACSM3	RNU6-…	SMG1P5	CTF1	PDXDC…	DDX19B	IST1	PLCG2	RN7SK…	SPATA…	TCF25	RN7SL…
CELLMARKERMSIGDB_DESCARTES_MAIN_FETAL_RET…																														
CELLMARKERMSIGDB_DESCARTES_MAIN_FETAL_SAT…																														
CELLMARKERMSIGDB_DESCARTES_MAIN_FETAL_SCH…																														
CELLMARKERMSIGDB_DESCARTES_MAIN_FETAL_SLC…	CDIP1	ROGDI	TEKT5	NOMO2	SMG1P1	RABEP2	ZNF747	FBXL1…	ITFG1…	TENT4B	TRAPP…	VPS9D…	SPATA…	VMO1	DERL2	MED31	TVP23C	TRIM1…	SDF2	ERAL1	NSRP1	MIR47…	ENSG0…	CCT6B	LIG3	RAD51D	KLHL11	HROB	ATXN7…	RNU6-…
CELLMARKERMSIGDB_DESCARTES_MAIN_FETAL_SMO…																														
CELLMARKERMSIGDB_DESCARTES_MAIN_FETAL_STE…																														
CELLMARKERMSIGDB_DESCARTES_MAIN_FETAL_SYM…																														
CELLMARKERMSIGDB_DESCARTES_MAIN_FETAL_UNI…																														
CELLMARKERMSIGDB_DESCARTES_MAIN_FETAL_URE…																														
CELLMARKERMSIGDB_DESCARTES_MAIN_FETAL_VAS…																														
CELLMARKERMSIGDB_DESCARTES_MAIN_FETAL_VIS…																														
CELLMARKERMSIGDB_DURANTE_ADULT_OLFACTORY_…																														
CELLMARKERMSIGDB_DURANTE_ADULT_OLFACTORY_…																														
CELLMARKERMSIGDB_DURANTE_ADULT_OLFACTORY_…																														
CELLMARKERMSIGDB_DURANTE_ADULT_OLFACTORY_…																														
CELLMARKERMSIGDB_DURANTE_ADULT_OLFACTORY_…																														
CELLMARKERMSIGDB_DURANTE_ADULT_OLFACTORY_…																														
CELLMARKERMSIGDB_DURANTE_ADULT_OLFACTORY_…																														
CELLMARKERMSIGDB_DURANTE_ADULT_OLFACTORY_…																														
CELLMARKERMSIGDB_DURANTE_ADULT_OLFACTORY_…																														
CELLMARKERMSIGDB_DURANTE_ADULT_OLFACTORY_…																														
CELLMARKERMSIGDB_DURANTE_ADULT_OLFACTORY_…																														
CELLMARKERMSIGDB_DURANTE_ADULT_OLFACTORY_…																														
CELLMARKERMSIGDB_DURANTE_ADULT_OLFACTORY_…																														
CELLMARKERMSIGDB_DURANTE_ADULT_OLFACTORY_…																														
CELLMARKERMSIGDB_DURANTE_ADULT_OLFACTORY_…																														
CELLMARKERMSIGDB_DURANTE_ADULT_OLFACTORY_…																														
CELLMARKERMSIGDB_FAN_EMBRYONIC_CTX_ASTROC…																														
CELLMARKERMSIGDB_FAN_EMBRYONIC_CTX_BIG_GR…	HLA-E	SLC9A…	FHL3	TAGLN2	TPM4	TUBB6	IGFBP4	SCARB1	ARHGA…	TMEM1…	TNFAI…	PON2	EFEMP2	PMP22	EPSTI1	ABCG2	PCDH12	OAS2	ETS2	TSPAN9	ITGA5	SLC39…	GSDMD	NOTCH4	CPD	FLI1	PDGFB	FERMT2	MT2A	CD9
CELLMARKERMSIGDB_FAN_EMBRYONIC_CTX_BIG_GR…	TSPAN9	MECR	TPI1	CYP26…	CYB5R3	ST6GA…	KCNIP4	ATP6A…	ENO2	ATP13…	SEZ6L2	PHEX-…	ADM2	SELEN…	NIPAL3	SHISA2	C1orf…	FAM16…	ST6GA…	TPBG	PPL	POLR2L	UQCR11	PIN1	SLC25…	TMEM47	ATP6V…	S100A…	SNCA	TSPAN…
CELLMARKERMSIGDB_FAN_EMBRYONIC_CTX_BIG_GR…																														
CELLMARKERMSIGDB_FAN_EMBRYONIC_CTX_BIG_GR…	NCKAP…	APOE	RGS2	POLD4	AP1B1	GPX1	GIMAP4	NEAT1	C12or…	RHOB	SERPI…	PTPN6	MGST2	MIS18…	SOCS6	CD37	ENTPD1	VMP1	H2AJ	DDIT4	NFKBIA	GPSM3	HVCN1	EVI2A	ITPR2	STOM	CMTM6	TNFRS…	ADAM9	HSPA1B
CELLMARKERMSIGDB_FAN_EMBRYONIC_CTX_BRAIN_…	ADCY4	FRY	TMEM88	TIMP1	GBP4	FHL3	IGFBP2	PLXND1	TCF4	MLEC	BLCAP	PGRMC1	FOXC1	TLE1	SERIN…	CCDC1…	SMTN	MSRB3	CYB5A	KLHL5	ALPL	MECOM	PROM1	SESTD1	MFAP2	SLC1A4	KLF4	ISG15	SLC52…	LSR
CELLMARKERMSIGDB_FAN_EMBRYONIC_CTX_BRAIN_…	VSTM4	DOCK6	LRRC32	SNHG18	MYO6	FRMD6	CDC42…	ITGA4	PHACT…	GMDS	AKAP12	AASS	MPRIP	DPY19…	EPN2	ARHGA…	ECM2	CPPED1	LZTS1	AKIRI…	PLAGL1	GJA4	RNF24	ILK	JAK1	TNS1	ST3GA…	GALNT…	MYH9	ANGPT…
CELLMARKERMSIGDB_FAN_EMBRYONIC_CTX_EX_1_E…																														
CELLMARKERMSIGDB_FAN_EMBRYONIC_CTX_IN_2_I…																														
CELLMARKERMSIGDB_FAN_EMBRYONIC_CTX_IN_3_I…																														
CELLMARKERMSIGDB_FAN_EMBRYONIC_CTX_IN_5_I…																														
CELLMARKERMSIGDB_FAN_EMBRYONIC_CTX_IN_6_I…																														
CELLMARKERMSIGDB_FAN_EMBRYONIC_CTX_MICROG…																														
CELLMARKERMSIGDB_FAN_EMBRYONIC_CTX_OLIG	SPATA…	GIPC1	FMNL2	DEPTOR	MITF	TECPR2	FAM17…	PLD1	PKP4	PACS2	GDE1	PICALM	ZFYVE…	HSPA2	ELAVL3	LPGAT1	TJP2	BROX	CBR1	NOL4L	COL16…	AGPAT4	RALGDS	PPFIB…	PLEKH…	MAP7D1	PRIMA1	MOSPD2	APP	C12or…
CELLMARKERMSIGDB_FAN_EMBRYONIC_CTX_OPC																														

Table S5: Cell state marker gene list

CELLMARKERCAN_ACINAR_CELLS																														
CELLMARKERMSIGDB_FAN_OVARY_CL1_GPRC5A_TNF…	C11or…	TXNL1	MTDH	HSPH1	C1QBP	COL6A1	FUBP1	SAP18	UBL3	H2AX	NAA15	DNAJA2	XBP1	RNPS1	ZNF207	PSMD12	NASP	PPIL4	FUNDC2	LAMA4	RPL8	EIF3J	EFEMP1	EIF5B	RBBP6	NGDN	BST2	RPL7L1	RNF139	ATF1
CELLMARKERMSIGDB_FAN_OVARY_CL11_MURAL_GRA…	PHACT…	SLC25…	CLK1	DAPL1	PPFIB…	FST	HK1	TARS1	WASF1	NDP	MCL1	RIOK3	MED13L	SRSF2	FXR1	PDLIM1	POR	BEX2	PHF6	TRIB2	IGFBP2	ATP5M…	TNIP1	HSP90…	LAMC1	MT-CYB	RBBP6	DPYSL3	UBC	SNRPN
CELLMARKERMSIGDB_FAN_OVARY_CL13_MONOCYTE_…	ISG15	SDCBP	WARS1	THEMI…	AP2S1	HLA-A	GLIPR2	MAP2K3	CKLF	EVI2A	MYO9B	HSPA6	RAP1B	ZNF706	CAPZA1	ATP6V…	MAP2K1	COX7B	ACTR3	BLOC1…	ZFYVE…	TPP1	CDC42…	IL10RA	LIMD2	PGK1	GLA	RGS10	ATP6V…	PTP4A2
CELLMARKERMSIGDB_FAN_OVARY_CL14_MATURE_SM…	AP2M1	NDUFA1	ARL6I…	PDK4	HSPA1B	ROMO1	DBI	GSTP1	SERPI…	SPCS1	SEPTI…	CHMP5	UQCRQ	HMGB1	CIRBP	CALM3	TOMM7	ATP5M…	DNAJB1	PHPT1	APLP2	RSRP1	PARK7	TBCB	CAPZA2	ISCU	NDUFA5	RAB11B	REEP5	ID3
CELLMARKERMSIGDB_FAN_OVARY_CL15_SMALL_ANT…	DPM1	MOAP1	PTTG1	SKIL	JPT1	FHOD3	B3GNT2	GADD4…	ING3	TJP1	EFNB2	FDFT1	YBX3	TXNL1	SLC39…	ANKH	HSPA14	LAPTM…	ATF7I…	MT-CO3	OSER1	HDAC2	NDUFA4	ACIN1	MGST3	CDH2	DCTN6	SPATA…	MYO1B	SERTA…
CELLMARKERMSIGDB_FAN_OVARY_CL18_B_LYMPHOC…	ATP6V…	FKBP2	SPON2	DDX39A	ARPC4	RPS26	B4GAL…	NDUFV2	RPS11	VDAC3	JTB	ERP29	PABPC1	ACTR2	SEC13	SERP1	GTF2B	REPIN1	RHOG	ENSA	SUPT4…	ARPC5L	TACC1	PPP1CA	CAPZA1	DNAJA1	PTP4A2	ATP6V…	RBX1	ARPC1B
CELLMARKERMSIGDB_FAN_OVARY_CL2_PUTATIVE_E…																														
CELLMARKERMSIGDB_FAN_OVARY_CL5_HEALTHY_SE…	SLC38…	ITM2C	CSNK1…	RPS26	EIF4A3	PRKAR…	ARF6	CLK1	EEF2	LRRFI…	PFDN4	ATP6A…	RAB34	UBXN4	TLE4	SRSF11	MALSU1	TAX1B…	TPST1	SPTBN1	CTSK	ATP5M…	NUCKS1	LGALS1	EMP2	MYO6	PLD3	PPP2CA	MMP14	GCLM
CELLMARKERMSIGDB_FAN_OVARY_CL6_PUTATIVE_E…	CLK1	SERPI…	RHOB	SERPI…	HMGN2	BSG	FKBP8	CCNL1	RNH1	SMARC…	ADM	RPLP0	PCMTD1	ALDH9…	IMPDH2	PGRMC1	MORF4…	EIF3E	LAMP2	ERGIC2	CUTA	KLHDC2	TMEM5…	ILK	HTRA1	CAVIN1	MPHOS…	NFKBIA	TTC3	PDLIM3
CELLMARKERMSIGDB_FAN_OVARY_CL8_MATURE_CUM…	OCIAD2	VEGFA	RCN2	PET100	NAA38	CMTM3	CMTM8	NDUFC2	CHL1	TXNDC…	DYNLL1	NDUFA…	ACSL4	NDUFA4	FHL2	NDUFA…	GSS	PRDX4	COPS9	NBEAL1	ROMO1	ADCK2	TRMT1…	MRPL51	COX6B1	ENTPD6	ANAPC…	HINT2	RNASE1	SNRPF
CELLMARKERMSIGDB_FAN_OVARY_CL9_PUTATIVE_A…	SPAG9	ARL6I…	TPM3	ENY2	UBB	NOP16	BAG3	ENO1	SNU13	APP	GPX1	FTL	ARPC5L	SNRPB	NUDC	RAB11A	CEBPD	RAB7A	EIF4A1	TPT1	EIF2S2	CARHS…	EIF5A	SNX3	SRSF5	PPFIB…	MT-ND2	NME1	DNAJB6	ARGLU1
CELLMARKERMSIGDB_GAO_ESOPHAGUS_25W_C1_CIL…	LRIG1	LRRC1…	LRRC23	LRRC27	LRRC46	LRRC61	LRRC71	LRRC7…	LRRIQ1	LRWD1	LTBP1	LZTFL1	CFAP91	MAP9	MAPK15	MAPRE3	MARCH…	MAT1A	MB	MDH1B	MDM1	MIPEP…	MLF1	MLLT1	MLPH	MOB1B	MORN2	MORN3	MPDZ	MRLN
CELLMARKERMSIGDB_GAO_ESOPHAGUS_25W_C2_KRT…																														
CELLMARKERMSIGDB_GAO_ESOPHAGUS_25W_C3_FGF…																														
CELLMARKERMSIGDB_GAO_LARGE_INTESTINE_24W_…																														
CELLMARKERMSIGDB_GAO_LARGE_INTESTINE_24W_…																														
CELLMARKERMSIGDB_GAO_LARGE_INTESTINE_24W_…	RASGE…	RASSF5	RGS19	RHBDF2	RIC1	RILPL2	RIPK2	RNASE6	RNF14…	RSAD2	RUNX3	SAMSN1	SECTM1	SFMBT2	SGK1	SGPP1	SH2B3	SIGLE…	SIGLE…	SLA	SLAMF7	SLAMF8	SLC15…	SLC1A4	SLC23…	SLC25…	SLC35…	SLC37…	SLC7A5	SLC8B1
CELLMARKERMSIGDB_GAO_LARGE_INTESTINE_24W_…																														
CELLMARKERMSIGDB_GAO_LARGE_INTESTINE_24W_…																														
CELLMARKERMSIGDB_GAO_LARGE_INTESTINE_24W_…																														
CELLMARKERMSIGDB_GAO_LARGE_INTESTINE_ADUL…	SLC29…	SLC38…	SLC5A8	SLC7A2	SMARC…	SMOC1	SNAP25	SOGA3	SOX12	SPART	SPHK1	SPOCK1	SPRED3	SPTBN4	SSTR5…	ST18	STK33	STMN2	STMN3	STX1A	STXBP…	SV2C	SYN1	SYNPR	SYP	SYT1	SYT4	SYT5	TAF6L	TCEAL3
CELLMARKERMSIGDB_GAO_LARGE_INTESTINE_ADUL…	PDZD4	PFN1P2	PHKA1	PHLDA3	PIPSL	PKP1	PLA2G…	PLEK	PLXDC1	PLXNC1	PMCHL2	POLQ	POLR3G	POM12…	PPEF2	PPIEL	PPP1R…	PREX1	PRND	PRPF4	PRR12	PRR7	PSORS…	QKI	QRFPR	RAB11…	RAB36	RALYL	RAMP3	RBBP9
CELLMARKERMSIGDB_GAO_LARGE_INTESTINE_ADUL…																														
CELLMARKERMSIGDB_GAO_LARGE_INTESTINE_ADUL…	PHGDH	PIK3CG	PLAAT3	PLEKH…	PNOC	POU2A…	POU2F2	PPM1K	PPP1R…	PPP1R…	PRDM1	PRKCB	PTPN7	PTPRC	PTPRC…	PTPRS	RAB30	RAB34	RASA3	RASAL3	RASGR…	RASSF5	RCSD1	RELT	RGCC	RGS1	RGS19	RHOH	RHOQ	RNF122
CELLMARKERMSIGDB_GAO_LARGE_INTESTINE_ADUL…	LGI4	LHFPL6	LIFR	LIMCH1	LIMS2	LINC0…	LINC0…	LMOD1	ENSG0…	LINC0…	C2orf…	RBM15…	LOX	LRP1	LRRC32	LRRC49	LRRN3	LTBP2	LUM	LY6E	MAF	MAGI2…	MAMDC2	MAP1B	MAP7D3	MAPK10	MCAM	MCTP1	MEG3	MEIS2
CELLMARKERMSIGDB_GAO_SMALL_INTESTINE_24W_…																														
CELLMARKERMSIGDB_GAO_SMALL_INTESTINE_24W_…																														
CELLMARKERMSIGDB_GAO_SMALL_INTESTINE_24W_…																														
CELLMARKERMSIGDB_GAO_SMALL_INTESTINE_24W_…																														
CELLMARKERMSIGDB_GAO_SMALL_INTESTINE_24W_…																														
CELLMARKERMSIGDB_GAO_STOMACH_24W_C1_PROCR…																														
CELLMARKERMSIGDB_GAO_STOMACH_24W_C2_TFF2P…																														
CELLMARKERMSIGDB_GAO_STOMACH_24W_C4_PARIE…																														
CELLMARKERMSIGDB_GAO_STOMACH_24W_C5_PUTAT…																														
CELLMARKERMSIGDB_GAUTAM_EYE_IRIS_CILIARY_…	AUTS2	HAGH	CISD1	BYSL	HIPK2	TMEM1…	ISY1	CNOT7	SAFB	GLTP	RAB1B	ROCK2	SCN1B	COMMD2	EVA1C	ATP5F…	PSENEN	SREK1…	MLST8	DDX5	SPR	PCTP	LY6G5C	PLEKH…	DDX21	ILF3	NAALA…	CREB3…	ZNF578	ZNF800
CELLMARKERMSIGDB_GAUTAM_EYE_IRIS_CILIARY_…																														
CELLMARKERMSIGDB_GAUTAM_EYE_IRIS_CILIARY_…																														
CELLMARKERMSIGDB_GAUTAM_EYE_IRIS_CILIARY_…																														
CELLMARKERMSIGDB_GAUTAM_EYE_IRIS_CILIARY_…																														
CELLMARKERMSIGDB_HAY_BONE_MARROW_CD34_POS…																														
CELLMARKERMSIGDB_HAY_BONE_MARROW_CD34_POS…																														
CELLMARKERMSIGDB_HAY_BONE_MARROW_CD34_POS…																														
CELLMARKERMSIGDB_HAY_BONE_MARROW_CD34_POS…																														
CELLMARKERMSIGDB_HAY_BONE_MARROW_CD8_T_CE…																														
CELLMARKERMSIGDB_HAY_BONE_MARROW_EARLY_ER…																														
CELLMARKERMSIGDB_HAY_BONE_MARROW_EOSINOPH…																														
CELLMARKERMSIGDB_HAY_BONE_MARROW_NAIVE_T_…	NSMCE3	FXYD7	MPHOS…	RIPOR2	SATB1	SLC38…	TBC1D4	CUTALP	TTC39…	CNN2	LINC0…	PIM2	SLC25…	APBB1	SCML4	SSR2	TNRC6B	ANAPC…	ZBTB25	RBL2	ORMDL3	MPZL3	TMEM2…	USP10	CFAP36	MHENCR	P2RY10	WNT7A	LYPD3	MDS2
CELLMARKERMSIGDB_HAY_BONE_MARROW_NEUTROPH…	NPTN	RILPL2	ARHGE…	LYST	LAMTO…	ASGR2	GABAR…	GNAQ	PRR13	AQP9	SMARC…	FPR2	CSGAL…	SLC24…	ZDHHC…	COX4I1	TNNT1	TGFB1	HLX	UBR4	SLC15…	APOBR	PTCH2	ATP5F…	CHMP4B	GAS7	RASSF4	TFEC	WLS	TET2
CELLMARKERMSIGDB_HAY_BONE_MARROW_NK_CELLS	PPP2R…	PRR5L	ZNF600	MIR14…	ADRB2	RGS9	RBM39	PSMB10	TCF25	EBP	ST6GA…	CBLB	USB1	CERS4	RPS6K…	APOBE…	RSRP1	ARHGA…	TSC22…	MAPK1	LDB2	PAFAH2	TLE1	SELEN…	NFATC3	SLFN13	PRR5	LAMP1	AGK	PLEKH…
CELLMARKERMSIGDB_HAY_BONE_MARROW_PRE_DEND…																														
CELLMARKERMSIGDB_HAY_BONE_MARROW_PRO_B	ACOT7	TRA2B	TP53I…	TERF1	NPPC	HNRNP…	DCLRE…	TTC7A	ARHGA…	EIF4G2	CSRP2	SET	NTAN1	CD47	TLCD3B	MAGOH	NCAPH2	MAPRE1	ATP2A3	C1orf…	SF3B2	QPRT	CTCF	H2AC16	CCP110	RDM1	IFT22	EWSR1	HNRNP…	H2AC17
CELLMARKERMSIGDB_HAY_BONE_MARROW_STROMAL	CNTFR	APOB	TENM2	SULF1	NLGN4X	TMEM1…	PLIN1	SYDE1	SELEN…	LRATD1	DAAM2	SGCB	PLEKH…	OLFML1	MROCKI	GAREM1	TJP1	ASPA	FHL1	DCLK1	SCUBE1	COL6A3	ATF3	RGS3	MMP14	BOC	KIRRE…	CRH	NRXN2	ADH1B
CELLMARKERMSIGDB_HE_LIM_SUN_FETAL_LUNG_C0…	RNF150	DCLK1	MAP1A	TBC1D7	HAGH	SYNM	CPXM2	RAB9B	CUEDC1	RHOQ	PPP2R…	STRN3	BCL11A	PLCB3	WASF1	FZD3	GEMIN7	MAPK14	MSC-A…	LRATD2	MRAS	NPDC1	INPP4A	RASSF3	ELAC2	ST6GA…	TOB1	HOXC4	METTL…	NUDT14
CELLMARKERMSIGDB_HE_LIM_SUN_FETAL_LUNG_C0…																														
CELLMARKERMSIGDB_HE_LIM_SUN_FETAL_LUNG_C0…																														
CELLMARKERMSIGDB_HE_LIM_SUN_FETAL_LUNG_C0…	TAF10	RAB38	IL6R	UQCRHL	YDJC	PDGFRL	ADAM33	SLC38…	NPNT	TOM1L1	SYNPR	DICER1	EPHA3	GOLGA…	RHPN2	ZNF738	MACRO…	TSPAN…	HAGH	NXN	FREM2	CYRIB	ITLN1	TUBB4A	HSPA1…	SEC11C	EPHA7	SLC48…	ADAMT…	SH3BP4
CELLMARKERMSIGDB_HE_LIM_SUN_FETAL_LUNG_C0…																														
CELLMARKERMSIGDB_HE_LIM_SUN_FETAL_LUNG_C0…																														
CELLMARKERMSIGDB_HE_LIM_SUN_FETAL_LUNG_C0…	DBNDD2	MET	C1RL	SLC39…	BHLHE…	OPTN	SOX6	TGFBR2	PCOLC…	RSPO3	SLC25…	IGSF9	OCIAD2	ZBTB7C	CYP27…	BCO2	INMT	SORBS2	PSMB9	RBP7	PPL	FAM10…	TMEM8B	EPS8L2	DPP7	HSPG2	TMCO3	DOCK11	SLC25…	FSTL3
CELLMARKERMSIGDB_HE_LIM_SUN_FETAL_LUNG_C0…	BICD1	SPC24	CDC45	ANLN	DLG2	THSD4	CDKN3	FST	H2BC21	PROM1	MXD3	H4C11	B3GAL…	SPAG5	GPR37	KIF18A	OIP5	H2BC9	KIF11	BUB1	CAPN5	SGO1	MOB3B	CXXC4	MCM10	ECT2	SKA3	CENPO	PGP	H2AC13
CELLMARKERMSIGDB_HE_LIM_SUN_FETAL_LUNG_C0…																														
CELLMARKERMSIGDB_HE_LIM_SUN_FETAL_LUNG_C0…	CRLS1	BOK	C21or…	TGM1	CFB	SLC48…	CD22	BDNF	COL4A6	GEM	KLF2	ATP11A	GABAR…	MRGPRF	SLC10…	ADAMT…	FAAH	ADAMT…	BNC1	CSDC2	PLXNB1	NMU	UBXN6	PNPLA4	SLC25…	PTGIS	A4GALT	CRABP2	TMBIM4	DCTN6
CELLMARKERMSIGDB_HE_LIM_SUN_FETAL_LUNG_C0…																														
CELLMARKERMSIGDB_HE_LIM_SUN_FETAL_LUNG_C1…																														
CELLMARKERMSIGDB_HE_LIM_SUN_FETAL_LUNG_C1…																														
CELLMARKERMSIGDB_HE_LIM_SUN_FETAL_LUNG_C1…																														
CELLMARKERMSIGDB_HE_LIM_SUN_FETAL_LUNG_C1…	SPDL1	TUBB3	PCGF6	BRCA2	CENPO	DTL	CDC25B	C5orf…	CPXM1	POLE	LRRC49	CHST6	ZNF695	MTHFD2	GRK6	PACC1	CCNJ	CLEC1…	GPSM2	GAS2	HOMER3	SLC5A6	LDLRA…	CENAT…	THNSL2	EXOSC6	GREB1	DONSON	MMS22L	C6orf…
CELLMARKERMSIGDB_HE_LIM_SUN_FETAL_LUNG_C1…	ST6GA…	BIK	DACH1	KLHL32	CNNM1	LINC0…	CENPF	SRGAP1	TPST2	RGS7	TESK1	RIMS2	VSTM2A	GOLGA…	CENPS	OPTN	SLC6A8	HORMA…	THBS3	SMOC1	FAM22…	MAST1	MEGF9	DLL3	POU2F2	ZDHHC8	DACH2	MAPK15	BCORL1	AKT3
CELLMARKERMSIGDB_HE_LIM_SUN_FETAL_LUNG_C1…																														
CELLMARKERMSIGDB_HE_LIM_SUN_FETAL_LUNG_C1…																														
CELLMARKERMSIGDB_HE_LIM_SUN_FETAL_LUNG_C1…																														
CELLMARKERMSIGDB_HE_LIM_SUN_FETAL_LUNG_C1…	MPZL3	ADORA…	IKBKE	GBP3	CTSW	PLAT	PAQR4	TIAM1	SLC30…	DOCK9	CAPNS2	ABCG1	LMTK3	SLC22…	OPTN	AMN	DLK1	KRT13	SLCO4…	ZBTB7C	WDR49	SOSTD…	ARAP2	TRIM22	ARHGA…	CNTN1	ACKR3	SRGAP1	EGR3	RASL1…
CELLMARKERMSIGDB_HE_LIM_SUN_FETAL_LUNG_C1…	SYT7	PTPRN	PDGFD	RTN1	MIR20…	COMTD1	KCND3	TAMAL…	SLC6A8	RAB30	TMEM1…	ELOVL7	CHN2	USP41	RAB6B	THSD4	KCNH3	DUSP10	ASB13	SMOC1	STMN2	RAPGE…	SLIT1	TIMP1	RAB26	PNPLA7	NLRP1	TMOD2	COL22…	CFC1B
CELLMARKERMSIGDB_HE_LIM_SUN_FETAL_LUNG_C1…	NEIL3	EOGT	MARK1	OPTN	RIC3	FXYD5	FGF3	TNFRS…	NTRK3	KIAA1…	SH3BP1	LAMP5	PFKP	BCL2A1	ADAMT…	LTB	WNT10B	TNS4	LSAMP	ROPN1	SEPTI…	RELL2	GABRP	SIRPA	NAAA	ROBO2	CHST15	FEZ1	WNT10A	GPX2
CELLMARKERMSIGDB_HE_LIM_SUN_FETAL_LUNG_C1…	SH3BP4	AVPI1	CHAF1B	CITED4	NIPAL1	LINC0…	ZNF524	BCL3	CNNM4	AREG	ITGB8	LPAR6	NRAD1	CLDN8	KRT6B	TMEM71	CASC8	UBXN8	ANPEP	B3GNT3	SLCO1…	SECTM1	DHRS12	CLMN	SLC44…	TSLP	CRLF3	PF4V1	HEY2	PAX1
CELLMARKERMSIGDB_HE_LIM_SUN_FETAL_LUNG_C2…	PLBD2	PAPSS2	EIF2A…	DNMT3A	EDC3	MMD	ANKH	ZNF58…	PPFIB…	MMP2-…	C6orf…	HMGXB3	MORN2	ZER1	RIDA	SLC37…	PCYT2	CD209	SQOR	GSAP	SLC46…	SAMD9L	CD5L	FCGBP	KIAA1…	CHCHD6	SLC48…	PLEKH…	FBXW2	RARRE…
CELLMARKERMSIGDB_HE_LIM_SUN_FETAL_LUNG_C2…	FUT8	MYO9A	ENOX1	IFT57	CKB	LY75	ISG20	TSPAN…	FAM98B	CD38	TSPOA…	ZDHHC…	H3C10	IQCE	ADORA3	TSPAN…	B4GAL…	PIGZ	ZBED3	ACOT7	ESAM	GRAP2	C10or…	RBBP8	CDCA7	MACRO…	GOLGA…	EGLN2	SEL1L3	AP1S3
CELLMARKERMSIGDB_HE_LIM_SUN_FETAL_LUNG_C2…																														
CELLMARKERMSIGDB_HE_LIM_SUN_FETAL_LUNG_C2…																														
CELLMARKERMSIGDB_HE_LIM_SUN_FETAL_LUNG_C2…																														
CELLMARKERMSIGDB_HE_LIM_SUN_FETAL_LUNG_C2…	UBR5-…	SVIP	MEG3	ACOT2	ZNF506	TNFSF8	CAVIN1	OBI1	SMARC…	ESAM	LMAN2L	NT5C3B	ACTR6	UMPS	PFKP	ATP2A3	INTS4	TP53B…	BPNT1	PHGDH	TESMIN	HARS2	INPP4B	LXN	GRAP	ZNF606	TRBV28	LINC0…	SAMD3	MED9
CELLMARKERMSIGDB_HE_LIM_SUN_FETAL_LUNG_C2…	SMIM3	DENND…	MAP3K5	SLC39…	BBC3	SLC39…	EMILI…	SEPTI…	MAP4K2	CYB5R1	RAB13	KIFAP3	SLC44…	SCYL2	CALCO…	TVP23B	COPS4	USP20	HBQ1	LAPTM…	MDM1	MTHFD…	ATG4D	SLC30…	TBC1D…	LRRC32	VAPB	HBG1	CLDN5	FN3K
CELLMARKERMSIGDB_HE_LIM_SUN_FETAL_LUNG_C2…																														
CELLMARKERMSIGDB_HE_LIM_SUN_FETAL_LUNG_C2…	FBXO3	SENP5	TRAPP…	DLEU2	POLR1C	PDK1	RAB30	ANKRD9	TTC33	MAD2L…	H2BC12	BPGM	MTURN	SMTN	CRKL	BMT2	CXCL3	THUMP…	H4C16	USP12	RBM7	PXMP4	LRRC8B	ZNF567	SOS1	SPHK1	RDH11	UTY	PDZK1…	CRYM
CELLMARKERMSIGDB_HE_LIM_SUN_FETAL_LUNG_C2…	CENPM	ABHD10	NT5DC1	EBP	CDC23	EZH2	GPATC…	OCIAD2	PYCR2	SLC38…	SLC2A6	CD200	BTLA	TRERF1	CENPF	MPHOS…	RRM1	RAD51C	ODF2	IGSF8	LEMD3	NUP88	RAD9A	SMC2	DYNC2…	HEMK1	CEP20	SMC4	CDH23	ATAD5
CELLMARKERMSIGDB_HE_LIM_SUN_FETAL_LUNG_C2…																														
CELLMARKERMSIGDB_HE_LIM_SUN_FETAL_LUNG_C2…																														
CELLMARKERMSIGDB_HE_LIM_SUN_FETAL_LUNG_C2…																														
CELLMARKERMSIGDB_HE_LIM_SUN_FETAL_LUNG_C3…																														
CELLMARKERMSIGDB_HE_LIM_SUN_FETAL_LUNG_C3…	TRIP13	STIL	H4C6	NMNAT3	AURKB	SKA1	ZBTB7A	RACGA…	PMM1	MCM4	JAZF1	CDKN2C	HPRT1	CHAC2	SLC20…	RAD51…	SPC25	H3C3	H3C8	PCK2	ARHGA…	PRIM1	SLC11…	NCAPD2	H2BC7	DDI2	DPF3	BOP1	RPS6K…	FAH
CELLMARKERMSIGDB_HE_LIM_SUN_FETAL_LUNG_C3…	PHF23	CFP	MAD2L1	SLC16…	ARFIP2	MZT1	DUSP3	RAB32	TMSB1…	MEX3A	LMNB2	SMC2	SIGMA…	ALG8	KLHDC…	SMAD6	FLAD1	ACAT2	TTC9C	KCNK6	TMEM1…	ORAI1	CEBPB	DALRD3	CDC20	RNF24	UBE2T	MARK4	NUDT1	PSAT1
CELLMARKERMSIGDB_HE_LIM_SUN_FETAL_LUNG_C3…	CALHM6	SHC2	SCNN1B	FHIT	TMEM3…	C1orf…	DSEL	CALHM2	PRKAG2	GLIS3	OAS1	KLHL3	METTL…	SGCD	ADAMT…	PLEKH…	PLA2G…	DOCK8	DTX4	SPR	APBA2	VSTM4	SCG3	CPXM2	PGM5-…	SCRN1	SMAD9	PCLO	TMOD2	PDE7B
CELLMARKERMSIGDB_HE_LIM_SUN_FETAL_LUNG_C3…	EMILI…	NTN1	TGFBR…	RHOD	RAB11…	SCARA3	IQSEC3	IQCA1	ACKR2	CRTAC1	SDC3	MGST1	COL5A2	MID1	STOX2	ACSL5	ATP9A	SPOCK3	PPARG	SH3PX…	F8	DAPK1	EMID1	C15or…	MTSS2	SCNN1B	PRKAG2	CYP39…	CERS6	MVP
CELLMARKERMSIGDB_HE_LIM_SUN_FETAL_LUNG_C3…																														
CELLMARKERMSIGDB_HE_LIM_SUN_FETAL_LUNG_C3…	DNASE2	CHST3	IFIH1	ABCA2	NPLOC4	ANOS1	SPEG	NCOA7	PTPRJ	EPS8	CERCAM	COPZ2	LYPD2	STON1	EVA1C	FGFRL1	RND3	FBLN1	ERAP1	CUBN	TRH	FOXC2	LDLR	GUCY1…	IL18BP	ABCA3	TRIM22	TNFRS…	ARSJ	CDKN1A
CELLMARKERMSIGDB_HE_LIM_SUN_FETAL_LUNG_C3…	HSPA2	SLC38…	NMD3	TUBB2B	GAA	CASP6	CNST	TMC6	CALHM2	RPRD1A	MIR99…	KCNQ1…	NAPG	MMP16	CPE	NIT2	EML3	HACD2	RASGR…	PPARG	LMBR1L	TOP2A	RABGA…	UNC119	SLC6A6	AGAP1	BCHE	NPY1R	NAV2	BMX
CELLMARKERMSIGDB_HE_LIM_SUN_FETAL_LUNG_C3…																														
CELLMARKERMSIGDB_HE_LIM_SUN_FETAL_LUNG_C4…																														
CELLMARKERMSIGDB_HE_LIM_SUN_FETAL_LUNG_C4…																														
CELLMARKERMSIGDB_HE_LIM_SUN_FETAL_LUNG_C4…																														
CELLMARKERMSIGDB_HE_LIM_SUN_FETAL_LUNG_C4…																														
CELLMARKERMSIGDB_HE_LIM_SUN_FETAL_LUNG_C4…	MGME1	CCDC18	CEP55	MSH2	TOPBP1	TMEM18	KIF14	FANCD2	C1orf…	CTBP1	MTRFR	RAB27A	NSL1	PRPS1	UBALD2	MRPL55	DBF4	ARHGA…	HTRA2	ARL6I…	FN3KRP	ATG16…	ITGB3…	RAD9A	TMEM1…	PDS5B	RIF1	SGO2	CDKN1A	H3C10
CELLMARKERMSIGDB_HE_LIM_SUN_FETAL_LUNG_C4…	ACD	SPC24	TIMEL…	PDS5B	BAZ1B	CEP55	RFC5	GPN3	DCLRE…	MASTL	TOB1	NUP58	MZT1	MCMBP	EXOSC8	SSBP2	POLD2	H2AC12	WDR62	E2F2	RPL39L	GTF3C5	BUB1B	GSPT1	ATAD5	MLST8	LRR1	SHCBP1	H4C6	CCDC18
CELLMARKERMSIGDB_HE_LIM_SUN_FETAL_LUNG_C4…																														

CELLMARKERCAN_ACINAR_CELLS																														
CELLMARKERMSIGDB_HE_LIM_SUN_FETAL_LUNG_C4…																														
CELLMARKERMSIGDB_HE_LIM_SUN_FETAL_LUNG_C4…																														
CELLMARKERMSIGDB_HE_LIM_SUN_FETAL_LUNG_C4…																														
CELLMARKERMSIGDB_HE_LIM_SUN_FETAL_LUNG_C5…																														
CELLMARKERMSIGDB_HE_LIM_SUN_FETAL_LUNG_C5…	ZDHHC…	CDIP1	ADPRH	CDK4	TCIRG1	MPEG1	PLGRKT	PRKD3	GLS	ECE1	MPHOS…	HSPBP1	DALRD3	TYK2	DERL1	IL2RG	UROS	MS4A6A	RAB11…	ETFB	CD1C	RNASE6	NFAT5	RAB30	ITGAL	WASHC3	RRBP1	CCDC47	NRG4	TOR3A
CELLMARKERMSIGDB_HE_LIM_SUN_FETAL_LUNG_C5…																														
CELLMARKERMSIGDB_HE_LIM_SUN_FETAL_LUNG_C5…																														
CELLMARKERMSIGDB_HE_LIM_SUN_FETAL_LUNG_C5…	CENPE	PARP2	CCHCR1	SVIP	POLD1	NCAPD3	DHRS4…	H2AC12	PCNT	KIF23	CCNF	PRPSA…	PARPBP	RMI1	MT1X	HMGXB4	FANCL	PLK1	C21or…	CSTF2T	CLEC1…	DDX11	KIF4A	CRNDE	KIF18A	SMC04	H3C8	SORBS3	C4orf…	PIMREG
CELLMARKERMSIGDB_HE_LIM_SUN_FETAL_LUNG_C6…																														
CELLMARKERMSIGDB_HE_LIM_SUN_FETAL_LUNG_C7…																														
CELLMARKERMSIGDB_HE_LIM_SUN_FETAL_LUNG_C7…																														
CELLMARKERMSIGDB_HE_LIM_SUN_FETAL_LUNG_C7…																														
CELLMARKERMSIGDB_HE_LIM_SUN_FETAL_LUNG_C7…																														
CELLMARKERMSIGDB_HE_LIM_SUN_FETAL_LUNG_C7…	TMEM54	DNAJC…	ATP1A3	PFKP	GCHFR	SV2A	TH	UNC5D	FAR2	EID2B	MACRO…	GATA3…	SYN1	CHST8	EFNA3	FGF10	CHRFA…	PAK3	GRM8	ACSS2	F12	CELF5	GNE	RIMS1	SPOCK1	KCNK3	PLXNC1	TRIM67	ANKS1A	LUZP2
CELLMARKERMSIGDB_HE_LIM_SUN_FETAL_LUNG_C7…																														
CELLMARKERMSIGDB_HU_FETAL_RETINA_AMACRINE																														
CELLMARKERMSIGDB_HU_FETAL_RETINA_BIPOLAR																														
CELLMARKERMSIGDB_HU_FETAL_RETINA_FIBROBLA…	HMGA2	RNU4A…	PLEKH…	EFNB2	DDR2	EXTL2	NHS	ROCK2	PTCH1	FAM11…	ALCAM	MAGED2	SIX2	GPC4	KLF2	ANTXR1	ANXA5	ZFP36…	NFKBIA	TUSC3	RBMS1	SNHG18	GSTO1	FOXP2	PLBD1	TCEAL9	FKBP7	FBLIM1	SDC1	AMOTL1
CELLMARKERMSIGDB_HU_FETAL_RETINA_HORIZONT…																														
CELLMARKERMSIGDB_HU_FETAL_RETINA_MICROGLIA	CASP1	ABCG2	VAV1	MYLIP	PREX1	ABI3	CMTM7	NFKBIZ	GAS6	GPSM3	SIRPA	MEF2A	SFT2D1	FMNL3	ACTR2	CXCL16	ARRB2	COLGA…	PRR13	C12or…	P2RX4	RCSD1	CYTH4	PLEKH…	TNFRS…	HLA-A	FGD2	PLAUR	GRB2	ARHGA…
CELLMARKERMSIGDB_HU_FETAL_RETINA_MULLER																														
CELLMARKERMSIGDB_HU_FETAL_RETINA_PHOTOREC…																														
CELLMARKERMSIGDB_HU_FETAL_RETINA_RGC	STX7	MAPK10	CHN1	RAB10	FASN	DYNLR…	CPNE9	JUP	ACSL3	ISLR2	PHACT…	NLN	KLHL1	BEND5	PFDN2	SOX11	DACH2	PRRT2	MTCH2	LSS	MFSD10	FAXC	PPP1R…	JAKMI…	TAF9	TP53I…	CPLX2	CNR1	GNAI1	SNAP25
CELLMARKERMSIGDB_LAKE_ADULT_KIDNEY_C1_EPI…																														
CELLMARKERMSIGDB_LAKE_ADULT_KIDNEY_C10_TH…	PAPOLA	RPL5	LAMTO…	TAF7	UBA52	PDIA3	TAX1B…	ANXA2	COX7C	PFDN5	HADHB	RPL32	RPL24	RPL18	HADHA	TNS3	RPS27	ACSL4	DEPTOR	RPS25	HLA-E	CD164	REEP5	EEF2	OSMR	CD81	RPL30	NCL	ADGRG1	RPL37A
CELLMARKERMSIGDB_LAKE_ADULT_KIDNEY_C12_TH…	ATP5I…	RPL21	RPS24	RPS13	REEP5	ITM2C	TUFM	ATP5F…	UBB	HIP1	RPSA	NDUFA5	SORT1	TBC1D4	RPL10A	SLC4A7	RPS25	FOXP2	PPARG…	PLPPR1	RPL27	SGIP1	GNAI1	VEPH1	ERICH1	RPL41	CLDN10	UQCRC2	RPL35	DZIP1
CELLMARKERMSIGDB_LAKE_ADULT_KIDNEY_C18_CO…	RPS25	MTUS1	APLP2	ATP5F…	CCT6A	SERBP1	EEF2	ACADVL	LLGL2	RPL34	RPL32	SPTBN1	NUCKS1	YBX1	HNRNPU	RPL12	SMARC…	COX7C	SEPTI…	FAM10…	ACAT1	DCDC2	PTTG1…	SRSF3	ATP1B1	IVNS1…	HSPA9	RSRP1	MTATP…	RPS24
CELLMARKERMSIGDB_LAKE_ADULT_KIDNEY_C19_CO…	RPS17	CNN3	RPL41	BMPR1B	TAX1B…	RPL12	GNAS	FAU	COX5B	TM9SF3	MOB1B	ACTR2	RPL34	PHLDB2	PGK1	NORAD	VAPA	UBB	ST6GA…	EZR	SERF2	CLIP1	ANXA2	TFCP2…	MYL6	UBXN4	MPPED2	EPRS1	HNRNPU	ARHGE…
CELLMARKERMSIGDB_LAKE_ADULT_KIDNEY_C25_EN…																														
CELLMARKERMSIGDB_LAKE_ADULT_KIDNEY_C28_IN…																														
CELLMARKERMSIGDB_LAKE_ADULT_KIDNEY_C29_UN…																														
CELLMARKERMSIGDB_LAKE_ADULT_KIDNEY_C5_PRO…	PPP6R2	EBNA1…	SLC17…	CHRM3	LSAMP	RIMS2	RHOBT…	TTTY14	PIP5K…	ZNF708	SLC47…	SPEF2	SLC43…	RNF152	TLN2	ALDH6…	NQO2	C4orf…	ADGRV1	GMDS-…	FRMD4B	BHMT	TPD52	ANKRD…	NR6A1	ACACB	SLC1A1	KDM2A	NCOR1	SNX29
CELLMARKERMSIGDB_MANNO_MIDBRAIN_NEUROTYPE…																														
CELLMARKERMSIGDB_MANNO_MIDBRAIN_NEUROTYPE…	MAB21…	MAGI2	MAP1B	MAP2	MAP3K…	MAP3K9	MAST1	MAST2	MED12L	MEG3	MIAT	MIR12…	MIR39…	MLLT11	MMP24	MSANT…	MTURN	MTUS2	MUC16	MUC19	MYO5A	MYRIP	MYT1L	NAP1L3	NAV3	NCAM1	NCDN	NEB	NEDD4L	NEGR1
CELLMARKERMSIGDB_MANNO_MIDBRAIN_NEUROTYPE…	KCTD8	KIAA1…	SHTN1	KIF21A	KIF21B	KIF3A	KIF3C	KIF5C	KIFC2	KLC1	KLHL1	KLHL13	KLHL29	KLHL32	KLHL35	KSR2	L1CAM	LDB2	LGI2	LHFPL4	LINGO1	LMO4	LMTK3	LMX1B	DRAIC	LONRF2	LPCAT4	LPIN1	PLPPR4	LRRC3B
CELLMARKERMSIGDB_MANNO_MIDBRAIN_NEUROTYPE…	LRRC3B	LRRC49	LRRC55	LRRC7	LRRTM2	LRRTM4	LY6H	MAP1B	MAP2	MAP7D2	MAPRE2	MAST1	MCC	MEG3	MGAT4C	MIAT	MIR39…	MLLT11	MMP24	MPP2	MPPED2	MTURN	MTUS2	MYCBP2	MYO5A	MYRIP	MYT1	MYT1L	NALCN	NAP1L3
CELLMARKERMSIGDB_MANNO_MIDBRAIN_NEUROTYPE…	FOLH1B	FOS	FOSB	FOXC1	FOXF1	FOXF2	FOXL2	FOXL2…	FOXN3	FOXQ1	FRMD4B	FRMD6	FRMD8	FSCN1	FSTL1	FUT1	FXYD5	FZD4	FZD6	GAB1	GABRE	GADD4…	GADD4…	GALNT…	GAS6	GATA2	CASTO…	GBP4	GBP6	GDF15
CELLMARKERMSIGDB_MANNO_MIDBRAIN_NEUROTYPE…	IKBKE	IKZF1	IL13R…	IL16	IL17RA	IL18	IL1B	IL1R1	IL27RA	IL4R	IL6R	IL6ST	IL7R	INPP5D	INPPL1	IPCEF1	IRAK2	IRF1	IRF8	ITGAV	ITGB2	ITM2B	ITPR2	ITPRI…	ITSN2	JAK3	JUNB	ANOS1	KBTBD8	KCNE3
CELLMARKERMSIGDB_MANNO_MIDBRAIN_NEUROTYPE…	GPSM1	GRIA1	GRIA2	GRIA4	GRID2	GRIK2	GRIN3A	GRIP2	GRM4	GRM5	GRM7	GRM8	GSE1	GUCY1…	HCN3	HERC2…	NSG2	HOOK1	HPCAL4	HS3ST1	HS6ST3	ICA1L	IDH3A	IGF1	IGFBP3	IKZF4	INA	INHBA…	IRGQ	JAG2
CELLMARKERMSIGDB_MANNO_MIDBRAIN_NEUROTYPE…	SH3PX…	SHISA2	SHISA6	SHROO…	SIM1	SLC17…	SLC22…	SLC37…	SLC38…	SLC4A8	SMIM18	SMPD3	SNAP25	SNAP91	SOX11	SOX4	SPINT2	SRGAP1	SRRM4	SRSF12	ST6GA…	ST8SI…	STMN1	STMN2	SV2A	SVOP	SYBU	SYCP2	SYN3	SYT4
CELLMARKERMSIGDB_MANNO_MIDBRAIN_NEUROTYPE…	NCAN	NCDN	NCS1	NDRG4	NEDD4L	NELL1	NFASC	NKAIN2	NKX2-2	NLN	NMNAT2	NNAT	NOL4	NOVA1	NOVA2	NPTXR	NREP	NRXN1	NRXN2	NRXN3	NSG1	NTNG1	NUAK1	NXPH2	ONECU…	OPCML	OPTN	OSBPL6	PAK3	PAK5
CELLMARKERMSIGDB_MANNO_MIDBRAIN_NEUROTYPE…	PLCL1	PLCXD3	PLXNA3	PLXNA4	PMFBP1	POU6F2	PPFIA2	PPP2R…	PPP2R…	PRKACB	PRKAR…	PRMT8	PRPH	PRRT2	PSD2	RAB11…	RAB15	RAB3A	RAB6B	RAB9B	RAPGE…	RAPH1	RASA4	RBFOX1	RBFOX2	RBMS3	RCOR2	REEP1	RELN	RHOU
CELLMARKERMSIGDB_MANNO_MIDBRAIN_NEUROTYPE…	PLAT	PLEKH…	PLLP	PMP2	PMP22	PLPP4	PPP2R…	PRDM8	PREX1	PRKCA	PRKCQ…	PSD	PTN	PTPRE	PTPRT	PTPRZ1	PXDC1	QKI	RAB31	RAB9A	RARG	RFTN2	RIMS1	RIT2	RNF14…	ROBO3	RTKN2	RUNDC…	SALL3	SAPCD2
CELLMARKERMSIGDB_MANNO_MIDBRAIN_NEUROTYPE…	FOXS1	FRMD4B	FRMD6	FRMD8	FRZB	FSTL1	FZD1	GAB1	GABRE	GADD4…	GALNT…	GAS6	CASTO…	GBGT1	GDF15	GEM	GGT1	GGT5	GGTLC2	GIMAP2	GIPC3	GJA4	GJA5	GJC1	GLA	GLRX	GLS	GLT8D2	GMFG	GNG11
CELLMARKERMSIGDB_MANNO_MIDBRAIN_NEUROTYPE…	SP5	SPAG5	SPC25	SPDL1	STK17B	STON1	STON1…	SYT2	TACC3	TDP1	TET1	TGIF2	TIMEL…	TK1	NEMP2	CEMIP2	TMEM97	TMPO	TMSB1…	TOP2A	TP53T…	TPX2	TRIM59	TROAP	TSIX	TTK	TYMS	UBE2C	UBE2T	UHRF1
CELLMARKERMSIGDB_MANNO_MIDBRAIN_NEUROTYPE…	SFXN2	SGO1	SGO2	SHROO…	SIVA1	SKA3	SLC39…	SLIT2	SMAD6	SMC2	SMC4	SNORA…	SNORD…	SNRPB	SOX2	SOX21	SPAG5	SPC25	SPDL1	STK17B	STON1	SYT2	TACC3	TCF12	TCF3	TENM4	TET1	TFF3	TIMEL…	TK1
CELLMARKERMSIGDB_MANNO_MIDBRAIN_NEUROTYPE…	PROM1	PRR11	PRTG	PTGR1	PTPRO	PTTG1	PTX3	RACGA…	RAD51…	RAN	RBM24	RBMX	RCN1	RCOR2	REST	RFC4	RFX4	RMST	RNASE…	RNF175	RPA3	RPL13…	RPL18	RPL23A	RPL26	RPL36…	RPS14	RPS19	RPS3	RRM2
CELLMARKERMSIGDB_MANNO_MIDBRAIN_NEUROTYPE…	SERPI…	SERTM1	SFRP2	SH3RF1	SHROO…	SKA3	SLC39…	SLC7A…	SLIT2	SOX2	SP5	SPAG17	SPAG6	SPC25	PLP1	STAR	STON1	SULF1	SYT2	TACC3	TBL1X	DYNLT5	TFF3	TMEM1…	CEMIP2	TMEM47	TMEM98	TMSB1…	TOP2A	TOX
CELLMARKERMSIGDB_MANNO_MIDBRAIN_NEUROTYPE…	PSAT1	PSD3	PTK2B	PTPRO	PXDC1	QKI	RAB2A	RAI14	RCN1	REST	RFTN2	RFX4	RGMA	RGS4	RHOJ	RMST	RNF182	ROPN1L	RPGR	RSPH1	RSPH9	RSPO2	RTKN2	S1PR1	SAMD13	SCD	SCN1A	SERTA…	SERTM1	SHROO…
CELLMARKERMSIGDB_MANNO_MIDBRAIN_NEUROTYPE…	HTRA1	HYDIN	ICA1L	ID4	IER2	IFI27…	IFI44L	IGDCC3	IGFBP2	IGSF5	IL5RA	ILDR2	INHBB	INPPL1	INSIG1	INTU	IQCA1	IQCG	IQGAP2	IRX1	IRX2	IRX4	ITGAV	ITGB5	ITGB8	ITM2C	ITPR2	JAG1	JUN	ANOS1
CELLMARKERMSIGDB_MANNO_MIDBRAIN_NEUROTYPE…	MNS1	MPC1	MPZ	MRC2	MT1F	MT3	MTSS2	MTTP	MYBL2	NACC2	NADK2	NCAN	NCAPG	NCKAP5	NDNF	NDP	NDRG2	NES	NFATC2	NFIA	NFIB	NFIX	NKAIN4	NKX2-2	NLGN4X	NOTCH1	NOTCH2	NPAS3	NPTXR	NRARP
CELLMARKERMSIGDB_MANNO_MIDBRAIN_NEUROTYPE…	QKI	RAB13	RAB31	RAB9A	RACGA…	RASSF2	RAVER1	RELN	RFC3	RHOJ	RIT2	RPE65	SCAMP2	SCD5	SCG3	SCHIP1	SCN1A	SCRG1	SCUBE2	SDC3	SEC14…	SERPI…	SGO2	SH3D19	SHISA3	SKA3	SLC1A2	SLC1A3	SLC25…	SLC4A4
CELLMARKERMSIGDB_MANNO_MIDBRAIN_NEUROTYPE…	IFI27…	IFI35	IFT57	IFT88	IGFBP2	IGFBP5	IGFBP…	IL33	INHBB	IQCA1	IQCG	IQGAP2	IRS1	ITGA6	ITGB5	ITGB8	ITIH5	ITM2C	ITPKB	ITSN1	JAM2	JUN	KCNAB1	KCNE5	KCNG1	KCNIP1	KCNK2	KCNMA1	KCNMB1	KCNMB4
CELLMARKERMSIGDB_MANNO_MIDBRAIN_NEUROTYPE…	RSBN1	RTN1	RUNDC…	RUNX1…	SAMD14	SAMD5	SBK1	SCARA5	SCG3	SCN3A	SCRT2	SEMA3C	SEPTI…	SEZ6L2	SH3BP5	SHANK2	SHISA6	SLC16…	SLC17…	SLC1A2	SLC22…	SLC37…	SLC38…	SLC6A…	SLC8A2	SMAP2	SMIM18	SMPD3	SNAP25	SNAP91
CELLMARKERMSIGDB_MANNO_MIDBRAIN_NEUROTYPE…	NCDN	NCOA7	NDFIP1	NDRG4	NEGR1	NELL1	NFASC	NKAIN2	NOL4	NPTXR	NRXN1	NRXN2	NSG1	OLFM3	OPCML	OPTN	PAK6	PAQR8	PCDH1…	PCDH19	PCDH7	PCDH9	PCDHA7	PCSK1N	PDE11A	PDZD7	MEST	PEG3	PFKP	PGM2L1
CELLMARKERMSIGDB_MENON_FETAL_KIDNEY_0_CAP…																														
CELLMARKERMSIGDB_MENON_FETAL_KIDNEY_10_IM…																														
CELLMARKERMSIGDB_MENON_FETAL_KIDNEY_2_NEP…																														
CELLMARKERMSIGDB_MENON_FETAL_KIDNEY_3_STR…																														
CELLMARKERMSIGDB_MENON_FETAL_KIDNEY_6_COL…																														
CELLMARKERMSIGDB_MURARO_PANCREAS_ACINAR_C…	FBXO25	CXCL12	S100A…	PHGDH	CFI	C5	RPL18A	GPRC5B	GPT2	B2M	FUT4	SLC38…	RNF213	NHSL2	CEMIP2	RDH10	EHD4	ARHGA…	FKBP11	SLC7A…	TST	TRIM47	AQP8	SEL1L3	RPL36…	EGFR	MYL12B	CD2AP	CNN2	IDH2
CELLMARKERMSIGDB_MURARO_PANCREAS_ALPHA_CE…	RPS6K…	SERTA…	KLHL3	G6PC3	CD82	BUD23	BACE1	GDA	STMN1	PDE4D	TMEM9	CKB	WDR59	NRXN1	GPR13…	WFS1	IL6R	BEX5	NPDC1	PLPP5	HERC5	CHKA	ARL3	LRPAP1	PTPRD	GGA2	UNC13A	ARNT2	NPTXR	DNER
CELLMARKERMSIGDB_MURARO_PANCREAS_BETA_CELL	LYSMD2	CAPN13	CACYBP	DOCK10	PPL	CENPC	CLCN3	CFL2	DNAJB1	PDE8B	FBXO17	NANOS1	SLC25…	NEO1	UBE3C	TMOD2	SLC2A1	LARP1	PLAGL1	C21or…	SEMA6D	ZNF91	CCSER2	LPIN1	TMEM1…	PHACT…	RANBP2	ZHX3	EPB41…	PI4KA
CELLMARKERMSIGDB_MURARO_PANCREAS_ENDOTHEL…	DGKD	GPX1	TGFBR1	LHFPL6	TMEM47	FABP5	ERBIN	VASH1	FSTL1	DENND…	SULF2	SNTB2	ADGRL2	PRCP	SYNPO	FRMD8	RAPGE…	CTNNA1	ETS2	MARCK…	PIM3	LXN	SYNM	ATAD3C	JAM3	LHFPL2	BNIP2	CDK2A…	DHRS3	RCC2
CELLMARKERMSIGDB_MURARO_PANCREAS_EPSILON_…																														
CELLMARKERMSIGDB_MURARO_PANCREAS_MESENCHY…	PTK7	NOTCH2	MSN	LXN	SHMT2	MT2A	CTSK	LDHB	PRDM1	NUPR1	SSH1	MYH9	ID3	THBS1	NES	FMNL3	NUMBL	SEC23A	JARID2	DSE	EDNRB	GPC6	CCN2	MRAS	IRX4	ITGB3	ZFP36…	ARHGE…	IFITM1	SH3KB…
CELLMARKERMSIGDB_RUBENSTEIN_SKELETAL_MUSC…	RPS4X	RPL27	RAP1A	RPL23	RPL34	S100A6	ENY2	SUB1	FCGRT	SEC11A	TMSB10	SPG21	COX4I1	M6PR	CFL1	ZNF706	COX7C	RPS12	CD74	ATP1B3	GLIPR2	SNX3	CSTB	RPL15	RPL23A	HSBP1	TPM3	NDUFB1	NOP10	ACTR3
CELLMARKERMSIGDB_RUBENSTEIN_SKELETAL_MUSC…	ATP5M…	TMEM2…	RPS20	EIF3K	SGCE	DBI	MOCS2	SNHG32	SMDT1	NDUFA5	NHP2	PYURF	ZFYVE…	MT-ND5	HNRNPR	UQCRF…	TMEM1…	GNAS	ST13	ATP5MF	DNAJC7	RPL28	LMNA	ATP5M…	CHCHD2	SNRPF	CCT3	DUT	TUBB	TIMM13
CELLMARKERMSIGDB_RUBENSTEIN_SKELETAL_MUSC…	BRK1	JUN	RPL41	PTMA	H4C3	LMNA	ATP5ME	RPL7	NPM1	SIVA1	EID1	GTF3C6	ATP5MK	RPL15	MT1M	ITGB1	ISCU	AK1	LSM5	CCDC1…	PHPT1	MFF	SGCE	PARL	METTL5	CYB5R3	NDUFB9	PKM	PRDX3	PFDN5
CELLMARKERMSIGDB_TRAVAGLINI_LUNG_ALVEOLAR…	PEBP4	B3GNT2	SBDS	PRDX1	CFLAR	LPCAT3	SPTBN1	LGALS3	GALK1	FLNA	RASSF8	MT2A	TGFBR2	HLA-C	PEAK1	SLC7A6	CRIP2	FAS	PTGS2	BCAP31	SEMA6D	DUSP1	DOCK11	PLEKH…	TMEM1…	MT1E	RNF213	PGM1	TSPAN4	ATOH8
CELLMARKERMSIGDB_TRAVAGLINI_LUNG_BASOPHIL…	DYNLT5	MBOAT7	MAPRE3	CDCA7L	SYTL3	SBDS	GALNT6	TMEM1…	LGALS…	SVIP	DNAJA1	GIHCG	ARHGD…	CD22	ALDH1…	NFE2L3	HNRNP…	SERTA…	STARD…	IL4R	PROCR	CSF2RB	LMNB1	FOXP1	PMP22	ASRGL1	RELB	TNFRS…	TFG	PAG1
CELLMARKERMSIGDB_TRAVAGLINI_LUNG_CAPILLAR…																														
CELLMARKERMSIGDB_TRAVAGLINI_LUNG_CAPILLAR…	GTF3A	PTPRN2	UBA52	HLA-D…	BCL6B	MTIF3	B2M	FOXN3	NDEL1	SLU7	ARPP19	ARHGA…	OTUD6…	RPL27	FGD5-…	FYTTD1	TUSC2	FBXO21	ARMCX3	ADIPO…	SZRD1	FNBP1L	DPYSL3	PAK4	EMP3	CPNE2	SYNJ2…	ZNF66…	CFAP36	MMP24…
CELLMARKERMSIGDB_TRAVAGLINI_LUNG_CD4_MEMO…																														
CELLMARKERMSIGDB_TRAVAGLINI_LUNG_EREG_DEN…	AHR	BCL3	ANKH	MALT1	MT1X	MIR44…	DDX21	IL1RN	LILRB3	RNASE1	ZSWIM6	PKIB	SKI	FKBP5	ATP6V…	SIPA1…	MACRO…	TNFRS…	ATP5MK	GSTO1	DUSP4	GRINA	HAVCR2	DBI	RNF145	SAP30	SPHK1	GNAI2	FGR	ETV6
CELLMARKERMSIGDB_TRAVAGLINI_LUNG_IGSF21_D…																														
CELLMARKERMSIGDB_TRAVAGLINI_LUNG_MESOTHEL…	FLNC	TMEM1…	PPA2	MARCO	LY96	SPSB1	SYNCR…	GCHFR	CBX3	STXBP2	MZT2A	UBE2D1	TUBB3	STAP2	NCOA7	ERP29	SDC4	VAMP8	HLA-D…	HDLBP	PTGER3	ANXA3	HLA-D…	RPA3	TMEM1…	MT1G	MGARP	MSANT…	GATA6	MRPL36
CELLMARKERMSIGDB_TRAVAGLINI_LUNG_MYOFIBRO…																														
CELLMARKERMSIGDB_TRAVAGLINI_LUNG_NATURAL_…																														
CELLMARKERMSIGDB_TRAVAGLINI_LUNG_NEUTROPH…	NDUFS6	MKNK2	CCDC8…	FGD4	TMUB1	LRP10	TLR2	MARCKS	SLC6A6	ULK1	MSL1	MHENCR	CYTH1	UQCRHL	SLC16…	NBPF10	HAL	SUN2	LCP2	PPP3CA	PSMA6	TMEM2…	PKM	FKBP8	NCF1B	SIPA1…	BCL3	FAM10…	ARHGA…	RPL12…
CELLMARKERMSIGDB_TRAVAGLINI_LUNG_OLR1_CLA…	PTGS2	SMIM3	SNAI1	RAB13	HNRNPM	OSM	RABGE…	ENO1	MAP2K3	SQOR	HMOX1	TRAF1	ATP1A1	CD55	PSAP	H3P6	DUSP1	FGR	LGALS1	SDCBP	ERGIC1	BATF3	ADM	MYL6	INSIG1	MS4A7	SERPI…	IRAK1	PCNX1	PITPNA
CELLMARKERMSIGDB_TRAVAGLINI_LUNG_PLATELET…	ACTB	CABP5	RPA1	RASGR…	PROS1	SLC39…	CMPK1	IFRD1	MLH3	SRC	RABGA…	TSPOA…	PLEKH…	PCP2	FXYD3	TAL1	DERA	MPST	ITGB5	MYL6	FHL2	BBLN	CENPT	CAPS	SSX2IP	MFAP3L	PLEK	IRX3	YPEL5	DNM3
CELLMARKERMSIGDB_TRAVAGLINI_LUNG_PROLIFER…	CALML3	RASSF1	ACOT7	ISLR	CYP2S1	RFC2	AP2S1	TRIM7	SKA2	COQ2	RHNO1	KNSTRN	BCL7C	SERPI…	GSS	BCL11A	CD320	LMNB2	NUDT8	RPA1	THOC3	PRPS2	AQP3	RPL4	HAUS4	LINC0…	MACRO…	CHCHD3	ISYNA1	BARD1
CELLMARKERMSIGDB_TRAVAGLINI_LUNG_PROLIFER…	MCM7	GLA	TIMP2	ATL3	FBXO5	CTSL	CREG1	NMB	CLIC4	GLDN	SLC11…	CD81	TEDC1	LRRCC1	SEPTI…	GINS2	ACAT1	COQ2	ADTRP	PGP	IFIT3	SEPTI…	NCAPH2	SNRNP…	NUDT1	MTX1	COA6	PDLIM7	ABHD5	KNSTRN
CELLMARKERMSIGDB_TRAVAGLINI_LUNG_PROXIMAL…	SNHG8	PCNA	UBA52	GCHFR	CTSB	MDH2	STEAP1	RPS13	NOB1	UQCRC2	TNFAI…	IGBP1	SERPI…	ATP5PB	ATP5M…	GMNN	CYP24…	TLE5	CTSC	HMGA1	BBLN	GDI2	CYP2J2	IGFBP6	ARG2	VILL	PDLIM1	HEY1	ASCL2	ATP5F…
CELLMARKERMSIGDB_TRAVAGLINI_LUNG_SEROUS_C…																														
CELLMARKERMSIGDB_TRAVAGLINI_LUNG_SIGNALIN…																														
CELLMARKERMSIGDB_TRAVAGLINI_LUNG_TREM2_DE…	PEPD	LGALS9	TIMM8B	DUSP23	CBR1	DPYSL2	COLGA…	GNB4	STEAP3	RNF13	DHRS4	CYB5A	FCGR1A	PDK4	MGST3	EIF4E…	PCK2	RAB32	SLAMF7	ACTN1	ME1	HAVCR2	SLC49…	CYFIP1	STAT1	MRAS	SYNGR2	SQOR	TSPAN4	TALDO1
CELLMARKERMSIGDB_TRAVAGLINI_LUNG_VASCULAR…																														
CELLMARKERMSIGDB_TRAVAGLINI_LUNG_VEIN_CELL																														
CELLMARKERMSIGDB_VANGURP_PANCREATIC_ALPHA…																														
CELLMARKERMSIGDB_VANGURP_PANCREATIC_DELTA…																														
CELLMARKERMSIGDB_VANGURP_PANCREATIC_GAMMA…																														
CELLMARKERMSIGDB_ZHENG_CORD_BLOOD_C1_PUTA…																														
CELLMARKERMSIGDB_ZHENG_CORD_BLOOD_C10_MUL…																														
CELLMARKERMSIGDB_ZHENG_CORD_BLOOD_C2_PUTA…																														
CELLMARKERMSIGDB_ZHENG_CORD_BLOOD_C4_PUTA…																														
CELLMARKERMSIGDB_ZHENG_CORD_BLOOD_C5_SIMI…																														
CELLMARKERMSIGDB_ZHENG_CORD_BLOOD_C6_HSC_…																														
CELLMARKERMSIGDB_ZHENG_CORD_BLOOD_C7_PUTA…																														
CELLMARKERMSIGDB_ZHENG_CORD_BLOOD_C8_PUTA…																														
CELLMARKERMSIGDB_ZHONG_PFC_C1_ASTROCYTE																														
CELLMARKERMSIGDB_ZHONG_PFC_C1_DLX5_POS_IN…																														
CELLMARKERMSIGDB_ZHONG_PFC_C2_ASCL1_POS_A…																														

Table S5: Cell state marker gene list

CELLMARKERCAN_ACINAR_CELLS																														
CELLMARKERMSIGDB_ZHONG_PFC_C2_UNKNOWN_NPC																														
CELLMARKERMSIGDB_ZHONG_PFC_C3_ASTROCYTE	CAPN10	DOT1L	MCM7	USP32…	ARSA	BRAP	MDGA1	UBALD2	LPL	RERE	STK11…	LRRC7	MTURN	CAMK2…	CIC	RBM33	NPY1R	STAG3	GRIK2	ANKRD…	CELF5	CNTN1	TBC1D…	SMARC…	RRAGC	ACD	ZDHHC…	SYT5	MCM3AP	PKD1
CELLMARKERMSIGDB_ZHONG_PFC_C3_HOPX_POS_OPC																														
CELLMARKERMSIGDB_ZHONG_PFC_C3_MICROGLIA	KIAA0…	CDC42…	GLRA2	AGAP3	SASH1	MGLL	CELF3	PRKCB	KIF1A	NFASC	SHISA7	KDM5B	NRXN2	STMN3	PCSK1N	SATB2	TMEM1…	RRAS2	LIMCH1	SNX32	BMERB1	RGS11	CARMI…	RFPL1S	FBXL16	SRCIN1	SNCB	KIF3C	NIPSN…	CACNB3
CELLMARKERMSIGDB_ZHONG_PFC_C3_UNKNOWN_INP																														
CELLMARKERMSIGDB_ZHONG_PFC_C4_UNKNOWN_INP																														
CELLMARKERMSIGDB_ZHONG_PFC_C6_PLXNA4_POS_…																														
CELLMARKERMSIGDB_ZHONG_PFC_C7_ORG_UNDERGO…																														
CELLMARKERMSIGDB_ZHONG_PFC_C8_ORG_PROLIFE…																														
CELLMARKERMSIGDB_ZHONG_PFC_C8_UNKNOWN_NEU…																														
CELLMARKERMSIGDB_ZHONG_PFC_C9_ORG_OTHER																														
CELLMARKERMSIGDB_ZHONG_PFC_HES1_POS_C1_NPC																														
CELLMARKERMSIGDB_ZHONG_PFC_MAJOR_TYPES_AS…	RAP1G…	BRINP3	ALCAM	LRRN3	MGLL	QKI	HES1	CRYL1	HSPB1	VIM	SEMA6D	THBS3	SPRY2	MASP1	TSPAN3	PRR5	ITGAV	ZFYVE…	EFEMP2	BCHE	LIFR	NLGN3	SLC3A2	TNFRS…	CBS	SCRG1	FXYD1	SOX2	DDR1	UBL3
CELLMARKERMSIGDB_ZHONG_PFC_MAJOR_TYPES_EX…																														
CELLMARKERMSIGDB_ZHONG_PFC_MAJOR_TYPES_MI…	BLNK	RAB3I…	IGFLR1	ARHGA…	IRAG2	AP1B1	SOCS6	SORL1	TAGAP	FLI1	ENTPD1	SIRPA	ANXA4	FTL	EGFL7	TLN1	VAV1	IPCEF1	MAF	ADA2	SLC9A9	C5AR1	PTPRE	FES	RCSD1	CNPY3	NR4A2	WASF2	NR4A1	LYN
CELLMARKERMSIGDB_ZHONG_PFC_MAJOR_TYPES_OPC																														

Table S5: Cell state marker gene list

CELLMARKERCAN_ACINAR_CELLS																														
CELLMARKERCAN_ADIPOCYTE_PROGENITOR_CELLS																														
CELLMARKERCAN_ADRENERGIC_NEURONS																														
CELLMARKERCAN_AIRWAY_EPITHELIAL_CELLS																														
CELLMARKERCAN_ALPHA_CELLS																														
CELLMARKERCAN_ALVEOLAR_MACROPHAGES																														
CELLMARKERCAN_ASTROCYTES																														
CELLMARKERCAN_B_CELLS																														
CELLMARKERCAN_B_CELLS_MEMORY																														
CELLMARKERCAN_B_CELLS_NAIVE																														
CELLMARKERCAN_BASOPHILS																														
CELLMARKERCAN_BERGMANN_GLIA																														
CELLMARKERCAN_BETA_CELLS																														
CELLMARKERCAN_CAJAL_RETZIUS_CELLS																														
CELLMARKERCAN_CARDIAC_STEM_AND_PRECURSOR_…																														
CELLMARKERCAN_CARDIOMYOCYTES																														
CELLMARKERCAN_CHOLANGIOCYTES																														
CELLMARKERCAN_CHOLINERGIC_NEURONS																														
CELLMARKERCAN_CHONDROCYTES																														
CELLMARKERCAN_CHOROID_PLEXUS_CELLS																														
CELLMARKERCAN_CILIATED_CELLS																														
CELLMARKERCAN_DELTA_CELLS																														
CELLMARKERCAN_DOPAMINERGIC_NEURONS																														
CELLMARKERCAN_DUCTAL_CELLS																														
CELLMARKERCAN_EMBRYONIC_STEM_CELLS																														
CELLMARKERCAN_ENTERIC_GLIA_CELLS																														
CELLMARKERCAN_ENTERIC_NEURONS																														
CELLMARKERCAN_ENTEROCYTES																														
CELLMARKERCAN_ENTEROENDOCRINE_CELLS																														
CELLMARKERCAN_EPENDYMAL_CELLS																														
CELLMARKERCAN_EPSILON_CELLS																														
CELLMARKERCAN_FOLLICULAR_CELLS																														
CELLMARKERCAN_FOVEOLAR_CELLS																														
CELLMARKERCAN_GAMMA_DELTA_T_CELLS																														
CELLMARKERCAN_GERM_CELLS																														
CELLMARKERCAN_GLUTAMINERGIC_NEURONS																														
CELLMARKERCAN_GRANULOSA_CELLS																														
CELLMARKERCAN_HEMATOPOIETIC_STEM_CELLS																														
CELLMARKERCAN_IMMATURE_NEURONS																														
CELLMARKERCAN_INTERNEURONS																														
CELLMARKERCAN_IONOCYTES																														
CELLMARKERCAN_KERATINOCYTES																														
CELLMARKERCAN_KUPFFER_CELLS																														
CELLMARKERCAN_LEYDIG_CELLS																														
CELLMARKERCAN_LOOP_OF_HENLE_CELLS																														
CELLMARKERCAN_LUMINAL_EPITHELIAL_CELLS																														
CELLMARKERCAN_LUTEAL_CELLS																														
CELLMARKERCAN_MEGAKARYOCYTES																														
CELLMARKERCAN_MELANOCYTES																														
CELLMARKERCAN_MESANGIAL_CELLS																														
CELLMARKERCAN_MESOTHELIAL_CELLS																														
CELLMARKERCAN_MICROFOLD_CELLS																														
CELLMARKERCAN_MICROGLIA																														
CELLMARKERCAN_MONOCYTES																														
CELLMARKERCAN_MOTOR_NEURONS																														
CELLMARKERCAN_MULLER_CELLS																														
CELLMARKERCAN_MYELOID_DERIVED_SUPPRESSOR_…																														
CELLMARKERCAN_MYOBLASTS																														
CELLMARKERCAN_MYOEPITHELIAL_CELLS																														
CELLMARKERCAN_MYOFIBROBLASTS																														
CELLMARKERCAN_NEUROBLASTS																														
CELLMARKERCAN_NEUROENDOCRINE_CELLS																														
CELLMARKERCAN_NEURONS																														
CELLMARKERCAN_NEUTROPHILS																														
CELLMARKERCAN_NK_CELLS																														
CELLMARKERCAN_NORADRENERGIC_NEURONS																														
CELLMARKERCAN_NUOCYTES																														
CELLMARKERCAN_OLIGODENDROCYTES																														
CELLMARKERCAN_OSTEOBLASTS																														
CELLMARKERCAN_OSTEOCLAST_PRECURSOR_CELLS																														
CELLMARKERCAN_OSTEOCLASTS																														
CELLMARKERCAN_OSTEOCYTES																														
CELLMARKERCAN_PANETH_CELLS																														
CELLMARKERCAN_PARATHYROID_CHIEF_CELLS																														
CELLMARKERCAN_PERICYTES																														
CELLMARKERCAN_PERITUBULAR_MYOID_CELLS																														
CELLMARKERCAN_PHOTORECEPTOR_CELLS																														
CELLMARKERCAN_PLASMA_CELLS																														
CELLMARKERCAN_PLASMACYTOID_DENDRITIC_CELLS																														
CELLMARKERCAN_PODOCYTES																														
CELLMARKERCAN_PROXIMAL_TUBULE_CELLS																														
CELLMARKERCAN_PULMONARY_ALVEOLAR_TYPE_I_C…																														
CELLMARKERCAN_PULMONARY_ALVEOLAR_TYPE_II_…																														
CELLMARKERCAN_PURKINJE_NEURONS																														
CELLMARKERCAN_RETICULOCYTES																														
CELLMARKERCAN_RETINAL_GANGLION_CELLS																														
CELLMARKERCAN_RETINAL_PROGENITOR_CELLS																														
CELLMARKERCAN_SATELLITE_CELLS																														
CELLMARKERCAN_SCHWANN_CELLS																														
CELLMARKERCAN_SEROTONERGIC_NEURONS																														
CELLMARKERCAN_SERTOLI_CELLS																														
CELLMARKERCAN_SMOOTH_MUSCLE_CELLS																														
CELLMARKERCAN_STROMAL_CELLS																														
CELLMARKERCAN_T_CELLS																														
CELLMARKERCAN_T_CYTOTOXIC_CELLS																														
CELLMARKERCAN_T_FOLLICULAR_HELPER_CELLS																														
CELLMARKERCAN_T_MEMORY_CELLS																														
CELLMARKERCAN_TRANSIENT_CELLS																														
CELLMARKERCAN_TRICHOCYTES																														
CELLMARKERCAN_TRIGEMINAL_NEURONS																														
CELLMARKERCAN_TROPHOBLAST_CELLS																														
CELLMARKERCAN_TROPHOBLAST_PROGENITOR_CELLS																														
CELLMARKERCAN_TUFT_CELLS																														
CELLMARKERCANENDOTHELIALCELLSAORTA																														
CELLMARKERCUR_N_ABNORMAL_MYELOID_CELL																														

CELLMARKERCAN_ACINAR_CELLS																														
CELLMARKERCUR_N_ABNORMAL_PLASMA_CELL																														
CELLMARKERCUR_N_ACTIVATED_DENDRITIC_CELL																														
CELLMARKERCUR_N_ACTIVATED_EFFECTOR_CELL																														
CELLMARKERCUR_N_ACTIVATED_HEPATIC_STELLAT…																														
CELLMARKERCUR_N_ACTIVATED_LANGERHANS_CELL																														
CELLMARKERCUR_N_ACTIVATED_MACROPHAGE																														
CELLMARKERCUR_N_ACTIVATED_MEMORY_T_CELL																														
CELLMARKERCUR_N_ACTIVATED_NATURAL_KILLER_…																														
CELLMARKERCUR_N_ACTIVATED_REGULATORY_T_CE…																														
CELLMARKERCUR_N_ADIPOCYTE_PROGENITOR_CELL																														
CELLMARKERCUR_N_ADRENAL_MEDULLA_CELL																														
CELLMARKERCUR_N_ADULT_SPERMATOGONIA																														
CELLMARKERCUR_N_ADVENTITIAL_CELL																														
CELLMARKERCUR_N_AEROCYTE																														
CELLMARKERCUR_N_AIRWAY_SMOOTH_MUSCLE_CELL																														
CELLMARKERCUR_N_ALVEOLAR_CELL_TYPE_1																														
CELLMARKERCUR_N_ALVEOLAR_CELL_TYPE_2																														
CELLMARKERCUR_N_ALVEOLAR_EPITHELIAL_CELL_…																														
CELLMARKERCUR_N_ALVEOLAR_EPITHELIAL_CELL_…																														
CELLMARKERCUR_N_ALVEOLAR_PNEUMOCYTE_TYPE_I																														
CELLMARKERCUR_N_ALVEOLAR_PNEUMOCYTE_TYPE_…																														
CELLMARKERCUR_N_AMACRINE_CELL																														
CELLMARKERCUR_N_AMNIOTIC_FLUID_STEM_CELL																														
CELLMARKERCUR_N_ANTERIOR_CELL																														
CELLMARKERCUR_N_ANTERIOR_HINDBRAIN_CELL																														
CELLMARKERCUR_N_ANTI_TUMOR_IMMUNE_CELL																														
CELLMARKERCUR_N_ANTIGEN_PRESENTATION_CANC…																														
CELLMARKERCUR_N_ASTROCYTE																														
CELLMARKERCUR_N_ATRIAL_CELL																														
CELLMARKERCUR_N_ATRIOVENTRICULAR_CANAL_CE…																														
CELLMARKERCUR_N_ATYPICAL_MEMORY_B_CELL																														
CELLMARKERCUR_N_B_2_CELL																														
CELLMARKERCUR_N_B1_CELL																														
CELLMARKERCUR_N_B10_REGULATORY_B_CELL																														
CELLMARKERCUR_N_BASAL_CELL																														
CELLMARKERCUR_N_BASAL_EPITHELIAL_CELL																														
CELLMARKERCUR_N_BASAL_KERATINOCYTE																														
CELLMARKERCUR_N_BASAL_LIKE_CELL																														
CELLMARKERCUR_N_BASAL_PROGENITOR_CELL																														
CELLMARKERCUR_N_BASOPHIL																														
CELLMARKERCUR_N_BILIARY_CELL																														
CELLMARKERCUR_N_BONE_MARROW_STEM_CELL																														
CELLMARKERCUR_N_BONE_SPECIFIC_CELL																														
CELLMARKERCUR_N_BRAIN_CELL																														
CELLMARKERCUR_N_CANCER_CELL	PRSS1	TYR	MITF	RANBP2	S100A1	ARID1A	APC	BIRC5	CDK1	SMARC…	COL3A1	PDGFRB	CDH17	NTRK3	CD8A	MPO	BATF3	TMOD1												
CELLMARKERCUR_N_CANCER_STEM_LIKE_CELL																														
CELLMARKERCUR_N_CAPILLARY_CELL																														
CELLMARKERCUR_N_CAR_T_CELL																														
CELLMARKERCUR_N_CARDIAC_CELL																														
CELLMARKERCUR_N_CARDIOMYOCYTE																														
CELLMARKERCUR_N_CARDIOSPHERE_DERIVED_CELL																														
CELLMARKERCUR_N_CARDIOVASCULAR_PROGENITOR…																														
CELLMARKERCUR_N_CAUDAL_CORTEX_CELL																														
CELLMARKERCUR_N_CD14_MONOCYTE																														
CELLMARKERCUR_N_CD16_MONOCYTE																														
CELLMARKERCUR_N_CD4_CD28_T_CELL																														
CELLMARKERCUR_N_CD4_PERIPHERALLY_DERIVED_…																														
CELLMARKERCUR_N_CD4_T_CELL																														
CELLMARKERCUR_N_CD56BRIGHT_NATURAL_KILLER…																														
CELLMARKERCUR_N_CD56DIM_NATURAL_KILLER_CE…																														
CELLMARKERCUR_N_CD8_T_CELL																														
CELLMARKERCUR_N_CENTRAL_MEMORY_T_CELL																														
CELLMARKERCUR_N_CENTRAL_MEMORY_T_HELPER_1…																														
CELLMARKERCUR_N_CENTRAL_NERVOUS_SYSTEM_ST…																														
CELLMARKERCUR_N_CHANDELIER_CELL																														
CELLMARKERCUR_N_CHIEF_CELL																														
CELLMARKERCUR_N_CHONDROCYTE																														
CELLMARKERCUR_N_CHROMAFFIN_CELL																														
CELLMARKERCUR_N_CIRCULATING_ANGIOGENIC_CE…																														
CELLMARKERCUR_N_CIRCULATING_FETAL_CELL																														
CELLMARKERCUR_N_CIRCULATING_NATURAL_KILLE…																														
CELLMARKERCUR_N_CIRCULATING_PRECURSOR_CELL																														
CELLMARKERCUR_N_CIRCULATING_PROGENITOR_CE…																														
CELLMARKERCUR_N_CIRCULATING_T_FOLLICULAR_…																														
CELLMARKERCUR_N_CIRCULATING_TUMOR_CELL																														
CELLMARKERCUR_N_CLASS_SWITCHED_PLASMABLAST																														
CELLMARKERCUR_N_CLASSICAL_B_CELL																														
CELLMARKERCUR_N_CLASSICAL_CANCER_ASSOCIAT…																														
CELLMARKERCUR_N_CLASSICAL_MONOCYTE																														
CELLMARKERCUR_N_CLAUDIN_LOW_CELL																														
CELLMARKERCUR_N_COLLECTING_DUCTAL_CELL																														
CELLMARKERCUR_N_COLONCYTE																														
CELLMARKERCUR_N_COLORECTAL_STEM_CELL																														
CELLMARKERCUR_N_COLUMNAR_EPITHELIAL_CELL																														
CELLMARKERCUR_N_COMMON_LYMPHOID_PROGENITO…																														
CELLMARKERCUR_N_COMMON_MYELOID_PROGENITOR…																														
CELLMARKERCUR_N_COMPLEMENT_SECRETING_CANC…																														
CELLMARKERCUR_N_CONE_PHOTORECEPTOR_CELL																														
CELLMARKERCUR_N_CONNECTING_TUBULAR_CELL																														
CELLMARKERCUR_N_CONVENTIONAL_CD4_T_CELL																														
CELLMARKERCUR_N_CORNEAL_ENDOTHELIAL_CELL																														
CELLMARKERCUR_N_CORNEAL_EPITHELIAL_CELL																														
CELLMARKERCUR_N_CORNEOCYTE_LIKE_CELL																														
CELLMARKERCUR_N_CORTICAL_CELL																														
CELLMARKERCUR_N_CORTICAL_STROMAL_CELL																														
CELLMARKERCUR_N_CORTICOTROPH_TUMOR_CELL																														
CELLMARKERCUR_N_CRANIAL_NEURAL_CREST_CELL																														
CELLMARKERCUR_N_CRYPT_CELL																														
CELLMARKERCUR_N_CRYPT_STEM_CELL																														
CELLMARKERCUR_N_CYCLING_B_CELL																														
CELLMARKERCUR_N_CYCLING_EPITHELIAL_CELL																														
CELLMARKERCUR_N_CYTOTOXIC_CD8_T_CELL																														
CELLMARKERCUR_N_CYTOTOXIC_DOUBLE_POSITIVE…																														
CELLMARKERCUR_N_CYTOTOXIC_T_CELL																														

CELLMARKERCAN_ACINAR_CELLS																														
CELLMARKERCUR_N_CYTOTOXIC_T_CELL_2																														
CELLMARKERCUR_N_DAMAGE_ASSOCIATED_TRANSIE…																														
CELLMARKERCUR_N_DARK_ZONE_CELL																														
CELLMARKERCUR_N_DEDIFFERENTIATION_ADIPOCY…																														
CELLMARKERCUR_N_DEEP_LAYER_NEURON																														
CELLMARKERCUR_N_DEFINITIVE_ENDODERM_CELL																														
CELLMARKERCUR_N_DELTA_CELL																														
CELLMARKERCUR_N_DEMYELINATING_MICROGLIAL_…																														
CELLMARKERCUR_N_DENDRITIC_CELL_2A																														
CELLMARKERCUR_N_DENDRITIC_CELL_LINEAGE																														
CELLMARKERCUR_N_DENTAL_PULP_CELL																														
CELLMARKERCUR_N_DERMAL_CELL																														
CELLMARKERCUR_N_DEUTEROSOMAL_CELL																														
CELLMARKERCUR_N_DIFFUSE_GASTRIC_CANCER_CE…																														
CELLMARKERCUR_N_DISTAL_CELL																														
CELLMARKERCUR_N_DISTAL_STEM_CELL																														
CELLMARKERCUR_N_DISTAL_TUBULAR_CELL																														
CELLMARKERCUR_N_DIVIDING_CELL																														
CELLMARKERCUR_N_DIVIDING_PLASMA_B_CELL																														
CELLMARKERCUR_N_DIVIDING_T_CELL																														
CELLMARKERCUR_N_DOPAMINERGIC_NEURON																														
CELLMARKERCUR_N_DORSAL_ANTERIOR_HINDBRAIN…																														
CELLMARKERCUR_N_DORSAL_LATERAL_GANGLIONIC…																														
CELLMARKERCUR_N_DORSAL_MEDIAL_GANGLIONIC_…																														
CELLMARKERCUR_N_DOUBLE_NEGATIVE_B_CELL																														
CELLMARKERCUR_N_DOUBLE_NEGATIVE_T_CELL																														
CELLMARKERCUR_N_DOUBLE_POSITIVE_T_CELL																														
CELLMARKERCUR_N_DOUBLE_POSITIVE_TREG_CELL																														
CELLMARKERCUR_N_EARLY_HEMANGIOBLAST																														
CELLMARKERCUR_N_EARLY_PROXIMAL_TUBULE_CELL																														
CELLMARKERCUR_N_EARLY_RESPONSE_SECRETORY_…																														
CELLMARKERCUR_N_EFFECTOR_CHONDROCYTE																														
CELLMARKERCUR_N_EFFECTOR_MEMORY_T_CELL																														
CELLMARKERCUR_N_EFFECTOR_NATURAL_KILLER_C…																														
CELLMARKERCUR_N_ELONGATING_CELL																														
CELLMARKERCUR_N_EMBRYONIC_CELL																														
CELLMARKERCUR_N_EMBRYONIC_STEM_CELL																														
CELLMARKERCUR_N_ENDOCARDIAL_CELL																														
CELLMARKERCUR_N_ENDOCRINE_PROGENITOR_CELL																														
CELLMARKERCUR_N_ENDODERM_PROGENITOR_CELL																														
CELLMARKERCUR_N_ENDODERMAL_CELL																														
CELLMARKERCUR_N_ENDOMETRIAL_STROMAL_CELL																														
CELLMARKERCUR_N_ENDOMETRIAL_STROMAL_STEM_…																														
CELLMARKERCUR_N_ENDOTHELIAL_PRECURSOR_CELL																														
CELLMARKERCUR_N_ENSHEATHING_GLIAL_CELL																														
CELLMARKERCUR_N_ENTERIC_NEURON																														
CELLMARKERCUR_N_EOSINOPHIL																														
CELLMARKERCUR_N_EPENDYMAL_CELL																														
CELLMARKERCUR_N_EPIBLAST_CELL																														
CELLMARKERCUR_N_EPICARDIAL_CELL																														
CELLMARKERCUR_N_EPICARDIUM_DERIVED_CELL																														
CELLMARKERCUR_N_EPIDERMAL_CELL																														
CELLMARKERCUR_N_EPIDERMAL_STEM_CELL																														
CELLMARKERCUR_N_EPITHELIAL_CELL	TG	DIO2	TWIST1	CLDN1	DSP	CTNNB1	GJB3	GJB2	GJA1	DDR1	PROM1	ERBB2	SOX9	VDR	EHF	KLF4	F11R	WNT7A	PLCG1	TGFBR2	CBX4	NGFR	PRSS8	HR	CDSN	PRDM1	KLF5	GRHL3	RNF186	PAX8
CELLMARKERCUR_N_EPITHELIAL_DUCTAL_CELL																														
CELLMARKERCUR_N_EPITHELIAL_MESENCHYMAL_CE…																														
CELLMARKERCUR_N_EPITHELIOID_MACROPHAGE																														
CELLMARKERCUR_N_EPSILON_CELL																														
CELLMARKERCUR_N_ERYTHROBLAST																														
CELLMARKERCUR_N_ERYTHROID_CELL																														
CELLMARKERCUR_N_ERYTHROID_PROGENITOR_CELL																														
CELLMARKERCUR_N_ESOPHAGEAL_CELL																														
CELLMARKERCUR_N_ESOPHAGEAL_PROGENITOR_CELL																														
CELLMARKERCUR_N_EXCITATORY_NEURON																														
CELLMARKERCUR_N_EXHAUSTED_DOUBLE_POSITIVE…																														
CELLMARKERCUR_N_EXTRAVILLOUS_TROPHOBLAST																														
CELLMARKERCUR_N_F_PERICYTE																														
CELLMARKERCUR_N_FETAL_MYOBLAST																														
CELLMARKERCUR_N_FIBROBLAST_LIKE_CELL																														
CELLMARKERCUR_N_FIBROCHONDROCYTE																														
CELLMARKERCUR_N_FIBROCHONDROCYTE_PROGENIT…																														
CELLMARKERCUR_N_FOAM_CELL																														
CELLMARKERCUR_N_FOLLICULAR_B_CELL																														
CELLMARKERCUR_N_FOLLICULAR_CELL																														
CELLMARKERCUR_N_FOLLICULAR_DENDRITIC_CELL																														
CELLMARKERCUR_N_FOREBRAIN_CELL																														
CELLMARKERCUR_N_GABAERGIC_NEURON																														
CELLMARKERCUR_N_GANGLION_CELL																														
CELLMARKERCUR_N_GASTRIC_NEUROENDOCRINE_CE…																														
CELLMARKERCUR_N_GASTRIC_STEM_CELL																														
CELLMARKERCUR_N_GERM_CELL																														
CELLMARKERCUR_N_GERMINAL_CENTER_B_CELL																														
CELLMARKERCUR_N_GERMINAL_CENTER_CELL																														
CELLMARKERCUR_N_GERMINAL_CENTRE_B_CELL																														
CELLMARKERCUR_N_GLIAL_CELL																														
CELLMARKERCUR_N_GLIOMA_STEM_CELL																														
CELLMARKERCUR_N_GLOBOSE_BASAL_CELL																														
CELLMARKERCUR_N_GLOMERULAR_PARIETAL_EPITH…																														
CELLMARKERCUR_N_GLUTAMATERGIC_NEURON																														
CELLMARKERCUR_N_GOBLET_CELL																														
CELLMARKERCUR_N_GONADOTROPE																														
CELLMARKERCUR_N_GONADOTROPH_CELL																														
CELLMARKERCUR_N_GRANULOCYTE																														
CELLMARKERCUR_N_GRANULOCYTE_MONOCYTE_PROG…																														
CELLMARKERCUR_N_GRANULOCYTIC_UNK																														
CELLMARKERCUR_N_GRANULOSA_CELL																														
CELLMARKERCUR_N_HAEMATOPOIETIC_CELL																														
CELLMARKERCUR_N_HAIR_FOLLICLE_CELL																														
CELLMARKERCUR_N_HEMANGIOBLAST																														
CELLMARKERCUR_N_HEMATOPOIETIC_CELL																														
CELLMARKERCUR_N_HEMATOPOIETIC_PRECURSOR_C…																														
CELLMARKERCUR_N_HEMATOPOIETIC_PROGENITOR_…																														
CELLMARKERCUR_N_HEPATIC_CELL																														
CELLMARKERCUR_N_HEPATIC_PROGENITOR_CELL																														

CELLMARKERCAN_ACINAR_CELLS																														
CELLMARKERCUR_N_HEPATIC_STELLATE_CELL																														
CELLMARKERCUR_N_HEPATOBLAST																														
CELLMARKERCUR_N_HEPATOCYTE																														
CELLMARKERCUR_N_HISTIOCYTE																														
CELLMARKERCUR_N_HMSC_TREATED_B_CELL																														
CELLMARKERCUR_N_HOFBAUER_CELL																														
CELLMARKERCUR_N_HOMEOSTATIC_CHONDROCYTE																														
CELLMARKERCUR_N_HOMEOSTATIC_MICROGLIAL_CE…																														
CELLMARKERCUR_N_HORIZONTAL_BASAL_CELL																														
CELLMARKERCUR_N_HORIZONTAL_CELL																														
CELLMARKERCUR_N_HYPOBLAST_CELL																														
CELLMARKERCUR_N_IDIOPATHIC_PULMONARY_FIBR…																														
CELLMARKERCUR_N_IGA_MEMORY_B_CELL																														
CELLMARKERCUR_N_IGG_MEMORY_B_CELL																														
CELLMARKERCUR_N_IGM_MEMORY_B_CELL																														
CELLMARKERCUR_N_IGM_ONLY_B_CELL																														
CELLMARKERCUR_N_IL7R_T_HELPER_CELL																														
CELLMARKERCUR_N_IMMATURE_ERYTHROID_CELL																														
CELLMARKERCUR_N_IMMATURE_MYELOID_CELL																														
CELLMARKERCUR_N_IMMATURE_NEUTROPHIL																														
CELLMARKERCUR_N_IMMATURE_TRANSITIONAL_B_C…																														
CELLMARKERCUR_N_IMMUNE_CELL																														
CELLMARKERCUR_N_INDUCED_PLURIPOTENT_STEM_…																														
CELLMARKERCUR_N_INFILTRATING_MACROPHAGE																														
CELLMARKERCUR_N_INFLAMMATORY_CANCER_ASSOC…																														
CELLMARKERCUR_N_INFLAMMATORY_DENDRITIC_CE…																														
CELLMARKERCUR_N_INFLAMMATORY_MONOCYTE																														
CELLMARKERCUR_N_INHIBITORY_NEURON																														
CELLMARKERCUR_N_INNATE_LYMPHOID_CELL																														
CELLMARKERCUR_N_INNER_CELL_MASS_CELL																														
CELLMARKERCUR_N_INNER_RADIAL_GLIAL_CELL																														
CELLMARKERCUR_N_INTER_MEDIATE_PROGENITOR_…																														
CELLMARKERCUR_N_INTERCALATED_CELL																														
CELLMARKERCUR_N_INTERFERON_RESPONSIVE_SEC…																														
CELLMARKERCUR_N_INTERMEDIATE_MONOCYTE																														
CELLMARKERCUR_N_INTERMEDIATE_MONOCYTE_CELL																														
CELLMARKERCUR_N_INTERSTITIAL_MACROPHAGE																														
CELLMARKERCUR_N_INTERSTITIAL_PROGENITOR_C…																														
CELLMARKERCUR_N_INTESTINAL_EPITHELIAL_STE…																														
CELLMARKERCUR_N_INTESTINAL_GASTRIC_CANCER…																														
CELLMARKERCUR_N_INTESTINAL_METAPLASIA_CELL																														
CELLMARKERCUR_N_INTRAEPITHELIAL_LYMPHOCYT…																														
CELLMARKERCUR_N_IONOCYTE																														
CELLMARKERCUR_N_IRIS_PUTTATIVE_STEM_CELL																														
CELLMARKERCUR_N_KUPFFER_CELL																														
CELLMARKERCUR_N_LACTOTROPH_CELL																														
CELLMARKERCUR_N_LANGERHANS_CELL																														
CELLMARKERCUR_N_LEUKOCYTE																														
CELLMARKERCUR_N_LIMBAL_EPITHELIAL_STEM_CE…																														
CELLMARKERCUR_N_LIMBAL_STEM_CELL																														
CELLMARKERCUR_N_LIPOFIBROBLAST																														
CELLMARKERCUR_N_LIVER_PROGENITOR_CELL																														
CELLMARKERCUR_N_LIVER_STEM_CELL																														
CELLMARKERCUR_N_LMMLEYDIG_CELL																														
CELLMARKERCUR_N_LONOCYTE																														
CELLMARKERCUR_N_LOOP_OF_HENLE_CELL																														
CELLMARKERCUR_N_LOOP_PROGENITOR_CELL																														
CELLMARKERCUR_N_LOW_DENSITY_NEUTROPHIL																														
CELLMARKERCUR_N_LUMINAL_PROGENITOR_CELL																														
CELLMARKERCUR_N_LUNG_CELL																														
CELLMARKERCUR_N_LYMPH_NODE_CELL																														
CELLMARKERCUR_N_LYMPHATIC_CELL																														
CELLMARKERCUR_N_LYMPHATIC_ENDOTHELIAL_CELL																														
CELLMARKERCUR_N_LYMPHOBLASTOID_CELL																														
CELLMARKERCUR_N_LYMPHOCYTE																														
CELLMARKERCUR_N_LYMPHOID_CELL																														
CELLMARKERCUR_N_LYMPHOID_PROGENITOR_CELL																														
CELLMARKERCUR_N_LYMPHOID_STRUCTURE_CELL																														
CELLMARKERCUR_N_M_PERICYTE																														
CELLMARKERCUR_N_M1_MACROPHAGE																														
CELLMARKERCUR_N_M1_MICROGLIAL_CELL																														
CELLMARKERCUR_N_M2_MACROPHAGE																														
CELLMARKERCUR_N_MACROGLIAL_CELL																														
CELLMARKERCUR_N_MALIGNANT_CELL																														
CELLMARKERCUR_N_MALIGNANT_PLASMA_CELL																														
CELLMARKERCUR_N_MARGINAL_ASSOCIATED_MACRO…																														
CELLMARKERCUR_N_MAST_CELL																														
CELLMARKERCUR_N_MATURE_ASTROCYTE																														
CELLMARKERCUR_N_MATURE_B_CELL																														
CELLMARKERCUR_N_MATURE_DENDRITIC_CELL																														
CELLMARKERCUR_N_MATURE_ENDOTHELIAL_CELL																														
CELLMARKERCUR_N_MATURE_LUMINAL_CELL																														
CELLMARKERCUR_N_MATURE_NEURON																														
CELLMARKERCUR_N_MATURE_NEURONAL																														
CELLMARKERCUR_N_MATURE_OLIGODENDROCYTE																														
CELLMARKERCUR_N_MATURE_OSTEOBLAST																														
CELLMARKERCUR_N_MATURE_PODOCYTE																														
CELLMARKERCUR_N_MEDIAL_AND_DISTAL_SEGMENT…																														
CELLMARKERCUR_N_MEDIAL_GANGLIONIC_EMINENC…																														
CELLMARKERCUR_N_MEGAKARYOCYTE																														
CELLMARKERCUR_N_MEGAKARYOCYTE_ERYTHROID_C…																														
CELLMARKERCUR_N_MEGAKARYOCYTE_ERYTHROID_P…																														
CELLMARKERCUR_N_MEGAKARYOCYTE_PROGENITOR_…																														
CELLMARKERCUR_N_MELANOMA_CELL																														
CELLMARKERCUR_N_MEMORY_B_CELL																														
CELLMARKERCUR_N_MEMORY_B_CELL_PRECURSOR																														
CELLMARKERCUR_N_MEMORY_CD27_B_CELL																														
CELLMARKERCUR_N_MEMORY_CD4_T_CELL																														
CELLMARKERCUR_N_MEMORY_DOUBLE_POSITIVE_T_…																														
CELLMARKERCUR_N_MEMORY_REGULATORY_T_CELL																														
CELLMARKERCUR_N_MEMORY_T_CELL																														
CELLMARKERCUR_N_MERKEL_CELL																														
CELLMARKERCUR_N_MESENCHYMAL_CELL																														
CELLMARKERCUR_N_MESENCHYMAL_EPITHELIAL_TR…																														

CELLMARKERCAN_ACINAR_CELLS																														
CELLMARKERCUR_N_MESENCHYMAL_GLIOBLASTOMA_…																														
CELLMARKERCUR_N_MESENCHYMAL_STEM_CELL																														
CELLMARKERCUR_N_MESENCHYMAL_STROMAL_CELL																														
CELLMARKERCUR_N_MESENDODERMAL_CELL																														
CELLMARKERCUR_N_MESODERMAL_EMBRYONIC_STEM…																														
CELLMARKERCUR_N_MESODERMAL_PROGENITOR_CELL																														
CELLMARKERCUR_N_METAPLASTIC_STEM_CELL																														
CELLMARKERCUR_N_MEYLOID_CELL																														
CELLMARKERCUR_N_MICROGLIAL_CELL	RPL38	RPL10A	RPS3A	ZNF662	CSTB	RPL14	CD5	RPL3	SDF2L1	NRP1	ARRDC4	RPL22	FXYD6	PRDX1	MEIS1	RPS2	IL18BP	VPS13C	ACTR3B	APLP2	ANXA5	PLBD2	RPL13	CRLF2	CPD	RPL27A	LSR	VAT1	RAMP1	RPL35
CELLMARKERCUR_N_MICROVILLAR_CELL																														
CELLMARKERCUR_N_MIDBRAIN_DOPAMINERGIC_NEU…																														
CELLMARKERCUR_N_MONO_MACROPHAGE																														
CELLMARKERCUR_N_MONOCYTE_DERIVED_CELL																														
CELLMARKERCUR_N_MONOCYTE_DERIVED_MYELOID_…																														
CELLMARKERCUR_N_MONOCYTE_LINEAGE																														
CELLMARKERCUR_N_MONOCYTE_PRECURSOR																														
CELLMARKERCUR_N_MONOCYTE_PROGENITOR_CELL																														
CELLMARKERCUR_N_MUELLER_GLIA_CELL																														
CELLMARKERCUR_N_MULTIPOTENT_MESENCHYMAL_S…																														
CELLMARKERCUR_N_MULTIPOTENT_RETINAL_PROGE…																														
CELLMARKERCUR_N_MULTIPOTENT_STEM_CELL																														
CELLMARKERCUR_N_MULTIPOTENT_STROMAL_CELL																														
CELLMARKERCUR_N_MUSCLE_CELL																														
CELLMARKERCUR_N_MUSCLE_DERIVED_CELL																														
CELLMARKERCUR_N_MUSCLE_SATELLITE_CELL																														
CELLMARKERCUR_N_MYELOBLAST																														
CELLMARKERCUR_N_MYELOID_DENDRITIC_CELL																														
CELLMARKERCUR_N_MYELOID_DENDRITIC_CELL_1																														
CELLMARKERCUR_N_MYELOID_DERIVED_CELL																														
CELLMARKERCUR_N_MYELOID_DERIVED_SUPPRESSO…																														
CELLMARKERCUR_N_MYELOID_PROGENITOR_CELL																														
CELLMARKERCUR_N_MYO_EPITHELIAL_CELL																														
CELLMARKERCUR_N_MYOBLAST																														
CELLMARKERCUR_N_MYOCYTE																														
CELLMARKERCUR_N_MYOFIBROBLAST																														
CELLMARKERCUR_N_MYOGENIC_ENDOTHELIAL_CELL																														
CELLMARKERCUR_N_MYOGENIC_PROGENITOR_CELL																														
CELLMARKERCUR_N_MYOID_CELL																														
CELLMARKERCUR_N_NAIVE_B_CELL																														
CELLMARKERCUR_N_NAIVE_CD8_T_CELL																														
CELLMARKERCUR_N_NAIVE_DOUBLE_POSITIVE_T_C…																														
CELLMARKERCUR_N_NAIVE_PROGENITOR_CELL																														
CELLMARKERCUR_N_NAIVE_T_CELL																														
CELLMARKERCUR_N_NASCENT_NEPHRON_CELL																														
CELLMARKERCUR_N_NATURAL_KILLER_CD56_BRIGH…																														
CELLMARKERCUR_N_NATURAL_KILLER_CD56_DIM_C…																														
CELLMARKERCUR_N_NEGATIVE_MESENCHYMAL_STEM…																														
CELLMARKERCUR_N_NEURAL_CELL																														
CELLMARKERCUR_N_NEURAL_CREST_STEM_CELL																														
CELLMARKERCUR_N_NEURAL_CREST_STEMLIKE_CELL																														
CELLMARKERCUR_N_NEURAL_PRECURSOR_CELL																														
CELLMARKERCUR_N_NEURAL_STEM_CELL																														
CELLMARKERCUR_N_NEUROECTODERM_CELL																														
CELLMARKERCUR_N_NEURONAL_INTERMEDIATE_PRO…																														
CELLMARKERCUR_N_NEURONAL_PROGENITOR_CELL																														
CELLMARKERCUR_N_NEUTROPHIL_PROGENITOR_CELL																														
CELLMARKERCUR_N_NON_NEURON																														
CELLMARKERCUR_N_NON_PT_TUBULAR_CELL																														
CELLMARKERCUR_N_NON_RECIRCULATING_TISSUE_…																														
CELLMARKERCUR_N_NONMYELINATING_SCHWANN_CE…																														
CELLMARKERCUR_N_NOTOCHORD_CELL																														
CELLMARKERCUR_N_NUCLEATED_CELL																														
CELLMARKERCUR_N_NUCLEUS_PULPOSUS_PROGENIT…																														
CELLMARKERCUR_N_OLIGODENDROCYTE																														
CELLMARKERCUR_N_OSTEOBLAST																														
CELLMARKERCUR_N_OUTER_RADIAL_GLIA																														
CELLMARKERCUR_N_OVALBUMIN_SPECIFIC_REGULA…																														
CELLMARKERCUR_N_OVARIAN_CELL																														
CELLMARKERCUR_N_OVARIAN_STROMAL_CELL																														
CELLMARKERCUR_N_P53_LIKE_CELL																														
CELLMARKERCUR_N_PAN_B_CELL																														
CELLMARKERCUR_N_PAN_CANCER_ASSOCIATED_FIB…																														
CELLMARKERCUR_N_PAN_MACROPHAGE																														
CELLMARKERCUR_N_PAN_MONOCYTE																														
CELLMARKERCUR_N_PAN_NEURONAL_CELL																														
CELLMARKERCUR_N_PAN_TROPHECTODERM_CELL																														
CELLMARKERCUR_N_PANCREATIC_ENDODERM_CELL																														
CELLMARKERCUR_N_PANCREATIC_POLYPEPTIDE_CE…																														
CELLMARKERCUR_N_PANCREATIC_PROGENITOR_CELL																														
CELLMARKERCUR_N_PANCREATIC_STEM_CELL																														
CELLMARKERCUR_N_PANETH_CELL																														
CELLMARKERCUR_N_PANNEURON																														
CELLMARKERCUR_N_PANVASCULAR_CELL																														
CELLMARKERCUR_N_PAPILLARY_FIBROBLAST																														
CELLMARKERCUR_N_PARIETAL_CELL																														
CELLMARKERCUR_N_PARVALBUMIN_INTERNEURON																														
CELLMARKERCUR_N_PATELET																														
CELLMARKERCUR_N_PATHOGENIC_T_PERIPHERAL_H…																														
CELLMARKERCUR_N_PELVIC_EPITHELIAL_CELL																														
CELLMARKERCUR_N_PERICYTE																														
CELLMARKERCUR_N_PERIODONTAL_LIGAMENT_STEM…																														
CELLMARKERCUR_N_PERIOSTEUM_DERIVED_PROGEN…																														
CELLMARKERCUR_N_PERITUBULAR_MYOID_CELL																														
CELLMARKERCUR_N_PERIVASCULAR_LIKE_CELL																														
CELLMARKERCUR_N_PHAGOCYTE																														
CELLMARKERCUR_N_PHOTORECEPTOR_CELL																														
CELLMARKERCUR_N_PIT_CELL																														
CELLMARKERCUR_N_PIT_MUCOUS_CELL																														
CELLMARKERCUR_N_PITUITARY_STEM_CELL																														
CELLMARKERCUR_N_PLACENTAL_TISSUE_CELL																														
CELLMARKERCUR_N_PLACODE_CELL																														
CELLMARKERCUR_N_PLASMABLAST_B_CELL																														
CELLMARKERCUR_N_PLATELET																														
CELLMARKERCUR_N_PLURIPOTENT_STEM_CELL																														

CELLMARKERCAN_ACINAR_CELLS																														
CELLMARKERCUR_N_PODOCYTE_PRECURSOR																														
CELLMARKERCUR_N_PORTAL_FIBROBLAST																														
CELLMARKERCUR_N_POSTERIOR_CELL																														
CELLMARKERCUR_N_PRE_B_CELL																														
CELLMARKERCUR_N_PRE_CONVENTIONAL_DENDRITI…																														
CELLMARKERCUR_N_PRE_DENDRITIC_CELL																														
CELLMARKERCUR_N_PRECURSOR_CELL																														
CELLMARKERCUR_N_PREHYPERTROPHIC_CHONDROCY…																														
CELLMARKERCUR_N_PRESOMITE_CELL																														
CELLMARKERCUR_N_PRETUBULAR_AGGREGATE_CELL																														
CELLMARKERCUR_N_PRIMITIVE_ENDODERM_CELL																														
CELLMARKERCUR_N_PRINCIPAL_CELL																														
CELLMARKERCUR_N_PRO_B_CELL																														
CELLMARKERCUR_N_PRO_INFLAMMATORY_MACROPHA…																														
CELLMARKERCUR_N_PRO_NEUTROPHIL																														
CELLMARKERCUR_N_PROLIFERATING_CELL																														
CELLMARKERCUR_N_PROLIFERATIVE_CHONDROCYTE																														
CELLMARKERCUR_N_PROLIFERATIVE_DUCTAL_CELL																														
CELLMARKERCUR_N_PROLIFERATIVE_NEURAL_PROG…																														
CELLMARKERCUR_N_PRONEURAL_GLIOBLASTOMA_CE…																														
CELLMARKERCUR_N_PROSPERMATOGONIA																														
CELLMARKERCUR_N_PROSTATE_CELL																														
CELLMARKERCUR_N_PROXIMAL_CELL																														
CELLMARKERCUR_N_PROXIMAL_SEGMENT_CELL																														
CELLMARKERCUR_N_PROXIMAL_STEM_CELL																														
CELLMARKERCUR_N_PROXIMAL_TUBULE_CELL																														
CELLMARKERCUR_N_PULMONARY_NEUROENDOCRINE_…																														
CELLMARKERCUR_N_PYRAMIDAL_CELL																														
CELLMARKERCUR_N_QUIESCENT_T_STEM_CELL																														
CELLMARKERCUR_N_REGULATORY_B_CELL																														
CELLMARKERCUR_N_REGULATORY_INNATE_LYMPHOI…																														
CELLMARKERCUR_N_RENAL_TUBULAR_CELL																														
CELLMARKERCUR_N_RESIDENT_MEMORY_T_CELL																														
CELLMARKERCUR_N_RESPIRATORY_HORIZONTAL_BA…																														
CELLMARKERCUR_N_RESPIRATORY_SECRETORY_CELL																														
CELLMARKERCUR_N_RESPONDER_T_CELL																														
CELLMARKERCUR_N_RESPONDING_CONVENTIONAL_T…																														
CELLMARKERCUR_N_RESTING_MEMORY_B_CELL																														
CELLMARKERCUR_N_RESTING_NAIVE_B_CELL																														
CELLMARKERCUR_N_RESTING_REGULATORY_T_CELL																														
CELLMARKERCUR_N_RETICULAR_FIBROBLAST																														
CELLMARKERCUR_N_RETINA_CELL																														
CELLMARKERCUR_N_ROD_CELL																														
CELLMARKERCUR_N_SATELLITE_CELL																														
CELLMARKERCUR_N_SEPTUM_TRANSVERSUM_MESENC…																														
CELLMARKERCUR_N_SEROUS_CELL																														
CELLMARKERCUR_N_SIDE_POPULATION_CELL																														
CELLMARKERCUR_N_SKELETAL_MUSCLE_CELL																														
CELLMARKERCUR_N_SKELETAL_MUSCLE_STEM_CELL																														
CELLMARKERCUR_N_SMOOTH_MUSCLE_CELL																														
CELLMARKERCUR_N_SMOOTH_MUSCLE_LIKE_CELL																														
CELLMARKERCUR_N_SOMATIC_CELL																														
CELLMARKERCUR_N_SOMATIC_STEM_CELL																														
CELLMARKERCUR_N_SOMATOSTATIN_INTERNEURON																														
CELLMARKERCUR_N_SPECIALIST_ANTIGEN_PRESEN…																														
CELLMARKERCUR_N_SPERMATOGENIC_CELL																														
CELLMARKERCUR_N_SPERMATOGONIAL_CELL																														
CELLMARKERCUR_N_SPERMATOGONIAL_STEM_CELL																														
CELLMARKERCUR_N_SQUAMOUS_DIFFERENTIATION_…																														
CELLMARKERCUR_N_STELLATE_CELL																														
CELLMARKERCUR_N_STEM_CELL_DERIVED_ALPHA_C…																														
CELLMARKERCUR_N_STEM_CELL_DERIVED_BETA_CE…																														
CELLMARKERCUR_N_STEM_CELL_DERIVED_ENDOTHE…																														
CELLMARKERCUR_N_STEM_LIKE_CD8_T_CELL																														
CELLMARKERCUR_N_STROMAL_CELL																														
CELLMARKERCUR_N_SUBPALLIUM_CELL																														
CELLMARKERCUR_N_SUBPLATE_CELL																														
CELLMARKERCUR_N_SUPPRESSIVE_MONOCYTE																														
CELLMARKERCUR_N_SUPPRESSOR_T_CELL																														
CELLMARKERCUR_N_SUPRABASAL_CELL																														
CELLMARKERCUR_N_SURFACE_ECTODERM_CELL																														
CELLMARKERCUR_N_SUSTENTACULAR_CELL																														
CELLMARKERCUR_N_SYMPATHOADRENAL_CELL																														
CELLMARKERCUR_N_SYNCYTIOTROPHOBLAST																														
CELLMARKERCUR_N_SYNTHETIC_SMOOTH_MUSCLE_C…																														
CELLMARKERCUR_N_T_CELL_LARGE_GRANULAR_LYM…																														
CELLMARKERCUR_N_T_CELL_LINEAGE																														
CELLMARKERCUR_N_T_HELPER_CELL																														
CELLMARKERCUR_N_T_MEMORY_STEM_CELL																														
CELLMARKERCUR_N_T2_MZP_REGULATORY_B_CELL																														
CELLMARKERCUR_N_TENDON_CELL																														
CELLMARKERCUR_N_TERMINAL_DIFFERENTIATED_B…																														
CELLMARKERCUR_N_THALAMIC_CELL																														
CELLMARKERCUR_N_THECA_INTERNA_CELL																														
CELLMARKERCUR_N_THYMIC_EPITHELIAL_CELL																														
CELLMARKERCUR_N_TISSUE_LIKE_MEMORY_B_CELL																														
CELLMARKERCUR_N_TISSUE_RESIDENT_CELL																														
CELLMARKERCUR_N_TISSUE_RESIDENT_EFFECTOR_…																														
CELLMARKERCUR_N_TISSUE_RESIDENT_MACROPHAGE																														
CELLMARKERCUR_N_TISSUE_RESIDENT_MEMORY_LI…																														
CELLMARKERCUR_N_TOLDC_INDUCED_REGULATORY_…																														
CELLMARKERCUR_N_TONSIL_CELL																														
CELLMARKERCUR_N_TRABECULAR_MESHWORK_CELL																														
CELLMARKERCUR_N_TRANSITIONAL_B_CELL																														
CELLMARKERCUR_N_TRANSITIONAL_DENDRITIC_CE…																														
CELLMARKERCUR_N_TRUNCATED_RADIAL_GLIAL_CE…																														
CELLMARKERCUR_N_TUBULAR_CELL																														
CELLMARKERCUR_N_TUMOR_ASSOCIATED_MICROGLI…																														
CELLMARKERCUR_N_TUMOR_ENDOTHELIAL_CELL																														
CELLMARKERCUR_N_TUMOR_EPITHELIAL_CELL																														
CELLMARKERCUR_N_TUMOR_INFILTRATING_REGULA…																														
CELLMARKERCUR_N_TUMOR_INFILTRATING_T_CELL																														
CELLMARKERCUR_N_TUMOR_INITIATING_CELL																														
CELLMARKERCUR_N_TUMOR_REGULATORY_T_CELL																														

CELLMARKERCAN_ACINAR_CELLS																														
CELLMARKERCUR_N_UMBRELLA_CELL																														
CELLMARKERCUR_N_UNRESTRICTED_SOMATIC_STEM…																														
CELLMARKERCUR_N_UNSWITCHED_PLASMABLAST																														
CELLMARKERCUR_N_URETERIC_BUD_CELL																														
CELLMARKERCUR_N_UROTHELIAL_CELL																														
CELLMARKERCUR_N_UROTHELIUM_CELL																														
CELLMARKERCUR_N_VASCULATURE_CELL																														
CELLMARKERCUR_N_VEIN_CELL																														
CELLMARKERCUR_N_VENOUS_CELL																														
CELLMARKERCUR_N_VENTRAL_LATERAL_GANGLIONI…																														
CELLMARKERCUR_N_VENTRAL_MEDIAL_GANGLIONIC…																														
CELLMARKERCUR_N_VENTRICULAR_CELL																														
CELLMARKERCUR_N_YOUNG_NEURON																														
CELLMARKERCUR_N_ZONA_FASCICULATA_CELL																														
CELLMARKERCUR_N_ZONA_GLOMERULOSA_CELL																														
CELLMARKERCUR_N_ZONA_RETICULARIS_CELL																														
CELLMARKERCUR_N_ZYGOTE																														
CELLMARKERCURNACTIVATEDEFFECTORMEMORYCD4T…																														
CELLMARKERCURNACTIVATEDTISSUERESIDENTMEMO…																														
CELLMARKERCURNACTIVATEDTISSUERESIDENTMEMO…																														
CELLMARKERCURNALPHACELLACELL																														
CELLMARKERCURNALVEOLARTYPEIIATIICELL																														
CELLMARKERCURNATYPICALBCELLABC																														
CELLMARKERCURNAXLCD11CPLASMACYTOIDDENDRIT…																														
CELLMARKERCURNAXLCD123PLASMACYTOIDDENDRIT…																														
CELLMARKERCURNBASALFOREBRAINCHOLINERGICNE…																														
CELLMARKERCURNBRUSHCELLTUFTCELL																														
CELLMARKERCURNCANCERASSOCIATEDFIBROBLASTC…																														
CELLMARKERCURNCAPSULARFIBROBLASTFB2																														
CELLMARKERCURNCCR7DENDRITICCELL																														
CELLMARKERCURNCD106MESENCHYMALSTEMCELL																														
CELLMARKERCURNCD14MACROPHAGE																														
CELLMARKERCURNCD16DENDRITICCELL																														
CELLMARKERCURNCD25LAG3TCELL																														
CELLMARKERCURNCD34GRANULOCYTICPROGENITORC…																														
CELLMARKERCURNCD34HEMATOPOIETICSTEMCELL																														
CELLMARKERCURNCD34PREBCELL																														
CELLMARKERCURNCD34PROGENITORCELL																														
CELLMARKERCURNCD34THYMOCYTE																														
CELLMARKERCURNCD40LGTHELPERCELL																														
CELLMARKERCURNCD4CD25REGULATORYTCELL																														
CELLMARKERCURNCD4CENTRALMEMORYLIKETTCMLIK…																														
CELLMARKERCURNCD4CYTOTOXICT1CELL																														
CELLMARKERCURNCD4CYTOTOXICT2CELL																														
CELLMARKERCURNCD4THELPERCELL																														
CELLMARKERCURNCHRONICALLYACTIVATEDCD4TCELL																														
CELLMARKERCURNCHRONICALLYACTIVATEDCD8TCELL																														
CELLMARKERCURNCLASSSWITCHEDMEMORYBIGGU03B…																														
CELLMARKERCURNCLEC10ADENDRITICCELL																														
CELLMARKERCURNCLEC9ADENDRITICCELL																														
CELLMARKERCURNCONVENTIONALDENDRITICCELL2B…																														
CELLMARKERCURNCONVENTIONALDENDRITICCELL2B…																														
CELLMARKERCURNCORTICALSOMATOSTATINSSTINTE…																														
CELLMARKERCURNCYTOTOXICCD4T2CELL																														
CELLMARKERCURNCYTOTOXICCD4TCELL																														
CELLMARKERCURND1MSNMEDIUMSPINYNEURON																														
CELLMARKERCURND2MSNMEDIUMSPINYNEURON																														
CELLMARKERCURNDISTALTUBULELOOPOFHENLECELL																														
CELLMARKERCURNEARLYCD34MEGAKARYOCYTEERYTH…																														
CELLMARKERCURNEARLYTCELLPROGENITORETP																														
CELLMARKERCURNEFFECTORMEMORYCD4TCELL																														
CELLMARKERCURNEFFECTORREGULATORYTETREGCELL																														
CELLMARKERCURNEFFECTORTTEFFCELL																														
CELLMARKERCURNEXHAUSTEDCD4TCELL																														
CELLMARKERCURNEXHAUSTEDCD8TCELL																														
CELLMARKERCURNEXHAUSTEDCD8TPROGENITORCELL																														
CELLMARKERCURNEXHAUSTEDTTEXCELL																														
CELLMARKERCURNEXTRATELENCEPHALICETCELL																														
CELLMARKERCURNFINALLYHIGHLYEFFECTORTEMRAM…																														
CELLMARKERCURNFOLLICULARREGULATORYTTFRCELL																														
CELLMARKERCURNFOXP3NATURALREGULATORYTTREG…																														
CELLMARKERCURNGAMMADELTAU03B3DTCELL																														
CELLMARKERCURNGERMINALCENTREGCLIKECELL																														
CELLMARKERCURNGLOMERULARENDOTHELIALCELLECG																														
CELLMARKERCURNGRANULOCYTICMYELOIDDERIVEDS…																														
CELLMARKERCURNGRBREGULATORYBCELL																														
CELLMARKERCURNGZMKTCELL																														
CELLMARKERCURNHIGHLYDIFFERENTIATEDEFFECTO…																														
CELLMARKERCURNINDUCEDREGULATORYTTREGCELL																														
CELLMARKERCURNINFLAMMATORYCANCERASSOCIATE…																														
CELLMARKERCURNINNATELYMPHOIDCELLILCS																														
CELLMARKERCURNINTRATHYMICFIBROBLASTFB1																														
CELLMARKERCURNITOCELLHEPATICSTELLATECELL																														
CELLMARKERCURNLYMPHANGIOLEIOMYOMATOSISCEL…																														
CELLMARKERCURNMARGINALZONEMZLIKEBCELL																														
CELLMARKERCURNMEMORYSTEMCELLTSCM																														
CELLMARKERCURNMICROGLIADERIVEDTUMORASSOCI…																														
CELLMARKERCURNMKERYTHCELL																														
CELLMARKERCURNMYOFIBROBLASTICCANCERASSOCI…																														
CELLMARKERCURNNAIVEREGULATORYTTREGCELL																														
CELLMARKERCURNNATURALREGULATORYTTREGCELL																														
CELLMARKERCURNPERIPHERALBLOODMONONUCLEARC…																														
CELLMARKERCURNPITUITARYSTEMCELLPSC																														
CELLMARKERCURNPLASMACYTOIDDENDRITICCELLPDC																														
CELLMARKERCURNPOLYMORPHONUCLEARMYELOIDDER…																														
CELLMARKERCURNPOLYMORPHONUCLEARNEUTROPHIL…																														
CELLMARKERCURNPOSTERIORPRESOMITICMESODERM…																														
CELLMARKERCURNPROLIFERATIVECD4TCELL																														
CELLMARKERCURNPROLIFERATIVECD8TCELL																														
CELLMARKERCURNREDBLOODCELLERYTHROCYTE																														
CELLMARKERCURNREGULATORYBBREGCELL																														
CELLMARKERCURNREGULATORYCD4TCELL																														
CELLMARKERCURNREGULATORYCD8TCELL																														
CELLMARKERCURNREGULATORYTTREGCELL																														

Table S5: Cell state marker gene list

CELLMARKERCAN_ACINAR_CELLS																														
CELLMARKERCURNREGULATORYTTREGCELL																														
CELLMARKERCURNRENALVESICLECOMMASHAPEDBODY…																														
CELLMARKERCURNRESIDENTMEMORYCD8TCELL																														
CELLMARKERCURNREVERTANTMEMORYCD8TTEMRACELL																														
CELLMARKERCURNSCARASSOCIATEDMACROPHAGESAM																														
CELLMARKERCURNSENESCENTCD8TCELL																														
CELLMARKERCURNSTEMPROGENITORCELL																														
CELLMARKERCURNSWITCHEDMEMORYBCELLSWMB																														
CELLMARKERCURNT1TRANSITIONALBCELL																														
CELLMARKERCURNT2TRANSITIONALBCELL																														
CELLMARKERCURNTCELLNATURALKILLERCELL																														
CELLMARKERCURNTERMINALEFFECTORCD8TCELL																														
CELLMARKERCURNTERMINALLYDIFFERENTIATEDCD8…																														
CELLMARKERCURNTFOLLICULARHELPERTFHCELL																														
CELLMARKERCURNTHELPER1TH1CELL																														
CELLMARKERCURNTHELPER1TH1CELL																														
CELLMARKERCURNTHELPER2TH2CELL																														
CELLMARKERCURNTHELPER2TH2CELL																														
CELLMARKERCURNTHELPER9TH9CELL																														
CELLMARKERCURNTHELPERTHCELL																														
CELLMARKERCURNTHYMICEPITHELIALCELLTEC																														
CELLMARKERCURNTISSUERESIDENTMEMORYCD4TCELL																														
CELLMARKERCURNTISSUERESIDENTMEMORYCD8TCELL																														
CELLMARKERCURNTISSUERESIDENTMEMORYTTRMCELL																														
CELLMARKERCURNTPERIPHERALHELPERTPHCELL																														
CELLMARKERCURNTRANSIENTAMPLIFYINGTACELL																														
CELLMARKERCURNTRANSITIONALEXHAUSTEDCD8TCE…																														
CELLMARKERCURNTUMORINFILTRATINGLYMPHOCYTE…																														
CELLMARKERCURNUNSWITCHEDMEMORYBCELLUNSWMB																														
CELLMARKERCURNVASARECTAENDOTHELIALCELLECVR																														
CELLMARKERCURNVONECONOMONEURONVEN																														
CELLMARKERCURNYOLKSACDERIVEDMYELOIDBIASED…																														
CELLMARKEREXP_ACINAR_CELLS																														
CELLMARKEREXP_ADRENERGIC_NEURONS																														
CELLMARKEREXP_AIRWAY_EPITHELIAL_CELLS																														
CELLMARKEREXP_AIRWAY_SMOOTH_MUSCLE_CELLS																														
CELLMARKEREXP_ALPHA_CELLS																														
CELLMARKEREXP_ANTERIOR_PITUITARY_GLAND_CE…																														
CELLMARKEREXP_ASTROCYTES																														
CELLMARKEREXP_B_CELLS																														
CELLMARKEREXP_B_CELLS_MEMORY																														
CELLMARKEREXP_B_CELLS_NAIVE																														
CELLMARKEREXP_BASAL_CELLS																														
CELLMARKEREXP_BASOPHILS																														
CELLMARKEREXP_BERGMANN_GLIA																														
CELLMARKEREXP_BETA_CELLS																														
CELLMARKEREXP_CAJAL_RETZIUS_CELLS																														
CELLMARKEREXP_CHOLANGIOCYTES																														
CELLMARKEREXP_CHOLINERGIC_NEURONS																														
CELLMARKEREXP_CHONDROCYTES																														
CELLMARKEREXP_DELTA_CELLS																														
CELLMARKEREXP_DISTAL_TUBULE_CELLS																														
CELLMARKEREXP_DUCTAL_CELLS																														
CELLMARKEREXP_EMBRYONIC_STEM_CELLS																														
CELLMARKEREXP_ENTERIC_NEURONS																														
CELLMARKEREXP_ENTEROENDOCRINE_CELLS																														
CELLMARKEREXP_EPENDYMAL_CELLS																														
CELLMARKEREXP_EPSILON_CELLS																														
CELLMARKEREXP_ERYTHROID_LIKE_AND_ERYTHROI…																														
CELLMARKEREXP_GAMMA_DELTA_T_CELLS																														
CELLMARKEREXP_GOBLET_CELLS																														
CELLMARKEREXP_HEMANGIOBLASTS																														
CELLMARKEREXP_HEMATOPOIETIC_STEM_CELLS																														
CELLMARKEREXP_HEPATIC_STELLATE_CELLS																														
CELLMARKEREXP_HIS_BUNDLE_CELLS																														
CELLMARKEREXP_IMMATURE_NEURONS																														
CELLMARKEREXP_JUXTAGLOMERULAR_CELLS																														
CELLMARKEREXP_KERATINOCYTES																														
CELLMARKEREXP_KIDNEY_PROGENITOR_CELLS																														
CELLMARKEREXP_KUPFFER_CELLS																														
CELLMARKEREXP_LEYDIG_CELLS																														
CELLMARKEREXP_LOOP_OF_HENLE_CELLS																														
CELLMARKEREXP_LUMINAL_EPITHELIAL_CELLS																														
CELLMARKEREXP_MAMMARY_EPITHELIAL_CELLS																														
CELLMARKEREXP_MEGAKARYOCYTES																														
CELLMARKEREXP_MELANOCYTES																														
CELLMARKEREXP_MESANGIAL_CELLS																														
CELLMARKEREXP_MESOTHELIAL_CELLS																														
CELLMARKEREXP_MICROFOLD_CELLS																														
CELLMARKEREXP_MICROGLIA																														
CELLMARKEREXP_MONOCYTES																														
CELLMARKEREXP_MULLER_CELLS																														
CELLMARKEREXP_MYOBLASTS																														
CELLMARKEREXP_MYOCYTES																														
CELLMARKEREXP_MYOFIBROBLASTS																														
CELLMARKEREXP_NEUROBLASTS																														
CELLMARKEREXP_NEUTROPHILS																														
CELLMARKEREXP_NK_CELLS																														
CELLMARKEREXP_NORADRENERGIC_NEURONS																														
CELLMARKEREXP_OLIGODENDROCYTES																														
CELLMARKEREXP_OSTEOBLASTS																														
CELLMARKEREXP_OSTEOCLAST_PRECURSOR_CELLS																														
CELLMARKEREXP_OSTEOCLASTS																														
CELLMARKEREXP_OSTEOCYTES																														
CELLMARKEREXP_PANETH_CELLS																														
CELLMARKEREXP_PARATHYROID_CHIEF_CELLS																														
CELLMARKEREXP_PERICYTES																														
CELLMARKEREXP_PHOTORECEPTOR_CELLS																														
CELLMARKEREXP_PLASMA_CELLS																														
CELLMARKEREXP_PLASMACYTOID_DENDRITIC_CELLS																														
CELLMARKEREXP_PODOCYTES																														
CELLMARKEREXP_PRINCIPAL_CELLS																														
CELLMARKEREXP_PROXIMAL_TUBULE_CELLS																														
CELLMARKEREXP_PULMONARY_ALVEOLAR_TYPE_I_C…																														

CELLMARKERCAN_ACINAR_CELLS																														
CELLMARKEREXP_PULMONARY_ALVEOLAR_TYPE_II_…																														
CELLMARKEREXP_PURKINJE_FIBER_CELLS																														
CELLMARKEREXP_PURKINJE_NEURONS																														
CELLMARKEREXP_PYRAMIDAL_CELLS																														
CELLMARKEREXP_RETICULOCYTES																														
CELLMARKEREXP_RETINAL_GANGLION_CELLS																														
CELLMARKEREXP_SALIVARY_MUCOUS_CELLS																														
CELLMARKEREXP_SATELLITE_CELLS																														
CELLMARKEREXP_SCHWANN_CELLS																														
CELLMARKEREXP_SEROTONERGIC_NEURONS																														
CELLMARKEREXP_SERTOLI_CELLS																														
CELLMARKEREXP_SMOOTH_MUSCLE_CELLS																														
CELLMARKEREXP_SPERMATOZOA																														
CELLMARKEREXP_STROMAL_CELLS																														
CELLMARKEREXP_T_CELLS																														
CELLMARKEREXP_T_CELLS_NAIVE																														
CELLMARKEREXP_T_CYTOTOXIC_CELLS																														
CELLMARKEREXP_T_HELPER_CELLS																														
CELLMARKEREXP_T_MEMORY_CELLS																														
CELLMARKEREXP_TRICHOCYTES																														
CELLMARKEREXP_TRIGEMINAL_NEURONS																														
CELLMARKEREXP_TROPHOBLAST_CELLS																														
CELLMARKEREXP_TROPHOBLAST_PROGENITOR_CELLS																														
CELLMARKEREXP_TUFT_CELLS																														
CELLMARKEREXP_VASCULAR_SMOOTH_MUSCLE_CELLS																														
CELLMARKEREXPENDOTHELIALCELLSAORTA																														
CELLMARKEREXPNEURALSTEMPRECURSORCELLS																														
CELLMARKERMSIGDB_AIZARANI_LIVER_C12_NK_NK…																														
CELLMARKERMSIGDB_AIZARANI_LIVER_C22_RESID…																														
CELLMARKERMSIGDB_AIZARANI_LIVER_C28_NK_NK…																														
CELLMARKERMSIGDB_AIZARANI_LIVER_C29_MVECS…	ASAP1	RBMS3	CGNL1	TMCC3	S100A…	TMSB10	NOS3	ACTN1	LUZP1	TFPI2	AKAP12	CNTNA…	DPYSL2	ITM2A	KCTD12	NPDC1	SPAG9	KLF3	PTTG1…	MYO1C	SPRY4	PREX2	CD55	NOTCH4	HSP90…	ATP1B3	NOP56	PDLIM5	YWHAE	PTPN12
CELLMARKERMSIGDB_AIZARANI_LIVER_C30_HEPAT…	MEGF9	ADCY1	LRRC27	RCL1	LRP6	ABCC3	SIN3B	PART1	KCNA7	MAST4	MLXIP	RNF41	PPP1R…	BHMT2	LINC0…	POLR2…	WNT2B	MYO1B	MUC3A	PTP4A1	UCKL1…	ACSM5	UBR4	DDR1-…	SLC31…	CCDC30	ZNF556	SLC28…	DESI1	COL18…
CELLMARKERMSIGDB_AIZARANI_LIVER_C8_RESIDE…																														
CELLMARKERMSIGDB_AIZARANI_LIVER_C9_LSECS_1	AFF1	PRKD3	FCHSD2	EPB41…	SDCBP	GOLGB1	TANC1	CHD7	ADGRL2	KDM6B	SNX5	ITGA9	ANKRD…	TM4SF1	CLEC1…	CRIM1	MYADM	PHF10	EIF4G3	PLK2	TSPAN…									
CELLMARKERMSIGDB_BUSSLINGER_DUODENAL_BCHE…																														
CELLMARKERMSIGDB_BUSSLINGER_DUODENAL_DIFF…	EIF5B	EEF2	TMSB10	UQCRQ	FAU	HMGCS2	TMA7	COX4I1	PKM	MAOA	PFN1	RPL19	CALM2	CLIC1	NDUFA4	SUCLG1	SLC25…	EGR1	ALDH1…	TSPAN8	HSPA8	YWHAB								
CELLMARKERMSIGDB_BUSSLINGER_DUODENAL_EC_C…																														
CELLMARKERMSIGDB_BUSSLINGER_DUODENAL_IMMU…	RAP1A	PNRC1	TMIGD2	GZMB	TSC22…	PIK3CD	RPS25	MYO1G	ABRACL	ICAM3	FAM78A	CYTH4	GRAMD…	NMUR1	HERC1	RAB8B	PTPN4	NBEAL2	JAK3	PTPN6	PLEKH…	GLIPR2	SFMBT2	UNC13D	AAK1	PARP8	PRKCB	PLXDC1	WAS	IFI16
CELLMARKERMSIGDB_BUSSLINGER_DUODENAL_STEM…	CLDN4	RPS11	HMGB1	ACTG1	NUCKS1	EIF4G2	ONECU…	TPD52	HNRNP…	EIF5B	RPL27	CHCHD2	RPL38	PABPC1	UBXN4	RPS9	GSTP1	COX7C	HNRNP…	YWHAZ	IER2	FCGRT	SPINT2	ATP5M…	KRT8	TPT1	HSPA8	RPL23		
CELLMARKERMSIGDB_BUSSLINGER_DUODENAL_TUFT…																														
CELLMARKERMSIGDB_BUSSLINGER_ESOPHAGEAL_EA…																														
CELLMARKERMSIGDB_BUSSLINGER_ESOPHAGEAL_PR…																														
CELLMARKERMSIGDB_BUSSLINGER_GASTRIC_CHIEF…																														
CELLMARKERMSIGDB_BUSSLINGER_GASTRIC_G_CEL…																														
CELLMARKERMSIGDB_BUSSLINGER_GASTRIC_ISTHM…	RPN1	TIMP1	NOP56	FERMT1	BHLHE…	PPARG	PSD3	TCERG1	NCL	GAPDH	SEMA3B	FOXA2	GADD4…	MUC1	CLMN	RPN2	PLXNA2	CORO2A	SMIM14	RHOA	DNAJB…	SH3RF1	SLC44…	USH1C	SLC39…	SLK	PRKCD	NUCKS1	ENSA	FHL2
CELLMARKERMSIGDB_BUSSLINGER_GASTRIC_NECK_…																														
CELLMARKERMSIGDB_BUSSLINGER_GASTRIC_OXYNT…																														
CELLMARKERMSIGDB_BUSSLINGER_GASTRIC_PREZY…																														
CELLMARKERMSIGDB_BUSSLINGER_GASTRIC_REG3A…																														
CELLMARKERMSIGDB_CUI_DEVELOPING_HEART_5TH…																														
CELLMARKERMSIGDB_CUI_DEVELOPING_HEART_5TH…																														
CELLMARKERMSIGDB_CUI_DEVELOPING_HEART_C1_…																														
CELLMARKERMSIGDB_CUI_DEVELOPING_HEART_C2_…																														
CELLMARKERMSIGDB_CUI_DEVELOPING_HEART_COM…																														
CELLMARKERMSIGDB_CUI_DEVELOPING_HEART_COR…																														
CELLMARKERMSIGDB_CUI_DEVELOPING_HEART_END…																														
CELLMARKERMSIGDB_CUI_DEVELOPING_HEART_LEF…																														
CELLMARKERMSIGDB_CUI_DEVELOPING_HEART_RIG…																														
CELLMARKERMSIGDB_CUI_DEVELOPING_HEART_SMO…																														
CELLMARKERMSIGDB_DESCARTES_FETAL_ADRENAL_…																														
CELLMARKERMSIGDB_DESCARTES_FETAL_CEREBELL…																														
CELLMARKERMSIGDB_DESCARTES_FETAL_CEREBELL…																														
CELLMARKERMSIGDB_DESCARTES_FETAL_CEREBELL…	CDKN1A	ADPGK	TANGO2	MAFB	NFATC1	CXCL16	TMEM1…	KBTBD…	UCP2	LAIR1	RAB39A	CTSL	RGS1	FCGR1…	SCIMP	SLC2A5	MILR1	CCDC8…	PIK3CG	EVI2B	ITGB3	CD180	ERMAP	IL10RA	MYOZ2	NLRP3	MAP3K8	ALOX5…	PTGS1	TRAF3…
CELLMARKERMSIGDB_DESCARTES_FETAL_CEREBELL…																														
CELLMARKERMSIGDB_DESCARTES_FETAL_CEREBELL…																														
CELLMARKERMSIGDB_DESCARTES_FETAL_CEREBELL…																														
CELLMARKERMSIGDB_DESCARTES_FETAL_CEREBELL…	HIGD1B	C2CD4B	HEYL	BHLHE…	SYDE1	POLR2L	RFLNB	ADCY4	MYLK2	EFNA1	HTRA3	PGF	SLC9A1	TBXA2R	PRKCH…	QPCT	TPT1P…	NODAL	FZD4	KCNE4	S100A…	SHE	EFEMP2	MIR36…	STARD8	FOXF1	NRARP	TNFAI…	GATA2	VAMP3
CELLMARKERMSIGDB_DESCARTES_FETAL_CEREBRUM…																														
CELLMARKERMSIGDB_DESCARTES_FETAL_CEREBRUM…																														
CELLMARKERMSIGDB_DESCARTES_FETAL_CEREBRUM…	POC1B…	USF1	MPND	ZNF46…	MFSD4B	H2BC21	PCYT2	FLYWC…	RMND1	CFAP2…	RHD	GRM3-…	LINC0…	CEP44	LAT	LIPC	BBC3	DUOX1	ZNF287	RNU6-…	MYO18B	CCDC1…	WDR24	DCLRE…	SH3BP…	ZSCAN…	MZT1	FFAR2	HSD17…	LINC0…
CELLMARKERMSIGDB_DESCARTES_FETAL_CEREBRUM…	GDPD4	BATF	TMEM5…	CDKN2…	TMIGD2	TFCP2…	NLRC4	GPR132	CHIT1	LINC0…	ARHGA…	SIGLE…	ARL5C	MS4A14	MS4A4A	CD5L	ENSG0…	SERPI…	CAPG	NCF4	CALCB	ALDH1…	DENND…	OSCAR	LINC0…	C19or…	LY86-…	TNFSF8	P2RY8	LINC0…
CELLMARKERMSIGDB_DESCARTES_FETAL_CEREBRUM…																														
CELLMARKERMSIGDB_DESCARTES_FETAL_CEREBRUM…																														
CELLMARKERMSIGDB_DESCARTES_FETAL_CEREBRUM…	PCDH1	OAS2	MOB2	SOCS3	LINC0…	A4GALT	DEPP1	APOL4	LINC0…	LUM	LINC0…	MT2A	SLC22…	GPRC5C	SOX17	FOXL2	SLC52…	SIGIRR	FAM16…	CARD10	D21S2…	CETP	SH3TC…	KANK4	SLC16…	ATP5M…	USHBP1	CCDC8…	ST6GA…	IFIT3
CELLMARKERMSIGDB_DESCARTES_FETAL_EYE_ASTR…																														
CELLMARKERMSIGDB_DESCARTES_FETAL_EYE_GANG…																														
CELLMARKERMSIGDB_DESCARTES_FETAL_EYE_LENS…																														
CELLMARKERMSIGDB_DESCARTES_FETAL_EYE_PHOT…																														
CELLMARKERMSIGDB_DESCARTES_FETAL_EYE_RETI…																														
CELLMARKERMSIGDB_DESCARTES_FETAL_EYE_SMOO…																														
CELLMARKERMSIGDB_DESCARTES_FETAL_EYE_STRO…																														
CELLMARKERMSIGDB_DESCARTES_FETAL_EYE_VASC…																														
CELLMARKERMSIGDB_DESCARTES_FETAL_HEART_EL…																														
CELLMARKERMSIGDB_DESCARTES_FETAL_HEART_EN…																														
CELLMARKERMSIGDB_DESCARTES_FETAL_HEART_LY…																														
CELLMARKERMSIGDB_DESCARTES_FETAL_HEART_SC…																														
CELLMARKERMSIGDB_DESCARTES_FETAL_HEART_SM…																														
CELLMARKERMSIGDB_DESCARTES_FETAL_HEART_ST…																														
CELLMARKERMSIGDB_DESCARTES_FETAL_HEART_VI…																														
CELLMARKERMSIGDB_DESCARTES_FETAL_INTESTIN…																														
CELLMARKERMSIGDB_DESCARTES_FETAL_INTESTIN…																														
CELLMARKERMSIGDB_DESCARTES_FETAL_INTESTIN…																														
CELLMARKERMSIGDB_DESCARTES_FETAL_INTESTIN…																														
CELLMARKERMSIGDB_DESCARTES_FETAL_INTESTIN…																														
CELLMARKERMSIGDB_DESCARTES_FETAL_INTESTIN…																														
CELLMARKERMSIGDB_DESCARTES_FETAL_KIDNEY_M…																														
CELLMARKERMSIGDB_DESCARTES_FETAL_KIDNEY_U…	ENSG0…	SPRR1A	UGT1A1	ERVE-1	HSPD1…	BOLL	CXCL17	MIR20…	NECTI…	KRT23	AARD	ARL14	ODAM	FAM13…	SSTR5	ENSG0…	MOGAT3	GJB4												
CELLMARKERMSIGDB_DESCARTES_FETAL_LIVER_ER…																														
CELLMARKERMSIGDB_DESCARTES_FETAL_LIVER_HE…																														
CELLMARKERMSIGDB_DESCARTES_FETAL_LIVER_HE…	SERPI…	UROC1	LINC0…	SERPI…	PERP	NPC1L1	TAT	SLC26…	PROZ	SLC25…	FGFR4	SERPI…	A1BG	GLYAT	CYP2J2	MLIP	TMEM82	EFHD1	LPA	TTPA	SAA4	SLC6A…	VIL1	FFAR4	GREB1	ACSL3…	GGCX	CACNA…	C6	ASPDH
CELLMARKERMSIGDB_DESCARTES_FETAL_LIVER_ME…	MGAM	PRX	CSPG5	TTC23L	COL4A3	SLC8A2	RRAD	HS3ST5	KCNJ15	GOLGA…	LINC0…	TMSB1…	MAD2L…	DNAI7	TEKT5	VIT	PIANP	LINC0…	ZNF649	TAF1A…	ASAH1…	LINC0…	ARHGE…	CBX8	PHF21B	PNMA2	EIF2A…	ODAD3	ACOXL	
CELLMARKERMSIGDB_DESCARTES_FETAL_LUNG_BRO…																														
CELLMARKERMSIGDB_DESCARTES_FETAL_LUNG_CIL…	AKAP14	CCDC96	IQCD	LCA5L	C10or…	MAK	SLC9C2	C17or…	LRRC43	RWDD3…	DYNLT4	DNAAF8	CDHR1…	PITPN…	ENSG0…	ARMH1	RSPH14	RABL2B	PLPP2	CATIP	MYCBP…	TEX26	KIF9	LRRC34	KCNRG	CFAP2…	FAM16…	GLB1L2	ZNF19	COL28…
CELLMARKERMSIGDB_DESCARTES_FETAL_LUNG_LYM…																														

CELLMARKERCAN_ACINAR_CELLS																														
CELLMARKERMSIGDB_DESCARTES_FETAL_LUNG_NEU…																														
CELLMARKERMSIGDB_DESCARTES_FETAL_LUNG_STR…																														
CELLMARKERMSIGDB_DESCARTES_FETAL_LUNG_VAS…																														
CELLMARKERMSIGDB_DESCARTES_FETAL_MUSCLE_L…																														
CELLMARKERMSIGDB_DESCARTES_FETAL_MUSCLE_S…																														
CELLMARKERMSIGDB_DESCARTES_FETAL_MUSCLE_S…																														
CELLMARKERMSIGDB_DESCARTES_FETAL_MUSCLE_V…																														
CELLMARKERMSIGDB_DESCARTES_FETAL_PANCREAS…																														
CELLMARKERMSIGDB_DESCARTES_FETAL_PANCREAS…																														
CELLMARKERMSIGDB_DESCARTES_FETAL_PANCREAS…																														
CELLMARKERMSIGDB_DESCARTES_FETAL_PANCREAS…																														
CELLMARKERMSIGDB_DESCARTES_FETAL_PANCREAS…																														
CELLMARKERMSIGDB_DESCARTES_FETAL_PANCREAS…																														
CELLMARKERMSIGDB_DESCARTES_FETAL_PANCREAS…																														
CELLMARKERMSIGDB_DESCARTES_FETAL_PLACENTA…																														
CELLMARKERMSIGDB_DESCARTES_FETAL_PLACENTA…																														
CELLMARKERMSIGDB_DESCARTES_FETAL_PLACENTA…																														
CELLMARKERMSIGDB_DESCARTES_FETAL_PLACENTA…																														
CELLMARKERMSIGDB_DESCARTES_FETAL_PLACENTA…																														
CELLMARKERMSIGDB_DESCARTES_FETAL_SPLEEN_E…																														
CELLMARKERMSIGDB_DESCARTES_FETAL_SPLEEN_S…																														
CELLMARKERMSIGDB_DESCARTES_FETAL_SPLEEN_V…																														
CELLMARKERMSIGDB_DESCARTES_FETAL_STOMACH_…	SCGB2…	TTC16	CST6	PRR29	HAGHL	IQCD	RSPH1…	FAM16…	ZMYND…	C2orf…	ZNF20	LRRC27	SPAG8	GON7	RSPH1…	FAM16…	ENKD1	SAMD15	WDR86…	ANKRD…	WDR90	ZC2HC…	LINC0…	SNRPF…	TTC9-…	CYP4B1	LINC0…	LRRC71	CD164…	LINC0…
CELLMARKERMSIGDB_DESCARTES_FETAL_STOMACH_…																														
CELLMARKERMSIGDB_DESCARTES_FETAL_STOMACH_…																														
CELLMARKERMSIGDB_DESCARTES_FETAL_STOMACH_…																														
CELLMARKERMSIGDB_DESCARTES_FETAL_STOMACH_…																														
CELLMARKERMSIGDB_DESCARTES_FETAL_STOMACH_…																														
CELLMARKERMSIGDB_DESCARTES_FETAL_STOMACH_…																														
CELLMARKERMSIGDB_DESCARTES_FETAL_STOMACH_…																														
CELLMARKERMSIGDB_DESCARTES_FETAL_STOMACH_…																														
CELLMARKERMSIGDB_DESCARTES_FETAL_STOMACH_…																														
CELLMARKERMSIGDB_DESCARTES_FETAL_STOMACH_…																														
CELLMARKERMSIGDB_DESCARTES_FETAL_STOMACH_…																														
CELLMARKERMSIGDB_DESCARTES_FETAL_THYMUS_T…																														
CELLMARKERMSIGDB_DESCARTES_MAIN_FETAL_ACI…																														
CELLMARKERMSIGDB_DESCARTES_MAIN_FETAL_ADR…																														
CELLMARKERMSIGDB_DESCARTES_MAIN_FETAL_AST…																														
CELLMARKERMSIGDB_DESCARTES_MAIN_FETAL_BIP…																														
CELLMARKERMSIGDB_DESCARTES_MAIN_FETAL_BRO…																														
CELLMARKERMSIGDB_DESCARTES_MAIN_FETAL_CAR…																														
CELLMARKERMSIGDB_DESCARTES_MAIN_FETAL_CIL…	C7orf…	ABCA13	METTL…	TMEM2…	CCDC1…	SRI	CFAP69	AKAP9	IFT22	LRWD1	FBXL13	EFCAB…	CDHR3	LINC0…	IQUB	LRGUK	TTC26	WDR86…	RNF32	DYNC2…	PCM1	NAT1	INTS10	LINC0…	CLXN	RB1CC1	TMEM68	PPP1R…	CSPP1	LINC0…
CELLMARKERMSIGDB_DESCARTES_MAIN_FETAL_DUC…																														
CELLMARKERMSIGDB_DESCARTES_MAIN_FETAL_END…																														
CELLMARKERMSIGDB_DESCARTES_MAIN_FETAL_ENS…																														
CELLMARKERMSIGDB_DESCARTES_MAIN_FETAL_EPI…																														
CELLMARKERMSIGDB_DESCARTES_MAIN_FETAL_GAN…																														
CELLMARKERMSIGDB_DESCARTES_MAIN_FETAL_GOB…																														
CELLMARKERMSIGDB_DESCARTES_MAIN_FETAL_HEM…																														
CELLMARKERMSIGDB_DESCARTES_MAIN_FETAL_HOR…																														
CELLMARKERMSIGDB_DESCARTES_MAIN_FETAL_INH…																														
CELLMARKERMSIGDB_DESCARTES_MAIN_FETAL_INH…																														
CELLMARKERMSIGDB_DESCARTES_MAIN_FETAL_INT…																														
CELLMARKERMSIGDB_DESCARTES_MAIN_FETAL_ISL…																														
CELLMARKERMSIGDB_DESCARTES_MAIN_FETAL_LYM…																														
CELLMARKERMSIGDB_DESCARTES_MAIN_FETAL_MES…																														
CELLMARKERMSIGDB_DESCARTES_MAIN_FETAL_MET…																														
CELLMARKERMSIGDB_DESCARTES_MAIN_FETAL_MYE…																														
CELLMARKERMSIGDB_DESCARTES_MAIN_FETAL_NEU…																														
CELLMARKERMSIGDB_DESCARTES_MAIN_FETAL_OLI…																														
CELLMARKERMSIGDB_DESCARTES_MAIN_FETAL_PDE…																														
CELLMARKERMSIGDB_DESCARTES_MAIN_FETAL_PDE…	DPH1-…	SLC16…	TMEM2…	POLR2A	PER1	MFSD6L	ALDH3…	LYRM9	LRRC3…	STAC2	MIEN1	SP2	EFCAB3	MAP2K6	ARMC7	RAB40B	TWSG1…	IMPA2	ZNF397	LINC0…	KCNG2	PET100	FBN3	ZNF558	C3P1	TSPAN…	ZNF441	GCDH	BRME1	HAUS8
CELLMARKERMSIGDB_DESCARTES_MAIN_FETAL_RET…																														
CELLMARKERMSIGDB_DESCARTES_MAIN_FETAL_SAT…																														
CELLMARKERMSIGDB_DESCARTES_MAIN_FETAL_SCH…																														
CELLMARKERMSIGDB_DESCARTES_MAIN_FETAL_SLC…	RSAD1	RNU6-…	TMEM1…	TUBB8B	RPL6P…	ST8SI…	SKA1	ELAC1	PHLPP1	ATP9B	REXO1	RANBP3	QTRT1	ZNF627	FAM32A	LINC0…	GPATC…	LIPE-…	TOMM40	RNU6-…	ZNF296	ZNF541	ZNF841	ZNF845	ZNF211	MRPS26	MCM8	BTBD3…	BPIFB1	SPAG4
CELLMARKERMSIGDB_DESCARTES_MAIN_FETAL_SMO…																														
CELLMARKERMSIGDB_DESCARTES_MAIN_FETAL_STE…																														
CELLMARKERMSIGDB_DESCARTES_MAIN_FETAL_SYM…																														
CELLMARKERMSIGDB_DESCARTES_MAIN_FETAL_UNI…																														
CELLMARKERMSIGDB_DESCARTES_MAIN_FETAL_URE…																														
CELLMARKERMSIGDB_DESCARTES_MAIN_FETAL_VAS…																														
CELLMARKERMSIGDB_DESCARTES_MAIN_FETAL_VIS…																														
CELLMARKERMSIGDB_DURANTE_ADULT_OLFACTORY_…																														
CELLMARKERMSIGDB_DURANTE_ADULT_OLFACTORY_…																														
CELLMARKERMSIGDB_DURANTE_ADULT_OLFACTORY_…																														
CELLMARKERMSIGDB_DURANTE_ADULT_OLFACTORY_…																														
CELLMARKERMSIGDB_DURANTE_ADULT_OLFACTORY_…																														
CELLMARKERMSIGDB_DURANTE_ADULT_OLFACTORY_…																														
CELLMARKERMSIGDB_DURANTE_ADULT_OLFACTORY_…																														
CELLMARKERMSIGDB_DURANTE_ADULT_OLFACTORY_…																														
CELLMARKERMSIGDB_DURANTE_ADULT_OLFACTORY_…																														
CELLMARKERMSIGDB_DURANTE_ADULT_OLFACTORY_…																														
CELLMARKERMSIGDB_DURANTE_ADULT_OLFACTORY_…																														
CELLMARKERMSIGDB_DURANTE_ADULT_OLFACTORY_…																														
CELLMARKERMSIGDB_DURANTE_ADULT_OLFACTORY_…																														
CELLMARKERMSIGDB_DURANTE_ADULT_OLFACTORY_…																														
CELLMARKERMSIGDB_DURANTE_ADULT_OLFACTORY_…																														
CELLMARKERMSIGDB_DURANTE_ADULT_OLFACTORY_…																														
CELLMARKERMSIGDB_FAN_EMBRYONIC_CTX_ASTROC…																														
CELLMARKERMSIGDB_FAN_EMBRYONIC_CTX_BIG_GR…	PLXND1	LTBR	APOLD1	NOTCH1	SAMM50	NAMPT	MFAP2	TES	TIMP3	CDH11	SLC52…	RAPGE…	LAPTM…	SNRK	CDC42…	SIPA1	CAST	ACTN4	NR3C1	SORBS3	VIM	JAM2	JAG1	CRIP2	FKBP1A	ESYT2	SELEN…	SYPL1	PSMB8	FLNA
CELLMARKERMSIGDB_FAN_EMBRYONIC_CTX_BIG_GR…	HSPB8	GNAL	TMEM2…	AKAP1	MAP4	ACLY	MAP7D2	NCS1	MXRA7	NDUFA…	KIAA0…	CLEC2L	PGAM1	FANK1	STX1A	MRPL41	SCG5	RAB6B	WIF1	SERTM1	PFKP	GALR2	FSTL4	ARHGA…	HMGCS1	NELL1	SCGB1…	AHNAK	B3GNT4	AGPAT2
CELLMARKERMSIGDB_FAN_EMBRYONIC_CTX_BIG_GR…																														
CELLMARKERMSIGDB_FAN_EMBRYONIC_CTX_BIG_GR…	HLA-E	CYFIP1	CKS2	SALL1	LINC0…	GPN3	RIN2	CCND1	HSPA5	PSAP	IGF1	BST2	TLN1	GAS6	RNF130	FMNL3	GLUL	LIMS1	CAPZB	GNB4	GNG7	LAP3	CD81	SCAMP2	DOCK4	DAGLB	SLC1A3	CHCHD7	GLRX	KCTD12
CELLMARKERMSIGDB_FAN_EMBRYONIC_CTX_BRAIN_…	SLCO1…	SEMA3G	PCDH12	RASIP1	SOX18	TMEM5…	NEIL1	SPARC…	PPA1	C12or…	NOS3	DACH1	ABLIM3	PELO	CHST12	PKP4	ST6GA…	PLEKH…	LDLRA…	IGF1R	PTPRM	ARL2	TMEM4…	DLL4	TUBB6	SLC2A3	IGF2B…	UNC5B	IFI44L	CDC42…
CELLMARKERMSIGDB_FAN_EMBRYONIC_CTX_BRAIN_…	PRKG1	CA4	PPFIB…	TNFAI…	DENND…	ARHGE…	NFATC4	PARD3	ANXA6	EMP2	CYC1	HCFC1…	BMP1	LURAP…	ARHGA…	PID1	GOLIM4	SGPL1	LIMA1	NT5DC2										
CELLMARKERMSIGDB_FAN_EMBRYONIC_CTX_EX_1_E…																														
CELLMARKERMSIGDB_FAN_EMBRYONIC_CTX_IN_2_I…																														
CELLMARKERMSIGDB_FAN_EMBRYONIC_CTX_IN_3_I…																														
CELLMARKERMSIGDB_FAN_EMBRYONIC_CTX_IN_5_I…																														
CELLMARKERMSIGDB_FAN_EMBRYONIC_CTX_IN_6_I…																														
CELLMARKERMSIGDB_FAN_EMBRYONIC_CTX_MICROG…																														
CELLMARKERMSIGDB_FAN_EMBRYONIC_CTX_OLIG	HIPK2	RTKN	SGK2	SLCO1…	QKI	DCTN1	RETRE…	IGSF8	RHOG	GNG7	PAQR6	NTM-A…	RAB40B	DOCK9	HMOX2	AGAP1	CCNY	PREX1	DNAJC6	FRYL	MPC1	TFEB	FAM13C	PRKCQ…	MARCH…	ASPA	TYRO3	BBX	DENND…	PACRG…
CELLMARKERMSIGDB_FAN_EMBRYONIC_CTX_OPC																														

Table S5: Cell state marker gene list

CELLMARKERCAN_ACINAR_CELLS																														
CELLMARKERMSIGDB_FAN_OVARY_CL1_GPRC5A_TNF…	GSPT1	GRPEL1	SRRM2	SPTSSA	PPP4R2	STRAP	MTHFD2	NAP1L1	EIF4G2	RPL10	MAPRE1	LUZP1	UTP11	PNRC2	WDR1	RPS5	SQSTM1	GSN	TMEM8…	EWSR1	TXNRD1	COLEC…	SSB	XRCC6	LITAF	CEBPZ	TUBA1A	PALLD	UBE2N	CAMLG
CELLMARKERMSIGDB_FAN_OVARY_CL11_MURAL_GRA…	RAB5IF	CMTM6	ME2	UBE2H	FOXP1	CEBPD	SIGLE…	RYBP	TMEM1…	KCNQ1…	IL6ST	BEX3	CSRP2	SRSF3	BRD4	H1-0	SELEN…	RPSA	IRF4	CCT6A	HNRNPD	RBMS1	MAP4K4	TLE3	DCUN1…	TNFRS…	MT-CO1	UBE2S	MSI2	IFI27
CELLMARKERMSIGDB_FAN_OVARY_CL13_MONOCYTE_…	SPNS1	TAOK3	RGCC	ACOT9	EHD1	SNAP23	LIMS1	SYNGR2	AKAP13	CRIP1	CYTOR	SNX3	GNG5	MIR22…	PPP1CA	CAP1	TMEM1…	HSPB1	BAX	BNIP3L	ABL2	DNTTI…	TXNRD1	NABP1	CD63	ATP5MF	NUMB	MBP	H3-3A	TAPBP
CELLMARKERMSIGDB_FAN_OVARY_CL14_MATURE_SM…	SERF2	PTMA	YWHAQ	DUT	TPM4	HMGN2	ATP6A…	TUBA1A	UBL5	RAB2A	COX6B1	COX5B	OST4	UBB	RAC1	UQCRB	PEBP1	DYNLL1	ALKBH7	EPS8	MT-ND3	LGALS…	TAF7							
CELLMARKERMSIGDB_FAN_OVARY_CL15_SMALL_ANT…	GOLPH3	CKAP2	PUM1	CHSY1	PNN	EZH2	FNDC3B	LPL	SUMO1	HSPA4	MFAP2	WEE1	YAP1	CMTM8	PPP1R…	TUFT1	HSP90…	THAP9…	NAV2	RPS25	RANBP1	TMEM1…	RNMT	CALM2	ZC3HA…	TBCA	UBC	CENAT…	CCT6A	CPSF6
CELLMARKERMSIGDB_FAN_OVARY_CL18_B_LYMPHOC…	TMEM2…	GRSF1	TRIB1	RPN2	PAK1	ARHGD…	P4HB	RPL28	RBM3	MOB4	ANKRD…	YTHDF2	TUBB4B	SAR1B	ARHGD…	SNRPG	ATP6V…	CACYBP	SRSF3	NAA50	TUBB6	CYRIB	RAB8A	RNF149	PHF20…	IDS	SEC61…	CCDC12	SMC4	VCP
CELLMARKERMSIGDB_FAN_OVARY_CL2_PUTATIVE_E…																														
CELLMARKERMSIGDB_FAN_OVARY_CL5_HEALTHY_SE…	GADD4…	TLE1	BOD1	TMEM59	SCD5	MDH2	PTTG1	GRHPR	COX6C	CCT4	JMJD6	ACAA1	GYPC	THAP9…	CCT2	C5orf…	RHBDD2	SRSF6	EPS8	LAMP2	MTX2	PSMA2	STRAP	HNRNP…	MEG3	NPM3	NOP58	FERMT2	BORCS7	DDIT4
CELLMARKERMSIGDB_FAN_OVARY_CL6_PUTATIVE_E…	NOP53	MBNL2	AK3	HADHB	CYB5R3	PRDX4	GDI2	CALU	REXO2	SYPL1	WASF2	RHOC	IFI27…	SGK1	MEG3	FBXO21	SAT2													
CELLMARKERMSIGDB_FAN_OVARY_CL8_MATURE_CUM…	ANP32B	SEC61G	H3-3A	SNRPD1	ECHDC3	POLR2I	RPL26…	FDFT1	NAV2	NDUFB2	GREB1	ATOX1	CMTM6	TP53I3	DPY30	MARCK…	COX6A1	TMA7	SMARC…	ERH	STMP1	CASP6	C7orf…	PPDPF	COMT	MRPL11	TRAPP…	USO1	HDDC2	PIN1
CELLMARKERMSIGDB_FAN_OVARY_CL9_PUTATIVE_A…	MAP2K3	BNIP2	LDHA	ARID4B	LRRFI…	CTNNA1	MAT2A	ARPC1B	TMEM1…	TRIR	ARHGD…	GNAS	AHSA1	MRPL14	ANKRD…	RCN1	HNRNPM	SAFB2	STAT3	MRPL32	MAFF	INSIG1	CNIH1	TUBA1C	SLIRP	TNFRS…	SNHG7	EIF3I	EIF3J	RHOC
CELLMARKERMSIGDB_GAO_ESOPHAGUS_25W_C1_CIL…	MS4A8	NBEA	NEK10	NEK5	NELL2	NHLRC4	NOVA1	NPHP1	NPIPA1	NRP2	NWD1	NXF3	ODF3B	OR7E3…	OSGEP	OTUD1	OXTR	P4HTM	PACRG	PECR	PFN2	PHTF1	PIH1D2	PLCB2	PLCG2	PLEKH…	PNO1	POLQ	PPP1R…	PPP1R…
CELLMARKERMSIGDB_GAO_ESOPHAGUS_25W_C2_KRT…																														
CELLMARKERMSIGDB_GAO_ESOPHAGUS_25W_C3_FGF…																														
CELLMARKERMSIGDB_GAO_LARGE_INTESTINE_24W_…																														
CELLMARKERMSIGDB_GAO_LARGE_INTESTINE_24W_…																														
CELLMARKERMSIGDB_GAO_LARGE_INTESTINE_24W_…	SLC9A9	SLCO2…	SLFN11	SNN	SNX10	SNX20	SOD2	SPI1	SPIC	SPPL2A	SRGN	ST3GA…	STAB1	STK26	STX11	TBXAS1	TFEC	THBD	TLR2	TLR4	PACC1	TNF	TNFAI…	TNFAI…	TNFRS…	TNFSF…	TPP1	TRAF1	TSPAN…	TYROBP
CELLMARKERMSIGDB_GAO_LARGE_INTESTINE_24W_…																														
CELLMARKERMSIGDB_GAO_LARGE_INTESTINE_24W_…																														
CELLMARKERMSIGDB_GAO_LARGE_INTESTINE_24W_…																														
CELLMARKERMSIGDB_GAO_LARGE_INTESTINE_ADUL…	TDRP	TFPI	TGM3	THSD7A	TM7SF2	TMEM1…	TMEM1…	TMEM6…	TMOD1	TNNC1	TPH1	TRAM1…	TSPAN5	TTC23L	TTC41P	TTR	TUBA1A	TUBB2B	UCN3	UNC13A	VAT1L	VGF	VNN3P	VWA5B2	WASF3	DNAI3	ZFR2	ZNF460	ZP2	
CELLMARKERMSIGDB_GAO_LARGE_INTESTINE_ADUL…	RBM26…	RBP7	RBPMS	RCAN2	RDH8	RHBDL3	RMI2	RNF215	RPS2P…	SAP25	SCN11A	SDK1	SENP3	SERPI…	SERPI…	SH2D7	SKI	SLC12…	SLC16…	SLC18…	SLC1A2	SLC22…	SLC24…	SLC7A…	SLC9A…	SNTA1	SNTN	SPESP1	SSPN	ST3GA…
CELLMARKERMSIGDB_GAO_LARGE_INTESTINE_ADUL…																														
CELLMARKERMSIGDB_GAO_LARGE_INTESTINE_ADUL…	RRAGD	RUNX2	RUNX3	S100A4	SAMSN1	SASH3	SCPEP1	SEC14…	SELEN…	SELPLG	SEPTI…	SEPTI…	SFMBT2	SIT1	SKAP1	SLA	SLAMF1	SLAMF7	SLC12…	SLC1A4	SLC2A3	SLC47…	SLC7A5	SLC7A7	SLFN11	SNAI3	SP140	SPAG4	SPINK2	SPOCK2
CELLMARKERMSIGDB_GAO_LARGE_INTESTINE_ADUL…	MEIS3	MFAP4	MFGE8	MGP	MMP11	MMP19	MMP2	MMRN2	MRC2	IRAG1	MSC-A…	MSN	MSRB3	MX2	MXRA5	MXRA7	MXRA8	MYCT1	MYL9	NDST1	NDUFA…	NDUFA…	NEGR1	NES	NEXN	NFATC4	NID1	NKX2-3	NNMT	NOTCH3
CELLMARKERMSIGDB_GAO_SMALL_INTESTINE_24W_…																														
CELLMARKERMSIGDB_GAO_SMALL_INTESTINE_24W_…																														
CELLMARKERMSIGDB_GAO_SMALL_INTESTINE_24W_…																														
CELLMARKERMSIGDB_GAO_SMALL_INTESTINE_24W_…																														
CELLMARKERMSIGDB_GAO_SMALL_INTESTINE_24W_…																														
CELLMARKERMSIGDB_GAO_STOMACH_24W_C1_PROCR…																														
CELLMARKERMSIGDB_GAO_STOMACH_24W_C2_TFF2P…																														
CELLMARKERMSIGDB_GAO_STOMACH_24W_C4_PARIE…																														
CELLMARKERMSIGDB_GAO_STOMACH_24W_C5_PUTAT…																														
CELLMARKERMSIGDB_GAUTAM_EYE_IRIS_CILIARY_…	RBBP6	SNAPIN	SH3BP4	DLC1	CAPZA1	SNRNP…	BPGM	ZDHHC9	AP3D1	TRAPP…	FN3KRP	SRPRB	POGLU…	AGO3	ASAP2	TESK1	SBF2	IFNAR2	TJP1	LBHD1	TMEM2…	RDH14	SLC52…	CDH2	BTBD7	CNN2	MYO10	RNF213	ACKR3	NDUFB9
CELLMARKERMSIGDB_GAUTAM_EYE_IRIS_CILIARY_…																														
CELLMARKERMSIGDB_GAUTAM_EYE_IRIS_CILIARY_…																														
CELLMARKERMSIGDB_GAUTAM_EYE_IRIS_CILIARY_…																														
CELLMARKERMSIGDB_GAUTAM_EYE_IRIS_CILIARY_…																														
CELLMARKERMSIGDB_HAY_BONE_MARROW_CD34_POS…																														
CELLMARKERMSIGDB_HAY_BONE_MARROW_CD34_POS…																														
CELLMARKERMSIGDB_HAY_BONE_MARROW_CD34_POS…																														
CELLMARKERMSIGDB_HAY_BONE_MARROW_CD34_POS…																														
CELLMARKERMSIGDB_HAY_BONE_MARROW_CD8_T_CE…																														
CELLMARKERMSIGDB_HAY_BONE_MARROW_EARLY_ER…																														
CELLMARKERMSIGDB_HAY_BONE_MARROW_EOSINOPH…																														
CELLMARKERMSIGDB_HAY_BONE_MARROW_NAIVE_T_…	SBDS	LBH	PATJ	SEPTI…	CCDC57	LSR	MTFP1	AKTIP	PLK3	PAG1	ADD3	TMEM6…	GRAMD…	ARHGA…	NMT2	FBLN7	FAM17…	PPP1R2	TRAF1	LINS1	BLOC1…	LINC0…	SIT1	UBASH…	RNF157	SMCHD1	CYLD	VSIG1	RASA3	CYTH1
CELLMARKERMSIGDB_HAY_BONE_MARROW_NEUTROPH…	FES	LAMP2	RASGR…	UBXN11	JDP2	PTAFR	FAM12…	COX7B	PRNP	NLRP12	PADI2	SLC36…	ATOX1	MOB1A	CPD	RAB5IF	AGTPB…	GLT1D1	DOK3	MBOAT7	PTPN12	VAPA	EMB	AKIRI…	KIF13A	PRKACA	TOM1	NDUFA…	NAGA	FAR1
CELLMARKERMSIGDB_HAY_BONE_MARROW_NK_CELLS	APOL6	GSAP	APOBE…	LINC0…	STK10	DSTN	SLC1A7	IDI1	KLHDC4	TBCC	LRP10	MED10	POLR2…	POLR3…	ZNF276	NAALA…	VIPR2	PTPRA	FUT11	CAP1	PTGER2	ZMAT4	VPS37B	KIR3D…	SMKR1	RAP1G…	TOX-DT	MIR18…	STX8	ARL6I…
CELLMARKERMSIGDB_HAY_BONE_MARROW_PRE_DEND…																														
CELLMARKERMSIGDB_HAY_BONE_MARROW_PRO_B	RBM8A	NUDT5	RBBP8	RPRML	SRSF9	PPP1CC	PIM1	IMMP1L	H2BC18	HP1BP3	ACAT2	N4BP2	KHDRB…	H2BC7	IKZF2	HIRIP3	DERA	HAUS8	RTKN2	TRAIP	CAMK2D									
CELLMARKERMSIGDB_HAY_BONE_MARROW_STROMAL	TNS3	SPRY1	TNFRS…	TMEM98	TNFRS…	NDN	FST	RCN3	NRP1	RRAD	GPR88	PXDC1	NID1	HTRA1	DMD	TSPAN8	CBLN2	RGS16	NCKAP5	CRISP…	FBXO17	RARRE…	CLMP	CFI	PLEKH…	FGFR1	CH25H	ZNF503	THSD4	CACNA…
CELLMARKERMSIGDB_HE_LIM_SUN_FETAL_LUNG_C0…	ABHD1…	ITGB1…	SRF	ASAP2	SLC2A8	PRKAB2	KPNA2	BTN2A2	CDK5	DMD	BMPR1B	RPS6K…	MTHFD…	CTPS1	RAB3C	CACNA…	HSPG2	PGF	ARMC9	CCNQ	DMKN	CISD1	PCCA	IL17B	ADAMT…	CRYL1	MMP11	TMEM51	TK1	HOMER2
CELLMARKERMSIGDB_HE_LIM_SUN_FETAL_LUNG_C0…																														
CELLMARKERMSIGDB_HE_LIM_SUN_FETAL_LUNG_C0…																														
CELLMARKERMSIGDB_HE_LIM_SUN_FETAL_LUNG_C0…	CPNE2	STX11	FZD2	PSMG4	HOXC4	PICK1	CFI	GDE1	FLNB	CHST9	ANK3	DOK6	GPRC5…	CAP2	SVIP	ITM2A	EDN3	MIR99…	TMEM1…	ALCAM	CADM4	TRAPP…	CERS4	C1GAL…	KIAA1…	BICDL1	ASXL3	ARL4C	CRACR…	THNSL2
CELLMARKERMSIGDB_HE_LIM_SUN_FETAL_LUNG_C0…																														
CELLMARKERMSIGDB_HE_LIM_SUN_FETAL_LUNG_C0…																														
CELLMARKERMSIGDB_HE_LIM_SUN_FETAL_LUNG_C0…	LINC0…	VIPR2	PDZD2	DPYD	BCAT2	TOR1A…	CHRDL1	TNFAI…	C21or…	GSG1L	BMP3	CAMK1D	PALM	BCR	IFI27	P4HA2	NMI	SPINT1	MYO5B	TSPAN7	SLC7A4	RRAS	TM7SF2	CTXN1	FN3K	GOLM2	MIEN1	CCND3	TRNP1	CTSE
CELLMARKERMSIGDB_HE_LIM_SUN_FETAL_LUNG_C0…	LRR1	DKKL1	CENPL	CENPP	PDE3A	CDC25C	FANCA	ESCO2	BUB1B	TROAP	AURKA	KIF14	PAWR	COL23…	BRCA2	FOXM1	KNSTRN													
CELLMARKERMSIGDB_HE_LIM_SUN_FETAL_LUNG_C0…																														
CELLMARKERMSIGDB_HE_LIM_SUN_FETAL_LUNG_C0…	KIRRE…	CRB2	SP100	PKHD1…	RAB33A	KLK5	RIC3	TOM1L1	TRNP1	DCBLD2	SPEG	LRRC42	HTRA1	PODN	POMGN…	MTHFS	SLC38…	ADARB1	MYO1C	SLC4A2	ABHD1…	WASF1	CDC42…	CYBC1	NXPH2	CARNS1	COL8A2	ASPHD1	DOCK11	DNAJB…
CELLMARKERMSIGDB_HE_LIM_SUN_FETAL_LUNG_C0…																														
CELLMARKERMSIGDB_HE_LIM_SUN_FETAL_LUNG_C1…																														
CELLMARKERMSIGDB_HE_LIM_SUN_FETAL_LUNG_C1…																														
CELLMARKERMSIGDB_HE_LIM_SUN_FETAL_LUNG_C1…																														
CELLMARKERMSIGDB_HE_LIM_SUN_FETAL_LUNG_C1…	EDIL3	CNFN	STRA6	SPIND…	SKA3	PLCL2	ZSWIM6	FBXO5	SERPI…	KIT	HSPB8	BRI3BP	RFX6	CCDC18	PSMC3…	MYO9B	CHAF1B	H2BC11	ABHD3	E2F5	RMI1	KIF14	MICA	HSPA2	CEP57…	HDC	TEAD4	ANK2	PCDH18	FANCA
CELLMARKERMSIGDB_HE_LIM_SUN_FETAL_LUNG_C1…	GIT1	MIR7-…	HTR2C	WASF1	GNG2	LINC0…	SDC3	ARHGE…	LRRC24	STK32A	ELAVL4	TCEAL2	KAT2B	ITGB8	ST8SI…	ACAP3	LARP6	SV2A	REEP2	RUNDC…	A4GALT	NAAA	TEX2	NPIPB4	TRAM2	ACTL6B	CRMP1	SDK1	PLXNC1	TP53I…
CELLMARKERMSIGDB_HE_LIM_SUN_FETAL_LUNG_C1…																														
CELLMARKERMSIGDB_HE_LIM_SUN_FETAL_LUNG_C1…																														
CELLMARKERMSIGDB_HE_LIM_SUN_FETAL_LUNG_C1…																														
CELLMARKERMSIGDB_HE_LIM_SUN_FETAL_LUNG_C1…	TMEM1…	ACSL4	CDC42…	TRERF1	MAB21…	MCC	TLL1	SEMA3D	NAMPT…	CNTN3	SLC16…	ARSJ	TMEM1…	LNX1	SPTBN2	RNF150	TM4SF1	STXBP6												
CELLMARKERMSIGDB_HE_LIM_SUN_FETAL_LUNG_C1…	RASA4	NEURL1	ERC2	NAALA…	CCDC1…	RCOR2	ZMAT3	NEURL…	EGLN3	GPM6A	KCNA4	BTBD17	CPEB3	TIGAR	PPP2R…	WIPI1	TCEAL5	NRG1	TMEM51	ATL1	TBC1D…									
CELLMARKERMSIGDB_HE_LIM_SUN_FETAL_LUNG_C1…	DMRTA2	SEMA6D	SPRY4	CSTA	CCDC3	TYMP	FOXC1	LGALS9	SLIT3	LPL	CEP55	STOX1	OSR1	RND1	NUDT11	TNFAI…	NUPR1	SLC35…	ABCA13	EYA2	FST	OCA2	RFLNA	GINS3	NPB	ABCG1	HEY1	MGP	CCL2	KCNN4
CELLMARKERMSIGDB_HE_LIM_SUN_FETAL_LUNG_C1…	GHR	CHRM3	LINC0…	KRT23	ATP12A	S100B	PDF	CALCRL	CXCL8	BTBD6	PEX11G	GFM1	INHBA	LGR4	ANG	SZT2	BAK1	N4BP3	CSTA	PDZK1…	LGALS9	PITX1	GRTP1	FMOD	DENND…	FOXQ1	NIPAL2	FAM83H	INHBB	C17or…
CELLMARKERMSIGDB_HE_LIM_SUN_FETAL_LUNG_C2…	UBASH…	GBA1	C11or…	SERIN…	SELEN…	CCZ1B	DENND…	BACE2	SLC44…	PHACT…	TTC27	ERRFI1	HSD3B7	IFIH1	NIBAN2	DCHS1	MYO7A	LINC0…	OPTN	MED31	SMUG1	NUAK1	FNDC3B	SOCS6	S1PR1	SDSL	KCNJ5	ACOT2	SDC2	AP1AR
CELLMARKERMSIGDB_HE_LIM_SUN_FETAL_LUNG_C2…	TMEM3…	CRIP3	PELO	CTNNA…	NCKAP5	TMEM1…	GFOD1	SP140	NOP2	KIAA0…	NAA40	SPATS2	PRELI…	TMEM1…	ARSB	NBPF12	DUSP4	B3GNT7	STRADB	NMB	NAE1	CHRFA…	SCARF1	TMEM1…	TMEM1…	H2AC17	RAB3IP	CCDC1…	MBOAT2	KIAA1…
CELLMARKERMSIGDB_HE_LIM_SUN_FETAL_LUNG_C2…																														
CELLMARKERMSIGDB_HE_LIM_SUN_FETAL_LUNG_C2…																														
CELLMARKERMSIGDB_HE_LIM_SUN_FETAL_LUNG_C2…																														
CELLMARKERMSIGDB_HE_LIM_SUN_FETAL_LUNG_C2…	RCN1	ARHGE…	XXYLT…	CHRM3	NTHL1	MMAB	LCK	EMCN	MKS1	EFNA1	LINC0…	ZEB1	FUT8	ZNF254	FHIP2A	BRF2	CALCRL	PIGU	CLIP3	STRBP	CD3E	DLK1	PXDN	STARD9	ZNF521	ZNF574	CD79A	MTA3	PRKCQ	CRACD
CELLMARKERMSIGDB_HE_LIM_SUN_FETAL_LUNG_C2…	NBEAL2	ASB8	TMEM6…	PRUNE1	TANGO2	CASS4	ATP8A1	CTNNA…	SLMAP	VPS26B	VANGL1	CGRRF1	CXCL2	CD40LG	MACIR	C12or…	HBD	SRSF8	PLOD2	TSPAN…	WFDC1	FAM10…	TRPC6	FYN	MEST	SIAE	WDR44	ANKRD9	CKAP5	ITFG1
CELLMARKERMSIGDB_HE_LIM_SUN_FETAL_LUNG_C2…																														
CELLMARKERMSIGDB_HE_LIM_SUN_FETAL_LUNG_C2…	CDKL1	LANCL3	NUDT4	LMBRD2	ATG4D	AQP10	PKHD1…	PDGFA	ELOVL7	MFAP3L	CEP44	DIPK1B	KHDC4	OPTN	TRIM23	H2BC8	PTGIR	PLD6	SERPI…	GAB1	TNFSF4	ALOX12	UBE2H	PIM2	SGMS1	ZNF185	CPEB2	GDF15	MCTP2	TWSG1
CELLMARKERMSIGDB_HE_LIM_SUN_FETAL_LUNG_C2…	NOL10	SLC1A4	ZWINT	EOLA1	ACAT2	H2AX	LSG1	RCAN3	RRP9	SLC2A…	SARS2	NUSAP1	PIGO	STK26	GMNN	AKAP8	ZNF830	GPATC…	REXO4	PRMT7	MVD	MCM4	CALCRL	GON7	PTPRC…	SCN9A	LMNB2	LIG1	BEND6	SIGLE…
CELLMARKERMSIGDB_HE_LIM_SUN_FETAL_LUNG_C2…																														
CELLMARKERMSIGDB_HE_LIM_SUN_FETAL_LUNG_C2…																														
CELLMARKERMSIGDB_HE_LIM_SUN_FETAL_LUNG_C2…																														
CELLMARKERMSIGDB_HE_LIM_SUN_FETAL_LUNG_C3…																														
CELLMARKERMSIGDB_HE_LIM_SUN_FETAL_LUNG_C3…	CCNE1	HELLS	RCL1	ANLN	TUBB1	EXOSC3	MRC2	S100A4	KIF20A	C8orf…	SLC48…	ERI1	CENPH	CD82	PCCB	KIF4A	SYNGR1	TF	MLH1	NEIL3	ZWILCH	H4C11	ACSS1	EXOSC9	TTK	KIF11	CEP152	CCP110	RMI2	H2AC4
CELLMARKERMSIGDB_HE_LIM_SUN_FETAL_LUNG_C3…	SIPA1…	ENAH	ARMT1	AK4	VEGFA	HOXB4	TEX30	TOR2A	PPIF	UBL4A	H2AC25	CENPF	GOLM1	GFER	SYT1	NPAS2	UCP1	USB1	CTPS1	MMAB	EMC9	MPHOS…	THOP1	NAGA	PELO	NLE1	UNC119	FARSB	ACYP1	IFFO1
CELLMARKERMSIGDB_HE_LIM_SUN_FETAL_LUNG_C3…	CLIP3	PLD1	NFIA-…	OSBP	LYPD6	TSHZ1	DOCK5	CYP39…	LRCH2	LRIG3	HOXB4	NLGN4X	FZD10	GCNT2	CTTNB…	PAIP2B	CYP4X1	DOC2B	MFSD4A	LINC0…	KCNN3	CYP1B1	TSPYL2	PSEN2	PTGR3	RPP25	ADAM22	TM7SF2	GAS7	PTPN3
CELLMARKERMSIGDB_HE_LIM_SUN_FETAL_LUNG_C3…	ANKDD…	ENTRE…	CDKN1A	FCGR2A	GLIS3	GALNT…	UBAP1L	ADAMT…	FRZB	HSD3B7	APH1B	ABHD2	ASB13	TTN	NPNT	PLEKH…	USP13	HS3ST1	KLKB1	MITF	DNMBP	ST3GA…	VSTM4	MAN2A1	KIF3C	SCRN1	STK32B	GNB5	SLC26…	SUSD4
CELLMARKERMSIGDB_HE_LIM_SUN_FETAL_LUNG_C3…																														
CELLMARKERMSIGDB_HE_LIM_SUN_FETAL_LUNG_C3…	GBP2	SNX18	CLN8	TM6SF1	LMTK2	NCAM1	TLR5	PLD5	CALHM5	SH3RF2	SMTNL2	SLC8A1	SLC26…	BAMBI	PEG10	MCTP1	NMT2	ZBTB16	APPAT	CDO1	HAS2	MYRIP	EFEMP1	RTN4R…	LDLRA…	ZNF217	LOXL1	CRABP2	DPYD	SELL
CELLMARKERMSIGDB_HE_LIM_SUN_FETAL_LUNG_C3…	SLC35…	ADAM22	ZC3H8	MAPK7	SLC16…	POLB	TRMT9B	B3GNT8	MET	APBA2	RUVBL1	ABHD2	URI1	TRIP6	MIGA1	RASSF8	FAM98A	PDGFA	BSCL2	TULP4	PDE6G	DUSP2	UCHL1	COQ8A	OSTM1	NTN1	TANC2	GCNT2	CARS1	RUFY1
CELLMARKERMSIGDB_HE_LIM_SUN_FETAL_LUNG_C3…																														
CELLMARKERMSIGDB_HE_LIM_SUN_FETAL_LUNG_C4…																														
CELLMARKERMSIGDB_HE_LIM_SUN_FETAL_LUNG_C4…																														
CELLMARKERMSIGDB_HE_LIM_SUN_FETAL_LUNG_C4…																														
CELLMARKERMSIGDB_HE_LIM_SUN_FETAL_LUNG_C4…																														
CELLMARKERMSIGDB_HE_LIM_SUN_FETAL_LUNG_C4…	TM7SF3	REEP4	TEX30	TPGS2	POLA2	E2F3	CCNF	JADE1	H2AC4	DEPDC…	TTF2	VRK1	TEDC2	NUP107	PRC1	TP53I…	VPS26A	CX3CR1	CKAP5	KEAP1	MGST3	CBFB	FAF1	TPRKB	SMCO4	ENOSF1	CHRAC1	MLH1	CHAF1A	TUBGC…
CELLMARKERMSIGDB_HE_LIM_SUN_FETAL_LUNG_C4…	DSCC1	ACOT7	CTNNA…	H2BC18	COMMD4	SRPK1	NFATC…	POLD3	AURKA	LMF2	NCAPH2	CDCA7L	H2BC15	AUTS2	H2AC4	GMPS	CTDSP…	ATP1B3	ASRGL1	USP39	POLQ	CDK2	E2F8	IGFBP2	ORC6	MSH2	MCM4	MPHOS…	ACTL6A	PBRM1
CELLMARKERMSIGDB_HE_LIM_SUN_FETAL_LUNG_C4…																														

Table S5: Cell state marker gene list

CELLMARKERCAN_ACINAR_CELLS																														
CELLMARKERMSIGDB_HE_LIM_SUN_FETAL_LUNG_C4…																														
CELLMARKERMSIGDB_HE_LIM_SUN_FETAL_LUNG_C4…																														
CELLMARKERMSIGDB_HE_LIM_SUN_FETAL_LUNG_C4…																														
CELLMARKERMSIGDB_HE_LIM_SUN_FETAL_LUNG_C5…																														
CELLMARKERMSIGDB_HE_LIM_SUN_FETAL_LUNG_C5…	MRPL17	KDM2A	CINP	LPCAT1	GNG8	ADTRP	MCM2	TANK	SNX9	TSEN15	COMMD…	BCAT2	NENF	MTX1	GNL2	PNOC	PTPN22	CHD3	LMAN1	HILPDA	SAMSN1	ZBTB17	PELP1	CISD1	DPH2	CCDC8…	SDHAF3	CISD2	THEM4	ORMDL2
CELLMARKERMSIGDB_HE_LIM_SUN_FETAL_LUNG_C5…																														
CELLMARKERMSIGDB_HE_LIM_SUN_FETAL_LUNG_C5…																														
CELLMARKERMSIGDB_HE_LIM_SUN_FETAL_LUNG_C5…	KNSTRN	NPY	MELK	PXDN	H2BC15	RFWD3	POLD3	UCK2	GINS3	PLCB1	DNAJC…	FADS1	PIDD1	PLK4	PBK	CHD3	ALDH7…	FADS2	CENPA	WDHD1	CTBP2	SKA3	TROAP	CEP41	PGRMC1	H3C15	GSN	LMNB2	RFC3	RNF168
CELLMARKERMSIGDB_HE_LIM_SUN_FETAL_LUNG_C6…																														
CELLMARKERMSIGDB_HE_LIM_SUN_FETAL_LUNG_C7…																														
CELLMARKERMSIGDB_HE_LIM_SUN_FETAL_LUNG_C7…																														
CELLMARKERMSIGDB_HE_LIM_SUN_FETAL_LUNG_C7…																														
CELLMARKERMSIGDB_HE_LIM_SUN_FETAL_LUNG_C7…																														
CELLMARKERMSIGDB_HE_LIM_SUN_FETAL_LUNG_C7…	NALCN	SCML4	CTIF	GRIA2	MTMR7	CAMSA…	ABCC8	CTXN2	F8A1	BCAS4	RPS6K…	PPM1E	ASTN1	MARCH…	FABP3	SCD5	LINGO2	EIF4E3	SHANK2	DIABLO	HCN3	GABRA3	RGS11	LRFN2	DIRAS1	NT5E	ABCA3	TANC2	ATP8A2	FAM17…
CELLMARKERMSIGDB_HE_LIM_SUN_FETAL_LUNG_C7…																														
CELLMARKERMSIGDB_HU_FETAL_RETINA_AMACRINE																														
CELLMARKERMSIGDB_HU_FETAL_RETINA_BIPOLAR																														
CELLMARKERMSIGDB_HU_FETAL_RETINA_FIBROBLA…	PHLDB2	EPHB4	ETS2	SNORD…	LRIG3	LUZP1	SEC24D	PTH1R	ANPEP	MT-TC	ZFP36…	BCAP29	PLXNC1	CYBA	KCNQ1…	MAFF	WASF2	GNG11	KLF4	PARVA	DNAJB1	CRTAP	SMARC…	S100A…	LOXL2	LEPROT	SLC2A3	MAGT1	P3H1	HLA-A
CELLMARKERMSIGDB_HU_FETAL_RETINA_HORIZONT…																														
CELLMARKERMSIGDB_HU_FETAL_RETINA_MICROGLIA	DNAJB1	SIPA1	ARHGA…	TUT7	ITGAM	FOS	CAPZB	MOB1A	LGALS9	SPI1	PRKCD	METTL…	LCP2	GRK2	MGST2	CEBPB	RHOB	TMC6	MILR1	ACTR3	MYL12A	PPT1	LPXN	ARHGA…	CHCHD7	ARPC3	QKI	HSPA1A	HSPA1B	SMAP2
CELLMARKERMSIGDB_HU_FETAL_RETINA_MULLER																														
CELLMARKERMSIGDB_HU_FETAL_RETINA_PHOTOREC…																														
CELLMARKERMSIGDB_HU_FETAL_RETINA_RGC	DCTN2	SLC2A6	RTL8C	BEX1	RAP1G…	RAB2A	GTF2A2	MAP4	CALM2	SYT1	GRIA2	TMEM5…	FYN	LCOR	NTRK1	DCLK1	KIF5C	GDAP1	PHYHI…	BLOC1…	DBI	TMEM5…	GAPDH	TPD52	ARPP19	VKORC1	TLE4	SEZ6L2	SH3BG…	LNPK
CELLMARKERMSIGDB_LAKE_ADULT_KIDNEY_C1_EPI…																														
CELLMARKERMSIGDB_LAKE_ADULT_KIDNEY_C10_TH…	CLINT1	PNPLA8	OCIAD1	PRDX6	LINC0…	HNRNPM	EEF1D	GSPT1	STAT3	RPL41	ZMYND8	DSTN	APP	HLA-B	RPL12	OSBPL3	NUFIP2	ATP5F…	RPS24	SF3B1	TOP2B	PLPP3	ITGB1	DIAPH1	PPP2R…	HIPK3	RALGA…	RPS28	RPS9	CTNNA1
CELLMARKERMSIGDB_LAKE_ADULT_KIDNEY_C12_TH…	RHOBT…	SDHA	EPS8L2	RPL12	KIFC3	MGST3	IMMT	AIF1L	P4HB	RPL31	GHITM	MAGED1	S100A…	HPN	RPL35A	RPL15	FLNB	TPT1	SRP14	RPLP0	EEF2	RPL37	PIK3R1	IVNS1…	KCTD1	RPL6	RPL4	TACC1	RPL23	RPLP2
CELLMARKERMSIGDB_LAKE_ADULT_KIDNEY_C18_CO…	CNDP2	YWHAZ	CD2AP	RPL37A	HNRNP…	ANAPC…	HNRNPR	RHOBT…	ACSL4	HNRNP…	CSDE1	PCM1	SF3B1	UBXN4	MYO10	SEPTI…	DSTN	RNF213	TMSB10	JAK1	CD46	RPL23	HLA-A	CCNI	STYL2	KTN1	TPR	PAPOLA	EEF1D	RPS28
CELLMARKERMSIGDB_LAKE_ADULT_KIDNEY_C19_CO…	BTG1	SERBP1	RHOA	HSPA9	ENO1	GPBP1	APLP2	TMF1	ANAPC…	UBE2D3	JAK1	PAPOLA	PKM	HNRNP…	FUBP1	NFE2L2	YWHAB	ACTG1	CSDE1	KMT2E	RETRE…	CALM1	ZFAND5	WWP1	SLC38…	RASD1	RPS28	RALBP1	PSAP	SF3B1
CELLMARKERMSIGDB_LAKE_ADULT_KIDNEY_C25_EN…																														
CELLMARKERMSIGDB_LAKE_ADULT_KIDNEY_C28_IN…																														
CELLMARKERMSIGDB_LAKE_ADULT_KIDNEY_C29_UN…																														
CELLMARKERMSIGDB_LAKE_ADULT_KIDNEY_C5_PRO…	DOK6	DYNC2…	CUX1	ENOX1	FMN1	PRLR	DMD	FAM22…	AK9	CHD2	ZNF82…	PHF20	KAT6B	WDR13	GCNT2	BCL2	TAPT1…	WWOX	PPP1R…	CCAR1	KCNJ15	PLCG2	SMG6	CACNA…	SHROO…	PHF14	PRRC2C	CRADD	OPTN	EPS8L2
CELLMARKERMSIGDB_MANNO_MIDBRAIN_NEUROTYPE…																														
CELLMARKERMSIGDB_MANNO_MIDBRAIN_NEUROTYPE…	NFE2L3	NFIX	NLGN4X	NLK	NMNAT2	NNAT	NPY1R	NR4A2	NREP	NRXN1	NRXN2	NRXN3	NSG1	NTM	NTNG1	NTSR1	NYAP1	OLFM3	OPRK1	OPTN	OR4N2	PAK3	PAK6	PAK5	PAPPA	PARP8	PBX1	PBX3	PCDH1	PCDHG…
CELLMARKERMSIGDB_MANNO_MIDBRAIN_NEUROTYPE…	LRRC55	LRRC7	LRRTM2	LRRTM4	LSAMP	MAB21…	MAN2A2	MAP1B	MAP2	MAP3K9	MAP6	MAPK8	MARCH…	MARK1	MAST1	MAST2	MCF2L	MEG3	MFSD6	MIAT	MID1	MIR12…	MLLT11	MMD	MMP24	MSANT…	MTURN	MTUS2	MYEF2	MYRIP
CELLMARKERMSIGDB_MANNO_MIDBRAIN_NEUROTYPE…	NAPB	NCAM1	NDRG4	NEDD4L	NETO2	NFASC	NIPA1	NKAIN1	NKAIN2	NLN	NMNAT2	NNAT	NOL4	NPAS4	NPY1R	NR4A2	NREP	NRXN2	NRXN3	NSG1	NTM	NTSR1	NYAP1	OLFM3	OPRK1	OPTN	OSBPL6	PACS1	PAK3	PAK5
CELLMARKERMSIGDB_MANNO_MIDBRAIN_NEUROTYPE…	GDPD5	GGT5	GIMAP1	GIMAP4	GIMAP5	GIMAP6	GIMAP7	GIPC3	GJA1	GJA4	GJC2	GLRX	GMFG	GNA14	GNAI2	GNG11	GNG5	GPCPD1	GPD1L	ADGRF5	ADGRA2	GPR146	GPR155	GPR4	GRAP	TAMAL…	GRN	GRPEL2	GUK1	GZMA
CELLMARKERMSIGDB_MANNO_MIDBRAIN_NEUROTYPE…	KCNK13	KCNK3	KCNK6	KCTD12	KIAA1…	KIF14	KLF2	KLF3	KRT14	KRT17	KRT5	LAIR1	LAPTM5	LAT2	LCP1	P3H2	LGMN	LHFPL2	LILRA2	LILRB4	LIMS1	LIPA	LITAF	LPAR5	LPAR6	LPCAT2	LPL	LRRC8C	LRRK2	LTBR
CELLMARKERMSIGDB_MANNO_MIDBRAIN_NEUROTYPE…	JAKMI…	JAKMI…	JPH3	JPH4	ANOS1	KCNA3	KCNC1	KCNC2	KCND2	KCNIP4	KCNIP…	KCNK6	KCNMA1	KCTD7	KCTD8	FGF7P3	KHDRB…	ARFGE…	KIAA1…	KIF21A	KIF21B	KIF3A	KIF3B	KIF5C	KIFC2	KIRRE…	KIRRE…	KLC1	KLHL1	KLHL32
CELLMARKERMSIGDB_MANNO_MIDBRAIN_NEUROTYPE…	SYT6	SYT7	TAC1	TAGLN3	TBC1D3	TENM1	THSD7A	TMEM1…	TMEM1…	TMEM1…	TMEM1…	TMEM3…	TMSB1…	TOX3	TRIM36	TRIM67	TRMT13	TSC22…	TSIX	TTC9B	TUBA1A	TUBB2B	TUBB3	TULP4	UBE2Q…	UCHL1	UNC13A	USP44	VASH2	VWDE
CELLMARKERMSIGDB_MANNO_MIDBRAIN_NEUROTYPE…	PAPPA	PAPPA2	PAQR5	PAX5	PBX3	PCDH1…	PCDH7	PCDH9	PCGF3	PDE11A	PDP1	PDS5B	PDZD7	PEG3	PGM2L1	PHACT…	PHF21B	PHYHI…	PIANP	PITPN…	PLEKH…	PLXNA2	PLXNA3	PLXNA4	PLXNC1	PNMA2	PNOC	POU2F1	POU2F2	PLPP2
CELLMARKERMSIGDB_MANNO_MIDBRAIN_NEUROTYPE…	RIMBP2	RIPPL…	RNF157	RNF165	TRIM8	RTN1	RUFY2	RUNDC…	RUNDC…	RUSC1	RXRG	SCG5	SCN3A	SCN8A	SCN9A	SCRT2	SEMA3A	SEMA3C	SEMA3D	SEMA3E	SEMA4D	SEMA6D	SEPTI…	SEPTI…	SEZ6L2	SH3PX…	SHROO…	SIM2	SLC18…	SLC1A4
CELLMARKERMSIGDB_MANNO_MIDBRAIN_NEUROTYPE…	SCAMP2	SCARB1	SCD5	SCG3	SCN1A	SCN3A	SCRG1	SCUBE2	SDC3	SEL1L3	SERIN…	SERPI…	SEZ6	SEZ6L	SGO1	SGO2	SH3D19	SHC3	SIRT2	SKA3	SLC15…	SLC16…	SLC1A2	SLC22…	SLC25…	SLC35…	SLC44…	SLC6A1	SLC7A…	SLC9A…
CELLMARKERMSIGDB_MANNO_MIDBRAIN_NEUROTYPE…	GNG12	GP1BB	GPC1	GPER1	GPNMB	ADGRF5	ADGRA2	ADGRD1	GPR21	GPR4	GPRC5C	GPX8	GRM3	GRN	GSN	GSTK1	GUCY1…	GUCY1…	GUCY1…	HAAO	HADHB	HBA1	HBA2	HBB	HBEGF	HBG1	HBG2	HCST	HES1	HES4
CELLMARKERMSIGDB_MANNO_MIDBRAIN_NEUROTYPE…	VEPH1	VIM	VIT	WEE1	WNT5A	WNT7A	XRCC3	YAP1	ZC3H1…	ZFP36…	ZFP36…	ZGRF1	ZNF395	ZNF519	ZNF738	ZNF878	ZWINT													
CELLMARKERMSIGDB_MANNO_MIDBRAIN_NEUROTYPE…	TMEM1…	TMEM1…	CEMIP2	TMEM47	TMPO	TMSB1…	TOP2A	TOX3	TPBG	TPX2	TRABD…	TRIM59	TROAP	TSPAN…	TSPAN…	TSPAN…	TTK	TWIST1	TXLNB	TYMS	UBE2C	UBE2T	UHRF1	VEPH1	VIM	VIT	VRK1	VSTM2L	WEE1	WNT1
CELLMARKERMSIGDB_MANNO_MIDBRAIN_NEUROTYPE…	RTKN2	SALL1	SALL4	SCARN…	SDC1	SDC2	SEPTI…	SFRP2	SFXN2	SGO1	SGO2	SHROO…	SIVA1	SKA3	SLC4A1	SLC6A8	SMC2	SMC4	SNORD…	SNRPC	SNRPF	SOX2	SOX21	SPAG5	SPC25	SPDL1	STMN1	STON1	SVIP	SYNE2
CELLMARKERMSIGDB_MANNO_MIDBRAIN_NEUROTYPE…	TOX3	TPBG	TRABD…	TRAM2	TRIM59	TROAP	TSPAN…	TTK	TTR	TWIST1	TXLNB	TYMS	UCP2	UHRF1	VIM	VSTM2L	WEE1	WNT1	WNT5A	YAP1	ZFP36…	ZFP36…	ZMAT4	ZNF497						
CELLMARKERMSIGDB_MANNO_MIDBRAIN_NEUROTYPE…	SLC16…	SLC19…	SLC25…	SLIT1	SLIT2	SNTG1	SOX2	SOX21	SOX5	SOX6	SOX9	SPAG1	SPAG17	SPAG6	SPEF1	ITPRI…	DENND…	STOM	STON1	STON2	SULF2	TACR1	TAGLN2	TBC1D…	TCF12	DYNLT5	TEKT1	TENM3	TENM4	TES
CELLMARKERMSIGDB_MANNO_MIDBRAIN_NEUROTYPE…	KAT2B	KBTBD…	KCNE5	KCNIP1	KCNJ1	KCNK1	KCNMB1	KCNN3	KCNQ2	KCTD12	KCTD21	MYORG	CEP126	KIF1A	KIF9	KIRRE…	KLF15	KLHDC…	KLHL25	KLHL32	KRT14	KRT17	KRT5	LAMA4	LAMB2	LDLRA…	CNMD	P3H4	LFNG	LGALS…
CELLMARKERMSIGDB_MANNO_MIDBRAIN_NEUROTYPE…	NSMF	NTRK2	NTRK3	NUF2	NUSAP1	OBSL1	OMG	OPHN1	OSBPL6	PARD3B	PAX3	PAX7	PBK	PBXIP1	PCDH1	PDE1C	PDE4C	PDLIM3	PDLIM5	PDPN	PEA15	PEAK1	PHGDH	PHYHI…	PIAS2	PIF1	PIFO	PLCD3	PLIN3	PLP1
CELLMARKERMSIGDB_MANNO_MIDBRAIN_NEUROTYPE…	SLC6A1	SLC6A9	SLC7A…	SLITR…	SMOC1	SNORD…	SOX2	SOX2-…	SOX8	SOX9	SOX9-…	SPAG17	SPARC…	SPATA6	SPEF1	SPRED1	SPRY1	SPRY2	SPRY4	TAGLN2	TAOK3	TEKT1	TGFB2…	THSD4	TIMP4	TMBIM1	TMEM2…	TMEM2…	TNC	TNFRS…
CELLMARKERMSIGDB_MANNO_MIDBRAIN_NEUROTYPE…	KCNN3	KCTD12	CEP126	KIF5C	KIF9	KIFC2	KIRRE…	KLF15	KLF9	KLHDC…	KLHL32	LAMB2	LAMP1	LANCL2	LCA5	LDLRA…	CNMD	P3H4	LGALS…	LGI3	LIFR	LIMCH1	LIPA	LIPG	LITAF	LIX1	LIX1L	LMBRD1	LMCD1	ENSG0…
CELLMARKERMSIGDB_MANNO_MIDBRAIN_NEUROTYPE…	SNCG	SORCS3	SOX11	SOX4	SRCIN1	SRRM4	SSBP3	ST6GA…	ST8SI…	CDK7	STMN1	STMN2	STMN4	STXBP1	SV2A	SYBU	SYP	SYT13	SYT7	TAGLN3	TBC1D…	TENM2	TERF2…	TES	THSD7A	TMEM1…	TMEM1…	TMEM1…	TMEM3…	TRHDE
CELLMARKERMSIGDB_MANNO_MIDBRAIN_NEUROTYPE…	PHACT…	PI4KA	PIGZ	PITPN…	PITRM1	PKIA	PLCB1	PLCH2	PLCXD3	PLXNC1	PNCK	PNMA2	PNMA8B	POU2F2	PPFIA2	PPFIA4	PPM1H	PPP2R…	PRKACB	PRKAR…	PRKAR…	PRKCB	PRKCZ	PRRT2	PRRT4	PSD	PSD2	PTPRM	PTPRR	QDPR
CELLMARKERMSIGDB_MENON_FETAL_KIDNEY_0_CAP…																														
CELLMARKERMSIGDB_MENON_FETAL_KIDNEY_10_IM…																														
CELLMARKERMSIGDB_MENON_FETAL_KIDNEY_2_NEP…																														
CELLMARKERMSIGDB_MENON_FETAL_KIDNEY_3_STR…																														
CELLMARKERMSIGDB_MENON_FETAL_KIDNEY_6_COL…																														
CELLMARKERMSIGDB_MURARO_PANCREAS_ACINAR_C…	PPIC	FTH1	SLC12…	MOB3B	TMEM4…	FA2H	LAMB3	PYCR1	NOP53	NECTI…	NRARP	PABPC3	HSPB8	LDHA	RDX	LMO7	HSPB1	ZNF704	RPL22…	PGM1	NAMPT	CAMK1D	RETSAT	TNFAI…	ANXA4	CCNG1	TUBA1C	MTHFD2	MCCC1	PRR15L
CELLMARKERMSIGDB_MURARO_PANCREAS_ALPHA_C…	ABLIM1	LDLRA…	CEACA…	ENSG0…	ALPK3	GFOD2	RTL5	GPC6	LINC0…	SSBP2	ATP2B1	NR4A2	HNF1A…	APH1B	STXBP6	ZNRF1	STMN2	NLGN2	GRM4	TBL1X	HPS1	PIGT	RAB3C	RAB26	C12or…	CPNE3	RAB3G…	NME4	GRK3	SDC2
CELLMARKERMSIGDB_MURARO_PANCREAS_BETA_CELL	SELEN…	CLCN4	SNAP91	CACNA…	TMED6	GTF3C1	CMTM8	TERF2…	PPP2CB	KATNA…	INSIG1	MCC	PPP1R…	PNMA2	MT1F	RPH3AL	RPL5	NEFM	ATP6V…	NCOA3	SLC4A…	CADPS	INA	INPP5F	BNIP3	CIRBP	ALDOC	PHF1	SC5D	ANKH
CELLMARKERMSIGDB_MURARO_PANCREAS_ENDOTHEL…	SKAP2	KBTBD2	CDR2L	PGM2L1	CBL	ATP1B3	TSC22…	CYGB	PELO	PTPN12	HPCAL1	BGN	MECOM	TM4SF1	KCTD20	UTRN	ECE1	PDGFA	SPG7	CBLB	TBC1D8	LBH	COL5A3	TMSB10	RSU1	SH3KB…	SOX4	IFITM3	ZNF503	RIPOR1
CELLMARKERMSIGDB_MURARO_PANCREAS_EPSILON_…																														
CELLMARKERMSIGDB_MURARO_PANCREAS_MESENCHY…	SSC5D	GJD3	CSGAL…	P4HA3	NOTCH…	MFAP4	MGP	NR2F2	CHST15	PLOD1	IL33	G0S2	CKAP4	DLC1	S100A…	A2M	ADAM12	TFPI	SLC38…	PARVA	SUSD2	REST	AKAP12	AMOTL1	HYCC1	QKI	PTGS2	PMEPA1	ACTN1	CCDC7…
CELLMARKERMSIGDB_RUBENSTEIN_SKELETAL_MUSC…	SSB	ACTR2	PPP1CA	RPL7	MT-CO1	EEF1B2	MRPL52	SARAF	RPL36A	HERPU…	TSC22…	METTL9	TIMP1	EIF3L	RPL17	CLEC2B	DYNLT1	BLOC1…	KLF4	ATP5MJ	COX5A	COMMD6	TXNDC…	PGK1	POLE4	PLSCR1	UQCRF…	GSTP1	RPS4Y1	NDUFB3
CELLMARKERMSIGDB_RUBENSTEIN_SKELETAL_MUSC…	SRSF3	HSBP1	CCT4	CNBP	GADD4…	SDHC	PMP22	SEC63	MRPL20	ESF1	MDH2	NDUFS6	TAF7	BEX4	ARL6I…	U2AF1	NDUFS4	PARK7	PRDX3	RAN	HIGD2A	FAM16…	LSM5	HADHA	ARF4	DDAH2	TUBA1B			
CELLMARKERMSIGDB_RUBENSTEIN_SKELETAL_MUSC…	ARPC2	RPL6	TCEA1	COX20	NDUFA…	MRPL32	RSU1	NPTN	DBI	NDUFB2	SPCS1	MRPL33	DYNLT1	COX17	PRDX2	FAM16…	TMEM2…	MT2A	VDAC3	MT1E	EPS8	ATP5F…	KLHL42	NDUFA8	ATP5F…	DCTN2	ATP5PB	MT-ND2	BBX	FHL1
CELLMARKERMSIGDB_TRAVAGLINI_LUNG_ALVEOLAR…	MVB12B	PARP14	ARPC5	NXN	PON2	SDC1	ACTG1	HEBP1	AGRN	SDCBP	CYSTM1	KCNN3	SPINT2	OSGIN2	RNF128	FZD5	VCL	PTPN21	RANBP…	PFKFB3	ECEL1…	FKBP9	LAMC1	SLC7A7	CD63	SLC39…	SFTA1P	RIN2	CLIP4	TMEM59
CELLMARKERMSIGDB_TRAVAGLINI_LUNG_BASOPHIL…	PRDX1	SMIM3	MEIS2	RPL37A	MIR44…	KLRG1	RAB37	MACIR	PIGA	SIK1	RBMX	MALAT1	TMEM88	HNRNP…	NSMCE1	MORF4…	IL5RA	LAX1	EMP3	HSD17…	SYTL2	ALS2	ADRB2	WDR45B	UTS2	BTG2	PRXL2C	RPL24	ANKRD9	HNRNPU
CELLMARKERMSIGDB_TRAVAGLINI_LUNG_CAPILLAR…																														
CELLMARKERMSIGDB_TRAVAGLINI_LUNG_CAPILLAR…	TTC1	MOCS2	SNX5	FCN3	LRCH1	BNIP3	PDLIM2	S100A6	C9orf…	STXBP6	SVIP	ERGIC2	LGALS1	ZC3H13	CETN2	IRF1-…	PPP2R…	R3HCC1	MXRA7	UBE2Q2	RPL37	RPL22	IFI6	TAF15	RPS20	NOSTR…	TNFSF…	MYL12A	BTN3A1	RPS25
CELLMARKERMSIGDB_TRAVAGLINI_LUNG_CD4_MEMO…																														
CELLMARKERMSIGDB_TRAVAGLINI_LUNG_EREG_DEN…	EMILI…	ZYX	ARHGA…	ZDHHC…	ENG	PRELI…	CYTOR	GNB1	GGTA1	TLR4	PLIN3	SLC31…	ATP2B…	RNF130	SYAP1	GNG5	DPYD	ANXA2	ITGAX	PDE4A	HCLS1	TUBA1C	S100A6	TTYH3	IGSF6	C4orf…	PSTPI…	BRI3	RUNX1	DDX3Y
CELLMARKERMSIGDB_TRAVAGLINI_LUNG_IGSF21_D…																														
CELLMARKERMSIGDB_TRAVAGLINI_LUNG_MESOTHEL…	AK6	SERPI…	NONO	RPL32	FLNB	PDXK	RPL13	RPL13A	EPGN	SLC9A…	RPLP1	IGBP1	C2	NDUFA…	KDELR3	TPBG	SERPI…	MAN2B1	MARCK…	TUBB2A	MUC16	ISOC2	TNFRS…	PLA2G…	FAM11…	RPL26	COL5A2	TYMP	MRPL12	DUSP6
CELLMARKERMSIGDB_TRAVAGLINI_LUNG_MYOFIBRO…																														
CELLMARKERMSIGDB_TRAVAGLINI_LUNG_NATURAL_…																														
CELLMARKERMSIGDB_TRAVAGLINI_LUNG_NEUTROPH…	CCNJL	PRR13	METTL…	ZBTB7A	BRD4	RTN3	CEBPZ	ARHGE…	ADM	CREB5	FTH1P8	DNAH17	FAM89B	ZFAS1	FTH1P7	MEFV	BST1	NDUFB3	RUBCNL	WDFY3	CNPPD1	TNFAI…	PLPPR2	MAST3	PPP1R…	ZNF638	BTG2	TKT	PGD	NOP10
CELLMARKERMSIGDB_TRAVAGLINI_LUNG_OLR1_CLA…	ACTB	LAIR1	STEAP4	IL6	VDR	FNDC3A	PIK3R5	CYRIA	CDKN1A	HS3ST…	SLC1A3	PDLIM7	SLC43…	SIGLE…	IL4I1	SKIL	CCL7	ZFP36…	TOP1	ECE1	TLE3	ASPH	CASP4	SERP1	TOM1	TNFAI…	PKM	EMILI…	MAFB	METRNL
CELLMARKERMSIGDB_TRAVAGLINI_LUNG_PLATELET…	SYTL4	NT5M	BMP6	RHOBT…	PNMA1	MPL	LEPROT	RGS6	AGR3	CYP4B1	PIP4K…	GABAR…	TUBB4B	GUCY1…	EIF4G2	MYCT1	VAPA	LINC0…	LINC0…	ENDOD1	BCL2L1	CYTOR	TSPAN…	NORAD	TPPP3	CFAP1…	AP2M1	TBC1D…	CAMTA1	WFDC2
CELLMARKERMSIGDB_TRAVAGLINI_LUNG_PROLIFER…	PPIA	BEND5	SERPI…	H1-4	FERMT1	LOC38…	OAF	RACK1	PXMP2	GUSB	ZNF581	RPS7	TSKU	CCT2	SUN2	LRRCC1	CXCL14	EIF2D	DLEU1	TP63	DCK	TEX30	MZT1	RND3	GOT2	KCTD1	KRT19	PAICS	ADSL	PERP
CELLMARKERMSIGDB_TRAVAGLINI_LUNG_PROLIFER…	SVIL	CD68	AKIP1	CXCL3	CBR1	MAGOHB	SERIN…	IL17RB	ADPGK	MSRB2	UBB	GCHFR	CD151	POLR3K	RPL26…	LAMP2	KPNA2	ARHGA…	ZDHHC…	MZT1	NIT2	EMC9	ANPEP	RMDN3	ATP2C1	PIMREG	BCL2L…	CD59	ACAT2	BTG3
CELLMARKERMSIGDB_TRAVAGLINI_LUNG_PROXIMAL…	PKM	HAUS4	RPL23A	RPL27A	CLSTN1	LINC0…	VDAC2	STIM2	HSPA2	PDCD4	PLPP2	SMC2	LAMP1	NET1	RPS12	AOPEP	RGS12	ABHD1…	GSTK1	EPRS1	TFAP2A	MEIS2	PRXL2A	C1QBP	ST6GA…	EIF3G	CCNB1…	SDC1	POLR1D	TXNIP
CELLMARKERMSIGDB_TRAVAGLINI_LUNG_SEROUS_C…																														
CELLMARKERMSIGDB_TRAVAGLINI_LUNG_SIGNALIN…																														
CELLMARKERMSIGDB_TRAVAGLINI_LUNG_TREM2_DE…	RRAS	ARHGA…	MARCKS	QPCT	PLEKH…	PGD	SCCPDH	RHOB	PLPP3	ANXA5	NAA20	ATP6V…	GCHFR	DST	ATG7	VDAC1	IL3RA	HMOX1	LACTB	MNDA	MTCH2	HSD17…	GPCPD1	MFSD12	CLIC2	SLC29…	BLVRB	RASSF4	ISOC2	VOPP1
CELLMARKERMSIGDB_TRAVAGLINI_LUNG_VASCULAR…																														
CELLMARKERMSIGDB_TRAVAGLINI_LUNG_VEIN_CELL																														
CELLMARKERMSIGDB_VANGURP_PANCREATIC_ALPHA…																														
CELLMARKERMSIGDB_VANGURP_PANCREATIC_DELTA…																														
CELLMARKERMSIGDB_VANGURP_PANCREATIC_GAMMA…																														
CELLMARKERMSIGDB_ZHENG_CORD_BLOOD_C1_PUTA…																														
CELLMARKERMSIGDB_ZHENG_CORD_BLOOD_C10_MUL…																														
CELLMARKERMSIGDB_ZHENG_CORD_BLOOD_C2_PUTA…																														
CELLMARKERMSIGDB_ZHENG_CORD_BLOOD_C4_PUTA…																														
CELLMARKERMSIGDB_ZHENG_CORD_BLOOD_C5_SIMI…																														
CELLMARKERMSIGDB_ZHENG_CORD_BLOOD_C6_HSC_…																														
CELLMARKERMSIGDB_ZHENG_CORD_BLOOD_C7_PUTA…																														
CELLMARKERMSIGDB_ZHENG_CORD_BLOOD_C8_PUTA…																														
CELLMARKERMSIGDB_ZHONG_PFC_C1_ASTROCYTE																														
CELLMARKERMSIGDB_ZHONG_PFC_C1_DLX5_POS_IN…																														
CELLMARKERMSIGDB_ZHONG_PFC_C2_ASCL1_POS_A…																														

Table S5: Cell state marker gene list

CELLMARKERCAN_ACINAR_CELLS																														
CELLMARKERMSIGDB_ZHONG_PFC_C2_UNKNOWN_NPC																														
CELLMARKERMSIGDB_ZHONG_PFC_C3_ASTROCYTE	CUL9	PHACT…	JPH4	MAST3	MEIS3	GEMIN5	RAB3A	PRPF18	TRIM41	CHGA	SNAP91	EVI5L	ELAVL3	SYP	CNKSR2	CBFA2…	NPIPA5	ARHGA…	CTTNB…	GAP43	ME3	SLC45…	BFAR	ACVR2B	GOLGA…	HEATR…	IBA57	VPS16	NEAT1	DYNC1…
CELLMARKERMSIGDB_ZHONG_PFC_C3_HOPX_POS_OPC																														
CELLMARKERMSIGDB_ZHONG_PFC_C3_MICROGLIA	CPLX1	GNG8	AMPD2	TUBB3	MOK	PPP1R…	VSTM2B	MPPED1	EXD3	TUBB2A	CXXC4	ACTN2	MFSD10	CACNA…	ANKRD…	THRA	LY6H	VSTM2L	STMN2	TENM2	AGTPB…	NAP1L3	SEPTI…	SYBU	PNMA3	SSBP3	GRIN1	RNU6-2	RFNG	FBXO3
CELLMARKERMSIGDB_ZHONG_PFC_C3_UNKNOWN_INP																														
CELLMARKERMSIGDB_ZHONG_PFC_C4_UNKNOWN_INP																														
CELLMARKERMSIGDB_ZHONG_PFC_C6_PLXNA4_POS_…																														
CELLMARKERMSIGDB_ZHONG_PFC_C7_ORG_UNDERGO…																														
CELLMARKERMSIGDB_ZHONG_PFC_C8_ORG_PROLIFE…																														
CELLMARKERMSIGDB_ZHONG_PFC_C8_UNKNOWN_NEU…																														
CELLMARKERMSIGDB_ZHONG_PFC_C9_ORG_OTHER																														
CELLMARKERMSIGDB_ZHONG_PFC_HES1_POS_C1_NPC																														
CELLMARKERMSIGDB_ZHONG_PFC_MAJOR_TYPES_AS…	RCN1	LEPROT	EMC2	GNG5	DOK5	CAMK2G	JUN	ABCD3	SHISA4	GNPTG	SNX3	SMIM30	SOAT1	FADS2	HMGCS1	B3GLCT	TMEM1…	SERPI…	SLC6A1	ELN	ZFP36…	BBS2	FABP5	PLEC	APBB2	KLHDC…	DCXR	DARS1	LIPA	P3H4
CELLMARKERMSIGDB_ZHONG_PFC_MAJOR_TYPES_EX…																														
CELLMARKERMSIGDB_ZHONG_PFC_MAJOR_TYPES_MI…	FAM78A	FOS	ANXA11	FGD2	CASP1	GNB4	OTUD1	SMAP2	IER2	TMBIM1	LPCAT2	MFSD1	LIMS1	ALOX5	WDR91	RB1	PLTP	PPT1	ARHGA…	RHOB	CYFIP1	RNF135	EGR1	B3GNT5	RNF213	GPX1	STOM	DPP7	SERPI…	PLCB2
CELLMARKERMSIGDB_ZHONG_PFC_MAJOR_TYPES_OPC																														

CELLMARKERCAN_ACINAR_CELLS																														
CELLMARKERCAN_ADIPOCYTE_PROGENITOR_CELLS																														
CELLMARKERCAN_ADRENERGIC_NEURONS																														
CELLMARKERCAN_AIRWAY_EPITHELIAL_CELLS																														
CELLMARKERCAN_ALPHA_CELLS																														
CELLMARKERCAN_ALVEOLAR_MACROPHAGES																														
CELLMARKERCAN_ASTROCYTES																														
CELLMARKERCAN_B_CELLS																														
CELLMARKERCAN_B_CELLS_MEMORY																														
CELLMARKERCAN_B_CELLS_NAIVE																														
CELLMARKERCAN_BASOPHILS																														
CELLMARKERCAN_BERGMANN_GLIA																														
CELLMARKERCAN_BETA_CELLS																														
CELLMARKERCAN_CAJAL_RETZIUS_CELLS																														
CELLMARKERCAN_CARDIAC_STEM_AND_PRECURSOR_…																														
CELLMARKERCAN_CARDIOMYOCYTES																														
CELLMARKERCAN_CHOLANGIOCYTES																														
CELLMARKERCAN_CHOLINERGIC_NEURONS																														
CELLMARKERCAN_CHONDROCYTES																														
CELLMARKERCAN_CHOROID_PLEXUS_CELLS																														
CELLMARKERCAN_CILIATED_CELLS																														
CELLMARKERCAN_DELTA_CELLS																														
CELLMARKERCAN_DOPAMINERGIC_NEURONS																														
CELLMARKERCAN_DUCTAL_CELLS																														
CELLMARKERCAN_EMBRYONIC_STEM_CELLS																														
CELLMARKERCAN_ENTERIC_GLIA_CELLS																														
CELLMARKERCAN_ENTERIC_NEURONS																														
CELLMARKERCAN_ENTEROCYTES																														
CELLMARKERCAN_ENTEROENDOCRINE_CELLS																														
CELLMARKERCAN_EPENDYMAL_CELLS																														
CELLMARKERCAN_EPSILON_CELLS																														
CELLMARKERCAN_FOLLICULAR_CELLS																														
CELLMARKERCAN_FOVEOLAR_CELLS																														
CELLMARKERCAN_GAMMA_DELTA_T_CELLS																														
CELLMARKERCAN_GERM_CELLS																														
CELLMARKERCAN_GLUTAMINERGIC_NEURONS																														
CELLMARKERCAN_GRANULOSA_CELLS																														
CELLMARKERCAN_HEMATOPOIETIC_STEM_CELLS																														
CELLMARKERCAN_IMMATURE_NEURONS																														
CELLMARKERCAN_INTERNEURONS																														
CELLMARKERCAN_IONOCYTES																														
CELLMARKERCAN_KERATINOCYTES																														
CELLMARKERCAN_KUPFFER_CELLS																														
CELLMARKERCAN_LEYDIG_CELLS																														
CELLMARKERCAN_LOOP_OF_HENLE_CELLS																														
CELLMARKERCAN_LUMINAL_EPITHELIAL_CELLS																														
CELLMARKERCAN_LUTEAL_CELLS																														
CELLMARKERCAN_MEGAKARYOCYTES																														
CELLMARKERCAN_MELANOCYTES																														
CELLMARKERCAN_MESANGIAL_CELLS																														
CELLMARKERCAN_MESOTHELIAL_CELLS																														
CELLMARKERCAN_MICROFOLD_CELLS																														
CELLMARKERCAN_MICROGLIA																														
CELLMARKERCAN_MONOCYTES																														
CELLMARKERCAN_MOTOR_NEURONS																														
CELLMARKERCAN_MULLER_CELLS																														
CELLMARKERCAN_MYELOID_DERIVED_SUPPRESSOR_…																														
CELLMARKERCAN_MYOBLASTS																														
CELLMARKERCAN_MYOEPITHELIAL_CELLS																														
CELLMARKERCAN_MYOFIBROBLASTS																														
CELLMARKERCAN_NEUROBLASTS																														
CELLMARKERCAN_NEUROENDOCRINE_CELLS																														
CELLMARKERCAN_NEURONS																														
CELLMARKERCAN_NEUTROPHILS																														
CELLMARKERCAN_NK_CELLS																														
CELLMARKERCAN_NORADRENERGIC_NEURONS																														
CELLMARKERCAN_NUOCYTES																														
CELLMARKERCAN_OLIGODENDROCYTES																														
CELLMARKERCAN_OSTEOBLASTS																														
CELLMARKERCAN_OSTEOCLAST_PRECURSOR_CELLS																														
CELLMARKERCAN_OSTEOCLASTS																														
CELLMARKERCAN_OSTEOCYTES																														
CELLMARKERCAN_PANETH_CELLS																														
CELLMARKERCAN_PARATHYROID_CHIEF_CELLS																														
CELLMARKERCAN_PERICYTES																														
CELLMARKERCAN_PERITUBULAR_MYOID_CELLS																														
CELLMARKERCAN_PHOTORECEPTOR_CELLS																														
CELLMARKERCAN_PLASMA_CELLS																														
CELLMARKERCAN_PLASMACYTOID_DENDRITIC_CELLS																														
CELLMARKERCAN_PODOCYTES																														
CELLMARKERCAN_PROXIMAL_TUBULE_CELLS																														
CELLMARKERCAN_PULMONARY_ALVEOLAR_TYPE_I_C…																														
CELLMARKERCAN_PULMONARY_ALVEOLAR_TYPE_II_…																														
CELLMARKERCAN_PURKINJE_NEURONS																														
CELLMARKERCAN_RETICULOCYTES																														
CELLMARKERCAN_RETINAL_GANGLION_CELLS																														
CELLMARKERCAN_RETINAL_PROGENITOR_CELLS																														
CELLMARKERCAN_SATELLITE_CELLS																														
CELLMARKERCAN_SCHWANN_CELLS																														
CELLMARKERCAN_SEROTONERGIC_NEURONS																														
CELLMARKERCAN_SERTOLI_CELLS																														
CELLMARKERCAN_SMOOTH_MUSCLE_CELLS																														
CELLMARKERCAN_STROMAL_CELLS																														
CELLMARKERCAN_T_CELLS																														
CELLMARKERCAN_T_CYTOTOXIC_CELLS																														
CELLMARKERCAN_T_FOLLICULAR_HELPER_CELLS																														
CELLMARKERCAN_T_MEMORY_CELLS																														
CELLMARKERCAN_TRANSIENT_CELLS																														
CELLMARKERCAN_TRICHOCYTES																														
CELLMARKERCAN_TRIGEMINAL_NEURONS																														
CELLMARKERCAN_TROPHOBLAST_CELLS																														
CELLMARKERCAN_TROPHOBLAST_PROGENITOR_CELLS																														
CELLMARKERCAN_TUFT_CELLS																														
CELLMARKERCANENDOTHELIALCELLSAORTA																														
CELLMARKERCUR_N_ABNORMAL_MYELOID_CELL																														

CELLMARKERCAN_ACINAR_CELLS																														
CELLMARKERCUR_N_ABNORMAL_PLASMA_CELL																														
CELLMARKERCUR_N_ACTIVATED_DENDRITIC_CELL																														
CELLMARKERCUR_N_ACTIVATED_EFFECTOR_CELL																														
CELLMARKERCUR_N_ACTIVATED_HEPATIC_STELLAT…																														
CELLMARKERCUR_N_ACTIVATED_LANGERHANS_CELL																														
CELLMARKERCUR_N_ACTIVATED_MACROPHAGE																														
CELLMARKERCUR_N_ACTIVATED_MEMORY_T_CELL																														
CELLMARKERCUR_N_ACTIVATED_NATURAL_KILLER_…																														
CELLMARKERCUR_N_ACTIVATED_REGULATORY_T_CE…																														
CELLMARKERCUR_N_ADIPOCYTE_PROGENITOR_CELL																														
CELLMARKERCUR_N_ADRENAL_MEDULLA_CELL																														
CELLMARKERCUR_N_ADULT_SPERMATOGONIA																														
CELLMARKERCUR_N_ADVENTITIAL_CELL																														
CELLMARKERCUR_N_AEROCYTE																														
CELLMARKERCUR_N_AIRWAY_SMOOTH_MUSCLE_CELL																														
CELLMARKERCUR_N_ALVEOLAR_CELL_TYPE_1																														
CELLMARKERCUR_N_ALVEOLAR_CELL_TYPE_2																														
CELLMARKERCUR_N_ALVEOLAR_EPITHELIAL_CELL_…																														
CELLMARKERCUR_N_ALVEOLAR_EPITHELIAL_CELL_…																														
CELLMARKERCUR_N_ALVEOLAR_PNEUMOCYTE_TYPE_I																														
CELLMARKERCUR_N_ALVEOLAR_PNEUMOCYTE_TYPE_…																														
CELLMARKERCUR_N_AMACRINE_CELL																														
CELLMARKERCUR_N_AMNIOTIC_FLUID_STEM_CELL																														
CELLMARKERCUR_N_ANTERIOR_CELL																														
CELLMARKERCUR_N_ANTERIOR_HINDBRAIN_CELL																														
CELLMARKERCUR_N_ANTI_TUMOR_IMMUNE_CELL																														
CELLMARKERCUR_N_ANTIGEN_PRESENTATION_CANC…																														
CELLMARKERCUR_N_ASTROCYTE																														
CELLMARKERCUR_N_ATRIAL_CELL																														
CELLMARKERCUR_N_ATRIOVENTRICULAR_CANAL_CE…																														
CELLMARKERCUR_N_ATYPICAL_MEMORY_B_CELL																														
CELLMARKERCUR_N_B_2_CELL																														
CELLMARKERCUR_N_B1_CELL																														
CELLMARKERCUR_N_B10_REGULATORY_B_CELL																														
CELLMARKERCUR_N_BASAL_CELL																														
CELLMARKERCUR_N_BASAL_EPITHELIAL_CELL																														
CELLMARKERCUR_N_BASAL_KERATINOCYTE																														
CELLMARKERCUR_N_BASAL_LIKE_CELL																														
CELLMARKERCUR_N_BASAL_PROGENITOR_CELL																														
CELLMARKERCUR_N_BASOPHIL																														
CELLMARKERCUR_N_BILIARY_CELL																														
CELLMARKERCUR_N_BONE_MARROW_STEM_CELL																														
CELLMARKERCUR_N_BONE_SPECIFIC_CELL																														
CELLMARKERCUR_N_BRAIN_CELL																														
CELLMARKERCUR_N_CANCER_CELL																														
CELLMARKERCUR_N_CANCER_STEM_LIKE_CELL																														
CELLMARKERCUR_N_CAPILLARY_CELL																														
CELLMARKERCUR_N_CAR_T_CELL																														
CELLMARKERCUR_N_CARDIAC_CELL																														
CELLMARKERCUR_N_CARDIOMYOCYTE																														
CELLMARKERCUR_N_CARDIOSPHERE_DERIVED_CELL																														
CELLMARKERCUR_N_CARDIOVASCULAR_PROGENITOR…																														
CELLMARKERCUR_N_CAUDAL_CORTEX_CELL																														
CELLMARKERCUR_N_CD14_MONOCYTE																														
CELLMARKERCUR_N_CD16_MONOCYTE																														
CELLMARKERCUR_N_CD4_CD28_T_CELL																														
CELLMARKERCUR_N_CD4_PERIPHERALLY_DERIVED_…																														
CELLMARKERCUR_N_CD4_T_CELL																														
CELLMARKERCUR_N_CD56BRIGHT_NATURAL_KILLER…																														
CELLMARKERCUR_N_CD56DIM_NATURAL_KILLER_CE…																														
CELLMARKERCUR_N_CD8_T_CELL																														
CELLMARKERCUR_N_CENTRAL_MEMORY_T_CELL																														
CELLMARKERCUR_N_CENTRAL_MEMORY_T_HELPER_1…																														
CELLMARKERCUR_N_CENTRAL_NERVOUS_SYSTEM_ST…																														
CELLMARKERCUR_N_CHANDELIER_CELL																														
CELLMARKERCUR_N_CHIEF_CELL																														
CELLMARKERCUR_N_CHONDROCYTE																														
CELLMARKERCUR_N_CHROMAFFIN_CELL																														
CELLMARKERCUR_N_CIRCULATING_ANGIOGENIC_CE…																														
CELLMARKERCUR_N_CIRCULATING_FETAL_CELL																														
CELLMARKERCUR_N_CIRCULATING_NATURAL_KILLE…																														
CELLMARKERCUR_N_CIRCULATING_PRECURSOR_CELL																														
CELLMARKERCUR_N_CIRCULATING_PROGENITOR_CE…																														
CELLMARKERCUR_N_CIRCULATING_T_FOLLICULAR_…																														
CELLMARKERCUR_N_CIRCULATING_TUMOR_CELL																														
CELLMARKERCUR_N_CLASS_SWITCHED_PLASMABLAST																														
CELLMARKERCUR_N_CLASSICAL_B_CELL																														
CELLMARKERCUR_N_CLASSICAL_CANCER_ASSOCIAT…																														
CELLMARKERCUR_N_CLASSICAL_MONOCYTE																														
CELLMARKERCUR_N_CLAUDIN_LOW_CELL																														
CELLMARKERCUR_N_COLLECTING_DUCTAL_CELL																														
CELLMARKERCUR_N_COLONCYTE																														
CELLMARKERCUR_N_COLORECTAL_STEM_CELL																														
CELLMARKERCUR_N_COLUMNAR_EPITHELIAL_CELL																														
CELLMARKERCUR_N_COMMON_LYMPHOID_PROGENITO…																														
CELLMARKERCUR_N_COMMON_MYELOID_PROGENITOR…																														
CELLMARKERCUR_N_COMPLEMENT_SECRETING_CANC…																														
CELLMARKERCUR_N_CONE_PHOTORECEPTOR_CELL																														
CELLMARKERCUR_N_CONNECTING_TUBULAR_CELL																														
CELLMARKERCUR_N_CONVENTIONAL_CD4_T_CELL																														
CELLMARKERCUR_N_CORNEAL_ENDOTHELIAL_CELL																														
CELLMARKERCUR_N_CORNEAL_EPITHELIAL_CELL																														
CELLMARKERCUR_N_CORNEOCYTE_LIKE_CELL																														
CELLMARKERCUR_N_CORTICAL_CELL																														
CELLMARKERCUR_N_CORTICAL_STROMAL_CELL																														
CELLMARKERCUR_N_CORTICOTROPH_TUMOR_CELL																														
CELLMARKERCUR_N_CRANIAL_NEURAL_CREST_CELL																														
CELLMARKERCUR_N_CRYPT_CELL																														
CELLMARKERCUR_N_CRYPT_STEM_CELL																														
CELLMARKERCUR_N_CYCLING_B_CELL																														
CELLMARKERCUR_N_CYCLING_EPITHELIAL_CELL																														
CELLMARKERCUR_N_CYTOTOXIC_CD8_T_CELL																														
CELLMARKERCUR_N_CYTOTOXIC_DOUBLE_POSITIVE…																														
CELLMARKERCUR_N_CYTOTOXIC_T_CELL																														

CELLMARKERCAN_ACINAR_CELLS																														
CELLMARKERCUR_N_CYTOTOXIC_T_CELL_2																														
CELLMARKERCUR_N_DAMAGE_ASSOCIATED_TRANSIE…																														
CELLMARKERCUR_N_DARK_ZONE_CELL																														
CELLMARKERCUR_N_DEDIFFERENTIATION_ADIPOCY…																														
CELLMARKERCUR_N_DEEP_LAYER_NEURON																														
CELLMARKERCUR_N_DEFINITIVE_ENDODERM_CELL																														
CELLMARKERCUR_N_DELTA_CELL																														
CELLMARKERCUR_N_DEMYELINATING_MICROGLIAL_…																														
CELLMARKERCUR_N_DENDRITIC_CELL_2A																														
CELLMARKERCUR_N_DENDRITIC_CELL_LINEAGE																														
CELLMARKERCUR_N_DENTAL_PULP_CELL																														
CELLMARKERCUR_N_DERMAL_CELL																														
CELLMARKERCUR_N_DEUTEROSOMAL_CELL																														
CELLMARKERCUR_N_DIFFUSE_GASTRIC_CANCER_CE…																														
CELLMARKERCUR_N_DISTAL_CELL																														
CELLMARKERCUR_N_DISTAL_STEM_CELL																														
CELLMARKERCUR_N_DISTAL_TUBULAR_CELL																														
CELLMARKERCUR_N_DIVIDING_CELL																														
CELLMARKERCUR_N_DIVIDING_PLASMA_B_CELL																														
CELLMARKERCUR_N_DIVIDING_T_CELL																														
CELLMARKERCUR_N_DOPAMINERGIC_NEURON																														
CELLMARKERCUR_N_DORSAL_ANTERIOR_HINDBRAIN…																														
CELLMARKERCUR_N_DORSAL_LATERAL_GANGLIONIC…																														
CELLMARKERCUR_N_DORSAL_MEDIAL_GANGLIONIC_…																														
CELLMARKERCUR_N_DOUBLE_NEGATIVE_B_CELL																														
CELLMARKERCUR_N_DOUBLE_NEGATIVE_T_CELL																														
CELLMARKERCUR_N_DOUBLE_POSITIVE_T_CELL																														
CELLMARKERCUR_N_DOUBLE_POSITIVE_TREG_CELL																														
CELLMARKERCUR_N_EARLY_HEMANGIOBLAST																														
CELLMARKERCUR_N_EARLY_PROXIMAL_TUBULE_CELL																														
CELLMARKERCUR_N_EARLY_RESPONSE_SECRETORY_…																														
CELLMARKERCUR_N_EFFECTOR_CHONDROCYTE																														
CELLMARKERCUR_N_EFFECTOR_MEMORY_T_CELL																														
CELLMARKERCUR_N_EFFECTOR_NATURAL_KILLER_C…																														
CELLMARKERCUR_N_ELONGATING_CELL																														
CELLMARKERCUR_N_EMBRYONIC_CELL																														
CELLMARKERCUR_N_EMBRYONIC_STEM_CELL																														
CELLMARKERCUR_N_ENDOCARDIAL_CELL																														
CELLMARKERCUR_N_ENDOCRINE_PROGENITOR_CELL																														
CELLMARKERCUR_N_ENDODERM_PROGENITOR_CELL																														
CELLMARKERCUR_N_ENDODERMAL_CELL																														
CELLMARKERCUR_N_ENDOMETRIAL_STROMAL_CELL																														
CELLMARKERCUR_N_ENDOMETRIAL_STROMAL_STEM_…																														
CELLMARKERCUR_N_ENDOTHELIAL_PRECURSOR_CELL																														
CELLMARKERCUR_N_ENSHEATHING_GLIAL_CELL																														
CELLMARKERCUR_N_ENTERIC_NEURON																														
CELLMARKERCUR_N_EOSINOPHIL																														
CELLMARKERCUR_N_EPENDYMAL_CELL																														
CELLMARKERCUR_N_EPIBLAST_CELL																														
CELLMARKERCUR_N_EPICARDIAL_CELL																														
CELLMARKERCUR_N_EPICARDIUM_DERIVED_CELL																														
CELLMARKERCUR_N_EPIDERMAL_CELL																														
CELLMARKERCUR_N_EPIDERMAL_STEM_CELL																														
CELLMARKERCUR_N_EPITHELIAL_CELL	CRYAB	OCIAD2	LAMC2	UBE2C	ADH7	CRABP2	TOP2A	CDKN2A	CEACA…	LY6D	SELEN…																			
CELLMARKERCUR_N_EPITHELIAL_DUCTAL_CELL																														
CELLMARKERCUR_N_EPITHELIAL_MESENCHYMAL_CE…																														
CELLMARKERCUR_N_EPITHELIOID_MACROPHAGE																														
CELLMARKERCUR_N_EPSILON_CELL																														
CELLMARKERCUR_N_ERYTHROBLAST																														
CELLMARKERCUR_N_ERYTHROID_CELL																														
CELLMARKERCUR_N_ERYTHROID_PROGENITOR_CELL																														
CELLMARKERCUR_N_ESOPHAGEAL_CELL																														
CELLMARKERCUR_N_ESOPHAGEAL_PROGENITOR_CELL																														
CELLMARKERCUR_N_EXCITATORY_NEURON																														
CELLMARKERCUR_N_EXHAUSTED_DOUBLE_POSITIVE…																														
CELLMARKERCUR_N_EXTRAVILLOUS_TROPHOBLAST																														
CELLMARKERCUR_N_F_PERICYTE																														
CELLMARKERCUR_N_FETAL_MYOBLAST																														
CELLMARKERCUR_N_FIBROBLAST_LIKE_CELL																														
CELLMARKERCUR_N_FIBROCHONDROCYTE																														
CELLMARKERCUR_N_FIBROCHONDROCYTE_PROGENIT…																														
CELLMARKERCUR_N_FOAM_CELL																														
CELLMARKERCUR_N_FOLLICULAR_B_CELL																														
CELLMARKERCUR_N_FOLLICULAR_CELL																														
CELLMARKERCUR_N_FOLLICULAR_DENDRITIC_CELL																														
CELLMARKERCUR_N_FOREBRAIN_CELL																														
CELLMARKERCUR_N_GABAERGIC_NEURON																														
CELLMARKERCUR_N_GANGLION_CELL																														
CELLMARKERCUR_N_GASTRIC_NEUROENDOCRINE_CE…																														
CELLMARKERCUR_N_GASTRIC_STEM_CELL																														
CELLMARKERCUR_N_GERM_CELL																														
CELLMARKERCUR_N_GERMINAL_CENTER_B_CELL																														
CELLMARKERCUR_N_GERMINAL_CENTER_CELL																														
CELLMARKERCUR_N_GERMINAL_CENTRE_B_CELL																														
CELLMARKERCUR_N_GLIAL_CELL																														
CELLMARKERCUR_N_GLIOMA_STEM_CELL																														
CELLMARKERCUR_N_GLOBOSE_BASAL_CELL																														
CELLMARKERCUR_N_GLOMERULAR_PARIETAL_EPITH…																														
CELLMARKERCUR_N_GLUTAMATERGIC_NEURON																														
CELLMARKERCUR_N_GOBLET_CELL																														
CELLMARKERCUR_N_GONADOTROPE																														
CELLMARKERCUR_N_GONADOTROPH_CELL																														
CELLMARKERCUR_N_GRANULOCYTE																														
CELLMARKERCUR_N_GRANULOCYTE_MONOCYTE_PROG…																														
CELLMARKERCUR_N_GRANULOCYTIC_UNK																														
CELLMARKERCUR_N_GRANULOSA_CELL																														
CELLMARKERCUR_N_HAEMATOPOIETIC_CELL																														
CELLMARKERCUR_N_HAIR_FOLLICLE_CELL																														
CELLMARKERCUR_N_HEMANGIOBLAST																														
CELLMARKERCUR_N_HEMATOPOIETIC_CELL																														
CELLMARKERCUR_N_HEMATOPOIETIC_PRECURSOR_C…																														
CELLMARKERCUR_N_HEMATOPOIETIC_PROGENITOR_…																														
CELLMARKERCUR_N_HEPATIC_CELL																														
CELLMARKERCUR_N_HEPATIC_PROGENITOR_CELL																														

CELLMARKERCAN_ACINAR_CELLS																														
CELLMARKERCUR_N_HEPATIC_STELLATE_CELL																														
CELLMARKERCUR_N_HEPATOBLAST																														
CELLMARKERCUR_N_HEPATOCYTE																														
CELLMARKERCUR_N_HISTIOCYTE																														
CELLMARKERCUR_N_HMSC_TREATED_B_CELL																														
CELLMARKERCUR_N_HOFBAUER_CELL																														
CELLMARKERCUR_N_HOMEOSTATIC_CHONDROCYTE																														
CELLMARKERCUR_N_HOMEOSTATIC_MICROGLIAL_CE…																														
CELLMARKERCUR_N_HORIZONTAL_BASAL_CELL																														
CELLMARKERCUR_N_HORIZONTAL_CELL																														
CELLMARKERCUR_N_HYPOBLAST_CELL																														
CELLMARKERCUR_N_IDIOPATHIC_PULMONARY_FIBR…																														
CELLMARKERCUR_N_IGA_MEMORY_B_CELL																														
CELLMARKERCUR_N_IGG_MEMORY_B_CELL																														
CELLMARKERCUR_N_IGM_MEMORY_B_CELL																														
CELLMARKERCUR_N_IGM_ONLY_B_CELL																														
CELLMARKERCUR_N_IL7R_T_HELPER_CELL																														
CELLMARKERCUR_N_IMMATURE_ERYTHROID_CELL																														
CELLMARKERCUR_N_IMMATURE_MYELOID_CELL																														
CELLMARKERCUR_N_IMMATURE_NEUTROPHIL																														
CELLMARKERCUR_N_IMMATURE_TRANSITIONAL_B_C…																														
CELLMARKERCUR_N_IMMUNE_CELL																														
CELLMARKERCUR_N_INDUCED_PLURIPOTENT_STEM_…																														
CELLMARKERCUR_N_INFILTRATING_MACROPHAGE																														
CELLMARKERCUR_N_INFLAMMATORY_CANCER_ASSOC…																														
CELLMARKERCUR_N_INFLAMMATORY_DENDRITIC_CE…																														
CELLMARKERCUR_N_INFLAMMATORY_MONOCYTE																														
CELLMARKERCUR_N_INHIBITORY_NEURON																														
CELLMARKERCUR_N_INNATE_LYMPHOID_CELL																														
CELLMARKERCUR_N_INNER_CELL_MASS_CELL																														
CELLMARKERCUR_N_INNER_RADIAL_GLIAL_CELL																														
CELLMARKERCUR_N_INTER_MEDIATE_PROGENITOR_…																														
CELLMARKERCUR_N_INTERCALATED_CELL																														
CELLMARKERCUR_N_INTERFERON_RESPONSIVE_SEC…																														
CELLMARKERCUR_N_INTERMEDIATE_MONOCYTE																														
CELLMARKERCUR_N_INTERMEDIATE_MONOCYTE_CELL																														
CELLMARKERCUR_N_INTERSTITIAL_MACROPHAGE																														
CELLMARKERCUR_N_INTERSTITIAL_PROGENITOR_C…																														
CELLMARKERCUR_N_INTESTINAL_EPITHELIAL_STE…																														
CELLMARKERCUR_N_INTESTINAL_GASTRIC_CANCER…																														
CELLMARKERCUR_N_INTESTINAL_METAPLASIA_CELL																														
CELLMARKERCUR_N_INTRAEPITHELIAL_LYMPHOCYT…																														
CELLMARKERCUR_N_IONOCYTE																														
CELLMARKERCUR_N_IRIS_PUTTATIVE_STEM_CELL																														
CELLMARKERCUR_N_KUPFFER_CELL																														
CELLMARKERCUR_N_LACTOTROPH_CELL																														
CELLMARKERCUR_N_LANGERHANS_CELL																														
CELLMARKERCUR_N_LEUKOCYTE																														
CELLMARKERCUR_N_LIMBAL_EPITHELIAL_STEM_CE…																														
CELLMARKERCUR_N_LIMBAL_STEM_CELL																														
CELLMARKERCUR_N_LIPOFIBROBLAST																														
CELLMARKERCUR_N_LIVER_PROGENITOR_CELL																														
CELLMARKERCUR_N_LIVER_STEM_CELL																														
CELLMARKERCUR_N_LMMLEYDIG_CELL																														
CELLMARKERCUR_N_LONOCYTE																														
CELLMARKERCUR_N_LOOP_OF_HENLE_CELL																														
CELLMARKERCUR_N_LOOP_PROGENITOR_CELL																														
CELLMARKERCUR_N_LOW_DENSITY_NEUTROPHIL																														
CELLMARKERCUR_N_LUMINAL_PROGENITOR_CELL																														
CELLMARKERCUR_N_LUNG_CELL																														
CELLMARKERCUR_N_LYMPH_NODE_CELL																														
CELLMARKERCUR_N_LYMPHATIC_CELL																														
CELLMARKERCUR_N_LYMPHATIC_ENDOTHELIAL_CELL																														
CELLMARKERCUR_N_LYMPHOBLASTOID_CELL																														
CELLMARKERCUR_N_LYMPHOCYTE																														
CELLMARKERCUR_N_LYMPHOID_CELL																														
CELLMARKERCUR_N_LYMPHOID_PROGENITOR_CELL																														
CELLMARKERCUR_N_LYMPHOID_STRUCTURE_CELL																														
CELLMARKERCUR_N_M_PERICYTE																														
CELLMARKERCUR_N_M1_MACROPHAGE																														
CELLMARKERCUR_N_M1_MICROGLIAL_CELL																														
CELLMARKERCUR_N_M2_MACROPHAGE																														
CELLMARKERCUR_N_MACROGLIAL_CELL																														
CELLMARKERCUR_N_MALIGNANT_CELL																														
CELLMARKERCUR_N_MALIGNANT_PLASMA_CELL																														
CELLMARKERCUR_N_MARGINAL_ASSOCIATED_MACRO…																														
CELLMARKERCUR_N_MAST_CELL																														
CELLMARKERCUR_N_MATURE_ASTROCYTE																														
CELLMARKERCUR_N_MATURE_B_CELL																														
CELLMARKERCUR_N_MATURE_DENDRITIC_CELL																														
CELLMARKERCUR_N_MATURE_ENDOTHELIAL_CELL																														
CELLMARKERCUR_N_MATURE_LUMINAL_CELL																														
CELLMARKERCUR_N_MATURE_NEURON																														
CELLMARKERCUR_N_MATURE_NEURONAL																														
CELLMARKERCUR_N_MATURE_OLIGODENDROCYTE																														
CELLMARKERCUR_N_MATURE_OSTEOBLAST																														
CELLMARKERCUR_N_MATURE_PODOCYTE																														
CELLMARKERCUR_N_MEDIAL_AND_DISTAL_SEGMENT…																														
CELLMARKERCUR_N_MEDIAL_GANGLIONIC_EMINENC…																														
CELLMARKERCUR_N_MEGAKARYOCYTE																														
CELLMARKERCUR_N_MEGAKARYOCYTE_ERYTHROID_C…																														
CELLMARKERCUR_N_MEGAKARYOCYTE_ERYTHROID_P…																														
CELLMARKERCUR_N_MEGAKARYOCYTE_PROGENITOR_…																														
CELLMARKERCUR_N_MELANOMA_CELL																														
CELLMARKERCUR_N_MEMORY_B_CELL																														
CELLMARKERCUR_N_MEMORY_B_CELL_PRECURSOR																														
CELLMARKERCUR_N_MEMORY_CD27_B_CELL																														
CELLMARKERCUR_N_MEMORY_CD4_T_CELL																														
CELLMARKERCUR_N_MEMORY_DOUBLE_POSITIVE_T_…																														
CELLMARKERCUR_N_MEMORY_REGULATORY_T_CELL																														
CELLMARKERCUR_N_MEMORY_T_CELL																														
CELLMARKERCUR_N_MERKEL_CELL																														
CELLMARKERCUR_N_MESENCHYMAL_CELL																														
CELLMARKERCUR_N_MESENCHYMAL_EPITHELIAL_TR…																														

Table S5: Cell state marker gene list

CELLMARKERCAN_ACINAR_CELLS																														
CELLMARKERCUR_N_MESENCHYMAL_GLIOBLASTOMA_…																														
CELLMARKERCUR_N_MESENCHYMAL_STEM_CELL																														
CELLMARKERCUR_N_MESENCHYMAL_STROMAL_CELL																														
CELLMARKERCUR_N_MESENDODERMAL_CELL																														
CELLMARKERCUR_N_MESODERMAL_EMBRYONIC_STEM…																														
CELLMARKERCUR_N_MESODERMAL_PROGENITOR_CELL																														
CELLMARKERCUR_N_METAPLASTIC_STEM_CELL																														
CELLMARKERCUR_N_MEYLOID_CELL																														
CELLMARKERCUR_N_MICROGLIAL_CELL	LGI2	HPSE	RFX2	CD63	CTSE	PLEKH…	GPNMB	CD300…	SELEN…	RPL7	NPC2	PSAT1	APOC4	RPL12	MAFF	CADM1	FOXP1	SCD5	DPP7	ANXA3	MIF	LILRB4	COX6A1	ALDOA	VENTX	CLDN15	ULK3	CXCL16	NEXN	TNFRS…
CELLMARKERCUR_N_MICROVILLAR_CELL																														
CELLMARKERCUR_N_MIDBRAIN_DOPAMINERGIC_NEU…																														
CELLMARKERCUR_N_MONO_MACROPHAGE																														
CELLMARKERCUR_N_MONOCYTE_DERIVED_CELL																														
CELLMARKERCUR_N_MONOCYTE_DERIVED_MYELOID_…																														
CELLMARKERCUR_N_MONOCYTE_LINEAGE																														
CELLMARKERCUR_N_MONOCYTE_PRECURSOR																														
CELLMARKERCUR_N_MONOCYTE_PROGENITOR_CELL																														
CELLMARKERCUR_N_MUELLER_GLIA_CELL																														
CELLMARKERCUR_N_MULTIPOTENT_MESENCHYMAL_S…																														
CELLMARKERCUR_N_MULTIPOTENT_RETINAL_PROGE…																														
CELLMARKERCUR_N_MULTIPOTENT_STEM_CELL																														
CELLMARKERCUR_N_MULTIPOTENT_STROMAL_CELL																														
CELLMARKERCUR_N_MUSCLE_CELL																														
CELLMARKERCUR_N_MUSCLE_DERIVED_CELL																														
CELLMARKERCUR_N_MUSCLE_SATELLITE_CELL																														
CELLMARKERCUR_N_MYELOBLAST																														
CELLMARKERCUR_N_MYELOID_DENDRITIC_CELL																														
CELLMARKERCUR_N_MYELOID_DENDRITIC_CELL_1																														
CELLMARKERCUR_N_MYELOID_DERIVED_CELL																														
CELLMARKERCUR_N_MYELOID_DERIVED_SUPPRESSO…																														
CELLMARKERCUR_N_MYELOID_PROGENITOR_CELL																														
CELLMARKERCUR_N_MYO_EPITHELIAL_CELL																														
CELLMARKERCUR_N_MYOBLAST																														
CELLMARKERCUR_N_MYOCYTE																														
CELLMARKERCUR_N_MYOFIBROBLAST																														
CELLMARKERCUR_N_MYOGENIC_ENDOTHELIAL_CELL																														
CELLMARKERCUR_N_MYOGENIC_PROGENITOR_CELL																														
CELLMARKERCUR_N_MYOID_CELL																														
CELLMARKERCUR_N_NAIVE_B_CELL																														
CELLMARKERCUR_N_NAIVE_CD8_T_CELL																														
CELLMARKERCUR_N_NAIVE_DOUBLE_POSITIVE_T_C…																														
CELLMARKERCUR_N_NAIVE_PROGENITOR_CELL																														
CELLMARKERCUR_N_NAIVE_T_CELL																														
CELLMARKERCUR_N_NASCENT_NEPHRON_CELL																														
CELLMARKERCUR_N_NATURAL_KILLER_CD56_BRIGH…																														
CELLMARKERCUR_N_NATURAL_KILLER_CD56_DIM_C…																														
CELLMARKERCUR_N_NEGATIVE_MESENCHYMAL_STEM…																														
CELLMARKERCUR_N_NEURAL_CELL																														
CELLMARKERCUR_N_NEURAL_CREST_STEM_CELL																														
CELLMARKERCUR_N_NEURAL_CREST_STEMLIKE_CELL																														
CELLMARKERCUR_N_NEURAL_PRECURSOR_CELL																														
CELLMARKERCUR_N_NEURAL_STEM_CELL																														
CELLMARKERCUR_N_NEUROECTODERM_CELL																														
CELLMARKERCUR_N_NEURONAL_INTERMEDIATE_PRO…																														
CELLMARKERCUR_N_NEURONAL_PROGENITOR_CELL																														
CELLMARKERCUR_N_NEUTROPHIL_PROGENITOR_CELL																														
CELLMARKERCUR_N_NON_NEURON																														
CELLMARKERCUR_N_NON_PT_TUBULAR_CELL																														
CELLMARKERCUR_N_NON_RECIRCULATING_TISSUE_…																														
CELLMARKERCUR_N_NONMYELINATING_SCHWANN_CE…																														
CELLMARKERCUR_N_NOTOCHORD_CELL																														
CELLMARKERCUR_N_NUCLEATED_CELL																														
CELLMARKERCUR_N_NUCLEUS_PULPOSUS_PROGENIT…																														
CELLMARKERCUR_N_OLIGODENDROCYTE																														
CELLMARKERCUR_N_OSTEOBLAST																														
CELLMARKERCUR_N_OUTER_RADIAL_GLIA																														
CELLMARKERCUR_N_OVALBUMIN_SPECIFIC_REGULA…																														
CELLMARKERCUR_N_OVARIAN_CELL																														
CELLMARKERCUR_N_OVARIAN_STROMAL_CELL																														
CELLMARKERCUR_N_P53_LIKE_CELL																														
CELLMARKERCUR_N_PAN_B_CELL																														
CELLMARKERCUR_N_PAN_CANCER_ASSOCIATED_FIB…																														
CELLMARKERCUR_N_PAN_MACROPHAGE																														
CELLMARKERCUR_N_PAN_MONOCYTE																														
CELLMARKERCUR_N_PAN_NEURONAL_CELL																														
CELLMARKERCUR_N_PAN_TROPHECTODERM_CELL																														
CELLMARKERCUR_N_PANCREATIC_ENDODERM_CELL																														
CELLMARKERCUR_N_PANCREATIC_POLYPEPTIDE_CE…																														
CELLMARKERCUR_N_PANCREATIC_PROGENITOR_CELL																														
CELLMARKERCUR_N_PANCREATIC_STEM_CELL																														
CELLMARKERCUR_N_PANETH_CELL																														
CELLMARKERCUR_N_PANNEURON																														
CELLMARKERCUR_N_PANVASCULAR_CELL																														
CELLMARKERCUR_N_PAPILLARY_FIBROBLAST																														
CELLMARKERCUR_N_PARIETAL_CELL																														
CELLMARKERCUR_N_PARVALBUMIN_INTERNEURON																														
CELLMARKERCUR_N_PATELET																														
CELLMARKERCUR_N_PATHOGENIC_T_PERIPHERAL_H…																														
CELLMARKERCUR_N_PELVIC_EPITHELIAL_CELL																														
CELLMARKERCUR_N_PERICYTE																														
CELLMARKERCUR_N_PERIODONTAL_LIGAMENT_STEM…																														
CELLMARKERCUR_N_PERIOSTEUM_DERIVED_PROGEN…																														
CELLMARKERCUR_N_PERITUBULAR_MYOID_CELL																														
CELLMARKERCUR_N_PERIVASCULAR_LIKE_CELL																														
CELLMARKERCUR_N_PHAGOCYTE																														
CELLMARKERCUR_N_PHOTORECEPTOR_CELL																														
CELLMARKERCUR_N_PIT_CELL																														
CELLMARKERCUR_N_PIT_MUCOUS_CELL																														
CELLMARKERCUR_N_PITUITARY_STEM_CELL																														
CELLMARKERCUR_N_PLACENTAL_TISSUE_CELL																														
CELLMARKERCUR_N_PLACODE_CELL																														
CELLMARKERCUR_N_PLASMABLAST_B_CELL																														
CELLMARKERCUR_N_PLATELET																														
CELLMARKERCUR_N_PLURIPOTENT_STEM_CELL																														

Table S5: Cell state marker gene list

CELLMARKERCAN_ACINAR_CELLS																														
CELLMARKERCUR_N_PODOCYTE_PRECURSOR																														
CELLMARKERCUR_N_PORTAL_FIBROBLAST																														
CELLMARKERCUR_N_POSTERIOR_CELL																														
CELLMARKERCUR_N_PRE_B_CELL																														
CELLMARKERCUR_N_PRE_CONVENTIONAL_DENDRITI…																														
CELLMARKERCUR_N_PRE_DENDRITIC_CELL																														
CELLMARKERCUR_N_PRECURSOR_CELL																														
CELLMARKERCUR_N_PREHYPERTROPHIC_CHONDROCY…																														
CELLMARKERCUR_N_PRESOMITE_CELL																														
CELLMARKERCUR_N_PRETUBULAR_AGGREGATE_CELL																														
CELLMARKERCUR_N_PRIMITIVE_ENDODERM_CELL																														
CELLMARKERCUR_N_PRINCIPAL_CELL																														
CELLMARKERCUR_N_PRO_B_CELL																														
CELLMARKERCUR_N_PRO_INFLAMMATORY_MACROPHA…																														
CELLMARKERCUR_N_PRO_NEUTROPHIL																														
CELLMARKERCUR_N_PROLIFERATING_CELL																														
CELLMARKERCUR_N_PROLIFERATIVE_CHONDROCYTE																														
CELLMARKERCUR_N_PROLIFERATIVE_DUCTAL_CELL																														
CELLMARKERCUR_N_PROLIFERATIVE_NEURAL_PROG…																														
CELLMARKERCUR_N_PRONEURAL_GLIOBLASTOMA_CE…																														
CELLMARKERCUR_N_PROSPERMATOGONIA																														
CELLMARKERCUR_N_PROSTATE_CELL																														
CELLMARKERCUR_N_PROXIMAL_CELL																														
CELLMARKERCUR_N_PROXIMAL_SEGMENT_CELL																														
CELLMARKERCUR_N_PROXIMAL_STEM_CELL																														
CELLMARKERCUR_N_PROXIMAL_TUBULE_CELL																														
CELLMARKERCUR_N_PULMONARY_NEUROENDOCRINE_…																														
CELLMARKERCUR_N_PYRAMIDAL_CELL																														
CELLMARKERCUR_N_QUIESCENT_T_STEM_CELL																														
CELLMARKERCUR_N_REGULATORY_B_CELL																														
CELLMARKERCUR_N_REGULATORY_INNATE_LYMPHOI…																														
CELLMARKERCUR_N_RENAL_TUBULAR_CELL																														
CELLMARKERCUR_N_RESIDENT_MEMORY_T_CELL																														
CELLMARKERCUR_N_RESPIRATORY_HORIZONTAL_BA…																														
CELLMARKERCUR_N_RESPIRATORY_SECRETORY_CELL																														
CELLMARKERCUR_N_RESPONDER_T_CELL																														
CELLMARKERCUR_N_RESPONDING_CONVENTIONAL_T…																														
CELLMARKERCUR_N_RESTING_MEMORY_B_CELL																														
CELLMARKERCUR_N_RESTING_NAIVE_B_CELL																														
CELLMARKERCUR_N_RESTING_REGULATORY_T_CELL																														
CELLMARKERCUR_N_RETICULAR_FIBROBLAST																														
CELLMARKERCUR_N_RETINA_CELL																														
CELLMARKERCUR_N_ROD_CELL																														
CELLMARKERCUR_N_SATELLITE_CELL																														
CELLMARKERCUR_N_SEPTUM_TRANSVERSUM_MESENC…																														
CELLMARKERCUR_N_SEROUS_CELL																														
CELLMARKERCUR_N_SIDE_POPULATION_CELL																														
CELLMARKERCUR_N_SKELETAL_MUSCLE_CELL																														
CELLMARKERCUR_N_SKELETAL_MUSCLE_STEM_CELL																														
CELLMARKERCUR_N_SMOOTH_MUSCLE_CELL																														
CELLMARKERCUR_N_SMOOTH_MUSCLE_LIKE_CELL																														
CELLMARKERCUR_N_SOMATIC_CELL																														
CELLMARKERCUR_N_SOMATIC_STEM_CELL																														
CELLMARKERCUR_N_SOMATOSTATIN_INTERNEURON																														
CELLMARKERCUR_N_SPECIALIST_ANTIGEN_PRESEN…																														
CELLMARKERCUR_N_SPERMATOGENIC_CELL																														
CELLMARKERCUR_N_SPERMATOGONIAL_CELL																														
CELLMARKERCUR_N_SPERMATOGONIAL_STEM_CELL																														
CELLMARKERCUR_N_SQUAMOUS_DIFFERENTIATION_…																														
CELLMARKERCUR_N_STELLATE_CELL																														
CELLMARKERCUR_N_STEM_CELL_DERIVED_ALPHA_C…																														
CELLMARKERCUR_N_STEM_CELL_DERIVED_BETA_CE…																														
CELLMARKERCUR_N_STEM_CELL_DERIVED_ENDOTHE…																														
CELLMARKERCUR_N_STEM_LIKE_CD8_T_CELL																														
CELLMARKERCUR_N_STROMAL_CELL																														
CELLMARKERCUR_N_SUBPALLIUM_CELL																														
CELLMARKERCUR_N_SUBPLATE_CELL																														
CELLMARKERCUR_N_SUPPRESSIVE_MONOCYTE																														
CELLMARKERCUR_N_SUPPRESSOR_T_CELL																														
CELLMARKERCUR_N_SUPRABASAL_CELL																														
CELLMARKERCUR_N_SURFACE_ECTODERM_CELL																														
CELLMARKERCUR_N_SUSTENTACULAR_CELL																														
CELLMARKERCUR_N_SYMPATHOADRENAL_CELL																														
CELLMARKERCUR_N_SYNCYTIOTROPHOBLAST																														
CELLMARKERCUR_N_SYNTHETIC_SMOOTH_MUSCLE_C…																														
CELLMARKERCUR_N_T_CELL_LARGE_GRANULAR_LYM…																														
CELLMARKERCUR_N_T_CELL_LINEAGE																														
CELLMARKERCUR_N_T_HELPER_CELL																														
CELLMARKERCUR_N_T_MEMORY_STEM_CELL																														
CELLMARKERCUR_N_T2_MZP_REGULATORY_B_CELL																														
CELLMARKERCUR_N_TENDON_CELL																														
CELLMARKERCUR_N_TERMINAL_DIFFERENTIATED_B…																														
CELLMARKERCUR_N_THALAMIC_CELL																														
CELLMARKERCUR_N_THECA_INTERNA_CELL																														
CELLMARKERCUR_N_THYMIC_EPITHELIAL_CELL																														
CELLMARKERCUR_N_TISSUE_LIKE_MEMORY_B_CELL																														
CELLMARKERCUR_N_TISSUE_RESIDENT_CELL																														
CELLMARKERCUR_N_TISSUE_RESIDENT_EFFECTOR_…																														
CELLMARKERCUR_N_TISSUE_RESIDENT_MACROPHAGE																														
CELLMARKERCUR_N_TISSUE_RESIDENT_MEMORY_LI…																														
CELLMARKERCUR_N_TOLDC_INDUCED_REGULATORY_…																														
CELLMARKERCUR_N_TONSIL_CELL																														
CELLMARKERCUR_N_TRABECULAR_MESHWORK_CELL																														
CELLMARKERCUR_N_TRANSITIONAL_B_CELL																														
CELLMARKERCUR_N_TRANSITIONAL_DENDRITIC_CE…																														
CELLMARKERCUR_N_TRUNCATED_RADIAL_GLIAL_CE…																														
CELLMARKERCUR_N_TUBULAR_CELL																														
CELLMARKERCUR_N_TUMOR_ASSOCIATED_MICROGLI…																														
CELLMARKERCUR_N_TUMOR_ENDOTHELIAL_CELL																														
CELLMARKERCUR_N_TUMOR_EPITHELIAL_CELL																														
CELLMARKERCUR_N_TUMOR_INFILTRATING_REGULA…																														
CELLMARKERCUR_N_TUMOR_INFILTRATING_T_CELL																														
CELLMARKERCUR_N_TUMOR_INITIATING_CELL																														
CELLMARKERCUR_N_TUMOR_REGULATORY_T_CELL																														

Table S5: Cell state marker gene list

CELLMARKERCAN_ACINAR_CELLS																														
CELLMARKERCUR_N_UMBRELLA_CELL																														
CELLMARKERCUR_N_UNRESTRICTED_SOMATIC_STEM…																														
CELLMARKERCUR_N_UNSWITCHED_PLASMABLAST																														
CELLMARKERCUR_N_URETERIC_BUD_CELL																														
CELLMARKERCUR_N_UROTHELIAL_CELL																														
CELLMARKERCUR_N_UROTHELIUM_CELL																														
CELLMARKERCUR_N_VASCULATURE_CELL																														
CELLMARKERCUR_N_VEIN_CELL																														
CELLMARKERCUR_N_VENOUS_CELL																														
CELLMARKERCUR_N_VENTRAL_LATERAL_GANGLIONI…																														
CELLMARKERCUR_N_VENTRAL_MEDIAL_GANGLIONIC…																														
CELLMARKERCUR_N_VENTRICULAR_CELL																														
CELLMARKERCUR_N_YOUNG_NEURON																														
CELLMARKERCUR_N_ZONA_FASCICULATA_CELL																														
CELLMARKERCUR_N_ZONA_GLOMERULOSA_CELL																														
CELLMARKERCUR_N_ZONA_RETICULARIS_CELL																														
CELLMARKERCUR_N_ZYGOTE																														
CELLMARKERCURNACTIVATEDEFFECTORMEMORYCD4T…																														
CELLMARKERCURNACTIVATEDTISSUERESIDENTMEMO…																														
CELLMARKERCURNACTIVATEDTISSUERESIDENTMEMO…																														
CELLMARKERCURNALPHACELLACELL																														
CELLMARKERCURNALVEOLARTYPEIIATIICELL																														
CELLMARKERCURNATYPICALBCELLABC																														
CELLMARKERCURNAXLCD11CPLASMACYTOIDDENDRIT…																														
CELLMARKERCURNAXLCD123PLASMACYTOIDDENDRIT…																														
CELLMARKERCURNBASALFOREBRAINCHOLINERGICNE…																														
CELLMARKERCURNBRUSHCELLTUFTCELL																														
CELLMARKERCURNCANCERASSOCIATEDFIBROBLASTC…																														
CELLMARKERCURNCAPSULARFIBROBLASTFB2																														
CELLMARKERCURNCCR7DENDRITICCELL																														
CELLMARKERCURNCD106MESENCHYMALSTEMCELL																														
CELLMARKERCURNCD14MACROPHAGE																														
CELLMARKERCURNCD16DENDRITICCELL																														
CELLMARKERCURNCD25LAG3TCELL																														
CELLMARKERCURNCD34GRANULOCYTICPROGENITORC…																														
CELLMARKERCURNCD34HEMATOPOIETICSTEMCELL																														
CELLMARKERCURNCD34PREBCELL																														
CELLMARKERCURNCD34PROGENITORCELL																														
CELLMARKERCURNCD34THYMOCYTE																														
CELLMARKERCURNCD40LGTHELPERCELL																														
CELLMARKERCURNCD4CD25REGULATORYTCELL																														
CELLMARKERCURNCD4CENTRALMEMORYLIKETTCMLIK…																														
CELLMARKERCURNCD4CYTOTOXICT1CELL																														
CELLMARKERCURNCD4CYTOTOXICT2CELL																														
CELLMARKERCURNCD4THELPERCELL																														
CELLMARKERCURNCHRONICALLYACTIVATEDCD4TCELL																														
CELLMARKERCURNCHRONICALLYACTIVATEDCD8TCELL																														
CELLMARKERCURNCLASSSWITCHEDMEMORYBIGGU03B…																														
CELLMARKERCURNCLEC10ADENDRITICCELL																														
CELLMARKERCURNCLEC9ADENDRITICCELL																														
CELLMARKERCURNCONVENTIONALDENDRITICCELL2B…																														
CELLMARKERCURNCONVENTIONALDENDRITICCELL2B…																														
CELLMARKERCURNCORTICALSOMATOSTATINSSTINTE…																														
CELLMARKERCURNCYTOTOXICCD4T2CELL																														
CELLMARKERCURNCYTOTOXICCD4TCELL																														
CELLMARKERCURND1MSNMEDIUMSPINYNEURON																														
CELLMARKERCURND2MSNMEDIUMSPINYNEURON																														
CELLMARKERCURNDISTALTUBULELOOPOFHENLECELL																														
CELLMARKERCURNEARLYCD34MEGAKARYOCYTEERYTH…																														
CELLMARKERCURNEARLYTCELLPROGENITORETP																														
CELLMARKERCURNEFFECTORMEMORYCD4TCELL																														
CELLMARKERCURNEFFECTORREGULATORYTETREGCELL																														
CELLMARKERCURNEFFECTORTTEFFCELL																														
CELLMARKERCURNEXHAUSTEDCD4TCELL																														
CELLMARKERCURNEXHAUSTEDCD8TCELL																														
CELLMARKERCURNEXHAUSTEDCD8TPROGENITORCELL																														
CELLMARKERCURNEXHAUSTEDTTEXCELL																														
CELLMARKERCURNEXTRATELENCEPHALICETCELL																														
CELLMARKERCURNFINALLYHIGHLYEFFECTORTEMRAM…																														
CELLMARKERCURNFOLLICULARREGULATORYTTFRCELL																														
CELLMARKERCURNFOXP3NATURALREGULATORYTTREG…																														
CELLMARKERCURNGAMMADELTAU03B3DTCELL																														
CELLMARKERCURNGERMINALCENTREGCLIKECELL																														
CELLMARKERCURNGLOMERULARENDOTHELIALCELLECG																														
CELLMARKERCURNGRANULOCYTICMYELOIDDERIVEDS…																														
CELLMARKERCURNGRBREGULATORYBCELL																														
CELLMARKERCURNGZMKTCELL																														
CELLMARKERCURNHIGHLYDIFFERENTIATEDEFFECTO…																														
CELLMARKERCURNINDUCEDREGULATORYTTREGCELL																														
CELLMARKERCURNINFLAMMATORYCANCERASSOCIATE…																														
CELLMARKERCURNINNATELYMPHOIDCELLILCS																														
CELLMARKERCURNINTRATHYMICFIBROBLASTFB1																														
CELLMARKERCURNITOCELLHEPATICSTELLATECELL																														
CELLMARKERCURNLYMPHANGIOLEIOMYOMATOSISCEL…																														
CELLMARKERCURNMARGINALZONEMZLIKEBCELL																														
CELLMARKERCURNMEMORYSTEMCELLTSCM																														
CELLMARKERCURNMICROGLIADERIVEDTUMORASSOCI…																														
CELLMARKERCURNMKERYTHCELL																														
CELLMARKERCURNMYOFIBROBLASTICCANCERASSOCI…																														
CELLMARKERCURNNAIVEREGULATORYTTREGCELL																														
CELLMARKERCURNNATURALREGULATORYTTREGCELL																														
CELLMARKERCURNPERIPHERALBLOODMONONUCLEARC…																														
CELLMARKERCURNPITUITARYSTEMCELLPSC																														
CELLMARKERCURNPLASMACYTOIDDENDRITICCELLPDC																														
CELLMARKERCURNPOLYMORPHONUCLEARMYELOIDDER…																														
CELLMARKERCURNPOLYMORPHONUCLEARNEUTROPHIL…																														
CELLMARKERCURNPOSTERIORPRESOMITICMESODERM…																														
CELLMARKERCURNPROLIFERATIVECD4TCELL																														
CELLMARKERCURNPROLIFERATIVECD8TCELL																														
CELLMARKERCURNREDBLOODCELLERYTHROCYTE																														
CELLMARKERCURNREGULATORYBBREGCELL																														
CELLMARKERCURNREGULATORYCD4TCELL																														
CELLMARKERCURNREGULATORYCD8TCELL																														
CELLMARKERCURNREGULATORYTTREGCELL																														

CELLMARKERCAN_ACINAR_CELLS																														
CELLMARKERCURNREGULATORYTTREGCELL																														
CELLMARKERCURNRENALVESICLECOMMASHAPEDBODY…																														
CELLMARKERCURNRESIDENTMEMORYCD8TCELL																														
CELLMARKERCURNREVERTANTMEMORYCD8TTEMRACELL																														
CELLMARKERCURNSCARASSOCIATEDMACROPHAGESAM																														
CELLMARKERCURNSENESCENTCD8TCELL																														
CELLMARKERCURNSTEMPROGENITORCELL																														
CELLMARKERCURNSWITCHEDMEMORYBCELLSWMB																														
CELLMARKERCURNT1TRANSITIONALBCELL																														
CELLMARKERCURNT2TRANSITIONALBCELL																														
CELLMARKERCURNTCELLNATURALKILLERCELL																														
CELLMARKERCURNTERMINALEFFECTORCD8TCELL																														
CELLMARKERCURNTERMINALLYDIFFERENTIATEDCD8…																														
CELLMARKERCURNTFOLLICULARHELPERTFHCELL																														
CELLMARKERCURNTHELPER1TH1CELL																														
CELLMARKERCURNTHELPER1TH1CELL																														
CELLMARKERCURNTHELPER2TH2CELL																														
CELLMARKERCURNTHELPER2TH2CELL																														
CELLMARKERCURNTHELPER9TH9CELL																														
CELLMARKERCURNTHELPERTHCELL																														
CELLMARKERCURNTHYMICEPITHELIALCELLTEC																														
CELLMARKERCURNTISSUERESIDENTMEMORYCD4TCELL																														
CELLMARKERCURNTISSUERESIDENTMEMORYCD8TCELL																														
CELLMARKERCURNTISSUERESIDENTMEMORYTTRMCELL																														
CELLMARKERCURNTPERIPHERALHELPERTPHCELL																														
CELLMARKERCURNTRANSIENTAMPLIFYINGTACELL																														
CELLMARKERCURNTRANSITIONALEXHAUSTEDCD8TCE…																														
CELLMARKERCURNTUMORINFILTRATINGLYMPHOCYTE…																														
CELLMARKERCURNUNSWITCHEDMEMORYBCELLUNSWMB																														
CELLMARKERCURNVASARECTAENDOTHELIALCELLECVR																														
CELLMARKERCURNVONECONOMONEURONVEN																														
CELLMARKERCURNYOLKSACDERIVEDMYELOIDBIASED…																														
CELLMARKEREXP_ACINAR_CELLS																														
CELLMARKEREXP_ADRENERGIC_NEURONS																														
CELLMARKEREXP_AIRWAY_EPITHELIAL_CELLS																														
CELLMARKEREXP_AIRWAY_SMOOTH_MUSCLE_CELLS																														
CELLMARKEREXP_ALPHA_CELLS																														
CELLMARKEREXP_ANTERIOR_PITUITARY_GLAND_CE…																														
CELLMARKEREXP_ASTROCYTES																														
CELLMARKEREXP_B_CELLS																														
CELLMARKEREXP_B_CELLS_MEMORY																														
CELLMARKEREXP_B_CELLS_NAIVE																														
CELLMARKEREXP_BASAL_CELLS																														
CELLMARKEREXP_BASOPHILS																														
CELLMARKEREXP_BERGMANN_GLIA																														
CELLMARKEREXP_BETA_CELLS																														
CELLMARKEREXP_CAJAL_RETZIUS_CELLS																														
CELLMARKEREXP_CHOLANGIOCYTES																														
CELLMARKEREXP_CHOLINERGIC_NEURONS																														
CELLMARKEREXP_CHONDROCYTES																														
CELLMARKEREXP_DELTA_CELLS																														
CELLMARKEREXP_DISTAL_TUBULE_CELLS																														
CELLMARKEREXP_DUCTAL_CELLS																														
CELLMARKEREXP_EMBRYONIC_STEM_CELLS																														
CELLMARKEREXP_ENTERIC_NEURONS																														
CELLMARKEREXP_ENTEROENDOCRINE_CELLS																														
CELLMARKEREXP_EPENDYMAL_CELLS																														
CELLMARKEREXP_EPSILON_CELLS																														
CELLMARKEREXP_ERYTHROID_LIKE_AND_ERYTHROI…																														
CELLMARKEREXP_GAMMA_DELTA_T_CELLS																														
CELLMARKEREXP_GOBLET_CELLS																														
CELLMARKEREXP_HEMANGIOBLASTS																														
CELLMARKEREXP_HEMATOPOIETIC_STEM_CELLS																														
CELLMARKEREXP_HEPATIC_STELLATE_CELLS																														
CELLMARKEREXP_HIS_BUNDLE_CELLS																														
CELLMARKEREXP_IMMATURE_NEURONS																														
CELLMARKEREXP_JUXTAGLOMERULAR_CELLS																														
CELLMARKEREXP_KERATINOCYTES																														
CELLMARKEREXP_KIDNEY_PROGENITOR_CELLS																														
CELLMARKEREXP_KUPFFER_CELLS																														
CELLMARKEREXP_LEYDIG_CELLS																														
CELLMARKEREXP_LOOP_OF_HENLE_CELLS																														
CELLMARKEREXP_LUMINAL_EPITHELIAL_CELLS																														
CELLMARKEREXP_MAMMARY_EPITHELIAL_CELLS																														
CELLMARKEREXP_MEGAKARYOCYTES																														
CELLMARKEREXP_MELANOCYTES																														
CELLMARKEREXP_MESANGIAL_CELLS																														
CELLMARKEREXP_MESOTHELIAL_CELLS																														
CELLMARKEREXP_MICROFOLD_CELLS																														
CELLMARKEREXP_MICROGLIA																														
CELLMARKEREXP_MONOCYTES																														
CELLMARKEREXP_MULLER_CELLS																														
CELLMARKEREXP_MYOBLASTS																														
CELLMARKEREXP_MYOCYTES																														
CELLMARKEREXP_MYOFIBROBLASTS																														
CELLMARKEREXP_NEUROBLASTS																														
CELLMARKEREXP_NEUTROPHILS																														
CELLMARKEREXP_NK_CELLS																														
CELLMARKEREXP_NORADRENERGIC_NEURONS																														
CELLMARKEREXP_OLIGODENDROCYTES																														
CELLMARKEREXP_OSTEOBLASTS																														
CELLMARKEREXP_OSTEOCLAST_PRECURSOR_CELLS																														
CELLMARKEREXP_OSTEOCLASTS																														
CELLMARKEREXP_OSTEOCYTES																														
CELLMARKEREXP_PANETH_CELLS																														
CELLMARKEREXP_PARATHYROID_CHIEF_CELLS																														
CELLMARKEREXP_PERICYTES																														
CELLMARKEREXP_PHOTORECEPTOR_CELLS																														
CELLMARKEREXP_PLASMA_CELLS																														
CELLMARKEREXP_PLASMACYTOID_DENDRITIC_CELLS																														
CELLMARKEREXP_PODOCYTES																														
CELLMARKEREXP_PRINCIPAL_CELLS																														
CELLMARKEREXP_PROXIMAL_TUBULE_CELLS																														
CELLMARKEREXP_PULMONARY_ALVEOLAR_TYPE_I_C…																														

CELLMARKERCAN_ACINAR_CELLS																														
CELLMARKEREXP_PULMONARY_ALVEOLAR_TYPE_II_…																														
CELLMARKEREXP_PURKINJE_FIBER_CELLS																														
CELLMARKEREXP_PURKINJE_NEURONS																														
CELLMARKEREXP_PYRAMIDAL_CELLS																														
CELLMARKEREXP_RETICULOCYTES																														
CELLMARKEREXP_RETINAL_GANGLION_CELLS																														
CELLMARKEREXP_SALIVARY_MUCOUS_CELLS																														
CELLMARKEREXP_SATELLITE_CELLS																														
CELLMARKEREXP_SCHWANN_CELLS																														
CELLMARKEREXP_SEROTONERGIC_NEURONS																														
CELLMARKEREXP_SERTOLI_CELLS																														
CELLMARKEREXP_SMOOTH_MUSCLE_CELLS																														
CELLMARKEREXP_SPERMATOZOA																														
CELLMARKEREXP_STROMAL_CELLS																														
CELLMARKEREXP_T_CELLS																														
CELLMARKEREXP_T_CELLS_NAIVE																														
CELLMARKEREXP_T_CYTOTOXIC_CELLS																														
CELLMARKEREXP_T_HELPER_CELLS																														
CELLMARKEREXP_T_MEMORY_CELLS																														
CELLMARKEREXP_TRICHOCYTES																														
CELLMARKEREXP_TRIGEMINAL_NEURONS																														
CELLMARKEREXP_TROPHOBLAST_CELLS																														
CELLMARKEREXP_TROPHOBLAST_PROGENITOR_CELLS																														
CELLMARKEREXP_TUFT_CELLS																														
CELLMARKEREXP_VASCULAR_SMOOTH_MUSCLE_CELLS																														
CELLMARKEREXPENDOTHELIALCELLSAORTA																														
CELLMARKEREXPNEURALSTEMPRECURSORCELLS																														
CELLMARKERMSIGDB_AIZARANI_LIVER_C12_NK_NK…																														
CELLMARKERMSIGDB_AIZARANI_LIVER_C22_RESID…																														
CELLMARKERMSIGDB_AIZARANI_LIVER_C28_NK_NK…																														
CELLMARKERMSIGDB_AIZARANI_LIVER_C29_MVECS…																														
CELLMARKERMSIGDB_AIZARANI_LIVER_C30_HEPAT…	RANBP…	PHKA2	APMAP	RN7SL…	RSPH3	FBXO22	MFAP5	STAT3	FMN1	MDM2	PLIN4	KIR3D…	PEDS1	LYRM7	C15or…	TNRC6A	GLYCTK	STXBP…	FEM1A	TMEM2…	ELL2	UBN2	AP1M1	ADGRG6	TAF8	RN7SL…	RNF115	BCL6	GTF2H2	ULK1
CELLMARKERMSIGDB_AIZARANI_LIVER_C8_RESIDE…																														
CELLMARKERMSIGDB_AIZARANI_LIVER_C9_LSECS_1																														
CELLMARKERMSIGDB_BUSSLINGER_DUODENAL_BCHE…																														
CELLMARKERMSIGDB_BUSSLINGER_DUODENAL_DIFF…																														
CELLMARKERMSIGDB_BUSSLINGER_DUODENAL_EC_C…																														
[truncated: 1,270,323 more chars]
